# Impact of NSD1 Alternative Transcripts in Actin Filament Formation and Cellular Division Pathways in Fibroblasts

**DOI:** 10.3390/genes15091117

**Published:** 2024-08-24

**Authors:** Giuseppina Conteduca, Davide Cangelosi, Chiara Baldo, Alessia Arado, Barbara Testa, Ryan T. Wagner, Keith D. Robertson, Franck Dequiedt, Lane Fitzsimmons, Michela Malacarne, Gilberto Filaci, Domenico A. Coviello

**Affiliations:** 1 Biotherapy Unit, IRCCS San Martino, 16132 Genoa, Italy; giusy_conteduca@alice.it (G.C.);; 2 Clinical Bioinformatics Unit, IRCCS Istituto Giannina Gaslini, 16147 Genoa, Italy; davidecangelosi@gaslini.org; 3 Laboratory of Human Genetics, IRCCS Istituto Giannina Gaslini, 16147 Genoa, Italy; 4 Department of Molecular Pharmacology and Experimental Therapeutics, Mayo Clinic, Rochester, MN 55905, USA; 5 GIGA-Molecular Biology of Diseases Laboratory of Gene Expression and Cancer, University of Liege, 4000 Liège, Belgium; 6 Renaissance School of Medicine, Stony Brook University, Stony Brook, NY 11794, USA; 7 Department of Internal Medicine (DIMI), University of Genoa, 16132 Genoa, Italy

**Keywords:** *NSD1*, isoforms, cell cycle, cytoskeleton, neoplastic pathways

## Abstract

Germline variants in the NSD1 gene are responsible for Sotos syndrome, while somatic variants promote neoplastic cell transformation. Our previous studies revealed three alternative RNA isoforms of *NSD1* present in fibroblast cell lines (FBs): the canonical full transcript and 2 alternative transcripts, termed AT2 (NSD1 Δ5Δ7) and AT3 (*NSD1* Δ19–23 at the 5′ end). The precise molecular pathways affected by each specific isoform of *NSD1* are uncharacterized to date. To elucidate the role of these isoforms, their expression was suppressed by siRNA knockdown in FBs and protein expression and transcriptome data was explored. We demonstrate that one gene target of *NSD1* isoform AT2 is ARP3 actin-related protein 3 homolog B (*ACTR3B*). We show that loss of both canonical *NSD1* and AT2 isoforms impaired the ability of fibroblasts to regulate the actin cytoskeleton, and we observed that this caused selective loss of stress fibers. Our findings provide novel insights into *NSD1* function by distinguishing isoform function and demonstrating an essential role of *NSD1* in regulating the actin cytoskeleton and stress fiber formation in fibroblasts.

## 1. Introduction

Sotos syndrome (SoS) (OMIM #117550) is an autosomal dominant disorder with an incidence of 1:14,000 live births [1]. Clinical manifestations include overgrowth (increased height, macrosomia, and macrocephaly), distinctive facial features, and learning and intellectual disabilities [2,3,4]. Mutations that cause loss of function of the nuclear receptor SET domain containing protein-1 (*NSD1*) (NM 022455.4) are responsible for SoS. In mouse models, *NSD1* knockout mutations result in phenotypes concordant with those of patients with SoS, including overgrowth, limitations of motor learning and special memory and nervous system deficits [5,6,7]. The *NSD1* gene product is a SET-domain histone lysine methyltransferase that interacts with nuclear receptors [8,9]. *NSD1* functions to regulate chromatin, and *NSD1* mutations result in genome-wide methylation alterations [4,10,11]. Though this broad epigenetic concept is well understood, the specific alterations in pathways and their connections to pathogenic states in humans remain largely undefined. 

In our previous study, we identified two alternative transcripts of the *NSD1* gene in fibroblasts isolated from Sotos patients and healthy controls [12] (Figure S1). We described AT2 (NSD1 Δ5Δ7) and AT3 (NSD1 Δ19–23 5′ end) isoforms, characterized by the skipping of exons 5 and 7, and the skipping of exon 19 to the 5′ region of exon 23, respectively. In silico analysis of NSD1 protein structure showed that isoform 2 (encoded by AT2) is a shortened protein containing the PWWP1 domain but not the catalytic SET domain. This observation suggests that the NSD1 protein may have an alternative function beyond its established role in methyltransferase activity. Analysis predicting the structure of isoform 3 (encoded by AT3) revealed that this truncated isoform lacks C-terminal domains: PHD3 and PHD4 [13].

NSD1 activity is highly regulated, as evidenced by the presence of several overlapping mechanisms that set specific thresholds for NSD1 activation in different tissues and cells, in response to various stimuli [13]. Splicing plays an important role in gene regulation, especially during the development of tissues and organs. Alternative splicing is known to contribute to physiological functions in various developmental processes, as isoforms with specialized functions can be used to tailor expression to specific developmental stages [14]. Splicing has also been found to be highly disrupted in a broad array of cancer types, suggesting its involvement in regulating cell growth and proliferation [15]. Even more, not all detected alternative splicing events ultimately produce functional proteins due to various reasons: (a) the transcript may be non-coding, resulting in it never being translated into a polypeptide, (b) the stability of the transcript may be affected, leading to changes in its persistence or degradation rates, (c) the localization of the mRNA may be altered, disrupting the transcript’s or protein’s function [16]. Through these mechanisms, alternative splicing enables fine-tuned regulation of gene expression. It follows that disruption of such processes can result in growth and developmental abnormalities, as in cancers and congenital disease. 

**Figure 1:**
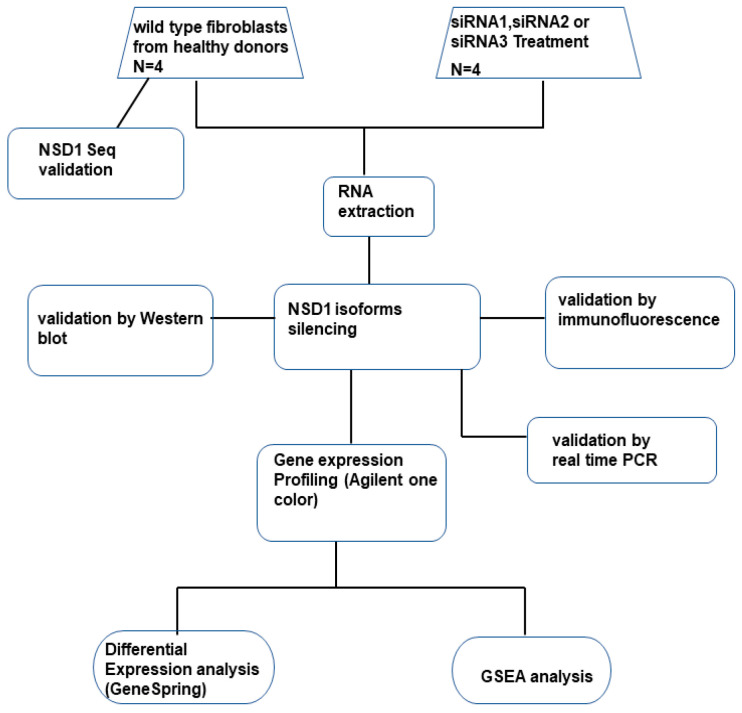
Schematic representation of the study strategy. Significant modulation of target genes between the wild-type fibroblast group and siRNA treated cells was calculated by a GeneSpring differential expression analysis tool. A Gene Set Enrichment Analysis (GSEA) identified statistically significantly modulated gene sets.

The *NSD1* gene exhibits widespread expression across various organs, including at elevated levels in most tissues of the healthy brain, pancreas, male reproductive tract, and hematopoietic organs [17]. Our studies on fibroblasts derived from SoS patients and healthy controls demonstrated that *NSD1* mutations result in changes in the expression of long noncoding RNAs and genes associated with neoplastic differentiation, particularly those that regulate the G2/M checkpoint [18]. Despite *NSD1*’s ubiquitous expression, specific characterization of each isoform, including the expression profile and precise biological role of each, remains limited. To elucidate the impact of AT2 and AT3 isoforms on noncoding RNA and mRNA expression, we generated transcriptional profiles in fibroblasts from two healthy controls and treated with siRNAs, targeting the NSD1 AT2 and AT3 isoforms specifically. A flowchart outlining the key steps of the study is shown in Figure 1.

## 2. Materials and Methods

### 2.1. Patients 

This study was conducted with the approval of the Ethics Committee of the Liguria Region (Approval #OG01IGG, 12 July 2021). Written informed consent was obtained from four healthy participants (two male and two female), selected based on the availability of skin biopsy. The fibroblast cell lines were available at the Gaslini Genetic Biobank (20GBG0075F, 20GBG0076F, GGB16417M, 21GBG0125M).

### 2.2. Cell Culture 

Fibroblast culture was maintained in RPMI-1640 medium (Thermo Fisher Scientific, Grand Island, NY, USA), complemented with 10% fetal calf serum (FCS), 2 mM L-glutamine, 100 U/mL penicillin and 100 µg/mL streptomycin (Euroclone S.p.a, Milan, Italy). In order to verify the absence of mycoplasma contamination, we used the mycoplasma detection kit (Lonza, Basel, Switzerland). Cell lines between passages 2 and 15 were used for all experiments.

### 2.3. Analysis of NSD1 Sequence

QIAamp^®^ DNA Blood kit (Qiagen, Milan, Italy) was used to extract DNA from fibroblasts according to the manufacturer’s protocol. PCR amplifications were performed using platinum-Taq DNA polymerase (Thermo Fisher Scientific, Carlsbad, CA, USA) and specific primers for the 23 different NSD1 exons as described [12]. We sequenced PCR products with the ABI BigDye Terminator Ready Reaction Mix (Thermo Fisher Scientific, Foster City, CA, USA) and analyzed them on an ABI 3130XL Genetic Analyzer (Applied Biosystems, Foster City, CA, USA) according to the manufacturer’s instructions. We aligned the sequences with SeqScape analysis software V.2.5 (Thermo Fisher Scientific, Foster City, CA, USA). The absence of deletion/duplication in the 5q35.3 region encompassing the NSD1 gene, array-CGH was verify with CGH 8 × 60 K (Agilent Technologies, Santa Clara, CA, USA). The data were analyzed with the Agilent Cytogenomics 4.0.3.12 software (Agilent Technologies, Santa Clara, CA, USA). We reported all genomic positions according to the human genome assembly (GRCh37/hg19).

### 2.4. siRNA Transfection

Fibroblasts were plated 24 h prior to transfection in 6-well plates (2 × 10^5^/well) for RNA analysis and in 8-well chamber slides for immunofluorescence tests. Three NSD1-specific siRNAs (Eurofins Genomics, Ebersberg, Germany) were used for silencing AT2 (siRNA-AT2), AT3 (siRNA-AT3) and the canonical NSD1 isoform (siRNA-AT1). We also designed a specific siRNA that binds the sequence of all NSD1 isoforms in order to completely silence NSD1, as described [19,20]. For siRNA design, we used siDirect v2.0. tool (http://sidirect2.rnai.jp/, accessed on 6 May 2022). The details of the siRNA duplexes are included in Table S4. We designed siRNA-AT2 specific for the AT2 NSD1 isoform, on the overlapping sequence between exon 4 and exon 6, while siRNA-AT3 specific for AT3 NSD1 isoform was designed on the overlapping sequence between exon 18 and exon 23. Single strands of siRNA were annealed. The annealing buffer contained 10 mM Tris, pH 7.5, and 20 mM NaCl in RNAase free water. Samples were heated to 95 °C for 1 min, then cooled and annealed at room temperature for 12–16 h. The siRNAs were then precipitated and resuspended in RNAase-free water. Fibroblasts (4 × 10^5^) were transfected with 20 nM of each specific siRNA using DOTAP liposomal transfection reagent (Roche Applied Science, Monza, Italy) according to the manufacturer’s instructions. The cells were transfected with active siRNA-AT1, siRNA-AT2, and siRNA-AT3, as well as with anti-Cy3 siRNA and anti- PPIA, and analyzed at various time points: 6, 24, and 48, hours post-transfection. Cells treated with the Cy3-specific and anti-PPIA siRNA were used as controls. NSD1 silencing by siRNA was validated by real-time PCR and immunofluorescence analysis. To confirm that NSD1 knockdown was induced by specific NSD1 RNA interference treatment, we used anti-PPIA siRNA as control knockdown rescue, because PPIA is a constitutively expressed gene in a wide range of cell types (Figure S2A).

### 2.5. Indirect Immunofluorescence

Fibroblast cells (1 × 10^4^/well) were transfected with siRNA-AT1, siRNA-AT2, siRNA-AT3 and anti-Cy3 siRNAs in an 8-well chamber slide in the same manner as described in 2.4. At 24 h post-transfection, immunofluorescence was used to quantify the NSD1 protein expression as a consequence of the siRNAs. Untransfected fibroblasts (1 × 10^4^/well) were grown in concurrent experiments. The medium in the plate was aspirated, washed with PBS (pH 7.4), and fixed at room temperature for 10 min in PBS containing 4% paraformaldehyde. PBS was used to briefly wash the fixed cells, and nonspecific binding was blocked using 5% BSA in PBS. We used an anti-NSD1 antibody that recognized an N-terminal epitope on the NSD1 protein (mouse monoclonal antibody, clone 1K47, Santa Cruz, CA, USA), and used an anti-NSD1 antibody that recognized an epitope encoded by exon 5 (rabbit polyclonal anti-NSD1 primary antibody, HPA048433, Sigma, CA, USA), at a dilution of 1/500 incubated overnight at 4 °C. Eight-well chamber slides were washed in PBS and incubated at room temperature for 1 h in the dark with either Alexa Fluor™ 594 conjugated donkey anti-rabbit secondary antibody, or Alexa Fluor™ 594 goat anti-mouse antibody (Sigma, CA, USA) at a dilution of 1/500. PBS was used to wash the cells three times. To confirm that the results were specific to the fibroblasts, not an artefact, an experiment with anti-PPIA siRNA (positive control for repression) and anti-Cy3 siRNA (negative control for repression) transfected cells was performed concurrently. For β-actin, mouse monoclonal anti-β-actin (clone C-2, Sigma, CA, USA) at a dilution of 1/100 and goat anti-mouse secondary antibody conjugated with Alexa Fluor™ 488 at a dilution of 1/500 were used. For β-tubulin a rabbit polyclonal antibody at a dilution of 1/500 (Thermo Fisher Scientific, Carlsbad, CA, USA) and an Alexa Fluor™ 594 conjugated donkey anti-rabbit secondary antibody were used. The fluorescence images were obtained using a digital camera (AX7O, Olympus, MI, Italy).

### 2.6. Gene Expression Profiling

Gene expression profiling was performed as previously described by Conteduca et al. [18]. Briefly, TRIzol reagent was used for RNA extraction (Thermo Fisher Scientific, MA, USA). We verified RNA quality with a NanoDrop ND-1000, an Agilent 2100 bioanalyzer. We performed microarray hybridization using the Agilent One Color microarray gene expression kit and the SurePrint G3, 8 × 60 K Human Gene Expression V3 array (Agilent Technologies, Santa Clara, CA, USA) as described [18]. Raw data were extracted using Feature Extraction (version 12.0.1.1; Agilent Technologies). Next, data preprocessing, including normalization and filtering, was carried out with the Genespring software (version 14.3; Agilent Technologies). Raw data were normalized by a 75-percentile shift, log2-transformed and shifted to the median of all samples. The microarray data were deposited in GEO under the accession number GSE253402 (http://www.ncbi.nlm.nih.gov/geo/query/acc.cgi?acc=GSE253402, accessed on 17 January 2024), GSE253403 (http://www.ncbi.nlm.nih.gov/geo/query/acc.cgi?acc=GSE253403, accessed on 17 January 2024), GSE253404 (http://www.ncbi.nlm.nih.gov/geo/query/acc.cgi?acc=GSE253404, accessed on 17 January 2024). Only samples of excellent quality were used for subsequent analyses in order to reduce potential biases introduced by analyzing low-quality specimens.

### 2.7. Quantitative Real-Time RT–PCR

We performed real-time quantitative PCR to confirm the silencing effect on NSD1 isoforms by siRNA-AT1, siRNA-AT2, siRNA-AT3 and siRNA4, and to confirm results obtained by gene expression array analysis (primers listed in Table S5). Exon-overlapping primers were used to quantitatively determine gene expression in real time [12]. Total cellular RNA was extracted and reverse transcribed according to the manufacturer’s protocol. Synthesis of cDNA was carried out from 400 ng of the total RNA using the Advantage RT cDNA Kit (Clontech, Mountain View, CA, USA) following the manufacturer’s instructions. Specifically, samples were incubated at 42 °C for 90 min, followed by at 90 °C for 2 min. Quantitative real-time PCR was conducted using LightCycler 480 SYBR Green I Master (Roche Diagnostics, Mannheim, Germany) in a 15 µL reaction mixture. GAPDH served as an internal control and was used to normalize gene expression values with the 2^−∆∆Ct^ method [21]. Each experiment was performed in triplicate for robustness (Figures S2A and S3).

### 2.8. Western Blotting

Wild type fibroblasts obtained from healthy donors and treated with NSD1 siRNA-AT1, siRNA-AT2, and siRNA-AT3 were lysed in RIPA buffer (NaCl 150 mM, Nonidet P-40 1%, sodium deoxycholate (DOC) 0.5%, sodium dodecyl sulfate (SDS) 0.1%, Tris-HCl (pH 7.4) 50 mM). For protein analysis by Western blotting, whole cell lysates (50 µg) for each sample were loaded and separated by SDS/PAGE using a blot 4–12% Bis Tris Plus (Thermo Fisher Scientific, CA, USA). Proteins were transferred to Immobilon-P PVDF membranes (Millipore, Burlington, MA, USA), and BSA in Tris-buffered-saline (TBS) was used to block membranes. Next, membranes were probed with primary antibodies diluted in TBS/0.05% Tween-20 (TBS-T)/5% BSA followed by the appropriate secondary antibody anti-mouse or anti-rabbit horseradish peroxidase (HRP)-conjugated reagent. An ECL Substrate (Bio-Rad, CA, USA) was used for detection. Images were acquired using Alliance Q9 Advanced Chemiluminescence Imager (UVITEC). We performed Western blotting analysis with either a mouse monoclonal antibody that recognized an N-terminal epitope of the NSD1 protein (1:1000, clone K47, Santa Cruz, USA), or with an anti-NSD1 antibody that recognized an epitope encoded by exon 5 (Rabbit polyclonal anti-NSD1 primary antibody, HPA070333, Sigma, USA), or with a β-actin mouse monoclonal antibody (1:5000, clone C-2, Sigma, USA), or a mouse monoclonal antibody anti-GAPDH (1:5000, clone FF26A, Sigma, USA). We used anti-rabbit or anti-mouse horseradish peroxidase-linked antibodies as secondary antibodies (Thermo Fisher Scientific, CA, USA) (Figure S2B).

### 2.9. Bioinformatic Analysis

The bioinformatic analysis was performed as previously described by Conteduca et al. [19] on 4 fibroblast cell lines in separate independent experiments (20GBG0075F, 20GBG0076F, GGB16417M, 21GBG0125M).

Based on the normalized fluorescence signal values of the lncRNA/mRNA probes, this analysis showed differential expression.

Protein–protein functional interactions among the differentially expressed genes were evaluated with the STRING database (http://stringdb.org, accessed on 24 May 2023) [22] using default parameters. An extension of the network was analyzed to identify potential indirect interactions between the differentially expressed genes. To address these limitations, a gene set enrichment analysis (GSEA) [23] was used to test the enrichment of functionally related gene sets in FB cell lines. Chemical and genetic perturbations (C2.CGP), hallmark (H), gene ontology biological processes (C5.GO.BP) and gene set collections retrieved from the Molecular Signature Database (MSigDB) v7.4 [24]. were used for enrichment analyses. GSEA calculated an enrichment score (ES) and a normalized enrichment score (NES) for each gene set, with the statistical significance of the NES estimated, using an empirical permutation test using 1000 gene permutations in order to obtain the nominal *p*-value (NOM *p*-value). The gene sets analyzed contained between 15 and 250 genes. Gene sets with nominal *p*-values < 0.05 and FDR q-values < 0.05 were considered significantly enriched.

## 3. Results

### 3.1. Effect of Anti-NSD1 siRNAs on NSD1 Isoform Expression

**Figure 2:**
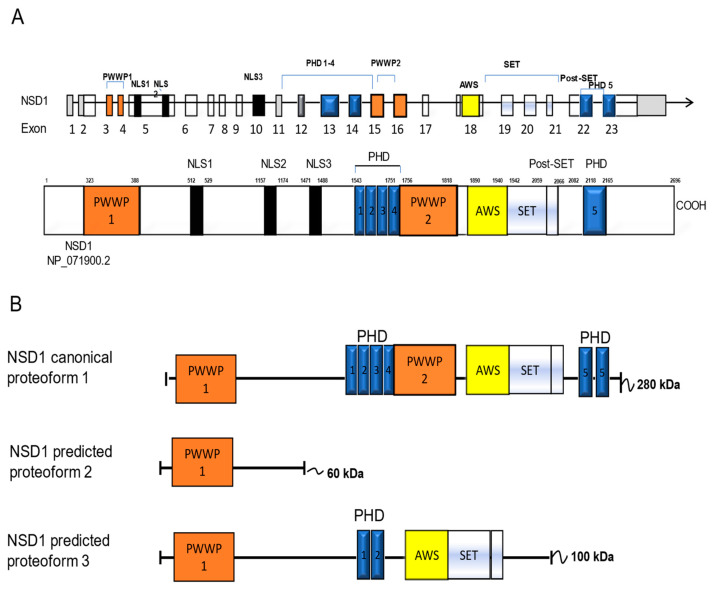
Schematic representation of the NSD1 isoforms. (**A**) NSD1 gene (**top**) and NSD1 canonical protein (**bottom**) with all functional domains indicated. Open boxes denote coding exons and grey boxes denote the 5′ and 3′ untranslated regions. (**B**) Modular domain architecture of putative NSD1 protein isoforms. Predicted protein domains were detected using PFAM motif markseq server (https://www.genome.jp/tools/motif/, accessed on 26 July 2023) and trRosetta tool (https://yanglab.nankai.edu.cn/trRosetta/, accessed on 2 October 2023). Colored boxes highlight specific functional domains. PWWP: proline-tryptophan-tryptophan-proline domain; NLS: nuclear localization signal; PHD: plant homeodomain domain; AWS: associated with SET domains; SET: Su(var)3–9, Enhancer-of-zeste, Trithorax domain.

To determine the specific roles of the AT2 and AT3 encoded polypeptides, each with unique functional domains with respect to the canonical NSD1 isoform, we established a workflow integrating both in vitro and in silico experiments. Our objective was to analyze the molecular pathways up- and down-regulated by the NSD1 AT2, AT3, and AT1 isoforms. A flowchart outlining the key steps of the study is shown in Figure 1 and a diagram of the NSD1 canonical gene and NSD1 predicted domain architecture for each NSD1 isoforms is displayed in Figure 2A,B.

To ensure the specificity of individual siRNAs for their target mRNA, a critical consideration in studies using siRNA, we designed three NSD1 siRNAs (anti-NSD1 siRNA-AT1, anti-NSD1 siRNA-AT2, and anti-NSD1 siRNA-AT3) against each of the specific RNA isoforms of the NSD1 gene (Figure S1). The effect of siRNA-mediated silencing on the levels of NSD1 protein and RNA was assessed by RT–PCR (Figure S2A) and Western blot (Figure S2B). Following fibroblast transfection, we observed maximal knockdown of NSD1 expression between 6 and 24 h.

### 3.2. NSD1 Protein Isoform AT3 Exhibits Distinct Subcellular Localization with Respect to the Long Canonical Isoform in Fibroblasts

**Figure 3:**
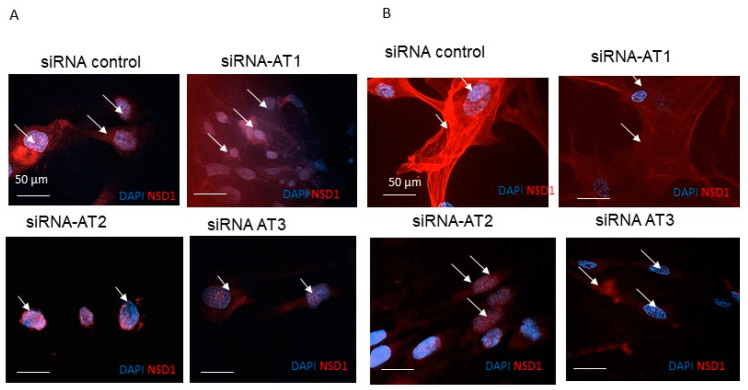
Expression analysis of NSD1 isoforms in fibroblasts by immunofluorescence. (**A**) Representative image of IF with rabbit polyclonal NSD1 antibody, HPA070333 for the exon 5 epitope. (**B**) Representative image of IF with mouse monoclonal NSD1 antibody, sc-130470, clone K47, binding to the N-terminal epitope. After 24 h of post anti-NSD1 siRNA treatment and untreated control cells, DAPI was used to stain the cell nucleus to locate cells and Alexa fluor 595-labelled NSD1 antibodies for NSD1 AT2, NSD1 AT3, and NSD1 AT1. (×100 magnification; blue = DAPI; red = NSD1. Scale bar = 50 µm).The arrows indicate NSD1 red signals.

Immunostaining wild-type (WT) fibroblasts with antibodies against an NSD1 N-terminal epitope that identifies AT1 and AT2 isoforms, or an NSD1 exon 5 epitope that identifies AT1 and AT3, showed a positive NSD1 signal both in the cytoplasm and the nucleus (Figure 3A). When we treated fibroblasts with siRNA-AT1, immunofluorescence staining with an antibody that recognized the NSD1 exon 5 epitope (AT1 and AT3), only the nuclear signal was detected. In contrast, the antibody that recognized the NSD1 N-terminal epitope displayed both the cytoplasmatic and nuclear signal (Figure 3B).

When we treated fibroblasts with siRNA-AT2 or siRNA-AT3 and stained with antibodies that recognize the *NSD1* exon 5 epitope or NSD1 N terminal-epitope, we observed both cytoplasmatic and nuclear signals. This suggested that the AT2 isoform contributes to the cytoplasmatic signal (Figure 3A,B). It is also of interest that we observe that the nuclear-specific AT3 isoform retains the SET domain whereas AT2 does not, thus AT2’s cytosolic functions are independent of the catalytic activity. We also designed a specific siRNA that binds the sequence of all NSD1 isoforms in order to completely silence NSD1 (Figure S3).

### 3.3. NSD1 AT2 and NSD1 AT3 Isoforms May Mediate Transcriptomic Effects on BCL2, KRAS and p53 Signaling Pathways

Fibroblasts obtained from four different healthy donors were screened to confirm the absence of NSD1 mutations. To silence specific NSD1 isoforms (canonical AT1, AT2 and AT3), wild-type fibroblasts were treated with siRNA-AT1, siRNA-AT2 or siRNA-AT3 in three independent experiments, achieving isoform silencing after 24 h of treatment. Data presentation is identical and independent for all four different fibroblasts cell lines. We showed mean value data.

**Figure 4:**
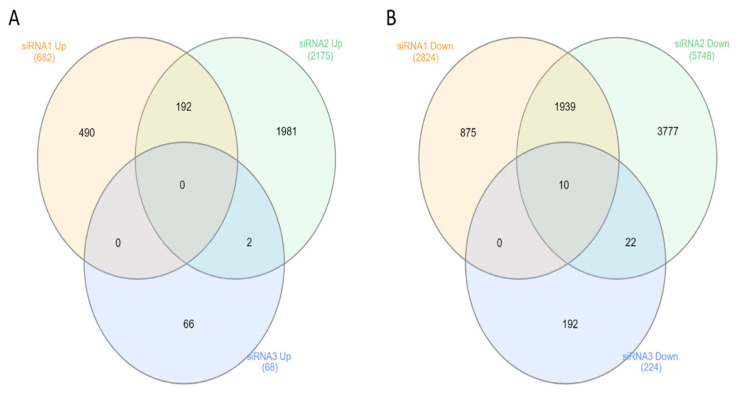
Venn diagram comparing shared and unique DEGs following NSD1 siRNA-AT1, siRNA-AT2 and siRNA-AT3 treatment of fibroblasts. Common and uniquely upregulated genes (**A**) and downregulated genes (**B**) are indicated.

Gene expression analysis identified 4174 significantly differentially expressed mRNAs (DEGs) after anti-NSD1 siRNA-AT1 treatment (Table S1). The most upregulated gene in the *NSD1* siRNA-AT1 treated samples was associated with the *FGFR1* oncogene partner 2 gene (*FGFR1OP2*) (fold change, 5.8, *p* < 0.05), and the most downregulated gene was associated with the polymerase RNA I polypeptide C *(POLR1C*) gene (fold change, −3.4, *p* < 0.05). Differential expression analysis identified 9785 significantly differentially expressed mRNAs after anti-NSD1 siRNA-AT2 treatment (Table S2). The most upregulated gene was associated with the *KISS-1* metastasis-suppressor (*KISS1*) gene (fold change, 6.6, *p* < 0.05), and the most downregulated was associated with the T-cell lymphoma invasion and metastasis 1 (*TIAM1*) gene (fold change, −5.5, *p* < 0.05). A differential expression analysis identified 337 significantly differentially expressed mRNAs after anti-NSD1 siRNA-AT3 treatment (Table S3). The most upregulated gene was associated with the doublecortin-like kinase 1 (*DCLK1*) gene (fold change, 0.9, *p* < 0.05), and the most downregulated was associated with the fem-1 homolog a (*FEM1A*) gene (fold change, −1.3, *p* < 0.05). The number of shared DEGs between wild type and siRNA-AT1, siRNA-AT2 and siRNA-AT3 treated fibroblasts is summarized as a Venn diagram (Figure 4). This analysis revealed a general exclusivity across the subsets of deregulated genes but showed a common pattern of genes regulated by isoforms 1, NSD1 AT2 isoform and NSD1 AT3 isoform. Following siRNA treatment, we observed concordant genes regulated under isoform 1 knockdown conditions (wild type vs. NSD1 siRNA-AT1 + wild type vs. NSD1 siRNA-AT2 + wild type vs. NSD1 siRNA-AT3), in both datasets of silenced isoform 2 and isoform 3 genes. Therefore, these DEGs represent common gene sets regulated by NSD1 AT1 and NSD1 AT2. We combined these concordant lists of NSD1-regulated genes by anti NSD1 siRNA-AT3 specific for silencing of NSD1 AT3 isoform (two genes positively and 47 negatively regulated including in fibroblasts treated with anti NSD1 siRNA-AT3), to describe the role of specific NSD1 isoforms in fibroblast molecular pathways. We observed that there were DEG uniquely regulated by NSD1 isoform AT3. The most relevant downregulated genes after siRNA -AT1 treatment were fibroblast growth factors (*FGF1*, *FGF3*) and *KRAS* (Table 1, Figure S4A). In addition, genes coding for proteins involved in the cytoskeleton and stress fiber formation *ACTR2*, *ACTR3*, *ARPC2*, and *ARPC3*, were significantly downregulated after NSD1 AT2 knockdown (Table 2, Figure S4B). Anti-NSD1 siRNA-AT3 treatment also resulted in significantly decreased expression of the prostaglandin E2 receptor *FEM1A* and *FEM1B* genes, and neural precursor cell, *NEDD* (Table 3, Figure S4C). To confirm differential gene expression induced by siRNA treatment, mRNA levels of a representative gene were quantified by RT–PCR (Figure S4).

**Figure 5:**
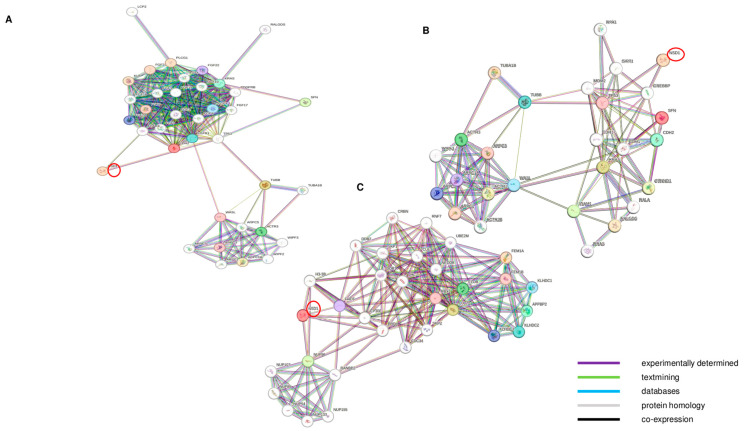
Protein–protein interaction network among differentially expressed genes between wild type fibroblasts group and treated with NSD1 siRNA-AT1 (**A**) or with NSD1 siRNA-AT2 (**B**) or with NSD1 siRNA-AT3 (**C**). Protein–protein interaction networks were generated using the STRING database to assess potential functional interactions among selected genes involved in actin cytoskeleton organization, cell differentiation and cell cycle regulation. Networks were expanded to assess likely indirect interactions. Nodes represent gene products, and edges represent protein–protein associations. Only the associations with an evidence score higher than 0.3 are shown, with colors indicating different kinds of evidence. The legend for evidence type is shown in the bottom right corner. Networks showed that the significantly modulated genes in the distinct experiments did not show direct interactions with NSD1, but they did show indirect interactions with a subset of other related gene products. Red circle showed *NSD1* gene.

Network analysis performed using the STRING-DB software, v11.0 [22] showed more connections among these genes than expected by chance (PPI interaction *p*-value < 0.05), indicating their potential involvement in common biological processes. Knowing that indirect interactions likely exist between differentially expressed genes, we also expanded our gene network (Figure 5). In the expanded network, the significantly modulated genes did not display any direct interaction with *NSD1*. These genes did display interactions with a subset of other gene products, including *KRAS*, *BCL2*, p53, β tubulin and actin-related protein 3. These gene products are known to interact with *NSD1* [25,26,27,28]. In particular, we observed that the *NSD1* AT2 isoform is implicated in *KRAS* and β actin protein-associated molecular pathways. In addition, the *NSD1* AT3 isoform is involved in *EP300*, *H4C6*, *H3-3B*, *DDB1*, *FEM1A* and *FEM1B*-related pathways. These results suggest that *NSD1* potentially functions to regulate genes involved in cell differentiation and proliferation, and that *NSD1* AT2 and *NSD1* AT3 isoforms contribute to the regulation of pathways distinct from those regulated by the canonical *NSD1* isoform.

### 3.4. Gene Set Enrichment Analysis to Perform an Unbiased Biological Interpretation of the Data

**Figure 6:**
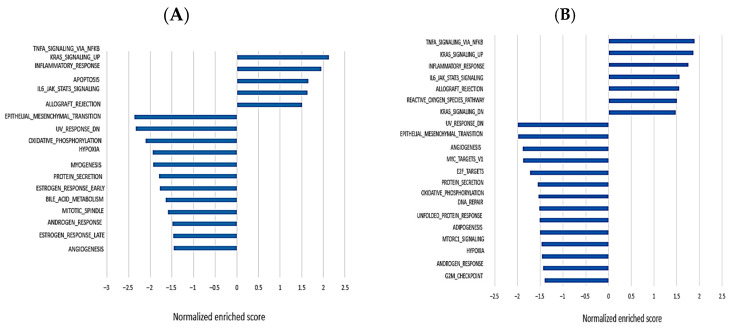
Lists of statistically significantly enriched gene sets from the Hallmark collection. Gene Set Enrichment Analysis (GSEA) was used to perform an unbiased biological reasoning of the expression data in different treatments. Gene sets with nominal *p* − values < 0.05 and FDR q − values < 0.05 were considered significantly enriched. Plots report the normalized enriched score of gene sets in decreasing order. The *Y* −axis reports the gene set names. (**A**) Gene sets enriched in the group treated with NSD1 siRNA-AT2. (**B**) Gene sets enriched in the group treated with NSD1 siRNA-AT3. It is evident that siRNA-AT2 treatment induces a downregulation of the mitotic spindle pathway, which is not observed after treatment with siRNA-AT3.

Gene set enrichment analysis (GSEA) computationally assesses the coordinated expression modulation of functionally related genes between two groups [23]. GSEA analysis was performed using the gene sets included in the chemical and genetic perturbation, hallmark (H), and C2 gene ontology collections from the MSigDB database [24]. There were 89 significantly enriched biological processes and pathways in the anti-NSD1-siRNA-AT2 treated fibroblasts compared to control fibroblasts (Figure 6A), and in the anti NSD1-siRNA-AT3 treated fibroblasts compared to untreated fibroblasts (Figure 6B). After NSD1 AT2 silencing, the most over-represented gene sets were related to cell cycle and proliferation, cell differentiation, and P53-mediated cell cycle arrest (Figure S5). The most under-represented gene sets were related to cell cycle G2/M checkpoint and epithelial mesenchymal transition pathway (Figure S6). In the treated fibroblasts after *NSD1* AT3 isoform knockdown, we observed that upregulated genes were mainly involved in *TNFA* signaling, *NFKB*, *KRAS* signaling, and the inflammatory response. The most under-represented gene sets were *E2F* pathway, *MYC* targets, the cell cycle G2/M, androgen response, and the unfolded protein response pathway (Figure 6B). Additionally, we examined differentially regulated genes from AT2 knock-down vs AT3 knock-down and observed that they target distinct subsets of genes. Specifically, AT2-modulated genes contributed to actin cytoskeleton and stress fiber formation, while AT3-regulated genes were involved in cell cycle regulation and in tumoral and neoplastic development.

### 3.5. NSD1 Is Involved in the Cytoskeleton Actin Stress Fiber (SFs) Organization Pathway

**Figure 7:**
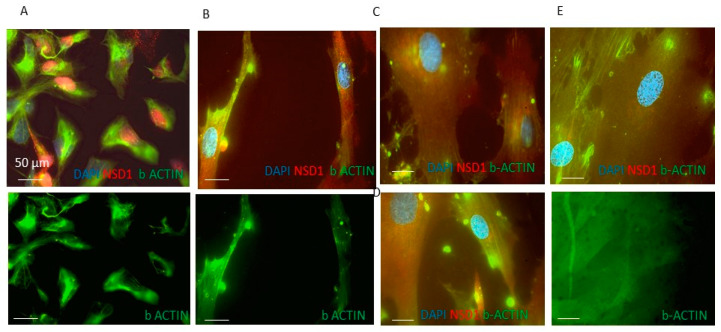
NSD1 loss in fibroblasts (FBs) impairs actin cytoskeleton organization and stress fiber structure. (**A**) Representative images of FBs 24 h post-transfection with anti NSD1 siRNA-AT2 showed a morphological transition into an amoeboid phenotype and (**B**) FBs not transfected with siRNA displayed a typical flat and elongated structure. (**C**) FBs transfected with anti-NSD1 siRNA-AT1 showed NSD1 isoform 1 knockdown and normal β-actin expression. (**D**) FBs transfected with anti Cy3 siRNA control. (**E**) FBs transfected with anti NSD1 siRNA-AT3 (×100 magnification; blue = DAPI; red = NSD1; green = β-actin. Scale bar = 50 µm). Immunofluorescence images with NSD1 antibody, sc-130470, clone K47, confirmed that AT2 isoform silencing changed stress fiber organization in fibroblasts, therefore we observed an ameboid phenotype induced by siRNA-AT2 treatment (**A**) with respect to the canonical flat, elongated morphology characteristic of fibroblasts. Since the cells in culture were heterogeneous, some differences of fluorescence intensity can be noted compared to the same treatment condition in Figure 3.

To investigate the potential role of NSD1 in cytoskeleton formation and fiber positioning in mitosis and meiosis, we performed immunofluorescence analysis with anti-β Actin antibody after siRNA-AT1, siRNA-AT2 or siRNA-AT3 treatment. Fibroblast cultures were treated with siRNA targeting each NSD1 isoform individually for 24h with or without anti-Cy3 siRNA control (Figure 7). Interestingly, we observed that NSD1 canonical protein AT1 and NSD1 AT2 isoform were co-localized with β-actin proteins in a cell context-dependent manner on SFs (Figure 7B,D). Using immunofluorescence staining, we confirmed NSD1 knockdown after siRNA treatment, and we observed that NSD1 AT2 isoform silencing induced a morphological transition into an amoeboid phenotype in fibroblasts, with respect to wild-type cells, characterized by a flat, elongated morphology and processes extending out from the ends of the cell body (Figure 7A,B and Figure S7).

## 4. Discussion

A more nuanced understanding of the biological functions of the *NSD1* gene will lead to insights into the etiologies of SoS and cancer. *NSD1* encodes for a histone methyltransferase that regulates protein transcription by modifying chromatin. NSD1’s *mono-* and *di*-methylation of H3K36 is mainly associated with regulating gene expression, DNA repair, and alternative splicing [29,30]. An expanding body of research indicates that the range of biological functions regulated by NSD1 is broad and complex [31,32,33].

Acquired somatic variants in NSD1 drive neoplastic cell transformation, whereas germline mutations cause Sos. NSD1 mutations can inhibit cellular differentiation by inducing alternate methylation of H3K36 [34,35]. This inhibition can promote oncogenesis, as evidenced by the frequent occurrence of NSD1 mutations in cancer [36,37]. The association between NSD1 and SoS is rooted in the gene’s role in regulating developmental processes. It is reported that NSD1 is an important gene for early post-implantation and embryonic development [38], aligning with its involvement in a congenital disease characterized by developmental delay and overgrowth.

In this study, we investigated which genes are up- or down-regulated by the NSD1 canonical isoform AT1 and the NSD1 isoforms AT2 and AT3 after RNA interference using siRNA-AT1, siRNA-AT2 and siRNA-AT3. We used fibroblast cell line fibroblasts cell lines because they were used as in vitro model for patients carrying NSD1 mutation affected by Sotos Syndrome. We already have produced iPSC from those cell lines [39,40].

Our results showed that NSD1 AT1 canonical isoform and NSD1 AT3 isoform are involved in the control of different genes important for neoplastic pathways and cell cycle regulation, while NSD1 AT2 isoform was involved in cytoskeleton organization and actin fiber distribution. Expression Microarray analysis revealed 4174 significantly differentially expressed mRNAs after NSD1 canonical AT1 isoform silencing, 9785 significantly differentially expressed mRNAs after NSD1 AT2 silencing and 337 after NSD1 AT3 silencing. To better understand the potential underlying mechanisms of the DEG, we also performed in silico GSEA analyses.

The results demonstrated that genes involved in meiotic and mitotic division are up- or down-regulated by specific NSD1 isoforms. Relevant points of regulation included checkpoints that control transitions between cell cycle phases and the onset of cell senescence. Abnormalities in regulation of this nature have been determined to be associated with neoplastic disease and tumor development [41]. Furthermore, we found that the KRAS signaling pathway, TNFA signaling via *NFKB,* and apoptosis by *CDKN1A* via TP53-signaling were remarkably upregulated in fibroblasts after NSD1 AT1 or AT3 isoform silencing. On the other hand, genes involved in DNA repair, cell cycle G2/M checkpoint and epithelial mesenchymal transition pathways were remarkably downregulated in fibroblasts after NSD1 AT1 and NSD1 AT3 silencing. Among the differentially expressed genes, KiSS-1 was the most upregulated by NSD1 AT2 (FC = 6.6). The KiSS-1 protein inhibits melanoma and breast carcinoma metastasis [42,43]. This metastasis-suppressor protein may interfere with the migration of cancer cells toward signals promoting their invasion. Additionally, changing the cytoskeleton structure has been proposed as a mechanism by which the KiSS-1 protein suppresses metastasis [44]. T-cell lymphoma invasion and metastasis 1 (*TIAM1*) was the most downregulated gene by the NSD1 AT2 isoform (FC= −5.5). Therefore, when integrated with the previously reported evidence, our study suggests the potential importance of the AT2 isoform (along with the NSD1 AT1 canonical protein) in regulating genes involved in cell cycle, mitotic translation, and G2/M checkpoint.

In this study, comprehensive gene expression analysis was performed after gene expression suppression by siRNA. Our results showed a change in the gene group, whose expression differed both as a direct result of the of gene expression suppression and also as a secondary consequence due to the suppression of the first gene. Further research should be carried out to distinguish between the direct and indirect expression consequences.

According to the reported involvement of *NSD1* in tumorigenesis [45,46], our study may help to clarify which distinct pathways of genes associated to tumor are specifically regulated by different NSD1 isoforms. In addition, in our study, *FEM1A* was the most down-regulated gene after NSD1 AT3 knockdown (FC= −1.3) and *DCLK1* was the most up-regulated gene after anti-NSD1 siRNA-AT3 knockdown (FC = 0.9). *FEM1A* encodes for fem-1 homolog A protein, a negative regulator of inflammatory response and ubiquitin-dependent protein catabolic process [47]. *DCLK1* encodes for the doublecortin-like kinase 1, which binds microtubules and regulates microtubule polymerization [48]. This kinase is also important during brain development due to its involvement in the calcium-signaling pathway that controls neuronal migration [48].

We also observed that the AT1 canonical and AT2 isoforms co-localized with β-actin proteins in a cell context-dependent manner on cytoskeleton actin stress fibers. In recent years the idea of the “histone code” has been reconceptualized as a “tubulin code” to describe how post-translational modifications (PTMs), like histone H3 lysine 36 trimethylation (H3K36me3). of chromatin distinctly label subsets of microtubules in the cytoskeleton [49]. Other PTMs, such as phosphorylation, detyrosination, polyglutamylation, polyglycylation and acetylation, are concentrated on specific microtubule structures. like neuronal axons, primary cilia, centrioles and basal bodies.

Microtubule-associated proteins (MAPs) are able to recognize PTMs and promote dynamic changes in microtubules during mitosis [50]. The role of PTMs associated with the mitotic spindle and midbody microtubules in mitotic polymerization and depolymerization remains unclear [51,52,53]. Moreover, in cancer, where defects in genes involved in chromatin remodeling of H3K36me3 are detected with high frequency, research has focused on chromatin and the consequences of H3K36me loss on the epigenome. Our results highlight that the NSD1 AT2 isoform could cooperate with the long canonical AT1 protein in regulating cytoskeleton fibers during mitosis and meiosis processes. Currently, we propose our manuscript that shows the first functional evidence based on transcriptomics and immunohistochemistry In addition to gene expression analysis and immunostaining assay, more validation is necessary to confirm our data regarding cell cycle assay and gene silencing on up-regulated and down-regulated target of NSD1 isoforms.

## 5. Conclusions

In summary, this study suggests that in fibroblasts the AT2 NSD1 isoform plays a role in β-actin interaction and in stress fiber organization, while NSD1 isoform AT3 promotes AT1 canonical isoform activity as a check point of cell differentiation and cell division, preventing neoplastic transformation. These expression signatures may be useful tools to understand the function of NSD1 in different cellular processes and to determine the specific impact of NSD1 isoforms on the mechanisms of tumorigenesis and in embryogenesis.

Further studies are necessary to clarify the detailed molecular mechanism of interaction between NSD1 and β-actin, as well as its impact on cytoskeleton actin stress fiber organization.

## Figures and Tables

**Table 1:** Relevant significant differentially expressed probe sets after anti-NSD1 siRNA-AT1 treatment.

Probe Set ID	Gene Symbol	Gene Name	Seq. Name	Log_2_ Fold Change	*p*-Value	*p*-Value Adjusted
A_33_P3389286	*SFN*	stratifin	NM_006142	5.2	1.61 × 10^−1^	0.002
*A_33_P3321205*	*BEGAIN*	brain-enriched guanylate kinase-associated	NM_001159531	*1*	*0.002*	*0.011*
A_33_P3363898	*TUBG1*	tubulin, γ 1	NM_001070	−0.29	0.011	0.03
A_32_P377880	*GDNF*	glial cell derived neurotrophic factor	NM_001190468	−0.4	0.009	0.025
A_23_P80694	*ACTR8*	ARP8 actin-related protein 8 homolog	NM_022899	−0.5	0.003	0.014
A_23_P377376	*ACTR2*	ARP2 actin-related protein 2 homolog	NM_001005386	−0.5	0.003	0.013
A_33_P3330283	*SP1*	Sp1 transcription factor	NM_138473	−0.6	0.022	0.049
A_24_P726336	*PHACTR2*	phosphatase and actin regulator 2	NM_001100164	−0.6	0.018	0.043
A_33_P3363674	*NFYC*	Nuclear transcription factor Y	NM_001142590	−0.7	0.0019	0.010
A_33_P3260016	*NEDD1*	neural precursor cell expressed, developmentally down-regulated 1	NM_152905	−0.7	0.003	0.014
A_24_P251969	*FGF1*	fibroblast growth factor 1	NM_000800	−0.8	1.13 × 10^−2^	0.029
A_23_P153256	*FGF3*	fibroblast growth factor 3	NM_005247	−0.8	8.64 × 10^−6^	0.035
A_23_P123193	*ACTR3B*	ARP3 actin-related protein 3 homolog B	NM_020445	−0.9	2.3 × 10^−2^	0.002
A_23_P162596	*ACTR6*	ARP6 actin-related protein 6 homolog	NM_022496	−1	0.006	0.019
A_23_P311201	*ACTR3*	ARP3 actin-related protein 3 homolog	NM_005721	−1.1	6.2 × 10^−2^	0.031
A_23_P208961	*KRAS*	Kirsten rat sarcoma viral oncogene homolog (KRAS)	NM_004985	−1.1	8.00 × 10^−6^	0.039
A_33_P3363799	*NCAM1*	neural cell adhesion molecule 1	NM_001242607	−3.1	0.011	0.030
A_22_P00001924	*DDB1*	damage-specific DNA binding protein 1	NM_001923	−3.9	7.04 × 10^−2^	0.029

**Table 2:** Relevant significant differentially expressed probe sets after anti-NSD1 siRNA-AT2 treatment.

Probe Set ID	Gene Symbol	Gene Name	Seq. Name	Log_2_ Fold Change	*p*-Value	*p*-Value Adjusted
A_23_P77493	*TUBB3*	tubulin, β 3 class III	NM_006086	1	0.002	0.006
*A_23_P102122*	*ARPC2*	actin-related protein 2/3 complex, subunit 2, 34 kDa	NM_152862	−0.2	0.007	0.010
A_23_P377376	*ACTR2*	ARP2 actin-related protein 2	NM_001005386	−0.4	7.9 × 10^−3^	0.004
*A_24_P167473*	*ARPC3*	actin-related protein 2/3 complex, subunit 3, 21 kDa	NM_001278556	−0.5	0.002	0.006
A_33_P3416946	*ACTR10*	actin-related protein 10 homolog	NM_018477	−0.7	0.013	0.014
*A_24_P72479*	*ARPC1A*	actin-related protein 2/3 complex, subunit 1A, 41 kDa	NM_006409	−0.8	0.005	0.009
A_23_P19291	*TUBB2A*	tubulin, β 2A class IIa	NM_001069	−0.8	0.002	0.006
*A_33_P3338909*	*ARPC5*	actin-related protein 2/3 complex, subunit 5, 16 kDa	NM_001270439	−0.9	9.12 × 10^−2^	0.004
A_23_P60283	*PHACTR2*	phosphatase and actin regulator 2	NM_014721	−1	0.001	0.005
*A_21_P0014389*	*ACTR3*	ARP3 actin-related protein 3 homolog	NM_005721	−1	1.96 × 10^−3^	0.003
A_23_P162596	*ACTR6*	ARP6 actin-related protein 6 homolog	NM_022496	−1	5.24 × 10^−2^	0.002
*A_23_P123193*	*ACTR3B*	ARP3 actin-related protein 3 homolog B	NM_005247	−1.1	0.002	0.007

**Table 3:** Relevant significant differentially expressed probe sets after anti-NSD1 siRNA-AT3 treatment.

Probe Set ID	Gene Symbol	Gene Name	Seq. Name	Log_2_ Fold Change	*p*-Value	*p*-Value Adjusted
A_23_P344555	*NEDD9*	neural precursor cell expressed, developmentally down-regulated 9	NM_006403	−0.05	1.13 × 10^−2^	0.0013
A_33_P3270009	*NCOA1*	nuclear receptor coactivator 1	NM_147223	−0.4	8.13 × 10^−1^	0.01
A_32_P75902	*MEIOB*	meiosis specific with OB domains	NM_152764	−0.6	8.08 × 10^−8^	0.01
A_23_P76774	*GSC*	goosecoid homeobox	NM_173849	−1.1	1.92 × 10^−2^	0.002
A_33_P3302389	*FEM1A*	fem^−1^ homolog a	NM_018708	−1.3	2.22 × 10^−10^	1.74 × 10^−6^

## Data Availability

The data presented in this study are available in the article and Supplementary Materials.

## Supplementary Materials

The following Supporting Information can be downloaded at: https://www.mdpi.com/article/10.3390/genes15091117/s1, Figure S1: Schematic exon-intron map of NSD1; Figure S2: Real-time PCR and Western blot results; Figure S3: real-time validation; Figure S4: GSEA plots relative to Apoptosis (A) and P53 Pathway (B); Figure S5: GSEA plots relative to epithelial mesenchymal transition (A) and G2M checkpoint (B); Figure S6: Enrichment plots of representative down represented biological processes in fibroblasts after siRNA-AT2 treatment by GSEA. (**A**) Plots are relative to epithelial mesenchymal transition (**B**) and G2M checkpoint; Figure S7: NSD1 AT2 loss in fibroblasts (FBs) impairs actin cytoskeleton organization and stress fiber structure. Representative images of FBs 24h post transfection with anti NSD1 siRNA-AT2 showed a morphological transition into an amoeboid phenotype and FBs not transfected with morphological displayed a typical flat and elongated structure. FBs transfected with anti NSD1 siRNA-AT1, or NSD1 siRNA-AT3 showed NSD1 isoform-AT1 or NSD1-AT3 knockdown and normal B actin expression. FBs transfected with siRNA control. (×100 magnification; blue = DAPI; red = NSD1; green = B actin. Scale bar = 50 µm). Table S1: GSEA results for AT1; Table S2: GSEA results for AT2; Table S3: GSEA results for AT3; Table S4: Characteristics of Anti-NSD1 siRNA-AT1, Anti-NSD1 siRNA-AT2, Anti-NSD1 siRNA-AT3, Anti PPIA and Anti-Cy3 siRNA Duplexes and Their Position in the NSD1 and PPIA mRNAs; Table S5: Primers of differentially expressed genes used for validation by real-time PCR.
