# Supplementary material for: Impact of NSD1 Alternative Transcripts in Actin Filament Formation and Cellular Division Pathways in Fibroblasts
Source: Genes (Basel). 2024 Aug 24;15(9):1117. doi: 10.3390/genes15091117 (PMC11431170; doi:10.3390/genes15091117)
Supplement: Supplementary file 1 [file genes-15-01117-s001.zip › genes-3110090-supplementary.pdf]

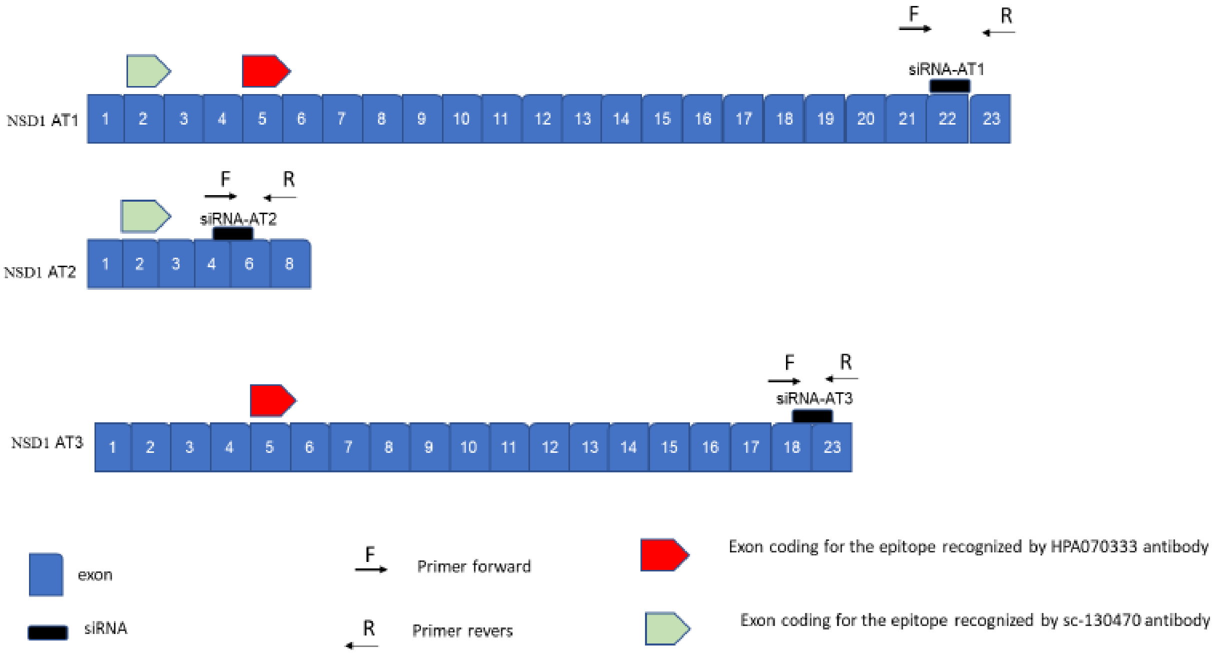

Figure. S1. Schematic exon-intron map of NSD1 showing the alternative splicing and the locations of each specific siRNA. The black arrows show primer sites for isoform gene expression analysis, Exons coding for each epitope recognized by two different NSD1 antibody are shown.

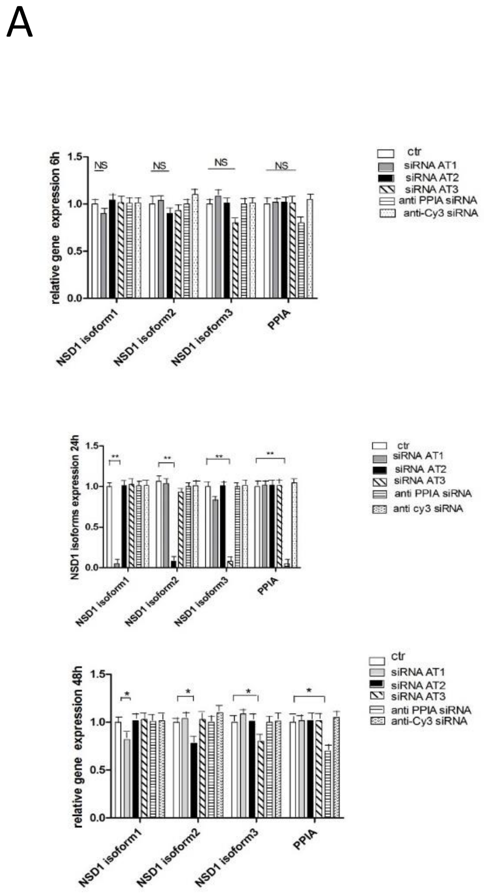

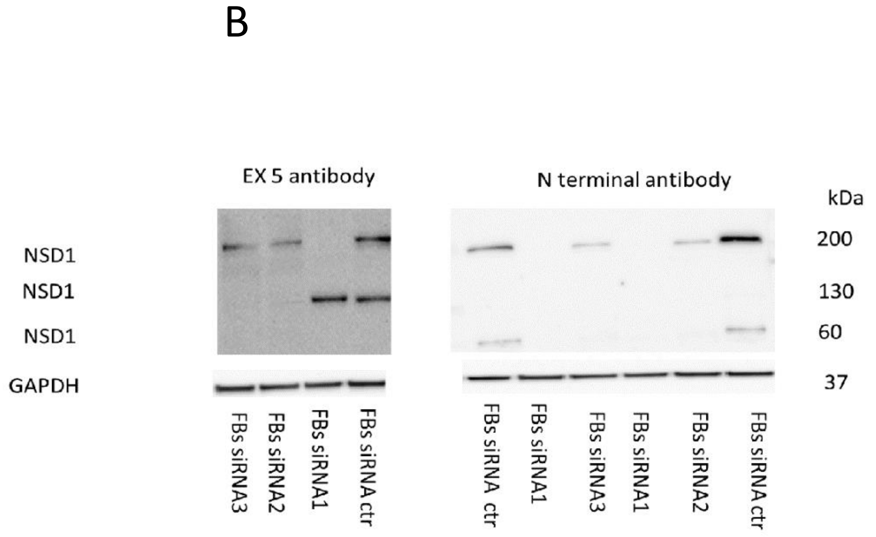

Figure. S2. Real time PCR and western blot shows Isoform-specific knock-down after specific RNA interference treatment. (**A**) Time-dependent effect of anti NSD1 siRNA-AT1, siRNA-AT2 and siRNA-AT3 on NSD1 isoforms expression as determined by real time PCR. Fibroblasts were transfected with anti NSD1 siRNA-AT1, or anti NSD1 siRNA-AT2 or anti NSD1 siRNA-AT3 or anti-Cy3 siRNA or anti PPIA siRNA and harvested at 6h(a), 24h (b), 48h (c) after transfection. NSD1 expression was significantly decreased after 24 hours from the transfection. We test b actin expression after anti b actin treatment, to confirm specific NSD1 silencing induced by Anti NSD1 siRNA-AT1, siRNA-AT2 and siRNA-AT3. Each bar reports the mean ± SD of three independent experiments. * $p < 0.05$, ** $p < 0.01$. (**B**) A representative figure of western bolts performed on all 4 FBs line was showed.Cells were untreated or transfected without NSD1 siRNA or with NSD1 siRNA-AT2 or NSD1 siRNA-AT3 and control siRNA ctr. Whole-cell extracts were prepared 24 h later and analysed for NSD1 and GAPDH levels. We used two NSD1 antibody, specific for N terminal (AT1 and AT2) and ex5 epitope of NSD1 protein (AT1 and AT3). Western blots results confirmed the presence in the fibroblasts of different NSD1 isoforms, that the two antibody used can recognize in different way, we can observed that N terminal epitope of NSD1 AT3 were not recognized by the sc-130470 and the epitope coding from exon5 recognized by HPA070333 antibody were not present in the NSD1 AT2 isoforms.

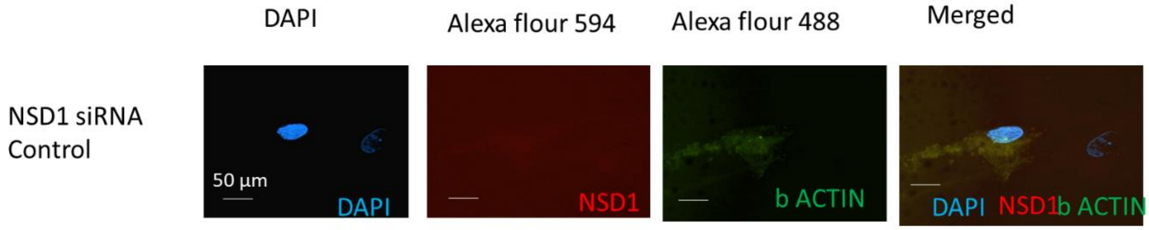

Figure. S3. Expression analysis of NSD1 isoforms in fibroblasts by immunofluorescence. After 24h of post anti-NSD1 siRNA control, DAPI was used to stain the cell nucleus to locate cells and Alexa fluor 595-labelled NSD1 antibodies for NSD1 AT2, NSD1 AT3, and NSD1 AT1, Alexa fluor 488 for b Actin (×100 magnification; blue = DAPI; red= NSD1; green= b Actin. Scale bar = 50 µm).

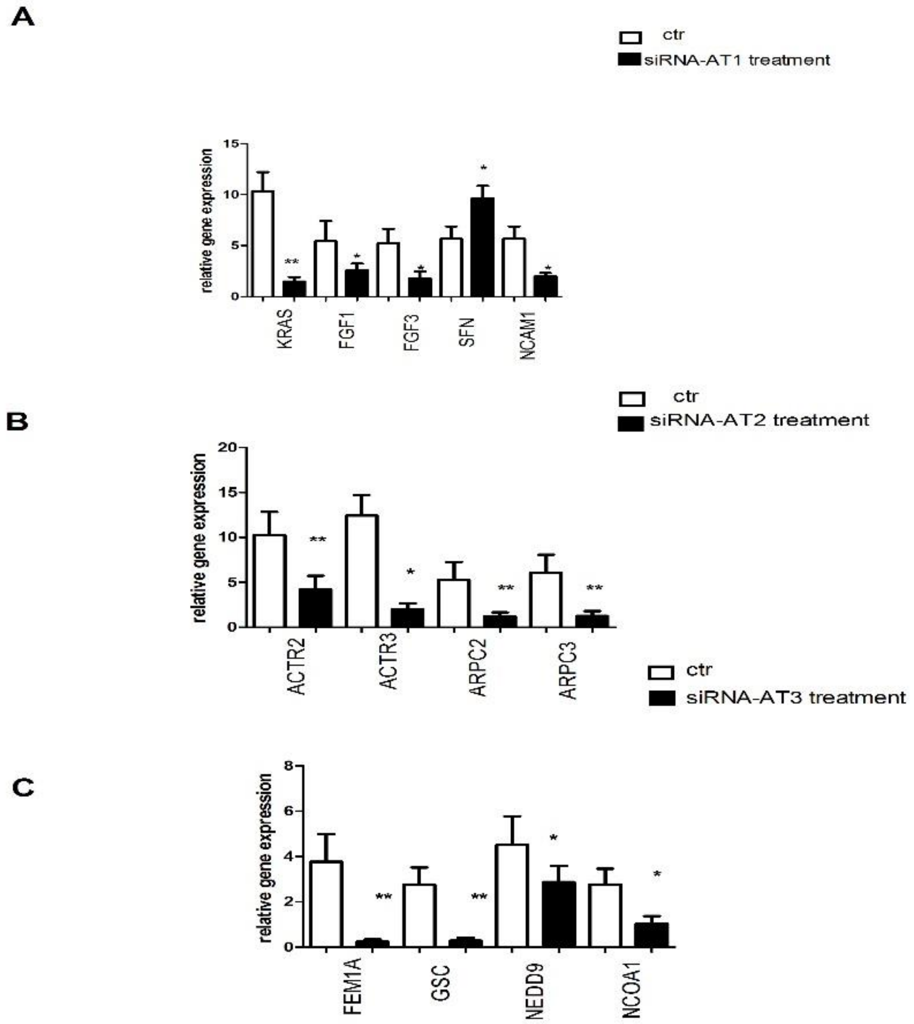

Figure S4. Real time PCR validation of differentially expressed genes. To confirm differential gene expression induced by siRNA treatment, mRNA levels of a representative gene were quantified by RT-PCR (**A**) after siRNA-AT1 treatment, (**B**) after siRNA-AT2 treatment, (**C**) after siRNA-AT3 treatment. Results were consistent with the findings obtained from the microarray analysis. Data are presented as the mean ± standard deviation. *$P<0.05$, **$P<0.01$ vs. white bars: wild type control samples; balack bars: fibroblasts after siRNA treatment.

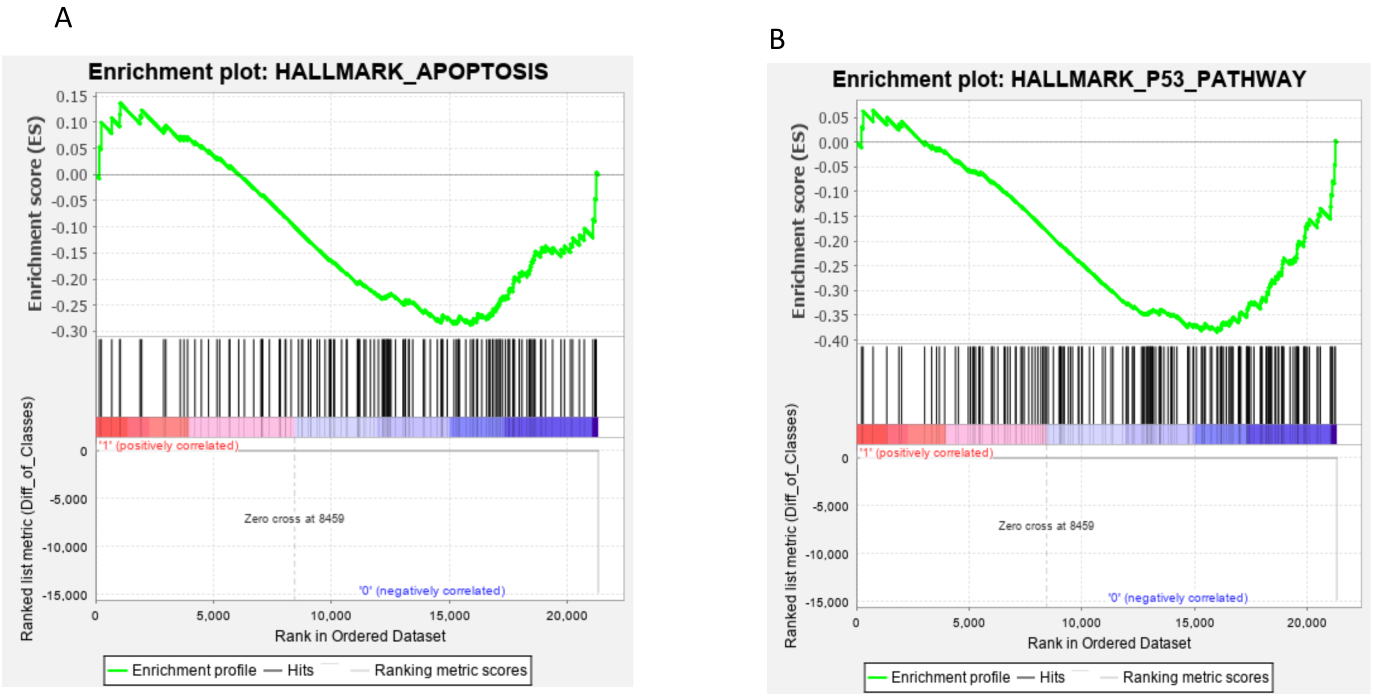

Figure. S5. Enrichment plots of representative over represented biological processes in fibroblasts after siRNA-AT2 treatment by GSEA. (**A**) Plots are relative to Apoptosis (**B**) and P53 Pathway

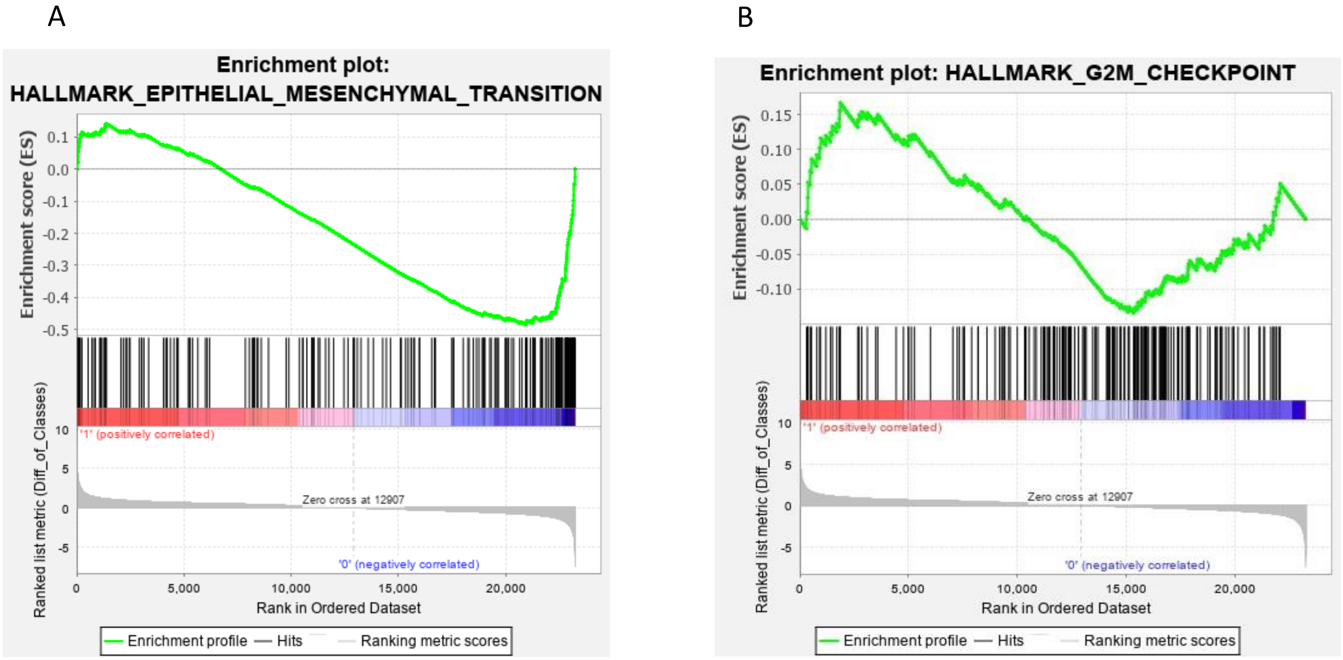

Figure. S6. Enrichment plots of representative down represented biological processes in fibroblasts after siRNA-AT2 treatment by GSEA. (**A**) Plots are relative to epithelial mesenchymal transition (**B**) and G2M checkpoint

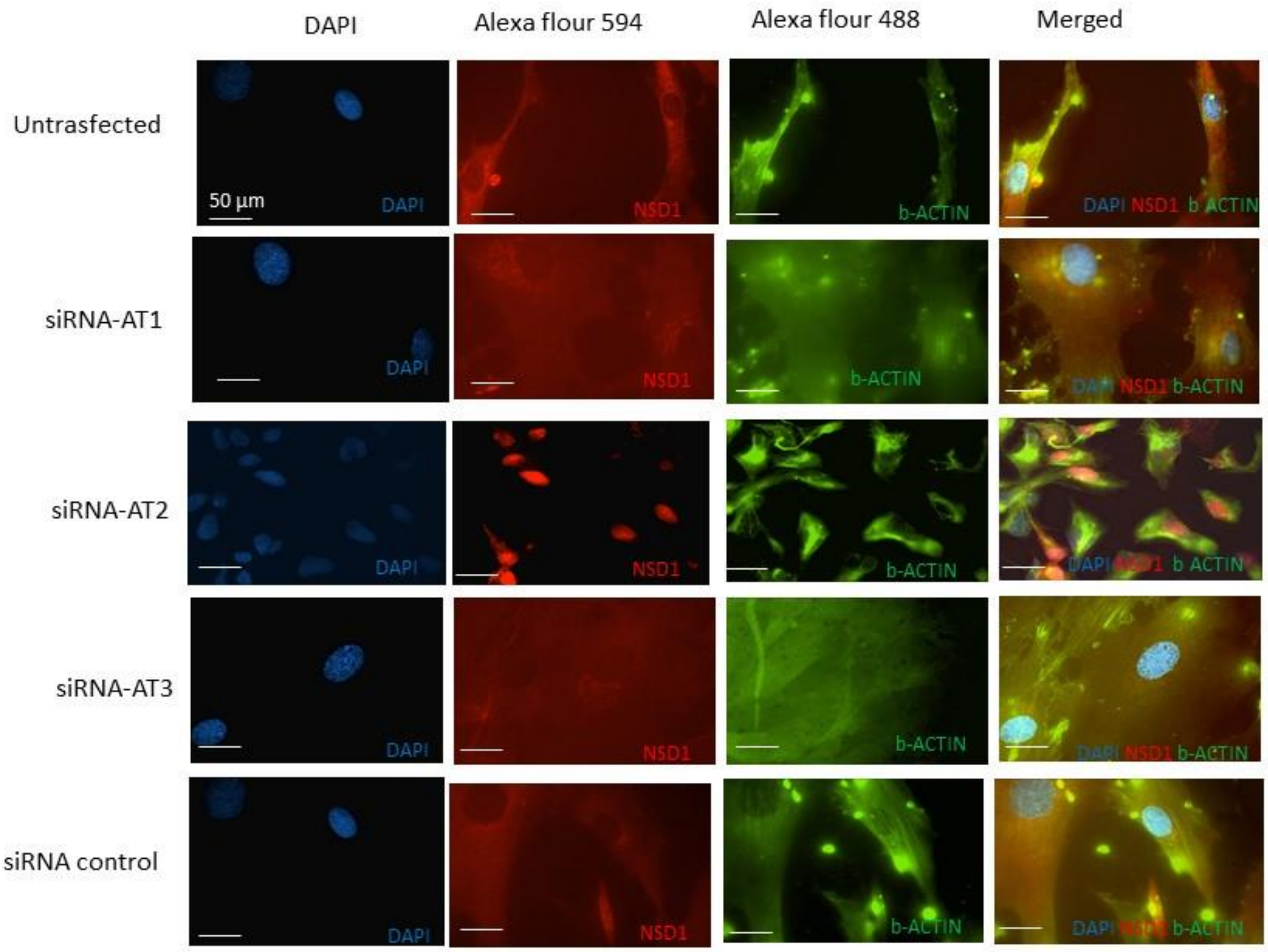

Figure. S7. NSD1 AT2 loss in fibroblasts (FBs) impairs actin cytoskeleton organization and stress fiber structure. Representative images of FBs 24h post transfection with anti NSD1 siRNA-AT2 showed a morphological transition into an amoeboid phenotype and FBs not transfected with morphological displayed a typical flat and elongated structure. FBs transfected with anti NSD1 siRNA-AT1, or NSD1 siRNA-AT3 showed NSD1 isoform-AT1 or NSD1-AT3 knockdown and normal B actin expression. FBs transfected with siRNA control. (×100 magnification; blue = DAPI; red = NSD1; green = B actin. Scale bar=50 µm).

Table S1. Differentially expressed genes untreated vs siRNA1 treated FBs

Probe set ID	Gene symbol	Gene name	2Log Fold Change	p.value
A_24_P349039	*REV1*	REV1, polymerase (DNA directed) [NM_016316]	-1.5450763	0.03421058
A_23_P115346	*ARHGAP31*	Rho GTPase activating protein 31 [NM_020754]	-1.31134589	0.024948223
A_32_P120604	*EIF2D*	eukaryotic translation initiation factor 2D [NM_006893]	-1.0086936	0.028573772
A_23_P120845	*SNX29*	sorting nexin 29 [BC029857]	-1.33134577	0.028573772
A_33_P3417950	*XBP1*	X-box binding protein 1 [NM_005080]	-1.1208981	0.026497496
A_32_P57237	*AKAP8*	A kinase (PRKA) anchor protein 8 [NM_005858]	-0.7092318	0.028192867
A_32_P783	*RPL21*	ribosomal protein L21 [NM_000982]	-0.934458	0.031606767
A_23_P146497	*RPL37A*	ribosomal protein L37a [NM_000998]	-0.90442467	0.03576752
A_33_P3383606	*PPP1R26*	protein phosphatase 1, regulatory subunit 26 [NM_014811]	-1.3393368	0.040555127
(+)E1A_r60_a22	*KCP*	kielin/chordin-like protein [NM_001135914]	0.7746044	0.03403978
A_33_P3251332	*APTR*	Alu-mediated CDKN1A/p21 transcriptional regulator [NR_038361]	1.1884073	0.03679443
A_33_P3272189	*HEATR5A*	HEAT repeat containing 5A [NM_015473]	-0.81438714	0.038003992
A_33_P3362321	*MFSD9*	major facilitator superfamily domain containing 9 [NM_032718]	-0.97848177	0.031603426
A_33_P3248602	*FHL2*	four and a half LIM domains 2 [NM_201555]	-0.9183239	0.02453978
A_24_P376787	*DUX4*	double homeobox 4 [NM_001293798]	-1.3802385	0.027942823
A_22_P00020384	*ZNF496*	zinc finger protein 496 [NM_032752]	0.99665016	0.037232194
A_23_P202860	*SYNJ2BP-COX16*	SYNJ2BP-COX16 readthrough [NM_001202547]	1.050983	0.03777906
A_24_P250499	*CYP2R1*	cytochrome P450, family 2, subfamily R, polypeptide 1 [NM_024514]	-0.91526693	0.03912857
A_24_P26554	*RPRD1B*	regulation of nuclear pre-mRNA domain containing 1B [NM_021215]	-1.7605554	0.024948223
A_24_P942030	*LYRM4*	LYR motif containing 4 [NM_020408]	-0.6188984	0.03777906
A_19_P00318404	*VAMP4*	vesicle-associated membrane protein 4 [NM_003762]	-1.2698624	0.02598378
A_33_P3379004	*LINC00989*	long intergenic non-protein coding RNA 989 [NR_038826]	-1.4128125	0.039682448

A_33_P6570282	*ZNF555*	zinc finger protein 555 [NM_152791]	1.087305	0.042693015
A_33_P3332547	*LINC00674*	long intergenic non-protein coding RNA 674 [NR_027418]	1.0966197	0.042526342
A_23_P125233	*IQCJ-SCHIP1*	IQCJ-SCHIP1 readthrough [NM_001197113]	-1.4990084	0.027942823
A_21_P0012654	*CNN1*	calponin 1, basic, smooth muscle [NM_001299]	-0.8731186	0.028189372
A_24_P291401	*ETFDH*	electron-transferring-flavoprotein dehydrogenase [NM_004453]	-2.3719945	0.034969777
A_23_P19115	*TMEM150A*	transmembrane protein 150A [NM_001031738]	-2.241376011	0.034342222
A_24_P227585	*GSPT2*	G1 to S phase transition 2 [NM_018094]	-1.1320736	0.029808238
A_33_P3268174	*GPALPP1*	GPALPP motifs containing 1 [NM_018559]	-1.1230536	0.04854581
A_24_P167984	*KBTBD6*	kelch repeat and BTB (POZ) domain containing 6 [NM_152903]	-0.6284245	0.035515364
A_23_P68942	*ATMIN*	ATM interactor [NM_015251]	-0.9464761	0.042475477
A_33_P3244818	*RPL3*	ribosomal protein L3 [NM_000967]	-1.0543827	0.04915393
A_23_P2474	*RSBN1L*	round spermatid basic protein 1-like [NM_198467]	-0.8178018	0.031603426
A_22_P00011599	*COPS7A*	COP9 signalosome subunit 7A [NM_016319]	-1.0988209	0.04881034
A_33_P3402526	*SNORD5*	small nucleolar RNA, C/D box 5 [NR_003033]	-1.33097276	0.040555127
A_32_P104746	*CSGALNACT2*	chondroitin sulfate N-acetylgalactosaminyltransferase 2 [NM_018590]	-0.8308151	0.033989865
A_33_P3221353	*ZFYVE28*	zinc finger, FYVE domain containing 28 [NM_020972]	1.5122744	0.04418471
A_33_P3290573	*VWA8*	von Willebrand factor A domain containing 8 [NM_001009814]	-1.0725269	0.032580614
A_21_P0010951	*FAM46A*	family with sequence similarity 46, member A [NM_017633]	-1.1590922	0.03669668
(+)E1A_r60_a135	*PDCD2*	programmed cell death 2 [NM_002598]	1.0266968	0.033095628
A_23_P153441	*CC2D1A*	coiled-coil and C2 domain containing 1A [ENST00000589138]	-0.9536866	0.03947304
A_23_P304511	*HOOK2*	hook microtubule-tethering protein 2 [NM_013312]	-1.1815007	0.043984637
A_23_P377819	*ZNF397*	zinc finger protein 397 [NM_032347]	1.8723375	0.03170058
A_23_P40025	*SRSF5*	serine/arginine-rich splicing factor 5 [NM_001039465]	-0.9367549	0.027751032
A_23_P94795	*DAZAP2*	DAZ associated protein 2 [NM_014764]	-1.1206213	0.048163485

A_33_P3299525	*TEAD4*	TEA domain family member 4 [NM_003213]	-1.672665	0.02453978
A_23_P94338	*KCTD21*	potassium channel tetramerization domain containing 21 [NM_001029859]	-0.8662739	0.028546385
A_33_P6817399	*ENPP2*	ectonucleotide pyrophosphatase/phosphodiesterase 2 (ENPP2) [NM_006209]	-0.7210753	0.049715545
A_23_P99771	*STARD3*	StAR-related lipid transfer [NM_001165937]	-1.6630183	0.028573772
A_24_P124973	*PNMA1*	paraneoplastic Ma antigen 1 [NM_006029]	-2.2059531	0.04208062
A_23_P91619	*NDNL2*	necdin-like 2 [NM_138704]	1.408341	0.046535324
A_32_P72940	*MIF*	macrophage migration inhibitory factor [NM_002415]	-0.55798644	0.02598378
A_33_P3777584	*RPL35*	ribosomal protein L35 [NM_007209]	-0.65366983	0.042994436
A_23_P89249	*SERAC1*	serine active site containing 1 [NM_032861]	-0.7336428	0.026497496
A_22_P00003766	*ERBB2*	erb-b2 receptor tyrosine kinase 2 [NM_001005862]	-1.1099892	0.027942823
A_23_P45970	*ZNF593*	zinc finger protein 593 (ZNF593) [NM_015871]	-1.5520568	0.04610539
A_23_P419795	*C1orf109*	chromosome 1 open reading frame 109 [NM_017850]	-0.95614904	0.03403978
A_32_P196115	*SBF2*	SET binding factor 2 [NM_030962]	-0.93105125	0.027942823
A_32_P131367	*ZSCAN25*	zinc finger and SCAN domain containing 25 [NM_145115]	-0.84376234	0.024948223
A_23_P38446	*RNF13*	ring finger protein 13 [NM_007282]	-1.2865602	0.04895638
A_21_P0013792	*TNFAIP1*	tumor necrosis factor, alpha-induced protein 1 [NM_021137]	-1.4750215	0.036667652
A_22_P00003974	*CCNG2*	cyclin G2 [NM_004354]	-1.1755918	0.029808238
A_23_P9056	*SEC23IP*	SEC23 interacting protein [NM_007190]	1.154816	0.043343782
A_23_P78563	*RB1CC1*	RB1-inducible coiled-coil 1 [NM_014781]	-1.678393302	0.0442831
A_33_P3213419	*UBL5*	ubiquitin-like 5 (UBL5) [NM_024292]	1.2320868	0.04733213
A_23_P46429	*RPS7*	ribosomal protein S7 [NM_001011]	-1.2012198	0.040741067
(+)E1A_r60_1	*CYR61*	cysteine-rich, angiogenic inducer, 61 [NM_001554]	-0.6091768	0.046256438
A_21_P0009753	*SGCD*	sarcoglycan, delta (35kDa dystrophin-associated glycoprotein) [NM_172244]	-1.0644426	0.028573772

A_23_P132444	*DSCR3*	Down syndrome critical region 3 [NM_006052]	-2.1577806	0.035935212
A_33_P3231981	*TCEA1*	transcription elongation factor A [NM_006756]	-1.494643	0.027751032
A_19_P00318152	*PARK7*	parkinson protein 7 [NM_007262]	-2.7533734	0.0380295
A_19_P00808120	*GEMIN5*	gem (nuclear organelle) associated protein 5 [NM_015465]	-1.26476	0.0460751
A_24_P56388	*FAM200B*	family with sequence similarity 200, member B [NM_001145191]	-0.7633934	0.03432043
(+)E1A_r60_a97	*HIF1A*	hypoxia inducible factor 1, alpha subunit [NM_181054]	-1.8560724	0.048760455
A_23_P54929	*LAIR1*	leukocyte-associated immunoglobulin-like receptor 1 (LAIR1) [NM_002287]	-1.0920852	0.040091004
A_21_P0007550	*LYRM1*	LYR motif containing 1 [NM_020424]	-0.71983266	0.046325136
A_23_P359655	*ESF1*	ESF1, nucleolar pre-rRNA processing protein [NM_016649]	1.1503044	0.048506733
A_23_P15123	*ZNF664*	zinc finger protein 664 (ZNF664) [NM_152437]	-1.2579515	0.037339266
A_23_P100220	*UBFD1*	ubiquitin family domain containing 1 [NM_019116]	0.98249054	0.043439556
A_23_P413788	*ESRP2*	epithelial splicing regulatory protein 2 [NM_024939]	-0.9314496	0.030362261
A_23_P218706	*FUT11*	fucosyltransferase 11 (alpha (1,3) fucosyltransferase) [NM_173540]	-1.178092	0.03136619
A_33_P3294449	*ZNF343*	zinc finger protein 343 (ZNF343) [NM_024325]	-0.8645179	0.032155342
A_33_P3403418	*DCAF6*	DDB1 and CUL4 associated factor 6 [NM_001017977]	3.7845683	0.024948223
A_33_P3225298	*CTNNA1*	catenin (cadherin-associated protein), alpha 1, 102kDa [NM_001903]	-0.73232985	0.045638688
A_33_P3267208	*XKR9*	XK, Kell blood group complex subunit-related family, member 9 [NM_001287258]	-0.86554617	0.041527454
A_32_P144920	*CSNK1G1*	casein kinase 1, gamma 1 [NM_022048]	-0.6491004	0.04639083
A_23_P120316	*NECAP1*	NECAP endocytosis associated 1 [NM_015509]	-1.1219978	0.03297842
A_23_P73835	*MTHFD2*	methylenetetrahydrofolate dehydrogenase (NADP+ dependent) 2, [NM_006636]	0.6932458	0.045874957
A_23_P122001	*MOSPD1*	motile sperm domain containing 1 [NM_019556]	-0.46894664	0.031083314
A_24_P191588	*MSH3*	mutS homolog 3 [NM_002439]	-0.83464664	0.031377234
A_23_P29124	*ALAS1*	5'-aminolevulinate synthase 1 [NM_000688]	-1.5916013	0.024948223

A_33_P3279431	*GP1BB*	glycoprotein Ib (platelet), beta polypeptide [NM_000407]	-1.1302638	0.038309526
A_24_P134789	*GOSR1*	golgi SNAP receptor complex member 1 [NM_001007024]	-0.70789903	0.038904693
A_21_P0010263	*KRTAP10-10*	keratin associated protein 10-10 [NM_181688]	-1.5227883	0.049327243
A_23_P30020	*YPEL5*	yippee-like 5 (Drosophila) [NM_001127401]	-1.6707284	0.049715545
A_23_P24633	*PLA2G12A*	phospholipase A2, group XIIA [NM_030821]	-1.3069816	0.036667652
A_23_P131435	*THYN1*	thymocyte nuclear protein 1 [NM_199297]	-1.4838992	0.02737376
A_23_P53018	*CD302*	CD302 molecule [NM_014880]	-1.2864618	0.03912857
A_23_P27649	*HRASLS5*	HRAS-like suppressor family, member 5 [NM_054108]	-0.7430994	0.0335366
A_32_P126311	*ZNF433*	zinc finger protein 433 [NM_001080411]	-0.9255164	0.045593053
A_21_P0014822	*SLC38A9*	solute carrier family 38 [NM_173514]	-1.101095189	0.0335366
A_23_P40952	*ATP6V1G1*	ATPase, H+ transporting, lysosomal 13kDa, V1 subunit G1 [NM_004888]	-0.75080836	0.030293303
A_23_P170901	*RAF1*	Raf-1 proto-oncogene, serine/threonine kinase [NM_002880]	-0.9878893	0.0322713
A_23_P105313	*PACRG*	PARK2 co-regulated [NM_152410]	-0.538684	0.040911164
A_24_P396702	*EIF2B1*	eukaryotic translation initiation factor 2B, subunit 1 alpha [NM_001414]	-0.82067746	0.030993799
A_24_P167338	*CD302*	CD302 molecule [NM_014880]	-0.7476463	0.04644307
ERCC-00148_99	*RAB30*	RAB30, member RAS oncogene family [NM_014488]	1.3554621	0.03590667
A_23_P63010	*PSMD10*	proteasome (prosome, macropain) 26S subunit, non-ATPase, 10 [ENST00000338548]	-1.2887968	0.028573772
A_23_P37892	*CERS2*	ceramide synthase 2 [NM_181746]	-1.0144821	0.043911606
A_23_P259442	*GPT2*	glutamic pyruvate transaminase 2 [NM_133443]	1.4596268	0.042907383
A_23_P55917	*CPE*	carboxypeptidase E [NM_001873]	-0.56497	0.028573772
A_21_P0009234	*SYT3*	synaptotagmin III [NM_032298]	-0.8098888	0.03808641
A_23_P398275	*MGST3*	microsomal glutathione S-transferase 3 [NM_004528]	-4.2532845	0.041929405
A_24_P927474	*TP53I3*	tumor protein p53 inducible protein 3 [NM_004881]	1.2475635	0.032155342
A_33_P3351765	*ZNF185*	zinc finger protein 185 [NM_001178106]	-1.322678	0.030915326

A_23_P201445	*KLHDC10*	kelch domain containing 10 [NM_014997]	-2.3405216	0.046205126
A_23_P414519	*DESI2*	desumoylating isopeptidase 2 [NM_016076]	-1.1189889	0.03403978
A_24_P195794	*NRN1*	neuritin 1 [NM_016588]	-3.8293924	0.029963305
A_23_P359738	*TRMT10A*	tRNA methyltransferase 10 homolog A [NM_152292]	-0.6697643	0.04793344
A_23_P114405	*EPC2*	enhancer of polycomb homolog 2 [NM_015630]	-1.6400964	0.03856101
A_24_P185036	*MORF4L2*	mortality factor 4 like 2 [NM_012286]	-1.4207518	0.042850092
A_33_P3286278	*CMTR2*	cap methyltransferase 2[NM_018348]	-0.89000213	0.03922557
A_23_P422724	*GRN*	granulin [NM_002087]	-1.0413703	0.037240293
A_33_P3271725	*PPIC*	peptidylprolyl isomerase C [NM_000943]	-1.4560999	0.035515364
A_23_P98022	*CCDC33*	coiled-coil domain containing 33 [NM_025055]	-1.2909067	0.049327243
A_33_P3404470	*SIRT1*	sirtuin 1 [NM_012238]	-0.88696814	0.034614928
A_23_P121533	*UNC93B1*	unc-93 homolog B1 (UNC93B1) [NM_030930]	-1.6661814	0.04820357
A_33_P3238543	*SPON2*	Spondin 2, extracellular matrix protein (SPON2)[NM_012445]	-0.46923253	0.03808641
A_33_P3395743	*MAFG-AS1*	MAFG antisense RNA 1 (head to head) (MAFG-AS1), long non-coding RNA [NR_015454]	-1.5880268	0.035673555
A_23_P30474	*VWA1*	Von Willebrand factor A domain containing 1 (VWA1) [NM_022834]	-1.616615	0.034334898
A_23_P77401	*WDR70*	WD repeat domain 70 (WDR70) [NM_018034]	3.4904237	0.03683928
A_23_P88046	*CPPED1*	calcineurin-like phosphoesterase domain containing 1 (CPPED1) [NM_018340]	-1.0894947	0.03912857
A_33_P3628481	*CARKD*	Carbohydrate kinase domain containing (CARKD) [NM_018210]	-1.2512784	0.0442831
A_23_P370142	*MGC27345*	Uncharacterized protein MGC27345 (MGC27345)[NR_046216]	-0.9990175	0.038727812
A_23_P49539	*PAFAH1B2*	Platelet-activating factor acetylhydrolase 1b, catalytic subunit 2 (PAFAH1B2) [NM_002572]	-1.0763955	0.038287252
A_33_P3225690	*BAHCC1*	BAH domain and coiled-coil containing 1 (BAHCC1) [NM_001291324]	-0.62797976	0.047708835
A_24_P12435	*ZNF516*	zinc finger protein 516 (ZNF516) [NM_014643]	-0.88622373	0.036667652

A_33_P3228762	*NCOA7*	Nuclear receptor coactivator 7 (NCOA7) [NM_181782]	-0.7333505	0.04434183
A_32_P110243	*SLC25A36*	Solute carrier family 25 (pyrimidine nucleotide carrier), member 36 (SLC25A36) [NM_001104647]	-0.6671869	0.03177512
A_23_P24215	*RPS20P27*	Ribosomal protein S20 pseudogene 27[BC071734]	-0.48976088	0.044186465
A_24_P294842	*TBC1D12*	TBC1 domain family, member 12 (TBC1D12) [NM_015188]	-0.8389652	0.03136619
A_32_P74409	*ATXN1*	Ataxin 1 (ATXN1), transcript variant 1, mRNA [NM_000332]	-1.1138685	0.03273351
A_24_P55250	*C11orf96*	chromosome 11 open reading frame 96 (C11orf96) [NM_001145033]	-0.7326619	0.043335356
A_23_P111132	*HDDC2*	HD domain containing 2 (HDDC2) [NM_016063]	-1.5787908	0.026808027
A_23_P105276	*HSPA1A*	heat shock 70kDa protein 1A (HSPA1A) [NM_005345]	-2.0961125	0.034210585
A_33_P3376644	*ZNF84*	zinc finger protein 84 (ZNF84) [NM_003428]	-2.3383312	0.02919588
A_23_P324994	*SENP6*	SUMO1/sentrin specific peptidase 6 (SENP6) [NM_015571]	-1.0569596	0.03297842
A_33_P3223923	*KLHL7*	kelch-like family member 7 (KLHL7)[NM_018846]	-0.33204338	0.04639083
A_23_P11543	*PDIA3*	protein disulfide isomerase family A, member 3 (PDIA3) [NM_005313]	-0.7189888	0.03273351
A_33_P3329098	*FUCA1*	fucosidase, alpha-L- 1, tissue (FUCA1) [NM_000147]	-1.6313813	0.032155342
A_23_P369456	*WTAP*	Wilms tumor 1 associated protein (WTAP) [NM_004906]	-1.6977592	0.03403978
A_23_P205531	*SYS1*	Sys1 golgi trafficking protein (SYS1) [NM_033542]	-1.5707605	0.049603622
A_21_P0000366	*RNASE4*	ribonuclease, RNase A family, 4 (RNASE4) [NM_001282192]	-1.8152766	0.027942823
A_33_P3314212	*SNORD11*	small nucleolar RNA, C/D box 11 (SNORD11) [NR_003031]	-0.29128593	0.0419793
A_23_P112825	*PNPT1*	polyribonucleotide nucleotidyltransferase 1 (PNPT1)[NM_033109]	-1.972919	0.046129715
A_33_P3331366	*LCMT1*	leucine carboxyl methyltransferase 1 (LCMT1) [NM_016309]	1.757093	0.0442831
A_33_P3221868	*TRIM25*	tripartite motif containing 25 (TRIM25) [NM_005082]	-1.0563985	0.038180117
A_32_P112493	*TMEM216*	transmembrane protein 216 (TMEM216) [NM_001173990]	0.9701724	0.045638688
A_32_P196047	*PKDCC*	protein kinase domain containing, cytoplasmic (PKDCC) [NM_138370]	-0.6271581	0.044364784
A_32_P112623	*DPY19L4*	dpy-19-like 4 (DPY19L4) [NM_181787]	-0.78145957	0.03811336

A_21_P0001482	*FAM27E2*	family with sequence similarity 27, member E2 (FAM27E2)[NR_103714]	-0.78948617	0.024948223
A_33_P3305974	*GSTP1*	glutathione S-transferase pi 1 (GSTP1) [NM_000852]	-2.9554944	0.03428924
A_24_P355876	*SNAP23*	synaptosomal-associated protein, 23kDa (SNAP23), transcript variant 1, mRNA [NM_003825]	-1.0185579	0.029541323
A_33_P3273000	*TMBIM6*	transmembrane BAX inhibitor motif containing 6 (TMBIM6), transcript variant 1, mRNA [NM_003217]	1.2178541	0.0376762
A_33_P3247255	*PTK2B*	cDNA FLJ46514 fis, clone THYMU3032798, highly similar to Focal adhesion kinase 2 (EC 2.7.1.112). [AK128371]	-0.6351998	0.035186797
A_33_P3359748	*TMCO3*	transmembrane and coiled-coil domains 3 (TMCO3), mRNA [NM_017905]	-1.2518985	0.02622961
A_32_P42054	*SARS*	seryl-tRNA synthetase [Source:HGNC Symbol;Acc:HGNC:10537] [ENST00000369923]	-0.5613925	0.046464663
A_33_P3419733	*DND1*	DND microRNA-mediated repression inhibitor 1 (DND1), mRNA [NM_194249]	1.6310196	0.031091278
A_33_P3289835	*DNAJC5*	DnaJ (Hsp40) homolog, subfamily C, member 5 (DNAJC5), mRNA [NM_025219]	-1.298963	0.042907383
(+)E1A_r60_n11	*OSBPL9*	oxysterol binding protein-like 9 [Source:HGNC Symbol;Acc:HGNC:16386] [ENST00000531819]	-1.0045393	0.040161368
A_23_P106299	*DHX36*	DEAH (Asp-Glu-Ala-His) box polypeptide 36 (DHX36), transcript variant 1, mRNA [NM_020865]	-0.7031659	0.034342222
A_33_P3312301	*SERF2*	small EDRK-rich factor 2 (SERF2), transcript variant 3, mRNA [NM_001018108]	-0.7775632	0.027942823
A_23_P109333	*CIT*	citron rho-interacting serine/threonine kinase (CIT), transcript variant 1, mRNA [NM_001206999]	1.8588545	0.03233256
A_23_P120594	*C21orf33*	chromosome 21 open reading frame 33 (C21orf33), transcript variant 1, mRNA [NM_004649]	-0.68432635	0.035186797
A_22_P00018931	*ACSS1*	acyl-CoA synthetase short-chain family member 1 (ACSS1), transcript variant 1, mRNA [NM_032501]	-0.5834114	0.04237416
A_24_P261052	*lnc-C8A-1*	LNCipedia lincRNA (lnc-C8A-1), lincRNA [lnc-C8A-1:1]	0.6722955	0.034686882

A_33_P3224680	*MTMR9*	myotubularin related protein 9 (MTMR9), [NM_015458]	-0.46119902	0.046789277
A_23_P6771	*TRMT13*	tRNA methyltransferase 13 homolog (S. cerevisiae) [ENST00000370143]	-2.7707474	0.027751032
A_23_P128940	*LMCD1*	LIM and cysteine-rich domains 1 (LMCD1) [NM_014583]	1.016168	0.028573772
A_23_P337790	*VCPKMT*	valosin containing protein lysine (K) methyltransferase (VCPKMT) [NM_024558]	-1.1641183	0.046464663
A_24_P188377	*SHPRH*	SNF2 histone linker PHD RING helicase, E3 ubiquitin protein ligase (SHPRH) [NM_001042683]	-1.2032222	0.04325316
A_33_P3322864	*CD55*	CD55 molecule, decay accelerating factor for complement (Cromer blood group) (CD55) [NM_000574]	-3.9278066	0.03970365
A_33_P3310430	*HES6*	hes family bHLH transcription factor 6 (HES6) [NM_018645]	-1.9735352	0.034210585
A_33_P3238196	*FAM86B2*	Family with sequence similarity 86, member B2 (FAM86B2)[NM_001137610]	-1.6107229	0.02453978
A_24_P44931	*ZNF33A*	zinc finger protein 33A (ZNF33A) [NM_001278171]	-1.7469429	0.027751032
A_23_P254415	*MPND*	MPN domain containing (MPND) [NM_032868]	1.1139222	0.046624914
A_33_P3357445	*RPSAP58*	ribosomal protein SA pseudogene 58 (RPSAP58) [NR_003662]	-1.3041537	0.04284872
A_32_P44568	*TDG*	thymine-DNA glycosylase (TDG), mRNA [NM_003211]	-1.2424641	0.03223675
A_19_P00811264	*LDHA*	lactate dehydrogenase A (LDHA), transcript variant 1, mRNA [NM_005566]	-0.7350086	0.027942823
A_23_P37484	*lnc-TSHZ1-1*	LNCipedia lincRNA (lnc-TSHZ1-1), lincRNA [lnc-TSHZ1-1:1]	-0.9577904	0.029808238
A_33_P3321657	*CHSY1*	chondroitin sulfate synthase 1 (CHSY1) [NM_014918]	-1.4695343	0.043335356
A_23_P159255	*HSPG2*	heparan sulfate proteoglycan 2 (HSPG2)[NM_001291860]	-1.180403	0.03808641
A_24_P122403	*PTPRM*	protein tyrosine phosphatase, receptor type, M (PTPRM) [NM_002845]	-1.3277766	0.044785164
A_22_P00006006	*TCEB3*	transcription elongation factor B (SIII), polypeptide 3 (110kDa, elongin A) (TCEB3), [NM_003198]	-1.167784	0.045316998
A_22_P00020312	*LOC283332*	uncharacterized LOC283332 (LOC283332), long non-coding RNA [NR_026948]	-1.0257123	0.03849178
A_23_P53736	*lnc-FKBP2-1*	Q2AC61_9BURK (Q2AC61) HrpW, partial (3%) [THC2716184]	-1.3160713	0.032261204

A_23_P156209	*FBXO21*	F-box protein 21 (FBXO21)[NM_033624]	-0.7590576	0.034210585
A_24_P318593	*ZNF622*	zinc finger protein 622 (ZNF622)[NM_033414]	1.5864384	0.031569958
A_19_P00321890	*SCRN2*	secernin 2 (SCRN2) [NM_138355]	-0.58171654	0.04542028
A_24_P270769	*LINC00894*	clone 161455-2-3 B cell expressed mRNA from chromosome X. [U66048]	-0.6983776	0.04976317
A_24_P751074	*VPS35*	vacuolar protein sorting 35 homolog (S. cerevisiae) (VPS35) [NM_018206]	-0.4643099	0.024948223
A_24_P273143	*ETS1*	v-ets avian erythroblastosis virus E26 oncogene homolog 1 (ETS1) [NM_005238]	-0.72160625	0.03880133
A_23_P163639	*LINC00152*	long intergenic non-protein coding RNA 152 (LINC00152), transcript variant 1, long non-coding RNA [NR_024204]	0.5937645	0.024948223
A_23_P127948	*ANKRD11*	ankyrin repeat domain 11 (ANKRD11) [NM_013275]	-0.76861405	0.048182435
A_23_P121196	*ADM*	adrenomedullin (ADM) [NM_001124]	-0.97104186	0.033091284
A_21_P0003476	*TMEM43*	transmembrane protein 43 (TMEM43) [NM_024334]	-0.52122974	0.028573772
A_21_P0000289	*XPC*	xeroderma pigmentosum, complementation group C (XPC)[NM_004628]	-1.3997946	0.04050197
A_32_P195401	*SNORD45A*	small nucleolar RNA, C/D box 45A (SNORD45A), small nucleolar RNA [NR_002749]	-1.5569849	0.031377234
A_23_P126486	*FAM117B*	family with sequence similarity 117, member B (FAM117B), mRNA [NM_173511]	2.6115446	0.030915326
A_24_P345822	*CROCCP2*	ciliary rootlet coiled-coil, rootletin pseudogene 2 (CROCCP2), non-coding RNA [NR_026752]	-1.5532935	0.030829152
A_24_P288954	*TFG*	TRK-fused gene (TFG), transcript variant 1, mRNA [NM_006070]	2.2321372	0.031091278
A_21_P0012616	*SRRD*	SRR1 domain containing (SRRD), mRNA [NM_001013694]	-1.6241874	0.031377234
A_23_P58877	*XLOC_l2_010854*	BROAD Institute lincRNA (XLOC_l2_010854), lincRNA [TCONS_l2_00020780]	-1.3134468	0.04800724
A_23_P127233	*GOPC*	golgi-associated PDZ and coiled-coil motif containing (GOPC), transcript variant 1, mRNA [NM_020399]	-0.80346274	0.039863296
A_23_P19322	*SMNDC1*	survival motor neuron domain containing 1 (SMNDC1), mRNA [NM_005871]	-0.6979828	0.04393096

A_33_P3274930	*SAYSD1*	SAYSVFN motif domain containing 1 (SAYSD1), mRNA [NM_018322]	0.8359247	0.031569958
A_21_P0000213	*lnc-RAB28-4*	LNCipedia lincRNA (lnc-RAB28-4), lincRNA [lnc-RAB28-4:1]	-0.80096906	0.03428924
A_33_P3353030	*SNORD42B*	small nucleolar RNA, C/D box 42B (SNORD42B), small nucleolar RNA [NR_000013]	-0.9435713	0.03436134
A_23_P127175	*UCN*	urocortin (UCN), mRNA [NM_003353]	-1.841545	0.028573772
A_21_P0000120	*SAR1A*	secretion associated, Ras related GTPase 1A (SAR1A) [NM_020150]	0.8390791	0.044452492
A_19_P00317759	*KCNJ18*	potassium channel, inwardly rectifying subfamily J, member 18 (KCNJ18) [NM_001194958]	2.396163	0.033692434
A_23_P310911	*lnc-NR5A2-1*	clone HA_003012 unknown mRNA [EU250746]	-0.35253462	0.04041675
A_23_P113803	*BLMH*	bleomycin hydrolase (BLMH)[NM_000386]	-0.47345284	0.04958916
A_23_P215634	*KATNA1*	katanin p60 (ATPase containing) subunit A 1 (KATNA1) [NM_007044]	-3.1888762	0.03032242
A_33_P3275668	*IGFBP3*	insulin-like growth factor binding protein 3 (IGFBP3) [NM_001013398]	2.2521062	0.03808641
A_23_P160869	*BAG6*	BCL2-associated athanogene 6 (BAG6) [NM_004639]	-1.3225769	0.038230915
A_23_P127367	*LRIG2*	leucine-rich repeats and immunoglobulin-like domains 2 (LRIG2)[NM_014813]	-1.1003768	0.034210585
A_24_P406714	*POLD4*	polymerase (DNA-directed), delta 4, accessory subunit (POLD4)[NM_021173]	-2.6369576	0.028546385
A_22_P00003944	*MLLT10*	myeloid/lymphoid or mixed-lineage leukemia (trithorax homolog, Drosophila); (MLLT10) [NM_004641]	-0.34639782	0.03257772
A_23_P203888	*RPS25*	ribosomal protein S25 (RPS25) [NM_001028]	-1.4252169	0.03138659
A_23_P24104	*MMP19*	matrix metallopeptidase 19 (MMP19) [NM_002429]	-1.0708293	0.043335356
A_23_P257417	*PLAU*	plasminogen activator, urokinase (PLAU) [NM_002658]	0.8470447	0.029088594
A_33_P3238461	*CLHC1*	clathrin heavy chain linker domain containing 1 (CLHC1) [NM_152385]	-0.61888236	0.027751032
A_23_P12173	*SF3B3*	splicing factor 3b, subunit 3, 130kDa (SF3B3) [NM_012426]	4.5903025	0.030915326
A_33_P3420167	*CRTC2*	CREB regulated transcription coactivator 2 (CRTC2) [NM_181715]	-1.1049178	0.0453208
A_22_P00002543	*DNAJB2*	DnaJ (Hsp40) homolog, subfamily B, member 2 (DNAJB2) [NM_006736]	0.61712575	0.04375756

A_33_P3267612	*lnc-C17orf63-1*	_Pan1 cDNA clone IMAGE:2229110 3' similar to TR:Q61382 Q61382 TNF RECEPTOR ASSOCIATED FACTOR 4 ; [AI632035]	-1.1353787	0.03126305
A_33_P3405043	*MIR143HG*	MIR143 host gene (non-protein coding) (MIR143HG) [NR_105059]	-1.0887897	0.04690382
A_23_P138514	*CIRBP*	cold inducible RNA binding protein [Source:HGNC Symbol;Acc:HGNC:1982] [ENST00000621399]	2.0505276	0.02598378
A_23_P50942	*COMMD3*	COMM domain containing 3 (COMMD3) [NM_012071]	-1.1034833	0.04159686
A_23_P50907	*RAB3GAP1*	RAB3 GTPase activating protein subunit 1 (catalytic) (RAB3GAP1) [NM_012233]	-0.4975628	0.046256438
A_23_P130194	*ITGAV*	integrin, alpha V (ITGAV) [NM_002210]	-2.0388637	0.031569958
A_33_P3236646	*PYCR1*	pyrroline-5-carboxylate reductase 1 (PYCR1) [NM_006907]	-1.0218153	0.043512966
A_24_P273799	*MIA3*	melanoma inhibitory activity family, member 3 (MIA3) [NM_198551]	-0.8062556	0.02737376
A_21_P0014389	*ZNF641*	zinc finger protein 641 (ZNF641) [NM_152320]	-1.2542503	0.026440267
A_23_P311201	*ACTR3*	ARP3 actin-related protein 3 homolog (yeast) (ACTR3) [NM_005721]	-1.1392481	0.031430684
A_24_P67534	*SRSF10*	serine/arginine-rich splicing factor 10 (SRSF10) [NM_054016]	-0.45433116	0.030293303
A_22_P00008937	*SMIM10*	small integral membrane protein 10 (SMIM10) [NM_001163438]	-1.2193161	0.034210585
A_23_P66637	*C12orf80*	chromosome 12 open reading frame 80 (C12orf80) [NM_001242696]	-1.0110378	0.04360087
A_23_P330908	*SGCA*	sarcoglycan, alpha (50kDa dystrophin-associated glycoprotein) (SGCA) [NM_000023]	-0.545748	0.049493678
A_23_P134744	*DERL1*	derlin 1 (DERL1) [NM_024295]	1.0553466	0.041831266
A_23_P141974	*RNF122*	ring finger protein 122 (RNF122) [NM_024787]	1.4006957	0.024948223
A_24_P303097	*TPM4*	tropomyosin 4 (TPM4), transcript variant Tpm4.2 [NM_003290]	-3.907391	0.024948223
A_32_P26969	*SNX25*	sorting nexin 25 (SNX25) [NM_031953]	-1.1068553	0.028546385
A_23_P256735	*ERICH1*	glutamate-rich 1 (ERICH1) [NM_207332]	-2.8042421	0.029808238
A_21_P0010415	*CPQ*	carboxypeptidase Q (CPQ) [NM_016134]	-0.84673595	0.049758088
A_23_P1492	*lnc-APOL1-1*	LNCipedia lincRNA (lnc-APOL1-1), lincRNA [lnc-APOL1-1:1]	-1.5289882	0.0442831

A_22_P00018823	*AVPI1*	arginine vasopressin-induced 1 (AVPI1)[NM_021732]	-0.78264993	0.03977008
A_21_P0010580	*DYNC1LI2*	dynein, cytoplasmic 1, light intermediate chain 2 (DYNC1LI2)[NM_006141]	2.1612577	0.013003563
A_23_P134078	*XLOC_l2_000657*	BROAD Institute lincRNA (XLOC_l2_000657), lincRNA [TCONS_l2_00000881]	0.76264447	0.03808641
A_23_P168812	*CDYL*	chromodomain protein, Y-like (CDYL) [NM_004824]	1.4392571	0.04644307
A_23_P22682	*FAM200A*	family with sequence similarity 200, member A (FAM200A) [NM_145111]	-0.8295312	0.04590026
A_23_P114947	*ARMCX1*	armadillo repeat containing, X-linked 1 (ARMCX1)[NM_016608]	-0.7569703	0.044449862
A_33_P3242873	*RGS2*	regulator of G-protein signaling 2 (RGS2) [NM_002923]	-1.9023821	0.04642224
A_33_P3380797	*MPC1*	mitochondrial pyruvate carrier 1 (MPC1)[NM_001270879]	-1.1855162	0.034540452
A_23_P153256	*FGF3*	fibroblast growth factor 3 (FGF3) [NM_005247]	-0.8632917	0.035227668
A_33_P3261167	*ZNF773*	zinc finger protein 773 [Source:HGNC Symbol;Acc:HGNC:30487] [ENST00000598770]	2.2102036	0.04639083
A_33_P3223239	*GNS*	glucosamine (N-acetyl)-6-sulfatase (GNS) [NM_002076]	-1.2138257	0.044672105
A_33_P3213064	*ST7L*	suppression of tumorigenicity 7 like (ST7L) [NM_138729]	0.62609667	0.048182435
A_22_P00010628	*STAT2*	signal transducer and activator of transcription 2, 113kDa (STAT2)[NM_005419]	-1.6437122	0.031377234
A_21_P0011712	*lnc-NDUFS8-1*	LNCipedia lincRNA (lnc-NDUFS8-1) [lnc-NDUFS8-1:13]	-0.93913555	0.031606767
A_33_P3415445	*CPOX*	coproporphyrinogen oxidase (CPOX) [NM_000097]	-1.0209731	0.048681058
A_24_P361457	*MAP3K2*	mitogen-activated protein kinase kinase kinase 2 (MAP3K2) [NM_006609]	-0.7762156	0.04528742
A_23_P123330	*ENDOV*	endonuclease V (ENDOV) [NM_173627]	-1.6278505	0.04542028
A_23_P119095	*RPL30*	ribosomal protein L30 (RPL30) [NM_000989]	-0.53480023	0.04610903
A_23_P129246	*PPP1R13L*	protein phosphatase 1, regulatory subunit 13 like (PPP1R13L) [NM_006663]	-0.81774974	0.037229434
A_21_P0013505	*PLEKHO2*	pleckstrin homology domain containing, family O member 2 (PLEKHO2) [NM_025201]	-1.6071416	0.024948223

A_33_P3372979	*KIAA0368*	KIAA0368 (KIAA0368) [NM_001080398]	-1.6721249	0.034293942
A_33_P3309665	*LINC00294*	long intergenic non-protein coding RNA 294 (LINC00294)[NR_015451]	-1.1983485	0.027751032
A_22_P00009821	*ZFP3*	ZFP3 zinc finger protein (ZFP3)[NM_153018]	-1.301095	0.03136619
A_21_P0014876	*lnc-METAP1-2*	LNCipedia lincRNA (lnc-METAP1-2)[lnc-METAP1-2:1]	-1.0066402	0.013003563
A_33_P3419806	*PDE4DIP*	phosphodiesterase 4D interacting protein (PDE4DIP) [NM_014644]	-0.6660881	0.03912857
A_24_P563545	*RPS29*	ribosomal protein S29 (RPS29) [NM_001032]	2.4803872	0.030915326
A_23_P204609	*SLC30A7*	solute carrier family 30 (zinc transporter), member 7 (SLC30A7)[NM_133496]	0.45735005	0.04434183
A_33_P3284596	*VEZT*	vezatin, adherens junctions transmembrane protein (VEZT) [NM_017599]	-1.997011	0.03403978
A_23_P346421	*ARL5C*	ADP-ribosylation factor-like 5C (ARL5C) [NM_001143968]	-0.94516563	0.029573848
A_22_P00019143	*ZNF532*	zinc finger protein 532 (ZNF532) [NM_018181]	-1.1502234	0.033433396
A_24_P272290	*lnc-DHX15-1*	PREDICTED: Homo sapiens uncharacterized LOC729175 (LOC729175) [XM_001129558]	-0.5794139	0.03504642
A_23_P14886	*PXDC1*	PX domain containing 1 (PXDC1)[NM_183373]	0.54548264	0.04253229
(+)E1A_r60_a20	*GNPTG*	N-acetylglucosamine-1-phosphate transferase, gamma subunit (GNPTG) [NM_032520]	-0.64663064	0.039628718
A_22_P00014709	*XLOC_l2_005557*	BROAD Institute lincRNA (XLOC_l2_005557) [TCONS_l2_00010291]	-2.2238173	0.04369149
A_33_P3333863	*lnc-SLC25A47-3*	LNCipedia lincRNA (lnc-SLC25A47-3), lincRNA [lnc-SLC25A47-3:1]	-1.3728642	0.02737376
A_24_P257359	*CCZ1*	CCZ1 vacuolar protein trafficking and biogenesis associated homolog (S. cerevisiae) (CCZ1)[NM_015622]	1.3198333	0.040091004
A_23_P2801	*RPL15*	ribosomal protein L15 (RPL15) [NM_002948]	-0.4858498	0.04253229
A_24_P374516	*ELF1*	E74-like factor 1 (ets domain transcription factor) (ELF1) [NM_172373]	0.8039978	0.03808641
A_33_P3234317	*TMSB4X*	thymosin beta 4, X-linked (TMSB4X) [NM_021109]	-1.5421059	0.031603426
A_33_P3351529	*RRAS2*	related RAS viral (r-ras) oncogene homolog 2 (RRAS2), transcript variant 1, mRNA [NM_012250]	-0.96857756	0.04555549

A_22_P00002044	*SIRT5*	sirtuin 5 (SIRT5) [NM_031244]	-1.9976214	0.04488468
A_23_P119141	*SCAMP1*	secretory carrier membrane protein 1 (SCAMP1) [NM_004866]	-1.2695926	0.029808238
A_23_P92948	*KEAP1*	kelch-like ECH-associated protein 1 (KEAP1) [NM_203500]	-0.6965003	0.048964206
A_23_P371682	*PRRC1*	proline-rich coiled-coil 1 (PRRC1)[NM_130809]	-1.2967902	0.028192867
A_23_P320159	*GPC6*	glypican 6 (GPC6) [NM_005708]	-0.9281703	0.034210585
A_23_P65174	*TCEANC*	transcription elongation factor A (SII) N-terminal and central domain containing (TCEANC)[NM_001297563]	-0.24312356	0.044364784
A_23_P155477	*PHF11*	PHD finger protein 11 (PHF11) [NM_001040443]	-0.83372396	0.033825807
A_33_P3314176	*C3orf18*	chromosome 3 open reading frame 18 (C3orf18)[NM_016210]	-2.871241	0.03655047
A_19_P00805291	*FAM46C*	family with sequence similarity 46, member C (FAM46C) [NM_017709]	-1.2354417	0.024948223
A_24_P324814	*ACTR8*	ARP8 actin-related protein 8 homolog (yeast) (ACTR8) [NM_022899]	-0.6444578	0.028573772
A_23_P121064	*CEP95*	centrosomal protein 95kDa (CEP95) [NM_138363]	4.7985477	0.04390963
A_33_P3306146	*PTX3*	pentraxin 3, long (PTX3) [NM_002852]	-1.3560493	0.033146918
A_23_P105957	*PLAU*	plasminogen activator, urokinase (PLAU) [NM_001145031]	-1.118956	0.048715595
A_33_P3339103	*ACTN1*	actinin, alpha 1 (ACTN1) [NM_001102]	-0.8046825	0.049758088
A_23_P119418	*POLR1C*	polymerase (RNA) I polypeptide C, 30kDa (POLR1C) [NM_203290]	-3.5765295	0.04434183
A_23_P257726	*URI1*	URI1, prefoldin-like chaperone (URI1) [NM_003796]	4.2638073	0.046959724
A_24_P48723	*C9orf156*	chromosome 9 open reading frame 156 (C9orf156) [NM_016481]	-0.95739716	0.031606767
A_33_P3391275	*PTGIS*	prostaglandin I2 (prostacyclin) synthase (PTGIS) [NM_000961]	-0.48127505	0.033198114
A_22_P00023557	*LINC00494*	long intergenic non-protein coding RNA 494 (LINC00494), long non-coding RNA [NR_026958]	-1.0688871	0.04542028
A_22_P00001924	*DDB1*	damage-specific DNA binding protein 1, 127kDa (DDB1), mRNA [NM_001923]	-3.963927	0.029808238
A_32_P208120	*lnc-BAI3-3*	RST28357 Athersys RAGE Library Homo sapiens cDNA, mRNA sequence [BG208723]	1.3297048	0.04929023
A_33_P3861706	*CAMK1D*	calcium/calmodulin-dependent protein kinase ID [Source:HGNC	1.5727237	0.026497496

		Symbol;Acc:HGNC:19341] [ENST00000615792]		
A_23_P205228	*MTMR10*	myotubularin related protein 10 (MTMR10) [NM_017762]	-0.65336514	0.04173021
A_21_P0014476	*ATP7B*	ATPase, Cu++ transporting, beta polypeptide (ATP7B)[NM_000053]	1.0504236	0.048618473
A_23_P50217	*CASP2*	caspase 2, apoptosis-related cysteine peptidase (CASP2) [NM_032982]	-0.81255287	0.044770036
A_33_P3279441	*ZNF671*	zinc finger protein 671 (ZNF671) [NM_024833]	-1.5710194	0.048372902
A_22_P00014836	*GOSR2*	golgi SNAP receptor complex member 2 (GOSR2), transcript variant C, [NM_001012511]	0.9432064	0.034210585
A_21_P0009752	*LOC101927963*	PREDICTED: Homo sapiens uncharacterized LOC101927963 (LOC101927963), transcript variant X3, ncRNA [XR_426361]	1.2845259	0.03777906
A_23_P19673	*lnc-UQCRFS1-9*	LNCipedia lincRNA (lnc-UQCRFS1-9), lincRNA [lnc-UQCRFS1-9:21]	-1.3277475	0.03738947
A_23_P6223	*SGK1*	serum/glucocorticoid regulated kinase 1 (SGK1) [NM_005627]	-0.66464597	0.04621581
A_33_P3352873	*SCAF4*	SR-related CTD-associated factor 4 (SCAF4) [NM_020706]	-0.6119468	0.031430684
A_24_P902728	*BCKDHB*	branched chain keto acid dehydrogenase E1, beta polypeptide (BCKDHB) [NM_000056]	-1.4379054	0.028479112
A_33_P3391603	*CAPRIN1*	cell cycle associated protein 1 (CAPRIN1) [NM_203364]	-1.798953	0.024948223
A_24_P277934	*LAMA4*	laminin, alpha 4 (LAMA4) [NM_001105209]	-0.7439035	0.038287252
A_23_P395524	*COL1A2*	collagen, type I, alpha 2 (COL1A2), [NM_000089]	-1.5714221	0.024948223
A_22_P00012710	*PWP1*	PWP1 homolog (S. cerevisiae) (PWP1) [NM_007062]	-1.6007417	0.035227668
A_23_P18325	*FLJ10038*	uncharacterized protein FLJ10038 (FLJ10038), long non-coding RNA [NR_026891]	-0.89847517	0.031569958
A_24_P173234	*PDCD10*	programmed cell death 10 (PDCD10) [NM_007217]	-0.59294456	0.047916878
A_23_P411296	*ZNF613*	zinc finger protein 613 (ZNF613) [NM_024840]	1.7814869	0.03136619
A_23_P151436	*CEBPB*	CCAAT/enhancer binding protein (C/EBP), beta (CEBPB) [NM_005194]	1.3333142	0.048398294
A_23_P103282	*ALG5*	ALG5, dolichyl-phosphate beta-glucosyltransferase (ALG5) [NM_013338]	-1.2966933	0.033198114
A_23_P39910	*TMEM59*	transmembrane protein 59 (TMEM59) [NM_004872]	-1.3491119	0.028573772

A_23_P162589	*COA5*	cytochrome c oxidase assembly factor 5 (COA5) [NM_001008215]	-0.471431	0.04434183
A_23_P27285	*VDR*	vitamin D (1,25- dihydroxyvitamin D3) receptor (VDR) [NM_001017535]	-0.6185152	0.042296566
A_33_P3311083	*MPPE1*	metallophosphoesterase 1 (MPPE1) [NM_023075]	-1.288346	0.028573772
A_33_P3361257	*PHLDB3*	pleckstrin homology-like domain, family B, member 3 [Source:HGNC Symbol;Acc:HGNC:30499] [ENST00000599242]	-1.4324273	0.04250221
A_19_P00316344	*NOP16*	NOP16 nucleolar protein (NOP16) [NM_016391]	-2.0124238	0.027751032
A_22_P00006419	*lnc-C9orf69-2*	clone IMAGE:2905626 [BC007236]	-0.95968246	0.028573772
A_24_P97836	*OAZ2*	ornithine decarboxylase antizyme 2 (OAZ2) [NM_002537]	-0.41145512	0.040555127
A_33_P3308332	*RAD17*	RAD17 homolog (S. pombe) (RAD17) [NM_002873]	0.84532607	0.036131933
A_32_P162150	*PLEKHB1*	pleckstrin homology domain containing, family B (evectins) member 1 (PLEKHB1) [NM_021200]	2.4385934	0.02737376
A_33_P3275199	*TAB3*	TGF-beta activated kinase 1/MAP3K7 binding protein 3 (TAB3) [NM_152787]	-0.64281017	0.03811336
A_33_P3869455	*JRK*	Jrk homolog (mouse) (JRK) [NM_003724]	-1.0789757	0.041768707
A_23_P130482	*ATXN8*	ataxin 8 (ATXN8) mRNA, partial cds. [DQ641254]	1.5911984	0.0370712
A_23_P205007	*ZNF211*	zinc finger protein 211 (ZNF211) [NM_198855]	-1.0726712	0.03436134
A_23_P76015	*IPO5*	importin 5 (IPO5) [NM_002271]	0.7459551	0.04831641
A_23_P201628	*ARHGEF17*	Rho guanine nucleotide exchange factor (GEF) 17 (ARHGEF17) [NM_014786]	1.7935195	0.032958407
A_24_P234415	*LAMC1*	laminin, gamma 1 (formerly LAMB2) (LAMC1)[NM_002293]	-1.2394358	0.035935212
A_24_P406301	*STAC*	SH3 and cysteine rich domain (STAC) [NM_003149]	-1.1476793	0.040555127
A_32_P407245	*NDUFB2*	NADH dehydrogenase (ubiquinone) 1 beta subcomplex, 2, 8kDa (NDUFB2) [NM_004546]	-0.45974165	0.026440267
A_24_P229871	*DNAJC22*	DnaJ (Hsp40) homolog, subfamily C, member 22 (DNAJC22) [NM_024902]	-1.436507	0.044689838
A_23_P156017	*LINC00469*	long intergenic non-protein coding RNA 469 [Source:HGNC Symbol;Acc:HGNC:26863] [ENST00000321800]	-4.6434555	0.02598378
A_23_P108835	*GOLPH3*	golgi phosphoprotein 3 (coat-protein) (GOLPH3) [NM_022130]	-0.6553783	0.04438234

A_33_P3308223	*YPEL5*	yippee-like 5 (Drosophila) (YPEL5) [NM_016061]	0.9527017	0.03138659
A_21_P0012393	*PDE4DIP*	phosphodiesterase 4D interacting protein (PDE4DIP) [NM_001198834]	1.5329344	0.03889491
A_24_P166663	*XLOC_l2_009883*	BROAD Institute lincRNA (XLOC_l2_009883), lincRNA [TCONS_l2_00018854]	-0.61764187	0.0460751
A_23_P24433	*CDK6*	cyclin-dependent kinase 6 (CDK6) [NM_001259]	-0.7903559	0.028573772
A_23_P103661	*CTSF*	cathepsin F (CTSF) [NM_003793]	-0.8816616	0.042621672
A_32_P64096	*YY1AP1*	YY1 associated protein 1 (YY1AP1) [NM_139118]	-2.1754298	0.030293303
A_33_P3231076	*ZKSCAN2*	zinc finger with KRAB and SCAN domains 2 (ZKSCAN2) [NM_001012981]	-1.2524282	0.03679443
A_23_P63153	*PAXIP1-AS2*	PAXIP1 antisense RNA 2 (PAXIP1-AS2), transcript variant 2, long non-coding RNA [NR_024477]	-1.5904632	0.033095628
A_24_P136470	*DDX20*	DEAD (Asp-Glu-Ala-Asp) box polypeptide 20 (DDX20) [NM_007204]	-0.866441	0.03777906
A_23_P28652	*BTN2A3P*	butyrophilin, subfamily 2, member A3, pseudogene (BTN2A3P), non-coding RNA [NR_027795]	-0.9513946	0.03770023
A_33_P3368109	*ATRAID*	all-trans retinoic acid-induced differentiation factor (ATRAID)[NM_016085]	-1.1680421	0.04114316
A_23_P390384	*EIF4B*	eukaryotic translation initiation factor 4B (EIF4B) [NM_001417]	-0.26871103	0.04124206
A_23_P39647	*ZNF630*	zinc finger protein 630 (ZNF630) [NM_001037735]	-0.86810726	0.044610508
A_21_P0014585	*SLC4A3*	solute carrier family 4 (anion exchanger), member 3 (SLC4A3)[NM_005070]	2.0038538	0.02453978
A_22_P00019433	*TMEM44-AS1*	TMEM44 antisense RNA 1 (TMEM44-AS1), transcript variant 1, long non-coding RNA [NR_047573]	-1.1969995	0.039287783
A_23_P252362	*lnc-SLC16A3-5*	LNCipedia lincRNA (lnc-SLC16A3-5), lincRNA [lnc-SLC16A3-5:1]	-1.7003227	0.037765756
A_23_P419947	*MRPS30*	mitochondrial ribosomal protein S30 (MRPS30) [NM_016640]	-1.7082901	0.04747978
A_19_P00321110	*MLF1*	myeloid leukemia factor 1 (MLF1) [NM_022443]	1.2229005	0.02598378
A_33_P3379962	*TIAM2*	T-cell lymphoma invasion and metastasis2 [ENST00000449545]	0.96221876	0.04622315
A_24_P33156	*HLA-A*	major histocompatibility complex, class I, A (HLA-A), transcript variant 1 (A*03:01:0:01 allele), [NM_002116]	-0.6295137	0.038988598

A_23_P71419	*AFMID*	arylformamidase (AFMID) [NM_001010982]	-1.5075325	0.028573772
A_32_P98927	*COPS5*	COP9 signalosome subunit 5 (COPS5) [NM_006837]	-0.79696274	0.032286324
A_24_P198820	*STAG3L4*	stromal antigen 3-like 4 (pseudogene) (STAG3L4) [NR_040585]	-1.0891556	0.04705478
A_23_P210358	*CDK12*	cyclin-dependent kinase 12 (CDK12) [NM_016507]	-0.9237248	0.029573848
A_33_P3258346	*LIMS1*	LIM and senescent cell antigen-like domains 1 (LIMS1) [NM_004987]	-0.94130254	0.046726856
A_22_P00010666	*XAF1*	XIAP associated factor 1 (XAF1) [NM_017523]	-1.6439564	0.033433396
A_33_P3324675	*lnc-NEO1-1*	LNCipedia lincRNA (lnc-NEO1-1), lincRNA [lnc-NEO1-1:2]	-0.55272055	0.033289474
A_23_P105900	*MXI1*	MAX interactor 1, dimerization protein (MXI1) [NM_130439]	-1.6646986	0.03338082
A_23_P84922	*BTF3P11*	basic transcription factor 3 pseudogene 11 (BTF3P11), non-coding RNA [NR_026983]	0.7668858	0.041067377
A_23_P593	*HDAC8*	histone deacetylase 8 (HDAC8) [NM_018486]	2.023187	0.030362261
A_24_P368544	*GPBP1L1*	GC-rich promoter binding protein 1-like 1 (GPBP1L1) [NM_021639]	-1.9144114	0.048182435
A_22_P00007888	*SLC25A26*	solute carrier family 25 (S-adenosylmethionine carrier), member 26 (SLC25A26) [NM_173471]	-1.0272373	0.04590026
A_23_P134384	*LINC00092*	long intergenic non-protein coding RNA 92 [Source:HGNC Symbol;Acc:HGNC:31408] [ENST00000583864]	-1.569181	0.029573848
A_24_P188878	*PHF14*	PHD finger protein 14 (PHF14) [NM_014660]	-0.6341176	0.040113345
A_23_P166	*RPL34*	ribosomal protein L34 (RPL34) [NM_033625]	-0.8293757	0.044452492
A_33_P3347301	*MOB3C*	MOB kinase activator 3C (MOB3C) [NM_145279]	1.9589846	0.04792962
A_21_P0001610	*LOC100127947*	Homo sapiens PP632 unknown mRNA. [AY240960]	-0.67980254	0.042994436
A_24_P30557	*lnc-FMOD-1*	LNCipedia lincRNA (lnc-FMOD-1), lincRNA [lnc-FMOD-1:1]	-0.74871516	0.039802827
A_24_P102880	*TBX5*	T-box 5 (TBX5) [NM_000192]	-0.4823002	0.03887779
A_33_P3311730	*NAV1*	neuron navigator 1 (NAV1) [NM_020443]	-1.4158387	0.040200267
A_33_P3250018	*USP8*	ubiquitin specific peptidase 8 (USP8)[NM_005154]	-2.264703	0.047831167
A_24_P943613	*HCFC2*	host cell factor C2 (HCFC2) [NM_013320]	-1.2986982	0.028192867

A_23_P100711	*TBC1D1*	TBC1 (tre-2/USP6, BUB2, cdc16) domain family, member 1 (TBC1D1) [NM_015173]	1.6883308	0.03591672
A_23_P54000	*PMP22*	peripheral myelin protein 22 (PMP22) [NM_000304]	-1.1535019	0.027942823
A_23_P258071	*SNX6*	sorting nexin 6 (SNX6) [NM_021249]	-1.442006	0.030589065
A_23_P121141	*RNF113A*	ring finger protein 113A (RNF113A)[NM_006978]	-0.8697579	0.033433396
A_21_P0011020	*ASTE1*	asteroid homolog 1 (Drosophila) (ASTE1) [NM_014065]	-2.0939126	0.032261204
A_33_P3233891	*XLOC_l2_002790*	BROAD Institute lincRNA (XLOC_l2_002790), lincRNA [TCONS_l2_00005211]	-0.9060976	0.045638688
A_23_P34018	*SRP72*	signal recognition particle 72kDa (SRP72)[NM_006947]	-0.65799206	0.02598378
A_23_P350187	*RPL39*	ribosomal protein L39 (RPL39)[NM_001000]	-1.0096126	0.035935212
A_23_P87902	*TET2*	s tet methylcytosine dioxygenase 2 (TET2) [NM_001127208]	-2.899284	0.03702924
A_21_P0011956	*DYRK4*	dual-specificity tyrosine-(Y)-phosphorylation regulated kinase 4 (DYRK4)[NM_003845]	-0.7123122	0.031377234
A_32_P182941	*XLOC_l2_008221*	BROAD Institute lincRNA (XLOC_l2_008221), lincRNA [TCONS_l2_00014844]	-0.5391693	0.042850092
A_33_P3245539	*RPS3*	ribosomal protein S3 (RPS3) [NM_001005]	-0.81674767	0.0381616
A_33_P3380567	*PNMA1*	paraneoplastic Ma antigen 1 (PNMA1) [NM_006029]	-1.0930986	0.03925921
A_22_P00006862	*SHARPIN*	SHANK-associated RH domain interactor (SHARPIN) [NM_030974]	-1.4621521	0.033433396
A_23_P64019	*TCEB3-AS1*	TCEB3 antisense RNA 1 (TCEB3-AS1), long non-coding RNA [NR_038280]	-1.0246938	0.031367566
A_23_P55688	*MTMR2*	myotubularin related protein 2 (MTMR2) [NM_201278]	-1.165791	0.042526342
A_23_P13183	*ZNF416*	zinc finger protein 416 (ZNF416) [NM_017879]	-0.88652706	0.048681058
A_23_P409553	*EXT2*	exostosin glycosyltransferase 2 (EXT2) [NM_000401]	1.5083396	0.02737376
A_23_P167358	*PPM1A*	protein phosphatase, Mg2+/Mn2+ dependent, 1A (PPM1A) [NM_177951]	-1.0753418	0.045331508
A_33_P3216869	*C4orf29*	chromosome 4 open reading frame 29 (C4orf29)[NM_001039717]	-0.78461576	0.04369149
A_23_P108342	*CRABP1*	cellular retinoic acid binding protein 1 (CRABP1)[NM_004378]	-1.0549614	0.027751032
A_23_P142146	*ZNF571*	zinc finger protein 571 (ZNF571) [NM_016536]	-0.82117456	0.04747978

A_24_P96762	*RPL18*	ribosomal protein L18 (RPL18) [NM_000979]	-1.4704704	0.031394158
A_23_P164341	*TRNAU1AP*	tRNA selenocysteine 1 associated protein 1 (TRNAU1AP)[NM_017846]	0.96872205	0.04250221
A_32_P62211	*VAMP2*	vesicle-associated membrane protein 2 (synaptobrevin 2) (VAMP2) [NM_014232]	-1.1891592	0.049967773
A_33_P3377045	*LOC101928433*	PREDICTED: Homo sapiens uncharacterized LOC101928433 (LOC101928433), ncRNA [XR_241982]	-0.8229982	0.03840554
A_23_P73457	*CDC37L1*	cell division cycle 37-like 1 [Source:HGNC Symbol;Acc:HGNC:17179] [ENST00000381854]	-0.86736536	0.04219537
A_24_P77681	*RUFY1*	RUN and FYVE domain containing 1 (RUFY1) [NM_025158]	1.2096372	0.04173021
A_23_P122216	*PAIP1*	poly(A) binding protein interacting protein 1 (PAIP1) [NM_006451]	-0.7344475	0.046256438
A_23_P171143	*LOX*	lysyl oxidase (LOX)[NM_002317]	-1.6318358	0.024948223
A_22_P00023177	*TSPAN6*	tetraspanin 6 (TSPAN6) [NM_003270]	-0.7626245	0.043971255
A_33_P3381771	*lnc-VTI1A-3*	cDNA: FLJ21484 fis, clone COL05256. [AK025137]	-1.0656025	0.049413078
A_33_P3297141	*GID4*	GID complex subunit 4 (GID4)[NM_024052]	-1.688977	0.03297842
A_33_P3246163	*WDR48*	WD repeat domain 48 (WDR48) [NM_020839]	-0.8957326	0.040555127
A_33_P3310296	*RPL5*	ribosomal protein L5 (RPL5)[NM_000969]	-1.2543305	0.03946874
A_23_P7325	*SLC35A1*	solute carrier family 35 (CMP-sialic acid transporter), member A1 (SLC35A1)[NM_001168398]	-1.1903514	0.041965257
A_23_P371076	*BST1*	bone marrow stromal cell antigen 1 (BST1) [NM_004334]	-1.0084918	0.03403978
A_23_P134935	*KLF12*	Kruppel-like factor 12 [Source:HGNC Symbol;Acc:HGNC:6346] [ENST00000472022]	1.1445949	0.02598378
A_33_P3216803	*DUSP4*	dual specificity phosphatase 4 (DUSP4) [NM_001394]	-1.2022314	0.03811336
A_24_P156501	*NARS*	asparaginyl-tRNA synthetase (NARS) [NM_004539]	1.3214377	0.03912857
A_33_P3301174	*EBF1*	early B-cell factor 1 (EBF1) [NM_024007]	-2.670631	0.036667652
A_23_P259741	*TMED2*	transmembrane emp24 domain trafficking protein 2 (TMED2) [NM_006815]	0.6832813	0.046464663

A_23_P19702	*SATB1*	SATB homeobox 1 (SATB1) [NM_002971]	-1.0771968	0.036044724
A_24_P156049	*TAB2*	TGF-beta activated kinase 1/MAP3K7 binding protein 2 (TAB2) [NM_015093]	-2.2151816	0.031578258
A_33_P3260575	*SLC39A6*	solute carrier family 39 (zinc transporter), member 6 (SLC39A6) [NM_012319]	-1.1786654	0.029808238
A_24_P35478	*CERCAM*	cerebral endothelial cell adhesion molecule (CERCAM) [NM_016174]	-0.9821393	0.047591254
A_23_P26954	*PARD3*	par-3 family cell polarity regulator (PARD3) [NM_019619]	-0.7871756	0.029397016
A_33_P3407424	*VAT1*	vesicle amine transport 1 (VAT1) [NM_006373]	-1.0908955	0.031606767
A_23_P148446	*CDC42EP1*	CDC42 effector protein (Rho GTPase binding) 1 (CDC42EP1) [NM_152243]	-1.0939116	0.027751032
A_21_P0006310	*UBE2A*	ubiquitin-conjugating enzyme E2A (UBE2A) [NM_003336]	-1.040755	0.034614928
A_23_P337729	*lnc-C9orf152-1*	LNCipedia lincRNA (lnc-C9orf152-1), lincRNA [lnc-C9orf152-1:1]	-1.1423087	0.035227668
A_33_P3300495	*TMEM180*	transmembrane protein 180 (TMEM180) [NM_024789]	-1.2995492	0.042693015
A_32_P41065	*PLGLB1*	plasminogen-like B1 [ENST00000409310]	-0.9592509	0.045638688
A_23_P320185	*TMCC1*	transmembrane and coiled-coil domain family 1 (TMCC1) [NM_001017395]	0.67562973	0.04733213
A_24_P169773	*NDUFA11*	NADH dehydrogenase (ubiquinone) 1 alpha subcomplex, 11, 14.7kDa (NDUFA11) [NM_175614]	-1.3240306	0.02598378
A_23_P53288	*CTAGE4*	CTAGE family, member 4 (CTAGE4) [NM_198495]	-1.9531019	0.031603426
A_33_P3841368	*CNPY2*	canopy FGF signaling regulator 2 (CNPY2) [NM_014255]	-1.0718185	0.038287252
A_22_P00021380	*FBXO25*	F-box protein 25 [ENST00000382824]	-0.67473936	0.036667652
A_23_P32454	*STMND1*	stathmin domain containing 1 (STMND1) [NM_001190766]	-0.8524315	0.031367566
A_24_P941167	*TG*	thyroglobulin (TG) [NM_003235]	-1.0412967	0.0442831
A_33_P3306207	*APOL6*	apolipoprotein L, 6 (APOL6) [NM_030641]	-1.070023	0.04438234
A_33_P3373375	*KLRG2*	killer cell lectin-like receptor subfamily G, member 2 (KLRG2)[NM_198508]	1.4008852	0.046325136
A_33_P3365856	*CD81*	CD81 molecule (CD81) [NM_004356]	-1.6728735	0.038180117
A_23_P383435	*lnc-AL117340.1-2*	fs15d03.y2 Human Lens cDNA (Normalized): fs Homo sapiens cDNA clone fs15d03 5' [CD674797]	-1.663166	0.031334452

A_23_P64669	*ZNHIT3*	zinc finger, HIT-type containing 3 (ZNHIT3) [NM_004773]	1.2264937	0.048760455
A_23_P124476	*ZCCHC8*	zinc finger, CCHC domain containing 8 (ZCCHC8) [NM_017612]	-0.5165627	0.03403978
A_22_P00006716	*CLCN3*	chloride channel, voltage-sensitive 3 (CLCN3)[NM_173872]	-0.9924026	0.048312414
A_23_P35309	*lnc-FRMD4B-2*	MGC7.1.1.1.1.H08.F.1 NIH_MGC_331 Homo sapiens cDNA clone MGC7.1.1.1.1.H08, [DR731266]	-0.8130054	0.032155342
A_21_P0005184	*TAF5L*	TAF5-like RNA polymerase II, p300/CBP-associated factor (PCAF)-associated factor, 65kDa (TAF5L)[NM_014409]	-0.46979493	0.03452819
A_23_P112634	*lnc-WDR27-1*	LNCipedia lincRNA (lnc-WDR27-1)[lnc-WDR27-1:7]	-1.1082741	0.034210585
A_23_P2725	*SMIM14*	small integral membrane protein 14 (SMIM14) [NM_174921]	2.006503	0.02598378
A_21_P0005658	*RPL21*	ribosomal protein L21 (RPL21) [NM_000982]	-0.66761553	0.041257963
A_24_P204214	*PSAP*	prosaposin (PSAP) [NM_001042465]	-1.3289267	0.040555127
A_23_P398372	*NCOR1*	nuclear receptor corepressor 1 (NCOR1) [NM_006311]	2.5984128	0.030362261
A_23_P38864	*C9orf69*	chromosome 9 open reading frame 69 (C9orf69) [NM_001256526]	-1.594238	0.036427937
A_33_P3410409	*RABAC1*	Rab acceptor 1 (prenylated) (RABAC1) [NM_006423]	-1.4780507	0.04645665
A_24_P40229	*LAMP2*	lysosomal-associated membrane protein 2 (LAMP2) [NM_001122606]	-0.8775766	0.046040986
A_22_P00017061	*CRNKL1*	crooked neck pre-mRNA splicing factor 1 (CRNKL1)[NM_016652]	-0.79782367	0.034037594
A_23_P202881	*PCCB*	propionyl CoA carboxylase, beta polypeptide (PCCB) [NM_000532]	-1.0298179	0.03510091
A_33_P3243554	*FEZ1*	fasciculation and elongation protein zeta 1 (zygin I) (FEZ1) [NM_005103]	-1.582109	0.039863296
A_21_P0001705	*SEPT6*	septin 6 (SEPT6) [NM_145802]	-1.2206358	0.028192867
A_21_P0000310	*LINC00869*	long intergenic non-protein coding RNA 869 (LINC00869), transcript variant 8, long non-coding RNA [NR_111952]	-0.7881894	0.046256438
A_24_P10137	*SNORA12*	small nucleolar RNA, H/ACA box 12 (SNORA12) [NR_002954]	-0.6373021	0.0415208
A_23_P159539	*RGCC*	regulator of cell cycle (RGCC) [NM_014059]	-1.6704284	0.04438234
A_33_P3209386	*ASMTL*	acetylserotonin O-methyltransferase-like (ASMTL)[NM_004192]	-1.5799012	0.04091952

A_33_P3751889	*TXNDC17*	thioredoxin domain containing 17 (TXNDC17) [NM_032731]	-1.5858469	0.04610903
A_23_P318604	*TOM1*	target of myb1 (chicken) (TOM1) [NM_001135732]	3.9812367	0.028573772
A_21_P0011843	*CYHR1*	cysteine/histidine-rich 1 [ENST00000528663]	-1.464699	0.039287783
A_23_P150053	*PGM5*	phosphoglucomutase 5 [ENST00000604870]	-1.0067583	0.031493735
A_33_P3296592	*ACTA2*	actin, alpha 2, smooth muscle, aorta (ACTA2)[NM_001613]	-0.9976337	0.031377234
A_33_P3405444	*C11orf30*	chromosome 11 open reading frame 30 (C11orf30) [NM_001300942]	-0.63506055	0.030362261
A_21_P0012200	*WASL*	Wiskott-Aldrich syndrome-like (WASL) [NM_003941]	2.050686	0.034810174
A_23_P102611	*RRAS2*	related RAS viral (r-ras) oncogene homolog 2 (RRAS2) [NM_012250]	-0.8109564	0.048681058
A_24_P551028	*WISP2*	WNT1 inducible signaling pathway protein 2 (WISP2) [NM_003881]	-0.9290211	0.047591254
A_23_P2317	*SPOPL*	speckle-type POZ protein-like (SPOPL)[NM_001001664]	-0.8876688	0.03794058
A_23_P58967	*DDN*	dendrin (DDN) [NM_015086]	-1.3267813	0.03428924
A_33_P3322909	*ZC3H11A*	zinc finger CCCH-type containing 11A (ZC3H11A) [NM_014827]	-6.1075945	0.034293942
A_23_P69383	*CUL4A*	cullin 4A (CUL4A) [NM_001278513]	-1.0274518	0.04663971
A_21_P0006079	*PARP9*	poly (ADP-ribose) polymerase family, member 9 (PARP9) [NM_031458]	1.1884462	0.033091284
A_23_P100441	*LINC01506*	long intergenic non-protein coding RNA 1506 (LINC01506), long non-coding RNA [NR_109769]	-0.5425663	0.02737376
A_33_P3354851	*FAM192A*	family with sequence similarity 192, member A (FAM192A) [NM_024946]	-1.0815408	0.02737376
A_23_P77160	*SLC19A2*	solute carrier family 19 (thiamine transporter), member 2 (SLC19A2), [NM_006996]	-0.79924345	0.028573772
A_23_P102258	*RNF111*	finger protein 111 (RNF111) [NM_017610]	-0.68228966	0.043911606
A_23_P141636	*MRPL53*	mitochondrial ribosomal protein L53 (MRPL53) [NM_053050]	0.6956444	0.043335356
A_23_P42498	*EIF4A3*	eukaryotic translation initiation factor 4A3 (EIF4A3)[NM_014740]	-0.8124148	0.034375925
A_23_P8185	*SNX3*	sorting nexin 3 (SNX3)NM_003795]	-0.99894756	0.030915326
A_33_P3262789	*DYNLT1*	dynein, light chain, Tctex-type 1 (DYNLT1)[NM_006519]	-0.61816835	0.048371606
A_23_P159688	*REEP6*	receptor accessory protein 6 (REEP6) [NM_138393]	-0.42740083	0.04414394
A_24_P112750	*TBC1D25*	TBC1 domain family, member 25 (TBC1D25) [NM_002536]	-1.0350504	0.046256438

A_33_P3214096	*TFCP2*	transcription factor CP2 (TFCP2) [NM_005653]	-0.964756	0.03912857
A_23_P15944	*ATF3*	activating transcription factor 3 (ATF3)[NM_001040619]	-2.522894	0.03073435
A_24_P23411	*ELAC1*	elaC ribonuclease Z 1 (ELAC1)[NM_018696]	-0.7479146	0.036129165
A_21_P0000746	*ARMCX3*	armadillo repeat containing, X-linked 3 (ARMCX3) [NM_016607]	-0.88490057	0.0442831
A_23_P131737	*LINC00933*	long intergenic non-protein coding RNA 933 (LINC00933) [NR_038273]	1.3164592	0.025423726
A_33_P3261927	*VPS54*	vacuolar protein sorting 54 homolog (S. cerevisiae) (VPS54) [NM_016516]	-0.83547515	0.031526815
A_24_P418816	*ZNF789*	zinc finger protein 789 (ZNF789)[NM_213603]	-2.4452672	0.033221602
A_23_P115316	*GPX7*	glutathione peroxidase 7 (GPX7) [NM_015696]	-1.3065625	0.03716006
A_21_P0000174	*TOR3A*	torsin family 3, member A (TOR3A) [NM_022371]	-0.7507756	0.048247673
A_23_P212545	*C5orf56*	cDNA, FLJ99232. [AK309191]	-0.8629443	0.033091284
A_24_P318939	*RAB5A*	RAB5A, member RAS oncogene family (RAB5A) [NM_004162]	-1.9622283	0.031091278
A_33_P3348204	*ZNF337*	zinc finger protein 337 (ZNF337) [NM_015655]	-1.1644403	0.03431701
A_23_P159305	*FAM218A*	family with sequence similarity 218, member A (FAM218A) [NM_153027]	0.8936249	0.03220128
A_32_P129527	*TAF15*	TAF15 RNA polymerase II, TATA box binding protein (TBP)-associated factor, 68kDa (TAF15)[NM_139215]	-0.86800927	0.02453978
A_33_P3884230	*ERMARD*	ER membrane-associated RNA degradation (ERMARD) [NM_018341]	-0.78273374	0.028573772
A_23_P121956	*NFIX*	nuclear factor I/X (CCAAT-binding transcription factor) (NFIX) [NM_002501]	-1.4606888	0.036009874
A_33_P3394615	*THG1L*	tRNA-histidine guanylyltransferase 1-like (S. cerevisiae) (THG1L), [NM_017872]	-0.4500065	0.03709602
A_33_P3410599	*FUNDC2*	FUN14 domain containing 2 (FUNDC2) [NM_023934]	-1.3387452	0.04643008
A_23_P74320	*FAM46A*	family with sequence similarity 46, member A (FAM46A) [NM_017633]	-0.603168	0.027751032
A_32_P460973	*SCYL3*	SCY1-like 3 (S. cerevisiae) (SCYL3)[NM_020423]	-1.1108708	0.04811439
A_22_P00010567	*HLA-E*	major histocompatibility complex, class I, E (HLA-E) [NM_005516]	-0.8733053	0.038003992
A_33_P3293753	*lnc-NDE1-3*	LNCipedia lincRNA (lnc-NDE1-3), lincRNA [lnc-NDE1-3:1]	-2.228703	0.028573772

A_33_P3381127	*KRTAP10-9*	keratin associated protein 10-9 (KRTAP10-9)[NM_198690]	-0.61895245	0.04740209
A_32_P170003	*FAS*	Fas cell surface death receptor (FAS) [NM_000043]	-0.9530616	0.03871793
A_23_P422981	*TRAF3IP1*	TNF receptor-associated factor 3 interacting protein 1 (TRAF3IP1)[NM_015650]	1.4600474	0.029979022
A_33_P3360216	*FBXO36*	F-box protein 36 (FBXO36)[NM_174899]	0.96534276	0.039287783
A_21_P0013848	*HIST1H2AI*	histone cluster 1, H2ai (HIST1H2AI)[NM_003509]	-1.4779952	0.027942823
A_23_P76823	*FOXN3*	forkhead box N3 (FOXN3) [NM_005197]	-0.7119075	0.041929405
A_22_P00017749	*ADSSL1*	s adenylosuccinate synthase like 1 (ADSSL1) [NM_199165]	-0.9143262	0.043885134
A_33_P3215193	*lnc-YIF1A-1*	LNCipedia lincRNA (lnc-YIF1A-1), lincRNA [lnc-YIF1A-1:2]	3.9451385	0.043335356
A_23_P305140	*NMNAT1*	nicotinamide nucleotide adenylyltransferase 1 (NMNAT1) [NM_001297779]	-1.5533938	0.03811336
A_33_P3274245	*C10orf32*	chromosome 10 open reading frame 32 (C10orf32) [NM_144591]	-1.068036	0.0471361
A_22_P00000406	*ENDOV*	endonuclease V (ENDOV) [NM_173627]	-0.96830785	0.044364784
A_33_P3529859	*LOC102724508*	uncharacterized LOC102724508 (LOC102724508) [NR_110881]	-1.1493335	0.02737376
A_23_P311087	*KDM2B*	lysine (K)-specific demethylase 2B (KDM2B) [NM_032590]	-1.036428	0.040741067
A_23_P35082	*ZNF281*	zinc finger protein 281 (ZNF281) [NM_012482]	1.7267693	0.027019119
A_33_P3389478	*SESN2*	sestrin 2 (SESN2) [NM_031459]	0.8543267	0.029979022
A_23_P74435	*SNAPC3*	small nuclear RNA activating complex, polypeptide 3, 50kDa [ENST00000380799]	-0.71815646	0.04855821
A_23_P95879	*C1orf50*	chromosome 1 open reading frame 50 (C1orf50)[NM_024097]	-2.1697967	0.03205687
A_24_P72064	*RPL38*	ribosomal protein L38 (RPL38)[NM_000999]	-1.35993	0.04573629
A_32_P194821	*GHR*	growth hormone receptor (GHR) [NM_000163]	-1.179367	0.041418947
A_33_P3402217	*RPL21*	ribosomal protein L21 (RPL21) [NM_000982]	-0.9827776	0.028573772
A_21_P0011423	*NUPR1*	nuclear protein, transcriptional regulator, 1 (NUPR1) [NM_001042483]	-1.6786766	0.04854581
A_24_P411899	*XLOC_l2_005175*	BROAD Institute lincRNA (XLOC_l2_005175)[TCONS_l2_00009664]	-0.82127064	0.049758088

A_33_P3277178	*RNF19A*	ring finger protein 19A, RBR E3 ubiquitin protein ligase (RNF19A) [NM_183419]	-1.3628753	0.03403978
A_23_P208143	*SSPO*	SCO-spondin [ENST00000472850]	-1.5796412	0.025407597
A_23_P500892	*ZNF397*	zinc finger protein 397 (ZNF397) [NM_001135178]	-0.68217665	0.039942726
A_24_P322771	*TUB*	tubby bipartite transcription factor (TUB) [NM_003320]	-1.3848615	0.027942823
A_33_P3843285	*TFF1*	trefoil factor 1 (TFF1) [NM_003225]	1.2443979	0.02737376
A_33_P3308158	*MAGI1-IT1*	PREDICTED: Homo sapiens MAGI1 intronic transcript 1 (non-protein coding) (MAGI1-IT1) [XR_110023]	-1.6619766	0.033719208
A_33_P3240532	*lnc-AC073343.1-1*	LNCipedia lincRNA (lnc-AC073343.1-1), lincRNA [lnc-AC073343.1-1:3]	0.71561724	0.03403978
A_21_P0013475	*RGL1*	ral guanine nucleotide dissociation stimulator-like 1 (RGL1) [NM_015149]	1.3731437	0.035769645
A_33_P3251896	*UFC1*	ubiquitin-fold modifier conjugating enzyme 1 (UFC1)[NM_016406]	0.44954503	0.03591672
A_33_P3356607	*APBB2*	amyloid beta (A4) precursor protein-binding, family B, member 2 (APBB2) [NM_004307]	-1.1150488	0.03668808
A_33_P3564409	*WIPI2*	WD repeat domain, phosphoinositide interacting 2 (WIPI2)[NM_001033518]	-1.523.262	0.03871793
A_23_P21162	*FOXF2*	forkhead box F2 (FOXF2) [NM_001452]	-1.3097049	0.033412892
A_22_P00009979	*TCTEX1D2*	Tctex1 domain containing 2 (TCTEX1D2) [NM_152773]	-0.61325043	0.047831167
A_24_P50368	*LOC100129781*	PREDICTED: Homo sapiens uncharacterized LOC100129781 (LOC100129781) [XR_109259]	1.3899285	0.04792962
A_24_P49190	*BLID*	BH3-like motif containing, cell death inducer (BLID)[NM_001001786]	-3.4577324	0.02737376
A_23_P354208	*C17orf58*	chromosome 17 open reading frame 58 (C17orf58) [NM_181655]	-0.9730737	0.034422405
A_23_P166899	*WDR81*	WD repeat domain 81 (WDR81) [NM_152348]	-2.100637	0.041027144
A_23_P89550	*DNAJB11*	DnaJ (Hsp40) homolog, subfamily B, member 11 (DNAJB11) [NM_016306]	-2.3332844	0.027751032
A_33_P3475737	*NLRP1*	NLR family, pyrin domain containing 1 (NLRP1)[NM_033004]	-2.0828018	0.04542028
A_23_P143559	*ZBTB8A*	zinc finger and BTB domain containing 8A (ZBTB8A) [NM_001040441]	-1.1421616	0.032914247
A_33_P3343090	*CLTCL1*	clathrin, heavy chain-like 1 (CLTCL1) [NM_007098]	-0.8564277	0.034342222
A_24_P384569	*MAP1S*	microtubule-associated protein 1S (MAP1S) [NM_018174]	-2.0777621	0.035560552

A_21_P0012564	*EWSR1*	EWS RNA-binding protein 1 (EWSR1) [NM_001163287]	-0.7476669	0.044506002
A_24_P31421	*XLOC_l2_010508*	BROAD Institute lincRNA (XLOC_l2_010508), lincRNA [TCONS_l2_00020322]	-1.6465974	0.034293942
A_33_P3663705	*GLTSCR1*	glioma tumor suppressor candidate region gene 1 (GLTSCR1) [NM_015711]	-1.017942	0.04462891
A_23_P115046	*CROCC*	ciliary rootlet coiled-coil, rootletin (CROCC) [NM_014675]	0.90933967	0.03420731
A_24_P328819	*EIF2B3*	eukaryotic translation initiation factor 2B, subunit 3 gamma, 58kDa (EIF2B3) [NM_020365]	-1.5847394	0.04434183
A_23_P70991	*TRAPPC11*	trafficking protein particle complex 11 (TRAPPC11) [NM_199053]	1.1104482	0.03273351
A_33_P3645805	*AIMP2*	aminoacyl tRNA synthetase complex-interacting multifunctional protein 2 (AIMP2) [NM_006303]	-0.82779765	0.029808238
A_32_P49616	*MGA*	MGA, MAX dimerization protein (MGA) [NM_001164273]	-1.0850153	0.03639509
A_33_P3250963	*EEF1B2*	eukaryotic translation elongation factor 1 beta 2 (EEF1B2) [NM_001959]	-1.1928089	0.034999274
A_24_P315500	*TP53TG1*	TP53 target 1 (non-protein coding) (TP53TG1)[NR_015381]	-0.55802006	0.031127423
A_33_P3265872	*NFYC-AS1*	NFYC antisense RNA 1 (NFYC-AS1)A [NR_024567]	-0.94925857	0.037136003
A_33_P3416350	*LOC101927497*	uncharacterized LOC101927497 (LOC101927497) [NR_110086]	-0.69020337	0.031091278
A_23_P214739	*ITSN2*	intersectin 2 (ITSN2) [NM_147152]	-1.0671442	0.046523497
A_24_P889720	*FBXL4*	F-box and leucine-rich repeat protein 4 (FBXL4) [NM_012160]	-1.2660748	0.047685638
A_32_P52206	*UBC*	ubiquitin C (UBC) [NM_021009]	-2.1801784	0.036129165
A_23_P4628	*TYW1*	tRNA-yW synthesizing protein 1 homolog (S. cerevisiae) (TYW1) [NM_018264]	1.3947531	0.03126305
A_22_P00019501	*ZNF606*	zinc finger protein 606 (ZNF606) [NM_025027]	-0.919466	0.031649236
A_24_P139191	*WDR45B*	WD repeat domain 45B (WDR45B) [NM_019613]	-0.49273545	0.04550219
A_23_P48070	*ITCH*	itchy E3 ubiquitin protein ligase (ITCH) [NM_031483]	-0.8688936	0.030362261
A_22_P00022304	*ING4*	inhibitor of growth family, member 4 (ING4) [NM_016162]	1.2963481	0.024948223
A_33_P3223874	*lnc-SUSD2-1*	BX100553 Soares_NFL_T_GBC_S1 [BX100553]	-0.53466964	0.043741588

A_23_P128543	*LOC102724679*	PREDICTED: Homo sapiens uncharacterized LOC102724679 (LOC102724679), ncRNA [XR_425307]	-0.9066307	0.024948223
A_33_P3366484	*MED4*	mediator complex subunit 4 (MED4) [NM_014166]	1.0996248	0.027751032
A_23_P311232	*HIBCH*	3-hydroxyisobutyryl-CoA hydrolase (HIBCH) [NM_198047]	-1.1502788	0.046464663
A_24_P285623	*CLASP1*	cytoplasmic linker associated protein 1 (CLASP1) [NM_015282]	1.1589669	0.03136619
A_33_P3345389	*DGUOK*	deoxyguanosine kinase (DGUOK) [NM_080916]	-1.041913	0.042850092
A_33_P3353051	*C20orf194*	chromosome 20 open reading frame 194 (C20orf194) [NM_001009984]	-1.43985	0.027942823
A_23_P73429	*C6orf48*	chromosome 6 open reading frame 48 (C6orf48) [NM_001287483]	-0.64809704	0.026587285
A_33_P3362521	*HCLS1*	hematopoietic cell-specific Lyn substrate 1 (HCLS1) [NM_005335]	-0.8995261	0.042850092
A_23_P21776	*CCDC174*	coiled-coil domain containing 174 (CCDC174) [NM_016474]	-0.6007386	0.03504642
A_23_P132784	*SNAPC5*	small nuclear RNA activating complex, polypeptide 5, 19kDa [ENST00000316634]	-1.1757894	0.042907383
A_24_P339869	*FXR1*	fragile X mental retardation, autosomal homolog 1 (FXR1) [NM_001013439]	-2.9997954	0.033091284
A_33_P3390335	*ZBTB21*	zinc finger and BTB domain containing 21 (ZBTB21)[NM_020727]	-1.0832549	0.042529937
A_33_P3320393	*RSU1*	Ras suppressor protein 1 (RSU1) [NM_012425]	-0.40859434	0.04542028
A_22_P00025249	*MRPS16*	mitochondrial ribosomal protein S16 (MRPS16)[NM_016065]	-1.015986	0.034008626
A_24_P57700	*lnc-CEACAM18-2*	DB453801 RIKEN full-length enriched human cDNA library, testis Homo sapiens cDNA clone H013063A19 5' [DB453801]	-0.5391167	0.027751032
A_23_P16225	*ZHX3*	zinc fingers and homeoboxes 3 (ZHX3) [NM_015035]	-1.5823016	0.035700265
A_23_P209805	*BEST2*	bestrophin 2 (BEST2) [NM_017682]	-0.54217625	0.038034573
A_21_P0014001	*NAB1*	NGFI-A binding protein 1 (EGR1 binding protein 1) (NAB1) [NM_005966]	0.89189166	0.0296305
A_23_P314115	*NNT-AS1*	NNT antisense RNA 1 (NNT-AS1) [NR_073113]	-0.91682553	0.030362261
A_24_P329924	*BMI1*	BMI1 proto-oncogene, polycomb ring finger (BMI1) [NM_005180]	1.5923547	0.046497274
A_23_P127676	*SIK3*	SIK family kinase 3 (SIK3) [NM_025164]	-0.511898	0.048182435

A_23_P436117	*CTR9*	CTR9, Paf1/RNA polymerase II complex component (CTR9) [NM_014633]	0.45840862	0.04873285
A_23_P378690	*HMG20A*	high mobility group 20A (HMG20A) [NM_018200]	-1.3035536	0.034635574
A_23_P80156	*TMEM64*	transmembrane protein 64 (TMEM64) [NM_001008495]	-0.7986665	0.0442831
A_21_P0013300	*PRMT2*	protein arginine methyltransferase 2 (PRMT2) [NM_206962]	-0.6662345	0.047932126
A_21_P0014496	*ZNF345*	zinc finger protein 345 (ZNF345) [NM_003419]	-1.316029	0.02598378
A_23_P127565	*LOC101927752*	PREDICTED: uncharacterized LOC101927752 (LOC101927752) [XR_245324]	-1.3820229	0.02598378
A_24_P135255	*LAYN*	layilin (LAYN) [NM_178834]	-1.277792	0.037795458
A_33_P3288700	*SUGT1P3*	SUGT1 pseudogene 3 (SUGT1P3)[NR_003365]	-1.1621977	0.02737376
A_21_P0009754	*FAM207A*	family with sequence similarity 207, member A (FAM207A)[NM_058190]	-0.96547955	0.029541323
A_23_P68155	*lnc-UQCRFS1-9*	LNCipedia lincRNA (lnc-UQCRFS1-9), lincRNA [lnc-UQCRFS1-9:23]	-1.369755	0.03504642
A_24_P835500	*IFIH1*	interferon induced with helicase C domain 1 (IFIH1) [NM_022168]	0.8578123	0.037923194
A_23_P109774	*ITPRIPL2*	inositol 1,4,5-trisphosphate receptor interacting protein-like 2 (ITPRIPL2) [NM_001034841]	-0.7864812	0.046120346
A_33_P3341731	*ZBTB11*	zinc finger and BTB domain containing 11 (ZBTB11) [NM_014415]	-1.4546185	0.029573848
A_33_P3402474	*TFDP2*	transcription factor Dp-2 (E2F dimerization partner 2) (TFDP2) [NM_001178138]	-0.57803875	0.034552876
A_23_P82351	*ATAT1*	alpha tubulin acetyltransferase 1 (ATAT1) [NM_001031722]	-0.76963097	0.03504642
A_23_P200829	*BBS9*	Bardet-Biedl syndrome 9 (BBS9) [NM_198428]	0.9862201	0.03912857
A_21_P0007163	*SRGAP2*	SLIT-ROBO Rho GTPase activating protein 2 (SRGAP2) [NM_015326]	-1.2223063	0.036129165
A_33_P3366120	*lnc-APOA4-1*	LNCipedia lincRNA (lnc-APOA4-1), lincRNA [lnc-APOA4-1:1]	-0.7989781	0.027751032
A_23_P123193	*FLNA*	filamin A, alpha (FLNA) [NM_001110556]	-0.75617504	0.048238702
A_32_P13795	*ACTR3B*	ARP3 actin-related protein 3 homolog B (yeast) (ACTR3B) [NM_020445]	-1.0937747	0.037395623
A_33_P3262485	*DNAJC27*	DnaJ (Hsp40) homolog, subfamily C, member 27 (DNAJC27) [NM_016544]	1.5423985	0.04784218
A_23_P410998	*ZNF563*	zinc finger protein 563 (ZNF563) [NM_145276]	-1.0899936	0.03840762

A_23_P14708	*RAB5B*	RAB5B, member RAS oncogene family (RAB5B) [NM_002868]	-0.94746524	0.03497811
A_33_P3305467	*ZNF280D*	zinc finger protein 280D (ZNF280D) [NM_017661]	-0.8861207	0.028573772
A_23_P78526	*YIF1A*	Yip1 interacting factor homolog A (S. cerevisiae) (YIF1A) [NM_020470]	-0.69931275	0.039189726
A_23_P259054	*CEACAM19*	carcinoembryonic antigen-related cell adhesion molecule 19 (CEACAM19) [NM_020219]	-1.3078029	0.038287252
A_32_P139229	*SNX14*	sorting nexin 14 (SNX14) [NM_153816]	-1.8553212	0.029642703
A_23_P109821	*ZNF543*	zinc finger protein 543 (ZNF543) [NM_213598]	-0.8106627	0.049567465
A_23_P25224	*TADA3*	transcriptional adaptor 3 (TADA3)[NM_006354]	0.4331408	0.04590026
A_22_P00023755	*YBX3*	Y box binding protein 3 (YBX3) [NM_003651]	-1.1002454	0.046464663
A_22_P00003920	*B4GALT1-AS1*	B4GALT1 antisense RNA 1 (B4GALT1-AS1) [NR_108108]	-0.3405851	0.03811336
A_23_P84782	*CES2*	carboxylesterase 2 (CES2) [NM_003869]	-0.5867646	0.048238702
A_23_P46017	*THAP4*	THAP domain containing 4 (THAP4) [NM_015963]	-1.8177198	0.028573772
A_19_P00316547	*CCBL2*	cysteine conjugate-beta lyase 2 (CCBL2) [NM_001008661]	-1.3147317	0.03220128
A_23_P43326	*HNRNPA0*	heterogeneous nuclear ribonucleoprotein A0 (HNRNPA0) [NM_006805]	-1.4741222	0.027751032
A_33_P3226212	*SPTLC1*	serine palmitoyltransferase, long chain base subunit 1 (SPTLC1) [NM_006415]	1.5005975	0.042269725
A_23_P145068	*JAM2*	junctional adhesion molecule 2 (JAM2) [NM_001270408]	1.1459873	0.02737376
A_33_P3317815	*MTO1*	mitochondrial tRNA translation optimization 1 (MTO1) [NM_012123]	-1.1028444	0.029573848
A_23_P208961	*KRAS*	Kirsten rat sarcoma viral oncogene homolog (KRAS) [NM_004985]	-1.0147654	0.034334898
A_33_P3289218	*MUM1*	melanoma associated antigen (mutated) 1 (MUM1) [NM_032853]	-4.7438416	0.029573848
A_23_P87560	*AAK1*	AP2 associated kinase 1 (AAK1) [NM_014911]	-1.2318206	0.037613362
A_22_P00003927	*BTG1*	B-cell translocation gene 1, anti-proliferative (BTG1) [NM_001731]	-1.662955	0.033477332
A_33_P3310293	*lnc-CETP-1*	clone IMAGE:4426859 [BC015447]	-0.5797031	0.042907383
A_23_P165879	*PKIG*	protein kinase (cAMP-dependent, catalytic) inhibitor gamma (PKIG)[NM_181805]	-0.7000558	0.047941346

A_24_P37519	*RAB10*	RAB10, member RAS oncogene family (RAB10) [NM_016131]	-2.1312797	0.036009874
A_23_P6891	*LZTFL1*	leucine zipper transcription factor-like 1 (LZTFL1)[NM_020347]	-0.939409	0.04792962
A_23_P170839	*EIF1B*	eukaryotic translation initiation factor 1B (EIF1B) [NM_005875]	-1.5958953	0.028573772
A_33_P3335865	*SEPT2*	septin 2 (SEPT2) [NM_001008491]	-0.884053	0.04041675
A_23_P422212	*WDR35*	WD repeat domain 35 (WDR35) [NM_001006657]	-1.1577566	0.036364187
A_22_P00011014	*SLC35F3*	solute carrier family 35, member F3 (SLC35F3) [NM_173508]	-1.6271783	0.038727812
A_24_P255845	*lnc-NSUN6-3*	LNCipedia lincRNA (lnc-NSUN6-3)[lnc-NSUN6-3:1]	-0.98958325	0.02737376
A_23_P40693	*LOC100129931*	uncharacterized LOC100129931 (LOC100129931) [NR_033828]	5.7419763	0.028192867
A_33_P3271925	*EP300*	E1A binding protein p300 (EP300) [NM_001429]	1.3232142	0.042850092
A_23_P406105	*ARFGEF2*	ADP-ribosylation factor guanine nucleotide-exchange factor 2 (brefeldin A-inhibited) (ARFGEF2) [NM_006420]	0.50927895	0.030385435
A_22_P00015513	*GCN1L1*	GCN1 general control of amino-acid synthesis 1-like 1 (yeast) (GCN1L1)[NM_006836]	1.3228894	0.03912857
A_23_P40039	*LOC101929294*	uncharacterized LOC101929294 (LOC101929294) [NR_125808]	-0.8607179	0.029642703
A_32_P209094	*AMMECR1L*	AMMECR1-like (AMMECR1L) [NM_031445]	-0.879281	0.026578737
A_33_P3263851	*FGGY*	FGGY carbohydrate kinase domain containing (FGGY)[NM_018291]	-0.46970862	0.028542664
A_33_P3405946	*LOC400863*	uncharactered LOC400863 (LOC400863)[NM_001288961]	1.2577894	0.033091284
A_33_P3319261	*RBM39*	RNA binding motif protein 39 (RBM39) [NM_184234]	-1.092315	0.028573772
A_23_P156319	*EXD3*	exonuclease 3'-5' domain containing 3 (EXD3) [NM_001286823]	-1.3751755	0.03436134
A_33_P3274428	*LARP1*	La ribonucleoprotein domain family, member 1 (LARP1) [NM_015315]	0.7612712	0.03403978
A_23_P124427	*PPP1R12B*	protein phosphatase 1, regulatory subunit 12B (PPP1R12B) [NM_002481]	-1.1655188	0.03709602
A_33_P3420655	*NEK1*	NIMA-related kinase 1 (NEK1) [NM_012224]	-1.0068262	0.032155342
A_33_P3423859	*KDM4A*	lysine (K)-specific demethylase 4A (KDM4A)[NM_014663]	-0.8483445	0.030293303
A_24_P332623	*PPP1R3D*	protein phosphatase 1, regulatory subunit 3D (PPP1R3D) [NM_006242]	-0.99876064	0.049065307

A_33_P3333985	*CTAGE11P*	CTAGE family, member 11, pseudogene (CTAGE11P) [NR_027466]	-1.4322133	0.028619371
A_33_P3732466	*lnc-PRAGMIN.1-3*	LNCipedia lincRNA (lnc-PRAGMIN.1-3) [lnc-PRAGMIN.1-3:3]	-1.1944908	0.030915326
A_24_P32118	*LOC100506282*	PREDICTED: uncharacterized LOC100506282 (LOC100506282) [XR_110899]	-0.832154	0.047886103
A_24_P250535	*ZFYVE26*	zinc finger, FYVE domain containing 26 (ZFYVE26) [NM_015346]	-0.73099	0.037221838
A_33_P3372666	*TMX4*	thioredoxin-related transmembrane protein 4 (TMX4) [NM_021156]	-1.2216281	0.02737376
A_23_P62920	*PDGFA*	platelet-derived growth factor alpha polypeptide (PDGFA) [NM_033023]	-1.0752507	0.034607448
A_33_P3329187	*KIFAP3*	kinesin-associated protein 3 (KIFAP3) [NM_014970]	-0.62758666	0.045638688
A_23_P74950	*DNMT1*	DNA (cytosine-5-)-methyltransferase 1 (DNMT1) [NM_001130823]	-1.1671332	0.042529937
A_23_P503182	*RCC2*	regulator of chromosome condensation 2 (RCC2) [NM_018715]	-1.8023428	0.037383392
A_33_P3323068	*ABR*	active BCR-related (ABR) [NM_021962]	-0.84304357	0.042693015
A_21_P0007126	*AGPAT4*	1-acylglycerol-3-phosphate O-acyltransferase 4 (AGPAT4) [NM_020133]	1.1598432	0.04041675
A_21_P0012245	*ELMOD2*	ELMO/CED-12 domain containing 2 (ELMOD2) [NM_153702]	-0.42838523	0.03616732
A_23_P63050	*CECR7*	cat eye syndrome chromosome region, candidate 7 (non-protein coding) (CECR7) [NR_015352]	-0.8164116	0.03136619
A_23_P105873	*UROD*	uroporphyrinogen decarboxylase (UROD) [NM_000374]	-1.8259219	0.037560765
A_23_P151662	*TRPC4*	transient receptor potential cation channel, subfamily C, member 4 (TRPC4) [NM_016179]	-1.1010083	0.0420299
A_24_P109652	*MAX*	MYC associated factor X (MAX) [NM_197957]	-0.83303094	0.031899918
A_23_P117782	*PEAK1*	pseudopodium-enriched atypical kinase 1 (PEAK1) [NM_024776]	-0.8788882	0.03668808
A_22_P00021779	*LARP6*	La ribonucleoprotein domain family, member 6 (LARP6) [NM_018357]	-4.371522	0.027942823
A_19_P00317653	*lnc-C16orf42-1*	LNCipedia lincRNA (lnc-C16orf42-1) [lnc-C16orf42-1:2]	-0.30086222	0.02695598
A_23_P37441	*CAND1*	cullin-associated and neddylation-dissociated 1 [ENST00000545606]	-1.0940387	0.02737376
A_33_P3249773	*B2M*	beta-2-microglobulin (B2M) [NM_004048]	2.2595778	0.025407597
A_24_P300777	*TATDN3*	TatD DNase domain containing 3 (TATDN3) [NM_001146171]	0.715005	0.042775135

A_23_P120048	*ADAM8*	ADAM metallopeptidase domain 8 (ADAM8) [NM_001109]	-1.2087631	0.042304065
A_23_P100203	*BAZ2B*	bromodomain adjacent to zinc finger domain, 2B (BAZ2B) [NM_013450]	-1.5093763	0.024948223
A_22_P00004432	*HSBP1*	heat shock factor binding protein 1 (HSBP1) [NM_001537]	-0.84279555	0.047982622
A_23_P40217	*lnc-CORO6-1*	full length insert cDNA clone YW04H08. [AF086010]	1.7617254	0.040555127
A_23_P32861	*DOK5*	docking protein 5 (DOK5) [NM_018431]	-0.59540296	0.033091284
A_32_P161913	*NMD3*	NMD3 ribosome export adaptor (NMD3) [NM_015938]	-1.6338837	0.03403978
A_23_P409386	*TMEM240*	transmembrane protein 240 (TMEM240) [NM_001114748]	1.8942363	0.03032242
A_23_P100486	*SLC25A22*	solute carrier family 25 (mitochondrial carrier: glutamate), member 22 (SLC25A22) [NM_024698]	-1.5109173	0.033091284
A_23_P113972	*VKORC1*	vitamin K epoxide reductase complex, subunit 1 (VKORC1) [NM_206824]	-1.2012869	0.04792962
A_33_P3825869	*EXOC1*	exocyst complex component 1 (EXOC1)[NM_001024924]	-1.0941734	0.04797014
A_33_P3318946	*CACNA1C*	calcium channel, voltage-dependent, L type, alpha 1C subunit (CACNA1C) [NM_199460]	1.6618383	0.027751032
A_33_P3303086	*HAPLN2*	hyaluronan and proteoglycan link protein 2 (HAPLN2) [NM_021817]	-0.54645306	0.047137905
A_24_P944458	*HNRNPLL*	heterogeneous nuclear ribonucleoprotein L-like (HNRNPLL) [NM_138394]	-1.1782854	0.032893
A_24_P850428	*INSIG2*	insulin induced gene 2 (INSIG2)[NM_016133]	-0.4996171	0.03946874
A_24_P323941	*ZBTB44*	zinc finger and BTB domain containing 44 (ZBTB44) [NM_014155]	-0.6923539	0.028573772
A_23_P211631	*FAM209A*	family with sequence similarity 209, member A (FAM209A) [NM_001012971]	-2.09823	0.036131933
A_33_P3402565	*FBLN1*	fibulin 1 (FBLN1) [NM_006486]	-0.53333354	0.042693015
A_23_P62932	*DSP*	desmoplakin (DSP) [NM_004415]	-1.576618	0.03808641
A_23_P138253	*ATP1B1*	ATPase, Na+/K+ transporting, beta 1 polypeptide (ATP1B1) [NM_001677]	0.7650663	0.04219537
A_23_P317105	*CHTOP*	chromatin target of PRMT1 (CHTOP) [NM_015607]	-1.1425638	0.03639509
A_21_P0011303	*AKAP10*	A kinase (PRKA) anchor protein 10 (AKAP10) [NM_007202]	-1.8122928	0.045006614
A_23_P252913	*PDCD6IPP2*	PDCD6IP pseudogene 2 (PDCD6IPP2) [NR_037599]	-1.0705812	0.04638335

A_23_P24987	*BBS7*	Bardet-Biedl syndrome 7 (BBS7) [NM_018190]	-0.79530984	0.04973461
A_23_P308954	*TSPAN31*	tetraspanin 31 (TSPAN31) [NM_005981]	-1.0512155	0.02453978
A_33_P3249349	*BHLHB9*	basic helix-loop-helix domain containing, class B, 9 (BHLHB9) [NM_030639]	-0.97301024	0.034342222
A_33_P3216664	*PRAME*	preferentially expressed antigen in melanoma[ENST00000406503]	-1.191832	0.04159686
A_24_P296508	*LOC151174*	uncharacterized LOC151174 (LOC151174) [NR_026925]	-1.1781763	0.035700265
A_33_P3304963	*SLC43A2*	solute carrier family 43 (amino acid system L transporter), member 2 (SLC43A2) [NM_152346]	-1.4311596	0.030915326
A_23_P416142	*LRRC27*	leucine rich repeat containing 27 (LRRC27) [NM_001143759]	1.5702844	0.028573772
A_23_P351837	*DLG1*	discs, large homolog 1 (Drosophila) (DLG1) [NM_004087]	3.451889	0.03718595
A_32_P163169	*KLHL35*	kelch-like family member 35 (KLHL35)[NM_001039548]	0.79711646	0.033867702
A_23_P355623	*VDAC1*	voltage-dependent anion channel 1 (VDAC1) [NM_003374]	-1.9721086	0.03974276
A_23_P83818	*ANKIB1*	ankyrin repeat and IBR domain containing 1 (ANKIB1) [NM_019004]	-1.1699191	0.047591254
A_21_P0012112	*COL5A1*	collagen, type V, alpha 1 (COL5A1) [NM_000093]	-1.4778587	0.024948223
A_33_P3356910	*XLOC_l2_008560*	BROAD Institute lincRNA (XLOC_l2_008560) [TCONS_l2_00016171]	-1.7785442	0.049413078
A_33_P3338733	*TCEAL6*	transcription elongation factor A (SII)-like 6 (TCEAL6) [NM_001006938]	-1.3916931	0.046680246
A_21_P0006730	*MITF*	microphthalmia-associated transcription factor (MITF) [NM_198159]	-1.945363	0.044610508
A_22_P00004878	*LOC101929988*	PREDICTED: uncharacterized LOC101929988 (LOC101929988) [XR_253363]	-0.8928487	0.03912857
A_23_P26522	*TMED10*	transmembrane emp24-like trafficking protein 10 (yeast) (TMED10) [NM_006827]	2.4737835	0.03205687
A_23_P310086	*AQP8*	aquaporin 8 (AQP8)[NM_001169]	1.0717032	0.035560552
A_33_P3367830	*BEND6*	BEN domain containing 6 (BEND6)[NM_152731]	-1.0343643	0.038904693
A_23_P111288	*EFEMP2*	EGF containing fibulin-like extracellular matrix protein 2 (EFEMP2) [NM_016938]	-1.2062018	0.026808027

A_21_P0006725	*TMEM14C*	transmembrane protein 14C (TMEM14C) [NM_016462]	1.172533	0.046680246
A_23_P94128	*LINC00702*	long intergenic non-protein coding RNA 702 [ENST00000454470]	-1.3065861	0.02598378
A_33_P3396473	*NEIL2*	nei endonuclease VIII-like 2 (E. coli) (NEIL2) [NM_145043]	-0.7490241	0.030915326
A_23_P258978	*PPP6R2*	protein phosphatase 6, regulatory subunit 2 (PPP6R2)[NM_001242898]	-0.58315563	0.042994436
A_24_P944049	*GOLGA1*	golgin A1 (GOLGA1) [NM_002077]	-0.80762446	0.034210585
A_24_P299474	*CEP68*	centrosomal protein 68kDa (CEP68) [NM_015147]	0.97485435	0.04462891
A_22_P00005585	*TENM2*	teneurin transmembrane protein 2 (TENM2) [NM_001122679]	-0.53417903	0.031606767
A_33_P3565787	*SLC50A1*	solute carrier family 50 (sugar efflux transporter), member 1 [ENST00000506037]	-1.0085223	0.037804034
A_24_P925664	*PSMG3-AS1*	PSMG3 antisense RNA 1 (head to head) (PSMG3-AS1) [NR_027329]	-1.4861087	0.04488468
A_24_P860797	*MDM2*	MDM2 proto-oncogene, E3 ubiquitin protein ligase (MDM2) [NM_002392]	-3.5016053	0.029573848
A_22_P00003512	*PAIP2B*	poly(A) binding protein interacting protein 2B (PAIP2B) [NM_020459]	0.7988106	0.04542028
A_24_P379165	*lnc-CCL1-1*	BX106859 Soares_testis_NHT [BX106859]	-1.3175871	0.029573848
A_33_P3224595	*FOXO4*	forkhead box O4 (FOXO4)[NM_005938]	-1.0640411	0.027751032
A_24_P23258	*OFD1*	oral-facial-digital syndrome 1 (OFD1) [NM_003611]	-1.5284042	0.041768707
A_33_P3335725	*GRAMD4*	GRAM domain containing 4 (GRAMD4) [NM_015124]	1.1714932	0.04831641
A_23_P121356	*INSR*	insulin receptor (INSR) [NM_000208]	-1.3212445	0.04881034
A_33_P3365392	*BBX*	bobby sox homolog (Drosophila) (BBX)[NM_020235]	-1.4303585	0.04825923
A_23_P120254	*LOC401557*	uncharacterized LOC401557 (LOC401557) [NR_046107]	-1.1867139	0.04934116
A_23_P323166	*DUSP22*	dual specificity phosphatase 22 (DUSP22) [NM_020185]	-1.055877	0.027942823
A_23_P80122	*SRRM2*	serine/arginine repetitive matrix 2 (SRRM2) [NM_016333]	-0.5203553	0.030589065
A_33_P3393836	*WRB*	tryptophan rich basic protein (WRB) [NM_004627]	1.1440845	0.04551316
A_33_P3831566	*NT5C3A*	5'-nucleotidase, cytosolic IIIA (NT5C3A) [NM_001166118]	-0.77528214	0.033853088
A_33_P3347928	*FPGT*	fucose-1-phosphate guanylyltransferase (FPGT) [NM_003838]	-1.3050127	0.048182435

A_33_P3262012	*CCNL1*	cyclin L1 (CCNL1)[NM_020307]	-0.60496974	0.048019953
A_23_P503200	*LINC00704*	long intergenic non-protein coding RNA 704 (LINC00704) [NR_024475]	-0.7284514	0.034698997
A_21_P0009450	*PHF10*	PHD finger protein 10 (PHF10) [NM_018288]	0.48516768	0.04135566
A_21_P0000367	*lnc-LRRC30-1*	LNCipedia lincRNA (lnc-LRRC30-1) [lnc-LRRC30-1:1]	-0.9077923	0.030293303
A_23_P105747	*SNORA32*	small nucleolar RNA, H/ACA box 32 (SNORA32) [NR_003032]	3.7874317	0.042693015
A_33_P7699408	*APPL2*	adaptor protein, phosphotyrosine interaction, PH domain and leucine zipper containing 2 (APPL2) [NM_018171]	-1.9413466	0.04159686
A_23_P8311	*LOC101928973*	uncharacterized LOC101928973 (LOC101928973) [NR_125966]	2.0905733	0.04217344
A_23_P155332	*TDP2*	tyrosyl-DNA phosphodiesterase 2 (TDP2) [NM_016614]	-1.1908939	0.036667652
A_33_P3461039	*PCNP*	PEST proteolytic signal containing nuclear protein (PCNP) [NM_020357]	-1.5675522	0.03403978
A_23_P208325	*lnc-MTERFD3-1*	cDNA FLJ31150 fis, clone IMR322001534. [AK055712]	1.0696341	0.033091284
A_33_P3268284	*ZNF235*	zinc finger protein 235 (ZNF235) [NM_004234]	-1.2431273	0.039574593
A_33_P3290914	*GDI2*	GDP dissociation inhibitor 2 (GDI2) [NM_001494]	-0.7135846	0.046497274
A_23_P65870	*SMC1A*	structural maintenance of chromosomes 1A (SMC1A) [NM_001281463]	-0.8841492	0.047832783
A_21_P0013806	*FBXO22*	F-box protein 22 (FBXO22) [NM_012170]	-0.7477438	0.04591278
A_23_P7697	*HADHB*	hydroxyacyl-CoA dehydrogenase/3-ketoacyl-CoA thiolase/enoyl-CoA hydratase [NM_001281513]	-1.2500777	0.03286765
A_23_P127128	*SNX2*	sorting nexin 2 (SNX2) [NM_003100]	-1.2625936	0.012632321
A_24_P212531	*DNAJC1*	DnaJ (Hsp40) homolog, subfamily C, member 1 (DNAJC1) [NM_022365]	-0.75099254	0.04368986
A_24_P219785	*B3GALNT2*	beta-1,3-N-acetylgalactosaminyltransferase 2 (B3GALNT2)[NM_152490]	-1.2301533	0.02737376
A_24_P370670	*CALM3*	calmodulin 3 (phosphorylase kinase, delta) (CALM3)[NM_005184]	-1.2475858	0.04817637
A_21_P0012391	*ZMYM6NB*	ZMYM6 neighbor (ZMYM6NB) [NM_001195156]	-1.4819375	0.028546385
A_23_P27346	*MIR31HG*	MIR31 host gene (non-protein coding) (MIR31HG) [NR_027054]	-0.83690906	0.04971531

A_24_P110983	*SMAD4*	SMAD family member 4 (SMAD4) [NM_005359]	-1.3541607	0.041872468
A_22_P00003295	*AKT3*	v-akt murine thymoma viral oncogene homolog 3 (AKT3) [NM_005465]	-1.4969263	0.024948223
A_33_P3357651	*STARD4-AS1*	STARD4 antisense RNA 1 (STARD4-AS1) [NR_040093]	0.42192483	0.04705478
A_23_P152356	*KRTAP10-12*	keratin associated protein 10-12 (KRTAP10-12) [NM_198699]	-1.1913211	0.045638688
A_24_P753161	*ZNF200*	zinc finger protein 200 (ZNF200) [NM_003454]	-1.1345193	0.031377234
A_23_P142310	*BMPR2*	bone morphogenetic protein receptor, type II (serine/threonine kinase) (BMPR2) [NM_001204]	-2.6138196	0.031899918
A_23_P205200	*MKNK2*	MAP kinase interacting serine/threonine kinase 2 (MKNK2) [NM_017572]	0.4518954	0.029808238
A_23_P7221	*DHRS12*	dehydrogenase/reductase (SDR family) member 12 (DHRS12) [NM_024705]	-0.97844505	0.042693015
A_23_P62967	*RPL34*	ribosomal protein L34 (RPL34) [NM_033625]	-1.1400527	0.035560552
A_32_P99902	*DISC1*	disrupted in schizophrenia 1 (DISC1) [NM_018662]	-1.5074449	0.038287252
A_23_P203391	*C15orf40*	chromosome 15 open reading frame 40 (C15orf40) [NM_144597]	-1.7222502	0.03136619
A_22_P00024094	*ASRGL1*	asparaginase like 1 (ASRGL1) [NM_001083926]	-0.9666238	0.042496953
A_23_P30495	*lnc-BEST4-1*	LNCipedia lincRNA (lnc-BEST4-1), lincRNA [lnc-BEST4-1:2]	-2.3629045	0.041277196
A_24_P179351	*HMGCR*	3-hydroxy-3-methylglutaryl-CoA reductase (HMGCR)[NM_000859]	-0.49181408	0.047831167
A_24_P152468	*TPT1*	tumor protein, translationally-controlled 1 (TPT1) [NM_003295]	1.6661806	0.031091278
A_23_P51397	*LOC100128364*	PREDICTED: uncharacterized LOC100128364 (LOC100128364) [XR_108776]	-0.76027566	0.042440254
A_33_P3622802	*ENAH*	enabled homolog (Drosophila) (ENAH) [NM_001008493]	-0.9605767	0.046680246
A_33_P3228385	*ABI1*	abl-interactor 1 (ABI1) [NM_005470]	-0.9896579	0.045638688
A_24_P105298	*ATPIF1*	ATPase inhibitory factor 1 (ATPIF1) [NM_016311]	1.3405317	0.030589065
A_24_P228667	*H3F3A*	H3 histone, family 3A (H3F3A) [NM_002107]	-0.86928225	0.029088594
A_23_P14636	*MRPL40*	mitochondrial ribosomal protein L40 (MRPL40) [NM_003776]	-1.8221754	0.024948223
A_23_P93938	*MFAP1*	microfibrillar-associated protein 1 (MFAP1) [NM_005926]	-0.7146031	0.047932126

A_23_P110606	*NACAD*	NAC alpha domain containing (NACAD) [NM_001146334]	-0.4580104	0.031377234
A_33_P3413987	*MAML1*	mastermind-like 1 (Drosophila) (MAML1) [NM_014757]	-0.5459103	0.049986046
A_24_P411186	*SERPING1*	serpin peptidase inhibitor, clade G (C1 inhibitor), member 1 (SERPING1) [NM_000062]	-0.76456165	0.042693015
A_23_P19313	*BCL11A*	B-cell CLL/lymphoma 11A (zinc finger protein) (BCL11A) [NM_022893]	-0.9592931	0.035935212
A_22_P00017999	*TBP*	TATA box binding protein (TBP)[NM_003194]	-1.287248	0.042140156
A_24_P397584	*lnc-ZNF132-1*	cDNA FLJ38721 fis, clone KIDNE2010052. [AK096040]	-2.2428904	0.048681058
A_22_P00008745	*TBCC*	tubulin folding cofactor C (TBCC) [NM_003192]	-1.8130152	0.033583984
A_24_P98277	*TRAF3IP2-AS1*	TRAF3IP2 antisense RNA 1 (TRAF3IP2-AS1) [NR_034108]	-0.79554445	0.04877656
A_22_P00016954	*GOLGA7*	golgin A7 (GOLGA7) [NM_001002296]	-0.78078836	0.030915326
A_22_P00010214	*lnc-TSPYL6-2*	LNCipedia lincRNA (lnc-TSPYL6-2), lincRNA [lnc-TSPYL6-2:1]	-0.7397418	0.041275464
A_23_P3204	*lnc-NR5A2-1*	LNCipedia lincRNA (lnc-NR5A2-1), lincRNA [lnc-NR5A2-1:1]	-0.6974091	0.035560552
A_23_P114057	*MAPK6*	mitogen-activated protein kinase 6 (MAPK6) [NM_002748]	1.4096828	0.036667652
A_23_P340318	*SEMA4C*	sema domain, immunoglobulin domain (Ig), transmembrane domain (TM) and short cytoplasmic domain, (semaphorin) 4C (SEMA4C) [NM_017789]	1.2161268	0.026208585
A_23_P500601	*C11orf31*	chromosome 11 open reading frame 31 (C11orf31) [NM_170746]	2.6677315	0.032261204
A_23_P164674	*TRIM4*	tripartite motif containing 4 (TRIM4)[NM_033017]	-1.0913408	0.027942823
A_33_P3377110	*ZNF225*	zinc finger protein 225 (ZNF225) [NM_013362]	-0.35872602	0.046129715
A_22_P00000427	*CYB561D1*	cytochrome b561 family, member D1 (CYB561D1) [NM_001134404]	-0.5615666	0.03439591
A_24_P235305	*BOLA3-AS1*	BOLA3 antisense RNA 1 (head to head) (BOLA3-AS1) RNA [NR_045637]	-0.77091545	0.04784006
A_22_P00024468	*ZNF706*	zinc finger protein 706 (ZNF706) [NM_001042510]	-0.6719254	0.028573772
A_23_P500271	*lnc-CTNNA2-1*	1UV0_A Chain A, Pancreatitis-Associated Protein 1 From Human. {Homo sapiens} (exp=-1; wgp=0; cg=0) [THC2550001]	-1.1750287	0.040277198
A_23_P168592	*IRF5*	interferon regulatory factor 5 (IRF5) [NM_001098627]	0.8074669	0.028178396

A_23_P390172	*CCDC126*	coiled-coil domain containing 126 (CCDC126) [NM_138771]	-0.91311	0.029808238
A_32_P160883	*RNASEL*	ribonuclease L (2',5'-oligoisoadenylate synthetase-dependent) (RNASEL) [NM_021133]	1.9636431	0.04229927
A_23_P133345	*NEDD4*	neural precursor cell expressed, developmentally down-regulated 4, E3 ubiquitin protein ligase (NEDD4) [NM_198400]	1.6341454	0.037395623
A_33_P3262890	*CLINT1*	clathrin interactor 1 (CLINT1) [NM_014666]	-1.191639	0.034549404
A_23_P155103	*SERBP1*	SERPINE1 mRNA binding protein 1 (SERBP1)[NM_001018067]	-1.4432788	0.04531519
A_24_P312325	*ADSL*	adenylosuccinate lyase (ADSL) [NM_000026]	-0.4580457	0.029808238
A_23_P47991	*lnc-AF131215.3.1-1*	mRNA for hypothetical protein (C8ORF15). [AJ312027]	-0.85508156	0.044664014
A_33_P3258041	*MED13L*	mediator complex subunit 13-like (MED13L) [NM_015335]	-1.1907923	0.02737376
A_22_P00017678	*GGPS1*	geranylgeranyl diphosphate synthase 1 (GGPS1) [NM_001037277]	-0.9000025	0.049065307
A_33_P3392192	*LOC101927885*	PREDICTED: uncharacterized LOC101927885 (LOC101927885) [XR_245288]	1.123713	0.031377234
A_32_P16258	*LINC00856*	long intergenic non-protein coding RNA 856 [ENST00000510550]	-1.9410237	0.035278592
A_22_P00003276	*EXOC6B*	exocyst complex component 6B [ENST00000272427]	-3.8783503	0.030915326
A_32_P23624	*lnc-CALML5-1*	DC396132 TESTI2 cDNA clone TESTI2015443 5' [DC396132]	-2.1965373	0.027751032
A_23_P434430	*ABCB10*	ATP-binding cassette, sub-family B (MDR/TAP), member 10 (ABCB10) [NM_012089]	0.984067	0.03126305
A_33_P3264612	*ZNF439*	zinc finger protein 439 (ZNF439) [NM_152262]	-1.0190071	0.03403978
A_32_P67259	*TPCN2*	two pore segment channel 2 (TPCN2) [NM_139075]	0.9423687	0.030294474
A_33_P3272165	*SDHA*	succinate dehydrogenase complex, subunit A, flavoprotein (Fp) (SDHA) [NM_004168]	-1.8750243	0.028573772
A_23_P130169	*NDUFAF6*	NADH dehydrogenase (ubiquinone) complex I, assembly factor 6 (NDUFAF6) [NM_152416]	-0.9039238	0.028573772
A_23_P313031	*TBKBP1*	TBK1 binding protein 1 (TBKBP1) [NM_014726]	-0.8439059	0.028573772
A_22_P00025022	*LOC101930506*	PREDICTED: uncharacterized LOC101930506 (LOC101930506) [XR_249340]	-0.6891949	0.049758088

A_33_P3275290	*lnc-LRRC49-2*	LNCipedia lincRNA (lnc-LRRC49-2)[lnc-LRRC49-2:1]	-0.5400675	0.030589065
A_23_P31109	*GLT8D1*	glycosyltransferase 8 domain containing 1 (GLT8D1) [NM_001010983]	0.76656854	0.04733987
A_32_P4626	*GINM1*	glycoprotein integral membrane 1 (GINM1) [NM_138785]	1.7821137	0.027751032
A_23_P306507	*LOC101928076*	PREDICTED: uncharacterized LOC101928076 (LOC101928076) [XR_241970]	1.8019952	0.037765756
A_23_P404481	*KRAS*	Kirsten rat sarcoma viral oncogene homolog (KRAS)[NM_033360]	-1.1004884	0.03912857
A_33_P3370787	*S1PR1*	sphingosine-1-phosphate receptor 1 (S1PR1) [NM_001400]	-0.7771359	0.04864667
A_23_P101551	*EPHB2*	EPH receptor B2 (EPHB2) [NM_004442]	-2.3048806	0.042496953
A_33_P3305208	*BCAT2*	branched chain amino-acid transaminase 2, mitochondrial (BCAT2) [NM_001190]	-1.9215724	0.024948223
A_33_P3227731	*PPOX*	protoporphyrinogen oxidase (PPOX) [NM_000309]	2.0878391	0.04713994
A_33_P3309501	*FGF1*	fibroblast growth factor 1 (acidic) (FGF1) [NM_000800]	-0.888967	0.029524941
A_22_P00023959	*MEG3*	maternally expressed 3 (non-protein coding) [ENST00000398460]	-0.8767933	0.04089562
A_33_P3382924	*lnc-SZT2-1*	Q8CQ43_STAES (Q8CQ43) Teichoic acid biosynthesis protein, partial (6%) [THC2545859]	-1.1214001	0.038287252
A_33_P3325018	*SPARC*	secreted protein, acidic, cysteine-rich (osteonectin) (SPARC) [NM_003118]	-1.5456142	0.042296566
A_23_P21134	*ERLEC1*	endoplasmic reticulum lectin 1 (ERLEC1) [NM_015701]	0.8728695	0.036345065
A_33_P3234667	*DDIT3*	DNA-damage-inducible transcript 3 (DDIT3) [NM_004083]	1.0636153	0.042994436
A_24_P945113	*ZKSCAN1*	zinc finger with KRAB and SCAN domains 1 (ZKSCAN1) [NM_001287054]	-1.6962512	0.026721999
A_21_P0000034	*ACVRL1*	activin A receptor type II-like 1 (ACVRL1)[NM_000020]	-0.4029691	0.045638688
A_23_P83278	*COX11*	COX11 cytochrome c oxidase copper chaperone (COX11) [NM_004375]	-2.3125882	0.024948223
A_24_P56130	*CHMP5*	charged multivesicular body protein 5 (CHMP5) [NM_016410]	-0.9096255	0.03436134
A_23_P205255	*MYL6*	myosin, light chain 6, alkali, smooth muscle and non-muscle (MYL6) [NM_079423]	-1.920497	0.027751032
A_23_P81650	*WDR20*	WD repeat domain 20 (WDR20) [NM_001242417]	-1.1166899	0.047996294

A_33_P7718819	*C5orf15*	chromosome 5 open reading frame 15 (C5orf15) [NM_020199]	-0.8523302	0.047094904
A_24_P174367	*EXOSC2*	exosome component 2 (EXOSC2) [NM_014285]	-0.9664313	0.036427937
A_23_P94636	*PPP1R2*	protein phosphatase 1, regulatory (inhibitor) subunit 2 (PPP1R2) [NM_006241]	-0.6590543	0.04217344
A_33_P3390032	*RC3H2*	ring finger and CCCH-type domains 2 (RC3H2)[NM_018835]	-0.8445749	0.03503775
A_33_P3349474	*EXOC7*	exocyst complex component 7 (EXOC7) [NM_001145297]	-0.8128085	0.04135566
A_23_P128067	*TRAPPC2*	trafficking protein particle complex 2 (TRAPPC2) [NM_001011658]	-0.79662627	0.029573848
A_24_P318897	*RPL41*	ribosomal protein L41 (RPL41)[NM_001035267]	-0.82895845	0.045638688
A_24_P937855	*SNX21*	sorting nexin family member 21 (SNX21) [NM_001042633]	-0.32280988	0.029573848
A_24_P315184	*SIKE1*	suppressor of IKBKE 1 (SIKE1) [NM_001102396]	-1.E10892883224	0.043106858
A_24_P418809	*CSNK1A1*	casein kinase 1, alpha 1 [ENST00000412431]	-0.90638685	0.04253229
A_23_P349882	*GNAS*	GNAS complex locus (GNAS) [NM_001077489]	-0.8284637	0.031377234
A_23_P143551	*PDCL*	phosducin-like (PDCL) [NM_005388]	-0.97340965	0.031303514
A_23_P154500	*ATP6V1E1*	ATPase, H+ transporting, lysosomal 31kDa, V1 subunit E1 (ATP6V1E1)[NM_001696]	-0.95424813	0.024948223
A_24_P925314	*DNMT3A*	DNA (cytosine-5-)-methyltransferase 3 alpha (DNMT3A) [NM_175629]	-1.1047524	0.046256438
A_33_P3557274	*GM2A*	GM2 ganglioside activator (GM2A) [NM_000405]	-0.9788822	0.036667652
A_32_P104478	*LOC100996924*	PREDICTED: uncharacterized LOC100996924 (LOC100996924)[XM_003846648]	-0.9599766	0.035560552
A_23_P205778	*FGD6*	FYVE, RhoGEF and PH domain containing 6 (FGD6) [NM_018351]	-0.71546865	0.038283803
A_23_P27315	*GNB5*	guanine nucleotide binding protein (G protein), beta 5 (GNB5)[NM_016194]	-0.7822211	0.027751032
A_21_P0011675	*EMILIN2*	elastin microfibril interfacer 2 (EMILIN2) [NM_032048]	1.2240126	0.04041675
A_23_P146077	*PPP1R3C*	protein phosphatase 1, regulatory subunit 3C (PPP1R3C)[NM_005398]	-1.5554926	0.03811336
A_33_P3364869	*ZNF395*	zinc finger protein 395 (ZNF395) [NM_018660]	-1.6528983	0.03957154
A_22_P00017751	*NAMPT*	cDNA FLJ13279 fis, clone OVARC1001055, moderately similar to	-2.046718	0.04726856

		PRE-B CELL ENHANCING FACTOR PRECURSOR. [AK023341]		
A_33_P3374215	*lnc-YIF1A-3*	Q9BIU8_ARGTR (Q9BIU8) Flagelliform silk protein (Fragment), partial (3%) [THC2774970]	-1.2650867	0.047170673
A_33_P3225983	*PPP2R2D*	protein phosphatase 2, regulatory subunit B, delta (PPP2R2D) [NM_018461]	2.5086424	0.042440254
A_33_P3336484	*DUSP11*	dual specificity phosphatase 11 (RNA/RNP complex 1-interacting) (DUSP11) [NM_003584]	2.0422282	0.033198114
A_32_P181638	*NPIPA5*	nuclear pore complex interacting protein family, member A5 (NPIPA5) [NM_001277325]	-0.6971939	0.048247673
A_23_P94683	*BVES*	blood vessel epicardial substance (BVES) [NM_147147]	-1.750382	0.04434183
A_24_P63522	*NFX1*	nuclear transcription factor, X-box binding 1 (NFX1)[NM_002504]	-1.1724516	0.027751032
A_23_P143120	*HMGCS1*	3-hydroxy-3-methylglutaryl-CoA synthase 1 (soluble) (HMGCS1) [NM_002130]	-1.1839987	0.045743782
A_23_P11331	*ADAM17*	ADAM metallopeptidase domain 17 (ADAM17) [NM_003183]	-1.2839769	0.027942823
A_23_P144999	*TCEAL8*	transcription elongation factor A (SII)-like 8 (TCEAL8) [NM_153333]	-0.85626763	0.049413078
A_19_P00804417	*RAPGEF6*	Rap guanine nucleotide exchange factor (GEF) 6 (RAPGEF6) [NM_016340]	-1.2665156	0.03770023
A_33_P3329916	*ARHGEF12*	Rho guanine nucleotide exchange factor (GEF) 12 (ARHGEF12) [NM_015313]	-0.82735896	0.048398294
A_24_P98263	*RPL6*	ribosomal protein L6 (RPL6) [NM_001024662]	-1.4062979	0.04644307
A_23_P52127	*EBAG9*	estrogen receptor binding site associated, antigen, 9 (EBAG9) [NM_004215]	-1.3583434	0.028546385
A_24_P156576	*ACBD6*	acyl-CoA binding domain containing 6 (ACBD6) [NM_032360]	-0.49922583	0.03138659
A_23_P27367	*GEMIN8*	gem (nuclear organelle) associated protein 8 (GEMIN8) [NM_017856]	-0.3316937	0.04573629
A_24_P419087	*KDSR*	3-ketodihydrosphingosine reductase (KDSR) [NM_002035]	-0.7995832	0.028546385
A_19_P00322898	*AVIL*	advillin (AVIL) [NM_006576]	-1.297153	0.027751032
A_23_P67424	*CA5BP1*	carbonic anhydrase VB pseudogene 1 (CA5BP1) [NR_026551]	-1.5810817	0.040555127
A_23_P110846	*ZNF461*	zinc finger protein 461 (ZNF461) [NM_153257]	-0.9767754	0.029808238

A_23_P163278	*CNOT8*	CCR4-NOT transcription complex, subunit 8 (CNOT8) [NM_004779]	-1.5918677	0.04192822
A_24_P316074	*PARP16*	poly (ADP-ribose) polymerase family, member 16 (PARP16) [NM_017851]	-0.70566297	0.049281515
A_21_P0005567	*SIPA1L3*	signal-induced proliferation-associated 1 like 3 (SIPA1L3) [NM_015073]	-1.8171633	0.03138659
A_24_P312041	*lnc-LAT2-1*	ALU8_HUMAN (P39195) Alu subfamily SX sequence contamination warning entry, partial (18%) [THC2723591]	-1.5310072	0.046908576
A_24_P270890	*PLAGL1*	pleiomorphic adenoma gene-like 1 (PLAGL1)[NM_006718]	-0.79095554	0.030578624
A_23_P106002	*SPOP*	speckle-type POZ protein (SPOP) [NM_001007226]	1.664034	0.027751032
A_33_P3237487	*NFKBIA*	nuclear factor of kappa light polypeptide gene enhancer in B-cells inhibitor, alpha (NFKBIA) [NM_020529]	1.7294734	0.025407597
A_33_P3223472	*COL5A2*	s collagen, type V, alpha 2 (COL5A2) [NM_000393]	-1.3518991	0.034008626
A_23_P350551	*AMZ2*	archaelysin family metallopeptidase 2 (AMZ2)[NM_016627]	1.1218241	0.041931406
A_24_P159036	*C12orf57*	chromosome 12 open reading frame 57 (C12orf57) [NM_138425]	-0.9275486	0.045400333
A_33_P3269203	*RPL36AL*	ribosomal protein L36a-like (RPL36AL) [NM_001001]	-1.835777	0.047832783
A_23_P81241	*SERPINH1*	serpin peptidase inhibitor, clade H (heat shock protein 47), member 1, (collagen binding protein 1) (SERPINH1) [NM_001207014]	-1.0178571	0.042693015
A_32_P28939	*NDFIP1*	Nedd4 family interacting protein 1 (NDFIP1) [NM_030571]	-1.1569283	0.029808238
A_24_P70002	*ALKBH2*	alkB, alkylation repair homolog 2 (E. coli) (ALKBH2) [NM_001001655]	-1.1530191	0.02453978
A_23_P431319	*LATS2*	large tumor suppressor kinase 2 (LATS2) [NM_014572]	-1.4132967	0.034750238
A_33_P3287218	*YIPF6*	Yip1 domain family, member 6 (YIPF6), transcript variant A [NM_173834]	-1.0391827	0.046726856
A_23_P21838	*GSTK1*	glutathione S-transferase kappa 1 (GSTK1)[NM_001143679]	-0.84173346	0.049603622
A_19_P00801324	*CNP*	2',3'-cyclic nucleotide 3' phosphodiesterase (CNP)[NM_033133]	-1.5566368	0.034750238
A_23_P126037	*LINC01355*	long intergenic non-protein coding RNA 1355 (LINC01355) [NR_110616]	-0.7313246	0.042907383
A_23_P109345	*RLF*	rearranged L-myc fusion (RLF) [NM_012421]	-0.74690586	0.04590026
A_33_P3267296	*PTTG1IP*	pituitary tumor-transforming 1 interacting protein (PTTG1IP) [NM_004339]	-0.62050533	0.028573772

A_22_P00006750	*FKBP11*	FK506 binding protein 11, 19 kDa (FKBP11)[NM_016594]	-1.3524162	0.03590667
A_33_P3305472	*ZNFX1*	zinc finger, NFX1-type containing 1 (ZNFX1)[NM_021035]	-1.266109	0.03804464
A_23_P47565	*SYNGR1*	synaptogyrin 1 (SYNGR1)[NM_145731]	1.8652108	0.027751032
A_23_P320261	*LDHA*	lactate dehydrogenase A (LDHA) [NM_005566]	-0.94292337	0.037471827
A_23_P250380	*DMKN*	dermokine (DMKN) [NM_001035516]	1.4648541	0.04236895
A_21_P0011408	*MAN2B2*	mannosidase, alpha, class 2B, member 2 (MAN2B2) [NM_015274]	-1.8412454	0.04705478
A_24_P215765	*ULK4P3*	ULK4 pseudogene 3 (ULK4P3) [NR_026859]	-1.0540397	0.03510091
A_33_P3297205	*ATP10A*	ATPase, class V, type 10A (ATP10A)[NM_024490]	-1.6208001	0.046700664
A_21_P0010686	*C22orf29*	chromosome 22 open reading frame 29 (C22orf29) [NM_024627]	-1.2770456	0.0298189
A_24_P345498	*PIK3R2*	phosphoinositide-3-kinase, regulatory subunit 2 (beta) (PIK3R2) [NM_005027]	-1.2236068	0.044286337
A_23_P50008	*XRCC5*	X-ray repair complementing defective repair in Chinese hamster cells 5 (double-strand-break rejoining) (XRCC5) [NM_021141]	-1.4320986	0.02695598
A_23_P343935	*TTC19*	tetratricopeptide repeat domain 19 (TTC19)[NM_017775]	-0.90927327	0.046045035
A_24_P244952	*EGLN1*	egl-9 family hypoxia-inducible factor 1 (EGLN1) [NM_022051]	-0.5310652	0.027751032
A_24_P703830	*SMG1*	SMG1 phosphatidylinositol 3-kinase-related kinase (SMG1) [NM_015092]	-1.243057	0.04641682
A_33_P3398667	*NANOS3*	nanos homolog 3 (Drosophila) (NANOS3) [NM_001098622]	-0.7985482	0.047414985
A_33_P3212650	*ZSCAN30*	zinc finger and SCAN domain containing 30 (ZSCAN30) [NM_001166012]	-1.2854868	0.04439921
A_23_P134614	*HDDC2*	containing 2 [ENST00000609477]	-1.76949	0.046535324
A_21_P0011364	*COG5*	component of oligomeric golgi complex 5 (COG5) [NM_006348]	-1.2003453	0.027751032
A_33_P3322373	*WHAMMP1*	WAS protein homolog associated with actin, golgi membranes and microtubules pseudogene 1 (WHAMMP1) [NR_036650]	0.94278526	0.02737376
A_33_P3373707	*GBGT1*	globoside alpha-1,3-N-acetylgalactosaminyltransferase 1 (GBGT1) [NM_001282632]	-1.24643	0.027189178
A_24_P466374	*STX7*	syntaxin 7 (STX7) [NM_003569]	-0.8806708	0.049758088

A_23_P18824	*EYA3*	EYA transcriptional coactivator and phosphatase 3 (EYA3) [NM_001990]	-1.17292	0.033095628
A_23_P26254	*PAPD7*	PAP associated domain containing 7 (PAPD7) [NM_006999]	1.1991065	0.036041338
A_24_P911676	*NDUFAF1*	NADH dehydrogenase (ubiquinone) complex I, assembly factor 1 (NDUFAF1) [NM_016013]	-1.1502358	0.049271874
A_23_P98402	*SOX4*	SRY (sex determining region Y)-box 4 (SOX4) [NM_003107]	-0.41085786	0.02737376
A_24_P389916	*SIDT2*	SID1 transmembrane family, member 2 (SIDT2) [NM_001040455]	-0.81702375	0.03510091
A_33_P3389023	*LRRC32*	leucine rich repeat containing 32 (LRRC32)[NM_005512]	-0.8563721	0.027751032
A_23_P132669	*SRD5A3*	steroid 5 alpha-reductase 3 (SRD5A3)[NM_024592]	-1.1281523	0.045638688
A_23_P112311	*GLT8D1*	glycosyltransferase 8 domain containing 1 (GLT8D1) [NM_001010983]	-1.0610574	0.028192867
A_24_P356338	*TRIM32*	tripartite motif containing 32 (TRIM32) [NM_012210]	-2.838784	0.04103169
A_33_P3417260	*GABARAPL2*	GABA(A) receptor-associated protein-like 2 (GABARAPL2) [NM_007285]	-1.2151797	0.031377234
A_33_P3302796	*NMRK1*	nicotinamide riboside kinase 1 (NMRK1) [NM_017881]	-1.2969439	0.045638688
A_33_P3227375	*U2AF2*	U2 small nuclear RNA auxiliary factor 2 (U2AF2)[NM_007279]	-1.1672606	0.029808238
A_23_P60225	*THBS2*	thrombospondin 2 (THBS2), mRNA [NM_003247]	-0.9868285	0.036667652
A_23_P108657	*GRHPR*	glyoxylate reductase/hydroxypyruvate reductase (GRHPR) [NM_012203]	-1.5926361	0.03683928
A_23_P11279	*WDSUB1*	WD repeat, sterile alpha motif and U-box domain containing 1 (WDSUB1) [NM_152528]	0.75266093	0.047879543
A_21_P0007470	*ALG13*	ALG13, UDP-N-acetylglucosaminyltransferase subunit (ALG13) [NM_018466]	-2.0622501	0.046051342
A_33_P3240392	*lnc-BLID-1*	LNCipedia lincRNA (lnc-BLID-1), lincRNA [lnc-BLID-1:17]	-0.6152641	0.027019119
A_23_P169576	*FOXO3*	forkhead box O3 (FOXO3) [NM_001455]	-1.518693	0.03298269
A_23_P327426	*EXOC6*	exocyst complex component 6 (EXOC6) [NM_019053]	-0.78216046	0.03662822
A_33_P3386765	*TIPRL*	TOR signaling pathway regulator (TIPRL) [NM_152902]	-2.495742	0.02598378
A_23_P205875	*ABHD14A*	abhydrolase domain containing 14A (ABHD14A)NM_015407]	-0.8318972	0.028573772
A_23_P15582	*SCAND2P*	SCAN domain containing 2 pseudogene (SCAND2P) [NR_004859]	1.072977	0.043814994

A_33_P3336422	*XYLT2*	xylosyltransferase II (XYLT2) [NM_022167]	-0.9284723	0.034614928
A_22_P00012802	*ZBED5-AS1*	ZBED5 antisense RNA 1 (ZBED5-AS1) [NR_034137]	-1.5408254	0.02453978
A_24_P56194	*lnc-RANBP9-1*	cDNA clone IMAGE:4865340, partial cds. [BC064478]	-0.89885235	0.04208062
A_23_P390097	*CREBL2*	cAMP responsive element binding protein-like 2 (CREBL2) [NM_001310]	-0.62767667	0.038727812
A_24_P21056	*TTC39B*	tetratricopeptide repeat domain 39B (TTC39B)[NM_152574]	0.93571055	0.049413078
A_23_P29630	*PHF14*	PHD finger protein 14 (PHF14) [NM_014660]	1.0663252	0.04971531
A_33_P3211924	*SPCS1*	signal peptidase complex subunit 1 homolog (S. cerevisiae) (SPCS1) [NM_014041]	-1.2546548	0.04532058
A_33_P3404531	*RCOR1*	REST corepressor 1 (RCOR1) [NM_015156]	-1.455379	0.029808238
A_23_P165657	*ZCWPW1*	zinc finger, CW type with PWWP domain 1 (ZCWPW1) [NM_001258008]	-1.3605559	0.03811336
A_23_P215296	*SLC20A1*	solute carrier family 20 (phosphate transporter), member 1 (SLC20A1) [NM_005415]	-0.5174897	0.049235344
A_22_P00015589	*CDK13*	cyclin-dependent kinase 13 (CDK13)[NM_003718]	-0.7769413	0.038684342
A_19_P00812587	*STX6*	syntaxin 6 (STX6) [NM_005819]	-1.3930787	0.047941346
A_23_P79426	*RPL5*	ribosomal protein L5 (RPL5) [NM_000969]	0.70541334	0.046040986
A_24_P134834	*CAB39*	calcium binding protein 39 (CAB39) [NM_016289]	-0.3234894	0.034999274
A_33_P3235491	*RHBDD1*	rhomboid domain containing 1 (RHBDD1) [NM_032276]	-0.7546818	0.024948223
A_23_P99405	*SLC8B1*	solute carrier family 8 (sodium/lithium/calcium exchanger), member B1 (SLC8B1) [NM_024959]	-1.0614233	0.032893
A_23_P14948	*ZMYM2*	zinc finger, MYM-type 2 (ZMYM2) [NM_003453]	-0.52977675	0.046256438
A_19_P00325158	*MBTPS1*	membrane-bound transcription factor peptidase, site 1 (MBTPS1)[NM_003791]	0.7720442	0.032155342
A_23_P87011	*MALAT1*	metastasis associated lung adenocarcinoma transcript 1 (non-protein coding) (MALAT1) [NR_002819]	-0.96691585	0.031091278
A_24_P342086	*TAGLN*	transgelin (TAGLN)[NM_001001522]	-0.98650026	0.034210585

A_33_P3577120	*WWP2*	WW domain containing E3 ubiquitin protein ligase 2 (WWP2) [NM_001270455]	-0.54105663	0.03974276
A_24_P319715	*TBL3*	transducin (beta)-like 3 [Source:HGNC Symbol;Acc:HGNC:11587] [ENST00000568546]	1.1932806	0.0402784
A_32_P155506	*PDIA6*	protein disulfide isomerase family A, member 6 (PDIA6) [NM_005742]	-1.1884947	0.034210585
A_21_P0003931	*UBE2E2*	ubiquitin-conjugating enzyme E2E 2 (UBE2E2) [NM_152653]	-0.936647	0.045708336
A_32_P2738	*MIR143HG*	MIR143 host gene (non-protein coding) [Source:HGNC Symbol;Acc:HGNC:42872] [ENST00000505254]	0.80724484	0.04639083
A_33_P3250323	*TCTN3*	tectonic family member 3 (TCTN3) [NM_015631]	-1.3300548	0.030971156
A_33_P3384284	*TMLHE*	trimethyllysine hydroxylase, epsilon (TMLHE)[NM_018196]	0.32493067	0.02453978
A_23_P216307	*SUPT20HL1*	suppressor of Ty 20 homolog (S. cerevisiae)-like 1 (SUPT20HL1) [NM_001136234]	-1.2018528	0.026440267
A_21_P0010098	*RUNX1T1*	runt-related transcription factor 1; translocated to, 1 (cyclin D-related) (RUNX1T1) [NM_004349]	0.69419897	0.033412892
A_23_P1029	*lnc-C20orf166-2*	LNCipedia lincRNA (lnc-C20orf166-2) [lnc-C20orf166-2:1]	-1.0542747	0.03590667
A_23_P135730	*MFAP2*	microfibrillar-associated protein 2 (MFAP2) [NM_017459]	2.0365763	0.03716438
A_33_P3230976	*ZNF627*	zinc finger protein 627 (ZNF627) [NM_145295]	-1.4290291	0.027751032
A_23_P217778	*PREP*	prolyl endopeptidase (PREP) [NM_002726]	1.0502717	0.047727138
A_23_P502832	*MSL3*	male-specific lethal 3 homolog (Drosophila) (MSL3) [NM_078629]	-1.5983689	0.029808238
A_33_P3224795	*RBM12*	RNA binding motif protein 12 (RBM12) [NM_006047]	-1.6163789	0.048238702
A_24_P922631	*IKZF5*	IKAROS family zinc finger 5 (Pegasus) (IKZF5) [NM_001271840]	-0.47168177	0.045740407
A_33_P3244141	*C5orf58*	chromosome 5 open reading frame 58 (C5orf58) [NM_001102609]	-1.0792116	0.0442831
A_33_P3336652	*NDEL1*	nudE neurodevelopment protein 1-like 1 (NDEL1) [NM_001025579]	-0.6456592	0.0419793
A_23_P56746	*NDUFC2*	NADH dehydrogenase (ubiquinone) 1, subcomplex unknown, 2, 14.5kDa (NDUFC2) [NM_001204054]	-0.5600514	0.042656552
A_23_P56798	*FAP*	fibroblast activation protein, alpha (FAP)[NM_004460]	4.055725	0.04533123

A_23_P142830	*ACP1*	acid phosphatase 1, soluble (ACP1) [NM_004300]	-0.68243784	0.03754682
A_23_P168882	*PLA2R1*	phospholipase A2 receptor 1, 180kDa (PLA2R1)[NM_007366]	-1.2082182	0.03338082
A_24_P174341	*TP53INP1*	tumor protein p53 inducible nuclear protein 1 (TP53INP1)[NM_033285]	-1.2010782	0.04455951 6
A_23_P53614	*CCNT2*	cyclin T2 (CCNT2)[NM_058241]	-0.648216	0.04881034
A_33_P332105 0	*BRAP*	BRCA1 associated protein (BRAP) [NM_006768]	-1.0779623	0.0442831
A_24_P286079	*PHACTR2*	phosphatase and actin regulator 2 (PHACTR2) [NM_001100165]	-1.5543269	0.04792134
A_23_P69683	*AFF4*	AF4/FMR2 family, member 4 (AFF4) [NM_014423]	-1.1469814	0.03343686
A_23_P166421	*SEC24B*	SEC24 family member B (SEC24B) [NM_006323]	-0.7897373	0.04009100 4
A_24_P229025	*TBC1D10A*	TBC1 domain family, member 10A (TBC1D10A) [NM_031937]	-1.2648892	0.04876218
A_33_P322830 5	*GRIA3*	glutamate receptor, ionotropic, AMPA 3 (GRIA3) [NM_001256743]	-1.4030054	0.04269301 5
A_21_P000009 2	*ARHGAP26*	Rho GTPase activating protein 26 (ARHGAP26) [NM_015071]	-1.7126715	0.03872781 2
A_23_P352684	*ZBED6*	zinc finger, BED-type containing 6 (ZBED6), mRNA [NM_001174108]	-0.56712365	0.03878296 5
A_23_P99360	*DCAF5*	DDB1 and CUL4 associated factor 5 (DCAF5) [NM_003861]	-2.4062803	0.0442831
A_32_P120043	*TRIM13*	tripartite motif containing 13 (TRIM13) [NM_213590]	-4.0891986	0.04573629
A_23_P157022	*FAM26E*	family with sequence similarity 26, member E (FAM26E) [NM_153711]	-0.9236454	0.03084976 6
A_24_P942068	*ZNF786*	zinc finger protein 786 (ZNF786) [NM_152411]	-0.8433656	0.0442831
A_23_P2661	*TANC2*	tetratricopeptide repeat, ankyrin repeat and coiled-coil containing 2 (TANC2)[NM_025185]	-0.78355473	0.03421058 5
A_23_P40194	*RAP1B*	RAP1B, member of RAS oncogene family (RAP1B) [NM_015646]	-1.6011577	0.04641176
A_23_P161439	*DDX27*	DEAD (Asp-Glu-Ala-Asp) box polypeptide 27 (DDX27) [NM_017895]	-0.7993535	0.03800399 2
A_23_P137909	*ADIRF*	adipogenesis regulatory factor (ADIRF) [NM_006829]	-1.3469903	0.03403978
A_23_P89123	*HIST3H3*	histone cluster 3, H3 (HIST3H3) [NM_003493]	-0.87852	0.03421058 5
A_24_P390403	*CFDP1*	craniofacial development protein 1 (CFDP1) [NM_006324]	-0.41980553	0.04109702 6
A_23_P417942	*RTF1*	Rtf1, Paf1/RNA polymerase II complex component, homolog (S. cerevisiae) (RTF1) [NM_015138]	3.2053678	0.04850673 3

A_23_P58443	*FNBP1L*	formin binding protein 1-like (FNBP1L)[NM_001024948]	-0.69396925	0.024948223
A_24_P375599	*ANKHD1-EIF4EBP3*	ANKHD1-EIF4EBP3 readthrough (ANKHD1-EIF4EBP3) [NM_020690]	-1.3270171	0.029808238
A_23_P78438	*CRBN*	cereblon (CRBN) [NM_016302]	-2.4693832	0.030589065
A_21_P0014782	*ELP2*	elongator acetyltransferase complex subunit 2 (ELP2)[NM_018255]	-0.9629884	0.028573772
A_33_P3358626	*XLOC_l2_003758*	BROAD Institute lincRNA (XLOC_l2_003758) [TCONS_l2_00006892]	-1.2455941	0.02737376
A_33_P3262181	*TAF4B*	TAF4b RNA polymerase II, TATA box binding protein (TBP)-associated factor, 105kDa (TAF4B) [NM_001293725]	-0.8488035	0.035515364
A_23_P252775	*APOBEC3F*	apolipoprotein B mRNA editing enzyme, catalytic polypeptide-like 3F (APOBEC3F) [NM_001006666]	2.3789344	0.03403978
A_33_P3400758	*APTX*	aprataxin (APTX) [NM_175073]	1.618492	0.04015477
A_23_P153676	*ZNF555*	zinc finger protein 555 (ZNF555), transcript variant 1, mRNA [NM_152791]	-1.1942405	0.038180117
A_23_P370097	*TLE2*	transducin-like enhancer of split 2 (TLE2), transcript variant 1, mRNA [NM_003260]	-0.8484211	0.048618473
A_32_P162187	*TMEM237*	transmembrane protein 237 (TMEM237), transcript variant 1, mRNA [NM_001044385]	-0.40487453	0.04041675
A_23_P17880	*C2*	complement component 2 (C2), transcript variant 1, mRNA [NM_000063]	-1.2383864	0.031207845
A_33_P3247403	*DNAL4*	dynein, axonemal, light chain 4 (DNAL4), mRNA [NM_005740]	-1.633189	0.03273351
A_33_P3228837	*TOR3A*	torsin family 3, member A (TOR3A), mRNA [NM_022371]	-1.2699676	0.046256438
A_21_P0007110	*CD8A*	CD8a molecule (CD8A) [NM_001145873]	-1.882272	0.03668808
A_33_P3332130	*lnc-WAPAL-1*	LNCipedia lincRNA (lnc-WAPAL-1), lincRNA [lnc-WAPAL-1:1]	-0.97613	0.042884316
A_33_P3220728	*PGRMC1*	progesterone receptor membrane component 1 (PGRMC1)[NM_001282621]	0.6512856	0.047938656
A_23_P42738	*RREB1*	ras responsive element binding protein 1 (RREB1)[NM_001003699]	1.5387058	0.035220377
A_33_P3329508	*FAM220A*	family with sequence similarity 220, member A (FAM220A) [NM_001037163]	1.3376223	0.024948223

A_24_P377225	*LRRC27*	leucine rich repeat containing 27 (LRRC27) [NM_001143757]	-0.8733089	0.044286337
A_33_P3242863	*USP46*	ubiquitin specific peptidase 46 (USP46) [NM_022832]	-1.177133	0.034210585
A_23_P23542	*NT5M*	5',3'-nucleotidase, mitochondrial (NT5M)[NM_020201]	-1.532841	0.036044724
A_33_P3256113	*RPAP2*	RNA polymerase II associated protein 2 (RPAP2) [NM_024813]	1.316316	0.027942823
A_21_P0000040	*GUSBP1*	glucuronidase, beta pseudogene 1 (GUSBP1) [NR_027026]	-0.7997964	0.02737376
A_23_P310331	*ZBTB24*	zinc finger and BTB domain containing 24 (ZBTB24) [NM_001164313]	-1.4414525	0.032261204
A_22_P00015976	*RANBP3*	RAN binding protein 3 (RANBP3)[NM_003624]	-0.95838785	0.030578624
A_33_P3221528	*lnc-TENC1-1*	LNCipedia lincRNA (lnc-TENC1-1), lincRNA [lnc-TENC1-1:1]	-2.3006477	0.028546385
A_22_P00006907	*IREB2*	iron-responsive element binding protein 2 (IREB2) [NM_004136]	1.1237549	0.026339147
A_23_P59950	*SEC14L1*	SEC14-like 1 (S. cerevisiae) (SEC14L1)[NM_001204408]	1.3930945	0.027751032
A_23_P159382	*SLC39A14*	solute carrier family 39 (zinc transporter), member 14 (SLC39A14)[NM_015359]	-1.0627351	0.03028147
A_32_P80068	*SEC24D*	SEC24 family member D (SEC24D) [NM_014822]	1.4792598	0.034342222
A_23_P432947	*CLEC2D*	C-type lectin domain family 2, member D (CLEC2D) [NM_001004419]	0.41048253	0.031603426
A_33_P3243337	*GREM1*	gremlin 1, DAN family BMP antagonist (GREM1) [NM_013372]	-0.6888887	0.0442831
A_23_P147495	*MYDGF*	myeloid-derived growth factor (MYDGF) [NM_019107]	-1.5869044	0.042269725
A_23_P409623	*BCORL1*	BCL6 corepressor-like 1 (BCORL1) [NM_021946]	-1.0752537	0.045925915
A_21_P0007329	*PPFIBP2*	PTPRF interacting protein, binding protein 2 (liprin beta 2) (PPFIBP2) [NM_003621]	-2.235006	0.033433396
A_23_P15402	*lnc-CCDC34-1*	BX096431 Soares placenta Nb2HP [BX096431]	-0.6714568	0.038322687
A_23_P87973	*SAT2*	spermidine/spermine N1-acetyltransferase family member 2 (SAT2) [NM_133491]	-0.84598327	0.042905256
A_24_P83075	*TRIM13*	tripartite motif containing 13 (TRIM13) [NM_213590]	-0.50287306	0.04135566
A_23_P27724	*LINC00205*	PREDICTED: putative uncharacterized protein encoded by LINC00205-like (LOC102723489)[XM_006724071]	-1.8697556	0.03777906
A_33_P3289145	*SEPW1*	selenoprotein W, 1 (SEPW1) [NM_003009]	-1.012713	0.034306627

A_32_P122402	*COPS8*	COP9 signalosome subunit 8 (COPS8) [NM_198189]	-0.4002156	0.034210585
A_19_P00320948	*LOC441455*	makorin ring finger protein 1 pseudogene (LOC441455) [NR_026792]	1.9222522	0.02737376
A_21_P0009343	*FAM200B*	family with sequence similarity 200, member B [ENST00000507992]	-2.0341477	0.035769645
A_32_P486693	*TBX2-AS1*	TBX2 antisense RNA 1 (TBX2-AS1) [NR_125749]	-1.2005204	0.03889047
A_21_P0007382	*NRIP3*	nuclear receptor interacting protein 3 (NRIP3) [NM_020645]	-0.66372454	0.041768707
A_23_P159956	*lnc-GPR83-2*	LNCipedia lincRNA (lnc-GPR83-2) [lnc-GPR83-2:1]	-1.3614389	0.041768707
A_23_P49021	*MID2*	midline 2 (MID2)[NM_012216]	2.820403	0.040976647
A_23_P252052	*WDR61*	WD repeat domain 61 (WDR61) [NM_025234]	0.99344563	0.04881034
A_33_P3300212	*FILIP1L*	filamin A interacting protein 1-like (FILIP1L) [NM_182909]	1.2841655	0.048964206
A_23_P323898	*BLCAP*	bladder cancer associated protein (BLCAP) [NM_001167820]	-1.188858	0.03136619
A_33_P3357049	*ZSCAN12*	zinc finger and SCAN domain containing 12 (ZSCAN12) [NR_028077]	-1.3948822	0.038463872
A_23_P141146	*RPS15AP10*	ribosomal protein S15a pseudogene 10 (RPS15AP10) [NR_026768]	-1.9548764	0.034750238
A_23_P258944	*FBXL20*	F-box and leucine-rich repeat protein 20 (FBXL20) [NM_032875]	-0.69226533	0.02737376
A_23_P85164	*DNAJB9*	DnaJ (Hsp40) homolog, subfamily B, member 9 (DNAJB9) [NM_012328]	-0.46671975	0.04976886
A_23_P333218	*DNASE1L1*	deoxyribonuclease I-like 1 [ENST00000309585]	-1.0969431	0.031091278
A_33_P3296198	*ERGIC1*	endoplasmic reticulum-golgi intermediate compartment (ERGIC) 1 (ERGIC1) [NM_001031711]	-0.72757035	0.036129165
A_23_P74467	*C5orf63*	chromosome 5 open reading frame 63 (C5orf63) [NM_001164479]	-0.9235542	0.03912857
A_23_P216489	*KIAA0907*	KIAA0907 (KIAA0907) [NM_014949]	-2.0110497	0.042621672
A_23_P12866	*GNE*	glucosamine (UDP-N-acetyl)-2-epimerase/N-acetylmannosamine kinase (GNE) [NM_005476]	-0.5306466	0.031569958
A_33_P3344618	*PSTK*	phosphoseryl-tRNA kinase (PSTK) [NM_153336]	-0.5965437	0.04926936
A_24_P384755	*TFEB*	transcription factor EB (TFEB) [NM_007162]	-0.95356077	0.047708835
A_23_P43273	*NOL10*	nucleolar protein 10 (NOL10) [NM_024894]	-0.48304963	0.048182435

A_23_P94921	*EXT1*	exostosin glycosyltransferase 1 (EXT1) [NM_000127]	-1.4249834	0.027751032
A_23_P165574	*SLC20A2*	solute carrier family 20 (phosphate transporter), member 2 (SLC20A2)[NM_006749]	-1.1357478	0.040930405
A_23_P118462	*IWS1*	IWS1 homolog (S. cerevisiae) (IWS1)[NM_017969]	-0.8313581	0.025407597
A_23_P343837	*OVCA2*	ovarian tumor suppressor candidate 2 (OVCA2) [NM_080822]	-1.0356858	0.045638688
A_23_P144311	*PARP11*	poly (ADP-ribose) polymerase family, member 11 (PARP11) [NM_020367]	-1.0851147	0.03933512
A_24_P56270	*POLR2B*	polymerase (RNA) II (DNA directed) polypeptide B, 140kDa (POLR2B) [NM_000938]	-0.7069858	0.04622053
A_32_P85676	*DYRK2*	dual-specificity tyrosine-(Y)-phosphorylation regulated kinase 2 (DYRK2) [NM_006482]	-0.60871935	0.041931406
A_33_P3272231	*STK32B*	serine/threonine kinase 32B (STK32B) [NM_018401]	-1.0570439	0.03911937
A_33_P3212839	*MFSD2A*	major facilitator superfamily domain containing 2A (MFSD2A) [NM_001136493]	-0.8514955	0.03808641
A_33_P3784283	*ZNF701*	zinc finger protein 701 (ZNF701) [NM_001172655]	-2.531923	0.035935212
A_33_P3224878	*JAK1*	Janus kinase 1 (JAK1) [NM_002227]	0.9308188	0.037221838
A_23_P51699	*ITGA4*	integrin, alpha 4 (antigen CD49D, alpha 4 subunit of VLA-4 receptor) (ITGA4) [NM_000885]	-1.0732243	0.043335356
A_33_P3671291	*ARHGEF2*	Rho/Rac guanine nucleotide exchange factor (GEF) 2 (ARHGEF2) [NM_004723]	-0.49114946	0.04434183
A_23_P141394	*SNORA12*	EST91069 Synovial sarcoma Homo sapiens cDNA 5' end [AA378382]	-1.1080908	0.04468573
A_32_P190049	*WIPI1*	WD repeat domain, phosphoinositide interacting 1 (WIPI1) [NM_017983]	-1.0133809	0.047756795
A_24_P2648	*LRRC58*	leucine rich repeat containing 58 (LRRC58)NM_001099678]	-1.501694	0.04524507
A_33_P3346841	*PTPN14*	protein tyrosine phosphatase, non-receptor type 14 (PTPN14) [NM_005401]	-2.1894808	0.03946874
A_21_P0014915	*YTHDF2*	YTH N(6)-methyladenosine RNA binding protein 2 (YTHDF2) [NM_001173128]	-1.0249498	0.02798001
A_33_P3387566	*SMIM13*	small integral membrane protein 13 (SMIM13)[NM_001135575]	-0.6542793	0.031965

A_23_P52031	*FAM215A*	family with sequence similarity 215, member A (non-protein coding) (FAM215A)[NR_026770]	-1.0069777	0.028502433
A_22_P00003346	*PGM1*	phosphoglucomutase 1 (PGM1) [NM_002633]	1.6016592	0.047996294
A_32_P220715	*lnc-CBLB-4*	Q6JHZ7_HUMAN (Q6JHZ7) HCV-NS5ATP5 binding protein 1, partial (23%) [THC2676175]	-1.1768073	0.031493735
A_32_P58074	*MAP1LC3B*	microtubule-associated protein 1 light chain 3 beta (MAP1LC3B) [NM_022818]	-0.6683937	0.03800859
A_33_P3351894	*RPS3A*	ribosomal protein S3A (RPS3A) [NM_001006]	-1.5561416	0.041414
A_23_P204782	*MIF-AS1*	MIF antisense RNA 1 (MIF-AS1) [NR_038911]	1.6205924	0.027751032
A_33_P3271395	*MDM1*	Mdm1 nuclear protein homolog (mouse) (MDM1) [NM_020128]	-1.131073	0.038727812
A_33_P3336915	*LOC100129534*	small nuclear ribonucleoprotein polypeptide N pseudogene (LOC100129534) [NR_024489]	-1.1877074	0.03136619
A_23_P17345	*SUMF2*	sulfatase modifying factor 2 (SUMF2) [NM_015411]	-0.5499334	0.043712083
A_23_P319013	*MAFB*	v-maf avian musculoaponeurotic fibrosarcoma oncogene homolog B (MAFB) [NM_005461]	-1.8117894	0.044186465
A_23_P111961	*ZNF383*	zinc finger protein 383 (ZNF383) [NM_152604]	-0.8831567	0.029994998
A_23_P56314	*MAK16*	MAK16 homolog (S. cerevisiae) (MAK16) [NM_032509]	-0.83972836	0.029808238
A_22_P00004927	*UQCR11*	ubiquinol-cytochrome c reductase, complex III subunit XI (UQCR11)[NM_006830]	-2.7489824	0.038137045
A_22_P00002716	*lnc-DBN1-2*	LNCipedia lincRNA (lnc-DBN1-2)[lnc-DBN1-2:2]	-1.0048208	0.042994436
A_21_P0003208	*lnc-C20orf197-3*	LNCipedia lincRNA (lnc-C20orf197-3)[lnc-C20orf197-3:13]	-0.8427236	0.03804464
A_22_P00019317	*lnc-KIAA0226-3*	LNCipedia lincRNA (lnc-KIAA0226-3) [lnc-KIAA0226-3:1]	-0.72616625	0.027751032
A_21_P0008390	*lnc-NMNAT3-3*	LNCipedia lincRNA (lnc-NMNAT3-3) [lnc-NMNAT3-3:2]	1.604915	0.037804034
A_33_P3319982	*lnc-DLK1-6*	LNCipedia lincRNA (lnc-DLK1-6) [lnc-DLK1-6:9]	3.164266	0.040218875
A_33_P3331376	*lnc-TMC7-1*	clone IMAGE:4424208, mRNA. [BC015443]	-1.3777751	0.039287783
A_32_P208403	*EPHB2*	EPH receptor B2 (EPHB2) [NM_004442]	1.3712131	0.029573848

A_33_P3364459	*GNG2*	guanine nucleotide binding protein (G protein), gamma 2 (GNG2) [NM_053064]	1.0411206	0.03880133
A_33_P3405168	*GMPR2*	guanosine monophosphate reductase 2 (GMPR2) [NM_001283021]	1.2713765	0.02737376
A_33_P7465707	*MMAA*	methylmalonic aciduria (cobalamin deficiency) cblA type (MMAA) [NM_172250]	2.13267	0.024948223
A_23_P61674	*KRAS*	Kirsten rat sarcoma viral oncogene homolog (KRAS) [NM_004985]	-1.5519644	0.037923194
A_22_P00004662	*CLK4*	CDC-like kinase 4 (CLK4) [NM_020666]	-0.7440041	0.045316625
A_33_P3405004	*TMEM185A*	transmembrane protein 185A (TMEM185A)[NM_032508]	-1.4109595	0.026497496
A_33_P3250268	*NFXL1*	nuclear transcription factor, X-box binding-like 1 (NFXL1) [NM_001278624]	-1.5902101	0.029808238
A_23_P307400	*GOLGA6L7P*	golgin A6 family-like 7, pseudogene [ENST00000567390]	0.9717261	0.024948223
A_23_P119627	*CEP95*	centrosomal protein 95kDa (CEP95) [NM_138363]	-0.9432844	0.042693015
A_23_P108751	*NDUFA13*	NADH dehydrogenase (ubiquinone) 1 alpha subcomplex, 13 (NDUFA13) [NM_015965]	-0.6022399	0.028573772
A_33_P3379726	*FHL2*	four and a half LIM domains 2 (FHL2) [NM_001039492]	1.5825474	0.031367566
A_24_P291598	*CCDC106*	coiled-coil domain containing 106 (CCDC106) [NM_013301]	-1.2482895	0.042440254
A_33_P3225250	*USP4*	ubiquitin specific peptidase 4 (proto-oncogene) (USP4) [NM_003363]	-0.9271281	0.046045035
A_24_P244356	*ALKBH3*	alkB, alkylation repair homolog 3 (E. coli) (ALKBH3) [NM_139178]	-1.2183976	0.027751032
A_23_P37347	*NLRX1*	NLR family member X1 (NLRX1) [NM_001282144]	-1.1307251	0.028573772
A_24_P217365	*SNW1*	SNW domain containing 1 (SNW1) [NM_012245]	-0.8134248	0.027751032
A_33_P3251989	*ANKRD28*	ankyrin repeat domain 28 (ANKRD28) [NM_015199]	-1.0441896	0.047591254
A_21_P0010886	*CRIPT*	cysteine-rich PDZ-binding protein (CRIPT) [NM_014171]	-0.92879367	0.0335366
A_21_P0012455	*XLOC_l2_002033*	BROAD Institute lincRNA (XLOC_l2_002033) [TCONS_l2_00003633]	-0.5925255	0.043670435
A_33_P3341365	*LOC101927056*	PREDICTED: uncharacterized LOC101927056 (RP11-379B18.5) [XR_425623]	-0.6555279	0.030362261
A_33_P3217689	*RNF216*	ring finger protein 216 (RNF216) [NM_207111]	-1.3014677	0.029808238

A_33_P3391578	*JMY*	junction mediating and regulatory protein, p53 cofactor (JMY)[NM_152405]	-0.68191457	0.036344
A_23_P75299	*RPL23AP64*	ribosomal protein L23a pseudogene 64 (RPL23AP64) [NR_003040]	-1.1676403	0.049603622
A_23_P404134	*LHPP*	phospholysine phosphohistidine inorganic pyrophosphate phosphatase (LHPP) [NM_022126]	-1.0274493	0.042526342
A_33_P3330099	*TOX4*	TOX high mobility group box family member 4 [ENST00000448790]	-1.4695392	0.048271507
A_23_P321959	*ARSD*	arylsulfatase D (ARSD) [NM_001669]	0.85025465	0.043540128
A_33_P3413927	*SFT2D1*	SFT2 domain containing 1 (SFT2D1) [NM_145169]	-1.7523863	0.027751032
A_24_P184555	*VEZT*	vezatin, adherens junctions transmembrane protein (VEZT) [NM_017599]	-1.0057389	0.029808238
A_23_P31085	*PXN*	paxillin (PXN) [NM_002859]	-1.7533257	0.030692566
A_33_P3211604	*CCDC28A*	coiled-coil domain containing 28A (CCDC28A) [NM_015439]	-0.9225206	0.03957154
A_33_P3593774	*RAP1B*	RAP1B, member of RAS oncogene family (RAP1B)[NM_015646]	-0.76197886	0.027751032
A_24_P360078	*PIK3R3*	phosphoinositide-3-kinase, regulatory subunit 3 (gamma) (PIK3R3) [NM_001303429]	0.6043627	0.04793844
A_33_P3318187	*LRBA*	LPS-responsive vesicle trafficking, beach and anchor containing (LRBA)[NM_006726]	-0.66327477	0.04462891
A_33_P3276475	*CENPBD1*	CENPB DNA-binding domains containing 1 (CENPBD1) [NM_145039]	-1.0601583	0.028546385
A_23_P79842	*CHMP1B*	charged multivesicular body protein 1B (CHMP1B)[NM_020412]	0.4264356	0.037204653
A_33_P3327961	*PIGT*	phosphatidylinositol glycan anchor biosynthesis, class T (PIGT) [NM_015937]	-1.0330768	0.035278592
A_33_P8579064	*ZNF615*	zinc finger protein 615 (ZNF615) [NM_001199324]	-1.0850767	0.02737376
A_22_P00015730	*ZNF518A*	protein 518A [ENST00000484770]	-0.5458302	0.03515347
A_33_P3277611	*SZT2*	seizure threshold 2 homolog (mouse) (SZT2) [NM_015284]	-1.5280364	0.026808027
A_22_P00005682	*TMEM8C*	transmembrane protein 8C (TMEM8C)[NM_001080483]	-0.86691344	0.03683928
A_33_P3289820	*lnc-EIF6-1*	LNCipedia lincRNA (lnc-EIF6-1)[lnc-EIF6-1:9]	-0.9281022	0.041768707
A_33_P3216319	*IQSEC1*	IQ motif and Sec7 domain 1 (IQSEC1)[NM_001134382]	-1.2533544	0.044141345

A_23_P91468	*ZCCHC14*	zinc finger, CCHC domain containing 14 (ZCCHC14)[NM_015144]	1.4437644	0.04434183
A_33_P3214635	*PSMA7*	proteasome (prosome, macropain) subunit, alpha type, 7 (PSMA7)[NM_002792]	-1.0337678	0.047996294
A_23_P257201	*FECH*	ferrochelatase (FECH) [NM_001012515]	-0.5519695	0.029642703
A_33_P3365408	*RNF146*	ring finger protein 146 (RNF146)[NM_030963]	-0.84363425	0.034037594
A_23_P98057	*C1RL-AS1*	C1RL antisense RNA 1 (C1RL-AS1)[NR_026947]	-0.6251395	0.028573772
A_24_P363745	*ZNF32*	zinc finger protein 32 (ZNF32) [NM_001005368]	-0.72414315	0.04447692
A_21_P0009578	*LRRC14*	Homo sapiens leucine rich repeat containing 14 (LRRC14), transcript variant 2, mRNA [NM_014665]	-1750238	0.045638688
A_21_P0013333	*lnc-FBXO15-4*	LNCipedia lincRNA (lnc-FBXO15-4), lincRNA [lnc-FBXO15-4:1]	-0.18317588	0.038287252
A_24_P940620	*FAM185A*	family with sequence similarity 185, member A (FAM185A)[NM_001145268]	-1.5265822	0.02737376
A_23_P121250	*CCDC57*	coiled-coil domain containing 57 [ENST00000327026]	-2.3796139	0.040171996
A_23_P417282	*EIF4A2*	eukaryotic translation initiation factor 4A2 (EIF4A2) [NM_001967]	2.408771	0.028573772
A_24_P487736	*IGF1R*	insulin-like growth factor 1 receptor (IGF1R) [NM_000875]	-0.6099621	0.031606767
A_22_P00023841	*CXorf23*	chromosome X open reading frame 23 (CXorf23) [NM_198279]	1.2232511	0.04103619
A_22_P00012561	*lnc-HLCS-1*	LNCipedia lincRNA (lnc-HLCS-1), lincRNA [lnc-HLCS-1:1]	-1.377007	0.049901493
A_21_P0010857	*LOC100506476*	uncharacterized LOC100506476 (LOC100506476) [NR_109995]	-1.454351	0.029541323
A_23_P94860	*lnc-FAS-1*	PREDICTED: Fas cell surface death receptor (FAS) [XM_006717819]	1.0717818	0.03558176
A_23_P209564	*NUDT9*	nudix (nucleoside diphosphate linked moiety X)-type motif 9 (NUDT9) [NM_024047]	-2.4531674	0.037136003
A_22_P00015965	*CYBRD1*	cytochrome b reductase 1 (CYBRD1) [NM_024843]	-1.5333073	0.040555127
A_24_P36868	*lnc-TEFM-2*	DKFZp781H0240_r1 781 (synonym: hlcc4) cDNA clone DKFZp781H0240 5' [BX493510]	-1.5537586	0.03136619
A_33_P3379506	*WDR26*	WD repeat domain 26 (WDR26)[NM_025160]	-0.701942	0.046256438
A_33_P3341224	*MITD1*	MIT, microtubule interacting and transport, domain containing 1 (MITD1)[NM_138798]	-1.6935457	0.041067377

A_33_P3337931	*CDK17*	cyclin-dependent kinase 17 (CDK17) [NM_001170464]	2.0800428	0.03974276
A_24_P135322	*UBE2Q1*	ubiquitin-conjugating enzyme E2Q family member 1 (UBE2Q1) [NM_017582]	-0.8398042	0.045638688
A_23_P54116	*NRP1*	neuropilin 1 (NRP1) [NM_001024629]	1.1769377	0.038782965
A_24_P645765	*DAAM1*	dishevelled associated activator of morphogenesis 1 (DAAM1) [NM_014992]	-0.38467574	0.04680539
A_23_P344037	*KLHL42*	kelch-like family member 42 (KLHL42) [NM_020782]	-0.7903676	0.024948223
A_23_P301360	*CHFR*	checkpoint with forkhead and ring finger domains, E3 ubiquitin protein ligase (CHFR) [NM_018223]	-1.1436349	0.027019119
A_21_P0001911	*ZNF572*	zinc finger protein 572 (ZNF572) [NM_152412]	-2.3443904	0.032914247
A_32_P144596	*NIFK-AS1*	NIFK antisense RNA 1 (NIFK-AS1) [NR_037858]	-1.111621	0.04555549
A_23_P162945	*TNKS*	tankyrase, TRF1-interacting ankyrin-related ADP-ribose polymerase (TNKS) [NM_003747]	-0.79002565	0.045740407
A_23_P34930	*SRP54*	signal recognition particle 54kDa (SRP54) [NM_003136]	-1.3832421	0.04192822
A_23_P123454	*BCAS2*	breast carcinoma amplified sequence 2 (BCAS2)[NM_005872]	-0.9704541	0.042656552
A_23_P46894	*NUDT18*	nudix (nucleoside diphosphate linked moiety X)-type motif 18 (NUDT18) [NM_024815]	-0.6033392	0.03136619
A_23_P128744	*CHAT*	choline O-acetyltransferase (CHAT)[NM_020549]	-0.64445823	0.024948223
A_33_P3360097	*BDKRB1*	bradykinin receptor B1 (BDKRB1) [NM_000710]	-0.73673695	0.037741717
A_32_P108254	*APRT*	adenine phosphoribosyltransferase (APRT) [NM_000485]	-0.93006283	0.035935212
A_23_P63038	*FAM20A*	family with sequence similarity 20, member A (FAM20A) [NM_017565]	1.6339862	0.033726536
A_21_P0010084	*P3H1*	prolyl 3-hydroxylase 1 (P3H1) [NM_022356]	3.3607311	0.042440254
A_33_P3306526	*LOC100270804*	uncharacterized LOC100270804 (LOC100270804) [NR_026885]	-0.8042407	0.0442831
A_21_P0005923	*DCAF5*	DDB1 and CUL4 associated factor 5 (DCAF5) [NM_001284208]	-1.2969087	0.03808641
A_23_P200685	*LOC100506990*	uncharacterized LOC100506990 (LOC100506990) [NR_040092]	-1.1942623	0.024948223
A_33_P3415500	*MARC2*	mitochondrial amidoxime reducing component 2 (MARC2) [NM_017898]	-0.940335	0.048949

A_21_P0013646	*TRIO*	trio Rho guanine nucleotide exchange factor [ENST00000620511]	-0.8451537	0.033853088
A_23_P14105	*lnc-C5orf47-2*	LNCipedia lincRNA (lnc-C5orf47-2) [lnc-C5orf47-2:1]	-1.5168839	0.029808238
A_23_P389102	*RCBTB2*	regulator of chromosome condensation (RCC1) and BTB (POZ) domain containing protein 2 (RCBTB2) [NM_001268]	-0.9774995	0.034635574
A_21_P0005092	*MYO1D*	myosin ID (MYO1D) [NM_015194]	-1.0629215	0.024948223
A_23_P348383	*LOC101929484*	PREDICTED: uncharacterized LOC101929484 (LOC101929484) [XR_245642]	1.1285785	0.026497496
A_23_P170290	*CC2D2A*	coiled-coil and C2 domain containing 2A (CC2D2A)[NM_001080522]	-0.61061454	0.04434183
A_21_P0003658	*TMEM57*	transmembrane protein 57 (TMEM57) [NM_018202]	-3.0248194	0.031126594
A_33_P3292854	*lnc-USP38-1*	LNCipedia lincRNA (lnc-USP38-1) [lnc-USP38-1:1]	-2.531516	0.04856388
A_23_P90099	*CALR*	calreticulin (CALR) [NM_004343]	-0.9962672	0.03718595
A_21_P0009196	*TMEM205*	transmembrane protein 205 (TMEM205) [NM_198536]	-1.395197	0.04964876
A_23_P315286	*TMEM92-AS1*	TMEM92 antisense RNA 1 (TMEM92-AS1) [NR_125805]	1.210367	0.031091278
A_23_P131208	*R3HDM4*	R3H domain containing 4 (R3HDM4) [NM_138774]	-0.89302856	0.04976156
A_33_P3323999	*NR4A2*	nuclear receptor subfamily 4, group A, member 2 (NR4A2) [NM_006186]	-0.6545613	0.028573772
A_23_P87013	*SBF1*	SET binding factor 1 (SBF1) [NM_002972]	1.0917846	0.045437407
A_33_P7514500	*TAGLN*	transgelin (TAGLN) [NM_001001522]	-0.803258	0.039750393
A_23_P348063	*RRP7B*	ribosomal RNA processing 7 homolog B (S. cerevisiae) (RRP7B) [NR_002184]	2.6842437	0.029051661
A_32_P95823	*SYNGR1*	synaptogyrin 1 (SYNGR1) [NM_004711]	-1.3409374	0.031569958
DCP_22_0	*UBXN4*	UBX domain protein 4 (UBXN4) [NM_014607]	-1.3920935	0.024948223
A_24_P256692	*DAK*	dihydroxyacetone kinase 2 homolog (S. cerevisiae) (DAK) [NM_015533]	-1.0899653	0.03718595
A_33_P3393821	*MIA3*	melanoma inhibitory activity family, member 3 (MIA3)[NM_198551]	-1.3607495	0.03136619
A_33_P3343750	*C1R*	complement component 1, r subcomponent (C1R) [NM_001733]	1.209141	0.035278592
A_22_P00024677	*ZNF567*	zinc finger protein 567 (ZNF567), transcript variant 1, mRNA [NM_001300979]	-1.1945331	0.034210585

A_23_P168229	*LOC101927901*	uncharacterized LOC101927901 (LOC101927901)[NR_110063]	-0.90960616	0.034210585
A_33_P3386506	*TXNDC5*	thioredoxin domain containing 5 (endoplasmic reticulum) (TXNDC5) [NM_030810]	-1.880291	0.031377234
A_32_P23010	*NFKBIL1*	nuclear factor of kappa light polypeptide gene enhancer in B-cells inhibitor-like 1 (NFKBIL1) [NM_001144961]	-0.77184075	0.03136619
A_23_P385217	*SDHAF1*	succinate dehydrogenase complex assembly factor 1 (SDHAF1) [NM_001042631]	1.0401605	0.03297842
A_23_P17012	*ARL8B*	ADP-ribosylation factor-like 8B (ARL8B) [NM_018184]	-1.0471566	0.04820357
A_23_P203790	*SCRN3*	secernin 3 (SCRN3) [NM_024583]	-1.7333856	0.03403978
A_23_P204472	*OS9*	osteosarcoma amplified 9, endoplasmic reticulum lectin (OS9) [NM_006812]	-0.94496804	0.04733213
A_33_P3415526	*RPLP0*	ribosomal protein, large, P0 (RPLP0) [NM_053275]	-0.57590145	0.03403978
A_24_P134074	*FBXO11*	F-box protein 11 (FBXO11) [NM_001190274]	-1.1813177	0.034635574
A_23_P419107	*RPS19*	ribosomal protein S19 (RPS19) [NM_001022]	-0.9532206	0.028192867
A_22_P00011160	*TCP11L2*	t-complex 11, testis-specific-like 2 (TCP11L2) [NM_152772]	-1.4186459	0.047043864
A_33_P3315704	*lnc-OBSCN-1*	(lnc-OBSCN-1)[lnc-OBSCN-1:1]	0.49726054	0.04405224
A_33_P3308456	*INO80D*	INO80 complex subunit D (INO80D) [NM_017759]	-1.2871945	0.04959708
A_23_P106708	*PRAC2*	prostate cancer susceptibility candidate 2 (PRAC2) [NM_001282275]	1.0117611	0.039287783
A_21_P0010535	*RPS2*	ribosomal protein S2 (RPS2) [NM_002952]	1.5166508	0.02737376
A_33_P3364443	*XLOC_l2_000297*	BROAD Institute lincRNA (XLOC_l2_000297) [TCONS_l2_00000385]	0.91682696	0.03440522
A_23_P35591	*CRNDE*	colorectal neoplasia differentially expressed (non-protein coding) [ENST00000560208]	1.2915338	0.027942823
A_23_P414899	*EXOSC1*	exosome component 1 (EXOSC1) [NM_016046]	-0.5293965	0.044286337
A_23_P48358	*TTC17*	tetratricopeptide repeat domain 17 (TTC17) [NM_018259]	-0.9525027	0.042850092
A_23_P1206	*PCCA*	propionyl CoA carboxylase, alpha polypeptide (PCCA) [NM_000282]	-1.5889219	0.034999274
A_23_P151970	*RPS24*	ribosomal protein S24 (RPS24) [NM_001026]	-0.71365803	0.04747978

A_33_P3396159	*FEM1B*	fem-1 homolog b (C. elegans) (FEM1B) [NM_015322]	-0.8739709	0.043106858
A_23_P11237	*CCDC6*	coiled-coil domain containing 6 (CCDC6) [NM_005436]	-1.1975749	0.031377234
A_23_P120660	*TAF1*	TAF1 RNA polymerase II, TATA box binding protein (TBP)-associated factor, 250kDa (TAF1) [NM_004606]	-0.6707971	0.027751032
A_23_P144911	*RPS21*	ribosomal protein S21 (RPS21) [NM_001024]	-1.2497281	0.03138659
A_21_P0010663	*EGFLAM*	EGF-like, fibronectin type III and laminin G domains (EGFLAM) [NM_152403]	-0.61130816	0.04555549
A_24_P27412	*XLOC_l2_001206*	BROAD Institute lincRNA (XLOC_l2_001206) [TCONS_l2_00001638]	-1.45496	0.042639446
A_23_P24365	*SNUPN*	snurportin 1 (SNUPN) [NM_005701]	-0.5859129	0.028583162
A_23_P37514	*ANKRD49*	ankyrin repeat domain 49 (ANKRD49) [NM_017704]	5.013453	0.0376762
A_32_P194779	*C15orf39*	chromosome 15 open reading frame 39 (C15orf39) [NM_015492]	1.3339349	0.024948223
A_33_P3396607	*ZBTB34*	zinc finger and BTB domain containing 34 (ZBTB34) [NM_001099270]	-0.9089157	0.042529937
A_23_P83463	*UGDH*	UDP-glucose 6-dehydrogenase (UGDH) [NM_003359]	-0.7304916	0.03223675
A_33_P3350207	*PHPT1*	phosphohistidine phosphatase 1 (PHPT1) [NM_014172]	-1.255634	0.030829152
A_33_P3369760	*HCFC1R1*	host cell factor C1 regulator 1 (XPO1 dependent) (HCFC1R1) [NM_017885]	-0.7831487	0.036667652
A_23_P162336	*GLIPR2*	GLI pathogenesis-related 2 (GLIPR2)[NM_001287011]	0.85302716	0.044186465
A_33_P3250394	*CNOT2*	CCR4-NOT transcription complex, subunit 2 (CNOT2) [NM_014515]	-1.12766	0.03466831
A_23_P20107	*TRPC2*	transient receptor potential cation channel, subfamily C, member 2, pseudogene [ENST00000451043]	-0.84209466	0.0439664
A_24_P330971	*GSTK1*	glutathione S-transferase kappa 1 (GSTK1) [NM_015917]	-1.8729022	0.024948223
A_22_P00016681	*EIF3K*	eukaryotic translation initiation factor 3, subunit K (EIF3K)[NM_013234]	-0.6064159	0.024948223
A_33_P3397693	*lnc-TPPP2-1*	AY028430 N-myc downstream regulator 2 [THC2496327]	1.3026397	0.024948223
A_22_P00002012	*AGPS*	alkylglycerone phosphate synthase (AGPS) [NM_003659]	-0.9591901	0.031569958
A_23_P115922	*lnc-BDKRB1-1*	LNCipedia lincRNA (lnc-BDKRB1-1)[lnc-BDKRB1-1:1]	-0.822675	0.0429929

A_23_P303671	*EIF4EBP2*	eukaryotic translation initiation factor 4E binding protein 2 (EIF4EBP2) [NM_004096]	1.6888939	0.02453978
A_22_P00006290	*ECM2*	extracellular matrix protein 2, female organ and adipocyte specific (ECM2) [NM_001393]	-1.2929705	0.034210585
A_23_P149664	*RAD51-AS1*	RAD51 antisense RNA 1 (head to head) (RAD51-AS1) [NR_040058]	2.3183455	0.03774674
A_23_P152651	*TMEM183B*	transmembrane protein 183B (TMEM183B) [NM_001079809]	-0.65911585	0.04820357
A_33_P3521643	*DDX42*	DEAD (Asp-Glu-Ala-Asp) box helicase 42 (DDX42)[NM_007372]	-2.4420686	0.036667652
A_23_P152548	*PPP1R12C*	protein phosphatase 1, regulatory subunit 12C (PPP1R12C)[NM_001271618]	1.6785325	0.04236566
A_21_P0000731	*SCPEP1*	serine carboxypeptidase 1 (SCPEP1)[NM_021626]	-1.115597	0.029808238
A_24_P167877	*LINC00968*	long intergenic non-protein coding RNA 968 (LINC00968) [NR_038236]	-0.9469814	0.024948223
A_33_P3221563	*NPIPB5*	nuclear pore complex interacting protein family, member B5 (NPIPB5) [NM_001135865]	-0.6180914	0.036339592
A_23_P404108	*ARMC5*	armadillo repeat containing 5 (ARMC5) [NM_024742]	-1.2284975	0.041254643
A_21_P0012294	*CCSER2*	coiled-coil serine-rich protein 2 (CCSER2) [NM_018999]	-1.7550992	0.029019035
A_33_P3299170	*PDE4D*	phosphodiesterase 4D, cAMP-specific (PDE4D)[NM_001165899]	-0.7533469	0.028546385
A_23_P162982	*LUC7L*	LUC7-like (S. cerevisiae) (LUC7L) [NM_201412]	-0.8411939	0.02453978
A_23_P54170	*DHRS4*	dehydrogenase/reductase (SDR family) member 4 (DHRS4) [NM_021004]	1.0949636	0.032456078
A_23_P66608	*MED6*	mediator complex subunit 6 (MED6) [NM_005466]	1.5687019	0.049758088
A_23_P75430	*KAT2A*	K(lysine) acetyltransferase 2A (KAT2A) [NM_021078]	-0.49631375	0.049901493
A_23_P214222	*SMCO4*	single-pass membrane protein with coiled-coil domains 4 (SMCO4)[NM_020179]	-0.43929574	0.048681058
A_22_P00017039	*MARCKS*	myristoylated alanine-rich protein kinase C substrate [ENST00000612661]	-0.6344967	0.048238702
A_24_P30141	*PP12719*	pp12719 mRNA, complete cds. [AF318328]	-1.60519	0.03136619
A_33_P3395758	*FRA10AC1*	fragile site, folic acid type, rare, fra(10)(q23.3) or fra(10)(q24.2) candidate 1 (FRA10AC1) [NM_145246]	-1.3273821	0.04545722

A_24_P192197	*C14orf28*	chromosome 14 open reading frame 28 (C14orf28) [NM_001017923]	1.4203134	0.03840762
A_32_P43812	*WRNIP1*	Werner helicase interacting protein 1 (WRNIP1) [NM_130395]	-2.2823873	0.024948223
A_23_P113572	*DCUN1D4*	DCN1, defective in cullin neddylation 1, domain containing 4 (DCUN1D4)[NM_001040402]	-0.5596637	0.043971255
A_32_P31771	*CD19*	CD19 molecule (CD19) [NM_001770]	-1.7045869	0.03716006
A_22_P00010189	*KIAA1715*	KIAA1715 (KIAA1715)[NM_030650]	-1.0512168	0.03536427
A_33_P3282556	*LOC101926937*	PREDICTED: uncharacterized LOC101926937 (LOC101926937)[XR_244896]	-1.1663678	0.028573772
A_33_P3253799	*TMEM204*	transmembrane protein 204 (TMEM204) [NM_024600]	1.6691892	0.04929023
A_24_P277955	*RUFY1*	RUN and FYVE domain containing 1 (RUFY1)[NM_001040451]	-0.7928795	0.046056066
A_23_P104741	*FIS1*	fission 1 (mitochondrial outer membrane) homolog (S. cerevisiae) (FIS1), mRNA [NM_016068]	1.6364509	0.03403978
A_23_P111672	*KIRREL3*	kin of IRRE like 3 (Drosophila) (KIRREL3) [NM_032531]	-1.2458355	0.02598378
A_33_P3319920	*TES*	testis derived transcript (3 LIM domains) (TES) [NM_152829]	-1.4325801	0.034466885
A_21_P0014799	*KDM4B*	lysine (K)-specific demethylase 4B (KDM4B) [NM_015015]	-0.8964186	0.04367992
A_33_P3381943	*GOLGA2*	golgin A2 (GOLGA2) [NM_004486]	-2.1198516	0.034698997
A_23_P209625	*DMTF1*	cyclin D binding myb-like transcription factor 1 (DMTF1) [NR_024549]	-1.255107	0.0419793
A_22_P00023573	*CYP1B1*	cytochrome P450, family 1, subfamily B, polypeptide 1 (CYP1B1) [NM_000104]	0.8295312	0.03808641
A_22_P00016858	*lnc-MAB21L2-1*	cDNA FLJ39676 fis, clone SMINT2009832. [AK096995]	-1.2659698	0.031899918
A_24_P322741	*lnc-TRPT1-1*	LNCipedia lincRNA (lnc-TRPT1-1) [lnc-TRPT1-1:1]	-1.3021382	0.049327243
A_33_P3864411	*IL10RB*	interleukin 10 receptor, beta (IL10RB) [NM_000628]	-1.9718812	0.030915326
A_33_P3248586	*ACTR3BP5*	ACTR3B pseudogene 5 (ACTR3BP5) [NR_045000]	1.5860931	0.028573772
A_21_P0008478	*DUX4*	double homeobox 4 (DUX4) [NM_001293798]	-1.2068208	0.04348676
A_23_P73763	*lnc-SERPINA12-1*	LNCipedia lincRNA (lnc-SERPINA12-1) [lnc-SERPINA12-1:1]	-0.9722051	0.02453978
A_19_P00808072	*LAGE3*	L antigen family, member 3 (LAGE3) [NM_006014]	2.0424175	0.044449862

A_32_P153388	*lnc-RTL1-2*	LNCipedia lincRNA (lnc-RTL1-2), lincRNA [lnc-RTL1-2:1]	0.9204091	0.04217344
A_23_P410312	*GULP1*	GULP, engulfment adaptor PTB domain containing 1 (GULP1) [NM_016315]	2.4886827	0.041609965
A_33_P3333826	*C12orf76*	chromosome 12 open reading frame 76 (C12orf76)[NM_207435]	-0.9434593	0.040241584
A_33_P3321342	*UBE2Q2*	ubiquitin-conjugating enzyme E2Q family member 2 (UBE2Q2) [NM_173469]	1.4026155	0.03152038
A_23_P204751	*INSIG2*	insulin induced gene 2 (INSIG2) [NM_016133]	1.4653782	0.04705478
A_33_P3369190	*ASIC1*	acid sensing (proton gated) ion channel 1 (ASIC1) [NM_020039]	-1.4423264	0.028583162
A_23_P82206	*PAM*	peptidylglycine alpha-amidating monooxygenase (PAM) [NM_001177306]	-0.6171431	0.03974276
A_21_P0006253	*ECHDC1*	ethylmalonyl-CoA decarboxylase 1 (ECHDC1) [NM_018479]	-1.6994855	0.03616732
A_23_P83234	*lnc-PTPRD-3*	LNCipedia lincRNA (lnc-PTPRD-3)[lnc-PTPRD-3:1]	-1.7299509	0.031640545
A_23_P145501	*ZBTB6*	zinc finger and BTB domain containing 6 (ZBTB6) [NM_006626]	-1.883978	0.03233256
A_23_P157449	*MED23*	mediator complex subunit 23 (MED23)[NM_004830]	-0.60796577	0.049758088
A_23_P218096	*POLR2K*	polymerase (RNA) II (DNA directed) polypeptide K, 7.0kDa (POLR2K) [NM_005034]	0.79347837	0.034342222
A_23_P200199	*RBM26*	RNA binding motif protein 26 (RBM26) [NM_022118]	-1.2526457	0.038424533
A_23_P424316	*TXNDC12*	thioredoxin domain containing 12 (endoplasmic reticulum) (TXNDC12) [NM_015913]	-1.2025397	0.03152038
A_33_P3370461	*TCF20*	transcription factor 20 (AR1) (TCF20) [NM_005650]	-0.613049	0.038003992
A_33_P3290949	*PHF20*	PHD finger protein 20 (PHF20) [NM_016436]	-1.0090599	0.026808027
A_24_P419028	*PTCD3*	pentatricopeptide repeat domain 3 [ENST00000487043]	-1.4175766	0.031603426
A_22_P00016771	*MOP-1*	mRNA for MOP-1, [AB014771]	-1.2077446	0.027751032
A_23_P321855	*TRIM31-AS1*	TRIM31 antisense RNA 1 (TRIM31-AS1) [NR_126470]	-1.6224581	0.034751743
A_22_P00012600	*ARHGEF7*	Rho guanine nucleotide exchange factor (GEF) 7 (ARHGEF7)[NM_003899]	-1.1209514	0.024948223
A_33_P3347452	*lnc-PTPRCAP-1*	LNCipedia lincRNA (lnc-PTPRCAP-1) [lnc-PTPRCAP-1:1]	1.0821539	0.02919588

A_24_P128312	*RPS6KA2*	ribosomal protein S6 kinase, 90kDa, polypeptide 2 (RPS6KA2) [NM_021135]	2.074296	0.027942823
A_24_P66125	*ZNF79*	zinc finger protein 79 (ZNF79) [NM_007135]	-0.6461425	0.03451892
A_33_P3370848	*STAG2*	stromal antigen 2 (STAG2) [NM_001042749]	0.5763366	0.042527765
A_24_P91472	*ETV2*	ets variant 2 (ETV2) [NM_014209]	-1.3336687	0.048681058
A_33_P3378509	*ALKBH7*	alkB, alkylation repair homolog 7 (E. coli) (ALKBH7) [NM_032306]	-1.245525	0.042310614
A_23_P119196	*USP38*	ubiquitin specific peptidase 38 (USP38) [NM_001290326]	-0.9765696	0.034999274
A_23_P161522	*KLF2*	Kruppel-like factor 2 (KLF2)[NM_016270]	-0.5856175	0.02453978
A_33_P3285251	*TMEM134*	transmembrane protein 134 (TMEM134) [NM_025124]	-1.129106	0.030915326
A_24_P277807	*FAM225B*	family with sequence similarity 225, member B (non-protein coding) (FAM225B) [NR_024376]	-1.5311668	0.031127423
A_33_P3401301	*SNX3*	sorting nexin 3 (SNX3)[NM_003795]	-0.8782398	0.03668808
A_33_P3264528	*RPL39*	ribosomal protein L39 (RPL39)[NM_001000]	1.2419918	0.04855276
A_23_P156826	*HOXA11*	homeobox A11 (HOXA11) [NM_005523]	1.8683883	0.031377234
A_33_P3219434	*ADTRP*	androgen-dependent TFPI-regulating protein (ADTRP) [NM_032744]	-1.7481753	0.03912857
A_33_P3215557	*MARC2*	mitochondrial amidoxime reducing component 2 (MARC2) [NM_017898]	-0.8469601	0.03227331
A_22_P00023080	*ADAMTS7*	ADAM metallopeptidase with thrombospondin type 1 motif, 7 (ADAMTS7) [NM_014272]	-1.8326088	0.049730826
A_33_P3349651	*LRIG2*	leucine-rich repeats and immunoglobulin-like domains 2 [ENST00000361127]	0.87413526	0.04976156
A_33_P3233700	*SWAP70*	SWAP switching B-cell complex 70kDa subunit (SWAP70) [NM_015055]	-1.6217967	0.03297842
A_33_P3262495	*HPN-AS1*	HPN antisense RNA 1 (HPN-AS1) [NR_024561]	1.2007196	0.031569958
A_23_P414895	*ZNF503*	zinc finger protein 503 (ZNF503)[NM_032772]	-1.3799357	0.028573772
A_23_P66117	*TTC17*	tetratricopeptide repeat domain 17 (TTC17) [NM_018259]	-1.3788364	0.031603426
A_22_P00016108	*ITFG3*	integrin alpha FG-GAP repeat containing 3 (ITFG3) [NM_032039]	0.93766505	0.0415662
A_23_P211814	*LOC102724814*	uncharacterized LOC102724814 (LOC102724814) [NR_110555]	-1.5950174	0.041687127

A_21_P0004024	*MAP4*	microtubule-associated protein 4 (MAP4) [NM_002375]	-0.40536287	0.048238702
A_33_P3349145	*lnc-RASA1-3*	LNCipedia lincRNA (lnc-RASA1-3) [lnc-RASA1-3:1]	-0.9528835	0.033099733
A_23_P11664	*TTLL1*	tubulin tyrosine ligase-like family member 1 (TTLL1) [NM_012263]	-0.55497986	0.026208585
A_33_P3257755	*SRSF11*	serine/arginine-rich splicing factor 11 (SRSF11) [NM_004768]	-1.2675965	0.027751032
A_24_P178148	*C2orf49*	chromosome 2 open reading frame 49 (C2orf49) [NM_024093]	-1.7122147	0.02737376
A_19_P00812349	*LOC100129455*	PREDICTED: uncharacterized LOC100129455 (LOC100129455) [XR_249246]	-1.0350175	0.04217344
A_32_P138004	*ZNF805*	zinc finger protein 805 (ZNF805)[NM_001023563]	0.6389215	0.048931535
A_23_P215931	*FAM45A*	family with sequence similarity 45, member A (FAM45A) [NM_207009]	-1.1487367	0.025734548
A_23_P99917	*LEPROTL1*	leptin receptor overlapping transcript-like 1 (LEPROTL1) [NM_015344]	1.9025528	0.0376762
A_24_P99071	*WDR73*	WD repeat domain 73 (WDR73) [NM_032856]	-1.4726774	0.043079574
A_23_P34142	*IPO5*	importin 5 (IPO5)[NM_002271]	-0.8551669	0.027751032
A_32_P143048	*WBP5*	WW domain binding protein 5 (WBP5) [NM_016303]	-0.97821736	0.036187515
A_21_P0008252	*ZFYVE9*	zinc finger, FYVE domain containing 9 (ZFYVE9)[NM_004799]	-0.8245361	0.036959786
A_33_P3354858	*lnc-TSC22D1-1*	LNCipedia lincRNA (lnc-TSC22D1-1) [lnc-TSC22D1-1:4]	-1.2275698	0.033692434
A_24_P383901	*COQ10B*	coenzyme Q10 homolog B (S. cerevisiae) (COQ10B) [NM_025147]	-0.6992004	0.030896025
A_33_P3216337	*lnc-FLYWCH2-1*	CM4-HT1152-161200-627-h03 HT1152 H [BF843649]	1.8173944	0.032286324
A_33_P3376071	*FTSJ1*	FtsJ RNA methyltransferase homolog 1 (E. coli) (FTSJ1) [NM_177439]	-0.6434194	0.035278592
A_23_P44264	*IFIT5*	interferon-induced protein with tetratricopeptide repeats 5 (IFIT5)[NM_012420]	1.5571287	0.040555127
A_23_P211985	*EMX2*	empty spiracles homeobox 2 (EMX2) [NM_004098]	-0.8184004	0.04135566
A_33_P3248424	*SNRK*	SNF related kinase (SNRK) [NM_017719]	-0.5920055	0.047879543
A_23_P364478	*OTULIN*	OTU deubiquitinase with linear linkage specificity (OTULIN) [NM_138348]	-0.8309839	0.03973283
A_23_P52207	*FAM175B*	family with sequence similarity 175, member B (FAM175B) [NM_032182]	-1.0868812	0.040555127

A_33_P3313595	*BAMBI*	BMP and activin membrane-bound inhibitor (BAMBI) [NM_012342]	-1.6787795	0.036041338
A_32_P208350	*CDK11B*	cyclin-dependent kinase 11B (CDK11B)[NM_033487]	0.5704035	0.04964876
A_23_P46369	*TDRD9*	tudor domain containing 9 (TDRD9)[NM_153046]	-1.4905264	0.04434183
A_24_P124662	*RAB13*	RAB13, member RAS oncogene family (RAB13) [NM_002870]	-4.0234785	0.028573772
A_23_P211244	*MAPKAPK5*	mitogen-activated protein kinase-activated protein kinase 5 (MAPKAPK5) [NM_139078]	-1.0201957	0.033095628
A_23_P50504	*PRMT2*	protein arginine methyltransferase 2 (PRMT2) [NM_206962]	2.3240619	0.040091004
A_22_P00010494	*FTL*	ferritin, light polypeptide (FTL) [NM_000146]	-1.6002494	0.038283803
A_24_P273666	*XLOC_l2_005415*	BROAD Institute lincRNA (XLOC_l2_005415) [TCONS_l2_00010039]	-1.0589023	0.03808641
A_22_P00013852	*GNAS*	GNAS complex locus (GNAS)[NM_001077489]	-0.56225234	0.04831641
A_22_P00013508	*LOC400620*	uncharacterized LOC400620 (LOC400620) [NR_040020]	1.1234332	0.047727138
A_24_P74571	*LOC102725057*	PREDICTED: uncharacterized LOC102725057 (LOC102725057) [XR_424658]	2.1539025	0.027751032
A_24_P928052	*CBY1*	chibby homolog 1 (Drosophila) (CBY1) [NM_001002880]	-1.0645857	0.039287783
A_33_P3295009	*NRP1*	neuropilin 1 (NRP1) [NM_003873]	1.8325863	0.026497496
A_32_P8546	*lnc-AEBP1-1*	AGENCOURT_6793813 NIH_MGC_99 [BQ059925]	-0.8997302	0.04009655
DCP_20_1	*LINC00473*	long intergenic non-protein coding RNA 473 (LINC00473) [NR_026860]	-1.2861896	0.035935212
A_21_P0005101	*FAM160B1*	family with sequence similarity 160, member B1 (FAM160B1) [NM_020940]	0.5676803	0.032155342
A_32_P218355	*lnc-WRNIP1-2*	LNCipedia lincRNA (lnc-WRNIP1-2) [lnc-WRNIP1-2:20]	3.291873	0.045638688
A_22_P00013715	*C6orf132*	chromosome 6 open reading frame 132 (C6orf132) [NM_001164446]	1.3070679	0.04881034
A_33_P3423600	*EWSAT1*	long intergenic non-protein coding RNA 277 [ENST00000559914]	-0.8899767	0.029808238
A_21_P0006174	*OXLD1*	oxidoreductase-like domain containing 1 (OXLD1) [NM_001039842]	-1.2700039	0.03683928
A_33_P3298024	*lnc-C9orf79-1*	LNCipedia lincRNA (lnc-C9orf79-1) [lnc-C9orf79-1:2]	-1.5030308	0.029573848
A_24_P181295	*ABCC3*	ATP-binding cassette, sub-family C (CFTR/MRP), member 3 (ABCC3) [NM_001144070]	0.587106	0.04792962

A_24_P248185	*C14orf37*	chromosome 14 open reading frame 37 (C14orf37)[NM_001001872]	-0.8908961	0.040091004
A_22_P00025333	*PIANP*	PILR alpha associated neural protein (PIANP) [NM_153685]	1.370866	0.03136619
A_33_P3415895	*MARCH8*	membrane-associated ring finger (C3HC4) 8, E3 ubiquitin protein ligase (MARCH8) [NM_145021]	-0.9590378	0.028189372
A_23_P213592	*NRBF2*	nuclear receptor binding factor 2 (NRBF2)[NM_030759]	-1.2813313	0.047591254
A_24_P106357	*RNF44*	ring finger protein 44 (RNF44) [NM_014901]	1.3792526	0.04217344
A_23_P212696	*WDR36*	WD repeat domain 36 (WDR36) [NM_139281]	-0.5905945	0.033091284
A_23_P91350	*FSTL1*	follistatin-like 1 (FSTL1) [NM_007085]	-1.275721	0.031606767
A_23_P59787	*GPCPD1*	glycerophosphocholine phosphodiesterase GDE1 homolog (S. cerevisiae) (GPCPD1) [NM_019593]	-1.0878153	0.038424533
A_24_P11315	*LUC7L2*	LUC7-like 2 (S. cerevisiae) (LUC7L2) [NM_016019]	-0.8380487	0.04217344
A_24_P20777	*OLFML3*	olfactomedin-like 3 (OLFML3) [NM_020190]	-1.283994	0.036891155
A_23_P4353	*RPL37*	ribosomal protein L37 (RPL37) [NM_000997]	-0.5579756	0.031377234
A_23_P63816	*WSB1*	WD repeat and SOCS box containing 1 (WSB1) [NM_015626]	-0.69984704	0.030826582
DCP_22_4	*NRBF2*	nuclear receptor binding factor 2 (NRBF2) [NM_030759]	-1.5695126	0.034750238
A_24_P21752	*RPAP3*	RNA polymerase II associated protein 3 (RPAP3) [NM_024604]	-0.7585046	0.048172984
A_33_P3328837	*TBRG1*	transforming growth factor beta regulator 1 (TBRG1) [NM_032811]	-1.6275979	0.028573772
A_33_P3311046	*TRIP12*	thyroid hormone receptor interactor 12, mRNA (cDNA clone IMAGE:5742743), with apparent retained intron. [BC037956]	-0.7720661	0.042693015
A_23_P115467	*OR2M7*	olfactory receptor, family 2, subfamily M, member 7 (OR2M7) [NM_001004691]	-1.A14320320741	0.031603426
A_23_P107036	*S100A5*	S100 calcium binding protein A5 (S100A5) [NM_002962]	-1.1205695	0.013003563
A_33_P3232280	*TMEM11*	transmembrane protein 11 (TMEM11) [NM_003876]	-0.55698925	0.031606767
A_23_P94159	*ASUN*	asunder spermatogenesis regulator (ASUN) [NM_018164]	2.96139	0.027189178
A_23_P255153	*FBXO25*	F-box protein 25 (FBXO25) [NM_183421]	2.0963624	0.028573772

A_21_P0012646	*RBMX2*	RNA binding motif protein, X-linked 2 (RBMX2) [NM_016024]	-0.41749832	0.028192867
A_23_P327698	*PRDX6*	peroxiredoxin 6 (PRDX6) [NM_004905]	-0.8324154	0.033091284
A_23_P129085	*LMBRD2*	LMBR1 domain containing 2 (LMBRD2) [NM_001007527]	-1.3282963	0.039628718
A_23_P356122	*SPESP1*	sperm equatorial segment protein 1 (SPESP1) [NM_145658]	-1.4779395	0.03297842
A_33_P3260974	*ZNF451*	zinc finger protein 451 (ZNF451) [NM_001031623]	-0.80553555	0.04747978
A_23_P66137	*NUCB1*	nucleobindin 1 (NUCB1) [NM_006184]	1.9959706	0.034210585
A_24_P44596	*SOX8*	SRY (sex determining region Y)-box 8 (SOX8) [NM_014587]	-1.004557	0.044286337
A_33_P3290343	*MON1B*	MON1 secretory trafficking family member B (MON1B) [NM_014940]	2.4557133	0.030915326
A_24_P350644	*CYP1B1*	cytochrome P450, family 1, subfamily B, polypeptide 1 (CYP1B1) [NM_000104]	-1.6125952	0.036131933
A_33_P3405068	*PRRC2B*	proline-rich coiled-coil 2B (PRRC2B) [NM_013318]	1.1549199	0.026497496
A_23_P90659	*NAV1*	neuron navigator 1 (NAV1) [NM_020443]	-0.75703335	0.042621672
A_23_P381714	*LAPTM4A*	lysosomal protein transmembrane 4 alpha (LAPTM4A) [NM_014713]	-3.144054	0.039899252
A_23_P43049	*CA13*	carbonic anhydrase XIII (CA13) [NM_198584]	-0.713137	0.031972177
A_33_P3219596	*DCTN6*	dynactin 6 (DCTN6) [NM_006571]	-1.5334754	0.024948223
A_33_P3214310	*LINC00925*	long intergenic non-protein coding RNA 925 (LINC00925) [NR_015411]	-0.76915646	0.045638688
A_22_P00002061	*FOXP1*	forkhead box P1 (FOXP1)[NM_001012505]	-1.2943276	0.038322687
A_21_P0014443	*lnc-BLID-1*	LNCipedia lincRNA (lnc-BLID-1)[lnc-BLID-1:8]	-1.0227382	0.046325136
A_32_P11894	*ZSCAN32*	Homo sapiens zinc finger and SCAN domain containing 32 (ZSCAN32), transcript variant 2, mRNA [NM_017810]	-1.9941115	0.030441707
A_23_P77048	*C12orf65*	Homo sapiens chromosome 12 open reading frame 65 (C12orf65), transcript variant 1, mRNA [NM_152269]	-2.4429982	0.03136619
A_21_P0010032	*SLC25A29*	Homo sapiens solute carrier family 25 (mitochondrial carnitine/acylcarnitine carrier), member 29 (SLC25A29), transcript variant 1, mRNA [NM_001039355]	1.0290575	0.035278592

A_24_P328969	*Q5T8U4*	Q5T8U4_HUMAN (Q5T8U4) Ribosomal protein L7a, partial (94%) [THC2577892]	-1.3050487	0.024948223
A_19_P00324470	*YIPF5*	Homo sapiens Yip1 domain family, member 5 (YIPF5), transcript variant 2, mRNA [NM_030799]	-1.2693503	0.03138659
A_23_P145247	*MIR143HG*	Homo sapiens MIR143 host gene (non-protein coding) (MIR143HG), long non-coding RNA [NR_105059]	-0.845529	0.040930405
A_33_P3214298	*ZSCAN26*	Homo sapiens zinc finger and SCAN domain containing 26 (ZSCAN26), [NM_001023560]	-1.2448692	0.02598378
A_24_P33461	*IMPDH2*	Homo sapiens IMP (inosine 5'-monophosphate) dehydrogenase 2 (IMPDH2), mRNA [NM_000884]	-1.3918779	0.027751032
A_21_P0010595	*AEBP2*	Homo sapiens AE binding protein 2 (AEBP2), transcript variant 1, mRNA [NM_153207]	-0.8740276	0.042850092
A_23_P141208	*FBXO25*	Homo sapiens F-box protein 25 (FBXO25), transcript variant 2, mRNA [NM_183420]	-0.88817185	0.049901493
A_23_P87072	*DHRS7B*	Homo sapiens dehydrogenase/reductase (SDR family) member 7B (DHRS7B), mRNA [NM_015510]	1.5493672	0.02737376
A_23_P127533	*PANX3*	Homo sapiens pannexin 3 (PANX3), mRNA [NM_052959]	-0.65717524	0.045638688
A_33_P3215953	*DCUN1D5*	Homo sapiens DCN1, defective in cullin neddylation 1, domain containing 5 (DCUN1D5), mRNA [NM_032299]	-1.341779	0.035769645
A_23_P63896	*MPZL1*	Homo sapiens myelin protein zero-like 1 (MPZL1), transcript variant 2, mRNA [NM_024569]	-0.7340386	0.036377493
A_33_P3300308	*FAS*	Homo sapiens Fas cell surface death receptor (FAS), transcript variant 1, mRNA [NM_000043]	1.3164997	0.03623899
A_21_P0005787	*MAP1LC3A*	Homo sapiens microtubule-associated protein 1 light chain 3 alpha (MAP1LC3A) [NM_032514]	-0.4513302	0.04705478
A_21_P0012457	*LOC101927815*	Homo sapiens uncharacterized LOC101927815 (LOC101927815), long non-coding RNA [NR_125425]	-0.65585977	0.03770505
A_23_P44956	*XLOC_l2_010330*	BROAD Institute lincRNA (XLOC_l2_010330), lincRNA [TCONS_l2_00019490]	-1.730591	0.027751032
A_33_P3265359	*RPL35A*	Homo sapiens ribosomal protein L35a (RPL35A), [NM_000996]	-1.3103799	0.03138659

A_32_P4199	*HES6*	Homo sapiens hes family bHLH transcription factor 6 (HES6), [NM_018645]	1.2416524	0.047933746
A_24_P201404	*RNF152*	Homo sapiens ring finger protein 152 (RNF152), [NM_173557]	-0.7466948	0.048182435
A_24_P244470	*C11orf54*	Homo sapiens chromosome 11 open reading frame 54 (C11orf54), [NM_014039]	-0.4545512	0.030293303
A_23_P117146	*RNF41*	Homo sapiens ring finger protein 41, E3 ubiquitin protein ligase (RNF41), [NM_194358]	0.91604507	0.039287783
A_23_P435501	*PRDM4*	Homo sapiens PR domain containing 4 (PRDM4), [NM_012406]	2.085948	0.0442831
A_24_P481783	*SERINC3*	Homo sapiens serine incorporator 3 (SERINC3), [NM_198941]	-0.90518224	0.0479607
A_22_P00015296	*UBAP1L*	Homo sapiens ubiquitin associated protein 1-like (UBAP1L), [NM_001163692]	-1.3949229	0.04219537
A_24_P261383	*EIF3J-AS1*	Homo sapiens EIF3J antisense RNA 1 (head to head) (EIF3J-AS1), [NR_034170]	-1.1120521	0.033091284
A_33_P3335966	*TAF1D*	Homo sapiens TATA box binding protein (TBP)-associated factor, RNA polymerase I, D, 41kDa (TAF1D), [NM_024116]	-0.8803816	0.04041675
A_23_P89410	*TPM1*	Homo sapiens tropomyosin 1 (alpha) (TPM1), [NM_001018005]	-0.5335864	0.037765756
A_23_P69437	*BECN1*	Homo sapiens beclin 1, autophagy related (BECN1), [NM_003766]	-1.2653449	0.025525203
A_23_P40108	*YEATS2*	Homo sapiens YEATS domain containing 2 (YEATS2), [NM_018023]	-1.5289203	0.034008626
A_33_P6461662	*COL9A3*	Homo sapiens collagen, type IX, alpha 3 (COL9A3), [NM_001853]	-2.5061314	0.024948223
A_23_P320530	*VPS11*	Homo sapiens vacuolar protein sorting 11 homolog (S. cerevisiae) (VPS11), [NM_001290185]	-0.8111117	0.025407597
A_23_P114883	*ZNF780A*	Homo sapiens zinc finger protein 780A (ZNF780A), [NM_001010880]	-1.1030486	0.04643008
A_23_P47247	*FMOD*	Homo sapiens fibromodulin (FMOD), [NM_002023]	3.9391203	0.04823297
A_23_P65278	*TCP11L1*	Homo sapiens t-complex 11, testis-specific-like 1 (TCP11L1), [NM_018393]	-1.0957775	0.027751032
A_23_P218637	*NBEA*	Homo sapiens neurobeachin (NBEA), [NM_015678]	-1.2053009	0.027942823
A_23_P145388	*RGPD5*	Homo sapiens RANBP2-like and GRIP domain containing 5 (RGPD5), [NM_005054]	-3.9368384	0.04237416

A_33_P3235990	*MTCH1*	Homo sapiens mitochondrial carrier 1 (MTCH1), [NM_014341]	-1.125717	0.03770023
A_33_P3289207	*PTPN21*	Homo sapiens protein tyrosine phosphatase, non-receptor type 21 (PTPN21), [NM_007039]	-2.1883483	0.043439556
A_22_P00003387	*AAK1*	Homo sapiens cDNA clone IMAGE:5226225. [BC090950]	-1.0835121	0.030891629
A_24_P62860	*lnc-CCDC107-2*	Q8AGK8_9DELA (Q8AGK8) Gag polyprotein, partial (9%) [THC2505157]	-0.61524916	0.042850092
A_23_P28688	*STAM2*	Homo sapiens signal transducing adaptor molecule (SH3 domain and ITAM motif) 2 (STAM2), [NM_005843]	-1.1220748	0.04458629
A_23_P148519	*CPSF3*	Homo sapiens cleavage and polyadenylation specific factor 3, 73kDa (CPSF3), [NM_016207]	0.41152495	0.03974276
A_23_P121082	*FAM156A*	Homo sapiens family with sequence similarity 156, member A (FAM156A), [NM_014138]	1.5070639	0.045638688
A_23_P372660	*GBE1*	Homo sapiens glucan (1,4-alpha-), branching enzyme 1 (GBE1), [NM_000158]	-1.334714	0.047941346
A_19_P00800555	*GAPVD1*	Homo sapiens GTPase activating protein and VPS9 domains 1 (GAPVD1), [NM_015635]	-0.7462327	0.03273351
A_33_P3353259	*lnc-UQCRFS1-7*	Q982T4_RHILO (Q982T4) Mll8506 protein, partial (12%) [THC2612479]	-0.48202822	0.02453978
A_33_P3424803	*FAM220A*	Homo sapiens family with sequence similarity 220, member A (FAM220A), [NM_001037163]	-0.54990834	0.041414
A_33_P3369311	*HLA-C*	Human MHC class I HLA-Cw1 gene, complete cds. [M26429]	-1.6577435	0.02737376
A_23_P70384	*BBX*	Homo sapiens bobby sox homolog (Drosophila) (BBX), [NM_001142568]	-1.1718953	0.042656552
A_23_P93704	*RNF8*	Homo sapiens ring finger protein 8, E3 ubiquitin protein ligase (RNF8), [NM_003958]	-2.0564485	0.040555127
A_23_P128408	*C7orf61*	Homo sapiens chromosome 7 open reading frame 61 (C7orf61), [NM_001004323]	-1.6446579	0.028573772
A_32_P20523	*TRIAP1*	Homo sapiens TP53 regulated inhibitor of apoptosis 1 (TRIAP1), [NM_016399]	-0.8566852	0.03436134
A_24_P721898	*TIGD2*	Homo sapiens tigger transposable element derived 2 (TIGD2), [NM_145715]	-0.96621865	0.028573772
A_23_P200507	*BTBD7*	Homo sapiens BTB (POZ) domain containing 7 (BTBD7), transcript variant 1, mRNA [NM_001002860]	1.5026584	0.028573772

A_24_P368943	*CNIH4*	Homo sapiens cornichon family AMPA receptor auxiliary protein 4 (CNIH4), transcript variant 1, mRNA [NM_014184]	-0.7896061	0.046523497
A_24_P379765	*EVX1*	Homo sapiens even-skipped homeobox 1 (EVX1), mRNA [NM_001989]	-1.1176351	0.026666366
A_33_P3307894	*MOB1A*	Homo sapiens MOB kinase activator 1A (MOB1A), mRNA [NM_018221]	-0.40981793	0.04438234
A_33_P3270599	*DPEP3*	Homo sapiens dipeptidase 3 (DPEP3), transcript variant 1, mRNA [NM_022357]	1.9579582	0.033643603
A_23_P213678	*TPM2*	Homo sapiens tropomyosin 2 (beta) (TPM2), transcript variant Tpm2.3, mRNA [NM_001301226]	-0.46414113	0.030385435
A_33_P3888365	*PAM*	Homo sapiens peptidylglycine alpha-amidating monooxygenase (PAM), transcript variant 1, mRNA [NM_000919]	-0.8499241	0.03718595
A_22_P00021143	*RSBN1*	Homo sapiens round spermatid basic protein 1 (RSBN1), mRNA [NM_018364]	1.5533892	0.030915326
A_24_P386323	*lnc-CCDC166-1*	zu66e09.r1 Soares_testis_NHT Homo sapiens cDNA clone IMAGE:742984 5', mRNA sequence [AA405962]	-1.2284219	0.033091284
A_32_P170925	*RABEPK*	Homo sapiens Rab9 effector protein with kelch motifs (RABEPK), transcript variant 1, mRNA [NM_005833]	-1.4551599	0.035438698
A_22_P00010904	*TXNRD3*	Homo sapiens thioredoxin reductase 3 (TXNRD3), transcript variant 1, mRNA [NM_052883]	-0.98812526	0.036667652
A_33_P3242264	*MFAP5*	Homo sapiens microfibrillar associated protein 5 (MFAP5), transcript variant 1, mRNA [NM_003480]	-0.7823725	0.049901493
A_33_P3416937	*NSUN4*	Homo sapiens NOP2/Sun domain family, member 4 (NSUN4), transcript variant 1, mRNA [NM_199044]	-1.2234532	0.02453978
A_23_P94591	*FAM206A*	family with sequence similarity 206, member A [Source:HGNC Symbol;Acc:HGNC:1364] [ENST00000374624]	0.88415325	0.033526238
A_24_P942002	*TMEM141*	Homo sapiens transmembrane protein 141 (TMEM141), [NM_032928]	-3.0268285	0.034210585
A_24_P766208	*ACAP2*	Homo sapiens ArfGAP with coiled-coil, ankyrin repeat and PH domains 2 (ACAP2), [NM_012287]	-0.30995798	0.024948223
A_23_P22614	*RPL3*	Homo sapiens ribosomal protein L3 (RPL3), [NM_000967]	-1.3202057	0.040287334

A_33_P3315763	*SEPT6*	Homo sapiens septin 6 (SEPT6), transcript variant V, mRNA [NM_145802]	-0.7887712	0.0420182
A_33_P3728979	*LOC143286*	Homo sapiens mRNA; cDNA DKFZp586E171 (from clone DKFZp586E171) [AL049428]	-0.78481674	0.027751032
A_23_P107724	*FAM151B*	Homo sapiens family with sequence similarity 151, member B (FAM151B), [NM_205548]	-3.2010684	0.03770023
A_24_P295590	*ZNF112*	Homo sapiens zinc finger protein 112 (ZNF112), [NM_013380]	-0.803236	0.043885134
A_23_P46170	*RASSF4*	Homo sapiens Ras association (RalGDS/AF-6) domain family member 4 (RASSF4), mRNA [NM_032023]	1.0654105	0.049862977
A_22_P00002900	*MED8*	Homo sapiens mediator complex subunit 8 (MED8), transcript variant 5, mRNA [NM_201542]	-1.3769125	0.02971716
A_23_P23171	*CTBP1-AS2*	Homo sapiens CTBP1 antisense RNA 2 (head to head) (CTBP1-AS2), long non-coding RNA [NR_033339]	-0.86866516	0.03136619
A_24_P256337	*AGO4*	Homo sapiens argonaute RISC catalytic component 4 (AGO4), mRNA [NM_017629]	-0.76193625	0.035186797
A_23_P26687	*ZNF644*	Homo sapiens zinc finger protein 644 (ZNF644), transcript variant 1, mRNA [NM_201269]	-0.7238929	0.034750238
A_23_P214969	*TMEM186*	Homo sapiens transmembrane protein 186 (TMEM186), mRNA [NM_015421]	1.2902383	0.024948223
A_23_P64990	*CITED2*	Cbp/p300-interacting transactivator, with Glu/Asp-rich carboxy-terminal domain, 2 (CITED2), [NM_006079]	-0.8108913	0.031162176
A_33_P3369766	*RAD52*	Homo sapiens RAD52 homolog (S. cerevisiae) (RAD52), [NM_134424]	-0.88354665	0.02453978
A_33_P3363674	*MED4*	Homo sapiens mediator complex subunit 4 (MED4), [NM_014166]	-0.6567251	0.027751032
A_33_P3300867	*NFYC*	Homo sapiens nuclear transcription factor Y, gamma (NFYC), [NM_001142590]	-2.0436072	0.024948223
A_23_P143029	*lnc-PAICS-1*	Homo sapiens cDNA FLJ42646 fis, clone BRACE3026075. [AK124637]	-0.88121486	0.04434183
A_22_P00018036	*HOXD11*	Homo sapiens homeobox D11 (HOXD11), [NM_021192]	-1.7402682	0.033526238
A_21_P0000063	*lnc-ZNF236-1*	LNCipedia lincRNA (lnc-ZNF236-1), lincRNA [lnc-ZNF236-1:1]	-0.6405563	0.04881034
A_23_P146885	*MDFIC*	Homo sapiens MyoD family inhibitor domain containing (MDFIC), [NM_001166346]	0.87670904	0.036667652

A_23_P97309	*UTS2R*	Homo sapiens urotensin 2 receptor (UTS2R), mRNA [NM_018949]	-3.1617265	0.047933746
A_23_P81492	*CASP9*	Homo sapiens caspase 9, apoptosis-related cysteine peptidase (CASP9), [NM_001229]	3.4586306	0.03136619
A_23_P250283	*RPS14*	Homo sapiens ribosomal protein S14 (RPS14), [NM_001025071]	1.5862203	0.027942823
A_33_P3375910	*RAB1A*	Homo sapiens RAB1A, member RAS oncogene family (RAB1A), transcript variant 1, mRNA [NM_004161]	-2.1567304	0.049049266
A_23_P209694	*RRBP1*	ribosome binding protein 1 [Source:HGNC Symbol;Acc:HGNC:10448] [ENST00000610403]	-0.91638416	0.03319862
A_33_P3283044	*PAPOLG*	Homo sapiens poly(A) polymerase gamma (PAPOLG), mRNA [NM_022894]	-0.92729545	0.045638688
A_24_P268160	*MTCL1*	Homo sapiens microtubule crosslinking factor 1 (MTCL1), mRNA [NM_015210]	1.4272232	0.042269725
A_33_P3219651	*DRAM2*	Homo sapiens DNA-damage regulated autophagy modulator 2 (DRAM2), mRNA [NM_178454]	-0.4860167	0.030441707
A_23_P168771	*BMPER*	Homo sapiens BMP binding endothelial regulator (BMPER), mRNA [NM_133468]	-0.88175786	0.03576752
A_24_P337334	*CCDC146*	Homo sapiens coiled-coil domain containing 146 (CCDC146), mRNA [NM_020879]	-1.5964485	0.04501488
A_23_P112241	*DCTD*	Homo sapiens dCMP deaminase (DCTD), transcript variant 1, mRNA [NM_001012732]	-1.9363142	0.03403978
A_33_P3323822	*DNAJB5*	Homo sapiens DnaJ (Hsp40) homolog, subfamily B, member 5 (DNAJB5), transcript variant 3, mRNA [NM_012266]	-1.1321335	0.038322687
A_23_P257795	*GATAD2B*	Homo sapiens GATA zinc finger domain containing 2B (GATAD2B), mRNA [NM_020699]	-1.6047379	0.032993637
A_33_P3272330	*NDUFA2*	Homo sapiens NADH dehydrogenase (ubiquinone) 1 alpha subcomplex, 2, 8kDa (NDUFA2), transcript variant 1, mRNA [NM_002488]	-1.155525	0.02598378
A_23_P24926	*DNMT3A*	Homo sapiens DNA (cytosine-5-)-methyltransferase 3 alpha (DNMT3A), transcript variant 1, mRNA [NM_175629]	-1.1658146	0.039538864

A_33_P3366146	*FNTA*	Homo sapiens farnesyltransferase, CAAX box, alpha (FNTA), transcript variant 1, mRNA [NM_002027]	-2.0232632	0.04764094
A_33_P3466016	*SRSF5*	Homo sapiens serine/arginine-rich splicing factor 5 (SRSF5), transcript variant 1, mRNA [NM_001039465]	-0.784518	0.032779668
A_23_P216476	*LOC200830*	Homo sapiens cDNA FLJ30391 fis, clone BRACE2008336. [AK054953]	-1.0673071	0.0460751
A_21_P0009522	*ZBTB5*	Homo sapiens zinc finger and BTB domain containing 5 (ZBTB5), mRNA [NM_014872]	-0.7753391	0.03297842
A_23_P417331	*TRAF3IP2-AS1*	Homo sapiens TRAF3IP2 antisense RNA 1 (TRAF3IP2-AS1), transcript variant 4, long non-coding RNA [NR_034111]	1.1380959	0.030385435
A_23_P56759	*RPS6KA3*	Homo sapiens ribosomal protein S6 kinase, 90kDa, polypeptide 3 (RPS6KA3), mRNA [NM_004586]	-1.3255768	0.030438775
A_33_P3218925	*KRCC1*	Homo sapiens lysine-rich coiled-coil 1 (KRCC1), mRNA [NM_016618]	0.84834087	0.044401187
A_23_P61202	*STEAP2*	Homo sapiens STEAP family member 2, metalloreductase (STEAP2), transcript variant 4, mRNA [NM_001244944]	0.9067151	0.042667523
A_23_P162846	*FAM207A*	Homo sapiens family with sequence similarity 207, member A (FAM207A), mRNA [NM_058190]	-1.4628657	0.04590026
A_33_P3240263	*LAMP1*	Homo sapiens lysosomal-associated membrane protein 1 (LAMP1), mRNA [NM_005561]	-1.3356273	0.046789277
A_33_P3343206	*POMP*	Homo sapiens proteasome maturation protein (POMP), mRNA [NM_015932]	-1.3089409	0.027751032
A_23_P253921	*MBD1*	Homo sapiens methyl-CpG binding domain protein 1 (MBD1), transcript variant 11, mRNA [NM_001204142]	-0.97714615	0.036131933
A_23_P207399	*ZKSCAN7*	Homo sapiens zinc finger with KRAB and SCAN domains 7 (ZKSCAN7), transcript variant 1, mRNA [NM_018651]	-1.0692098	0.049704313
A_33_P3235716	*NBR1*	Homo sapiens neighbor of BRCA1 gene 1 (NBR1), transcript variant 1, mRNA [NM_005899]	0.63131016	0.03403978
A_22_P00015781	*SPSB3*	Homo sapiens splA/ryanodine receptor domain and SOCS box containing 3 (SPSB3), mRNA [NM_080861]	-0.61435384	0.03073435
A_23_P28538	*lnc-TAOK3-4*	LNCipedia lincRNA (lnc-TAOK3-4), lincRNA [lnc-TAOK3-4:2]	-0.79412794	0.033853088
A_32_P99100	*MRPS5*	Homo sapiens mitochondrial ribosomal protein S5 (MRPS5), mRNA [NM_031902]	-1.3301072	0.036660764

A_33_P3274560	*PTPRK*	Homo sapiens protein tyrosine phosphatase, receptor type, K (PTPRK), transcript variant 2, mRNA [NM_002844]	-1.0490351	0.028573772
A_24_P219053	*SLC19A3*	solute carrier family 19 (thiamine transporter), member 3 [Source:HGNC Symbol;Acc:HGNC:16266] [ENST00000409456]	1.5773016	0.029808238
A_23_P26810	*GPALPP1*	GPALPP motifs containing 1 [Source:HGNC Symbol;Acc:HGNC:20298] [ENST00000497558]	-0.8385792	0.033198114
A_23_P29803	*TP53*	tumor protein p53 (TP53) [NM_000546]	0.56671786	0.045006614
A_23_P319895	*POLR2H*	polymerase (RNA) II (DNA directed) polypeptide H (POLR2H) [NM_006232]	2.0167782	0.028189372
A_24_P60972	*SETD1B*	SET domain containing 1B (SETD1B) [NM_015048]	-1.8222613	0.047831167
A_21_P0014624	*TSTD2*	Homo sapiens thiosulfate sulfurtransferase (rhodanese)-like domain containing 2 (TSTD2), mRNA [NM_139246]	-1.133402	0.049526483
A_33_P3245218	*LOC102725057*	PREDICTED: Homo sapiens uncharacterized LOC102725057 (LOC102725057), ncRNA [XR_424658]	-0.7650492	0.033433396
A_24_P724040	*TENM2*	Homo sapiens teneurin transmembrane protein 2 (TENM2), mRNA [NM_001122679]	-0.819318	0.032155342
A_33_P3391290	*SNRPB2*	Homo sapiens small nuclear ribonucleoprotein polypeptide B (SNRPB2), transcript variant 1, mRNA [NM_003092]	-0.6496351	0.03403978
A_23_P93311	*NREP*	Homo sapiens neuronal regeneration related protein (NREP), transcript variant 11, mRNA [NM_001142483]	-0.8690355	0.047218688
A_23_P46844	*DDR1*	Homo sapiens discoidin domain receptor tyrosine kinase 1 (DDR1), transcript variant 2, mRNA [NM_013993]	-3.9656315	0.02737376
A_33_P3289466	*TRIM8*	Homo sapiens tripartite motif containing 8 (TRIM8), mRNA [NM_030912]	-0.8592338	0.04390963
A_33_P3296940	*CDC16*	Homo sapiens cell division cycle 16 (CDC16), transcript variant 2, mRNA [NM_001078645]	-1.4005313	0.03889491
A_23_P11800	*FNDC3B*	Homo sapiens fibronectin type III domain containing 3B (FNDC3B),	-0.49441957	0.041929405

		transcript variant 1, mRNA [NM_022763]		
A_23_P129358	*CAMK2N1*	Homo sapiens calcium/calmodulin-dependent protein kinase II inhibitor 1 (CAMK2N1), mRNA [NM_018584]	0.7930381	0.048690848
A_24_P380330	*SETD6*	Homo sapiens SET domain containing 6 (SETD6), transcript variant 2, mRNA [NM_024860]	-0.949661	0.034210585
A_23_P57941	*PANK3*	Homo sapiens pantothenate kinase 3 (PANK3), mRNA [NM_024594]	-0.9285157	0.036667652
A_23_P36513	*RBM6*	Homo sapiens RNA binding motif protein 6 (RBM6), transcript variant 1, mRNA [NM_005777]	2.1054811	0.038727812
A_33_P3354267	*PRKAG1*	Homo sapiens protein kinase, AMP-activated, gamma 1 non-catalytic subunit (PRKAG1), transcript variant 4, mRNA [NM_001206710]	-0.56175125	0.031972177
A_19_P00810474	*AKIRIN1*	Homo sapiens akirin 1 (AKIRIN1), transcript variant 1, mRNA [NM_024595]	-0.94601756	0.031603426
A_24_P240065	*CIC*	capicua transcriptional repressor [Source:HGNC Symbol;Acc:HGNC:14214] [ENST00000572681]	-1.1810076	0.027942823
A_22_P00021338	*CHMP3*	Homo sapiens charged multivesicular body protein 3 (CHMP3), transcript variant 1, mRNA [NM_016079]	-0.6459131	0.046497274
A_24_P207150	*GPATCH2L*	Homo sapiens G patch domain containing 2-like (GPATCH2L), transcript variant 1, mRNA [NM_017926]	-0.7297573	0.027751032
A_33_P3368675	*UBE3A*	Homo sapiens ubiquitin protein ligase E3A (UBE3A), transcript variant 3, mRNA [NM_130839]	0.86561394	0.044286337
A_24_P200023	*C6orf120*	Homo sapiens chromosome 6 open reading frame 120 (C6orf120), mRNA [NM_001029863]	-1.1862803	0.034210585
A_21_P0013795	*IL1R1*	Homo sapiens interleukin 1 receptor, type I (IL1R1), transcript variant 1, mRNA [NM_000877]	-0.96802175	0.03403978
A_33_P3338360	*RPL36A*	Homo sapiens ribosomal protein L36a (RPL36A), transcript variant 1, mRNA [NM_021029]	-0.8255026	0.038966544
A_24_P80181	*SCARNA13*	Homo sapiens small Cajal body-specific RNA 13 (SCARNA13), guide RNA [NR_003002]	-0.8993357	0.045740407

A_23_P310483	*TMEM127*	Homo sapiens transmembrane protein 127 (TMEM127), transcript variant 1, mRNA [NM_017849]	-1.0319071	0.031606767
A_23_P150080	*C8orf58*	Homo sapiens chromosome 8 open reading frame 58 (C8orf58), transcript variant 1, mRNA [NM_001013842]	-0.7254948	0.034210585
A_33_P3422030	*RPP38*	Homo sapiens ribonuclease P/MRP 38kDa subunit (RPP38), transcript variant 1, mRNA [NM_183005]	-1.437034	0.047831167
A_33_P3504659	*FXYD5*	FXYD domain containing ion transport regulator 5 [Source:HGNC Symbol;Acc:HGNC:4029] [ENST00000392218]	-0.86487603	0.033095628
A_23_P48166	*CASP10*	Homo sapiens caspase 10, apoptosis-related cysteine peptidase (CASP10), transcript variant 1, mRNA [NM_032977]	-0.9507656	0.039100364
A_24_P330773	*TWF1*	Homo sapiens twinfilin actin-binding protein 1 (TWF1), transcript variant 2, mRNA [NM_002822]	-0.7488349	0.042529937
A_33_P3241646	*CALCOCO2*	Homo sapiens calcium binding and coiled-coil domain 2 (CALCOCO2), transcript variant 3, mRNA [NM_005831]	1.2147633	0.047932126
A_22_P00004708	*PANK3*	Homo sapiens pantothenate kinase 3 (PANK3), mRNA [NM_024594]	-1.2763003	0.024948223
A_24_P32887	*lnc-CTD-3080F16.3.1-1*	LNCipedia lincRNA (lnc-CTD-3080F16.3.1-1), lincRNA [lnc-CTD-3080F16.3.1-1:1]	-1.111902	0.04369149
A_23_P210330	*BRD3*	Homo sapiens bromodomain containing 3 (BRD3), mRNA [NM_007371]	-1.423567	0.034342222
A_21_P0000095	*LGALSL*	Homo sapiens lectin, galactoside-binding-like (LGALSL), mRNA [NM_014181]	-1.0731977	0.047832783
A_23_P374351	*HDHD1*	Homo sapiens haloacid dehalogenase-like hydrolase domain containing 1 (HDHD1), transcript variant 3, mRNA [NM_001178135]	1.8338989	0.034210585
A_21_P0002639	*KCTD20*	Homo sapiens potassium channel tetramerization domain containing 20 (KCTD20), transcript variant 1, mRNA [NM_173562]	-1.2597556	0.0369263
A_33_P3316639	*lnc-TMEM18-6*	LNCipedia lincRNA (lnc-TMEM18-6), lincRNA [lnc-TMEM18-6:1]	-1.5177553	0.04965512
A_23_P69226	*CHMP3*	Homo sapiens charged multivesicular body protein 3 (CHMP3), transcript variant 3, mRNA [NM_001193517]	-1.4340467	0.042304065

A_21_P0006078	*EMC3*	Homo sapiens ER membrane protein complex subunit 3 (EMC3), mRNA [NM_018447]	-2.2084782	0.031746764
A_33_P3423845	*LINC01410*	Homo sapiens long intergenic non-protein coding RNA 1410 (LINC01410), long non-coding RNA [NR_121647]	1.7183592	0.026339147
A_33_P3629678	*ATP11A*	Homo sapiens ATPase, class VI, type 11A (ATP11A), transcript variant 1, mRNA [NM_015205]	-1.0174854	0.03136619
A_21_P0007892	*COL5A1*	Homo sapiens collagen, type V, alpha 1 (COL5A1), transcript variant 1, mRNA [NM_000093]	1.6443123	0.047832783
A_22_P00016483	*lnc-TMEM116-1*	Homo sapiens mRNA; cDNA DKFZp686P0929 (from clone DKFZp686P0929). [AL833501]	1.8030218	0.024948223
A_23_P145485	*LOC148413*	Homo sapiens uncharacterized LOC148413 (LOC148413), long non-coding RNA [NR_015434]	-0.8131119	0.031640545
A_24_P272061	*ULBP2*	Homo sapiens UL16 binding protein 2 (ULBP2), mRNA [NM_025217]	-1.9220407	0.04622315
A_22_P00017112	*RPL13A*	Homo sapiens ribosomal protein L13a (RPL13A), transcript variant 1, mRNA [NM_012423]	1.1088184	0.038287252
A_23_P216549	*ADAM12*	Homo sapiens ADAM metallopeptidase domain 12 (ADAM12), transcript variant 1, mRNA [NM_003474]	-1.2014699	0.029573848
A_23_P155257	*RUSC2*	Homo sapiens RUN and SH3 domain containing 2 (RUSC2), transcript variant 2, mRNA [NM_014806]	1.1634759	0.026467344
A_24_P189739	*FOXP1*	Homo sapiens forkhead box P1 (FOXP1), transcript variant 1, mRNA [NM_032682]	-1.887226	0.039287783
A_24_P157342	*DUSP16*	Homo sapiens dual specificity phosphatase 16 (DUSP16), mRNA [NM_030640]	-3.0124338	0.043402363
A_23_P108785	*BRK1*	Homo sapiens BRICK1, SCAR/WAVE actin-nucleating complex subunit (BRK1), mRNA [NM_018462]	-1.0394257	0.047941346
A_33_P3308050	*ACTR3*	Homo sapiens ARP3 actin-related protein 3 homolog (yeast) (ACTR3), transcript variant 1, mRNA [NM_005721]	-1.6134312	0.029573848
A_32_P140706	*EIF4E2*	Homo sapiens eukaryotic translation initiation factor 4E family member 2 (EIF4E2), transcript variant 1, mRNA [NM_004846]	-1.0350546	0.04024844
A_33_P3314386	*PMS2*	Homo sapiens PMS2 postmeiotic segregation increased 2 (S. cerevisiae)	-0.775428	0.042850092

		(PMS2), transcript variant 1, mRNA [NM_000535]		
A_33_P3405384	*TOR1AIP1*	Homo sapiens torsin A interacting protein 1 (TOR1AIP1), transcript variant 1, mRNA [NM_001267578]	-1.3364677	0.034750238
A_23_P30307	*MCMDC2*	Homo sapiens minichromosome maintenance domain containing 2 (MCMDC2), transcript variant 1, mRNA [NM_173518]	-1.2306852	0.028573772
A_33_P3231162	*MED7*	Homo sapiens mediator complex subunit 7 (MED7), transcript variant 2, mRNA [NM_004270]	-0.61066	0.039750393
A_23_P31654	*ZNF776*	Homo sapiens zinc finger protein 776 (ZNF776), mRNA [NM_173632]	-0.5663016	0.04309562
DCP_20_0	*RPL8*	Homo sapiens ribosomal protein L8 (RPL8), transcript variant 1, mRNA [NM_000973]	-1.302343	0.024948223
A_24_P813147	*AHCYL2*	Homo sapiens adenosylhomocysteinase-like 2 (AHCYL2), transcript variant 1, mRNA [NM_015328]	-1.3716896	0.028546385
A_23_P129101	*TUBB8*	Homo sapiens tubulin, beta 8 class VIII (TUBB8), mRNA [NM_177987]	-0.69708866	0.033692434
A_23_P54758	*HEXA*	Homo sapiens hexosaminidase A (alpha polypeptide) (HEXA), mRNA [NM_000520]	1.2546344	0.039287783
A_21_P0012609	*GDE1*	Homo sapiens glycerophosphodiester phosphodiesterase 1 (GDE1), mRNA [NM_016641]	-0.93715477	0.024948223
A_24_P261724	*CCR6*	Homo sapiens chemokine (C-C motif) receptor 6 (CCR6), transcript variant 2, mRNA [NM_031409]	-1.1202794	0.04488468
A_23_P150064	*RNF10*	Homo sapiens ring finger protein 10 (RNF10), mRNA [NM_014868]	-1.2957919	0.04542028
A_33_P3242733	*MMRN2*	Homo sapiens multimerin 2 (MMRN2), mRNA [NM_024756]	-0.43862754	0.04621581
A_23_P37265	*PKDCC*	Homo sapiens protein kinase domain containing, cytoplasmic (PKDCC), mRNA [NM_138370]	0.9951468	0.046325136
A_23_P96990	*AP5M1*	Homo sapiens adaptor-related protein complex 5, mu 1 subunit (AP5M1), transcript variant 1, mRNA [NM_018229]	-0.9829201	0.026440267
A_23_P51548	*NVL*	Homo sapiens nuclear VCP-like (NVL), transcript variant 1, mRNA [NM_002533]	2.585289	0.03808641

A_23_P142724	*MGST3*	Homo sapiens microsomal glutathione S-transferase 3 (MGST3), mRNA [NM_004528]	-2.3873343	0.030293303
A_33_P3381827	*RPL37A*	Homo sapiens ribosomal protein L37a (RPL37A), mRNA [NM_000998]	-1.0992327	0.04371038
A_33_P3292126	*OSBPL2*	Homo sapiens oxysterol binding protein-like 2 (OSBPL2), transcript variant 3, mRNA [NM_001278649]	-0.7197227	0.047218688
A_21_P0000833	*XLOC_l2_013837*	BROAD Institute lincRNA (XLOC_l2_013837), lincRNA [TCONS_l2_00026575]	-0.85007995	0.04971531
A_33_P3389540	*SENCR*	Homo sapiens smooth muscle and endothelial cell enriched migration/differentiation-associated long non-coding RNA (SENCR), long non-coding RNA [NR_038908]	-0.8232321	0.027751032
A_23_P29855	*TMCO1*	Homo sapiens transmembrane and coiled-coil domains 1 (TMCO1), transcript variant 1, mRNA [NM_019026]	1.546926	0.034635574
A_32_P117422	*USO1*	Homo sapiens USO1 vesicle transport factor (USO1), transcript variant 2, mRNA [NM_003715]	-2.2453258	0.048690848
A_23_P300033	*PMS2P4*	Homo sapiens postmeiotic segregation increased 2 pseudogene 4 (PMS2P4), transcript variant 2, non-coding RNA [NR_022007]	-1.4197812	0.046256438
A_33_P3289391	*PDGFRA*	Homo sapiens platelet-derived growth factor receptor, alpha polypeptide (PDGFRA), mRNA [NM_006206]	-1.0936294	0.04620576
A_22_P00011225	*SPIN1*	Homo sapiens spindlin 1 (SPIN1), mRNA [NM_006717]	-0.50133926	0.047098655
A_23_P201079	*lnc-OPRL1-1*	LNCipedia lincRNA (lnc-OPRL1-1), lincRNA [lnc-OPRL1-1:1]	-1.1677382	0.024948223
A_23_P331943	*PRDM2*	Homo sapiens PR domain containing 2, with ZNF domain (PRDM2), transcript variant 1, mRNA [NM_012231]	-1.5263339	0.031972177
A_23_P122863	*TMEM181*	Homo sapiens transmembrane protein 181 (TMEM181), mRNA [NM_020823]	-0.8161816	0.045638688
A_24_P61864	*GRB10*	Homo sapiens growth factor receptor-bound protein 10 (GRB10), transcript variant 4, mRNA [NM_001001555]	1.2745184	0.035207514
A_24_P142743	*CCDC47*	Homo sapiens coiled-coil domain containing 47 (CCDC47), mRNA [NM_020198]	-1.72812	0.026038945
A_33_P3255782	*CNN2*	calponin 2 [Source:HGNC Symbol;Acc:HGNC:2156] [ENST00000564572]	-0.7155609	0.039287783

A_23_P160167	*HOPX*	Homo sapiens HOP homeobox (HOPX), transcript variant 5, mRNA [NM_001145460]	-1.8895314	0.045316625
A_23_P45389	*TSPAN1*	Homo sapiens tetraspanin 1 (TSPAN1), mRNA [NM_005727]	-0.8974604	0.035078283
A_23_P50418	*RAB9A*	Homo sapiens RAB9A, member RAS oncogene family (RAB9A), transcript variant 1, mRNA [NM_004251]	1.8873371	0.04009291
A_23_P19164	*ZNF791*	Homo sapiens zinc finger protein 791 (ZNF791), mRNA [NM_153358]	1.9962463	0.049603622
A_23_P132526	*TTC1*	Homo sapiens tetratricopeptide repeat domain 1 (TTC1), transcript variant 1, mRNA [NM_003314]	1.278882	0.040555127
A_23_P167595	*PIK3R4*	Homo sapiens phosphoinositide-3-kinase, regulatory subunit 4 (PIK3R4), mRNA [NM_014602]	-1.7241602	0.028573772
A_23_P46149	*UBE2B*	Homo sapiens ubiquitin-conjugating enzyme E2B (UBE2B), mRNA [NM_003337]	-0.6538819	0.042255856
A_33_P7477724	*GPR137B*	Homo sapiens G protein-coupled receptor 137B (GPR137B), mRNA [NM_003272]	-0.90192765	0.034698997
A_33_P3393941	*lnc-VAMP1-1*	LNCipedia lincRNA (lnc-VAMP1-1), lincRNA [lnc-VAMP1-1:6]	-1.1237171	0.03136619
A_33_P3367917	*MBTPS1*	Homo sapiens membrane-bound transcription factor peptidase, site 1 (MBTPS1), mRNA [NM_003791]	-0.87278783	0.033853576
A_33_P3249529	*SSH2*	Homo sapiens slingshot protein phosphatase 2 (SSH2), transcript variant 3, mRNA [NM_001282130]	-0.71480876	0.031091278
A_33_P3381762	*PCNX*	Homo sapiens pecanex homolog (Drosophila) (PCNX), mRNA [NM_014982]	-1.1687458081	0.030441707
A_23_P340251	*XLOC_l2_010493*	BROAD Institute lincRNA (XLOC_l2_010493), lincRNA [TCONS_l2_00019671]	1.9088635	0.034750238
A_24_P188941	*RAB2A*	Homo sapiens RAB2A, member RAS oncogene family (RAB2A), transcript variant 1, mRNA [NM_002865]	-0.500168	0.047831167
A_33_P3519683	*NPM1*	Homo sapiens nucleophosmin (nucleolar phosphoprotein B23, numatrin) (NPM1), transcript variant 1, mRNA [NM_002520]	1.0851176	0.024948223
A_24_P139901	*ZBTB8OS*	Homo sapiens zinc finger and BTB domain containing 8 opposite strand (ZBTB8OS), mRNA [NM_178547]	1.5587596	0.03601631

A_21_P0010044	*GYPC*	Homo sapiens glycophorin C (Gerbich blood group) (GYPC), transcript variant 1, mRNA [NM_002101]	-0.99163556	0.031377234
A_24_P397386	*lnc-PLTP-1*	LNCipedia lincRNA (lnc-PLTP-1), lincRNA [lnc-PLTP-1:1]	-0.55642605	0.048618574
A_23_P41818	*LIFR*	Homo sapiens leukemia inhibitory factor receptor alpha (LIFR), transcript variant 2, mRNA [NM_002310]	-0.53998494	0.03754682
A_23_P48455	*ZFR*	Homo sapiens zinc finger RNA binding protein (ZFR), mRNA [NM_016107]	-1.3421918	0.03032242
A_24_P191417	*AMN*	Homo sapiens amnion associated transmembrane protein (AMN), mRNA [NM_030943]	0.6829832	0.042994436
A_24_P142228	*NAB1*	Homo sapiens NGFI-A binding protein 1 (EGR1 binding protein 1) (NAB1), mRNA [NM_005966]	-1.9833406	0.03012347
A_32_P109036	*RPL13*	Homo sapiens ribosomal protein L13 (RPL13), transcript variant 2, mRNA [NM_033251]	-0.71242833	0.045346793
A_33_P3353581	*ZNF493*	Homo sapiens zinc finger protein 493 (ZNF493), transcript variant 3, mRNA [NM_001076678]	1.0930239	0.042799078
A_33_P3214314	*IGBP1*	Homo sapiens immunoglobulin (CD79A) binding protein 1 (IGBP1), mRNA [NM_001551]	-0.7162459	0.048618473
A_23_P82762	*CCSER2*	Homo sapiens coiled-coil serine-rich protein 2 (CCSER2), transcript variant 3, mRNA [NM_001284241]	-0.525703	0.048690848
A_22_P00010513	*ZNF596*	Homo sapiens zinc finger protein 596 (ZNF596), transcript variant 1, mRNA [NM_001042416]	-0.88201296	0.03376993
A_33_P3336720	*lnc-NBPF6-2*	Homo sapiens cDNA clone IMAGE:5753455. [BC053880]	-0.92954016	0.046965998
A_22_P00024512	*HAMP*	Homo sapiens hepcidin antimicrobial peptide (HAMP), mRNA [NM_021175]	-1.147958	0.03220128
A_23_P436476	*JARID2-AS1*	Homo sapiens JARID2 antisense RNA 1 (JARID2-AS1), long non-coding RNA [NR_120502]	-1.9265239	0.031377234
A_24_P52004	*RBM34*	Homo sapiens RNA binding motif protein 34 (RBM34), transcript variant 1, mRNA [NM_015014]	-0.25542107	0.03138659
A_23_P66525	*PDS5A*	Homo sapiens PDS5 cohesin associated factor A (PDS5A), transcript variant 1, mRNA [NM_001100399]	1.3934294	0.03811336
A_24_P100742	*HS3ST3A1*	heparan sulfate (glucosamine) 3-O-sulfotransferase 3A1 [Source:HGNC Symbol;Acc:HGNC:5196] [ENST00000284110]	1.1822231	0.026497496

A_24_P810697	*ADD1*	Homo sapiens adducin 1 (alpha) (ADD1), transcript variant 2, mRNA [NM_014189]	-0.8348887	0.036131933
A_23_P91487	*MXRA7*	Homo sapiens matrix-remodelling associated 7 (MXRA7), transcript variant 2, mRNA [NM_001008529]	-1.1282746	0.036427937
A_23_P255317	*RWDD2B*	Homo sapiens RWD domain containing 2B (RWDD2B), mRNA [NM_016940]	1.425252	0.028573772
A_23_P123563	*RNF14*	Homo sapiens ring finger protein 14 (RNF14), transcript variant 1, mRNA [NM_004290]	-1.2004281	0.03708757
A_23_P501007	*RPS6*	Homo sapiens ribosomal protein S6 (RPS6), mRNA [NM_001010]	-0.8518021	0.03718595
A_22_P00012456	*EFEMP1*	Homo sapiens EGF containing fibulin-like extracellular matrix protein 1 (EFEMP1), transcript variant 2, mRNA [NM_001039348]	-0.88184977	0.029808238
A_23_P88817	*LINC00176*	Homo sapiens long intergenic non-protein coding RNA 176 (LINC00176), transcript variant 2, long non-coding RNA [NR_027687]	-0.8340435	0.028573772
A_33_P3213374	*MLYCD*	Homo sapiens malonyl-CoA decarboxylase (MLYCD), mRNA [NM_012213]	-0.49194738	0.036131933
A_23_P373568	*CITED2*	Homo sapiens Cbp/p300-interacting transactivator, with Glu/Asp-rich carboxy-terminal domain, 2 (CITED2), transcript variant 1, mRNA [NM_006079]	-2.2584755	0.029808238
A_33_P3280044	*FUZ*	Homo sapiens fuzzy planar cell polarity protein (FUZ), transcript variant 1, mRNA [NM_025129]	1.6598899	0.043984637
A_33_P3228739	*ANKRD11*	Homo sapiens ankyrin repeat domain 11 (ANKRD11), transcript variant 1, mRNA [NM_001256182]	-1.4628893	0.047708835
A_24_P194508	*LRRC3C*	Homo sapiens leucine rich repeat containing 3C (LRRC3C), mRNA [NM_001195545]	-1.6955299	0.024948223
A_33_P3353816	*SPIN2B*	Homo sapiens spindlin family, member 2B (SPIN2B), transcript variant 3, mRNA [NM_001006683]	-0.32453656	0.042850092
A_24_P12413	*ITGA4*	Homo sapiens integrin, alpha 4 (antigen CD49D, alpha 4 subunit of VLA-4 receptor) (ITGA4), mRNA [NM_000885]	-0.78027534	0.03403978
A_23_P420256	*TRAM2*	Homo sapiens translocation associated membrane protein 2 (TRAM2), mRNA [NM_012288]	1.0903364	0.040277198

A_23_P380998	*C2orf43*	Homo sapiens chromosome 2 open reading frame 43 (C2orf43), transcript variant 1, mRNA [NM_021925]	-0.67210555	0.041609965
A_23_P97632	*R3HDM1*	Homo sapiens R3H domain containing 1 (R3HDM1), transcript variant 2, mRNA [NM_015361]	-2.4294477	0.033583984
A_22_P00000129	*EPRS*	Homo sapiens glutamyl-prolyl-tRNA synthetase (EPRS), mRNA [NM_004446]	-1.586894	0.030294474
A_23_P404565	*RIMKLB*	Homo sapiens ribosomal modification protein rimK-like family member B (RIMKLB), transcript variant 3, non-coding RNA [NR_123740]	-1.453893	0.04793844
A_33_P3331451	*SLC2A11*	Homo sapiens solute carrier family 2 (facilitated glucose transporter), member 11 (SLC2A11), transcript variant 1, mRNA [NM_030807]	-0.41988972	0.038727812
A_23_P16743	*TGFBR1*	Homo sapiens transforming growth factor, beta receptor 1 (TGFBR1), transcript variant 1, mRNA [NM_004612]	-1.2357967	0.024948223
A_33_P3284019	*MGAT5*	Homo sapiens mannosyl (alpha-1,6-)-glycoprotein beta-1,6-N-acetyl-glucosaminyltransferase (MGAT5), mRNA [NM_002410]	-1.8266768	0.032993637
A_23_P33759	*FOXP4*	Homo sapiens forkhead box P4 (FOXP4), transcript variant 1, mRNA [NM_001012426]	-0.76396567	0.032261204
A_22_P00019757	*DHRS3*	Homo sapiens dehydrogenase/reductase (SDR family) member 3 (DHRS3), mRNA [NM_004753]	-0.91928744	0.034342222
A_23_P217609	*RPS3A*	Homo sapiens ribosomal protein S3A (RPS3A), transcript variant 1, mRNA [NM_001006]	-2.0512335	0.02737376
A_23_P426472	*RPL36A*	Homo sapiens ribosomal protein L36a (RPL36A), transcript variant 1, mRNA [NM_021029]	-2.3460045	0.028583162
A_23_P30805	*ZNF45*	Homo sapiens zinc finger protein 45 (ZNF45), mRNA [NM_003425]	2.1746922	0.033392303
A_23_P28969	*HIST1H4J*	Homo sapiens histone cluster 1, H4j (HIST1H4J), mRNA [NM_021968]	-1.1223025	0.029751904
A_24_P206047	*CHMP4B*	Homo sapiens charged multivesicular body protein 4B (CHMP4B), mRNA [NM_176812]	-0.7385876	0.03208779
A_22_P00010880	*SLC25A4*	Homo sapiens solute carrier family 25 (mitochondrial carrier; adenine	-1.0936605	0.03319862

		nucleotide translocator), member 4 (SLC25A4), mRNA [NM_001151]		
A_33_P3223082	*lnc-NPB-3*	BC001513 VPS18 protein {Homo sapiens} (exp=-1; wgp=0; cg=0), partial (4%) [THC2772516]	-1.0167922	0.041628964
A_32_P97169	*MRPL19*	Homo sapiens mitochondrial ribosomal protein L19 (MRPL19), mRNA [NM_014763]	-1.037066	0.028573772
A_33_P3362148	*GPC6*	Homo sapiens glypican 6 (GPC6), mRNA [NM_005708]	-0.8928208	0.03668808
A_33_P3311371	*TTC28-AS1*	Homo sapiens TTC28 antisense RNA 1 (TTC28-AS1), transcript variant 1, long non-coding RNA [NR_026963]	-0.854104	0.04525541
A_33_P3274304	*PDLIM2*	Homo sapiens PDZ and LIM domain 2 (mystique) (PDLIM2), transcript variant 3, mRNA [NM_198042]	-0.5649414	0.045331508
A_33_P3256868	*WIPF2*	Homo sapiens WAS/WASL interacting protein family, member 2 (WIPF2), mRNA [NM_133264]	-3.3287973	0.03436134
A_32_P104000	*ZNF83*	Homo sapiens zinc finger protein 83 (ZNF83), transcript variant 10, mRNA [NM_001277945]	-0.8101095	0.036667652
A_33_P3378920	*DCUN1D3*	Homo sapiens DCN1, defective in cullin neddylation 1, domain containing 3 (DCUN1D3), mRNA [NM_173475]	-0.6974459	0.039287783
A_24_P65616	*RBM14-RBM4*	Homo sapiens RBM14-RBM4 readthrough (RBM14-RBM4), transcript variant 1, mRNA [NM_001198845]	-0.7269837	0.030578624
A_23_P500206	*PVR*	Homo sapiens poliovirus receptor (PVR), transcript variant 1, mRNA [NM_006505]	-0.9361894	0.042034253
A_22_P00015046	*IL17RE*	Homo sapiens interleukin 17 receptor E (IL17RE), transcript variant 5, mRNA [NM_153483]	-0.9805131	0.047266874
A_23_P74178	*NPR1*	natriuretic peptide receptor 1 [Source:HGNC Symbol;Acc:HGNC:7943] [ENST00000413826]	-0.8240938	0.03138659
A_24_P56484	*HCRTR1*	Homo sapiens hypocretin (orexin) receptor 1 (HCRTR1), mRNA [NM_001525]	-1.2955347	0.03770023
A_23_P58912	*BRMS1L*	Homo sapiens breast cancer metastasis-suppressor 1-like (BRMS1L), mRNA [NM_032352]	0.97796524	0.045708336
A_33_P3336657	*SLC35A1*	Homo sapiens solute carrier family 35 (CMP-sialic acid transporter), member	1.5734866	0.033091284

		A1 (SLC35A1), transcript variant 1, mRNA [NM_006416]		
A_23_P134008	*PLEKHM3*	Homo sapiens pleckstrin homology domain containing, family M, member 3 (PLEKHM3), mRNA [NM_001080475]	2.2223067	0.02453978
A_23_P20558	*USP45*	ubiquitin specific peptidase 45 [Source:HGNC Symbol;Acc:HGNC:20080] [ENST00000472914]	-1.2095639	0.03590667
A_21_P0010721	*CDC37L1*	cell division cycle 37-like 1 [Source:HGNC Symbol;Acc:HGNC:17179] [ENST00000381854]	-1.3279985	0.03621218
A_23_P367816	*LINC00869*	long intergenic non-protein coding RNA 869 [Source:EntrezGene;Acc:57234] [ENST00000430442]	-0.6419797	0.028573772
A_22_P00017580	*SNX29*	Homo sapiens sorting nexin 29 (SNX29), mRNA [NM_032167]	-0.26939866	0.03683928
A_23_P140648	*lnc-WDR67-2*	LNCipedia lincRNA (lnc-WDR67-2), lincRNA [lnc-WDR67-2:1]	-1.7832335	0.02453978
A_21_P0001273	*CYFIP1*	Homo sapiens cytoplasmic FMR1 interacting protein 1 (CYFIP1), transcript variant 1, mRNA [NM_014608]	2.3461585	0.038034573
A_33_P3883912	*lnc-LDLRAP1-1*	PREDICTED: Homo sapiens low density lipoprotein receptor adaptor protein 1 (LDLRAP1), transcript variant X3, mRNA [XM_006710560]	-1.032983	0.028573772
A_23_P380951	*ZCCHC10*	Homo sapiens zinc finger, CCHC domain containing 10 (ZCCHC10), transcript variant 4, mRNA [NM_017665]	-1.8673086	0.049235344
A_22_P00017575	*ZNF420*	Homo sapiens zinc finger protein 420 (ZNF420), mRNA [NM_144689]	-1.1097976	0.037765756
A_23_P409541	*lnc-WDR63-1*	LNCipedia lincRNA (lnc-WDR63-1), lincRNA [lnc-WDR63-1:1]	-1.4856855	0.03273351
A_23_P24077	*POLR1D*	Homo sapiens polymerase (RNA) I polypeptide D, 16kDa (POLR1D), transcript variant 2, mRNA [NM_152705]	-1.063733	0.04610903
DCP_20_3	*C10orf54*	Homo sapiens chromosome 10 open reading frame 54 (C10orf54), mRNA [NM_022153]	-1.017708	0.04519146
A_24_P16036	*lnc-LRIG2-4*	Homo sapiens mRNA; cDNA DKFZp686L05231 (from clone DKFZp686L05231). [BX648855]	-0.9393964	0.048182435

A_23_P382045	*PTRHD1*	Homo sapiens peptidyl-tRNA hydrolase domain containing 1 (PTRHD1), mRNA [NM_001013663]	-1.7112745	0.029808238
A_23_P74716	*TULP4*	Homo sapiens tubby like protein 4 (TULP4), transcript variant 1, mRNA [NM_020245]	1.5119938	0.042529937
A_23_P23102	*DEDD*	Homo sapiens death effector domain containing (DEDD), transcript variant 1, mRNA [NM_032998]	-0.71016246	0.041931406
A_23_P60565	*ZSCAN20*	Homo sapiens zinc finger and SCAN domain containing 20 (ZSCAN20), mRNA [NM_145238]	-0.69691586	0.036667652
A_24_P194962	*ZNF354A*	Homo sapiens zinc finger protein 354A (ZNF354A), mRNA [NM_005649]	-0.73964405	0.028573772
A_23_P398491	*lnc-KATNAL2-4*	LNCipedia lincRNA (lnc-KATNAL2-4), lincRNA [lnc-KATNAL2-4:1]	-0.73874736	0.037221838
A_21_P0000414	*C15orf57*	Homo sapiens chromosome 15 open reading frame 57 (C15orf57), transcript variant 4, mRNA [NM_001289132]	-1.3447165	0.024948223
A_23_P389118	*SNORD114-17*	Homo sapiens small nucleolar RNA, C/D box 114-17 (SNORD114-17), small nucleolar RNA [NR_003210]	-1.5775735	0.034810174
A_22_P00020414	*ANO6*	Homo sapiens anoctamin 6 (ANO6), transcript variant 1, mRNA [NM_001025356]	1.1478828	0.02453978
A_23_P360964	*lnc-TTC7B-2*	HY047944 RIKEN full-length enriched human cDNA library, testis Homo sapiens cDNA clone H04D168N20, mRNA sequence [HY047944]	-2.4713244	0.034293942
A_33_P3420496	*DACT3*	Homo sapiens dishevelled-binding antagonist of beta-catenin 3 (DACT3), transcript variant 1, mRNA [NM_145056]	0.89010125	0.04864667
A_32_P68746	*SNORA53*	Homo sapiens small nucleolar RNA, H/ACA box 53 (SNORA53), small nucleolar RNA [NR_003015]	-1.420701	0.04864667
A_22_P00002125	*SMU1*	Homo sapiens smu-1 suppressor of mec-8 and unc-52 homolog (C. elegans) (SMU1), mRNA [NM_018225]	1.4880992	0.028573772
A_33_P3243622	*lnc-BOLA2B-1*	Q25UY8_MYCVN (Q25UY8) Initiation factor 2:Small GTP-binding protein domain, partial (3%) [THC2532449]	-1.4380137	0.039525595
A_24_P302998	*DCAF8*	Homo sapiens DDB1 and CUL4 associated factor 8 (DCAF8), transcript variant 1, mRNA [NM_015726]	1.6532702	0.03668808
A_24_P239606	*ATP5I*	Homo sapiens ATP synthase, H+ transporting, mitochondrial Fo	-0.8474478	0.044286337

		complex, subunit E (ATP5I), transcript variant 1, mRNA [NM_007100]		
A_23_P215154	*GADD45B*	Homo sapiens growth arrest and DNA-damage-inducible, beta (GADD45B), mRNA [NM_015675]	0.8639292	0.045740407
A_24_P232365	*NUB1*	Homo sapiens negative regulator of ubiquitin-like proteins 1 (NUB1), transcript variant 2, mRNA [NM_016118]	-1.135091	0.048430257
A_33_P3286536	*APBB1IP*	Homo sapiens amyloid beta (A4) precursor protein-binding, family B, member 1 interacting protein (APBB1IP), mRNA [NM_019043]	-0.56166935	0.033583984
A_24_P849801	*FUT4*	Homo sapiens fucosyltransferase 4 (alpha (1,3) fucosyltransferase, myeloid-specific) (FUT4), mRNA [NM_002033]	-2.025649	0.02737376
A_32_P198303	*RPL22*	Homo sapiens ribosomal protein L22 (RPL22), mRNA [NM_000983]	-0.99324435	0.03338082
A_33_P3263747	*DNAJC24*	Homo sapiens DnaJ (Hsp40) homolog, subfamily C, member 24 (DNAJC24), mRNA [NM_181706]	-3.474266	0.028551029
A_24_P58881	*ISCA1*	Homo sapiens iron-sulfur cluster assembly 1 (ISCA1), mRNA [NM_030940]	-1.433409	0.028573772
A_32_P108655	*ELFN1*	Homo sapiens extracellular leucine-rich repeat and fibronectin type III domain containing 1 (ELFN1), mRNA [NM_001128636]	1.1756722	0.03403978
A_24_P945147	*AK4*	Homo sapiens adenylate kinase 4 (AK4), transcript variant 1, mRNA [NM_001005353]	-0.9902372	0.028573772
A_21_P0000209	*RABEP1*	Homo sapiens rabaptin, RAB GTPase binding effector protein 1 (RABEP1), transcript variant 1, mRNA [NM_004703]	-0.82959443	0.03403978
A_24_P360763	*SNORD4B*	Homo sapiens small nucleolar RNA, C/D box 4B (SNORD4B), small nucleolar RNA [NR_000009]	-1.2163516	0.031526815
A_33_P3221432	*CREM*	Homo sapiens cAMP responsive element modulator (CREM), transcript variant 2, mRNA [NM_001881]	-2.5191271	0.03770023
A_23_P69586	*ZNF284*	Homo sapiens zinc finger protein 284 (ZNF284), mRNA [NM_001037813]	-1.4260969	0.03227331
A_23_P309803	*FAT1*	Homo sapiens FAT atypical cadherin 1 (FAT1), mRNA [NM_005245]	0.9974394	0.035935212
A_21_P0005995	*ZNF777*	Homo sapiens zinc finger protein 777 (ZNF777), mRNA [NM_015694]	-1.1852514	0.047941346

A_23_P132595	*lnc-DMRT2-1*	LNCipedia lincRNA (lnc-DMRT2-1), lincRNA [lnc-DMRT2-1:3]	-0.8064921	0.028192867
A_33_P3243168	*VGLL4*	Homo sapiens vestigial-like family member 4 (VGLL4), transcript variant 2, mRNA [NM_014667]	-0.55935115	0.045740407
A_23_P116512	*MZF1*	Homo sapiens myeloid zinc finger 1 (MZF1), transcript variant 2, mRNA [NM_198055]	-0.6065512	0.04696214
A_33_P3325023	*PRR5L*	Homo sapiens proline rich 5 like (PRR5L), transcript variant 2, mRNA [NM_024841]	1.6091187	0.030915326
A_32_P17635	*ERLEC1*	Homo sapiens endoplasmic reticulum lectin 1 (ERLEC1), transcript variant 1, mRNA [NM_015701]	-1.9444363	0.027751032
A_24_P75920	*SRSF8*	Homo sapiens serine/arginine-rich splicing factor 8 (SRSF8), transcript variant 1, mRNA [NM_032102]	-0.6761343	0.042994436
A_33_P3266923	*CCDC159*	Homo sapiens coiled-coil domain containing 159 (CCDC159), mRNA [NM_001080503]	-1.7541275	0.03233256
A_23_P380181	*LMTK2*	Homo sapiens lemur tyrosine kinase 2 (LMTK2), mRNA [NM_014916]	-0.83073115	0.029979022
A_23_P30162	*LMO4*	Homo sapiens LIM domain only 4 (LMO4), mRNA [NM_006769]	-0.4173286	0.039863296
A_24_P383076	*MFSD8*	Homo sapiens major facilitator superfamily domain containing 8 (MFSD8), mRNA [NM_152778]	-0.943826	0.049682755
A_24_P111996	*AASDH*	Homo sapiens aminoadipate-semialdehyde dehydrogenase (AASDH), transcript variant 1, mRNA [NM_181806]	0.7893417	0.028192867
A_33_P3210622	*HFE*	Homo sapiens hemochromatosis (HFE), transcript variant 1, mRNA [NM_000410]	-1.2880073	0.046120346
A_33_P3380883	*ASB13*	Homo sapiens ankyrin repeat and SOCS box containing 13 (ASB13), transcript variant 1, mRNA [NM_024701]	-0.57190794	0.040171996
A_21_P0000525	*XPA*	Homo sapiens xeroderma pigmentosum, complementation group A (XPA), transcript variant 2, non-coding RNA [NR_027302]	-0.73409504	0.045740407
A_23_P143817	*LOC285847*	Homo sapiens uncharacterized LOC285847 (LOC285847), long non-coding RNA [NR_027117]	-0.47883958	0.02453978
A_33_P3414487	*MYLK*	Homo sapiens myosin light chain kinase (MYLK), transcript variant 1, mRNA [NM_053025]	1.5449307	0.02453978

A_21_P0014670	*WBSCR22*	Homo sapiens Williams Beuren syndrome chromosome region 22 (WBSCR22), transcript variant 1, mRNA [NM_001202560]	-1.3020043	0.041527454
A_33_P3333975	*RPS10*	Homo sapiens ribosomal protein S10 (RPS10), transcript variant 2, mRNA [NM_001014]	-1.5336376	0.033723384
A_23_P114466	*CBWD5*	Homo sapiens COBW domain containing 5 (CBWD5), transcript variant 2, mRNA [NM_001286835]	-0.6208439	0.037593983
A_22_P00014694	*TBL1Y*	Homo sapiens transducin (beta)-like 1, Y-linked (TBL1Y), transcript variant 1, mRNA [NM_033284]	-1.1382377	0.048689365
A_23_P143906	*SLC25A29*	Homo sapiens solute carrier family 25 (mitochondrial carnitine/acylcarnitine carrier), member 29 (SLC25A29), transcript variant 2, mRNA [NM_001291813]	-0.6846044	0.047665294
A_23_P76983	*MLF1*	Homo sapiens myeloid leukemia factor 1 (MLF1), transcript variant 1, mRNA [NM_022443]	-1.3866329	0.027751032
A_33_P3313635	*CCDC176*	Homo sapiens coiled-coil domain containing 176 (CCDC176), mRNA [NM_025057]	0.76795244	0.034999274
A_33_P3301559	*lnc-BOLA2B-1*	601344576F1 NIH_MGC_8 Homo sapiens cDNA clone IMAGE:3677250 5', mRNA sequence [BE561442]	-1.0100251	0.046120346
A_33_P3282693	*CLUAP1*	Homo sapiens clusterin associated protein 1 (CLUAP1), transcript variant 1, mRNA [NM_015041]	-1.7496138	0.030915326
A_22_P00011692	*OR3A4P*	Homo sapiens olfactory receptor, family 3, subfamily A, member 4 pseudogene (OR3A4P), non-coding RNA [NR_024128]	-1.7222704	0.024948223
A_23_P126393	*lnc-PDHX-1*	Homo sapiens full length insert cDNA clone ZE10H04. [AF086543]	1.631529	0.03601631
A_24_P22079	*SETDB1*	Homo sapiens SET domain, bifurcated 1 (SETDB1), transcript variant 2, mRNA [NM_012432]	-1.176813	0.04217344
A_23_P39542	*FOXO1*	Homo sapiens forkhead box O1 (FOXO1), mRNA [NM_002015]	0.6585378	0.03655047
A_23_P76291	*C2orf76*	Homo sapiens chromosome 2 open reading frame 76 (C2orf76), mRNA [NM_001017927]	3.1277342	0.048771914
A_33_P3393106	*PRR4*	Homo sapiens proline rich 4 (lacrimal) (PRR4), transcript variant 2, mRNA [NM_007244]	-0.38761717	0.04383085

A_23_P115573	*FAM188A*	Homo sapiens family with sequence similarity 188, member A (FAM188A), mRNA [NM_024948]	-2.8989987	0.033412892
A_24_P383330	*SHISA4*	Homo sapiens shisa family member 4 (SHISA4), transcript variant 1, mRNA [NM_198149]	-0.9801142	0.049235344
A_33_P3338116	*LOC100129763*	Homo sapiens cDNA FLJ44135 fis, clone THYMU2009134. [AK126123]	-1.1952271	0.043670435
A_23_P256663	*LAMB2*	Homo sapiens laminin, beta 2 (laminin S) (LAMB2), mRNA [NM_002292]	-1.1710997	0.044286337
A_24_P8088	*GALR3*	Homo sapiens galanin receptor 3 (GALR3), mRNA [NM_003614]	-1.2226523	0.03794058
A_21_P0009442	*RIOK1*	Homo sapiens RIO kinase 1 (RIOK1), transcript variant 2, mRNA [NM_153005]	0.628775	0.042260204
A_23_P125717	*LINC01540*	Homo sapiens long intergenic non-protein coding RNA 1540 (LINC01540), transcript variant 1, long non-coding RNA [NR_110429]	-1.027627	0.028573772
A_33_P3298661	*NAP1L3*	Homo sapiens nucleosome assembly protein 1-like 3 (NAP1L3), mRNA [NM_004538]	-2.2448	0.04823297
A_23_P352535	*ZNF148*	Homo sapiens zinc finger protein 148 (ZNF148), mRNA [NM_021964]	-0.70280385	0.04434183
A_24_P941441	*PPP1R16B*	Homo sapiens protein phosphatase 1, regulatory subunit 16B (PPP1R16B), transcript variant 1, mRNA [NM_015568]	1.9164943	0.04447692
A_21_P0013984	*GNA13*	Homo sapiens guanine nucleotide binding protein (G protein), alpha 13 (GNA13), transcript variant 1, mRNA [NM_006572]	-1.5750893	0.03297842
A_24_P337657	*AK4*	Homo sapiens adenylate kinase 4 (AK4), transcript variant 1, mRNA [NM_001005353]	-0.68582916	0.031578258
A_21_P0003892	*SRF*	Homo sapiens serum response factor (c-fos serum response element-binding transcription factor) (SRF), transcript variant 1, mRNA [NM_003131]	1.5011568	0.041182548
A_23_P344451	*CYBRD1*	Homo sapiens cytochrome b reductase 1 (CYBRD1), transcript variant 1, mRNA [NM_024843]	-2.5241609	0.03405808
A_24_P242609	*HDGFRP3*	Homo sapiens hepatoma-derived growth factor, related protein 3 (HDGFRP3), mRNA [NM_016073]	-1.0343323	0.036041338
A_23_P415827	*KLHL12*	Homo sapiens kelch-like family member 12 (KLHL12), transcript variant 2, mRNA [NM_021633]	1.0256678	0.033095628

A_33_P3394789	*SEPT8*	Homo sapiens septin 8 (SEPT8), transcript variant 2, mRNA [NM_015146]	-0.8393727	0.043885134
A_19_P00811940	*LOC100653296*	PREDICTED: Homo sapiens uncharacterized LOC100653296 (LOC100653296), misc_RNA [XR_424298]	-0.736896	0.041547265
A_33_P3236177	*lnc-SLC35A5-1*	ALU1_HUMAN (P39188) Alu subfamily J sequence contamination warning entry, partial (13%) [THC2669783]	-0.8797881	0.03702924
A_24_P365807	*ANG*	Homo sapiens angiogenin, ribonuclease, RNase A family, 5 (ANG), transcript variant 1, mRNA [NM_001145]	-1.0696275	0.028192867
A_23_P251680	*EFNB1*	Homo sapiens ephrin-B1 (EFNB1), mRNA [NM_004429]	1.0946678	0.048931535
A_33_P7431456	*COMT*	Homo sapiens catechol-O-methyltransferase (COMT), transcript variant 1, mRNA [NM_000754]	-2.347587	0.035777844
DCP_1_4	*HLA-E*	Homo sapiens major histocompatibility complex, class I, E (HLA-E), mRNA [NM_005516]	-1.1950058	0.03136619
A_33_P3416420	*ELN*	Homo sapiens elastin (ELN), transcript variant 13, mRNA [NM_001278939]	-1.0337846	0.048182435
A_24_P287691	*ZUFSP*	Homo sapiens zinc finger with UFM1-specific peptidase domain (ZUFSP), mRNA [NM_145062]	-1.1203349	0.037765756
A_23_P66402	*AP3S2*	Homo sapiens adaptor-related protein complex 3, sigma 2 subunit (AP3S2), transcript variant 1, mRNA [NM_005829]	1.9195886	0.04964876
A_33_P3213377	*MED9*	Homo sapiens mediator complex subunit 9 (MED9), mRNA [NM_018019]	-3.9973402	0.03805463
A_23_P111865	*IMMP2L*	Homo sapiens IMP2 inner mitochondrial membrane peptidase-like (S. cerevisiae) (IMMP2L), transcript variant 1, mRNA [NM_032549]	-1.7906562	0.042886477
A_24_P941625	*ZSCAN21*	Homo sapiens zinc finger and SCAN domain containing 21 (ZSCAN21), mRNA [NM_145914]	-0.6505308	0.046072803
A_33_P3421827	*ZNF70*	Homo sapiens zinc finger protein 70 (ZNF70), mRNA [NM_021916]	-0.630372	0.048371606
A_33_P3289296	*HBZ*	Homo sapiens hemoglobin, zeta (HBZ), mRNA [NM_005332]	-2.022667	0.030293303
A_24_P186124	*TMEM37*	Homo sapiens transmembrane protein 37 (TMEM37), mRNA [NM_183240]	-1.2283243	0.027751032

A_24_P382489	*MTERF4*	Homo sapiens mitochondrial transcription termination factor 4 (MTERF4), transcript variant 1, mRNA [NM_182501]	-0.7126692	0.047879543
A_33_P3378689	*SLC27A1*	solute carrier family 27 (fatty acid transporter), member 1 [Source:HGNC Symbol;Acc:HGNC:10995] [ENST00000594962]	0.6011397	0.02737376
A_23_P22672	*WAC*	Homo sapiens WW domain containing adaptor with coiled-coil (WAC), transcript variant 1, mRNA [NM_016628]	1.4887035	0.043984637
A_22_P00014975	*ALG13*	Homo sapiens ALG13, UDP-N-acetylglucosaminyltransferase subunit (ALG13), transcript variant 7, mRNA [NM_001257231]	-0.5228436	0.03911937
A_33_P3319491	*SMARCC2*	Homo sapiens SWI/SNF related, matrix associated, actin dependent regulator of chromatin, subfamily c, member 2 (SMARCC2), transcript variant 1, mRNA [NM_003075]	-1.9116899	0.029808238
A_23_P91221	*AZIN1*	Homo sapiens antizyme inhibitor 1 (AZIN1), transcript variant 1, mRNA [NM_015878]	-0.80795956	0.043665398
A_21_P0000599	*PKIG*	Homo sapiens protein kinase (cAMP-dependent, catalytic) inhibitor gamma (PKIG), transcript variant 1, mRNA [NM_181805]	-0.90366864	0.04767117
A_23_P59397	*MKRN1*	Homo sapiens makorin ring finger protein 1 (MKRN1), transcript variant 1, mRNA [NM_013446]	0.49643776	0.03947304
A_33_P3221284	*RSPH3*	Homo sapiens radial spoke 3 homolog (Chlamydomonas) (RSPH3), mRNA [NM_031924]	-0.6176021	0.047996294
A_33_P3372451	*CCDC80*	Homo sapiens coiled-coil domain containing 80 (CCDC80), transcript variant 1, mRNA [NM_199511]	-2.4309914	0.032938797
A_33_P3244921	*DNPH1*	Homo sapiens 2'-deoxynucleoside 5'-phosphate N-hydrolase 1 (DNPH1), transcript variant 2, mRNA [NM_199184]	-0.5178425	0.03947304
A_24_P140391	*VWA9*	Homo sapiens von Willebrand factor A domain containing 9 (VWA9), transcript variant 1, mRNA [NM_001136043]	-0.93083286	0.036057316
A_23_P133470	*PIGY*	Homo sapiens phosphatidylinositol glycan anchor biosynthesis, class Y (PIGY), mRNA [NM_001042616]	-2.1257012	0.03136619

A_33_P3412975	*PJA2*	Homo sapiens praja ring finger 2, E3 ubiquitin protein ligase (PJA2), mRNA [NM_014819]	-0.6134202	0.03912857
A_23_P106204	*SIX5*	Homo sapiens SIX homeobox 5 (SIX5), mRNA [NM_175875]	-1.0335214	0.035560552
A_21_P0013440	*GSTZ1*	Homo sapiens glutathione S-transferase zeta 1 (GSTZ1), transcript variant 1, mRNA [NM_145870]	-1.0315678	0.02737376
A_33_P3287611	*LOC102725415*	Homo sapiens cDNA clone IMAGE:5278284. [BC037920]	-1.0006192	0.042907383
A_24_P322635	*KRT3*	Homo sapiens keratin 3, type II (KRT3), mRNA [NM_057088]	-0.63910216	0.04208062
A_23_P69521	*ELMO2*	Homo sapiens engulfment and cell motility 2 (ELMO2), transcript variant 2, mRNA [NM_182764]	-1.2715521	0.028573772
A_24_P271014	*CCNI*	Homo sapiens cyclin I (CCNI), mRNA [NM_006835]	0.5377268	0.034342222
A_23_P128930	*PPP4R1*	Homo sapiens protein phosphatase 4, regulatory subunit 1 (PPP4R1), transcript variant 1, mRNA [NM_001042388]	1.8930818	0.028192867
A_23_P63681	*PSMC6*	Homo sapiens proteasome (prosome, macropain) 26S subunit, ATPase, 6 (PSMC6), mRNA [NM_002806]	-1.4309314	0.045513812
A_21_P0000251	*IDE*	Homo sapiens insulin-degrading enzyme (IDE), transcript variant 1, mRNA [NM_004969]	-1.3443089	0.03601631
A_33_P3215288	*SNORA65*	Homo sapiens small nucleolar RNA, H/ACA box 65 (SNORA65), small nucleolar RNA [NR_002449]	-0.7818293	0.03405808
A_33_P3421520	*MIR646HG*	Homo sapiens cDNA FLJ46426 fis, clone THYMU3013897. [AK128288]	-0.97903967	0.043911606
A_21_P0001710	*IKZF5*	Homo sapiens IKAROS family zinc finger 5 (Pegasus) (IKZF5), transcript variant 1, mRNA [NM_001271840]	-0.6192164	0.038727812
A_24_P355649	*lnc-NAV1-1*	LNCipedia lincRNA (lnc-NAV1-1), lincRNA [lnc-NAV1-1:2]	-0.68647176	0.03419059
A_33_P3357580	*FLI1*	Homo sapiens Fli-1 proto-oncogene, ETS transcription factor (FLI1), transcript variant 1, mRNA [NM_002017]	0.7848347	0.048398294
A_23_P4161	*MRTO4*	Homo sapiens mRNA turnover 4 homolog (S. cerevisiae) (MRTO4), mRNA [NM_016183]	-0.59928596	0.03136619
A_23_P57807	*ARSG*	arylsulfatase G [Source:HGNC Symbol;Acc:HGNC:24102] [ENST00000448504]	-1.2805378	0.03808641

A_33_P3257903	*MKRN2*	Homo sapiens makorin ring finger protein 2 (MKRN2), transcript variant 1, mRNA [NM_014160]	-3.4216566	0.024948223
A_23_P90679	*GSTA4*	Homo sapiens glutathione S-transferase alpha 4 (GSTA4), mRNA [NM_001512]	0.6708436	0.044286337
A_22_P00024420	*STRADB*	Homo sapiens STE20-related kinase adaptor beta (STRADB), transcript variant 1, mRNA [NM_018571]	-0.87871224	0.03738295
A_23_P42322	*lnc-GCGR-1*	AU155391 OVARC1 Homo sapiens cDNA clone OVARC1001844 3', mRNA sequence [AU155391]	-0.78485847	0.037585977
A_22_P00001252	*COL11A2*	Homo sapiens collagen, type XI, alpha 2 (COL11A2), transcript variant 1, mRNA [NM_080680]	-1.9968296	0.031578258
A_33_P3286724	*lnc-ANKRD34B-1*	LNCipedia lincRNA (lnc-ANKRD34B-1), lincRNA [lnc-ANKRD34B-1:1]	-0.8811965	0.043712083
A_23_P255057	*PLD2*	Homo sapiens phospholipase D2 (PLD2), transcript variant 1, mRNA [NM_002663]	1.4227655	0.040442128
A_23_P58521	*SLC35A5*	Homo sapiens solute carrier family 35, member A5 (SLC35A5), mRNA [NM_017945]	-1.1415875	0.035278592
A_33_P3357530	*ERCC8*	Homo sapiens excision repair cross-complementation group 8 (ERCC8), transcript variant 2, mRNA [NM_001007233]	1.5916133	0.047350444
A_33_P3283713	*SLC12A7*	Homo sapiens solute carrier family 12 (potassium/chloride transporter), member 7 (SLC12A7), mRNA [NM_006598]	-1.0279027	0.038230915
A_23_P81248	*ABHD14A*	Homo sapiens abhydrolase domain containing 14A (ABHD14A), mRNA [NM_015407]	-1.1436594	0.02919588
A_33_P3315021	*TAF7*	Homo sapiens TAF7 RNA polymerase II, TATA box binding protein (TBP)-associated factor, 55kDa (TAF7), mRNA [NM_005642]	-0.89612114	0.034210585
A_33_P3287959	*RPL23AP7*	ribosomal protein L23a pseudogene 7 [Source:HGNC Symbol;Acc:HGNC:17336] [ENST00000416673]	-2.5216389	0.0369263
A_24_P398781	*RASA4*	Homo sapiens RAS p21 protein activator 4 (RASA4), transcript variant 1, mRNA [NM_006989]	-1.5825169	0.024948223
A_24_P159837	*IFT43*	Homo sapiens intraflagellar transport 43 (IFT43), transcript variant 1, mRNA [NM_052873]	-0.48594782	0.04639083

A_24_P808100	*ZNF302*	Homo sapiens zinc finger protein 302 (ZNF302), transcript variant 15, mRNA [NM_018675]	-1.6739657	0.033433396
A_33_P3407042	*MCTS1*	Homo sapiens malignant T cell amplified sequence 1 (MCTS1), transcript variant 1, mRNA [NM_014060]	-2.0036407	0.039287783
A_24_P942354	*B3GALT6*	Homo sapiens UDP-Gal:betaGal beta 1,3-galactosyltransferase polypeptide 6 (B3GALT6), mRNA [NM_080605]	-1.7644765	0.026467344
A_22_P00015868	*PITPNA*	Homo sapiens phosphatidylinositol transfer protein, alpha (PITPNA), mRNA [NM_006224]	-1.28329	0.03297842
A_24_P137897	*TBX18*	T-box 18 [Source:HGNC Symbol;Acc:HGNC:11595] [ENST00000330469]	-1.2791144	0.030252501
A_23_P164638	*IFRD1*	Homo sapiens interferon-related developmental regulator 1 (IFRD1), transcript variant 2, mRNA [NM_001007245]	-0.5654552	0.033786207
A_23_P166248	*ZNF419*	Homo sapiens zinc finger protein 419 (ZNF419), transcript variant 2, mRNA [NM_024691]	-0.6700764	0.041692626
A_33_P3309636	*RCAN1*	Homo sapiens regulator of calcineurin 1 (RCAN1), transcript variant 1, mRNA [NM_004414]	-1.4522554	0.041768707
A_32_P22501	*C11orf58*	Homo sapiens chromosome 11 open reading frame 58 (C11orf58), mRNA [NM_014267]	-1.4741476	0.026440267
A_23_P257003	*EIF3J-AS1*	Homo sapiens EIF3J antisense RNA 1 (head to head) (EIF3J-AS1), transcript variant 1, long non-coding RNA [NR_034170]	-1.6687031	0.034293942
A_21_P0008391	*PCSK5*	Homo sapiens proprotein convertase subtilisin/kexin type 5 (PCSK5), transcript variant 2, mRNA [NM_006200]	-1.551049	0.028573772
A_23_P46095	*lnc-DLK1-9*	LNCipedia lincRNA (lnc-DLK1-9), lincRNA [lnc-DLK1-9:1]	-1.1139648	0.029808238
A_23_P140170	*C1orf131*	Homo sapiens chromosome 1 open reading frame 131 (C1orf131), transcript variant 1, mRNA [NM_152379]	-0.94248575	0.02737376
A_24_P282309	*SEC23A*	Homo sapiens Sec23 homolog A (S. cerevisiae) (SEC23A), mRNA [NM_006364]	-3.3145342	0.02737376

A_23_P387523	*MYOF*	Homo sapiens myoferlin (MYOF), transcript variant 2, mRNA [NM_133337]	1.4784282	0.039287783
A_24_P20746	*ZBTB40*	Homo sapiens zinc finger and BTB domain containing 40 (ZBTB40), transcript variant 1, mRNA [NM_001083621]	-1.183134	0.02453978
A_24_P322369	*PRR16*	Homo sapiens proline rich 16 (PRR16), transcript variant 2, mRNA [NM_016644]	-0.82348543	0.03808641
A_23_P206324	*VPS4B*	Homo sapiens vacuolar protein sorting 4 homolog B (S. cerevisiae) (VPS4B), mRNA [NM_004869]	-0.72314525	0.039863296
A_21_P0003678	*HSDL1*	Homo sapiens hydroxysteroid dehydrogenase like 1 (HSDL1), transcript variant 1, mRNA [NM_031463]	-1.009451	0.03431701
A_32_P38637	*lnc-SAP30-2*	LNCipedia lincRNA (lnc-SAP30-2), lincRNA [lnc-SAP30-2:2]	-1.2746578	0.027751032
A_33_P3306177	*KRBA1*	Homo sapiens KRAB-A domain containing 1 (KRBA1), transcript variant 1, mRNA [NM_032534]	-1.0590093	0.02737376
A_23_P87279	*TAPT1*	Homo sapiens transmembrane anterior posterior transformation 1 (TAPT1), mRNA [NM_153365]	-0.93990314	0.043712083
A_33_P3408305	*TRPM5*	Homo sapiens transient receptor potential cation channel, subfamily M, member 5 (TRPM5), mRNA [NM_014555]	-2.47621	0.038309526
A_33_P3396886	*CERS3*	Homo sapiens ceramide synthase 3 (CERS3), transcript variant 4, mRNA [NM_178842]	-0.92437595	0.027751032
A_33_P3215575	*C11orf52*	Homo sapiens chromosome 11 open reading frame 52 (C11orf52), mRNA [NM_080659]	-1.1651037	0.02737376
A_33_P3344816	*ARHGEF10L*	Homo sapiens Rho guanine nucleotide exchange factor (GEF) 10-like (ARHGEF10L), transcript variant 1, mRNA [NM_018125]	2.0968585	0.045486424
A_24_P100761	*ZNF813*	Homo sapiens zinc finger protein 813 (ZNF813), mRNA [NM_001004301]	1.0046412	0.049758088
A_23_P110961	*BCAS3*	Homo sapiens breast carcinoma amplified sequence 3 (BCAS3), transcript variant 2, mRNA [NM_017679]	-1.6492817	0.045740407
A_33_P3370404	*MPC1*	Homo sapiens mitochondrial pyruvate carrier 1 (MPC1), transcript variant 1, mRNA [NM_016098]	-1.1999959	0.03297842

A_23_P18806	*PANX1*	Homo sapiens pannexin 1 (PANX1), mRNA [NM_015368]	-0.810645	0.03436134
A_24_P924591	*YIPF5*	Homo sapiens Yip1 domain family, member 5 (YIPF5), transcript variant 1, mRNA [NM_001024947]	-1.0580932	0.047879543
A_23_P209032	*VEZF1*	Homo sapiens vascular endothelial zinc finger 1 (VEZF1), mRNA [NM_007146]	-1.092433	0.04250221
A_33_P3297978	*ZNF302*	Homo sapiens zinc finger protein 302 (ZNF302), transcript variant 1, mRNA [NM_018443]	-0.58184385	0.048019953
A_23_P355385	*MYO1E*	Homo sapiens myosin IE (MYO1E), mRNA [NM_004998]	-0.7087252	0.047886103
A_22_P00006772	*PPP6C*	Homo sapiens protein phosphatase 6, catalytic subunit (PPP6C), transcript variant 2, mRNA [NM_002721]	-0.33401176	0.044286337
A_32_P4403	*lnc-FUS-1*	Homo sapiens mRNA; cDNA DKFZp667F205 (from clone DKFZp667F205). [AL713703]	-0.96176404	0.035515364
A_33_P3344951	*C16orf72*	Homo sapiens chromosome 16 open reading frame 72 (C16orf72), mRNA [NM_014117]	-1.4374272	0.037765756
A_23_P157147	*JMJD7*	Homo sapiens jumonji domain containing 7 (JMJD7), mRNA [NM_001114632]	-0.45077416	0.046962585
A_33_P3393694	*ZDHHC4*	Homo sapiens zinc finger, DHHC-type containing 4 (ZDHHC4), transcript variant 3, mRNA [NM_018106]	1.6508491	0.041527454
A_23_P204503	*GLTSCR2*	Homo sapiens glioma tumor suppressor candidate region gene 2 (GLTSCR2), mRNA [NM_015710]	-1.076782	0.026497496
A_33_P3529860	*PRKAB1*	Homo sapiens protein kinase, AMP-activated, beta 1 non-catalytic subunit (PRKAB1), mRNA [NM_006253]	1.0064058	0.03811336
A_33_P3300273	*KDM2B*	Homo sapiens lysine (K)-specific demethylase 2B (KDM2B), transcript variant 2, mRNA [NM_001005366]	-0.799309	0.029642703
A_21_P0014899	*C1orf52*	Homo sapiens chromosome 1 open reading frame 52 (C1orf52), transcript variant 1, mRNA [NM_198077]	-0.8511522	0.042260204
DCP_1_2	*SARM1*	Homo sapiens sterile alpha and TIR motif containing 1 (SARM1), mRNA [NM_015077]	-0.5942807	0.028573772
A_23_P340722	*SNRPE*	Homo sapiens small nuclear ribonucleoprotein polypeptide E (SNRPE), mRNA [NM_003094]	-0.3626118	0.036364187
A_23_P91702	*XPOT*	Homo sapiens exportin, tRNA (XPOT), mRNA [NM_007235]	-1.2835773	0.04412469

A_23_P139929	*EIF3D*	Homo sapiens eukaryotic translation initiation factor 3, subunit D (EIF3D), mRNA [NM_003753]	1.8208463	0.036667652
A_33_P3554053	*ERP29*	Homo sapiens endoplasmic reticulum protein 29 (ERP29), transcript variant 1, mRNA [NM_006817]	0.6113791	0.038322687
A_23_P54230	*LINC00106*	UI-H-ED1-axv-n-20-0-UI.s1 NCI_CGAP_ED1 Homo sapiens cDNA clone IMAGE:5834323 3', mRNA sequence [BQ009527]	-2.2671494	0.035935212
A_23_P143713	*ZC3H14*	Homo sapiens zinc finger CCCH-type containing 14 (ZC3H14), transcript variant 2, mRNA [NM_207660]	-0.88861775	0.042693015
A_23_P97250	*APOBEC3G*	Homo sapiens apolipoprotein B mRNA editing enzyme, catalytic polypeptide-like 3G (APOBEC3G), mRNA [NM_021822]	-1.2810476	0.028546385
A_33_P3289113	*GON4L*	Homo sapiens gon-4-like (C. elegans) (GON4L), transcript variant 3, mRNA [NM_001282860]	1.363306	0.027751032
A_33_P3307447	*COX11*	cytochrome c oxidase assembly homolog 11 (yeast) [Source:HGNC Symbol;Acc:HGNC:2261] [ENST00000572088]	-0.621551	0.049393076
A_21_P0010927	*RPL32P3*	ribosomal protein L32 pseudogene 3 [Source:HGNC Symbol;Acc:HGNC:27024] [ENST00000359025]	-0.90170896	0.03403978
A_23_P70168	*LINC00502*	Homo sapiens long intergenic non-protein coding RNA 502 (LINC00502), long non-coding RNA [NR_047467]	-0.79682106	0.03136619
A_23_P94204	*TARS*	Homo sapiens threonyl-tRNA synthetase (TARS), transcript variant 1, mRNA [NM_152295]	-1.3732728	0.033726536
A_33_P3531857	*OXR1*	Homo sapiens oxidation resistance 1 (OXR1), transcript variant 2, mRNA [NM_181354]	-0.52017456	0.03323599
A_24_P294703	*DARS*	Homo sapiens aspartyl-tRNA synthetase (DARS), transcript variant 1, mRNA [NM_001349]	1.7128823	0.042738907
A_22_P00007979	*PRRC1*	Homo sapiens proline-rich coiled-coil 1 (PRRC1), transcript variant 2, mRNA [NM_130809]	-0.6903234	0.030915326
A_33_P3328666	*IDS*	Homo sapiens iduronate 2-sulfatase (IDS), transcript variant 2, mRNA [NM_006123]	-0.9459026	0.046256438

A_23_P167444	*CLTB*	Homo sapiens clathrin, light chain B (CLTB), transcript variant 2, mRNA [NM_007097]	-0.9674759	0.04465309
A_23_P214658	*NSA2*	Homo sapiens NSA2 ribosome biogenesis homolog (S. cerevisiae) (NSA2), transcript variant 1, mRNA [NM_014886]	-0.89384925	0.038287252
A_21_P0000413	*PBX2*	Homo sapiens pre-B-cell leukemia homeobox 2 (PBX2), mRNA [NM_002586]	-1.1893976	0.034825742
A_23_P351467	*SNORD114-16*	Homo sapiens small nucleolar RNA, C/D box 114-16 (SNORD114-16), small nucleolar RNA [NR_003209]	1.0360215	0.02453978
A_24_P333802	*CMAHP*	Homo sapiens cytidine monophospho-N-acetylneuraminic acid hydroxylase, pseudogene (CMAHP), transcript variant 1, non-coding RNA [NR_002174]	-1.5449846	0.02487949
A_33_P3303372	*MAGT1*	Homo sapiens magnesium transporter 1 (MAGT1), mRNA [NM_032121]	-1.4131738	0.03205687
A_19_P00322711	*PARD3*	Homo sapiens par-3 family cell polarity regulator (PARD3), transcript variant 9, mRNA [NM_001184792]	1.0555078	0.047218688
A_33_P3271111	*LINC00662*	Homo sapiens long intergenic non-protein coding RNA 662 (LINC00662), long non-coding RNA [NR_027301]	-1.6389973	0.046853997
A_24_P153511	*NINJ1*	Homo sapiens ninjurin 1 (NINJ1), mRNA [NM_004148]	-1.030225	0.03403978
A_33_P3313075	*OSBPL8*	Homo sapiens oxysterol binding protein-like 8 (OSBPL8), transcript variant 1, mRNA [NM_020841]	-1.2822531	0.029808238
A_33_P3400943	*OMA1*	Homo sapiens OMA1 zinc metallopeptidase (OMA1), mRNA [NM_145243]	0.97955173	0.04846618
A_33_P3408962	*CDIP1*	Homo sapiens cell death-inducing p53 target 1 (CDIP1), transcript variant 1, mRNA [NM_001199054]	-1.0683804	0.04820357
A_23_P17490	*SCN8A*	Homo sapiens sodium channel, voltage gated, type VIII alpha subunit (SCN8A), transcript variant 1, mRNA [NM_014191]	-2.763247	0.028192867
A_32_P31182	*TMEM230*	Homo sapiens transmembrane protein 230 (TMEM230), transcript variant 2, mRNA [NM_001009924]	-0.88218546	0.03504642
A_24_P165656	*RPL7*	Homo sapiens ribosomal protein L7 (RPL7), mRNA [NM_000971]	-1.9510095	0.03403978
A_23_P135257	*PRKD3*	Homo sapiens protein kinase D3 (PRKD3), mRNA [NM_005813]	-0.576396	0.031569958

A_24_P116242	*PRSS3*	Homo sapiens protease, serine, 3 (PRSS3), transcript variant 2, mRNA [NM_002771]	-1.3429972	0.033091284
A_24_P339429	*KLHDC2*	Homo sapiens kelch domain containing 2 (KLHDC2), mRNA [NM_014315]	-1.8115501	0.013003563
A_33_P3387300	*KCNJ12*	Homo sapiens potassium channel, inwardly rectifying subfamily J, member 12 (KCNJ12), mRNA [NM_021012]	-1.283234	0.035560552
A_23_P8339	*FXR1*	Homo sapiens fragile X mental retardation, autosomal homolog 1 (FXR1), transcript variant 2, mRNA [NM_001013438]	2.6388001	0.046325136
A_33_P3374463	*MRPL18*	Homo sapiens mitochondrial ribosomal protein L18 (MRPL18), mRNA [NM_014161]	-1.6162434	0.02737376
A_33_P3308914	*MFAP3*	Homo sapiens microfibrillar-associated protein 3 (MFAP3), transcript variant 3, mRNA [NM_001242336]	-2.8897424	0.03136619
A_33_P3420486	*CIB2*	Homo sapiens calcium and integrin binding family member 2 (CIB2), transcript variant 4, mRNA [NM_001301224]	-0.92481524	0.029808238
A_21_P0000553	*HIF1A*	Homo sapiens hypoxia inducible factor 1, alpha subunit (basic helix-loop-helix transcription factor) (HIF1A), transcript variant 2, mRNA [NM_181054]	-1.0469558	0.030915326
A_23_P345942	*MTERF4*	Homo sapiens mitochondrial transcription termination factor 4 (MTERF4), transcript variant 2, non-coding RNA [NR_028049]	-2.36122	0.028573772
A_23_P72568	*NDUFAF2*	Homo sapiens NADH dehydrogenase (ubiquinone) complex I, assembly factor 2 (NDUFAF2), mRNA [NM_174889]	1.2822582	0.029808238
A_22_P00000959	*SNX4*	Homo sapiens sorting nexin 4 (SNX4), transcript variant 1, mRNA [NM_003794]	-2.0348384	0.03220128
A_33_P3414312	*lnc-AL020996.1-1*	NM_115901 FK506 binding {Arabidopsis thaliana} (exp=-1; wgp=0; cg=0), partial (6%) [THC2686321]	-1.3635844	0.044750392
A_24_P74487	*USP32*	Homo sapiens ubiquitin specific peptidase 32 (USP32), mRNA [NM_032582]	-0.7458172	0.027751032
A_22_P00013055	*SMIM11*	Homo sapiens small integral membrane protein 11 (SMIM11), mRNA [NM_058182]	-0.8130401	0.02737376

A_23_P385938	*DLC1*	Homo sapiens DLC1 Rho GTPase activating protein (DLC1), transcript variant 1, mRNA [NM_182643]	-0.7114231	0.042693015
A_24_P385611	*PAN3*	Homo sapiens PAN3 poly(A) specific ribonuclease subunit (PAN3), mRNA [NM_175854]	1.8680317	0.04876218
A_24_P252794	*SP100*	Homo sapiens SP100 nuclear antigen (SP100), transcript variant 2, mRNA [NM_003113]	1.25799	0.03912857
A_22_P00012823	*CUL2*	Homo sapiens cullin 2 (CUL2), transcript variant 3, mRNA [NM_003591]	-1.2918315	0.04791796
A_23_P119130	*LOC728095*	Homo sapiens uncharacterized LOC728095 (CTB-174D11.1), long non-coding RNA [NR_109897]	-0.7392186	0.046120346
A_32_P128656	*RPS19*	Homo sapiens ribosomal protein S19 (RPS19), mRNA [NM_001022]	-1.0134845	0.04876218
A_33_P3881056	*MID1*	Homo sapiens midline 1 (MID1), transcript variant 1, mRNA [NM_000381]	-1.5538377	0.047043864
A_23_P415633	*LOC100996405*	PREDICTED: Homo sapiens uncharacterized LOC100996405 (LOC100996405), mRNA [XM_003846604]	1.780586	0.032261204
A_23_P63243	*SGK494*	Homo sapiens uncharacterized serine/threonine-protein kinase SgK494 (SGK494), mRNA [NM_001174103]	-0.9018111	0.027751032
A_24_P209113	*C1orf43*	Homo sapiens chromosome 1 open reading frame 43 (C1orf43), transcript variant 2, mRNA [NM_138740]	-0.75501317	0.029979022
A_33_P3286608	*NDFIP1*	Homo sapiens Nedd4 family interacting protein 1 (NDFIP1), mRNA [NM_030571]	-1.1485766	0.043712083
A_23_P158096	*PGBD2*	Homo sapiens piggyBac transposable element derived 2 (PGBD2), transcript variant 1, mRNA [NM_170725]	0.42607352	0.037249662
A_23_P203819	*COL27A1*	Homo sapiens collagen, type XXVII, alpha 1 (COL27A1), mRNA [NM_032888]	-0.9207553	0.034210585
A_23_P150350	*GOLGA3*	Homo sapiens golgin A3 (GOLGA3), transcript variant 1, mRNA [NM_005895]	-0.85950273	0.03338082
A_21_P0013930	*C11orf1*	Homo sapiens chromosome 11 open reading frame 1 (C11orf1), mRNA [NM_022761]	-0.83767736	0.045638688

A_22_P00004541	*NSMCE2*	Homo sapiens non-SMC element 2, MMS21 homolog (S. cerevisiae) (NSMCE2), mRNA [NM_173685]	-0.7108491	0.02453978
A_33_P3252989	*PRRT3-AS1*	Homo sapiens PRRT3 antisense RNA 1 (PRRT3-AS1), long non-coding RNA [NR_046734]	-0.6726785	0.038309526
A_32_P18072	*ZSWIM8*	Homo sapiens zinc finger, SWIM-type containing 8 (ZSWIM8), transcript variant 1, mRNA [NM_015037]	-0.76755214	0.045638688
A_33_P3224045	*lnc-RLBP1-1*	LNCipedia lincRNA (lnc-RLBP1-1), lincRNA [lnc-RLBP1-1:1]	-1.2328181	0.02598378
A_23_P5912	*DAPK3*	Homo sapiens death-associated protein kinase 3 (DAPK3), mRNA [NM_001348]	-3.1746016	0.03504642
A_23_P92954	*YTHDF1*	Homo sapiens YTH N(6)-methyladenosine RNA binding protein 1 (YTHDF1), mRNA [NM_017798]	-0.7586501	0.044610508
A_33_P3232277	*HSD17B4*	Homo sapiens hydroxysteroid (17-beta) dehydrogenase 4 (HSD17B4), transcript variant 2, mRNA [NM_000414]	3.048894	0.028573772
A_23_P98015	*CCNG1*	Homo sapiens cyclin G1 (CCNG1), transcript variant 1, mRNA [NM_004060]	-1.0387824	0.037240352
A_33_P3248354	*CUTC*	Homo sapiens cutC copper transporter (CUTC), mRNA [NM_015960]	-0.5291569	0.028573772
A_23_P415706	*OLFM1*	Homo sapiens olfactomedin 1 (OLFM1), transcript variant 2, mRNA [NM_006334]	-1.1686921	0.037548345
A_24_P928604	*GPR133*	Homo sapiens G protein-coupled receptor 133 (GPR133), mRNA [NM_198827]	-0.9384959	0.028573772
A_23_P13438	*CYP4F8*	Homo sapiens cytochrome P450, family 4, subfamily F, polypeptide 8 (CYP4F8), mRNA [NM_007253]	-0.7383473	0.04555549
A_33_P3705884	*BTBD10*	Homo sapiens BTB (POZ) domain containing 10 (BTBD10), transcript variant 2, mRNA [NM_032320]	1.4325252	0.04525541
A_33_P3233565	*LINC00662*	long intergenic non-protein coding RNA 662 [Source:HGNC Symbol;Acc:HGNC:27122] [ENST00000561521]	-1.1924826	0.026497496
A_24_P135276	*UROS*	Homo sapiens uroporphyrinogen III synthase (UROS), mRNA [NM_000375]	1.5079049	0.029994998
A_24_P377489	*USP42*	Homo sapiens ubiquitin specific peptidase 42 (USP42), mRNA [NM_032172]	-1.0984766	0.045316998

A_33_P3338693	*CCZ1*	Homo sapiens CCZ1 vacuolar protein trafficking and biogenesis associated homolog (S. cerevisiae) (CCZ1), mRNA [NM_015622]	-1.4773223	0.034375925
A_32_P129968	*SNAP25*	Homo sapiens synaptosomal-associated protein, 25kDa (SNAP25), transcript variant 1, mRNA [NM_003081]	-0.6314132	0.046072803
A_21_P0010881	*ZNF284*	zinc finger protein 284 [Source:HGNC Symbol;Acc:HGNC:13078] [ENST00000421176]	-0.65190864	0.040555127
A_33_P3303176	*FGFR1OP2*	Homo sapiens FGFR1 oncogene partner 2 (FGFR1OP2), transcript variant 3, mRNA [NM_001171888]	5.8670664	0.02453978
A_24_P127021	*MRGPRG*	Homo sapiens MAS-related GPR, member G (MRGPRG), mRNA [NM_001164377]	-1.0094899	0.035278592
A_33_P3392977	*ST13*	Homo sapiens suppression of tumorigenicity 13 (colon carcinoma) (Hsp70 interacting protein) (ST13), transcript variant 1, mRNA [NM_003932]	-0.9426849	0.034999274
A_24_P896205	*CCNDBP1*	Homo sapiens cyclin D-type binding-protein 1 (CCNDBP1), transcript variant 1, mRNA [NM_012142]	-0.9640341	0.031377234
A_23_P6561	*TBX2-AS1*	Homo sapiens TBX2 antisense RNA 1 (TBX2-AS1), transcript variant 1, long non-coding RNA [NR_125749]	2.4205291	0.04080239
A_33_P3269359	*EBLN2*	Homo sapiens endogenous Bornavirus-like nucleoprotein 2 (EBLN2), mRNA [NM_018029]	-1.3278131	0.041182548
A_23_P103503	*SPPL3*	Homo sapiens signal peptide peptidase like 3 (SPPL3), mRNA [NM_139015]	-1.1531942	0.028573772
A_22_P00004317	*MDM4*	Homo sapiens MDM4, p53 regulator (MDM4), transcript variant 1, mRNA [NM_002393]	2.8162951	0.039024077
A_22_P00001136	*PSMD5-AS1*	Homo sapiens PSMD5 antisense RNA 1 (head to head) (PSMD5-AS1), long non-coding RNA [NR_024408]	-1.0499216	0.03273351
A_23_P35989	*HNRNPKP3*	Homo sapiens heterogeneous nuclear ribonucleoprotein K pseudogene 3 (HNRNPKP3), non-coding RNA [NR_033868]	-1.1697444	0.02598378
A_21_P0014351	*ALKBH3*	Homo sapiens alkB, alkylation repair homolog 3 (E. coli) (ALKBH3), mRNA [NM_139178]	-0.57742596	0.03032242

A_32_P141724	*SP2-AS1*	Homo sapiens SP2 antisense RNA 1 (SP2-AS1), transcript variant 1, long non-coding RNA [NR_103856]	-1.4520673	0.030578624
A_23_P41734	*COMMD7*	Homo sapiens COMM domain containing 7 (COMMD7), transcript variant 1, mRNA [NM_053041]	2.04149	0.045543194
A_24_P219114	*RNF130*	Homo sapiens ring finger protein 130 (RNF130), transcript variant 1, mRNA [NM_018434]	-1.0022254	0.034306627
A_23_P92154	*SEL1L*	Homo sapiens sel-1 suppressor of lin-12-like (C. elegans) (SEL1L), transcript variant 1, mRNA [NM_005065]	-1.2863939	0.031578258
A_23_P48886	*MBD4*	Homo sapiens methyl-CpG binding domain protein 4 (MBD4), transcript variant 1, mRNA [NM_003925]	-0.60364807	0.046205126
A_24_P35891	*ADAM10*	Homo sapiens ADAM metallopeptidase domain 10 (ADAM10), mRNA [NM_001110]	-0.7860477	0.042994436
A_24_P235266	*ZNF219*	Homo sapiens zinc finger protein 219 (ZNF219), transcript variant 1, mRNA [NM_016423]	-0.9107847	0.035935212
A_21_P0011822	*GRB10*	Homo sapiens growth factor receptor-bound protein 10 (GRB10), transcript variant 4, mRNA [NM_001001555]	-0.9847872	0.034342222
A_33_P3397233	*lnc-ZAP70-2*	LNCipedia lincRNA (lnc-ZAP70-2), lincRNA [lnc-ZAP70-2:2]	-0.71666855	0.048527416
A_33_P3333810	*LOC101929256*	PREDICTED: Homo sapiens uncharacterized LOC101929256 (LOC101929256), ncRNA [XR_242748]	-1.9471712	0.03136619
A_23_P166023	*UBE2V2*	Homo sapiens ubiquitin-conjugating enzyme E2 variant 2 (UBE2V2), mRNA [NM_003350]	-2.0191407	0.027751032
A_23_P256716	*PFDN4*	Homo sapiens prefoldin subunit 4 (PFDN4), mRNA [NM_002623]	2.5031545	0.032779668
A_23_P1280	*SMARCA5*	Homo sapiens SWI/SNF related, matrix associated, actin dependent regulator of chromatin, subfamily a, member 5 (SMARCA5), mRNA [NM_003601]	-1.4799063	0.029808238
A_23_P500734	*ASCC1*	Homo sapiens activating signal cointegrator 1 complex subunit 1 (ASCC1), transcript variant 1, mRNA [NM_001198799]	-0.77334476	0.035227668
A_23_P376759	*POGZ*	Homo sapiens pogo transposable element with ZNF domain (POGZ), transcript variant 1, mRNA [NM_015100]	-0.54360795	0.04649562
A_33_P3388466	*DUSP11*	Homo sapiens dual specificity phosphatase 11 (RNA/RNP complex 1-	-0.6666731	0.045316998

		interacting) (DUSP11), mRNA [NM_003584]		
A_24_P223124	*BTN3A1*	Homo sapiens butyrophilin, subfamily 3, member A1 (BTN3A1), transcript variant 1, mRNA [NM_007048]	-0.9695622	0.03136619
A_33_P3317543	*FNDC3B*	Homo sapiens fibronectin type III domain containing 3B (FNDC3B), transcript variant 1, mRNA [NM_022763]	-0.5963997	0.045346793
A_24_P886040	*GTF2IRD2B*	Homo sapiens GTF2I repeat domain containing 2B (GTF2IRD2B), mRNA [NM_001003795]	-1.523357	0.02598378
A_22_P00019571	*DCP2*	Homo sapiens decapping mRNA 2 (DCP2), transcript variant 1, mRNA [NM_152624]	-0.60786396	0.04676237
A_23_P153236	*SNAPC1*	Homo sapiens small nuclear RNA activating complex, polypeptide 1, 43kDa (SNAPC1), mRNA [NM_003082]	-1.4190888	0.032155342
A_33_P3231140	*CHMP2A*	Homo sapiens charged multivesicular body protein 2A (CHMP2A), transcript variant 2, mRNA [NM_014453]	-0.91288656	0.04114316
A_22_P00003546	*ANKRD50*	Homo sapiens ankyrin repeat domain 50 (ANKRD50), transcript variant 2, mRNA [NM_001167882]	-1.2273654	0.026578737
A_24_P26073	*PIGBOS1*	PREDICTED: Homo sapiens uncharacterized LOC101928527 (LOC101928527), transcript variant X2, ncRNA [XR_243152]	-1.1785021	0.028573772
A_32_P32653	*LRPPRC*	Homo sapiens leucine-rich pentatricopeptide repeat containing (LRPPRC), mRNA [NM_133259]	-1.0803086	0.028573772
A_23_P64712	*SENP5*	Homo sapiens SUMO1/sentrin specific peptidase 5 (SENP5), mRNA [NM_152699]	-1.641714	0.03136619
A_33_P3212823	*TCTN2*	Homo sapiens tectonic family member 2 (TCTN2), transcript variant 1, mRNA [NM_024809]	-0.8449278	0.028189372
A_33_P3319870	*NPIPB5*	Homo sapiens nuclear pore complex interacting protein family, member B5 (NPIPB5), mRNA [NM_001135865]	-0.834917	0.043885134
A_21_P0011047	*GREM1*	Homo sapiens gremlin 1, DAN family BMP antagonist (GREM1), transcript variant 2, mRNA [NM_001191323]	-0.922704	0.033989865
A_33_P3252915	*SMYD3*	Homo sapiens SET and MYND domain containing 3 (SMYD3), transcript variant 2, mRNA [NM_022743]	-1.4897845	0.02453978

A_24_P167614	*RNF7*	Homo sapiens ring finger protein 7 (RNF7), transcript variant 5, non-coding RNA [NR_037702]	-1.3068058	0.04512774
A_22_P00024090	*INTS6*	Homo sapiens integrator complex subunit 6 (INTS6), transcript variant 1, mRNA [NM_012141]	-1.4378263	0.034210585
A_24_P103060	*lnc-GBP5-2*	Homo sapiens leucine rich repeat containing 8 family, member B, mRNA (cDNA clone IMAGE:6195030). [BC053565]	-1.5755551	0.032261204
A_23_P82693	*MCFD2*	Homo sapiens multiple coagulation factor deficiency 2 (MCFD2), transcript variant 1, mRNA [NM_139279]	-1.1121901	0.04590026
A_24_P46577	*PABPC1*	Homo sapiens poly(A) binding protein, cytoplasmic 1 (PABPC1), mRNA [NM_002568]	-0.9482604	0.044286337
A_23_P210091	*ZNRD1*	Homo sapiens zinc ribbon domain containing 1 (ZNRD1), transcript variant a, mRNA [NM_170783]	3.1477387	0.029263772
A_21_P0006870	*SUPT7L*	Homo sapiens suppressor of Ty 7 (S. cerevisiae)-like (SUPT7L), transcript variant 1, mRNA [NM_014860]	-0.81348723	0.04733987
A_33_P3252785	*lnc-ANTXRL-3*	LNCipedia lincRNA (lnc-ANTXRL-3), lincRNA [lnc-ANTXRL-3:1]	-1.0793953	0.047932126
A_24_P185158	*PLAC9*	placenta-specific 9 [Source:HGNC Symbol;Acc:HGNC:19255] [ENST00000372263]	-1.2262044	0.03071273
A_33_P3795707	*FAM134C*	Homo sapiens family with sequence similarity 134, member C (FAM134C), transcript variant 1, mRNA [NM_178126]	-0.8093515	0.0442831
A_23_P127964	*SNORA16B*	CU457294 Homo sapiens ORESTES from keratinocytes Homo sapiens cDNA, mRNA sequence [CU457294]	-0.62825185	0.034210585
A_23_P21485	*PRCP*	Homo sapiens prolylcarboxypeptidase (angiotensinase C) (PRCP), transcript variant 2, mRNA [NM_199418]	-3.035503	0.02453978
A_24_P16815	*PID1*	Homo sapiens phosphotyrosine interaction domain containing 1 (PID1), transcript variant 1, mRNA [NM_017933]	-1.0750461	0.045708336
A_33_P7518452	*ZMYM3*	Homo sapiens zinc finger, MYM-type 3 (ZMYM3), transcript variant 2, mRNA [NM_005096]	-0.48928142	0.028573772
A_23_P83028	*LRIG2*	leucine-rich repeats and immunoglobulin-like domains 2 [Source:HGNC	-0.81202745	0.045740407

		Symbol;Acc:HGNC:20889] [ENST00000361127]		
A_23_P77818	*RECK*	Homo sapiens reversion-inducing-cysteine-rich protein with kazal motifs (RECK), mRNA [NM_021111]	-2.1957712	0.033583984
A_23_P50775	*ATP5H*	Homo sapiens ATP synthase, H+ transporting, mitochondrial Fo complex, subunit d (ATP5H), transcript variant 1, mRNA [NM_006356]	-0.88552636	0.04793844
A_23_P207517	*LRFN3*	Homo sapiens leucine rich repeat and fibronectin type III domain containing 3 (LRFN3), mRNA [NM_024509]	-1.2731613	0.03403978
A_33_P3268124	*PDK2*	Homo sapiens pyruvate dehydrogenase kinase, isozyme 2 (PDK2), transcript variant 1, mRNA [NM_002611]	-1.5012681	0.034009747
A_23_P56505	*ZNF654*	Homo sapiens zinc finger protein 654 (ZNF654), mRNA [NM_018293]	-0.4221106	0.034342222
A_32_P141768	*ITGA4*	Homo sapiens integrin, alpha 4 (antigen CD49D, alpha 4 subunit of VLA-4 receptor) (ITGA4), mRNA [NM_000885]	-0.660557	0.035278592
A_23_P54692	*AGPAT4*	Homo sapiens 1-acylglycerol-3-phosphate O-acyltransferase 4 (AGPAT4), mRNA [NM_020133]	-0.7348952	0.036667652
A_24_P98249	*LOC101927910*	PREDICTED: Homo sapiens keratin-associated protein 5-5-like (LOC101927910), mRNA [XM_005256332]	-1.004162	0.02737376
A_22_P00020040	*TACC1*	Homo sapiens transforming, acidic coiled-coil containing protein 1 (TACC1), transcript variant 1, mRNA [NM_006283]	-1.2672486	0.04434183
A_23_P342744	*lnc-SPAG1-4*	HY037890 RIKEN full-length enriched human cDNA library, testis Homo sapiens cDNA clone H04D132H10, mRNA sequence [HY037890]	-0.828027	0.029808238
A_23_P21747	*LIX1L*	Homo sapiens Lix1 homolog (chicken) like (LIX1L), mRNA [NM_153713]	2.4241662	0.03338082
A_23_P200936	*CABP5*	Homo sapiens calcium binding protein 5 (CABP5), mRNA [NM_019855]	-0.7209014	0.033412892
A_33_P3268487	*MTR*	Homo sapiens 5-methyltetrahydrofolate-homocysteine methyltransferase (MTR), transcript variant 1, mRNA [NM_000254]	2.5941033	0.03947304
A_21_P0008535	*ABAT*	Homo sapiens 4-aminobutyrate aminotransferase (ABAT), transcript variant 2, mRNA [NM_000663]	-1.1988558	0.047727138

A_22_P00010775	*lnc-DLGAP5-2*	LNCipedia lincRNA (lnc-DLGAP5-2), lincRNA [lnc-DLGAP5-2:1]	1.4480661	0.029051661
A_23_P99226	*TPM1*	Homo sapiens tropomyosin 1 (alpha) (TPM1), transcript variant Tpm1.5, mRNA [NM_000366]	-2.6389532	0.03840762
A_23_P41888	*SIRT4*	Homo sapiens sirtuin 4 (SIRT4), mRNA [NM_012240]	1.5288179	0.034306627
A_24_P270144	*PHYKPL*	Homo sapiens 5-phosphohydroxy-L-lysine phospho-lyase (PHYKPL), transcript variant 2, mRNA [NM_153373]	-1.9652114	0.03297842
A_24_P58054	*CD63*	Homo sapiens CD63 molecule (CD63), transcript variant 3, mRNA [NM_001257389]	-2.5002358	0.02737376
A_23_P140848	*SLC9A8*	Homo sapiens solute carrier family 9, subfamily A (NHE8, cation proton antiporter 8), member 8 (SLC9A8), transcript variant 2, mRNA [NM_015266]	-1.0353442	0.028573772
A_33_P3320772	*MPHOSPH6*	Homo sapiens M-phase phosphoprotein 6 (MPHOSPH6), mRNA [NM_005792]	-0.9080144	0.04434183
A_33_P3398840	*ATXN1*	Homo sapiens ataxin 1 (ATXN1), transcript variant 1, mRNA [NM_000332]	-0.83266085	0.03576752
A_33_P3374085	*ZBED1*	Homo sapiens zinc finger, BED-type containing 1 (ZBED1), transcript variant 3, mRNA [NM_001171135]	-0.69976187	0.03804464
A_33_P3235690	*LRRFIP1*	Homo sapiens leucine rich repeat (in FLII) interacting protein 1 (LRRFIP1), transcript variant 3, mRNA [NM_001137552]	0.61027086	0.04368986
A_22_P00000579	*THOC2*	Homo sapiens THO complex 2 (THOC2), mRNA [NM_001081550]	-1.3167846	0.024948223
A_33_P3371144	*lnc-AC136604.1-1*	LNCipedia lincRNA (lnc-AC136604.1-1), lincRNA [lnc-AC136604.1-1:4]	-1.8410571	0.04217344
A_23_P761	*ZNF484*	Homo sapiens zinc finger protein 484 (ZNF484), transcript variant 2, mRNA [NM_001007101]	-1.2168374	0.042850092
A_33_P3309231	*PSMB4*	Homo sapiens proteasome (prosome, macropain) subunit, beta type, 4 (PSMB4), mRNA [NM_002796]	-1.6537092	0.048182435
A_33_P3250083	*HSCB*	Homo sapiens HscB mitochondrial iron-sulfur cluster co-chaperone (HSCB), mRNA [NM_172002]	-1.1666526	0.036395732
A_23_P83438	*NFATC4*	Homo sapiens nuclear factor of activated T-cells, cytoplasmic, calcineurin-dependent 4 (NFATC4),	-0.42192444	0.034293942

		transcript variant 1, mRNA [NM_001136022]		
A_23_P423331	*UBE2Z*	Homo sapiens ubiquitin-conjugating enzyme E2Z (UBE2Z), mRNA [NM_023079]	-0.28116652	0.02737376
A_23_P55682	*NTNG2*	Homo sapiens netrin G2 (NTNG2), mRNA [NM_032536]	-0.94574714	0.04638335
A_33_P3306983	*ZSCAN18*	Homo sapiens zinc finger and SCAN domain containing 18 (ZSCAN18), transcript variant 3, mRNA [NM_023926]	-1.2348301	0.02737376
A_23_P153084	*C11orf31*	Homo sapiens chromosome 11 open reading frame 31 (C11orf31), mRNA [NM_170746]	-0.7006112	0.047933746
A_23_P2705	*RALBP1*	Homo sapiens ralA binding protein 1 (RALBP1), mRNA [NM_006788]	-2.499384	0.029808238
A_33_P3234984	*LPAR6*	Homo sapiens lysophosphatidic acid receptor 6 (LPAR6), transcript variant 1, mRNA [NM_005767]	-0.6513026	0.04873285
A_24_P940125	*IFT81*	Homo sapiens intraflagellar transport 81 (IFT81), transcript variant 2, mRNA [NM_031473]	-0.6946411	0.038732324
A_33_P3350823	*CNOT6*	Homo sapiens CCR4-NOT transcription complex, subunit 6 (CNOT6), mRNA [NM_001303241]	-0.93811893	0.04250221
A_24_P331560	*ZNF639*	Homo sapiens zinc finger protein 639 (ZNF639), transcript variant 1, mRNA [NM_016331]	-0.9079366	0.03032242
A_33_P3290403	*STS*	Homo sapiens steroid sulfatase (microsomal), isozyme S (STS), mRNA [NM_000351]	-1.2581202	0.03227331
A_22_P00013027	*IMPA2*	Homo sapiens inositol(myo)-1(or 4)-monophosphatase 2 (IMPA2), mRNA [NM_014214]	-0.34514302	0.047982622
A_24_P214598	*GACAT1*	Homo sapiens gastric cancer associated transcript 1 (non-protein coding) (GACAT1), transcript variant 2, long non-coding RNA [NR_126370]	-0.6857527	0.04459763
A_33_P3308132	*PPM1K*	Homo sapiens protein phosphatase, Mg2+/Mn2+ dependent, 1K (PPM1K), mRNA [NM_152542]	-1.2507871	0.027942823
A_24_P527404	*LOC102725127*	PREDICTED: Homo sapiens uncharacterized LOC102725127 (LOC102725127), ncRNA [XR_433669]	1.7612562	0.04855821
A_33_P3347168	*BMPR1A*	Homo sapiens bone morphogenetic protein receptor, type IA (BMPR1A), mRNA [NM_004329]	2.4752133	0.028573772

DCP_1_1	*TBL1X*	Homo sapiens transducin (beta)-like 1X-linked (TBL1X), transcript variant 1, mRNA [NM_005647]	-1.3171358	0.026440267
A_21_P0008352	*CDC42EP5*	Homo sapiens CDC42 effector protein (Rho GTPase binding) 5 (CDC42EP5), mRNA [NM_145057]	-0.58784825	0.043439556
A_24_P80135	*lnc-EXD2-1*	Q21RF1_9BURK (Q21RF1) Biotin--acetyl-CoA-carboxylase ligase, partial (6%) [THC2728144]	0.7535713	0.029808238
A_33_P7289027	*PTPN18*	Homo sapiens protein tyrosine phosphatase, non-receptor type 18 (brain-derived) (PTPN18), transcript variant 1, mRNA [NM_014369]	1.5652311	0.040091004
A_23_P101237	*LOC401320*	Homo sapiens uncharacterized LOC401320 (LOC401320), long non-coding RNA [NR_038889]	-1.7526724	0.035935212
A_21_P0005657	*C18orf21*	Homo sapiens chromosome 18 open reading frame 21 (C18orf21), transcript variant 1, mRNA [NM_031446]	-1.028047	0.04041675
A_21_P0003089	*ZNF823*	Homo sapiens zinc finger protein 823 (ZNF823), transcript variant 1, mRNA [NM_001080493]	-1.1082553	0.02453978
A_23_P8754	*PDCD6*	Homo sapiens programmed cell death 6 (PDCD6), transcript variant 6, non-coding RNA [NR_073609]	-0.72571445	0.026440267
A_24_P183264	*AASS*	Homo sapiens aminoadipate-semialdehyde synthase (AASS), mRNA [NM_005763]	-1.2256663	0.038287252
A_33_P3226832	*BTF3*	Homo sapiens basic transcription factor 3 (BTF3), transcript variant 1, mRNA [NM_001037637]	-1.0174546	0.027751032
A_23_P88703	*F3*	Homo sapiens coagulation factor III (thromboplastin, tissue factor) (F3), transcript variant 1, mRNA [NM_001993]	-0.9098656	0.034698997
A_33_P3326285	*TP53BP1*	Homo sapiens tumor protein p53 binding protein 1 (TP53BP1), transcript variant 3, mRNA [NM_005657]	-1.8411546	0.030915326
A_21_P0000315	*GAS5*	Homo sapiens growth arrest-specific 5 (non-protein coding) (GAS5), long non-coding RNA [NR_002578]	-0.7328393	0.03403978
A_21_P0007762	*SNORA18*	Homo sapiens small nucleolar RNA, H/ACA box 18 (SNORA18), small nucleolar RNA [NR_002959]	-1.1126621	0.038287252
A_24_P59667	*MAPKAPK5-AS1*	Homo sapiens MAPKAPK5 antisense RNA 1 (MAPKAPK5-AS1), long non-coding RNA [NR_015404]	-0.7657392	0.028192867

A_33_P3255409	*JAK3*	Homo sapiens Janus kinase 3 (JAK3), mRNA [NM_000215]	-1.3714497	0.034404512
A_23_P117546	*PIH1D1*	Homo sapiens PIH1 domain containing 1 (PIH1D1), mRNA [NM_017916]	-1.3741145	0.03947304
A_22_P00013416	*SOS2*	Homo sapiens son of sevenless homolog 2 (Drosophila) (SOS2), mRNA [NM_006939]	-0.70834917	0.030915326
A_21_P0014134	*lnc-XRCC2-4*	LNCipedia lincRNA (lnc-XRCC2-4), lincRNA [lnc-XRCC2-4:1]	-1.8516214	0.024948223
A_33_P3409154	*EVC*	PREDICTED: Homo sapiens Ellis van Creveld syndrome (EVC), transcript variant X11, misc_RNA [XR_427477]	-1.039521	0.044286337
A_23_P406135	*SLC22A23*	Homo sapiens solute carrier family 22, member 23 (SLC22A23), transcript variant 1, mRNA [NM_015482]	1.411799	0.048760455
A_23_P26928	*IFT172*	Homo sapiens intraflagellar transport 172 (IFT172), mRNA [NM_015662]	0.838264	0.03974276
A_23_P385206	*CCDC103*	family with sequence similarity 187, member A [Source:HGNC Symbol;Acc:HGNC:35153] [ENST00000331733]	-1.2709286	0.047170673
A_23_P50897	*STX12*	Homo sapiens syntaxin 12 (STX12), mRNA [NM_177424]	-2.1274796	0.03297842
A_23_P435697	*NIFK*	Homo sapiens nucleolar protein interacting with the FHA domain of MKI67 (NIFK), mRNA [NM_032390]	-0.98164463	0.030971156
A_24_P916496	*EDRF1*	Homo sapiens erythroid differentiation regulatory factor 1 (EDRF1), transcript variant 2, mRNA [NM_015608]	-1.1375756	0.048690848
A_23_P502808	*PRKCA*	Homo sapiens protein kinase C, alpha (PRKCA), mRNA [NM_002737]	-0.95042586	0.042850092
A_33_P3292560	*PRIMA1*	Homo sapiens proline rich membrane anchor 1 (PRIMA1), mRNA [NM_178013]	-1.0867991	0.031430684
A_33_P3300757	*SURF1*	Homo sapiens surfeit 1 (SURF1), transcript variant 1, mRNA [NM_003172]	-0.82059383	0.0442831
A_22_P00011520	*DOPEY1*	Homo sapiens dopey family member 1 (DOPEY1), transcript variant 2, mRNA [NM_001199942]	-0.4341879	0.033583984
A_33_P3221293	*lnc-PAXIP1-1*	LNCipedia lincRNA (lnc-PAXIP1-1), lincRNA [lnc-PAXIP1-1:1]	1.5418966	0.02724032
A_33_P3231432	*DFNA5*	Homo sapiens deafness, autosomal dominant 5 (DFNA5), transcript variant 1, mRNA [NM_004403]	-0.81574863	0.028573772
A_24_P383999	*LDHB*	Homo sapiens lactate dehydrogenase B (LDHB), transcript variant 2, mRNA [NM_001174097]	-0.6507151	0.04639083

A_23_P304450	*RPS3A*	Homo sapiens ribosomal protein S3A (RPS3A), transcript variant 1, mRNA [NM_001006]	1.3163409	0.04555549
A_23_P140029	*GATA6*	Homo sapiens GATA binding protein 6 (GATA6), mRNA [NM_005257]	-1.6618682	0.02737376
A_21_P0000176	*UBL3*	Homo sapiens ubiquitin-like 3 (UBL3), mRNA [NM_007106]	-1.5764639	0.038287252
A_23_P109768	*CDH13*	Homo sapiens cadherin 13 (CDH13), transcript variant 5, mRNA [NM_001220491]	-1.6193779	0.03573528
A_33_P3354151	*RNF7*	Homo sapiens ring finger protein 7 (RNF7), transcript variant 1, mRNA [NM_014245]	-5.8032436	0.029808238
A_33_P3260342	*BOLA1*	Homo sapiens bolA family member 1 (BOLA1), mRNA [NM_016074]	-1.155851	0.03912857
A_23_P154605	*NFASC*	Homo sapiens neurofascin (NFASC), transcript variant 1, mRNA [NM_001005388]	-3.380847	0.02453978
A_23_P216679	*SULF2*	Homo sapiens sulfatase 2 (SULF2), transcript variant 1, mRNA [NM_018837]	-0.67850256	0.04105627
A_32_P225604	*CDC14B*	Homo sapiens cell division cycle 14B (CDC14B), transcript variant 2, mRNA [NM_033331]	-1.1380625	0.036129165
A_23_P566	*RPL5*	Homo sapiens ribosomal protein L5 (RPL5), mRNA [NM_000969]	-1.3256073	0.03691742
A_23_P42265	*FOXJ3*	Homo sapiens forkhead box J3 (FOXJ3), transcript variant 1, mRNA [NM_014947]	-4.5814686	0.026038945
A_24_P382017	*APOM*	Homo sapiens apolipoprotein M (APOM), transcript variant 1, mRNA [NM_019101]	-0.8515456	0.028192867
A_33_P3213392	*RRN3*	Homo sapiens RRN3 RNA polymerase I transcription factor homolog (S. cerevisiae) (RRN3), transcript variant 1, mRNA [NM_018427]	-0.82951164	0.041275464
A_33_P3315929	*TMEM214*	Homo sapiens transmembrane protein 214 (TMEM214), transcript variant 1, mRNA [NM_017727]	-1.0650585	0.029524941
A_22_P00006199	*PTBP3*	Homo sapiens polypyrimidine tract binding protein 3 (PTBP3), transcript variant 5, mRNA [NM_001244897]	-1.422592	0.040091004
A_33_P3378047	*lnc-FAM40A-1*	Q5T8M4_HUMAN (Q5T8M4) Aristaless-like homeobox 3, partial (13%) [THC2511655]	1.4100506	0.029808238
A_24_P21985	*SESTD1*	Homo sapiens SEC14 and spectrin domains 1 (SESTD1), mRNA [NM_178123]	-0.6164112	0.047982622

A_33_P3699445	*FOXJ2*	Homo sapiens forkhead box J2 (FOXJ2), mRNA [NM_018416]	-0.5195146	0.028573772
A_33_P3417990	*VAMP7*	Homo sapiens vesicle-associated membrane protein 7 (VAMP7), transcript variant 1, mRNA [NM_005638]	-1.2262475	0.037613362
A_33_P3311917	*VRK2*	Homo sapiens vaccinia related kinase 2 (VRK2), transcript variant 8, mRNA [NM_001288837]	-1.1349528	0.042621672
A_23_P77669	*HCV-NS5ATP5*	Q6JHZ7_HUMAN (Q6JHZ7) HCV-NS5ATP5 binding protein 1, partial (29%) [THC2537043]	-1.0185705	0.049413078
A_24_P171182	*ZNF821*	Homo sapiens zinc finger protein 821 (ZNF821), transcript variant 4, mRNA [NM_017530]	-1.585288	0.03274125
A_33_P3364240	*ACBD3*	Homo sapiens acyl-CoA binding domain containing 3 (ACBD3), mRNA [NM_022735]	-0.72920996	0.026440267
A_24_P89872	*PAEP*	Homo sapiens progestagen-associated endometrial protein (PAEP), transcript variant 2, mRNA [NM_002571]	-1.3985021	0.041272104
A_33_P3384562	*ZNF189*	Homo sapiens zinc finger protein 189 (ZNF189), transcript variant 2, mRNA [NM_197977]	-0.92496085	0.03808641
A_23_P207367	*PDLIM7*	Homo sapiens PDZ and LIM domain 7 (enigma) (PDLIM7), transcript variant 4, mRNA [NM_213636]	-1.3083847	0.034614928
A_23_P365226	*STAT5A*	Homo sapiens signal transducer and activator of transcription 5A (STAT5A), transcript variant 2, mRNA [NM_003152]	0.88672996	0.04653647
A_23_P302005	*FAM161B*	Homo sapiens family with sequence similarity 161, member B (FAM161B), mRNA [NM_152445]	-1.0156341	0.029808238
A_23_P139500	*STON1*	Homo sapiens stonin 1 (STON1), transcript variant 2, mRNA [NM_006873]	-0.83619833	0.028573772
A_23_P157580	*BHLHE41*	Homo sapiens basic helix-loop-helix family, member e41 (BHLHE41), mRNA [NM_030762]	-1.2388828	0.028573772
A_33_P3315554	*SDCBP*	Homo sapiens syndecan binding protein (syntenin) (SDCBP), transcript variant 1, mRNA [NM_005625]	-0.9473388	0.036667652
A_33_P3402570	*ANKMY1*	Homo sapiens ankyrin repeat and MYND domain containing 1 (ANKMY1), transcript variant 3, mRNA [NM_001282771]	-1.6014348	0.026440267

A_21_P0011314	*CCDC59*	Homo sapiens coiled-coil domain containing 59 (CCDC59), transcript variant 1, mRNA [NM_014167]	-2.3745565	0.03887779
A_23_P79231	*INAFM2*	Homo sapiens InaF-motif containing 2 (INAFM2), mRNA [NM_001301268]	-1.318164	0.03974276
A_23_P143484	*CREB1*	Homo sapiens cAMP responsive element binding protein 1 (CREB1), transcript variant B, mRNA [NM_134442]	-0.6937004	0.044186465
A_23_P101811	*PIGP*	Homo sapiens phosphatidylinositol glycan anchor biosynthesis, class P (PIGP), transcript variant 1, mRNA [NM_153681]	-0.90628767	0.035769645
A_33_P3292179	*ZNF14*	Homo sapiens zinc finger protein 14 (ZNF14), mRNA [NM_021030]	-0.7891104	0.049681563
A_23_P215819	*ABCA9*	Homo sapiens ATP-binding cassette, sub-family A (ABC1), member 9 (ABCA9), mRNA [NM_080283]	-1.0464516	0.034210585
A_23_P65240	*ZNF655*	Homo sapiens zinc finger protein 655 (ZNF655), transcript variant 2, mRNA [NM_024061]	-0.8246155	0.031127423
A_23_P401700	*COL4A1*	Homo sapiens collagen, type IV, alpha 1 (COL4A1), transcript variant 1, mRNA [NM_001845]	-1.4070675	0.036667652
A_33_P3251289	*APBB1IP*	Homo sapiens amyloid beta (A4) precursor protein-binding, family B, member 1 interacting protein (APBB1IP), mRNA [NM_019043]	-0.4412606	0.036667652
A_24_P170763	*VIMP*	Homo sapiens VCP-interacting membrane protein (VIMP), transcript variant 2, mRNA [NM_018445]	1.019537	0.04726856
A_24_P941988	*KHNYN*	Homo sapiens KH and NYN domain containing (KHNYN), transcript variant 1, mRNA [NM_015299]	-4.662704	0.042621672
A_33_P3343106	*SPAST*	Homo sapiens spastin (SPAST), transcript variant 1, mRNA [NM_014946]	-3.5251231	0.027751032
A_23_P62901	*ETV2*	Homo sapiens ets variant 2 (ETV2), transcript variant 1, mRNA [NM_014209]	-1.5297661	0.034210585
A_23_P502678	*BTG2*	Homo sapiens BTG family, member 2 (BTG2), mRNA [NM_006763]	-0.8997884	0.033412892
A_24_P406601	*TM2D2*	Homo sapiens TM2 domain containing 2 (TM2D2), transcript variant 2, mRNA [NM_031940]	-0.6111492	0.04434183
A_33_P3217500	*OLFM1*	Homo sapiens olfactomedin 1 (OLFM1), transcript variant 1, mRNA [NM_014279]	-0.90552294	0.027751032

A_33_P3406828	*MTA3*	Homo sapiens metastasis associated 1 family, member 3 (MTA3), transcript variant 3, mRNA [NM_020744]	-0.83378214	0.03974276
A_33_P3352432	*MAFIP*	Homo sapiens MAFF interacting protein (pseudogene) (MAFIP), transcript variant 1, non-coding RNA [NR_046439]	-0.8501409	0.027751032
A_22_P00023764	*KRBOX4*	Homo sapiens KRAB box domain containing 4 (KRBOX4), transcript variant 3, mRNA [NM_001129899]	-0.99527454	0.034999274
A_21_P0012079	*NEXN-AS1*	Homo sapiens NEXN antisense RNA 1 (NEXN-AS1), long non-coding RNA [NR_103535]	-1.4913758	0.0442831
A_23_P92057	*MIR4435-1HG*	Homo sapiens MIR4435-1 host gene (non-protein coding) (MIR4435-1HG), transcript variant 2, long non-coding RNA [NR_024373]	1.4311038	0.032893
A_33_P3399090	*PIK3CA*	Homo sapiens phosphatidylinositol-4,5-bisphosphate 3-kinase, catalytic subunit alpha (PIK3CA), mRNA [NM_006218]	-0.6268168	0.046265304
A_19_P00321009	*DIXDC1*	Homo sapiens DIX domain containing 1 (DIXDC1), transcript variant 1, mRNA [NM_001037954]	1.5135274	0.04043569
A_33_P3210965	*LINC01133*	Homo sapiens long intergenic non-protein coding RNA 1133 (LINC01133), long non-coding RNA [NR_038849]	-0.2829907	0.042850092
A_23_P84651	*TCTN1*	Homo sapiens tectonic family member 1 (TCTN1), transcript variant 1, mRNA [NM_001082538]	-0.8056766	0.04438234
A_23_P45108	*CNBP*	Homo sapiens CCHC-type zinc finger, nucleic acid binding protein (CNBP), transcript variant 3, mRNA [NM_003418]	-1.8311996	0.036667652
A_33_P3356210	*QRICH1*	Homo sapiens glutamine-rich 1 (QRICH1), transcript variant 1, mRNA [NM_017730]	-2.8900106	0.029979022
A_33_P3388815	*NCR3LG1*	Homo sapiens natural killer cell cytotoxicity receptor 3 ligand 1 (NCR3LG1), mRNA [NM_001202439]	-1.061068	0.04401559
A_24_P118489	*FPGT*	Homo sapiens fucose-1-phosphate guanylyltransferase (FPGT), transcript variant 1, mRNA [NM_003838]	-0.8954594	0.04934116
A_21_P0014791	*WHAMM*	Homo sapiens WAS protein homolog associated with actin, golgi membranes and microtubules (WHAMM), mRNA [NM_001080435]	-1.0071001	0.044364784

A_23_P88404	*5H9 antigen*	Q5J7W6_HUMAN (Q5J7W6) Growth-inhibiting gene 2 protein (5H9 antigen), partial (33%) [THC2507863]	1.3748263	0.028573772
A_24_P330303	*TGFB3*	Homo sapiens transforming growth factor, beta 3 (TGFB3), mRNA [NM_003239]	-1.1803628	0.03840762
A_32_P168247	*FRMD6*	Homo sapiens FERM domain containing 6 (FRMD6), transcript variant 1, mRNA [NM_001042481]	-1.3422453	0.04434183
A_22_P00005910	*COX6A1*	Homo sapiens cytochrome c oxidase subunit VIa polypeptide 1 (COX6A1), mRNA [NM_004373]	1.9558665	0.02737376
A_23_P23141	*LINC01252*	Homo sapiens long intergenic non-protein coding RNA 1252 (LINC01252), long non-coding RNA [NR_033890]	-1.362084	0.029808238
A_24_P111054	*H3F3A*	Homo sapiens H3 histone, family 3A (H3F3A), mRNA [NM_002107]	-2.1291072	0.02598378
A_22_P00013498	*SLC2A5*	solute carrier family 2 (facilitated glucose/fructose transporter), member 5 [Source:HGNC Symbol;Acc:HGNC:11010] [ENST00000377414]	-0.47629875	0.036044724
A_23_P2582	*lnc-RP11-362K2.2.1-1*	LNCipedia lincRNA (lnc-RP11-362K2.2.1-1), lincRNA [lnc-RP11-362K2.2.1-1:1]	1.4060966	0.040911164
A_24_P136182	*HDAC7*	Homo sapiens histone deacetylase 7 (HDAC7), transcript variant 1, mRNA [NM_015401]	-1.0202222	0.028573772
A_21_P0003710	*RPS2P32*	Homo sapiens ribosomal protein S2 pseudogene 32 (RPS2P32), non-coding RNA [NR_026676]	2.5595639	0.028573772
A_33_P3414157	*lnc-FAM53A-1*	LNCipedia lincRNA (lnc-FAM53A-1), lincRNA [lnc-FAM53A-1:2]	1.1239173	0.04726856
A_24_P807031	*MLPH*	Homo sapiens melanophilin (MLPH), transcript variant 1, mRNA [NM_024101]	-1.0108747	0.028573772
A_21_P0013685	*ATP6AP1L*	Homo sapiens ATPase, H+ transporting, lysosomal accessory protein 1-like (ATP6AP1L), mRNA [NM_001017971]	-1.0851605	0.029979022
A_33_P3353996	*SMARCA4*	Homo sapiens SWI/SNF related, matrix associated, actin dependent regulator of chromatin, subfamily a, member 4 (SMARCA4), transcript variant 3, mRNA [NM_003072]	0.960396	0.033546355
A_33_P3354990	*PPP1R3G*	Homo sapiens protein phosphatase 1, regulatory subunit 3G (PPP1R3G), mRNA [NM_001145115]	-11602178	0.043837514

A_23_P169428	*SYNJ2*	synaptojanin 2 [Source:HGNC Symbol;Acc:HGNC:11504] [ENST00000449320]	-11266899	0.044481665
A_22_P00004142	*TRUB2*	Homo sapiens TruB pseudouridine (psi) synthase family member 2 (TRUB2), mRNA [NM_015679]	-0.48741525	0.049986046
A_23_P67980	*lnc-CLDN6-2*	LNCipedia lincRNA (lnc-CLDN6-2), lincRNA [lnc-CLDN6-2:1]	-0.9700098	0.034334898
A_33_P3254634	*KLF7*	Homo sapiens Kruppel-like factor 7 (ubiquitous) (KLF7), transcript variant 1, mRNA [NM_003709]	0.8533906	0.034999274
A_21_P0003882	*PDIA5*	Homo sapiens protein disulfide isomerase family A, member 5 (PDIA5), transcript variant 1, mRNA [NM_006810]	-1.1199961	0.041684493
A_23_P206454	*lnc-CTBP1-1*	LNCipedia lincRNA (lnc-CTBP1-1), lincRNA [lnc-CTBP1-1:1]	3.9643092	0.036131933
A_33_P3209522	*CENPBD1*	Homo sapiens CENPB DNA-binding domains containing 1 (CENPBD1), mRNA [NM_145039]	-1.8575921	0.02695598
A_33_P3214199	*SLFN5*	Homo sapiens schlafen family member 5 (SLFN5), mRNA [NM_144975]	-1.6273656	0.033091284
A_21_P0003889	*ZNF532*	Homo sapiens zinc finger protein 532 (ZNF532), mRNA [NM_018181]	3.4354708	0.027942823
A_24_P395814	*FLJ36777*	uncharacterized LOC730971 [Source:EntrezGene;Acc:730971] [ENST00000504402]	-0.61699635	0.031603426
A_33_P6468355	*CGB*	Homo sapiens chorionic gonadotropin, beta polypeptide (CGB), mRNA [NM_000737]	-1.2859935	0.031377234
A_33_P3355071	*TP73-AS1*	Homo sapiens TP73 antisense RNA 1 (TP73-AS1), transcript variant 1, long non-coding RNA [NR_033711]	-1.1259352	0.044839047
A_23_P17955	*MLLT10*	Homo sapiens myeloid/lymphoid or mixed-lineage leukemia (trithorax homolog, Drosophila); translocated to, 10 (MLLT10), transcript variant 1, mRNA [NM_004641]	1.9868469	0.048220593
A_21_P0013236	*FBXL2*	Homo sapiens F-box and leucine-rich repeat protein 2 (FBXL2), transcript variant 1, mRNA [NM_012157]	-2.2000818	0.042440254
A_24_P362646	*GTF2I*	Homo sapiens general transcription factor IIi (GTF2I), transcript variant 1, mRNA [NM_032999]	-0.69417673	0.028583162
A_23_P165984	*TXNDC9*	Homo sapiens thioredoxin domain containing 9 (TXNDC9), mRNA [NM_005783]	-0.8721893	0.04390963

A_33_P3412149	*ZSWIM3*	Homo sapiens zinc finger, SWIM-type containing 3 (ZSWIM3), transcript variant 1, mRNA [NM_080752]	-1.9027332	0.048182435
A_33_P3318465	*CLCC1*	Homo sapiens chloride channel CLIC-like 1 (CLCC1), transcript variant 1, mRNA [NM_001048210]	-0.21392192	0.040692493
A_21_P0000339	*SUMO1*	Homo sapiens small ubiquitin-like modifier 1 (SUMO1), transcript variant 1, mRNA [NM_003352]	-1.2499297	0.031303514
A_24_P914513	*SNORA58*	Homo sapiens small nucleolar RNA, H/ACA box 58 (SNORA58), small nucleolar RNA [NR_002985]	-0.82284796	0.030293303
A_24_P292470	*BCKDHB*	Homo sapiens branched chain keto acid dehydrogenase E1, beta polypeptide (BCKDHB), transcript variant 1, mRNA [NM_183050]	-1.1620893	0.036131933
A_21_P0005109	*UCP3*	Homo sapiens uncoupling protein 3 (mitochondrial, proton carrier) (UCP3), transcript variant short, mRNA [NM_022803]	-0.9128131	0.049724605
A_24_P71468	*lnc-CD83-2*	LNCipedia lincRNA (lnc-CD83-2), lincRNA [lnc-CD83-2:2]	-1.0056858	0.03716006
A_33_P3215023	*QPCT*	Homo sapiens glutaminyl-peptide cyclotransferase (QPCT), mRNA [NM_012413]	-1.662809	0.039863296
A_33_P3352664	*C6orf89*	Homo sapiens chromosome 6 open reading frame 89 (C6orf89), transcript variant 1, mRNA [NM_152734]	1.3407452	0.029857725
A_23_P259292	*SP7*	Homo sapiens Sp7 transcription factor (SP7), transcript variant 3, mRNA [NM_001300837]	2.2868176	0.045400333
A_19_P00320440	*C1QTNF5*	Homo sapiens C1q and tumor necrosis factor related protein 5 (C1QTNF5), transcript variant 1, mRNA [NM_015645]	-1.6867766	0.024948223
A_21_P0011623	*LINC00662*	long intergenic non-protein coding RNA 662 [Source:HGNC Symbol;Acc:HGNC:27122] [ENST00000586248]	-0.9920573	0.04501488
A_23_P161183	*lnc-LCLAT1-2*	LNCipedia lincRNA (lnc-LCLAT1-2), lincRNA [lnc-LCLAT1-2:3]	-1.5627254	0.02598378
A_21_P0008758	*ZDHHC6*	Homo sapiens zinc finger, DHHC-type containing 6 (ZDHHC6), transcript variant 1, mRNA [NM_022494]	-1.3942542	0.042269725
A_33_P3771039	*lnc-MAN2C1-3*	LNCipedia lincRNA (lnc-MAN2C1-3), lincRNA [lnc-MAN2C1-3:1]	1.5957952	0.02453978

A_23_P168443	*TM9SF2*	Homo sapiens transmembrane 9 superfamily member 2 (TM9SF2), mRNA [NM_004800]	-0.91939235	0.029808238
A_24_P17722	*EPHB4*	Homo sapiens EPH receptor B4 (EPHB4), mRNA [NM_004444]	2.2954607	0.03403978
A_33_P3286046	*TBC1D9B*	Homo sapiens TBC1 domain family, member 9B (with GRAM domain) (TBC1D9B), transcript variant 1, mRNA [NM_198868]	-1.035157	0.029808238
A_23_P88865	*DPCD*	Homo sapiens deleted in primary ciliary dyskinesia homolog (mouse) (DPCD), mRNA [NM_015448]	-0.9305699	0.035186797
A_33_P3265704	*CMTM3*	Homo sapiens CKLF-like MARVEL transmembrane domain containing 3 (CMTM3), transcript variant 1, mRNA [NM_144601]	-1.E22781414117	0.036726505
A_33_P3333317	*UFD1L*	Homo sapiens ubiquitin fusion degradation 1 like (yeast) (UFD1L), transcript variant 1, mRNA [NM_005659]	-0.43785262	0.027751032
A_23_P360209	*OPTN*	Homo sapiens optineurin (OPTN), transcript variant 1, mRNA [NM_001008211]	-1.1639544	0.03233256
A_23_P151614	*ND3*	mitochondrially encoded NADH dehydrogenase 3 [Source:HGNC Symbol;Acc:HGNC:7458] [ENST00000361227]	-0.7457735	0.031303514
A_33_P3400547	*PSME1*	Homo sapiens proteasome (prosome, macropain) activator subunit 1 (PA28 alpha) (PSME1), transcript variant 1, mRNA [NM_006263]	-0.83371776	0.029019035
A_33_P3395403	*MED21*	Homo sapiens mediator complex subunit 21 (MED21), transcript variant 1, mRNA [NM_004264]	-0.5947213	0.04020063
A_33_P3387050	*LOC100996724*	PREDICTED: Homo sapiens myomegalin-like (LOC100996724), transcript variant X8, mRNA [XM_006710081]	1.751964	0.036667652
A_23_P87964	*C8orf82*	Homo sapiens chromosome 8 open reading frame 82 (C8orf82), mRNA [NM_001001795]	-0.5435805	0.04555549
A_32_P10272	*ESD*	Homo sapiens esterase D (ESD), mRNA [NM_001984]	-0.8066887	0.04277177
A_33_P3424272	*PMS2P5*	Homo sapiens postmeiotic segregation increased 2 pseudogene 5 (PMS2P5), transcript variant 1, non-coding RNA [NR_027775]	-0.9267452	0.041527454

A_23_P57667	*NAA60*	Homo sapiens N(alpha)-acetyltransferase 60, NatF catalytic subunit (NAA60), transcript variant 1, mRNA [NM_001083601]	-0.68828607	0.02737376
A_23_P115703	*PLXNA1*	Homo sapiens plexin A1 (PLXNA1), mRNA [NM_032242]	-1.4019278	0.03032242
A_23_P5601	*PCGF6*	Homo sapiens polycomb group ring finger 6 (PCGF6), transcript variant 1, mRNA [NM_001011663]	-0.5900264	0.030293303
A_23_P86195	*DOK1*	Homo sapiens docking protein 1, 62kDa (downstream of tyrosine kinase 1) (DOK1), transcript variant 1, mRNA [NM_001381]	-0.7128482	0.035244066
A_33_P3419938	*SLC44A3*	Homo sapiens solute carrier family 44, member 3 (SLC44A3), transcript variant 2, mRNA [NM_152369]	-0.832659	0.032261204
A_33_P3221438	*VHL*	Homo sapiens von Hippel-Lindau tumor suppressor, E3 ubiquitin protein ligase (VHL), transcript variant 1, mRNA [NM_000551]	-0.9535896	0.04217344
A_22_P00025917	*XXYLT1*	Homo sapiens xyloside xylosyltransferase 1 (XXYLT1), mRNA [NM_152531]	-0.9170353	0.04447692
A_23_P79155	*LOC100506603*	Homo sapiens uncharacterized LOC100506603 (LOC100506603), transcript variant 1, long non-coding RNA [NR_104183]	-2184292	0.048182435
A_24_P79808	*GPR39*	Homo sapiens G protein-coupled receptor 39 (GPR39), mRNA [NM_001508]	-0.8842976	0.031526815
A_23_P18276	*PBXIP1*	Homo sapiens pre-B-cell leukemia homeobox interacting protein 1 (PBXIP1), mRNA [NM_020524]	-0.51841205	0.034210585
A_33_P3243649	*RBM5*	Homo sapiens RNA binding motif protein 5 (RBM5), transcript variant 1, mRNA [NM_005778]	-1.6309304	0.03436134
A_33_P3285038	*LOC100131831*	Homo sapiens cDNA FLJ26174 fis, clone ADG03920. [AK129685]	1.3948197	0.048182435
A_23_P38365	*CERS5*	Homo sapiens ceramide synthase 5 (CERS5), transcript variant 2, mRNA [NM_001281731]	-0.79995805	0.036667652
A_33_P3329597	*TLK2*	Homo sapiens tousled-like kinase 2 (TLK2), transcript variant A, mRNA [NM_006852]	-0.5741535	0.035769645
A_21_P0012925	*BLOC1S3*	Homo sapiens biogenesis of lysosomal organelles complex-1, subunit 3 (BLOC1S3), mRNA [NM_212550]	1.5812652	0.046325136

A_23_P85053	*XLOC_l2_012210*	BROAD Institute lincRNA (XLOC_l2_012210), lincRNA [TCONS_l2_00023087]	-0.46574262	0.04281463
A_24_P184305	*ZRSR2*	Homo sapiens zinc finger (CCCH type), RNA-binding motif and serine/arginine rich 2 (ZRSR2), mRNA [NM_005089]	-1.2231337	0.04250221
A_24_P191312	*BBS1*	Homo sapiens Bardet-Biedl syndrome 1 (BBS1), mRNA [NM_024649]	-0.60519475	0.027189178
A_33_P3297277	*SLC1A4*	Homo sapiens solute carrier family 1 (glutamate/neutral amino acid transporter), member 4 (SLC1A4), transcript variant 1, mRNA [NM_003038]	-0.80679256	0.046325136
A_21_P0001790	*LTA4H*	Homo sapiens leukotriene A4 hydrolase (LTA4H), transcript variant 3, mRNA [NM_001256644]	-1.2465223	0.04591278
A_23_P133075	*LOC101927438*	Homo sapiens uncharacterized LOC101927438 (LOC101927438), long non-coding RNA [NR_110224]	-3.8819642	0.041275464
A_23_P332399	*TBCK*	Homo sapiens TBC1 domain containing kinase (TBCK), transcript variant 4, mRNA [NM_033115]	-0.97540665	0.029150521
A_23_P160318	*GULP1*	Homo sapiens GULP, engulfment adaptor PTB domain containing 1 (GULP1), transcript variant 1, mRNA [NM_016315]	-0.69661856	0.036667652
A_33_P3264424	*COL16A1*	Homo sapiens collagen, type XVI, alpha 1 (COL16A1), mRNA [NM_001856]	1.0593367	0.03590667
A_24_P234792	*MFAP3L*	Homo sapiens microfibrillar-associated protein 3-like (MFAP3L), transcript variant 1, mRNA [NM_021647]	1.7451346	0.044770036
A_33_P3370226	*CSNK1G3*	Homo sapiens casein kinase 1, gamma 3 (CSNK1G3), transcript variant 4, mRNA [NM_001044723]	-0.948977	0.04079467
A_24_P879740	*RPL7*	Homo sapiens ribosomal protein L7 (RPL7), mRNA [NM_000971]	-0.9519628	0.031526815
A_23_P121875	*MAP1B*	Homo sapiens microtubule-associated protein 1B (MAP1B), mRNA [NM_005909]	-2.9806852	0.036841016
A_23_P316460	*C5orf28*	Homo sapiens chromosome 5 open reading frame 28 (C5orf28), mRNA [NM_022483]	-0.7826829	0.03319862
A_33_P3340342	*ZBED6CL*	Homo sapiens ZBED6 C-terminal like (ZBED6CL), mRNA [NM_138434]	-1.0134747	0.03560228
A_32_P113114	*CMTM3*	Homo sapiens CKLF-like MARVEL transmembrane domain containing 3 (CMTM3), transcript variant 1, mRNA [NM_144601]	-0.67092675	0.03900215

A_23_P30050	*ZNF561*	Homo sapiens zinc finger protein 561 (ZNF561), mRNA [NM_152289]	-0.9757428	0.036290724
A_24_P323545	*SLC30A9*	Homo sapiens solute carrier family 30 (zinc transporter), member 9 (SLC30A9), mRNA [NM_006345]	1.788779	0.047708835
A_22_P00002137	*MYH14*	Homo sapiens myosin, heavy chain 14, non-muscle (MYH14), transcript variant 1, mRNA [NM_001077186]	-1.4023588	0.042034253
A_33_P3382423	*lnc-BPHL-1*	LNCipedia lincRNA (lnc-BPHL-1), lincRNA [lnc-BPHL-1:1]	-1.0956194	0.044286337
A_23_P43946	*ZNF428*	Homo sapiens zinc finger protein 428 (ZNF428), mRNA [NM_182498]	-0.7218165	0.044444222
A_23_P28318	*SARNP*	Homo sapiens SAP domain containing ribonucleoprotein (SARNP), transcript variant 1, mRNA [NM_033082]	0.62854123	0.02474944
A_33_P3375646	*NDUFAF7*	Homo sapiens NADH dehydrogenase (ubiquinone) complex I, assembly factor 7 (NDUFAF7), transcript variant 1, mRNA [NM_144736]	0.70783764	0.042656552
A_33_P3271196	*GTPBP6*	Homo sapiens cDNA FLJ56662 complete cds, highly similar to Homo sapiens GTP binding protein 6 (GTPBP6), mRNA. [AK296003]	-0.4813677	0.041831266
A_23_P400066	*AMOTL1*	Homo sapiens angiomotin like 1 (AMOTL1), transcript variant 1, mRNA [NM_130847]	-0.85367703	0.044364784
A_23_P105794	*RALGAPB*	Homo sapiens Ral GTPase activating protein, beta subunit (non-catalytic) (RALGAPB), transcript variant 1, mRNA [NM_020336]	-0.7134524	0.03808641
A_23_P55998	*EPSTI1*	Homo sapiens epithelial stromal interaction 1 (breast) (EPSTI1), transcript variant 2, mRNA [NM_033255]	1.7984608	0.043670435
A_24_P303589	*SLC1A5*	Homo sapiens solute carrier family 1 (neutral amino acid transporter), member 5 (SLC1A5), transcript variant 1, mRNA [NM_005628]	0.87911314	0.042693015
A_23_P59630	*CUL1*	Homo sapiens cullin 1 (CUL1), mRNA [NM_003592]	-0.91381145	0.028573772
A_23_P147109	*PMS2P3*	postmeiotic segregation increased 2 pseudogene 3 [Source:HGNC Symbol;Acc:HGNC:9128] [ENST00000437568]	-2.2195024	0.04639083
A_33_P3317548	*TOR4A*	Homo sapiens torsin family 4, member A (TOR4A), mRNA [NM_017723]	-1.8646594	0.043374248

A_24_P268786	*LINC01478*	Homo sapiens long intergenic non-protein coding RNA 1478 (LINC01478), long non-coding RNA [NR_110792]	-0.75378984	0.030293303
A_24_P942730	*MYNN*	Homo sapiens myoneurin (MYNN), transcript variant 1, mRNA [NM_018657]	1.7455846	0.03668808
A_23_P70688	*ZSCAN29*	Homo sapiens zinc finger and SCAN domain containing 29 (ZSCAN29), mRNA [NM_152455]	0.8478765	0.03129854
A_33_P3244803	*LY86*	Homo sapiens lymphocyte antigen 86 (LY86), mRNA [NM_004271]	2.1889815	0.030896025
A_23_P301372	*ACOX1*	Homo sapiens acyl-CoA oxidase 1, palmitoyl (ACOX1), transcript variant 3, mRNA [NM_001185039]	-1.0232303	0.029088594
A_33_P3330443	*TAPT1*	Homo sapiens transmembrane anterior posterior transformation 1 (TAPT1), mRNA [NM_153365]	-1.2761942	0.024948223
A_23_P130304	*FAM110B*	Homo sapiens family with sequence similarity 110, member B (FAM110B), mRNA [NM_147189]	1.1851829	0.036339592
A_23_P326009	*TXNL4A*	thioredoxin-like 4A [Source:HGNC Symbol;Acc:HGNC:30551] [ENST00000585769]	-0.78656864	0.03297842
A_19_P00321734	*ZNF471*	Homo sapiens zinc finger protein 471 (ZNF471), mRNA [NM_020813]	-0.9896753	0.033726536
A_23_P210829	*LINC00881*	long intergenic non-protein coding RNA 881 [Source:HGNC Symbol;Acc:HGNC:48567] [ENST00000474477]	-1.8803407	0.032983456
A_22_P00011719	*PCMTD2*	Homo sapiens protein-L-isoaspartate (D-aspartate) O-methyltransferase domain containing 2 (PCMTD2), transcript variant 1, mRNA [NM_018257]	-0.41066912	0.048398294
A_33_P3354322	*lnc-PEA15-1*	Q9Z0G8_RAT (Q9Z0G8) SH3 domain binding protein (CR16), partial (4%) [THC2569838]	-0.8807465	0.041496843
A_33_P6820772	*GPX1*	Homo sapiens glutathione peroxidase 1 (GPX1), transcript variant 2, mRNA [NM_201397]	1.9662677	0.03601631
A_33_P3324383	*PDCD4-AS1*	Homo sapiens PDCD4 antisense RNA 1 (PDCD4-AS1), long non-coding RNA [NR_026932]	-0.96343243	0.045952834
A_23_P258251	*FIGNL2*	Homo sapiens fidgetin-like 2 (FIGNL2), mRNA [NM_001013690]	1.3169448	0.034750238
A_24_P88565	*ENOX2*	Homo sapiens ecto-NOX disulfide-thiol exchanger 2 (ENOX2), transcript variant 2, mRNA [NM_182314]	-1.1185665	0.03431529

A_24_P237757	*SLC50A1*	Homo sapiens solute carrier family 50 (sugar efflux transporter), member 1 (SLC50A1), transcript variant 1, mRNA [NM_018845]	1.3389504	0.034210585
A_33_P3253807	*ZMYM2*	zinc finger, MYM-type 2 [Source:HGNC Symbol;Acc:HGNC:12989] [ENST00000382881]	-2.182809	0.047233704
A_23_P93988	*CEBPG*	Homo sapiens CCAAT/enhancer binding protein (C/EBP), gamma (CEBPG), transcript variant 1, mRNA [NM_001806]	-0.9949248	0.04591315
A_22_P00009028	*ARHGEF5*	Homo sapiens Rho guanine nucleotide exchange factor (GEF) 5 (ARHGEF5), mRNA [NM_005435]	-0.9395367	0.047375914
A_23_P146908	*CCDC183-AS1*	Homo sapiens CCDC183 antisense RNA 1 (CCDC183-AS1), long non-coding RNA [NR_024580]	-0.6817179	0.038322687
A_24_P356130	*STX8*	Homo sapiens syntaxin 8 (STX8), transcript variant 1, mRNA [NM_004853]	-0.81456494	0.03136619
A_33_P3269723	*MAP2K5*	Homo sapiens mitogen-activated protein kinase kinase 5 (MAP2K5), transcript variant 2, mRNA [NM_002757]	-1.5016122	0.027751032
A_24_P68819	*ZSWIM7*	Homo sapiens zinc finger, SWIM-type containing 7 (ZSWIM7), transcript variant 1, mRNA [NM_001042697]	1.173993	0.04792962
A_23_P343237	*PPP1R21*	Homo sapiens protein phosphatase 1, regulatory subunit 21 (PPP1R21), transcript variant 2, mRNA [NM_152994]	-0.9486058	0.027751032
A_23_P218841	*ANGEL2*	Homo sapiens angel homolog 2 (Drosophila) (ANGEL2), transcript variant 1, mRNA [NM_144567]	-1.2729398	0.037395623
A_24_P216765	*NGLY1*	Homo sapiens N-glycanase 1 (NGLY1), transcript variant 1, mRNA [NM_018297]	-0.7277279	0.031493735
A_23_P205031	*TOMM20*	Homo sapiens translocase of outer mitochondrial membrane 20 homolog (yeast) (TOMM20), mRNA [NM_014765]	-1.2593116	0.036667652
A_33_P3259183	*COL4A2*	Homo sapiens collagen, type IV, alpha 2 (COL4A2), mRNA [NM_001846]	-0.7320355	0.03623899
A_33_P3259522	*FAM78B*	Homo sapiens family with sequence similarity 78, member B (FAM78B), mRNA [NM_001017961]	-0.6928997	0.04015477

A_23_P143016	*CDCP2*	Homo sapiens CUB domain containing protein 2 (CDCP2), mRNA [NM_201546]	-0.68347996	0.02453978
A_33_P3270509	*ARID5A*	Homo sapiens AT rich interactive domain 5A (MRF1-like) (ARID5A), mRNA [NM_212481]	-3.0585132	0.033726536
A_33_P3235568	*SIAH1*	Homo sapiens siah E3 ubiquitin protein ligase 1 (SIAH1), transcript variant 1, mRNA [NM_003031]	-1.0549738	0.03515347
A_22_P00010118	*CAPZB*	Homo sapiens capping protein (actin filament) muscle Z-line, beta (CAPZB), transcript variant 3, mRNA [NM_001206541]	1.8070818	0.03436134
A_23_P84334	*ZNF658*	Homo sapiens zinc finger protein 658 (ZNF658), mRNA [NM_033160]	-1.0278376	0.046256438
A_33_P3216277	*TMEM39A*	Homo sapiens transmembrane protein 39A (TMEM39A), transcript variant 1, mRNA [NM_018266]	-0.624768	0.040911164
A_24_P935881	*LOC101059976*	PREDICTED: Homo sapiens arf-GAP with GTPase, ANK repeat and PH domain-containing protein 2-like (LOC101059976), mRNA [XM_003959913]	-0.6318814	0.04591315
A_23_P51231	*SERF1B*	Homo sapiens small EDRK-rich factor 1B (centromeric) (SERF1B), transcript variant 2, mRNA [NM_022978]	-0.835442	0.032580614
A_23_P100420	*RUNX3*	Homo sapiens runt-related transcription factor 3 (RUNX3), transcript variant 1, mRNA [NM_001031680]	-1.2477047	0.032155342
A_23_P16915	*ZCCHC14*	Homo sapiens zinc finger, CCHC domain containing 14 (ZCCHC14), mRNA [NM_015144]	0.609014	0.038322687
A_24_P414376	*QPCT*	Homo sapiens glutaminyl-peptide cyclotransferase (QPCT), mRNA [NM_012413]	-0.79188156	0.032914247
A_33_P3396120	*KLF3*	Homo sapiens Kruppel-like factor 3 (basic) (KLF3), mRNA [NM_016531]	-3.0717576	0.044286337
A_23_P98532	*ZNF594*	Homo sapiens zinc finger protein 594 (ZNF594), mRNA [NM_032530]	-1.1275334	0.032286324
A_24_P311771	*ZDHHC5*	Homo sapiens zinc finger, DHHC-type containing 5 (ZDHHC5), mRNA [NM_015457]	-1.7475247	0.026098706
A_33_P3759592	*ZFR*	Homo sapiens zinc finger RNA binding protein (ZFR), mRNA [NM_016107]	-1.5964732	0.033772174
A_33_P3392525	*FLJ13773*	Homo sapiens cDNA FLJ13773 fis, clone PLACE4000323. [AK023835]	-0.6365829	0.039287783

A_24_P129277	*ARL4D*	Homo sapiens ADP-ribosylation factor-like 4D (ARL4D), mRNA [NM_001661]	-1.1261268	0.034210585
A_23_P78099	*NOD1*	Homo sapiens nucleotide-binding oligomerization domain containing 1 (NOD1), mRNA [NM_006092]	-0.9957049	0.02737376
A_19_P00316219	*VTN*	Homo sapiens vitronectin (VTN), mRNA [NM_000638]	-0.8698044	0.034037594
A_24_P417162	*DAPK2*	Homo sapiens death-associated protein kinase 2 (DAPK2), mRNA [NM_014326]	-0.9142035	0.038309526
A_33_P3221303	*TBL1X*	Homo sapiens transducin (beta)-like 1X-linked (TBL1X), transcript variant 1, mRNA [NM_005647]	1.137438	0.048986726
A_33_P3407925	*CCR10*	Homo sapiens chemokine (C-C motif) receptor 10 (CCR10), mRNA [NM_016602]	-1.3339369	0.028192867
A_33_P3246244	*VMP1*	Homo sapiens vacuole membrane protein 1 (VMP1), mRNA [NM_030938]	-2.6973152	0.02737376
A_23_P47879	*ATF4*	Homo sapiens activating transcription factor 4 (ATF4), transcript variant 1, mRNA [NM_001675]	-1.2566558	0.031606767
A_23_P171336	*STAT6*	Homo sapiens signal transducer and activator of transcription 6, interleukin-4 induced (STAT6), transcript variant 2, mRNA [NM_003153]	1.6678079	0.039287783
A_23_P432034	*NXF3*	Homo sapiens nuclear RNA export factor 3 (NXF3), mRNA [NM_022052]	0.7361953	0.046256438
A_23_P34983	*CCDC117*	Homo sapiens coiled-coil domain containing 117 (CCDC117), transcript variant 1, mRNA [NM_173510]	-0.7880931	0.027751032
A_21_P0012571	*JTB*	Homo sapiens jumping translocation breakpoint (JTB), mRNA [NM_006694]	-1.9623834	0.03957154
A_23_P21324	*lnc-LUZP1-1*	LNCipedia lincRNA (lnc-LUZP1-1), lincRNA [lnc-LUZP1-1:1]	-1.1768168	0.028573772
A_23_P82959	*TWIST2*	Homo sapiens twist family bHLH transcription factor 2 (TWIST2), transcript variant 1, mRNA [NM_001271893]	-0.9324298	0.031603426
A_33_P3269806	*FOXH1*	Homo sapiens forkhead box H1 (FOXH1), mRNA [NM_003923]	-1.0968149	0.034210585
A_23_P214091	*ZNF616*	Homo sapiens zinc finger protein 616 (ZNF616), mRNA [NM_178523]	1.3616287	0.030589065
A_24_P111912	*LYPLA1*	Homo sapiens lysophospholipase I (LYPLA1), transcript variant 1, mRNA [NM_006330]	-0.6814842	0.041768707
A_33_P3299066	*FAM172A*	Homo sapiens family with sequence similarity 172, member A (FAM172A),	1.368206	0.047656383

		transcript variant 1, mRNA [NM_032042]		
A_21_P0011306	*NR4A2*	Homo sapiens nuclear receptor subfamily 4, group A, member 2 (NR4A2), mRNA [NM_006186]	-0.870934	0.029808238
A_23_P414913	*SYNGR2*	Homo sapiens synaptogyrin 2 (SYNGR2), mRNA [NM_004710]	-1.1079383	0.03977008
A_33_P3214129	*GLIPR2*	Homo sapiens GLI pathogenesis-related 2 (GLIPR2), transcript variant 1, mRNA [NM_022343]	-1.180447	0.02737376
A_23_P61524	*LOC728061*	Homo sapiens cDNA: FLJ21498 fis, clone COL05627. [AK025151]	2.6474981	0.024948223
A_33_P3479999	*CCDC71*	Homo sapiens coiled-coil domain containing 71 (CCDC71), mRNA [NM_022903]	1.0859337	0.044849187
A_24_P150874	*LOC494150*	Homo sapiens prohibitin pseudogene, mRNA (cDNA clone IMAGE:4547239). [BC014228]	-2.437121	0.02453978
A_23_P18493	*GNA13*	Homo sapiens guanine nucleotide binding protein (G protein), alpha 13 (GNA13), transcript variant 1, mRNA [NM_006572]	-2.2640307	0.03136619
A_23_P40847	*PTPN13*	Homo sapiens protein tyrosine phosphatase, non-receptor type 13 (APO-1/CD95 (Fas)-associated phosphatase) (PTPN13), transcript variant 4, mRNA [NM_080685]	-1.9501894	0.04009655
A_33_P3329592	*CHST2*	Homo sapiens carbohydrate (N-acetylglucosamine-6-O) sulfotransferase 2 (CHST2), mRNA [NM_004267]	-0.9314132	0.030385435
A_21_P0006803	*BLOC1S3*	Homo sapiens biogenesis of lysosomal organelles complex-1, subunit 3 (BLOC1S3), mRNA [NM_212550]	-1.3220778	0.03510091
A_24_P769359	*lnc-EBF3-3*	LNCipedia lincRNA (lnc-EBF3-3), lincRNA [lnc-EBF3-3:1]	-1.2671633	0.027942823
A_23_P139648	*WNK1*	Homo sapiens WNK lysine deficient protein kinase 1 (WNK1), transcript variant 4, mRNA [NM_001184985]	-1.8479913	0.029808238
A_23_P337934	*IAPP*	Homo sapiens islet amyloid polypeptide (IAPP), mRNA [NM_000415]	0.7409446	0.04690382
A_24_P823684	*FBLIM1*	Homo sapiens filamin binding LIM protein 1 (FBLIM1), transcript variant 1, mRNA [NM_017556]	-0.82480705	0.036542445
A_33_P3217834	*HSP90AB1*	Homo sapiens heat shock protein 90kDa alpha (cytosolic), class B	-0.8136928	0.026187742

		member 1 (HSP90AB1), transcript variant 2, mRNA [NM_007355]		
A_33_P3233170	*SMARCAL1*	Homo sapiens SWI/SNF related, matrix associated, actin dependent regulator of chromatin, subfamily a-like 1 (SMARCAL1), transcript variant 1, mRNA [NM_014140]	0.65770864	0.03668808
A_23_P164451	*ISG15*	Homo sapiens ISG15 ubiquitin-like modifier (ISG15), mRNA [NM_005101]	-0.6796844	0.04531519
A_21_P0013931	*TBX2*	Homo sapiens T-box 2 (TBX2), mRNA [NM_005994]	-1.334394	0.028573772
A_24_P416997	*MRS2*	Homo sapiens MRS2 magnesium transporter (MRS2), transcript variant 3, mRNA [NM_001286265]	-1.0805757	0.04250221
A_24_P1919	*APOL3*	Homo sapiens apolipoprotein L, 3 (APOL3), transcript variant beta/a, mRNA [NM_145641]	3.1284604	0.034342222
A_33_P3243153	*NF1*	Homo sapiens neurofibromin 1 (NF1), transcript variant 2, mRNA [NM_000267]	1.523535	0.02737376
A_23_P39602	*GFPT1*	Homo sapiens glutamine--fructose-6-phosphate transaminase 1 (GFPT1), transcript variant 1, mRNA [NM_001244710]	-1.369706	0.042850092
A_24_P82880	*NCOA1*	Homo sapiens nuclear receptor coactivator 1 (NCOA1), transcript variant 3, mRNA [NM_147233]	-1.6528428	0.049976278
A_33_P3326432	*TPM4*	Homo sapiens tropomyosin 4 (TPM4), transcript variant Tpm4.2, mRNA [NM_003290]	-0.5815386	0.036129165
A_24_P21447	*SEPW1*	Homo sapiens selenoprotein W, 1 (SEPW1), mRNA [NM_003009]	-0.53483003	0.042656552
A_23_P156025	*SURF6*	Homo sapiens surfeit 6 (SURF6), transcript variant 1, mRNA [NM_006753]	-2.1355119	0.02737376
A_24_P360722	*IRX2*	Homo sapiens iroquois homeobox 2 (IRX2), transcript variant 1, mRNA [NM_033267]	-1.1426706	0.028573772
A_23_P56709	*DIP2C*	Homo sapiens DIP2 disco-interacting protein 2 homolog C (Drosophila) (DIP2C), mRNA [NM_014974]	-1.9092722	0.027942823
A_23_P425516	*RNF103*	Homo sapiens ring finger protein 103 (RNF103), transcript variant 1, mRNA [NM_005667]	-0.7459626	0.047266874
A_33_P3321150	*TSPAN17*	Homo sapiens tetraspanin 17 (TSPAN17), transcript variant 1, mRNA [NM_012171]	-1.3806112	0.032261204

A_33_P3250148	*CUL3*	Homo sapiens cullin 3 (CUL3), transcript variant 1, mRNA [NM_003590]	-1.1755699	0.042693015
A_23_P10591	*SP8*	Homo sapiens Sp8 transcription factor (SP8), transcript variant 2, mRNA [NM_198956]	-1.2740551	0.03510091
A_33_P3241521	*METRNL*	Homo sapiens meteorin, glial cell differentiation regulator-like (METRNL), mRNA [NM_001004431]	-0.23230952	0.0380295
A_22_P00022364	*EBF1*	Homo sapiens early B-cell factor 1 (EBF1), transcript variant 1, mRNA [NM_001290360]	-0.5702564	0.047933746
A_33_P3309471	*LOC100507054*	PREDICTED: Homo sapiens uncharacterized LOC100507054 (LOC100507054), ncRNA [XR_108535]	0.61652887	0.048618473
A_23_P154306	*PTPRQ*	Homo sapiens protein tyrosine phosphatase, receptor type, Q (PTPRQ), mRNA [NM_001145026]	-1.6834166	0.03136619
A_33_P3269598	*TANK*	Homo sapiens TRAF family member-associated NFKB activator (TANK), transcript variant 1, mRNA [NM_004180]	-2.1465828	0.046120346
A_33_P3322192	*ERCC6L2*	Homo sapiens excision repair cross-complementation group 6-like 2 (ERCC6L2), transcript variant 1, mRNA [NM_020207]	-1.873087	0.036379445
A_32_P184727	*STX17*	Homo sapiens syntaxin 17 (STX17), mRNA [NM_017919]	-1.0326533	0.04217344
A_33_P3266165	*KPNB1*	Homo sapiens karyopherin (importin) beta 1 (KPNB1), transcript variant 1, mRNA [NM_002265]	-1.0855156	0.039682448
A_24_P138361	*TNFSF12*	Homo sapiens tumor necrosis factor (ligand) superfamily, member 12 (TNFSF12), transcript variant 1, mRNA [NM_003809]	-0.7008772	0.030002516
A_23_P46182	*EIF3M*	Homo sapiens eukaryotic translation initiation factor 3, subunit M (EIF3M), mRNA [NM_006360]	-0.4124334	0.048295442
A_33_P3270317	*RPS8*	Homo sapiens ribosomal protein S8 (RPS8), mRNA [NM_001012]	-0.7946818	0.02598378
A_33_P3281572	*SLC18A3*	Homo sapiens solute carrier family 18 (vesicular acetylcholine transporter), member 3 (SLC18A3), mRNA [NM_003055]	-0.808015	0.037221838
A_23_P90333	*CMAHP*	Homo sapiens cytidine monophospho-N-acetylneuraminic acid hydroxylase, pseudogene (CMAHP), transcript	-1.2371794	0.035438698

		variant 1, non-coding RNA [NR_002174]		
A_23_P362637	*ZNF404*	Homo sapiens zinc finger protein 404 (ZNF404), mRNA [NM_001033719]	-0.8550453	0.033198114
A_33_P3275381	*UBR2*	Homo sapiens ubiquitin protein ligase E3 component n-recognin 2 (UBR2), transcript variant 1, mRNA [NM_015255]	1.7458882	0.027751032
A_21_P0006566	*MAP9*	Homo sapiens microtubule-associated protein 9 (MAP9), mRNA [NM_001039580]	-2.0967004	0.042564467
A_21_P0005117	*lnc-RAB40AL-1*	BX114424 Soares placenta Nb2HP Homo sapiens cDNA clone IMAGp998L02197, mRNA sequence [BX114424]	-1.1740865	0.024948223
A_22_P00010250	*SUPV3L1*	Homo sapiens suppressor of var1, 3-like 1 (S. cerevisiae) (SUPV3L1), transcript variant 1, mRNA [NM_003171]	-1.4322617	0.042469725
A_24_P358164	*MIR100HG*	Homo sapiens mir-100-let-7a-2 cluster host gene (non-protein coding) (MIR100HG), long non-coding RNA [NR_024430]	0.7556763	0.041277196
A_33_P3265290	*RPSAP9*	Homo sapiens ribosomal protein SA pseudogene 9 (RPSAP9), non-coding RNA [NR_026890]	-1.7388902	0.029808238
A_32_P70724	*RPL24*	Homo sapiens ribosomal protein L24 (RPL24), mRNA [NM_000986]	1.2718648	0.031603426
A_22_P00011122	*KDM5B*	Homo sapiens lysine (K)-specific demethylase 5B (KDM5B), mRNA [NM_006618]	-1.0061672	0.03297842
A_23_P65768	*lnc-NUMB-2*	BX101396 Soares_testis_NHT Homo sapiens cDNA clone IMAGp998D114108, mRNA sequence [BX101396]	-0.5395293	0.04147798
A_23_P5441	*RSL24D1*	Homo sapiens ribosomal L24 domain containing 1 (RSL24D1), mRNA [NM_016304]	-0.9613993	0.038283803
A_23_P201918	*ABCB6*	Homo sapiens ATP-binding cassette, sub-family B (MDR/TAP), member 6 (Langereis blood group) (ABCB6), mRNA [NM_005689]	-1.1744943	0.047832783
A_23_P4190	*ABCB10*	Homo sapiens ATP-binding cassette, sub-family B (MDR/TAP), member 10 (ABCB10), mRNA [NM_012089]	1.205969	0.0442831
A_23_P122052	*ACSF2*	Homo sapiens acyl-CoA synthetase family member 2 (ACSF2), transcript variant 2, mRNA [NM_025149]	-1.225816	0.028573772

A_33_P3243717	*GPX8*	Homo sapiens glutathione peroxidase 8 (putative) (GPX8), mRNA [NM_001008397]	-1.1705825	0.03794058
A_22_P00008083	*ZNF248*	Homo sapiens zinc finger protein 248 (ZNF248), transcript variant 1, mRNA [NM_021045]	-1.3502641	0.037523273
A_22_P00005683	*LOC101929761*	PREDICTED: Homo sapiens uncharacterized LOC101929761 (LOC101929761), transcript variant X2, ncRNA [XR_428452]	-1.4002	0.037795458
A_19_P00315780	*MMP24-AS1*	Homo sapiens MMP24 antisense RNA 1 (MMP24-AS1), transcript variant 1, long non-coding RNA [NR_102705]	-0.90396833	0.042925138
A_33_P3248420	*WDR20*	Homo sapiens WD repeat domain 20 (WDR20), transcript variant 1, mRNA [NM_181291]	-1.0169041	0.03668808
A_23_P371787	*FAM185A*	Homo sapiens family with sequence similarity 185, member A (FAM185A), transcript variant 1, mRNA [NM_001145268]	-1.2538649	0.029808238
A_33_P3283971	*SUSD6*	Homo sapiens sushi domain containing 6 (SUSD6), mRNA [NM_014734]	-0.92935085	0.046962585
A_23_P259328	*NFKBIL1*	Homo sapiens nuclear factor of kappa light polypeptide gene enhancer in B-cells inhibitor-like 1 (NFKBIL1), transcript variant 1, mRNA [NM_005007]	-0.7794278	0.042621672
A_22_P00000010	*PEX3*	Homo sapiens peroxisomal biogenesis factor 3 (PEX3), mRNA [NM_003630]	-1.1875299	0.044444222
A_24_P360269	*ADAM12*	Homo sapiens ADAM metallopeptidase domain 12 (ADAM12), transcript variant 1, mRNA [NM_003474]	-0.58989364	0.03403978
A_33_P3283601	*RNASET2*	Homo sapiens ribonuclease T2 (RNASET2), mRNA [NM_003730]	-0.8662009	0.028583162
A_23_P105664	*LOC389033*	Homo sapiens placenta-specific 9 pseudogene (LOC389033), non-coding RNA [NR_026740]	-0.85004914	0.04968268
A_23_P211047	*CCDC59*	Homo sapiens coiled-coil domain containing 59 (CCDC59), transcript variant 1, mRNA [NM_014167]	-3.3663034	0.04438234
A_24_P417189	*BACH1*	Homo sapiens BTB and CNC homology 1, basic leucine zipper transcription factor 1 (BACH1), transcript variant 1, mRNA [NM_206866]	-1.2892904	0.04206122
A_33_P3332438	*DUSP9*	Homo sapiens dual specificity phosphatase 9 (DUSP9), mRNA [NM_001395]	-2.402437	0.024948223

A_24_P235783	*GINM1*	Homo sapiens glycoprotein integral membrane 1 (GINM1), mRNA [NM_138785]	-0.6609311	0.035935212
A_33_P3316696	*SF1*	splicing factor 1 [Source:HGNC Symbol;Acc:HGNC:12950] [ENST00000463343]	-0.6673228	0.046256438
A_23_P202458	*SETD7*	Homo sapiens SET domain containing (lysine methyltransferase) 7 (SETD7), mRNA [NM_030648]	-1.1534165	0.03233256
A_19_P00322762	*ZNF22*	Homo sapiens zinc finger protein 22 (ZNF22), mRNA [NM_006963]	0.55953336	0.0419793
A_24_P154037	*ZNF83*	zinc finger protein 83 [Source:HGNC Symbol;Acc:HGNC:13158] [ENST00000598190]	1.4127743	0.04800724
A_33_P3284939	*IRS2*	Homo sapiens insulin receptor substrate 2 (IRS2), mRNA [NM_003749]	-0.6171222	0.033583984
A_24_P50753	*TMEM189*	Homo sapiens transmembrane protein 189 (TMEM189), transcript variant 1, mRNA [NM_199129]	-0.7696037	0.031195676
A_33_P3329878	*NUDT4*	Homo sapiens nudix (nucleoside diphosphate linked moiety X)-type motif 4 (NUDT4), transcript variant 2, mRNA [NM_199040]	0.8557642	0.024948223
A_33_P3344603	*RNF216*	Homo sapiens ring finger protein 216 (RNF216), transcript variant 1, mRNA [NM_207111]	-2.2707324	0.036131933
A_23_P86493	*ZNF280D*	Homo sapiens zinc finger protein 280D (ZNF280D), transcript variant 5, mRNA [NM_001288589]	-1.5546591	0.027751032
A_23_P342934	*LBX1*	Homo sapiens ladybird homeobox 1 (LBX1), mRNA [NM_006562]	-1.0947325	0.03297842
A_33_P3317850	*TLE3*	Homo sapiens transducin-like enhancer of split 3 (TLE3), transcript variant 1, mRNA [NM_005078]	-1.5225906	0.033095628
A_24_P248240	*RIOK3*	Homo sapiens RIO kinase 3 (RIOK3), mRNA [NM_003831]	-1.1346489	0.046624914
A_23_P125042	*SYT11*	Homo sapiens synaptotagmin XI (SYT11), mRNA [NM_152280]	-2.0139394	0.03403978
A_32_P230828	*ZNF222*	Homo sapiens zinc finger protein 222 (ZNF222), transcript variant 2, mRNA [NM_013360]	-0.90266585	0.04250221
A_33_P3251054	*GAS5*	Homo sapiens growth arrest-specific 5 (non-protein coding) (GAS5), long non-coding RNA [NR_002578]	-1.3440473	0.04733213
A_22_P00003471	*RNF38*	Homo sapiens ring finger protein 38 (RNF38), transcript variant 2, mRNA [NM_194328]	-1.1531422	0.04253229

A_33_P3297444	*lnc-CCDC69-1*	LNCipedia lincRNA (lnc-CCDC69-1), lincRNA [lnc-CCDC69-1:1]	-1.4655617	0.047617503
A_21_P0003259	*ABTB1*	Homo sapiens ankyrin repeat and BTB (POZ) domain containing 1 (ABTB1), transcript variant 1, mRNA [NM_032548]	-2.2894905	0.04079467
A_22_P00015126	*lnc-SERPINI1-9*	LNCipedia lincRNA (lnc-SERPINI1-9), lincRNA [lnc-SERPINI1-9:6]	-0.7783608	0.0429929
A_33_P3307197	*lnc-SNX27-1*	LNCipedia lincRNA (lnc-SNX27-1), lincRNA [lnc-SNX27-1:1]	2.8469467	0.027942823
A_23_P38732	*PTGFRN*	Homo sapiens prostaglandin F2 receptor inhibitor (PTGFRN), mRNA [NM_020440]	-0.6642363	0.04555549
A_23_P309865	*CDH2*	Homo sapiens cadherin 2, type 1, N-cadherin (neuronal) (CDH2), mRNA [NM_001792]	-1.3404522	0.04919617
A_33_P3379017	*ZNF449*	Homo sapiens zinc finger protein 449 (ZNF449), mRNA [NM_152695]	1.3238811	0.04079467
A_23_P60240	*HIC2*	Homo sapiens hypermethylated in cancer 2 (HIC2), mRNA [NM_015094]	-4.6719117	0.024948223
A_23_P430181	*PIGO*	Homo sapiens phosphatidylinositol glycan anchor biosynthesis, class O (PIGO), transcript variant 1, mRNA [NM_032634]	-3.2400198	0.032893
A_33_P3357918	*ZBTB3*	Homo sapiens zinc finger and BTB domain containing 3 (ZBTB3), mRNA [NM_024784]	-1.3183956	0.024948223
A_24_P192627	*PGLS*	Homo sapiens 6-phosphogluconolactonase (PGLS), mRNA [NM_012088]	-1.7915826	0.029573848
A_23_P388900	*MLLT3*	Homo sapiens myeloid/lymphoid or mixed-lineage leukemia (trithorax homolog, Drosophila); translocated to, 3 (MLLT3), transcript variant 1, mRNA [NM_004529]	-0.5476335	0.038230915
A_33_P3423425	*SLC22A15*	Homo sapiens solute carrier family 22, member 15 (SLC22A15), mRNA [NM_018420]	-0.8367598	0.044186465
A_32_P37592	*ZNF770*	Homo sapiens zinc finger protein 770 (ZNF770), mRNA [NM_014106]	-0.927602	0.026587285
A_22_P00017379	*SCARNA17*	Homo sapiens small Cajal body-specific RNA 17 (SCARNA17), guide RNA [NR_003003]	0.58782303	0.042656552
A_24_P13230	*lnc-UVRAG-2*	DA940890 SPLEN2 Homo sapiens cDNA clone SPLEN2014945 5', mRNA sequence [DA940890]	-1.7436639	0.048502974

A_33_P3417123	*RAB6A*	Homo sapiens RAB6A, member RAS oncogene family (RAB6A), transcript variant 1, mRNA [NM_002869]	-2.5527058	0.032611065
A_33_P3250253	*C6orf203*	Homo sapiens chromosome 6 open reading frame 203 (C6orf203), transcript variant 1, mRNA [NM_016487]	1.7326624	0.042440254
A_33_P3229241	*LOC100131150*	Homo sapiens cDNA FLJ38875 fis, clone MESAN2013936. [AK096194]	-1.3542922	0.029808238
A_33_P3242014	*HIST2H2BF*	histone cluster 2, H2bf [Source:HGNC Symbol;Acc:HGNC:24700] [ENST00000369167]	1.9143215	0.030293303
A_23_P143935	*PHC1*	Homo sapiens polyhomeotic homolog 1 (Drosophila) (PHC1), mRNA [NM_004426]	-0.9818156	0.03800715
A_23_P334870	*PIGZ*	Homo sapiens phosphatidylinositol glycan anchor biosynthesis, class Z (PIGZ), mRNA [NM_025163]	1.2520276	0.034477387
A_33_P3341239	*TMEM217*	Homo sapiens transmembrane protein 217 (TMEM217), transcript variant 1, mRNA [NM_145316]	-1.0556275	0.03933512
A_33_P3217704	*CDK16*	Homo sapiens cyclin-dependent kinase 16 (CDK16), transcript variant 1, mRNA [NM_006201]	-1.1020617	0.04173021
A_23_P206018	*FAM214B*	Homo sapiens family with sequence similarity 214, member B (FAM214B), mRNA [NM_025182]	-0.6084714	0.038782965
A_33_P3387170	*TPM1*	Homo sapiens tropomyosin 1 (alpha) (TPM1), transcript variant Tpm1.6, mRNA [NM_001018004]	-1.532507	0.02598378
A_33_P3216150	*MATN1-AS1*	Homo sapiens MATN1 antisense RNA 1 (MATN1-AS1), long non-coding RNA [NR_034182]	-0.58400077	0.042440254
A_32_P230825	*PRB4*	Homo sapiens proline-rich protein BstNI subfamily 4 (PRB4), transcript variant 1, mRNA [NM_002723]	-1.4008374	0.029979022
A_33_P3349827	*MLF1*	Homo sapiens myeloid leukemia factor 1 (MLF1), transcript variant 4, mRNA [NM_001195432]	1.1036466	0.039264042
A_33_P3377060	*RBM3*	Homo sapiens RNA binding motif (RNP1, RRM) protein 3 (RBM3), mRNA [NM_006743]	-0.8028642	0.045638688
A_21_P0000651	*SSR1*	Homo sapiens signal sequence receptor, alpha (SSR1), transcript variant 1, mRNA [NM_003144]	-1.9841967	0.047879543
A_33_P3293446	*HERC2P7*	Homo sapiens hect domain and RLD 2 pseudogene 7 (HERC2P7), non-coding RNA [NR_036470]	-1.7630283	0.026440267

A_24_P649747	*KIAA1462*	Homo sapiens KIAA1462 (KIAA1462), mRNA [NM_020848]	-1.3144261	0.045605946
A_24_P333421	*BMS1*	Homo sapiens BMS1 ribosome biogenesis factor (BMS1), mRNA [NM_014753]	-0.6011382	0.036044724
A_23_P167040	*ZNF862*	Homo sapiens zinc finger protein 862 (ZNF862), mRNA [NM_001099220]	-1.2654576	0.034210585
A_23_P210425	*PDIA5*	Homo sapiens protein disulfide isomerase family A, member 5 (PDIA5), transcript variant 1, mRNA [NM_006810]	-1.5167603	0.037136003
A_22_P00002059	*MYL9*	Homo sapiens myosin, light chain 9, regulatory (MYL9), transcript variant 2, mRNA [NM_181526]	-0.88246083	0.028573772
A_33_P3244424	*MIR100HG*	Homo sapiens mir-100-let-7a-2 cluster host gene (non-protein coding) (MIR100HG), long non-coding RNA [NR_024430]	-0.7964599	0.048707504
A_19_P00326558	*SNRPE*	Homo sapiens small nuclear ribonucleoprotein polypeptide E (SNRPE), mRNA [NM_003094]	-1.8079836	0.02737376
A_22_P00017736	*ELMOD3*	Homo sapiens ELMO/CED-12 domain containing 3 (ELMOD3), transcript variant 1, mRNA [NM_032213]	-0.8375032	0.028573772
A_33_P3279581	*NUFIP2*	Homo sapiens nuclear fragile X mental retardation protein interacting protein 2 (NUFIP2), mRNA [NM_020772]	1.6644439	0.033853088
A_24_P223163	*ARV1*	Homo sapiens ARV1 homolog (S. cerevisiae) (ARV1), mRNA [NM_022786]	2.6251998	0.02737376
A_24_P104512	*NAF1*	Homo sapiens nuclear assembly factor 1 ribonucleoprotein (NAF1), transcript variant 1, mRNA [NM_138386]	-0.75463724	0.04705478
A_24_P67806	*EVPL*	Homo sapiens envoplakin (EVPL), mRNA [NM_001988]	1.016999	0.044186465
A_23_P163711	*MTR*	Homo sapiens 5-methyltetrahydrofolate-homocysteine methyltransferase (MTR), transcript variant 1, mRNA [NM_000254]	-0.8962178	0.044364784
A_22_P00004148	*FAM57B*	Homo sapiens family with sequence similarity 57, member B (FAM57B), mRNA [NM_031478]	1.0092139	0.043335356
A_22_P00013517	*lnc-CLEC18B-2*	LNCipedia lincRNA (lnc-CLEC18B-2), lincRNA [lnc-CLEC18B-2:2]	1.7275975	0.026808027
A_33_P3294237	*ERICH6-AS1*	Homo sapiens ERICH6 antisense RNA 1 (ERICH6-AS1), transcript variant 1, long non-coding RNA [NR_121674]	-1.411146	0.03136619

A_32_P235796	*MTF1*	Homo sapiens metal-regulatory transcription factor 1 (MTF1), mRNA [NM_005955]	2.7291026	0.046680246
A_24_P269814	*BBS12*	Homo sapiens Bardet-Biedl syndrome 12 (BBS12), transcript variant 2, mRNA [NM_152618]	-0.99264014	0.03388051
A_24_P58337	*PLEKHA1*	Homo sapiens pleckstrin homology domain containing, family A (phosphoinositide binding specific) member 1 (PLEKHA1), transcript variant 2, mRNA [NM_001001974]	-0.75608784	0.032893
A_33_P3255229	*FTH1*	Homo sapiens ferritin, heavy polypeptide 1 (FTH1), mRNA [NM_002032]	-1.692465	0.034210585
A_23_P69431	*SETD7*	Homo sapiens cDNA clone IMAGE:5312439. [BC066361]	-1.4561416	0.027751032
A_33_P3333560	*RPL4*	Homo sapiens ribosomal protein L4 (RPL4), mRNA [NM_000968]	-1.5197952	0.03945833
A_23_P18887	*FBXO34*	Homo sapiens F-box protein 34 (FBXO34), transcript variant 2, mRNA [NM_152231]	0.9233462	0.047727138
A_23_P9280	*MCCC2*	Homo sapiens methylcrotonoyl-CoA carboxylase 2 (beta) (MCCC2), mRNA [NM_022132]	-1.1971798	0.03138659
A_23_P134809	*LOC389765*	Homo sapiens kinesin family member 27 pseudogene (LOC389765), non-coding RNA [NR_029410]	1.7044469	0.03716006
A_24_P47182	*NSMAF*	Homo sapiens neutral sphingomyelinase (N-SMase) activation associated factor (NSMAF), transcript variant 1, mRNA [NM_003580]	-1.08774	0.042656552
A_33_P3268408	*VCL*	Homo sapiens vinculin (VCL), transcript variant 1, mRNA [NM_014000]	-1.2521452	0.040555127
A_33_P3342430	*MSTO1*	Homo sapiens misato homolog 1 (Drosophila), mRNA (cDNA clone IMAGE:4828998). [BC070067]	-1.0843334	0.033583984
A_24_P378987	*ZADH2*	Homo sapiens zinc binding alcohol dehydrogenase domain containing 2 (ZADH2), mRNA [NM_175907]	-1.1447473	0.027751032
A_21_P0000410	*DHRSX*	Homo sapiens dehydrogenase/reductase (SDR family) X-linked (DHRSX), mRNA [NM_145177]	-0.36877632	0.024948223
A_22_P00015986	*SNORD114-13*	Homo sapiens small nucleolar RNA, C/D box 114-13 (SNORD114-13), small nucleolar RNA [NR_003206]	-1649749	0.026038945

A_23_P386320	*RBM39*	Homo sapiens RNA binding motif protein 39 (RBM39), transcript variant 1, mRNA [NM_184234]	-0.89781046	0.043983467
A_23_P92410	*MFI2*	Homo sapiens antigen p97 (melanoma associated) identified by monoclonal antibodies 133.2 and 96.5 (MFI2), transcript variant 2, mRNA [NM_033316]	0.9624002	0.03969308
A_24_P399362	*CASP3*	Homo sapiens caspase 3, apoptosis-related cysteine peptidase (CASP3), transcript variant alpha, mRNA [NM_004346]	1.460577	0.044325642
A_33_P3412900	*PSMG2*	Homo sapiens proteasome (prosome, macropain) assembly chaperone 2 (PSMG2), transcript variant 1, mRNA [NM_020232]	-0.60274166	0.030915326
A_33_P3349444	*CBLN3*	Homo sapiens cerebellin 3 precursor (CBLN3), mRNA [NM_001039771]	-12469174	0.027751032
A_32_P24581	*NAGA*	Homo sapiens N-acetylgalactosaminidase, alpha- (NAGA), mRNA [NM_000262]	-0.7175374	0.043837514
A_22_P00009218	*RPS27A*	Homo sapiens ribosomal protein S27a (RPS27A), transcript variant 1, mRNA [NM_002954]	-1.1289326	0.036443383
A_24_P267997	*lnc-LPPR5.1-1*	LNCipedia lincRNA (lnc-LPPR5.1-1), lincRNA [lnc-LPPR5.1-1:1]	-0.9393985	0.04250221
A_22_P00017059	*TOR1AIP1*	Homo sapiens torsin A interacting protein 1 (TOR1AIP1), transcript variant 2, mRNA [NM_015602]	-0.56695175	0.039287783
A_22_P00015264	*ADAM8*	Homo sapiens ADAM metallopeptidase domain 8 (ADAM8), transcript variant 2, mRNA [NM_001164489]	-1.6677871	0.040555127
A_22_P00013430	*SPANXA2-OT1*	Homo sapiens SPANXA2 overlapping transcript 1 (SPANXA2-OT1), long non-coding RNA [NR_037183]	1.5176948	0.031091278
A_33_P3291454	*BTG1*	Homo sapiens B-cell translocation gene 1, anti-proliferative (BTG1), mRNA [NM_001731]	-12785338	0.024948223
A_24_P941759	*CCDC172*	Homo sapiens coiled-coil domain containing 172 (CCDC172), mRNA [NM_198515]	1.8606278	0.027751032
A_24_P271049	*G2E3*	Homo sapiens G2/M-phase specific E3 ubiquitin protein ligase (G2E3), mRNA [NM_017769]	0.9329677	0.047266874
A_23_P128613	*C18orf32*	Homo sapiens chromosome 18 open reading frame 32 (C18orf32), transcript variant 2, mRNA [NM_001035005]	-14174721	0.03808641

A_33_P3338928	*KDELC1*	Homo sapiens KDEL (Lys-Asp-Glu-Leu) containing 1 (KDELC1), mRNA [NM_024089]	2.3339128	0.047094904
A_23_P252808	*DAB2*	Homo sapiens Dab, mitogen-responsive phosphoprotein, homolog 2 (Drosophila) (DAB2), transcript variant 1, mRNA [NM_001343]	-0.38197958	0.030699471
A_23_P35354	*WBP1*	Homo sapiens WW domain binding protein 1 (WBP1), mRNA [NM_012477]	-2.0923991	0.049976278
A_33_P3318861	*CWF19L1*	Homo sapiens CWF19-like 1, cell cycle control (S. pombe) (CWF19L1), transcript variant 1, mRNA [NM_018294]	-0.6996291	0.042994436
A_23_P165891	*DYTN*	Homo sapiens dystrotelin (DYTN), mRNA [NM_001093730]	-1.3978677	0.029808238
A_24_P276816	*TAF1B*	Homo sapiens TATA box binding protein (TBP)-associated factor, RNA polymerase I, B, 63kDa (TAF1B), mRNA [NM_005680]	-0.6896441	0.042526342
A_23_P404091	*RCOR3*	Homo sapiens REST corepressor 3 (RCOR3), transcript variant 4, mRNA [NM_018254]	-0.9436034	0.0268412
A_33_P3279940	*GRPEL2*	Homo sapiens GrpE-like 2, mitochondrial (E. coli) (GRPEL2), mRNA [NM_152407]	1.7720606	0.031603426
A_33_P3270802	*TM2D1*	Homo sapiens TM2 domain containing 1 (TM2D1), mRNA [NM_032027]	-1.3204188	0.03510091
A_23_P500773	*CPSF3L*	Homo sapiens cleavage and polyadenylation specific factor 3-like (CPSF3L), transcript variant 1, mRNA [NM_001256456]	-1.2262871	0.03032242
A_23_P119789	*MAP3K7*	Homo sapiens mitogen-activated protein kinase kinase kinase 7 (MAP3K7), transcript variant A, mRNA [NM_003188]	-1.4534047	0.024948223
A_23_P406702	*TMEM185B*	Homo sapiens transmembrane protein 185B (TMEM185B), mRNA [NM_024121]	-0.9265372	0.049758088
A_23_P132536	*CAMSAP2*	Homo sapiens calmodulin regulated spectrin-associated protein family, member 2 (CAMSAP2), transcript variant 2, mRNA [NM_203459]	-0.61189437	0.038684342
A_24_P324314	*TRAK1*	Homo sapiens trafficking protein, kinesin binding 1 (TRAK1), transcript variant 1, mRNA [NM_001042646]	-1.1466328	0.02598378
A_24_P129232	*MXRA7*	Homo sapiens matrix-remodelling associated 7 (MXRA7), transcript variant 2, mRNA [NM_001008529]	-0.997112	0.033091284

A_23_P73114	*SERINC1*	Homo sapiens serine incorporator 1 (SERINC1), mRNA [NM_020755]	-0.96418476	0.03835606
A_23_P259172	*PROS1*	Homo sapiens protein S (alpha) (PROS1), mRNA [NM_000313]	-1.4498653	0.034549404
A_23_P90143	*SSR4*	Homo sapiens signal sequence receptor, delta (SSR4), transcript variant 2, mRNA [NM_006280]	-1.3110135	0.028222434
A_23_P360797	*RPL13A*	Homo sapiens ribosomal protein L13a (RPL13A), transcript variant 1, mRNA [NM_012423]	-1.0354342	0.035515364
A_33_P3637909	*NTF3*	Homo sapiens neurotrophin 3 (NTF3), transcript variant 2, mRNA [NM_002527]	-2.0889244	0.032958407
A_23_P94216	*UBE2D4*	Homo sapiens ubiquitin-conjugating enzyme E2D 4 (putative) (UBE2D4), mRNA [NM_015983]	-1.0647305	0.035207514
A_22_P00013798	*LONRF1*	Homo sapiens LON peptidase N-terminal domain and ring finger 1 (LONRF1), mRNA [NM_152271]	-1.1489952	0.03136619
A_24_P135753	*LOC101928433*	PREDICTED: Homo sapiens uncharacterized LOC101928433 (LOC101928433), ncRNA [XR_241982]	-1.7422744	0.037560765
A_33_P3258274	*PTPLB*	protein tyrosine phosphatase-like (proline instead of catalytic arginine), member b [Source:HGNC Symbol;Acc:HGNC:9640] [ENST00000383657]	-0.865572	0.037795458
A_32_P197489	*TFPI*	tissue factor pathway inhibitor (lipoprotein-associated coagulation inhibitor) [Source:HGNC Symbol;Acc:HGNC:11760] [ENST00000481132]	-1.8467423	0.0376762
A_24_P419276	*KLF13*	Homo sapiens Kruppel-like factor 13 (KLF13), transcript variant 1, mRNA [NM_015995]	-1.2978673	0.024948223
A_33_P3410372	*ZNF248*	Homo sapiens zinc finger protein 248 (ZNF248), transcript variant 1, mRNA [NM_021045]	-0.68370503	0.03662822
A_23_P57709	*NUDT10*	Homo sapiens nudix (nucleoside diphosphate linked moiety X)-type motif 10 (NUDT10), mRNA [NM_153183]	1.115917	0.041609965
A_21_P0014195	*PCOLCE2*	Homo sapiens procollagen C-endopeptidase enhancer 2 (PCOLCE2), mRNA [NM_013363]	-0.4921203	0.041768707
A_22_P00002608	*LGALS2*	Homo sapiens cDNA FLJ27172 fis, clone SYN01847. [AK130682]	-0.9193932	0.041628964

A_22_P00003206	*SSTR5-AS1*	Homo sapiens SSTR5 antisense RNA 1 (SSTR5-AS1), long non-coding RNA [NR_027242]	-4.123674	0.034549404
A_23_P80643	*LINC01301*	long intergenic non-protein coding RNA 1301 [Source:HGNC Symbol;Acc:HGNC:50464] [ENST00000532232]	1.81133	0.028573772
A_33_P3320804	*SETMAR*	Homo sapiens SET domain and mariner transposase fusion gene (SETMAR), transcript variant 1, mRNA [NM_006515]	-0.58913034	0.04915393
A_32_P234738	*IP6K1*	Homo sapiens inositol hexakisphosphate kinase 1 (IP6K1), transcript variant 3, mRNA [NM_001242829]	0.95472807	0.04971531
A_24_P250765	*RPL21*	Homo sapiens ribosomal protein L21 (RPL21), mRNA [NM_000982]	0.62416613	0.03947304
A_22_P00016228	*PRKG1*	Homo sapiens protein kinase, cGMP-dependent, type I (PRKG1), transcript variant 2, mRNA [NM_006258]	-1.1888384	0.04390963
A_23_P106481	*lnc-TM9SF2-2*	LNCipedia lincRNA (lnc-TM9SF2-2), lincRNA [lnc-TM9SF2-2:1]	-0.81906325	0.028573772
A_33_P3402763	*EMC4*	Homo sapiens ER membrane protein complex subunit 4 (EMC4), transcript variant 1, mRNA [NM_016454]	-1.3676896	0.04055477
A_23_P251316	*NIFK*	Homo sapiens nucleolar protein interacting with the FHA domain of MKI67 (NIFK), mRNA [NM_032390]	-1.2045642	0.04253229
A_24_P916718	*CTNND1*	Homo sapiens catenin (cadherin-associated protein), delta 1 (CTNND1), transcript variant 3, mRNA [NM_001331]	1.0052663	0.033433396
A_33_P3738458	*ZNF467*	Homo sapiens zinc finger protein 467 (ZNF467), mRNA [NM_207336]	-1.1742465	0.031091278
A_23_P155417	*TNS1*	Homo sapiens tensin 1 (TNS1), mRNA [NM_022648]	-1.0900232	0.034210585
A_33_P3422787	*ABHD14B*	Homo sapiens abhydrolase domain containing 14B (ABHD14B), transcript variant 1, mRNA [NM_032750]	-0.8201921	0.047591254
A_23_P81158	*lnc-TM4SF4-2*	Homo sapiens cDNA, FLJ99482. [AK309441]	1.4633311	0.02737376
A_33_P3281468	*ADH1C*	Homo sapiens alcohol dehydrogenase 1C (class I), gamma polypeptide (ADH1C), mRNA [NM_000669]	-0.62573904	0.028573772
A_23_P95165	*STARD9*	Homo sapiens StAR-related lipid transfer (START) domain containing 9 (STARD9), mRNA [NM_020759]	-0.57626057	0.0442831

A_33_P3393537	*SEMA4B*	Homo sapiens sema domain, immunoglobulin domain (Ig), transmembrane domain (TM) and short cytoplasmic domain, (semaphorin) 4B (SEMA4B), transcript variant 1, mRNA [NM_020210]	1.4091786	0.037221838
A_33_P3262327	*PTAFR*	Homo sapiens platelet-activating factor receptor (PTAFR), transcript variant 2, mRNA [NM_001164722]	2.4455276	0.030293303
A_33_P3256510	*THADA*	Homo sapiens thyroid adenoma associated (THADA), transcript variant 1, mRNA [NM_022065]	1.0353153	0.044846643
A_24_P341019	*KCNK12*	Homo sapiens potassium channel, two pore domain subfamily K, member 12 (KCNK12), mRNA [NM_022055]	1.1349132	0.0442831
A_32_P10396	*TMEM230*	Homo sapiens transmembrane protein 230 (TMEM230), transcript variant 2, mRNA [NM_001009924]	2.0266051	0.027019119
A_23_P118105	*WDFY3*	Homo sapiens WD repeat and FYVE domain containing 3 (WDFY3), mRNA [NM_014991]	-1.7240564	0.02598378
A_33_P3375496	*NDUFB10*	Homo sapiens NADH dehydrogenase (ubiquinone) 1 beta subcomplex, 10, 22kDa (NDUFB10), mRNA [NM_004548]	2.0036004	0.03136619
A_33_P6501835	*MBD2*	Homo sapiens methyl-CpG binding domain protein 2 (MBD2), transcript variant 1, mRNA [NM_003927]	-1.1934458	0.02737376
A_23_P165722	*LOC101929128*	Homo sapiens uncharacterized LOC101929128 (LOC101929128), long non-coding RNA [NR_125431]	-0.9711776	0.043665398
A_23_P256933	*EIF4E2*	Homo sapiens eukaryotic translation initiation factor 4E family member 2 (EIF4E2), transcript variant 1, mRNA [NM_004846]	1.2542306	0.047266874
A_22_P00019657	*RPL24*	Homo sapiens ribosomal protein L24 (RPL24), mRNA [NM_000986]	-0.9549429	0.03403978
A_23_P374767	*lnc-NFAT5-1*	Q4RE65_TETNG (Q4RE65) Chromosome 2 SCAF15135, whole genome shotgun sequence. (Fragment), partial (3%) [THC2561515]	1.2527158	0.04135566
A_32_P180265	*UBE2D4*	Homo sapiens ubiquitin-conjugating enzyme E2D 4 (putative) (UBE2D4), mRNA [NM_015983]	-1.05047	0.032261204
A_21_P0003404	*STK36*	Homo sapiens serine/threonine kinase 36 (STK36), transcript variant 1, mRNA [NM_015690]	1.2800422	0.031965

A_22_P00021090	*C4orf27*	Homo sapiens chromosome 4 open reading frame 27 (C4orf27), mRNA [NM_017867]	-2.0072567	0.045740407
A_23_P24709	*lnc-MDH1B-2*	LNCipedia lincRNA (lnc-MDH1B-2), lincRNA [lnc-MDH1B-2:1]	-1.1077656	0.035935212
A_23_P80827	*OSBP*	Homo sapiens oxysterol binding protein (OSBP), mRNA [NM_002556]	1.7988596	0.0370712
A_24_P83615	*FYTTD1*	Homo sapiens forty-two-three domain containing 1 (FYTTD1), transcript variant 2, mRNA [NM_001011537]	-1.2606568	0.04610903
A_23_P258018	*NLRP1*	Homo sapiens NLR family, pyrin domain containing 1 (NLRP1), transcript variant 5, mRNA [NM_001033053]	-1.3662403	0.045316998
A_33_P3331307	*MYL5*	Homo sapiens myosin, light chain 5, regulatory (MYL5), mRNA [NM_002477]	-1.3638098	0.029979022
A_23_P90220	*CDC42EP3*	Homo sapiens CDC42 effector protein (Rho GTPase binding) 3 (CDC42EP3), transcript variant 1, mRNA [NM_006449]	-1.3209145	0.049759135
A_32_P206293	*ZNF585A*	Homo sapiens zinc finger protein 585A (ZNF585A), transcript variant 1, mRNA [NM_152655]	-1.1873472	0.045638688
A_23_P169197	*ZNF322*	Homo sapiens zinc finger protein 322 (ZNF322), transcript variant 1, mRNA [NM_001242797]	-1.3805376	0.02737376
A_24_P724153	*HSDL2*	Homo sapiens hydroxysteroid dehydrogenase like 2 (HSDL2), transcript variant 1, mRNA [NM_032303]	-1.5147836	0.034210585
A_23_P386411	*MAT2B*	Homo sapiens methionine adenosyltransferase II, beta (MAT2B), transcript variant 2, mRNA [NM_182796]	-1.2779922	0.028573772
A_23_P7074	*PPID*	Homo sapiens peptidylprolyl isomerase D (PPID), mRNA [NM_005038]	-0.6388228	0.04591278
A_24_P282343	*NOA1*	Homo sapiens nitric oxide associated 1 (NOA1), mRNA [NM_032313]	1.7859628	0.044286337
A_22_P00012283	*CDKL5*	Homo sapiens cyclin-dependent kinase-like 5 (CDKL5), transcript variant I, mRNA [NM_003159]	-0.97496843	0.043984637
A_23_P19291	*PPP2R2D*	Homo sapiens protein phosphatase 2, regulatory subunit B, delta (PPP2R2D), transcript variant 3, mRNA [NM_001291310]	-0.76665044	0.03205687

A_22_P00014120	*TUBB2A*	Homo sapiens tubulin, beta 2A class IIa (TUBB2A), mRNA [NM_001069]	-0.77053875	0.042850092
A_22_P00005923	*lnc-SCAP-1*	Homo sapiens cDNA clone IMAGE:5272313. [BC036224]	-1.6431661	0.04469611
A_33_P3304754	*HOXA11-AS*	Homo sapiens HOXA11 antisense RNA (HOXA11-AS), antisense RNA [NR_002795]	0.28174597	0.045740407
A_32_P6221	*TPP2*	tripeptidyl peptidase II [Source:HGNC Symbol;Acc:HGNC:12016] [ENST00000490010]	-0.73717266	0.036667652
A_24_P255005	*FAM86C2P*	Homo sapiens family with sequence similarity 86, member C2, pseudogene (FAM86C2P), non-coding RNA [NR_024249]	-1.2488925	0.025734548
A_23_P94319	*ZNF24*	Homo sapiens zinc finger protein 24 (ZNF24), mRNA [NM_006965]	0.8631455	0.03668808
A_23_P338952	*KBTBD11*	Homo sapiens kelch repeat and BTB (POZ) domain containing 11 (KBTBD11), mRNA [NM_014867]	-1.4376901	0.026208585
A_33_P3297562	*S100PBP*	Homo sapiens S100P binding protein (S100PBP), transcript variant 1, mRNA [NM_022753]	-0.4320646	0.028573772
A_22_P00015645	*IRX2*	Homo sapiens iroquois homeobox 2 (IRX2), transcript variant 1, mRNA [NM_033267]	-1.3131366	0.029979022
A_32_P184279	*lnc-SUPT6H-3*	LNCipedia lincRNA (lnc-SUPT6H-3), lincRNA [lnc-SUPT6H-3:1]	-4.48956	0.02737376
A_33_P3589819	*CCDC6*	Homo sapiens coiled-coil domain containing 6 (CCDC6), mRNA [NM_005436]	-1.2571857	0.024948223
A_33_P3213204	*MIATNB*	Homo sapiens cDNA: FLJ22849 fis, clone KAIA987. [AK026502]	-1.3234015	0.03770023
A_23_P203299	*MAGEF1*	Homo sapiens melanoma antigen family F, 1 (MAGEF1), mRNA [NM_022149]	1.4686241	0.024948223
A_33_P3416946	*RCN1*	Homo sapiens reticulocalbin 1, EF-hand calcium binding domain (RCN1), mRNA [NM_002901]	-1.2111511	0.03245403
A_23_P333129	*ACTR10*	Homo sapiens actin-related protein 10 homolog (S. cerevisiae) (ACTR10), mRNA [NM_018477]	0.9923872	0.030152684
A_24_P151121	*IGF2R*	Homo sapiens insulin-like growth factor 2 receptor (IGF2R), mRNA [NM_000876]	-0.9469795	0.0442831
A_33_P3335920	*FAM195B*	Homo sapiens family with sequence similarity 195, member B (FAM195B), transcript variant 1, mRNA [NM_207368]	-0.81859183	0.04735342

A_22_P00008212	*SYNE1*	Homo sapiens spectrin repeat containing, nuclear envelope 1 (SYNE1), transcript variant 1, mRNA [NM_182961]	0.53736496	0.03590667
A_21_P0012376	*NCOA3*	Homo sapiens nuclear receptor coactivator 3 (NCOA3), transcript variant 1, mRNA [NM_181659]	-0.65649575	0.034306627
A_23_P144916	*GEMIN2*	Homo sapiens gem (nuclear organelle) associated protein 2 (GEMIN2), transcript variant gamma, mRNA [NM_001009183]	-1.7920648	0.03403978
A_33_P3276718	*GFPT2*	Homo sapiens glutamine-fructose-6-phosphate transaminase 2 (GFPT2), mRNA [NM_005110]	-1.9159251	0.039287783
A_23_P33045	*HGF*	Homo sapiens hepatocyte growth factor (hepapoietin A; scatter factor) (HGF), transcript variant 5, mRNA [NM_001010934]	-0.9646807	0.044288937
A_33_P3775741	*RPL26*	Homo sapiens ribosomal protein L26 (RPL26), mRNA [NM_000987]	-1.1946319	0.028321572
A_33_P3288359	*RPL32P3*	Homo sapiens ribosomal protein L32 pseudogene 3 (RPL32P3), non-coding RNA [NR_003111]	-1.3527079	0.035935212
A_22_P00000650	*PSMB10*	Homo sapiens proteasome (prosome, macropain) subunit, beta type, 10 (PSMB10), mRNA [NM_002801]	4.9194946	0.042907383
A_24_P35169	*lnc-ACSS3-2*	LNCipedia lincRNA (lnc-ACSS3-2), lincRNA [lnc-ACSS3-2:1]	-0.9679687	0.040555127
A_23_P433676	*GATAD1*	Homo sapiens GATA zinc finger domain containing 1 (GATAD1), transcript variant 1, mRNA [NM_021167]	-1.0455014	0.035560552
A_23_P31747	*ZNF529*	Homo sapiens zinc finger protein 529 (ZNF529), transcript variant 2, mRNA [NM_020951]	-0.8444791	0.03536427
A_22_P00008991	*C8orf76*	Homo sapiens chromosome 8 open reading frame 76 (C8orf76), mRNA [NM_032847]	1.7793627	0.04976156
A_22_P00015552	*GRTP1-AS1*	Homo sapiens GRTP1 antisense RNA 1 (GRTP1-AS1), transcript variant 2, long non-coding RNA [NR_120385]	-1.2113172	0.02737376
A_24_P677634	*lnc-STOM-1*	Homo sapiens cDNA FLJ27200 fis, clone SYN03061. [AK130710]	-0.75862163	0.024948223
A_24_P134319	*LOC493754*	Homo sapiens RAB guanine nucleotide exchange factor (GEF) 1 pseudogene (LOC493754), transcript variant 6, non-coding RNA [NR_111977]	-0.95488024	0.037221838
A_33_P3220422	*ADNP*	Homo sapiens activity-dependent neuroprotector homeobox (ADNP),	1.0206733	0.03431701

		transcript variant 3, mRNA [NM_001282531]		
A_24_P186346	*POM121L12*	Homo sapiens POM121 transmembrane nucleoporin-like 12 (POM121L12), mRNA [NM_182595]	-0.7159425	0.0442831
A_23_P37283	*ANAPC1*	Homo sapiens anaphase promoting complex subunit 1 (ANAPC1), mRNA [NM_022662]	-0.81517464	0.029573848
A_33_P3313704	*CGRRF1*	Homo sapiens cell growth regulator with ring finger domain 1 (CGRRF1), mRNA [NM_006568]	-0.6199839	0.031377234
A_23_P406438	*LOC100128374*	PREDICTED: Homo sapiens uncharacterized LOC100128374 (LOC100128374), misc_RNA [XR_112969]	2.259705	0.042693015
A_21_P0014370	*SRPK2*	Homo sapiens SRSF protein kinase 2 (SRPK2), transcript variant 2, mRNA [NM_182691]	-1.2789543	0.048323337
A_23_P59202	*SIKE1*	Homo sapiens suppressor of IKBKE 1 (SIKE1), transcript variant 1, mRNA [NM_001102396]	1.0297654	0.036667652
A_23_P149813	*TAF11*	Homo sapiens TAF11 RNA polymerase II, TATA box binding protein (TBP)-associated factor, 28kDa (TAF11), transcript variant 1, mRNA [NM_005643]	-1.1106639	0.049941108
A_24_P191847	*RSU1*	Homo sapiens Ras suppressor protein 1 (RSU1), transcript variant 1, mRNA [NM_012425]	1.4628425	0.028619371
A_24_P380679	*PTCD2*	Homo sapiens pentatricopeptide repeat domain 2 (PTCD2), transcript variant 1, mRNA [NM_024754]	-0.7234466	0.033726536
A_21_P0000116	*LSMEM1*	Homo sapiens leucine-rich single-pass membrane protein 1 (LSMEM1), transcript variant 1, mRNA [NM_182597]	-0.9408795	0.035769645
A_33_P3335124	*MID1*	Homo sapiens midline 1 (MID1), transcript variant 6, mRNA [NM_001193278]	-1.3781947	0.045445263
A_23_P435407	*RPS2*	Homo sapiens ribosomal protein S2 (RPS2), mRNA [NM_002952]	-0.9723232	0.024948223
A_24_P171873	*GPC4*	Homo sapiens glypican 4 (GPC4), mRNA [NM_001448]	-1.6639479	0.037560765
A_21_P0013960	*FBXO4*	Homo sapiens F-box protein 4 (FBXO4), transcript variant 1, mRNA [NM_012176]	-1.1417457	0.03590667
A_33_P3384775	*LOC101930246*	PREDICTED: Homo sapiens uncharacterized LOC101930246	-1.3626783	0.033091284

		(LOC101930246), transcript variant X2, ncRNA [XR_250239]		
A_33_P3281816	*ZBTB21*	zinc finger and BTB domain containing 21 [Source:HGNC Symbol;Acc:HGNC:13083] [ENST00000398497]	-3.9499643	0.029642703
A_33_P3383856	*CAP1*	Homo sapiens CAP, adenylate cyclase-associated protein 1 (yeast) (CAP1), transcript variant 1, mRNA [NM_006367]	-1.1923752	0.04958916
A_33_P3353948	*TREX2*	Homo sapiens three prime repair exonuclease 2 (TREX2), mRNA [NM_080701]	1.5435814	0.041929405
A_24_P9671	*lnc-BCL2L11-2*	LNCipedia lincRNA (lnc-BCL2L11-2), lincRNA [lnc-BCL2L11-2:2]	-0.5211355	0.04733987
A_23_P307955	*DNAJA1*	Homo sapiens DnaJ (Hsp40) homolog, subfamily A, member 1 (DNAJA1), mRNA [NM_001539]	1.1274607	0.046944562
A_22_P00021933	*SIRT5*	Homo sapiens sirtuin 5 (SIRT5), transcript variant 2, mRNA [NM_031244]	1.7297593	0.03257772
A_23_P204579	*P4HA2*	Homo sapiens prolyl 4-hydroxylase, alpha polypeptide II (P4HA2), transcript variant 1, mRNA [NM_004199]	1.7418882	0.029573848
A_24_P148094	*TDG*	Homo sapiens thymine-DNA glycosylase (TDG), mRNA [NM_003211]	-0.6361232	0.024948223
A_24_P414712	*LEPROT*	Homo sapiens leptin receptor overlapping transcript (LEPROT), transcript variant 1, mRNA [NM_017526]	-0.4919804	0.03136619
A_23_P71094	*BRPF3*	Homo sapiens bromodomain and PHD finger containing, 3 (BRPF3), mRNA [NM_015695]	-1.3026965	0.031377234
A_32_P49848	*GCC1*	Homo sapiens GRIP and coiled-coil domain containing 1 (GCC1), mRNA [NM_024523]	-1.0125623	0.027751032
A_23_P62276	*RHOQ*	Homo sapiens ras homolog family member Q (RHOQ), mRNA [NM_012249]	-0.65599346	0.040161368
A_23_P57760	*NKAP*	Homo sapiens NFKB activating protein (NKAP), mRNA [NM_024528]	-0.83794594	0.049413078
A_23_P147984	*PXYLP1*	Homo sapiens 2-phosphoxylose phosphatase 1 (PXYLP1), transcript variant 1, mRNA [NM_152282]	-1.3972986	0.034750238
A_24_P306561	*TRIP12*	Homo sapiens thyroid hormone receptor interactor 12 (TRIP12),	-1.3029201	0.03668808

		transcript variant 3, mRNA [NM_004238]		
A_23_P306215	*TCF25*	Homo sapiens transcription factor 25 (basic helix-loop-helix) (TCF25), mRNA [NM_014972]	-1.1114874	0.030578624
A_24_P342591	*FAM84A*	Homo sapiens family with sequence similarity 84, member A (FAM84A), mRNA [NM_145175]	-1.8334163	0.04792962
A_24_P589266	*RERE*	Homo sapiens arginine-glutamic acid dipeptide (RE) repeats (RERE), transcript variant 1, mRNA [NM_012102]	-0.36018375	0.045638688
A_33_P3256560	*SH3RF3*	Homo sapiens SH3 domain containing ring finger 3 (SH3RF3), mRNA [NM_001099289]	-0.36635783	0.032914247
A_22_P00025115	*ZER1*	Homo sapiens zyg-11 related, cell cycle regulator (ZER1), mRNA [NM_006336]	1.541755	0.038322687
A_23_P164237	*DDX26B*	Homo sapiens DEAD/H (Asp-Glu-Ala-Asp/His) box polypeptide 26B (DDX26B), mRNA [NM_182540]	0.82236123	0.030915326
A_32_P58425	*UTP6*	Homo sapiens UTP6, small subunit (SSU) processome component, homolog (yeast) (UTP6), mRNA [NM_018428]	-1.1401496	0.024948223
A_24_P570049	*PHF13*	Homo sapiens PHD finger protein 13 (PHF13), mRNA [NM_153812]	1.4731405	0.04792962
A_24_P271527	*PPARA*	Homo sapiens peroxisome proliferator-activated receptor alpha (PPARA), transcript variant 5, mRNA [NM_005036]	-1.5413387	0.03244441
A_23_P256342	*JOSD1*	Homo sapiens Josephin domain containing 1 (JOSD1), mRNA [NM_014876]	-1.5886377	0.042693015
A_24_P363005	*SNX13*	Homo sapiens sorting nexin 13 (SNX13), mRNA [NM_015132]	-0.49950287	0.033853576
A_24_P242361	*UBE2D3*	Homo sapiens ubiquitin-conjugating enzyme E2D 3 (UBE2D3), transcript variant 2, mRNA [NM_181886]	-1.220966	0.024948223
A_23_P19619	*HS2ST1*	Homo sapiens heparan sulfate 2-O-sulfotransferase 1 (HS2ST1), transcript variant 1, mRNA [NM_012262]	-0.9964287	0.028551029
A_24_P294719	*HIVEP1*	Homo sapiens human immunodeficiency virus type I enhancer binding protein 1 (HIVEP1), mRNA [NM_002114]	-1.1847405	0.030441707
A_24_P43092	*FAF2*	Homo sapiens Fas associated factor family member 2 (FAF2), mRNA [NM_014613]	-1.1302948	0.03668808

A_22_P00004292	*RPL39*	Homo sapiens ribosomal protein L39 (RPL39), mRNA [NM_001000]	-0.76204336	0.042994436
A_23_P164536	*lnc-CNOT6-4*	LNCipedia lincRNA (lnc-CNOT6-4), lincRNA [lnc-CNOT6-4:1]	-0.913136	0.03205687
A_33_P3214874	*PIK3C3*	Homo sapiens phosphatidylinositol 3-kinase, catalytic subunit type 3 (PIK3C3), mRNA [NM_002647]	-1.0391555	0.02598378
A_33_P3416762	*KPNA6*	Homo sapiens karyopherin alpha 6 (importin alpha 7) (KPNA6), mRNA [NM_012316]	-1.6169574	0.03403978
A_23_P419239	*LYPLA1*	Homo sapiens lysophospholipase I (LYPLA1), transcript variant 1, mRNA [NM_006330]	0.6755611	0.027942823
A_24_P376391	*ETNK1*	Homo sapiens ethanolamine kinase 1 (ETNK1), transcript variant 1, mRNA [NM_018638]	-0.7092836	0.03403978
A_19_P00322495	*PLXND1*	Homo sapiens plexin D1 (PLXND1), mRNA [NM_015103]	-0.48738998	0.031578258
A_23_P205997	*TSTD3*	Homo sapiens thiosulfate sulfurtransferase (rhodanese)-like domain containing 3 (TSTD3), mRNA [NM_001195131]	-1.0483634	0.03706845
A_21_P0005656	*APH1B*	Homo sapiens APH1B gamma secretase subunit (APH1B), transcript variant 1, mRNA [NM_031301]	-0.8974722	0.042693015
A_33_P3302681	*LOC100506990*	Homo sapiens uncharacterized LOC100506990 (LOC100506990), transcript variant 2, long non-coding RNA [NR_040092]	-0.8147764	0.027942823
A_23_P324754	*TLE4*	transducin-like enhancer of split 4 [Source:HGNC Symbol;Acc:HGNC:11840] [ENST00000455913]	-1.0053903	0.033392303
A_21_P0005606	*CEMIP*	Homo sapiens cell migration inducing protein, hyaluronan binding (CEMIP), transcript variant 3, mRNA [NM_018689]	-0.74412256	0.04250221
A_23_P304489	*lnc-SBDS-10*	LNCipedia lincRNA (lnc-SBDS-10), lincRNA [lnc-SBDS-10:1]	-1.7330667	0.028573772
A_23_P94118	*FAM19A5*	Homo sapiens family with sequence similarity 19 (chemokine (C-C motif)-like), member A5 (FAM19A5), transcript variant 2, mRNA [NM_015381]	-1.3305504	0.03032242
A_22_P00005677	*GTF2E2*	Homo sapiens general transcription factor IIE, polypeptide 2, beta 34kDa (GTF2E2), mRNA [NM_002095]	-1.476532	0.04976156

A_21_P0000590	*lnc-EIF4EBP1-1*	Homo sapiens, clone IMAGE:4431109, mRNA. [BC015455]	2.9467635	0.03947304
A_33_P3243657	*ZEB2*	Homo sapiens zinc finger E-box binding homeobox 2 (ZEB2), transcript variant 3, non-coding RNA [NR_033258]	-3.6680593	0.02598378
A_22_P00019235	*TIMM10B*	Homo sapiens translocase of inner mitochondrial membrane 10 homolog B (yeast) (TIMM10B), mRNA [NM_012192]	-0.8283706	0.031430684
A_24_P158421	*LOC101559451*	Homo sapiens uncharacterized LOC101559451 (LOC101559451), long non-coding RNA [NR_103482]	-1.301246	0.028573772
A_23_P77073	*SAR1A*	Homo sapiens secretion associated, Ras related GTPase 1A (SAR1A), transcript variant 2, mRNA [NM_020150]	-0.8937503	0.031606767
A_33_P3249877	*SPPL2A*	Homo sapiens signal peptide peptidase like 2A (SPPL2A), mRNA [NM_032802]	-0.36882734	0.028192867
A_23_P36689	*BEST3*	Homo sapiens bestrophin 3 (BEST3), transcript variant 4, mRNA [NM_001282614]	1.8668717	0.03032242
A_23_P374288	*LRRC23*	Homo sapiens leucine rich repeat containing 23 (LRRC23), transcript variant 2, mRNA [NM_006992]	-1.1229451	0.03319862
A_24_P26897	*CASC3*	Homo sapiens cancer susceptibility candidate 3 (CASC3), mRNA [NM_007359]	-0.8629365	0.033723384
A_22_P00013992	*INPP5A*	Homo sapiens inositol polyphosphate-5-phosphatase, 40kDa (INPP5A), mRNA [NM_005539]	2.3383603	0.029088594
A_23_P21425	*lnc-RUNDC3A-1*	AGENCOURT_8824882 NIH_MGC_18 Homo sapiens cDNA clone IMAGE:6423362 5', mRNA sequence [BQ943064]	-0.61534953	0.039287783
A_21_P0000583	*GPR119*	Homo sapiens G protein-coupled receptor 119 (GPR119), mRNA [NM_178471]	-0.6691341	0.04820357
A_32_P61857	*LOC439994*	Homo sapiens uncharacterized LOC439994 (LOC439994), long non-coding RNA [NR_029408]	-1.1277802	0.027751032
A_23_P216568	*KIAA1468*	Homo sapiens KIAA1468 (KIAA1468), mRNA [NM_020854]	1.3695962	0.048238702
A_23_P203137	*FAM206A*	Homo sapiens family with sequence similarity 206, member A (FAM206A), mRNA [NM_017832]	2.1735082	0.031126594
A_33_P3336262	*UBE4A*	Homo sapiens ubiquitination factor E4A (UBE4A), transcript variant 1, mRNA [NM_004788]	-1.0710235	0.03126305

A_23_P34968	*CFAP74*	cilia and flagella associated protein 74 [Source:HGNC Symbol;Acc:HGNC:29368] [ENST00000412120]	-0.97114116	0.048527416
A_23_P98085	*SCNM1*	Homo sapiens sodium channel modifier 1 (SCNM1), transcript variant 2, mRNA [NM_001204856]	-0.5809779	0.04336959
A_23_P201386	*PTEN*	Homo sapiens phosphatase and tensin homolog (PTEN), mRNA [NM_000314]	-0.8077445	0.029808238
A_23_P162734	*DDAH1*	Homo sapiens dimethylarginine dimethylaminohydrolase 1 (DDAH1), transcript variant 1, mRNA [NM_012137]	1.7193091	0.02737376
A_22_P00014982	*RNF6*	Homo sapiens ring finger protein (C3H2C3 type) 6 (RNF6), transcript variant 1, mRNA [NM_005977]	-0.8518464	0.032286324
A_24_P914817	*lnc-SMC1B-1*	LNCipedia lincRNA (lnc-SMC1B-1), lincRNA [lnc-SMC1B-1:1]	-0.8249646	0.03297842
A_23_P104109	*MKLN1*	Homo sapiens muskelin 1, intracellular mediator containing kelch motifs (MKLN1), transcript variant 2, mRNA [NM_013255]	-1.6888695	0.029808238
A_24_P105564	*RPS6KC1*	Homo sapiens ribosomal protein S6 kinase, 52kDa, polypeptide 1 (RPS6KC1), transcript variant 1, mRNA [NM_012424]	-1.0341663	0.02737376
A_23_P37623	*PRKAB2*	Homo sapiens protein kinase, AMP-activated, beta 2 non-catalytic subunit (PRKAB2), transcript variant 1, mRNA [NM_005399]	1.4145643	0.047932126
A_23_P136172	*GOLGA8A*	Homo sapiens golgin A8 family, member A (GOLGA8A), transcript variant 1, mRNA [NM_181077]	-1.1683959	0.04964876
A_33_P3280779	*TMEM68*	Homo sapiens transmembrane protein 68 (TMEM68), transcript variant 2, mRNA [NM_152417]	-0.8485472	0.032456078
A_23_P32217	*TMEM254*	Homo sapiens transmembrane protein 254 (TMEM254), transcript variant 9, mRNA [NM_001270374]	-1.6230412	0.0480697
A_33_P3327702	*TOPORS*	Homo sapiens topoisomerase I binding, arginine/serine-rich, E3 ubiquitin protein ligase (TOPORS), transcript variant 1, mRNA [NM_005802]	-1.2542238	0.048681058
A_33_P3285893	*SYMPK*	Homo sapiens symplekin (SYMPK), mRNA [NM_004819]	-0.96267706	0.03811336

A_24_P378019	*SCRN1*	Homo sapiens secernin 1 (SCRN1), transcript variant 1, mRNA [NM_001145513]	-1.1420811	0.028573772
A_23_P382775	*IRF7*	Homo sapiens interferon regulatory factor 7 (IRF7), transcript variant d, mRNA [NM_004031]	-0.6974895	0.047555834
A_33_P3306267	*BBC3*	Homo sapiens BCL2 binding component 3 (BBC3), transcript variant 4, mRNA [NM_014417]	-0.79063743	0.024948223
A_33_P3314550	*PAX6*	Homo sapiens paired box 6 (PAX6), transcript variant 1, mRNA [NM_000280]	1.1751077	0.04976886
A_33_P3376781	*RAB3A*	Homo sapiens RAB3A, member RAS oncogene family (RAB3A), mRNA [NM_002866]	-1.4920609	0.045006614
A_23_P200216	*TIMP2*	Homo sapiens TIMP metallopeptidase inhibitor 2 (TIMP2), mRNA [NM_003255]	2.427184	0.030915326
A_33_P3374589	*MAGOH*	Homo sapiens mago-nashi homolog, proliferation-associated (Drosophila) (MAGOH), mRNA [NM_002370]	2.5466344	0.03436134
A_32_P228501	*FAM101B*	Homo sapiens family with sequence similarity 101, member B (FAM101B), mRNA [NM_182705]	-1.012002	0.028573772
A_23_P366328	*CDC26*	Homo sapiens cell division cycle 26 (CDC26), mRNA [NM_139286]	-0.4438865	0.03319862
A_19_P00322929	*VPS37A*	Homo sapiens vacuolar protein sorting 37 homolog A (S. cerevisiae) (VPS37A), transcript variant 1, mRNA [NM_152415]	0.8661927	0.032261204
A_33_P3404749	*HTR7P1*	Homo sapiens 5-hydroxytryptamine (serotonin) receptor 7 pseudogene 1 (HTR7P1), non-coding RNA [NR_002774]	-0.8768191	0.024948223
A_33_P3316394	*FMN1*	Homo sapiens formin 1 (FMN1), transcript variant 1, mRNA [NM_001277313]	-1.0931765	0.027751032
A_24_P310756	*ZNF419*	Homo sapiens zinc finger protein 419 (ZNF419), transcript variant 1, mRNA [NM_001098491]	-1.217738	0.031606767
A_33_P3240674	*FBXO28*	Homo sapiens F-box protein 28 (FBXO28), transcript variant 1, mRNA [NM_015176]	0.76512927	0.044364784
A_21_P0010986	*BRD3*	Homo sapiens bromodomain containing 3 (BRD3), mRNA [NM_007371]	-2.3038683	0.03298269

A_23_P30275	*U2AF1*	Homo sapiens U2 small nuclear RNA auxiliary factor 1 (U2AF1), transcript variant c, mRNA [NM_001025204]	-1.3665777	0.036667652
A_33_P3248654	*PCYOX1L*	Homo sapiens prenylcysteine oxidase 1 like (PCYOX1L), transcript variant 1, mRNA [NM_024028]	-0.79074806	0.03794058
A_33_P3311637	*SARS*	Homo sapiens seryl-tRNA synthetase (SARS), transcript variant 1, mRNA [NM_006513]	-1.6547371	0.027751032
A_33_P3243997	*SCFD1*	Homo sapiens sec1 family domain containing 1 (SCFD1), transcript variant 1, mRNA [NM_016106]	-1.2619846	0.036667652
A_23_P215787	*PSME4*	Homo sapiens proteasome (prosome, macropain) activator subunit 4 (PSME4), mRNA [NM_014614]	-1.6986836	0.028056882
A_23_P410613	*HBP1*	Homo sapiens HMG-box transcription factor 1 (HBP1), transcript variant 2, mRNA [NM_012257]	-1.2255887	0.027019119
A_24_P913115	*TMEM263*	Homo sapiens transmembrane protein 263 (TMEM263), mRNA [NM_152261]	-0.81779236	0.04405224
A_24_P108242	*PTEN*	Homo sapiens phosphatase and tensin homolog (PTEN), mRNA [NM_000314]	-0.68027127	0.043983467
A_23_P421221	*GOSR2*	Homo sapiens golgi SNAP receptor complex member 2 (GOSR2), transcript variant A, mRNA [NM_004287]	-1.8502071	0.027751032
A_23_P314191	*R3HCC1*	Homo sapiens R3H domain and coiled-coil containing 1 (R3HCC1), transcript variant 1, mRNA [NM_001136108]	-0.9808967	0.033726536
A_32_P155247	*ZDHHC17*	Homo sapiens zinc finger, DHHC-type containing 17 (ZDHHC17), mRNA [NM_015336]	-0.8067501	0.034999274
A_23_P57059	*FTL*	Homo sapiens ferritin, light polypeptide (FTL), mRNA [NM_000146]	-0.6050034	0.024948223
A_33_P3320368	*STAU1*	Homo sapiens staufen double-stranded RNA binding protein 1 (STAU1), transcript variant T3, mRNA [NM_017453]	-1.1022397	0.039287783
A_24_P43391	*TMEM161A*	Homo sapiens transmembrane protein 161A (TMEM161A), transcript variant 1, mRNA [NM_017814]	-1.2429707	0.033723384
A_23_P396666	*TMEM165*	Homo sapiens transmembrane protein 165 (TMEM165), transcript variant 1, mRNA [NM_018475]	-0.98019475	0.04219537
A_23_P110345	*TBC1D2B*	Homo sapiens TBC1 domain family, member 2B (TBC1D2B), transcript variant 2, mRNA [NM_015079]	-0.49784878	0.046726856

A_23_P356484	*CHIC2*	Homo sapiens cysteine-rich hydrophobic domain 2 (CHIC2), mRNA [NM_012110]	-0.9979484	0.030441707
A_24_P205364	*RPS10*	Homo sapiens ribosomal protein S10 (RPS10), transcript variant 2, mRNA [NM_001014]	-1.5239472	0.048390456
A_24_P219552	*SHMT1*	Homo sapiens serine hydroxymethyltransferase 1 (soluble) (SHMT1), transcript variant 1, mRNA [NM_004169]	-0.7568965	0.03840554
A_22_P00011073	*NFE2L1*	Homo sapiens nuclear factor, erythroid 2-like 1 (NFE2L1), mRNA [NM_003204]	-0.9356494	0.027942823
A_23_P130064	*lnc-NUB1-1*	LNCipedia lincRNA (lnc-NUB1-1), lincRNA [lnc-NUB1-1:4]	-0.9455519	0.04610903
A_23_P98382	*SNF8*	Homo sapiens SNF8, ESCRT-II complex subunit (SNF8), mRNA [NM_007241]	-0.5804462	0.03591672
A_23_P50815	*TIMM8B*	Homo sapiens translocase of inner mitochondrial membrane 8 homolog B (yeast) (TIMM8B), transcript variant 1, mRNA [NM_012459]	-0.59631354	0.030293303
A_33_P3295650	*TTYH1*	Homo sapiens tweety family member 1 (TTYH1), transcript variant 1, mRNA [NM_020659]	0.7473234	0.04831641
A_21_P0013937	*APBA1*	Homo sapiens amyloid beta (A4) precursor protein-binding, family A, member 1 (APBA1), mRNA [NM_001163]	-0.8204901	0.044364784
A_32_P46571	*HAPLN3*	Homo sapiens hyaluronan and proteoglycan link protein 3 (HAPLN3), mRNA [NM_178232]	1.8090798	0.029808238
A_33_P3383756	*RHBDL2*	Homo sapiens rhomboid, veinlet-like 2 (Drosophila) (RHBDL2), mRNA [NM_017821]	2.1861007	0.024948223
A_23_P201432	*WHSC1L1*	Homo sapiens Wolf-Hirschhorn syndrome candidate 1-like 1 (WHSC1L1), transcript variant short, mRNA [NM_017778]	1.2215936	0.03152038
A_33_P3344039	*ADSS*	Homo sapiens adenylosuccinate synthase (ADSS), mRNA [NM_001126]	-2.2548892	0.031526815
A_24_P780052	*RABL2A*	Homo sapiens RAB, member of RAS oncogene family-like 2A (RABL2A), transcript variant 2, mRNA [NM_007082]	-1.3186787	0.035935212
A_33_P3231557	*RPSAP58*	Homo sapiens ribosomal protein SA pseudogene 58 (RPSAP58), non-coding RNA [NR_003662]	-0.7793889	0.032155342

A_23_P59798	*CDC14B*	Homo sapiens cell division cycle 14B (CDC14B), transcript variant 1, mRNA [NM_003671]	-1.3548143	0.04405224
A_23_P406785	*MKRN1*	Homo sapiens makorin ring finger protein 1 (MKRN1), transcript variant 1, mRNA [NM_013446]	-0.61010545	0.03136619
A_32_P208178	*C9orf50*	Homo sapiens chromosome 9 open reading frame 50 (C9orf50), mRNA [NM_199350]	-0.47724342	0.047879543
A_24_P637982	*RPS3A*	Homo sapiens ribosomal protein S3A (RPS3A), transcript variant 1, mRNA [NM_001006]	-1.2887485	0.03590667
A_24_P100673	*C1orf122*	Homo sapiens chromosome 1 open reading frame 122 (C1orf122), transcript variant 1, mRNA [NM_198446]	-0.6530172	0.042693015
A_23_P44932	*EMC4*	Homo sapiens ER membrane protein complex subunit 4 (EMC4), transcript variant 1, mRNA [NM_016454]	-0.9890877	0.032261204
A_22_P00012919	*EIF2A*	Homo sapiens eukaryotic translation initiation factor 2A, 65kDa (EIF2A), mRNA [NM_032025]	-0.4706106	0.03319862
A_22_P00022847	*NMNAT1*	Homo sapiens nicotinamide nucleotide adenylyltransferase 1 (NMNAT1), transcript variant 2, mRNA [NM_001297778]	-0.9458916	0.034210585
A_33_P3318696	*lnc-RNF186-1*	Homo sapiens cDNA: FLJ23530 fis, clone LNG06055. [AK027183]	-0.99454784	0.034578066
A_33_P3358521	*CCBL2*	Homo sapiens cysteine conjugate-beta lyase 2 (CCBL2), transcript variant 1, mRNA [NM_001008661]	-1.4808657	0.024948223
A_33_P3231542	*RIC1*	Homo sapiens RAB6A GEF complex partner 1 (RIC1), transcript variant 1, mRNA [NM_020829]	-1.5220307	0.042994436
A_23_P209426	*ZFHX3*	Homo sapiens zinc finger homeobox 3 (ZFHX3), transcript variant A, mRNA [NM_006885]	1.2815835	0.030915326
A_33_P3325275	*TRAK2*	Homo sapiens trafficking protein, kinesin binding 2 (TRAK2), mRNA [NM_015049]	-1.8904226	0.042459987
A_23_P314145	*NRSN2*	Homo sapiens neurensin 2 (NRSN2), mRNA [NM_024958]	-0.90567446	0.044286337
A_23_P54649	*UST*	Homo sapiens uronyl-2-sulfotransferase (UST), mRNA [NM_005715]	-1.2197502	0.049070477
A_22_P00012060	*TRADD*	Homo sapiens TNFRSF1A-associated via death domain (TRADD), mRNA [NM_003789]	-0.8715308	0.029573848

A_24_P71700	*lnc-PLOD2-1*	LNCipedia lincRNA (lnc-PLOD2-1), lincRNA [lnc-PLOD2-1:1]	1.6755579	0.028573772
A_22_P00003043	*ZBTB47*	Homo sapiens zinc finger and BTB domain containing 47 (ZBTB47), mRNA [NM_145166]	-1.732418	0.031603426
A_33_P3279640	*TLE4*	Homo sapiens transducin-like enhancer of split 4 (TLE4), transcript variant 3, mRNA [NM_007005]	-2.076426	0.03702924
A_33_P3346403	*HCN2*	Homo sapiens hyperpolarization activated cyclic nucleotide gated potassium channel 2 (HCN2), mRNA [NM_001194]	-1.2217901	0.027751032
A_23_P28263	*PTMA*	Homo sapiens prothymosin, alpha (PTMA), transcript variant 1, mRNA [NM_001099285]	1.6366947	0.047708835
A_23_P207927	*CTDSP1*	Homo sapiens CTD (carboxy-terminal domain, RNA polymerase II, polypeptide A) small phosphatase 1 (CTDSP1), transcript variant 1, mRNA [NM_021198]	-1.8160787	0.02737376
A_21_P0000236	*C17orf80*	Homo sapiens chromosome 17 open reading frame 80 (C17orf80), transcript variant 4, mRNA [NM_001288770]	-1.7119999	0.02598378
A_23_P205830	*SNORA64*	Homo sapiens small nucleolar RNA, H/ACA box 64 (SNORA64), small nucleolar RNA [NR_002326]	-1.1386597	0.04820357
A_33_P3371904	*BTBD1*	Homo sapiens BTB (POZ) domain containing 1 (BTBD1), transcript variant 1, mRNA [NM_025238]	-1.0831293	0.027942823
A_23_P372331	*ASTN2*	Homo sapiens astrotactin 2 (ASTN2), transcript variant 4, mRNA [NM_198188]	-1.3013759	0.031377234
A_23_P160618	*TRNT1*	Homo sapiens tRNA nucleotidyl transferase, CCA-adding, 1 (TRNT1), transcript variant 1, mRNA [NM_182916]	-1.0441457	0.047879543
A_23_P26713	*SH2D2A*	Homo sapiens SH2 domain containing 2A (SH2D2A), transcript variant 2, mRNA [NM_003975]	-0.68623567	0.045743782
A_33_P3245709	*RPL23*	Homo sapiens ribosomal protein L23 (RPL23), mRNA [NM_000978]	-1.5651288	0.030971156
A_23_P165698	*RALGAPB*	Homo sapiens Ral GTPase activating protein, beta subunit (non-catalytic) (RALGAPB), transcript variant 1, mRNA [NM_020336]	-0.8456743	0.043024775

A_33_P3237634	*C2orf49*	Homo sapiens chromosome 2 open reading frame 49 (C2orf49), transcript variant 1, mRNA [NM_024093]	2.1500785	0.02453978
A_33_P3371320	*TSC22D3*	Homo sapiens TSC22 domain family, member 3 (TSC22D3), transcript variant 2, mRNA [NM_004089]	-0.431467	0.03403978
A_21_P0011727	*BRE*	Homo sapiens brain and reproductive organ-expressed (TNFRSF1A modulator) (BRE), transcript variant 6, mRNA [NM_001261840]	-0.819587	0.027751032
A_24_P153576	*NUTM2B-AS1*	Homo sapiens NUTM2B antisense RNA 1 (NUTM2B-AS1), transcript variant 3, long non-coding RNA [NR_120613]	-1.9134254	0.03510091
A_33_P3219785	*SHPRH*	Homo sapiens SNF2 histone linker PHD RING helicase, E3 ubiquitin protein ligase (SHPRH), transcript variant 2, mRNA [NM_173082]	-0.75067276	0.02919588
A_23_P144453	*SOWAHC*	Homo sapiens sosondowah ankyrin repeat domain family member C (SOWAHC), mRNA [NM_023016]	2.6554353	0.03668808
A_33_P3294654	*MRFAP1*	Homo sapiens Morf4 family associated protein 1 (MRFAP1), transcript variant 1, mRNA [NM_033296]	-1.3242584	0.031869426
A_24_P918266	*RSG1*	Homo sapiens REM2 and RAB-like small GTPase 1 (RSG1), mRNA [NM_030907]	-0.96479726	0.033583984
A_33_P3353791	*TMTC3*	Homo sapiens transmembrane and tetratricopeptide repeat containing 3 (TMTC3), mRNA [NM_181783]	-1.0302678	0.03770023
A_33_P3344492	*ITGA1*	Homo sapiens integrin, alpha 1 (ITGA1), mRNA [NM_181501]	-0.75035	0.027751032
A_21_P0000019	*SCARNA1*	Homo sapiens small Cajal body-specific RNA 1 (SCARNA1), guide RNA [NR_002997]	-0.8011913	0.029979022
A_23_P30223	*KDM4C*	Homo sapiens lysine (K)-specific demethylase 4C (KDM4C), transcript variant 3, mRNA [NM_001146695]	-1.1443044	0.032155342
A_23_P162879	*SRD5A1*	Homo sapiens steroid-5-alpha-reductase, alpha polypeptide 1 (3-oxo-5 alpha-steroid delta 4-dehydrogenase alpha 1) (SRD5A1), mRNA [NM_001047]	-0.6390528	0.03136619
A_21_P0014801	*APOPT1*	Homo sapiens apoptogenic 1, mitochondrial (APOPT1), transcript variant 1, mRNA [NM_032374]	-0.6813183	0.02737376
A_33_P3270852	*KLHDC4*	kelch domain containing 4 [Source:HGNC	-1.0421265	0.03912857

		Symbol;Acc:HGNC:25272] [ENST00000567298]		
A_23_P216038	*RPS29*	Homo sapiens ribosomal protein S29 (RPS29), transcript variant 1, mRNA [NM_001032]	-0.8695912	0.025407597
A_23_P50137	*PHF20L1*	PHD finger protein 20-like 1 [Source:HGNC Symbol;Acc:HGNC:24280] [ENST00000395383]	-0.8002677	0.034306627
A_24_P309360	*MEX3C*	Homo sapiens mex-3 RNA binding family member C (MEX3C), mRNA [NM_016626]	-1.2743179	0.047233704
A_24_P402898	*TM9SF3*	Homo sapiens transmembrane 9 superfamily member 3 (TM9SF3), mRNA [NM_020123]	-0.83307934	0.032261204
A_24_P365327	*OTUD4*	Homo sapiens OTU deubiquitinase 4 (OTUD4), transcript variant 3, mRNA [NM_001102653]	-1.574934	0.033726536
A_23_P398172	*LSM14A*	Homo sapiens LSM14A, SCD6 homolog A (S. cerevisiae) (LSM14A), transcript variant 2, mRNA [NM_015578]	-1.1493821	0.028546385
A_33_P3311618	*FAM135A*	Homo sapiens family with sequence similarity 135, member A (FAM135A), transcript variant 2, mRNA [NM_020819]	-0.9777079	0.028189372
A_23_P83976	*RABGGTB*	Homo sapiens Rab geranylgeranyltransferase, beta subunit (RABGGTB), transcript variant 1, mRNA [NM_004582]	-1.0786397	0.0415208
A_33_P3240538	*CEP112*	Homo sapiens centrosomal protein 112kDa (CEP112), transcript variant 3, mRNA [NM_001199165]	-0.83120275	0.028546385
A_32_P202759	*TUBE1*	Homo sapiens tubulin, epsilon 1 (TUBE1), mRNA [NM_016262]	-1.5355645	0.042693015
A_23_P254442	*FAM171B*	Homo sapiens family with sequence similarity 171, member B (FAM171B), mRNA [NM_177454]	-1.1873279	0.043971255
A_23_P142322	*LOC155060*	Homo sapiens AI894139 pseudogene (LOC155060), non-coding RNA [NR_036573]	-1.0683293	0.0442831
A_23_P380928	*CIRBP*	Homo sapiens cold inducible RNA binding protein (CIRBP), transcript variant 1, mRNA [NM_001280]	-1.4170747	0.029573848
A_33_P3349045	*ARPC4-TTLL3*	Homo sapiens ARPC4-TTLL3 readthrough (ARPC4-TTLL3), mRNA [NM_001198793]	0.8726351	0.031603426

A_33_P3381245	*IL4R*	Homo sapiens interleukin 4 receptor (IL4R), transcript variant 4, mRNA [NM_001257407]	-1.1029999	0.04945554
A_24_P264664	*PDCD5*	Homo sapiens programmed cell death 5 (PDCD5), mRNA [NM_004708]	-0.8220372	0.041108888
A_23_P214977	*CHD2*	Homo sapiens chromodomain helicase DNA binding protein 2 (CHD2), transcript variant 1, mRNA [NM_001271]	-0.47356728	0.044286337
A_23_P149221	*SEC63*	Homo sapiens SEC63 homolog (S. cerevisiae) (SEC63), mRNA [NM_007214]	1.124527	0.04024844
A_33_P3375665	*HECTD3*	Homo sapiens HECT domain containing E3 ubiquitin protein ligase 3 (HECTD3), mRNA [NM_024602]	-0.42833564	0.031127423
A_33_P3406245	*FOXO3*	Homo sapiens forkhead box O3 (FOXO3), transcript variant 1, mRNA [NM_001455]	-0.9181821	0.03716438
A_24_P416411	*TAF1A*	Homo sapiens TATA box binding protein (TBP)-associated factor, RNA polymerase I, A, 48kDa (TAF1A), transcript variant 1, mRNA [NM_005681]	-1.3410648	0.03136619
A_23_P259189	*PEX12*	Homo sapiens peroxisomal biogenesis factor 12 (PEX12), mRNA [NM_000286]	-0.9330424	0.049758088
A_22_P00013169	*CLIC4*	Homo sapiens chloride intracellular channel 4 (CLIC4), mRNA [NM_013943]	-0.9289195	0.029808238
A_22_P00009296	*lnc-RNF13-2*	AGENCOURT_14302541 NIH_MGC_173 Homo sapiens cDNA 5', mRNA sequence [CD388559]	-0.9467176	0.03338082
A_22_P00000026	*lnc-LRRC36-1*	Homo sapiens PP10897 mRNA, complete cds. [AF370400]	-1.4650903	0.048213527
A_23_P141520	*EMX2OS*	Homo sapiens EMX2 opposite strand/antisense RNA (EMX2OS), long non-coding RNA [NR_002791]	-0.9400127	0.037431587
A_33_P3524912	*C17orf49*	Homo sapiens chromosome 17 open reading frame 49 (C17orf49), transcript variant 2, mRNA [NM_174893]	2.540513	0.027942823
A_24_P362540	*LOC283038*	Homo sapiens uncharacterized LOC283038 (LOC283038), long non-coding RNA [NR_033848]	2.0531578	0.027942823
A_33_P3235454	*ASAP2*	Homo sapiens ArfGAP with SH3 domain, ankyrin repeat and PH domain 2 (ASAP2), transcript variant 1, mRNA [NM_003887]	-2.1657274	0.027942823

A_33_P3215059	*LIMD1-AS1*	Homo sapiens LIMD1 antisense RNA 1 (LIMD1-AS1), long non-coding RNA [NR_033947]	1.1227896	0.024948223
A_22_P00021971	*NSMCE1*	Homo sapiens non-SMC element 1 homolog (S. cerevisiae) (NSMCE1), mRNA [NM_145080]	-1.2452223	0.03800715
A_32_P389118	*lnc-EIF4E3-1*	Homo sapiens cDNA FLJ12191 fis, clone MAMMA1000843. [AK022253]	1.5313742	0.03808641
A_24_P245815	*HEATR5B*	Homo sapiens HEAT repeat containing 5B (HEATR5B), mRNA [NM_019024]	1.5114746	0.044286337
A_23_P8664	*ASPHD2*	Homo sapiens aspartate beta-hydroxylase domain containing 2 (ASPHD2), mRNA [NM_020437]	-0.9918663	0.029808238
A_23_P134953	*DMTF1*	Homo sapiens cyclin D binding myb-like transcription factor 1 (DMTF1), transcript variant 1, mRNA [NM_021145]	-3.4234388	0.029573848
A_23_P145463	*PLIN2*	Homo sapiens perilipin 2 (PLIN2), transcript variant 1, mRNA [NM_001122]	-0.65860885	0.03808641
A_19_P00805679	*SLC35B3*	Homo sapiens solute carrier family 35 (adenosine 3'-phospho 5'-phosphosulfate transporter), member B3 (SLC35B3), transcript variant 1, mRNA [NM_015948]	-2.2171712	0.03272157
A_33_P3389153	*LOC730102*	Homo sapiens quinone oxidoreductase-like protein 2 pseudogene (LOC730102), non-coding RNA [NR_037167]	-0.7702388	0.03403978
A_23_P5568	*STK10*	Homo sapiens serine/threonine kinase 10 (STK10), mRNA [NM_005990]	-0.974736	0.042656552
A_33_P3418766	*SFT2D3*	Homo sapiens SFT2 domain containing 3 (SFT2D3), mRNA [NM_032740]	-1.544948	0.03811336
A_33_P3215739	*ZNF532*	Homo sapiens zinc finger protein 532 (ZNF532), mRNA [NM_018181]	2.0131378	0.030438775
A_22_P00018829	*LNP1*	Homo sapiens leukemia NUP98 fusion partner 1 (LNP1), mRNA [NM_001085451]	-1.3074448	0.02453978
A_23_P73747	*LOC102724571*	PREDICTED: Homo sapiens uncharacterized LOC102724571 (LOC102724571), transcript variant X3, misc_RNA [XR_426651]	-0.9424729	0.028573772
A_23_P69720	*ARMCX2*	Homo sapiens armadillo repeat containing, X-linked 2 (ARMCX2), transcript variant 2, mRNA [NM_014782]	-0.9871645	0.03933512
A_33_P3260100	*ANXA5*	Homo sapiens annexin A5 (ANXA5), mRNA [NM_001154]	1.1128231	0.04971531

A_33_P3330149	*CCDC167*	Homo sapiens coiled-coil domain containing 167 (CCDC167), mRNA [NM_138493]	-1.6270427	0.030589065
A_33_P3292387	*PAX6*	Homo sapiens paired box 6 (PAX6), transcript variant 1, mRNA [NM_000280]	-0.88464946	0.03032242
A_23_P10497	*LRRIQ3*	Homo sapiens leucine-rich repeats and IQ motif containing 3 (LRRIQ3), mRNA [NM_001105659]	-1.3354263	0.04792962
A_21_P0004312	*ATP9B*	Homo sapiens ATPase, class II, type 9B (ATP9B), mRNA [NM_198531]	-0.95210284	0.040612318
A_33_P3402773	*MIR143HG*	MIR143 host gene (non-protein coding) [Source:HGNC Symbol;Acc:HGNC:42872] [ENST00000519898]	2.181802	0.036542445
A_23_P122852	*RPL7A*	Homo sapiens ribosomal protein L7a (RPL7A), mRNA [NM_000972]	-0.64972156	0.048541743
A_23_P202071	*SMARCD3*	Homo sapiens SWI/SNF related, matrix associated, actin dependent regulator of chromatin, subfamily d, member 3 (SMARCD3), transcript variant 2, mRNA [NM_003078]	-2.597348	0.027751032
A_23_P152305	*CELF2*	Homo sapiens CUGBP, Elav-like family member 2 (CELF2), transcript variant 3, mRNA [NM_001025077]	1.4350766	0.027942823
A_33_P3416124	*CDH11*	Homo sapiens cadherin 11, type 2, OB-cadherin (osteoblast) (CDH11), mRNA [NM_001797]	-0.6957145	0.036057316
A_33_P6570838	*LOC100130872*	Homo sapiens uncharacterized LOC100130872 (LOC100130872), long non-coding RNA [NR_024569]	-0.6618333	0.040669635
A_24_P185604	*MPV17L*	MPV17 mitochondrial membrane protein-like [Source:HGNC Symbol;Acc:HGNC:26827] [ENST00000287594]	-1.5054848	0.028573772
A_23_P93464	*NDRG3*	Homo sapiens NDRG family member 3 (NDRG3), transcript variant 1, mRNA [NM_032013]	-1.1567731	0.049704313
A_22_P00001341	*BCKDHB*	Homo sapiens branched chain keto acid dehydrogenase E1, beta polypeptide (BCKDHB), transcript variant 2, mRNA [NM_000056]	1.7033985	0.02737376
A_33_P3394933	*MIR612*	Homo sapiens microRNA 612 (MIR612), microRNA [NR_030343]	1.2315409	0.031394158
A_19_P00321259	*P4HA2*	Homo sapiens prolyl 4-hydroxylase, alpha polypeptide II (P4HA2), transcript variant 2, mRNA [NM_001017973]	-0.94721806	0.029979022

A_19_P00804777	*LINC01133*	Homo sapiens long intergenic non-protein coding RNA 1133 (LINC01133), long non-coding RNA [NR_038849]	-1.5130308	0.033583984
A_22_P00020897	*LINC00662*	long intergenic non-protein coding RNA 662 [Source:HGNC Symbol;Acc:HGNC:27122] [ENST00000586954]	1.0526097	0.04831641
A_23_P162322	*lnc-RPP38-2*	Homo sapiens mRNA; cDNA DKFZp761F0115 (from clone DKFZp761F0115). [CR749680]	-1.4339128	0.02737376
A_23_P342668	*WNT10B*	Homo sapiens wingless-type MMTV integration site family, member 10B (WNT10B), mRNA [NM_003394]	-2.588562	0.032261204
A_33_P3288871	*AKAP17A*	Homo sapiens A kinase (PRKA) anchor protein 17A (AKAP17A), transcript variant 1, mRNA [NM_005088]	-1.6122599	0.024948223
A_23_P96965	*PPP1R3B*	Homo sapiens protein phosphatase 1, regulatory subunit 3B (PPP1R3B), transcript variant 1, mRNA [NM_001201329]	1.4189618	0.030915326
A_23_P36183	*SYNC*	Homo sapiens syncoilin, intermediate filament protein (SYNC), transcript variant 1, mRNA [NM_030786]	-0.27192262	0.04462891
A_23_P35256	*GTF2H1*	Homo sapiens general transcription factor IIH, polypeptide 1, 62kDa (GTF2H1), transcript variant 1, mRNA [NM_005316]	2.5761983	0.028573772
A_33_P3365596	*SDSL*	Homo sapiens serine dehydratase-like (SDSL), mRNA [NM_138432]	-1.2389443	0.044141345
A_24_P141214	*NAPB*	Homo sapiens N-ethylmaleimide-sensitive factor attachment protein, beta (NAPB), transcript variant 1, mRNA [NM_001283018]	-1.009774	0.031603426
A_23_P257609	*STOM*	Homo sapiens stomatin (STOM), transcript variant 2, mRNA [NM_198194]	-1.1255193	0.036667652
A_33_P3412695	*RPL29*	Homo sapiens ribosomal protein L29 (RPL29), mRNA [NM_000992]	-1.0977212	0.027751032
A_33_P3343305	*RUNX3*	Homo sapiens runt-related transcription factor 3 (RUNX3), transcript variant 1, mRNA [NM_001031680]	-1.6551387	0.03136619
A_33_P3271126	*NUTM2B*	Homo sapiens NUT family member 2B (NUTM2B), mRNA [NM_001278495]	2.2815845	0.029994998
A_21_P0007761	*DDX60L*	Homo sapiens DEAD (Asp-Glu-Ala-Asp) box polypeptide 60-like (DDX60L), transcript variant 1, mRNA [NM_001012967]	1.9805547	0.02598378

A_23_P135616	*MAPKAPK5-AS1*	Homo sapiens MAPKAPK5 antisense RNA 1 (MAPKAPK5-AS1), long non-coding RNA [NR_015404]	-1.1947569	0.036345065
A_33_P3289561	*STX18*	Homo sapiens syntaxin 18 (STX18), mRNA [NM_016930]	-1.7387526	0.04642224
A_33_P3329108	*SLC25A3P1*	Homo sapiens solute carrier family 25 (mitochondrial carrier; phosphate carrier), member 3 pseudogene 1 (SLC25A3P1), non-coding RNA [NR_002314]	-0.9009901	0.04531519
A_24_P356	*MTAP*	Homo sapiens methylthioadenosine phosphorylase (MTAP), mRNA [NM_002451]	-1.0684272	0.028551029
A_22_P00014248	*AAK1*	AP2 associated kinase 1 [Source:HGNC Symbol;Acc:HGNC:19679] [ENST00000406297]	-1.3443137	0.034210585
A_24_P98555	*FBXO15*	Homo sapiens F-box protein 15 (FBXO15), transcript variant 1, mRNA [NM_152676]	1.2490377	0.048317578
A_24_P101101	*FAM45A*	Homo sapiens family with sequence similarity 45, member A (FAM45A), transcript variant 2, mRNA [NM_001303111]	0.6717342	0.0376762
A_24_P296907	*AKR1C3*	Homo sapiens aldo-keto reductase family 1, member C3 (AKR1C3), transcript variant 1, mRNA [NM_003739]	1.0592335	0.03804464
A_33_P3332492	*THAP8*	Homo sapiens THAP domain containing 8 (THAP8), mRNA [NM_152658]	-1.0841899	0.0442831
A_33_P3216442	*FANK1*	Homo sapiens fibronectin type III and ankyrin repeat domains 1 (FANK1), mRNA [NM_145235]	-1.1827705	0.02737376
A_33_P3239849	*COL11A2*	Homo sapiens collagen, type XI, alpha 2 (COL11A2), transcript variant 1, mRNA [NM_080680]	-0.7387893	0.036857422
A_33_P3283064	*GPX1*	Homo sapiens glutathione peroxidase 1 (GPX1), transcript variant 2, mRNA [NM_201397]	-1.5440251	0.03452508
A_23_P125078	*RHBDL2*	Homo sapiens rhomboid, veinlet-like 2 (Drosophila) (RHBDL2), mRNA [NM_017821]	-1.056164	0.024948223
A_23_P6836	*SLC26A11*	Homo sapiens solute carrier family 26 (anion exchanger), member 11 (SLC26A11), transcript variant 2, mRNA [NM_173626]	-1.1490352	0.02737376
A_24_P121846	*IP6K2*	Homo sapiens inositol hexakisphosphate kinase 2 (IP6K2),	1.7920558	0.02453978

		transcript variant 1, mRNA [NM_016291]		
A_33_P3356216	*ZNF132*	Homo sapiens zinc finger protein 132 (ZNF132), mRNA [NM_003433]	-1.0987617	0.030915326
A_33_P3224780	*ARCN1*	Homo sapiens archain 1 (ARCN1), transcript variant 1, mRNA [NM_001655]	-0.7181182	0.02598378
A_33_P3344399	*SCGB2B2*	Homo sapiens secretoglobin, family 2B, member 2 (SCGB2B2), mRNA [NM_001025591]	-1.6725508	0.037095673
A_22_P00010050	*LRRC49*	Homo sapiens leucine rich repeat containing 49 (LRRC49), transcript variant 3, mRNA [NM_001199018]	-0.81079644	0.03889491
A_23_P23839	*ADNP-AS1*	Homo sapiens ADNP antisense RNA 1 (ADNP-AS1), transcript variant 2, long non-coding RNA [NR_110008]	0.85103506	0.049327243
A_23_P102037	*LGR6*	Homo sapiens leucine-rich repeat containing G protein-coupled receptor 6 (LGR6), transcript variant 1, mRNA [NM_001017403]	1.138071	0.042570952
A_23_P321201	*COQ10B*	Homo sapiens coenzyme Q10 homolog B (S. cerevisiae) (COQ10B), mRNA [NM_025147]	-1.1729854	0.03136619
A_24_P232790	*DENND5A*	Homo sapiens DENN/MADD domain containing 5A (DENND5A), transcript variant 1, mRNA [NM_015213]	-0.8096332	0.024948223
A_23_P59616	*CCDC177*	Homo sapiens coiled-coil domain containing 177 (CCDC177), mRNA [NM_001271507]	2.2004168	0.033888694
A_33_P3422679	*GTF2IRD2*	Homo sapiens GTF2I repeat domain containing 2 (GTF2IRD2), transcript variant 1, mRNA [NM_173537]	-0.67876333	0.030915326
A_22_P00014756	*FAM149B1*	Homo sapiens family with sequence similarity 149, member B1 (FAM149B1), mRNA [NM_173348]	-0.9423237	0.028192867
A_33_P3339051	*LOC101060091*	Homo sapiens uncharacterized LOC101060091 (LOC101060091), long non-coding RNA [NR_110174]	2.2791696	0.03403978
A_33_P3378545	*ZNF428*	Homo sapiens zinc finger protein 428 (ZNF428), mRNA [NM_182498]	-1.5982577	0.028573772
A_23_P104996	*lnc-DIO2-3*	DB092099 TESTI4 Homo sapiens cDNA clone TESTI4041634 5', mRNA sequence [DB092099]	-1.6552781	0.042440254
A_21_P0005463	*BEST1*	Homo sapiens bestrophin 1 (BEST1), transcript variant 1, mRNA [NM_004183]	-1.919042	0.043485392
A_23_P77965	*lnc-COX19-1*	LNCipedia lincRNA (lnc-COX19-1), lincRNA [lnc-COX19-1:1]	-0.71822244	0.028573772

A_24_P233915	*HEATR6*	Homo sapiens HEAT repeat containing 6 (HEATR6), mRNA [NM_022070]	2.7198386	0.027019119
A_23_P208009	*MED8*	Homo sapiens mediator complex subunit 8 (MED8), transcript variant 4, mRNA [NM_001001653]	-1.6600108	0.028573772
A_22_P00011687	*SEC11C*	Homo sapiens SEC11 homolog C (S. cerevisiae) (SEC11C), mRNA [NM_033280]	1.8428288	0.04173021
A_23_P416581	*LOC339807*	Homo sapiens uncharacterized LOC339807 (LOC339807), long non-coding RNA [NR_034023]	-1.2593148	0.036311693
A_22_P00012594	*GNAZ*	Homo sapiens guanine nucleotide binding protein (G protein), alpha z polypeptide (GNAZ), mRNA [NM_002073]	-0.758938	0.028192867
A_24_P135902	*SNUPN*	Homo sapiens snurportin 1 (SNUPN), transcript variant 1, mRNA [NM_005701]	2.1036344	0.03590667
A_23_P254081	*RPS2*	Homo sapiens ribosomal protein S2 (RPS2), mRNA [NM_002952]	1.6302631	0.036667652
A_23_P35609	*LIAS*	Homo sapiens lipoic acid synthetase (LIAS), transcript variant 1, mRNA [NM_006859]	2.478366	0.030362261
A_23_P133095	*UROS*	Homo sapiens uroporphyrinogen III synthase (UROS), mRNA [NM_000375]	-1.2824391	0.03338082
A_33_P3352980	*RAPGEF2*	Homo sapiens Rap guanine nucleotide exchange factor (GEF) 2 (RAPGEF2), mRNA [NM_014247]	-1.0892026	0.03601631
A_23_P254863	*HIST1H4K*	Homo sapiens histone cluster 1, H4k (HIST1H4K), mRNA [NM_003541]	-0.3533649	0.042970333
A_23_P415006	*NIM1K*	Homo sapiens NIM1 serine/threonine protein kinase (NIM1K), transcript variant 1, mRNA [NM_153361]	-0.5208928	0.035935212
A_33_P3308153	*RAB11FIP5*	Homo sapiens RAB11 family interacting protein 5 (class I) (RAB11FIP5), mRNA [NM_015470]	1.4533479	0.03811336
A_23_P47426	*ADA*	Homo sapiens adenosine deaminase (ADA), mRNA [NM_000022]	-1.7572927	0.04950784
A_23_P157607	*ACAD8*	Homo sapiens acyl-CoA dehydrogenase family, member 8 (ACAD8), mRNA [NM_014384]	-0.9907377	0.03403978
A_23_P59637	*INTS10*	Homo sapiens integrator complex subunit 10 (INTS10), mRNA [NM_018142]	1.306989	0.028573772
A_23_P141893	*DOCK4*	Homo sapiens dedicator of cytokinesis 4 (DOCK4), mRNA [NM_014705]	-1.7232385	0.03138659

A_33_P3209356	*PPM1N*	Homo sapiens protein phosphatase, Mg2+/Mn2+ dependent, 1N (putative) (PPM1N), mRNA [NM_001080401]	-1.4839532	0.034210585
A_23_P330788	*RFWD2*	Homo sapiens ring finger and WD repeat domain 2, E3 ubiquitin protein ligase (RFWD2), transcript variant 1, mRNA [NM_022457]	-0.91506815	0.03136619
A_21_P0010135	*IQSEC2*	Homo sapiens IQ motif and Sec7 domain 2 (IQSEC2), transcript variant 2, mRNA [NM_015075]	1.0518739	0.046384484
A_24_P40594	*LSM1*	Homo sapiens LSM1, U6 small nuclear RNA associated (LSM1), transcript variant 1, mRNA [NM_014462]	-1.0976527	0.032345347
A_21_P0007924	*HOMEZ*	Homo sapiens homeobox and leucine zipper encoding (HOMEZ), mRNA [NM_020834]	0.57995933	0.043911606
A_23_P216689	*lnc-SGCG-1*	LNCipedia lincRNA (lnc-SGCG-1), lincRNA [lnc-SGCG-1:1]	1.020518	0.039287783
A_21_P0004621	*BRD3*	Homo sapiens bromodomain containing 3 (BRD3), mRNA [NM_007371]	-0.69141626	0.029573848
A_19_P00804950	*BCL11A*	Homo sapiens B-cell CLL/lymphoma 11A (zinc finger protein) (BCL11A), transcript variant 2, mRNA [NM_018014]	3.031857	0.0370712
A_23_P97365	*lnc-AC114947.1.1-2*	ALU1_HUMAN (P39188) Alu subfamily J sequence contamination warning entry, partial (13%) [THC2658268]	-0.7880897	0.027942823
A_23_P213754	*LRRC8D*	Homo sapiens leucine rich repeat containing 8 family, member D (LRRC8D), transcript variant 2, mRNA [NM_018103]	1.494302	0.04041675
A_21_P0003809	*PAIP2*	Homo sapiens poly(A) binding protein interacting protein 2 (PAIP2), transcript variant 1, mRNA [NM_001033112]	-2.3847394	0.027751032
A_22_P00023057	*lnc-CLDN24-2*	LNCipedia lincRNA (lnc-CLDN24-2), lincRNA [lnc-CLDN24-2:3]	-1.1831207	0.030293303
A_22_P00012938	*IL13RA1*	Homo sapiens interleukin 13 receptor, alpha 1 (IL13RA1), mRNA [NM_001560]	-1.4021546	0.044286337
A_33_P3394405	*lnc-RCSD1-1*	Q4BPG8_BURVI (Q4BPG8) DNA-directed DNA polymerase , partial (3%) [THC2658218]	-1.576765	0.03621218
A_33_P3317317	*LOC727721*	DB238770 TRACH3 Homo sapiens cDNA clone TRACH3034794 5', mRNA sequence [DB238770]	1.2116269	0.031603426

A_33_P3356075	*KRTAP5-4*	Homo sapiens keratin associated protein 5-4 (KRTAP5-4), mRNA [NM_001012709]	-0.8898735	0.046624914
A_33_P3312754	*LPIN1*	Homo sapiens lipin 1 (LPIN1), transcript variant 4, mRNA [NM_001261429]	0.76818687	0.031578258
A_23_P109420	*LOC102467146*	Homo sapiens uncharacterized LOC102467146 (LOC102467146), long non-coding RNA [NR_104663]	1.6813636	0.027942823
A_23_P17074	*BMS1*	Homo sapiens BMS1 ribosome biogenesis factor (BMS1), mRNA [NM_014753]	1.906207	0.024948223
A_33_P3364112	*CCDC115*	Homo sapiens coiled-coil domain containing 115 (CCDC115), transcript variant 1, mRNA [NM_032357]	0.8394454	0.044286337
A_33_P3273409	*FRS2*	Homo sapiens fibroblast growth factor receptor substrate 2 (FRS2), transcript variant 3, mRNA [NM_001278351]	2.0566337	0.027019119
A_24_P414332	*SLC35E1*	Homo sapiens solute carrier family 35, member E1 (SLC35E1), mRNA [NM_024881]	-0.56098604	0.049427208
A_23_P129486	*AFF1*	Homo sapiens AF4/FMR2 family, member 1 (AFF1), transcript variant 2, mRNA [NM_005935]	-0.73510695	0.044286337
A_32_P45168	*MSRB1*	Homo sapiens methionine sulfoxide reductase B1 (MSRB1), mRNA [NM_016332]	-0.6880674	0.030441707
A_22_P00000102	*IL6ST*	Homo sapiens interleukin 6 signal transducer (IL6ST), transcript variant 1, mRNA [NM_002184]	-0.7932177	0.042850092
A_23_P149099	*SNHG6*	Homo sapiens small nucleolar RNA host gene 6 (non-protein coding) (SNHG6), long non-coding RNA [NR_002599]	-20002403	0.028573772
A_33_P3259028	*DDOST*	Homo sapiens dolichyl-diphosphooligosaccharide--protein glycosyltransferase subunit (non-catalytic) (DDOST), mRNA [NM_005216]	-0.65988517	0.027942823
A_33_P3258320	*TRIM24*	tripartite motif containing 24 [Source:HGNC Symbol;Acc:HGNC:11812] [ENST00000343526]	-0.3998959	0.035560552
A_24_P317874	*ZNF562*	zinc finger protein 562 [Source:HGNC Symbol;Acc:HGNC:25950] [ENST00000585350]	-1.6621599	0.04041675

A_23_P55376	*KIN*	Homo sapiens Kin17 DNA and RNA binding protein (KIN), transcript variant 1, mRNA [NM_012311]	-0.8683769	0.02737376
A_23_P130149	*KANSL1*	Homo sapiens KAT8 regulatory NSL complex subunit 1 (KANSL1), transcript variant 2, mRNA [NM_015443]	-0.60900736	0.029642703
A_23_P113283	*ENO3*	Homo sapiens enolase 3 (beta, muscle) (ENO3), transcript variant 1, mRNA [NM_001976]	1.3215187	0.02598378
A_24_P687594	*ZMAT3*	Homo sapiens zinc finger, matrin-type 3 (ZMAT3), transcript variant 1, mRNA [NM_022470]	-0.78861976	0.03403978
A_33_P3399101	*LIX1L*	Homo sapiens Lix1 homolog (chicken) like (LIX1L), mRNA [NM_153713]	-0.4256444	0.045638688
A_33_P3318564	*CTSL*	cathepsin L [Source:HGNC Symbol;Acc:HGNC:2537] [ENST00000342020]	-1.3298887	0.038727812
A_32_P205110	*lnc-LIN28B-1*	Q5T552_HUMAN (Q5T552) OTTHUMP00000016913, complete [THC2740889]	-1.0837051	0.027942823
A_33_P3212092	*FOXC1*	Homo sapiens forkhead box C1 (FOXC1), mRNA [NM_001453]	-0.96730876	0.031606767
A_22_P00003291	*PDCD4*	Homo sapiens programmed cell death 4 (neoplastic transformation inhibitor) (PDCD4), transcript variant 2, mRNA [NM_145341]	0.96388763	0.042621672
A_24_P277349	*NDST2*	Homo sapiens N-deacetylase/N-sulfotransferase (heparan glucosaminyl) 2 (NDST2), mRNA [NM_003635]	1.0893824	0.042693015
A_21_P0000152	*SEC31A*	Homo sapiens SEC31 homolog A (S. cerevisiae) (SEC31A), transcript variant 5, mRNA [NM_001077207]	-2.266687	0.037923194
A_23_P360626	*CD44*	Homo sapiens CD44 molecule (Indian blood group) (CD44), transcript variant 8, mRNA [NM_001202557]	-0.9660503	0.031430684
A_33_P3318911	*PLD6*	Homo sapiens phospholipase D family, member 6 (PLD6), mRNA [NM_178836]	-0.6041881	0.038727812
A_23_P115785	*INPP5K*	Homo sapiens inositol polyphosphate-5-phosphatase K (INPP5K), transcript variant 3, mRNA [NM_001135642]	-0.89312315	0.028573772
A_24_P7157	*FANK1*	Homo sapiens fibronectin type III and ankyrin repeat domains 1 (FANK1), mRNA [NM_145235]	2.1724446	0.04368986
A_19_P00322941	*RIMKLB*	Homo sapiens ribosomal modification protein rimK-like family member B	0.8885215	0.04792962

		(RIMKLB), transcript variant 1, mRNA [NM_020734]		
A_21_P0007412	*MIR143HG*	MIR143 host gene (non-protein coding) [Source:HGNC Symbol;Acc:HGNC:42872] [ENST00000518014]	-0.78071356	0.04610903
A_23_P100455	*lnc-BLID-1*	LNCipedia lincRNA (lnc-BLID-1), lincRNA [lnc-BLID-1:18]	-1.4037186	0.04164901
A_23_P430902	*MTHFSD*	Homo sapiens methenyltetrahydrofolate synthetase domain containing (MTHFSD), transcript variant 3, mRNA [NM_022764]	-1.8773229	0.034334898
A_23_P393425	*MORN4*	Homo sapiens MORN repeat containing 4 (MORN4), transcript variant 1, mRNA [NM_178832]	-4.6306467	0.033412892
A_23_P93792	*PAPD4*	Homo sapiens PAP associated domain containing 4 (PAPD4), transcript variant 3, mRNA [NM_173797]	-1.7562617	0.03912857
A_24_P925062	*PMS2*	Homo sapiens PMS2 postmeiotic segregation increased 2 (S. cerevisiae) (PMS2), transcript variant 1, mRNA [NM_000535]	-1.7022289	0.04348676
A_21_P0012461	*MXRA7*	Homo sapiens matrix-remodelling associated 7 (MXRA7), transcript variant 1, mRNA [NM_001008528]	-1.3113856	0.03138659
A_23_P58588	*XLOC_l2_010348*	BROAD Institute lincRNA (XLOC_l2_010348), lincRNA [TCONS_l2_00019530]	-1.436636	0.027942823
A_32_P187663	*SLIT3*	Homo sapiens slit homolog 3 (Drosophila) (SLIT3), transcript variant 2, mRNA [NM_003062]	-1.2302052	0.031965
A_23_P15727	*ZNF596*	Homo sapiens zinc finger protein 596 (ZNF596), transcript variant 1, mRNA [NM_001042416]	-1.1965439	0.034210585
A_33_P3296303	*FKBP10*	Homo sapiens FK506 binding protein 10, 65 kDa (FKBP10), mRNA [NM_021939]	-0.98345196	0.029808238
A_21_P0009377	*CHMP5*	Homo sapiens charged multivesicular body protein 5 (CHMP5), transcript variant 1, mRNA [NM_016410]	-2.752685	0.029808238
A_33_P3220833	*lnc-NLGN2-1*	LNCipedia lincRNA (lnc-NLGN2-1), lincRNA [lnc-NLGN2-1:2]	-1.8655103	0.030915326
A_33_P3270034	*PAK2*	Homo sapiens p21 protein (Cdc42/Rac)-activated kinase 2 (PAK2), mRNA [NM_002577]	-1.1819124	0.03912857

A_32_P179676	*NCOA4*	Homo sapiens nuclear receptor coactivator 4 (NCOA4), transcript variant 1, mRNA [NM_001145260]	-1.37917	0.034960467
A_33_P3854030	*TOB2*	Homo sapiens transducer of ERBB2, 2 (TOB2), mRNA [NM_016272]	1.2746749	0.040555127
A_33_P3386716	*MBD5*	Homo sapiens methyl-CpG binding domain protein 5 (MBD5), mRNA [NM_018328]	-1.4167199	0.03609871
A_23_P361584	*PPP1R15B*	Homo sapiens protein phosphatase 1, regulatory subunit 15B (PPP1R15B), mRNA [NM_032833]	-0.7718086	0.047727138
A_33_P3262665	*TMEM154*	Homo sapiens transmembrane protein 154 (TMEM154), mRNA [NM_152680]	-1.0870819	0.03297842
A_33_P3318122	*MAP7D3*	Homo sapiens MAP7 domain containing 3 (MAP7D3), transcript variant 1, mRNA [NM_024597]	-1.294104	0.027751032
A_33_P3384543	*C16orf62*	Homo sapiens chromosome 16 open reading frame 62 (C16orf62), transcript variant 1, mRNA [NM_020314]	-0.90088725	0.037741717
A_33_P3331346	*POMGNT1*	Homo sapiens protein O-linked mannose N-acetylglucosaminyltransferase 1 (beta 1,2-) (POMGNT1), transcript variant 2, mRNA [NM_001243766]	-1.7588933	0.02453978
A_23_P208850	*TRIM65*	Homo sapiens tripartite motif containing 65 (TRIM65), transcript variant 1, mRNA [NM_173547]	-1.3621153	0.048506733
A_32_P130265	*RPS16*	Homo sapiens ribosomal protein S16 (RPS16), mRNA [NM_001020]	-0.9425037	0.034293942
A_33_P3292851	*ZC3H11A*	Homo sapiens zinc finger CCCH-type containing 11A (ZC3H11A), mRNA [NM_014827]	-0.32614347	0.04842329
A_23_P408675	*UBE2I*	PREDICTED: Homo sapiens ubiquitin-conjugating enzyme E2I (UBE2I), transcript variant X3, mRNA [XM_006720938]	-0.49763662	0.02598378
A_33_P3685216	*BTAF1*	Homo sapiens BTAF1 RNA polymerase II, B-TFIID transcription factor-associated, 170kDa (BTAF1), mRNA [NM_003972]	-0.567786	0.024948223
A_23_P359870	*A1BG*	Homo sapiens alpha-1-B glycoprotein (A1BG), mRNA [NM_130786]	-1.0416348	0.02453978
A_32_P46214	*CLIC4*	Homo sapiens chloride intracellular channel 4 (CLIC4), mRNA [NM_013943]	-1.5211592	0.02453978
A_33_P7796746	*SLC9A9*	Homo sapiens solute carrier family 9, subfamily A (NHE9, cation proton	-0.59548306	0.036129165

		antiporter 9), member 9 (SLC9A9), mRNA [NM_173653]		
A_24_P415327	*TONSL*	Homo sapiens tonsoku-like, DNA repair protein (TONSL), mRNA [NM_013432]	1.3200363	0.049383525
A_21_P0005513	*HPS1*	Homo sapiens Hermansky-Pudlak syndrome 1 (HPS1), transcript variant 1, mRNA [NM_000195]	-1.6277404	0.04947425
A_23_P120472	*lnc-SGCE-1*	LNCipedia lincRNA (lnc-SGCE-1), lincRNA [lnc-SGCE-1:2]	-2.92319	0.035186797
A_33_P3342295	*TFAP2C*	Homo sapiens transcription factor AP-2 gamma (activating enhancer binding protein 2 gamma) (TFAP2C), mRNA [NM_003222]	-0.89269924	0.031569958
A_33_P3335257	*VSX1*	Homo sapiens visual system homeobox 1 (VSX1), transcript variant 2, mRNA [NM_199425]	-0.643284	0.035207514
A_19_P00810806	*CATSPERD*	Homo sapiens catsper channel auxiliary subunit delta (CATSPERD), mRNA [NM_152784]	-0.48163193	0.028573772
A_24_P29686	*SMIM2-AS1*	Homo sapiens SMIM2 antisense RNA 1 (SMIM2-AS1), transcript variant 1, long non-coding RNA [NR_104064]	1.2104733	0.049418923
A_23_P124044	*ZMIZ2*	Homo sapiens zinc finger, MIZ-type containing 2 (ZMIZ2), transcript variant 1, mRNA [NM_031449]	-1.8776381	0.039899252
A_24_P314351	*DEAF1*	Homo sapiens DEAF1 transcription factor (DEAF1), transcript variant 1, mRNA [NM_021008]	1.5730848	0.034210585
A_24_P64233	*ZBTB33*	Homo sapiens zinc finger and BTB domain containing 33 (ZBTB33), transcript variant 2, mRNA [NM_006777]	1.6219044	0.037464894
A_33_P3364904	*ALDH1B1*	Homo sapiens aldehyde dehydrogenase 1 family, member B1 (ALDH1B1), mRNA [NM_000692]	-1.2222956	0.030578624
A_24_P368735	*ZNF662*	Homo sapiens zinc finger protein 662 (ZNF662), transcript variant 1, mRNA [NM_207404]	-0.44259122	0.046325136
A_23_P45945	*ARL10*	Homo sapiens ADP-ribosylation factor-like 10 (ARL10), mRNA [NM_173664]	-1.0499688	0.034306627
A_23_P361841	*EIF1*	Homo sapiens eukaryotic translation initiation factor 1 (EIF1), mRNA [NM_005801]	-2.1741714	0.044286337
A_24_P173746	*RIMKLB*	Homo sapiens ribosomal modification protein rimK-like family member B (RIMKLB), transcript variant 1, mRNA [NM_020734]	-1.4317006	0.048398294

A_23_P33093	*RALGPS2*	Homo sapiens Ral GEF with PH domain and SH3 binding motif 2 (RALGPS2), transcript variant 10, mRNA [NM_152663]	-0.7206672	0.03794058
A_33_P3231242	*ST6GALNAC5*	Homo sapiens ST6 (alpha-N-acetyl-neuraminyl-2,3-beta-galactosyl-1,3)-N-acetylgalactosaminide alpha-2,6-sialyltransferase 5 (ST6GALNAC5), mRNA [NM_030965]	-0.65647644	0.029573848
A_33_P3244828	*SEC11A*	Homo sapiens SEC11 homolog A (S. cerevisiae) (SEC11A), transcript variant 2, mRNA [NM_014300]	-1.0047194	0.031377234
A_23_P253029	*RPL6*	Homo sapiens ribosomal protein L6 (RPL6), transcript variant 1, mRNA [NM_001024662]	-0.71048516	0.036427937
A_32_P9842	*BOK*	Homo sapiens BCL2-related ovarian killer (BOK), mRNA [NM_032515]	-0.66136265	0.03338082
A_23_P348264	*MTMR14*	Homo sapiens myotubularin related protein 14 (MTMR14), transcript variant 1, mRNA [NM_001077526]	1.6415606	0.024948223
A_22_P00017373	*LETM2*	Homo sapiens leucine zipper-EF-hand containing transmembrane protein 2 (LETM2), transcript variant 2, mRNA [NM_144652]	-0.8709178	0.032261204
A_22_P00015763	*lnc-UTS2-1*	603205994F1 NIH_MGC_97 Homo sapiens cDNA clone IMAGE:5271719 5', mRNA sequence [BI464920]	-1.0864301	0.049418923
A_21_P0009964	*ZNF547*	zinc finger protein 547 [Source:HGNC Symbol;Acc:HGNC:26432] [ENST00000282282]	2.7597334	0.03452508
A_23_P104509	*lnc-NCOA3-10*	DKFZp686M2229_r1 686 (synonym: hlcc3) Homo sapiens cDNA clone DKFZp686M2229 5', mRNA sequence [BX489122]	-1.7212591	0.03434428
A_21_P0003497	*FAM53B*	Homo sapiens family with sequence similarity 53, member B (FAM53B), mRNA [NM_014661]	0.48207095	0.03601631
A_23_P201636	*LINC01365*	long intergenic non-protein coding RNA 1365 [Source:HGNC Symbol;Acc:HGNC:50603] [ENST00000512219]	-1.5046465	0.028573772
A_24_P307289	*LAMC2*	Homo sapiens laminin, gamma 2 (LAMC2), transcript variant 1, mRNA [NM_005562]	0.79554266	0.045322645
A_23_P252283	*TMEM95*	Homo sapiens transmembrane protein 95 (TMEM95), mRNA [NM_198154]	1.0671369	0.049327243

A_32_P30831	*RNF135*	Homo sapiens ring finger protein 135 (RNF135), transcript variant 1, mRNA [NM_032322]	1.6697936	0.04864667
A_23_P254756	*RAD51-AS1*	Homo sapiens RAD51 antisense RNA 1 (head to head) (RAD51-AS1), long non-coding RNA [NR_040058]	-2.8481653	0.03770023
A_23_P106798	*CD164*	Homo sapiens CD164 molecule, sialomucin (CD164), transcript variant 1, mRNA [NM_006016]	2.823608	0.045638688
A_23_P350895	*SIAH1*	Homo sapiens siah E3 ubiquitin protein ligase 1 (SIAH1), transcript variant 1, mRNA [NM_003031]	-0.4533994	0.031303514
A_23_P33407	*MEFV*	Homo sapiens Mediterranean fever (MEFV), transcript variant 1, mRNA [NM_000243]	-0.82487303	0.04591315
A_33_P3283669	*HERC2*	Homo sapiens HECT and RLD domain containing E3 ubiquitin protein ligase 2 (HERC2), mRNA [NM_004667]	-1.2317822	0.049758088
A_33_P3389286	*ATP1A3*	Homo sapiens ATPase, Na+/K+ transporting, alpha 3 polypeptide (ATP1A3), transcript variant 3, mRNA [NM_001256214]	-1.370114	0.03233256
A_23_P10815	*SFN*	Homo sapiens stratifin (SFN), mRNA [NM_006142]	2.5351858	0.024948223
A_22_P00012038	*PUM1*	Homo sapiens pumilio RNA-binding family member 1 (PUM1), transcript variant 1, mRNA [NM_001020658]	-1.1727967	0.04080239
A_24_P363679	*MTURN*	Homo sapiens maturin, neural progenitor differentiation regulator homolog (Xenopus) (MTURN), mRNA [NM_152793]	1.4525095	0.03136619
A_23_P8281	*ATP6V1H*	Homo sapiens ATPase, H+ transporting, lysosomal 50/57kDa, V1 subunit H (ATP6V1H), transcript variant 1, mRNA [NM_015941]	6.6479416	0.026808027
A_23_P139396	*IFNGR1*	Homo sapiens interferon gamma receptor 1 (IFNGR1), mRNA [NM_000416]	-0.7018206	0.036044724
A_23_P144746	*C11orf73*	Homo sapiens chromosome 11 open reading frame 73 (C11orf73), transcript variant 1, mRNA [NM_016401]	-2.128181	0.028573772
A_33_P3282836	*ZNF454*	Homo sapiens zinc finger protein 454 (ZNF454), transcript variant 2, mRNA [NM_182594]	-1.4750911	0.028573772
A_24_P392690	*RPS28*	Homo sapiens ribosomal protein S28 (RPS28), mRNA [NM_001031]	-1.7674205	0.03220128

A_23_P39814	*FAM117A*	Homo sapiens family with sequence similarity 117, member A (FAM117A), mRNA [NM_030802]	-0.9872186	0.033546355
A_33_P3290800	*CIR1*	Homo sapiens corepressor interacting with RBPJ, 1 (CIR1), mRNA [NM_004882]	-0.4473114	0.04092333
A_24_P297098	*DDX26B*	Homo sapiens DEAD/H (Asp-Glu-Ala-Asp/His) box polypeptide 26B (DDX26B), mRNA [NM_182540]	-0.98029727	0.03708757
A_33_P3231653	*PHF20*	Homo sapiens PHD finger protein 20 (PHF20), mRNA [NM_016436]	-1.4210721	0.033412892
A_23_P95050	*GOLM1*	Homo sapiens golgi membrane protein 1 (GOLM1), transcript variant 1, mRNA [NM_016548]	-0.87816805	0.041254643
A_33_P3230264	*ARFIP1*	Homo sapiens ADP-ribosylation factor interacting protein 1 (ARFIP1), transcript variant 1, mRNA [NM_001025595]	-1.2677383	0.04971531
A_33_P3365666	*GPC3*	Homo sapiens glypican 3 (GPC3), transcript variant 1, mRNA [NM_001164617]	-0.8164253	0.027751032
A_23_P310582	*LOC100130654*	Homo sapiens cDNA FLJ37924 fis, clone CTONG2000218. [AK095243]	-2.0128922	0.03338082
A_33_P3248329	*ST7L*	Homo sapiens suppression of tumorigenicity 7 like (ST7L), transcript variant 4, mRNA [NM_138729]	2.6060507	0.03136619
A_33_P3354683	*ZFAND3*	Homo sapiens zinc finger, AN1-type domain 3 (ZFAND3), mRNA [NM_021943]	2.806858	0.024948223
A_23_P133058	*PIGS*	Homo sapiens phosphatidylinositol glycan anchor biosynthesis, class S (PIGS), mRNA [NM_033198]	1.1938826	0.032893
A_33_P3215803	*MRFAP1L1*	Homo sapiens Morf4 family associated protein 1-like 1 (MRFAP1L1), mRNA [NM_203462]	-0.5771485	0.047266874
A_23_P27332	*EMR1*	Homo sapiens egf-like module containing, mucin-like, hormone receptor-like 1 (EMR1), transcript variant 1, mRNA [NM_001974]	-0.8857305	0.03504642
A_23_P202594	*TCF4*	Homo sapiens transcription factor 4 (TCF4), transcript variant 2, mRNA [NM_003199]	-0.84569937	0.04881034
A_24_P356453	*MCMBP*	Homo sapiens minichromosome maintenance complex binding protein (MCMBP), transcript variant 1, mRNA [NM_024834]	-1.0571555	0.028573772

A_24_P311926	*COA3*	Homo sapiens cytochrome c oxidase assembly factor 3 (COA3), mRNA [NM_001040431]	0.8425594	0.040555127
A_23_P210608	*HLA-G*	Homo sapiens major histocompatibility complex, class I, G (HLA-G), mRNA [NM_002127]	-1.3097771	0.03136619
A_23_P146512	*ZNF217*	Homo sapiens zinc finger protein 217 (ZNF217), mRNA [NM_006526]	-0.64440775	0.037560765
A_23_P79836	*GOLM1*	Homo sapiens golgi membrane protein 1 (GOLM1), transcript variant 1, mRNA [NM_016548]	-0.6770489	0.03887779
A_23_P97990	*SERINC3*	Homo sapiens serine incorporator 3 (SERINC3), transcript variant 1, mRNA [NM_006811]	-1.3205066	0.049603622
A_23_P112666	*HTRA1*	Homo sapiens HtrA serine peptidase 1 (HTRA1), mRNA [NM_002775]	-1.3844807	0.040555127
A_24_P410017	*FOXE3*	Homo sapiens forkhead box E3 (FOXE3), mRNA [NM_012186]	-1.2860227	0.040218875
A_24_P49687	*POTEI*	Homo sapiens POTE ankyrin domain family, member I (POTEI), mRNA [NM_001277406]	-0.7224912	0.032261204
A_33_P3350508	*CD274*	Homo sapiens CD274 molecule (CD274), transcript variant 1, mRNA [NM_014143]	-0.7031975	0.03138659
A_23_P148600	*FTO*	Homo sapiens fat mass and obesity associated (FTO), mRNA [NM_001080432]	-0.74841684	0.028573772
A_23_P368896	*INE1*	Homo sapiens inactivation escape 1 (non-protein coding) (INE1), long non-coding RNA [NR_024616]	2.8854694	0.02737376
A_23_P34144	*SNX12*	Homo sapiens sorting nexin 12 (SNX12), transcript variant 2, mRNA [NM_013346]	3.2310853	0.034825742
A_33_P3244753	*MAGEH1*	Homo sapiens melanoma antigen family H, 1 (MAGEH1), mRNA [NM_014061]	-0.6767759	0.03829215
A_33_P3229161	*DRP2*	Homo sapiens dystrophin related protein 2 (DRP2), transcript variant 1, mRNA [NM_001939]	-1.7697198	0.03403978
A_23_P212213	*UTP14C*	Homo sapiens UTP14, U3 small nucleolar ribonucleoprotein, homolog C (yeast) (UTP14C), mRNA [NM_021645]	-0.8797739	0.031526815
A_33_P3402500	*THUMPD3*	Homo sapiens THUMP domain containing 3 (THUMPD3), transcript variant 1, mRNA [NM_015453]	-0.5717468	0.038287252

A_33_P3244181	*TMEM14C*	Homo sapiens transmembrane protein 14C (TMEM14C), transcript variant 1, mRNA [NM_001165258]	1.4234782	0.027942823
A_33_P3363016	*HSBP1*	Homo sapiens heat shock factor binding protein 1 (HSBP1), mRNA [NM_001537]	-0.74015397	0.024948223
A_24_P70888	*BRF1*	Homo sapiens BRF1, RNA polymerase III transcription initiation factor 90 kDa subunit (BRF1), transcript variant 8, mRNA [NM_001242790]	-0.78180844	0.03947304
A_24_P808522	*PLXNB2*	Homo sapiens plexin B2 (PLXNB2), mRNA [NM_012401]	-1.0061661	0.043665398
A_21_P0004807	*RPS14*	Homo sapiens ribosomal protein S14 (RPS14), transcript variant 3, mRNA [NM_005617]	-0.9463324	0.029088594
A_22_P00017915	*LOC101929484*	PREDICTED: Homo sapiens uncharacterized LOC101929484 (LOC101929484), ncRNA [XR_245642]	1.0653613	0.03136619
A_33_P3273732	*ZNF883*	Homo sapiens zinc finger protein 883 (ZNF883), mRNA [NM_001101338]	-1.0260878	0.029573848
A_22_P00017446	*lnc-BRD3-1*	LNCipedia lincRNA (lnc-BRD3-1), lincRNA [lnc-BRD3-1:2]	-0.9352314	0.04462891
A_32_P157481	*lnc-VPS4A-1*	df23h09.y1 Morton Fetal Cochlea Homo sapiens cDNA clone IMAGE:2484352 5', mRNA sequence [AW021463]	-2.4254131	0.043712083
A_33_P3375668	*ERCC4*	Homo sapiens excision repair cross-complementation group 4 (ERCC4), mRNA [NM_005236]	1.4213214	0.03590667
A_33_P3355371	*ZFP90*	Homo sapiens ZFP90 zinc finger protein (ZFP90), mRNA [NM_133458]	3.012382	0.024948223
A_22_P00016429	*TTC9C*	tetratricopeptide repeat domain 9C [Source:HGNC Symbol;Acc:HGNC:28432] [ENST00000294161]	-1.107987	0.047996294
A_21_P0011109	*HIF1A-AS1*	Homo sapiens HIF1A antisense RNA 1 (HIF1A-AS1), long non-coding RNA [NR_047116]	-0.872591	0.02453978
A_33_P3314974	*RPS27*	Homo sapiens ribosomal protein S27 (RPS27), mRNA [NM_001030]	-0.48024338	0.03601631
A_33_P3415321	*PARD6G-AS1*	Homo sapiens PARD6G antisense RNA 1 (PARD6G-AS1), transcript variant 1, long non-coding RNA [NR_028339]	-1.3423198	0.044286337
A_24_P238744	*ST7-OT4*	Homo sapiens ST7 overlapping transcript 4 (ST7-OT4), long non-coding RNA [NR_002329]	-2.3723319	0.03800859

A_33_P3318796	*POTEM*	Homo sapiens POTE ankyrin domain family, member M (POTEM), mRNA [NM_001145442]	-1.3856262	0.04855821
A_33_P3215128	*FSTL3*	Homo sapiens follistatin-like 3 (secreted glycoprotein) (FSTL3), mRNA [NM_005860]	1.1606112	0.03702924
A_23_P110941	*IBA57*	Homo sapiens IBA57, iron-sulfur cluster assembly homolog (S. cerevisiae) (IBA57), mRNA [NM_001010867]	1.4079628	0.042850092
A_23_P153562	*GSTA4*	Homo sapiens glutathione S-transferase alpha 4 (GSTA4), mRNA [NM_001512]	-1.0493296	0.024948223
A_32_P86739	*C5AR1*	Homo sapiens complement component 5a receptor 1 (C5AR1), mRNA [NM_001736]	-1.8045454	0.034614928
A_23_P44760	*CASC10*	Homo sapiens cancer susceptibility candidate 10 (CASC10), mRNA [NM_001010911]	-1.5014246	0.02598378
A_23_P423197	*SETD2*	Homo sapiens SET domain containing 2 (SETD2), mRNA [NM_014159]	-1.7271497	0.034210585
A_33_P3327454	*RXRA*	Homo sapiens retinoid X receptor, alpha (RXRA), transcript variant 1, mRNA [NM_002957]	0.91843414	0.048715595
A_23_P25121	*RSPRY1*	Homo sapiens ring finger and SPRY domain containing 1 (RSPRY1), mRNA [NM_133368]	-1.1547893	0.04733213
A_23_P70398	*FKBP11*	Homo sapiens FK506 binding protein 11, 19 kDa (FKBP11), transcript variant 1, mRNA [NM_016594]	-1.1636416	0.0267966
A_19_P00319465	*VEGFA*	Homo sapiens vascular endothelial growth factor A (VEGFA), transcript variant 6, mRNA [NM_001025370]	-1.2613788	0.02724032
A_23_P68628	*MAVS*	Homo sapiens mitochondrial antiviral signaling protein (MAVS), transcript variant 1, mRNA [NM_020746]	-0.90108323	0.042799078
A_23_P395585	*NECAB3*	Homo sapiens N-terminal EF-hand calcium binding protein 3 (NECAB3), transcript variant 2, mRNA [NM_031232]	-1.564332	0.024948223
A_23_P3355	*C3orf22*	chromosome 3 open reading frame 22 [Source:HGNC Symbol;Acc:HGNC:28534] [ENST00000318225]	-1.0667498	0.028573772
A_33_P3210909	*POLG*	Homo sapiens polymerase (DNA directed), gamma (POLG), transcript variant 1, mRNA [NM_002693]	-0.7655106	0.027751032

A_23_P214354	*SEC13*	Homo sapiens SEC13 homolog (S. cerevisiae) (SEC13), transcript variant 5, mRNA [NM_001278946]	0.8761965	0.043670435
A_33_P3225278	*EXOC2*	Homo sapiens exocyst complex component 2 (EXOC2), transcript variant 1, mRNA [NM_018303]	0.58148324	0.04445317
A_21_P0011573	*ZNF18*	Homo sapiens zinc finger protein 18 (ZNF18), transcript variant 1, mRNA [NM_144680]	-1.0787241	0.03403978
A_24_P70993	*SLC34A3*	Homo sapiens solute carrier family 34 (type II sodium/phosphate cotransporter), member 3 (SLC34A3), transcript variant 2, mRNA [NM_001177317]	1.453181	0.028321572
A_23_P300781	*CD99*	Homo sapiens CD99 molecule (CD99), transcript variant 1, mRNA [NM_002414]	-1.1421459	0.028573772
A_22_P00024993	*CNOT4*	Homo sapiens CCR4-NOT transcription complex, subunit 4 (CNOT4), transcript variant 1, mRNA [NM_013316]	1.2778577	0.03136619
A_22_P00016609	*LOC102724231*	long intergenic non-protein coding RNA 694 [Source:HGNC Symbol;Acc:HGNC:44570] [ENST00000416124]	1.0914733	0.024948223
A_23_P47614	*lnc-TOLLIP-1*	LNCipedia lincRNA (lnc-TOLLIP-1), lincRNA [lnc-TOLLIP-1:1]	-0.8920981	0.031091278
A_23_P202565	*PHLDA2*	Homo sapiens pleckstrin homology-like domain, family A, member 2 (PHLDA2), mRNA [NM_003311]	-1.0797501	0.03138659
A_24_P142024	*SHOC2*	Homo sapiens soc-2 suppressor of clear homolog (C. elegans) (SHOC2), transcript variant 1, mRNA [NM_007373]	3.4499152	0.031377234
A_32_P229132	*CHMP4A*	Homo sapiens charged multivesicular body protein 4A (CHMP4A), mRNA [NM_014169]	1.1514242	0.030385435
A_23_P369899	*FMN2*	Homo sapiens formin 2 (FMN2), mRNA [NM_020066]	2.220687	0.04458629
A_23_P37560	*TMEM158*	Homo sapiens transmembrane protein 158 (gene/pseudogene) (TMEM158), mRNA [NM_015444]	-1.239109	0.046384484
A_24_P89426	*PEX11A*	Homo sapiens peroxisomal biogenesis factor 11 alpha (PEX11A), transcript variant 1, mRNA [NM_003847]	-0.9631917	0.033433396
A_24_P218074	*APOM*	Homo sapiens apolipoprotein M (APOM), transcript variant 1, mRNA [NM_019101]	-1.2094138	0.042639446

A_33_P3224675	*ZNF467*	Homo sapiens zinc finger protein 467 (ZNF467), mRNA [NM_207336]	3.2565916	0.048183426
A_33_P3395146	*TRMT13*	tRNA methyltransferase 13 homolog (S. cerevisiae) [Source:HGNC Symbol;Acc:HGNC:25502] [ENST00000370139]	-0.9177104	0.029573848
A_33_P3378126	*LOC646762*	Homo sapiens uncharacterized LOC646762 (LOC646762), long non-coding RNA [NR_024278]	-1.0361155	0.024948223
A_33_P3389363	*FBXO32*	Homo sapiens F-box protein 32 (FBXO32), transcript variant 1, mRNA [NM_058229]	-2.3336215	0.03403978
A_23_P98345	*C19orf54*	chromosome 19 open reading frame 54 [Source:HGNC Symbol;Acc:HGNC:24758] [ENST00000597507]	-1.7169106	0.04383085
A_23_P60591	*SNX19*	Homo sapiens sorting nexin 19 (SNX19), transcript variant 1, mRNA [NM_014758]	-0.91536856	0.04639083
A_33_P3269803	*DNAJC7*	Homo sapiens DnaJ (Hsp40) homolog, subfamily C, member 7 (DNAJC7), transcript variant 1, mRNA [NM_003315]	-3.9855123	0.04319268
A_22_P00018123	*CLSTN3*	Homo sapiens calsyntenin 3 (CLSTN3), mRNA [NM_014718]	1.1894596	0.049776267
A_24_P149124	*lnc-ZNF484-1*	hm04h10.x1 NCI_CGAP_Thy8 Homo sapiens cDNA clone IMAGE:3011683 3' similar to contains Alu repetitive element;contains element LTR7 repetitive element ;, mRNA sequence [AW873248]	-0.9683518	0.034210585
A_24_P55465	*NREP*	Homo sapiens neuronal regeneration related protein (NREP), transcript variant 1, mRNA [NM_004772]	-0.8361883	0.028192867
A_33_P3332690	*MTPN*	Homo sapiens myotrophin (MTPN), mRNA [NM_145808]	-0.6220576	0.040555127
A_21_P0013986	*SUZ12P1*	Homo sapiens suppressor of zeste 12 homolog pseudogene, mRNA (cDNA clone IMAGE:5736798). [BC047718]	-1.4912739	0.03510091
A_23_P97457	*DPY30*	Homo sapiens dpy-30 homolog (C. elegans) (DPY30), mRNA [NM_032574]	-1.1136882	0.046416454
A_23_P128624	*AIDA*	Homo sapiens axin interactor, dorsalization associated (AIDA), mRNA [NM_022831]	-0.9663074	0.047218688
A_33_P3214179	*CARS2*	Homo sapiens cysteinyl-tRNA synthetase 2, mitochondrial (putative) (CARS2), mRNA [NM_024537]	-1.5112113	0.035769645

A_22_P00000222	*KIZ*	Homo sapiens kizuna centrosomal protein (KIZ), transcript variant 1, mRNA [NM_018474]	-0.97342163	0.02453978
A_33_P3252781	*LOC100506585*	Homo sapiens uncharacterized LOC100506585 (LOC100506585), long non-coding RNA [NR_038966]	-1.1455938	0.03338082
A_19_P00322354	*PLAC9*	Homo sapiens placenta-specific 9 (PLAC9), mRNA [NM_001012973]	0.820115	0.042965095
A_23_P148297	*MAGI2-AS3*	Homo sapiens MAGI2 antisense RNA 3 (MAGI2-AS3), transcript variant 1, long non-coding RNA [NR_038343]	-1.3187333	0.033853576
A_33_P3228315	*SH3BGRL*	Homo sapiens SH3 domain binding glutamate-rich protein like (SH3BGRL), mRNA [NM_003022]	1.1870916	0.04590026
A_24_P827037	*CARD14*	Homo sapiens caspase recruitment domain family, member 14 (CARD14), transcript variant 2, mRNA [NM_052819]	-3.340938	0.02453978
A_23_P3819	*LRRC15*	Homo sapiens leucine rich repeat containing 15 (LRRC15), transcript variant 2, mRNA [NM_130830]	1.3285339	0.03974276
A_33_P3339036	*ZNF747*	Homo sapiens zinc finger protein 747 (ZNF747), mRNA [NM_023931]	-1.06076	0.049413078
A_23_P86216	*MECP2*	Homo sapiens methyl CpG binding protein 2 (MECP2), transcript variant 2, mRNA [NM_001110792]	1.7003977	0.028573772
A_33_P3289976	*PSMA5*	Homo sapiens proteasome (prosome, macropain) subunit, alpha type, 5 (PSMA5), transcript variant 1, mRNA [NM_002790]	-2.7827504	0.027942823
A_22_P00025334	*lnc-TRMT2B-1*	Homo sapiens cDNA FLJ34403 fis, clone HCHON2001607. [AK091722]	-0.9006373	0.04273615
A_21_P0011387	*LINC01119*	Homo sapiens long intergenic non-protein coding RNA 1119 (LINC01119), long non-coding RNA [NR_024452]	-0.7817228	0.047266874
A_23_P159775	*GPR89B*	Homo sapiens G protein-coupled receptor 89B (GPR89B), mRNA [NM_016334]	1.4865773	0.035207514
A_33_P3332112	*GABRE*	Homo sapiens gamma-aminobutyric acid (GABA) A receptor, epsilon (GABRE), mRNA [NM_004961]	-1.0557452	0.036667652
A_33_P3231297	*FAS*	Homo sapiens Fas cell surface death receptor (FAS), transcript variant 1, mRNA [NM_000043]	-0.63380146	0.034210585
A_23_P65262	*CREG1*	Homo sapiens cellular repressor of E1A-stimulated genes 1 (CREG1), mRNA [NM_003851]	-1.0528828	0.04663971

A_33_P3407065	*N4BP2L2*	Homo sapiens NEDD4 binding protein 2-like 2 (N4BP2L2), transcript variant 3, mRNA [NM_001278432]	-1.32025	0.03233256
A_21_P0011142	*KIF2A*	Homo sapiens kinesin heavy chain member 2A (KIF2A), transcript variant 3, mRNA [NM_001243952]	-2.3147135	0.025423726
A_24_P162287	*NAP1L1*	Homo sapiens nucleosome assembly protein 1-like 1 (NAP1L1), transcript variant 1, mRNA [NM_139207]	-1.5648677	0.024948223
A_22_P00001148	*CEP250*	Homo sapiens centrosomal protein 250kDa (CEP250), mRNA [NM_007186]	-1.265218	0.02598378
A_33_P3271323	*lnc-AMIGO1-1*	LNCipedia lincRNA (lnc-AMIGO1-1), lincRNA [lnc-AMIGO1-1:1]	-1.1864957	0.044419996
A_24_P273203	*RHOQ*	Homo sapiens ras homolog family member Q (RHOQ), mRNA [NM_012249]	-1.0933914	0.048398294
A_23_P66563	*STK19*	Homo sapiens serine/threonine kinase 19 (STK19), transcript variant 2, mRNA [NM_032454]	-0.9963901	0.03912857
A_23_P14876	*SMG8*	Homo sapiens SMG8 nonsense mediated mRNA decay factor (SMG8), mRNA [NM_018149]	-1.1866355	0.028573772
A_24_P290013	*SRP14*	Homo sapiens signal recognition particle 14kDa (homologous Alu RNA binding protein) (SRP14), transcript variant 1, mRNA [NM_003134]	0.84418267	0.0438955
A_24_P24790	*FAM86B3P*	Homo sapiens family with sequence similarity 86, member B3, pseudogene (FAM86B3P), transcript variant 3, non-coding RNA [NR_024363]	-1.3751544	0.034750238
A_23_P160481	*DCAF13*	Homo sapiens DDB1 and CUL4 associated factor 13 (DCAF13), mRNA [NM_015420]	-0.69452715	0.030915326
A_23_P86470	*DCAF6*	Homo sapiens DDB1 and CUL4 associated factor 6 (DCAF6), transcript variant 1, mRNA [NM_018442]	-1.3543689	0.02737376
A_32_P210642	*CH25H*	Homo sapiens cholesterol 25-hydroxylase (CH25H), mRNA [NM_003956]	-0.67364645	0.04411239
A_33_P3411985	*EGFL7*	Homo sapiens EGF-like-domain, multiple 7 (EGFL7), transcript variant 2, mRNA [NM_201446]	-1.0153439	0.044286337
A_24_P218587	*KANSL3*	Homo sapiens KAT8 regulatory NSL complex subunit 3 (KANSL3), transcript variant 1, mRNA [NM_001115016]	-4.213048	0.026467344
A_33_P3217347	*MED17*	Homo sapiens mediator complex subunit 17 (MED17), mRNA [NM_004268]	-2.0836213	0.033583984

A_23_P63929	*PJA1*	Homo sapiens praja ring finger 1, E3 ubiquitin protein ligase (PJA1), transcript variant 1, mRNA [NM_145119]	1.414707	0.042440254
A_23_P411814	*WDR11*	Homo sapiens WD repeat domain 11 (WDR11), mRNA [NM_018117]	-1.0346429	0.029808238
A_23_P333063	*OSTC*	Homo sapiens oligosaccharyltransferase complex subunit (non-catalytic) (OSTC), transcript variant 1, mRNA [NM_021227]	-0.96217084	0.030915326
A_23_P104413	*SMARCE1*	Homo sapiens SWI/SNF related, matrix associated, actin dependent regulator of chromatin, subfamily e, member 1 (SMARCE1), mRNA [NM_003079]	-0.91281223	0.04792962
A_22_P00010163	*DUX4*	Homo sapiens double homeobox 4 (DUX4), mRNA [NM_001293798]	-1.45348	0.031569958
A_23_P168898	*lnc-MRPS17-1*	Homo sapiens cDNA FLJ12163 fis, clone MAMMA1000594. [AK022225]	-1.6382539	0.02737376
A_21_P0001989	*RPS20*	Homo sapiens ribosomal protein S20 (RPS20), transcript variant 2, mRNA [NM_001023]	-1.5555683	0.029642703
A_23_P165061	*lnc-TMEM18-11*	LNCipedia lincRNA (lnc-TMEM18-11), lincRNA [lnc-TMEM18-11:1]	-0.6128265	0.044186465
A_24_P272352	*AES*	Homo sapiens amino-terminal enhancer of split (AES), transcript variant 1, mRNA [NM_198969]	-0.2624198	0.047879543
A_23_P75083	*CATSPERG*	Homo sapiens catsper channel auxiliary subunit gamma (CATSPERG), mRNA [NM_021185]	1.3042103	0.036131933
A_21_P0000163	*ZMYND11*	Homo sapiens zinc finger, MYND-type containing 11 (ZMYND11), transcript variant 2, mRNA [NM_212479]	-0.8757582	0.027751032
A_23_P129801	*RNF223*	Homo sapiens ring finger protein 223 (RNF223), mRNA [NM_001205252]	-0.95514745	0.030896025
A_23_P2431	*RAB40B*	Homo sapiens RAB40B, member RAS oncogene family (RAB40B), mRNA [NM_006822]	1.8658013	0.024948223
A_33_P3464555	*C3AR1*	Homo sapiens complement component 3a receptor 1 (C3AR1), mRNA [NM_004054]	-0.7491963	0.03403978
A_24_P201879	*CAMK1D*	calcium/calmodulin-dependent protein kinase ID [Source:HGNC Symbol;Acc:HGNC:19341] [ENST00000619168]	1.6284475	0.033095628
A_23_P151368	*UTP14C*	Homo sapiens UTP14, U3 small nucleolar ribonucleoprotein, homolog	-0.78941894	0.027942823

		C (yeast) (UTP14C), mRNA [NM_021645]		
A_32_P98732	*N6AMT2*	Homo sapiens N-6 adenine-specific DNA methyltransferase 2 (putative) (N6AMT2), mRNA [NM_174928]	3.2672048	0.031377234
A_21_P0008580	*GCM1*	Homo sapiens glial cells missing homolog 1 (Drosophila) (GCM1), mRNA [NM_003643]	2.3657691	0.041254643
A_33_P3220919	*LOC101929151*	PREDICTED: Homo sapiens uncharacterized LOC101929151 (LOC101929151), ncRNA [XR_243177]	-0.9055244	0.028573772
A_21_P0008224	*ADRBK2*	Homo sapiens adrenergic, beta, receptor kinase 2 (ADRBK2), mRNA [NM_005160]	-0.5294969	0.029808238
A_23_P29036	*XLOC_l2_006025*	BROAD Institute lincRNA (XLOC_l2_006025), lincRNA [TCONS_l2_00011143]	0.98827344	0.03977008
A_23_P397376	*IFNGR2*	Homo sapiens interferon gamma receptor 2 (interferon gamma transducer 1) (IFNGR2), mRNA [NM_005534]	-1.8330271	0.035679508
A_23_P58002	*MAF*	Homo sapiens v-maf avian musculoaponeurotic fibrosarcoma oncogene homolog (MAF), transcript variant 2, mRNA [NM_001031804]	-1.0479951	0.0402021
A_32_P69166	*TCTA*	Homo sapiens T-cell leukemia translocation altered (TCTA), mRNA [NM_022171]	2.9203372	0.028573772
A_23_P155556	*ANKRD42*	Homo sapiens ankyrin repeat domain 42 (ANKRD42), transcript variant 4, mRNA [NM_182603]	-0.93717957	0.040922645
A_33_P3273444	*CLDND1*	Homo sapiens claudin domain containing 1 (CLDND1), transcript variant 6, mRNA [NM_001040199]	-0.7723927	0.04971531
A_21_P0000032	*SCHIP1*	Homo sapiens schwannomin interacting protein 1 (SCHIP1), transcript variant 1, mRNA [NM_014575]	-1.6128689	0.042210005
A_32_P226567	*KIRREL3*	Homo sapiens kin of IRRE like 3 (Drosophila) (KIRREL3), transcript variant 2, mRNA [NM_001161707]	-0.8640887	0.036667652
A_23_P141389	*UPF3A*	Homo sapiens UPF3 regulator of nonsense transcripts homolog A (yeast) (UPF3A), transcript variant 1, mRNA [NM_023011]	-0.9555812	0.03887779
A_24_P921477	*RPL27*	Homo sapiens ribosomal protein L27 (RPL27), mRNA [NM_000988]	-1.6268353	0.034810174

A_33_P3295870	*METTL23*	Homo sapiens methyltransferase like 23 (METTL23), transcript variant 6, mRNA [NM_001206987]	-1.7228993	0.027019119
A_33_P3234641	*FAM166A*	Homo sapiens family with sequence similarity 166, member A (FAM166A), mRNA [NM_001001710]	-1.107282	0.0376762
A_24_P144620	*ACP1*	Homo sapiens acid phosphatase 1, soluble (ACP1), transcript variant 4, mRNA [NM_001040649]	-0.6085234	0.028573772
A_21_P0003024	*TMEM242*	Homo sapiens transmembrane protein 242 (TMEM242), mRNA [NM_018452]	0.7840849	0.0442831
A_23_P74229	*LOC101928948*	PREDICTED: Homo sapiens uncharacterized LOC101928948 (LOC101928948), transcript variant X3, ncRNA [XR_425705]	-0.942923	0.028573772
A_32_P66020	*STK40*	Homo sapiens serine/threonine kinase 40 (STK40), transcript variant 3, mRNA [NM_032017]	-0.7297294	0.03136619
A_23_P313389	*SNX29*	Homo sapiens sorting nexin 29 (SNX29), mRNA [NM_032167]	-1.3399732	0.030362261
A_23_P300076	*UGCG*	Homo sapiens UDP-glucose ceramide glucosyltransferase (UGCG), mRNA [NM_003358]	-1.3592083	0.037136003
A_23_P98763	*IQCG*	Homo sapiens IQ motif containing G (IQCG), transcript variant 1, mRNA [NM_032263]	-1.3682766	0.030386643
A_23_P93009	*LRTOMT*	Homo sapiens leucine rich transmembrane and O-methyltransferase domain containing (LRTOMT), transcript variant 1, mRNA [NM_145309]	-0.8812539	0.038322687
A_24_P195037	*SRP19*	Homo sapiens signal recognition particle 19kDa (SRP19), transcript variant 6, mRNA [NM_001204199]	-1.5594792	0.02453978
A_23_P315836	*C1orf52*	Homo sapiens chromosome 1 open reading frame 52 (C1orf52), transcript variant 1, mRNA [NM_198077]	-1.617068	0.049603622
A_21_P0014203	*BAIAP2*	Homo sapiens BAI1-associated protein 2 (BAIAP2), transcript variant 2, mRNA [NM_017451]	-0.36949497	0.0442831
A_22_P00006179	*lnc-HTR1B-2*	LNCipedia lincRNA (lnc-HTR1B-2), lincRNA [lnc-HTR1B-2:1]	-1.0276443	0.027751032
A_33_P3334590	*LOC100507642*	Homo sapiens uncharacterized LOC100507642 (LOC100507642), transcript variant 2, long non-coding RNA [NR_108065]	3.0001326	0.03991377

A_23_P395911	*MTMR10*	Homo sapiens myotubularin related protein 10 (MTMR10), mRNA [NM_017762]	-0.7118256	0.04964876
A_33_P3284933	*FBXO17*	F-box protein 17 [Source:HGNC Symbol;Acc:HGNC:18754] [ENST00000601394]	1.1205941	0.02598378
A_22_P00001020	*IL27*	Homo sapiens interleukin 27 (IL27), mRNA [NM_145659]	0.6285478	0.0471361
A_21_P0007311	*lnc-AL391421.1-3*	LNCipedia lincRNA (lnc-AL391421.1-3), lincRNA [lnc-AL391421.1-3:1]	-0.99317026	0.047266874
A_32_P193288	*lnc-C11orf89-1*	LNCipedia lincRNA (lnc-C11orf89-1), lincRNA [lnc-C11orf89-1:3]	1.6862864	0.024948223
A_33_P3258324	*RPL18A*	Homo sapiens ribosomal protein L18a (RPL18A), mRNA [NM_000980]	1.4602346	0.034810174
A_23_P337550	*LOC102724279*	Homo sapiens uncharacterized LOC102724279 (LOC102724279), mRNA [NM_001302493]	1.6756104	0.030915326
A_23_P75839	*OTUD5*	Homo sapiens OTU deubiquitinase 5 (OTUD5), transcript variant 1, mRNA [NM_017602]	1.3895172	0.024948223
A_24_P36285	*TSG101*	Homo sapiens tumor susceptibility 101 (TSG101), mRNA [NM_006292]	-0.8881283	0.034750238
A_24_P203726	*ZNF224*	Homo sapiens zinc finger protein 224 (ZNF224), mRNA [NM_013398]	1.5541186	0.024948223
A_23_P79122	*C14orf166*	Homo sapiens chromosome 14 open reading frame 166 (C14orf166), mRNA [NM_016039]	-0.5547649	0.036297325
A_33_P3326927	*USE1*	Homo sapiens unconventional SNARE in the ER 1 homolog (S. cerevisiae) (USE1), mRNA [NM_018467]	-1.1777762	0.036041338
A_33_P3210059	*ZNF19*	Homo sapiens zinc finger protein 19 (ZNF19), mRNA [NM_006961]	-0.8146376	0.028303366
A_33_P3339276	*SF3A1*	Homo sapiens splicing factor 3a, subunit 1, 120kDa (SF3A1), mRNA [NM_005877]	-0.6735764	0.049704313
A_21_P0011990	*ACSM5*	Homo sapiens acyl-CoA synthetase medium-chain family member 5 (ACSM5), mRNA [NM_017888]	-1.1909006	0.044286337
A_24_P85942	*LINC00607*	Homo sapiens long intergenic non-protein coding RNA 607 (LINC00607), long non-coding RNA [NR_037195]	-1.2678759	0.049758088
A_24_P336848	*GCC2*	Homo sapiens GRIP and coiled-coil domain containing 2 (GCC2), transcript variant 1, mRNA [NM_181453]	-0.48280782	0.04792962
A_23_P342185	*ACYP2*	Homo sapiens acylphosphatase 2, muscle type (ACYP2), mRNA [NM_138448]	1.7824951	0.02737376

A_23_P169154	*WAPAL*	Homo sapiens wings apart-like homolog (Drosophila) (WAPAL), mRNA [NM_015045]	0.9918536	0.03403978
A_23_P1170	*ERMP1*	Homo sapiens endoplasmic reticulum metallopeptidase 1 (ERMP1), mRNA [NM_024896]	-0.92547673	0.048371606
A_33_P3605969	*ZFAND4*	Homo sapiens zinc finger, AN1-type domain 4 (ZFAND4), transcript variant 1, mRNA [NM_174890]	-1.4945501	0.048170313
A_23_P211561	*FAXDC2*	Homo sapiens fatty acid hydroxylase domain containing 2 (FAXDC2), mRNA [NM_032385]	-0.7588339	0.043879047
A_23_P92730	*MEI1*	Homo sapiens meiosis inhibitor 1 (MEI1), mRNA [NM_152513]	-1.2182289	0.02453978
A_23_P116614	*HSPB3*	Homo sapiens heat shock 27kDa protein 3 (HSPB3), mRNA [NM_006308]	1.0400846	0.039287783
A_23_P208293	*ME3*	Homo sapiens malic enzyme 3, NADP(+)-dependent, mitochondrial (ME3), transcript variant 2, mRNA [NM_001014811]	-1.2627451	0.04793844
A_21_P0014380	*PVRL2*	Homo sapiens poliovirus receptor-related 2 (herpesvirus entry mediator B) (PVRL2), transcript variant delta, mRNA [NM_001042724]	-2.9195545	0.041527454
A_33_P6608390	*BHLHE23*	Homo sapiens basic helix-loop-helix family, member e23 (BHLHE23), mRNA [NM_080606]	-1.3152086	0.049516298
A_24_P450285	*YTHDF3*	YTH domain family, member 3 [Source:HGNC Symbol;Acc:HGNC:26465] [ENST00000518373]	-1.0518973	0.03811336
A_23_P395595	*CCDC153*	Homo sapiens coiled-coil domain containing 153 (CCDC153), mRNA [NM_001145018]	-0.7751039	0.047832783
A_33_P3226070	*FNBP4*	Homo sapiens formin binding protein 4 (FNBP4), mRNA [NM_015308]	-1.1336701	0.03136619
A_33_P3412095	*lnc-CRIPAK-1*	LNCipedia lincRNA (lnc-CRIPAK-1), lincRNA [lnc-CRIPAK-1:1]	-0.85722446	0.042656552
A_21_P0014477	*PEX7*	Homo sapiens peroxisomal biogenesis factor 7 (PEX7), mRNA [NM_000288]	-0.8582004	0.049758088
A_32_P157965	*TCEA2*	Homo sapiens transcription elongation factor A (SII), 2 (TCEA2), transcript variant 1, mRNA [NM_003195]	-1.3557142	0.036857422
A_23_P50591	*EIF2S2*	Homo sapiens eukaryotic translation initiation factor 2, subunit 2 beta, 38kDa (EIF2S2), mRNA [NM_003908]	1.3037294	0.02453978

A_22_P00014249	*KCNK6*	Homo sapiens potassium channel, two pore domain subfamily K, member 6 (KCNK6), mRNA [NM_004823]	1.5942558	0.033091284
A_33_P3298251	*PPIP5K1*	Homo sapiens diphosphoinositol pentakisphosphate kinase 1 (PPIP5K1), transcript variant 5, mRNA [NM_001130858]	-2.2445765	0.026440267
A_23_P149206	*lnc-AC127496.3-6*	DKFZp686P2320_r1 686 (synonym: hlcc3) Homo sapiens cDNA clone DKFZp686P2320 5', mRNA sequence [AL603067]	0.9455849	0.033583984
A_21_P0000298	*B4GALT2*	Homo sapiens UDP-Gal:betaGlcNAc beta 1,4- galactosyltransferase, polypeptide 2 (B4GALT2), transcript variant 2, mRNA [NM_003780]	-0.48354948	0.031578258
A_24_P88800	*SNORD8*	Homo sapiens small nucleolar RNA, C/D box 8 (SNORD8), small nucleolar RNA [NR_002916]	1.4056072	0.038003992
A_33_P3368139	*COX7A2L*	Homo sapiens cytochrome c oxidase subunit VIIa polypeptide 2 like (COX7A2L), mRNA [NM_004718]	1.9355829	0.028573772
A_32_P18547	*MAP3K1*	Homo sapiens mitogen-activated protein kinase kinase kinase 1, E3 ubiquitin protein ligase (MAP3K1), mRNA [NM_005921]	-0.67309207	0.028573772
A_33_P3371055	*YBEY*	Homo sapiens ybeY metallopeptidase (putative) (YBEY), transcript variant 1, mRNA [NM_058181]	-0.6507532	0.036667652
A_23_P348911	*RAB1A*	Homo sapiens RAB1A, member RAS oncogene family (RAB1A), transcript variant 1, mRNA [NM_004161]	-1.3385763	0.03644264
A_24_P329635	*LINC01126*	Homo sapiens long intergenic non-protein coding RNA 1126 (LINC01126), long non-coding RNA [NR_027251]	-1.4760658	0.028573772
A_33_P3296308	*TSC1*	Homo sapiens tuberous sclerosis 1 (TSC1), transcript variant 1, mRNA [NM_000368]	-0.591825	0.049049266
A_22_P00015829	*DCAF16*	Homo sapiens DDB1 and CUL4 associated factor 16 (DCAF16), mRNA [NM_017741]	-0.6923341	0.04267235
A_22_P00006229	*LOC284930*	Homo sapiens uncharacterized LOC284930 (LOC284930), long non-coding RNA [NR_122046]	1.4255631	0.03136619
A_21_P0010912	*LOC100507054*	PREDICTED: Homo sapiens uncharacterized LOC100507054 (LOC100507054), ncRNA [XR_108535]	-0.59497136	0.02737376

A_24_P262407	*XLOC_l2_002176*	BROAD Institute lincRNA (XLOC_l2_002176), lincRNA [TCONS_l2_00003853]	-0.5927841	0.047831167
A_24_P100551	*THRA*	Homo sapiens thyroid hormone receptor, alpha (THRA), transcript variant 1, mRNA [NM_199334]	1.3333062	0.028546385
A_22_P00017498	*SH3RF1*	Homo sapiens SH3 domain containing ring finger 1 (SH3RF1), mRNA [NM_020870]	-1.662814	0.04371038
A_22_P00019886	*lnc-VWF-2*	LNCipedia lincRNA (lnc-VWF-2), lincRNA [lnc-VWF-2:3]	-1.0842783	0.045638688
A_33_P3360942	*HNF4A-AS1*	Homo sapiens HNF4A antisense RNA 1 (HNF4A-AS1), long non-coding RNA [NR_109949]	-1.0989732	0.049441572
A_23_P24234	*SCAMP1*	Homo sapiens secretory carrier membrane protein 1 (SCAMP1), transcript variant 1, mRNA [NM_004866]	-1.4202089	0.04954669
A_23_P28485	*OPN4*	Homo sapiens opsin 4 (OPN4), transcript variant 2, mRNA [NM_001030015]	-1.9723828	0.048238702
A_24_P114255	*GCA*	Homo sapiens grancalcin, EF-hand calcium binding protein (GCA), mRNA [NM_012198]	1.6180363	0.02695598
A_33_P3315600	*MBOAT2*	Homo sapiens membrane bound O-acyltransferase domain containing 2 (MBOAT2), mRNA [NM_138799]	-0.9865978	0.040692493
A_23_P201731	*YY1*	Homo sapiens YY1 transcription factor (YY1), mRNA [NM_003403]	1.4835229	0.046129715
A_23_P250571	*TRAF5*	Homo sapiens TNF receptor-associated factor 5 (TRAF5), transcript variant 1, mRNA [NM_004619]	-2.4520788	0.046657376
A_33_P3325467	*DMXL1*	Homo sapiens Dmx-like 1 (DMXL1), transcript variant 2, mRNA [NM_005509]	-1.154463	0.035560552
A_21_P0008499	*SMIM12*	Homo sapiens small integral membrane protein 12 (SMIM12), transcript variant 1, mRNA [NM_001164824]	-0.64616734	0.040764265
A_23_P23438	*lnc-CDCA4-1*	LNCipedia lincRNA (lnc-CDCA4-1), lincRNA [lnc-CDCA4-1:1]	-1.9676623	0.048163652
A_23_P65217	*SEMA4A*	Homo sapiens sema domain, immunoglobulin domain (Ig), transmembrane domain (TM) and short cytoplasmic domain, (semaphorin) 4A (SEMA4A), transcript variant 1, mRNA [NM_022367]	-1.0733219	0.028546385

A_23_P380298	*TGDS*	Homo sapiens TDP-glucose 4,6-dehydratase (TGDS), mRNA [NM_014305]	-0.4860263	0.03403978
A_23_P72117	*LZTS3*	Homo sapiens leucine zipper, putative tumor suppressor family member 3 (LZTS3), mRNA [NM_001282533]	0.64313823	0.035278592
A_24_P254551	*SMPDL3A*	Homo sapiens sphingomyelin phosphodiesterase, acid-like 3A (SMPDL3A), transcript variant 1, mRNA [NM_006714]	1.5054749	0.03297842
A_22_P00016948	*ARHGEF9*	Homo sapiens Cdc42 guanine nucleotide exchange factor (GEF) 9 (ARHGEF9), transcript variant 1, mRNA [NM_015185]	-1.2796954	0.047727138
A_33_P3283611	*KANTR*	Homo sapiens KDM5C adjacent non-coding transcript (KANTR), long non-coding RNA [NR_110456]	-1.5575525	0.04676237
A_33_P3295578	*IFIT3*	Homo sapiens interferon-induced protein with tetratricopeptide repeats 3 (IFIT3), transcript variant 3, mRNA [NM_001289758]	-0.7392936	0.027942823
A_24_P303815	*TCEAL8*	Homo sapiens transcription elongation factor A (SII)-like 8 (TCEAL8), transcript variant 1, mRNA [NM_153333]	-0.7390226	0.038424533
A_23_P259692	*UHRF2*	Homo sapiens ubiquitin-like with PHD and ring finger domains 2, E3 ubiquitin protein ligase (UHRF2), transcript variant 1, mRNA [NM_152896]	1.295282	0.036667652
A_24_P391526	*PSAT1*	Homo sapiens phosphoserine aminotransferase 1 (PSAT1), transcript variant 1, mRNA [NM_058179]	-1.3707001	0.03032242
A_22_P00005863	*MAGED1*	Homo sapiens melanoma antigen family D, 1 (MAGED1), transcript variant 1, mRNA [NM_001005333]	-1.4249746	0.04792134
A_24_P943997	*ERN1*	endoplasmic reticulum to nucleus signaling 1 [Source:HGNC Symbol;Acc:HGNC:3449] [ENST00000433197]	-0.91290414	0.028573772
A_33_P3418120	*ARL5B*	Homo sapiens ADP-ribosylation factor-like 5B (ARL5B), mRNA [NM_178815]	-1.0600142	0.045740407
A_33_P3281616	*IPPK*	Homo sapiens inositol 1,3,4,5,6-pentakisphosphate 2-kinase (IPPK), mRNA [NM_022755]	-1.0919365	0.03576752
A_23_P19663	*CDPF1*	Homo sapiens cysteine-rich, DPF motif domain containing 1 (CDPF1), mRNA [NM_207327]	-1.2812401	0.03683928

A_24_P195476	*CTGF*	Homo sapiens connective tissue growth factor (CTGF), mRNA [NM_001901]	-1.0521773	0.045638688
A_33_P3287310	*PMS2*	Homo sapiens PMS2 postmeiotic segregation increased 2 (S. cerevisiae) (PMS2), transcript variant 1, mRNA [NM_000535]	-0.61056	0.03840762
A_33_P3385957	*JUP*	Homo sapiens junction plakoglobin (JUP), transcript variant 1, mRNA [NM_002230]	-2.7865715	0.024948223
A_33_P3219803	*TTLL1*	Homo sapiens tubulin tyrosine ligase-like family member 1 (TTLL1), transcript variant 1, mRNA [NM_012263]	-0.5934352	0.04792962
A_22_P00024462	*PTMA*	Homo sapiens prothymosin, alpha (PTMA), transcript variant 1, mRNA [NM_001099285]	2.172818	0.028189372
A_33_P3392077	*RARA-AS1*	Homo sapiens RARA antisense RNA 1 (RARA-AS1), long non-coding RNA [NR_110861]	-1.7461627	0.044286337
A_23_P106016	*TP53I3*	Homo sapiens tumor protein p53 inducible protein 3 (TP53I3), transcript variant 1, mRNA [NM_004881]	-2.072209	0.024948223
A_23_P423427	*PRKD1*	Homo sapiens protein kinase D1 (PRKD1), mRNA [NM_002742]	-1.2960975	0.034293942
A_23_P25204	*FAM171B*	Homo sapiens family with sequence similarity 171, member B (FAM171B), mRNA [NM_177454]	-1.367087	0.032893
A_33_P3350227	*SLC25A3*	Homo sapiens solute carrier family 25 (mitochondrial carrier; phosphate carrier), member 3 (SLC25A3), transcript variant 3, mRNA [NM_213611]	-1.9627382	0.047941346
A_32_P176550	*MED22*	Homo sapiens mediator complex subunit 22 (MED22), transcript variant c, mRNA [NM_181491]	-1.5613201	0.044286337
A_23_P160226	*JMY*	Homo sapiens junction mediating and regulatory protein, p53 cofactor (JMY), mRNA [NM_152405]	1.6946129	0.03668808
A_33_P3651282	*MROH7*	Homo sapiens maestro heat-like repeat family member 7 (MROH7), transcript variant 1, mRNA [NM_001039464]	-0.7926276	0.046824332
A_22_P00014156	*RILPL1*	Homo sapiens Rab interacting lysosomal protein-like 1 (RILPL1), mRNA [NM_178314]	-1.4505131	0.03388051
A_23_P155907	*ZNF618*	Homo sapiens zinc finger protein 618 (ZNF618), mRNA [NM_133374]	0.86057204	0.045740407

A_24_P191067	*METAP1*	Homo sapiens methionyl aminopeptidase 1 (METAP1), mRNA [NM_015143]	-1.1285	0.0420299
A_23_P79931	*CLSTN1*	Homo sapiens calsyntenin 1 (CLSTN1), transcript variant 1, mRNA [NM_001009566]	-0.91067106	0.02737376
A_24_P313334	*ATRN*	Homo sapiens attractin (ATRN), transcript variant 2, mRNA [NM_139322]	-0.9360895	0.044286337
A_32_P119616	*SERF2*	Homo sapiens small EDRK-rich factor 2 (SERF2), transcript variant 4, mRNA [NM_001199877]	-0.80416626	0.048949
A_23_P55880	*ETFA*	Homo sapiens electron-transfer-flavoprotein, alpha polypeptide (ETFA), transcript variant 1, mRNA [NM_000126]	-0.6982694	0.040442128
A_33_P3281191	*ZNF564*	Homo sapiens zinc finger protein 564 (ZNF564), mRNA [NM_144976]	-1.0149349	0.045876816
A_22_P00018324	*NID1*	Homo sapiens nidogen 1 (NID1), mRNA [NM_002508]	-1.3272476	0.024948223
A_23_P3979	*SSPO*	Homo sapiens SCO-spondin (SSPO), mRNA [NM_198455]	-1.0444782	0.028573772
A_33_P3267410	*MRPS7*	Homo sapiens mitochondrial ribosomal protein S7 (MRPS7), mRNA [NM_015971]	-1.5351584	0.031569958
A_23_P4522	*KLLN*	Homo sapiens killin, p53-regulated DNA replication inhibitor (KLLN), mRNA [NM_001126049]	-1.466588	0.029808238
A_24_P143127	*TXNL1*	Homo sapiens thioredoxin-like 1 (TXNL1), transcript variant 1, mRNA [NM_004786]	1.6230338	0.028573772
A_33_P3247644	*GRIA3*	Homo sapiens glutamate receptor, ionotropic, AMPA 3 (GRIA3), transcript variant 2, mRNA [NM_000828]	-0.81294274	0.03485871
A_33_P3284463	*MTURN*	Homo sapiens maturin, neural progenitor differentiation regulator homolog (Xenopus) (MTURN), mRNA [NM_152793]	-0.5694548	0.034648914
A_22_P00015282	*ASAH1*	Homo sapiens N-acylsphingosine amidohydrolase (acid ceramidase) 1 (ASAH1), transcript variant 1, mRNA [NM_177924]	-0.48046345	0.03515347
A_23_P259621	*NBPF1*	Homo sapiens neuroblastoma breakpoint family, member 1 (NBPF1), mRNA [NM_017940]	-2.1550949	0.030915326
A_33_P3233005	*LAT2*	Homo sapiens linker for activation of T cells family, member 2 (LAT2),	-1.8210977	0.024948223

		transcript variant 1, mRNA [NM_032464]		
A_23_P356565	*MBTD1*	Homo sapiens mbt domain containing 1 (MBTD1), mRNA [NM_017643]	-0.80353117	0.024948223
A_33_P3311717	*RRP8*	Homo sapiens ribosomal RNA processing 8, methyltransferase, homolog (yeast) (RRP8), mRNA [NM_015324]	-1.4317187	0.037923194
A_21_P0000429	*TGIF1*	Homo sapiens TGFB-induced factor homeobox 1 (TGIF1), transcript variant 5, mRNA [NM_173209]	-0.8625513	0.039287783
A_33_P3474175	*SNORD113-1*	Homo sapiens small nucleolar RNA, C/D box 113-1 (SNORD113-1), small nucleolar RNA [NR_003229]	-0.95681965	0.038782965
A_32_P34920	*SFTA1P*	AGENCOURT_10018270 NIH_MGC_142 Homo sapiens cDNA clone IMAGE:6495000 5', mRNA sequence [BU601128]	-0.39067358	0.042621672
A_23_P15876	*FOXD1*	Homo sapiens forkhead box D1 (FOXD1), mRNA [NM_004472]	0.9012767	0.031526815
A_23_P332509	*ALPK2*	Homo sapiens alpha-kinase 2 (ALPK2), mRNA [NM_052947]	1.4765688	0.032557126
A_22_P00017343	*NBAS*	Homo sapiens neuroblastoma amplified sequence (NBAS), transcript variant 1, mRNA [NM_015909]	-3.4323618	0.03126305
A_33_P3327108	*lnc-USP47-2*	LNCipedia lincRNA (lnc-USP47-2), lincRNA [lnc-USP47-2:1]	-1.5148447	0.02598378
A_23_P31903	*NLK*	Homo sapiens nemo-like kinase (NLK), mRNA [NM_016231]	-1.9755442	0.03338082
A_33_P3384442	*VPS28*	Homo sapiens vacuolar protein sorting 28 homolog (S. cerevisiae) (VPS28), transcript variant 2, mRNA [NM_183057]	-0.8369446	0.040161368
A_23_P65699	*LAMA5*	laminin, alpha 5 [Source:HGNC Symbol;Acc:HGNC:6485] [ENST00000370677]	0.87145245	0.03390937
A_24_P398064	*SPG11*	Homo sapiens spastic paraplegia 11 (autosomal recessive) (SPG11), transcript variant 1, mRNA [NM_025137]	-0.93005085	0.03754682
A_23_P85726	*RGP1*	RGP1 retrograde golgi transport homolog (S. cerevisiae) [Source:HGNC Symbol;Acc:HGNC:21965] [ENST00000378078]	-0.84166193	0.026808027
A_23_P204998	*METTL18*	Homo sapiens methyltransferase like 18 (METTL18), mRNA [NM_033418]	-0.4032187	0.049603622
A_23_P395954	*FARP1*	Homo sapiens FERM, RhoGEF (ARHGEF) and pleckstrin domain	-0.75586486	0.028573772

		protein 1 (chondrocyte-derived) (FARP1), transcript variant 1, mRNA [NM_005766]		
A_23_P257503	*SSH2*	slingshot protein phosphatase 2 [Source:HGNC Symbol;Acc:HGNC:30580] [ENST00000394848]	-0.80097395	0.047137905
A_24_P166613	*HERC1*	Homo sapiens HECT and RLD domain containing E3 ubiquitin protein ligase family member 1 (HERC1), mRNA [NM_003922]	-1.0167959	0.042693015
A_23_P215198	*EPDR1*	Homo sapiens ependymin related 1 (EPDR1), transcript variant 1, mRNA [NM_017549]	-0.27694222	0.031606767
A_33_P3234989	*TYW1*	Homo sapiens tRNA-yW synthesizing protein 1 homolog (S. cerevisiae) (TYW1), mRNA [NM_018264]	1.3520074	0.04250221
A_33_P3384721	*IFT81*	Homo sapiens intraflagellar transport 81 (IFT81), transcript variant 1, mRNA [NM_014055]	-0.42917034	0.04879768
A_23_P14515	*NDUFB8*	Homo sapiens NADH dehydrogenase (ubiquinone) 1 beta subcomplex, 8, 19kDa (NDUFB8), transcript variant 1, mRNA [NM_005004]	-2.892419	0.035278592
A_23_P95594	*ACOT4*	Homo sapiens acyl-CoA thioesterase 4 (ACOT4), mRNA [NM_152331]	-0.9037538	0.03590667
A_33_P3424112	*NAT1*	Homo sapiens N-acetyltransferase 1 (arylamine N-acetyltransferase) (NAT1), transcript variant 5, mRNA [NM_000662]	-0.6398068	0.04383085
A_21_P0014809	*DHX40*	Homo sapiens DEAH (Asp-Glu-Ala-His) box polypeptide 40 (DHX40), transcript variant 1, mRNA [NM_024612]	-0.7616298	0.03668808
A_23_P58102	*FAM136A*	Homo sapiens family with sequence similarity 136, member A (FAM136A), mRNA [NM_032822]	-1.6202415	0.04369149
A_23_P366812	*EXOSC7*	Homo sapiens exosome component 7 (EXOSC7), transcript variant 1, mRNA [NM_015004]	-0.7919132	0.048964206
A_22_P00024217	*AQP5*	Homo sapiens aquaporin 5 (AQP5), mRNA [NM_001651]	-0.57091707	0.048715595
A_33_P3401138	*lnc-AP3M1-1*	Homo sapiens cDNA FLJ30962 fis, clone HCASM2000560. [AK055524]	-0.44319487	0.03136619
A_23_P14734	*CETN3*	Homo sapiens centrin, EF-hand protein, 3 (CETN3), transcript variant 2, mRNA [NM_004365]	1.708235	0.043712083
A_21_P0009597	*RPS27L*	Homo sapiens ribosomal protein S27-like (RPS27L), mRNA [NM_015920]	1.4892621	0.02737376

A_24_P90216	*LOC102723376*	Homo sapiens uncharacterized LOC102723376 (LOC102723376), long non-coding RNA [NR_110795]	1.3996205	0.029573848
A_33_P3404950	*LGR4*	Homo sapiens leucine-rich repeat containing G protein-coupled receptor 4 (LGR4), mRNA [NM_018490]	-1.1757615	0.03702924
A_32_P186981	*MINOS1*	Homo sapiens mitochondrial inner membrane organizing system 1 (MINOS1), transcript variant 5, mRNA [NM_001204083]	-0.900624	0.03485871
A_33_P3402171	*RPL17*	Homo sapiens ribosomal protein L17 (RPL17), transcript variant 1, mRNA [NM_000985]	1.2038926	0.04935762
A_22_P00019526	*EIF2S1*	Homo sapiens eukaryotic translation initiation factor 2, subunit 1 alpha, 35kDa (EIF2S1), mRNA [NM_004094]	-2.833966	0.028573772
A_33_P3277012	*TRIM52-AS1*	Homo sapiens TRIM52 antisense RNA 1 (head to head) (TRIM52-AS1), transcript variant 4, long non-coding RNA [NR_102762]	-1.2560109	0.02737376
A_33_P3298425	*DFFB*	Homo sapiens DNA fragmentation factor, 40kDa, beta polypeptide (caspase-activated DNase) (DFFB), transcript variant 2, mRNA [NM_004402]	-1.3038816	0.026497496
A_24_P320545	*LMO4*	Homo sapiens LIM domain only 4 (LMO4), mRNA [NM_006769]	1.1488233	0.029088594
A_24_P944299	*PTK7*	Homo sapiens protein tyrosine kinase 7 (PTK7), transcript variant PTK7-1, mRNA [NM_002821]	1.0680187	0.026098706
A_24_P64918	*CDC42BPA*	Homo sapiens CDC42 binding protein kinase alpha (DMPK-like) (CDC42BPA), transcript variant B, mRNA [NM_003607]	0.6638586	0.03947304
A_22_P00003891	*ZBTB1*	Homo sapiens zinc finger and BTB domain containing 1 (ZBTB1), transcript variant 2, mRNA [NM_014950]	-1.4045887	0.038309526
A_23_P355455	*lnc-CEP152-1*	LNCipedia lincRNA (lnc-CEP152-1), lincRNA [lnc-CEP152-1:1]	-1.848559	0.04080239
A_33_P3410859	*TBC1D5*	Homo sapiens TBC1 domain family, member 5 (TBC1D5), transcript variant 2, mRNA [NM_014744]	-1.0896745	0.02453978
A_23_P91891	*LOC729603*	Homo sapiens calcineurin-like EF-hand protein 1 pseudogene (LOC729603), non-coding RNA [NR_003288]	-1.2058283	0.036339592
A_33_P3381464	*COPB2*	Homo sapiens coatomer protein complex, subunit beta 2 (beta prime)	0.75370425	0.048163652

		(COPB2), transcript variant 1, mRNA [NM_004766]		
A_33_P3214159	*SLC16A11*	Homo sapiens solute carrier family 16, member 11 (SLC16A11), mRNA [NM_153357]	-0.40823177	0.047680285
A_23_P42514	*CDH2*	Homo sapiens cadherin 2, type 1, N-cadherin (neuronal) (CDH2), mRNA [NM_001792]	2.036927	0.028573772
A_22_P00005871	*C6orf62*	Homo sapiens chromosome 6 open reading frame 62 (C6orf62), mRNA [NM_030939]	-0.9865413	0.047982622
A_23_P210690	*lnc-ESCO1-2*	BX099285 Soares_fetal_lung_NbHL19W Homo sapiens cDNA clone IMAGp998M05679, mRNA sequence [BX099285]	1.947094	0.037472438
A_33_P3323368	*TRIB3*	Homo sapiens tribbles pseudokinase 3 (TRIB3), transcript variant 1, mRNA [NM_021158]	-3.1757207	0.034210585
A_23_P27075	*LINC00921*	Homo sapiens long intergenic non-protein coding RNA 921 (LINC00921), long non-coding RNA [NR_033904]	-0.82087827	0.04348676
A_19_P00315705	*GABARAP*	Homo sapiens GABA(A) receptor-associated protein (GABARAP), mRNA [NM_007278]	0.62918216	0.034210585
A_33_P3481987	*LOC101927354*	Homo sapiens uncharacterized LOC101927354 (LOC101927354), long non-coding RNA [NR_108073]	-2.393445	0.035515364
A_22_P00003050	*SLC16A12*	Homo sapiens solute carrier family 16, member 12 (SLC16A12), mRNA [NM_213606]	-0.74776006	0.032893
A_22_P00025961	*PBDC1*	Homo sapiens polysaccharide biosynthesis domain containing 1 (PBDC1), transcript variant 1, mRNA [NM_016500]	2.464511	0.027942823
A_32_P175539	*lnc-CNPY3-1*	602038318F1 NCI_CGAP_Brn64 Homo sapiens cDNA clone IMAGE:4185940 5', mRNA sequence [BF341178]	-1.1483567	0.027751032
A_33_P3289356	*RCN2*	Homo sapiens reticulocalbin 2, EF-hand calcium binding domain (RCN2), transcript variant 1, mRNA [NM_002902]	1.6466765	0.04217344
A_23_P133868	*CD58*	Homo sapiens CD58 molecule (CD58), transcript variant 1, mRNA [NM_001779]	-0.72964555	0.035207514
A_23_P372925	*ZKSCAN4*	Homo sapiens zinc finger with KRAB and SCAN domains 4 (ZKSCAN4), mRNA [NM_019110]	0.95019114	0.030441707

A_23_P18292	*CIPC*	Homo sapiens CLOCK-interacting pacemaker (CIPC), mRNA [NM_033426]	-1.2847457	0.046763405
A_33_P3231888	*RPL14*	Homo sapiens ribosomal protein L14 (RPL14), transcript variant 1, mRNA [NM_001034996]	-1.3588731	0.034334898
A_33_P3385436	*C5orf15*	Homo sapiens chromosome 5 open reading frame 15 (C5orf15), mRNA [NM_020199]	-0.94280887	0.04390963
A_22_P00011316	*PLAC8L1*	Homo sapiens PLAC8-like 1 (PLAC8L1), mRNA [NM_001029869]	-0.7832274	0.031603426
A_22_P00013214	*ZFYVE16*	Homo sapiens zinc finger, FYVE domain containing 16 (ZFYVE16), transcript variant 2, mRNA [NM_001105251]	-1.4394698	0.033146918
A_33_P3388745	*ZNRD1-AS1*	Homo sapiens ZNRD1 antisense RNA 1 (ZNRD1-AS1), long non-coding RNA [NR_026751]	-1.4238945	0.037336178
A_23_P5325	*LOC100132207*	Homo sapiens cDNA FLJ41345 fis, clone BRAWH2002761. [AK123339]	1.3962688	0.031091278
A_22_P00005232	*ERCC3*	Homo sapiens excision repair cross-complementation group 3 (ERCC3), transcript variant 1, mRNA [NM_000122]	-0.9899602	0.044839047
A_23_P31335	*PGM5*	Homo sapiens phosphoglucomutase 5 (PGM5), mRNA [NM_021965]	0.72818613	0.03947304
A_33_P3220381	*ZNF12*	Homo sapiens zinc finger protein 12 (ZNF12), transcript variant 1, mRNA [NM_016265]	-0.8349742	0.044364784
A_33_P3287418	*TRAPPC13*	Homo sapiens trafficking protein particle complex 13 (TRAPPC13), transcript variant 5, mRNA [NM_001243737]	1.0645196	0.024948223
A_23_P121945	*ARHGAP35*	Homo sapiens Rho GTPase activating protein 35 (ARHGAP35), mRNA [NM_004491]	-1.067687	0.027751032
A_21_P0014707	*SNCB*	Homo sapiens synuclein, beta (SNCB), transcript variant 1, mRNA [NM_001001502]	-1.2494559	0.040555127
A_33_P3269278	*TOMM20L*	Homo sapiens translocase of outer mitochondrial membrane 20 homolog (yeast)-like (TOMM20L), mRNA [NM_207377]	-1.6827118	0.03576752
A_32_P155776	*ZNF461*	Homo sapiens zinc finger protein 461 (ZNF461), transcript variant 1, mRNA [NM_153257]	1.0590414	0.03590667
A_33_P3239347	*POTEKP*	POTE ankyrin domain family, member K, pseudogene [Source:HGNC	-1.45675	0.04043569

		Symbol;Acc:HGNC:30182] [ENST00000397487]		
A_21_P0009794	*NKX3-1*	Homo sapiens NK3 homeobox 1 (NKX3-1), transcript variant 1, mRNA [NM_006167]	1.6618253	0.028321572
A_23_P394605	*lnc-VN1R2-1*	LNCipedia lincRNA (lnc-VN1R2-1), lincRNA [lnc-VN1R2-1:2]	-1.4457906	0.048398294
A_23_P129577	*SEC24A*	Homo sapiens SEC24 family member A (SEC24A), transcript variant 1, mRNA [NM_021982]	-0.72193027	0.027751032
A_24_P7594	*TIGD7*	Homo sapiens tigger transposable element derived 7 (TIGD7), mRNA [NM_033208]	-1.5298917	0.035186797
A_22_P00010367	*APOL6*	Homo sapiens apolipoprotein L, 6 (APOL6), mRNA [NM_030641]	1.1038758	0.042260204
A_33_P3401284	*lnc-SAP30-5*	LNCipedia lincRNA (lnc-SAP30-5), lincRNA [lnc-SAP30-5:33]	-0.8143302	0.03504642
A_24_P74371	*RMRP*	Homo sapiens RNA component of mitochondrial RNA processing endoribonuclease (RMRP), RNase MRP RNA [NR_003051]	-1.7783821	0.027751032
A_23_P214046	*CTSA*	Homo sapiens cathepsin A (CTSA), transcript variant 1, mRNA [NM_000308]	1.9763713	0.024948223
A_23_P255805	*FBXW11*	Homo sapiens F-box and WD repeat domain containing 11 (FBXW11), transcript variant 3, mRNA [NM_012300]	-2.1044188	0.047708835
A_23_P64121	*ZNF7*	Homo sapiens zinc finger protein 7 (ZNF7), transcript variant 2, mRNA [NM_003416]	0.9048113	0.036427937
A_33_P3221999	*KIAA1549L*	Homo sapiens KIAA1549-like (KIAA1549L), mRNA [NM_012194]	-0.88723356	0.03363671
A_23_P73548	*GSDMB*	Homo sapiens gasdermin B (GSDMB), transcript variant 3, mRNA [NM_001165958]	-0.91029	0.04761801
A_23_P163942	*GRIPAP1*	Homo sapiens GRIP1 associated protein 1 (GRIPAP1), mRNA [NM_020137]	-1.1100183	0.031430684
A_24_P313993	*SUPT4H1*	Homo sapiens suppressor of Ty 4 homolog 1 (S. cerevisiae) (SUPT4H1), transcript variant 1, mRNA [NM_003168]	-0.8945806	0.037137717
A_23_P101532	*CAPS*	Homo sapiens calcyphosine (CAPS), transcript variant 1, mRNA [NM_004058]	-1.4991038	0.026208585
A_23_P139919	*RPS11*	Homo sapiens ribosomal protein S11 (RPS11), mRNA [NM_001015]	-0.8408039	0.043335356

A_24_P278460	*CHST11*	Homo sapiens carbohydrate (chondroitin 4) sulfotransferase 11 (CHST11), transcript variant 1, mRNA [NM_018413]	-0.7691431	0.024948223
A_23_P73540	*FAR1*	Homo sapiens fatty acyl CoA reductase 1 (FAR1), mRNA [NM_032228]	-1.410997	0.027751032
A_23_P318646	*CCDC120*	Homo sapiens coiled-coil domain containing 120 (CCDC120), transcript variant 3, mRNA [NM_033626]	-0.7527032	0.03808641
A_24_P42014	*RPS10*	Homo sapiens ribosomal protein S10 (RPS10), transcript variant 2, mRNA [NM_001014]	-1.1026802	0.032261204
A_23_P393727	*HSF1*	Homo sapiens heat shock transcription factor 1 (HSF1), mRNA [NM_005526]	-2.0756254	0.029573848
A_21_P0000711	*NRP2*	Homo sapiens neuropilin 2 (NRP2), transcript variant 1, mRNA [NM_201266]	-0.8333876	0.03912857
A_33_P3381259	*SRD5A3-AS1*	Homo sapiens SRD5A3 antisense RNA 1 (SRD5A3-AS1), long non-coding RNA [NR_037969]	-1.18417	0.044449862
A_23_P20743	*lnc-DNAI1-1*	Q6UWT2_HUMAN (Q6UWT2) GAAI470, partial (64%) [THC2753675]	-0.5831876	0.032155342
A_33_P3288839	*TMEM246*	Homo sapiens transmembrane protein 246 (TMEM246), transcript variant 1, mRNA [NM_032342]	-1.5668316	0.028573772
A_32_P94	*C14orf37*	Homo sapiens chromosome 14 open reading frame 37 (C14orf37), mRNA [NM_001001872]	-1.4091712	0.04465309
A_23_P73530	*LHFPL4*	Homo sapiens lipoma HMGIC fusion partner-like 4 (LHFPL4), mRNA [NM_198560]	1.8327804	0.044449862
A_33_P3262452	*MTMR1*	Homo sapiens myotubularin related protein 1 (MTMR1), mRNA [NM_003828]	-3.1021855	0.029573848
A_23_P203737	*PEX26*	Homo sapiens peroxisomal biogenesis factor 26 (PEX26), transcript variant 2, mRNA [NM_001127649]	-1.8939946	0.031091278
A_23_P125668	*INTS4*	Homo sapiens integrator complex subunit 4 (INTS4), mRNA [NM_033547]	1.512424	0.02453978
A_33_P3229037	*SLC25A43*	Homo sapiens solute carrier family 25, member 43 (SLC25A43), mRNA [NM_145305]	-1.0285062	0.04114316
A_21_P0014008	*lnc-VSTM2B-1*	LNCipedia lincRNA (lnc-VSTM2B-1), lincRNA [lnc-VSTM2B-1:2]	-0.83198	0.041018903
A_22_P00008393	*ZNF322*	Homo sapiens zinc finger protein 322 (ZNF322), transcript variant 1, mRNA [NM_001242797]	-1.6587948	0.035278592

A_23_P338233	*EHD4-AS1*	Homo sapiens EHD4 antisense RNA 1 (EHD4-AS1), long non-coding RNA [NR_120332]	1.5814772	0.03716006
A_24_P414786	*BCDIN3D*	Homo sapiens BCDIN3 domain containing (BCDIN3D), mRNA [NM_181708]	1.1190321	0.043885134
A_24_P300483	*CDC40*	Homo sapiens cell division cycle 40 (CDC40), mRNA [NM_015891]	-1.1902729	0.024948223
A_23_P46333	*C7orf25*	Homo sapiens chromosome 7 open reading frame 25 (C7orf25), transcript variant 2, mRNA [NM_024054]	1.222575	0.03773257
A_33_P3310232	*MTF2*	Homo sapiens metal response element binding transcription factor 2 (MTF2), transcript variant 1, mRNA [NM_007358]	-0.98826194	0.029979022
A_24_P272873	*L2HGDH*	Homo sapiens L-2-hydroxyglutarate dehydrogenase (L2HGDH), mRNA [NM_024884]	-1.1134218	0.049413078
A_22_P00010973	*RPL13AP3*	Homo sapiens ribosomal protein L13a pseudogene 3 (RPL13AP3), non-coding RNA [NR_004844]	-0.8041276	0.035679508
A_33_P3315258	*lnc-NRIP2-2*	LNCipedia lincRNA (lnc-NRIP2-2), lincRNA [lnc-NRIP2-2:1]	-0.99883306	0.028573772
A_24_P206776	*CHD1L*	Homo sapiens chromodomain helicase DNA binding protein 1-like (CHD1L), transcript variant 1, mRNA [NM_004284]	-0.52681345	0.033095628
A_23_P394448	*CRYAB*	Homo sapiens crystallin, alpha B (CRYAB), transcript variant 1, mRNA [NM_001885]	-1.7576156	0.042621672
A_22_P00013057	*DPY19L1*	Homo sapiens dpy-19-like 1 (C. elegans) (DPY19L1), mRNA [NM_015283]	0.63967973	0.031367566
A_24_P312519	*LOC284648*	Homo sapiens uncharacterized LOC284648 (LOC284648), long non-coding RNA [NR_036490]	0.5200305	0.02919588
A_24_P403561	*PBLD*	Homo sapiens phenazine biosynthesis-like protein domain containing (PBLD), transcript variant 2, mRNA [NM_001033083]	-1.5899222	0.048398294
A_22_P00021911	*LRP4*	Homo sapiens low density lipoprotein receptor-related protein 4 (LRP4), mRNA [NM_002334]	-1.1673578	0.0442831
A_22_P00011639	*lnc-BRF1-2*	LNCipedia lincRNA (lnc-BRF1-2), lincRNA [lnc-BRF1-2:1]	1.5210601	0.03136619
A_21_P0007619	*LOC101927206*	PREDICTED: Homo sapiens uncharacterized LOC101927206 (LOC101927206), ncRNA [XR_241444]	-1.1052728	0.03138659

A_23_P14273	*SNAI3-AS1*	Homo sapiens SNAI3 antisense RNA 1 (SNAI3-AS1), transcript variant 1, long non-coding RNA [NR_024402]	1.9042835	0.02453978
A_23_P13073	*ZFYVE21*	Homo sapiens zinc finger, FYVE domain containing 21 (ZFYVE21), transcript variant 2, mRNA [NM_024071]	1.4548765	0.026808027
A_22_P00000203	*PUS3*	Homo sapiens pseudouridylate synthase 3 (PUS3), transcript variant 1, mRNA [NM_031307]	1.7184854	0.028573772
A_33_P3313640	*ITPR2*	Homo sapiens inositol 1,4,5-trisphosphate receptor, type 2 (ITPR2), mRNA [NM_002223]	-0.56848115	0.03808641
A_33_P3271051	*EEF1G*	Homo sapiens eukaryotic translation elongation factor 1 gamma (EEF1G), mRNA [NM_001404]	0.8773473	0.028573772
A_33_P3224819	*CYTH3*	Homo sapiens cytohesin 3 (CYTH3), mRNA [NM_004227]	-1.7676852	0.049603622
A_24_P787947	*SLC45A4*	Homo sapiens solute carrier family 45, member 4 (SLC45A4), transcript variant 1, mRNA [NM_001286646]	0.59189665	0.044227738
A_33_P3403356	*YPEL2*	Homo sapiens yippee-like 2 (Drosophila) (YPEL2), mRNA [NM_001005404]	-0.8452208	0.037395623
A_22_P00000269	*IDS*	Homo sapiens iduronate 2-sulfatase (IDS), transcript variant 1, mRNA [NM_000202]	-0.93596256	0.03601631
A_23_P202170	*lnc-AC007557.1.1-3*	LNCipedia lincRNA (lnc-AC007557.1.1-3), lincRNA [lnc-AC007557.1.1-3:1]	-1.5604899	0.034210585
A_33_P3383422	*MGEA5*	Homo sapiens meningioma expressed antigen 5 (hyaluronidase) (MGEA5), transcript variant 1, mRNA [NM_012215]	-0.86137366	0.04929023
A_33_P3236661	*DHRS4*	Homo sapiens dehydrogenase/reductase (SDR family) member 4 (DHRS4), transcript variant 6, mRNA [NM_001282991]	-1.1683975	0.04250221
A_21_P0007407	*PCDH9*	Homo sapiens protocadherin 9, mRNA (cDNA clone MGC:167030 IMAGE:8860363), complete cds. [BC150296]	1.3278177	0.046944562
A_23_P361469	*lnc-RP11-158I9.5.1-2*	LNCipedia lincRNA (lnc-RP11-158I9.5.1-2), lincRNA [lnc-RP11-158I9.5.1-2:1]	-0.9180393	0.047414247
A_23_P171237	*METTL6*	Homo sapiens methyltransferase like 6 (METTL6), transcript variant 1, mRNA [NM_152396]	-0.6442525	0.024948223
A_23_P1523	*ACRC*	Homo sapiens acidic repeat containing (ACRC), mRNA [NM_052957]	1.8207917	0.04959708

A_21_P0011448	*RHOD*	Homo sapiens ras homolog family member D (RHOD), transcript variant 1, mRNA [NM_014578]	1.0474837	0.030252501
A_22_P00004698	*XLOC_l2_005314*	BROAD Institute lincRNA (XLOC_l2_005314), lincRNA [TCONS_l2_00009861]	-0.8629443	0.029808238
A_33_P3303449	*LOC100287098*	Homo sapiens uncharacterized LOC100287098 (LOC100287098), long non-coding RNA [NR_109770]	-2.8058786	0.02598378
A_33_P3240497	*LEPR*	Homo sapiens leptin receptor (LEPR), transcript variant 5, mRNA [NM_001198688]	-0.7699751	0.04159686
A_22_P00014899	*ODF3L2*	Homo sapiens outer dense fiber of sperm tails 3-like 2 (ODF3L2), mRNA [NM_182577]	0.5029931	0.031526815
A_33_P3277728	*lnc-SLC9A1-1*	LNCipedia lincRNA (lnc-SLC9A1-1), lincRNA [lnc-SLC9A1-1:1]	-1.244583	0.0442831
A_22_P00001250	*ITSN1*	Homo sapiens intersectin 1 (SH3 domain protein) (ITSN1), transcript variant 2, mRNA [NM_001001132]	1.0008354	0.036131933
A_22_P00010319	*MGC45922*	Homo sapiens uncharacterized LOC284365 (MGC45922), long non-coding RNA [NR_038359]	1.9005554	0.028573772
A_24_P398898	*lnc-MUC5B-1*	LNCipedia lincRNA (lnc-MUC5B-1), lincRNA [lnc-MUC5B-1:1]	-0.45555863	0.03403978
A_23_P152028	*MORF4L1*	Homo sapiens mortality factor 4 like 1 (MORF4L1), transcript variant 2, mRNA [NM_206839]	1.3966318	0.042976096
A_23_P301984	*TRIP4*	Homo sapiens thyroid hormone receptor interactor 4 (TRIP4), mRNA [NM_016213]	0.91278946	0.03403978
A_23_P145694	*C8orf48*	Homo sapiens chromosome 8 open reading frame 48 (C8orf48), mRNA [NM_001007090]	2.2451134	0.034342222
A_33_P3261565	*ASNS*	Homo sapiens asparagine synthetase (glutamine-hydrolyzing) (ASNS), transcript variant 2, mRNA [NM_001673]	-12098992	0.034306627
A_23_P4474	*ZRANB1*	Homo sapiens zinc finger, RAN-binding domain containing 1 (ZRANB1), mRNA [NM_017580]	-0.6954949	0.036667652
A_24_P288448	*IER3IP1*	Homo sapiens immediate early response 3 interacting protein 1 (IER3IP1), mRNA [NM_016097]	-0.64989007	0.04217344
A_23_P2097	*RASSF2*	Ras association (RalGDS/AF-6) domain family member 2 [Source:HGNC Symbol;Acc:HGNC:9883] [ENST00000478553]	-1.4405406	0.02453978

A_23_P84836	*TRIM68*	Homo sapiens tripartite motif containing 68 (TRIM68), mRNA [NM_018073]	-0.81862086	0.028192867
A_21_P0006850	*NPEPPS*	Homo sapiens aminopeptidase puromycin sensitive (NPEPPS), mRNA [NM_006310]	-0.5579903	0.04590026
A_33_P3317725	*lnc-LYZL1-2*	LNCipedia lincRNA (lnc-LYZL1-2), lincRNA [lnc-LYZL1-2:1]	1.176883	0.046958555
A_33_P3383696	*LINC00243*	Homo sapiens cDNA FLJ40693 fis, clone THYMU2025042. [AK098012]	-0.35252976	0.04645665
A_21_P0014048	*SPEG*	Homo sapiens SPEG complex locus (SPEG), transcript variant 1, mRNA [NM_005876]	-1.2312021	0.03808641
A_22_P00008931	*RBFOX3*	Homo sapiens RNA binding protein, fox-1 homolog (C. elegans) 3 (RBFOX3), mRNA [NM_001082575]	1.7199757	0.029808238
A_24_P763243	*EIF2S2*	Homo sapiens eukaryotic translation initiation factor 2, subunit 2 beta, 38kDa (EIF2S2), mRNA [NM_003908]	-1.904012	0.035769645
A_23_P165989	*EEF1A1*	Homo sapiens eukaryotic translation elongation factor 1 alpha 1 (EEF1A1), mRNA [NM_001402]	3.6881835	0.032261204
A_32_P76853	*NEURL2*	Homo sapiens neuralized E3 ubiquitin protein ligase 2 (NEURL2), transcript variant 1, mRNA [NM_080749]	1.2140244	0.031964067
A_33_P3232504	*GOLGA8R*	Homo sapiens golgin A8 family, member R (GOLGA8R), mRNA [NM_001282484]	-0.8614358	0.03504642
A_23_P7827	*CYSRT1*	Homo sapiens cysteine-rich tail protein 1 (CYSRT1), mRNA [NM_199001]	-0.7150931	0.03515347
A_23_P76882	*FAM26F*	Homo sapiens family with sequence similarity 26, member F (FAM26F), transcript variant 1, mRNA [NM_001010919]	-0.9036115	0.049070477
A_23_P150609	*CCNB1IP1*	Homo sapiens cyclin B1 interacting protein 1, E3 ubiquitin protein ligase (CCNB1IP1), transcript variant 4, mRNA [NM_182852]	-0.8093934	0.042461615
A_23_P217068	*IGF2*	Homo sapiens insulin-like growth factor 2 (IGF2), transcript variant 1, mRNA [NM_000612]	-0.99978614	0.041978877
A_32_P91042	*RPL12*	Homo sapiens ribosomal protein L12 (RPL12), mRNA [NM_000976]	-2.474249	0.041609965
A_23_P338890	*LOC100129034*	Homo sapiens uncharacterized LOC100129034 (LOC100129034), long non-coding RNA [NR_027406]	-1.2531731	0.03436134
A_33_P3308263	*PTPN1*	Homo sapiens protein tyrosine phosphatase, non-receptor type 1	3.7808442	0.04902804

		(PTPN1), transcript variant 1, mRNA [NM_002827]		
A_33_P3328659	*DIRC2*	Homo sapiens disrupted in renal carcinoma 2 (DIRC2), mRNA [NM_032839]	-0.6962128	0.027751032
A_24_P307014	*CELSR1*	Homo sapiens cadherin, EGF LAG seven-pass G-type receptor 1 (CELSR1), mRNA [NM_014246]	-1.2759508	0.04657141
A_23_P89163	*BRD2*	Homo sapiens bromodomain containing 2 (BRD2), transcript variant 1, mRNA [NM_005104]	0.55196285	0.03313523
A_23_P94703	*EXOC7*	Homo sapiens exocyst complex component 7 (EXOC7), transcript variant 1, mRNA [NM_001013839]	-0.72014904	0.040555127
A_33_P3409277	*TOR1B*	Homo sapiens torsin family 1, member B (torsin B) (TOR1B), mRNA [NM_014506]	-1.3678508	0.045708336
A_23_P160354	*LOC100128882*	PREDICTED: Homo sapiens uncharacterized LOC100128882 (LOC100128882), transcript variant X3, misc_RNA [XR_429794]	-0.6184731	0.029862247
A_23_P212728	*AKT3*	Homo sapiens v-akt murine thymoma viral oncogene homolog 3 (AKT3), transcript variant 2, mRNA [NM_181690]	-0.95327663	0.032261204
A_22_P00019134	*TBC1D23*	Homo sapiens TBC1 domain family, member 23 (TBC1D23), transcript variant 1, mRNA [NM_001199198]	-0.8295648	0.04488468
A_33_P3220390	*lnc-CBX1-1*	LNCipedia lincRNA (lnc-CBX1-1), lincRNA [lnc-CBX1-1:1]	1.9904025	0.02598378
A_33_P3318852	*WTAP*	Homo sapiens Wilms tumor 1 associated protein, mRNA (cDNA clone IMAGE:5399821), partial cds. [BC028180]	-1.1687335	0.0370712
A_21_P0000312	*TBC1D8B*	Homo sapiens TBC1 domain family, member 8B (with GRAM domain) (TBC1D8B), transcript variant 2, mRNA [NM_198881]	-1.0906432	0.027751032
A_23_P129829	*SNORA14B*	Homo sapiens small nucleolar RNA, H/ACA box 14B (SNORA14B), small nucleolar RNA [NR_002956]	-0.44120106	0.037030667
A_21_P0010748	*ORMDL3*	Homo sapiens ORMDL sphingolipid biosynthesis regulator 3 (ORMDL3), mRNA [NM_139280]	2.1698856	0.034052864
A_33_P3220160	*RPS23*	Homo sapiens ribosomal protein S23 (RPS23), mRNA [NM_001025]	1.04286	0.035278592
A_32_P34116	*RAC2*	Homo sapiens cDNA FLJ39605 fis, clone SKNSH2005981, weakly similar	-0.93543863	0.035227668

		to RAS-RELATED C3 BOTULINUM TOXIN SUBSTRATE 2. [AK096924]		
A_24_P321634	*NUS1*	Homo sapiens nuclear undecaprenyl pyrophosphate synthase 1 homolog (S. cerevisiae) (NUS1), mRNA [NM_138459]	-1.4273823	0.04264674
A_19_P00322573	*ZMYM5*	Homo sapiens zinc finger, MYM-type 5 (ZMYM5), transcript variant 1, mRNA [NM_001039650]	-1.1484439	0.037593983
A_33_P3405360	*LINC01372*	Homo sapiens long intergenic non-protein coding RNA 1372 (LINC01372), long non-coding RNA [NR_108104]	1.668066	0.04402506
A_33_P3372682	*PDCL*	Homo sapiens phosducin-like (PDCL), mRNA [NM_005388]	-0.91561437	0.039878353
A_32_P118258	*GTSE1-AS1*	Homo sapiens GTSE1 antisense RNA 1 (head to head) (GTSE1-AS1), long non-coding RNA [NR_024009]	-0.92237467	0.04639083
A_21_P0000543	*RPL21*	Homo sapiens ribosomal protein L21 (RPL21), mRNA [NM_000982]	-0.93647903	0.028573772
A_32_P31992	*ATP1A1-AS1*	Homo sapiens ATP1A1 antisense RNA 1 (ATP1A1-AS1), transcript variant 2, long non-coding RNA [NR_027645]	-1.723411	0.027751032
A_24_P193498	*ZHX2*	Homo sapiens zinc fingers and homeoboxes 2 (ZHX2), mRNA [NM_014943]	-0.7862198	0.03922557
A_23_P354827	*TM2D3*	Homo sapiens TM2 domain containing 3 (TM2D3), transcript variant 1, mRNA [NM_078474]	-0.619822	0.034334898
A_21_P0013671	*ZNF550*	Homo sapiens zinc finger protein 550 (ZNF550), transcript variant 1, mRNA [NM_001277090]	2.5905075	0.02724032
A_23_P58953	*XLOC_l2_015295*	BROAD Institute lincRNA (XLOC_l2_015295), lincRNA [TCONS_l2_00029481]	-1.9869895	0.04434183
A_24_P160401	*NQO2*	Homo sapiens NAD(P)H dehydrogenase, quinone 2 (NQO2), transcript variant 3, mRNA [NM_000904]	-0.80831504	0.040442128
A_23_P20494	*CDCP1*	Homo sapiens CUB domain containing protein 1 (CDCP1), transcript variant 2, mRNA [NM_178181]	-0.94858795	0.049413078
A_33_P3285430	*NDRG1*	Homo sapiens N-myc downstream regulated 1 (NDRG1), transcript variant 2, mRNA [NM_006096]	1.5801508	0.03190556
A_22_P00003121	*DNM1L*	dynamin 1-like [Source:HGNC Symbol;Acc:HGNC:2973] [ENST00000547640]	-0.95538515	0.04217344

A_32_P50417	*CAB39L*	Homo sapiens calcium binding protein 39-like (CAB39L), transcript variant 2, mRNA [NM_001079670]	-2.0987234	0.029573848
A_21_P0012320	*PLEKHO1*	Homo sapiens pleckstrin homology domain containing, family O member 1 (PLEKHO1), mRNA [NM_016274]	2.729108	0.029573848
A_32_P100683	*SCUBE2*	Homo sapiens signal peptide, CUB domain, EGF-like 2 (SCUBE2), transcript variant 1, mRNA [NM_020974]	-0.7684641	0.024948223
A_24_P319923	*TTC28*	Homo sapiens tetratricopeptide repeat domain 28 (TTC28), mRNA [NM_001145418]	1.329773	0.04041675
A_23_P7066	*MYLK*	Homo sapiens myosin light chain kinase (MYLK), transcript variant 1, mRNA [NM_053025]	-1.0660685	0.047665294
A_23_P208208	*RPL9*	Homo sapiens ribosomal protein L9 (RPL9), transcript variant 2, mRNA [NM_001024921]	-1.4519986	0.028573772
A_22_P00008002	*ZNF649*	Homo sapiens zinc finger protein 649 (ZNF649), mRNA [NM_023074]	-1.0406541	0.03468858
A_24_P944616	*BLCAP*	Homo sapiens bladder cancer associated protein (BLCAP), transcript variant 1, mRNA [NM_006698]	-0.82120204	0.033412892
A_23_P128166	*HP1BP3*	Homo sapiens heterochromatin protein 1, binding protein 3 (HP1BP3), mRNA [NM_016287]	-0.7742007	0.04733213
A_21_P0013818	*RAB21*	Homo sapiens RAB21, member RAS oncogene family (RAB21), mRNA [NM_014999]	1.3064511	0.024948223
A_19_P00315627	*XLOC_l2_015752*	BROAD Institute lincRNA (XLOC_l2_015752), lincRNA [TCONS_l2_00030522]	-0.8566341	0.047218688
A_24_P288685	*LOC101930053*	PREDICTED: Homo sapiens uncharacterized LOC101930053 (LOC101930053), transcript variant X1, ncRNA [XR_428440]	-0.7366223	0.033726536
A_23_P61960	*IL13RA1*	Homo sapiens interleukin 13 receptor, alpha 1 (IL13RA1), mRNA [NM_001560]	-0.45106557	0.042850092
A_33_P3379157	*ATP6V0E2*	Homo sapiens ATPase, H+ transporting V0 subunit e2 (ATP6V0E2), transcript variant 1, mRNA [NM_145230]	1.0378017	0.03515347
A_19_P00321656	*MTCH1*	Homo sapiens mitochondrial carrier 1 (MTCH1), transcript variant 2, mRNA [NM_001271641]	-2.162055	0.049758088
A_21_P0014566	*DDAH1*	Homo sapiens dimethylarginine dimethylaminohydrolase 1 (DDAH1),	-1.0070053	0.02737376

		transcript variant 2, mRNA [NM_001134445]		
A_33_P3263906	*ARHGAP24*	Rho GTPase activating protein 24 [Source:HGNC Symbol;Acc:HGNC:25361] [ENST00000509709]	-1.4768548	0.03668808
A_23_P208674	*TUBE1*	tubulin, epsilon 1 [Source:HGNC Symbol;Acc:HGNC:20775] [ENST00000368657]	-2.5201292	0.028573772
A_21_P0014295	*EMC10*	Homo sapiens ER membrane protein complex subunit 10 (EMC10), transcript variant 1, mRNA [NM_175063]	1.9144123	0.034342222
A_33_P3217230	*PIGBOS1*	PREDICTED: Homo sapiens uncharacterized LOC101928527 (LOC101928527), transcript variant X2, ncRNA [XR_243152]	-1.3269808	0.028573772
A_23_P122876	*ZNF91*	Homo sapiens zinc finger protein 91 (ZNF91), transcript variant 1, mRNA [NM_003430]	-2.2942734	0.036588036
A_23_P163647	*TAF6*	Homo sapiens TAF6 RNA polymerase II, TATA box binding protein (TBP)-associated factor, 80kDa (TAF6), transcript variant 1, mRNA [NM_005641]	-0.92092943	0.031367566
A_22_P00003321	*ECI1*	Homo sapiens enoyl-CoA delta isomerase 1 (ECI1), transcript variant 1, mRNA [NM_001919]	-1.00832	0.032357268
A_23_P164258	*lnc-CARHSP1-1*	fs10h06.y1 Human Lens cDNA (Normalized): fs Homo sapiens cDNA clone fs10h06 5', mRNA sequence [CD674525]	-0.9441696	0.048182435
A_33_P3313421	*PIPOX*	Homo sapiens pipecolic acid oxidase (PIPOX), mRNA [NM_016518]	0.53796107	0.033719625
A_23_P59349	*UHRF1BP1*	Homo sapiens UHRF1 binding protein 1 (UHRF1BP1), mRNA [NM_017754]	-1.6103008	0.029808238
A_23_P15108	*HECA*	Homo sapiens headcase homolog (Drosophila) (HECA), mRNA [NM_016217]	1.5302749	0.02453978
A_23_P71440	*YPEL3*	Homo sapiens yippee-like 3 (Drosophila) (YPEL3), transcript variant 1, mRNA [NM_031477]	1.6695235	0.03233256
A_23_P213247	*GOLGA7*	Homo sapiens golgin A7 (GOLGA7), transcript variant 2, mRNA [NM_001002296]	-1.1095971	0.037634917
A_33_P3370930	*FBXL5*	Homo sapiens F-box and leucine-rich repeat protein 5 (FBXL5), transcript variant 1, mRNA [NM_012161]	-1.5430079	0.031526815

A_22_P00010071	*LAMB1*	laminin, beta 1 [Source:HGNC Symbol;Acc:HGNC:6486] [ENST00000393559]	-1.4809965	0.04456819
A_23_P104617	*ADCK3*	Homo sapiens aarF domain containing kinase 3 (ADCK3), mRNA [NM_020247]	-1.2422268	0.038904693
A_23_P156156	*GYLTL1B*	Homo sapiens glycosyltransferase-like 1B (GYLTL1B), transcript variant 1, mRNA [NM_152312]	1.497092	0.045740407
A_33_P3322499	*ZCCHC9*	Homo sapiens zinc finger, CCHC domain containing 9 (ZCCHC9), transcript variant 1, mRNA [NM_032280]	1.8053664	0.02598378
A_33_P3239148	*ZNF833P*	Homo sapiens zinc finger protein 833, pseudogene (ZNF833P), non-coding RNA [NR_028594]	-0.67251587	0.04129315
A_24_P347624	*ZNF548*	Homo sapiens zinc finger protein 548 (ZNF548), transcript variant 1, mRNA [NM_001172773]	1.2000631	0.047728516
A_23_P170857	*SNURF*	Homo sapiens SNRPN upstream reading frame (SNURF), transcript variant 2, mRNA [NM_022804]	-0.7010076	0.045638688
A_33_P3361202	*IL1RAP*	Homo sapiens interleukin 1 receptor accessory protein (IL1RAP), transcript variant 1, mRNA [NM_002182]	2.4989343	0.02453978
A_22_P00013971	*CCSAP*	Homo sapiens centriole, cilia and spindle-associated protein (CCSAP), mRNA [NM_145257]	-0.94539326	0.035269484
A_23_P29384	*lnc-RTN2-1*	LNCipedia lincRNA (lnc-RTN2-1), lincRNA [lnc-RTN2-1:1]	-0.45199898	0.038287252
A_23_P159741	*ZNF502*	Homo sapiens zinc finger protein 502 (ZNF502), transcript variant 1, mRNA [NM_033210]	-1.4602373	0.028573772
A_33_P3294162	*BCOR*	Homo sapiens BCL6 corepressor (BCOR), transcript variant 1, mRNA [NM_017745]	-1.309613	0.03755321
A_24_P82419	*PMPCB*	Homo sapiens peptidase (mitochondrial processing) beta (PMPCB), mRNA [NM_004279]	1.3482001	0.043485392
A_23_P27167	*H3F3C*	Homo sapiens H3 histone, family 3C (H3F3C), mRNA [NM_001013699]	-0.8398547	0.036667652
A_24_P113824	*RNASEH1*	Homo sapiens ribonuclease H1 (RNASEH1), transcript variant 1, mRNA [NM_002936]	-1.1393349	0.041761205
A_23_P60376	*TMEM50A*	Homo sapiens transmembrane protein 50A (TMEM50A), mRNA [NM_014313]	-0.628275	0.03012347
A_21_P0005915	*EDF1*	Homo sapiens endothelial differentiation-related factor 1 (EDF1),	-0.682556	0.049603622

		transcript variant beta, mRNA [NM_153200]		
A_23_P133629	*lnc-SLC45A4-1*	LNCipedia lincRNA (lnc-SLC45A4-1), lincRNA [lnc-SLC45A4-1:1]	-0.5557769	0.04041675
A_19_P00812250	*CDC23*	Homo sapiens cell division cycle 23 (CDC23), mRNA [NM_004661]	-1.1929406	0.047816608
A_23_P94494	*SMAD4*	Homo sapiens SMAD family member 4 (SMAD4), mRNA [NM_005359]	-0.9091563	0.0442831
A_23_P75097	*TLE4*	Homo sapiens transducin-like enhancer of split 4 (TLE4), transcript variant 3, mRNA [NM_007005]	1.5036573	0.040706977
A_22_P00012059	*PDZD7*	Homo sapiens PDZ domain containing 7 (PDZD7), transcript variant 2, mRNA [NM_024895]	-1.0980663	0.035935212
A_23_P79942	*PLLP*	plasmolipin [Source:HGNC Symbol;Acc:HGNC:18553] [ENST00000564376]	-0.8207061	0.024948223
A_32_P9575	*PANK2*	Homo sapiens pantothenate kinase 2 (PANK2), transcript variant 1, mRNA [NM_153638]	-0.80978185	0.046680246
A_22_P00000873	*MRPL45*	Homo sapiens mitochondrial ribosomal protein L45 (MRPL45), transcript variant 1, mRNA [NM_032351]	-0.85450125	0.026409145
A_21_P0014094	*lnc-AGRP-5*	Homo sapiens cDNA clone IMAGE:5271145. [BC038776]	0.6924167	0.02598378
A_22_P00001377	*CBWD5*	Homo sapiens COBW domain containing 5 (CBWD5), transcript variant 1, mRNA [NM_001024916]	-0.5994673	0.047094904
A_24_P28619	*ILF3-AS1*	Homo sapiens ILF3 antisense RNA 1 (head to head) (ILF3-AS1), long non-coding RNA [NR_024333]	0.8412788	0.037221838
A_33_P3409159	*ANGEL2*	Homo sapiens angel homolog 2 (Drosophila) (ANGEL2), transcript variant 1, mRNA [NM_144567]	-0.9525596	0.048182435
A_33_P3396877	*SLC22A23*	Homo sapiens solute carrier family 22, member 23 (SLC22A23), transcript variant 1, mRNA [NM_015482]	-1.1876154	0.029808238
A_24_P148796	*RPP38*	Homo sapiens ribonuclease P/MRP 38kDa subunit (RPP38), transcript variant 1, mRNA [NM_183005]	0.9265835	0.03032242
A_21_P0013042	*MST1*	Homo sapiens macrophage stimulating 1 (hepatocyte growth factor-like) (MST1), mRNA [NM_020998]	-0.68378544	0.042440254
A_33_P3369631	*XLOC_l2_012748*	BROAD Institute lincRNA (XLOC_l2_012748), lincRNA [TCONS_l2_00024457]	-2.6683655	0.028192867
A_22_P00009478	*EIF3J*	Homo sapiens eukaryotic translation initiation factor 3, subunit J (EIF3J),	-1.0581592	0.02737376

		transcript variant 1, mRNA [NM_003758]		
A_33_P3239736	*lnc-MAGEA4-1*	601440791F1 NIH_MGC_72 Homo sapiens cDNA clone IMAGE:3915647 5', mRNA sequence [BE622226]	-0.7495477	0.038287252
A_23_P255076	*WDR44*	Homo sapiens WD repeat domain 44 (WDR44), transcript variant 1, mRNA [NM_019045]	0.92696536	0.045543194
A_32_P184518	*RWDD2A*	Homo sapiens RWD domain containing 2A (RWDD2A), mRNA [NM_033411]	-1.2295562	0.032155342
A_24_P401637	*RPL21*	Homo sapiens ribosomal protein L21 (RPL21), mRNA [NM_000982]	1.9934382	0.04367142
A_33_P3265030	*LOC100130856*	Homo sapiens cDNA FLJ38408 fis, clone FEBRA2009029. [AK095727]	-0.5900522	0.043732516
A_23_P83453	*GP1BB*	Homo sapiens glycoprotein Ib (platelet), beta polypeptide (GP1BB), mRNA [NM_000407]	-0.95530933	0.04983421
A_33_P3287883	*SMARCC1*	Homo sapiens SWI/SNF related, matrix associated, actin dependent regulator of chromatin, subfamily c, member 1 (SMARCC1), mRNA [NM_003074]	-0.9099972	0.034342222
A_33_P3352407	*LOC100133331*	Homo sapiens uncharacterized LOC100133331 (LOC100133331), long non-coding RNA [NR_028327]	-0.49030396	0.036297325
A_33_P3270451	*PEX13*	Homo sapiens peroxisomal biogenesis factor 13 (PEX13), mRNA [NM_002618]	-0.5294769	0.036364187
A_33_P3227284	*TXNDC5*	Homo sapiens thioredoxin domain containing 5 (endoplasmic reticulum) (TXNDC5), transcript variant 1, mRNA [NM_030810]	1.2981975	0.043147165
A_24_P81841	*OGT*	Homo sapiens O-linked N-acetylglucosamine (GlcNAc) transferase (OGT), transcript variant 1, mRNA [NM_181672]	-0.84999514	0.03639509
A_21_P0000737	*CDKN1B*	Homo sapiens cyclin-dependent kinase inhibitor 1B (p27, Kip1) (CDKN1B), mRNA [NM_004064]	-0.9996132	0.040442128
A_23_P97296	*ZNF571-AS1*	Homo sapiens ZNF571 antisense RNA 1 (ZNF571-AS1), transcript variant 1, long non-coding RNA [NR_038247]	-1.0568931	0.033287887
A_22_P00012377	*NECAP2*	Homo sapiens NECAP endocytosis associated 2 (NECAP2), transcript variant 1, mRNA [NM_018090]	-1.0412031	0.03668808
A_22_P00002697	*lnc-PRICKLE4-1*	LNCipedia lincRNA (lnc-PRICKLE4-1), lincRNA [lnc-PRICKLE4-1:1]	-1.7057347	0.033095628
A_33_P3237784	*lnc-C2-2*	Homo sapiens mRNA; cDNA DKFZp686H08213 (from clone DKFZp686H08213). [BX648392]	-1.0758168	0.035186797

A_24_P48318	*PORCN*	Homo sapiens porcupine homolog (Drosophila) (PORCN), transcript variant F, mRNA [NM_001282167]	-1.0654495	0.028192867
A_33_P3303015	*CWC25*	Homo sapiens CWC25 spliceosome-associated protein homolog (S. cerevisiae) (CWC25), transcript variant 1, mRNA [NM_017748]	2.0322833	0.044444222
A_21_P0000219	*RPS9*	Homo sapiens ribosomal protein S9 (RPS9), mRNA [NM_001013]	1.3549047	0.036041338
A_33_P3342126	*SNORD34*	Homo sapiens small nucleolar RNA, C/D box 34 (SNORD34), small nucleolar RNA [NR_000019]	-0.8492031	0.02453978
A_23_P148785	*PRDM8*	Homo sapiens PR domain containing 8 (PRDM8), transcript variant 1, mRNA [NM_020226]	-0.9769691	0.039287783
A_22_P00002241	*SFT2D2*	Homo sapiens SFT2 domain containing 2 (SFT2D2), mRNA [NM_199344]	-0.97068214	0.037923194
A_22_P00016154	*GNPAT*	Homo sapiens glyceronephosphate O-acyltransferase (GNPAT), mRNA [NM_014236]	2.1973221	0.03800715
A_33_P3241482	*LARGE-AS1*	Homo sapiens LARGE antisense RNA 1 (LARGE-AS1), transcript variant 2, long non-coding RNA [NR_038950]	-0.8751358	0.031972177
A_23_P409093	*ZNF346*	Homo sapiens zinc finger protein 346 (ZNF346), mRNA [NM_012279]	1.4931828	0.028573772
A_21_P0013573	*ANO4*	Homo sapiens anoctamin 4 (ANO4), transcript variant 3, mRNA [NM_178826]	-0.6301959	0.04831641
A_24_P348083	*LOC101928195*	PREDICTED: Homo sapiens monofunctional C1-tetrahydrofolate synthase, mitochondrial-like (LOC101928195), transcript variant X6, misc_RNA [XR_252557]	2.3151088	0.02453978
A_23_P123622	*LOC102723428*	RNA polymerase I transcription factor homolog (S. cerevisiae) pseudogene 1 [Source:HGNC Symbol;Acc:HGNC:30548] [ENST00000551681]	-2.0570064	0.04740209
A_23_P159663	*NPR2*	Homo sapiens natriuretic peptide receptor 2 (NPR2), mRNA [NM_003995]	1.6474879	0.03912857
A_23_P14493	*UXT*	Homo sapiens ubiquitously-expressed, prefoldin-like chaperone (UXT), transcript variant 2, mRNA [NM_004182]	-2.001793	0.03136619
A_22_P00009735	*DNAAF2*	Homo sapiens dynein, axonemal, assembly factor 2 (DNAAF2), transcript variant 1, mRNA [NM_018139]	-0.34080118	0.04958916

A_19_P00315869	*BTBD9*	Homo sapiens BTB (POZ) domain containing 9 (BTBD9), transcript variant 1, mRNA [NM_052893]	-1.2777417	0.02453978
A_23_P101093	*lnc-DET1-1*	Homo sapiens cDNA FLJ33447 fis, clone BRAMY1000098. [AK090766]	-0.8602914	0.045740407
A_23_P7783	*COPZ2*	Homo sapiens coatomer protein complex, subunit zeta 2 (COPZ2), mRNA [NM_016429]	-1.1840559	0.027751032
A_22_P00017709	*KDM3B*	Homo sapiens lysine (K)-specific demethylase 3B (KDM3B), mRNA [NM_016604]	-0.79857445	0.033091284
A_21_P0000077	*LOC101929172*	Homo sapiens uncharacterized LOC101929172 (LOC101929172), long non-coding RNA [NR_104677]	1.0027041	0.03403978
A_24_P295379	*DZIP1L*	Homo sapiens DAZ interacting zinc finger protein 1-like (DZIP1L), transcript variant 2, mRNA [NM_001170538]	-0.73122835	0.027942823
A_33_P3708364	*GPR107*	Homo sapiens G protein-coupled receptor 107 (GPR107), transcript variant 3, mRNA [NM_020960]	-0.752218	0.049235344
A_33_P3237135	*LOC644285*	Homo sapiens cDNA FLJ44905 fis, clone BRAMY3005912. [AK126853]	1.6377976	0.028573772
A_21_P0009195	*MMP2*	Homo sapiens matrix metallopeptidase 2 (gelatinase A, 72kDa gelatinase, 72kDa type IV collagenase) (MMP2), transcript variant 1, mRNA [NM_004530]	-0.85669106	0.034342222
A_24_P633902	*lnc-PHB-2*	LNCipedia lincRNA (lnc-PHB-2), lincRNA [lnc-PHB-2:1]	-0.95577073	0.029573848
A_24_P145066	*RNF115*	Homo sapiens ring finger protein 115 (RNF115), mRNA [NM_014455]	-0.48685673	0.049516298
A_24_P932418	*KIF1B*	Homo sapiens kinesin family member 1B (KIF1B), transcript variant 2, mRNA [NM_183416]	-0.7910135	0.02737376
A_33_P3243857	*AP2A2*	Homo sapiens adaptor-related protein complex 2, alpha 2 subunit (AP2A2), transcript variant 2, mRNA [NM_012305]	0.7988148	0.044286337
A_33_P3268564	*ADAM10*	Homo sapiens ADAM metallopeptidase domain 10 (ADAM10), mRNA [NM_001110]	-1.1980603	0.034698997
A_23_P130089	*NCK2*	Homo sapiens NCK adaptor protein 2 (NCK2), transcript variant 3, mRNA [NM_001004722]	-1.3661315	0.035515364
A_23_P66241	*IFT20*	Homo sapiens intraflagellar transport 20 (IFT20), transcript variant 2, mRNA [NM_174887]	-0.97373843	0.03974276

A_21_P0010421	*MT1M*	Homo sapiens metallothionein 1M (MT1M), mRNA [NM_176870]	-0.8112509	0.038003992
A_23_P38795	*lnc-PPARA-2*	LNCipedia lincRNA (lnc-PPARA-2), lincRNA [lnc-PPARA-2:1]	-1.8116473	0.02737376
A_33_P3334313	*FPR1*	Homo sapiens formyl peptide receptor 1 (FPR1), transcript variant 2, mRNA [NM_002029]	-1.1965723	0.028573772
A_33_P3414242	*ACTR3B*	Homo sapiens ARP3 actin-related protein 3 homolog B (yeast) (ACTR3B), transcript variant 1, mRNA [NM_020445]	1.8189659	0.039863296
A_33_P3357149	*MOG*	Homo sapiens myelin oligodendrocyte glycoprotein (MOG), transcript variant beta2, mRNA [NM_001008229]	1.516008	0.024948223
A_23_P317800	*PEAK1*	Homo sapiens pseudopodium-enriched atypical kinase 1 (PEAK1), mRNA [NM_024776]	-1.1859367	0.02737376
A_19_P00321872	*ANAPC4*	Homo sapiens anaphase promoting complex subunit 4 (ANAPC4), transcript variant 2, mRNA [NM_013367]	1.1643369	0.04041675
A_23_P60354	*LINC00958*	Homo sapiens long intergenic non-protein coding RNA 958 (LINC00958), long non-coding RNA [NR_038904]	-0.45703462	0.04881034
A_22_P00015457	*SMARCA2*	Homo sapiens SWI/SNF related, matrix associated, actin dependent regulator of chromatin, subfamily a, member 2 (SMARCA2), transcript variant 2, mRNA [NM_139045]	-0.91568995	0.04876218
A_23_P423926	*DCP1A*	Homo sapiens decapping mRNA 1A (DCP1A), transcript variant 3, mRNA [NM_001290205]	1.1700191	0.0442831
A_23_P250118	*SS18L1*	Homo sapiens synovial sarcoma translocation gene on chromosome 18-like 1 (SS18L1), transcript variant 1, mRNA [NM_198935]	-1.808577	0.04404228
A_22_P00022141	*HSPBAP1*	Homo sapiens HSPB (heat shock 27kDa) associated protein 1 (HSPBAP1), mRNA [NM_024610]	2.123454	0.024948223
A_23_P132915	*lnc-TLCD2-2*	LNCipedia lincRNA (lnc-TLCD2-2), lincRNA [lnc-TLCD2-2:1]	-1.0219185	0.028573772
A_23_P39263	*FAM114A1*	Homo sapiens family with sequence similarity 114, member A1 (FAM114A1), transcript variant 1, mRNA [NM_138389]	-1.1840721	0.025407597
A_33_P3412160	*ZNF57*	Homo sapiens zinc finger protein 57 (ZNF57), mRNA [NM_173480]	-0.7362767	0.048398785

A_24_P65060	*CLCC1*	Homo sapiens chloride channel CLIC-like 1 (CLCC1), transcript variant 1, mRNA [NM_001048210]	1.7876391	0.049758088
A_24_P204244	*MEX3B*	Homo sapiens mex-3 RNA binding family member B (MEX3B), mRNA [NM_032246]	-0.9155314	0.033723384
A_33_P3331491	*ANXA2P1*	Homo sapiens annexin A2 pseudogene 1 (ANXA2P1), non-coding RNA [NR_001562]	-1.9399114	0.02737376
A_33_P3361027	*LOC728392*	Homo sapiens uncharacterized LOC728392 (LOC728392), mRNA [NM_001162371]	-0.63316053	0.027019119
A_23_P166779	*TFIP11*	Homo sapiens tuftelin interacting protein 11 (TFIP11), transcript variant 1, mRNA [NM_001008697]	-2.8888814	0.027751032
A_23_P144476	*LINC00312*	Homo sapiens long intergenic non-protein coding RNA 312 (LINC00312), long non-coding RNA [NR_024065]	-1.0715625	0.03403978
A_33_P3575854	*SPRY1*	Homo sapiens sprouty homolog 1, antagonist of FGF signaling (Drosophila) (SPRY1), transcript variant 2, mRNA [NM_199327]	0.79154056	0.034210585
A_24_P205019	*LOC642361*	Homo sapiens uncharacterized LOC642361 (LOC642361), long non-coding RNA [NR_029407]	-1.0240064	0.037560765
A_22_P00007742	*ZNF250*	Homo sapiens zinc finger protein 250 (ZNF250), transcript variant 1, mRNA [NM_021061]	-3.3793635	0.03709602
A_33_P3274851	*LINGO1-AS1*	Homo sapiens LINGO1 antisense RNA 1 (LINGO1-AS1), long non-coding RNA [NR_045123]	-0.88037956	0.048502974
A_33_P3240702	*COX5B*	Homo sapiens cytochrome c oxidase subunit Vb (COX5B), mRNA [NM_001862]	-0.7258208	0.042639446
A_19_P00315764	*RBBP8*	Homo sapiens retinoblastoma binding protein 8 (RBBP8), transcript variant 1, mRNA [NM_002894]	-1.5840659	0.031526815
A_33_P3390778	*LOC644277*	PREDICTED: Homo sapiens uncharacterized LOC644277 (RP11-794P6.3), misc_RNA [XR_110541]	1.6878064	0.040161368
A_23_P62890	*TRIM46*	Homo sapiens tripartite motif containing 46 (TRIM46), transcript variant 2, mRNA [NM_001256599]	-1.0934038	0.02798001
A_24_P161914	*GBP1*	Homo sapiens guanylate binding protein 1, interferon-inducible (GBP1), mRNA [NM_002053]	-1.1327833	0.044952072

A_33_P3289996	*ZNF667*	Homo sapiens zinc finger protein 667 (ZNF667), transcript variant 1, mRNA [NM_022103]	-0.9332042	0.030589065
A_32_P184796	*USP45*	Homo sapiens ubiquitin specific peptidase 45 (USP45), mRNA [NM_001080481]	-1.871643	0.043712083
A_23_P65481	*RPLP0*	Homo sapiens ribosomal protein, large, P0 (RPLP0), transcript variant 2, mRNA [NM_053275]	1.0393853	0.039036095
A_23_P259357	*TEP1*	Homo sapiens telomerase-associated protein 1 (TEP1), mRNA [NM_007110]	-1.1499196	0.048030753
A_24_P37540	*SLC35E4*	Homo sapiens solute carrier family 35, member E4 (SLC35E4), mRNA [NM_001001479]	-0.5093454	0.024948223
A_24_P387609	*ARPC4-TTLL3*	Homo sapiens ARPC4-TTLL3 readthrough (ARPC4-TTLL3), mRNA [NM_001198793]	-0.81176186	0.039287783
A_33_P3288924	*ISCA1*	Homo sapiens iron-sulfur cluster assembly 1 (ISCA1), mRNA [NM_030940]	-1.2728157	0.04733213
A_32_P98975	*HUWE1*	Homo sapiens HECT, UBA and WWE domain containing 1, E3 ubiquitin protein ligase (HUWE1), mRNA [NM_031407]	-0.8718219	0.04041675
A_33_P3211834	*C15orf57*	Homo sapiens chromosome 15 open reading frame 57 (C15orf57), transcript variant 2, mRNA [NM_052849]	-1.3507935	0.038034573
A_22_P00007479	*SIRPB1*	PREDICTED: Homo sapiens signal-regulatory protein beta 1 (SIRPB1), mRNA [XM_006710250]	1.4451861	0.041929405
A_22_P00011540	*lnc-GTPBP1-1*	yf55b03.s1 Soares infant brain 1NIB Homo sapiens cDNA clone IMAGE:25867 3', mRNA sequence [R37239]	-0.770909	0.032893
A_24_P284584	*CCDC80*	Homo sapiens coiled-coil domain containing 80 (CCDC80), transcript variant 1, mRNA [NM_199511]	1.7199585	0.028573772
A_23_P501877	*ZNF559*	Homo sapiens zinc finger protein 559 (ZNF559), transcript variant 2, mRNA [NM_032497]	1.0336984	0.048295442
A_23_P142255	*ZFP64*	Homo sapiens ZFP64 zinc finger protein (ZFP64), transcript variant 1, mRNA [NM_018197]	1.325874	0.03032242
A_21_P0009285	*SHD*	Homo sapiens Src homology 2 domain containing transforming protein D (SHD), mRNA [NM_020209]	-2.5967438	0.027751032

A_22_P00015970	*lnc-C1QTNF1-1*	LNCipedia lincRNA (lnc-C1QTNF1-1), lincRNA [lnc-C1QTNF1-1:1]	-0.81437135	0.02737376
A_33_P3251283	*lnc-TEKT3-1*	Homo sapiens mRNA; cDNA DKFZp686P21116 (from clone DKFZp686P21116). [AL833462]	-1.1409461	0.031377234
A_23_P164179	*CTAGE4*	Homo sapiens CTAGE family, member 4 (CTAGE4), mRNA [NM_198495]	1.3004202	0.02453978
A_23_P137948	*TOB1*	Homo sapiens transducer of ERBB2, 1 (TOB1), transcript variant 1, mRNA [NM_005749]	1.5705526	0.047933746
A_23_P125423	*NENF*	Homo sapiens neudesin neurotrophic factor (NENF), transcript variant 1, mRNA [NM_013349]	-1.915946	0.03840762
A_23_P148015	*C1R*	Homo sapiens complement component 1, r subcomponent (C1R), mRNA [NM_001733]	-1.2703123	0.027751032
A_22_P00007205	*AXIN2*	Homo sapiens axin 2 (AXIN2), mRNA [NM_004655]	-0.4587912	0.03576752
A_23_P163148	*MIR17HG*	Homo sapiens miR-17-92 cluster host gene (non-protein coding) (MIR17HG), transcript variant 1, long non-coding RNA [NR_027350]	-0.6980719	0.047727138
A_23_P47077	*VIPAS39*	Homo sapiens VPS33B interacting protein, apical-basolateral polarity regulator, spe-39 homolog (VIPAS39), transcript variant 2, mRNA [NM_022067]	-1.5363281	0.042693015
A_23_P112512	*BAG3*	Homo sapiens BCL2-associated athanogene 3 (BAG3), mRNA [NM_004281]	-1.2179914	0.04250221
A_23_P374294	*SLC25A51*	Homo sapiens solute carrier family 25, member 51 (SLC25A51), transcript variant 1, mRNA [NM_033412]	1.7429544	0.027751032
A_23_P96383	*CCDC7*	Homo sapiens coiled-coil domain containing 7 (CCDC7), transcript variant 1, mRNA [NM_145023]	-0.8713905	0.044191804
A_24_P294982	*SRPX*	Homo sapiens sushi-repeat containing protein, X-linked (SRPX), transcript variant 1, mRNA [NM_006307]	-1.2193935	0.047137905
A_23_P211878	*VTA1*	Homo sapiens vesicle (multivesicular body) trafficking 1 (VTA1), transcript variant 1, mRNA [NM_016485]	-0.8409361	0.034342222
A_33_P3231267	*FLNB*	Homo sapiens filamin B, beta (FLNB), transcript variant 2, mRNA [NM_001457]	-0.44905618	0.04794156
A_23_P112260	*lnc-ITGA2-1*	LNCipedia lincRNA (lnc-ITGA2-1), lincRNA [lnc-ITGA2-1:5]	-0.8748889	0.032155342

A_23_P201996	*GNG10*	Homo sapiens guanine nucleotide binding protein (G protein), gamma 10 (GNG10), transcript variant 1, mRNA [NM_001017998]	-1.0324674	0.032557126
A_33_P3351474	*WAC*	Homo sapiens WW domain containing adaptor with coiled-coil (WAC), transcript variant 2, mRNA [NM_100264]	-1.8970506	0.027942823
A_23_P336565	*SMARCAD1*	Homo sapiens SWI/SNF-related, matrix-associated actin-dependent regulator of chromatin, subfamily a, containing DEAD/H box 1 (SMARCAD1), transcript variant 1, mRNA [NM_001128429]	1.706299	0.029808238
A_22_P00007751	*FAM185A*	Homo sapiens family with sequence similarity 185, member A (FAM185A), transcript variant 1, mRNA [NM_001145268]	-1.2871032	0.0335366
A_23_P12463	*lnc-HMGB2-3*	LNCipedia lincRNA (lnc-HMGB2-3), lincRNA [lnc-HMGB2-3:3]	-0.75977546	0.048681058
A_23_P217015	*QSOX1*	Homo sapiens quiescin Q6 sulfhydryl oxidase 1 (QSOX1), transcript variant 1, mRNA [NM_002826]	-0.6301723	0.039656427
A_33_P3367994	*SET*	Homo sapiens SET nuclear proto-oncogene (SET), transcript variant 2, mRNA [NM_003011]	-0.8521469	0.02453978
A_33_P3396635	*TAF8*	Homo sapiens TAF8 RNA polymerase II, TATA box binding protein (TBP)-associated factor, 43kDa (TAF8), mRNA [NM_138572]	0.367766	0.031526815
A_33_P3383029	*SSX3*	Homo sapiens synovial sarcoma, X breakpoint 3 (SSX3), mRNA [NM_021014]	-0.22043486	0.04217344
A_23_P2922	*MXI1*	Homo sapiens MAX interactor 1, dimerization protein (MXI1), transcript variant 2, mRNA [NM_130439]	1.1925843	0.029808238
A_23_P54846	*MBIP*	Homo sapiens MAP3K12 binding inhibitory protein 1 (MBIP), transcript variant 1, mRNA [NM_016586]	-0.83102673	0.032313347
A_23_P317207	*HERPUD1*	Homo sapiens homocysteine-inducible, endoplasmic reticulum stress-inducible, ubiquitin-like domain member 1 (HERPUD1), transcript variant 1, mRNA [NM_014685]	1.6168777	0.025153033
A_33_P3323718	*ATXN7L2*	Homo sapiens ataxin 7-like 2 (ATXN7L2), mRNA [NM_153340]	-2.8883	0.048182435
A_24_P343271	*UACA*	Homo sapiens uveal autoantigen with coiled-coil domains and ankyrin	-0.7155955	0.048760455

		repeats (UACA), transcript variant 2, mRNA [NM_001008224]		
A_22_P00013693	*RMND1*	Homo sapiens required for meiotic nuclear division 1 homolog (S. cerevisiae) (RMND1), transcript variant 1, mRNA [NM_017909]	-1.1629616	0.027942823
A_24_P169544	*LOC100507464*	PREDICTED: Homo sapiens uncharacterized LOC100507464 (LOC100507464), ncRNA [XR_108905]	1.6136959	0.026213482
A_32_P149174	*ZNF17*	Homo sapiens zinc finger protein 17 (ZNF17), mRNA [NM_006959]	1.8038982	0.039863296
A_32_P167122	*C6orf226*	Homo sapiens chromosome 6 open reading frame 226 (C6orf226), mRNA [NM_001008739]	-0.6267721	0.03668808
A_22_P00013109	*RCOR3*	Homo sapiens REST corepressor 3 (RCOR3), transcript variant 4, mRNA [NM_018254]	1.3870914	0.04792962
A_24_P11965	*lnc-RIOK1-2*	Homo sapiens cDNA FLJ12098 fis, clone HEMBB1002623. [AK022160]	-1.0125074	0.036443383
A_32_P190488	*MRFAP1*	Homo sapiens Morf4 family associated protein 1 (MRFAP1), transcript variant 1, mRNA [NM_033296]	-0.45116192	0.04445317
A_33_P3243887	*IPO5P1*	Homo sapiens importin 5 pseudogene 1 (IPO5P1), transcript variant 2, non-coding RNA [NR_103742]	-1.007302	0.03623899
A_23_P259135	*IL11*	Homo sapiens interleukin 11 (IL11), transcript variant 1, mRNA [NM_000641]	-1.349122	0.04167923
A_21_P0001900	*NCOA6*	Homo sapiens nuclear receptor coactivator 6 (NCOA6), transcript variant 1, mRNA [NM_014071]	-0.93350667	0.032779668
A_33_P3365878	*GACAT1*	Homo sapiens gastric cancer associated transcript 1 (non-protein coding) (GACAT1), transcript variant 1, long non-coding RNA [NR_126369]	1.0218751	0.035227668
A_23_P301896	*BMP8B*	Homo sapiens bone morphogenetic protein 8b (BMP8B), mRNA [NM_001720]	2.136566	0.04820357
A_33_P3247599	*PRDM2*	Homo sapiens PR domain containing 2, with ZNF domain (PRDM2), transcript variant 2, mRNA [NM_015866]	-0.54916185	0.045743782
A_23_P61633	*ZNF805*	Homo sapiens zinc finger protein 805 (ZNF805), transcript variant 1, mRNA [NM_001023563]	0.85072327	0.03777906
A_24_P98385	*TNK2*	Homo sapiens tyrosine kinase, non-receptor, 2 (TNK2), transcript variant 1, mRNA [NM_005781]	1.2480228	0.028573772

A_21_P0000427	*RNF20*	Homo sapiens ring finger protein 20, E3 ubiquitin protein ligase (RNF20), mRNA [NM_019592]	-1.1920364	0.031606767
A_33_P3268763	*SNORD114-30*	Homo sapiens small nucleolar RNA, C/D box 114-30 (SNORD114-30), small nucleolar RNA [NR_003223]	-1.1346027	0.045007665
A_23_P254120	*TMUB1*	Homo sapiens transmembrane and ubiquitin-like domain containing 1 (TMUB1), transcript variant 1, mRNA [NM_031434]	-0.60155123	0.04487451
A_22_P00002166	*FBXO9*	Homo sapiens F-box protein 9 (FBXO9), transcript variant 2, mRNA [NM_033480]	-1.1223623	0.029808238
A_23_P12989	*FAM229A*	Homo sapiens family with sequence similarity 229, member A (FAM229A), mRNA [NM_001167676]	0.8772452	0.024948223
A_23_P416395	*PRDX5*	Homo sapiens peroxiredoxin 5 (PRDX5), transcript variant 1, mRNA [NM_012094]	-0.84354067	0.04817637
A_33_P3341509	*STC2*	Homo sapiens stanniocalcin 2 (STC2), mRNA [NM_003714]	-1.0631883	0.031965
A_24_P154006	*TRIM34*	Homo sapiens tripartite motif containing 34 (TRIM34), transcript variant 3, mRNA [NM_130390]	-0.9404401	0.04620816
A_23_P134755	*NDUFB9*	Homo sapiens NADH dehydrogenase (ubiquinone) 1 beta subcomplex, 9, 22kDa (NDUFB9), transcript variant 1, mRNA [NM_005005]	-3.2915783	0.040911164
A_23_P89187	*TRPS1*	Homo sapiens trichorhinophalangeal syndrome I (TRPS1), transcript variant 1, mRNA [NM_014112]	-1.5697494	0.029808238
A_23_P131240	*LASP1*	Homo sapiens LIM and SH3 protein 1 (LASP1), transcript variant 1, mRNA [NM_006148]	-0.98215973	0.04942863
A_23_P108028	*UBXN2A*	Homo sapiens UBX domain protein 2A (UBXN2A), mRNA [NM_181713]	-0.8866053	0.035278592
A_22_P00007468	*ZNF146*	Homo sapiens zinc finger protein 146 (ZNF146), transcript variant 1, mRNA [NM_007145]	-1.858863	0.04462891
A_23_P108932	*RBPMS-AS1*	Homo sapiens RBPMS antisense RNA 1 (RBPMS-AS1), long non-coding RNA [NR_046205]	-1.0699642	0.03343686
A_24_P132787	*RPL23AP32*	Homo sapiens ribosomal protein L23a pseudogene 32 (RPL23AP32), non-coding RNA [NR_002229]	-0.8107175	0.028573772
A_33_P3283485	*RAB18*	Homo sapiens RAB18, member RAS oncogene family (RAB18), transcript variant 1, mRNA [NM_021252]	-0.6414809	0.031603426

A_23_P170518	*GALNT16*	Homo sapiens polypeptide N-acetylgalactosaminyltransferase 16 (GALNT16), transcript variant 2, mRNA [NM_020692]	-2.3966055	0.02737376
A_21_P0012886	*DYM*	Homo sapiens dymeclin (DYM), mRNA [NM_017653]	-0.9167347	0.02598378
A_33_P3320548	*XLOC_l2_011911*	BROAD Institute lincRNA (XLOC_l2_011911), lincRNA [TCONS_l2_00022753]	1.8300402	0.027751032
A_21_P0013615	*NUPL2*	Homo sapiens nucleoporin like 2 (NUPL2), mRNA [NM_007342]	-1.420629	0.043215733
A_19_P00319682	*XLOC_l2_015098*	BROAD Institute lincRNA (XLOC_l2_015098), lincRNA [TCONS_l2_00029170]	-1.5162549	0.03220128
A_33_P3255165	*LRRFIP1*	Homo sapiens leucine rich repeat (in FLII) interacting protein 1 (LRRFIP1), transcript variant 1, mRNA [NM_001137550]	-2.2504358	0.029573848
A_23_P141549	*ACTA2-AS1*	Homo sapiens ACTA2 antisense RNA 1 (ACTA2-AS1), long non-coding RNA [NR_125373]	-1.111368	0.035515364
A_33_P3218955	*RPS7*	Homo sapiens ribosomal protein S7 (RPS7), mRNA [NM_001011]	1.9994973	0.03126305
A_24_P221883	*KDM6A*	Homo sapiens lysine (K)-specific demethylase 6A (KDM6A), transcript variant 1, mRNA [NM_001291415]	-1.5450763	0.038287252
A_23_P127475	*PARL*	presenilin associated, rhomboid-like (PARL) [NM_018622]	2.1681042	0.040338222
A_24_P147540	*CCS*	copper chaperone for superoxide dismutase (CCS) [NM_005125]	-0.69281125	0.031606767
A_23_P124837	*PRR36*	proline rich 36 (PRR36) [NM_001190467]	-1.2886072	0.04855821
A_33_P3305158	*LRP1*	low density lipoprotein receptor-related protein 1 (LRP1) [NM_002332]	-0.93254274	0.040200267
A_23_P31844	*ZNF621*	zinc finger protein 621 (ZNF621) [NM_198484]	-1.0991669	0.028573772
A_33_P3287745	*ATP6V1B2*	ATPase, H+ transporting, lysosomal 56/58kDa, V1 subunit B2 (ATP6V1B2) [NM_001693]	-2.60268	0.024948223
A_23_P65797	*KAT8*	K(lysine) acetyltransferase 8 (KAT8) [NM_182958]	-1.7180539	0.047938656
A_23_P151093	*KLHL25*	kelch-like family member 25 (KLHL25) [NM_022480]	-0.9360688	0.03136619
A_23_P136325	*YARS2*	tyrosyl-tRNA synthetase 2, mitochondrial (YARS2) [NM_001040436]	-1.167379	0.03403978
A_32_P5480	*WIPF2*	WAS/WASL interacting protein family, member 2 (WIPF2) [NM_133264]	-0.46065575	0.030362261

A_22_P00022587	*CERS6*	ceramide synthase 6 (CERS6) [NM_203463]	-0.56058216	0.049758088
A_33_P3344477	*lnc-NUMB-2*	BX101396 Soares_testis_NHT Homo sapiens cDNA clone IMAGp998D114108 [BX101396]	-1.1171238	0.048681058
A_23_P156890	*FERMT2*	fermitin family member 2 (FERMT2)[NM_001134999]	-0.8261066	0.045638688
A_33_P3345501	*TCF21*	transcription factor 21 (TCF21)[NM_003206]	-2.8490486	0.046072803
A_33_P3407895	*CD58*	CD58 molecule (CD58)[NM_001779]	-1.474921	0.030362261
A_33_P3423700	*RINL*	Ras and Rab interactor-like (RINL) [NM_001195833]	4.2048016	0.03323599
A_21_P0011874	*LOC100131829*	cDNA FLJ42008 fis, clone SPLEN2031724. [AK124002]	-1.2502238	0.034478147
A_33_P3299140	*XLOC_l2_007767*	BROAD Institute lincRNA (XLOC_l2_007767), lincRNA [TCONS_l2_00014252]	-0.9286828	0.031126594
A_23_P25989	*GTF3C5*	general transcription factor IIIC, polypeptide 5, 63kDa [ENST00000435745]	-1.3362757	0.036726505
A_24_P337397	*SETD3*	SET domain containing 3 (SETD3), [NM_032233]	-0.4270629	0.04371038
A_23_P60166	*ANKRA2*	ankyrin repeat, family A (RFXANK-like), 2 (ANKRA2) [NM_023039]	1.7751162	0.02598378
A_22_P00014639	*DEPTOR*	DEP domain containing MTOR-interacting protein (DEPTOR)[NM_022783]	2.853012	0.024948223
A_23_P115608	*lnc-SLC17A9-1*	LNCipedia lincRNA (lnc-SLC17A9-1), lincRNA [lnc-SLC17A9-1:1]	-2.065002	0.033091284
A_23_P79661	*ARHGAP21*	Rho GTPase activating protein 21 (ARHGAP21) [NM_020824]	-1.5746799	0.030362261
A_21_P0010844	*CCDC93*	coiled-coil domain containing 93 (CCDC93)[NM_019044]	-2.9756284	0.030385435
A_23_P115366	*XLOC_l2_001760*	BROAD Institute lincRNA (XLOC_l2_001760), lincRNA [TCONS_l2_00003281]	3.7082295	0.028573772
A_23_P254648	*CMPK1*	cytidine monophosphate (UMP-CMP) kinase 1, cytosolic (CMPK1)[NM_016308]	-0.8382011	0.047785223
A_23_P77568	*FBXW2*	F-box and WD repeat domain containing 2 (FBXW2), [NM_012164]	-1.7425125	0.024948223
A_24_P916614	*EIF3C*	eukaryotic translation initiation factor 3, subunit C (EIF3C) [NM_001037808]	1.7361699	0.027189178
A_23_P327069	*PTBP3*	polypyrimidine tract binding protein 3 (PTBP3) [NM_005156]	-1.0000535	0.028573772
A_24_P397247	*KIAA0232*	KIAA0232 (KIAA0232) [NM_014743]	-0.81224036	0.027942823

A_24_P88850	*SDAD1*	SDA1 domain containing 1 (SDAD1) [NM_018115]	-0.643553	0.049980104
A_23_P35343	*MRAS*	muscle RAS oncogene homolog (MRAS) [NM_012219]	-0.7079067	0.024948223
A_23_P82065	*ZNF248*	zinc finger protein 248 (ZNF248)[NM_021045]	-1.3585813	0.049730826
A_33_P3247175	*ELOVL4*	ELOVL fatty acid elongase 4 (ELOVL4) [NM_022726]	-0.9563474	0.03297842
A_33_P3286254	*C4orf47*	chromosome 4 open reading frame 47 (C4orf47) [NM_001114357]	-0.9531695	0.044364784
A_23_P358995	*AP3S1*	adaptor-related protein complex 3, sigma 1 subunit (AP3S1) [NM_001284]	-0.86660975	0.041172817
A_33_P3314192	*IBTK*	inhibitor of Bruton agammaglobulinemia tyrosine kinase (IBTK) [NM_015525]	-0.7595598	0.03485871
A_23_P476	*PACSIN2*	protein kinase C and casein kinase substrate in neurons 2 (PACSIN2) [NM_001184970]	-2.7424645	0.033412892
A_24_P83808	*MPZL1*	myelin protein zero-like 1 (MPZL1) [NM_003953]	-0.8119907	0.034698997
A_33_P3256282	*TRHDE-AS1*	TRHDE antisense RNA 1 (TRHDE-AS1) [NR_026836]	-1.1939632	0.045400333
A_22_P00021229	*KRTAP10-2*	keratin associated protein 10-2 [Source:HGNC Symbol;Acc:HGNC:22967] [ENST00000391621]	-0.46359396	0.046256438
A_24_P99838	*lnc-SHISA4-1*	cDNA FLJ45279 fis, clone BRHIP3001338. [AK127212]	-0.8022657	0.028619371
A_33_P3316223	*ZNF223*	zinc finger protein 223 (ZNF223) [NM_013361]	1.9006711	0.028573772
A_33_P3282252	*SNAPC1*	small nuclear RNA activating complex, polypeptide 1, 43kDa (SNAPC1) [NM_003082]	1.4136139	0.032286324
A_23_P77562	*CARS*	Homo sapiens cysteinyl-tRNA synthetase (CARS)[NM_001014437]	-0.99378145	0.03126305
A_22_P00017827	*TMEM219*	transmembrane protein 219 (TMEM219), transcript variant 2, mRNA [NM_194280]	-1.2961087	0.035560552
A_22_P00017244	*lnc-ZC3H12D-2*	RST31404 Athersys RAGE Library Homo sapiens cDNA, mRNA sequence [BG211832]	0.81661916	0.042907383
A_33_P3292241	*TPM2*	tropomyosin 2 (beta) (TPM2), transcript variant Tpm2.1, [NM_213674]	-0.75254387	0.047266874
A_23_P390704	*LOC100130285*	PREDICTED: Homo sapiens uncharacterized LOC100130285 (LOC100130285), misc_RNA [XR_424754]	1.7639865	0.03136619

A_33_P3311205	*STK38*	serine/threonine kinase 38 (STK38) [NM_007271]	-0.78964853	0.047043864
A_23_P76245	*PRPF40B*	PRP40 pre-mRNA processing factor 40 homolog B (S. cerevisiae) (PRPF40B) [NM_012272]	1.5287892	0.042850092
A_23_P433079	*SCN8A*	sodium channel, voltage gated, type VIII alpha subunit (SCN8A) [NM_014191]	1.1677563	0.028056882
A_33_P3248644	*DNAJC14*	DnaJ (Hsp40) homolog, subfamily C, member 14 (DNAJC14) [NM_032364]	-1.5514996	0.04456819
A_24_P371303	*QARS*	glutaminyl-tRNA synthetase (QARS) [NM_005051]	-0.67786103	0.042907383
A_24_P303480	*FAM208A*	family with sequence similarity 208, member A (FAM208A)[NM_015224]	-1.0338731	0.02598378
A_33_P3318746	*RAB32*	RAB32, member RAS oncogene family (RAB32) [NM_006834]	-0.42247584	0.029088594

Table S2. Differentially expressed genes untreated vs siRNA2 treated FBs

Probe set ID	Gene symbol	Gene name	2Log Fold Change	p.value
A_23_P208706	***CPED1***	Homo sapiens cadherin-like and PC-esterase domain containing 1 (CPED1), [NM_001105533]	-0.70044076	0.011517759
A_24_P333494	*CHAC2*	Homo sapiens ChaC, cation transport regulator homolog 2 (E. coli) (CHAC2), [NM_001008708]	0.47609758	0.013963695
A_33_P3330991	*BAX*	Homo sapiens BCL2-associated X protein (BAX), [NM_138764]	-1.4769621	0.0036517929
A_23_P83436	*FBXO42*	Homo sapiens F-box protein 42 (FBXO42), mRNA [NM_018994]	-0.6583867	0.0108836265
A_23_P101084	*PEPD*	Homo sapiens peptidase D (PEPD), transcript variant 1, mRNA [NM_000285]	-0.7448556	0.0098884255
A_23_P162120	*lnc-C21orf58-1*	Homo sapiens cDNA FLJ45665 fis, clone CTONG2027959. [AK127572]	-0.6424021	0.016435314
A_24_P291426	*SPATA22*	Homo sapiens spermatogenesis associated 22 (SPATA22), [NM_032598]	4.2906613	0.0032795896
A_23_P52610	*NUMA1*	Homo sapiens nuclear mitotic apparatus protein 1 (NUMA1), [NM_006185]	0.9922859	0.010648535
A_24_P349039	*REV1*	Homo sapiens REV1, polymerase (DNA directed) (REV1), [NM_016316]	-0.74829	0.009924457
A_33_P3227880	*DDB2*	Homo sapiens damage-specific DNA binding protein 2, 48kDa (DDB2), mRNA [NM_000107]	-1.0765746	0.008427824
A_22_P00021565	*ARHGAP31*	Homo sapiens Rho GTPase activating protein 31 (ARHGAP31), mRNA [NM_020754]	-1.0009328	0.0075709717
A_23_P215406	*BTN3A2*	Homo sapiens butyrophilin, subfamily 3, member A2 (BTN3A2), [NM_001197247]	-0.99151516	0.006191503
A_24_P92952	*SNORA78*	small nucleolar RNA host gene 9 (non-protein coding) [Source:HGNC Symbol;Acc:HGNC:33102] [ENST00000531523]	-0.6092805	0.00832791
A_32_P132317	*RAC1*	Homo sapiens ras-related C3 botulinum toxin substrate 1 (rho family, small GTP binding protein Rac1) (RAC1), transcript variant Rac1b, mRNA [NM_018890]	-0.72617	0.010839213

A_32_P120604	*ARID1A*	Homo sapiens AT rich interactive domain 1A (SWI-like) (ARID1A), transcript variant 1, mRNA [NM_006015]	0.7151692	0.011741186
A_23_P121806	*GPR155*	Homo sapiens G protein-coupled receptor 155 (GPR155), transcript variant 1, mRNA [NM_001033045]	-1.4604946	0.0039659217
A_23_P120845	*SNX29*	Homo sapiens sorting nexin 29, mRNA (cDNA clone IMAGE:5177668), partial cds. [BC029857]	-1.1827585	0.0048641856
A_23_P156708	*ENOPH1*	Homo sapiens enolase-phosphatase 1 (ENOPH1), transcript variant 1, mRNA [NM_021204]	-0.6535342	0.013793035
A_33_P3220445	*XBP1*	Homo sapiens X-box binding protein 1 (XBP1), transcript variant 1, mRNA [NM_005080]	-0.8897166	0.007801743
A_23_P123672	*TNXB*	Homo sapiens tenascin XB (TNXB), transcript variant XB-S, mRNA [NM_032470]	-2.529717	0.003956951
A_24_P288915	*SMAD5*	Homo sapiens SMAD family member 5 (SMAD5), transcript variant 2, mRNA [NM_001001419]	-0.7701853	0.015419179
A_33_P3417950	*TDRD7*	Homo sapiens tudor domain containing 7 (TDRD7), transcript variant 1, mRNA [NM_014290]	-1.0087049	0.008160913
A_33_P3763846	*AKAP8*	Homo sapiens A kinase (PRKA) anchor protein 8 (AKAP8), mRNA [NM_005858]	-0.44052717	0.01313138
A_19_P00319917	*GADD45G*	Homo sapiens growth arrest and DNA-damage-inducible, gamma (GADD45G), mRNA [NM_006705]	0.93060774	0.007415985
A_23_P71146	*MAPK12*	Homo sapiens mitogen-activated protein kinase 12 (MAPK12), transcript variant 2, mRNA [NM_001303252]	1.7206231	0.0108264685
A_21_P0000259	*NUP50-AS1*	Homo sapiens NUP50 antisense RNA 1 (head to head) (NUP50-AS1), transcript variant 1, long non-coding RNA [NR_038956]	-0.97299683	0.010527615
A_33_P3589217	*POLD2*	Homo sapiens polymerase (DNA directed), delta 2, accessory subunit (POLD2), transcript variant 2, mRNA [NM_006230]	1.0768609	0.0057028057
A_33_P3231739	*SNORD25*	Homo sapiens small nucleolar RNA, C/D box 25 (SNORD25), small nucleolar RNA [NR_002565]	1.0952668	0.007923661
A_33_P3223467	*SLC25A6*	Homo sapiens solute carrier family 25 (mitochondrial carrier; adenine nucleotide translocator), member 6 (SLC25A6), mRNA [NM_001636]	1.0077997	0.0060495283
A_23_P29204	*ELOVL2*	Homo sapiens ELOVL fatty acid elongase 2 (ELOVL2), mRNA [NM_017770]	-0.93687844	0.004833
A_23_P146497	*VCPIP1*	Homo sapiens valosin containing protein (p97)/p47 complex interacting protein 1 (VCPIP1), mRNA [NM_025054]	-1.0205032	0.005449271

A_23_P116898	*MTFP1*	Homo sapiens mitochondrial fission process 1 (MTFP1), transcript variant 1, mRNA [NM_016498]	0.525302	0.010909709
A_33_P3240951	*PPP1R26*	Homo sapiens protein phosphatase 1, regulatory subunit 26 (PPP1R26), mRNA [NM_014811]	-0.86006737	0.015431974
A_33_P3383606	*A2M*	Homo sapiens alpha-2-macroglobulin (A2M), mRNA [NM_000014]	-1.8271095	0.0071150893
A_23_P253074	*DPF3*	Homo sapiens D4, zinc and double PHD fingers, family 3 (DPF3), transcript variant 4, mRNA [NM_001280544]	0.9846692	0.00856985
(+)E1A_r60_a22	*KCP*	Homo sapiens kielin/chordin-like protein (KCP), transcript variant 1, mRNA [NM_001135914]	0.4551999	0.015502214
A_22_P00015397	*PIWIL2*	Homo sapiens piwi-like RNA-mediated gene silencing 2 (PIWIL2), transcript variant 2, mRNA [NM_018068]	2.9299266	0.004359316
A_23_P118392	*lnc-SRP54-1*	Homo sapiens cDNA clone IMAGE:4823416. [BC039097]	-0.5001618	0.01331803
A_24_P76879	*CORO1B*	Homo sapiens coronin, actin binding protein, 1B (CORO1B), transcript variant 1, mRNA [NM_020441]	1.027989	0.009848541
A_33_P3329467	*RASD1*	Homo sapiens RAS, dexamethasone-induced 1 (RASD1), transcript variant 1, mRNA [NM_016084]	0.8097829	0.0059353677
A_21_P0012155	*TSPYL1*	Homo sapiens TSPY-like 1 (TSPYL1), mRNA [NM_003309]	-0.67527866	0.014625823
A_19_P00318883	*STYXL1*	Homo sapiens serine/threonine/tyrosine interacting-like 1 (STYXL1), mRNA [NM_016086]	-0.72526115	0.009779963
A_23_P213014	*APTR*	Homo sapiens Alu-mediated CDKN1A/p21 transcriptional regulator (non-protein coding) (APTR), long non-coding RNA [NR_038361]	-0.8537056	0.014525657
A_23_P102364	*HEATR5A*	Homo sapiens HEAT repeat containing 5A (HEATR5A), mRNA [NM_015473]	-0.66512305	0.013823839
A_33_P3210379	*SLC2A9*	Homo sapiens solute carrier family 2 (facilitated glucose transporter), member 9 (SLC2A9), transcript variant 2, mRNA [NM_001001290]	-2.2600362	0.005255513
A_23_P98350	*NGEF*	Homo sapiens neuronal guanine nucleotide exchange factor (NGEF), transcript variant 1, mRNA [NM_019850]	-3.1702201	0.002819256
A_23_P61580	*SCGB3A1*	Homo sapiens secretoglobin, family 3A, member 1 (SCGB3A1), mRNA [NM_052863]	0.7185824	0.008242901
A_23_P119943	*BIRC3*	Homo sapiens baculoviral IAP repeat containing 3 (BIRC3), transcript variant 1, mRNA [NM_001165]	0.6983158	0.008536083
A_23_P102937	*NSUN6*	Homo sapiens NOP2/Sun domain family, member 6 (NSUN6), mRNA [NM_182543]	-0.33672148	0.013481679

A_21_P0000766	*IGFBP2*	Homo sapiens insulin-like growth factor binding protein 2, 36kDa (IGFBP2), mRNA [NM_000597]	-5.49483	0.0031827379
A_22_P00001741	*SUMO3*	Homo sapiens small ubiquitin-like modifier 3 (SUMO3), transcript variant 1, mRNA [NM_006936]	-0.6763079	0.005766287
A_33_P3397763	*TEKT4P2*	Homo sapiens tektin 4 pseudogene 2 (TEKT4P2), transcript variant 2, non-coding RNA [NR_038328]	-0.89285105	0.006573993
A_33_P3272189	*C11orf95*	Homo sapiens chromosome 11 open reading frame 95 (C11orf95), mRNA [NM_001144936]	0.6293619	0.010561557
A_22_P00016091	*TNFSF9*	Homo sapiens tumor necrosis factor (ligand) superfamily, member 9 (TNFSF9), mRNA [NM_003811]	-0.83127797	0.0072927927
A_23_P126623	*MFSD9*	Homo sapiens major facilitator superfamily domain containing 9 (MFSD9), mRNA [NM_032718]	-1.1146184	0.012830978
A_33_P3397418	*lnc-THOC1-1*	LNCipedia lincRNA (lnc-THOC1-1), lincRNA [lnc-THOC1-1:1]	-0.58319306	0.013327314
A_23_P81399	*PGD*	Homo sapiens phosphogluconate dehydrogenase (PGD), mRNA [NM_002631]	1.303128	0.007797447
A_24_P376787	*ZC3HAV1*	Homo sapiens zinc finger CCCH-type, antiviral 1 (ZC3HAV1), transcript variant 1, mRNA [NM_020119]	-1.0661266	0.00556941
A_23_P256632	*SQSTM1*	Homo sapiens sequestosome 1 (SQSTM1), transcript variant 1, mRNA [NM_003900]	1.8354533	0.010545495
A_33_P3412317	*ZNF496*	Homo sapiens zinc finger protein 496 (ZNF496), mRNA [NM_032752]	1.1035042	0.016498217
A_22_P00020384	*PGGT1B*	Homo sapiens protein geranylgeranyltransferase type I, beta subunit (PGGT1B), mRNA [NM_005023]	-0.603736	0.0117289275
A_33_P3356371	*SYNJ2BP-COX16*	Homo sapiens SYNJ2BP-COX16 readthrough (SYNJ2BP-COX16), transcript variant 1, mRNA [NM_001202547]	-0.7214455	0.004055852
A_19_P00321739	*RPL22*	Homo sapiens ribosomal protein L22 (RPL22), mRNA [NM_000983]	-0.4072657	0.005581195
A_23_P320739	*LINC00472*	Homo sapiens long intergenic non-protein coding RNA 472 (LINC00472), transcript variant 4, long non-coding RNA [NR_121614]	0.42587036	0.01073543
A_23_P149200	*MGST2*	Homo sapiens microsomal glutathione S-transferase 2 (MGST2), transcript variant 1, mRNA [NM_002413]	-1.3907036	0.007323471
A_23_P202860	*MEF2C*	Homo sapiens myocyte enhancer factor 2C (MEF2C), transcript variant 1, mRNA [NM_002397]	-1.6558856	0.0044707763
A_23_P160729	*CDC20*	Homo sapiens cell division cycle 20 (CDC20), mRNA [NM_001255]	0.9287774	0.008995857

A_33_P3388491	*CYP2R1*	Homo sapiens cytochrome P450, family 2, subfamily R, polypeptide 1 (CYP2R1), mRNA [NM_024514]	-1.8289489	0.0039497027
A_19_P00321556	*AP4B1*	Homo sapiens adaptor-related protein complex 4, beta 1 subunit (AP4B1), transcript variant 1, mRNA [NM_006594]	-0.7329488	0.014173936
A_23_P416212	*PQLC2*	Homo sapiens PQ loop repeat containing 2 (PQLC2), transcript variant 1, mRNA [NM_001040125]	1.0490125	0.015084378
A_23_P106145	*LINC01204*	Homo sapiens long intergenic non-protein coding RNA 1204 (LINC01204), transcript variant 2, long non-coding RNA [NR_104645]	-1.9408175	0.00641094
A_23_P253389	*HSPB9*	Homo sapiens heat shock protein, alpha-crystallin-related, B9 (HSPB9), mRNA [NM_033194]	0.98637277	0.012170573
A_24_P250499	*ERO1L*	ERO1-like (S. cerevisiae) [Source:HGNC Symbol;Acc:HGNC:13280] [ENST00000555069]	-0.7954559	0.0052959127
A_21_P0010703	*SLC4A2*	Homo sapiens solute carrier family 4 (anion exchanger), member 2 (SLC4A2), transcript variant 1, mRNA [NM_003040]	1.4705176	0.0052084886
A_23_P10121	*RPRD1B*	Homo sapiens regulation of nuclear pre-mRNA domain containing 1B (RPRD1B), mRNA [NM_021215]	-0.6038518	0.0067226742
A_22_P00025959	*lnc-C1orf177-2*	LNCipedia lincRNA (lnc-C1orf177-2), lincRNA [lnc-C1orf177-2:2]	0.9768491	0.01613151
A_23_P67992	*SFRP1*	Homo sapiens secreted frizzled-related protein 1 (SFRP1), mRNA [NM_003012]	1.2030308	0.0034310205
A_23_P431939	*PTPN14*	Homo sapiens protein tyrosine phosphatase, non-receptor type 14 (PTPN14), mRNA [NM_005401]	0.38173252	0.0079484265
A_24_P144377	*C1D*	Homo sapiens C1D nuclear receptor corepressor (C1D), transcript variant 1, mRNA [NM_006333]	-0.39747304	0.011048744
A_23_P133424	*MR1*	Homo sapiens major histocompatibility complex, class I-related (MR1), transcript variant 1, mRNA [NM_001531]	-0.95828867	0.011672903
A_23_P157007	*DEXI*	Homo sapiens Dexi homolog (mouse) (DEXI), mRNA [NM_014015]	-0.77468634	0.0053354
A_24_P686965	*SKP1*	Homo sapiens S-phase kinase-associated protein 1 (SKP1), transcript variant 1, mRNA [NM_006930]	-0.48402593	0.011743996
A_23_P16652	*TMEM176B*	Homo sapiens transmembrane protein 176B (TMEM176B), transcript variant 1, mRNA [NM_014020]	4.915075	0.014436519
A_23_P52336	*SH2D5*	Homo sapiens SH2 domain containing 5 (SH2D5), transcript variant 1, mRNA [NM_001103161]	1.2337608	0.0053387037

A_33_P3251108	*ZNF555*	Homo sapiens zinc finger protein 555 (ZNF555), transcript variant 1, mRNA [NM_152791]	-1.4882494	0.008706018
A_33_P3332547	*UNC5B*	Homo sapiens unc-5 homolog B (C. elegans) (UNC5B), transcript variant 1, mRNA [NM_170744]	0.8231432	0.008007196
A_23_P125233	*SLC33A1*	Homo sapiens solute carrier family 33 (acetyl-CoA transporter), member 1 (SLC33A1), transcript variant 2, mRNA [NM_001190992]	-0.8731644	0.005822303
A_21_P0012654	*IQCJ-SCHIP1*	Homo sapiens IQCJ-SCHIP1 readthrough (IQCJ-SCHIP1), transcript variant 1, mRNA [NM_001197113]	-1.3620689	0.004846843
A_21_P0011354	*CNN1*	Homo sapiens calponin 1, basic, smooth muscle (CNN1), mRNA [NM_001299]	-2.15633	0.0023340243
A_23_P427703	*LOC101928803*	PREDICTED: Homo sapiens uncharacterized LOC101928803 (LOC101928803), transcript variant X2, ncRNA [XR_251551]	-1.150672	0.008454407
A_23_P61447	*XLOC_l2_004844*	BROAD Institute lincRNA (XLOC_l2_004844), lincRNA [TCONS_l2_00008932]	1.516608	0.006769596
A_24_P355967	*MT1L*	Homo sapiens metallothionein 1L (gene/pseudogene) (MT1L), non-coding RNA [NR_001447]	1.6284982	0.0036402496
A_32_P86245	*ETFDH*	Homo sapiens electron-transferring-flavoprotein dehydrogenase (ETFDH), transcript variant 1, mRNA [NM_004453]	-1.1346524	0.0032554762
A_24_P227585	*HTR2A*	Homo sapiens 5-hydroxytryptamine (serotonin) receptor 2A, G protein-coupled (HTR2A), transcript variant 1, mRNA [NM_000621]	-3.8821058	0.0037353234
A_33_P3339070	*EFHC1*	Homo sapiens EF-hand domain (C-terminal) containing 1 (EFHC1), transcript variant A, mRNA [NM_018100]	-1.1853187	0.011110267
A_24_P190541	*GPALPP1*	Homo sapiens GPALPP motifs containing 1 (GPALPP1), mRNA [NM_018559]	-0.59137005	0.014104453
A_24_P79054	*LINC00704*	Homo sapiens long intergenic non-protein coding RNA 704 (LINC00704), long non-coding RNA [NR_024475]	1.9103131	0.012554233
A_24_P921321	*BRWD1*	Homo sapiens bromodomain and WD repeat domain containing 1 (BRWD1), transcript variant 1, mRNA [NM_018963]	-0.81679606	0.0072907214
A_23_P10785	*TGFB1*	Homo sapiens transforming growth factor, beta 1 (TGFB1), mRNA [NM_000660]	1.3437598	0.00915229
A_21_P0002073	*PTPRJ*	Homo sapiens protein tyrosine phosphatase, receptor type, J (PTPRJ), transcript variant 1, mRNA [NM_002843]	-0.98936677	0.003365343
A_33_P3244818	*VTI1A*	Homo sapiens vesicle transport through interaction with t-SNAREs 1A (VTI1A), mRNA [NM_145206]	-0.89107025	0.008159728

A_24_P129632	*RSBN1L*	Homo sapiens round spermatid basic protein 1-like (RSBN1L), mRNA [NM_198467]	-0.7903685	0.013706757
A_23_P311192	*PDXK*	pyridoxal (pyridoxine, vitamin B6) kinase [Source:HGNC Symbol;Acc:HGNC:8819] [ENST00000438837]	1.6260008	0.00511559
A_23_P90895	*DLG5*	Homo sapiens discs, large homolog 5 (Drosophila) (DLG5), [NM_004747]	-0.6060345	0.005800329
A_23_P42507	*SPTLC1*	Homo sapiens serine palmitoyltransferase, long chain base subunit 1 (SPTLC1), [NM_178324]	-1.0295069	0.005822303
A_22_P00012484	*PPP1R21*	Homo sapiens protein phosphatase 1, regulatory subunit 21 (PPP1R21), [NM_152994]	-0.90377283	0.0056002573
A_23_P55319	*PPIL4*	Homo sapiens peptidylprolyl isomerase (cyclophilin)-like 4 (PPIL4), [NM_139126]	-0.784612	0.008976597
A_23_P106859	*FLOT2*	Homo sapiens flotillin 2 (FLOT2), mRNA [NM_004475]	1.0088496	0.012525387
A_21_P0000368	*YWHAH*	Homo sapiens tyrosine 3-monooxygenase/tryptophan 5-monooxygenase activation protein, eta (YWHAH), [NM_003405]	-0.8393057	0.006165438
A_24_P274640	*EMC8*	Homo sapiens ER membrane protein complex subunit 8 (EMC8), [NM_006067]	-0.59092253	0.011307727
A_21_P0005503	*SNORD5*	Homo sapiens small nucleolar RNA, C/D box 5 (SNORD5), small nucleolar RNA [NR_003033]	-0.9099448	0.005142579
A_23_P393645	*GTF2H2C_2*	Homo sapiens GTF2H2 family member C, copy 2 (GTF2H2C_2), [NM_001042490]	-0.92416745	0.0044319443
A_32_P104746	*lnc-SBDS-2*	LNCipedia lincRNA (lnc-SBDS-2), lincRNA [lnc-SBDS-2:2]	-0.7051017	0.01179784
A_23_P10077	*ADAMTS13*	Homo sapiens ADAM metallopeptidase with thrombospondin type 1 motif, 13 (ADAMTS13), [NM_139025]	-1.0732559	0.005947757
A_21_P0001397	*ZFYVE28*	Homo sapiens zinc finger, FYVE domain containing 28 (ZFYVE28), [NM_020972]	1.3578033	0.0071432865
(+)E1A_r60_a107	*PNPLA2*	Homo sapiens patatin-like phospholipase domain containing 2 (PNPLA2), [NM_020376]	1.1849583	0.008106991
A_19_P00808461	*lnc-TSEN15-1*	LNCipedia lincRNA (lnc-TSEN15-1), lincRNA [lnc-TSEN15-1:1]	-1.1736588	0.002419416
A_33_P3366102	*ZNF37A*	Homo sapiens zinc finger protein 37A (ZNF37A), [NM_001007094]	-0.62718296	0.013730757
A_33_P3290573	*WASF1*	Homo sapiens WAS protein family, member 1 (WASF1), [NM_003931]	-0.6922748	0.0141058015
A_21_P0010951	*KLHL7*	Homo sapiens kelch-like family member 7 (KLHL7), [NM_001172428]	-0.7053471	0.013022237
A_23_P145146	*FAM46A*	Homo sapiens family with sequence similarity 46, member A (FAM46A), [NM_017633]	-1.4097297	0.005852893
A_22_P00007583	*LOC101929506*	PREDICTED: Homo sapiens uncharacterized LOC101929506 (LOC101929506), [XR_252776]	-0.90744406	0.012655556

A_23_P412577	*PDCD2*	Homo sapiens programmed cell death 2 (PDCD2), [NM_002598]	-0.6492102	0.011328784
A_24_P296457	*lnc-HDAC9-3*	LNCipedia lincRNA (lnc-HDAC9-3), [lnc-HDAC9-3:1]	-1.1398283	0.0029015075
A_23_P122563	*ANKRD29*	Homo sapiens ankyrin repeat domain 29 (ANKRD29), [NM_173505]	-1.9975395	0.0030069214
A_24_P334130	*PHKG2*	Homo sapiens phosphorylase kinase, gamma 2 (testis) (PHKG2), [NM_000294]	1.1277802	0.012448702
A_33_P3390013	*PFDN6*	Homo sapiens prefoldin subunit 6 (PFDN6), [NM_014260]	-0.70696354	0.0067833774
(+)E1A_r60_a135	*FN1*	Homo sapiens fibronectin 1 (FN1), [NM_054034]	-0.67328143	0.01387464
A_23_P209269	*ADAMTS1*	Homo sapiens ADAM metallopeptidase with thrombospondin type 1 motif, 1 (ADAMTS1), [NM_006988]	-1.1322892	0.00681888
A_24_P132039	*PPM1B*	Homo sapiens protein phosphatase, Mg2+/Mn2+ dependent, 1B (PPM1B), [NM_001033557]	-0.8482415	0.008470153
A_33_P3668049	*SNX16*	Homo sapiens sorting nexin 16 (SNX16), [NM_022133]	-1.0394547	0.006352343
A_33_P3297305	*RNF14*	Homo sapiens ring finger protein 14 (RNF14), [NM_004290]	-0.83284044	0.0039416323
A_22_P00009741	*RHOT1*	Homo sapiens ras homolog family member T1 (RHOT1), [NM_001288758]	-0.7094445	0.012611509
A_32_P88349	*ELMOD3*	Homo sapiens ELMO/CED-12 domain containing 3 (ELMOD3), [NM_032213]	0.8158282	0.010370833
A_23_P92967	*lnc-MDH1B-2*	LNCipedia lincRNA (lnc-MDH1B-2), lincRNA [lnc-MDH1B-2:1]	-1.1930197	0.009792738
A_33_P3408983	*LOC730257*	PREDICTED: Homo sapiens uncharacterized LOC730257 (LOC730257), [XR_426840]	0.75735015	0.012487623
A_24_P3140	*MOCS2*	Homo sapiens molybdenum cofactor synthesis 2 (MOCS2), [NM_004531]	-0.4965877	0.004851867
A_23_P57137	*APC*	Homo sapiens adenomatous polyposis coli (APC), [NM_001127511]	-0.87386197	0.005438366
A_23_P16523	*ZNF638*	Homo sapiens zinc finger protein 638 (ZNF638), [NM_014497]	-0.7126786	0.00410343
A_23_P304511	*AP5S1*	Homo sapiens adaptor-related protein complex 5, sigma 1 subunit (AP5S1), [NM_018347]	-0.58710974	0.013622508
A_23_P315789	*GDF15*	Homo sapiens growth differentiation factor 15 (GDF15), [NM_004864]	-1.2944835	0.0029691157
A_23_P377819	*ZNF397*	Homo sapiens zinc finger protein 397 (ZNF397), [NM_032347]	-1.9961092	0.005073928
A_23_P202496	*RFXAP*	Homo sapiens regulatory factor X-associated protein (RFXAP), [NM_000538]	-0.5424772	0.009808998
A_23_P40025	*SRSF5*	Homo sapiens serine/arginine-rich splicing factor 5 (SRSF5), [NM_001039465]	-0.3322603	0.012512322

A_23_P13899	*NOC3L*	Homo sapiens nucleolar complex associated 3 homolog (S. cerevisiae) (NOC3L), [NM_022451]	-0.496185	0.015891002
A_33_P3355014	*DAZAP2*	Homo sapiens DAZ associated protein 2 (DAZAP2), [NM_014764]	-0.94240683	0.0044240346
A_33_P3299525	*GAPDH*	Homo sapiens glyceraldehyde-3-phosphate dehydrogenase (GAPDH), [NM_002046]	0.38551566	0.011184008
A_22_P00014599	*TMEM229B*	Homo sapiens transmembrane protein 229B (TMEM229B), [NM_182526]	-1.8441373	0.01708899
A_23_P94338	*KCTD21*	Homo sapiens potassium channel tetramerization domain containing 21 (KCTD21), [NM_001029859]	-1.2417297	0.0054085264
A_33_P3318960	*lnc-SLC15A1-1*	Homo sapiens cDNA: FLJ23243 fis, clone COL01757. [AK026896]	0.43400025	0.012975978
A_32_P29806	*ENPP2*	Homo sapiens ectonucleotide pyrophosphatase/phosphodiesterase 2 (ENPP2), [NM_006209]	-2.1280468	0.0043581175
A_24_P941376	*POLR2F*	Homo sapiens polymerase (RNA) II (DNA directed) polypeptide F (POLR2F), [NM_001301131]	-0.5978458	0.0032554762
A_33_P3326904	*CRADD*	Homo sapiens CASP2 and RIPK1 domain containing adaptor with death domain (CRADD), [NM_003805]	-0.76158047	0.010093137
A_33_P3367301	*ZNF473*	Homo sapiens zinc finger protein 473 (ZNF473), [NM_015428]	0.39195153	0.016863305
A_24_P304723	*LOC441268*	Homo sapiens cDNA FLJ43176 fis, clone FCBBF3008944. [AK125166]	-0.7443146	0.0051692408
A_23_P200780	*GJD3*	Homo sapiens gap junction protein, delta 3, 31.9kDa (GJD3), [NM_152219]	-2.5645633	0.0035939496
A_23_P117683	*PPIB*	Homo sapiens peptidylprolyl isomerase B (cyclophilin B) (PPIB), [NM_000942]	-0.37097934	0.010369088
A_19_P00322533	*TGFBR3*	Homo sapiens transforming growth factor, beta receptor III (TGFBR3), [NM_003243]	-0.55570984	0.0035648923
A_33_P3372397	*HYPK*	Homo sapiens huntingtin interacting protein K (HYPK), [NM_016400]	-0.7244668	0.006653174
A_23_P254181	*CRNDE*	Homo sapiens colorectal neoplasia differentially expressed (non-protein coding) (CRNDE), [NR_110453]	-0.9399233	0.0034903844
A_23_P99771	*LINC01260*	Homo sapiens long intergenic non-protein coding RNA 1260 (LINC01260), [NR_034104]	-1.1101272	0.014183129
A_23_P65157	*ITGBL1*	Homo sapiens integrin, beta-like 1 (with EGF-like repeat domains) (ITGBL1), [NM_004791]	-1.5277996	0.0031941293
A_23_P21673	*PNMA1*	Homo sapiens paraneoplastic Ma antigen 1 (PNMA1), [NM_006029]	-0.9004938	0.0119310785
A_24_P124973	*COX17*	Homo sapiens COX17 cytochrome c oxidase copper chaperone (COX17), [NM_005694]	-0.5162554	0.011575981
A_33_P3311056	*FOCAD*	Homo sapiens focadhesin (FOCAD), [NM_017794]	-1.0370393	0.008289811

A_23_P91619	*NDNL2*	Homo sapiens necdin-like 2 (NDNL2), [NM_138704]	0.522037	0.016465655
A_21_P0010254	*SDHA*	Homo sapiens succinate dehydrogenase complex, subunit A, flavoprotein (Fp) (SDHA), [NM_004168]	-0.9196756	0.0050847097
A_24_P102895	*MIF*	Homo sapiens macrophage migration inhibitory factor (glycosylation-inhibiting factor) (MIF), [NM_002415]	-0.66287726	0.0027248112
A_23_P143374	*lnc-UMODL1-1*	LNCipedia lincRNA (lnc-UMODL1-1), lincRNA [lnc-UMODL1-1:2]	2.7506518	0.0025001846
A_23_P23719	*WDTC1*	Homo sapiens WD and tetratricopeptide repeats 1 (WDTC1), [NM_015023]	0.71484256	0.015933463
A_21_P0007218	*NINL*	Homo sapiens ninein-like (NINL), [NM_025176]	-0.65855604	0.008638155
A_33_P3276856	*VPS45*	Homo sapiens vacuolar protein sorting 45 homolog (S. cerevisiae) (VPS45), [NM_007259]	-0.73234797	0.010035188
A_23_P115137	*BCAS4*	breast carcinoma amplified sequence 4 [Source:HGNC Symbol;Acc:HGNC:14367] [ENST00000463943]	3.1536767	0.0034534817
A_24_P169386	*HYDIN*	Homo sapiens HYDIN, axonemal central pair apparatus protein (HYDIN), [NM_001198542]	1.1924071	0.00968922
A_23_P121686	*LAMTOR5*	Homo sapiens late endosomal/lysosomal adaptor, MAPK and MTOR activator 5 (LAMTOR5), [NM_006402]	-0.7666111	0.011334712
A_32_P151366	*AIMP1*	Homo sapiens aminoacyl tRNA synthetase complex-interacting multifunctional protein 1 (AIMP1), [NM_004757]	-1.3272684	0.004160909
A_32_P72940	*ZSCAN20*	zinc finger and SCAN domain containing 20 [Source:HGNC Symbol;Acc:HGNC:13093] [ENST00000373413]	-1.3745135	0.012318275
A_23_P373799	*SIRT5*	Homo sapiens sirtuin 5 (SIRT5), [NM_012241]	-0.61368686	0.0060155373
A_33_P3255544	*RPL35*	Homo sapiens ribosomal protein L35 (RPL35), [NM_007209]	-0.32938263	0.007740612
A_22_P00022877	*CWC22*	Homo sapiens CWC22 spliceosome-associated protein (CWC22), [NM_020943]	-0.5202603	0.005612091
A_33_P3777584	*RNF130*	Homo sapiens ring finger protein 130 (RNF130), [NM_001280801]	-0.70513433	0.015123205
A_23_P146811	*lnc-IL15RA-1*	AGENCOURT_6621625 NIH_MGC_125 Homo sapiens cDNA clone IMAGE:5722280 5',[BM906553]	-0.79434127	0.008819748
A_23_P89249	*SERAC1*	Homo sapiens serine active site containing 1 (SERAC1), [NM_032861]	-1.6681533	0.0066818837
A_22_P00003766	*FBXO38*	Homo sapiens F-box protein 38 (FBXO38), [NM_030793]	-0.7154479	0.0059050764
A_23_P359214	*ERBB2*	Homo sapiens erb-b2 receptor tyrosine kinase 2 (ERBB2), [NM_001005862]	-0.96529937	0.009811661

A_23_P45970	*LINC00842*	Homo sapiens long intergenic non-protein coding RNA 842 (LINC00842), [NR_033957]	0.8879297	0.0070324177
A_23_P148584	*ZNF593*	Homo sapiens zinc finger protein 593 (ZNF593), [NM_015871]	-0.6833907	0.014183129
A_23_P419795	*C1orf109*	Homo sapiens chromosome 1 open reading frame 109 (C1orf109), [NM_017850]	-0.70869863	0.005712846
A_33_P3332135	*DOCK11*	Homo sapiens dedicator of cytokinesis 11 (DOCK11), [NM_144658]	-1.4169881	0.0029471128
A_32_P131367	*SBF2*	Homo sapiens SET binding factor 2 (SBF2), [NM_030962]	-1.3169384	0.01080114
A_33_P3842770	*PHOSPHO1*	Homo sapiens phosphatase, orphan 1 (PHOSPHO1), [NM_001143804]	0.74026453	0.011738894
A_24_P406986	*RNF13*	Homo sapiens ring finger protein 13 (RNF13), [NM_007282]	-0.5654602	0.012620988
A_23_P38446	*LOC101928669*	PREDICTED: Homo sapiens uncharacterized LOC101928669 (LOC101928669), [XR_424064]	-0.71879005	0.0065307794
A_33_P3214052	*SLC43A3*	Homo sapiens solute carrier family 43, member 3 (SLC43A3), [NM_199329]	1.1055601	0.014349793
A_33_P3385870	*TNFAIP1*	Homo sapiens tumor necrosis factor, alpha-induced protein 1 (endothelial) (TNFAIP1), [NM_021137]	-0.925318	0.0031941293
A_22_P00003974	*lnc-SHPRH-2*	LNCipedia lincRNA (lnc-SHPRH-2), lincRNA [lnc-SHPRH-2:6]	0.58488053	0.009543431
A_33_P3271930	*CCNG2*	Homo sapiens cyclin G2 (CCNG2), [NM_004354]	-1.1548259	0.0024890031
A_23_P202602	*PYCR1*	Homo sapiens pyrroline-5-carboxylate reductase 1 (PYCR1), [NM_153824]	1.099001	0.0044813035
A_33_P3362326	*MARCH6*	Homo sapiens membrane-associated ring finger (C3HC4) 6, E3 ubiquitin protein ligase (MARCH6), [NM_005885]	-0.5615244	0.009466768
A_23_P435941	*SEC23IP*	Homo sapiens SEC23 interacting protein (SEC23IP), [NM_007190]	-0.5175168	0.008570872
A_23_P9056	*FHL2*	Homo sapiens mRNA; cDNA DKFZp781J01103 (from clone DKFZp781J01103). [CR936682]	-1.3321233	0.0036402496
A_23_P78563	*SAMD1*	Homo sapiens sterile alpha motif domain containing 1 (SAMD1), [NM_138352]	1.6489882	0.0069652614
A_33_P3251148	*RB1CC1*	Homo sapiens RB1-inducible coiled-coil 1 (RB1CC1), [NM_014781]	-1.0013981	0.007479662
A_32_P20367	*UBL5*	Homo sapiens ubiquitin-like 5 (UBL5), [NM_024292]	-0.72780323	0.0057328343
A_23_P46429	*TSPO*	Homo sapiens translocator protein (18kDa) (TSPO), [NM_001256530]	-0.59465545	0.003956951
A_33_P3351999	*RPS7*	Homo sapiens ribosomal protein S7 (RPS7), [NM_001011]	-0.3180602	0.0046701035
A_23_P118516	*CYR61*	Homo sapiens cysteine-rich, angiogenic inducer, 61 (CYR61), [NM_001554]	-0.77340645	0.0098884255

A_22_P00006710	*TVP23B*	Homo sapiens trans-golgi network vesicle protein 23 homolog B (S. cerevisiae) (TVP23B), [NM_016078]	-0.7438069	0.0034400926
A_33_P3267380	*HGC6.3*	Homo sapiens uncharacterized LOC100128124 (HGC6.3), [NM_001129895]	0.92642856	0.006655095
A_23_P1145	*SGCD*	Homo sapiens sarcoglycan, delta (35kDa dystrophin-associated glycoprotein) (SGCD), [NM_172244]	-1.7424376	0.0032554762
A_23_P258493	*ZNF562*	Homo sapiens zinc finger protein 562 (ZNF562), [NM_001130031]	-0.5816956	0.0074182227
A_23_P110811	*MTPAP*	Homo sapiens mitochondrial poly(A) polymerase (MTPAP), [NM_018109]	-0.39893413	0.016661061
A_33_P3282840	*LMNB1*	Homo sapiens lamin B1 (LMNB1), [NM_005573]	-0.56391877	0.01240238
A_21_P0009753	*COX7C*	Homo sapiens cytochrome c oxidase subunit VIIc (COX7C), [NM_001867]	-0.38080215	0.008564741
A_23_P83073	*RPS29*	Homo sapiens ribosomal protein S29 (RPS29), [NM_001030001]	-0.98798823	0.0031941293
A_23_P256455	*LINC00662*	long intergenic non-protein coding RNA 662 [Source:HGNC Symbol;Acc:HGNC:27122] [ENST00000590677]	-0.8918375	0.011057721
A_33_P3311646	*HIATL1*	Homo sapiens hippocampus abundant transcript-like 1 (HIATL1), [NM_032558]	-0.57375765	0.0070324177
A_22_P00016696	*RPA3*	Homo sapiens replication protein A3, 14kDa (RPA3), [NM_002947]	-0.73156804	0.0063513564
A_24_P140608	*lnc-TRA2A-1*	Q4G0Q7_HUMAN (Q4G0Q7) MGC72075 protein, partial (22%) [THC2615536]	1.0827988	0.013453215
A_23_P115762	*FOLR2*	Homo sapiens folate receptor 2 (fetal) (FOLR2), [NM_000803]	0.2604382	0.016549561
A_33_P3290602	*HBEGF*	Homo sapiens heparin-binding EGF-like growth factor (HBEGF),[NM_001945]	1.5673296	0.00631895
A_23_P122233	*ECD*	Homo sapiens ecdysoneless homolog (Drosophila) (ECD), [NM_007265]	-0.5800934	0.010894719
A_22_P00005153	*MMP24-AS1*	Homo sapiens MMP24 antisense RNA 1 (MMP24-AS1), [NR_102705]	-0.98400235	0.009787606
A_23_P6596	*MRPL22*	Homo sapiens mitochondrial ribosomal protein L22 (MRPL22), transcript variant 2, mRNA [NM_001014990]	-0.6789506	0.00652649
A_21_P0000081	*lnc-DHX57-2*	ALU1_HUMAN (P39188) Alu subfamily J sequence contamination warning entry, partial (7%) [THC2671955]	-1.6732069	0.008534892
A_24_P254346	*HES1*	Homo sapiens hes family bHLH transcription factor 1 (HES1), mRNA [NM_005524]	2.3569098	0.0130220065
A_23_P132444	*ZFAND1*	Homo sapiens zinc finger, AN1-type domain 1 (ZFAND1), transcript variant 3, mRNA [NM_001170797]	-0.5895803	0.01372088
A_33_P3231981	*DSCR3*	Homo sapiens Down syndrome critical region 3 (DSCR3), mRNA [NM_006052]	-1.0207448	0.007843468

A_24_P88554	*TCEA1*	Homo sapiens transcription elongation factor A (SII), 1 (TCEA1), transcript variant 1, mRNA [NM_006756]	-0.9873054	0.00477571
A_24_P132518	*PARK7*	Homo sapiens parkinson protein 7 (PARK7), transcript variant 1, mRNA [NM_007262]	-0.61235356	0.006632856
A_23_P404821	*PEX11B*	Homo sapiens peroxisomal biogenesis factor 11 beta (PEX11B), transcript variant 1, mRNA [NM_003846]	-0.95510006	0.01375843
A_22_P00003889	*IKBKB*	Homo sapiens inhibitor of kappa light polypeptide gene enhancer in B-cells, kinase beta (IKBKB), transcript variant 1, mRNA [NM_001556]	-0.55424184	0.0132579645
A_23_P211345	*KIAA1147*	Homo sapiens KIAA1147 (KIAA1147), mRNA [NM_001080392]	-1.2014724	0.0061321366
A_33_P3279620	*ZNF608*	Homo sapiens zinc finger protein 608 (ZNF608), mRNA [NM_020747]	2.3733063	0.0040942165
A_22_P00011151	*TBX1*	Homo sapiens T-box 1 (TBX1), transcript variant C, mRNA [NM_080647]	3.5262694	0.0035894548
A_23_P117582	*lnc-OBFC2A-1*	LNCipedia lincRNA (lnc-OBFC2A-1), lincRNA [lnc-OBFC2A-1:1]	-0.8833198	0.0069863
A_23_P211806	*OSBP2*	Homo sapiens oxysterol binding protein 2 (OSBP2), transcript variant 1, mRNA [NM_030758]	1.6916969	0.015589794
A_23_P48535	*JDP2*	Homo sapiens Jun dimerization protein 2 (JDP2), transcript variant 1, mRNA [NM_130469]	-1.0767076	0.006726202
A_23_P102508	*LRRFIP2*	Homo sapiens leucine rich repeat (in FLII) interacting protein 2 (LRRFIP2), transcript variant 2, mRNA [NM_017724]	-0.7457301	0.0031941293
A_23_P29495	*ARHGAP5*	Homo sapiens Rho GTPase activating protein 5 (ARHGAP5), transcript variant 1, mRNA [NM_001030055]	-1.4133751	0.005428736
A_23_P51410	*SLC5A6*	Homo sapiens solute carrier family 5 (sodium/multivitamin and iodide cotransporter), member 6 (SLC5A6), transcript variant 1, mRNA [NM_021095]	1.1367449	0.008537068
A_33_P3273290	*CTNNB1*	Homo sapiens catenin (cadherin-associated protein), beta 1, 88kDa (CTNNB1), transcript variant 1, mRNA [NM_001904]	-0.17209646	0.004788691
A_23_P45488	*SMYD3*	Homo sapiens SET and MYND domain containing 3 (SMYD3), transcript variant 2, mRNA [NM_022743]	-1.8973423	0.005261815
A_23_P319640	*ZNF28*	zinc finger protein 28 [Source:HGNC Symbol;Acc:HGNC:13073] [ENST00000464469]	-0.81175697	0.0059749396
A_33_P3248787	*FAM3A*	Homo sapiens family with sequence similarity 3, member A (FAM3A), transcript variant 1, mRNA [NM_021806]	0.7187319	0.014426925

A_24_P253251	*LBX2-AS1*	Homo sapiens LBX2 antisense RNA 1 (LBX2-AS1), long non-coding RNA [NR_024606]	1.6135265	0.0031941293
A_23_P36226	*SLC7A1*	Homo sapiens solute carrier family 7 (cationic amino acid transporter, y+ system), member 1 (SLC7A1), mRNA [NM_003045]	-0.48039246	0.0096329255
A_33_P3334121	*INF2*	Homo sapiens inverted formin, FH2 and WH2 domain containing (INF2), transcript variant 3, mRNA [NM_032714]	-0.87948847	0.016192868
A_21_P0001665	*SLC25A33*	Homo sapiens solute carrier family 25 (pyrimidine nucleotide carrier), member 33 (SLC25A33), mRNA [NM_032315]	-0.64439225	0.015936593
A_23_P15511	*lnc-APITD1-1*	LNCipedia lincRNA (lnc-APITD1-1), lincRNA [lnc-APITD1-1:1]	-0.89878	0.007720072
A_19_P00318152	*MMS22L*	Homo sapiens MMS22-like, DNA repair protein (MMS22L), mRNA [NM_198468]	-0.58513665	0.011319855
A_23_P45294	*DDX5*	Homo sapiens DEAD (Asp-Glu-Ala-Asp) box helicase 5 (DDX5), mRNA [NM_004396]	-0.49434778	0.00410343
A_21_P0000748	*SNHG11*	Homo sapiens small nucleolar RNA host gene 11 (non-protein coding) (SNHG11), long non-coding RNA [NR_003239]	1.1573807	0.015290136
A_23_P358221	*FAM199X*	Homo sapiens family with sequence similarity 199, X-linked (FAM199X), mRNA [NM_207318]	-0.3382402	0.010402669
A_23_P132260	*TCEB3-AS1*	Homo sapiens TCEB3 antisense RNA 1 (TCEB3-AS1), long non-coding RNA [NR_038280]	-1.2237061	0.0032522907
A_23_P122924	*UBXN7*	Homo sapiens UBX domain protein 7 (UBXN7), mRNA [NM_015562]	-0.32623142	0.008564741
A_33_P3392187	*PES1*	Homo sapiens pescadillo ribosomal biogenesis factor 1 (PES1), transcript variant 1, mRNA [NM_014303]	1.040891	0.010971502
A_19_P00808120	*INHBA*	Homo sapiens inhibin, beta A (INHBA), mRNA [NM_002192]	-1.7038536	0.0024818634
A_33_P3257027	*CCDC85A*	Homo sapiens coiled-coil domain containing 85A (CCDC85A), mRNA [NM_001080433]	-3.9643726	0.002135847
A_21_P0010617	*FAM200B*	Homo sapiens family with sequence similarity 200, member B (FAM200B), mRNA [NM_001145191]	-1.2676052	0.007501246
A_24_P56388	*FGF7*	Homo sapiens fibroblast growth factor 7 (FGF7), mRNA [NM_002009]	-0.2847082	0.008437608
(+)E1A_r60_a97	*HIF1A*	Homo sapiens hypoxia inducible factor 1, alpha subunit (basic helix-loop-helix transcription factor) (HIF1A), transcript variant 2, mRNA [NM_181054]	-0.817703	0.0033199077
A_23_P54929	*TRIP11*	Homo sapiens thyroid hormone receptor interactor 11 (TRIP11), mRNA [NM_004239]	-0.63760495	0.0067463443
A_22_P00019867	*LYRM1*	Homo sapiens LYR motif containing 1 (LYRM1), transcript variant 1, mRNA [NM_020424]	-0.889323	0.005947757

A_21_P0000269	*LOC644189*	Homo sapiens acyl-CoA thioesterase 4 pseudogene (LOC644189), non-coding RNA [NR_033748]	-1.7142814	0.005644507
A_22_P00012284	*lnc-GSTO2-2*	Q2U8N5_ASPOR (Q2U8N5) Predicted protein, partial (3%) [THC2646832]	0.6005881	0.005612091
A_23_P359655	*SNORA52*	Homo sapiens small nucleolar RNA, H/ACA box 52 (SNORA52), small nucleolar RNA [NR_002585]	1.0863055	0.007111782
A_23_P15123	*lnc-PPP2R2D-3*	LNCipedia lincRNA (lnc-PPP2R2D-3), lincRNA [lnc-PPP2R2D-3:1]	0.5517357	0.0046073813
A_23_P356616	*ZNF664*	Homo sapiens zinc finger protein 664 (ZNF664), transcript variant 1, mRNA [NM_152437]	-0.89241856	0.006573993
A_24_P19228	*UBFD1*	Homo sapiens ubiquitin family domain containing 1 (UBFD1), mRNA [NM_019116]	-0.77130073	0.016251752
A_23_P100220	*ABTB2*	Homo sapiens ankyrin repeat and BTB (POZ) domain containing 2 (ABTB2), mRNA [NM_145804]	-1.2027278	0.014212319
A_24_P226210	*GAMT*	Homo sapiens guanidinoacetate N-methyltransferase (GAMT), transcript variant 2, mRNA [NM_138924]	0.8475453	0.010440681
A_21_P0013302	*ESRP2*	Homo sapiens epithelial splicing regulatory protein 2 (ESRP2), mRNA [NM_024939]	2.4225965	0.0054365364
A_23_P31671	*CEP120*	Homo sapiens centrosomal protein 120kDa (CEP120), transcript variant 1, mRNA [NM_153223]	-0.54087216	0.007479662
A_21_P0014709	*UQCRB*	Homo sapiens ubiquinol-cytochrome c reductase binding protein (UQCRB), transcript variant 1, mRNA [NM_006294]	-0.6427481	0.0047988673
A_24_P45005	*lnc-FANCI-1*	Homo sapiens cDNA clone IMAGE:4838568. [BC031940]	-0.79236335	0.010773812
A_33_P3286157	*PAX8-AS1*	Homo sapiens PAX8 antisense RNA 1 (PAX8-AS1), transcript variant 2, long non-coding RNA [NR_047570]	-1.0118401	0.0054365364
A_23_P151075	*NPEPL1*	Homo sapiens aminopeptidase-like 1 (NPEPL1), transcript variant 1, mRNA [NM_024663]	-1.1028764	0.0061486606
A_23_P46396	*TNFRSF4*	Homo sapiens tumor necrosis factor receptor superfamily, member 4 (TNFRSF4), mRNA [NM_003327]	0.7390684	0.01661746
A_23_P500333	*ARHGDIB*	Homo sapiens Rho GDP dissociation inhibitor (GDI) beta (ARHGDIB), mRNA [NM_001175]	-1.4024599	0.005428736
A_23_P45059	*PTBP2*	Homo sapiens polypyrimidine tract binding protein 2 (PTBP2), transcript variant 6, mRNA [NM_021190]	-0.7997536	0.0037479755
A_33_P3364263	*SSBP3*	Homo sapiens single stranded DNA binding protein 3 (SSBP3), transcript variant 3, mRNA [NM_001009955]	1.2530909	0.0029471128

A_33_P3373985	*DOCK1*	Homo sapiens dedicator of cytokinesis 1 (DOCK1), transcript variant 2, mRNA [NM_001380]	-0.6639247	0.0036517929
A_23_P140256	*LBH*	Homo sapiens limb bud and heart development (LBH), mRNA [NM_030915]	-3.0581656	0.0031941293
A_19_P00325810	*PNP*	Homo sapiens purine nucleoside phosphorylase (PNP), [NM_000270]	0.54750085	0.016931703
A_23_P73835	*MTHFD2*	Homo sapiens methylenetetrahydrofolate dehydrogenase (NADP+ dependent) 2, methenyltetrahydrofolate cyclohydrolase (MTHFD2), [NM_006636]	-1.480989	0.0025001846
A_23_P122001	*KHDC1*	Homo sapiens KH homology domain containing 1 (KHDC1), [NR_027005]	1.212868	0.0074157496
A_24_P191588	*MOSPD1*	Homo sapiens motile sperm domain containing 1 (MOSPD1), [NM_019556]	-0.66492206	0.014104291
A_23_P29124	*MSH3*	Homo sapiens mutS homolog 3 (MSH3), [NM_002439]	-0.64266485	0.007123488
A_33_P3330079	*ALAS1*	Homo sapiens 5'-aminolevulinate synthase 1 (ALAS1), [NM_000688]	-0.5812967	0.0140423635
A_22_P00008291	*GP1BB*	Homo sapiens glycoprotein Ib (platelet), beta polypeptide (GP1BB), mRNA [NM_000407]	-1.674026	0.0057806885
A_33_P3279431	*LOC100128242*	PREDICTED: Homo sapiens uncharacterized LOC100128242 (LOC100128242), misc_RNA [XR_110532]	0.95553035	0.009874361
A_23_P34578	*lnc-ITGA11-1*	PREDICTED: Homo sapiens integrin, alpha 11 (ITGA11), [XM_005254228]	-1.4991989	0.0088250805
A_24_P66337	*GOSR1*	Homo sapiens golgi SNAP receptor complex member 1 (GOSR1), [NM_001007024]	-0.88954806	0.009737427
A_24_P263543	*GNL2*	Homo sapiens guanine nucleotide binding protein-like 2 (nucleolar) (GNL2), mRNA [NM_013285]	-0.4018519	0.01073543
A_24_P345377	*LCLAT1*	lysocardiolipin acyltransferase 1 [Source:HGNC Symbol;Acc:HGNC:26756] [ENST00000319406]	-1.5965906	0.006573993
A_21_P0003941	*GOLPH3L*	Homo sapiens golgi phosphoprotein 3-like (GOLPH3L), [NM_018178]	-0.70386046	0.016822075
A_33_P3407049	*ALDH1A1*	Homo sapiens aldehyde dehydrogenase 1 family, member A1 (ALDH1A1), [NM_000689]	-2.2268572	0.0048263967
A_33_P3281408	*LUCAT1*	lung cancer associated transcript 1 (non-protein coding) [Source:HGNC Symbol;Acc:HGNC:48498] [ENST00000513626]	1.6027503	0.00886592
A_33_P3279629	*YPEL5*	Homo sapiens yippee-like 5 (Drosophila) (YPEL5), transcript variant 1, mRNA [NM_001127401]	-1.125389	0.0037656627
A_33_P3285047	*ALG11*	Homo sapiens ALG11, alpha-1,2-mannosyltransferase (ALG11), [NM_001004127]	-0.6255653	0.00881236
A_23_P24633	*UCN2*	Homo sapiens urocortin 2 (UCN2), [NM_033199]	3.1834087	0.0029471128

A_23_P106720	*ZNF75A*	Homo sapiens zinc finger protein 75a (ZNF75A), [NM_001302109]	-0.7799487	0.0037685214
A_23_P31866	*THYN1*	Homo sapiens thymocyte nuclear protein 1 (THYN1), [NM_199297]	-0.46980312	0.009091813
A_23_P131435	*TBL3*	Homo sapiens transducin (beta)-like 3 (TBL3), [NM_006453]	1.9141259	0.0032831472
A_23_P170608	*MED30*	Homo sapiens mediator complex subunit 30 (MED30), [NM_080651]	-1.0695635	0.0032224318
A_23_P359647	*CD302*	Homo sapiens CD302 molecule (CD302), [NM_014880]	-1.2387658	0.010987073
A_24_P229726	*TSPYL2*	Homo sapiens TSPY-like 2 (TSPYL2), mRNA [NM_022117]	1.4490501	0.0053387037
A_24_P320526	*NFAT5*	Homo sapiens nuclear factor of activated T-cells 5, tonicity-responsive (NFAT5), [NM_138714]	-0.6589229	0.014357166
A_21_P0014573	*C6orf106*	Homo sapiens chromosome 6 open reading frame 106 (C6orf106), [NM_024294]	0.9082173	0.010854676
A_33_P3230478	*RRP12*	Homo sapiens ribosomal RNA processing 12 homolog (S. cerevisiae) (RRP12), [NM_015179]	0.7228968	0.015840316
A_23_P403955	*C1S*	Homo sapiens complement component 1, s subcomponent (C1S), [NM_201442]	0.76018524	0.016750982
A_24_P192301	*TARDBP*	Homo sapiens TAR DNA binding protein (TARDBP), mRNA [NM_007375]	-0.60399526	0.0029471128
A_21_P0011840	*WSCD2*	Homo sapiens WSC domain containing 2 (WSCD2), mRNA [NM_014653]	1.230182	0.005735339
A_21_P0010648	*SEMA3A*	Homo sapiens sema domain, immunoglobulin domain (Ig), short basic domain, secreted, (semaphorin) 3A (SEMA3A), mRNA [NM_006080]	2.2068162	0.0034812074
A_23_P27649	*LOC100507334*	Homo sapiens two pore channel 3 pseudogene (LOC100507334), [NR_037626]	0.44938406	0.01284574
A_23_P209499	*XLOC_l2_001086*	BROAD Institute lincRNA (XLOC_l2_001086), lincRNA [TCONS_l2_00001492]	-1.7908609	0.0027262743
A_24_P678104	*ZNF433*	Homo sapiens zinc finger protein 433 (ZNF433), [NM_001080411]	-0.8562858	0.005852893
A_32_P126311	*PAX3*	Homo sapiens paired box 3 (PAX3), [NM_181458]	-2.220285	0.0032554762
A_21_P0000464	*STMN3*	Homo sapiens stathmin-like 3 (STMN3), [NM_015894]	2.308778	0.00422039
A_21_P0012220	*SLC38A9*	Homo sapiens solute carrier family 38, member 9 (SLC38A9), [NM_173514]	-0.82629794	0.0085387705
A_23_P20882	*SNORD123*	Homo sapiens small nucleolar RNA, C/D box 123 (SNORD123), small nucleolar RNA [NR_003689]	-0.65619975	0.015710928
A_23_P110712	*ANKRD20A11P*	ankyrin repeat domain 20 family, member A11, pseudogene [ENST00000429521]	-1.6307449	0.005365543

A_21_P0009685	*ATP6V1G1*	Homo sapiens ATPase, H+ transporting, lysosomal 13kDa, V1 subunit G1 (ATP6V1G1), [NM_004888]	-0.7447572	0.00690275
A_23_P383819	*DUSP1*	Homo sapiens dual specificity phosphatase 1 (DUSP1), [NM_004417]	-0.13993911	0.011588712
A_23_P167096	*TMEM167A*	Homo sapiens transmembrane protein 167A (TMEM167A), [NM_174909]	-0.55237335	0.0055686985
A_24_P101201	*TBX3*	Homo sapiens T-box 3 (TBX3), [NM_016569]	1.7590486	0.008705128
A_23_P77630	*VEGFC*	Homo sapiens vascular endothelial growth factor C (VEGFC), [NM_005429]	0.5838756	0.011021474
A_21_P0001639	*PDIA3*	Homo sapiens protein disulfide isomerase family A, member 3 (PDIA3), [NM_005313]	-0.8198633	0.008168415
A_23_P127095	*MAP1LC3B*	Homo sapiens microtubule-associated protein 1 light chain 3 beta (MAP1LC3B), [NM_022818]	-0.3740437	0.009792738
A_23_P99027	*lnc-TAF5L-1*	LNCipedia lincRNA (lnc-TAF5L-1), lincRNA [lnc-TAF5L-1:1]	1.7225945	0.013353969
A_24_P673786	*USMG5*	Homo sapiens up-regulated during skeletal muscle growth 5 homolog (mouse) (USMG5), [NM_032747]	-0.7492084	0.0044240346
A_23_P112531	*PTPN11*	Homo sapiens protein tyrosine phosphatase, non-receptor type 11 (PTPN11), [NM_002834]	-0.65893537	0.010819448
A_22_P00019837	*PIP4K2A*	Homo sapiens phosphatidylinositol-5-phosphate 4-kinase, type II, alpha (PIP4K2A), [NM_005028]	-0.87349796	0.007503526
A_33_P3257973	*FAM102A*	Homo sapiens family with sequence similarity 102, member A (FAM102A), [NM_001035254]	-0.76885676	0.0044707763
A_23_P122007	*lnc-GTDC1-6*	LNCipedia lincRNA (lnc-GTDC1-6), lincRNA [lnc-GTDC1-6:1]	0.7809692	0.004588558
A_22_P00005034	*HELZ*	Homo sapiens helicase with zinc finger (HELZ), [NM_014877]	-0.80510706	0.0060757087
A_24_P544882	*C5orf30*	Homo sapiens chromosome 5 open reading frame 30 (C5orf30), [NM_033211]	-1.3876598	0.0032379
A_23_P132910	*lnc-DDX54-1*	LNCipedia lincRNA (lnc-DDX54-1), lincRNA [lnc-DDX54-1:1]	-0.71306056	0.010314042
A_23_P105313	*SSR1*	Homo sapiens signal sequence receptor, alpha (SSR1), [NM_003144]	-0.46521452	0.006543285
A_23_P337262	*RBM47*	Homo sapiens RNA binding motif protein 47 (RBM47), [NM_019027]	-0.8764981	0.0072648157
A_24_P396702	*EIF2B1*	Homo sapiens eukaryotic translation initiation factor 2B, subunit 1 alpha, 26kDa (EIF2B1), mRNA [NM_001414]	-0.61755615	0.009700162
A_24_P237753	*APCDD1*	Homo sapiens adenomatosis polyposis coli down-regulated 1 (APCDD1), [NM_153000]	3.452778	0.003190129
A_23_P71530	*CD302*	Homo sapiens CD302 molecule (CD302), [NM_014880]	-1.0892811	0.007370545
A_24_P182494	*NUP188*	Homo sapiens nucleoporin 188kDa (NUP188), [NM_015354]	1.5167032	0.007683989

A_33_P3335624	*TNFRSF11B*	Homo sapiens tumor necrosis factor receptor superfamily, member 11b (TNFRSF11B), [NM_002546]	0.9556622	0.005325502
A_33_P3233947	*DUSP10*	Homo sapiens dual specificity phosphatase 10 (DUSP10), [NM_007207]	1.0419993	0.007065473
A_33_P3275422	*PSMD10*	proteasome (prosome, macropain) 26S subunit, non-ATPase, 10 [Source:HGNC Symbol;Acc:HGNC:9555] [ENST00000338548]	-0.80467606	0.003363001
A_33_P3302586	*COX20*	Homo sapiens COX20 cytochrome c oxidase assembly factor (COX20), [NM_198076]	0.61685413	0.011376762
A_33_P3330283	*LINC00523*	Homo sapiens long intergenic non-protein coding RNA 523 (LINC00523), [NR_024096]	0.50913924	0.013130267
A_23_P117225	*GPR153*	Homo sapiens G protein-coupled receptor 153 (GPR153), [NM_207370]	2.0437775	0.00324898
A_23_P37892	*SP1*	Homo sapiens Sp1 transcription factor (SP1), [NM_138473]	-0.48929626	0.015087558
A_32_P167791	*ERCC5*	Homo sapiens excision repair cross-complementation group 5 (ERCC5), [NM_000123]	-0.40912727	0.016068412
A_33_P3342160	*GPT2*	Homo sapiens glutamic pyruvate transaminase (alanine aminotransferase) 2 (GPT2), [NM_133443]	-0.9631507	0.011360915
A_23_P76684	*ST13*	Homo sapiens suppression of tumorigenicity 13 (colon carcinoma) (Hsp70 interacting protein) (ST13), [NM_003932]	-0.65464216	0.010134572
A_33_P3334308	*ATG2B*	Homo sapiens autophagy related 2B (ATG2B), [NM_018036]	-1.0424538	0.0044240346
A_23_P259442	*RTN3*	Homo sapiens reticulon 3 (RTN3), transcript variant 1, mRNA [NM_006054]	-0.7506871	0.004324298
A_23_P372308	*ZXDB*	Homo sapiens zinc finger, X-linked, duplicated B (ZXDB), mRNA [NM_007157]	-0.527289	0.010807059
A_21_P0011599	*CPE*	Homo sapiens carboxypeptidase E (CPE), mRNA [NM_001873]	-3.8581128	0.0024890031
A_23_P38271	*RGMA*	Homo sapiens repulsive guidance molecule family member a (RGMA), transcript variant 4, mRNA [NM_020211]	-1.8822908	0.0073276176
A_21_P0000899	*XLOC_l2_006152*	BROAD Institute lincRNA (XLOC_l2_006152), lincRNA [TCONS_l2_00011380]	-1.1154872	0.0036240483
A_33_P3316555	*MYH2*	Homo sapiens myosin, heavy chain 2, skeletal muscle, adult (MYH2), transcript variant 1, mRNA [NM_017534]	-5.0773964	0.0028913463
A_23_P398275	*LOC100506990*	Homo sapiens uncharacterized LOC100506990 (LOC100506990), transcript variant 1, long non-coding RNA [NR_040091]	-1.860631	0.0029691157
A_33_P3258593	*MGST3*	Homo sapiens microsomal glutathione S-transferase 3 (MGST3), mRNA [NM_004528]	-1.1834002	0.0064825187
A_33_P6474088	*GOLGA2P7*	Homo sapiens golgin A2 pseudogene 7 (GOLGA2P7), non-coding RNA [NR_027001]	-0.9990255	0.011193861

A_23_P5392	*PRB1*	Homo sapiens proline-rich protein BstNI subfamily 1 (PRB1), transcript variant 1, mRNA [NM_005039]	0.68027556	0.016669532
A_19_P00321722	*HCG8*	Homo sapiens HLA complex group 8 (HCG8), long non-coding RNA [NR_103542]	-1.5660026	0.003667703
A_23_P14072	*TP53I3*	Homo sapiens tumor protein p53 inducible protein 3 (TP53I3), transcript variant 1, mRNA [NM_004881]	-1.2716167	0.010203489
A_24_P927474	*lnc-CCDC71L-1*	LNCipedia lincRNA (lnc-CCDC71L-1), lincRNA [lnc-CCDC71L-1:3]	0.5917449	0.011257593
A_23_P395426	*KRT8*	Homo sapiens keratin 8, type II (KRT8), [NM_002273]	0.677562	0.013596905
A_33_P3238290	*DIDO1*	Homo sapiens death inducer-obliterator 1 (DIDO1), [NM_022105]	-0.6475878	0.011110318
A_23_P24515	*KLHDC10*	Homo sapiens kelch domain containing 10 (KLHDC10), [NM_014997]	-0.7966812	0.014874616
A_22_P00008943	*FAM65C*	Homo sapiens family with sequence similarity 65, member C (FAM65C), [NM_001290268]	3.9341965	0.0032869747
A_24_P213354	*ACAT1*	Homo sapiens acetyl-CoA acetyltransferase 1 (ACAT1), [NM_000019]	-0.9748728	0.0048641856
A_23_P114423	*KRT81*	Homo sapiens keratin 81, type II (KRT81), [NM_002281]	0.85638857	0.009543431
A_23_P414519	*RGN*	Homo sapiens regucalcin (RGN), [NM_152869]	-1.3160689	0.0031941293
A_24_P195794	*HS1BP3-IT1*	Homo sapiens HS1BP3 intronic transcript 1 (non-protein coding) (HS1BP3-IT1), [NR_046836]	0.67933565	0.006543285
A_23_P398637	*NRN1*	Homo sapiens neuritin 1 (NRN1), A [NM_016588]	-1.4298284	0.0032379
A_23_P359738	*TRMT10A*	Homo sapiens tRNA methyltransferase 10 homolog A (S. cerevisiae) (TRMT10A), [NM_152292]	-0.69796455	0.013774471
A_23_P114405	*MGC45922*	Homo sapiens uncharacterized LOC284365 (MGC45922), [NR_038359]	1.1207365	0.003190129
A_23_P119102	*EPC2*	Homo sapiens enhancer of polycomb homolog 2 (Drosophila) (EPC2), [NM_015630]	-0.9672353	0.0054708454
A_24_P185036	*MORF4L2*	Homo sapiens mortality factor 4 like 2 (MORF4L2), [NM_012286]	-1.0493786	0.004160909
A_21_P0000496	*VASP*	Homo sapiens vasodilator-stimulated phosphoprotein (VASP), [NM_003370]	-1.1043512	0.009646789
A_33_P3286278	*CMTR2*	Homo sapiens cap methyltransferase 2 (CMTR2), [NM_018348]	-0.8303592	0.0031941293
A_23_P422724	*SNORD1A*	Homo sapiens small nucleolar RNA, C/D box 1A (SNORD1A), small nucleolar RNA [NR_004395]	0.85183364	0.004359316
A_23_P317683	*GRN*	Homo sapiens granulin (GRN), [NM_002087]	-0.89714766	0.0025001846
A_33_P3210203	*PPIC*	Homo sapiens peptidylprolyl isomerase C (cyclophilin C) (PPIC), [NM_000943]	-1.4983683	0.0031941293

A_23_P98022	*TRAPPC10*	Homo sapiens trafficking protein particle complex 10 (TRAPPC10), [NM_003274]	-0.55245733	0.0066119307
A_33_P3382086	*SPIRE2*	Homo sapiens spire-type actin nucleation factor 2 (SPIRE2), [NM_032451]	1.2601818	0.017047435
A_33_P3489737	*SIRT1*	Homo sapiens sirtuin 1 (SIRT1), transcript variant 1, [NM_012238]	-0.855169	0.0042136866
A_23_P121533	*FAM195A*	Homo sapiens family with sequence similarity 195, member A (FAM195A), [NM_138418]	0.7348978	0.017107433
A_33_P3383656	*NLN*	Homo sapiens neurolysin (metallopeptidase M3 family) (NLN), [NM_020726]	-0.61367244	0.014349793
A_23_P106741	*SPON2*	Homo sapiens spondin 2, extracellular matrix protein (SPON2), [NM_012445]	2.8272786	0.0024890031
A_33_P3321205	*DUSP28*	Homo sapiens dual specificity phosphatase 28 (DUSP28), [NM_001033575]	-0.96950006	0.0075099007
A_33_P8922891	*PSMD7*	Homo sapiens proteasome (prosome, macropain) 26S subunit, non-ATPase, 7 (PSMD7), [NM_002811]	-0.7574869	0.013011981
A_23_P11862	*BEGAIN*	Homo sapiens brain-enriched guanylate kinase-associated (BEGAIN), [NM_001159531]	0.9407593	0.012161595
A_21_P0007890	*LOC101927751*	PREDICTED: Homo sapiens uncharacterized LOC101927751 (LOC101927751), [XM_005255009]	-0.8735515	0.0041916566
A_23_P120125	*C1orf112*	Homo sapiens chromosome 1 open reading frame 112 (C1orf112), [NM_018186]	-0.3838904	0.0142741185
A_23_P30474	*COLEC11*	Homo sapiens collectin sub-family member 11 (COLEC11), [NM_199235]	0.6736605	0.011503178
A_23_P77401	*USP47*	Homo sapiens ubiquitin specific peptidase 47 (USP47), [NM_017944]	-0.6830237	0.0080137085
A_23_P4082	*WDR70*	Homo sapiens WD repeat domain 70 (WDR70), [NM_018034]	-0.8802189	0.0038456172
A_23_P209360	*CPPED1*	Homo sapiens calcineurin-like phosphoesterase domain containing 1 (CPPED1), [NM_018340]	-1.2357435	0.004884975
A_23_P305723	*CCT6B*	Homo sapiens chaperonin containing TCP1, subunit 6B (zeta 2) (CCT6B), [NM_006584]	-0.80580026	0.010154543
A_33_P3361741	*KLHL29*	Homo sapiens kelch-like family member 29 (KLHL29), [NM_052920]	-0.9848981	0.008876561
A_22_P00011217	*MIER1*	Homo sapiens mesoderm induction early response 1, transcriptional regulator (MIER1), [NM_020948]	-0.7677695	0.0067047477
A_33_P3228762	*DNAJC15*	Homo sapiens DnaJ (Hsp40) homolog, subfamily C, member 15 (DNAJC15), [NM_013238]	-0.72901934	0.012335783
A_33_P3410589	*SLC25A36*	Homo sapiens solute carrier family 25 (pyrimidine nucleotide carrier), member 36 (SLC25A36), [NM_001104647]	-0.7875261	0.008079168
A_24_P878388	*JADE1*	Homo sapiens jade family PHD finger 1 (JADE1), [NM_001287439]	-1.1256807	0.009387307

A_23_P66682	*FAM43A*	Homo sapiens family with sequence similarity 43, member A (FAM43A), [NM_153690]	-2.075702	0.0027248112
A_23_P154875	*HOXB6*	Homo sapiens homeobox B6 (HOXB6), [NM_018952]	-0.54949373	0.005690581
A_24_P23445	*GSTM5*	Homo sapiens glutathione S-transferase mu 5 (GSTM5), [NM_000851]	1.1462544	0.008208044
A_22_P00001790	*BACE2*	Homo sapiens beta-site APP-cleaving enzyme 2 (BACE2), [NM_012105]	-0.9655502	0.013619302
A_23_P106127	*RNU12*	Homo sapiens RNA, U12 small nuclear (RNU12), small nuclear RNA [NR_029422]	1.4040579	0.0038056036
A_32_P110243	*LOC101928521*	PREDICTED: Homo sapiens uncharacterized LOC101928521 (RP11-1191J2.2), [XR_246615]	-0.319978	0.010353839
A_23_P24215	*KIAA0586*	Homo sapiens KIAA0586 (KIAA0586), [NM_014749]	-0.8718768	0.0073130694
A_33_P3408711	*RPS20P27*	Homo sapiens ribosomal protein S20 pseudogene 27, mRNA (cDNA clone IMAGE:5549882). [BC071734]	0.5756212	0.016331382
(+)E1A_r60_n9	*TBC1D12*	Homo sapiens TBC1 domain family, member 12 (TBC1D12), [NM_015188]	-1.1246215	0.005512448
A_23_P208400	*FLNA*	Homo sapiens filamin A, alpha (FLNA), [NM_001456]	0.52437335	0.0064965826
A_24_P294842	*ADCK4*	Homo sapiens aarF domain containing kinase 4 (ADCK4), [NM_024876]	1.3093541	0.0039659217
A_33_P3368375	*C2orf47*	Homo sapiens chromosome 2 open reading frame 47 (C2orf47), [NM_024520]	-0.6068136	0.014409653
A_33_P3261625	*ATXN1*	Homo sapiens ataxin 1 (ATXN1), [NM_000332]	-1.0991559	0.005858282
A_23_P54963	*RANBP3*	Homo sapiens RAN binding protein 3 (RANBP3), transcript variant RANBP3-d, mRNA [NM_007322]	1.0856864	0.0037006992
A_19_P00813352	*LSM2*	Homo sapiens LSM2 homolog, U6 small nuclear RNA associated (S. cerevisiae) (LSM2), [NM_021177]	-0.75035524	0.0070571043
A_33_P3284453	*MRPL38*	Homo sapiens mitochondrial ribosomal protein L38 (MRPL38), [NM_032478]	1.2368506	0.004026994
A_24_P28811	*lnc-DYM-1*	LNCipedia lincRNA (lnc-DYM-1), lincRNA [lnc-DYM-1:1]	0.47138807	0.010391608
A_33_P3246318	*SOGA3*	Homo sapiens SOGA family member 3 (SOGA3), [NM_001012279]	-1.3270046	0.0065241847
A_33_P3335451	*CYP4F62P*	Homo sapiens cytochrome P450, family 4, subfamily F, polypeptide 62, pseudogene (CYP4F62P), [NR_103761]	0.8167572	0.0029695062
A_23_P118300	*RBX1*	Homo sapiens ring-box 1, E3 ubiquitin protein ligase (RBX1), [NM_014248]	-0.5347498	0.0038056036
A_23_P143474	*PARL*	Homo sapiens presenilin associated, rhomboid-like (PARL), [NM_018622]	-0.29427212	0.015961058
A_19_P00317360	*FAHD1*	Homo sapiens fumarylacetoacetate hydrolase domain containing 1 (FAHD1), [NM_031208]	-0.53192204	0.012161595

A_32_P74409	*ATP5O*	Homo sapiens ATP synthase, H+ transporting, mitochondrial F1 complex, O subunit (ATP5O), [NM_001697]	-0.7408037	0.002819256
A_33_P3243707	*ATP2C1*	Homo sapiens ATPase, Ca++ transporting, type 2C, member 1 (ATP2C1), [NM_001199182]	0.49430043	0.017077666
A_24_P186943	*C11orf96*	Homo sapiens chromosome 11 open reading frame 96 (C11orf96), [NM_001145033]	-2.0937881	0.0036517929
A_33_P3393170	*KLHL20*	Homo sapiens kelch-like family member 20 (KLHL20), [NM_014458]	-0.8743422	0.004213048
A_24_P50437	*ELN*	Homo sapiens elastin (ELN), [NM_000501]	-1.3415893	0.0061524836
A_24_P55250	*CAPN5*	Homo sapiens calpain 5 (CAPN5), [NM_004055]	-0.75565606	0.016394306
A_33_P3240767	*HDDC2*	Homo sapiens HD domain containing 2 (HDDC2), [NM_016063]	-0.6927788	0.011307727
A_23_P52147	*HSPA1A*	Homo sapiens heat shock 70kDa protein 1A (HSPA1A), mRNA [NM_005345]	-0.5613771	0.010561063
A_33_P3814721	*CIDEC*	Homo sapiens cell death-inducing DFFA-like effector c (CIDEC), [NM_022094]	-1.3027607	0.01034603
A_23_P133694	*TBCE*	Homo sapiens tubulin folding cofactor E (TBCE), [NM_001079515]	-0.69851977	0.0034885637
A_23_P19938	*INSC*	Homo sapiens inscuteable homolog (Drosophila) (INSC), [NM_001031853]	2.314537	0.004619079
A_33_P3396522	*SLC29A1*	Homo sapiens solute carrier family 29 (equilibrative nucleoside transporter), member 1 (SLC29A1), [NM_001078177]	1.2733511	0.006573993
A_24_P932416	*KDELR2*	Homo sapiens KDEL (Lys-Asp-Glu-Leu) endoplasmic reticulum protein retention receptor 2 (KDELR2), [NM_006854]	-0.78122854	0.0029691157
A_23_P324994	*POLR2G*	Homo sapiens polymerase (RNA) II (DNA directed) polypeptide G (POLR2G), [NM_002696]	-0.69991326	0.015081194
A_23_P300150	*TMEM14E*	Homo sapiens transmembrane protein 14E (TMEM14E), [NM_001123228]	-1.2294313	0.008897602
A_33_P3223923	*KLHL7*	Homo sapiens kelch-like family member 7 (KLHL7), transcript variant 2, mRNA [NM_018846]	-1.3955355	0.004829709
A_23_P11543	*NFATC1*	Homo sapiens nuclear factor of activated T-cells, cytoplasmic, calcineurin-dependent 1 (NFATC1), transcript variant 3, mRNA [NM_172387]	-0.83292633	0.012795951
A_23_P120335	*PDIA3*	Homo sapiens protein disulfide isomerase family A, member 3 (PDIA3), mRNA [NM_005313]	-0.80066353	0.007501246
A_33_P3326020	*FUCA1*	Homo sapiens fucosidase, alpha-L- 1, tissue (FUCA1), mRNA [NM_000147]	-1.6727782	0.0041523096
A_23_P18649	*MTX2*	Homo sapiens metaxin 2 (MTX2), transcript variant 1, mRNA [NM_006554]	-0.5846019	0.012211056

A_33_P3653330	*KANK3*	KN motif and ankyrin repeat domains 3 [Source:HGNC Symbol;Acc:HGNC:24796] [ENST00000593649]	1.6441647	0.013005072
A_23_P352435	*FAT4*	Homo sapiens FAT atypical cadherin 4 (FAT4), transcript variant 1, mRNA [NM_001291303]	-1.2299498	0.002804883
A_33_P3613358	*MAP2K4*	Homo sapiens mitogen-activated protein kinase kinase 4 (MAP2K4), transcript variant 2, mRNA [NM_001281435]	-0.27864796	0.0117289275
A_33_P3414017	*RGS12*	Homo sapiens regulator of G-protein signaling 12 (RGS12), [NM_002926]	0.80111647	0.008159287
A_23_P72537	*ZNF71*	Homo sapiens zinc finger protein 71 (ZNF71), [NM_021216]	-0.8581549	0.01368361
A_33_P3361337	*SH2D4A*	Homo sapiens SH2 domain containing 4A (SH2D4A), [NM_022071]	-2.1715508	0.004359316
A_23_P205531	*AIFM1*	Homo sapiens apoptosis-inducing factor, mitochondrion-associated, 1 (AIFM1), [NM_004208]	-0.65323234	0.0098649
A_21_P0000366	*TMEM184A*	Homo sapiens transmembrane protein 184A (TMEM184A), [NM_001097620]	1.1970099	0.0066119307
A_23_P334608	*RNASE4*	Homo sapiens ribonuclease, RNase A family, 4 (RNASE4), [NM_001282192]	-1.64616	0.0029497955
A_23_P381449	*SNORD11*	Homo sapiens small nucleolar RNA, C/D box 11 (SNORD11), small nucleolar RNA [NR_003031]	0.97118616	0.012157646
A_23_P169409	*GUSB*	Homo sapiens glucuronidase, beta (GUSB), [NM_000181]	-0.8603697	0.005958252
A_33_P3371224	*SP2*	Homo sapiens Sp2 transcription factor (SP2), [NM_003110]	-0.7297263	0.015772153
A_23_P126241	*RPP25L*	Homo sapiens ribonuclease P/MRP 25kDa subunit-like (RPP25L), [NM_148179]	-0.83998346	0.0068755653
A_33_P3214943	*ANO10*	Homo sapiens anoctamin 10 (ANO10), [NM_001204831]	-0.9309604	0.009658549
A_23_P11652	*EIF4G3*	Homo sapiens eukaryotic translation initiation factor 4 gamma, 3 (EIF4G3), [NM_003760]	-0.56815886	0.009431794
A_23_P119923	*SPOCK2*	Homo sapiens sparc/osteonectin, cwcv and kazal-like domains proteoglycan (testican) 2 (SPOCK2), [NM_001134434]	1.395339	0.009042921
A_23_P213661	*USP1*	Homo sapiens ubiquitin specific peptidase 1 (USP1), [NM_003368]	-0.51424766	0.008628575
A_23_P14083	*CNNM4*	Homo sapiens cyclin and CBS domain divalent metal cation transport mediator 4 (CNNM4), mRNA [NM_020184]	-0.83180135	0.009874361
A_23_P371824	*PPIP5K2*	Homo sapiens diphosphoinositol pentakisphosphate kinase 2 (PPIP5K2), transcript variant 2, mRNA [NM_015216]	-0.82211334	0.0036517929
A_32_P112493	*AMIGO2*	Homo sapiens adhesion molecule with Ig-like domain 2 (AMIGO2), transcript variant 2, mRNA [NM_181847]	-0.60642236	0.011057721

A_24_P328524	*TUFT1*	Homo sapiens tuftelin 1 (TUFT1), transcript variant 1, mRNA [NM_020127]	-1.8238237	0.005017109
A_32_P196047	*PKDCC*	Homo sapiens protein kinase domain containing, cytoplasmic (PKDCC), mRNA [NM_138370]	-1.539002	0.012773474
A_33_P331365 2	*KALRN*	Homo sapiens kalirin, RhoGEF kinase (KALRN), transcript variant 2, mRNA [NM_003947]	-2.959732	0.005254371 6
A_24_P291826	*DPY19L4*	Homo sapiens dpy-19-like 4 (C. elegans) (DPY19L4), mRNA [NM_181787]	-1.0992253	0.008811762
A_23_P27180	*SYTL3*	Homo sapiens synaptotagmin-like 3 (SYTL3), [NM_001009991]	1.7136087	0.006845927 3
A_23_P354387	*FAM27E2*	Homo sapiens family with sequence similarity 27, member E2 (FAM27E2), long non-coding RNA [NR_103714]	-2.6933215	0.010203489
A_33_P342213 3	*NSRP1*	Homo sapiens nuclear speckle splicing regulatory protein 1 (NSRP1), [NM_032141]	-0.65855306	0.005412784 4
A_33_P336312 0	*MYOF*	Homo sapiens myoferlin (MYOF), [NM_013451]	-1.1843555	0.002947112 8
A_23_P202658	*ADAP1*	Homo sapiens ArfGAP with dual PH domains 1 (ADAP1), [NM_006869]	1.1157862	0.010750983
A_33_P329838 7	*SCARNA10*	Homo sapiens small Cajal body-specific RNA 10 (SCARNA10), guide RNA [NR_004387]	-0.6308129	0.012344488
A_33_P330597 4	*GSTP1*	Homo sapiens glutathione S-transferase pi 1 (GSTP1), [NM_000852]	-0.69257855	0.002489003 1
A_23_P258964	*PLK1*	polo-like kinase 1 [Source:HGNC Symbol;Acc:HGNC:9077] [ENST00000570220]	1.2377714	0.010689566
A_24_P54178	*SNAP23*	Homo sapiens synaptosomal-associated protein, 23kDa (SNAP23), [NM_003825]	-1.06668	0.009572463
A_33_P324725 5	*IARS*	Homo sapiens isoleucyl-tRNA synthetase (IARS), [NM_013417]	-0.852127	0.008692265 5
A_23_P68472	*TMED5*	Homo sapiens transmembrane emp24 protein transport domain containing 5 (TMED5), [NM_016040]	-0.4698107	0.011728736
A_22_P000086 69	*TMCO3*	Homo sapiens transmembrane and coiled-coil domains 3 (TMCO3), [NM_017905]	-1.031329	0.005954033 7
A_32_P42054	*DPM1*	Homo sapiens dolichyl-phosphate mannosyltransferase polypeptide 1, catalytic subunit (DPM1), mRNA [NM_003859]	-1.0844915	0.002987366 4
A_23_P256948	*DND1*	Homo sapiens DND microRNA-mediated repression inhibitor 1 (DND1), [NM_194249]	0.7274992	0.007542933
A_23_P56894	*ERV3-1*	Homo sapiens endogenous retrovirus group 3, member 1 (ERV3-1), [NM_001007253]	-1.1970382	0.009129293
A_33_P378989 4	*MSC*	Homo sapiens musculin (MSC), mRNA [NM_005098]	1.8035582	0.003194129 3
A_23_P164057	*CYP20A1*	Homo sapiens cytochrome P450, family 20, subfamily A, polypeptide 1 (CYP20A1), mRNA [NM_177538]	-0.6969446	0.010730268

A_24_P263937	*SNORA75*	PM2-HT0338-051199-001-c04 HT0338 Homo sapiens cDNA, mRNA sequence [AW382724]	0.9504762	0.012858025
A_32_P25050	*MFAP4*	Homo sapiens microfibrillar-associated protein 4 (MFAP4), transcript variant 2, mRNA [NM_002404]	-0.56402606	0.012774024
(+)E1A_r60_n11	*CCDC23*	Homo sapiens coiled-coil domain containing 23 (CCDC23), mRNA [NM_199342]	-0.70745033	0.006442033
A_23_P426663	*RDH10*	Homo sapiens retinol dehydrogenase 10 (all-trans) (RDH10), mRNA [NM_172037]	-1.7935804	0.0028913463
A_23_P355517	*MITF*	Homo sapiens microphthalmia-associated transcription factor (MITF), transcript variant 1, mRNA [NM_198159]	2.0791795	0.00549504
A_23_P117157	*PRKAR1B*	Homo sapiens protein kinase, cAMP-dependent, regulatory, type I, beta (PRKAR1B), transcript variant 1, mRNA [NM_001164761]	0.72967964	0.013441296
A_23_P89780	*SYNPO2L*	Homo sapiens synaptopodin 2-like (SYNPO2L), transcript variant 2, mRNA [NM_024875]	2.7066698	0.0026564114
A_23_P47839	*SUCLA2*	Homo sapiens succinate-CoA ligase, ADP-forming, beta subunit (SUCLA2), mRNA [NM_003850]	-0.8883652	0.002909564
A_23_P333228	*LAMA3*	Homo sapiens laminin, alpha 3 (LAMA3), transcript variant 1, mRNA [NM_198129]	-2.7051709	0.0120937945
A_21_P0005007	*DDX55*	Homo sapiens DEAD (Asp-Glu-Ala-Asp) box polypeptide 55 (DDX55), mRNA [NM_020936]	-0.62841725	0.010203489
A_21_P0002529	*MARCH4*	Homo sapiens membrane-associated ring finger (C3HC4) 4, E3 ubiquitin protein ligase (MARCH4), mRNA [NM_020814]	-0.8994825	0.011772038
A_33_P3323847	*lnc-DEK-1*	LNCipedia lincRNA (lnc-DEK-1), lincRNA [lnc-DEK-1:1]	0.34381586	0.010707513
A_23_P109333	*lnc-SDPR-1*	LNCipedia lincRNA (lnc-SDPR-1), lincRNA [lnc-SDPR-1:1]	1.2422659	0.0051410845
A_23_P160828	*RECQL4*	Homo sapiens RecQ protein-like 4 (RECQL4), mRNA [NM_004260]	0.38013813	0.01032002
A_23_P204850	*C21orf33*	Homo sapiens chromosome 21 open reading frame 33 (C21orf33), transcript variant 1, mRNA [NM_004649]	-0.74808407	0.0065241847
A_23_P90804	*C1orf159*	Homo sapiens chromosome 1 open reading frame 159 (C1orf159), mRNA [NM_017891]	1.7558681	0.0056601833
A_23_P324327	*RB1*	Homo sapiens retinoblastoma 1 (RB1), mRNA [NM_000321]	-0.95044684	0.010273475
A_23_P357101	*MAP4K4*	Homo sapiens mitogen-activated protein kinase kinase kinase kinase 4 (MAP4K4), transcript variant 2, mRNA [NM_145686]	-0.61226815	0.013246798
A_23_P157758	*GPRC5B*	Homo sapiens G protein-coupled receptor, class C, group 5, member B (GPRC5B), mRNA [NM_016235]	2.7693295	0.0031941293
A_23_P207014	*APOBEC3F*	Homo sapiens apolipoprotein B mRNA editing enzyme, catalytic polypeptide-like 3F	-0.15681742	0.013143453

		(APOBEC3F), transcript variant 1, mRNA [NM_145298]		
A_23_P120594	*KIAA2026*	Homo sapiens KIAA2026 (KIAA2026), mRNA [NM_001017969]	-0.6234175	0.012911957
A_23_P114740	*RAD51C*	Homo sapiens RAD51 paralog C (RAD51C), transcript variant 2, mRNA [NM_002876]	-0.41523474	0.016428085
A_24_P115199	*ACSS1*	Homo sapiens acyl-CoA synthetase short-chain family member 1 (ACSS1), transcript variant 1, mRNA [NM_032501]	-2.578216	0.0027709126
A_23_P60016	*CFH*	Homo sapiens complement factor H (CFH), transcript variant 1, mRNA [NM_000186]	2.394916	0.0034885637
A_24_P261052	*FOXK1*	Homo sapiens forkhead box K1 (FOXK1), mRNA [NM_001037165]	-0.85464025	0.006613322
A_33_P3224680	*PTTG3P*	Homo sapiens pituitary tumor-transforming 3, pseudogene (PTTG3P), non-coding RNA [NR_002734]	-0.5410556	0.0052440073
A_33_P3281465	*MTMR9*	Homo sapiens myotubularin related protein 9 (MTMR9), mRNA [NM_015458]	-1.1983631	0.012732773
A_23_P6771	*TRMT13*	tRNA methyltransferase 13 homolog (S. cerevisiae) [Source:HGNC Symbol;Acc:HGNC:25502] [ENST00000370143]	-0.8759215	0.006397939
A_24_P134392	*SLC5A6*	Homo sapiens solute carrier family 5 (sodium/multivitamin and iodide cotransporter), member 6 (SLC5A6), transcript variant 1, mRNA [NM_021095]	1.1687007	0.007925045
A_23_P41437	*LMCD1*	Homo sapiens LIM and cysteine-rich domains 1 (LMCD1), transcript variant 1, mRNA [NM_014583]	-3.3593912	0.002819256
A_23_P128940	*HSPA13*	Homo sapiens heat shock protein 70kDa family, member 13 (HSPA13), mRNA [NM_006948]	-0.8630512	0.004213048
A_23_P337790	*UFSP2*	Homo sapiens UFM1-specific peptidase 2 (UFSP2), transcript variant 1, mRNA [NM_018359]	-0.86433095	0.0055521973
A_24_P305678	*VCPKMT*	Homo sapiens valosin containing protein lysine (K) methyltransferase (VCPKMT), transcript variant 1, mRNA [NM_024558]	-1.0529755	0.0046812603
A_33_P3333587	*SHPRH*	Homo sapiens SNF2 histone linker PHD RING helicase, E3 ubiquitin protein ligase (SHPRH), transcript variant 1, mRNA [NM_001042683]	-0.9728104	0.005852893
A_24_P188377	*PITPNB*	Homo sapiens phosphatidylinositol transfer protein, beta (PITPNB), transcript variant sp1, mRNA [NM_012399]	-0.5102794	0.014434375
A_23_P41804	*FBXL20*	Homo sapiens F-box and leucine-rich repeat protein 20 (FBXL20), transcript variant 1, mRNA [NM_032875]	-1.0390267	0.004701671
A_23_P432626	*CD55*	Homo sapiens CD55 molecule, decay accelerating factor for complement (Cromer	-1.2843204	0.009058357

		blood group) (CD55), transcript variant 1, mRNA [NM_000574]		
A_19_P00320028	*NKD2*	Homo sapiens naked cuticle homolog 2 (Drosophila) (NKD2), transcript variant 1, mRNA [NM_033120]	2.7090821	0.017038407
A_33_P3290667	*SLC25A40*	Homo sapiens solute carrier family 25, member 40 (SLC25A40), mRNA [NM_018843]	-0.5120249	0.014141527
A_33_P3310430	*LINC01004*	Homo sapiens long intergenic non-protein coding RNA 1004 (LINC01004), long non-coding RNA [NR_039981]	-1.7010827	0.0060695945
A_33_P3230818	*SELT*	Homo sapiens selenoprotein T (SELT), mRNA [NM_016275]	-0.42950648	0.014904488
A_33_P3238196	*FAM86B2*	Homo sapiens family with sequence similarity 86, member B2 (FAM86B2), mRNA [NM_001137610]	-1.4621091	0.0039497027
A_23_P101208	*RCAN2*	Homo sapiens regulator of calcineurin 2 (RCAN2), transcript variant 3, mRNA [NM_001251973]	-0.94425404	0.009924993
A_24_P146892	*ZNF33A*	Homo sapiens zinc finger protein 33A (ZNF33A), transcript variant 4, mRNA [NM_001278171]	-0.52980286	0.013432872
A_24_P149036	*CYB5A*	Homo sapiens cytochrome b5 type A (microsomal) (CYB5A), transcript variant 2, mRNA [NM_001914]	-0.7920062	0.010709687
A_23_P57277	*ORAI1*	Homo sapiens ORAI calcium release-activated calcium modulator 1 (ORAI1), mRNA [NM_032790]	0.7646828	0.015762169
A_23_P11295	*DPYSL3*	Homo sapiens dihydropyrimidinase-like 3 (DPYSL3), transcript variant 2, mRNA [NM_001387]	1.2571747	0.004884975
A_33_P3285077	*MAP3K7CL*	Homo sapiens MAP3K7 C-terminal like (MAP3K7CL), transcript variant 1, mRNA [NM_020152]	0.7706268	0.008559404
A_33_P3369567	*CMC4*	Homo sapiens C-x(9)-C motif containing 4 (CMC4), mRNA [NM_001018024]	-1.176034	0.0067319144
A_23_P160240	*COMMD8*	Homo sapiens COMM domain containing 8 (COMMD8), mRNA [NM_017845]	-0.8696296	0.0033094941
A_33_P3413845	*LSP1*	Homo sapiens lymphocyte-specific protein 1 (LSP1), transcript variant 6, mRNA [NM_001289005]	-1.083472	0.009049286
A_23_P110433	*ACP6*	Homo sapiens acid phosphatase 6, lysophosphatidic (ACP6), mRNA [NM_016361]	0.9805323	0.0077268425
A_23_P501887	*TIMM13*	Homo sapiens translocase of inner mitochondrial membrane 13 homolog (yeast) (TIMM13), mRNA [NM_012458]	-0.89520377	0.016092481
A_33_P3357445	*PPWD1*	Homo sapiens peptidylprolyl isomerase domain and WD repeat containing 1 (PPWD1), transcript variant 1, mRNA [NM_015342]	-1.0125372	0.0044240346

A_23_P119857	*DHPS*	Homo sapiens deoxyhypusine synthase (DHPS), transcript variant 2, mRNA [NM_013406]	0.79876715	0.011464552
A_23_P100654	*TDG*	Homo sapiens thymine-DNA glycosylase (TDG), mRNA [NM_003211]	-0.771225	0.010074762
A_33_P3376828	*TTC32*	Homo sapiens tetratricopeptide repeat domain 32 (TTC32), mRNA [NM_001008237]	-1.3783649	0.0038292732
A_21_P0004664	*ZBTB4*	Homo sapiens zinc finger and BTB domain containing 4 (ZBTB4), [NM_020899]	-0.5077233	0.00324898
A_19_P00801042	*CMTM7*	Homo sapiens CKLF-like MARVEL transmembrane domain containing 7 (CMTM7), [NM_138410]	1.3018464	0.010035188
A_22_P00015652	*ZNF90*	zinc finger protein 90 [Source:HGNC Symbol;Acc:HGNC:13165] [ENST00000469078]	-0.71513975	0.002804883
A_21_P0006885	*MAP3K5*	Homo sapiens mitogen-activated protein kinase kinase kinase 5 (MAP3K5), [NM_005923]	1.6445601	0.0054365364
A_23_P37484	*lnc-FAM22B-1*	LNCipedia lincRNA (lnc-FAM22B-1), [lnc-FAM22B-1:6]	-0.93988836	0.012512322
A_32_P72110	*TRPV4*	Homo sapiens transient receptor potential cation channel, subfamily V, member 4 (TRPV4), [NM_147204]	1.2935412	0.009106659
A_33_P3271410	*CHSY1*	Homo sapiens chondroitin sulfate synthase 1 (CHSY1), [NM_014918]	-1.3328016	0.0034766807
A_22_P00011809	*PVR*	Homo sapiens poliovirus receptor (PVR), [NM_006505]	-1.1255972	0.008976597
A_21_P0013556	*THAP9*	Homo sapiens THAP domain containing 9 (THAP9), [NM_024672]	-0.72339594	0.011021474
A_33_P3321657	*XLOC_l2_014711*	BROAD Institute lincRNA (XLOC_l2_014711), lincRNA [TCONS_l2_00028630]	-0.5148959	0.0037583082
A_23_P159255	*MBD6*	Homo sapiens methyl-CpG binding domain protein 6 (MBD6), [NM_052897]	0.8467525	0.01034603
A_23_P146274	*HSPG2*	Homo sapiens heparan sulfate proteoglycan 2 (HSPG2), [NM_001291860]	-1.0376136	0.0069557577
A_24_P122403	*PTPRM*	Homo sapiens protein tyrosine phosphatase, receptor type, M (PTPRM), [NM_002845]	-1.2005131	0.010036303
A_23_P253932	*STMN2*	Homo sapiens stathmin 2 (STMN2), [NM_007029]	-2.0989358	0.0031750489
A_22_P00020312	*TCEB3*	Homo sapiens transcription elongation factor B (SIII), polypeptide 3 (110kDa, elongin A) (TCEB3), [NM_003198]	-0.4080205	0.010773812
A_23_P16166	*FAIM*	Homo sapiens Fas apoptotic inhibitory molecule (FAIM), [NM_001033030]	-0.6531922	0.0058361553
A_23_P53736	*lnc-FKBP2-1*	Q2AC61_9BURK (Q2AC61) HrpW, partial (3%) [THC2716184]	-0.7595861	0.0044240346
A_23_P33326	*PNPLA6*	Homo sapiens patatin-like phospholipase domain containing 6 (PNPLA6), [NM_006702]	1.1511075	0.009874361
A_22_P00007695	*FBXO21*	Homo sapiens F-box protein 21 (FBXO21), [NM_033624]	-0.702343	0.013369118

A_23_P85682	*ADRA1B*	Homo sapiens adrenoceptor alpha 1B (ADRA1B), [NM_000679]	-3.490646	0.0029276514
A_19_P00321890	*lnc-HIST2H2AA3-1*	Homo sapiens mRNA; cDNA DKFZp686A01184 (from clone DKFZp686A01184). [BX648801]	-0.48943347	0.009294179
A_23_P159227	*NFIA*	Homo sapiens nuclear factor I/A (NFIA), [NM_005595]	-1.8814378	0.0053870557
A_22_P00017486	*LINC00894*	Homo sapiens clone 161455-2-3 B cell expressed mRNA from chromosome X. [U66048]	0.9199988	0.008127947
A_24_P270769	*ADAM15*	Homo sapiens ADAM metallopeptidase domain 15 (ADAM15), [NM_207191]	1.147768	0.0055594156
A_22_P00014749	*TMEM88B*	Homo sapiens transmembrane protein 88B (TMEM88B), [NM_001146685]	1.5433215	0.0044707763
A_33_P3214884	*VPS35*	Homo sapiens vacuolar protein sorting 35 homolog (S. cerevisiae) (VPS35), [NM_018206]	-0.6162832	0.015453676
A_23_P153320	*LOC101928861*	Homo sapiens uncharacterized LOC101928861 (LOC101928861), [NR_120513]	0.88118744	0.010126709
A_33_P3383371	*KPNA3*	Homo sapiens karyopherin alpha 3 (importin alpha 4) (KPNA3), [NM_002267]	-0.49267936	0.012756575
A_24_P273143	*ICAM1*	Homo sapiens intercellular adhesion molecule 1 (ICAM1), mRNA [NM_000201]	2.32963	0.005050209
A_24_P382119	*LINC00152*	Homo sapiens long intergenic non-protein coding RNA 152 (LINC00152), transcript variant 1, long non-coding RNA [NR_024204]	-0.5123091	0.010237655
A_23_P324718	*GUCA1B*	Homo sapiens guanylate cyclase activator 1B (retina) (GUCA1B), mRNA [NM_002098]	-0.5071732	0.009387307
A_33_P3310475	*MTMR4*	Homo sapiens myotubularin related protein 4 (MTMR4), mRNA [NM_004687]	-0.9782426	0.0044240346
A_23_P200325	*SYNJ1*	Homo sapiens synaptojanin 1 (SYNJ1), transcript variant 2, mRNA [NM_203446]	-0.83480805	0.014613322
A_23_P364766	*RABGAP1L*	Homo sapiens RAB GTPase activating protein 1-like (RABGAP1L), transcript variant 1, mRNA [NM_014857]	-1.0546916	0.0056819725
A_23_P156497	*TRIT1*	Homo sapiens tRNA isopentenyltransferase 1 (TRIT1), mRNA [NM_017646]	-0.7747574	0.007683989
A_21_P0013213	*SLX4IP*	Homo sapiens SLX4 interacting protein (SLX4IP), mRNA [NM_001009608]	-0.4698548	0.011057721
A_23_P127948	*ELOVL5*	Homo sapiens ELOVL fatty acid elongase 5 (ELOVL5), transcript variant 1, mRNA [NM_021814]	-0.7150397	0.006819766
A_23_P121196	*ZNF727*	PREDICTED: Homo sapiens zinc finger protein 727 (ZNF727), transcript variant X2, misc_RNA [XR_242241]	-1.0793873	0.015175275
A_23_P141021	*ADM*	Homo sapiens adrenomedullin (ADM), mRNA [NM_001124]	-1.6100321	0.0025741379
A_22_P00023679	*TMEM43*	Homo sapiens transmembrane protein 43 (TMEM43), mRNA [NM_024334]	-1.3492954	0.005734989

A_33_P3346132	*LPCAT2*	Homo sapiens lysophosphatidylcholine acyltransferase 2 (LPCAT2), mRNA [NM_017839]	-1.2816185	0.0054365364
A_23_P117778	*lnc-QPCT-3*	LNCipedia lincRNA (lnc-QPCT-3), lincRNA [lnc-QPCT-3:4]	-1.1205086	0.014013276
A_23_P88522	*RUFY2*	Homo sapiens RUN and FYVE domain containing 2 (RUFY2), [NM_017987]	-0.9684755	0.015272768
A_33_P3246985	*UBL7*	Homo sapiens ubiquitin-like 7 (UBL7), [NM_032907]	1.3924723	0.0076440917
A_23_P108641	*NMB*	Homo sapiens neuromedin B (NMB), [NM_021077]	-0.72081035	0.014728144
A_23_P116743	*PDE4DIP*	Homo sapiens phosphodiesterase 4D interacting protein (PDE4DIP), [NM_022359]	-0.9772949	0.007683969
A_33_P3336387	*FAM136A*	Homo sapiens family with sequence similarity 136, member A (FAM136A), [NM_032822]	-0.68077254	0.016348667
A_32_P195401	*LINC01089*	Homo sapiens long intergenic non-protein coding RNA 1089 (LINC01089), [NR_002809]	0.83716965	0.011341994
A_33_P3258091	*PAOX*	Homo sapiens polyamine oxidase (exo-N4-amino) (PAOX), [NM_207128]	0.8877548	0.013529562
A_22_P00003981	*FAM117B*	Homo sapiens family with sequence similarity 117, member B (FAM117B), [NM_173511]	-1.5273812	0.0072648157
A_23_P43141	*RNPEP*	Homo sapiens arginyl aminopeptidase (aminopeptidase B) (RNPEP), mRNA [NM_020216]	-1.3448534	0.0062004877
A_23_P126486	*EIF3E*	Homo sapiens eukaryotic translation initiation factor 3, subunit E (EIF3E), [NM_001568]	-0.5519829	0.004055852
A_24_P383609	*CROCCP2*	Homo sapiens ciliary rootlet coiled-coil, rootletin pseudogene 2 (CROCCP2), [NR_026752]	-1.5405736	0.004801003
A_24_P21887	*PDHA1*	Homo sapiens pyruvate dehydrogenase (lipoamide) alpha 1 (PDHA1), [NM_000284]	-0.6498975	0.01174194
A_23_P336554	*NANOS1*	Homo sapiens nanos homolog 1 (Drosophila) (NANOS1), mRNA [NM_199461]	-0.93073946	0.011360915
A_23_P106617	*PLCH2*	Homo sapiens phospholipase C, eta 2 (PLCH2), [NM_014638]	-0.44997883	0.00929522
A_23_P83498	*IL1RAP*	Homo sapiens interleukin 1 receptor accessory protein (IL1RAP), transcript variant 2, mRNA [NM_134470]	-0.45306623	0.012538933
A_33_P3245977	*WFDC1*	Homo sapiens WAP four-disulfide core domain 1 (WFDC1), transcript variant 1, mRNA [NM_021197]	2.1256454	0.0063513564
A_24_P230938	*IGF2BP1*	Homo sapiens insulin-like growth factor 2 mRNA binding protein 1 (IGF2BP1), transcript variant 1, mRNA [NM_006546]	0.95752597	0.006485141
A_23_P58877	*SHROOM1*	Homo sapiens shroom family member 1 (SHROOM1), transcript variant 2, mRNA [NM_133456]	0.90618163	0.01294906

A_19_P00317824	*MORN4*	Homo sapiens MORN repeat containing 4, mRNA (cDNA clone IMAGE:4690584), complete cds. [BC022054]	-1.2194483	0.0066045914
A_33_P3338724	*GOPC*	Homo sapiens golgi-associated PDZ and coiled-coil motif containing (GOPC), transcript variant 1, mRNA [NM_020399]	-0.7632909	0.006155321
A_23_P127233	*JPX*	JPX transcript, XIST activator (non-protein coding) [Source:HGNC Symbol;Acc:HGNC:37191] [ENST00000415215]	-0.9221504	0.0062702326
A_23_P19322	*LENG9*	Homo sapiens leukocyte receptor cluster (LRC) member 9 (LENG9), transcript variant 1, mRNA [NM_198988]	-1.1439991	0.011575981
A_22_P00025425	*SMNDC1*	Homo sapiens survival motor neuron domain containing 1 (SMNDC1), mRNA [NM_005871]	-0.9188564	0.0048260237
A_21_P0009158	*SAYSD1*	Homo sapiens SAYSVFN motif domain containing 1 (SAYSD1), mRNA [NM_018322]	-0.9591983	0.0053870557
A_33_P3274930	*lnc-ARMC6-1*	LNCipedia lincRNA (lnc-ARMC6-1), lincRNA [lnc-ARMC6-1:1]	1.0997517	0.006395745
A_33_P3353030	*KDM6A*	Homo sapiens lysine (K)-specific demethylase 6A (KDM6A), transcript variant 1, mRNA [NM_001291415]	-0.33726937	0.0073048193
A_23_P127175	*SHFM1*	Homo sapiens split hand/foot malformation (ectrodactyly) type 1 (SHFM1), mRNA [NM_006304]	-0.5386066	0.005377644
A_21_P0000120	*UCN*	Homo sapiens urocortin (UCN), mRNA [NM_003353]	-0.63735664	0.009864289
A_23_P121215	*SAR1A*	Homo sapiens secretion associated, Ras related GTPase 1A (SAR1A), transcript variant 2, mRNA [NM_020150]	-0.7363176	0.00544143
A_33_P3366221	*KCNJ18*	Homo sapiens potassium channel, inwardly rectifying subfamily J, member 18 (KCNJ18), mRNA [NM_001194958]	-4.4038215	0.0031941293
A_19_P00317759	*CAMK1*	Homo sapiens calcium/calmodulin-dependent protein kinase I (CAMK1), mRNA [NM_003656]	1.5602689	0.00978609
A_23_P310911	*NTNG1*	Homo sapiens netrin G1 (NTNG1), transcript variant 3, mRNA [NM_014917]	-0.9695072	0.013496047
A_23_P331908	*lnc-NR5A2-1*	Homo sapiens clone HA_003012 unknown mRNA. [EU250746]	2.0062516	0.0052045966
A_23_P52806	*BLMH*	Homo sapiens bleomycin hydrolase (BLMH), mRNA [NM_000386]	-0.76546985	0.004453573
A_23_P113803	*BACE1*	Homo sapiens beta-site APP-cleaving enzyme 1 (BACE1), transcript variant a, mRNA [NM_012104]	-1.5104886	0.0026950072
A_33_P3341429	*GYPA*	Homo sapiens glycophorin A (MNS blood group) (GYPA), mRNA [NM_002099]	0.8033737	0.017010119
A_33_P3229722	*KATNA1*	Homo sapiens katanin p60 (ATPase containing) subunit A 1 (KATNA1), transcript variant 1, mRNA [NM_007044]	-1.2268085	0.0034766807

A_23_P215634	*NEXN*	Homo sapiens nexilin (F actin binding protein) (NEXN), transcript variant 1, mRNA [NM_144573]	-0.64935255	0.005098191
A_33_P3275668	*PSMF1*	Homo sapiens proteasome (prosome, macropain) inhibitor subunit 1 (PI31) (PSMF1), transcript variant 1, mRNA [NM_006814]	1.0089245	0.010237655
A_23_P71415	*IGFBP3*	Homo sapiens insulin-like growth factor binding protein 3 (IGFBP3), transcript variant 1, mRNA [NM_001013398]	-2.6618638	0.00180587
A_22_P00002489	*BAG6*	Homo sapiens BCL2-associated athanogene 6 (BAG6), transcript variant 1, mRNA [NM_004639]	-0.7018337	0.016892996
A_23_P160869	*WDYHV1*	Homo sapiens WDYHV motif containing 1 (WDYHV1), transcript variant 1, mRNA [NM_018024]	-0.5953922	0.009039211
A_23_P127367	*lnc-C16orf45-1*	LNCipedia lincRNA (lnc-C16orf45-1), lincRNA [lnc-C16orf45-1:1]	-0.7255065	0.0064857528
A_21_P0013105	*LRIG2*	Homo sapiens leucine-rich repeats and immunoglobulin-like domains 2 (LRIG2), mRNA [NM_014813]	-0.6647517	0.011870703
A_32_P178945	*POLD4*	Homo sapiens polymerase (DNA-directed), delta 4, accessory subunit (POLD4), transcript variant 1, mRNA [NM_021173]	-1.0019345	0.006573993
A_33_P3361811	*YOD1*	Homo sapiens YOD1 deubiquitinase (YOD1), [NM_018566]	-1.0059707	0.007442623
A_23_P149775	*ATP8B1*	Homo sapiens ATPase, aminophospholipid transporter, class I, type 8B, member 1 (ATP8B1), [NM_005603]	-1.1227853	0.0025001846
A_33_P3618561	*NAA16*	Homo sapiens N(alpha)-acetyltransferase 16, NatA auxiliary subunit (NAA16), [NM_024561]	0.77411515	0.0025001846
A_22_P00002015	*ARHGAP12*	Homo sapiens Rho GTPase activating protein 12 (ARHGAP12), [NM_018287]	-1.0189173	0.004359316
A_33_P3272442	*LOC730961*	Homo sapiens cDNA clone IMAGE:4826905. [BC034282]	-0.49621782	0.01526263
A_22_P00003693	*lnc-BDNF-2*	Homo sapiens trapped 3' terminal exon, clone C2D9. [AJ011602]	0.7047826	0.013925071
A_33_P3259722	*ZSCAN16-AS1*	Homo sapiens ZSCAN16 antisense RNA 1 (ZSCAN16-AS1), [NR_103455]	-1.6477976	0.0030069214
A_23_P422933	*LOC101927650*	Homo sapiens uncharacterized LOC101927650 (LOC101927650), [NR_110918]	2.444348	0.0119697815
A_24_P417526	*TMEM222*	Homo sapiens transmembrane protein 222 (TMEM222), [NM_032125]	0.7606437	0.012728797
A_23_P112004	*ARHGAP20*	Homo sapiens Rho GTPase activating protein 20 (ARHGAP20), [NM_020809]	-1.4258231	0.0056123743
A_22_P00024912	*FRG1B*	Homo sapiens FSHD region gene 1 family, member B (FRG1B), [NR_003579]	-0.9530697	0.007206317
A_33_P3283083	*LRRC6*	Homo sapiens leucine rich repeat containing 6 (LRRC6),[NM_012472]	-2.7346616	0.010804776

A_24_P366165	*LOC102723366*	PREDICTED: Homo sapiens uncharacterized LOC102723366 (LOC102723366), ncRNA [XR_424492]	0.7854801	0.010642556
A_23_P131935	*INPP4B*	Homo sapiens inositol polyphosphate-4-phosphatase, type II, 105kDa (INPP4B), [NM_003866]	1.6494086	0.003162355
A_21_P0012106	*FERMT1*	Homo sapiens fermitin family member 1 (FERMT1), [NM_017671]	1.1147124	0.0071447864
A_23_P388871	*PLAU*	Homo sapiens plasminogen activator, urokinase (PLAU), [NM_002658]	3.5819368	0.0032831472
A_23_P257417	*LINC00607*	Homo sapiens long intergenic non-protein coding RNA 607 (LINC00607), [NR_037195]	-1.9431611	0.004297874
A_23_P157136	*HIST4H4*	Homo sapiens histone cluster 4, H4 (HIST4H4), mRNA [NM_175054]	1.3172168	0.003956336
A_33_P3341676	*CLHC1*	Homo sapiens clathrin heavy chain linker domain containing 1 (CLHC1), transcript variant 1, mRNA [NM_152385]	-1.251188	0.0048260237
A_33_P3238461	*SCIN*	Homo sapiens scinderin (SCIN), transcript variant 2, mRNA [NM_033128]	-1.559459	0.0059861154
A_22_P00002430	*MEF2A*	Homo sapiens myocyte enhancer factor 2A (MEF2A), transcript variant 5, mRNA [NM_001171894]	-1.0945593	0.0079484265
A_23_P109907	*lnc-C14orf37-1*	Homo sapiens cDNA clone IMAGE:5276765. [BC031314]	-1.737165	0.0042908904
A_23_P12173	*lnc-CTTN-3*	AGENCOURT_25900990 NIH_MGC_195 Homo sapiens cDNA clone IMAGE:7286376 5', mRNA sequence [CO247556]	0.701513	0.011772038
A_23_P357811	*ILDR1*	Homo sapiens immunoglobulin-like domain containing receptor 1 (ILDR1), transcript variant 2, mRNA [NM_175924]	-0.7973521	0.005644507
A_24_P252575	*CRTC2*	Homo sapiens CREB regulated transcription coactivator 2 (CRTC2), mRNA [NM_181715]	-0.93665975	0.0056601833
A_33_P3359115	*MBNL1*	Homo sapiens muscleblind-like splicing regulator 1 (MBNL1), transcript variant 1, mRNA [NM_021038]	-0.9636254	0.0039659217
A_23_P138125	*RABGAP1*	Homo sapiens RAB GTPase activating protein 1 (RABGAP1), mRNA [NM_012197]	-1.9071786	0.004026994
A_33_P3420167	*LMBRD1*	Homo sapiens LMBR1 domain containing 1 (LMBRD1), mRNA [NM_018368]	-1.1072747	0.0039522685
A_23_P205841	*FAIM3*	Homo sapiens Fas apoptotic inhibitory molecule 3 (FAIM3), transcript variant 1, mRNA [NM_005449]	2.9096687	0.0032522907
A_24_P219971	*MYO9A*	Homo sapiens myosin IXA (MYO9A), mRNA [NM_006901]	-0.94364595	0.008831622
A_33_P3217819	*GID8*	Homo sapiens GID complex subunit 8 (GID8), mRNA [NM_017896]	-0.6426869	0.012460022

A_24_P102981	*MGEA5*	Homo sapiens meningioma-expressed antigen 5s splice variant mRNA, complete cds. [AF307332]	-0.5817533	0.0122128595
A_33_P3267612	*CCNE2*	Homo sapiens cyclin E2 (CCNE2), mRNA [NM_057749]	0.79809844	0.0109915305
A_33_P3246613	*DNAJB2*	Homo sapiens DnaJ (Hsp40) homolog, subfamily B, member 2 (DNAJB2), transcript variant 2, mRNA [NM_006736]	-1.2273879	0.009242212
A_23_P64567	*MIR143HG*	Homo sapiens MIR143 host gene (non-protein coding) (MIR143HG), transcript variant 1, long non-coding RNA [NR_105059]	-1.4212191	0.0060077314
A_33_P3303355	*CCDC78*	Homo sapiens coiled-coil domain containing 78 (CCDC78), mRNA [NM_001031737]	1.0830445	0.005842833
A_22_P00003395	*PPME1*	Homo sapiens protein phosphatase methylesterase 1 (PPME1), transcript variant 1, mRNA [NM_016147]	0.9356001	0.010801365
A_33_P3328609	*ZCCHC18*	zinc finger, CCHC domain containing 18 [Source:HGNC Symbol;Acc:HGNC:32459] [ENST00000605784]	1.086248	0.0062159016
A_23_P377616	*LOC102725059*	PREDICTED: Homo sapiens uncharacterized LOC102725059 (LOC102725059), transcript variant X2, ncRNA [XR_433694]	-0.7799506	0.008989235
A_23_P138514	*PKP4*	Homo sapiens plakophilin 4 (PKP4), transcript variant 1, mRNA [NM_003628]	-1.0802376	0.008472645
A_24_P810290	*CIRBP*	cold inducible RNA binding protein [Source:HGNC Symbol;Acc:HGNC:1982] [ENST00000621399]	-2.0636473	0.0044813035
A_23_P50942	*COMMD3*	Homo sapiens COMM domain containing 3 (COMMD3), mRNA [NM_012071]	-1.0640916	0.0066818837
A_23_P309973	*PPAPDC1A*	Homo sapiens phosphatidic acid phosphatase type 2 domain containing 1A (PPAPDC1A), mRNA [NM_001030059]	0.8081839	0.013367055
A_24_P712562	*RAB3GAP1*	Homo sapiens RAB3 GTPase activating protein subunit 1 (catalytic) (RAB3GAP1), transcript variant 2, mRNA [NM_012233]	-1.2303443	0.0061486606
A_23_P89431	*PPP1R36*	Homo sapiens protein phosphatase 1, regulatory subunit 36 (PPP1R36), mRNA [NM_172365]	-0.70925725	0.0131854955
A_21_P0013993	*C17orf67*	Homo sapiens chromosome 17 open reading frame 67 (C17orf67), mRNA [NM_001085430]	-0.94990796	0.011674624
A_23_P50907	*CCL2*	Homo sapiens chemokine (C-C motif) ligand 2 (CCL2), mRNA [NM_002982]	2.6030285	0.003557021
A_33_P3285824	*FAM104B*	Homo sapiens family with sequence similarity 104, member B (FAM104B), transcript variant 1, mRNA [NM_138362]	-0.95672613	0.0075041396
A_23_P130194	*ITGAV*	Homo sapiens integrin, alpha V (ITGAV), transcript variant 1, mRNA [NM_002210]	-0.98970604	0.005581195

A_24_P273799	*C4orf3*	Homo sapiens chromosome 4 open reading frame 3 (C4orf3), transcript variant 1, mRNA [NM_001170330]	-1.1438687	0.0029695062
A_33_P3280666	*PYCR1*	Homo sapiens pyrroline-5-carboxylate reductase 1 (PYCR1), transcript variant 1, mRNA [NM_006907]	-0.8939915	0.005644507
A_23_P98483	*ZNF641*	Homo sapiens zinc finger protein 641 (ZNF641), transcript variant 1, mRNA [NM_152320]	-0.6603052	0.008705128
A_33_P3373892	*PHF21B*	Homo sapiens PHD finger protein 21B (PHF21B), transcript variant 1, mRNA [NM_138415]	0.9523824	0.01240238
A_21_P0014389	*ZBED5*	Homo sapiens zinc finger, BED-type containing 5 (ZBED5), transcript variant 1, mRNA [NM_021211]	-0.97975606	0.009630889
A_33_P3291569	*NAA35*	N(alpha)-acetyltransferase 35, NatC auxiliary subunit [Source:HGNC Symbol;Acc:HGNC:24340] [ENST00000376040]	-1.0987537	0.0051266714
A_23_P58266	*ACTR3*	Homo sapiens ARP3 actin-related protein 3 homolog (yeast) (ACTR3), transcript variant 1, mRNA [NM_005721]	-1.017873	0.0031750489
A_21_P0003757	*LUC7L3*	Homo sapiens LUC7-like 3 (S. cerevisiae) (LUC7L3), transcript variant 1, mRNA [NM_016424]	-0.75772923	0.009507545
A_24_P67534	*S100P*	Homo sapiens S100 calcium binding protein P (S100P), mRNA [NM_005980]	1.8200165	0.0051457407
A_23_P66637	*lnc-ARSJ-1*	LNCipedia lincRNA (lnc-ARSJ-1), lincRNA [lnc-ARSJ-1:6]	-1.0999181	0.009468359
A_33_P3418496	*SMIM10*	Homo sapiens small integral membrane protein 10 (SMIM10), mRNA [NM_001163438]	1.1364673	0.010626791
A_33_P3336592	*SGCA*	Homo sapiens sarcoglycan, alpha (50kDa dystrophin-associated glycoprotein) (SGCA), transcript variant 1, mRNA [NM_000023]	-3.7940688	0.003121324
A_23_P134744	*KLF13*	Homo sapiens cDNA FLJ45109 fis, clone BRAWH3034097. [AK127052]	1.16894	0.006111203
A_33_P3336622	*CARF*	Homo sapiens calcium responsive transcription factor (CARF), transcript variant 2, mRNA [NM_024744]	-1.119871	0.011156819
A_23_P258698	*RNF122*	Homo sapiens ring finger protein 122 (RNF122), mRNA [NM_024787]	-1.6042696	0.002535607
A_23_P141974	*ALDH3A2*	Homo sapiens aldehyde dehydrogenase 3 family, member A2 (ALDH3A2), transcript variant 1, mRNA [NM_001031806]	-1.2200661	0.007730128
A_23_P109269	*MANBA*	Homo sapiens mannosidase, beta A, lysosomal (MANBA), mRNA [NM_005908]	-0.8407781	0.011788845
A_24_P303097	*TPM4*	Homo sapiens tropomyosin 4 (TPM4), transcript variant Tpm4.2, mRNA [NM_003290]	-0.82949543	0.004160909

A_21_P0014494	*LAMA5*	Homo sapiens laminin, alpha 5 (LAMA5), mRNA [NM_005560]	1.3838621	0.016059838
A_23_P77174	*SNX25*	Homo sapiens sorting nexin 25 (SNX25), mRNA [NM_031953]	-1.1509372	0.008094475
A_24_P557479	*CTC-338M12.4*	Homo sapiens uncharacterized LOC101928649 (CTC-338M12.4), long non-coding RNA [NR_109909]	-0.97565794	0.013016004
A_24_P97526	*PIGB*	Homo sapiens phosphatidylinositol glycan anchor biosynthesis, class B (PIGB), mRNA [NM_004855]	-0.41892964	0.005752781
A_23_P386478	*XAF1*	Homo sapiens XIAP associated factor 1 (XAF1), transcript variant 1, mRNA [NM_017523]	-1.3768356	0.008298705
A_21_P0007429	*CMTM6*	Homo sapiens CKLF-like MARVEL transmembrane domain containing 6 (CMTM6), mRNA [NM_017801]	-0.7212131	0.00511559
A_32_P26969	*TNIP3*	Homo sapiens TNFAIP3 interacting protein 3 (TNIP3), transcript variant 1, mRNA [NM_024873]	-1.6399871	0.005852893
A_23_P16032	*lnc-RP1-239B22.1.1-1*	LNCipedia lincRNA (lnc-RP1-239B22.1.1-1), lincRNA [lnc-RP1-239B22.1.1-1:2]	0.9985497	0.00856985
A_23_P127140	*ERICH1*	Homo sapiens glutamate-rich 1 (ERICH1), transcript variant 1, mRNA [NM_207332]	-0.7767017	0.009795873
A_33_P3419460	*TRAPPC2B*	Homo sapiens trafficking protein particle complex 2B (TRAPPC2B), non-coding RNA [NR_002166]	-0.8226578	0.009817055
A_23_P256735	*RAB11FIP2*	Homo sapiens RAB11 family interacting protein 2 (class I) (RAB11FIP2), mRNA [NM_014904]	-1.2484447	0.011193861
A_23_P114155	*VAPA*	Homo sapiens VAMP (vesicle-associated membrane protein)-associated protein A, 33kDa (VAPA), transcript variant 1, mRNA [NM_003574]	-0.18654591	0.0096807415
A_33_P3511265	*CPQ*	Homo sapiens carboxypeptidase Q (CPQ), mRNA [NM_016134]	-1.6078511	0.0054341624
A_21_P0000200	*GPKOW*	Homo sapiens G patch domain and KOW motifs (GPKOW), mRNA [NM_015698]	-0.92172176	0.0115902675
A_23_P26854	*POSTN*	Homo sapiens periostin, osteoblast specific factor (POSTN), transcript variant 1, mRNA [NM_006475]	-1.4369125	0.0034885637
A_24_P364970	*MCPH1*	Homo sapiens microcephalin 1 (MCPH1), transcript variant 1, mRNA [NM_024596]	-0.9762727	0.009630487
A_23_P350886	*ARHGAP44*	Homo sapiens Rho GTPase activating protein 44 (ARHGAP44), mRNA [NM_014859]	-2.5249734	0.00477571
A_23_P103201	*DHX33*	Homo sapiens DEAH (Asp-Glu-Ala-His) box polypeptide 33 (DHX33), transcript variant 1, mRNA [NM_020162]	-0.6019366	0.012436811
A_33_P3374758	*GLTSCR1L*	Homo sapiens GLTSCR1-like (GLTSCR1L), mRNA [NM_015349]	-0.9107887	0.0052660066

A_21_P0010580	*PNRC2*	Homo sapiens proline-rich nuclear receptor coactivator 2 (PNRC2), mRNA [NM_017761]	-0.74888605	0.0057647754
A_23_P50399	*DYNC1LI2*	Homo sapiens dynein, cytoplasmic 1, light intermediate chain 2 (DYNC1LI2), transcript variant 1, mRNA [NM_006141]	-1.0909067	0.005215599
A_23_P106835	*XLOC_l2_000657*	BROAD Institute lincRNA (XLOC_l2_000657), lincRNA [TCONS_l2_00000881]	-0.89852184	0.013836516
A_23_P131348	*DCAF15*	Homo sapiens DDB1 and CUL4 associated factor 15 (DCAF15), mRNA [NM_138353]	1.8767667	0.005192162
A_32_P86763	*BBS2*	Homo sapiens Bardet-Biedl syndrome 2 (BBS2), mRNA [NM_031885]	-1.0909213	0.004359316
A_33_P3373541	*THUMPD2*	Homo sapiens THUMP domain containing 2 (THUMPD2), transcript variant 1, mRNA [NM_025264]	-0.6335355	0.008691653
A_24_P295543	*TGM2*	Homo sapiens transglutaminase 2 (TGM2), transcript variant 1, mRNA [NM_004613]	2.0480566	0.0066194455
A_24_P142095	*ELAC2*	Homo sapiens elaC ribonuclease Z 2 (ELAC2), transcript variant 2, mRNA [NM_173717]	0.7902782	0.012864988
A_32_P60065	*BLOC1S2*	Homo sapiens biogenesis of lysosomal organelles complex-1, subunit 2 (BLOC1S2), transcript variant 2, mRNA [NM_001001342]	-0.9493172	0.004437345
A_24_P143138	*AKAP13*	Homo sapiens A kinase (PRKA) anchor protein 13 (AKAP13), transcript variant 1, mRNA [NM_006738]	-0.99538493	0.01389265
A_23_P134078	*F2RL2*	Homo sapiens coagulation factor II (thrombin) receptor-like 2 (F2RL2), transcript variant 1, mRNA [NM_004101]	-3.4138541	0.003162355
A_24_P305764	*FGD1*	Homo sapiens FYVE, RhoGEF and PH domain containing 1 (FGD1), mRNA [NM_004463]	0.7514522	0.005852893
A_24_P9883	*CDYL*	Homo sapiens chromodomain protein, Y-like (CDYL), transcript variant 1, mRNA [NM_004824]	-0.9638862	0.0069557577
A_21_P0011684	*SMS*	Homo sapiens spermine synthase (SMS), transcript variant 1, mRNA [NM_004595]	-0.36017033	0.014307808
A_23_P78835	*AP5B1*	Homo sapiens adaptor-related protein complex 5, beta 1 subunit (AP5B1), mRNA [NM_138368]	0.72530854	0.015893938
A_32_P142652	*XLOC_l2_006665*	BROAD Institute lincRNA (XLOC_l2_006665), lincRNA [TCONS_l2_00012419]	0.8463179	0.0072226366
A_23_P22682	*ZNF787*	Homo sapiens zinc finger protein 787 (ZNF787), mRNA [NM_001002836]	0.75630474	0.011491425
A_33_P3267150	*ARMCX1*	Homo sapiens armadillo repeat containing, X-linked 1 (ARMCX1), mRNA [NM_016608]	-0.97632074	0.0043867165
A_23_P148959	*F11R*	Homo sapiens F11 receptor (F11R), mRNA [NM_016946]	-1.3896459	0.0034924159
A_23_P40049	*TM2D1*	Homo sapiens TM2 domain containing 1 (TM2D1), mRNA [NM_032027]	-1.0158416	0.0071605598

A_23_P114947	*TOP1*	Homo sapiens topoisomerase (DNA) I (TOP1), mRNA [NM_003286]	0.8472102	0.005899316
A_23_P121926	*CAD*	Homo sapiens carbamoyl-phosphate synthetase 2, aspartate transcarbamylase, and dihydroorotase (CAD), mRNA [NM_004341]	0.93455744	0.011110267
A_33_P3302125	*RGS2*	Homo sapiens regulator of G-protein signaling 2 (RGS2), mRNA [NM_002923]	1.93802	0.0031941293
A_33_P3242873	*SEPP1*	Homo sapiens selenoprotein P, plasma, 1 (SEPP1), transcript variant 1, mRNA [NM_005410]	2.5534027	0.002819256
A_33_P3334843	*LCE2A*	Homo sapiens late cornified envelope 2A (LCE2A), mRNA [NM_178428]	2.6551194	0.004759128
A_23_P111037	*MPC1*	Homo sapiens mitochondrial pyruvate carrier 1 (MPC1), transcript variant 2, mRNA [NM_001270879]	-1.697033	0.010004596
A_23_P90612	*PLD1*	phospholipase D1, phosphatidylcholine-specific [Source:HGNC Symbol;Acc:HGNC:9067] [ENST00000331659]	0.5575692	0.011590999
A_23_P404005	*HIST1H3A*	Homo sapiens histone cluster 1, H3a (HIST1H3A), mRNA [NM_003529]	-1.1690407	0.0037353234
A_22_P00002540	*MCM6*	Homo sapiens minichromosome maintenance complex component 6 (MCM6), mRNA [NM_005915]	-0.4633243	0.015322128
A_33_P3398526	*FAM58A*	Homo sapiens family with sequence similarity 58, member A (FAM58A), transcript variant 1, mRNA [NM_152274]	-0.5427654	0.010633319
A_33_P3377194	*lnc-C17orf62-1*	293T_111 SVA 5' RACE from 293T cell line Homo sapiens cDNA, mRNA sequence [GR564636]	0.8011272	0.011747539
A_22_P00013349	*BCL2L11*	Homo sapiens BCL2-like 11 (apoptosis facilitator) (BCL2L11), transcript variant 9, mRNA [NM_207002]	0.5579446	0.010567525
A_23_P153256	*ADRA1A*	Homo sapiens adrenoceptor alpha 1A (ADRA1A), transcript variant 4, mRNA [NM_033304]	0.36053327	0.009341755
A_33_P3404959	*LOC100507420*	Homo sapiens uncharacterized LOC100507420 (LOC100507420), transcript variant 1, long non-coding RNA [NR_121620]	-0.8223049	0.015647346
A_32_P305888	*ZNF773*	zinc finger protein 773 [Source:HGNC Symbol;Acc:HGNC:30487] [ENST00000598770]	-1.5761964	0.0072230194
A_33_P3261167	*C2orf68*	Homo sapiens chromosome 2 open reading frame 68 (C2orf68), mRNA [NM_001013649]	-1.3744282	0.005272309
A_21_P0000230	*SH3TC2*	Homo sapiens SH3 domain and tetratricopeptide repeats 2 (SH3TC2), mRNA [NM_024577]	-0.16511922	0.008318697
A_22_P00010404	*GNS*	Homo sapiens glucosamine (N-acetyl)-6-sulfatase (GNS), mRNA [NM_002076]	-0.9358792	0.008094475

A_33_P3388080	*SNORD107*	Homo sapiens small nucleolar RNA, C/D box 107 (SNORD107), small nucleolar RNA [NR_001293]	0.58685637	0.004437345
A_24_P110558	*lnc-MYO1D-1*	Homo sapiens cDNA clone IMAGE:5273098. [BC037534]	1.4845378	0.0024890031
A_23_P24960	*LINC01220*	long intergenic non-protein coding RNA 1220 [Source:HGNC Symbol;Acc:HGNC:49664] [ENST00000558575]	0.2641688	0.0111169275
A_21_P0004995	*IGIP*	Homo sapiens IgA-inducing protein (IGIP), mRNA [NM_001007189]	-1.3568103	0.0034766807
A_33_P3253975	*NARS2*	Homo sapiens asparaginyl-tRNA synthetase 2, mitochondrial (putative) (NARS2), transcript variant 1, mRNA [NM_024678]	-0.8745452	0.0065241847
A_22_P00000618	*lnc-ECI2-3*	LNCipedia lincRNA (lnc-ECI2-3), lincRNA [lnc-ECI2-3:1]	2.290902	0.006030567
A_32_P196854	*ACAT1*	Homo sapiens acetyl-CoA acetyltransferase 1 (ACAT1), mRNA [NM_000019]	-0.71553254	0.009431794
A_23_P41424	*FAM76A*	Homo sapiens family with sequence similarity 76, member A (FAM76A), transcript variant 1, mRNA [NM_001143912]	-1.0029956	0.0046812603
A_23_P133582	*TOMM22*	Homo sapiens translocase of outer mitochondrial membrane 22 homolog (yeast) (TOMM22), mRNA [NM_020243]	-0.52335733	0.010561557
A_33_P3213064	*SLC39A8*	Homo sapiens solute carrier family 39 (zinc transporter), member 8 (SLC39A8), transcript variant 1, mRNA [NM_022154]	1.2514075	0.0037096923
A_33_P3225522	*ETF1*	Homo sapiens eukaryotic translation termination factor 1 (ETF1), transcript variant 1, mRNA [NM_004730]	-0.4622335	0.008465412
A_22_P00010628	*STAT2*	Homo sapiens signal transducer and activator of transcription 2, 113kDa (STAT2), transcript variant 1, mRNA [NM_005419]	-1.0987127	0.0034310205
A_21_P0011712	*OAS2*	Homo sapiens 2'-5'-oligoadenylate synthetase 2, 69/71kDa (OAS2), transcript variant 3, mRNA [NM_001032731]	-1.2019173	0.015601476
A_23_P69637	*lnc-NDUFS8-1*	LNCipedia lincRNA (lnc-NDUFS8-1), lincRNA [lnc-NDUFS8-1:13]	-1.1487803	0.011889729
A_23_P103720	*OTUD4*	Homo sapiens OTU deubiquitinase 4 (OTUD4), transcript variant 2, mRNA [NM_017493]	-0.41140985	0.014821658
A_33_P3415445	*CPOX*	Homo sapiens coproporphyrinogen oxidase (CPOX), mRNA [NM_000097]	-0.6733248	0.0071110567
A_33_P3399172	*AGMAT*	Homo sapiens agmatine ureohydrolase (agmatinase) (AGMAT), mRNA [NM_024758]	-1.0814346	0.0072215945
A_33_P3214849	*MAP3K2*	Homo sapiens mitogen-activated protein kinase kinase kinase 2 (MAP3K2), mRNA [NM_006609]	-0.89400715	0.0066818837
A_23_P5586	*KDELC2*	Homo sapiens KDEL (Lys-Asp-Glu-Leu) containing 2 (KDELC2), mRNA [NM_153705]	-0.92708373	0.003542592

A_21_P0014288	*lnc-SMARCAL1-3*	LNCipedia lincRNA (lnc-SMARCAL1-3), lincRNA [lnc-SMARCAL1-3:2]	-1.5255679	0.0068711066
A_23_P44781	*MPHOSPH10*	Homo sapiens M-phase phosphoprotein 10 (U3 small nucleolar ribonucleoprotein) (MPHOSPH10), mRNA [NM_005791]	-0.300108	0.014663486
A_23_P123330	*ANKRD10-IT1*	Homo sapiens cDNA FLJ42239 fis, clone TKIDN2002329. [AK124233]	0.5670786	0.01658009
A_33_P3396492	*CDKAL1*	Homo sapiens CDK5 regulatory subunit associated protein 1-like 1 (CDKAL1), mRNA [NM_017774]	-0.96243674	0.00856985
A_23_P304991	*RPL30*	Homo sapiens ribosomal protein L30 (RPL30), mRNA [NM_000989]	-0.43304133	0.005787274
A_23_P168551	*CDH24*	Homo sapiens cadherin 24, type 2 (CDH24), transcript variant 1, mRNA [NM_022478]	1.1999419	0.00856985
A_21_P0012244	*HLCS*	Homo sapiens holocarboxylase synthetase (biotin-(proprionyl-CoA-carboxylase (ATP-hydrolysing)) ligase) (HLCS), transcript variant 1, mRNA [NM_000411]	-0.8882305	0.009938401
A_21_P0014188	*SLC29A4*	Homo sapiens solute carrier family 29 (equilibrative nucleoside transporter), member 4 (SLC29A4), transcript variant 1, mRNA [NM_001040661]	0.9302271	0.008427824
A_23_P202837	*XLOC_l2_009285*	BROAD Institute lincRNA (XLOC_l2_009285), lincRNA [TCONS_l2_00017563]	-1.6449525	0.011371365
A_33_P3258265	*LOC401317*	Homo sapiens cDNA clone IMAGE:30398108. [BC087859]	1.028421	0.00968922
A_22_P00012941	*CCND1*	Homo sapiens cyclin D1 (CCND1), mRNA [NM_053056]	1.6626948	0.004026994
A_33_P3245278	*SEMA6C*	Homo sapiens sema domain, transmembrane domain (TM), and cytoplasmic domain, (semaphorin) 6C (SEMA6C), transcript variant 2, mRNA [NM_030913]	0.47787768	0.011334134
A_23_P28169	*PTPRG*	Homo sapiens protein tyrosine phosphatase, receptor type, G (PTPRG), mRNA [NM_002841]	-1.1330494	0.0088250805
A_23_P119095	*C2orf69*	Homo sapiens chromosome 2 open reading frame 69 (C2orf69), mRNA [NM_153689]	-0.67573696	0.015893938
A_23_P129246	*ARL6IP6*	Homo sapiens ADP-ribosylation factor-like 6 interacting protein 6 (ARL6IP6), transcript variant 1, mRNA [NM_152522]	-0.58406687	0.0056601833
A_24_P416177	*PPP1R13L*	Homo sapiens protein phosphatase 1, regulatory subunit 13 like (PPP1R13L), transcript variant 2, mRNA [NM_006663]	-0.89587873	0.016739154
A_33_P3374833	*PLEKHO2*	Homo sapiens pleckstrin homology domain containing, family O member 2 (PLEKHO2), transcript variant 1, mRNA [NM_025201]	-1.1939993	0.010052036
A_33_P3431595	*ADCY7*	Homo sapiens adenylate cyclase 7 (ADCY7), transcript variant 1, mRNA [NM_001114]	-0.63035536	0.0038456172

A_23_P117797	*BLOC1S6*	Homo sapiens biogenesis of lysosomal organelles complex-1, subunit 6, pallidin (BLOC1S6), mRNA [NM_012388]	-0.9389057	0.0051985686
A_23_P138271	*C8orf31*	Homo sapiens chromosome 8 open reading frame 31 (C8orf31), transcript variant 1, mRNA [NM_173687]	-1.4153684	0.0041847117
A_33_P3391496	*CLN6*	Homo sapiens ceroid-lipofuscinosis, neuronal 6, late infantile, variant (CLN6), mRNA [NM_017882]	0.9534049	0.011959745
A_24_P258235	*ARL8A*	Homo sapiens ADP-ribosylation factor-like 8A (ARL8A), transcript variant 1, mRNA [NM_138795]	-1.0481604	0.0073276176
A_33_P3372979	*OR5L2*	Homo sapiens olfactory receptor, family 5, subfamily L, member 2 (OR5L2), mRNA [NM_001004739]	1.1557738	0.0086922655
A_24_P296070	*RAD50*	Homo sapiens RAD50 homolog (S. cerevisiae) (RAD50), mRNA [NM_005732]	-0.47729653	0.012706064
A_23_P135966	*LINC00294*	Homo sapiens long intergenic non-protein coding RNA 294 (LINC00294), long non-coding RNA [NR_015451]	-0.7153294	0.011485715
A_33_P3309665	*COG3*	Homo sapiens component of oligomeric golgi complex 3 (COG3), mRNA [NM_031431]	-0.83103466	0.0037017688
A_33_P3410700	*SLC25A14*	Homo sapiens solute carrier family 25 (mitochondrial carrier, brain), member 14 (SLC25A14), transcript variant 3, mRNA [NM_001282197]	-0.75793004	0.014564713
A_23_P53588	*ZFP3*	Homo sapiens ZFP3 zinc finger protein (ZFP3), mRNA [NM_153018]	-1.3099754	0.007501246
A_23_P24004	*ATP8*	mitochondrially encoded ATP synthase 8 [Source:HGNC Symbol;Acc:HGNC:7415] [ENST00000361851]	1.3587439	0.0067678746
A_23_P366394	*WNT5B*	Homo sapiens wingless-type MMTV integration site family, member 5B (WNT5B), transcript variant 2, mRNA [NM_030775]	0.6532538	0.014241321
A_33_P6805542	*IFIT2*	Homo sapiens interferon-induced protein with tetratricopeptide repeats 2 (IFIT2), mRNA [NM_001547]	-2.2142868	0.005057235
A_24_P93703	*ZAK*	Homo sapiens sterile alpha motif and leucine zipper containing kinase AZK (ZAK), transcript variant 1, mRNA [NM_016653]	-0.73657876	0.0068711066
A_24_P142118	*lnc-SERPINC1-1*	LNCipedia lincRNA (lnc-SERPINC1-1), lincRNA [lnc-SERPINC1-1:9]	-0.45318118	0.014538049
A_24_P354689	*TMEM198B*	Homo sapiens transmembrane protein 198B, pseudogene (TMEM198B), transcript variant 1, non-coding RNA [NR_036476]	-0.9015421	0.014977407
A_33_P3419806	*THBS1*	Homo sapiens thrombospondin 1 (THBS1), mRNA [NM_003246]	-0.7459605	0.01073543

A_23_P501805	*SPOCK1*	Homo sapiens sparc/osteonectin, cwcv and kazal-like domains proteoglycan (testican) 1 (SPOCK1), mRNA [NM_004598]	-0.69650704	0.009765222
A_23_P306867	*RPS29*	Homo sapiens ribosomal protein S29 (RPS29), transcript variant 1, mRNA [NM_001032]	-0.47868964	0.006161988
A_21_P0009233	*LIPT1*	Homo sapiens lipoyltransferase 1 (LIPT1), transcript variant 3, mRNA [NM_145197]	-0.87988114	0.016394967
A_24_P563545	*NR4A3*	Homo sapiens nuclear receptor subfamily 4, group A, member 3 (NR4A3), transcript variant 4, mRNA [NM_173199]	1.3181381	0.0039856653
A_21_P0000389	*lnc-PIPOX-1*	LNCipedia lincRNA (lnc-PIPOX-1), lincRNA [lnc-PIPOX-1:1]	-0.13192226	0.010822477
A_23_P40782	*SLC30A7*	Homo sapiens solute carrier family 30 (zinc transporter), member 7 (SLC30A7), transcript variant 1, mRNA [NM_133496]	-0.91660684	0.006219354
A_33_P3278043	*SNORD91B*	Homo sapiens small nucleolar RNA, C/D box 91B (SNORD91B), small nucleolar RNA [NR_003073]	0.49273875	0.013736626
A_22_P00000575	*COMMD2*	Homo sapiens COMM domain containing 2 (COMMD2), mRNA [NM_016094]	-1.2339817	0.00609916
A_23_P206510	*TRMT10A*	Homo sapiens tRNA methyltransferase 10 homolog A (S. cerevisiae) (TRMT10A), transcript variant 1, mRNA [NM_152292]	-0.60007703	0.0066818837
A_22_P00007594	*ORAI3*	Homo sapiens ORAI calcium release-activated calcium modulator 3 (ORAI3), mRNA [NM_152288]	-0.78662926	0.013556385
A_23_P39718	*GLG1*	Homo sapiens golgi glycoprotein 1 (GLG1), transcript variant 1, mRNA [NM_012201]	-0.7265165	0.0077546863
A_23_P75800	*lnc-HDDC3-1*	Q6AGI4_LEIXX (Q6AGI4) Competence protein F, partial (6%) [THC2626027]	0.9315181	0.015497578
A_21_P0010575	*FEZ2*	Homo sapiens fasciculation and elongation protein zeta 2 (zygin II) (FEZ2), transcript variant 2, mRNA [NM_001042548]	-1.2395356	0.0046008946
A_22_P00001171	*RAB3IL1*	Homo sapiens RAB3A interacting protein (rabin3)-like 1 (RAB3IL1), transcript variant 1, mRNA [NM_013401]	-1.3662062	0.013522089
A_21_P0004421	*lnc-ANAPC11-2*	LNCipedia lincRNA (lnc-ANAPC11-2), lincRNA [lnc-ANAPC11-2:1]	-0.27454835	0.011870703
A_24_P157087	*RBMS1*	Homo sapiens RNA binding motif, single stranded interacting protein 1 (RBMS1), transcript variant 1, mRNA [NM_016836]	-0.60896325	0.016888179
A_24_P272290	*LUCAT1*	Homo sapiens lung cancer associated transcript 1 (non-protein coding) (LUCAT1), transcript variant 1, long non-coding RNA [NR_103548]	1.1752898	0.014136575
A_23_P12329	*CASP8*	Homo sapiens caspase 8, apoptosis-related cysteine peptidase (CASP8), transcript variant B, mRNA [NM_033355]	-0.9520895	0.005512448

A_23_P209962	*PXDC1*	Homo sapiens PX domain containing 1 (PXDC1), mRNA [NM_183373]	-0.991985	0.013596905
A_23_P7253	*APH1A*	Homo sapiens APH1A gamma secretase subunit (APH1A), transcript variant 2, mRNA [NM_016022]	-0.6937553	0.011760672
A_23_P14886	*SMC6*	Homo sapiens structural maintenance of chromosomes 6 (SMC6), transcript variant 2, mRNA [NM_024624]	-0.6987517	0.0036050293
A_23_P122304	*LARP1B*	Homo sapiens La ribonucleoprotein domain family, member 1B (LARP1B), transcript variant 1, mRNA [NM_018078]	-0.3648328	0.015262972
A_33_P3294790	*GNPTG*	Homo sapiens N-acetylglucosamine-1-phosphate transferase, gamma subunit (GNPTG), mRNA [NM_032520]	-0.69777083	0.0153139625
A_33_P3321801	*HDAC2*	Homo sapiens histone deacetylase 2 (HDAC2), transcript variant 1, mRNA [NM_001527]	-0.50253636	0.010801365
A_22_P00017203	*LOC100272216*	Homo sapiens uncharacterized LOC100272216 (LOC100272216), long non-coding RNA [NR_027439]	1.3408192	0.007479662
A_33_P3396591	*lnc-UCK1-1*	LNCipedia lincRNA (lnc-UCK1-1), lincRNA [lnc-UCK1-1:1]	1.137984	0.005958252
A_24_P161355	*FAM92A1*	Homo sapiens family with sequence similarity 92, member A1 (FAM92A1), transcript variant 1, mRNA [NM_145269]	-0.6013293	0.006545542
A_24_P115621	*PCED1A*	Homo sapiens PC-esterase domain containing 1A (PCED1A), transcript variant 2, mRNA [NM_001271168]	0.7949921	0.0067244163
A_33_P3242109	*EIF4EBP2*	Homo sapiens eukaryotic translation initiation factor 4E binding protein 2 (EIF4EBP2), mRNA [NM_004096]	-0.7584116	0.0075397296
A_33_P3423300	*SLMO2*	Homo sapiens slowmo homolog 2 (Drosophila) (SLMO2), transcript variant 1, mRNA [NM_016045]	-0.5800753	0.00910296
(+)E1A_r60_a20	*lnc-NINJ2-2*	Homo sapiens cDNA clone IMAGE:4752264, partial cds. [BC039168]	-0.56604564	0.007286966
A_24_P87490	*TNPO2*	Homo sapiens transportin 2 (TNPO2), transcript variant 3, mRNA [NM_001136195]	-0.57228446	0.006726202
A_23_P137209	*TVP23C*	Homo sapiens trans-golgi network vesicle protein 23 homolog C (S. cerevisiae) (TVP23C), transcript variant 1, mRNA [NM_145301]	-0.9032676	0.006360746
A_33_P3300232	*UBA1*	Homo sapiens ubiquitin-like modifier activating enzyme 1 (UBA1), transcript variant 1, mRNA [NM_003334]	1.0393144	0.005470758
A_23_P157361	*CCZ1*	Homo sapiens CCZ1 vacuolar protein trafficking and biogenesis associated homolog (S. cerevisiae) (CCZ1), mRNA [NM_015622]	-0.8288146	0.0037856582

A_24_P257359	*HYDIN*	Homo sapiens HYDIN, axonemal central pair apparatus protein (HYDIN), transcript variant 1, mRNA [NM_001270974]	1.4069242	0.005057235
A_23_P397543	*WDR60*	Homo sapiens WD repeat domain 60 (WDR60), mRNA [NM_018051]	-1.2114794	0.012168611
A_24_P940006	*RPL15*	Homo sapiens ribosomal protein L15 (RPL15), transcript variant 1, mRNA [NM_002948]	-0.4217401	0.0061486606
A_22_P00011411	*LINC00174*	Homo sapiens long intergenic non-protein coding RNA 174 (LINC00174), long non-coding RNA [NR_026873]	-1.2950447	0.014864522
A_23_P2801	*EFNB3*	Homo sapiens ephrin-B3 (EFNB3), mRNA [NM_001406]	-1.7655227	0.005852893
A_33_P3300893	*USP27X-AS1*	Homo sapiens USP27X antisense RNA 1 (head to head) (USP27X-AS1), long non-coding RNA [NR_026742]	-1.1405681	0.004155979
A_24_P374516	*ELF1*	Homo sapiens E74-like factor 1 (ets domain transcription factor) (ELF1), transcript variant 1, mRNA [NM_172373]	-1.0032902	0.0044319443
A_33_P3344276	*MADD*	Homo sapiens MAP-kinase activating death domain (MADD), transcript variant 7, mRNA [NM_130475]	0.5668737	0.016746266
A_23_P144458	*TMSB4X*	Homo sapiens thymosin beta 4, X-linked (TMSB4X), mRNA [NM_021109]	-0.801022	0.0075709717
A_33_P3351944	*HS1BP3*	Homo sapiens HCLS1 binding protein 3 (HS1BP3), mRNA [NM_022460]	1.310069	0.0070515345
A_23_P19657	*CAMK2D*	Homo sapiens calcium/calmodulin-dependent protein kinase II delta (CAMK2D), transcript variant 3, mRNA [NM_001221]	-0.9815061	0.0076856846
A_33_P3234317	*EGFR*	Homo sapiens epidermal growth factor receptor (EGFR), transcript variant 3, mRNA [NM_201283]	-0.8276099	0.0073988605
A_33_P3221971	*LRP11*	Homo sapiens low density lipoprotein receptor-related protein 11 (LRP11), mRNA [NM_032832]	-0.5487859	0.010447919
A_33_P3271755	*RRAS2*	Homo sapiens related RAS viral (r-ras) oncogene homolog 2 (RRAS2), transcript variant 1, mRNA [NM_012250]	-1.1835055	0.003956951
A_33_P3292844	*GDPGP1*	Homo sapiens GDP-D-glucose phosphorylase 1 (GDPGP1), mRNA [NM_001013657]	-0.6486402	0.015801186
A_24_P48057	*SIRT5*	Homo sapiens sirtuin 5 (SIRT5), transcript variant 2, mRNA [NM_031244]	-0.8648001	0.008153665
A_33_P3227556	*NEUROG3*	Homo sapiens neurogenin 3 (NEUROG3), mRNA [NM_020999]	0.8601265	0.011225326
A_24_P205120	*IRX5*	Homo sapiens iroquois homeobox 5 (IRX5), transcript variant 1, mRNA [NM_005853]	-1.1372447	0.01316657
A_19_P00803472	*SPG7*	Homo sapiens spastic paraplegia 7 (pure and complicated autosomal recessive) (SPG7), transcript variant 2, mRNA [NM_199367]	1.5347997	0.00527838

A_24_P702813	*TPD52L1*	Homo sapiens tumor protein D52-like 1 (TPD52L1), transcript variant 2, mRNA [NM_001003395]	-2.2390277	0.0029355595
A_23_P127891	*lnc-GDF10-2*	LNCipedia lincRNA (lnc-GDF10-2), lincRNA [lnc-GDF10-2:1]	0.93981975	0.007415985
A_22_P00002044	*XPR1*	Homo sapiens xenotropic and polytropic retrovirus receptor 1 (XPR1), transcript variant 1, mRNA [NM_004736]	-1.2048836	0.006632856
A_24_P346886	*BDNF*	Homo sapiens brain-derived neurotrophic factor (BDNF), transcript variant 1, mRNA [NM_170735]	0.74834937	0.011646681
A_23_P3312	*SCAMP1*	Homo sapiens secretory carrier membrane protein 1 (SCAMP1), transcript variant 1, mRNA [NM_004866]	-0.9265578	0.006517467
A_23_P71328	*NDUFB8*	Homo sapiens NADH dehydrogenase (ubiquinone) 1 beta subcomplex, 8, 19kDa (NDUFB8), transcript variant 1, mRNA [NM_005004]	-0.9608803	0.01013214
A_23_P635	*ISLR*	Homo sapiens immunoglobulin superfamily containing leucine-rich repeat (ISLR), transcript variant 1, mRNA [NM_005545]	-1.1606485	0.00942879
A_23_P250564	*MATN2*	Homo sapiens matrilin 2 (MATN2), transcript variant 2, mRNA [NM_030583]	-2.7474995	0.004359316
A_24_P245108	*PMF1*	Homo sapiens polyamine-modulated factor 1 (PMF1), transcript variant 2, mRNA [NM_007221]	0.59457237	0.012509614
A_22_P00000187	*PRKCE*	Homo sapiens protein kinase C, epsilon (PRKCE), mRNA [NM_005400]	-1.1609659	0.0035476608
A_23_P37910	*USP7*	Homo sapiens ubiquitin specific peptidase 7 (herpes virus-associated) (USP7), transcript variant 3, mRNA [NM_001286458]	-0.26532903	0.014022445
A_23_P156683	*MIR133A1HG*	Homo sapiens MIR133A1 host gene (non-protein coding) (MIR133A1HG), long non-coding RNA [NR_110369]	-0.16427736	0.009943344
A_23_P144202	*MAPK3*	Homo sapiens mitogen-activated protein kinase 3 (MAPK3), transcript variant 1, mRNA [NM_002746]	0.86154103	0.009938745
A_23_P502170	*LTA*	Homo sapiens lymphotoxin alpha (LTA), transcript variant 2, mRNA [NM_000595]	0.26578012	0.016718669
A_23_P65712	*EEFSEC*	Homo sapiens eukaryotic elongation factor, selenocysteine-tRNA-specific (EEFSEC), mRNA [NM_021937]	0.9318556	0.014218263
A_24_P326491	*DYNC2LI1*	Homo sapiens dynein, cytoplasmic 2, light intermediate chain 1 (DYNC2LI1), transcript variant 2, mRNA [NM_015522]	-1.1500261	0.007831759
A_23_P371682	*FAM103A1*	Homo sapiens family with sequence similarity 103, member A1 (FAM103A1), mRNA [NM_031452]	-0.7929705	0.005428736

A_23_P320159	*MKX*	Homo sapiens mohawk homeobox (MKX), transcript variant 1, mRNA [NM_173576]	2.0733275	0.0031512622
A_23_P65174	*GPC6*	Homo sapiens glypican 6 (GPC6), mRNA [NM_005708]	-3.249513	0.010769289
A_23_P395075	*TCEANC*	Homo sapiens transcription elongation factor A (SII) N-terminal and central domain containing (TCEANC), transcript variant 2, mRNA [NM_001297563]	-1.2317595	0.0047990954
A_33_P3314176	*PHF11*	Homo sapiens PHD finger protein 11 (PHF11), transcript variant 1, mRNA [NM_001040443]	-0.9474871	0.010301823
A_23_P166491	*KDM3A*	Homo sapiens lysine (K)-specific demethylase 3A (KDM3A), transcript variant 1, mRNA [NM_018433]	-0.9079938	0.009794768
A_19_P00805291	*FAM46C*	Homo sapiens family with sequence similarity 46, member C (FAM46C), mRNA [NM_017709]	3.1108227	0.0028703364
A_33_P3339361	*SGSM3*	Homo sapiens small G protein signaling modulator 3 (SGSM3), transcript variant 1, mRNA [NM_015705]	0.9689121	0.013481679
A_32_P355396	*ARHGAP11A*	Homo sapiens Rho GTPase activating protein 11A (ARHGAP11A), transcript variant 2, mRNA [NM_199357]	-0.5423016	0.011893636
A_33_P3433156	*FABP5*	Homo sapiens fatty acid binding protein 5 (psoriasis-associated) (FABP5), mRNA [NM_001444]	-0.31532454	0.0039322884
A_23_P133133	*TECPR2*	Homo sapiens tectonin beta-propeller repeat containing 2 (TECPR2), transcript variant 1, mRNA [NM_014844]	-0.5274189	0.007488306
A_22_P00018366	*TRMU*	Homo sapiens tRNA 5-methylaminomethyl-2-thiouridylate methyltransferase (TRMU), transcript variant 7, mRNA [NM_001282785]	0.7752924	0.00978609
A_21_P0000661	*ALPK1*	Homo sapiens alpha-kinase 1 (ALPK1), transcript variant 1, mRNA [NM_025144]	-0.82054543	0.007889792
A_33_P3335596	*lnc-ZWINT-5*	C04987 Human heart cDNA (YNakamura) Homo sapiens cDNA clone 3NHC4411, mRNA sequence [C04987]	1.2783598	0.0034534817
A_24_P324814	*SNHG4*	Homo sapiens small nucleolar RNA host gene 4 (non-protein coding) (SNHG4), transcript variant 2, long non-coding RNA [NR_036536]	1.1690099	0.007993578
A_23_P55421	*C17orf62*	Homo sapiens chromosome 17 open reading frame 62 (C17orf62), transcript variant 4, mRNA [NM_001193653]	1.3330779	0.007474992
A_23_P48307	*CEP95*	Homo sapiens centrosomal protein 95kDa (CEP95), mRNA [NM_138363]	-0.6670106	0.007406785
A_23_P121064	*CBX8*	Homo sapiens chromobox homolog 8 (CBX8), mRNA [NM_020649]	1.1304797	0.006736978
A_32_P186474	*PABPC3*	Homo sapiens poly(A) binding protein, cytoplasmic 3 (PABPC3), mRNA [NM_030979]	-0.70687413	0.01120723

A_21_P0003091	*PTX3*	Homo sapiens pentraxin 3, long (PTX3), mRNA [NM_002852]	-3.6653047	0.0024890031
A_23_P110882	*RACGAP1*	Homo sapiens Rac GTPase activating protein 1 (RACGAP1), transcript variant 1, mRNA [NM_013277]	-0.8085263	0.012525387
A_32_P506600	*lnc-AC069257.9.1-5*	LNCipedia lincRNA (lnc-AC069257.9.1-5), lincRNA [lnc-AC069257.9.1-5:4]	-1.835394	0.0059353677
A_33_P3349552	*TSPYL4*	Homo sapiens TSPY-like 4 (TSPYL4), mRNA [NM_021648]	-1.1577442	0.010237655
A_33_P3306146	*RAN*	Homo sapiens RAN, member RAS oncogene family (RAN), transcript variant 1, mRNA [NM_006325]	-0.2620154	0.007571981
A_21_P0000679	*CASKIN1*	Homo sapiens CASK interacting protein 1 (CASKIN1), mRNA [NM_020764]	0.6646013	0.011745691
A_19_P00316340	*PLAU*	Homo sapiens plasminogen activator, urokinase (PLAU), transcript variant 2, mRNA [NM_001145031]	4.7402935	0.0033199077
A_33_P3252134	*LINC01578*	Homo sapiens long intergenic non-protein coding RNA 1578 (LINC01578), transcript variant 1, long non-coding RNA [NR_037601]	-0.6659964	0.0071447864
A_33_P3337540	*LOC100506860*	Homo sapiens uncharacterized LOC100506860 (LOC100506860), long non-coding RNA [NR_109780]	-0.5193327	0.017157277
A_33_P3339103	*IFNAR2*	Homo sapiens interferon (alpha, beta and omega) receptor 2 (IFNAR2), transcript variant 2, mRNA [NM_000874]	-0.44088894	0.007115449
A_23_P11353	*MTERF3*	Homo sapiens mitochondrial transcription termination factor 3 (MTERF3), transcript variant 1, mRNA [NM_015942]	-0.62874866	0.008331241
A_33_P3263193	*POLR1C*	Homo sapiens polymerase (RNA) I polypeptide C, 30kDa (POLR1C), mRNA [NM_203290]	-0.29254225	0.016038679
A_23_P119418	*ATP6AP2*	Homo sapiens ATPase, H+ transporting, lysosomal accessory protein 2 (ATP6AP2), mRNA [NM_005765]	-1.0648805	0.0046717683
A_33_P3257155	*RHNO1*	Homo sapiens RAD9-HUS1-RAD1 interacting nuclear orphan 1 (RHNO1), transcript variant 4, mRNA [NM_001257097]	-0.48263073	0.0044600246
A_24_P48723	*URI1*	Homo sapiens URI1, prefoldin-like chaperone (URI1), transcript variant 1, mRNA [NM_003796]	-0.7933998	0.004508921
A_21_P0000480	*SMAP1*	small ArfGAP 1 [Source:HGNC Symbol;Acc:HGNC:19651] [ENST00000439432]	-0.42093012	0.011358038
A_23_P258246	*PTGIS*	Homo sapiens prostaglandin I2 (prostacyclin) synthase (PTGIS), mRNA [NM_000961]	-3.6697054	0.0029471128
A_32_P208120	*DDB1*	Homo sapiens damage-specific DNA binding protein 1, 127kDa (DDB1), mRNA [NM_001923]	-0.7055223	0.009574714

A_33_P3861706	*UBP1*	Homo sapiens upstream binding protein 1 (LBP-1a) (UBP1), transcript variant 1, mRNA [NM_014517]	-0.32130262	0.0093693705
A_22_P00011910	*CAMK1D*	calcium/calmodulin-dependent protein kinase ID [Source:HGNC Symbol;Acc:HGNC:19341] [ENST00000615792]	1.9154586	0.0062702326
A_23_P155868	*MTMR10*	Homo sapiens myotubularin related protein 10 (MTMR10), mRNA [NM_017762]	-1.1535833	0.0059353677
A_23_P205228	*IDH1-AS1*	Homo sapiens IDH1 antisense RNA 1 (IDH1-AS1), long non-coding RNA [NR_046452]	-0.91677475	0.002419416
A_22_P00010013	*PGRMC2*	Homo sapiens progesterone receptor membrane component 2 (PGRMC2), mRNA [NM_006320]	-1.1287682	0.0039659217
A_21_P0014476	*ATP7B*	Homo sapiens ATPase, Cu++ transporting, beta polypeptide (ATP7B), transcript variant 1, mRNA [NM_000053]	-1.4829817	0.0065835547
A_33_P3235410	*lnc-MME-4*	BX094640 Soares_NFL_T_GBC_S1 Homo sapiens cDNA clone IMAGp998I184013, mRNA sequence [BX094640]	0.64247173	0.005403412
A_23_P15174	*PTPLA*	Homo sapiens protein tyrosine phosphatase-like (proline instead of catalytic arginine), member A, mRNA (cDNA clone IMAGE:4304680), with apparent retained intron. [BC027709]	-1.2290459	0.006573993
A_21_P0013343	*APBA1*	Homo sapiens amyloid beta (A4) precursor protein-binding, family A, member 1 (APBA1), mRNA [NM_001163]	1.3081928	0.0072763516
A_22_P00019325	*MT1F*	Homo sapiens metallothionein 1F (MT1F), transcript variant 1, mRNA [NM_005949]	-0.7787107	0.009630487
A_23_P141624	*XLOC_l2_013931*	BROAD Institute lincRNA (XLOC_l2_013931), lincRNA [TCONS_l2_00026784]	-1.6619648	0.0040699136
A_23_P13701	*lnc-MAP6-1*	Q6NSJ6_HUMAN (Q6NSJ6) Serine (Or cysteine) proteinase inhibitor, clade H, member 1,, partial (16%) [THC2603244]	0.61374295	0.01704795
A_21_P0011081	*KRTAP1-1*	Homo sapiens keratin associated protein 1-1 (KRTAP1-1), mRNA [NM_030967]	-2.7302246	0.006485141
A_22_P00003188	*TMBIM4*	Homo sapiens transmembrane BAX inhibitor motif containing 4 (TMBIM4), transcript variant 2, mRNA [NM_016056]	-0.85585755	0.006395745
A_23_P66017	*FAM86C1*	Homo sapiens family with sequence similarity 86, member C1 (FAM86C1), transcript variant 3, mRNA [NM_001099653]	-1.354569	0.0071605598
A_21_P0011401	*lnc-C9orf95-1*	LNCipedia lincRNA (lnc-C9orf95-1), lincRNA [lnc-C9orf95-1:1]	0.73527104	0.016782014
A_23_P4909	*PRRT2*	Homo sapiens proline-rich transmembrane protein 2 (PRRT2), transcript variant 1, mRNA [NM_145239]	0.6086009	0.007049496

A_21_P0013007	*LOC100996255*	Homo sapiens uncharacterized LOC100996255 (LOC100996255), long non-coding RNA [NR_102756]	-0.6822681	0.013936194
A_21_P0010267	*SNRNP70*	Homo sapiens small nuclear ribonucleoprotein 70kDa (U1) (SNRNP70), transcript variant 1, mRNA [NM_003089]	1.0520948	0.008106991
A_23_P50217	*lnc-TPTE-3*	LNCipedia lincRNA (lnc-TPTE-3), lincRNA [lnc-TPTE-3:3]	4.4634438	0.004359316
A_33_P3279441	*lnc-SHPRH-2*	LNCipedia lincRNA (lnc-SHPRH-2), lincRNA [lnc-SHPRH-2:7]	-0.4193511	0.013353969
A_21_P0009752	*ZNF671*	Homo sapiens zinc finger protein 671 (ZNF671), mRNA [NM_024833]	-1.2824506	0.007206317
A_22_P00000282	*GOSR2*	Homo sapiens golgi SNAP receptor complex member 2 (GOSR2), transcript variant C, mRNA [NM_001012511]	-1.2781522	0.005852893
A_32_P217510	*lnc-UQCRFS1-9*	LNCipedia lincRNA (lnc-UQCRFS1-9), lincRNA [lnc-UQCRFS1-9:21]	-0.9136075	0.007897626
A_33_P3628675	*MIR940*	microRNA 940 [Source:HGNC Symbol;Acc:HGNC:33683] [ENST00000567888]	1.301189	0.0051410845
A_23_P209430	*WDR75*	Homo sapiens WD repeat domain 75 (WDR75), transcript variant 1, mRNA [NM_032168]	-0.5368097	0.008346524
A_23_P19673	*FAM86B1*	Homo sapiens family with sequence similarity 86, member B1 (FAM86B1), transcript variant 1, mRNA [NM_001083537]	0.57587653	0.010791786
A_33_P3352873	*ALS2*	Homo sapiens amyotrophic lateral sclerosis 2 (juvenile) (ALS2), transcript variant 1, mRNA [NM_020919]	-0.7696877	0.009202373
A_24_P902728	*SGK1*	Homo sapiens serum/glucocorticoid regulated kinase 1 (SGK1), transcript variant 1, mRNA [NM_005627]	-1.2059472	0.007946583
A_23_P37359	*BCKDHB*	Homo sapiens branched chain keto acid dehydrogenase E1, beta polypeptide (BCKDHB), transcript variant 2, mRNA [NM_000056]	-1.3023942	0.0152601935
A_33_P3368500	*CAPRIN1*	Homo sapiens cell cycle associated protein 1 (CAPRIN1), transcript variant 2, mRNA [NM_203364]	-0.9680891	0.0039753253
A_23_P203957	*CIDEB*	Homo sapiens cell death-inducing DFFA-like effector b (CIDEB), mRNA [NM_014430]	1.5947372	0.007963782
A_23_P22926	*IFT52*	intraflagellar transport 52 [Source:HGNC Symbol;Acc:HGNC:15901] [ENST00000471199]	-1.0285592	0.009733482
A_23_P139509	*TMTC1*	Homo sapiens transmembrane and tetratricopeptide repeat containing 1 (TMTC1), transcript variant 2, mRNA [NM_175861]	0.49287942	0.016435314
A_33_P3280575	*GNB1*	Homo sapiens guanine nucleotide binding protein (G protein), beta polypeptide 1 (GNB1), transcript variant 1, mRNA [NM_002074]	-0.6252511	0.005014313

A_33_P3327818	*ERGIC2*	Homo sapiens ERGIC and golgi 2 (ERGIC2), mRNA [NM_016570]	-0.6752369	0.012416257
A_24_P643587	*AKAP7*	Homo sapiens A kinase (PRKA) anchor protein 7 (AKAP7), transcript variant gamma, mRNA [NM_016377]	-0.8997512	0.013050359
A_23_P395555	*LAMA4*	Homo sapiens laminin, alpha 4 (LAMA4), transcript variant 5, mRNA [NM_001105209]	-1.4727234	0.0032831472
A_33_P3361746	*ASB8*	Homo sapiens ankyrin repeat and SOCS box containing 8 (ASB8), mRNA [NM_024095]	-0.99574196	0.012965191
A_33_P3363188	*ZNF226*	Homo sapiens zinc finger protein 226 (ZNF226), transcript variant 1, mRNA [NM_001032372]	-0.9263844	0.0068644313
A_23_P395524	*TRPS1*	Homo sapiens trichorhinophalangeal syndrome I (TRPS1), transcript variant 1, mRNA [NM_014112]	-0.96936077	0.0077180467
A_24_P555170	*FLJ43315*	Homo sapiens asparagine synthetase pseudogene (FLJ43315), non-coding RNA [NR_033856]	-0.6328649	0.0093693705
A_23_P334990	*PWP1*	Homo sapiens PWP1 homolog (S. cerevisiae) (PWP1), mRNA [NM_007062]	-0.8785596	0.0052438728
A_22_P00015971	*UBE2Q2L*	Homo sapiens ubiquitin-conjugating enzyme E2Q family member 2-like (UBE2Q2L), mRNA [NM_001243531]	-0.93587506	0.016929097
A_23_P17287	*SUDS3*	Homo sapiens suppressor of defective silencing 3 homolog (S. cerevisiae) (SUDS3), mRNA [NM_022491]	-0.41662553	0.0037353234
A_33_P3471712	*lnc-TEKT5-1*	LNCipedia lincRNA (lnc-TEKT5-1), lincRNA [lnc-TEKT5-1:1]	-0.90711004	0.005348777
A_23_P122531	*IAH1*	Homo sapiens isoamyl acetate-hydrolyzing esterase 1 homolog (S. cerevisiae) (IAH1), mRNA [NM_001039613]	-0.9813704	0.008415294
A_33_P3221408	*C6orf48*	Homo sapiens chromosome 6 open reading frame 48 (C6orf48), transcript variant 1, mRNA [NM_001040437]	-1.0879705	0.005948276
A_32_P194962	*NTNG1*	Homo sapiens netrin G1 (NTNG1), transcript variant 1, mRNA [NM_001113226]	-1.9355271	0.0037856582
A_33_P3335740	*LOC284454*	Homo sapiens uncharacterized LOC284454 (LOC284454), long non-coding RNA [NR_036515]	3.066616	0.0041847117
A_23_P18325	*FLJ10038*	Homo sapiens uncharacterized protein FLJ10038 (FLJ10038), long non-coding RNA [NR_026891]	0.738253	0.011225326
A_33_P3231120	*PAMR1*	Homo sapiens peptidase domain containing associated with muscle regeneration 1 (PAMR1), transcript variant 1, mRNA [NM_015430]	1.2845614	0.011030013

A_23_P36825	*PDCD10*	Homo sapiens programmed cell death 10 (PDCD10), transcript variant 1, mRNA [NM_007217]	-0.82309246	0.0074381046
A_24_P303080	*CASD1*	Homo sapiens CAS1 domain containing 1 (CASD1), mRNA [NM_022900]	-0.62373865	0.008538553
A_33_P3344308	*GPRC5A*	Homo sapiens G protein-coupled receptor, class C, group 5, member A (GPRC5A), mRNA [NM_003979]	2.2729764	0.003365343
A_24_P242036	*MFSD8*	Homo sapiens major facilitator superfamily domain containing 8 (MFSD8), mRNA [NM_152778]	-1.080552	0.0058573913
A_23_P90357	*APTX*	Homo sapiens aprataxin (APTX), transcript variant 7, mRNA [NM_001195249]	-0.8316801	0.012911957
A_33_P3254756	*RRP7B*	Homo sapiens ribosomal RNA processing 7 homolog B (S. cerevisiae) (RRP7B), non-coding RNA [NR_002184]	0.7380028	0.0117289275
A_24_P602507	*TBXA2R*	Homo sapiens thromboxane A2 receptor (TBXA2R), transcript variant a, mRNA [NM_001060]	-0.75896955	0.015648095
A_33_P3303519	*UBA6-AS1*	Homo sapiens UBA6 antisense RNA 1 (head to head) (UBA6-AS1), long non-coding RNA [NR_015439]	-0.99324083	0.010837003
A_24_P173234	*HYKK*	Homo sapiens hydroxylysine kinase (HYKK), transcript variant 1, mRNA [NM_001013619]	-1.6528485	0.0052543716
A_24_P941359	*CLEC12B*	Homo sapiens C-type lectin domain family 12, member B (CLEC12B), transcript variant 2, mRNA [NM_205852]	0.79836893	0.014847365
A_33_P3322283	*ZNF613*	Homo sapiens zinc finger protein 613 (ZNF613), transcript variant 2, mRNA [NM_024840]	-1.1281394	0.011328784
A_23_P88069	*FAM65B*	Homo sapiens family with sequence similarity 65, member B (FAM65B), transcript variant 1, mRNA [NM_014722]	-3.5831242	0.0023340243
A_23_P14284	*AZI2*	Homo sapiens 5-azacytidine induced 2 (AZI2), transcript variant 4, mRNA [NM_001271650]	-0.9656114	0.0072350414
A_23_P207675	*LHFP*	Homo sapiens lipoma HMGIC fusion partner (LHFP), mRNA [NM_005780]	-0.88188624	0.008819748
A_21_P0014446	*VTI1B*	Homo sapiens vesicle transport through interaction with t-SNAREs 1B (VTI1B), mRNA [NM_006370]	-0.83298373	0.00456068
A_21_P0014015	*PLCD3*	Homo sapiens phospholipase C, delta 3 (PLCD3), mRNA [NM_133373]	2.9162512	0.0028193828
A_24_P194714	*HHIP*	hedgehog interacting protein [Source:HGNC Symbol;Acc:HGNC:14866] [ENST00000296575]	-1.8606005	0.013800824
A_33_P3369461	*CNP*	2',3'-cyclic nucleotide 3' phosphodiesterase [Source:HGNC Symbol;Acc:HGNC:2158] [ENST00000592861]	0.47293755	0.014535962

A_33_P3231447	*UBALD2*	Homo sapiens UBA-like domain containing 2 (UBALD2), mRNA [NM_182565]	-0.82567143	0.008537068
A_23_P104734	*AMIGO1*	Homo sapiens adhesion molecule with Ig-like domain 1 (AMIGO1), mRNA [NM_020703]	-2.1257894	0.009778202
A_19_P00319413	*ITGA6*	Homo sapiens integrin, alpha 6 (ITGA6), transcript variant 2, mRNA [NM_000210]	0.83954567	0.005920916
A_23_P151436	*STT3A*	Homo sapiens STT3A, subunit of the oligosaccharyltransferase complex (catalytic) (STT3A), transcript variant 2, mRNA [NM_152713]	-0.8765479	0.010663839
A_23_P103282	*ALG5*	Homo sapiens ALG5, dolichyl-phosphate beta-glucosyltransferase (ALG5), transcript variant 1, mRNA [NM_013338]	-0.62452674	0.0059821224
A_33_P3311170	*CRTC1*	Homo sapiens CREB regulated transcription coactivator 1 (CRTC1), transcript variant 3, mRNA [NM_001098482]	0.58582467	0.016859638
A_33_P3361513	*TMEM59*	Homo sapiens transmembrane protein 59 (TMEM59), mRNA [NM_004872]	-1.2613795	0.005348777
A_33_P3233273	*SLC7A5P1*	Homo sapiens solute carrier family 7 (amino acid transporter light chain, L system), member 5 pseudogene 1 (SLC7A5P1), non-coding RNA [NR_002593]	0.86585546	0.016164096
A_23_P39910	*NLE1*	Homo sapiens notchless homolog 1 (Drosophila) (NLE1), transcript variant 2, mRNA [NM_001014445]	1.1819465	0.010769289
A_24_P268196	*LRRC61*	Homo sapiens leucine rich repeat containing 61 (LRRC61), transcript variant 1, mRNA [NM_001142928]	2.3248625	0.013487576
A_23_P53126	*COA5*	Homo sapiens cytochrome c oxidase assembly factor 5 (COA5), mRNA [NM_001008215]	-1.4739658	0.0033868244
A_23_P20894	*LZIC*	leucine zipper and CTNNBIP1 domain containing [Source:HGNC Symbol;Acc:HGNC:17497] [ENST00000377223]	-0.71930546	0.014323062
A_33_P3323384	*LMO2*	Homo sapiens LIM domain only 2 (rhombotin-like 1) (LMO2), transcript variant 1, mRNA [NM_005574]	-1.0752326	0.011851147
A_23_P162589	*EHMT1*	Homo sapiens euchromatic histone-lysine N-methyltransferase 1 (EHMT1), transcript variant 1, mRNA [NM_024757]	0.97697294	0.015419179
A_23_P352799	*RRM2B*	Homo sapiens ribonucleotide reductase M2 B (TP53 inducible) (RRM2B), transcript variant 1, mRNA [NM_015713]	-0.7556054	0.009811661
A_23_P414978	*VDR*	Homo sapiens vitamin D (1,25-dihydroxyvitamin D3) receptor (VDR), transcript variant 2, mRNA [NM_001017535]	-1.4379749	0.004016376
A_23_P27285	*NPW*	Homo sapiens neuropeptide W (NPW), mRNA [NM_001099456]	1.4483969	0.014720503

A_33_P3279059	*NUDT14*	Homo sapiens nudix (nucleoside diphosphate linked moiety X)-type motif 14 (NUDT14), mRNA [NM_177533]	-1.6366436	0.009938745
A_33_P3423440	*MPPE1*	Homo sapiens metallophosphoesterase 1 (MPPE1), transcript variant 1, mRNA [NM_023075]	-0.53811264	0.016574716
A_33_P3429576	*RIPK1*	Homo sapiens receptor (TNFRSF)-interacting serine-threonine kinase 1 (RIPK1), mRNA [NM_003804]	-0.9974544	0.0041847117
A_33_P3216955	*ZNF720*	Homo sapiens zinc finger protein 720 (ZNF720), mRNA [NM_001130913]	-0.90724725	0.0052543716
A_33_P3213772	*AP3S1*	Homo sapiens adaptor-related protein complex 3, sigma 1 subunit (AP3S1), mRNA [NM_001284]	-0.7396076	0.0038292732
A_24_P943393	*ANAPC16*	Homo sapiens anaphase promoting complex subunit 16 (ANAPC16), transcript variant 1, mRNA [NM_001242546]	-0.94156015	0.00795954
A_33_P3842886	*SRGAP2*	Homo sapiens SLIT-ROBO Rho GTPase activating protein 2 (SRGAP2), transcript variant 4, mRNA [NM_001300952]	-1.5536709	0.0054714447
A_23_P349928	*AHNAK*	Homo sapiens AHNAK nucleoprotein (AHNAK), transcript variant 1, mRNA [NM_001620]	0.563765	0.013805346
A_23_P207842	*POLM*	Homo sapiens polymerase (DNA directed), mu (POLM), transcript variant 1, mRNA [NM_013284]	-0.94733405	0.015639378
A_21_P0004484	*SP100*	Homo sapiens SP100 nuclear antigen (SP100), transcript variant 2, mRNA [NM_003113]	-0.20108892	0.013595847
A_33_P3322328	*RARA*	Homo sapiens retinoic acid receptor, alpha (RARA), transcript variant 2, mRNA [NM_001024809]	0.73540014	0.006360746
A_23_P20225	*LINC01018*	Homo sapiens long intergenic non-protein coding RNA 1018 (LINC01018), transcript variant 1, long non-coding RNA [NR_024424]	0.519395	0.01065365
A_33_P3411632	*EPS15*	Homo sapiens epidermal growth factor receptor pathway substrate 15 (EPS15), transcript variant 1, mRNA [NM_001981]	-0.94166607	0.0039633843
A_23_P33022	*RRM2B*	Homo sapiens ribonucleotide reductase M2 B (TP53 inducible) (RRM2B), transcript variant 1, mRNA [NM_015713]	-1.4676684	0.0022181747
A_23_P10374	*TMEM121*	Homo sapiens transmembrane protein 121 (TMEM121), mRNA [NM_025268]	-0.75068164	0.016262818
A_23_P63067	*POLR2L*	Homo sapiens polymerase (RNA) II (DNA directed) polypeptide L, 7.6kDa (POLR2L), mRNA [NM_021128]	-0.17052934	0.005403412
A_23_P161156	*R3HDM2*	Homo sapiens R3H domain containing 2 (R3HDM2), mRNA [NM_014925]	-0.9317298	0.006229295

A_33_P3409996	*DAP3*	Homo sapiens death associated protein 3 (DAP3), transcript variant 1, mRNA [NM_033657]	-0.7528162	0.006587778
A_23_P83939	*ZNF438*	Homo sapiens zinc finger protein 438 (ZNF438), transcript variant 2, mRNA [NM_182755]	-1.0803971	0.006169501
A_33_P3322859	*AP5Z1*	Homo sapiens adaptor-related protein complex 5, zeta 1 subunit (AP5Z1), mRNA [NM_014855]	1.1101471	0.0054418826
A_21_P0012872	*SYAP1*	Homo sapiens synapse associated protein 1 (SYAP1), transcript variant 1, mRNA [NM_032796]	-0.32461527	0.010036303
A_23_P160862	*HES6*	Homo sapiens hes family bHLH transcription factor 6 (HES6), transcript variant 1, mRNA [NM_018645]	0.8470365	0.0031941293
A_22_P00005311	*XLOC_l2_011873*	BROAD Institute lincRNA (XLOC_l2_011873), lincRNA [TCONS_l2_00022667]	-1.2110801	0.005175472
A_23_P250813	*HNRNPU*	Homo sapiens heterogeneous nuclear ribonucleoprotein U (scaffold attachment factor A) (HNRNPU), transcript variant 1, mRNA [NM_031844]	-0.29160184	0.015347851
A_24_P97836	*lnc-DNAJC7-1*	LNCipedia lincRNA (lnc-DNAJC7-1), lincRNA [lnc-DNAJC7-1:1]	-0.4671061	0.0133306645
A_23_P78289	*WRN*	Homo sapiens Werner syndrome, RecQ helicase-like (WRN), mRNA [NM_000553]	-0.9538644	0.008095165
A_24_P605190	*RAD17*	Homo sapiens RAD17 homolog (S. pombe) (RAD17), transcript variant 8, mRNA [NM_002873]	-0.6100074	0.01531403
A_33_P3375934	*FAM104A*	Homo sapiens family with sequence similarity 104, member A (FAM104A), transcript variant 2, mRNA [NM_032837]	-0.83343077	0.013549144
A_32_P162150	*PGGT1B*	Homo sapiens protein geranylgeranyltransferase type I, beta subunit (PGGT1B), mRNA [NM_005023]	-0.71125126	0.010042705
A_24_P22488	*NAMPT*	Homo sapiens nicotinamide phosphoribosyltransferase (NAMPT), mRNA [NM_005746]	1.281537	0.007571981
A_23_P67042	*TAB3*	Homo sapiens TGF-beta activated kinase 1/MAP3K7 binding protein 3 (TAB3), mRNA [NM_152787]	-0.75286466	0.011367354
A_33_P3354539	*AXIN1*	Homo sapiens axin 1 (AXIN1), transcript variant 1, mRNA [NM_003502]	0.5628185	0.015810816
A_23_P164313	*MOCOS*	Homo sapiens molybdenum cofactor sulfurase (MOCOS), mRNA [NM_017947]	-1.6262081	0.0040942165
A_33_P3304247	*CHURC1*	Homo sapiens churchill domain containing 1 (CHURC1), transcript variant 1, mRNA [NM_145165]	-0.7905743	0.006173301

A_23_P154235	*MINK1*	Homo sapiens misshapen-like kinase 1 (MINK1), transcript variant 3, mRNA [NM_153827]	1.6735877	0.004206936
A_23_P205007	*NMI*	Homo sapiens N-myc (and STAT) interactor (NMI), mRNA [NM_004688]	-1.1889529	0.0067145773
A_23_P76015	*CGREF1*	Homo sapiens cell growth regulator with EF-hand domain 1 (CGREF1), transcript variant 1, mRNA [NM_006569]	-1.3040543	0.007072526
A_23_P58251	*IPO5*	Homo sapiens importin 5 (IPO5), mRNA [NM_002271]	-1.2169642	0.003365343
A_23_P201628	*ARHGEF17*	Homo sapiens Rho guanine nucleotide exchange factor (GEF) 17 (ARHGEF17), mRNA [NM_014786]	-1.0346124	0.0067377784
A_23_P111981	*CPZ*	Homo sapiens carboxypeptidase Z (CPZ), transcript variant 3, mRNA [NM_001014448]	1.4522523	0.009975308
A_33_P3292840	*LAMC1*	Homo sapiens laminin, gamma 1 (formerly LAMB2) (LAMC1), mRNA [NM_002293]	-1.5416849	0.005057235
A_24_P234415	*LYNX1*	Ly6/neurotoxin 1 [Source:HGNC Symbol;Acc:HGNC:29604] [ENST00000614491]	-1.2338604	0.012368504
A_23_P6869	*C11orf49*	Homo sapiens chromosome 11 open reading frame 49 (C11orf49), transcript variant 4, mRNA [NM_001003678]	-0.67485493	0.002535607
A_24_P406301	*STAC*	Homo sapiens SH3 and cysteine rich domain (STAC), transcript variant 1, mRNA [NM_003149]	-4.1165457	0.0022181747
A_33_P3252326	*TMEM115*	Homo sapiens transmembrane protein 115 (TMEM115), mRNA [NM_007024]	1.0273732	0.011883512
A_23_P14157	*NDUFB2*	Homo sapiens NADH dehydrogenase (ubiquinone) 1 beta subcomplex, 2, 8kDa (NDUFB2), mRNA [NM_004546]	-0.6759155	0.0024890031
A_23_P51085	*DZIP1*	Homo sapiens DAZ interacting zinc finger protein 1 (DZIP1), transcript variant 2, mRNA [NM_198968]	-0.568517	0.0075709717
A_24_P61753	*SEMA4F*	Homo sapiens sema domain, immunoglobulin domain (Ig), transmembrane domain (TM) and short cytoplasmic domain, (semaphorin) 4F (SEMA4F), transcript variant 1, mRNA [NM_004263]	-0.6604481	0.013124348
A_33_P3280237	*SPC25*	Homo sapiens SPC25, NDC80 kinetochore complex component (SPC25), mRNA [NM_020675]	-0.68430996	0.011678774
A_33_P3245126	*CLUH*	Homo sapiens clustered mitochondria (cluA/CLU1) homolog (CLUH), mRNA [NM_015229]	1.0839151	0.007733715
A_33_P3210139	*RAB11B-AS1*	Homo sapiens RAB11B antisense RNA 1 (RAB11B-AS1), long non-coding RNA [NR_038237]	-1.1579753	0.003609745

A_32_P407245	*ALDH3A2*	Homo sapiens aldehyde dehydrogenase 3 family, member A2 (ALDH3A2), transcript variant 2, mRNA [NM_000382]	-1.5131686	0.0071553676
A_24_P357266	*PCF11*	Homo sapiens PCF11 cleavage and polyadenylation factor subunit (PCF11), mRNA [NM_015885]	-0.959161	0.0052883234
A_21_P0000130	*DNAJC22*	Homo sapiens DnaJ (Hsp40) homolog, subfamily C, member 22 (DNAJC22), mRNA [NM_024902]	0.97868836	0.01285011
A_24_P71373	*GRPR*	Homo sapiens gastrin-releasing peptide receptor (GRPR), mRNA [NM_005314]	2.3289104	0.0029691157
A_24_P229871	*GS1-259H13.2*	Homo sapiens transmembrane protein 225-like (GS1-259H13.2), transcript variant 1, mRNA [NM_001195541]	-1.1523602	0.0049917083
A_23_P120062	*SLC9A1*	Homo sapiens solute carrier family 9, subfamily A (NHE1, cation proton antiporter 1), member 1 (SLC9A1), transcript variant 1, mRNA [NM_003047]	0.9518933	0.011828812
A_33_P3364964	*LINC00469*	long intergenic non-protein coding RNA 469 [Source:HGNC Symbol;Acc:HGNC:26863] [ENST00000321800]	1.1782379	0.0066128806
A_23_P156017	*GCFC2*	Homo sapiens GC-rich sequence DNA-binding factor 2 (GCFC2), transcript variant 1, mRNA [NM_003203]	-0.7471769	0.0059218835
A_23_P404606	*LOC100129596*	Homo sapiens cDNA FLJ27403 fis, clone WMC03327. [AK130913]	0.9791455	0.004437345
A_22_P00022810	*GOLPH3*	Homo sapiens golgi phosphoprotein 3 (coat-protein) (GOLPH3), mRNA [NM_022130]	-0.7790223	0.007510704
A_21_P0012393	*CREBRF*	Homo sapiens CREB3 regulatory factor (CREBRF), transcript variant 1, mRNA [NM_153607]	-1.0738983	0.0098649
A_23_P2203	*POFUT1*	Homo sapiens protein O-fucosyltransferase 1 (POFUT1), transcript variant 1, mRNA [NM_015352]	-0.7067246	0.013220456
A_21_P0007591	*XLOC_l2_009883*	BROAD Institute lincRNA (XLOC_l2_009883), lincRNA [TCONS_l2_00018854]	-1.98019	0.0035939496
A_24_P166663	*TAOK3*	Homo sapiens TAO kinase 3 (TAOK3), mRNA [NM_016281]	-0.9466088	0.010154543
A_33_P3313258	*CDK6*	Homo sapiens cyclin-dependent kinase 6 (CDK6), transcript variant 1, mRNA [NM_001259]	-0.9898059	0.002804883
A_21_P0000600	*LOC344887*	Homo sapiens NmrA-like family domain containing 1 pseudogene (LOC344887), non-coding RNA [NR_033752]	0.3809712	0.016607525
A_33_P3265920	*ATAD3B*	Homo sapiens ATPase family, AAA domain containing 3B (ATAD3B), mRNA [NM_031921]	1.4185491	0.008471989

A_33_P3230290	*IFT27*	Homo sapiens intraflagellar transport 27 (IFT27), transcript variant 4, non-coding RNA [NR_033531]	-1.4563863	0.0025001846
A_23_P24433	*PTPN4*	Homo sapiens protein tyrosine phosphatase, non-receptor type 4 (megakaryocyte) (PTPN4), mRNA [NM_002830]	-0.5955023	0.0072215945
A_33_P3222635	*ACAP3*	Homo sapiens ArfGAP with coiled-coil, ankyrin repeat and PH domains 3 (ACAP3), mRNA [NM_030649]	0.7575898	0.006285482
A_22_P00025748	*CTSF*	Homo sapiens cathepsin F (CTSF), mRNA [NM_003793]	-1.267473	0.008245779
A_33_P3839897	*XGY2*	Homo sapiens Xg pseudogene, Y-linked 2 (XGY2), non-coding RNA [NR_003254]	0.86400443	0.006719993
A_23_P103661	*lnc-HSPA4-4*	LNCipedia lincRNA (lnc-HSPA4-4), lincRNA [lnc-HSPA4-4:1]	-0.5725315	0.014817799
A_24_P316939	*RNU4ATAC*	HHAGE001732 Human liver regeneration after partial hepatectomy Homo sapiens cDNA, mRNA sequence [DW419002]	1.036424	0.013467389
A_21_P0008662	*YY1AP1*	Homo sapiens YY1 associated protein 1 (YY1AP1), transcript variant 2, mRNA [NM_139118]	-0.9652436	0.006773419
A_33_P3231076	*LRRFIP1*	Homo sapiens leucine rich repeat (in FLII) interacting protein 1 (LRRFIP1), transcript variant 1, mRNA [NM_001137550]	-1.3097808	0.003606253
A_22_P00013581	*lnc-IL16-2*	LNCipedia lincRNA (lnc-IL16-2), lincRNA [lnc-IL16-2:1]	-1.0322509	0.014502948
A_21_P0008231	*PAXIP1-AS2*	Homo sapiens PAXIP1 antisense RNA 2 (PAXIP1-AS2), transcript variant 2, long non-coding RNA [NR_024477]	-1.0693269	0.013335797
A_23_P63153	*FRMD6-AS2*	Homo sapiens FRMD6 antisense RNA 2 (FRMD6-AS2), transcript variant 2, long non-coding RNA [NR_051990]	-0.5781902	0.016159432
A_24_P255473	*lnc-TNFRSF19-5*	LNCipedia lincRNA (lnc-TNFRSF19-5), lincRNA [lnc-TNFRSF19-5:1]	0.24106711	0.00854209
A_33_P3225843	*DDX20*	Homo sapiens DEAD (Asp-Glu-Ala-Asp) box polypeptide 20 (DDX20), mRNA [NM_007204]	-0.77545357	0.014145028
A_23_P28652	*CCDC149*	Homo sapiens coiled-coil domain containing 149 (CCDC149), transcript variant 1, mRNA [NM_173463]	-1.8299198	0.0039659217
A_23_P257198	*FKBP1A*	Homo sapiens FK506 binding protein 1A, 12kDa (FKBP1A), transcript variant 2, mRNA [NM_054014]	0.9494047	0.007392104
A_22_P00007987	*ATRAID*	Homo sapiens all-trans retinoic acid-induced differentiation factor (ATRAID), transcript variant 1, mRNA [NM_016085]	-0.8809785	0.00477571
A_24_P365349	*NDUFS4*	Homo sapiens NADH dehydrogenase (ubiquinone) Fe-S protein 4, 18kDa (NADH-	-0.8011377	0.008159287

		coenzyme Q reductase) (NDUFS4), mRNA [NM_002495]		
A_33_P3378514	*LOC101928973*	Homo sapiens uncharacterized LOC101928973 (LOC101928973), long non-coding RNA [NR_125966]	0.7549374	0.011087365
A_24_P28977	*CACNG7*	Homo sapiens calcium channel, voltage-dependent, gamma subunit 7 (CACNG7), mRNA [NM_031896]	2.3123195	0.0026049376
A_23_P390384	*PDE5A*	Homo sapiens phosphodiesterase 5A, cGMP-specific (PDE5A), transcript variant 1, mRNA [NM_001083]	0.9537236	0.00789784
A_23_P134714	*TRPC1*	Homo sapiens transient receptor potential cation channel, subfamily C, member 1 (TRPC1), transcript variant 2, mRNA [NM_003304]	-0.8391456	0.011136462
A_33_P3356502	*ZNF630*	Homo sapiens zinc finger protein 630 (ZNF630), transcript variant 1, mRNA [NM_001037735]	-1.5788772	0.00422039
A_24_P46953	*HRSP12*	Homo sapiens heat-responsive protein 12 (HRSP12), mRNA [NM_005836]	-0.90736985	0.0060306
A_32_P170481	*RALB*	Homo sapiens v-ral simian leukemia viral oncogene homolog B (RALB), mRNA [NM_002881]	-1.3301309	0.0039830348
A_33_P3221253	*SGK3*	Homo sapiens serum/glucocorticoid regulated kinase family, member 3 (SGK3), transcript variant 1, mRNA [NM_013257]	-1.310866	0.0042918096
A_23_P134925	*LOC100240735*	Homo sapiens uncharacterized LOC100240735 (LOC100240735), long non-coding RNA [NR_026658]	-1.6385403	0.005348777
A_23_P131899	*LPP*	Homo sapiens LIM domain containing preferred translocation partner in lipoma (LPP), transcript variant 1, mRNA [NM_005578]	-0.9835007	0.003363001
A_23_P316612	*BNIP3L*	Homo sapiens BCL2/adenovirus E1B 19kDa interacting protein 3-like (BNIP3L), mRNA [NM_004331]	-0.8667095	0.002804883
A_21_P0001470	*SDCBP2*	Homo sapiens syndecan binding protein (syntenin) 2 (SDCBP2), transcript variant 1, mRNA [NM_080489]	0.9811852	0.012381021
A_23_P419947	*GLIS1*	Homo sapiens GLIS family zinc finger 1 (GLIS1), mRNA [NM_147193]	-2.5398474	0.0037583082
A_22_P00018620	*lnc-C1orf195-2*	LNCipedia lincRNA (lnc-C1orf195-2), lincRNA [lnc-C1orf195-2:1]	0.766976	0.010874895
A_23_P19182	*MLF1*	Homo sapiens myeloid leukemia factor 1 (MLF1), transcript variant 1, mRNA [NM_022443]	-1.6545436	0.002804883
A_23_P400235	*REEP2*	Homo sapiens receptor accessory protein 2 (REEP2), transcript variant 2, mRNA [NM_016606]	-1.118391	0.0073276176

A_33_P3395219	*TIAM2*	T-cell lymphoma invasion and metastasis 2 [Source:HGNC Symbol;Acc:HGNC:11806] [ENST00000449545]	-1.1458006	0.0072907214
A_33_P3379962	*MUT*	Homo sapiens methylmalonyl CoA mutase (MUT), mRNA [NM_000255]	-0.85186434	0.009367357
A_23_P71419	*NEXN-AS1*	Homo sapiens NEXN antisense RNA 1 (NEXN-AS1), long non-coding RNA [NR_103535]	1.2444957	0.010583646
A_32_P98927	*HLA-A*	Homo sapiens major histocompatibility complex, class I, A (HLA-A), transcript variant 1 (A*03:01:0:01 allele), mRNA [NM_002116]	-0.20718998	0.010237655
A_21_P0014502	*COPS5*	Homo sapiens COP9 signalosome subunit 5 (COPS5), mRNA [NM_006837]	-0.85288167	0.01214474
A_23_P71513	*STAG3L4*	Homo sapiens stromal antigen 3-like 4 (pseudogene) (STAG3L4), transcript variant 1, non-coding RNA [NR_040585]	-0.6937696	0.009629584
A_24_P285480	*EFR3A*	Homo sapiens EFR3 homolog A (S. cerevisiae) (EFR3A), mRNA [NM_015137]	-0.7851477	0.0035935708
A_33_P3403867	*HOTAIRM1*	Homo sapiens HOXA transcript antisense RNA, myeloid-specific 1 (HOTAIRM1), transcript variant 1, long non-coding RNA [NR_038366]	-1.0524472	0.009690877
A_23_P200792	*MRPL33*	Homo sapiens mitochondrial ribosomal protein L33 (MRPL33), transcript variant 1, mRNA [NM_004891]	-0.5044877	0.0037685214
A_24_P176493	*PMEPA1*	Homo sapiens prostate transmembrane protein, androgen induced 1 (PMEPA1), transcript variant 1, mRNA [NM_020182]	-0.7629959	0.015771633
A_33_P3258346	*NOTCH2*	Homo sapiens notch 2 (NOTCH2), transcript variant 1, mRNA [NM_024408]	-0.9143111	0.0073130694
A_23_P431569	*ATMIN*	Homo sapiens ATM interactor (ATMIN), transcript variant 1, mRNA [NM_015251]	-0.9993873	0.00800842
A_24_P166311	*XAF1*	Homo sapiens XIAP associated factor 1 (XAF1), transcript variant 1, mRNA [NM_017523]	-0.88820034	0.009506635
A_22_P00009871	*LOC100049716*	Homo sapiens uncharacterized LOC100049716 (LOC100049716), long non-coding RNA [NR_122124]	-1.3493303	0.006110945
A_33_P3422802	*ZBED8*	Homo sapiens zinc finger, BED-type containing 8 (ZBED8), transcript variant 2, mRNA [NM_022090]	-1.1079088	0.011747599
A_24_P6030	*lnc-MFAP4-1*	LNCipedia lincRNA (lnc-MFAP4-1), lincRNA [lnc-MFAP4-1:1]	0.89062506	0.005559843
A_23_P48217	*ULBP1*	Homo sapiens UL16 binding protein 1 (ULBP1), mRNA [NM_025218]	-0.93083763	0.014260212
A_23_P28105	*APOLD1*	Homo sapiens apolipoprotein L domain containing 1 (APOLD1), transcript variant 2, mRNA [NM_030817]	-1.95885	0.006573993
A_33_P3347869	*lnc-CELA3A-2*	Homo sapiens full length insert cDNA clone ZD58F06. [AF086337]	0.536568	0.009582734

A_23_P161399	*TSN*	Homo sapiens translin (TSN), transcript variant 1, mRNA [NM_004622]	-0.8428383	0.01082951
A_23_P105900	*C3*	Homo sapiens complement component 3 (C3), mRNA [NM_000064]	5.475241	0.0074153394
A_23_P50919	*MXI1*	Homo sapiens MAX interactor 1, dimerization protein (MXI1), transcript variant 2, mRNA [NM_130439]	-1.639381	0.0057647754
A_33_P3307875	*BTF3P11*	Homo sapiens basic transcription factor 3 pseudogene 11 (BTF3P11), non-coding RNA [NR_026983]	-0.49910975	0.0064668017
A_23_P139864	*SERPINE2*	Homo sapiens serpin peptidase inhibitor, clade E (nexin, plasminogen activator inhibitor type 1), member 2 (SERPINE2), transcript variant 1, mRNA [NM_006216]	-0.7057216	0.0027248112
A_23_P314760	*SPTSSA*	Homo sapiens serine palmitoyltransferase, small subunit A (SPTSSA), mRNA [NM_138288]	-0.8720126	0.005348777
A_24_P193600	*GSG1*	Homo sapiens germ cell associated 1 (GSG1), transcript variant 1, mRNA [NM_031289]	-0.69858176	0.009296599
A_21_P0005309	*PRKAG2*	Homo sapiens protein kinase, AMP-activated, gamma 2 non-catalytic subunit (PRKAG2), transcript variant a, mRNA [NM_016203]	-1.714822	0.0054643718
A_24_P350008	*ZNF174*	Homo sapiens zinc finger protein 174 (ZNF174), transcript variant 1, mRNA [NM_003450]	0.6639893	0.005985026
A_24_P368544	*ZNF850*	Homo sapiens zinc finger protein 850 (ZNF850), transcript variant 1, mRNA [NM_001193552]	0.9228581	0.010617712
A_24_P373174	*ZNF333*	Homo sapiens zinc finger protein 333 (ZNF333), transcript variant 1, mRNA [NM_032433]	-0.8020443	0.010698572
A_23_P62081	*SLC25A26*	Homo sapiens solute carrier family 25 (S-adenosylmethionine carrier), member 26 (SLC25A26), transcript variant 1, mRNA [NM_173471]	-0.66309655	0.009630487
A_24_P213643	*RAB27A*	Homo sapiens RAB27A, member RAS oncogene family (RAB27A), transcript variant 1, mRNA [NM_004580]	-0.560796	0.007870519
A_21_P0000090	*SCG5*	Homo sapiens secretogranin V (7B2 protein) (SCG5), transcript variant 2, mRNA [NM_003020]	-1.8476439	0.0024890031
A_21_P0000189	*TSPAN10*	Homo sapiens tetraspanin 10 (TSPAN10), transcript variant 2, mRNA [NM_031945]	0.9532317	0.006262615
A_24_P188878	*TSFM*	Homo sapiens Ts translation elongation factor, mitochondrial (TSFM), transcript variant 4, mRNA [NM_001172697]	-0.58035654	0.014904488
A_33_P3287892	*DR1*	Homo sapiens down-regulator of transcription 1, TBP-binding (negative cofactor 2) (DR1), mRNA [NM_001938]	-0.78545713	0.008943179

A_21_P0013528	*RPL34*	Homo sapiens ribosomal protein L34 (RPL34), transcript variant 2, mRNA [NM_033625]	-0.6276274	0.002804883
A_33_P3212782	*SLTM*	SAFB-like, transcription modulator [Source:HGNC Symbol;Acc:HGNC:20709] [ENST00000480144]	-1.3795046	0.0075689107
A_33_P3419339	*CALM2*	Homo sapiens calmodulin 2 (phosphorylase kinase, delta) (CALM2), mRNA [NM_001743]	-0.5924592	0.0032554762
A_24_P257971	*CERS6*	Homo sapiens ceramide synthase 6 (CERS6), transcript variant 1, mRNA [NM_001256126]	-2676149	0.0038422274
A_24_P30557	*GNAI3*	Homo sapiens guanine nucleotide binding protein (G protein), alpha inhibiting activity polypeptide 3 (GNAI3), mRNA [NM_006496]	-0.91237545	0.005701378
A_23_P102770	*SLC27A4*	Homo sapiens solute carrier family 27 (fatty acid transporter), member 4 (SLC27A4), mRNA [NM_005094]	1.0657042	0.010688684
A_24_P102880	*TBX5*	Homo sapiens T-box 5 (TBX5), transcript variant 1, mRNA [NM_000192]	-1.1074227	0.0049163844
A_33_P3330811	*NAA20*	Homo sapiens N(alpha)-acetyltransferase 20, NatB catalytic subunit (NAA20), transcript variant 1, mRNA [NM_016100]	-0.6513178	0.00856985
A_33_P3319815	*NAV1*	Homo sapiens neuron navigator 1 (NAV1), transcript variant 1, mRNA [NM_020443]	-0.7316948	0.016642986
A_23_P129058	*C8orf59*	Homo sapiens chromosome 8 open reading frame 59 (C8orf59), transcript variant 1, mRNA [NM_001099670]	-0.37090594	0.009737427
A_21_P0013932	*LINC01546*	Homo sapiens long intergenic non-protein coding RNA 1546 (LINC01546), long non-coding RNA [NR_038428]	-1.4045839	0.015418683
A_33_P3227467	*USP8*	Homo sapiens ubiquitin specific peptidase 8 (USP8), transcript variant 1, mRNA [NM_005154]	-0.7524969	0.010931504
A_23_P101281	*BNIP2*	Homo sapiens BCL2/adenovirus E1B 19kDa interacting protein 2 (BNIP2), mRNA [NM_004330]	-0.6354943	0.0048261923
A_21_P0011781	*UBE2J2*	Homo sapiens ubiquitin-conjugating enzyme E2, J2 (UBE2J2), transcript variant 3, mRNA [NM_194458]	0.81714505	0.0098649
A_19_P00804020	*ZNF587*	Homo sapiens zinc finger protein 587 (ZNF587), transcript variant 1, mRNA [NM_032828]	-0.7100281	0.009965863
A_33_P3250018	*PSG8*	Homo sapiens pregnancy specific beta-1-glycoprotein 8 (PSG8), transcript variant 2, mRNA [NM_001130167]	1.7595842	0.01065365
A_33_P3331856	*TLE1*	Homo sapiens transducin-like enhancer of split 1 (E(sp1) homolog, Drosophila) (TLE1), transcript variant 3, mRNA [NM_001303104]	-0.8718124	0.004218752
A_24_P943613	*HCFC2*	Homo sapiens host cell factor C2 (HCFC2), mRNA [NM_013320]	-1.0811467	0.010816889

A_33_P3327847	*PDE1C*	phosphodiesterase 1C, calmodulin-dependent 70kDa [Source:HGNC Symbol;Acc:HGNC:8776] [ENST00000396184]	-6.164423	0.015750945
A_24_P238333	*TBC1D1*	Homo sapiens TBC1 (tre-2/USP6, BUB2, cdc16) domain family, member 1 (TBC1D1), transcript variant 1, mRNA [NM_015173]	-0.996927	0.004803986
A_19_P00315584	*SGPL1*	Homo sapiens sphingosine-1-phosphate lyase 1 (SGPL1), mRNA [NM_003901]	-0.89489174	0.0126051465
A_23_P11685	*NSA2*	Homo sapiens NSA2 ribosome biogenesis homolog (S. cerevisiae) (NSA2), transcript variant 1, mRNA [NM_014886]	-1.1200014	0.010874326
A_24_P911094	*LOC100130691*	Homo sapiens uncharacterized LOC100130691 (LOC100130691), long non-coding RNA [NR_026966]	-1.13848	0.004829709
A_23_P100711	*PLA2G4A*	Homo sapiens phospholipase A2, group IVA (cytosolic, calcium-dependent) (PLA2G4A), mRNA [NM_024420]	-2.1345081	0.0029471128
A_24_P151834	*RABL2A*	RAB, member of RAS oncogene family-like 2A [Source:HGNC Symbol;Acc:HGNC:9799] [ENST00000376439]	-0.74495125	0.007977038
A_23_P54000	*PMP22*	Homo sapiens peripheral myelin protein 22 (PMP22), transcript variant 1, mRNA [NM_000304]	-2.2958517	0.0036789463
A_23_P32414	*ANKFY1*	ankyrin repeat and FYVE domain containing 1 [Source:HGNC Symbol;Acc:HGNC:20763] [ENST00000570934]	-0.6956286	0.016195282
A_23_P258071	*SNX6*	Homo sapiens sorting nexin 6 (SNX6), transcript variant 1, mRNA [NM_021249]	-1.3760178	0.0035470822
A_21_P0011020	*STK26*	Homo sapiens serine/threonine protein kinase 26 (STK26), transcript variant 1, mRNA [NM_016542]	-3.4103074	0.0030069214
A_23_P250994	*RNF113A*	Homo sapiens ring finger protein 113A (RNF113A), mRNA [NM_006978]	-0.9668209	0.012626196
A_23_P7761	*XLOC_l2_002790*	BROAD Institute lincRNA (XLOC_l2_002790), lincRNA [TCONS_l2_00005211]	-2.13572	0.0030069214
A_33_P3340468	*ANAPC10*	Homo sapiens anaphase promoting complex subunit 10 (ANAPC10), transcript variant 2, mRNA [NM_014885]	-0.7452528	0.011581112
A_33_P3308553	*C5orf22*	Homo sapiens chromosome 5 open reading frame 22 (C5orf22), mRNA [NM_018356]	-0.6695846	0.0051130913
A_23_P61406	*CENPI*	centromere protein I [Source:HGNC Symbol;Acc:HGNC:3968] [ENST00000403304]	0.62216926	0.011870703
A_22_P00014790	*SHC3*	Homo sapiens SHC (Src homology 2 domain containing) transforming protein 3 (SHC3), mRNA [NM_016848]	3.9412584	0.0027874883
A_23_P87902	*NDUFS6*	Homo sapiens NADH dehydrogenase (ubiquinone) Fe-S protein 6, 13kDa (NADH-	-0.409339	0.0054879813

		coenzyme Q reductase) (NDUFS6), mRNA [NM_004553]		
A_33_P3341716	*DYRK4*	Homo sapiens dual-specificity tyrosine-(Y)-phosphorylation regulated kinase 4 (DYRK4), transcript variant 1, mRNA [NM_003845]	-0.53167534	0.005928778
A_21_P0011956	*FZD6*	Homo sapiens frizzled class receptor 6 (FZD6), transcript variant 1, mRNA [NM_003506]	-0.16871266	0.016947122
A_21_P0011008	*SP4*	Homo sapiens Sp4 transcription factor (SP4), mRNA [NM_003112]	-0.85994047	0.0042032786
A_32_P182941	*XLOC_l2_008221*	BROAD Institute lincRNA (XLOC_l2_008221), lincRNA [TCONS_l2_00014844]	-1.1273205	0.004359316
A_23_P46627	*XLOC_l2_002651*	BROAD Institute lincRNA (XLOC_l2_002651), lincRNA [TCONS_l2_00005005]	0.7702514	0.016152125
A_21_P0013858	*RPS3*	Homo sapiens ribosomal protein S3 (RPS3), transcript variant 1, mRNA [NM_001005]	-0.27800032	0.011969343
A_33_P3380567	*ADIPOR1*	Homo sapiens adiponectin receptor 1 (ADIPOR1), transcript variant 1, mRNA [NM_015999]	-0.54333514	0.016817955
A_23_P218158	*TMLHE*	trimethyllysine hydroxylase, epsilon [Source:HGNC Symbol;Acc:HGNC:18308] [ENST00000449645]	-1.12634	0.015249244
A_23_P135548	*SHARPIN*	Homo sapiens SHANK-associated RH domain interactor (SHARPIN), transcript variant 1, mRNA [NM_030974]	-0.40813068	0.00410343
A_23_P137057	*SMEK1*	Homo sapiens SMEK homolog 1, suppressor of mek1 (Dictyostelium) (SMEK1), transcript variant 3, mRNA [NM_001284280]	-0.6160117	0.0037685214
A_23_P201264	*DPYD*	Homo sapiens dihydropyrimidine dehydrogenase (DPYD), transcript variant 1, mRNA [NM_000110]	-1.708668	0.002804883
A_23_P64019	*SLC25A5*	Homo sapiens solute carrier family 25 (mitochondrial carrier; adenine nucleotide translocator), member 5 (SLC25A5), mRNA [NM_001152]	-0.47483367	0.011959745
A_23_P352870	*NMNAT1*	Homo sapiens nicotinamide nucleotide adenylyltransferase 1 (NMNAT1), transcript variant 1, mRNA [NM_022787]	-0.6199169	0.016498217
A_33_P3223631	*MTMR2*	Homo sapiens myotubularin related protein 2 (MTMR2), transcript variant 2, mRNA [NM_201278]	-0.827625	0.009334625
A_23_P55688	*PVRL2*	Homo sapiens poliovirus receptor-related 2 (herpesvirus entry mediator B) (PVRL2), transcript variant alpha, mRNA [NM_002856]	1.1337197	0.010066772
A_23_P32036	*ZNF416*	Homo sapiens zinc finger protein 416 (ZNF416), mRNA [NM_017879]	-0.8942128	0.014548385
A_33_P3292660	*MRPS17*	Homo sapiens mitochondrial ribosomal protein S17 (MRPS17), mRNA [NM_015969]	-0.6592157	0.0037353234

A_23_P218111	*NMRK1*	Homo sapiens nicotinamide riboside kinase 1 (NMRK1), transcript variant 1, mRNA [NM_017881]	-1.2094517	0.0070515345
A_23_P133236	*GRIA3*	Homo sapiens glutamate receptor, ionotropic, AMPA 3 (GRIA3), transcript variant 3, mRNA [NM_001256743]	-2.540306	0.0031750489
A_33_P3415087	*SERPINA1*	Homo sapiens serpin peptidase inhibitor, clade A (alpha-1 antiproteinase, antitrypsin), member 1 (SERPINA1), transcript variant 2, mRNA [NM_001002236]	-1.5099223	0.009833635
A_33_P3229022	*PCDHB14*	Homo sapiens protocadherin beta 14 (PCDHB14), mRNA [NM_018934]	1.9017756	0.00556941
A_21_P0000158	*CLCN5*	Homo sapiens chloride channel, voltage-sensitive 5 (CLCN5), transcript variant 1, mRNA [NM_001127899]	-0.7653482	0.0140423635
A_23_P409553	*XPNPEP3*	Homo sapiens X-prolyl aminopeptidase (aminopeptidase P) 3, putative (XPNPEP3), transcript variant 2, mRNA [NM_001204827]	2.1636853	0.0039416323
A_33_P3224745	*EXT2*	Homo sapiens exostosin glycosyltransferase 2 (EXT2), transcript variant 1, mRNA [NM_000401]	-1.1325727	0.00862906
A_23_P167358	*PPM1A*	Homo sapiens protein phosphatase, Mg2+/Mn2+ dependent, 1A (PPM1A), transcript variant 2, mRNA [NM_177951]	-0.6888645	0.013460425
A_33_P3358824	*PLA2G15*	Homo sapiens phospholipase A2, group XV (PLA2G15), mRNA [NM_012320]	-0.7001776	0.016992087
A_21_P0006903	*C4orf29*	Homo sapiens chromosome 4 open reading frame 29 (C4orf29), mRNA [NM_001039717]	-0.7345525	0.011680649
A_21_P0012053	*PLOD1*	Homo sapiens procollagen-lysine, 2-oxoglutarate 5-dioxygenase 1 (PLOD1), mRNA [NM_000302]	0.71773505	0.0062296083
A_23_P88484	*lnc-BTRC-2*	LNCipedia lincRNA (lnc-BTRC-2), lincRNA [lnc-BTRC-2:1]	1.3350198	0.0032554762
A_33_P3343467	*FLJ43681*	Homo sapiens ribosomal protein L23a pseudogene (FLJ43681), non-coding RNA [NR_029406]	-0.8508086	0.015772918
A_22_P00005124	*DUT*	Homo sapiens deoxyuridine triphosphatase (DUT), transcript variant 1, mRNA [NM_001025248]	-0.94385284	0.0058573913
A_23_P254594	*LINC00957*	Homo sapiens long intergenic non-protein coding RNA 957 (LINC00957), transcript variant 2, long non-coding RNA [NR_015401]	-0.39434597	0.01305673
A_21_P0013662	*NAPA-AS1*	Homo sapiens NAPA antisense RNA 1 (NAPA-AS1), long non-coding RNA [NR_038452]	0.56538695	0.004449649
A_24_P192262	*GNRH1*	Homo sapiens gonadotropin-releasing hormone 1 (luteinizing-releasing hormone) (GNRH1), transcript variant 1, mRNA [NM_000825]	-1.4072174	0.0037926938

A_33_P3227666	*HSPB1*	Homo sapiens heat shock 27kDa protein 1 (HSPB1), mRNA [NM_001540]	-0.30249026	0.0065241847
A_21_P0001010	*RALA*	Homo sapiens v-ral simian leukemia viral oncogene homolog A (ras related) (RALA), mRNA [NM_005402]	-0.488905	0.008284906
A_23_P100074	*AGGF1*	Homo sapiens angiogenic factor with G patch and FHA domains 1 (AGGF1), mRNA [NM_018046]	-0.46042588	0.01482335
A_23_P142146	*lnc-RP3-377D14.1.1-7*	LNCipedia lincRNA (lnc-RP3-377D14.1.1-7), lincRNA [lnc-RP3-377D14.1.1-7:3]	-1.7147098	0.005428736
A_33_P3406004	*AVEN*	Homo sapiens apoptosis, caspase activation inhibitor (AVEN), mRNA [NM_020371]	-0.94234633	0.010640448
A_24_P58242	*RPL18*	Homo sapiens ribosomal protein L18 (RPL18), transcript variant 1, mRNA [NM_000979]	-0.514097	0.008800535
A_24_P96762	*YBEY*	Homo sapiens ybeY metallopeptidase (putative) (YBEY), transcript variant 1, mRNA [NM_058181]	-0.70291543	0.013936194
A_33_P3350583	*TRNAU1AP*	Homo sapiens tRNA selenocysteine 1 associated protein 1 (TRNAU1AP), transcript variant 1, mRNA [NM_017846]	-1.0996916	0.0049456456
A_23_P157569	*lnc-USP10-2*	LNCipedia lincRNA (lnc-USP10-2), lincRNA [lnc-USP10-2:1]	1.3678228	0.0116016995
A_23_P41246	*FAM122C*	Homo sapiens family with sequence similarity 122C (FAM122C), transcript variant 2, mRNA [NM_138819]	-1.3338196	0.015050633
A_22_P00007897	*ADHFE1*	Homo sapiens alcohol dehydrogenase, iron containing, 1 (ADHFE1), mRNA [NM_144650]	-1.2589337	0.008976597
A_24_P127121	*MFSD10*	Homo sapiens major facilitator superfamily domain containing 10 (MFSD10), transcript variant 1, mRNA [NM_001120]	0.81031036	0.01695468
A_21_P0000514	*lnc-HSP90AA1-2*	LNCipedia lincRNA (lnc-HSP90AA1-2), lincRNA [lnc-HSP90AA1-2:1]	1.1039335	0.010500704
A_32_P62211	*VTRNA1-2*	Homo sapiens vault RNA 1-2 (VTRNA1-2), vault RNA [NR_026704]	-1.2057816	0.0034310205
A_21_P0000406	*ZCCHC7*	Homo sapiens zinc finger, CCHC domain containing 7 (ZCCHC7), transcript variant 4, mRNA [NM_032226]	-0.93219376	0.0023561441
A_33_P3377045	*LOC101928433*	PREDICTED: Homo sapiens uncharacterized LOC101928433 (LOC101928433), ncRNA [XR_241982]	-1.1221588	0.009968179
A_23_P73457	*SNORD114-9*	Homo sapiens small nucleolar RNA, C/D box 114-9 (SNORD114-9), small nucleolar RNA [NR_003201]	0.16996673	0.012620988
A_23_P7172	*CDC37L1*	cell division cycle 37-like 1 [Source:HGNC Symbol;Acc:HGNC:17179] [ENST00000381854]	-1.413628	0.0049063917
A_24_P365180	*RUFY1*	Homo sapiens RUN and FYVE domain containing 1 (RUFY1), transcript variant 1, mRNA [NM_025158]	-1.1275477	0.013734197

A_23_P128967	*PGM2*	Homo sapiens phosphoglucomutase 2 (PGM2), mRNA [NM_018290]	-0.5603493	0.008090729
A_23_P111240	*DSEL*	Homo sapiens dermatan sulfate epimerase-like (DSEL), mRNA [NM_032160]	0.68744755	0.011958462
A_32_P414791	*ALDH6A1*	Homo sapiens aldehyde dehydrogenase 6 family, member A1 (ALDH6A1), transcript variant 1, mRNA [NM_005589]	-1.2528049	0.008488166
A_23_P60283	*PHACTR2*	Homo sapiens phosphatase and actin regulator 2 (PHACTR2), transcript variant 3, mRNA [NM_014721]	-1.0169612	0.005352383
A_23_P122216	*POLN*	Homo sapiens polymerase (DNA directed) nu (POLN), mRNA [NM_181808]	-0.50830495	0.004723954
A_22_P00023177	*XPA*	Homo sapiens xeroderma pigmentosum, complementation group A (XPA), transcript variant 1, mRNA [NM_000380]	-0.8080335	0.009438499
A_23_P147439	*LOX*	Homo sapiens lysyl oxidase (LOX), transcript variant 1, mRNA [NM_002317]	-1.9216816	0.0031941293
A_24_P67308	*lnc-VTI1A-3*	Homo sapiens cDNA: FLJ21484 fis, clone COL05256. [AK025137]	-1.2676105	0.0088250805
A_33_P3381771	*ATXN2L*	Homo sapiens ataxin 2-like (ATXN2L), transcript variant F, mRNA [NM_017492]	0.9088568	0.01011346
A_21_P0004789	*RPL19P12*	Homo sapiens ribosomal protein L19 pseudogene 12 (RPL19P12), non-coding RNA [NR_026660]	-0.37325042	0.01419689
A_33_P3297141	*GID4*	Homo sapiens GID complex subunit 4 (GID4), mRNA [NM_024052]	-1.3218815	0.0038422274
A_23_P12079	*lnc-KATNA1-1*	LNCipedia lincRNA (lnc-KATNA1-1), lincRNA [lnc-KATNA1-1:1]	-1.2151792	0.006573993
A_23_P74042	*WDR48*	Homo sapiens WD repeat domain 48 (WDR48), transcript variant 1, mRNA [NM_020839]	-1.3831242	0.007317922
A_32_P163125	*KCNC4*	Homo sapiens potassium channel, voltage gated Shaw related subfamily C, member 4 (KCNC4), transcript variant 3, mRNA [NM_001039574]	0.7458464	0.012532662
A_21_P0004423	*LPHN2*	Homo sapiens latrophilin 2 (LPHN2), transcript variant 1, mRNA [NM_012302]	-1.6711829	0.0077072685
A_24_P916378	*SGMS1*	Homo sapiens sphingomyelin synthase 1 (SGMS1), mRNA [NM_147156]	-1.0938861	0.008285209
A_33_P3394710	*lnc-ARRDC3-1*	LNCipedia lincRNA (lnc-ARRDC3-1), lincRNA [lnc-ARRDC3-1:1]	0.967128	0.014864522
A_23_P55174	*HNRNPLL*	heterogeneous nuclear ribonucleoprotein L-like [Source:HGNC Symbol;Acc:HGNC:25127] [ENST00000410076]	-0.5892419	0.0062257075
A_33_P3257817	*SLC35A1*	Homo sapiens solute carrier family 35 (CMP-sialic acid transporter), member A1 (SLC35A1), transcript variant 2, mRNA [NM_001168398]	-0.7478629	0.016888179

A_33_P3577671	*G6PC3*	Homo sapiens glucose 6 phosphatase, catalytic, 3 (G6PC3), transcript variant 1, mRNA [NM_138387]	1.1909236	0.0060804365
A_33_P3398917	*LOC102723701*	Homo sapiens uncharacterized LOC102723701 (LOC102723701), long non-coding RNA [NR_125821]	0.72502375	0.0151453735
A_23_P7325	*DPP8*	Homo sapiens dipeptidyl-peptidase 8 (DPP8), transcript variant 1, mRNA [NM_130434]	-0.7243602	0.009560995
A_23_P155463	*C15orf59*	Homo sapiens chromosome 15 open reading frame 59 (C15orf59), transcript variant 1, mRNA [NM_001039614]	-1.5874887	0.002804883
A_23_P103414	*BST1*	Homo sapiens bone marrow stromal cell antigen 1 (BST1), mRNA [NM_004334]	-3.5385475	0.003104718
A_22_P00014730	*LRRC2*	Homo sapiens leucine rich repeat containing 2 (LRRC2), mRNA [NM_024512]	-1.5691276	0.00511559
A_23_P501435	*YTHDF2*	Homo sapiens YTH N(6)-methyladenosine RNA binding protein 2 (YTHDF2), transcript variant 1, mRNA [NM_016258]	-0.53428984	0.013563653
A_23_P35131	*lnc-SLC2A4RG-1*	Q9VRI3_DROME (Q9VRI3) CG10918-PA, partial (8%) [THC2780915]	0.95816034	0.014306614
A_23_P4808	*CSRP2BP*	Homo sapiens CSRP2 binding protein (CSRP2BP), transcript variant 1, mRNA [NM_020536]	-0.9615907	0.00556786
A_33_P3824237	*SF3B4*	Homo sapiens splicing factor 3b, subunit 4, 49kDa (SF3B4), mRNA [NM_005850]	1.9459507	0.003363001
A_22_P00013536	*PTGER1*	Homo sapiens prostaglandin E receptor 1 (subtype EP1), 42kDa (PTGER1), mRNA [NM_000955]	0.96562856	0.010328656
A_22_P00022177	*LINC00857*	Homo sapiens long intergenic non-protein coding RNA 857 (LINC00857), long non-coding RNA [NR_038464]	1.6246086	0.0065689106
A_23_P140146	*lnc-RP11-422N16.3.1-1*	LNCipedia lincRNA (lnc-RP11-422N16.3.1-1), lincRNA [lnc-RP11-422N16.3.1-1:1]	0.47964165	0.0077180467
A_23_P53390	*lnc-UHRF2-1*	LNCipedia lincRNA (lnc-UHRF2-1), lincRNA [lnc-UHRF2-1:1]	-1.4310906	0.0025001846
A_23_P134935	*IFI27L2*	Homo sapiens interferon, alpha-inducible protein 27-like 2 (IFI27L2), mRNA [NM_032036]	-0.98588395	0.003956336
A_23_P202448	*PTPRB*	Homo sapiens protein tyrosine phosphatase, receptor type, B (PTPRB), transcript variant 2, mRNA [NM_002837]	-4.232866	0.00431082
A_23_P111995	*DUSP4*	Homo sapiens dual specificity phosphatase 4 (DUSP4), transcript variant 1, mRNA [NM_001394]	-1.7266382	0.005842833
A_23_P40453	*CXCL12*	Homo sapiens chemokine (C-X-C motif) ligand 12 (CXCL12), transcript variant 1, mRNA [NM_199168]	-1.2824626	0.0044813035

A_23_P101185	*LOXL2*	Homo sapiens lysyl oxidase-like 2 (LOXL2), mRNA [NM_002318]	-0.8260541	0.008870375
A_24_P156501	*CBR3*	Homo sapiens carbonyl reductase 3 (CBR3), mRNA [NM_001236]	-0.66572833	0.008298705
A_23_P160546	*NARS*	Homo sapiens asparaginyl-tRNA synthetase (NARS), mRNA [NM_004539]	-0.79568523	0.0062273466
A_23_P11705	*EBF1*	Homo sapiens early B-cell factor 1 (EBF1), transcript variant 2, mRNA [NM_024007]	-3.169417	0.0030924478
A_22_P00017726	*FAM63A*	Homo sapiens family with sequence similarity 63, member A (FAM63A), transcript variant 2, mRNA [NM_001040217]	-1.0706067	0.0105750235
A_24_P251969	*BSDC1*	Homo sapiens BSD domain containing 1 (BSDC1), transcript variant 2, mRNA [NM_018045]	-0.6501259	0.0039416323
A_33_P3230688	*KIZ*	Homo sapiens kizuna centrosomal protein (KIZ), transcript variant 1, mRNA [NM_018474]	-1.2304974	0.0055957953
A_33_P3301174	*FGF1*	Homo sapiens fibroblast growth factor 1 (acidic) (FGF1), transcript variant 1, mRNA [NM_000800]	-1.4602578	0.010138025
A_33_P3215078	*CDK10*	Homo sapiens cyclin-dependent kinase 10 (CDK10), transcript variant a, mRNA [NM_052988]	-1.0750366	0.011530419
A_22_P00003284	*TMED2*	Homo sapiens transmembrane emp24 domain trafficking protein 2 (TMED2), mRNA [NM_006815]	-0.6447846	0.0044240346
A_23_P132763	*CS*	Homo sapiens citrate synthase (CS), mRNA [NM_004077]	-0.50083643	0.01704795
A_24_P943472	*MIR205HG*	Homo sapiens MIR205 host gene (non-protein coding) (MIR205HG), mRNA [NM_001104548]	0.9214389	0.011575981
A_24_P876522	*VGLL3*	Homo sapiens vestigial-like family member 3 (VGLL3), mRNA [NM_016206]	-2.568134	0.0029691157
A_23_P252155	*NR1D2*	Homo sapiens nuclear receptor subfamily 1, group D, member 2 (NR1D2), transcript variant 1, mRNA [NM_005126]	-1.1420004	0.0029471128
A_24_P392060	*GPX8*	Homo sapiens glutathione peroxidase 8 (putative) (GPX8), mRNA [NM_001008397]	-0.75124645	0.0065117455
A_33_P3421219	*STRBP*	Homo sapiens spermatid perinuclear RNA binding protein (STRBP), transcript variant 1, mRNA [NM_018387]	-0.87211084	0.014834527
A_23_P259741	*SIMC1*	Homo sapiens SUMO-interacting motifs containing 1 (SIMC1), mRNA [NM_198567]	-0.3366546	0.016059838
A_33_P3278347	*lnc-SHCBP1L-1*	Homo sapiens cDNA FLJ10580 fis, clone NT2RP2003533. [AK001442]	-0.80800605	0.009679106
A_33_P3366053	*SATB1*	Homo sapiens SATB homeobox 1 (SATB1), transcript variant 1, mRNA [NM_002971]	-1.3895674	0.0061896956
A_23_P26457	*B3GALT5-AS1*	Homo sapiens B3GALT5 antisense RNA 1 (B3GALT5-AS1), transcript variant 1, long non-coding RNA [NR_026542]	0.4638496	0.005612162

A_23_P19702	*ADPRH*	Homo sapiens ADP-ribosylarginine hydrolase (ADPRH), transcript variant 1, mRNA [NM_001125]	-2.0272717	0.002804883
A_23_P337753	*HBA2*	Homo sapiens hemoglobin, alpha 2 (HBA2), mRNA [NM_000517]	1.667103	0.010533048
A_23_P53176	*TAB2*	Homo sapiens TGF-beta activated kinase 1/MAP3K7 binding protein 2 (TAB2), transcript variant 1, mRNA [NM_015093]	-0.9115587	0.008318697
A_24_P391260	*SP140L*	Homo sapiens SP140 nuclear body protein-like (SP140L), mRNA [NM_138402]	0.5183977	0.011479118
A_23_P215883	*FOLR1*	Homo sapiens folate receptor 1 (adult) (FOLR1), transcript variant 1, mRNA [NM_016725]	-0.79378384	0.009355827
A_22_P00020986	*PTTG1IP*	Homo sapiens pituitary tumor-transforming 1 interacting protein (PTTG1IP), transcript variant 1, mRNA [NM_004339]	-1.3915181	0.013250213
A_24_P156049	*NCALD*	Homo sapiens neurocalcin delta (NCALD), transcript variant 7, mRNA [NM_001040630]	-0.83482724	0.010860952
A_22_P00010633	*GATA2-AS1*	Homo sapiens GATA2 antisense RNA 1 (GATA2-AS1), long non-coding RNA [NR_125398]	0.99241096	0.0036517929
A_33_P3407424	*SLC39A6*	Homo sapiens solute carrier family 39 (zinc transporter), member 6 (SLC39A6), transcript variant 1, mRNA [NM_012319]	-0.9811267	0.0024890031
A_33_P3303577	*lnc-NDUFV3-1*	LNCipedia lincRNA (lnc-NDUFV3-1), lincRNA [lnc-NDUFV3-1:1]	-1.3181795	0.0037333963
A_33_P3362616	*CDC42EP1*	Homo sapiens CDC42 effector protein (Rho GTPase binding) 1 (CDC42EP1), mRNA [NM_152243]	-0.7640877	0.011377638
A_23_P212119	*TMEM110*	Homo sapiens transmembrane protein 110 (TMEM110), mRNA [NM_198563]	-0.842983	0.0036050293
A_33_P3290672	*PRR3*	Homo sapiens proline rich 3 (PRR3), transcript variant 1, mRNA [NM_025263]	-1.0587544	0.013594373
A_23_P148446	*GALNT15*	Homo sapiens polypeptide N-acetylgalactosaminyltransferase 15 (GALNT15), mRNA [NM_054110]	-0.9068193	0.010274393
A_33_P3244165	*SELT*	Homo sapiens selenoprotein T (SELT), mRNA [NM_016275]	-0.5520517	0.015378117
A_23_P85004	*UBE2A*	Homo sapiens ubiquitin-conjugating enzyme E2A (UBE2A), transcript variant 1, mRNA [NM_003336]	-0.8861003	0.011054281
A_23_P140301	*RNA28S5*	Homo sapiens RNA, 28S ribosomal 5 (RNA28S5), ribosomal RNA [NR_003287]	1.0175818	0.0150135
A_22_P00009484	*DIAPH2*	Homo sapiens diaphanous-related formin 2 (DIAPH2), transcript variant 12C, mRNA [NM_007309]	-0.6576756	0.0072714784
A_23_P352266	*PSMA3*	Homo sapiens proteasome (prosome, macropain) subunit, alpha type, 3 (PSMA3), transcript variant 1, mRNA [NM_002788]	-0.3842287	0.009689709

A_24_P413988	*lnc-MAGEB1-1*	LNCipedia lincRNA (lnc-MAGEB1-1), lincRNA [lnc-MAGEB1-1:1]	1.572819	0.010212053
A_24_P210637	*BCL2*	Homo sapiens B-cell CLL/lymphoma 2 (BCL2), transcript variant alpha, mRNA [NM_000633]	-1.2272627	0.007488306
A_23_P337729	*TGOLN2*	Homo sapiens trans-golgi network protein 2 (TGOLN2), transcript variant 1, mRNA [NM_006464]	-0.7747288	0.007206317
A_33_P3300495	*CHST14*	Homo sapiens carbohydrate (N-acetylgalactosamine 4-0) sulfotransferase 14 (CHST14), mRNA [NM_130468]	0.82678497	0.010310044
A_32_P41065	*TMEM180*	Homo sapiens transmembrane protein 180 (TMEM180), mRNA [NM_024789]	-1.5000671	0.015414845
A_23_P320185	*PLGLB1*	plasminogen-like B1 [Source:HGNC Symbol;Acc:HGNC:9072] [ENST00000409310]	-1.8245381	0.0031941293
A_21_P0010725	*TMCC1*	Homo sapiens transmembrane and coiled-coil domain family 1 (TMCC1), transcript variant 1, mRNA [NM_001017395]	-1.319942	0.007677732
A_23_P104054	*NDUFA11*	Homo sapiens NADH dehydrogenase (ubiquinone) 1 alpha subcomplex, 11, 14.7kDa (NDUFA11), transcript variant 1, mRNA [NM_175614]	-0.8466632	0.0074155424
A_23_P149545	*SUCO*	Homo sapiens SUN domain containing ossification factor (SUCO), transcript variant 2, mRNA [NM_016227]	-0.6218144	0.011172352
A_22_P00020359	*lnc-SOX11-3*	LNCipedia lincRNA (lnc-SOX11-3), lincRNA [lnc-SOX11-3:12]	1.6125839	0.0034964236
A_23_P18798	*HIST2H2BE*	Homo sapiens histone cluster 2, H2be (HIST2H2BE), mRNA [NM_003528]	1.6062188	0.0037856582
A_33_P3330211	*CTTNBP2NL*	CTTNBP2 N-terminal like [Source:HGNC Symbol;Acc:HGNC:25330] [ENST00000607039]	-0.97496915	0.011411947
A_23_P53288	*PCDHB9*	Homo sapiens protocadherin beta 9 (PCDHB9), mRNA [NM_019119]	1.3362588	0.0145460265
A_32_P58937	*AUTS2*	Homo sapiens autism susceptibility candidate 2 (AUTS2), transcript variant 3, mRNA [NM_001127232]	-2.1950274	0.005945938
A_33_P3390057	*CNPY2*	Homo sapiens canopy FGF signaling regulator 2 (CNPY2), transcript variant 1, mRNA [NM_014255]	-1.1964827	0.005142579
A_24_P683011	*MBTD1*	Homo sapiens mbt domain containing 1 (MBTD1), mRNA [NM_017643]	-0.56866986	0.012773474
A_33_P3348529	*TM4SF1*	Homo sapiens transmembrane 4 L six family member 1 (TM4SF1), mRNA [NM_014220]	0.8659742	0.0054879813
A_33_P3841368	*C8orf88*	Homo sapiens chromosome 8 open reading frame 88 (C8orf88), mRNA [NM_001190972]	-0.83202446	0.0056287716
A_24_P941167	*FBXO25*	F-box protein 25 [Source:HGNC Symbol;Acc:HGNC:13596] [ENST00000382824]	-0.96664983	0.014442452
A_21_P0000548	*TG*	Homo sapiens thyroglobulin (TG), mRNA [NM_003235]	-1.3344557	0.0031941293

A_24_P122137	*APOL6*	Homo sapiens apolipoprotein L, 6 (APOL6), mRNA [NM_030641]	-1.3305798	0.0059353677
A_24_P195724	*PDCD6IP*	Homo sapiens programmed cell death 6 interacting protein (PDCD6IP), transcript variant 4, mRNA [NM_001256192]	-0.7431137	0.010435809
A_23_P89589	*LIF*	Homo sapiens leukemia inhibitory factor (LIF), transcript variant 1, mRNA [NM_002309]	4.6046095	0.002804883
A_23_P149975	*PER1*	Homo sapiens period circadian clock 1 (PER1), mRNA [NM_002616]	2.2711685	0.0052637225
A_33_P3398156	*FRRS1*	Homo sapiens ferric-chelate reductase 1 (FRRS1), mRNA [NM_001013660]	-1.3156012	0.0062385905
A_24_P178503	*FAM107B*	Homo sapiens family with sequence similarity 107, member B (FAM107B), transcript variant 2, mRNA [NM_031453]	0.63531405	0.01027789
A_24_P38081	*CYS1*	Homo sapiens cystin 1 (CYS1), mRNA [NM_001037160]	-1.908114	0.0047066417
A_33_P3315906	*ABCC9*	Homo sapiens ATP-binding cassette, sub-family C (CFTR/MRP), member 9 (ABCC9), transcript variant SUR2A, mRNA [NM_005691]	0.8763998	0.008488166
A_33_P3271657	*FKBP5*	Homo sapiens FK506 binding protein 5 (FKBP5), transcript variant 1, mRNA [NM_004117]	0.6175328	0.012785839
A_23_P106661	*PTP4A3*	Homo sapiens protein tyrosine phosphatase type IVA, member 3 (PTP4A3), transcript variant 1, mRNA [NM_032611]	-1.465409	0.013197363
A_24_P175059	*HHIPL1*	Homo sapiens HHIP-like 1 (HHIPL1), transcript variant 1, mRNA [NM_001127258]	-2.2560813	0.0048989807
A_21_P0012703	*CMTM1*	Homo sapiens CKLF-like MARVEL transmembrane domain containing 1 (CMTM1), transcript variant 17, mRNA [NM_052999]	-0.7219865	0.010190244
A_32_P59486	*ATG5*	Homo sapiens autophagy related 5 (ATG5), transcript variant 1, mRNA [NM_004849]	-1.0709928	0.002538878
A_21_P0013654	*XLOC_l2_011415*	BROAD Institute lincRNA (XLOC_l2_011415), lincRNA [TCONS_l2_00021481]	0.54588675	0.006503996
A_33_P3272508	*SDHD*	Homo sapiens succinate dehydrogenase complex, subunit D, integral membrane protein (SDHD), transcript variant 1, mRNA [NM_003002]	-1.3939326	0.0062257075
A_23_P133245	*LOC102723780*	PREDICTED: Homo sapiens uncharacterized LOC102723780 (LOC102723780), ncRNA [XR_426514]	-1.0653872	0.0138141345
A_23_P212089	*MRPL57*	Homo sapiens mitochondrial ribosomal protein L57 (MRPL57), mRNA [NM_024026]	-0.6815468	0.01623537
A_22_P00017514	*IK*	Homo sapiens IK cytokine, down-regulator of HLA II (IK), mRNA [NM_006083]	-0.8144005	0.009452373
A_21_P0014458	*NFKBIZ*	Homo sapiens nuclear factor of kappa light polypeptide gene enhancer in B-cells inhibitor,	1.2754416	0.0064337924

		zeta (NFKBIZ), transcript variant 1, mRNA [NM_031419]		
A_23_P383435	*INTS6-AS1*	Homo sapiens INTS6 antisense RNA 1 (INTS6-AS1), long non-coding RNA [NR_103812]	-1.0576614	0.0029760713
A_23_P112135	*ZNHIT3*	Homo sapiens zinc finger, HIT-type containing 3 (ZNHIT3), transcript variant 1, mRNA [NM_004773]	-0.58226776	0.008017601
A_23_P124476	*ZCCHC8*	Homo sapiens zinc finger, CCHC domain containing 8 (ZCCHC8), mRNA [NM_017612]	-1.0400238	0.010447919
A_23_P251927	*TRAM1*	Homo sapiens translocation associated membrane protein 1 (TRAM1), mRNA [NM_014294]	-0.8416319	0.00910296
A_23_P2143	*CLCN3*	Homo sapiens chloride channel, voltage-sensitive 3 (CLCN3), transcript variant e, mRNA [NM_173872]	-0.7981479	0.010274393
A_23_P35309	*CHCHD3*	Homo sapiens coiled-coil-helix-coiled-coil-helix domain containing 3 (CHCHD3), mRNA [NM_017812]	-0.7899212	0.010960915
A_33_P3298159	*SPCS2*	Homo sapiens signal peptidase complex subunit 2 homolog (S. cerevisiae) (SPCS2), mRNA [NM_014752]	-0.9963631	0.0049676057
A_23_P130553	*TAF5L*	Homo sapiens TAF5-like RNA polymerase II, p300/CBP-associated factor (PCAF)-associated factor, 65kDa (TAF5L), transcript variant 1, mRNA [NM_014409]	-0.6026234	0.00180587
A_21_P0004863	*PTGDS*	Homo sapiens prostaglandin D2 synthase 21kDa (brain) (PTGDS), mRNA [NM_000954]	-2.0997891	0.0065723537
A_23_P104318	*SHKBP1*	Homo sapiens SH3KBP1 binding protein 1 (SHKBP1), mRNA [NM_138392]	1.215637	0.013348818
A_23_P112634	*DDIT4*	Homo sapiens DNA-damage-inducible transcript 4 (DDIT4), mRNA [NM_019058]	-1.1510161	0.004723954
A_33_P3358158	*SMIM14*	Homo sapiens small integral membrane protein 14 (SMIM14), mRNA [NM_174921]	-1.470689	0.0025001846
A_23_P361049	*LINC00467*	Homo sapiens long intergenic non-protein coding RNA 467 (LINC00467), long non-coding RNA [NR_026761]	-0.59586	0.01634805
A_23_P49646	*CRYBB2P1*	Homo sapiens crystallin, beta B2 pseudogene 1 (CRYBB2P1), transcript variant 1, non-coding RNA [NR_033733]	-0.68809104	0.015144069
A_23_P217475	*MYO1B*	Homo sapiens myosin IB (MYO1B), transcript variant 2, mRNA [NM_012223]	-1.06192	0.0034913097
A_23_P132793	*PRPSAP2*	Homo sapiens phosphoribosyl pyrophosphate synthetase-associated protein 2 (PRPSAP2), transcript variant 1, mRNA [NM_002767]	-0.71347135	0.011772038
A_33_P3336223	*IDS*	Homo sapiens iduronate 2-sulfatase (IDS), transcript variant 2, mRNA [NM_006123]	-0.954021	0.0062618116

A_33_P3373364	*MANF*	Homo sapiens mesencephalic astrocyte-derived neurotrophic factor (MANF), mRNA [NM_006010]	-1.1099911	0.0044813035
A_23_P2446	*CLIC4*	Homo sapiens chloride intracellular channel 4 (CLIC4), mRNA [NM_013943]	-1.4249797	0.0057647754
A_23_P210274	*TMEM5*	Homo sapiens transmembrane protein 5 (TMEM5), transcript variant 1, mRNA [NM_014254]	-0.65320677	0.007990728
A_22_P00016479	*TAS2R30*	Homo sapiens taste receptor, type 2, member 30 (TAS2R30), mRNA [NM_001097643]	-0.77439535	0.0064359996
A_33_P3375451	*MOB4*	Homo sapiens MOB family member 4, phocein (MOB4), transcript variant 1, mRNA [NM_015387]	-0.8159661	0.007647175
A_23_P2725	*lnc-TMEM86A-2*	Homo sapiens mRNA; cDNA DKFZp434F1626 (from clone DKFZp434F1626). [AL137366]	-0.6356034	0.011204836
A_24_P416159	*NYNRIN*	Homo sapiens NYN domain and retroviral integrase containing (NYNRIN), mRNA [NM_025081]	1.1954364	0.008276945
A_24_P127312	*RPL21*	Homo sapiens ribosomal protein L21 (RPL21), mRNA [NM_000982]	-0.21927282	0.013460425
A_21_P0005658	*RBL2*	Homo sapiens retinoblastoma-like 2 (RBL2), mRNA [NM_005611]	-0.9684837	0.0038056036
A_24_P307065	*RNF222*	Homo sapiens ring finger protein 222 (RNF222), mRNA [NM_001146684]	-0.46587998	0.011824575
A_33_P3235432	*DBNDD1*	Homo sapiens dysbindin (dystrobrevin binding protein 1) domain containing 1 (DBNDD1), transcript variant 1, mRNA [NM_001042610]	2.4906125	0.0057059666
A_22_P00017308	*NMNAT1*	Homo sapiens nicotinamide nucleotide adenylyltransferase 1 (NMNAT1), transcript variant 3, mRNA [NM_001297779]	3.3350267	0.0036517929
A_22_P00009053	*FLJ35934*	Homo sapiens FLJ35934 (FLJ35934), long non-coding RNA [NR_104343]	-0.98893	0.013938657
A_23_P90722	*MIR99AHG*	Homo sapiens mir-99a-let-7c cluster host gene (non-protein coding) (MIR99AHG), transcript variant 1, long non-coding RNA [NR_027790]	-0.64889127	0.009961446
A_21_P0013512	*LOC101928236*	PREDICTED: Homo sapiens uncharacterized LOC101928236 (LOC101928236), transcript variant X1, ncRNA [XR_241604]	-0.37263632	0.015203984
A_23_P353056	*PTPRN*	Homo sapiens protein tyrosine phosphatase, receptor type, N (PTPRN), transcript variant 1, mRNA [NM_002846]	1.0056466	0.011745691
A_33_P3379967	*C2CD2L*	Homo sapiens C2CD2-like (C2CD2L), transcript variant 1, mRNA [NM_014807]	-0.5350938	0.010455912
A_32_P133518	*PPFIBP1*	Homo sapiens PTPRF interacting protein, binding protein 1 (liprin beta 1) (PPFIBP1), transcript variant 1, mRNA [NM_003622]	-1.4460204	0.0058555133

A_33_P3415097	*HLA-F*	Homo sapiens major histocompatibility complex, class I, F (HLA-F), transcript variant 3, mRNA [NM_001098478]	-0.8184698	0.012713055
A_33_P3277140	*CLCN4*	Homo sapiens chloride channel, voltage-sensitive 4 (CLCN4), transcript variant 1, mRNA [NM_001830]	-1.6608913	0.010056441
A_23_P92362	*SLC9A6*	Homo sapiens solute carrier family 9, subfamily A (NHE6, cation proton antiporter 6), member 6 (SLC9A6), transcript variant 1, mRNA [NM_001042537]	-0.77747107	0.011030013
A_23_P422071	*MINOS1*	Homo sapiens mitochondrial inner membrane organizing system 1 (MINOS1), transcript variant 1, mRNA [NM_001032363]	-0.6070659	0.011566584
A_21_P0004864	*NDUFC1*	Homo sapiens NADH dehydrogenase (ubiquinone) 1, subcomplex unknown, 1, 6kDa (NDUFC1), transcript variant 2, mRNA [NM_002494]	-0.92666554	0.0059353677
A_33_P3392391	*B3GALT4*	Homo sapiens UDP-Gal:betaGlcNAc beta 1,3-galactosyltransferase, polypeptide 4 (B3GALT4), mRNA [NM_003782]	-1.997174	0.0044240346
A_23_P399255	*CPT1C*	Homo sapiens carnitine palmitoyltransferase 1C (CPT1C), transcript variant 3, mRNA [NM_001199752]	1.0108802	0.0061608143
A_23_P133284	*NDUFS5*	Homo sapiens NADH dehydrogenase (ubiquinone) Fe-S protein 5, 15kDa (NADH-coenzyme Q reductase) (NDUFS5), transcript variant 1, mRNA [NM_004552]	-1.1098602	0.004989007
A_23_P38864	*RNF182*	Homo sapiens ring finger protein 182 (RNF182), transcript variant 2, mRNA [NM_152737]	-3.6800194	0.010822477
A_23_P145074	*RIOK2*	Homo sapiens RIO kinase 2 (RIOK2), transcript variant 1, mRNA [NM_018343]	-0.542331	0.011730079
A_23_P428129	*RABAC1*	Homo sapiens Rab acceptor 1 (prenylated) (RABAC1), mRNA [NM_006423]	-0.75906926	0.0057647754
A_32_P342064	*PNRC1*	Homo sapiens proline-rich nuclear receptor coactivator 1 (PNRC1), mRNA [NM_006813]	-0.7822065	0.009710156
A_33_P3410409	*CDKN1C*	Homo sapiens cyclin-dependent kinase inhibitor 1C (p57, Kip2) (CDKN1C), transcript variant 1, mRNA [NM_000076]	-1.5409203	0.005255513
A_24_P40229	*FTH1*	Homo sapiens ferritin, heavy polypeptide 1 (FTH1), mRNA [NM_002032]	1.125835	0.009820808
A_23_P94095	*LAMP2*	Homo sapiens lysosomal-associated membrane protein 2 (LAMP2), transcript variant C, mRNA [NM_001122606]	-0.9043529	0.008159728
A_32_P794272	*CRNKL1*	Homo sapiens crooked neck pre-mRNA splicing factor 1 (CRNKL1), transcript variant 1, mRNA [NM_016652]	-0.7773359	0.009113534

A_22_P00017061	*ANKRD46*	Homo sapiens ankyrin repeat domain 46 (ANKRD46), transcript variant 2, mRNA [NM_198401]	-0.68881553	0.0072230194
A_33_P3243554	*GNAI2*	Homo sapiens guanine nucleotide binding protein (G protein), alpha inhibiting activity polypeptide 2 (GNAI2), transcript variant 5, mRNA [NM_001282619]	-0.5003635	0.0031750489
A_23_P20970	*CPSF7*	Homo sapiens cleavage and polyadenylation specific factor 7, 59kDa (CPSF7), transcript variant 1, mRNA [NM_024811]	-0.45442957	0.007683969
A_21_P0013714	*ATG12*	Homo sapiens autophagy related 12 (ATG12), transcript variant 1, mRNA [NM_004707]	-0.9462311	0.005173042
A_33_P3220929	*TMEM123*	Homo sapiens transmembrane protein 123 (TMEM123), mRNA [NM_052932]	-1.2025894	0.0029695062
A_21_P0001705	*ATR*	Homo sapiens ATR serine/threonine kinase (ATR), mRNA [NM_001184]	-0.46795848	0.016549561
A_21_P0000310	*JOSD2*	Homo sapiens Josephin domain containing 2 (JOSD2), transcript variant 3, mRNA [NM_001270641]	1.1660582	0.0059963865
A_33_P3276062	*LINC00869*	Homo sapiens long intergenic non-protein coding RNA 869 (LINC00869), transcript variant 8, long non-coding RNA [NR_111952]	-0.8238711	0.011328784
A_24_P10137	*SNORA12*	Homo sapiens small nucleolar RNA, H/ACA box 12 (SNORA12), small nucleolar RNA [NR_002954]	-1.7594874	0.00180587
A_21_P0006626	*RGCC*	Homo sapiens regulator of cell cycle (RGCC), mRNA [NM_014059]	3.9861012	0.0025741379
A_33_P3209386	*ASMTL*	Homo sapiens acetylserotonin O-methyltransferase-like (ASMTL), transcript variant 1, mRNA [NM_004192]	-1.3380628	0.01240238
A_24_P81298	*RRP12*	ribosomal RNA processing 12 homolog (S. cerevisiae) [Source:HGNC Symbol;Acc:HGNC:29100] [ENST00000621606]	-0.44942424	0.0096938675
A_24_P118196	*TXNDC17*	Homo sapiens thioredoxin domain containing 17 (TXNDC17), mRNA [NM_032731]	-0.9457729	0.008189544
A_23_P342138	*PPP6C*	Homo sapiens protein phosphatase 6, catalytic subunit (PPP6C), transcript variant 1, mRNA [NM_001123355]	-1.2642922	0.006937616
A_23_P23266	*GXYLT2*	Homo sapiens glucoside xylosyltransferase 2 (GXYLT2), mRNA [NM_001080393]	-2.0626693	0.005116237
A_19_P00322407	*ADAMTSL1*	Homo sapiens ADAMTS-like 1 (ADAMTSL1), transcript variant 4, mRNA [NM_001040272]	-2.1271665	0.0027248112
A_22_P00021677	*BLZF1*	basic leucine zipper nuclear factor 1 [Source:HGNC Symbol;Acc:HGNC:1065] [ENST00000367808]	-0.80058306	0.0053370837
A_23_P318604	*STXBP5-AS1*	Homo sapiens STXBP5 antisense RNA 1 (STXBP5-AS1), long non-coding RNA [NR_034115]	0.5440862	0.014705425

A_22_P00016008	*KRIT1*	Homo sapiens KRIT1, ankyrin repeat containing (KRIT1), transcript variant 4, mRNA [NM_194455]	-0.4166098	0.009724618
A_21_P0011843	*CYHR1*	cysteine/histidine-rich 1 [Source:HGNC Symbol;Acc:HGNC:17806] [ENST00000528663]	-0.711602	0.012329381
A_23_P150053	*lnc-TFB2M-2*	LNCipedia lincRNA (lnc-TFB2M-2), lincRNA [lnc-TFB2M-2:1]	-0.8477045	0.012168611
A_21_P0014388	*PGM5*	phosphoglucomutase 5 [Source:HGNC Symbol;Acc:HGNC:8908] [ENST00000604870]	2.1407022	0.0054418826
A_23_P83298	*ACTA2*	Homo sapiens actin, alpha 2, smooth muscle, aorta (ACTA2), transcript variant 2, mRNA [NM_001613]	-3.174201	0.0032074507
A_33_P3296592	*DNM2*	dynamin 2 [Source:HGNC Symbol;Acc:HGNC:2974] [ENST00000590787]	0.6808618	0.017157277
A_24_P355944	*PRRX2*	Homo sapiens paired related homeobox 2 (PRRX2), mRNA [NM_016307]	-0.48756737	0.007474992
A_24_P406754	*C11orf30*	Homo sapiens chromosome 11 open reading frame 30 (C11orf30), transcript variant 1, mRNA [NM_001300942]	-1.1320025	0.003609745
A_24_P140204	*EFNB2*	Homo sapiens ephrin-B2 (EFNB2), mRNA [NM_004093]	-1.3223693	0.005438366
A_33_P3405444	*LOXL4*	Homo sapiens lysyl oxidase-like 4 (LOXL4), mRNA [NM_032211]	-1.210926	0.0065142275
A_23_P9362	*PXK*	Homo sapiens PX domain containing serine/threonine kinase (PXK), transcript variant 1, mRNA [NM_017771]	-1.0756402	0.006503996
A_23_P36795	*WASL*	Homo sapiens Wiskott-Aldrich syndrome-like (WASL), mRNA [NM_003941]	-0.7926939	0.007889792
A_22_P00018253	*PSMB7*	Homo sapiens proteasome (prosome, macropain) subunit, beta type, 7 (PSMB7), mRNA [NM_002799]	-0.6118718	0.01540069
A_23_P13852	*SYT1*	Homo sapiens synaptotagmin I (SYT1), transcript variant 1, mRNA [NM_005639]	1.0940616	0.0074182227
A_21_P0002619	*CLUHP3*	Homo sapiens clustered mitochondria (cluA/CLU1) homolog pseudogene 3 (CLUHP3), long non-coding RNA [NR_024034]	0.9887936	0.0069863
A_24_P373844	*STRAP*	Homo sapiens serine/threonine kinase receptor associated protein (STRAP), mRNA [NM_007178]	-0.48239735	0.011851147
A_23_P99138	*lnc-AC007405.7.1-2*	LNCipedia lincRNA (lnc-AC007405.7.1-2), lincRNA [lnc-AC007405.7.1-2:1]	1.0372419	0.0074393568
A_23_P319565	*KCTD15*	Homo sapiens potassium channel tetramerization domain containing 15 (KCTD15), transcript variant 1, mRNA [NM_024076]	-0.7556495	0.01649328
A_23_P351734	*MRPL51*	Homo sapiens mitochondrial ribosomal protein L51 (MRPL51), mRNA [NM_016497]	-0.59731025	0.0044600246

A_33_P3297245	*PGBD3*	Homo sapiens piggyBac transposable element derived 3 (PGBD3), mRNA [NM_170753]	-1.4151267	0.002804883
A_23_P102611	*NPHP4*	Homo sapiens nephronophthisis 4 (NPHP4), transcript variant 1, mRNA [NM_015102]	-0.9931638	0.009304936
A_24_P551028	*RRAS2*	Homo sapiens related RAS viral (r-ras) oncogene homolog 2 (RRAS2), transcript variant 1, mRNA [NM_012250]	-1.2787795	0.005348777
A_24_P56317	*WISP2*	Homo sapiens WNT1 inducible signaling pathway protein 2 (WISP2), mRNA [NM_003881]	-5.4892225	0.0022181747
A_22_P00020404	*SPOPL*	Homo sapiens speckle-type POZ protein-like (SPOPL), mRNA [NM_001001664]	-0.9718196	0.0057647754
A_23_P58967	*MBNL2*	Homo sapiens muscleblind-like splicing regulator 2 (MBNL2), transcript variant 1, mRNA [NM_144778]	-0.84610647	0.011672903
A_32_P217655	*MIR137HG*	Homo sapiens MIR137 host gene (non-protein coding) (MIR137HG), long non-coding RNA [NR_046105]	-1.2515363	0.015833389
A_23_P122443	*ZC3H11A*	Homo sapiens zinc finger CCCH-type containing 11A (ZC3H11A), mRNA [NM_014827]	-0.6952784	0.011544451
A_23_P111273	*LOC645166*	Homo sapiens lymphocyte-specific protein 1 pseudogene (LOC645166), transcript variant 2, non-coding RNA [NR_027355]	-1.0902407	0.007901642
A_21_P0003290	*HIST1H1C*	Homo sapiens histone cluster 1, H1c (HIST1H1C), mRNA [NM_005319]	-0.49858472	0.016888233
A_23_P407112	*TBC1D7*	Homo sapiens TBC1 domain family, member 7 (TBC1D7), transcript variant 1, mRNA [NM_016495]	-0.725571	0.011078993
A_22_P00016147	*lnc-MINA-3*	LNCipedia lincRNA (lnc-MINA-3), lincRNA [lnc-MINA-3:2]	0.8886027	0.012161102
A_24_P941831	*SPATA18*	Homo sapiens spermatogenesis associated 18 (SPATA18), transcript variant 1, mRNA [NM_145263]	-1.5894523	0.0029675725
A_23_P81690	*lnc-TIMM9-2*	LNCipedia lincRNA (lnc-TIMM9-2), lincRNA [lnc-TIMM9-2:15]	-0.91569066	0.011743996
A_33_P3348797	*TMEM237*	Homo sapiens transmembrane protein 237 (TMEM237), transcript variant 1, mRNA [NM_001044385]	-0.90376204	0.007190758
A_22_P00015390	*COX7A2*	Homo sapiens cytochrome c oxidase subunit VIIa polypeptide 2 (liver) (COX7A2), transcript variant 1, mRNA [NM_001865]	-0.5073526	0.003667703
A_33_P3400699	*C5orf24*	Homo sapiens chromosome 5 open reading frame 24 (C5orf24), transcript variant 1, mRNA [NM_001135586]	-0.7077927	0.0073130694
A_33_P3265945	*lnc-SRGAP3-1*	LNCipedia lincRNA (lnc-SRGAP3-1), lincRNA [lnc-SRGAP3-1:21]	-0.33380502	0.016746266

A_23_P69383	*SLC26A5*	Homo sapiens solute carrier family 26 (anion exchanger), member 5 (SLC26A5), transcript variant b, mRNA [NM_206883]	-0.5528807	0.011069593
A_23_P121182	*PARP9*	Homo sapiens poly (ADP-ribose) polymerase family, member 9 (PARP9), transcript variant 1, mRNA [NM_031458]	-0.9002722	0.008362665
A_23_P213137	*LOC100129550*	Homo sapiens uncharacterized LOC100129550 (LOC100129550), long non-coding RNA [NR_024618]	-0.668313	0.0059005646
A_32_P74120	*HACL1*	Homo sapiens 2-hydroxyacyl-CoA lyase 1 (HACL1), transcript variant 1, mRNA [NM_012260]	-0.9399173	0.0070802537
A_23_P20275	*LNX1*	Homo sapiens ligand of numb-protein X 1, E3 ubiquitin protein ligase (LNX1), transcript variant 2, mRNA [NM_032622]	-1.307621	0.007662565
A_23_P167828	*LINC00869*	Homo sapiens long intergenic non-protein coding RNA 869 (LINC00869), transcript variant 8, long non-coding RNA [NR_111952]	-1.1895483	0.005997236
A_33_P3282394	*PLEKHF2*	Homo sapiens pleckstrin homology domain containing, family F (with FYVE domain) member 2 (PLEKHF2), mRNA [NM_024613]	-1.1671671	0.0038274636
A_23_P168828	*RWDD1*	Homo sapiens RWD domain containing 1 (RWDD1), transcript variant 2, mRNA [NM_016104]	-0.919342	0.004536782
A_33_P3449417	*MLLT1*	Homo sapiens myeloid/lymphoid or mixed-lineage leukemia (trithorax homolog, Drosophila); translocated to, 1 (MLLT1), mRNA [NM_005934]	-1.0066338	0.0029276514
A_23_P132644	*KLF10*	Homo sapiens Kruppel-like factor 10 (KLF10), transcript variant 1, mRNA [NM_005655]	0.7141454	0.0047272886
A_32_P177024	*KARS*	Homo sapiens lysyl-tRNA synthetase (KARS), transcript variant 1, mRNA [NM_001130089]	-0.71440953	0.007610532
A_23_P9061	*NCEH1*	Homo sapiens neutral cholesterol ester hydrolase 1 (NCEH1), transcript variant 2, mRNA [NM_020792]	0.55041647	0.008307647
A_23_P27400	*SBDS*	Homo sapiens Shwachman-Bodian-Diamond syndrome (SBDS), mRNA [NM_016038]	-0.9875729	0.007501246
A_33_P3397443	*EIF3H*	Homo sapiens eukaryotic translation initiation factor 3, subunit H (EIF3H), mRNA [NM_003756]	-0.77571845	0.0063443403
A_33_P3272539	*HAS1*	Homo sapiens hyaluronan synthase 1 (HAS1), transcript variant 1, mRNA [NM_001523]	3.9221597	0.0072359135
A_22_P00004333	*PKMYT1*	Homo sapiens protein kinase, membrane associated tyrosine/threonine 1 (PKMYT1), transcript variant 2, mRNA [NM_182687]	0.75632644	0.01564773
A_23_P30254	*PLEKHG5*	Homo sapiens pleckstrin homology domain containing, family G (with RhoGef domain)	0.9633504	0.013781849

		member 5 (PLEKHG5), transcript variant 8, mRNA [NM_001265594]		
A_23_P408996	*PLK2*	Homo sapiens polo-like kinase 2 (PLK2), transcript variant 1, mRNA [NM_006622]	-0.40431887	0.015414845
A_23_P77160	*RRP1*	Homo sapiens ribosomal RNA processing 1 (RRP1), mRNA [NM_003683]	1.0558646	0.01218671
A_23_P102258	*MBOAT1*	Homo sapiens membrane bound O-acyltransferase domain containing 1 (MBOAT1), transcript variant 1, mRNA [NM_001080480]	-0.19188184	0.01696804
A_23_P141636	*RNF111*	Homo sapiens ring finger protein 111 (RNF111), transcript variant 2, mRNA [NM_017610]	-0.75690144	0.01273111
A_23_P42498	*MRPL53*	Homo sapiens mitochondrial ribosomal protein L53 (MRPL53), mRNA [NM_053050]	-0.9141712	0.007571981
A_23_P8185	*EIF4A3*	Homo sapiens eukaryotic translation initiation factor 4A3 (EIF4A3), mRNA [NM_014740]	-0.47551563	0.00910823
A_33_P3262789	*SNX3*	Homo sapiens sorting nexin 3 (SNX3), transcript variant 1, mRNA [NM_003795]	-0.8756859	0.008346524
A_23_P138507	*DYNLT1*	Homo sapiens dynein, light chain, Tctex-type 1 (DYNLT1), transcript variant 1, mRNA [NM_006519]	-0.8377393	0.006726202
A_23_P136347	*REEP6*	Homo sapiens receptor accessory protein 6 (REEP6), mRNA [NM_138393]	-1.8170116	0.009851655
A_22_P00016998	*CDK1*	Homo sapiens cyclin-dependent kinase 1 (CDK1), transcript variant 1, mRNA [NM_001786]	-10748863	0.0037124872
A_24_P8220	*EPS8*	Homo sapiens epidermal growth factor receptor pathway substrate 8 (EPS8), mRNA [NM_004447]	-0.88758755	0.002819256
A_23_P123905	*DSCR9*	Homo sapiens Down syndrome critical region 9 (non-protein coding) (DSCR9), long non-coding RNA [NR_026719]	0.7997124	0.01012583
A_24_P112750	*HS6ST1*	Homo sapiens heparan sulfate 6-O-sulfotransferase 1 (HS6ST1), mRNA [NM_004807]	0.4556749	0.011196168
A_23_P11995	*EXOSC3*	Homo sapiens exosome component 3 (EXOSC3), transcript variant 1, mRNA [NM_016042]	-0.43113998	0.005403412
A_23_P60296	*TFCP2*	Homo sapiens transcription factor CP2 (TFCP2), transcript variant 1, mRNA [NM_005653]	-0.92247224	0.016281858
A_23_P323094	*PRDX1*	Homo sapiens peroxiredoxin 1 (PRDX1), transcript variant 1, mRNA [NM_002574]	-0.44986323	0.005695306
A_33_P3271490	*OSTF1*	Homo sapiens osteoclast stimulating factor 1 (OSTF1), mRNA [NM_012383]	-1.0887327	0.005365543
A_23_P15944	*PHC1*	Homo sapiens polyhomeotic homolog 1 (Drosophila) (PHC1), mRNA [NM_004426]	-0.62342095	0.015272768

A_24_P66528	*RBMS1*	Homo sapiens RNA binding motif, single stranded interacting protein 1 (RBMS1), transcript variant 1, mRNA [NM_016836]	-0.5420475	0.0072222846
A_24_P131580	*ELAC1*	Homo sapiens elaC ribonuclease Z 1 (ELAC1), mRNA [NM_018696]	-0.89398146	0.008471989
A_23_P71752	*SRP9*	Homo sapiens signal recognition particle 9kDa (SRP9), transcript variant 2, mRNA [NM_003133]	-1.0649325	0.0037856582
A_24_P23411	*ALPPL2*	Homo sapiens alkaline phosphatase, placental-like 2 (ALPPL2), mRNA [NM_031313]	0.58922184	0.016647754
A_33_P3240078	*ZFAND5*	Homo sapiens zinc finger, AN1-type domain 5 (ZFAND5), transcript variant c, mRNA [NM_006007]	-0.5532729	0.013657404
A_21_P0000746	*ARMCX3*	Homo sapiens armadillo repeat containing, X-linked 3 (ARMCX3), transcript variant 1, mRNA [NM_016607]	-1.1250763	0.0043581175
A_22_P00007350	*LINC00933*	Homo sapiens long intergenic non-protein coding RNA 933 (LINC00933), transcript variant 1, long non-coding RNA [NR_038273]	-1.6188879	0.0037685214
A_33_P3316671	*MGST1*	Homo sapiens microsomal glutathione S-transferase 1 (MGST1), transcript variant 3, mRNA [NM_145791]	-0.3555538	0.0074116928
A_33_P3339531	*lnc-GPX1-1*	LNCipedia lincRNA (lnc-GPX1-1), lincRNA [lnc-GPX1-1:1]	-0.80285007	0.015558877
A_22_P00020023	*CHADL*	Homo sapiens chondroadherin-like (CHADL), mRNA [NM_138481]	-0.97382617	0.0063397386
A_33_P3336257	*MAPKAP1*	Homo sapiens mitogen-activated protein kinase associated protein 1 (MAPKAP1), transcript variant 6, mRNA [NM_001006618]	0.75854194	0.011926954
A_23_P61371	*PDP2*	Homo sapiens pyruvate dehyrogenase phosphatase catalytic subunit 2 (PDP2), mRNA [NM_020786]	-0.6046718	0.008248755
A_23_P131737	*IRX1*	Homo sapiens iroquois homeobox 1 (IRX1), mRNA [NM_024337]	-1.7404408	0.0037583082
A_23_P164737	*TMEM173*	Homo sapiens transmembrane protein 173 (TMEM173), transcript variant 1, mRNA [NM_198282]	-1.1021011	0.005659222
A_33_P3333708	*VPS54*	Homo sapiens vacuolar protein sorting 54 homolog (S. cerevisiae) (VPS54), transcript variant 1, mRNA [NM_016516]	-0.80875134	0.0053727482
A_23_P208334	*MED26*	Homo sapiens mediator complex subunit 26 (MED26), mRNA [NM_004831]	-0.6942277	0.011091872
A_24_P329353	*PDE4A*	Homo sapiens phosphodiesterase 4A, cAMP-specific (PDE4A), transcript variant 4, mRNA [NM_006202]	1.2146889	0.0065735937
A_23_P388670	*MINA*	Homo sapiens MYC induced nuclear antigen (MINA), transcript variant 1, mRNA [NM_001042533]	-0.7287815	0.0035461378

A_23_P35617	*ORAI2*	Homo sapiens ORAI calcium release-activated calcium modulator 2 (ORAI2), transcript variant 2, mRNA [NM_032831]	0.92927545	0.011087365
A_23_P327519	*LTA4H*	Homo sapiens leukotriene A4 hydrolase (LTA4H), transcript variant 1, mRNA [NM_000895]	-0.8698639	0.00741874
A_23_P115316	*PLCE1*	Homo sapiens phospholipase C, epsilon 1 (PLCE1), transcript variant 1, mRNA [NM_016341]	-2.1771436	0.003121324
A_33_P3372426	*STARD4*	Homo sapiens StAR-related lipid transfer (START) domain containing 4 (STARD4), mRNA [NM_139164]	-1.3324418	0.0055818916
A_23_P154806	*TOR3A*	Homo sapiens torsin family 3, member A (TOR3A), mRNA [NM_022371]	-0.8874266	0.006665037
A_32_P155091	*ADAMTS5*	Homo sapiens ADAM metallopeptidase with thrombospondin type 1 motif, 5 (ADAMTS5), mRNA [NM_007038]	-0.33010215	0.0042127552
A_21_P0009155	*EPB41L1*	Homo sapiens erythrocyte membrane protein band 4.1-like 1 (EPB41L1), transcript variant 1, mRNA [NM_012156]	-1.9169118	0.0032224318
A_23_P212545	*ATXN2L*	Homo sapiens ataxin 2-like (ATXN2L), transcript variant B, mRNA [NM_145714]	-0.50081086	0.014244717
A_21_P0000119	*RAB5A*	Homo sapiens RAB5A, member RAS oncogene family (RAB5A), transcript variant 1, mRNA [NM_004162]	-0.7266068	0.0075343307
A_23_P23924	*TMEM254*	Homo sapiens transmembrane protein 254 (TMEM254), transcript variant 1, mRNA [NM_025125]	-1.5494183	0.0049917083
A_33_P3357620	*SUGCT*	Homo sapiens succinyl-CoA:glutarate-CoA transferase (SUGCT), transcript variant 1, mRNA [NM_001193311]	-1.4665875	0.0029392217
A_33_P3268954	*CAPN2*	Homo sapiens calpain 2, (m/II) large subunit (CAPN2), transcript variant 1, mRNA [NM_001748]	-0.41509673	0.0041350042
A_23_P6196	*PAGR1*	Homo sapiens PAXIP1 associated glutamate-rich protein 1 (PAGR1), mRNA [NM_024516]	-0.68856126	0.00986522
A_33_P3349521	*FBLL1*	Homo sapiens fibrillarin-like 1 (FBLL1), non-coding RNA [NR_024356]	-1.2437528	0.012161102
A_33_P3413597	*SCAND1*	Homo sapiens SCAN domain containing 1 (SCAND1), transcript variant 1, mRNA [NM_016558]	0.60908794	0.01188095
A_33_P3348204	*SARAF*	Homo sapiens store-operated calcium entry-associated regulatory factor (SARAF), transcript variant 1, mRNA [NM_016127]	-0.9885165	0.0024890031
A_23_P376188	*NDUFB3*	Homo sapiens NADH dehydrogenase (ubiquinone) 1 beta subcomplex, 3, 12kDa (NDUFB3), transcript variant 2, mRNA [NM_001257102]	-0.66304487	0.016766664

A_23_P159305	*FAM218A*	Homo sapiens family with sequence similarity 218, member A (FAM218A), mRNA [NM_153027]	-1.6960936	0.009501149
A_23_P2884	*BEND7*	Homo sapiens BEN domain containing 7 (BEND7), transcript variant 2, mRNA [NM_001100912]	-1.2687039	0.0057647754
A_32_P129527	*TAF15*	Homo sapiens TAF15 RNA polymerase II, TATA box binding protein (TBP)-associated factor, 68kDa (TAF15), transcript variant 1, mRNA [NM_139215]	-1.1181065	0.0072907214
A_33_P3367062	*PIGH*	Homo sapiens phosphatidylinositol glycan anchor biosynthesis, class H (PIGH), mRNA [NM_004569]	-0.5385402	0.011162851
A_23_P140748	*ERMARD*	Homo sapiens ER membrane-associated RNA degradation (ERMARD), transcript variant 1, mRNA [NM_018341]	-1.0716388	0.0065304036
A_23_P215484	*SWT1*	Homo sapiens SWT1 RNA endoribonuclease homolog (S. cerevisiae) (SWT1), transcript variant 1, mRNA [NM_017673]	-0.69060045	0.012525387
A_24_P349560	*NDRG4*	Homo sapiens NDRG family member 4 (NDRG4), transcript variant 3, mRNA [NM_022910]	1.6524006	0.008220364
A_19_P00319646	*CCL26*	Homo sapiens chemokine (C-C motif) ligand 26 (CCL26), mRNA [NM_006072]	-3.118366	0.003354357
A_24_P81965	*EIF4E*	Homo sapiens eukaryotic translation initiation factor 4E (EIF4E), transcript variant 1, mRNA [NM_001968]	-0.5016313	0.014053405
A_33_P3322589	*LINC00673*	Homo sapiens long intergenic non-protein coding RNA 673 (LINC00673), long non-coding RNA [NR_036488]	-1.1791025	0.0062013934
A_33_P6817354	*RAP2A*	Homo sapiens RAP2A, member of RAS oncogene family (RAP2A), mRNA [NM_021033]	-1.1125681	0.0037583082
A_23_P121956	*FANCL*	Homo sapiens Fanconi anemia, complementation group L (FANCL), transcript variant 1, mRNA [NM_001114636]	-0.20269375	0.013549144
A_23_P258164	*FGD5-AS1*	Homo sapiens FGD5 antisense RNA 1 (FGD5-AS1), transcript variant 1, long non-coding RNA [NR_046251]	-0.7696638	0.008541028
A_33_P3410599	*THG1L*	Homo sapiens tRNA-histidine guanylyltransferase 1-like (S. cerevisiae) (THG1L), mRNA [NM_017872]	-1.0539298	0.0061139446
A_23_P74320	*CORT*	Homo sapiens cortistatin (CORT), mRNA [NM_001302]	0.6472346	0.014116709
A_23_P117082	*FAM46A*	Homo sapiens family with sequence similarity 46, member A (FAM46A), mRNA [NM_017633]	-2.1964593	0.0022181747
A_22_P00010567	*SCYL3*	Homo sapiens SCY1-like 3 (S. cerevisiae) (SCYL3), transcript variant 1, mRNA [NM_020423]	-1.408032	0.006991915

A_23_P335428	*HEBP1*	Homo sapiens heme binding protein 1 (HEBP1), mRNA [NM_015987]	-1.0643669	0.0055818916
A_23_P62807	*lnc-NDE1-3*	LNCipedia lincRNA (lnc-NDE1-3), lincRNA [lnc-NDE1-3:1]	1.8836541	0.0025001846
A_24_P361006	*HIBADH*	Homo sapiens 3-hydroxyisobutyrate dehydrogenase (HIBADH), mRNA [NM_152740]	-1.0987853	0.011307727
A_33_P3349259	*SCCPDH*	Homo sapiens saccharopine dehydrogenase (putative) (SCCPDH), mRNA [NM_016002]	-0.7175501	0.007050734
A_22_P00017778	*NDUFA9*	Homo sapiens NADH dehydrogenase (ubiquinone) 1 alpha subcomplex, 9, 39kDa (NDUFA9), mRNA [NM_005002]	-0.3686939	0.014603465
A_33_P3381127	*TCAF1*	Homo sapiens family with sequence similarity 115, member A (FAM115A), transcript variant 1, mRNA [NM_014719]	1.4582081	0.011172963
A_23_P142849	*lnc-ZAP70-1*	LNCipedia lincRNA (lnc-ZAP70-1), lincRNA [lnc-ZAP70-1:1]	0.28896856	0.015061039
A_23_P358709	*FAS*	Homo sapiens Fas cell surface death receptor (FAS), transcript variant 1, mRNA [NM_000043]	-1.327961	0.005659222
A_23_P422981	*RND3*	Homo sapiens Rho family GTPase 3 (RND3), transcript variant 2, mRNA [NM_005168]	-0.2972128	0.008539769
A_23_P42884	*AHRR*	Homo sapiens aryl-hydrocarbon receptor repressor (AHRR), transcript variant 1, mRNA [NM_020731]	-0.3209254	0.009029172
A_21_P0007763	*FBXO36*	Homo sapiens F-box protein 36 (FBXO36), mRNA [NM_174899]	-0.84554774	0.007683834
A_23_P75038	*MRPS24*	Homo sapiens mitochondrial ribosomal protein S24 (MRPS24), mRNA [NM_032014]	-0.4913468	0.01214474
A_21_P0013848	*MAPKAPK5-AS1*	Homo sapiens MAPKAPK5 antisense RNA 1 (MAPKAPK5-AS1), long non-coding RNA [NR_015404]	-0.85616136	0.008046525
A_23_P152655	*DCLRE1A*	Homo sapiens DNA cross-link repair 1A (DCLRE1A), transcript variant 2, mRNA [NM_014881]	-0.52953434	0.014187497
A_23_P47885	*FOXN3*	Homo sapiens forkhead box N3 (FOXN3), transcript variant 2, mRNA [NM_005197]	-0.8146701	0.015334945
A_24_P453544	*ICAM2*	Homo sapiens intercellular adhesion molecule 2 (ICAM2), transcript variant 5, mRNA [NM_000873]	-1.2974463	0.010505778
A_24_P250650	*LRIG3*	Homo sapiens leucine-rich repeats and immunoglobulin-like domains 3 (LRIG3), transcript variant 2, mRNA [NM_153377]	-0.51915306	0.011768655
A_33_P3338186	*RABL2A*	Homo sapiens RAB, member of RAS oncogene family-like 2A (RABL2A), transcript variant 1, mRNA [NM_013412]	-0.9403877	0.0073276176

A_33_P3210399	*HEXDC*	Homo sapiens hexosaminidase (glycosyl hydrolase family 20, catalytic domain) containing (HEXDC), mRNA [NM_173620]	-0.74525785	0.0096329255
A_33_P3215193	*MMP1*	Homo sapiens matrix metallopeptidase 1 (interstitial collagenase) (MMP1), transcript variant 1, mRNA [NM_002421]	2.459429	0.0023561441
A_21_P0014703	*SLC14A1*	Homo sapiens solute carrier family 14 (urea transporter), member 1 (Kidd blood group) (SLC14A1), transcript variant 4, mRNA [NM_001146037]	3.4985151	0.0030069214
A_23_P305140	*NMNAT1*	Homo sapiens nicotinamide nucleotide adenylyltransferase 1 (NMNAT1), transcript variant 3, mRNA [NM_001297779]	-0.6583038	0.009690877
A_23_P94932	*LINC01128*	Homo sapiens long intergenic non-protein coding RNA 1128 (LINC01128), transcript variant 9, long non-coding RNA [NR_047526]	0.58054805	0.016766192
A_33_P3234804	*C10orf32*	Homo sapiens chromosome 10 open reading frame 32 (C10orf32), transcript variant 2, mRNA [NM_144591]	-0.76721597	0.0042509567
A_22_P00014157	*MMADHC*	Homo sapiens methylmalonic aciduria (cobalamin deficiency) cblD type, with homocystinuria (MMADHC), mRNA [NM_015702]	-0.6992233	0.0065866495
A_23_P311087	*CBR4*	Homo sapiens carbonyl reductase 4 (CBR4), mRNA [NM_032783]	-0.7755282	0.009237514
A_33_P3294578	*ZNF281*	Homo sapiens zinc finger protein 281 (ZNF281), transcript variant 1, mRNA [NM_012482]	-1.0333104	0.009794768
A_24_P133253	*FBXW7*	Homo sapiens F-box and WD repeat domain containing 7, E3 ubiquitin protein ligase (FBXW7), transcript variant 1, mRNA [NM_033632]	-1.4770467	0.0035912434
A_32_P150891	*PRKCI*	Homo sapiens protein kinase C, iota (PRKCI), mRNA [NM_002740]	-1.0511715	0.0036402496
A_33_P3331267	*KITLG*	Homo sapiens KIT ligand (KITLG), transcript variant b, mRNA [NM_000899]	-0.49380133	0.012865104
A_33_P3389478	*DIAPH3*	Homo sapiens diaphanous-related formin 3 (DIAPH3), transcript variant 1, mRNA [NM_001042517]	-0.692023	0.016260965
A_24_P754803	*SNAPC3*	small nuclear RNA activating complex, polypeptide 3, 50kDa [Source:HGNC Symbol;Acc:HGNC:11136] [ENST00000380799]	-1.2553607	0.010086547
A_33_P3407524	*C1orf50*	Homo sapiens chromosome 1 open reading frame 50 (C1orf50), transcript variant 1, mRNA [NM_024097]	-1.0182718	0.009018742
A_32_P194821	*SUV39H1*	Homo sapiens suppressor of variegation 3-9 homolog 1 (Drosophila) (SUV39H1), transcript variant 1, mRNA [NM_001282166]	1.276981	0.005734989

A_33_P3402217	*GHR*	Homo sapiens growth hormone receptor (GHR), transcript variant 1, mRNA [NM_000163]	-2.5777264	0.008897745
A_22_P00017805	*RPL21*	Homo sapiens ribosomal protein L21 (RPL21), mRNA [NM_000982]	-0.430062	0.008311857
A_23_P215525	*lnc-ZBTB25-1*	Homo sapiens cDNA FLJ40004 fis, clone STOMA2004194. [AK097323]	-0.7257447	0.0056666723
A_23_P134454	*NUPR1*	Homo sapiens nuclear protein, transcriptional regulator, 1 (NUPR1), transcript variant 1, mRNA [NM_001042483]	-0.97041196	0.00584425
A_21_P0000611	*OSBPL3*	Homo sapiens oxysterol binding protein-like 3 (OSBPL3), transcript variant 1, mRNA [NM_015550]	-0.9090297	0.009003274
A_21_P0000325	*CAV1*	Homo sapiens caveolin 1, caveolae protein, 22kDa (CAV1), transcript variant 1, mRNA [NM_001753]	-0.8712275	0.005255513
A_24_P411899	*FLJ20021*	Homo sapiens uncharacterized LOC90024 (FLJ20021), long non-coding RNA [NR_033874]	-0.9832077	0.004804688
A_23_P208143	*SNORA37*	Homo sapiens small nucleolar RNA, H/ACA box 37 (SNORA37), small nucleolar RNA [NR_002970]	-0.32660463	0.012806621
A_23_P500892	*RNF19A*	Homo sapiens ring finger protein 19A, RBR E3 ubiquitin protein ligase (RNF19A), transcript variant 1, mRNA [NM_183419]	-0.75107926	0.013079763
A_23_P76914	*ZNF397*	Homo sapiens zinc finger protein 397 (ZNF397), transcript variant 1, mRNA [NM_001135178]	-1.1595747	0.011747539
A_22_P00024064	*TUB*	Homo sapiens tubby bipartite transcription factor (TUB), transcript variant 1, mRNA [NM_003320]	-1.1309749	0.012477395
A_24_P89509	*SIX1*	Homo sapiens SIX homeobox 1 (SIX1), mRNA [NM_005982]	1.1573455	0.010035188
A_33_P3404097	*OGFR-AS1*	Homo sapiens OGFR antisense RNA 1 (OGFR-AS1), long non-coding RNA [NR_102430]	-1.2044326	0.009794768
A_23_P45524	*TRMT11*	Homo sapiens tRNA methyltransferase 11 homolog (S. cerevisiae) (TRMT11), mRNA [NM_001031712]	-0.68529665	0.006199031
A_33_P3407945	*PGM5P2*	Homo sapiens phosphoglucomutase 5 pseudogene 2 (PGM5P2), non-coding RNA [NR_002836]	-0.16384082	0.008956738
A_33_P3308158	*NGFRAP1*	Homo sapiens nerve growth factor receptor (TNFRSF16) associated protein 1 (NGFRAP1), transcript variant 3, mRNA [NM_014380]	-0.787328	0.0067145773
A_24_P185854	*TMEM19*	Homo sapiens transmembrane protein 19 (TMEM19), mRNA [NM_018279]	-0.8231295	0.0057522994
A_23_P411335	*DMD*	Homo sapiens dystrophin (DMD), transcript variant Dp427p2, mRNA [NM_004010]	-2.6824906	0.003986463

A_33_P3365524	*lnc-AC073343.1-1*	LNCipedia lincRNA (lnc-AC073343.1-1), lincRNA [lnc-AC073343.1-1:3]	0.62141895	0.0072648157
A_33_P3415663	*SGOL2*	Homo sapiens shugoshin-like 2 (S. pombe) (SGOL2), transcript variant 1, mRNA [NM_152524]	-0.5521644	0.007122443
A_22_P00025671	*SETD3*	Homo sapiens SET domain containing 3 (SETD3), transcript variant 2, mRNA [NM_199123]	-0.7561339	0.0064965826
A_23_P89199	*MBLAC2*	Homo sapiens metallo-beta-lactamase domain containing 2 (MBLAC2), mRNA [NM_203406]	-0.6685293	0.005822303
A_23_P425681	*IGFBP7*	insulin-like growth factor binding protein 7 [Source:HGNC Symbol;Acc:HGNC:5476] [ENST00000295666]	-2.2093081	0.0037705272
A_33_P3240532	*COPS3*	Homo sapiens COP9 signalosome subunit 3 (COPS3), transcript variant 1, mRNA [NM_003653]	-0.60876465	0.009039211
A_21_P0013475	*CCK*	Homo sapiens cholecystokinin (CCK), transcript variant 1, mRNA [NM_000729]	5.0834556	0.003363001
A_23_P15798	*RGL1*	Homo sapiens ral guanine nucleotide dissociation stimulator-like 1 (RGL1), transcript variant 1, mRNA [NM_015149]	-1.2309132	0.0035734554
A_23_P103905	*KRTAP4-12*	Homo sapiens keratin associated protein 4-12 (KRTAP4-12), mRNA [NM_031854]	1.083347	0.013369118
A_22_P00004633	*PARVA*	Homo sapiens parvin, alpha (PARVA), mRNA [NM_018222]	-1.089829	0.0061428803
A_33_P3257993	*UFC1*	Homo sapiens ubiquitin-fold modifier conjugating enzyme 1 (UFC1), mRNA [NM_016406]	-0.5719876	0.013411169
A_33_P3319401	*RNF125*	Homo sapiens ring finger protein 125, E3 ubiquitin protein ligase (RNF125), mRNA [NM_017831]	1.0841131	0.009450744
A_22_P00018322	*HSPB8*	Homo sapiens heat shock 22kDa protein 8 (HSPB8), mRNA [NM_014365]	0.5433244	0.007282378
A_22_P00000848	*ARHGAP21*	Rho GTPase activating protein 21 [Source:HGNC Symbol;Acc:HGNC:23725] [ENST00000476499]	-2.0132003	0.004213048
A_23_P13604	*SSPO*	Homo sapiens SCO-spondin (SSPO), mRNA [NM_198455]	0.5001347	0.015544047
A_23_P34396	*ZBED3-AS1*	Homo sapiens ZBED3 antisense RNA 1 (ZBED3-AS1), long non-coding RNA [NR_024398]	-0.26490083	0.014717759
A_32_P107876	*PEBP1*	Homo sapiens phosphatidylethanolamine binding protein 1 (PEBP1), mRNA [NM_002567]	-0.49383163	0.003230945
A_23_P110957	*RSRP1*	Homo sapiens arginine/serine-rich protein 1 (RSRP1), mRNA [NM_020317]	-1.3299191	0.005879462
A_23_P21162	*FRAS1*	Homo sapiens Fraser extracellular matrix complex subunit 1 (FRAS1), transcript variant 1, mRNA [NM_025074]	-2.5247107	0.004867804

A_24_P122682	*FOXF2*	Homo sapiens forkhead box F2 (FOXF2), mRNA [NM_001452]	-2.3897967	0.002419416
A_23_P349083	*TCTEX1D2*	Homo sapiens Tctex1 domain containing 2 (TCTEX1D2), mRNA [NM_152773]	-0.97888875	0.012626196
A_23_P379327	*RAB4A*	Homo sapiens RAB4A, member RAS oncogene family (RAB4A), transcript variant 1, mRNA [NM_004578]	-0.7715253	0.0062257075
A_24_P941051	*FCHO2*	Homo sapiens FCH domain only 2 (FCHO2), transcript variant 1, mRNA [NM_138782]	-1.3544269	0.0036402496
A_33_P3304252	*FAM63B*	Homo sapiens family with sequence similarity 63, member B (FAM63B), transcript variant 1, mRNA [NM_001040450]	-1.2542422	0.0069511216
A_22_P00009979	*CSTF2T*	Homo sapiens cleavage stimulation factor, 3' pre-RNA, subunit 2, 64kDa, tau variant (CSTF2T), mRNA [NM_015235]	-0.6595669	0.00955837
A_24_P50368	*MRPL32*	Homo sapiens mitochondrial ribosomal protein L32 (MRPL32), mRNA [NM_031903]	-0.51035684	0.00842156
A_23_P71379	*LOC100129781*	PREDICTED: Homo sapiens uncharacterized LOC100129781 (LOC100129781), transcript variant X2, ncRNA [XR_109259]	-1.6918076	0.0049014823
A_24_P49190	*BLID*	Homo sapiens BH3-like motif containing, cell death inducer (BLID), mRNA [NM_001001786]	-2.221666	0.0044600195
A_23_P18598	*PSCA*	Homo sapiens prostate stem cell antigen (PSCA), transcript variant 1, mRNA [NM_005672]	0.5943172	0.015820691
A_23_P502797	*C17orf58*	Homo sapiens chromosome 17 open reading frame 58 (C17orf58), transcript variant 1, mRNA [NM_181655]	-0.9668524	0.006395745
A_23_P166899	*PI4K2B*	Homo sapiens phosphatidylinositol 4-kinase type 2 beta (PI4K2B), mRNA [NM_018323]	-0.58394456	0.0044240346
A_23_P212800	*WDFY1*	Homo sapiens WD repeat and FYVE domain containing 1 (WDFY1), mRNA [NM_020830]	-1.0652893	0.0040229773
A_33_P3475737	*DNAJB11*	Homo sapiens DnaJ (Hsp40) homolog, subfamily B, member 11 (DNAJB11), mRNA [NM_016306]	-1.0214636	0.0075578676
A_19_P00802936	*FGF5*	Homo sapiens fibroblast growth factor 5 (FGF5), transcript variant 1, mRNA [NM_004464]	0.9274585	0.003542592
A_21_P0001290	*ZBTB8A*	Homo sapiens zinc finger and BTB domain containing 8A (ZBTB8A), transcript variant 1, mRNA [NM_001040441]	-1.0460641	0.009225704
A_23_P116387	*BRK1*	Homo sapiens BRICK1, SCAR/WAVE actin-nucleating complex subunit (BRK1), mRNA [NM_018462]	-0.8971596	0.0097600445
A_32_P382427	*LINC00853*	Homo sapiens long intergenic non-protein coding RNA 853 (LINC00853), long non-coding RNA [NR_047498]	-1.1894425	0.013624135

A_23_P133375	*INCENP*	Homo sapiens inner centromere protein antigens 135/155kDa (INCENP), transcript variant 1, mRNA [NM_001040694]	-0.8915999	0.009036186
A_33_P3276703	*PSMG3-AS1*	PSMG3 antisense RNA 1 (head to head) [Source:HGNC Symbol;Acc:HGNC:22230] [ENST00000533935]	-0.86403847	0.0074523045
A_24_P384569	*SLC25A46*	Homo sapiens solute carrier family 25, member 46 (SLC25A46), transcript variant 1, mRNA [NM_138773]	-0.8231187	0.0166364
A_33_P3397716	*VGF*	Homo sapiens VGF nerve growth factor inducible (VGF), mRNA [NM_003378]	1.2473025	0.0072350414
A_33_P3382944	*EWSR1*	Homo sapiens EWS RNA-binding protein 1 (EWSR1), transcript variant 5, mRNA [NM_001163287]	0.7734138	0.008289568
A_23_P122464	*lnc-GGCT-1*	Homo sapiens cDNA FLJ39921 fis, clone SPLEN2020417. [AK097240]	-1.6696099	0.01012932
A_23_P144684	*YJEFN3*	Homo sapiens YjeF N-terminal domain containing 3 (YJEFN3), transcript variant 1, mRNA [NM_198537]	0.97864175	0.012576259
A_22_P00008476	*ZSCAN9*	Homo sapiens zinc finger and SCAN domain containing 9 (ZSCAN9), transcript variant 2, mRNA [NM_006299]	-0.7377603	0.005073928
A_21_P0012564	*ANKRD32*	Homo sapiens ankyrin repeat domain 32 (ANKRD32), mRNA [NM_032290]	-0.3711901	0.013857311
A_33_P3209960	*XLOC_l2_010508*	BROAD Institute lincRNA (XLOC_l2_010508), lincRNA [TCONS_l2_00020322]	-0.66779923	0.008488166
A_33_P3663705	*LSM5*	Homo sapiens LSM5 homolog, U6 small nuclear RNA associated (S. cerevisiae) (LSM5), transcript variant 1, mRNA [NM_012322]	-0.6314807	0.0057647754
A_22_P00002062	*RASGRP2*	Homo sapiens RAS guanyl releasing protein 2 (calcium and DAG-regulated) (RASGRP2), transcript variant 2, mRNA [NM_153819]	-0.67130816	0.0058573913
A_23_P88602	*CROCC*	Homo sapiens ciliary rootlet coiled-coil, rootletin (CROCC), mRNA [NM_014675]	-0.83705187	0.015769914
A_23_P26674	*lnc-BLID-1*	LNCipedia lincRNA (lnc-BLID-1), lincRNA [lnc-BLID-1:15]	-1.8698561	0.010735533
A_33_P3388482	*MEIS2*	Homo sapiens Meis homeobox 2 (MEIS2), transcript variant a, mRNA [NM_170677]	-1.2790316	0.0039356006
A_32_P150735	*KIAA0430*	Homo sapiens KIAA0430 (KIAA0430), transcript variant 1, mRNA [NM_014647]	-1.2712281	0.0033148525
A_23_P70991	*TMEM120B*	Homo sapiens transmembrane protein 120B (TMEM120B), mRNA [NM_001080825]	1.0708091	0.012773474
A_33_P3303104	*AIMP2*	Homo sapiens aminoacyl tRNA synthetase complex-interacting multifunctional protein 2 (AIMP2), mRNA [NM_006303]	-1.1943915	0.005470758
A_32_P49616	*MYNN*	Homo sapiens myoneurin (MYNN), transcript variant 3, mRNA [NM_001185119]	-0.5413709	0.005092817

A_23_P77661	*KIAA0513*	Homo sapiens KIAA0513 (KIAA0513), transcript variant 2, mRNA [NM_014732]	-1.5309911	0.005581195
A_19_P00321911	*EEF1B2*	Homo sapiens eukaryotic translation elongation factor 1 beta 2 (EEF1B2), transcript variant 1, mRNA [NM_001959]	-0.44337535	0.008236777
A_33_P3365087	*ZNF720*	Homo sapiens zinc finger protein 720 (ZNF720), mRNA [NM_001130913]	-0.6118747	0.010244809
A_23_P345118	*IPW*	Homo sapiens imprinted in Prader-Willi syndrome (non-protein coding) (IPW), long non-coding RNA [NR_023915]	0.5625654	0.0065241847
A_33_P3421626	*PRR20B*	Homo sapiens proline rich 20B (PRR20B), mRNA [NM_001130404]	0.6946221	0.010976941
A_33_P3265872	*PIM1*	Homo sapiens Pim-1 proto-oncogene, serine/threonine kinase (PIM1), transcript variant 1, mRNA [NM_002648]	-1.7259538	0.0033868244
A_33_P3268378	*KIAA1147*	Homo sapiens KIAA1147 (KIAA1147), mRNA [NM_001080392]	-1.1310868	0.005098191
A_33_P3416350	*LOC101927497*	Homo sapiens uncharacterized LOC101927497 (LOC101927497), transcript variant 1, long non-coding RNA [NR_110086]	-2.583572	0.00461665
A_33_P3267532	*FAM167A*	Homo sapiens family with sequence similarity 167, member A (FAM167A), mRNA [NM_053279]	1.5732505	0.011732056
A_22_P00015288	*ITSN2*	Homo sapiens intersectin 2 (ITSN2), transcript variant 2, mRNA [NM_147152]	-0.52525485	0.007974485
A_23_P43226	*KCNJ15*	Homo sapiens potassium channel, inwardly rectifying subfamily J, member 15 (KCNJ15), transcript variant 1, mRNA [NM_170736]	1.0965444	0.01264829
A_23_P3994	*lnc-SPATC1-1*	PREDICTED: Homo sapiens sphingomyelin phosphodiesterase 5 (SMPD5), mRNA [XM_001714032]	1.1682324	0.0048181154
A_33_P3269678	*KCTD9*	Homo sapiens potassium channel tetramerization domain containing 9 (KCTD9), mRNA [NM_017634]	-0.6802907	0.0048717977
A_23_P214739	*TMEM256*	Homo sapiens transmembrane protein 256 (TMEM256), mRNA [NM_152766]	-0.578155	0.006485141
A_24_P889720	*MIR4435-1HG*	Homo sapiens MIR4435-1 host gene (non-protein coding) (MIR4435-1HG), transcript variant 1, long non-coding RNA [NR_015395]	-1.0611924	0.011307727
A_23_P206532	*FBXL4*	Homo sapiens F-box and leucine-rich repeat protein 4 (FBXL4), transcript variant 1, mRNA [NM_012160]	-0.7939897	0.0053870557
A_23_P207020	*UBC*	Homo sapiens ubiquitin C (UBC), mRNA [NM_021009]	0.18045978	0.010220027
A_24_P547010	*PHKB*	Homo sapiens phosphorylase kinase, beta (PHKB), transcript variant 2, mRNA [NM_001031835]	-1.1191711	0.007196166

A_24_P139191	*WDR45B*	Homo sapiens WD repeat domain 45B (WDR45B), mRNA [NM_019613]	-0.72342086	0.011368536
A_33_P3385561	*ITCH*	Homo sapiens itchy E3 ubiquitin protein ligase (ITCH), transcript variant 2, mRNA [NM_031483]	-0.5399589	0.012093684
A_24_P407323	*ING4*	Homo sapiens inhibitor of growth family, member 4 (ING4), transcript variant 1, mRNA [NM_016162]	-0.9754452	0.01716122
A_23_P118002	*PPM1A*	Homo sapiens protein phosphatase, Mg2+/Mn2+ dependent, 1A (PPM1A), transcript variant 2, mRNA [NM_177951]	-0.5058107	0.0044813035
A_33_P3261293	*CCDC74B*	Homo sapiens coiled-coil domain containing 74B (CCDC74B), transcript variant 1, mRNA [NM_207310]	-1.1444364	0.01368361
A_23_P15450	*UQCRC2*	Homo sapiens ubiquinol-cytochrome c reductase core protein II (UQCRC2), mRNA [NM_003366]	-0.78453946	0.006020368
A_23_P140511	*DKK3*	Homo sapiens dickkopf WNT signaling pathway inhibitor 3 (DKK3), transcript variant 1, mRNA [NM_015881]	0.88345695	0.013730757
A_24_P321581	*TMEM100*	Homo sapiens transmembrane protein 100 (TMEM100), transcript variant 2, mRNA [NM_018286]	-1.2976213	0.009711265
A_23_P254733	*CLPX*	Homo sapiens caseinolytic mitochondrial matrix peptidase chaperone subunit (CLPX), mRNA [NM_006660]	-0.81547993	0.016256094
A_33_P3242798	*SLC38A4*	Homo sapiens solute carrier family 38, member 4 (SLC38A4), transcript variant 1, mRNA [NM_018018]	1.7030609	0.0039633308
A_33_P3389638	*CENPU*	Homo sapiens centromere protein U (CENPU), transcript variant 1, mRNA [NM_024629]	-0.42915013	0.011821381
A_23_P55251	*CPS1*	Homo sapiens carbamoyl-phosphate synthase 1, mitochondrial (CPS1), transcript variant 2, mRNA [NM_001875]	0.20097476	0.011258094
A_19_P00325336	*NOP14*	Homo sapiens NOP14 nucleolar protein (NOP14), transcript variant 1, mRNA [NM_001291978]	-0.50424737	0.010134365
A_33_P3229196	*ITGA3*	Homo sapiens integrin, alpha 3 (antigen CD49C, alpha 3 subunit of VLA-3 receptor) (ITGA3), transcript variant a, mRNA [NM_002204]	1.3799175	0.0054365364
A_23_P35576	*CD151*	Homo sapiens CD151 molecule (Raph blood group) (CD151), transcript variant 1, mRNA [NM_004357]	0.2597256	0.0044813035
A_32_P162374	*ZNF518A*	Homo sapiens zinc finger protein 518A (ZNF518A), transcript variant 2, mRNA [NM_014803]	-0.60172117	0.0057506533

A_23_P128543	*KCNK15*	Homo sapiens potassium channel, two pore domain subfamily K, member 15 (KCNK15), mRNA [NM_022358]	0.9257021	0.0072384304
A_33_P3366484	*C18orf54*	Homo sapiens chromosome 18 open reading frame 54 (C18orf54), transcript variant 3, mRNA [NM_173529]	-0.6078247	0.013302637
A_24_P291973	*MED4*	Homo sapiens mediator complex subunit 4 (MED4), transcript variant 1, mRNA [NM_014166]	-1.1880836	0.0029071842
A_24_P406132	*HIBCH*	Homo sapiens 3-hydroxyisobutyryl-CoA hydrolase (HIBCH), transcript variant 2, mRNA [NM_198047]	-0.74973464	0.010769289
A_24_P285623	*MAPK13*	Homo sapiens mitogen-activated protein kinase 13 (MAPK13), transcript variant 1, mRNA [NM_002754]	1.704202	0.0049917083
A_23_P29836	*KBTBD4*	Homo sapiens kelch repeat and BTB (POZ) domain containing 4 (KBTBD4), transcript variant 2, mRNA [NM_016506]	-0.6262533	0.010874207
A_33_P3345389	*DGUOK*	Homo sapiens deoxyguanosine kinase (DGUOK), transcript variant 1, mRNA [NM_080916]	-0.7737949	0.008060008
A_23_P107855	*TMEM42*	transmembrane protein 42 [Source:HGNC Symbol;Acc:HGNC:28444] [ENST00000477126]	-1.4470854	0.0038144474
A_33_P3353051	*C20orf194*	Homo sapiens chromosome 20 open reading frame 194 (C20orf194), mRNA [NM_001009984]	-0.7576735	0.016320607
A_33_P3300179	*FIZ1*	Homo sapiens FLT3-interacting zinc finger 1 (FIZ1), mRNA [NM_032836]	1.2779144	0.006466261
A_23_P77859	*C6orf48*	Homo sapiens chromosome 6 open reading frame 48 (C6orf48), transcript variant 4, mRNA [NM_001287483]	-1.3523633	0.0054365364
A_33_P3354434	*TMEM88*	Homo sapiens transmembrane protein 88 (TMEM88), mRNA [NM_203411]	1.7699866	0.0033828178
A_23_P27584	*HCLS1*	Homo sapiens hematopoietic cell-specific Lyn substrate 1 (HCLS1), transcript variant 1, mRNA [NM_005335]	-2.427445	0.004723954
A_33_P6812319	*TRIM41*	Homo sapiens tripartite motif containing 41 (TRIM41), transcript variant 1, mRNA [NM_033549]	-0.9010706	0.0047719665
A_22_P00011026	*MYADM*	Homo sapiens myeloid-associated differentiation marker (MYADM), transcript variant 1, mRNA [NM_001020818]	-0.84741753	0.007099076
A_23_P318904	*NDUFV3*	NADH dehydrogenase (ubiquinone) flavoprotein 3, 10kDa [Source:HGNC Symbol;Acc:HGNC:7719] [ENST00000460259]	-0.49654588	0.014571856
A_23_P374689	*lnc-NT5DC2-1*	LNCipedia lincRNA (lnc-NT5DC2-1), lincRNA [lnc-NT5DC2-1:1]	-0.84378266	0.0067730513

A_33_P3263890	*SERTAD4*	Homo sapiens SERTA domain containing 4 (SERTAD4), mRNA [NM_019605]	-1.645627	0.005690581
A_23_P143190	*GAD1*	Homo sapiens glutamate decarboxylase 1 (brain, 67kDa) (GAD1), transcript variant GAD67, mRNA [NM_000817]	-1.7572497	0.0066194455
A_33_P3219475	*PRRX1*	Homo sapiens paired related homeobox 1 (PRRX1), transcript variant pmx-1a, mRNA [NM_006902]	-0.6270047	0.00590614
A_33_P3362521	*MYBL2*	Homo sapiens v-myb avian myeloblastosis viral oncogene homolog-like 2 (MYBL2), transcript variant 1, mRNA [NM_002466]	0.8243395	0.013354078
A_19_P00803507	*MSANTD3-TMEFF1*	Homo sapiens MSANTD3-TMEFF1 readthrough (MSANTD3-TMEFF1), mRNA [NM_001198812]	-1.1305478	0.006812377
A_21_P0002061	*CCDC174*	Homo sapiens coiled-coil domain containing 174 (CCDC174), mRNA [NM_016474]	-1477021	0.00519239
A_33_P3326617	*LOC101929666*	PREDICTED: Homo sapiens uncharacterized LOC101929666 (LOC101929666), ncRNA [XR_247062]	-0.6987389	0.008340001
A_24_P941708	*LOC101927577*	Homo sapiens uncharacterized LOC101927577 (LOC101927577), transcript variant 1, long non-coding RNA [NR_110264]	0.646552	0.0160096
A_19_P00324399	*FAHD1*	Homo sapiens fumarylacetoacetate hydrolase domain containing 1 (FAHD1), transcript variant 1, mRNA [NM_001018104]	-0.97209173	0.009081763
A_33_P3258141	*RUFY2*	Homo sapiens RUN and FYVE domain containing 2 (RUFY2), transcript variant 2, mRNA [NM_001042417]	-0.5108226	0.009765222
A_19_P00321332	*LINC01481*	Homo sapiens long intergenic non-protein coding RNA 1481 (LINC01481), transcript variant 1, long non-coding RNA [NR_120459]	-0.62510026	0.007205146
A_23_P132784	*CHUK*	Homo sapiens conserved helix-loop-helix ubiquitous kinase (CHUK), mRNA [NM_001278]	-0.5971644	0.0098649
A_33_P3390335	*NEAT1*	Homo sapiens nuclear paraspeckle assembly transcript 1 (non-protein coding) (NEAT1), long non-coding RNA [NR_028272]	-0.6511395	0.0075020376
A_33_P3320393	*FXR1*	Homo sapiens fragile X mental retardation, autosomal homolog 1 (FXR1), transcript variant 3, mRNA [NM_001013439]	-1.0772848	0.0074182227
A_24_P57700	*RSU1*	Homo sapiens Ras suppressor protein 1 (RSU1), transcript variant 1, mRNA [NM_012425]	-0.82107186	0.0037458793
A_23_P209449	*MRPS16*	Homo sapiens mitochondrial ribosomal protein S16 (MRPS16), mRNA [NM_016065]	-0.74092454	0.007794005
A_21_P0007250	*ZHX3*	Homo sapiens zinc fingers and homeoboxes 3 (ZHX3), mRNA [NM_015035]	-0.9368577	0.007929221
A_24_P321411	*FZD7*	Homo sapiens frizzled class receptor 7 (FZD7), mRNA [NM_003507]	-0.8177957	0.009968179

A_32_P116271	*RAB30-AS1*	Homo sapiens RAB30 antisense RNA 1 (head to head) (RAB30-AS1), long non-coding RNA [NR_038903]	-0.45829433	0.015154758
A_24_P230057	*PRKRIR*	Homo sapiens protein-kinase, interferon-inducible double stranded RNA dependent inhibitor, repressor of (P58 repressor) (PRKRIR), mRNA [NM_004705]	-0.41198105	0.0099648675
A_22_P00005210	*WDFY3-AS2*	Homo sapiens WDFY3 antisense RNA 2 (WDFY3-AS2), long non-coding RNA [NR_015359]	-0.9046833	0.00942855
A_23_P105392	*MEG9*	Homo sapiens maternally expressed 9 (non-protein coding) (MEG9), long non-coding RNA [NR_047664]	1.8553559	0.0057806885
A_23_P411188	*CA12*	Homo sapiens carbonic anhydrase XII (CA12), transcript variant 1, mRNA [NM_001218]	-1.1657231	0.00477571
A_23_P58466	*CCT2*	Homo sapiens chaperonin containing TCP1, subunit 2 (beta) (CCT2), transcript variant 1, mRNA [NM_006431]	-0.54227805	0.01011679
A_33_P3290082	*CHRNA10*	Homo sapiens cholinergic receptor, nicotinic, alpha 10 (neuronal) (CHRNA10), transcript variant 1, mRNA [NM_020402]	-0.58492327	0.012479078
A_33_P3251492	*SMN1*	Homo sapiens survival of motor neuron 1, telomeric (SMN1), transcript variant d, mRNA [NM_000344]	-0.11454175	0.007111782
A_23_P1641	*HPS4*	Homo sapiens Hermansky-Pudlak syndrome 4 (HPS4), transcript variant 1, mRNA [NM_022081]	0.72648716	0.0069557577
A_33_P3334292	*LOC100133669*	Homo sapiens uncharacterized LOC100133669 (LOC100133669), long non-coding RNA [NR_026913]	1.5182649	0.005787274
A_23_P60499	*RCE1*	Homo sapiens Ras converting CAAX endopeptidase 1 (RCE1), transcript variant 1, mRNA [NM_005133]	1.1190395	0.0050508454
A_23_P107644	*PPIAL4G*	peptidylprolyl isomerase A (cyclophilin A)-like 4G [Source:HGNC Symbol;Acc:HGNC:33996] [ENST00000419275]	-0.91349643	0.005512448
A_23_P314115	*ZNF462*	Homo sapiens zinc finger protein 462 (ZNF462), mRNA [NM_021224]	-0.7157314	0.009566855
A_33_P3277943	*SNRPD1*	Homo sapiens small nuclear ribonucleoprotein D1 polypeptide 16kDa (SNRPD1), transcript variant 1, mRNA [NM_006938]	-0.2790878	0.008471989
A_22_P00013451	*BMI1*	Homo sapiens BMI1 proto-oncogene, polycomb ring finger (BMI1), mRNA [NM_005180]	-0.6844933	0.006020368
A_33_P3373469	*CNOT6L*	Homo sapiens CCR4-NOT transcription complex, subunit 6-like (CNOT6L), transcript variant 1, mRNA [NM_001286790]	-1.126461	0.005612091

A_33_P3281867	*lnc-RP11-295K3.1.1-1*	AGENCOURT_10261047 NIH_MGC_107 Homo sapiens cDNA clone IMAGE:6586397 5', mRNA sequence [BU558156]	0.60491943	0.016435314
A_23_P6398	*PSMD2*	Homo sapiens proteasome (prosome, macropain) 26S subunit, non-ATPase, 2 (PSMD2), transcript variant 1, mRNA [NM_002808]	-0.24854377	0.0046008946
A_24_P11131	*AP1B1*	Homo sapiens adaptor-related protein complex 1, beta 1 subunit (AP1B1), transcript variant 1, mRNA [NM_001127]	1.623914	0.007887163
A_23_P76557	*SNORD35B*	Homo sapiens small nucleolar RNA, C/D box 35B (SNORD35B), small nucleolar RNA [NR_001285]	0.7821721	0.0141507
A_32_P12580	*SRRM1*	Homo sapiens serine/arginine repetitive matrix 1 (SRRM1), transcript variant 2, mRNA [NM_005839]	-0.3286062	0.016465655
A_21_P0000684	*NDUFA12*	Homo sapiens NADH dehydrogenase (ubiquinone) 1 alpha subcomplex, 12 (NDUFA12), transcript variant 1, mRNA [NM_018838]	-0.8467915	0.009237514
A_24_P329924	*SMAD2*	Homo sapiens SMAD family member 2 (SMAD2), transcript variant 2, mRNA [NM_001003652]	-0.67731416	0.01273111
A_33_P3222341	*LOC100506548*	Homo sapiens uncharacterized LOC100506548 (LOC100506548), long non-coding RNA [NR_037665]	-0.47317237	0.0054365364
A_23_P127676	*SIK3*	Homo sapiens SIK family kinase 3 (SIK3), transcript variant 1, mRNA [NM_025164]	-1.1064909	0.005057235
A_33_P3353672	*PITPNC1*	Homo sapiens phosphatidylinositol transfer protein, cytoplasmic 1 (PITPNC1), transcript variant 1, mRNA [NM_012417]	1.3802242	0.008106991
A_24_P183150	*CTR9*	Homo sapiens CTR9, Paf1/RNA polymerase II complex component (CTR9), mRNA [NM_014633]	-0.6609687	0.009762497
A_33_P3239195	*RAB28*	Homo sapiens RAB28, member RAS oncogene family (RAB28), transcript variant 2, mRNA [NM_004249]	-0.8271014	0.007797447
A_23_P64173	*CXCL3*	Homo sapiens chemokine (C-X-C motif) ligand 3 (CXCL3), mRNA [NM_002090]	3.261024	0.0039856653
A_33_P3302015	*CARD16*	Homo sapiens caspase recruitment domain family, member 16 (CARD16), transcript variant 1, mRNA [NM_001017534]	3.0371008	0.004034385
A_33_P3233150	*lnc-BTBD19-1*	LNCipedia lincRNA (lnc-BTBD19-1), lincRNA [lnc-BTBD19-1:1]	-0.7237606	0.0066411127
A_23_P137689	*ADAMTSL1*	Homo sapiens ADAMTS-like 1 (ADAMTSL1), transcript variant 4, mRNA [NM_001040272]	-0.7702471	0.007900263
A_32_P153725	*ZSWIM4*	Homo sapiens zinc finger, SWIM-type containing 4 (ZSWIM4), mRNA [NM_023072]	1.2519617	0.007740612

A_24_P122337	*OLFML2B*	Homo sapiens olfactomedin-like 2B (OLFML2B), transcript variant 2, mRNA [NM_015441]	0.7158084	0.003365343
A_24_P382187	*KIAA1033*	Homo sapiens KIAA1033 (KIAA1033), transcript variant 2, mRNA [NM_015275]	-0.8791585	0.0070447545
A_21_P0000103	*SYTL4*	Homo sapiens synaptotagmin-like 4 (SYTL4), transcript variant 1, mRNA [NM_080737]	-0.6656083	0.014394993
A_22_P00015672	*IGFBP4*	Homo sapiens insulin-like growth factor binding protein 4 (IGFBP4), mRNA [NM_001552]	1.0382006	0.0097552175
A_23_P138975	*MTRNR2L3*	Homo sapiens MT-RNR2-like 3 (MTRNR2L3), mRNA [NM_001190472]	-0.97540236	0.017113892
A_21_P0013300	*RBM7*	Homo sapiens RNA binding motif protein 7 (RBM7), transcript variant 2, mRNA [NM_016090]	-0.85203403	0.00180587
A_24_P127928	*GRIN3B*	Homo sapiens glutamate receptor, ionotropic, N-methyl-D-aspartate 3B (GRIN3B), mRNA [NM_138690]	-1.2068268	0.012387412
A_23_P404730	*RAB3GAP2*	Homo sapiens RAB3 GTPase activating protein subunit 2 (non-catalytic) (RAB3GAP2), mRNA [NM_012414]	-0.65903187	0.007264689
A_23_P127565	*LOC101927752*	PREDICTED: Homo sapiens uncharacterized LOC101927752 (LOC101927752), ncRNA [XR_245324]	-1.0668203	0.010047998
A_33_P3278435	*ARHGAP33*	Homo sapiens Rho GTPase activating protein 33 (ARHGAP33), transcript variant 1, mRNA [NM_052948]	1.0611684	0.008864373
A_23_P75255	*LAYN*	Homo sapiens layilin (LAYN), transcript variant 2, mRNA [NM_178834]	-0.76412886	0.009576729
A_24_P130936	*COA4*	Homo sapiens cytochrome c oxidase assembly factor 4 homolog (S. cerevisiae) (COA4), mRNA [NM_016565]	-0.83891153	0.0046155252
A_24_P366457	*VPS26A*	Homo sapiens vacuolar protein sorting 26 homolog A (S. pombe) (VPS26A), transcript variant 1, mRNA [NM_004896]	-0.7522471	0.0035555444
A_32_P52153	*DDX3Y*	Homo sapiens DEAD (Asp-Glu-Ala-Asp) box helicase 3, Y-linked (DDX3Y), transcript variant 2, mRNA [NM_004660]	-0.59988856	0.014104654
A_23_P68155	*UNC5B-AS1*	Homo sapiens UNC5B antisense RNA 1 (UNC5B-AS1), long non-coding RNA [NR_038453]	-1.3516217	0.012936874
A_23_P367899	*lnc-UQCRFS1-9*	LNCipedia lincRNA (lnc-UQCRFS1-9), lincRNA [lnc-UQCRFS1-9:23]	-0.8254065	0.016192868
A_23_P78685	*IFIH1*	Homo sapiens interferon induced with helicase C domain 1 (IFIH1), mRNA [NM_022168]	-1.2314595	0.0137527995
A_24_P835500	*EPOR*	Homo sapiens erythropoietin receptor (EPOR), transcript variant 1, mRNA [NM_000121]	0.89720535	0.0083446065

A_33_P3382595	*FARSA*	Homo sapiens phenylalanyl-tRNA synthetase, alpha subunit (FARSA), mRNA [NM_004461]	0.96953917	0.013167504
A_22_P00010734	*ITPRIPL2*	Homo sapiens inositol 1,4,5-trisphosphate receptor interacting protein-like 2 (ITPRIPL2), transcript variant 1, mRNA [NM_001034841]	-0.72167706	0.009334625
A_23_P53866	*RN7SK*	Homo sapiens RNA, 7SK small nuclear (RN7SK), small nuclear RNA [NR_001445]	0.2310395	0.009507545
A_23_P216468	*lnc-NIF3L1-1*	DA371222 BRTHA2 Homo sapiens cDNA clone BRTHA2001026 5', mRNA sequence [DA371222]	0.5508765	0.007549738
A_33_P3546070	*COG6*	Homo sapiens component of oligomeric golgi complex 6 (COG6), transcript variant 1, mRNA [NM_020751]	-0.78242725	0.006818124
A_32_P180958	*SLC1A1*	Homo sapiens solute carrier family 1 (neuronal/epithelial high affinity glutamate transporter, system Xag), member 1 (SLC1A1), mRNA [NM_004170]	-1.2690704	0.007078901
A_23_P109774	*PHF20L1*	Homo sapiens PHD finger protein 20-like 1 (PHF20L1), transcript variant 1, mRNA [NM_016018]	-0.884139	0.010384165
A_22_P00006358	*PCYOX1*	Homo sapiens prenylcysteine oxidase 1 (PCYOX1), mRNA [NM_016297]	-1.0267476	0.0085387705
A_24_P15062	*ZBTB11*	Homo sapiens zinc finger and BTB domain containing 11 (ZBTB11), mRNA [NM_014415]	-0.5444057	0.0062013385
A_23_P68866	*lnc-FARSB-1*	Homo sapiens cDNA FLJ33441 fis, clone BRACE2021932. [AK090760]	0.597609	0.016359903
A_33_P3384432	*ZNF490*	Homo sapiens zinc finger protein 490 (ZNF490), mRNA [NM_020714]	-0.84599257	0.011728736
A_33_P3402474	*UQCR10*	Homo sapiens ubiquinol-cytochrome c reductase, complex III subunit X (UQCR10), transcript variant 2, mRNA [NM_001003684]	-0.6658001	0.005172653
A_23_P81121	*TFDP2*	Homo sapiens transcription factor Dp-2 (E2F dimerization partner 2) (TFDP2), transcript variant 1, mRNA [NM_001178138]	-1.1762525	0.0054085264
A_23_P82351	*ATAT1*	Homo sapiens alpha tubulin acetyltransferase 1 (ATAT1), transcript variant 1, mRNA [NM_001031722]	-1.1177533	0.005098191
A_23_P200829	*EXOSC9*	Homo sapiens exosome component 9 (EXOSC9), transcript variant 2, mRNA [NM_005033]	-0.4124799	0.01007156
A_33_P3366120	*BBS9*	Homo sapiens Bardet-Biedl syndrome 9 (BBS9), transcript variant 2, mRNA [NM_198428]	-1.3721259	0.006664567
A_23_P123193	*SRGAP2*	Homo sapiens SLIT-ROBO Rho GTPase activating protein 2 (SRGAP2), transcript variant 1, mRNA [NM_015326]	-1.627754	0.0051410845
A_23_P52298	*FLNA*	Homo sapiens filamin A, alpha (FLNA), transcript variant 2, mRNA [NM_001110556]	0.35691237	0.012737636

A_32_P13795	*ACTR3B*	Homo sapiens ARP3 actin-related protein 3 homolog B (yeast) (ACTR3B), transcript variant 1, mRNA [NM_020445]	-1.1931734	0.0070008305
A_23_P410998	*NPM3*	Homo sapiens nucleophosmin/nucleoplasmin 3 (NPM3), mRNA [NM_006993]	-0.5202562	0.015313101
A_19_P00802141	*DNAJC27*	Homo sapiens DnaJ (Hsp40) homolog, subfamily C, member 27 (DNAJC27), transcript variant 1, mRNA [NM_016544]	-0.9307482	0.0060804365
A_23_P8640	*RAB5B*	Homo sapiens RAB5B, member RAS oncogene family (RAB5B), transcript variant 1, mRNA [NM_002868]	-0.42620727	0.007698106
A_23_P14708	*IPO7*	Homo sapiens importin 7 (IPO7), mRNA [NM_006391]	-0.53404695	0.004092341
A_33_P3305467	*GPER1*	Homo sapiens G protein-coupled estrogen receptor 1 (GPER1), transcript variant 3, mRNA [NM_001039966]	-1.3148849	0.003145806
A_23_P47226	*ZNF280D*	Homo sapiens zinc finger protein 280D (ZNF280D), transcript variant 1, mRNA [NM_017661]	-0.9892944	0.00557203
A_23_P259054	*YIF1A*	Homo sapiens Yip1 interacting factor homolog A (S. cerevisiae) (YIF1A), transcript variant 1, mRNA [NM_020470]	-1.0917536	0.009811661
A_23_P109821	*MAPK1*	Homo sapiens mitogen-activated protein kinase 1 (MAPK1), transcript variant 2, mRNA [NM_138957]	-0.5773425	0.016647164
A_23_P25224	*SNX14*	Homo sapiens sorting nexin 14 (SNX14), transcript variant 1, mRNA [NM_153816]	-0.8842823	0.0037856582
A_22_P00016518	*TADA3*	Homo sapiens transcriptional adaptor 3 (TADA3), transcript variant 1, mRNA [NM_006354]	-0.9081273	0.007666008
A_33_P3308744	*YBX3*	Homo sapiens Y box binding protein 3 (YBX3), transcript variant 1, mRNA [NM_003651]	-1.0666722	0.0076856846
A_23_P96590	*PLAC4*	Homo sapiens placenta-specific 4 (PLAC4), mRNA [NM_182832]	0.73382974	0.0044240346
A_22_P00013683	*LAMA4*	Homo sapiens laminin, alpha 4 (LAMA4), transcript variant 1, mRNA [NM_001105206]	-1.4390979	0.0113135865
A_22_P00023755	*GPRASP1*	Homo sapiens G protein-coupled receptor associated sorting protein 1 (GPRASP1), transcript variant 1, mRNA [NM_014710]	-1.3492509	0.012007281
A_24_P86240	*lnc-RP11-712L6.5.1-1*	DKFZp686N0126_r1 686 (synonym: hlcc3) Homo sapiens cDNA clone DKFZp686N0126 5', mRNA sequence [BX508466]	0.50878817	0.012911957
A_33_P3386297	*B4GALT1-AS1*	Homo sapiens B4GALT1 antisense RNA 1 (B4GALT1-AS1), transcript variant 1, long non-coding RNA [NR_108108]	-0.82001686	0.012509614
A_23_P46017	*BMP2K*	Homo sapiens BMP2 inducible kinase (BMP2K), transcript variant 1, mRNA [NM_198892]	-1.1326159	0.012279968

A_33_P3252730	*CCBL2*	Homo sapiens cysteine conjugate-beta lyase 2 (CCBL2), transcript variant 1, mRNA [NM_001008661]	-0.6982872	0.006573993
A_33_P3287105	*DYNC1LI1*	Homo sapiens dynein, cytoplasmic 1, light intermediate chain 1 (DYNC1LI1), mRNA [NM_016141]	-0.30432388	0.0140423635
A_33_P3315223	*ZNF788*	Homo sapiens zinc finger family member 788 (ZNF788), non-coding RNA [NR_027049]	-0.8992473	0.015933463
A_23_P396626	*HNRNPA0*	Homo sapiens heterogeneous nuclear ribonucleoprotein A0 (HNRNPA0), mRNA [NM_006805]	-0.65449715	0.005852893
A_32_P220750	*SPTLC1*	Homo sapiens serine palmitoyltransferase, long chain base subunit 1 (SPTLC1), transcript variant 1, mRNA [NM_006415]	-1.0658462	0.00511559
A_23_P422193	*SYNRG*	Homo sapiens synergin, gamma (SYNRG), transcript variant 1, mRNA [NM_007247]	-0.66620797	0.017155705
A_21_P0010448	*PLGLB1*	Homo sapiens plasminogen-like B1 (PLGLB1), mRNA [NM_001032392]	-1.7865932	0.0062516355
A_23_P110802	*SUV39H1*	Homo sapiens suppressor of variegation 3-9 homolog 1 (Drosophila) (SUV39H1), transcript variant 2, mRNA [NM_003173]	1.085128	0.009591824
A_33_P3226212	*lnc-SMC1B-2*	LNCipedia lincRNA (lnc-SMC1B-2), lincRNA [lnc-SMC1B-2:1]	-1.9249685	0.00968922
A_22_P00012872	*CENPH*	Homo sapiens centromere protein H (CENPH), mRNA [NM_022909]	-0.7485962	0.0066162148
A_24_P153853	*JAM2*	Homo sapiens junctional adhesion molecule 2 (JAM2), transcript variant 3, mRNA [NM_001270408]	-4.049053	0.0048452313
A_23_P145068	*TRIM37*	Homo sapiens tripartite motif containing 37 (TRIM37), transcript variant 2, mRNA [NM_001005207]	-0.78924346	0.011054281
A_23_P64058	*PLEKHH2*	Homo sapiens pleckstrin homology domain containing, family H (with MyTH4 domain) member 2 (PLEKHH2), mRNA [NM_172069]	-2.6350427	0.0032073085
A_33_P3405285	*MTO1*	Homo sapiens mitochondrial tRNA translation optimization 1 (MTO1), transcript variant 2, mRNA [NM_012123]	-1.0720377	0.008538553
A_33_P3317815	*RASGRP2*	Homo sapiens RAS guanyl releasing protein 2 (calcium and DAG-regulated) (RASGRP2), transcript variant 2, mRNA [NM_153819]	-1.1149714	0.010110854
A_33_P3417222	*GSG1*	Homo sapiens germ cell associated 1 (GSG1), transcript variant 3, mRNA [NM_001080554]	0.22197144	0.005255513
A_22_P00019191	*KRAS*	Homo sapiens Kirsten rat sarcoma viral oncogene homolog (KRAS), transcript variant b, mRNA [NM_004985]	-1.0440578	0.0034766807
A_23_P83134	*CD72*	CD72 molecule [Source:HGNC Symbol;Acc:HGNC:1696] [ENST00000378430]	0.7017963	0.013888596

A_23_P117558	*GAS1*	Homo sapiens growth arrest-specific 1 (GAS1), mRNA [NM_002048]	-0.9892771	0.0033868244
A_21_P0009509	*SUZ12P1*	Homo sapiens suppressor of zeste 12 homolog pseudogene 1 (SUZ12P1), non-coding RNA [NR_024187]	-0.68009645	0.006613322
A_23_P87560	*FKBP3*	Homo sapiens FK506 binding protein 3, 25kDa (FKBP3), mRNA [NM_002013]	-0.5904486	0.0032831472
A_33_P3279379	*LOC284240*	PREDICTED: Homo sapiens uncharacterized LOC284240 (LOC284240), misc_RNA [XR_248506]	0.16921885	0.012460022
A_23_P7056	*BTG1*	Homo sapiens B-cell translocation gene 1, anti-proliferative (BTG1), mRNA [NM_001731]	-1.8224306	0.0025739188
A_33_P3212490	*DSTNP2*	Homo sapiens destrin (actin depolymerizing factor) pseudogene 2 (DSTNP2), non-coding RNA [NR_033796]	-0.8155834	0.009722053
A_33_P3309319	*SDAD1*	Homo sapiens SDA1 domain containing 1 (SDAD1), transcript variant 1, mRNA [NM_018115]	0.45081306	0.010740848
A_23_P74269	*DICER1*	Homo sapiens dicer 1, ribonuclease type III (DICER1), transcript variant 3, mRNA [NM_001195573]	-0.7893331	0.0152601935
A_22_P00003927	*PIDD1*	Homo sapiens p53-induced death domain protein 1 (PIDD1), transcript variant 1, mRNA [NM_145886]	-0.8418188	0.015431974
A_23_P69242	*SRM*	Homo sapiens spermidine synthase (SRM), mRNA [NM_003132]	0.87782913	0.01268465
A_23_P140563	*lnc-CETP-1*	Homo sapiens, clone IMAGE:4426859, mRNA. [BC015447]	-1.0533026	0.00928559
A_24_P114183	*SUMF1*	Homo sapiens sulfatase modifying factor 1 (SUMF1), transcript variant 1, mRNA [NM_182760]	-1.1491022	0.0071724257
A_24_P12539	*TTC23*	Homo sapiens tetratricopeptide repeat domain 23 (TTC23), transcript variant 8, mRNA [NM_001288615]	-0.9114281	0.012911027
A_33_P3310293	*FDPS*	Homo sapiens farnesyl diphosphate synthase (FDPS), transcript variant 1, mRNA [NM_002004]	-0.5694618	0.015507675
A_33_P3268304	*KBTBD2*	Homo sapiens kelch repeat and BTB (POZ) domain containing 2 (KBTBD2), mRNA [NM_015483]	-0.76787657	0.011382023
A_23_P106056	*PKIG*	Homo sapiens protein kinase (cAMP-dependent, catalytic) inhibitor gamma (PKIG), transcript variant 1, mRNA [NM_181805]	-2.4858437	0.0031474202
A_23_P165879	*LIMS2*	Homo sapiens LIM and senescent cell antigen-like domains 2 (LIMS2), transcript variant 5, mRNA [NM_001161404]	-3.3387856	0.0039486005
A_23_P17914	*DAD1*	Homo sapiens defender against cell death 1 (DAD1), mRNA [NM_001344]	-0.5641451	0.011618324

A_24_P37519	*RAB10*	Homo sapiens RAB10, member RAS oncogene family (RAB10), mRNA [NM_016131]	-0.80190736	0.006506821
A_33_P3217073	*PNPLA3*	Homo sapiens patatin-like phospholipase domain containing 3 (PNPLA3), mRNA [NM_025225]	-1.1232864	0.010447919
A_33_P3419098	*LZTFL1*	Homo sapiens leucine zipper transcription factor-like 1 (LZTFL1), transcript variant 1, mRNA [NM_020347]	-0.7105751	0.0062283403
A_23_P200637	*GHITM*	Homo sapiens growth hormone inducible transmembrane protein (GHITM), mRNA [NM_014394]	-0.8566308	0.006155321
A_23_P6891	*PLEC*	Homo sapiens plectin (PLEC), transcript variant 6, mRNA [NM_201380]	0.755399	0.0147644505
A_33_P3279920	*FBXO44*	Homo sapiens F-box protein 44 (FBXO44), transcript variant 4, mRNA [NM_001014765]	1.4649897	0.0059128376
A_22_P00011218	*EIF1B*	Homo sapiens eukaryotic translation initiation factor 1B (EIF1B), mRNA [NM_005875]	-1.0650119	0.0040017776
A_23_P170839	*TARDBP*	Homo sapiens TAR DNA binding protein (TARDBP), mRNA [NM_007375]	-0.23786275	0.009987734
A_32_P101301	*lnc-OPN4-1*	LNCipedia lincRNA (lnc-OPN4-1), lincRNA [lnc-OPN4-1:2]	-0.5334697	0.007571981
A_23_P153037	*USP39*	Homo sapiens ubiquitin specific peptidase 39 (USP39), transcript variant 1, mRNA [NM_006590]	-0.7247682	0.0108927535
A_33_P3335865	*LOC102724352*	PREDICTED: Homo sapiens uncharacterized LOC102724352 (LOC102724352), ncRNA [XR_424996]	-1.3095934	0.0053150533
A_23_P422212	*ZNF624*	Homo sapiens zinc finger protein 624 (ZNF624), mRNA [NM_020787]	-0.7032436	0.017157277
A_22_P00025650	*WDR35*	Homo sapiens WD repeat domain 35 (WDR35), transcript variant 1, mRNA [NM_001006657]	-0.766331	0.011786233
A_22_P00003933	*SLC35F3*	Homo sapiens solute carrier family 35, member F3 (SLC35F3), transcript variant 1, mRNA [NM_173508]	4.8357344	0.00180587
A_33_P3415744	*PRKD2*	Homo sapiens protein kinase D2 (PRKD2), transcript variant 1, mRNA [NM_016457]	0.8414979	0.013992443
A_33_P3225268	*EIF1AY*	Homo sapiens eukaryotic translation initiation factor 1A, Y-linked (EIF1AY), transcript variant 1, mRNA [NM_004681]	-0.5658034	0.0045315274
A_33_P3335183	*ANKRD36B*	Homo sapiens ankyrin repeat domain 36B (ANKRD36B), mRNA [NM_025190]	-0.93717	0.014489432
A_33_P3410895	*QSOX1*	Homo sapiens quiescin Q6 sulfhydryl oxidase 1 (QSOX1), transcript variant 2, mRNA [NM_001004128]	1.4356363	0.005169007
A_23_P54816	*LRRC37A2*	Homo sapiens leucine rich repeat containing 37, member A2 (LRRC37A2), mRNA [NM_001006607]	1.1772709	0.009808998

A_23_P138967	*POR*	P450 (cytochrome) oxidoreductase [Source:HGNC Symbol;Acc:HGNC:9208] [ENST00000421059]	0.9153046	0.010960915
A_33_P3273298	*ATP6V0C*	Homo sapiens ATPase, H+ transporting, lysosomal 16kDa, V0 subunit c (ATP6V0C), transcript variant 1, mRNA [NM_001694]	-0.2892136	0.00943937
A_33_P3231814	*SDHD*	Homo sapiens succinate dehydrogenase complex, subunit D, integral membrane protein (SDHD), transcript variant 1, mRNA [NM_003002]	-1.4756756	0.0052430327
A_24_P154080	*ZNF813*	Homo sapiens zinc finger protein 813 (ZNF813), mRNA [NM_001004301]	-0.46025077	0.008459498
A_24_P287780	*ECE1*	Homo sapiens endothelin converting enzyme 1 (ECE1), transcript variant 1, mRNA [NM_001397]	1.1340919	0.004249292
A_23_P122915	*AMMECR1L*	Homo sapiens AMMECR1-like (AMMECR1L), transcript variant 1, mRNA [NM_031445]	-1.1370203	0.0064965826
A_32_P209094	*CHMP1A*	Homo sapiens charged multivesicular body protein 1A (CHMP1A), transcript variant 2, mRNA [NM_002768]	0.6455154	0.01109159
A_33_P3379371	*BRI3*	Homo sapiens brain protein I3 (BRI3), transcript variant 1, mRNA [NM_015379]	-0.6020594	0.008208044
A_22_P00008239	*FGGY*	Homo sapiens FGGY carbohydrate kinase domain containing (FGGY), transcript variant 2, mRNA [NM_018291]	-1.325489	0.007149221
A_19_P00315843	*RYK*	Homo sapiens receptor-like tyrosine kinase (RYK), transcript variant 1, mRNA [NM_001005861]	-0.72003484	0.009962962
A_24_P453921	*lnc-IRF2BP2-3*	LNCipedia lincRNA (lnc-IRF2BP2-3), lincRNA [lnc-IRF2BP2-3:1]	0.64384955	0.011025315
A_33_P3375710	*SCARNA16*	Homo sapiens small Cajal body-specific RNA 16 (SCARNA16), guide RNA [NR_003013]	-1.0852596	0.007569461
A_33_P3249354	*DPY19L1P1*	Homo sapiens DPY19L1 pseudogene 1 (DPY19L1P1), non-coding RNA [NR_036680]	-0.9192723	0.005045916
A_33_P3319261	*lnc-VAMP3-1*	PREDICTED: Homo sapiens calmodulin binding transcription activator 1 (CAMTA1), transcript variant X3, mRNA [XM_006710484]	1.874382	0.0053354
A_23_P156319	*SPDL1*	Homo sapiens spindle apparatus coiled-coil protein 1 (SPDL1), mRNA [NM_017785]	-0.47120497	0.010272991
A_22_P00015371	*EXD3*	Homo sapiens exonuclease 3'-5' domain containing 3 (EXD3), transcript variant 2, mRNA [NM_001286823]	-1.1489108	0.015479728
A_23_P32615	*LARP1*	Homo sapiens La ribonucleoprotein domain family, member 1 (LARP1), mRNA [NM_015315]	-0.83175874	0.006543285
A_23_P204252	*lnc-SRBD1-1*	LNCipedia lincRNA (lnc-SRBD1-1), lincRNA [lnc-SRBD1-1:1]	-0.7939163	0.01582957

A_23_P85893	*MNAT1*	Homo sapiens MNAT CDK-activating kinase assembly factor 1 (MNAT1), transcript variant 1, mRNA [NM_002431]	-0.4076657	0.0054708454
A_23_P124427	*M6PR*	Homo sapiens mannose-6-phosphate receptor (cation dependent) (M6PR), transcript variant 1, mRNA [NM_002355]	-0.36009744	0.01558317
A_19_P00319525	*GLMP*	Homo sapiens glycosylated lysosomal membrane protein (GLMP), transcript variant 1, mRNA [NM_144580]	-0.646554	0.007365646
A_23_P369746	*NEK1*	Homo sapiens NIMA-related kinase 1 (NEK1), transcript variant 2, mRNA [NM_012224]	-0.8098402	0.0043705306
A_23_P258814	*TSTD3*	Homo sapiens thiosulfate sulfurtransferase (rhodanese)-like domain containing 3 (TSTD3), mRNA [NM_001195131]	-0.84206647	0.007500294
A_33_P3340060	*DPH3P1*	Homo sapiens diphthamide biosynthesis 3 pseudogene 1 (DPH3P1), mRNA [NM_080750]	-0.46472147	0.0076570855
A_33_P3420655	*MKX-AS1*	Homo sapiens MKX antisense RNA 1 (MKX-AS1), long non-coding RNA [NR_121652]	2.022444	0.0072230194
A_21_P0014600	*RPL13A*	Homo sapiens ribosomal protein L13a (RPL13A), transcript variant 1, mRNA [NM_012423]	-0.30394292	0.0036402496
A_23_P211227	*KDM4A*	Homo sapiens lysine (K)-specific demethylase 4A (KDM4A), mRNA [NM_014663]	-1.0030661	0.01034603
A_19_P00811812	*POFUT2*	Homo sapiens protein O-fucosyltransferase 2 (POFUT2), transcript variant 3, mRNA [NM_133635]	1.3481737	0.010060375
A_21_P0005794	*MPRIP*	Homo sapiens myosin phosphatase Rho interacting protein (MPRIP), transcript variant 2, mRNA [NM_201274]	-0.5256097	0.015919259
A_33_P3732466	*SHROOM3*	Homo sapiens shroom family member 3 (SHROOM3), mRNA [NM_020859]	-0.90424156	0.013050359
A_23_P124892	*lnc-PRAGMIN.1-3*	LNCipedia lincRNA (lnc-PRAGMIN.1-3), lincRNA [lnc-PRAGMIN.1-3:3]	-1.0694332	0.010871661
A_32_P18470	*LOC100506282*	PREDICTED: Homo sapiens uncharacterized LOC100506282 (LOC100506282), transcript variant X1, misc_RNA [XR_110899]	-0.83202744	0.006736978
A_33_P3248953	*KISS1*	Homo sapiens KiSS-1 metastasis-suppressor (KISS1), mRNA [NM_002256]	6.6979713	0.0027248112
A_24_P522864	*TCEAL5*	Homo sapiens transcription elongation factor A (SII)-like 5 (TCEAL5), mRNA [NM_001012979]	-1.3954539	0.0044240346
A_23_P45871	*C5orf63*	Homo sapiens chromosome 5 open reading frame 63 (C5orf63), transcript variant 2, mRNA [NM_001164478]	-0.83654094	0.015292606
A_24_P133162	*MGC12916*	Homo sapiens uncharacterized protein MGC12916 (MGC12916), long non-coding RNA [NR_026880]	1.5878017	0.0073160357
A_21_P0000475	*IFI44L*	Homo sapiens interferon-induced protein 44-like (IFI44L), mRNA [NM_006820]	2.4876213	0.0046744854

A_23_P208540	*STPG1*	Homo sapiens sperm-tail PG-rich repeat containing 1 (STPG1), transcript variant 3, mRNA [NM_178122]	-1.6607261	0.0050497777
A_24_P32118	*SNORA70B*	Homo sapiens small nucleolar RNA, H/ACA box 70B (SNORA70B), small nucleolar RNA [NR_003707]	0.7414344	0.012806621
A_33_P3244931	*NDUFA3*	Homo sapiens NADH dehydrogenase (ubiquinone) 1 alpha subcomplex, 3, 9kDa (NDUFA3), mRNA [NM_004542]	-0.69234776	0.011969343
A_24_P250535	*ZFYVE26*	Homo sapiens zinc finger, FYVE domain containing 26 (ZFYVE26), mRNA [NM_015346]	-1.1635609	0.009241496
A_33_P3372666	*DBI*	Homo sapiens diazepam binding inhibitor (GABA receptor modulator, acyl-CoA binding protein) (DBI), transcript variant 2, mRNA [NM_001079863]	-0.68669146	0.013391542
A_23_P62920	*TMX4*	Homo sapiens thioredoxin-related transmembrane protein 4 (TMX4), mRNA [NM_021156]	-1.0035518	0.00706395
A_33_P3244951	*PDGFA*	Homo sapiens platelet-derived growth factor alpha polypeptide (PDGFA), transcript variant 2, mRNA [NM_033023]	-0.762143	0.012773474
A_23_P325661	*KIFAP3*	Homo sapiens kinesin-associated protein 3 (KIFAP3), transcript variant 1, mRNA [NM_014970]	-1.1779914	0.005014313
A_23_P24384	*PDE8A*	Homo sapiens phosphodiesterase 8A (PDE8A), transcript variant 3, mRNA [NM_001243137]	-0.5435447	0.01210552
A_23_P74950	*ZNF134*	Homo sapiens zinc finger protein 134 (ZNF134), mRNA [NM_003435]	-0.6997876	0.011021474
A_23_P139965	*CCDC88B*	Homo sapiens coiled-coil domain containing 88B (CCDC88B), mRNA [NM_032251]	-1.19040073158	0.005595418
A_33_P3376873	*RCC2*	Homo sapiens regulator of chromosome condensation 2 (RCC2), transcript variant 1, mRNA [NM_018715]	-0.7708542	0.0055818916
A_23_P169470	*SERP2*	Homo sapiens stress-associated endoplasmic reticulum protein family member 2 (SERP2), mRNA [NM_001010897]	-0.55418706	0.011958462
A_21_P0002852	*CDK9*	Homo sapiens cyclin-dependent kinase 9 (CDK9), mRNA [NM_001261]	-1.0366068	0.0054365364
A_21_P0006404	*SVIL-AS1*	Homo sapiens SVIL antisense RNA 1 (SVIL-AS1), transcript variant 4, long non-coding RNA [NR_110923]	-1.412566	0.0044319443
A_23_P305692	*SEMA3B-AS1*	Homo sapiens SEMA3B antisense RNA 1 (head to head) (SEMA3B-AS1), long non-coding RNA [NR_110702]	-1.3993353	0.013184759
A_33_P3268181	*LINC01204*	Homo sapiens long intergenic non-protein coding RNA 1204 (LINC01204), transcript variant 1, long non-coding RNA [NR_104644]	-1.7073008	0.0086035505

A_23_P214876	*ELMOD2*	Homo sapiens ELMO/CED-12 domain containing 2 (ELMOD2), mRNA [NM_153702]	-0.8670643	0.013620142
A_23_P416468	*LIMS2*	Homo sapiens LIM and senescent cell antigen-like domains 2 (LIMS2), transcript variant 5, mRNA [NM_001161404]	-1.1200515	0.010175607
A_23_P49499	*JARID2*	Homo sapiens jumonji, AT rich interactive domain 2 (JARID2), transcript variant 1, mRNA [NM_004973]	-0.9691286	0.006632856
A_23_P15073	*PIF1*	Homo sapiens PIF1 5'-to-3' DNA helicase (PIF1), transcript variant 2, mRNA [NM_025049]	-0.6971257	0.013864809
A_23_P502142	*ST6GALNAC2*	Homo sapiens ST6 (alpha-N-acetyl-neuraminyl-2,3-beta-galactosyl-1,3)-N-acetylgalactosaminide alpha-2,6-sialyltransferase 2 (ST6GALNAC2), mRNA [NM_006456]	-0.95620656	0.0068512554
A_24_P282043	*JMJD8*	Homo sapiens jumonji domain containing 8 (JMJD8), mRNA [NM_001005920]	0.37718695	0.00371792
A_32_P25253	*FYN*	Homo sapiens FYN proto-oncogene, Src family tyrosine kinase (FYN), transcript variant 1, mRNA [NM_002037]	-0.97571635	0.0129129235
A_23_P63050	*ZNF28*	Homo sapiens zinc finger protein 28 (ZNF28), transcript variant 1, mRNA [NM_006969]	-0.80785877	0.013335527
A_23_P105571	*ISCA1*	Homo sapiens iron-sulfur cluster assembly 1 (ISCA1), mRNA [NM_030940]	-0.4990578	0.0113135865
A_32_P35220	*UROD*	Homo sapiens uroporphyrinogen decarboxylase (UROD), transcript variant 1, mRNA [NM_000374]	-0.8599375	0.010237655
A_23_P105873	*CHPT1*	Homo sapiens choline phosphotransferase 1 (CHPT1), mRNA [NM_020244]	-0.8094079	0.0033199077
A_23_P304524	*CBWD5*	Homo sapiens COBW domain containing 5 (CBWD5), transcript variant 1, mRNA [NM_001024916]	-0.3873587	0.011845045
A_24_P109652	*TRPC4*	Homo sapiens transient receptor potential cation channel, subfamily C, member 4 (TRPC4), transcript variant alpha, mRNA [NM_016179]	-4.392831	0.0029085083
A_23_P48561	*DCLK2*	Homo sapiens doublecortin-like kinase 2 (DCLK2), transcript variant 1, mRNA [NM_001040260]	0.81086546	0.012168611
A_23_P117782	*PEAK1*	Homo sapiens pseudopodium-enriched atypical kinase 1 (PEAK1), mRNA [NM_024776]	-1.1957697	0.009646789
A_23_P30995	*EFS*	Homo sapiens embryonal Fyn-associated substrate (EFS), transcript variant 1, mRNA [NM_005864]	-0.91537267	0.010976066
A_24_P276932	*LARP6*	Homo sapiens La ribonucleoprotein domain family, member 6 (LARP6), transcript variant 1, mRNA [NM_018357]	-1.3792496	0.0060695945

A_24_P47681	*CYB5R4*	Homo sapiens cytochrome b5 reductase 4 (CYB5R4), mRNA [NM_016230]	-0.7403472	0.006573993
A_23_P37441	*ATP6V1C2*	Homo sapiens ATPase, H+ transporting, lysosomal 42kDa, V1 subunit C2 (ATP6V1C2), transcript variant 1, mRNA [NM_001039362]	0.5217686	0.0129849
A_33_P3821660	*CAND1*	cullin-associated and neddylation-dissociated 1 [Source:HGNC Symbol;Acc:HGNC:30688] [ENST00000545606]	-0.6584616	0.0025001846
A_23_P40217	*B2M*	Homo sapiens beta-2-microglobulin (B2M), mRNA [NM_004048]	-0.9856684	0.0027248112
A_23_P32861	*RABL6*	Homo sapiens RAB, member RAS oncogene family-like 6 (RABL6), transcript variant 4, mRNA [NM_001173989]	1.8523647	0.0047988673
A_33_P3224735	*DOK5*	Homo sapiens docking protein 5 (DOK5), transcript variant 1, mRNA [NM_018431]	-2.2748141	0.0043846127
A_23_P17430	*NMD3*	Homo sapiens NMD3 ribosome export adaptor (NMD3), mRNA [NM_015938]	-0.8816271	0.003162355
A_33_P3234864	*ZC3H12B*	Homo sapiens zinc finger CCCH-type containing 12B (ZC3H12B), mRNA [NM_001010888]	1.1875422	0.009713777
A_22_P00003672	*RBM38*	Homo sapiens RNA binding motif protein 38 (RBM38), transcript variant 1, mRNA [NM_017495]	1.0070176	0.015209785
A_23_P337726	*UTRN*	Homo sapiens utrophin (UTRN), mRNA [NM_007124]	-0.9557674	0.01225594
A_33_P3310780	*CDC42SE1*	CDC42 small effector 1 [Source:HGNC Symbol;Acc:HGNC:17719] [ENST00000491825]	1.4745817	0.0062310593
A_33_P3825869	*ATP6*	mitochondrially encoded ATP synthase 6 [Source:HGNC Symbol;Acc:HGNC:7414] [ENST00000361899]	0.87365097	0.0065241847
A_24_P398940	*CTTN*	Homo sapiens cortactin (CTTN), transcript variant 1, mRNA [NM_005231]	-0.5715823	0.016607525
A_23_P332908	*CACNA1C*	Homo sapiens calcium channel, voltage-dependent, L type, alpha 1C subunit (CACNA1C), transcript variant 1, mRNA [NM_199460]	-1.2488047	0.010769378
A_22_P00024803	*CASC4*	Homo sapiens cancer susceptibility candidate 4 (CASC4), transcript variant 1, mRNA [NM_138423]	-0.8041058	0.0039486005
A_23_P210176	*ZFC3H1*	Homo sapiens zinc finger, C3H1-type containing (ZFC3H1), mRNA [NM_144982]	-0.67068714	0.009391877
A_19_P00316415	*lnc-WWC2-1*	17000455083797 GRN_ES Homo sapiens cDNA 5', mRNA sequence [CN283862]	0.8001451	0.011493016
A_24_P413126	*ITGA6*	Homo sapiens integrin, alpha 6 (ITGA6), transcript variant 2, mRNA [NM_000210]	0.4660528	0.011137466
A_23_P204640	*lnc-ERP44-3*	Homo sapiens cDNA FLJ33647 fis, clone BRAMY2024374. [AK090966]	-2.743176	0.014603465

A_23_P103897	*PMEPA1*	Homo sapiens prostate transmembrane protein, androgen induced 1 (PMEPA1), transcript variant 1, mRNA [NM_020182]	-2.3987026	0.0036234485
A_33_P3303086	*NANOG*	Homo sapiens Nanog homeobox (NANOG), transcript variant 1, mRNA [NM_024865]	-1.773172	0.0068076253
A_23_P168669	*SPATA1*	spermatogenesis associated 1 [Source:HGNC Symbol;Acc:HGNC:14682] [ENST00000431031]	-0.6856263	0.011443248
A_23_P92727	*HNRNPLL*	Homo sapiens heterogeneous nuclear ribonucleoprotein L-like (HNRNPLL), transcript variant 1, mRNA [NM_138394]	-0.80636454	0.011346314
A_23_P401084	*CROT*	Homo sapiens carnitine O-octanoyltransferase (CROT), transcript variant 2, mRNA [NM_021151]	0.7999013	0.00652649
A_24_P944458	*RAI14*	Homo sapiens retinoic acid induced 14 (RAI14), transcript variant 1, mRNA [NM_015577]	0.6550019	0.0075218254
A_22_P00015897	*ZNF575*	Homo sapiens zinc finger protein 575 (ZNF575), mRNA [NM_174945]	-0.7968953	0.011676163
A_33_P3395675	*INSIG2*	Homo sapiens insulin induced gene 2 (INSIG2), mRNA [NM_016133]	-13980739	0.004437345
A_22_P00003497	*SNHG6*	Homo sapiens small nucleolar RNA host gene 6 (non-protein coding) (SNHG6), long non-coding RNA [NR_002599]	-0.7628078	0.013864809
A_23_P211631	*lnc-CCDC90B-1*	RST25001 Athersys RAGE Library Homo sapiens cDNA, mRNA sequence [BG205572]	1.8841492	0.0075538
A_23_P31399	*FBLN1*	Homo sapiens fibulin 1 (FBLN1), transcript variant D, mRNA [NM_006486]	-0.83542204	0.0064698174
A_24_P336577	*DSP*	Homo sapiens desmoplakin (DSP), transcript variant 1, mRNA [NM_004415]	-2.8142056	0.003162355
A_33_P3303602	*PON2*	Homo sapiens paraoxonase 2 (PON2), transcript variant 1, mRNA [NM_000305]	-0.666346	0.006155321
A_23_P23017	*FAM212B*	Homo sapiens family with sequence similarity 212, member B (FAM212B), transcript variant 1, mRNA [NM_019099]	-1.479455	0.005792148
A_33_P3360684	*DGKZ*	diacylglycerol kinase, zeta [Source:HGNC Symbol;Acc:HGNC:2857] [ENST00000527674]	0.40257832	0.015320854
A_23_P62932	*C1orf123*	Homo sapiens chromosome 1 open reading frame 123 (C1orf123), mRNA [NM_017887]	-1.0232613	0.0105530135
A_23_P312863	*CORO1B*	Homo sapiens coronin, actin binding protein, 1B (CORO1B), transcript variant 2, mRNA [NM_001018070]	1.0992502	0.0074182227
A_21_P0012116	*ATP1B1*	Homo sapiens ATPase, Na+/K+ transporting, beta 1 polypeptide (ATP1B1), mRNA [NM_001677]	-1.2584662	0.0065241847
A_23_P10182	*TUBB4B*	Homo sapiens tubulin, beta 4B class IVb (TUBB4B), mRNA [NM_006088]	0.6073162	0.01147035
A_23_P251051	*ZNF850*	Homo sapiens zinc finger protein 850 (ZNF850), transcript variant 1, mRNA [NM_001193552]	-1.0096762	0.00628389

A_23_P12272	*ACOX2*	Homo sapiens acyl-CoA oxidase 2, branched chain (ACOX2), mRNA [NM_003500]	-0.90769506	0.013662534
A_23_P205646	*NF2*	Homo sapiens neurofibromin 2 (merlin) (NF2), transcript variant 8, mRNA [NM_181832]	-0.88677764	0.0031827379
A_23_P138253	*PPP1R8*	Homo sapiens protein phosphatase 1, regulatory subunit 8 (PPP1R8), transcript variant 2, mRNA [NM_138558]	-0.8368721	0.010727068
A_33_P3224020	*MAP4K5*	Homo sapiens mitogen-activated protein kinase kinase kinase kinase 5 (MAP4K5), transcript variant 2, mRNA [NM_198794]	-1.165989	0.004588558
A_23_P317105	*CHTOP*	Homo sapiens chromatin target of PRMT1 (CHTOP), transcript variant 1, mRNA [NM_015607]	-0.81642747	0.00641094
A_21_P0011303	*ARAFP2*	Homo sapiens v-raf murine sarcoma 3611 viral oncogene homolog pseudogene, mRNA (cDNA clone IMAGE:5295529). [BC033982]	0.7660742	0.006639663
A_23_P24987	*AKAP10*	Homo sapiens A kinase (PRKA) anchor protein 10 (AKAP10), mRNA [NM_007202]	-0.3148746	0.00786361
A_23_P218997	*PDCD6IPP2*	Homo sapiens PDCD6IP pseudogene 2 (PDCD6IPP2), non-coding RNA [NR_037599]	-0.87860036	0.012989547
A_23_P308954	*TSPAN31*	Homo sapiens tetraspanin 31 (TSPAN31), mRNA [NM_005981]	-1.2439594	0.005154336
A_23_P93844	*PDCD6*	Homo sapiens programmed cell death 6 (PDCD6), transcript variant 1, mRNA [NM_013232]	-0.61533046	0.01421644
A_21_P0014885	*BHLHB9*	Homo sapiens basic helix-loop-helix domain containing, class B, 9 (BHLHB9), transcript variant 2, mRNA [NM_030639]	-1.6611724	0.005236083
A_33_P3390107	*TOMM7*	Homo sapiens translocase of outer mitochondrial membrane 7 homolog (yeast) (TOMM7), mRNA [NM_019059]	-0.8635666	0.0024890031
A_23_P407840	*LOC100506257*	Homo sapiens cDNA FLJ61763 complete cds. [AK295076]	0.93958926	0.014117065
A_23_P83599	*RNA18S5*	Homo sapiens RNA, 18S ribosomal 5 (RNA18S5), ribosomal RNA [NR_003286]	-0.576951	0.0134738535
A_23_P36611	*FNDC1*	Homo sapiens fibronectin type III domain containing 1 (FNDC1), mRNA [NM_032532]	-1.2724426	0.0053150533
A_23_P5131	*PRKAR1B*	Homo sapiens protein kinase, cAMP-dependent, regulatory, type I, beta (PRKAR1B), transcript variant 2, mRNA [NM_002735]	1.9782938	0.0051985686
A_33_P3216664	*APAF1*	Homo sapiens apoptotic peptidase activating factor 1 (APAF1), transcript variant 3, mRNA [NM_181861]	-0.7318889	0.008627961
A_24_P250227	*ISYNA1*	Homo sapiens inositol-3-phosphate synthase 1 (ISYNA1), transcript variant 1, mRNA [NM_016368]	1.1686008	0.013657404

A_23_P15182	*LOC151174*	Homo sapiens uncharacterized LOC151174 (LOC151174), transcript variant 1, long non-coding RNA [NR_026925]	2.8435917	0.014629385
A_33_P3337272	*NR1D1*	Homo sapiens nuclear receptor subfamily 1, group D, member 1 (NR1D1), mRNA [NM_021724]	2.012955	0.008298705
A_33_P3379841	*ARL2BP*	Homo sapiens ADP-ribosylation factor-like 2 binding protein (ARL2BP), mRNA [NM_012106]	-0.8324182	0.011448485
A_21_P0010750	*NRARP*	Homo sapiens NOTCH-regulated ankyrin repeat protein (NRARP), mRNA [NM_001004354]	1.2541518	0.013587454
A_23_P416142	*N4BP2L2-IT2*	Homo sapiens N4BPL2 intronic transcript 2 (non-protein coding) (N4BP2L2-IT2), long non-coding RNA [NR_026928]	0.25909096	0.013653012
A_23_P154771	*DLG1*	Homo sapiens discs, large homolog 1 (Drosophila) (DLG1), transcript variant 2, mRNA [NM_004087]	-1.1756736	0.005936798
A_32_P150030	*KLHL35*	Homo sapiens kelch-like family member 35 (KLHL35), mRNA [NM_001039548]	-1.0003675	0.015238087
A_33_P3415037	*DUSP15*	Homo sapiens dual specificity phosphatase 15 (DUSP15), transcript variant 1, mRNA [NM_080611]	0.7490241	0.007889792
A_23_P355623	*PPM1D*	Homo sapiens protein phosphatase, Mg2+/Mn2+ dependent, 1D (PPM1D), mRNA [NM_003620]	-1.4950738	0.006466261
A_21_P0010307	*VDAC2*	Homo sapiens voltage-dependent anion channel 2 (VDAC2), transcript variant 1, mRNA [NM_001184783]	-0.47480732	0.00842156
A_23_P56680	*ANKIB1*	Homo sapiens ankyrin repeat and IBR domain containing 1 (ANKIB1), mRNA [NM_019004]	-1.2159609	0.0039322884
A_22_P00005535	*LOC284825*	Homo sapiens uncharacterized LOC284825 (LOC284825), transcript variant 1, long non-coding RNA [NR_126010]	1.3469347	0.012609954
A_21_P0012112	*ATP5G3*	Homo sapiens ATP synthase, H+ transporting, mitochondrial Fo complex, subunit C3 (subunit 9) (ATP5G3), transcript variant 3, mRNA [NM_001002258]	-0.7231026	0.0057028057
A_33_P3413468	*SH3RF3-AS1*	Homo sapiens SH3RF3 antisense RNA 1 (SH3RF3-AS1), long non-coding RNA [NR_029193]	0.6136587	0.005255513
A_24_P95029	*XLOC_l2_008560*	BROAD Institute lincRNA (XLOC_l2_008560), lincRNA [TCONS_l2_00016171]	0.55746967	0.012993897
A_23_P87580	*EDA2R*	Homo sapiens ectodysplasin A2 receptor (EDA2R), transcript variant 3, mRNA [NM_001242310]	-0.9008179	0.01231797
A_33_P3326682	*TAX1BP1*	Homo sapiens Tax1 (human T-cell leukemia virus type I) binding protein 1 (TAX1BP1), transcript variant 1, mRNA [NM_006024]	-1.0939987	0.0066818837

A_22_P00023255	*ANP32D*	Homo sapiens acidic (leucine-rich) nuclear phosphoprotein 32 family, member D (ANP32D), mRNA [NM_012404]	-0.18940201	0.010314908
A_24_P376556	*SDHC*	Homo sapiens succinate dehydrogenase complex, subunit C, integral membrane protein, 15kDa (SDHC), transcript variant 1, mRNA [NM_003001]	-0.9459337	0.012773474
A_33_P3283122	*CYCS*	Homo sapiens cytochrome c, somatic (CYCS), mRNA [NM_018947]	-0.3494601	0.0039322884
A_33_P3356910	*SENP1*	Homo sapiens SUMO1/sentrin specific peptidase 1 (SENP1), transcript variant 2, mRNA [NM_001267595]	-0.6933839	0.008298705
A_23_P323751	*WWC2*	Homo sapiens WW and C2 domain containing 2 (WWC2), mRNA [NM_024949]	-0.52639836	0.014591436
A_24_P242820	*TCEAL6*	Homo sapiens transcription elongation factor A (SII)-like 6 (TCEAL6), mRNA [NM_001006938]	-1.1759632	0.006890667
A_23_P50946	*FAM83D*	Homo sapiens family with sequence similarity 83, member D (FAM83D), mRNA [NM_030919]	-0.56372494	0.010735533
A_23_P256158	*TSN*	Homo sapiens translin (TSN), transcript variant 1, mRNA [NM_004622]	-0.67224026	0.003422245
A_23_P115683	*RAMP1*	Homo sapiens receptor (G protein-coupled) activity modifying protein 1 (RAMP1), mRNA [NM_005855]	0.73487437	0.015156207
A_33_P3419545	*ADRA2C*	Homo sapiens adrenoceptor alpha 2C (ADRA2C), mRNA [NM_000683]	-5.9517174	0.002804883
A_21_P0007590	*HPS6*	Homo sapiens Hermansky-Pudlak syndrome 6 (HPS6), mRNA [NM_024747]	-0.6264148	0.013596905
A_33_P3358745	*CDADC1*	Homo sapiens cytidine and dCMP deaminase domain containing 1 (CDADC1), transcript variant 2, mRNA [NM_001193478]	-0.8724851	0.009169725
A_21_P0006730	*SEPP1*	Homo sapiens selenoprotein P, plasma, 1 (SEPP1), transcript variant 3, mRNA [NM_001093726]	2.4119868	0.0038773962
A_33_P3344127	*MITF*	Homo sapiens microphthalmia-associated transcription factor (MITF), transcript variant 1, mRNA [NM_198159]	2.5671139	0.0024890031
A_22_P00006752	*HIST1H2AC*	Homo sapiens histone cluster 1, H2ac (HIST1H2AC), mRNA [NM_003512]	0.487561	0.010976941
A_33_P3321507	*RAET1E-AS1*	Homo sapiens RAET1E antisense RNA 1 (RAET1E-AS1), transcript variant 1, long non-coding RNA [NR_045126]	-1.205411	0.0065308674
A_23_P212715	*LOC101928245*	PREDICTED: Homo sapiens uncharacterized LOC101928245 (LOC101928245), ncRNA [XR_243144]	-1.6044147	0.0068755653
A_33_P3314161	*TNRC18*	Homo sapiens trinucleotide repeat containing 18 (TNRC18), mRNA [NM_001080495]	0.99047065	0.0054910174

A_24_P917833	*CBLB*	Homo sapiens Cbl proto-oncogene B, E3 ubiquitin protein ligase (CBLB), mRNA [NM_170662]	-1.2402015	0.0076496457
A_33_P3330209	*YAF2*	Homo sapiens YY1 associated factor 2 (YAF2), transcript variant 1, mRNA [NM_001190979]	-0.92230237	0.008764855
A_33_P3287972	*TMED10*	Homo sapiens transmembrane emp24-like trafficking protein 10 (yeast) (TMED10), mRNA [NM_006827]	-0.8009686	0.0060066204
A_23_P310086	*AUTS2*	Homo sapiens autism susceptibility candidate 2 (AUTS2), transcript variant 3, mRNA [NM_001127232]	-2.719951	0.0041847117
A_33_P3367830	*RASA2*	RAS p21 protein activator 2 [Source:HGNC Symbol;Acc:HGNC:9872] [ENST00000452898]	-0.34192282	0.016946968
A_33_P3251538	*BEND6*	Homo sapiens BEN domain containing 6 (BEND6), mRNA [NM_152731]	-0.73857236	0.013549144
A_33_P3403615	*EFEMP2*	Homo sapiens EGF containing fibulin-like extracellular matrix protein 2 (EFEMP2), transcript variant 1, mRNA [NM_016938]	-0.6900315	0.014349793
A_21_P0012033	*MAPKAP1*	Homo sapiens mitogen-activated protein kinase associated protein 1 (MAPKAP1), transcript variant 6, mRNA [NM_001006618]	0.37778708	0.015868282
A_33_P3339397	*FKBP1A*	Homo sapiens FK506 binding protein 1A, 12kDa (FKBP1A), transcript variant 1, mRNA [NM_000801]	1.6795173	0.017031018
A_23_P251767	*lnc-DYNC1I2-1*	Homo sapiens cDNA FLJ14080 fis, clone HEMBB1002152. [AK024142]	-1.0906131	0.010937513
A_24_P706340	*SH3YL1*	Homo sapiens SH3 and SYLF domain containing 1 (SH3YL1), transcript variant 4, mRNA [NM_001282687]	-1.1876966	0.0090518845
A_33_P3441583	*BANP*	Homo sapiens BTG3 associated nuclear protein (BANP), transcript variant 1, mRNA [NM_017869]	0.8030628	0.016860068
A_33_P3268798	*FAM155A*	Homo sapiens family with sequence similarity 155, member A (FAM155A), mRNA [NM_001080396]	-2.130347	0.0060066204
A_23_P11286	*ADAT2*	Homo sapiens adenosine deaminase, tRNA-specific 2 (ADAT2), transcript variant 2, mRNA [NM_001286259]	-0.8488294	0.011324697
A_33_P3396473	*KLHL9*	Homo sapiens kelch-like family member 9 (KLHL9), mRNA [NM_018847]	-0.8679323	0.013594373
A_32_P36942	*HNRNPH2*	Homo sapiens heterogeneous nuclear ribonucleoprotein H2 (H') (HNRNPH2), transcript variant 1, mRNA [NM_019597]	-0.6490774	0.005449271
A_33_P3372257	*PPP6R2*	Homo sapiens protein phosphatase 6, regulatory subunit 2 (PPP6R2), transcript variant 1, mRNA [NM_001242898]	-0.78932923	0.013212187

A_24_P944049	*TYW5*	Homo sapiens tRNA-yW synthesizing protein 5 (TYW5), transcript variant 1, mRNA [NM_001039693]	-1.0007651	0.011499655
A_23_P93629	*HOMER1*	Homo sapiens homer homolog 1 (Drosophila) (HOMER1), transcript variant 1, mRNA [NM_004272]	-0.38702324	0.0044319443
A_24_P299474	*CEP68*	Homo sapiens centrosomal protein 68kDa (CEP68), mRNA [NM_015147]	-0.6125686	0.011454393
A_33_P3565787	*TRIM24*	Homo sapiens tripartite motif containing 24 (TRIM24), transcript variant 1, mRNA [NM_015905]	-0.8338616	0.003518271
A_33_P3300092	*TENM2*	Homo sapiens teneurin transmembrane protein 2 (TENM2), mRNA [NM_001122679]	-3.9531047	0.0026564114
A_24_P925664	*PSMG3-AS1*	Homo sapiens PSMG3 antisense RNA 1 (head to head) (PSMG3-AS1), transcript variant 1, long non-coding RNA [NR_027329]	-1.3582829	0.009784609
A_24_P860797	*NDST1*	Homo sapiens N-deacetylase/N-sulfotransferase (heparan glucosaminyl) 1 (NDST1), transcript variant 1, mRNA [NM_001543]	-1.3610525	0.0034310205
A_33_P3296871	*MDM2*	Homo sapiens MDM2 proto-oncogene, E3 ubiquitin protein ligase (MDM2), transcript variant 1, mRNA [NM_002392]	-1.3372271	0.0052543716
A_33_P3224595	*PAIP2B*	Homo sapiens poly(A) binding protein interacting protein 2B (PAIP2B), mRNA [NM_020459]	-1.3608967	0.004054826
A_23_P215751	*ZNF33B*	Homo sapiens zinc finger protein 33B (ZNF33B), mRNA [NM_006955]	-1.0745236	0.0067923404
A_32_P24376	*OFD1*	Homo sapiens oral-facial-digital syndrome 1 (OFD1), mRNA [NM_003611]	-1.401251	0.003956951
A_23_P166526	*NDUFA5*	Homo sapiens NADH dehydrogenase (ubiquinone) 1 alpha subcomplex, 5 (NDUFA5), transcript variant 1, mRNA [NM_005000]	-0.7237	0.0031941293
A_24_P23258	*KRTAP2-3*	Homo sapiens keratin associated protein 2-3 (KRTAP2-3), mRNA [NM_001165252]	1.5188491	0.0049917083
A_23_P44466	*RIBC2*	Homo sapiens RIB43A domain with coiled-coils 2 (RIBC2), mRNA [NM_015653]	-0.97220206	0.0130611155
A_22_P00019529	*GRAMD4*	Homo sapiens GRAM domain containing 4 (GRAMD4), mRNA [NM_015124]	-0.6246135	0.0067319144
A_23_P121602	*CCDC102B*	Homo sapiens coiled-coil domain containing 102B (CCDC102B), transcript variant 2, mRNA [NM_024781]	3.8865352	0.0054879813
A_23_P101615	*LOC102724096*	Homo sapiens uncharacterized LOC102724096 (LOC102724096), long non-coding RNA [NR_125874]	0.4791345	0.011025315
A_23_P137391	*SAP30*	Homo sapiens Sin3A-associated protein, 30kDa (SAP30), mRNA [NM_003864]	-1.0169023	0.0058047334

A_22_P00008323	*ZNF565*	Homo sapiens zinc finger protein 565 (ZNF565), mRNA [NM_001042474]	-0.7107738	0.015419179
A_23_P163467	*ENO1*	Homo sapiens enolase 1, (alpha) (ENO1), transcript variant 1, mRNA [NM_001428]	0.913862	0.011162851
A_24_P135406	*lnc-ITGBL1-1*	LNCipedia lincRNA (lnc-ITGBL1-1), lincRNA [lnc-ITGBL1-1:1]	-1.3185806	0.00969217
A_23_P167017	*C15orf52*	Homo sapiens chromosome 15 open reading frame 52 (C15orf52), mRNA [NM_207380]	-1.06285	0.0140423635
A_19_P00315647	*KCTD9*	Homo sapiens potassium channel tetramerization domain containing 9 (KCTD9), mRNA [NM_017634]	-0.82644725	0.007474992
A_23_P120254	*POPDC2*	Homo sapiens popeye domain containing 2 (POPDC2), mRNA [NM_022135]	-1.5430868	0.0055521973
A_22_P00005228	*LOC100507165*	PREDICTED: Homo sapiens uncharacterized LOC100507165 (LOC100507165), ncRNA [XR_110530]	-2.8350518	0.002419416
A_32_P130630	*DUSP22*	Homo sapiens dual specificity phosphatase 22 (DUSP22), transcript variant 2, mRNA [NM_020185]	-0.93323344	0.005734989
A_23_P80122	*SNHG23*	small nucleolar RNA host gene 23 (non-protein coding) [Source:HGNC Symbol;Acc:HGNC:50622] [ENST00000554369]	1.1595372	0.006678166
A_33_P3402116	*SLC16A7*	Homo sapiens solute carrier family 16 (monocarboxylate transporter), member 7 (SLC16A7), transcript variant 3, mRNA [NM_004731]	-0.9978802	0.0060705077
A_24_P399622	*WRB*	Homo sapiens tryptophan rich basic protein (WRB), transcript variant 1, mRNA [NM_004627]	-0.97646046	0.0039728633
A_24_P23245	*AGO1*	Homo sapiens argonaute RISC catalytic component 1 (AGO1), mRNA [NM_012199]	0.40962982	0.009450744
A_33_P3393836	*COPE*	Homo sapiens coatomer protein complex, subunit epsilon (COPE), transcript variant 3, mRNA [NM_199444]	1.0043137	0.016343247
A_23_P353035	*NDUFA6*	Homo sapiens NADH dehydrogenase (ubiquinone) 1 alpha subcomplex, 6, 14kDa (NDUFA6), mRNA [NM_002490]	-0.638262	0.011171237
A_33_P3402600	*NT5C3A*	Homo sapiens 5'-nucleotidase, cytosolic IIIA (NT5C3A), transcript variant 4, mRNA [NM_001166118]	-0.40706232	0.010104091
A_23_P154234	*IGFBP7*	Homo sapiens insulin-like growth factor binding protein 7 (IGFBP7), transcript variant 1, mRNA [NM_001553]	-1.0870163	0.0026564114
A_23_P112801	*POLE4*	Homo sapiens polymerase (DNA-directed), epsilon 4, accessory subunit (POLE4), mRNA [NM_019896]	-0.6409108	0.006678166
A_33_P3278313	*CHP1*	Homo sapiens calcineurin-like EF-hand protein 1 (CHP1), mRNA [NM_007236]	-0.9669661	0.0067226742

A_24_P904903	*FPGT*	Homo sapiens fucose-1-phosphate guanylyltransferase (FPGT), transcript variant 1, mRNA [NM_003838]	-1.0375952	0.007206317
A_21_P0010899	*MSRB3*	Homo sapiens methionine sulfoxide reductase B3 (MSRB3), transcript variant 3, mRNA [NM_001193460]	-0.74695134	0.007103236
A_21_P0006520	*DDX39B*	Homo sapiens DEAD (Asp-Glu-Ala-Asp) box polypeptide 39B (DDX39B), transcript variant 1, mRNA [NM_004640]	0.873366	0.0064680316
A_21_P0000428	*lnc-ATP2B3-1*	Homo sapiens cDNA clone IMAGE:4797878. [BC030106]	-0.93317705	0.0057328343
A_33_P3296499	*SNORD114-31*	Homo sapiens small nucleolar RNA, C/D box 114-31 (SNORD114-31), small nucleolar RNA [NR_003224]	0.9366834	0.007070614
A_33_P3397161	*CECR3*	Homo sapiens cat eye syndrome chromosome region, candidate 3 (non-protein coding) (CECR3), long non-coding RNA [NR_038398]	0.83480096	0.011427788
A_24_P701776	*PTPRK*	Homo sapiens protein tyrosine phosphatase, receptor type, K (PTPRK), transcript variant 5, mRNA [NM_001291983]	0.95797586	0.0115006585
A_32_P215113	*SHANK2-AS3*	Homo sapiens SHANK2 antisense RNA 3 (SHANK2-AS3), long non-coding RNA [NR_073536]	1.1484255	0.0037856582
A_32_P195647	*ARHGEF35*	Homo sapiens Rho guanine nucleotide exchange factor (GEF) 35 (ARHGEF35), mRNA [NM_001003702]	-1.4535275	0.0062071052
A_33_P3256344	*CCDC58*	Homo sapiens coiled-coil domain containing 58 (CCDC58), mRNA [NM_001017928]	-0.8226664	0.0046073813
A_19_P00800638	*KLHL15*	Homo sapiens kelch-like family member 15 (KLHL15), mRNA [NM_030624]	1.6890055	0.0024890031
A_23_P503200	*LINC01088*	Homo sapiens long intergenic non-protein coding RNA 1088 (LINC01088), long non-coding RNA [NR_038342]	0.7879128	0.010817938
A_21_P0000302	*LINC00704*	Homo sapiens long intergenic non-protein coding RNA 704 (LINC00704), long non-coding RNA [NR_024475]	1.8180325	0.005581195
A_24_P164505	*PHF10*	Homo sapiens PHD finger protein 10 (PHF10), transcript variant 1, mRNA [NM_018288]	-0.9817424	0.0057647754
A_33_P3313055	*SNORA8*	Homo sapiens small nucleolar RNA, H/ACA box 8 (SNORA8), small nucleolar RNA [NR_002920]	-1.2130737	0.0037856582
A_23_P213602	*FAM106CP*	Homo sapiens family with sequence similarity 106, member C, pseudogene (FAM106CP), long non-coding RNA [NR_026810]	0.8679341	0.0145022655
A_21_P0000367	*NOTCH3*	Homo sapiens notch 3 (NOTCH3), mRNA [NM_000435]	-2.2034838	0.0056002573
A_23_P105747	*CSNK1G3*	Homo sapiens casein kinase 1, gamma 3 (CSNK1G3), transcript variant 4, mRNA [NM_001044723]	-0.7900035	0.007102401

A_23_P321511	*SNORA32*	Homo sapiens small nucleolar RNA, H/ACA box 32 (SNORA32), small nucleolar RNA [NR_003032]	-1.1732173	0.0037093356
A_33_P3399363	*APPL2*	Homo sapiens adaptor protein, phosphotyrosine interaction, PH domain and leucine zipper containing 2 (APPL2), transcript variant 1, mRNA [NM_018171]	-0.93432516	0.002535607
A_24_P173754	*MARCH3*	Homo sapiens membrane-associated ring finger (C3HC4) 3, E3 ubiquitin protein ligase (MARCH3), mRNA [NM_178450]	1.1553682	0.007900263
A_24_P385336	*GPR155*	Homo sapiens G protein-coupled receptor 155 (GPR155), transcript variant 1, mRNA [NM_001033045]	-1.119066	0.0063259676
A_23_P110661	*C1orf21*	Homo sapiens chromosome 1 open reading frame 21 (C1orf21), mRNA [NM_030806]	-1.3094566	0.002804883
A_33_P3243093	*HIPK1*	Homo sapiens homeodomain interacting protein kinase 1 (HIPK1), transcript variant 2, mRNA [NM_152696]	0.8892654	0.0097600445
A_33_P3213822	*SKIV2L2*	Homo sapiens superkiller viralicidic activity 2-like 2 (S. cerevisiae) (SKIV2L2), mRNA [NM_015360]	-0.47477168	0.017107433
A_23_P8311	*RGS5*	Homo sapiens regulator of G-protein signaling 5 (RGS5), transcript variant 1, mRNA [NM_003617]	-0.24960595	0.01627333
A_23_P67162	*KCNK2*	Homo sapiens potassium channel, two pore domain subfamily K, member 2 (KCNK2), transcript variant 1, mRNA [NM_001017424]	-1.6544302	0.006605609
A_24_P185709	*TDP2*	Homo sapiens tyrosyl-DNA phosphodiesterase 2 (TDP2), mRNA [NM_016614]	-1.0119505	0.007566709
A_23_P110473	*PIN1*	Homo sapiens peptidylprolyl cis/trans isomerase, NIMA-interacting 1 (PIN1), transcript variant 1, mRNA [NM_006221]	0.69673496	0.014245707
A_23_P155332	*EPB41L1*	Homo sapiens erythrocyte membrane protein band 4.1-like 1 (EPB41L1), transcript variant 1, mRNA [NM_012156]	-1.3652492	0.007485581
A_23_P102109	*NAIP*	Homo sapiens NLR family, apoptosis inhibitory protein (NAIP), transcript variant 1, mRNA [NM_004536]	2.077096	0.0047132876
A_33_P3461039	*PCNP*	Homo sapiens PEST proteolytic signal containing nuclear protein (PCNP), mRNA [NM_020357]	-0.74594766	0.00856805
A_33_P3256660	*TUBA4A*	Homo sapiens tubulin, alpha 4a (TUBA4A), transcript variant 1, mRNA [NM_006000]	1.5173564	0.0031941293
A_33_P3268284	*lnc-MTERFD3-1*	Homo sapiens cDNA FLJ31150 fis, clone IMR322001534. [AK055712]	-0.79869276	0.006653682
A_24_P330691	*GDI2*	Homo sapiens GDP dissociation inhibitor 2 (GDI2), transcript variant 1, mRNA [NM_001494]	-0.5325667	0.013985718

A_23_P7697	*FBXO22*	Homo sapiens F-box protein 22 (FBXO22), transcript variant 2, mRNA [NM_012170]	-0.9265435	0.010503048
A_23_P127128	*SUMO2*	Homo sapiens small ubiquitin-like modifier 2 (SUMO2), transcript variant 1, mRNA [NM_006937]	-0.3968756	0.013076766
A_23_P139260	*SNX2*	Homo sapiens sorting nexin 2 (SNX2), transcript variant 1, mRNA [NM_003100]	-0.9951589	0.0068846266
A_23_P14673	*DNAJC1*	Homo sapiens DnaJ (Hsp40) homolog, subfamily C, member 1 (DNAJC1), mRNA [NM_022365]	-0.7812977	0.010369088
A_24_P370670	*SLC22A18*	Homo sapiens solute carrier family 22, member 18 (SLC22A18), transcript variant 2, mRNA [NM_183233]	-1.3775966	0.016798597
A_21_P0012391	*IGDCC4*	Homo sapiens immunoglobulin superfamily, DCC subclass, member 4 (IGDCC4), mRNA [NM_020962]	-1.7759566	0.004282122
A_23_P10442	*ZMYM6NB*	Homo sapiens ZMYM6 neighbor (ZMYM6NB), mRNA [NM_001195156]	-1.3101295	0.0050554653
A_23_P72387	*OSBPL1A*	Homo sapiens oxysterol binding protein-like 1A (OSBPL1A), transcript variant 2, mRNA [NM_080597]	-0.9014802	0.015292874
A_24_P110983	*SMAD4*	Homo sapiens SMAD family member 4 (SMAD4), mRNA [NM_005359]	-0.55381674	0.0031941293
A_23_P127150	*AFAP1*	Homo sapiens actin filament associated protein 1 (AFAP1), transcript variant A, mRNA [NM_001134647]	-1.3428937	0.009882768
A_22_P00003295	*AKT3*	Homo sapiens v-akt murine thymoma viral oncogene homolog 3 (AKT3), transcript variant 1, mRNA [NM_005465]	-1.6514066	0.005920916
A_24_P167473	*TUBGCP2*	Homo sapiens tubulin, gamma complex associated protein 2 (TUBGCP2), transcript variant 2, mRNA [NM_006659]	0.8711867	0.009091813
A_23_P152356	*STARD4-AS1*	Homo sapiens STARD4 antisense RNA 1 (STARD4-AS1), long non-coding RNA [NR_040093]	-1.9831963	0.004588558
A_24_P753161	*ARPC3*	Homo sapiens actin related protein 2/3 complex, subunit 3, 21kDa (ARPC3), transcript variant 1, mRNA [NM_001278556]	-0.5162053	0.006474282
A_23_P142310	*ZNF200*	Homo sapiens zinc finger protein 200 (ZNF200), transcript variant 1, mRNA [NM_003454]	-1.1232789	0.0051109
A_33_P3410459	*BMPR2*	Homo sapiens bone morphogenetic protein receptor, type II (serine/threonine kinase) (BMPR2), mRNA [NM_001204]	-1.0801703	0.0040942165
A_19_P00322357	*MKNK2*	Homo sapiens MAP kinase interacting serine/threonine kinase 2 (MKNK2), transcript variant 1, mRNA [NM_017572]	-1.3820231	0.0033490837

A_33_P3368452	*SCARB2*	Homo sapiens scavenger receptor class B, member 2 (SCARB2), transcript variant 2, mRNA [NM_001204255]	-0.81119484	0.007279599
A_23_P62967	*LOC101928738*	Homo sapiens uncharacterized LOC101928738 (LOC101928738), long non-coding RNA [NR_110851]	0.9749462	0.012515723
A_24_P40529	*MICA*	Homo sapiens MHC class I polypeptide-related sequence A (MICA), transcript variant 1*001, mRNA [NM_000247]	-1.2636199	0.009690877
A_23_P418413	*DISC1*	Homo sapiens disrupted in schizophrenia 1 (DISC1), transcript variant L, mRNA [NM_018662]	-3.3205938	0.003381037
A_32_P99902	*TMLHE*	Homo sapiens trimethyllysine hydroxylase, epsilon (TMLHE), transcript variant 1, mRNA [NM_018196]	-1.1520506	0.015771633
A_33_P3262537	*OXSR1*	Homo sapiens oxidative stress responsive 1 (OXSR1), mRNA [NM_005109]	-1.115252	0.0029355595
A_33_P3225937	*C15orf40*	Homo sapiens chromosome 15 open reading frame 40 (C15orf40), transcript variant 1, mRNA [NM_144597]	-0.6835067	0.012926502
A_23_P3042	*CRYZL1*	Homo sapiens crystallin, zeta (quinone reductase)-like 1 (CRYZL1), mRNA [NM_145858]	-0.7380768	0.009172592
A_33_P3393175	*PFDN5*	Homo sapiens prefoldin subunit 5 (PFDN5), transcript variant 1, mRNA [NM_002624]	-0.5041823	0.006538247
A_22_P00024094	*PPP2R5E*	Homo sapiens protein phosphatase 2, regulatory subunit B', epsilon isoform (PPP2R5E), transcript variant 1, mRNA [NM_006246]	-0.67015666	0.0065241847
A_33_P3402329	*CAPN7*	Homo sapiens calpain 7 (CAPN7), mRNA [NM_014296]	-0.84721744	0.0077699986
A_24_P124992	*lnc-BEST4-1*	LNCipedia lincRNA (lnc-BEST4-1), lincRNA [lnc-BEST4-1:2]	-0.99870867	0.00410343
A_23_P30495	*MIR503HG*	Homo sapiens MIR503 host gene (non-protein coding) (MIR503HG), long non-coding RNA [NR_024607]	-0.90626544	0.008189544
A_33_P3234899	*PSMA4*	Homo sapiens proteasome (prosome, macropain) subunit, alpha type, 4 (PSMA4), transcript variant 1, mRNA [NM_002789]	-0.5969197	0.0029695062
A_32_P58407	*HMGCR*	Homo sapiens 3-hydroxy-3-methylglutaryl-CoA reductase (HMGCR), transcript variant 1, mRNA [NM_000859]	-1.3679574	0.004402651
A_23_P401098	*PSMB3*	Homo sapiens proteasome (prosome, macropain) subunit, beta type, 3 (PSMB3), transcript variant 1, mRNA [NM_002795]	-0.6306279	0.013182338
A_33_P3278303	*KCND3*	potassium voltage-gated channel, Shal-related subfamily, member 3 [Source:HGNC Symbol;Acc:HGNC:6239] [ENST00000369697]	-1.0122126	0.010406291

A_23_P325040	*TTC39C*	Homo sapiens tetratricopeptide repeat domain 39C (TTC39C), transcript variant 2, mRNA [NM_153211]	-1.2320358	0.007264689
A_23_P51397	*TMPO*	Homo sapiens thymopoietin (TMPO), transcript variant 1, mRNA [NM_003276]	-0.15179078	0.011347969
A_33_P3622802	*ZNF75D*	Homo sapiens zinc finger protein 75D (ZNF75D), transcript variant 1, mRNA [NM_007131]	-1.1252898	0.014644525
A_23_P318581	*ENAH*	Homo sapiens enabled homolog (Drosophila) (ENAH), transcript variant 1, mRNA [NM_001008493]	-0.75619155	0.0035477025
A_33_P3329149	*ABI1*	Homo sapiens abl-interactor 1 (ABI1), transcript variant 1, mRNA [NM_005470]	-1.2049115	0.0071828146
A_33_P3482466	*CFAP97*	Homo sapiens cilia and flagella associated protein 97 (CFAP97), transcript variant 1, mRNA [NM_020827]	-0.7720135	0.009450744
A_32_P36694	*CDK13*	Homo sapiens cyclin-dependent kinase 13 (CDK13), transcript variant 2, mRNA [NM_031267]	1.700332	0.013426972
A_21_P0011605	*JAZF1*	Homo sapiens JAZF zinc finger 1 (JAZF1), mRNA [NM_175061]	-1.0611093	0.009383018
A_24_P273865	*CAPN10*	Homo sapiens calpain 10 (CAPN10), transcript variant 3, mRNA [NM_023085]	1.1510389	0.0038302224
A_33_P3228385	*AMZ2P1*	Homo sapiens archaelysin family metallopeptidase 2 pseudogene 1 (AMZ2P1), non-coding RNA [NR_026903]	-0.9234098	0.00943937
A_22_P00003666	*MED20*	Homo sapiens mediator complex subunit 20 (MED20), mRNA [NM_004275]	-0.7087674	0.011672903
A_23_P22263	*ATPIF1*	Homo sapiens ATPase inhibitory factor 1 (ATPIF1), transcript variant 1, mRNA [NM_016311]	-0.5923385	0.0024890031
A_33_P3278144	*BANP*	Homo sapiens BTG3 associated nuclear protein (BANP), transcript variant 2, mRNA [NM_079837]	-0.65538406	0.013029367
A_21_P0001773	*PCK2*	Homo sapiens phosphoenolpyruvate carboxykinase 2 (mitochondrial) (PCK2), transcript variant 2, mRNA [NM_001018073]	-0.5958023	0.015963586
A_24_P105298	*ANKRD42*	Homo sapiens ankyrin repeat domain 42 (ANKRD42), transcript variant 6, mRNA [NM_001300977]	-0.66934896	0.005057235
A_23_P88691	*LINC01119*	Homo sapiens long intergenic non-protein coding RNA 1119 (LINC01119), long non-coding RNA [NR_024452]	-1.3286839	0.0065033673
A_23_P203406	*H3F3A*	Homo sapiens H3 histone, family 3A (H3F3A), mRNA [NM_002107]	-0.6854708	0.0032831472
A_23_P64650	*CHRNA5*	Homo sapiens cholinergic receptor, nicotinic, alpha 5 (neuronal) (CHRNA5), mRNA [NM_000745]	-1.0872736	0.005920916

A_23_P14636	*GANAB*	Homo sapiens glucosidase, alpha; neutral AB (GANAB), transcript variant 3, mRNA [NM_198335]	-0.7833617	0.010665993
A_23_P136460	*TSFM*	Homo sapiens Ts translation elongation factor, mitochondrial (TSFM), transcript variant 2, mRNA [NM_005726]	-1.0389352	0.006937616
A_33_P3229958	*MFAP1*	Homo sapiens microfibrillar-associated protein 1 (MFAP1), mRNA [NM_005926]	-0.68528056	0.0076747234
A_33_P3280549	*FAM13B*	Homo sapiens family with sequence similarity 13, member B (FAM13B), transcript variant 1, mRNA [NM_016603]	-0.62379855	0.012878349
A_23_P24555	*SERPING1*	Homo sapiens serpin peptidase inhibitor, clade G (C1 inhibitor), member 1 (SERPING1), transcript variant 1, mRNA [NM_000062]	-1.2291281	0.012873199
A_23_P103398	*BCL11A*	Homo sapiens B-cell CLL/lymphoma 11A (zinc finger protein) (BCL11A), transcript variant 1, mRNA [NM_022893]	-2.2412724	0.007889792
A_24_P315921	*PHLDB1*	Homo sapiens pleckstrin homology-like domain, family B, member 1 (PHLDB1), transcript variant 1, mRNA [NM_015157]	1.0015056	0.01318143
A_23_P41854	*PSEN2*	Homo sapiens presenilin 2 (PSEN2), transcript variant 1, mRNA [NM_000447]	1.232518	0.008600396
A_33_P3278560	*lnc-PABPC4-2*	Homo sapiens cDNA FLJ36340 fis, clone THYMU2006468. [AK093659]	-0.7343622	0.012276114
A_23_P19313	*CARD6*	Homo sapiens caspase recruitment domain family, member 6 (CARD6), mRNA [NM_032587]	-0.71145177	0.015305683
A_22_P00017999	*ZIK1*	Homo sapiens zinc finger protein interacting with K protein 1 (ZIK1), mRNA [NM_001010879]	0.6081264	0.016416559
A_33_P3295200	*TBP*	Homo sapiens TATA box binding protein (TBP), transcript variant 1, mRNA [NM_003194]	-0.43988818	0.00793684
A_22_P00008745	*lnc-ZNF132-1*	Homo sapiens cDNA FLJ38721 fis, clone KIDNE2010052. [AK096040]	-1.1259649	0.012974829
A_23_P117068	*HAS1*	Homo sapiens hyaluronan synthase 1 (HAS1), transcript variant 1, mRNA [NM_001523]	4.1873975	0.0030069214
A_24_P98277	*TRAF3IP2-AS1*	Homo sapiens TRAF3IP2 antisense RNA 1 (TRAF3IP2-AS1), transcript variant 1, long non-coding RNA [NR_034108]	-1.5931379	0.006736978
A_33_P3531828	*SNRPF*	Homo sapiens small nuclear ribonucleoprotein polypeptide F (SNRPF), mRNA [NM_003095]	-0.41012433	0.004566164
A_23_P148916	*GOLGA7*	Homo sapiens golgin A7 (GOLGA7), transcript variant 2, mRNA [NM_001002296]	-0.677382	0.011403431
A_21_P0014428	*LARS*	Homo sapiens leucyl-tRNA synthetase (LARS), mRNA [NM_020117]	-1.1447262	0.0075928494
A_23_P148273	*OSBPL9*	Homo sapiens oxysterol binding protein-like 9 (OSBPL9), transcript variant 7, mRNA [NM_148909]	-1.0882969	0.005073928

A_22_P00016330	*LOC101927372*	PREDICTED: Homo sapiens uncharacterized LOC101927372 (LOC101927372), ncRNA [XR_244439]	-0.79352987	0.015948348
A_19_P00322183	*MAGT1*	Homo sapiens magnesium transporter 1 (MAGT1), mRNA [NM_032121]	-0.38555172	0.013706757
A_33_P3328445	*lnc-TMEM151A-1*	DA554227 HCHON2 Homo sapiens cDNA clone HCHON2004197 5', mRNA sequence [DA554227]	1.2024398	0.004832007
A_23_P3204	*lnc-NR5A2-1*	LNCipedia lincRNA (lnc-NR5A2-1), lincRNA [lnc-NR5A2-1:1]	1.6615729	0.00735003
A_33_P3348744	*ALOX12-AS1*	Homo sapiens cDNA FLJ90099 fis, clone HEMBA1006016. [AK074580]	-1.181501	0.010957296
A_23_P361419	*MAPK6*	Homo sapiens mitogen-activated protein kinase 6 (MAPK6), mRNA [NM_002748]	-0.96551126	0.0050847097
A_21_P0000477	*DEPDC1B*	Homo sapiens DEP domain containing 1B (DEPDC1B), transcript variant 1, mRNA [NM_018369]	-0.5652548	0.0056601833
A_33_P3367692	*C19orf47*	Homo sapiens chromosome 19 open reading frame 47 (C19orf47), transcript variant 1, mRNA [NM_001256440]	0.8875462	0.016644593
A_23_P114057	*SNORA11B*	Homo sapiens small nucleolar RNA, H/ACA box 11B (SNORA11B), small nucleolar RNA [NR_003709]	-0.4347473	0.007819978
A_23_P66158	*CFH*	Homo sapiens complement factor H (CFH), transcript variant 2, mRNA [NM_001014975]	1.7951612	0.011087365
A_23_P340318	*SEMA4C*	Homo sapiens sema domain, immunoglobulin domain (Ig), transmembrane domain (TM) and short cytoplasmic domain, (semaphorin) 4C (SEMA4C), mRNA [NM_017789]	-0.6561547	0.015141121
A_23_P63751	*KNOP1*	Homo sapiens lysine-rich nucleolar protein 1 (KNOP1), mRNA [NM_001012991]	-0.70705795	0.009002031
A_33_P3297050	*C11orf31*	Homo sapiens chromosome 11 open reading frame 31 (C11orf31), mRNA [NM_170746]	-0.6643148	0.003667498
A_33_P3251093	*PRDX3*	Homo sapiens peroxiredoxin 3 (PRDX3), transcript variant 1, mRNA [NM_006793]	-0.65071607	0.006818124
A_33_P3384835	*ALG2*	Homo sapiens ALG2, alpha-1,3/1,6-mannosyltransferase (ALG2), transcript variant 1, mRNA [NM_033087]	-0.5479603	0.005947757
A_23_P216630	*SLC36A1*	Homo sapiens solute carrier family 36 (proton/amino acid symporter), member 1 (SLC36A1), mRNA [NM_078483]	-1.023127	0.0058573913
A_23_P500601	*LOC101927100*	Homo sapiens uncharacterized LOC101927100 (LOC101927100), transcript variant 1, long non-coding RNA [NR_109874]	-1.4065171	0.008646254
A_21_P0013341	*SLC44A1*	Homo sapiens solute carrier family 44 (choline transporter), member 1 (SLC44A1), transcript variant 1, mRNA [NM_080546]	-0.62277865	0.010974453

A_33_P3318966	*TRIM4*	Homo sapiens tripartite motif containing 4 (TRIM4), transcript variant alpha, mRNA [NM_033017]	-0.90419817	0.011772038
A_33_P3377110	*METTL20*	Homo sapiens methyltransferase like 20 (METTL20), transcript variant 1, mRNA [NM_173802]	-0.74606526	0.011573572
A_23_P144877	*NDUFA4*	Homo sapiens NDUFA4, mitochondrial complex associated (NDUFA4), mRNA [NM_002489]	-0.5421908	0.0029471128
A_33_P3783235	*CYB561D1*	Homo sapiens cytochrome b561 family, member D1 (CYB561D1), transcript variant 5, mRNA [NM_001134404]	-1.2817359	0.007698106
A_32_P36235	*ATOX1*	Homo sapiens antioxidant 1 copper chaperone (ATOX1), mRNA [NM_004045]	-0.8321585	0.006352343
A_24_P940666	*IER2*	Homo sapiens immediate early response 2 (IER2), mRNA [NM_004907]	-0.5430116	0.005098191
A_23_P47034	*RDX*	Homo sapiens radixin (RDX), transcript variant 3, mRNA [NM_002906]	-0.72301954	0.010519517
A_23_P118150	*MED1*	Homo sapiens mediator complex subunit 1 (MED1), mRNA [NM_004774]	-0.36448148	0.0071450765
A_21_P0012935	*HHEX*	Homo sapiens hematopoietically expressed homeobox (HHEX), mRNA [NM_002729]	2.2744107	0.0031941293
A_23_P86252	*ARL6IP1*	Homo sapiens ADP-ribosylation factor-like 6 interacting protein 1 (ARL6IP1), mRNA [NM_015161]	-0.6276203	0.0068711066
A_21_P0002691	*XLOC_l2_012323*	BROAD Institute lincRNA (XLOC_l2_012323), lincRNA [TCONS_l2_00023270]	0.8087053	0.014536804
A_33_P3270102	*PIGC*	Homo sapiens phosphatidylinositol glycan anchor biosynthesis, class C (PIGC), transcript variant 2, mRNA [NM_002642]	-0.593626	0.011958462
A_33_P3291394	*lnc-KLF7-1*	LNCipedia lincRNA (lnc-KLF7-1), lincRNA [lnc-KLF7-1:6]	-1.2968605	0.00340924
A_23_P67618	*RNF126*	Homo sapiens ring finger protein 126 (RNF126), mRNA [NM_194460]	1.2127758	0.01317642
A_22_P00004297	*GPN1*	Homo sapiens GPN-loop GTPase 1 (GPN1), transcript variant 1, mRNA [NM_007266]	-0.6636676	0.008167038
A_24_P323815	*ZNF792*	Homo sapiens zinc finger protein 792 (ZNF792), mRNA [NM_175872]	1.6080711	0.0034766807
A_23_P429478	*CNPY3*	PREDICTED: Homo sapiens canopy FGF signaling regulator 3 (CNPY3), transcript variant X7, misc_RNA [XR_427823]	-0.77015	0.009938745
A_33_P3307735	*MYCBP2*	Homo sapiens MYC binding protein 2, E3 ubiquitin protein ligase (MYCBP2), mRNA [NM_015057]	-0.8206398	0.008570872
A_21_P0014683	*CCDC140*	Homo sapiens coiled-coil domain containing 140 (CCDC140), mRNA [NM_153038]	-2.206918	0.003365343
A_23_P390172	*OPHN1*	Homo sapiens oligophrenin 1 (OPHN1), mRNA [NM_002547]	-1.3096161	0.0037353234

A_21_P0014730	*LOC101928188*	PREDICTED: Homo sapiens uncharacterized LOC101928188 (LOC101928188), ncRNA [XR_243371]	1.3968521	0.015933463
A_22_P00024689	*RNASEL*	Homo sapiens ribonuclease L (2',5'-oligoisoadenylate synthetase-dependent) (RNASEL), mRNA [NM_021133]	-1.5366571	0.0031941293
A_33_P3309919	*lnc-C7orf23-1*	PREDICTED: Homo sapiens uncharacterized LOC101927420 (LOC101927420), mRNA [XM_006710189]	-0.8013549	0.01530907
A_32_P34387	*MALAT1*	Homo sapiens metastasis associated lung adenocarcinoma transcript 1 (non-protein coding) (MALAT1), long non-coding RNA [NR_002819]	0.8654818	0.0048641856
A_23_P167856	*RWDD1*	Homo sapiens RWD domain containing 1 (RWDD1), transcript variant 2, mRNA [NM_016104]	-1.0874641	0.007733715
A_32_P160883	*SPATA7*	Homo sapiens spermatogenesis associated 7 (SPATA7), transcript variant 1, mRNA [NM_018418]	-1.4122541	0.0038302224
A_32_P187599	*TMEM63B*	Homo sapiens transmembrane protein 63B (TMEM63B), mRNA [NM_018426]	0.9890365	0.006586824
A_23_P373927	*NEDD4*	Homo sapiens neural precursor cell expressed, developmentally down-regulated 4, E3 ubiquitin protein ligase (NEDD4), transcript variant 2, mRNA [NM_198400]	-0.5621307	0.0029691157
A_23_P44849	*SERBP1*	Homo sapiens SERPINE1 mRNA binding protein 1 (SERBP1), transcript variant 1, mRNA [NM_001018067]	-0.44257477	0.002419416
A_23_P344988	*ERP44*	endoplasmic reticulum protein 44 [Source:HGNC Symbol;Acc:HGNC:18311] [ENST00000262455]	-0.5777151	0.0062618116
A_33_P3359268	*KCTD13*	Homo sapiens potassium channel tetramerization domain containing 13 (KCTD13), transcript variant 1, mRNA [NM_178863]	0.6160472	0.011409725
A_33_P3299416	*ICK*	Homo sapiens intestinal cell (MAK-like) kinase (ICK), transcript variant 2, mRNA [NM_016513]	-1.8492204	0.004297874
A_19_P00812033	*HMG20B*	Homo sapiens high mobility group 20B (HMG20B), mRNA [NM_006339]	-0.7680397	0.007479662
A_23_P64204	*GRASP*	Homo sapiens GRP1 (general receptor for phosphoinositides 1)-associated scaffold protein (GRASP), transcript variant 1, mRNA [NM_181711]	-0.78089803	0.0075689107
A_23_P104146	*ZW10*	Homo sapiens zw10 kinetochore protein (ZW10), mRNA [NM_004724]	-0.8733255	0.008581556
A_23_P126908	*RELA*	Homo sapiens v-rel avian reticuloendotheliosis viral oncogene homolog A (RELA), transcript variant 1, mRNA [NM_021975]	0.5629805	0.014728144

A_23_P121716	*ZMYM4*	Homo sapiens zinc finger, MYM-type 4 (ZMYM4), mRNA [NM_005095]	-0.36946887	0.016931534
A_23_P49975	*TNFRSF14*	Homo sapiens tumor necrosis factor receptor superfamily, member 14 (TNFRSF14), transcript variant 1, mRNA [NM_003820]	-1.2098594	0.0072222846
A_23_P72668	*ANXA3*	Homo sapiens annexin A3 (ANXA3), mRNA [NM_005139]	0.7996266	0.010237655
A_24_P312325	*KRT10*	Homo sapiens keratin 10, type I (KRT10), mRNA [NM_000421]	-0.57309484	0.002466562
A_23_P143643	*SDPR*	Homo sapiens serum deprivation response (SDPR), mRNA [NM_004657]	-2.6148224	0.0035935708
A_23_P47991	*lnc-AF131215.3.1-1*	Homo sapiens mRNA for hypothetical protein (C8ORF15). [AJ312027]	0.9829893	0.004355716
A_23_P3212	*ASCC2*	Homo sapiens activating signal cointegrator 1 complex subunit 2 (ASCC2), transcript variant 1, mRNA [NM_032204]	0.79790914	0.008477025
A_33_P3258041	*MED13L*	Homo sapiens mediator complex subunit 13-like (MED13L), mRNA [NM_015335]	-1.2388058	0.0024890031
A_22_P00017678	*DTWD1*	Homo sapiens DTW domain containing 1 (DTWD1), transcript variant 1, mRNA [NM_020234]	-0.9866977	0.006573993
A_32_P37867	*GGPS1*	Homo sapiens geranylgeranyl diphosphate synthase 1 (GGPS1), transcript variant 2, mRNA [NM_001037277]	-0.92801714	0.0053083743
A_22_P00011747	*LOC101927885*	PREDICTED: Homo sapiens uncharacterized LOC101927885 (LOC101927885), ncRNA [XR_245288]	-1.9186548	0.0044707763
A_24_P225604	*KIAA1644*	Homo sapiens KIAA1644 (KIAA1644), mRNA [NM_001099294]	1.0830153	0.009795713
A_23_P154526	*lnc-PERP-2*	Q6DQW2_MANSE (Q6DQW2) CAPA, partial (7%) [THC2514262]	-0.8938156	0.012334684
A_24_P305662	*DNAJC10*	Homo sapiens DnaJ (Hsp40) homolog, subfamily C, member 10 (DNAJC10), transcript variant 1, mRNA [NM_018981]	-0.8947129	0.005734989
A_33_P3392192	*GRB14*	Homo sapiens growth factor receptor-bound protein 14 (GRB14), transcript variant 1, mRNA [NM_004490]	-1.3262674	0.011024329
A_21_P0013921	*TRMT2A*	Homo sapiens tRNA methyltransferase 2 homolog A (S. cerevisiae) (TRMT2A), transcript variant 1, mRNA [NM_022727]	0.79548514	0.0154827535
A_32_P74366	*PPCS*	Homo sapiens phosphopantothenoylcysteine synthetase (PPCS), transcript variant 4, mRNA [NM_001287507]	-0.9151547	0.009765222
A_33_P3416414	*NAA11*	Homo sapiens N(alpha)-acetyltransferase 11, NatA catalytic subunit (NAA11), mRNA [NM_032693]	0.7809856	0.01217032

A_33_P3215277	*VCPIP1*	valosin containing protein (p97)/p47 complex interacting protein 1 [Source:HGNC Symbol;Acc:HGNC:30897] [ENST00000310421]	-0.872108	0.014548385
A_33_P3284919	*TTBK2*	Homo sapiens tau tubulin kinase 2 (TTBK2), mRNA [NM_173500]	-1.0390623	0.004834548
A_23_P334282	*COPS8*	Homo sapiens COP9 signalosome subunit 8 (COPS8), transcript variant 2, mRNA [NM_198189]	-0.847108	0.006155321
A_19_P00812050	*SEMA6C*	Homo sapiens sema domain, transmembrane domain (TM), and cytoplasmic domain, (semaphorin) 6C (SEMA6C), transcript variant 1, mRNA [NM_001178061]	-0.6826976	0.010447919
A_24_P706752	*BMP2K*	Homo sapiens BMP2 inducible kinase (BMP2K), transcript variant 2, mRNA [NM_017593]	-0.5115992	0.010383267
A_32_P23624	*lnc-TBC1D19-3*	LNCipedia lincRNA (lnc-TBC1D19-3), lincRNA [lnc-TBC1D19-3:1]	0.6348371	0.010138025
A_33_P3272169	*PLA2G12A*	Homo sapiens phospholipase A2, group XIIA (PLA2G12A), mRNA [NM_030821]	-0.72357243	0.009010301
A_33_P3220837	*ABCB10*	Homo sapiens ATP-binding cassette, sub-family B (MDR/TAP), member 10 (ABCB10), mRNA [NM_012089]	-0.5200792	0.010971502
A_32_P231617	*CLIP2*	Homo sapiens CAP-GLY domain containing linker protein 2 (CLIP2), transcript variant 1, mRNA [NM_003388]	0.9248818	0.0057417424
A_33_P3264612	*MAFB*	Homo sapiens v-maf avian musculoaponeurotic fibrosarcoma oncogene homolog B (MAFB), mRNA [NM_005461]	-297936	0.0026950072
A_24_P76546	*TM4SF1*	Homo sapiens transmembrane 4 L six family member 1 (TM4SF1), mRNA [NM_014220]	1.039844	0.003422245
A_22_P00006607	*TPCN2*	Homo sapiens two pore segment channel 2 (TPCN2), mRNA [NM_139075]	-0.6877808	0.013806441
A_22_P00011288	*WASF2*	WAS protein family, member 2 [Source:HGNC Symbol;Acc:HGNC:12733] [ENST00000618852]	0.52803683	0.014548385
A_32_P96807	*lnc-FOXA1-2*	LNCipedia lincRNA (lnc-FOXA1-2), lincRNA [lnc-FOXA1-2:1]	1.7762562	0.0046675983
A_32_P149251	*lnc-OR7C2-1*	LNCipedia lincRNA (lnc-OR7C2-1), lincRNA [lnc-OR7C2-1:1]	0.80205363	0.0075689107
A_23_P201790	*RC3H1*	Homo sapiens ring finger and CCCH-type domains 1 (RC3H1), transcript variant 1, mRNA [NM_001300850]	-0.53019327	0.015419179
A_22_P00003481	*DNAJC18*	Homo sapiens DnaJ (Hsp40) homolog, subfamily C, member 18 (DNAJC18), mRNA [NM_152686]	-1.1675345	0.009763898
A_23_P18205	*PPP1R12B*	Homo sapiens protein phosphatase 1, regulatory subunit 12B (PPP1R12B), transcript variant 1, mRNA [NM_002481]	-19896834	0.004169345
A_23_P30956	*CCDC80*	coiled-coil domain containing 80 [Source:HGNC Symbol;Acc:HGNC:30649] [ENST00000473959]	-14105768	0.003190129

A_33_P3350413	*RAD54L2*	Homo sapiens RAD54-like 2 (S. cerevisiae) (RAD54L2), mRNA [NM_015106]	0.7380054	0.005057235
A_21_P0000360	*UFL1*	Homo sapiens UFM1-specific ligase 1 (UFL1), mRNA [NM_015323]	-0.6570747	0.009468359
A_23_P142634	*SVIL*	Homo sapiens cDNA, FLJ17314. [AK310272]	-0.6125934	0.010383267
A_24_P90878	*SNORA77*	Homo sapiens small nucleolar RNA, H/ACA box 77 (SNORA77), small nucleolar RNA [NR_003019]	-0.7722089	0.012156404
A_33_P3275290	*METTL5*	Homo sapiens methyltransferase like 5 (METTL5), transcript variant 1, mRNA [NM_014168]	-0.728605	0.010976066
A_23_P360754	*THUMPD1*	Homo sapiens THUMP domain containing 1 (THUMPD1), mRNA [NM_017736]	-0.9536777	0.0056962976
A_23_P203445	*GLT8D1*	Homo sapiens glycosyltransferase 8 domain containing 1 (GLT8D1), transcript variant 3, mRNA [NM_001010983]	-0.6548886	0.016716918
A_23_P54006	*ADAMTS4*	Homo sapiens ADAM metallopeptidase with thrombospondin type 1 motif, 4 (ADAMTS4), mRNA [NM_005099]	-0.9513588	0.0043846127
A_23_P31109	*UEVLD*	Homo sapiens UEV and lactate/malate dehyrogenase domains (UEVLD), transcript variant 1, mRNA [NM_001040697]	-0.5884773	0.006573993
A_33_P3218584	*HECTD1*	Homo sapiens HECT domain containing E3 ubiquitin protein ligase 1 (HECTD1), mRNA [NM_015382]	-0.8682425	0.004561848
A_23_P200551	*GINM1*	Homo sapiens glycoprotein integral membrane 1 (GINM1), mRNA [NM_138785]	-0.9057522	0.010995328
A_32_P4626	*POP5*	Homo sapiens processing of precursor 5, ribonuclease P/MRP subunit (S. cerevisiae) (POP5), transcript variant 1, mRNA [NM_015918]	-0.23766278	0.0074993544
A_23_P306507	*USP48*	Homo sapiens ubiquitin specific peptidase 48 (USP48), transcript variant 1, mRNA [NM_032236]	-0.7624328	0.008755959
A_33_P3357322	*LOC101928076*	PREDICTED: Homo sapiens uncharacterized LOC101928076 (LOC101928076), transcript variant X1, ncRNA [XR_241970]	-26434278	0.0025001846
A_23_P404481	*KRAS*	Homo sapiens Kirsten rat sarcoma viral oncogene homolog (KRAS), transcript variant a, mRNA [NM_033360]	-10122467	0.01210552
A_23_P19482	*SMC2*	Homo sapiens structural maintenance of chromosomes 2 (SMC2), transcript variant 1, mRNA [NM_001042550]	-0.5933799	0.008649646
A_33_P3370787	*S1PR1*	Homo sapiens sphingosine-1-phosphate receptor 1 (S1PR1), mRNA [NM_001400]	-0.987271	0.0036861196
A_23_P419714	*DDAH2*	Homo sapiens dimethylarginine dimethylaminohydrolase 2 (DDAH2), transcript variant 2, mRNA [NM_013974]	-1.7013502	0.0027348737

A_23_P201376	*EPHB2*	Homo sapiens EPH receptor B2 (EPHB2), transcript variant 2, mRNA [NM_004442]	1.6284063	0.0065142275
A_23_P101551	*BTBD11*	Homo sapiens BTB (POZ) domain containing 11 (BTBD11), transcript variant a, mRNA [NM_001018072]	1.3490319	0.011517759
A_33_P3273364	*SSX2IP*	Homo sapiens synovial sarcoma, X breakpoint 2 interacting protein (SSX2IP), transcript variant 5, mRNA [NM_014021]	0.39102405	0.0043125506
A_33_P3518572	*BCAT2*	Homo sapiens branched chain amino-acid transaminase 2, mitochondrial (BCAT2), transcript variant a, mRNA [NM_001190]	-1.1400115	0.0068771048
A_33_P3362296	*YIPF2*	Homo sapiens Yip1 domain family, member 2 (YIPF2), mRNA [NM_024029]	-1.1437142	0.009574714
A_23_P69670	*LINC00941*	Homo sapiens long intergenic non-protein coding RNA 941 (LINC00941), long non-coding RNA [NR_040245]	0.7497611	0.016766192
A_24_P216654	*KCNG1*	PREDICTED: Homo sapiens potassium voltage-gated channel, subfamily G, member 1 (KCNG1), transcript variant X2, mRNA [XM_006723786]	-1.1906928	0.0055197445
A_33_P3420402	*BLOC1S4*	Homo sapiens biogenesis of lysosomal organelles complex-1, subunit 4, cappuccino (BLOC1S4), mRNA [NM_018366]	-0.6240114	0.0072350414
A_33_P3227731	*SOAT1*	Homo sapiens sterol O-acyltransferase 1 (SOAT1), transcript variant 1, mRNA [NM_003101]	-0.7125712	0.012864231
A_23_P153286	*HACE1*	Homo sapiens HECT domain and ankyrin repeat containing E3 ubiquitin protein ligase 1 (HACE1), transcript variant 1, mRNA [NM_020771]	-0.5996326	0.0072350414
A_33_P3270863	*ZNF234*	Homo sapiens zinc finger protein 234 (ZNF234), transcript variant 1, mRNA [NM_006630]	-0.90388006	0.004522109
A_22_P00002634	*ATXN3*	Homo sapiens ataxin 3 (ATXN3), transcript variant reference, mRNA [NM_004993]	-1.5042604	0.0037583082
A_23_P213336	*XDH*	Homo sapiens xanthine dehydrogenase (XDH), mRNA [NM_000379]	1.7621378	0.0065613114
A_23_P200096	*lnc-C1orf177-1*	LNCipedia lincRNA (lnc-C1orf177-1), lincRNA [lnc-C1orf177-1:1]	0.43381515	0.016549561
A_22_P00015379	*FGF1*	Homo sapiens fibroblast growth factor 1 (acidic) (FGF1), transcript variant 1, mRNA [NM_000800]	-1.7870495	0.0044240346
A_23_P215051	*SPSB1*	Homo sapiens splA/ryanodine receptor domain and SOCS box containing 1 (SPSB1), mRNA [NM_025106]	0.8518053	0.014162805
A_32_P94722	*LOC101929494*	PREDICTED: Homo sapiens uncharacterized LOC101929494 (LOC101929494), transcript variant X3, ncRNA [XR_424822]	-1.7502997	0.013471701

A_33_P3382924	*ECHDC1*	Homo sapiens ethylmalonyl-CoA decarboxylase 1 (ECHDC1), transcript variant 2, mRNA [NM_018479]	-0.7426738	0.0035935708
A_33_P3325018	*BTBD9*	Homo sapiens BTB (POZ) domain containing 9 (BTBD9), transcript variant 1, mRNA [NM_052893]	-1.0157169	0.007867022
A_23_P21134	*SPARC*	Homo sapiens secreted protein, acidic, cysteine-rich (osteonectin) (SPARC), mRNA [NM_003118]	-1.3592739	0.0031827379
A_23_P205697	*ERLEC1*	Homo sapiens endoplasmic reticulum lectin 1 (ERLEC1), transcript variant 1, mRNA [NM_015701]	-0.49353078	0.010589583
A_33_P3234667	*DDIT3*	Homo sapiens DNA-damage-inducible transcript 3 (DDIT3), transcript variant 5, mRNA [NM_004083]	-1.6976739	0.0040017776
A_23_P203305	*DLST*	Homo sapiens dihydrolipoamide S-succinyltransferase (E2 component of 2-oxo-glutarate complex) (DLST), transcript variant 1, mRNA [NM_001933]	-0.6655927	0.0064972234
A_24_P945113	*ZKSCAN1*	Homo sapiens zinc finger with KRAB and SCAN domains 1 (ZKSCAN1), transcript variant 2, mRNA [NM_001287054]	-0.913846	0.0076779537
A_23_P170467	*IMMP1L*	IMP1 inner mitochondrial membrane peptidase-like (S. cerevisiae) [Source:HGNC Symbol;Acc:HGNC:26317] [ENST00000278200]	-0.48031157	0.014362992
A_23_P33607	*ACVRL1*	Homo sapiens activin A receptor type II-like 1 (ACVRL1), transcript variant 1, mRNA [NM_000020]	-1.8506355	0.0050360644
A_33_P3293254	*USP3*	Homo sapiens ubiquitin specific peptidase 3 (USP3), transcript variant 1, mRNA [NM_006537]	-0.40361905	0.013367055
A_23_P83278	*TEFM*	Homo sapiens transcription elongation factor, mitochondrial (TEFM), mRNA [NM_024683]	-0.5525632	0.0032869747
A_23_P167401	*GPSM1*	Homo sapiens G-protein signaling modulator 1 (GPSM1), transcript variant 2, mRNA [NM_015597]	1.390911	0.012240551
A_24_P56130	*CHMP5*	Homo sapiens charged multivesicular body protein 5 (CHMP5), transcript variant 1, mRNA [NM_016410]	-1.0266855	0.0054708454
A_24_P257348	*PCDHB11*	Homo sapiens protocadherin beta 11 (PCDHB11), mRNA [NM_018931]	1.5928264	0.012477395
A_23_P81650	*MYL6*	Homo sapiens myosin, light chain 6, alkali, smooth muscle and non-muscle (MYL6), transcript variant 2, mRNA [NM_079423]	-0.45037812	0.008048185
A_33_P3216994	*ARL6IP5*	Homo sapiens ADP-ribosylation factor-like 6 interacting protein 5 (ARL6IP5), mRNA [NM_006407]	-1.1445874	0.0029691157

A_33_P3317628	*C5orf15*	Homo sapiens chromosome 5 open reading frame 15 (C5orf15), mRNA [NM_020199]	-0.92715645	0.004026994
A_33_P3226605	*HERC4*	Homo sapiens HECT and RLD domain containing E3 ubiquitin protein ligase 4 (HERC4), transcript variant 5, mRNA [NM_001278187]	-0.61990356	0.01342566
A_23_P156880	*PKP3*	Homo sapiens plakophilin 3 (PKP3), transcript variant 1, mRNA [NM_007183]	1.7901803	0.0052438728
A_24_P64329	*PSIP1*	Homo sapiens PC4 and SFRS1 interacting protein 1 (PSIP1), transcript variant 2, mRNA [NM_033222]	-0.6771338	0.007989236
A_32_P50924	*ENPP1*	Homo sapiens ectonucleotide pyrophosphatase/phosphodiesterase 1 (ENPP1), mRNA [NM_006208]	-1.0160986	0.005512448
A_24_P280926	*STK32C*	Homo sapiens serine/threonine kinase 32C (STK32C), mRNA [NM_173575]	1.0759556	0.013050359
A_23_P129466	*HNRNPA1L2*	Homo sapiens heterogeneous nuclear ribonucleoprotein A1-like 2 (HNRNPA1L2), transcript variant 1, mRNA [NM_001011724]	-0.3390872	0.009630487
A_24_P174367	*ATF7IP2*	Homo sapiens activating transcription factor 7 interacting protein 2 (ATF7IP2), transcript variant 1, mRNA [NM_024997]	1.0643884	0.0035939496
A_23_P94636	*XLOC_l2_002033*	BROAD Institute lincRNA (XLOC_l2_002033), lincRNA [TCONS_l2_00003643]	2.0345297	0.005595418
A_33_P3390032	*PPP1R2*	Homo sapiens protein phosphatase 1, regulatory (inhibitor) subunit 2 (PPP1R2), transcript variant 2, mRNA [NM_006241]	-0.9914002	0.0049172705
A_22_P00023999	*RC3H2*	Homo sapiens ring finger and CCCH-type domains 2 (RC3H2), transcript variant 2, mRNA [NM_018835]	-0.6510001	0.016171155
A_33_P3296497	*EXOC7*	Homo sapiens exocyst complex component 7 (EXOC7), transcript variant 4, mRNA [NM_001145297]	-0.9034876	0.0141841015
A_21_P0011311	*lnc-C1orf31-1*	LNCipedia lincRNA (lnc-C1orf31-1), lincRNA [lnc-C1orf31-1:1]	0.5004602	0.007740612
A_33_P3326733	*PTPRK*	protein tyrosine phosphatase, receptor type, K [Source:HGNC Symbol;Acc:HGNC:9674] [ENST00000495748]	0.686907	0.008427824
A_23_P73660	*GOLGA2P6*	Homo sapiens golgin A2 pseudogene 6 (GOLGA2P6), non-coding RNA [NR_120609]	-0.79628134	0.016101176
A_23_P126706	*TRAPPC2*	Homo sapiens trafficking protein particle complex 2 (TRAPPC2), transcript variant 1, mRNA [NM_001011658]	-0.86430943	0.011377638
A_24_P937855	*SNX21*	Homo sapiens sorting nexin family member 21 (SNX21), transcript variant 4, mRNA [NM_001042633]	-1.5128651	0.005192162
A_24_P929369	*ANGPTL1*	Homo sapiens angiopoietin-like 1 (ANGPTL1), mRNA [NM_004673]	-1.0862304	0.013537862

A_24_P200942	*SIKE1*	Homo sapiens suppressor of IKBKE 1 (SIKE1), transcript variant 1, mRNA [NM_001102396]	-0.49000555	0.011409725
A_23_P63829	*AP4E1*	Homo sapiens adaptor-related protein complex 4, epsilon 1 subunit (AP4E1), transcript variant 1, mRNA [NM_007347]	-0.753844	0.01368361
A_23_P88909	*TSC22D4*	Homo sapiens TSC22 domain family, member 4 (TSC22D4), transcript variant 1, mRNA [NM_030935]	0.99628097	0.006573993
A_33_P3330716	*HSPA14*	Homo sapiens heat shock 70kDa protein 14 (HSPA14), transcript variant 1, mRNA [NM_016299]	-0.6743367	0.008717397
A_21_P0014870	*SYNGR3*	Homo sapiens synaptogyrin 3 (SYNGR3), mRNA [NM_004209]	1.8517267	0.013936194
A_23_P312174	*INO80D*	Homo sapiens INO80 complex subunit D (INO80D), mRNA [NM_017759]	-0.74930024	0.0039486005
A_33_P3293202	*ALMS1*	Homo sapiens Alstrom syndrome 1 (ALMS1), mRNA [NM_015120]	-0.8427746	0.007683084
A_22_P00024793	*SMAGP*	Homo sapiens small cell adhesion glycoprotein (SMAGP), transcript variant 1, mRNA [NM_001031628]	1.1114874	0.0064857528
A_33_P3427102	*C8orf37*	Homo sapiens chromosome 8 open reading frame 37 (C8orf37), mRNA [NM_177965]	-1.288642	0.0056932163
A_21_P0002502	*TMOD3*	tropomodulin 3 (ubiquitous) [Source:HGNC Symbol;Acc:HGNC:11873] [ENST00000308580]	-0.7163864	0.008289568
A_33_P3278265	*TTL*	Homo sapiens tubulin tyrosine ligase (TTL), mRNA [NM_153712]	-0.6007395	0.0036402496
A_24_P148026	*lnc-WDR33-1*	LNCipedia lincRNA (lnc-WDR33-1), lincRNA [lnc-WDR33-1:1]	0.7132211	0.0072714784
A_24_P345209	*PYROXD1*	Homo sapiens pyridine nucleotide-disulphide oxidoreductase domain 1 (PYROXD1), mRNA [NM_024854]	-0.66492087	0.006266305
A_33_P3263379	*lnc-C1orf86-1*	PREDICTED: Homo sapiens uncharacterized LOC100506504 (LOC100506504), mRNA [XM_006710074]	0.83695066	0.009604172
A_23_P18641	*DYRK3*	Homo sapiens dual-specificity tyrosine-(Y)-phosphorylation regulated kinase 3 (DYRK3), transcript variant 2, mRNA [NM_001004023]	-0.7443505	0.015731523
A_33_P3212432	*SNORD17*	Homo sapiens small nucleolar RNA, C/D box 17 (SNORD17), small nucleolar RNA [NR_003045]	0.582871	0.008835937
A_21_P0000124	*SNX25*	Homo sapiens sorting nexin 25 (SNX25), mRNA [NM_031953]	-0.78510237	0.00690275
A_33_P3773261	*DENND1B*	Homo sapiens DENN/MADD domain containing 1B (DENND1B), transcript variant 4, mRNA [NM_001195216]	-1.2587188	0.010550066
A_23_P32785	*ERRFI1*	Homo sapiens ERBB receptor feedback inhibitor 1 (ERRFI1), mRNA [NM_018948]	0.567976	0.005303325

A_24_P925314	*NQO2*	NAD(P)H dehydrogenase, quinone 2 [Source:HGNC Symbol;Acc:HGNC:7856] [ENST00000380455]	0.3451227	0.0102413045
A_33_P3379941	*C3orf38*	Homo sapiens chromosome 3 open reading frame 38 (C3orf38), mRNA [NM_173824]	-0.90979904	0.009711265
A_32_P104478	*GM2A*	Homo sapiens GM2 ganglioside activator (GM2A), transcript variant 1, mRNA [NM_000405]	-0.7500031	0.0054418826
A_23_P205778	*HMGXB3*	Homo sapiens HMG box domain containing 3 (HMGXB3), mRNA [NM_014983]	-0.5759092	0.013441296
A_23_P27315	*FGD6*	Homo sapiens FYVE, RhoGEF and PH domain containing 6 (FGD6), mRNA [NM_018351]	-0.79678077	0.006503996
A_21_P0009768	*GNB5*	Homo sapiens guanine nucleotide binding protein (G protein), beta 5 (GNB5), transcript variant 2, mRNA [NM_016194]	-1.2035835	0.0044074184
A_24_P94651	*EMILIN2*	Homo sapiens elastin microfibril interfacer 2 (EMILIN2), mRNA [NM_032048]	-3.6823947	0.0025001846
A_23_P9458	*lnc-ZNF404-1*	LNCipedia lincRNA (lnc-ZNF404-1), lincRNA [lnc-ZNF404-1:4]	-1.4106307	0.016205462
A_33_P3377691	*ARIH2*	Homo sapiens ariadne RBR E3 ubiquitin protein ligase 2 (ARIH2), mRNA [NM_006321]	-0.48989534	0.0101569565
A_21_P0000199	*POLR1E*	Homo sapiens polymerase (RNA) I polypeptide E, 53kDa (POLR1E), transcript variant 1, mRNA [NM_022490]	0.57626414	0.00906047
A_23_P253052	*C4orf46*	Homo sapiens chromosome 4 open reading frame 46 (C4orf46), transcript variant 1, mRNA [NM_001008393]	-0.4648487	0.0035939496
A_32_P214340	*HOXA6*	Homo sapiens homeobox A6 (HOXA6), mRNA [NM_024014]	1.5619371	0.011212515
A_23_P404685	*CD99L2*	Homo sapiens CD99 molecule-like 2 (CD99L2), transcript variant 1, mRNA [NM_031462]	-1.0837263	0.0056098294
A_33_P3383524	*lnc-AKIRIN1-1*	AF179597 connexin 59 {Homo sapiens} (exp=-1; wgp=0; cg=0), partial (10%) [THC2681034]	-0.6713115	0.016076444
A_24_P390928	*LCE1A*	Homo sapiens late cornified envelope 1A (LCE1A), mRNA [NM_178348]	0.69514245	0.0062664533
A_23_P35414	*LINC00923*	Homo sapiens long intergenic non-protein coding RNA 923 (LINC00923), transcript variant 1, long non-coding RNA [NR_024172]	1.708652	0.007875496
A_23_P256231	*TRAPPC6A*	Homo sapiens trafficking protein particle complex 6A (TRAPPC6A), transcript variant 1, mRNA [NM_024108]	-1.1705416	0.006807964
A_23_P80626	*PPP1R3C*	Homo sapiens protein phosphatase 1, regulatory subunit 3C (PPP1R3C), mRNA [NM_005398]	-1.4988699	0.0054341624
A_33_P3373437	*FBXO30*	Homo sapiens F-box protein 30 (FBXO30), mRNA [NM_032145]	-0.45004278	0.009938745
A_23_P167030	*PRRT3*	Homo sapiens proline-rich transmembrane protein 3 (PRRT3), mRNA [NM_207351]	-0.88352126	0.014864522

A_23_P146077	*PTH1R*	Homo sapiens parathyroid hormone 1 receptor (PTH1R), transcript variant 1, mRNA [NM_000316]	-1.7592639	0.0056239334
A_22_P00024433	*DDX17*	Homo sapiens DEAD (Asp-Glu-Ala-Asp) box helicase 17 (DDX17), transcript variant 1, mRNA [NM_006386]	-0.7379212	0.010427376
A_23_P364890	*ZNF395*	Homo sapiens zinc finger protein 395 (ZNF395), mRNA [NM_018660]	-1.4729676	0.010150118
A_23_P132226	*SASS6*	Homo sapiens spindle assembly 6 homolog (C. elegans) (SASS6), mRNA [NM_194292]	-0.8008221	0.014416893
A_24_P709377	*RNF40*	Homo sapiens ring finger protein 40, E3 ubiquitin protein ligase (RNF40), transcript variant 1, mRNA [NM_014771]	1.3971263	0.0029471128
A_23_P40315	*TPST2*	Homo sapiens tyrosylprotein sulfotransferase 2 (TPST2), transcript variant 1, mRNA [NM_001008566]	-1.1702769	0.005428736
A_33_P6809500	*PAX8-AS1*	Homo sapiens PAX8 antisense RNA 1 (PAX8-AS1), transcript variant 1, long non-coding RNA [NR_015377]	-1.0850753	0.00910823
A_33_P3364869	*DZANK1*	Homo sapiens double zinc ribbon and ankyrin repeat domains 1 (DZANK1), mRNA [NM_001099407]	-1.3299583	0.01147035
A_23_P209347	*LOC100506844*	Homo sapiens uncharacterized LOC100506844 (LOC100506844), long non-coding RNA [NR_038269]	-0.9743557	0.009794768
A_23_P85969	*NAMPT*	Homo sapiens cDNA FLJ13279 fis, clone OVARC1001055, moderately similar to PRE-B CELL ENHANCING FACTOR PRECURSOR. [AK023341]	3.4814236	0.0026869643
A_33_P3225983	*ANKRD44*	Homo sapiens ankyrin repeat domain 44 (ANKRD44), transcript variant B, mRNA [NM_153697]	-1.7596321	0.003703558
A_23_P43164	*ZNF326*	Homo sapiens zinc finger protein 326 (ZNF326), transcript variant 1, mRNA [NM_182976]	-0.758226	0.009848541
A_23_P207299	*SULF1*	Homo sapiens sulfatase 1 (SULF1), transcript variant 3, mRNA [NM_015170]	-0.72712713	0.006474282
A_21_P0009341	*DUSP11*	Homo sapiens dual specificity phosphatase 11 (RNA/RNP complex 1-interacting) (DUSP11), mRNA [NM_003584]	-0.79283524	0.005861645
A_22_P00009227	*RNFT1*	Homo sapiens ring finger protein, transmembrane 1 (RNFT1), mRNA [NM_016125]	-0.7728575	0.010115223
A_23_P130653	*WFDC21P*	Homo sapiens WAP four-disulfide core domain 21, pseudogene (WFDC21P), non-coding RNA [NR_030732]	0.5841305	0.012532662

A_23_P106544	*PCOLCE-AS1*	Homo sapiens PCOLCE antisense RNA 1 (PCOLCE-AS1), long non-coding RNA [NR_038910]	0.914241	0.00782212
A_22_P00007826	*RTBDN*	Homo sapiens retbindin (RTBDN), transcript variant 2, mRNA [NM_031429]	1.0160339	0.009271572
A_33_P3419334	*CMC2*	Homo sapiens C-x(9)-C motif containing 2 (CMC2), mRNA [NM_020188]	-0.5134151	0.016617304
A_23_P253200	*lnc-HPD-1*	LNCipedia lincRNA (lnc-HPD-1), lincRNA [lnc-HPD-1:1]	0.8375599	0.0130220065
A_32_P181638	*GNAI3*	Homo sapiens guanine nucleotide binding protein (G protein), alpha inhibiting activity polypeptide 3 (GNAI3), mRNA [NM_006496]	-0.9015519	0.0039659217
A_24_P267686	*RPL15*	Homo sapiens ribosomal protein L15 (RPL15), transcript variant 1, mRNA [NM_002948]	-0.49790433	0.0056601833
A_23_P110052	*BVES*	Homo sapiens blood vessel epicardial substance (BVES), transcript variant B, mRNA [NM_147147]	-0.85776395	0.00856985
A_24_P63522	*FOXL2*	Homo sapiens forkhead box L2 (FOXL2), mRNA [NM_023067]	-4.97773	0.002419416
A_21_P0014829	*lnc-SLC22A12-2*	LNCipedia lincRNA (lnc-SLC22A12-2), lincRNA [lnc-SLC22A12-2:1]	0.7953123	0.008967042
A_23_P11331	*HMGCS1*	Homo sapiens 3-hydroxy-3-methylglutaryl-CoA synthase 1 (soluble) (HMGCS1), transcript variant 2, mRNA [NM_002130]	-0.87907	0.0059821224
A_23_P399078	*TCEAL8*	Homo sapiens transcription elongation factor A (SII)-like 8 (TCEAL8), transcript variant 1, mRNA [NM_153333]	-0.78820515	0.0029471128
A_23_P144999	*C17orf53*	Homo sapiens chromosome 17 open reading frame 53 (C17orf53), transcript variant 1, mRNA [NM_024032]	1.2288823	0.0047066417
A_21_P0000098	*TIMP3*	Homo sapiens TIMP metallopeptidase inhibitor 3 (TIMP3), mRNA [NM_000362]	-0.8789971	0.0043857186
A_21_P0000087	*RAPGEF6*	Homo sapiens Rap guanine nucleotide exchange factor (GEF) 6 (RAPGEF6), transcript variant 2, mRNA [NM_016340]	-0.6979311	0.011760672
A_19_P00804417	*CARD8*	Homo sapiens caspase recruitment domain family, member 8 (CARD8), transcript variant 6, mRNA [NM_001184904]	-1.0617229	0.007498967
A_33_P3312735	*MCPH1*	Homo sapiens microcephalin 1 (MCPH1), transcript variant 2, mRNA [NM_001172574]	-0.6207722	0.0069250474
A_23_P74928	*PGAP1*	Homo sapiens post-GPI attachment to proteins 1 (PGAP1), mRNA [NM_024989]	-0.82648206	0.007479662
A_33_P3410296	*ZFP91*	Homo sapiens ZFP91 zinc finger protein (ZFP91), transcript variant 1, mRNA [NM_053023]	-1.0531603	0.0042136866
A_23_P414252	*MR1*	Homo sapiens major histocompatibility complex, class I-related (MR1), transcript variant 1, mRNA [NM_001531]	-0.8956996	0.013011981

A_19_P00318559	*DPYSL4*	Homo sapiens dihydropyrimidinase-like 4 (DPYSL4), mRNA [NM_006426]	0.95359033	0.0070101186
A_24_P98263	*SNX8*	Homo sapiens cDNA, FLJ96764, highly similar to Homo sapiens sorting nexin 8 (SNX8), mRNA. [AK315670]	1.0734372	0.012368307
A_24_P345993	*LINC01021*	long intergenic non-protein coding RNA 1021 [Source:HGNC Symbol;Acc:HGNC:48995] [ENST00000512067]	-1.9728364	0.0041847117
A_23_P52127	*EBAG9*	Homo sapiens estrogen receptor binding site associated, antigen, 9 (EBAG9), transcript variant 1, mRNA [NM_004215]	-0.7619178	0.0061608143
A_22_P00022560	*CANX*	Homo sapiens calnexin (CANX), transcript variant 1, mRNA [NM_001746]	-0.7717498	0.0135640865
A_33_P3246543	*ACBD6*	Homo sapiens acyl-CoA binding domain containing 6 (ACBD6), mRNA [NM_032360]	-0.8921433	0.0112419
A_22_P00009719	*lnc-MUSK-2*	Homo sapiens cDNA FLJ37814 fis, clone BRSSN2002859. [AK095133]	1.7498575	0.0057476712
A_33_P3405334	*MAT2B*	Homo sapiens methionine adenosyltransferase II, beta (MAT2B), transcript variant 2, mRNA [NM_182796]	-0.86098236	0.011193861
A_21_P0006215	*GM2A*	Homo sapiens GM2 ganglioside activator (GM2A), transcript variant 1, mRNA [NM_000405]	-0.9432942	0.006812377
A_23_P212552	*MAGI2-IT1*	Homo sapiens platelet receptor for type III collagen mRNA, partial cds. [AF521131]	0.9859098	0.004928666
A_22_P00015747	*lnc-DEC1-3*	LNCipedia lincRNA (lnc-DEC1-3), lincRNA [lnc-DEC1-3:1]	1.2715313	0.0047356286
A_33_P3242136	*TBL1XR1*	Homo sapiens transducin (beta)-like 1 X-linked receptor 1 (TBL1XR1), mRNA [NM_024665]	-0.5343633	0.00945292
A_23_P151280	*lnc-TAF15-1*	BX103036 Soares_testis_NHT Homo sapiens cDNA clone IMAGp998A114170, mRNA sequence [BX103036]	5.340726	0.0029015075
A_33_P3299754	*FGF1*	Homo sapiens fibroblast growth factor 1 (acidic) (FGF1), transcript variant 8, mRNA [NM_001257206]	0.91919947	0.010520434
A_33_P3494875	*COX14*	Homo sapiens COX14 cytochrome c oxidase assembly factor (COX14), transcript variant 1, mRNA [NM_032901]	-1.115127	0.0035238392
A_22_P00023527	*RAB18*	Homo sapiens RAB18, member RAS oncogene family (RAB18), transcript variant 5, mRNA [NM_001256412]	-0.60158175	0.010237655
A_23_P67424	*lnc-AC016745.1-2*	Homo sapiens cDNA FLJ36584 fis, clone TRACH2013450. [AK093903]	-0.90380013	0.00925584
A_24_P316074	*ZNF461*	Homo sapiens zinc finger protein 461 (ZNF461), transcript variant 1, mRNA [NM_153257]	-0.8902818	0.013194162

A_33_P3237699	*CNOT8*	Homo sapiens CCR4-NOT transcription complex, subunit 8 (CNOT8), transcript variant 1, mRNA [NM_004779]	-1.1617379	0.0025001846
A_21_P0004715	*RMND5A*	Homo sapiens required for meiotic nuclear division 5 homolog A (S. cerevisiae) (RMND5A), mRNA [NM_022780]	-0.55184716	0.014835472
A_23_P28246	*SIPA1L3*	Homo sapiens signal-induced proliferation-associated 1 like 3 (SIPA1L3), mRNA [NM_015073]	0.2758595	0.0150995245
A_22_P00002461	*SLC23A3*	Homo sapiens solute carrier family 23, member 3 (SLC23A3), transcript variant 1, mRNA [NM_144712]	-1.2152115	0.015346156
A_23_P398836	*MAN1C1*	Homo sapiens mannosidase, alpha, class 1C, member 1 (MAN1C1), transcript variant 1, mRNA [NM_020379]	-1.7293469	0.0044240346
A_33_P3380211	*lnc-C15orf41-5*	LNCipedia lincRNA (lnc-C15orf41-5), lincRNA [lnc-C15orf41-5:1]	-0.90242994	0.005640775
A_24_P270890	*TXNDC16*	Homo sapiens thioredoxin domain containing 16 (TXNDC16), transcript variant 1, mRNA [NM_020784]	-1.2253	0.005377619
A_33_P3326588	*AKAP9*	Homo sapiens A kinase (PRKA) anchor protein 9 (AKAP9), transcript variant 2, mRNA [NM_005751]	-0.661603	0.009113876
A_24_P11587	*SPOP*	Homo sapiens speckle-type POZ protein (SPOP), transcript variant 1, mRNA [NM_001007226]	-1.1215022	0.004288771
A_23_P106002	*TNFRSF10D*	Homo sapiens tumor necrosis factor receptor superfamily, member 10d, decoy with truncated death domain (TNFRSF10D), mRNA [NM_003840]	-0.9955213	0.0050508454
A_23_P33196	*PEX13*	Homo sapiens peroxisomal biogenesis factor 13 (PEX13), mRNA [NM_002618]	-0.89226335	0.0056025535
A_33_P3223472	*NFKBIA*	Homo sapiens nuclear factor of kappa light polypeptide gene enhancer in B-cells inhibitor, alpha (NFKBIA), mRNA [NM_020529]	1.2605586	0.00590614
A_23_P43255	*COL5A2*	Homo sapiens collagen, type V, alpha 2 (COL5A2), mRNA [NM_000393]	-1610492	0.0029497955
A_21_P0011685	*AMZ2*	Homo sapiens archaelysin family metallopeptidase 2 (AMZ2), transcript variant 1, mRNA [NM_016627]	-0.76309055	0.008683025
A_23_P319133	*FAM49B*	Homo sapiens family with sequence similarity 49, member B (FAM49B), transcript variant 2, mRNA [NM_016623]	-0.27561474	0.010711222
A_23_P350551	*XLOC_l2_006665*	BROAD Institute lincRNA (XLOC_l2_006665), lincRNA [TCONS_l2_00012420]	1853669	0.00578448
A_22_P00003349	*DNAJC10*	Homo sapiens DnaJ (Hsp40) homolog, subfamily C, member 10 (DNAJC10), transcript variant 1, mRNA [NM_018981]	-0.81030226	0.010468031

A_23_P170491	*C12orf57*	Homo sapiens chromosome 12 open reading frame 57 (C12orf57), transcript variant 1, mRNA [NM_138425]	-13167369	0.003956951
A_32_P9963	*LINC00882*	Homo sapiens long intergenic non-protein coding RNA 882 (LINC00882), long non-coding RNA [NR_028303]	-0.8128512	0.00427815
A_24_P159036	*TRAIP*	Homo sapiens TRAF interacting protein (TRAIP), mRNA [NM_005879]	-0.47019178	0.0032869747
A_22_P00006901	*HSF2*	Homo sapiens heat shock transcription factor 2 (HSF2), transcript variant 1, mRNA [NM_004506]	-0.6301976	0.009848541
A_23_P322704	*RPL36AL*	Homo sapiens ribosomal protein L36a-like (RPL36AL), mRNA [NM_001001]	-0.19330697	0.016068412
A_23_P71864	*SCARNA10*	Homo sapiens small Cajal body-specific RNA 10 (SCARNA10), guide RNA [NR_004387]	-0.7504129	0.005581195
A_33_P3269203	*FAM177A1*	Homo sapiens family with sequence similarity 177, member A1 (FAM177A1), transcript variant 2, mRNA [NM_001079519]	-0.9974735	0.0048260237
A_23_P42718	*FAM73B*	Homo sapiens family with sequence similarity 73, member B (FAM73B), mRNA [NM_032809]	0.95023066	0.0071118497
A_23_P81241	*SERPINH1*	Homo sapiens serpin peptidase inhibitor, clade H (heat shock protein 47), member 1, (collagen binding protein 1) (SERPINH1), transcript variant 1, mRNA [NM_001207014]	-11703584	0.0024890031
A_33_P3382380	*NFE2L3*	Homo sapiens nuclear factor, erythroid 2-like 3 (NFE2L3), mRNA [NM_004289]	28664808	0.0032831472
A_33_P3736691	*NDFIP1*	Homo sapiens Nedd4 family interacting protein 1 (NDFIP1), mRNA [NM_030571]	-0.9201853	0.007925653
A_33_P3247077	*UBQLN4*	ubiquilin 4 [Source:HGNC Symbol;Acc:HGNC:1237] [ENST00000368309]	0.57685995	0.008559404
A_23_P408353	*ZNF430*	Homo sapiens zinc finger protein 430 (ZNF430), transcript variant 1, mRNA [NM_025189]	0.28105628	0.013624135
A_23_P118749	*HLA-A*	Homo sapiens major histocompatibility complex, class I, A (HLA-A), transcript variant 1 (A*03:01:0:01 allele), mRNA [NM_002116]	0.44144946	0.0081359595
A_24_P70002	*ALKBH2*	Homo sapiens alkB, alkylation repair homolog 2 (E. coli) (ALKBH2), transcript variant 2, mRNA [NM_001001655]	-0.9272764	0.015919259
A_24_P268676	*DERL2*	Homo sapiens derlin 2 (DERL2), mRNA [NM_016041]	-0.6391587	0.011383506
A_24_P521994	*LATS2*	Homo sapiens large tumor suppressor kinase 2 (LATS2), mRNA [NM_014572]	-10201441	0.004326644
A_23_P431319	*BHLHE40*	Homo sapiens basic helix-loop-helix family, member e40 (BHLHE40), mRNA [NM_003670]	2314111	0.0032239521
A_21_P0000287	*KLHL24*	Homo sapiens kelch-like family member 24 (KLHL24), mRNA [NM_017644]	-0.91607285	0.0080137085

A_23_P21838	*YIPF6*	Homo sapiens Yip1 domain family, member 6 (YIPF6), transcript variant A, mRNA [NM_173834]	-0.6199844	0.0067360536
A_24_P288754	*SNORD47*	Homo sapiens small nucleolar RNA, C/D box 47 (SNORD47), small nucleolar RNA [NR_002746]	-0.5294676	0.0074182227
A_23_P47691	*CNP*	Homo sapiens 2',3'-cyclic nucleotide 3' phosphodiesterase (CNP), mRNA [NM_033133]	-0.622308	0.007050734
A_33_P3329949	*PIGA*	Homo sapiens phosphatidylinositol glycan anchor biosynthesis, class A (PIGA), transcript variant 1, mRNA [NM_002641]	-0.51368785	0.009787606
A_23_P71591	*TRIM21*	Homo sapiens tripartite motif containing 21 (TRIM21), mRNA [NM_003141]	0.84423125	0.005842833
A_23_P126037	*ANXA7*	Homo sapiens annexin A7 (ANXA7), transcript variant 2, mRNA [NM_004034]	-0.7092824	0.009430791
A_33_P3249489	*NOL8*	Homo sapiens nucleolar protein 8 (NOL8), transcript variant 1, mRNA [NM_017948]	-0.5688205	0.004169345
A_24_P769672	*RLF*	Homo sapiens rearranged L-myc fusion (RLF), mRNA [NM_012421]	-0.73634577	0.013513713
A_33_P3302025	*WDR89*	Homo sapiens WD repeat domain 89 (WDR89), transcript variant 3, mRNA [NM_001258272]	-0.5789737	0.006637152
A_21_P0009392	*C12orf73*	Homo sapiens chromosome 12 open reading frame 73 (C12orf73), mRNA [NM_001135570]	-0.77054137	0.00705397
A_33_P3343073	*ADAMTSL1*	Homo sapiens ADAMTS-like 1 (ADAMTSL1), transcript variant 2, mRNA [NM_052866]	-0.9601804	0.009627439
A_23_P109345	*lnc-RGS9-1*	LNCipedia lincRNA (lnc-RGS9-1), lincRNA [lnc-RGS9-1:2]	0.7847748	0.0030924478
A_23_P137984	*SMARCB1*	Homo sapiens SWI/SNF related, matrix associated, actin dependent regulator of chromatin, subfamily b, member 1 (SMARCB1), transcript variant 1, mRNA [NM_003073]	0.9844241	0.008481417
A_33_P3267296	*PTTG1IP*	Homo sapiens pituitary tumor-transforming 1 interacting protein (PTTG1IP), transcript variant 1, mRNA [NM_004339]	-13291618	0.0072907214
A_22_P00005225	*S100A10*	Homo sapiens S100 calcium binding protein A10 (S100A10), mRNA [NM_002966]	-0.77699137	0.0024890031
A_23_P204980	*FKBP11*	Homo sapiens FK506 binding protein 11, 19 kDa (FKBP11), transcript variant 1, mRNA [NM_016594]	-15289317	0.006173301
A_32_P118372	*MEG8*	maternally expressed 8 (non-protein coding) [Source:HGNC Symbol;Acc:HGNC:14574] [ENST00000556475]	0.80516917	0.008717397
A_32_P225816	*UGGT2*	Homo sapiens UDP-glucose glycoprotein glucosyltransferase 2 (UGGT2), mRNA [NM_020121]	-0.8906991	0.008857441
A_32_P59811	*INTU*	Homo sapiens inturned planar cell polarity protein (INTU), mRNA [NM_015693]	-17068701	0.0039416323

A_33_P3305472	*PRDM16*	Homo sapiens PR domain containing 16 (PRDM16), transcript variant 1, mRNA [NM_022114]	-23801453	0.017158885
A_24_P215765	*MYO18A*	Homo sapiens myosin XVIIIA (MYO18A), transcript variant 1, mRNA [NM_078471]	12143058	0.005470758
A_33_P3297205	*ULK4P3*	Homo sapiens ULK4 pseudogene 3 (ULK4P3), non-coding RNA [NR_026859]	-0.9734502	0.013983251
A_21_P0010686	*ATP10A*	Homo sapiens ATPase, class V, type 10A (ATP10A), mRNA [NM_024490]	-16990778	0.003210263
A_33_P3367855	*C22orf29*	Homo sapiens chromosome 22 open reading frame 29 (C22orf29), mRNA [NM_024627]	-11581867	0.0067161894
A_23_P50008	*PIK3R2*	Homo sapiens phosphoinositide-3-kinase, regulatory subunit 2 (beta) (PIK3R2), transcript variant 1, mRNA [NM_005027]	-0.76918507	0.0102413045
A_23_P343935	*TRA2A*	Homo sapiens transformer 2 alpha homolog (Drosophila) (TRA2A), transcript variant 1, mRNA [NM_013293]	-0.5941117	0.012437972
A_24_P244952	*TTC19*	Homo sapiens tetratricopeptide repeat domain 19 (TTC19), transcript variant 1, mRNA [NM_017775]	-0.67468923	0.01572632
A_24_P703830	*EGLN1*	Homo sapiens egl-9 family hypoxia-inducible factor 1 (EGLN1), mRNA [NM_022051]	-0.7395501	0.010824262
A_33_P3298356	*SMG1*	Homo sapiens SMG1 phosphatidylinositol 3-kinase-related kinase (SMG1), mRNA [NM_015092]	-0.9177968	0.0076747234
A_33_P3407195	*NANOS3*	Homo sapiens nanos homolog 3 (Drosophila) (NANOS3), mRNA [NM_001098622]	-0.90347177	0.005403412
A_24_P291658	*SH3GLB1*	Homo sapiens SH3-domain GRB2-like endophilin B1 (SH3GLB1), transcript variant 2, mRNA [NM_001206651]	-10822821	0.0039655184
A_33_P3212650	*BOD1L1*	Homo sapiens biorientation of chromosomes in cell division 1-like 1 (BOD1L1), mRNA [NM_148894]	-0.7786999	0.010115223
A_23_P362824	*ADH1A*	Homo sapiens alcohol dehydrogenase 1A (class I), alpha polypeptide (ADH1A), mRNA [NM_000667]	24417214	0.012921736
A_23_P134614	*HDDC2*	HD domain containing 2 [Source:HGNC Symbol;Acc:HGNC:21078] [ENST00000609477]	-0.7851753	0.008427824
A_21_P0005002	*CSTF1*	Homo sapiens cleavage stimulation factor, 3' pre-RNA, subunit 1, 50kDa (CSTF1), transcript variant 2, mRNA [NM_001324]	-0.35281733	0.017113892
A_21_P0011364	*COG5*	Homo sapiens component of oligomeric golgi complex 5 (COG5), transcript variant 1, mRNA [NM_006348]	-0.90912	0.009911271
A_23_P342091	*lnc-CCDC90A-5*	LNCipedia lincRNA (lnc-CCDC90A-5), lincRNA [lnc-CCDC90A-5:1]	0.5750854	0.004723954
A_33_P3335621	*WHAMMP1*	Homo sapiens WAS protein homolog associated with actin, golgi membranes and	-0.6188583	0.0029497955

		microtubules pseudogene 1 (WHAMMP1), non-coding RNA [NR_036650]		
A_33_P3369034	*OARD1*	Homo sapiens O-acyl-ADP-ribose deacylase 1 (OARD1), mRNA [NM_145063]	-0.88664514	0.0057806885
A_21_P0010949	*PSMD10*	Homo sapiens proteasome (prosome, macropain) 26S subunit, non-ATPase, 10 (PSMD10), transcript variant 2, mRNA [NM_170750]	-1344135	0.004588558
A_23_P88209	*BCAR1*	Homo sapiens breast cancer anti-estrogen resistance 1 (BCAR1), transcript variant 1, mRNA [NM_001170714]	-0.5694141	0.0044707763
A_23_P62270	*NUTM2B-AS1*	Homo sapiens NUTM2B antisense RNA 1 (NUTM2B-AS1), transcript variant 1, long non-coding RNA [NR_120611]	-0.5865338	0.015833624
A_33_P3287529	*SCFD1*	Homo sapiens sec1 family domain containing 1 (SCFD1), transcript variant 1, mRNA [NM_016106]	-0.581161	0.0068771048
A_33_P3373707	*KDM5C*	Homo sapiens lysine (K)-specific demethylase 5C (KDM5C), transcript variant 1, mRNA [NM_004187]	0.50019234	0.012277604
A_23_P25097	*SDF4*	Homo sapiens stromal cell derived factor 4 (SDF4), transcript variant 2, mRNA [NM_016176]	0.7324264	0.012609292
A_33_P3371089	*STX7*	Homo sapiens syntaxin 7 (STX7), mRNA [NM_003569]	-1075302	0.0049136993
A_32_P73045	*LLPH*	Homo sapiens LLP homolog, long-term synaptic facilitation (Aplysia) (LLPH), mRNA [NM_032338]	-0.6670265	0.0061896956
A_22_P00011030	*SGTB*	Homo sapiens small glutamine-rich tetratricopeptide repeat (TPR)-containing, beta (SGTB), mRNA [NM_019072]	-1042625	0.0065241847
A_23_P18824	*CMAS*	Homo sapiens cytidine monophosphate N-acetylneuraminic acid synthetase (CMAS), mRNA [NM_018686]	-0.33628848	0.011140108
A_23_P26254	*lnc-NT5E-1*	LNCipedia lincRNA (lnc-NT5E-1), lincRNA [lnc-NT5E-1:1]	16834487	0.014329686
A_23_P92629	*PAPD7*	Homo sapiens PAP associated domain containing 7 (PAPD7), transcript variant 1, mRNA [NM_006999]	-0.48540565	0.011156819
A_23_P74609	*NDUFAF1*	Homo sapiens NADH dehydrogenase (ubiquinone) complex I, assembly factor 1 (NDUFAF1), transcript variant 1, mRNA [NM_016013]	-0.60485035	0.016332418
A_33_P3311775	*CWC27*	Homo sapiens CWC27 spliceosome-associated protein homolog (S. cerevisiae) (CWC27), transcript variant 1, mRNA [NM_005869]	-0.45673177	0.01121547
A_24_P911676	*G0S2*	Homo sapiens G0/G1 switch 2 (G0S2), mRNA [NM_015714]	21300495	0.0056184647

A_23_P304897	*ZNF789*	Homo sapiens zinc finger protein 789 (ZNF789), transcript variant 2, mRNA [NM_001013258]	-0.69819957	0.012774933
A_33_P3418125	*SOX4*	Homo sapiens SRY (sex determining region Y)-box 4 (SOX4), mRNA [NM_003107]	-0.5577217	0.0043857186
A_23_P98402	*BDKRB2*	Homo sapiens bradykinin receptor B2 (BDKRB2), mRNA [NM_000623]	10505192	0.006772767
A_21_P0014909	*GLIPR1*	Homo sapiens GLI pathogenesis-related 1 (GLIPR1), mRNA [NM_006851]	-1069042	0.008272217
A_24_P389916	*SIDT2*	Homo sapiens SID1 transmembrane family, member 2 (SIDT2), mRNA [NM_001040455]	-14823182	0.0054438785
A_33_P3233754	*LRRC32*	Homo sapiens leucine rich repeat containing 32 (LRRC32), transcript variant 1, mRNA [NM_005512]	-15368698	0.0073898793
A_23_P132669	*ZNF154*	Homo sapiens zinc finger protein 154 (ZNF154), transcript variant 1, mRNA [NM_001085384]	-0.7705221	0.017002601
A_33_P3264815	*SRD5A3*	Homo sapiens steroid 5 alpha-reductase 3 (SRD5A3), mRNA [NM_024592]	-11431041	0.0037353234
A_23_P325080	*GLT8D1*	Homo sapiens glycosyltransferase 8 domain containing 1 (GLT8D1), transcript variant 3, mRNA [NM_001010983]	-12441604	0.0039486005
A_33_P3301940	*TLN2*	Homo sapiens talin 2 (TLN2), mRNA [NM_015059]	-0.8385207	0.014228801
A_23_P112311	*PTOV1*	Homo sapiens prostate tumor overexpressed 1 (PTOV1), mRNA [NM_017432]	1000084	0.01089527
A_23_P142714	*PDE7B*	Homo sapiens phosphodiesterase 7B (PDE7B), mRNA [NM_018945]	0.76919097	0.016256094
A_23_P154065	*TRIM32*	Homo sapiens tripartite motif containing 32 (TRIM32), transcript variant 1, mRNA [NM_012210]	-0.7225525	0.006736978
A_32_P104063	*SLC25A12*	Homo sapiens solute carrier family 25 (aspartate/glutamate carrier), member 12 (SLC25A12), transcript variant 1, mRNA [NM_003705]	-0.6387365	0.0057506533
A_33_P3394494	*TUBA4A*	Homo sapiens tubulin, alpha 4a (TUBA4A), transcript variant 1, mRNA [NM_006000]	11553875	0.016205462
A_33_P3417260	*CRNDE*	Homo sapiens colorectal neoplasia differentially expressed (non-protein coding) (CRNDE), transcript variant 3, long non-coding RNA [NR_110453]	-1079453	0.00422039
A_33_P3227375	*ELP5*	Homo sapiens elongator acetyltransferase complex subunit 5 (ELP5), transcript variant 4, mRNA [NM_203415]	-10991291	0.005947757
A_33_P3423551	*NMRK1*	Homo sapiens nicotinamide riboside kinase 1 (NMRK1), transcript variant 1, mRNA [NM_017881]	-11136053	0.0050554653

A_23_P108657	*THBS2*	Homo sapiens thrombospondin 2 (THBS2), mRNA [NM_003247]	-13063104	0.0054418826
A_24_P365025	*IER3*	Homo sapiens immediate early response 3 (IER3), mRNA [NM_003897]	12189052	0.012215527
A_32_P123514	*WDSUB1*	Homo sapiens WD repeat, sterile alpha motif and U-box domain containing 1 (WDSUB1), transcript variant 3, mRNA [NM_152528]	-10433046	0.0041946988
A_21_P0007470	*SPAG9*	Homo sapiens sperm associated antigen 9 (SPAG9), transcript variant 3, mRNA [NM_003971]	-11463141	0.0065241847
A_33_P3240392	*PABPC4L*	Homo sapiens poly(A) binding protein, cytoplasmic 4-like (PABPC4L), mRNA [NM_001114734]	0.7894844	0.012776862
A_33_P3270311	*lnc-BLID-1*	LNCipedia lincRNA (lnc-BLID-1), lincRNA [lnc-BLID-1:17]	-13371328	0.008208044
A_23_P120103	*FOXO3*	Homo sapiens forkhead box O3 (FOXO3), transcript variant 1, mRNA [NM_001455]	-0.8210866	0.0062516355
A_23_P401361	*HECW2*	Homo sapiens HECT, C2 and WW domain containing E3 ubiquitin protein ligase 2 (HECW2), mRNA [NM_020760]	-0.5427452	0.0054708454
A_23_P416686	*KCNS3*	Homo sapiens potassium voltage-gated channel, modifier subfamily S, member 3 (KCNS3), transcript variant 1, mRNA [NM_002252]	0.9027404	0.0034310205
A_23_P420361	*PITPNM2*	Homo sapiens phosphatidylinositol transfer protein, membrane-associated 2 (PITPNM2), transcript variant 1, mRNA [NM_020845]	-0.46758178	0.01226546
A_23_P327426	*GPR137*	Homo sapiens G protein-coupled receptor 137 (GPR137), transcript variant 3, mRNA [NM_020155]	15578347	0.016776491
A_24_P37441	*BRK1*	Homo sapiens BRICK1, SCAR/WAVE actin-nucleating complex subunit (BRK1), mRNA [NM_018462]	-0.7056076	0.0075041396
A_33_P3386765	*TIPRL*	Homo sapiens TOR signaling pathway regulator (TIPRL), transcript variant 1, mRNA [NM_152902]	-0.5417347	0.0072230194
A_33_P3402414	*PDK1*	Homo sapiens pyruvate dehydrogenase kinase, isozyme 1 (PDK1), transcript variant 2, mRNA [NM_002610]	-0.85802746	0.00422039
A_33_P3355232	*ABHD14A*	Homo sapiens abhydrolase domain containing 14A (ABHD14A), mRNA [NM_015407]	-15540631	0.0041946988
A_24_P416346	*UBLCP1*	Homo sapiens ubiquitin-like domain containing CTD phosphatase 1 (UBLCP1), mRNA [NM_145049]	-0.4897675	0.016377958
A_33_P3339231	*MICALCL*	Homo sapiens MICAL C-terminal like (MICALCL), mRNA [NM_032867]	0.5672784	0.00945292
A_23_P205875	*ETV4*	Homo sapiens ets variant 4 (ETV4), transcript variant 2, mRNA [NM_001079675]	26465883	0.004092341

A_23_P253012	*UBN2*	Homo sapiens ubinuclein 2 (UBN2), mRNA [NM_173569]	-0.5887136	0.013706757
A_23_P15582	*SCAND2P*	Homo sapiens SCAN domain containing 2 pseudogene (SCAND2P), transcript variant 1, non-coding RNA [NR_004859]	-0.72532547	0.009844283
A_23_P46039	*GRAMD1C*	Homo sapiens GRAM domain containing 1C (GRAMD1C), transcript variant 1, mRNA [NM_017577]	-0.63414043	0.005612091
A_23_P266	*XYLT2*	Homo sapiens xylosyltransferase II (XYLT2), transcript variant 1, mRNA [NM_022167]	-0.8293009	0.009630487
A_22_P00024775	*FCRLA*	Homo sapiens Fc receptor-like A (FCRLA), transcript variant 2, mRNA [NM_032738]	-17649641	0.0064965826
A_23_P25974	*PIN1P1*	Homo sapiens peptidylprolyl cis/trans isomerase, NIMA-interacting 1 pseudogene 1 (PIN1P1), non-coding RNA [NR_023916]	0.79588586	0.007900263
A_23_P154585	*lnc-S100A1-2*	LNCipedia lincRNA (lnc-S100A1-2), lincRNA [lnc-S100A1-2:1]	0.8267782	0.01251333
A_23_P206369	*TTC7B*	Homo sapiens tetratricopeptide repeat domain 7B (TTC7B), mRNA [NM_001010854]	-0.7877068	0.014013276
A_23_P408167	*SNX21*	Homo sapiens sorting nexin family member 21 (SNX21), transcript variant 4, mRNA [NM_001042633]	-10982708	0.0072139124
A_24_P943193	*TMEM208*	Homo sapiens transmembrane protein 208 (TMEM208), mRNA [NM_014187]	-0.5814323	0.0090693105
A_22_P00005218	*GPRASP2*	Homo sapiens G protein-coupled receptor associated sorting protein 2 (GPRASP2), transcript variant 1, mRNA [NM_001004051]	-0.5696862	0.008427824
A_23_P433369	*PREPL*	Homo sapiens prolyl endopeptidase-like (PREPL), transcript variant 1, mRNA [NM_006036]	-0.77893496	0.0066995043
A_21_P0005605	*MEG3*	Homo sapiens maternally expressed 3 (non-protein coding) (MEG3), transcript variant 5, long non-coding RNA [NR_033359]	24289412	0.005236083
A_33_P3382910	*HEATR5A*	Homo sapiens HEAT repeat containing 5A (HEATR5A), mRNA [NM_015473]	-0.98096484	0.008829352
A_24_P56194	*lnc-GUSB-2*	LNCipedia lincRNA (lnc-GUSB-2), lincRNA [lnc-GUSB-2:1]	14510919	0.010274393
A_23_P390097	*GIN1*	Homo sapiens gypsy retrotransposon integrase 1 (GIN1), mRNA [NM_017676]	-0.7747448	0.0057506533
A_23_P53856	*CREBL2*	Homo sapiens cAMP responsive element binding protein-like 2 (CREBL2), mRNA [NM_001310]	-0.74926305	0.012441637
A_23_P127793	*TTC39B*	Homo sapiens tetratricopeptide repeat domain 39B (TTC39B), transcript variant 1, mRNA [NM_152574]	-12379807	0.0025001846
A_24_P79070	*N4BP2L2*	Homo sapiens NEDD4 binding protein 2-like 2 (N4BP2L2), transcript variant 2, mRNA [NM_014887]	-0.96035284	0.006004045

A_33_P3495962	*EML3*	Homo sapiens echinoderm microtubule associated protein like 3 (EML3), transcript variant 3, mRNA [NM_153265]	0.9800129	0.013460425
A_33_P3360611	*GNG8*	Homo sapiens guanine nucleotide binding protein (G protein), gamma 8 (GNG8), mRNA [NM_033258]	12059778	0.0055054883
A_33_P3211924	*SNORA71A*	AGENCOURT_6611325 NIH_MGC_106 Homo sapiens cDNA clone IMAGE:5485440 5', mRNA sequence [BM918074]	0.779283	0.004404579
A_33_P3404531	*MPP2*	Homo sapiens membrane protein, palmitoylated 2 (MAGUK p55 subfamily member 2) (MPP2), transcript variant 9, mRNA [NM_001278381]	-11790802	0.010799395
A_24_P252846	*RCOR1*	Homo sapiens REST corepressor 1 (RCOR1), mRNA [NM_015156]	-0.7609346	0.010105655
A_22_P00023117	*ZCWPW1*	Homo sapiens zinc finger, CW type with PWWP domain 1 (ZCWPW1), transcript variant 2, mRNA [NM_001258008]	-0.8670513	0.017003933
A_23_P393607	*C11orf74*	Homo sapiens chromosome 11 open reading frame 74 (C11orf74), transcript variant 4, mRNA [NM_138787]	-0.7755673	0.0074074627
A_33_P3305790	*lnc-RFC2-2*	LNCipedia lincRNA (lnc-RFC2-2), lincRNA [lnc-RFC2-2:1]	-0.81990093	0.014260212
A_33_P3407606	*SNAP47*	Homo sapiens synaptosomal-associated protein, 47kDa (SNAP47), mRNA [NM_053052]	-0.7156594	0.016310623
A_23_P165657	*NOS3*	Homo sapiens nitric oxide synthase 3 (endothelial cell) (NOS3), transcript variant 1, mRNA [NM_000603]	10989734	0.00817131
A_22_P00015589	*MSN*	moesin [Source:HGNC Symbol;Acc:HGNC:7373] [ENST00000447323]	-19351611	0.003667498
A_19_P00812587	*SLC20A1*	Homo sapiens solute carrier family 20 (phosphate transporter), member 1 (SLC20A1), mRNA [NM_005415]	0.75835204	0.0032073085
A_21_P0010943	*STX6*	Homo sapiens syntaxin 6 (STX6), transcript variant 1, mRNA [NM_005819]	-0.7490339	0.013398949
A_23_P103433	*RPL5*	Homo sapiens ribosomal protein L5 (RPL5), mRNA [NM_000969]	-0.68511087	0.006096579
A_23_P12503	*OSCP1*	Homo sapiens organic solute carrier partner 1 (OSCP1), transcript variant 1, mRNA [NM_145047]	-14675629	0.004282122
A_23_P406227	*AKT1*	Homo sapiens v-akt murine thymoma viral oncogene homolog 1 (AKT1), transcript variant 1, mRNA [NM_005163]	0.64075726	0.0152601935
A_24_P134834	*NUP133*	Homo sapiens nucleoporin 133kDa (NUP133), mRNA [NM_018230]	-0.7943194	0.0059353677
A_33_P3380086	*SNAI3-AS1*	Homo sapiens SNAI3 antisense RNA 1 (SNAI3-AS1), transcript variant 2, long non-coding RNA [NR_024399]	-0.49061698	0.016901668

A_23_P27066	*RHBDD1*	Homo sapiens rhomboid domain containing 1 (RHBDD1), transcript variant 1, mRNA [NM_032276]	-10170411	0.0053221607
A_21_P0000371	*PXN*	paxillin [Source:HGNC Symbol;Acc:HGNC:9718] [ENST00000323871]	13497214	0.014104453
A_23_P328600	*UBE2G1*	Homo sapiens ubiquitin-conjugating enzyme E2G 1 (UBE2G1), mRNA [NM_003342]	-0.89005184	0.007900263
A_23_P99405	*SNORD7*	Homo sapiens small nucleolar RNA, C/D box 7 (SNORD7), small nucleolar RNA [NR_003037]	-0.69356436	0.009792738
A_21_P0011465	*SLC8B1*	Homo sapiens solute carrier family 8 (sodium/lithium/calcium exchanger), member B1 (SLC8B1), mRNA [NM_024959]	-0.6908531	0.012172706
A_24_P235049	*ZMYM2*	Homo sapiens zinc finger, MYM-type 2 (ZMYM2), transcript variant 1, mRNA [NM_003453]	-0.9407327	0.0034534817
A_33_P3224331	*SMG1*	Homo sapiens SMG1 phosphatidylinositol 3-kinase-related kinase (SMG1), mRNA [NM_015092]	0.84334093	0.005701378
A_19_P00325158	*MTHFD1L*	Homo sapiens methylenetetrahydrofolate dehydrogenase (NADP+ dependent) 1-like (MTHFD1L), transcript variant 2, mRNA [NM_015440]	-11133708	0.010133606
A_23_P87011	*DDX3Y*	Homo sapiens DEAD (Asp-Glu-Ala-Asp) box helicase 3, Y-linked (DDX3Y), transcript variant 2, mRNA [NM_004660]	-0.8566511	0.005612162
A_33_P3224495	*MALAT1*	Homo sapiens metastasis associated lung adenocarcinoma transcript 1 (non-protein coding) (MALAT1), long non-coding RNA [NR_002819]	0.9445119	0.0031750489
A_23_P201287	*TAGLN*	Homo sapiens transgelin (TAGLN), transcript variant 1, mRNA [NM_001001522]	-0.49794048	0.01669231
A_33_P3210343	*KCNQ5-IT1*	Homo sapiens KCNQ5 intronic transcript 1 (non-protein coding) (KCNQ5-IT1), long non-coding RNA [NR_120503]	0.5304248	0.01613243
A_24_P300302	*KIF1B*	Homo sapiens kinesin family member 1B (KIF1B), transcript variant 1, mRNA [NM_015074]	-13570554	0.005879462
A_23_P258418	*ETV6*	Homo sapiens ets variant 6 (ETV6), mRNA [NM_001987]	-10969816	0.0038302224
A_24_P337746	*ZNF300P1*	Homo sapiens zinc finger protein 300 pseudogene 1 (functional) (ZNF300P1), non-coding RNA [NR_026867]	-13929294	0.0026564114
A_22_P00022263	*TNIP2*	Homo sapiens TNFAIP3 interacting protein 2 (TNIP2), transcript variant 1, mRNA [NM_024309]	0.69008315	0.0056287716
A_21_P0000121	*RABGEF1*	Homo sapiens RAB guanine nucleotide exchange factor (GEF) 1 (RABGEF1), transcript variant 4, mRNA [NM_014504]	-10858772	0.0075020376

A_23_P84047	*lnc-AL353597.1-1*	LNCipedia lincRNA (lnc-AL353597.1-1), lincRNA [lnc-AL353597.1-1:1]	2026383	0.013800824
A_33_P3577120	*C19orf81*	Homo sapiens chromosome 19 open reading frame 81 (C19orf81), mRNA [NM_001195076]	0.439065	0.016752198
A_33_P3282390	*PABPC5*	Homo sapiens poly(A) binding protein, cytoplasmic 5 (PABPC5), mRNA [NM_080832]	-0.5396759	0.0031941293
A_22_P00007974	*TBL3*	transducin (beta)-like 3 [Source:HGNC Symbol;Acc:HGNC:11587] [ENST00000568546]	0.586129	0.011828812
A_22_P00004265	*GRM4*	Homo sapiens glutamate receptor, metabotropic 4 (GRM4), transcript variant 1, mRNA [NM_000841]	-0.96685725	0.011745691
A_32_P155506	*lnc-IDH3G-1*	BX364425 Homo sapiens NEUROBLASTOMA COT 25-NORMALIZED Homo sapiens cDNA clone CS0DC009YA06 5-PRIME, mRNA sequence [BX364425]	0.63355577	0.010391631
A_24_P706314	*lnc-CMTM5-1*	LNCipedia lincRNA (lnc-CMTM5-1), lincRNA [lnc-CMTM5-1:1]	0.53191197	0.01238315
A_23_P215832	*UBE2E2*	Homo sapiens ubiquitin-conjugating enzyme E2E 2 (UBE2E2), mRNA [NM_152653]	-0.27058977	0.008003523
A_24_P9090	*HNRNPA3*	Homo sapiens heterogeneous nuclear ribonucleoprotein A3 (HNRNPA3), mRNA [NM_194247]	-0.43143204	0.010314908
A_23_P133365	*ATP5J2*	Homo sapiens ATP synthase, H+ transporting, mitochondrial Fo complex, subunit F2 (ATP5J2), transcript variant 1, mRNA [NM_004889]	-0.44042224	0.017107433
A_21_P0003931	*HNRNPDL*	Homo sapiens heterogeneous nuclear ribonucleoprotein D-like (HNRNPDL), transcript variant 2, mRNA [NM_031372]	-0.48209387	0.008416942
A_24_P175989	*TCERG1*	Homo sapiens transcription elongation regulator 1 (TCERG1), transcript variant 1, mRNA [NM_006706]	-0.42040128	0.00558687
A_23_P149818	*MIR143HG*	MIR143 host gene (non-protein coding) [Source:HGNC Symbol;Acc:HGNC:42872] [ENST00000505254]	-51547475	0.0024890031
A_33_P3387771	*VPS29*	Homo sapiens vacuolar protein sorting 29 homolog (S. cerevisiae) (VPS29), transcript variant 1, mRNA [NM_016226]	-10086899	0.009441473
A_23_P372467	*UPF2*	Homo sapiens UPF2 regulator of nonsense transcripts homolog (yeast) (UPF2), transcript variant 1, mRNA [NM_080599]	-0.74247825	0.010396678
A_24_P145316	*USP9X*	Homo sapiens ubiquitin specific peptidase 9, X-linked (USP9X), transcript variant 3, mRNA [NM_001039590]	-0.6420469	0.0072230194
A_32_P90047	*AHSA2*	Homo sapiens AHA1, activator of heat shock 90kDa protein ATPase homolog 2 (yeast) (AHSA2), mRNA [NM_152392]	-0.94281316	0.0066162148

A_23_P127584	*DTNBP1*	Homo sapiens dystrobrevin binding protein 1 (DTNBP1), transcript variant 2, mRNA [NM_183040]	-0.60285527	0.010289405
A_32_P2738	*C4orf48*	Homo sapiens chromosome 4 open reading frame 48 (C4orf48), transcript variant 2, mRNA [NM_001141936]	-0.8220029	0.013454804
A_33_P3339860	*NNMT*	Homo sapiens nicotinamide N-methyltransferase (NNMT), mRNA [NM_006169]	-0.39347792	0.0094108535
A_24_P187774	*TCTN3*	Homo sapiens tectonic family member 3 (TCTN3), transcript variant 1, mRNA [NM_015631]	-0.71132785	0.009566855
A_32_P456537	*PLEKHA2*	Homo sapiens pleckstrin homology domain containing, family A (phosphoinositide binding specific) member 2 (PLEKHA2), mRNA [NM_021623]	-0.63362265	0.011091872
A_24_P186944	*SVEP1*	sushi, von Willebrand factor type A, EGF and pentraxin domain containing 1 [Source:HGNC Symbol;Acc:HGNC:15985] [ENST00000374461]	0.20417126	0.0117895575
A_33_P3338634	*TCP1*	Homo sapiens t-complex 1 (TCP1), transcript variant 1, mRNA [NM_030752]	-0.56355786	0.01282686
A_21_P0002588	*SYT14*	Homo sapiens synaptotagmin XIV (SYT14), transcript variant 5, mRNA [NM_001256006]	22224078	0.006149492
A_24_P282416	*NR3C1*	Homo sapiens nuclear receptor subfamily 3, group C, member 1 (glucocorticoid receptor) (NR3C1), transcript variant 5, mRNA [NM_001018077]	-0.847733	0.003365343
A_24_P647682	*lnc-FAM82A1-1*	LNCipedia lincRNA (lnc-FAM82A1-1), lincRNA [lnc-FAM82A1-1:1]	0.43018287	0.00862906
A_23_P216307	*ABL1*	Homo sapiens ABL proto-oncogene 1, non-receptor tyrosine kinase (ABL1), transcript variant b, mRNA [NM_007313]	10808834	0.012288998
A_33_P3374903	*RUNX1T1*	Homo sapiens runt-related transcription factor 1; translocated to, 1 (cyclin D-related) (RUNX1T1), transcript variant 1, mRNA [NM_004349]	-11085749	0.0066818837
A_23_P160406	*ZDHHC21*	Homo sapiens zinc finger, DHHC-type containing 21 (ZDHHC21), mRNA [NM_178566]	-0.78964376	0.012334684
A_23_P117515	*KCTD3*	Homo sapiens potassium channel tetramerization domain containing 3 (KCTD3), mRNA [NM_016121]	-0.9862094	0.004324298
A_23_P217778	*ZNF627*	Homo sapiens zinc finger protein 627 (ZNF627), transcript variant 1, mRNA [NM_145295]	-11650375	0.010230527
A_22_P00008240	*ARID4A*	Homo sapiens AT rich interactive domain 4A (RBP1-like) (ARID4A), transcript variant 1, mRNA [NM_002892]	-0.47836637	0.010839669

A_32_P357301	*MSL3*	Homo sapiens male-specific lethal 3 homolog (Drosophila) (MSL3), transcript variant 1, mRNA [NM_078629]	-0.65999895	0.011443248
A_33_P3224795	*LOC102724190*	Homo sapiens uncharacterized LOC102724190 (LOC102724190), long non-coding RNA [NR_110553]	0.926204	0.00795879
A_24_P922631	*PPHLN1*	Homo sapiens periphilin 1 (PPHLN1), transcript variant 1, mRNA [NM_016488]	-0.45274106	0.012999866
A_33_P3244141	*IKZF5*	Homo sapiens IKAROS family zinc finger 5 (Pegasus) (IKZF5), transcript variant 1, mRNA [NM_001271840]	-0.7699071	0.01171801
A_23_P97736	*C5orf58*	Homo sapiens chromosome 5 open reading frame 58 (C5orf58), mRNA [NM_001102609]	39453306	0.002804883
A_23_P54079	*NDEL1*	Homo sapiens nudE neurodevelopment protein 1-like 1 (NDEL1), transcript variant 1, mRNA [NM_001025579]	-0.51472163	0.009630487
A_23_P251893	*NCDN*	Homo sapiens neurochondrin (NCDN), transcript variant 3, mRNA [NM_014284]	0.898921	0.010550066
A_23_P324384	*OSGEP*	Homo sapiens O-sialoglycoprotein endopeptidase (OSGEP), mRNA [NM_017807]	-0.8491759	0.0025001846
A_33_P3232692	*BRAT1*	Homo sapiens BRCA1-associated ATM activator 1 (BRAT1), mRNA [NM_152743]	-0.9980077	0.005663018
A_33_P3348244	*RPS4Y2*	Homo sapiens ribosomal protein S4, Y-linked 2 (RPS4Y2), mRNA [NM_001039567]	-0.96760726	0.0037335544
A_33_P3336652	*IL24*	Homo sapiens interleukin 24 (IL24), transcript variant 3, mRNA [NM_001185156]	459034	0.0028288076
A_23_P162782	*BRI3*	Homo sapiens brain protein I3 (BRI3), transcript variant 1, mRNA [NM_015379]	-0.8524167	0.010154543
A_23_P56746	*NDUFC2*	Homo sapiens NADH dehydrogenase (ubiquinone) 1, subcomplex unknown, 2, 14.5kDa (NDUFC2), transcript variant 2, mRNA [NM_001204054]	-0.76069975	0.008358917
A_23_P152002	*ARGLU1*	Homo sapiens arginine and glutamate rich 1 (ARGLU1), mRNA [NM_018011]	-0.92840457	0.015010829
A_24_P183094	*FAP*	Homo sapiens fibroblast activation protein, alpha (FAP), transcript variant 1, mRNA [NM_004460]	-11491129	0.0025001846
A_23_P58606	*BCL2A1*	Homo sapiens BCL2-related protein A1 (BCL2A1), transcript variant 1, mRNA [NM_004049]	45146685	0.0072139124
A_33_P3374878	*ATP13A3*	Homo sapiens ATPase type 13A3 (ATP13A3), mRNA [NM_024524]	-0.38139838	0.002804883
A_33_P3303031	*CCNG1*	Homo sapiens cyclin G1 (CCNG1), transcript variant 1, mRNA [NM_004060]	-11196635	0.0027139152
A_23_P142830	*FAT4*	Homo sapiens FAT atypical cadherin 4 (FAT4), transcript variant 1, mRNA [NM_001291303]	-13500451	0.0053998437

A_32_P225355	*LOC643549*	Homo sapiens hypothetical protein LOC643549, mRNA (cDNA clone IMAGE:40147028). [BC133006]	10787263	0.004701671
A_23_P133068	*PLA2R1*	Homo sapiens phospholipase A2 receptor 1, 180kDa (PLA2R1), transcript variant 1, mRNA [NM_007366]	-10215667	0.007740612
A_21_P0012708	*CPEB2*	Homo sapiens cytoplasmic polyadenylation element binding protein 2 (CPEB2), transcript variant B, mRNA [NM_182485]	-0.8203142	0.0039416323
A_21_P0013318	*ANK2*	Homo sapiens ankyrin 2, neuronal (ANK2), transcript variant 1, mRNA [NM_001148]	-15810037	0.006474282
A_23_P63281	*CRCP*	Homo sapiens CGRP receptor component (CRCP), transcript variant 4, mRNA [NM_001142414]	-0.5491986	0.015868904
A_33_P3386955	*CHCHD7*	Homo sapiens coiled-coil-helix-coiled-coil-helix domain containing 7 (CHCHD7), transcript variant 1, mRNA [NM_001011667]	-0.63024753	0.0048233946
A_24_P409042	*CPTP*	Homo sapiens ceramide-1-phosphate transfer protein (CPTP), mRNA [NM_001029885]	14070781	0.010735533
A_23_P50646	*MRPS5*	Homo sapiens mitochondrial ribosomal protein S5 (MRPS5), mRNA [NM_031902]	-0.8029925	0.007807294
A_21_P0000592	*CDC42SE2*	Homo sapiens CDC42 small effector 2 (CDC42SE2), transcript variant 1, mRNA [NM_020240]	-0.5739464	0.006633698
A_23_P168882	*PINLYP*	Homo sapiens phospholipase A2 inhibitor and LY6/PLAUR domain containing (PINLYP), transcript variant 1, mRNA [NM_001193621]	-0.83397925	0.015648095
A_24_P174341	*SNORA70G*	Homo sapiens small nucleolar RNA, H/ACA box 70G (SNORA70G), small nucleolar RNA [NR_033335]	0.70795065	0.009731291
A_33_P3254510	*TP53INP1*	Homo sapiens tumor protein p53 inducible nuclear protein 1 (TP53INP1), transcript variant 1, mRNA [NM_033285]	-16968596	0.0037124872
A_23_P7144	*CCNT2*	Homo sapiens cyclin T2 (CCNT2), transcript variant b, mRNA [NM_058241]	-12618021	0.010909709
A_24_P286079	*CXCL1*	Homo sapiens chemokine (C-X-C motif) ligand 1 (melanoma growth stimulating activity, alpha) (CXCL1), transcript variant 1, mRNA [NM_001511]	2780225	0.0024890031
A_23_P69683	*PHACTR2*	Homo sapiens phosphatase and actin regulator 2 (PHACTR2), transcript variant 2, mRNA [NM_001100165]	-10470836	0.009497297
A_33_P3398331	*AFF4*	Homo sapiens AF4/FMR2 family, member 4 (AFF4), mRNA [NM_014423]	-0.96846986	0.0034534817
A_33_P3279009	*SEC24B*	Homo sapiens SEC24 family member B (SEC24B), transcript variant 1, mRNA [NM_006323]	-0.79644156	0.004358433

A_22_P00012531	*MMP24*	Homo sapiens matrix metallopeptidase 24 (membrane-inserted) (MMP24), mRNA [NM_006690]	-12720529	0.011696815
A_23_P157809	*HMX1*	Homo sapiens H6 family homeobox 1 (HMX1), mRNA [NM_018942]	0.42621943	0.0053756773
A_33_P3230594	*lnc-PSPC1-1*	UI-CF-EC1-abv-j-01-0-UI.s1 UI-CF-EC1 Homo sapiens cDNA clone UI-CF-EC1-abv-j-01-0-UI 3', mRNA sequence [BM970278]	-1137183	0.007501246
A_23_P99204	*PTGR1*	Homo sapiens prostaglandin reductase 1 (PTGR1), transcript variant 2, mRNA [NM_012212]	-0.637513	0.014939495
A_33_P3266255	*LOC100507547*	Homo sapiens uncharacterized LOC100507547 (LOC100507547), transcript variant 1, long non-coding RNA [NR_037169]	-10474188	0.0036556649
A_33_P3408665	*MAPKAPK5-AS1*	Homo sapiens MAPKAPK5 antisense RNA 1 (MAPKAPK5-AS1), long non-coding RNA [NR_015404]	-0.63721734	0.0057522994
A_22_P00012883	*CSGALNACT2*	Homo sapiens chondroitin sulfate N-acetylgalactosaminyltransferase 2 (CSGALNACT2), mRNA [NM_018590]	-0.14036906	0.01497713
A_33_P3364205	*lnc-RBM11-2*	Homo sapiens, clone IMAGE:3660074, mRNA. [BC048201]	-0.6564145	0.013501388
A_33_P3266265	*TBXA2R*	Homo sapiens thromboxane A2 receptor (TBXA2R), transcript variant a, mRNA [NM_001060]	30127525	0.0073189787
A_24_P229025	*SBDSP1*	Homo sapiens Shwachman-Bodian-Diamond syndrome pseudogene 1 (SBDSP1), transcript variant 3, non-coding RNA [NR_001588]	-15471302	0.009713893
A_32_P231446	*ABHD17B*	Homo sapiens abhydrolase domain containing 17B (ABHD17B), transcript variant 2, mRNA [NM_001025780]	-0.52239704	0.012163237
A_33_P3302115	*GRIA3*	Homo sapiens glutamate receptor, ionotropic, AMPA 3 (GRIA3), transcript variant 3, mRNA [NM_001256743]	-27194707	0.0025001846
A_33_P3275968	*HIPK1*	Homo sapiens homeodomain interacting protein kinase 1 (HIPK1), transcript variant 1, mRNA [NM_198268]	-0.68652797	0.0029471128
A_33_P3321996	*CAPRIN1*	Homo sapiens cell cycle associated protein 1 (CAPRIN1), transcript variant 1, mRNA [NM_005898]	-0.84739345	0.0053387037
A_33_P3228305	*SEL1L*	Homo sapiens sel-1 suppressor of lin-12-like (C. elegans) (SEL1L), transcript variant 1, mRNA [NM_005065]	-0.7854635	0.010931163
A_33_P3361417	*lnc-SDCCAG8-2*	DB053889 TESTI2 Homo sapiens cDNA clone TESTI2044585 5', mRNA sequence [DB053889]	13028994	0.007058774
A_33_P3344831	*ARHGAP26*	Homo sapiens Rho GTPase activating protein 26 (ARHGAP26), transcript variant 1, mRNA [NM_015071]	-31815982	0.0040526874

A_33_P3366903	*C6orf57*	Homo sapiens chromosome 6 open reading frame 57 (C6orf57), mRNA [NM_145267]	-10720377	0.0035734554
A_23_P345591	*TMEM45A*	Homo sapiens transmembrane protein 45A (TMEM45A), mRNA [NM_018004]	-14004748	0.004616648
A_33_P3251985	*CHST6*	Homo sapiens carbohydrate (N-acetylglucosamine 6-O) sulfotransferase 6 (CHST6), mRNA [NM_021615]	12173986	0.004404579
A_23_P126803	*PSMA2*	Homo sapiens proteasome (prosome, macropain) subunit, alpha type, 2 (PSMA2), mRNA [NM_002787]	-0.2955439	0.009466768
A_23_P72138	*MFSD1*	Homo sapiens major facilitator superfamily domain containing 1 (MFSD1), transcript variant 1, mRNA [NM_022736]	-0.5138726	0.008159287
A_33_P3315899	*ARPC5*	Homo sapiens actin related protein 2/3 complex, subunit 5, 16kDa (ARPC5), transcript variant 1, mRNA [NM_005717]	-11801028	0.0072222846
A_23_P352684	*MRPS22*	Homo sapiens mitochondrial ribosomal protein S22 (MRPS22), mRNA [NM_020191]	-0.5532787	0.0070571043
A_23_P151459	*DCAF5*	Homo sapiens DDB1 and CUL4 associated factor 5 (DCAF5), transcript variant 1, mRNA [NM_003861]	-0.9659918	0.008204847
A_22_P00001933	*TOMM5*	Homo sapiens translocase of outer mitochondrial membrane 5 homolog (yeast) (TOMM5), transcript variant 3, mRNA [NM_001134484]	-0.5252511	0.010820454
A_23_P99360	*MYCBP2*	Homo sapiens MYC binding protein 2, E3 ubiquitin protein ligase (MYCBP2), mRNA [NM_015057]	-0.6459739	0.011672903
A_23_P41541	*LOC101928880*	Homo sapiens uncharacterized LOC101928880 (LOC101928880), long non-coding RNA [NR_110944]	0.882687	0.005612091
A_21_P0014771	*TRIM13*	Homo sapiens tripartite motif containing 13 (TRIM13), transcript variant 3, mRNA [NM_213590]	-0.57964396	0.008404053
A_24_P203953	*PRMT9*	Homo sapiens protein arginine methyltransferase 9 (PRMT9), mRNA [NM_138364]	-0.74693626	0.009202373
A_33_P3351097	*LOC439951*	PREDICTED: Homo sapiens uncharacterized LOC439951 (LOC439951), misc_RNA [XR_171055]	0.735477	0.01543225
A_23_P157022	*FAM26E*	Homo sapiens family with sequence similarity 26, member E (FAM26E), mRNA [NM_153711]	-10648427	0.006639663
A_24_P390583	*ZNF786*	Homo sapiens zinc finger protein 786 (ZNF786), mRNA [NM_152411]	-0.60755056	0.013348818
A_24_P942068	*BLZF1*	Homo sapiens basic leucine zipper nuclear factor 1 (BLZF1), mRNA [NM_003666]	-0.5305877	0.0068629696
A_22_P00005112	*USP31*	Homo sapiens ubiquitin specific peptidase 31 (USP31), mRNA [NM_020718]	-0.5874834	0.01120723

A_19_P00330076	*TANC2*	Homo sapiens tetratricopeptide repeat, ankyrin repeat and coiled-coil containing 2 (TANC2), mRNA [NM_025185]	-14826516	0.004326644
A_23_P2661	*lnc-DHRS7B-3*	LNCipedia lincRNA (lnc-DHRS7B-3), lincRNA [lnc-DHRS7B-3:1]	0.7698395	0.0066119307
A_23_P391506	*lnc-SIK1-5*	LNCipedia lincRNA (lnc-SIK1-5), lincRNA [lnc-SIK1-5:4]	0.68232423	0.003121324
A_23_P40194	*RAP1B*	Homo sapiens RAP1B, member of RAS oncogene family (RAP1B), transcript variant 1, mRNA [NM_015646]	-0.66007566	0.0069260783
A_23_P258221	*IVNS1ABP*	Homo sapiens influenza virus NS1A binding protein (IVNS1ABP), mRNA [NM_006469]	-10430474	0.003162355
A_24_P11791	*DDX27*	Homo sapiens DEAD (Asp-Glu-Ala-Asp) box polypeptide 27 (DDX27), mRNA [NM_017895]	-0.6855626	0.007797447
A_23_P49082	*ABCC5*	Homo sapiens ATP-binding cassette, sub-family C (CFTR/MRP), member 5 (ABCC5), transcript variant 1, mRNA [NM_005688]	-0.8367698	0.007697845
A_23_P209731	*KPNA4*	Homo sapiens karyopherin alpha 4 (importin alpha 3) (KPNA4), mRNA [NM_002268]	-0.9170186	0.00659355
A_33_P3393582	*NUBP2*	Homo sapiens nucleotide binding protein 2 (NUBP2), transcript variant 1, mRNA [NM_012225]	12846688	0.0031474202
A_23_P161439	*ARMC9*	Homo sapiens armadillo repeat containing 9 (ARMC9), transcript variant 2, mRNA [NM_025139]	-0.5977079	0.008685628
A_21_P0003756	*CCNDBP1*	cyclin D-type binding-protein 1 [Source:HGNC Symbol;Acc:HGNC:1587] [ENST00000564630]	-21669288	0.0031941293
A_21_P0012569	*ADIRF*	Homo sapiens adipogenesis regulatory factor (ADIRF), mRNA [NM_006829]	18934729	0.0037353234
A_23_P89123	*lnc-ARSJ-1*	LNCipedia lincRNA (lnc-ARSJ-1), lincRNA [lnc-ARSJ-1:2]	-10928156	0.003956336
A_23_P217917	*CFDP1*	Homo sapiens craniofacial development protein 1 (CFDP1), mRNA [NM_006324]	-0.7851898	0.004404579
A_23_P417942	*RTF1*	Homo sapiens Rtf1, Paf1/RNA polymerase II complex component, homolog (S. cerevisiae) (RTF1), mRNA [NM_015138]	-0.5993286	0.010138943
A_23_P216149	*GSTM4*	Homo sapiens glutathione S-transferase mu 4 (GSTM4), transcript variant 2, mRNA [NM_147148]	30312657	0.0031750489
A_33_P3410849	*FNBP1L*	Homo sapiens formin binding protein 1-like (FNBP1L), transcript variant 1, mRNA [NM_001024948]	-2212138	0.0029691157
A_23_P56590	*TERF1*	Homo sapiens telomeric repeat binding factor (NIMA-interacting) 1 (TERF1), transcript variant 1, mRNA [NM_017489]	-0.37438774	0.012380959
A_23_P436138	*C8orf58*	Homo sapiens chromosome 8 open reading frame 58 (C8orf58), transcript variant 1, mRNA [NM_001013842]	0.5209186	0.01501352

A_24_P375599	*C1D*	Homo sapiens C1D nuclear receptor corepressor (C1D), transcript variant 1, mRNA [NM_006333]	-0.32467073	0.014904488
A_24_P80500	*MAX*	Homo sapiens MYC associated factor X (MAX), transcript variant 4, mRNA [NM_145114]	-11072562	0.012728797
A_23_P78438	*BDH2*	Homo sapiens 3-hydroxybutyrate dehydrogenase, type 2 (BDH2), mRNA [NM_020139]	-11385366	0.008717397
A_24_P314179	*CRBN*	Homo sapiens cereblon (CRBN), transcript variant 1, mRNA [NM_016302]	-0.9374451	0.0034766807
A_24_P407235	*ELP2*	Homo sapiens elongator acetyltransferase complex subunit 2 (ELP2), transcript variant 2, mRNA [NM_018255]	-10112739	0.00371792
A_33_P3358626	*ETS2*	Homo sapiens v-ets avian erythroblastosis virus E26 oncogene homolog 2 (ETS2), transcript variant 1, mRNA [NM_005239]	-0.805318	0.015502214
A_23_P31315	*CRY1*	Homo sapiens cryptochrome circadian clock 1 (CRY1), mRNA [NM_004075]	-0.30458862	0.0086152
A_24_P51061	*TAF4B*	Homo sapiens TAF4b RNA polymerase II, TATA box binding protein (TBP)-associated factor, 105kDa (TAF4B), transcript variant 1, mRNA [NM_001293725]	-0.6403159	0.016463466
A_23_P319859	*CBX3*	Homo sapiens chromobox homolog 3 (CBX3), transcript variant 2, mRNA [NM_016587]	-0.426141	0.007635518
A_24_P290263	*DCBLD2*	Homo sapiens discoidin, CUB and LCCL domain containing 2 (DCBLD2), mRNA [NM_080927]	15641854	0.0034534817
A_23_P432591	*EYA2*	Homo sapiens EYA transcriptional coactivator and phosphatase 2 (EYA2), transcript variant 1, mRNA [NM_005244]	-20492659	0.0044762716
A_33_P3262181	*CCDC125*	Homo sapiens coiled-coil domain containing 125 (CCDC125), transcript variant 1, mRNA [NM_176816]	-0.51443124	0.00874838
A_22_P00016145	*SDHC*	Homo sapiens succinate dehydrogenase complex, subunit C, integral membrane protein, 15kDa (SDHC), transcript variant 1, mRNA [NM_003001]	-0.81315035	0.00628389
A_33_P3400758	*APOBEC3F*	Homo sapiens apolipoprotein B mRNA editing enzyme, catalytic polypeptide-like 3F (APOBEC3F), transcript variant 2, mRNA [NM_001006666]	-12161391	0.0034903844
A_22_P00000867	*lnc-TIMM9-2*	LNCipedia lincRNA (lnc-TIMM9-2), lincRNA [lnc-TIMM9-2:12]	-0.6606191	0.010369088
A_33_P3268224	*ZNF555*	Homo sapiens zinc finger protein 555 (ZNF555), transcript variant 1, mRNA [NM_152791]	-13292319	0.0037856582
A_23_P370097	*AGRN*	Homo sapiens agrin (AGRN), mRNA [NM_198576]	16688218	0.004327546

A_33_P3255046	*SPATA5*	Homo sapiens spermatogenesis associated 5 (SPATA5), mRNA [NM_145207]	-0.40997806	0.00752686
A_21_P0007029	*TMEM237*	Homo sapiens transmembrane protein 237 (TMEM237), transcript variant 1, mRNA [NM_001044385]	-0.9916606	0.0061321366
A_22_P00003858	*FLJ37035*	uncharacterized LOC399821 [Source:EntrezGene;Acc:399821] [ENST00000419400]	-12020446	0.0046008946
A_23_P359245	*CHN1*	Homo sapiens chimerin 1 (CHN1), transcript variant 1, mRNA [NM_001822]	-18138187	0.0027248112
A_33_P3332130	*LINC00339*	Homo sapiens long intergenic non-protein coding RNA 339 (LINC00339), transcript variant 3, long non-coding RNA [NR_109762]	0.34230286	0.016493961
A_23_P79703	*MET*	Homo sapiens MET proto-oncogene, receptor tyrosine kinase (MET), transcript variant 2, mRNA [NM_000245]	-0.49536246	0.013774471
A_23_P42738	*PGRMC1*	Homo sapiens progesterone receptor membrane component 1 (PGRMC1), transcript variant 2, mRNA [NM_001282621]	-10358086	0.0034766807
A_33_P3262515	*HADHB*	Homo sapiens hydroxyacyl-CoA dehydrogenase/3-ketoacyl-CoA thiolase/enoyl-CoA hydratase (trifunctional protein), beta subunit (HADHB), transcript variant 1, mRNA [NM_000183]	-0.9096763	0.0050698896
A_33_P3411090	*FAM220A*	Homo sapiens family with sequence similarity 220, member A (FAM220A), mRNA [NM_001037163]	-0.93393636	0.005822303
A_33_P3413958	*RASA3*	Homo sapiens RAS p21 protein activator 3 (RASA3), mRNA [NM_007368]	-0.6795583	0.007819016
A_32_P225854	*ADAL*	Homo sapiens adenosine deaminase-like (ADAL), transcript variant 2, mRNA [NM_001012969]	-10030922	0.012649422
A_32_P412313	*OR7E47P*	Homo sapiens olfactory receptor, family 7, subfamily E, member 47 pseudogene (OR7E47P), transcript variant 1, non-coding RNA [NR_120437]	-0.8171782	0.008106991
A_23_P425066	*SPRED2*	Homo sapiens sprouty-related, EVH1 domain containing 2 (SPRED2), transcript variant 1, mRNA [NM_181784]	0.27549255	0.01042595
A_33_P3242863	*ASB6*	Homo sapiens ankyrin repeat and SOCS box containing 6 (ASB6), transcript variant 1, mRNA [NM_017873]	0.8855709	0.0031941293
A_23_P217098	*CRYBB2*	Homo sapiens crystallin, beta B2 (CRYBB2), mRNA [NM_000496]	-0.83258843	0.0140423635
A_23_P113634	*NT5M*	Homo sapiens 5',3'-nucleotidase, mitochondrial (NT5M), mRNA [NM_020201]	-19352598	0.0034884813

A_23_P50426	*VPS13A*	Homo sapiens vacuolar protein sorting 13 homolog A (S. cerevisiae) (VPS13A), transcript variant A, mRNA [NM_033305]	-0.5581281	0.015820691
A_33_P3256113	*CBFB*	Homo sapiens core-binding factor, beta subunit (CBFB), transcript variant 2, mRNA [NM_001755]	-0.74191755	0.015754001
A_24_P254177	*KANK2*	Homo sapiens KN motif and ankyrin repeat domains 2 (KANK2), transcript variant 1, mRNA [NM_015493]	-0.90503407	0.011821381
A_22_P00000040	*GUSBP1*	Homo sapiens glucuronidase, beta pseudogene 1 (GUSBP1), transcript variant 1, non-coding RNA [NR_027026]	-11141211	0.006315211
A_23_P121396	*SMIM7*	Homo sapiens small integral membrane protein 7 (SMIM7), transcript variant 2, mRNA [NM_024104]	-0.82145256	0.006513625
A_24_P375683	*HOTAIRM1*	Homo sapiens HOXA transcript antisense RNA, myeloid-specific 1 (HOTAIRM1), transcript variant 1, long non-coding RNA [NR_038366]	-0.83013195	0.004566164
A_22_P00025141	*DNAJC19*	Homo sapiens DnaJ (Hsp40) homolog, subfamily C, member 19 (DNAJC19), transcript variant 1, mRNA [NM_145261]	-0.6575337	0.014448771
A_23_P310331	*lnc-RNASET2-1*	LNCipedia lincRNA (lnc-RNASET2-1), lincRNA [lnc-RNASET2-1:1]	0.56493783	0.006262615
A_22_P00015976	*ZNF493*	Homo sapiens zinc finger protein 493 (ZNF493), transcript variant 2, mRNA [NM_145326]	-0.8913766	0.010404371
A_23_P103328	*RANBP3*	Homo sapiens RAN binding protein 3 (RANBP3), transcript variant RANBP3-a, mRNA [NM_003624]	-0.55417395	0.010474825
A_33_P3221528	*lnc-TENC1-1*	LNCipedia lincRNA (lnc-TENC1-1), lincRNA [lnc-TENC1-1:1]	0.59090745	0.014356328
A_23_P114282	*PTGER3*	Homo sapiens prostaglandin E receptor 3 (subtype EP3) (PTGER3), transcript variant 4, mRNA [NM_198714]	11351112	0.006543285
A_33_P3290687	*IREB2*	Homo sapiens iron-responsive element binding protein 2 (IREB2), mRNA [NM_004136]	-10459099	0.0071601756
A_33_P8991074	*MCTS1*	Homo sapiens malignant T cell amplified sequence 1 (MCTS1), transcript variant 1, mRNA [NM_014060]	-0.69223547	0.0032869747
A_24_P237389	*DNAJC1*	Homo sapiens DnaJ (Hsp40) homolog, subfamily C, member 1 (DNAJC1), mRNA [NM_022365]	-0.7662532	0.012873199
A_23_P153583	*LOC101927768*	Homo sapiens uncharacterized LOC101927768 (LOC101927768), long non-coding RNA [NR_125845]	-0.9498538	0.0071605598
A_33_P3233666	*EIF1AX*	Homo sapiens eukaryotic translation initiation factor 1A, X-linked (EIF1AX), mRNA [NM_001412]	-0.36080864	0.013604386

A_32_P35800	*PPP5C*	Homo sapiens protein phosphatase 5, catalytic subunit (PPP5C), transcript variant 1, mRNA [NM_006247]	14202429	0.009864289
A_23_P13364	*RPP30*	Homo sapiens ribonuclease P/MRP 30kDa subunit (RPP30), transcript variant 1, mRNA [NM_001104546]	-0.645229	0.015210516
A_23_P432947	*ICE1*	Homo sapiens interactor of little elongation complex ELL subunit 1 (ICE1), mRNA [NM_015325]	-0.7730634	0.0033868244
A_24_P236235	*NUCB2*	Homo sapiens nucleobindin 2 (NUCB2), mRNA [NM_005013]	-0.77508855	0.0042072237
A_23_P147495	*GREM1*	Homo sapiens gremlin 1, DAN family BMP antagonist (GREM1), transcript variant 1, mRNA [NM_013372]	-15631106	0.007479662
A_23_P409623	*FLRT2*	Homo sapiens fibronectin leucine rich transmembrane protein 2 (FLRT2), mRNA [NM_013231]	-11646297	0.0049347663
A_21_P0007329	*BCORL1*	Homo sapiens BCL6 corepressor-like 1 (BCORL1), mRNA [NM_021946]	-14060524	0.00609916
A_24_P237586	*PPFIBP2*	Homo sapiens PTPRF interacting protein, binding protein 2 (liprin beta 2) (PPFIBP2), transcript variant 1, mRNA [NM_003621]	-0.79518247	0.009737427
A_22_P00010615	*lnc-CCDC34-1*	BX096431 Soares placenta Nb2HP Homo sapiens cDNA clone IMAGp998K15235, mRNA sequence [BX096431]	-17129402	0.0051130913
A_23_P27822	*ANKRD37*	Homo sapiens ankyrin repeat domain 37 (ANKRD37), mRNA [NM_181726]	-0.85724545	0.005852893
A_33_P3369153	*lnc-NDUFS5-1*	LNCipedia lincRNA (lnc-NDUFS5-1), lincRNA [lnc-NDUFS5-1:1]	-10350157	0.005936798
A_33_P3252369	*GPATCH1*	Homo sapiens G patch domain containing 1 (GPATCH1), mRNA [NM_018025]	-0.9668357	0.0048260237
A_32_P34589	*KIF3C*	Homo sapiens kinesin family member 3C (KIF3C), mRNA [NM_002254]	-10974492	0.009270997
A_23_P15402	*TMEM182*	Homo sapiens transmembrane protein 182 (TMEM182), mRNA [NM_144632]	-0.78663194	0.005612091
A_33_P3302518	*RSRC1*	Homo sapiens arginine/serine-rich coiled-coil 1 (RSRC1), transcript variant 2, mRNA [NM_016625]	-0.44729185	0.00910823
A_24_P416131	*SAT2*	Homo sapiens spermidine/spermine N1-acetyltransferase family member 2 (SAT2), mRNA [NM_133491]	-0.82904077	0.00427815
A_32_P117354	*CCDC40*	Homo sapiens coiled-coil domain containing 40 (CCDC40), transcript variant 2, mRNA [NM_001243342]	0.67186487	0.014737409
A_23_P88381	*COTL1*	Homo sapiens coactosin-like F-actin binding protein 1 (COTL1), mRNA [NM_021149]	-0.28702495	0.010734322

A_23_P49842	*LIMCH1*	Homo sapiens LIM and calponin homology domains 1 (LIMCH1), transcript variant 1, mRNA [NM_014988]	-4213737	0.0026564114
A_23_P370588	*NUMB*	Homo sapiens numb homolog (Drosophila) (NUMB), transcript variant 1, mRNA [NM_001005743]	0.39124346	0.007700591
A_23_P253561	*UNC119*	Homo sapiens unc-119 homolog (C. elegans) (UNC119), transcript variant 1, mRNA [NM_005148]	0.7515356	0.013926392
A_24_P90097	*HOXB8*	Homo sapiens homeobox B8 (HOXB8), mRNA [NM_024016]	15525541	0.0031474202
A_23_P391689	*TTPAL*	Homo sapiens tocopherol (alpha) transfer protein-like (TTPAL), transcript variant 1, mRNA [NM_024331]	-10185739	0.007889792
A_22_P00021578	*ADD3*	Homo sapiens adducin 3 (gamma) (ADD3), transcript variant 1, mRNA [NM_016824]	-2082964	0.0024890031
A_24_P235429	*PET100*	Homo sapiens PET100 homolog (S. cerevisiae) (PET100), transcript variant 1, mRNA [NM_001171155]	-0.7579918	0.0037856582
A_23_P29638	*TSGA10IP*	Homo sapiens testis specific, 10 interacting protein (TSGA10IP), mRNA [NM_152762]	0.64320993	0.009711265
A_23_P138139	*ABCA1*	Homo sapiens ATP-binding cassette, sub-family A (ABC1), member 1 (ABCA1), mRNA [NM_005502]	-0.830491	0.010942903
A_22_P00000839	*NCKIPSD*	Homo sapiens NCK interacting protein with SH3 domain (NCKIPSD), transcript variant 2, mRNA [NM_184231]	14696974	0.0025001846
A_19_P00316675	*OMA1*	Homo sapiens OMA1 zinc metallopeptidase (OMA1), mRNA [NM_145243]	-0.9655922	0.008427824
A_23_P146058	*lnc-AGBL1-3*	LNCipedia lincRNA (lnc-AGBL1-3), lincRNA [lnc-AGBL1-3:1]	11972985	0.014644525
A_33_P3236082	*MEG3*	Homo sapiens maternally expressed 3 (non-protein coding) (MEG3), transcript variant 5, long non-coding RNA [NR_033359]	26732965	0.0028288076
A_33_P3325502	*ATP6V1C1*	Homo sapiens ATPase, H+ transporting, lysosomal 42kDa, V1 subunit C1 (ATP6V1C1), mRNA [NM_001695]	-0.6185236	0.0072350414
A_33_P3284763	*NSDHL*	Homo sapiens NAD(P) dependent steroid dehydrogenase-like (NSDHL), transcript variant 2, mRNA [NM_001129765]	-0.5379941	0.009856158
A_33_P3277579	*ARHGAP29*	Homo sapiens Rho GTPase activating protein 29 (ARHGAP29), mRNA [NM_004815]	-19529631	0.005752781
A_23_P27724	*DMD*	Homo sapiens dystrophin (DMD), transcript variant Dp140b, mRNA [NM_004021]	-16823752	0.012211056
A_33_P3289145	*ZFYVE28*	Homo sapiens zinc finger, FYVE domain containing 28 (ZFYVE28), transcript variant 4, mRNA [NM_001172657]	0.937621	0.01666397

A_24_P117672	*SEPW1*	Homo sapiens selenoprotein W, 1 (SEPW1), mRNA [NM_003009]	-21026442	0.0027248112
A_23_P168965	*COPS8*	Homo sapiens COP9 signalosome subunit 8 (COPS8), transcript variant 2, mRNA [NM_198189]	-0.6041042	0.0105877705
A_33_P3841819	*SCAF1*	Homo sapiens SR-related CTD-associated factor 1 (SCAF1), mRNA [NM_021228]	22057033	0.0029471128
A_24_P68019	*C8orf44*	Homo sapiens chromosome 8 open reading frame 44 (C8orf44), mRNA [NM_019607]	-0.7789102	0.004349054
A_33_P3226135	*THUMPD3-AS1*	Homo sapiens THUMPD3 antisense RNA 1 (THUMPD3-AS1), long non-coding RNA [NR_027007]	-0.5825773	0.010447919
A_32_P78783	*ZNF551*	Homo sapiens zinc finger protein 551 (ZNF551), transcript variant 1, mRNA [NM_138347]	-0.36297384	0.016899958
A_23_P124733	*ZNF778*	Homo sapiens zinc finger protein 778 (ZNF778), transcript variant 1, mRNA [NM_001201407]	-0.9424846	0.007889792
A_24_P325520	*SH3BP5L*	Homo sapiens SH3-binding domain protein 5-like (SH3BP5L), mRNA [NM_030645]	-0.63282275	0.014822511
A_21_P0000322	*COQ2*	Homo sapiens coenzyme Q2 4-hydroxybenzoate polyprenyltransferase (COQ2), mRNA [NM_015697]	-0.6029065	0.012320746
A_19_P00320948	*SORT1*	Homo sapiens sortilin 1 (SORT1), transcript variant 1, mRNA [NM_002959]	-15798136	0.004912971
A_23_P134684	*SNORA34*	Homo sapiens small nucleolar RNA, H/ACA box 34 (SNORA34), small nucleolar RNA [NR_002968]	-0.32947898	0.0052959127
A_23_P122439	*FAM200B*	family with sequence similarity 200, member B [Source:HGNC Symbol;Acc:HGNC:27740] [ENST00000507992]	-11837065	0.0037479755
A_21_P0009343	*HMBOX1*	Homo sapiens homeobox containing 1 (HMBOX1), transcript variant 1, mRNA [NM_024567]	-10470836	0.014705425
A_33_P3284472	*BTN2A2*	Homo sapiens butyrophilin, subfamily 2, member A2 (BTN2A2), transcript variant 2, mRNA [NM_181531]	-0.7444145	0.0065866495
A_21_P0010774	*TBX2-AS1*	Homo sapiens TBX2 antisense RNA 1 (TBX2-AS1), transcript variant 1, long non-coding RNA [NR_125749]	13813105	0.006573993
A_23_P426305	*DDX51*	Homo sapiens DEAD (Asp-Glu-Ala-Asp) box polypeptide 51 (DDX51), mRNA [NM_175066]	11307501	0.010822477
A_21_P0007382	*lnc-PPIAL4G-4*	Q3SWV3_HUMAN (Q3SWV3) LOC389833 protein (Fragment), partial (8%) [THC2542300]	-13955935	0.0044240346
A_23_P159956	*AOC3*	Homo sapiens amine oxidase, copper containing 3 (AOC3), transcript variant 1, mRNA [NM_003734]	-0.6314112	0.013926392

A_23_P49021	*lnc-GPR83-2*	LNCipedia lincRNA (lnc-GPR83-2), lincRNA [lnc-GPR83-2:1]	10076302	0.010734322
A_22_P00018111	*MID2*	Homo sapiens midline 2 (MID2), transcript variant 1, mRNA [NM_012216]	-15221465	0.0053354
A_23_P252052	*WDR61*	Homo sapiens WD repeat domain 61 (WDR61), transcript variant 2, mRNA [NM_025234]	-0.88768405	0.0059128376
A_23_P150316	*FILIP1L*	Homo sapiens filamin A interacting protein 1-like (FILIP1L), transcript variant 1, mRNA [NM_182909]	-0.84181195	0.01080114
A_23_P347048	*SEC16A*	Homo sapiens SEC16 homolog A (S. cerevisiae) (SEC16A), transcript variant 1, mRNA [NM_014866]	10107694	0.015502214
A_33_P3400424	*MMP12*	Homo sapiens matrix metallopeptidase 12 (macrophage elastase) (MMP12), mRNA [NM_002426]	-2102993	0.0037583082
A_24_P237486	*SGPP1*	Homo sapiens sphingosine-1-phosphate phosphatase 1 (SGPP1), mRNA [NM_030791]	-0.4139637	0.0074008796
A_23_P334709	*ZNF615*	Homo sapiens zinc finger protein 615 (ZNF615), transcript variant 1, mRNA [NM_001199324]	-0.44201925	0.010958166
A_33_P3357049	*MECP2*	Homo sapiens methyl CpG binding protein 2 (MECP2), transcript variant 1, mRNA [NM_004992]	-0.6228681	0.012617283
A_23_P63655	*FKBP9*	Homo sapiens FK506 binding protein 9, 63 kDa (FKBP9), transcript variant 1, mRNA [NM_007270]	-0.6534252	0.007871983
A_24_P382467	*RPS15AP10*	Homo sapiens ribosomal protein S15a pseudogene 10 (RPS15AP10), non-coding RNA [NR_026768]	-14498249	0.008289811
A_22_P00015997	*ATP5C1*	Homo sapiens ATP synthase, H+ transporting, mitochondrial F1 complex, gamma polypeptide 1 (ATP5C1), transcript variant 2, mRNA [NM_005174]	-0.64429617	0.008110664
A_33_P3269899	*SLC39A3*	Homo sapiens solute carrier family 39 (zinc transporter), member 3 (SLC39A3), transcript variant 1, mRNA [NM_144564]	0.86299735	0.009029172
A_24_P532180	*RECK*	Homo sapiens reversion-inducing-cysteine-rich protein with kazal motifs (RECK), mRNA [NM_021111]	-20795894	0.0034903844
A_23_P85164	*FBXL20*	Homo sapiens F-box and leucine-rich repeat protein 20 (FBXL20), transcript variant 1, mRNA [NM_032875]	-11454538	0.014763061
A_23_P333218	*LOC90246*	Homo sapiens uncharacterized LOC90246 (LOC90246), long non-coding RNA [NR_026954]	13487755	0.0043846127
A_23_P58390	*DNASE1L1*	deoxyribonuclease I-like 1 [Source:HGNC Symbol;Acc:HGNC:2957] [ENST00000309585]	-15028235	0.008691653

A_23_P74467	*ERGIC1*	Homo sapiens endoplasmic reticulum-golgi intermediate compartment (ERGIC) 1 (ERGIC1), mRNA [NM_001031711]	-0.82869434	0.0077546863
A_21_P0011398	*C4orf32*	Homo sapiens chromosome 4 open reading frame 32 (C4orf32), mRNA [NM_152400]	-0.96745276	0.010573547
A_23_P202004	*KIAA0907*	Homo sapiens KIAA0907 (KIAA0907), mRNA [NM_014949]	-10433056	0.006632856
A_32_P38467	*WHAMMP3*	WAS protein homolog associated with actin, golgi membranes and microtubules pseudogene 3 [Source:HGNC Symbol;Acc:HGNC:27892] [ENST00000622062]	-13113248	0.014502948
A_23_P73150	*PRTFDC1*	Homo sapiens phosphoribosyl transferase domain containing 1 (PRTFDC1), transcript variant 1, mRNA [NM_020200]	-0.7091307	0.0039416323
A_21_P0000280	*SNHG8*	Homo sapiens small nucleolar RNA host gene 8 (non-protein coding) (SNHG8), transcript variant 1, long non-coding RNA [NR_003584]	-0.7061665	0.01211171
A_19_P00320229	*TTC25*	Homo sapiens tetratricopeptide repeat domain 25 (TTC25), transcript variant 1, mRNA [NM_031421]	-10413445	0.010968563
A_33_P3301221	*SNORD60*	Homo sapiens small nucleolar RNA, C/D box 60 (SNORD60), small nucleolar RNA [NR_002736]	-0.45279032	0.011402858
A_23_P216489	*FLJ46906*	Homo sapiens uncharacterized LOC441172 (FLJ46906), long non-coding RNA [NR_033896]	13991784	0.0041847117
A_22_P00018539	*MORN1*	Homo sapiens MORN repeat containing 1 (MORN1), transcript variant 3, non-coding RNA [NR_125361]	-10741496	0.0054365364
A_23_P1912	*GNE*	Homo sapiens glucosamine (UDP-N-acetyl)-2-epimerase/N-acetylmannosamine kinase (GNE), transcript variant 2, mRNA [NM_005476]	-0.7899468	0.010158614
A_23_P255785	*ERICH6-AS1*	Homo sapiens ERICH6 antisense RNA 1 (ERICH6-AS1), transcript variant 1, long non-coding RNA [NR_121674]	-0.29619017	0.012982396
A_24_P165259	*ZP1*	Homo sapiens zona pellucida glycoprotein 1 (sperm receptor) (ZP1), mRNA [NM_207341]	-0.9015901	0.007321179
A_33_P3344618	*NCK1*	Homo sapiens NCK adaptor protein 1 (NCK1), transcript variant 1, mRNA [NM_006153]	-0.39806935	0.005787274
A_23_P200386	*PYCR2*	Homo sapiens pyrroline-5-carboxylate reductase family, member 2 (PYCR2), transcript variant 1, mRNA [NM_013328]	-0.658869	0.01083596
A_23_P103442	*TFEB*	Homo sapiens transcription factor EB (TFEB), transcript variant 1, mRNA [NM_007162]	-13635526	0.009014158
A_22_P00014697	*KHDRBS1*	Homo sapiens KH domain containing, RNA binding, signal transduction associated 1 (KHDRBS1), transcript variant 1, mRNA [NM_006559]	-0.29129928	0.011383506

A_33_P3313401	*MEAF6*	Homo sapiens MYST/Esa1-associated factor 6 (MEAF6), transcript variant 1, mRNA [NM_022756]	-0.39536756	0.010817855
A_33_P3271445	*TPT1*	Homo sapiens tumor protein, translationally-controlled 1 (TPT1), transcript variant 1, mRNA [NM_001286272]	-0.4798984	0.016490895
A_23_P344421	*CYCS*	Homo sapiens cytochrome c, somatic (CYCS), mRNA [NM_018947]	-0.49255368	0.0069250474
A_33_P3220437	*COL4A3BP*	Homo sapiens collagen, type IV, alpha 3 (Goodpasture antigen) binding protein (COL4A3BP), transcript variant 3, mRNA [NM_001130105]	-1132858	0.004646996
A_23_P161446	*ROBO4*	Homo sapiens roundabout, axon guidance receptor, homolog 4 (Drosophila) (ROBO4), transcript variant 1, mRNA [NM_019055]	35770059	0.0032831472
A_23_P94921	*TNFAIP8L1*	Homo sapiens tumor necrosis factor, alpha-induced protein 8-like 1 (TNFAIP8L1), transcript variant 1, mRNA [NM_001167942]	-0.70356345	0.009039211
A_23_P165574	*GDI2*	Homo sapiens GDP dissociation inhibitor 2 (GDI2), transcript variant 1, mRNA [NM_001494]	0.33109763	0.0059040254
A_22_P00007462	*SLC20A2*	Homo sapiens solute carrier family 20 (phosphate transporter), member 2 (SLC20A2), transcript variant 2, mRNA [NM_006749]	-14989421	0.0057647754
A_33_P3346610	*IWS1*	Homo sapiens IWS1 homolog (S. cerevisiae) (IWS1), mRNA [NM_017969]	-0.992993	0.01208643
A_23_P16944	*PKN2-AS1*	Homo sapiens PKN2 antisense RNA 1 (PKN2-AS1), long non-coding RNA [NR_110682]	-0.92729783	0.00410343
A_21_P0012918	*SDC1*	Homo sapiens syndecan 1 (SDC1), transcript variant 1, mRNA [NM_001006946]	-0.83912516	0.011958462
A_23_P130352	*OVCA2*	Homo sapiens ovarian tumor suppressor candidate 2 (OVCA2), mRNA [NM_080822]	-0.5812936	0.0031941293
A_33_P3842551	*XLOC_l2_012150*	BROAD Institute lincRNA (XLOC_l2_012150), lincRNA [TCONS_l2_00023029]	-0.56475645	0.012600716
A_23_P159937	*KCTD1*	Homo sapiens potassium channel tetramerization domain containing 1 (KCTD1), transcript variant 2, mRNA [NM_198991]	0.6473639	0.015502214
A_33_P3329991	*IKZF2*	Homo sapiens IKAROS family zinc finger 2 (Helios) (IKZF2), transcript variant 2, mRNA [NM_001079526]	18739849	0.003381037
A_22_P00009101	*SLC6A8*	Homo sapiens solute carrier family 6 (neurotransmitter transporter), member 8 (SLC6A8), transcript variant 1, mRNA [NM_005629]	0.7552311	0.007658092
A_24_P56270	*lnc-LIFR-1*	LNCipedia lincRNA (lnc-LIFR-1), lincRNA [lnc-LIFR-1:1]	0.63559854	0.014025799
A_32_P85676	*SNORD86*	Homo sapiens small nucleolar RNA, C/D box 86 (SNORD86), small nucleolar RNA [NR_004399]	0.62169933	0.009264408

A_33_P3272231	*DYRK2*	Homo sapiens dual-specificity tyrosine-(Y)-phosphorylation regulated kinase 2 (DYRK2), transcript variant 2, mRNA [NM_006482]	-15487945	0.0031941293
A_33_P3254412	*STK32B*	Homo sapiens serine/threonine kinase 32B (STK32B), mRNA [NM_018401]	-11974391	0.003609745
A_33_P3398697	*MFSD2A*	Homo sapiens major facilitator superfamily domain containing 2A (MFSD2A), transcript variant 1, mRNA [NM_001136493]	12077265	0.00970286
A_23_P18123	*ULK2*	Homo sapiens unc-51 like autophagy activating kinase 2 (ULK2), transcript variant 1, mRNA [NM_014683]	-15184038	0.006652346
A_33_P3784283	*PICK1*	Homo sapiens protein interacting with PRKCA 1 (PICK1), transcript variant 1, mRNA [NM_012407]	-1029946	0.007874565
A_32_P166693	*NLGN1*	Homo sapiens neuroligin 1 (NLGN1), mRNA [NM_014932]	-3565037	0.0028703364
A_23_P206022	*JAK1*	Homo sapiens Janus kinase 1 (JAK1), mRNA [NM_002227]	-0.9062853	0.008425284
A_33_P3224878	*HEG1*	Homo sapiens heart development protein with EGF-like domains 1 (HEG1), mRNA [NM_020733]	-0.7512486	0.0027248112
A_33_P3376017	*ITGA11*	Homo sapiens integrin, alpha 11 (ITGA11), mRNA [NM_001004439]	-2275762	0.0056819725
A_33_P3422822	*ITGA4*	Homo sapiens integrin, alpha 4 (antigen CD49D, alpha 4 subunit of VLA-4 receptor) (ITGA4), mRNA [NM_000885]	-10272126	0.017028617
A_22_P00010760	*lnc-KIAA0319L-1*	Homo sapiens mRNA for proteasome beta 2 subunit variant protein. [AB209470]	-0.93798524	0.0034310205
A_23_P334955	*GJC2*	Homo sapiens gap junction protein, gamma 2, 47kDa (GJC2), mRNA [NM_020435]	-0.88301706	0.007149221
A_23_P334263	*FAM167A*	Homo sapiens family with sequence similarity 167, member A (FAM167A), mRNA [NM_053279]	0.89008015	0.012164148
A_33_P3323463	*TNNI1*	Homo sapiens troponin I type 1 (skeletal, slow) (TNNI1), mRNA [NM_003281]	0.6803153	0.011209355
A_21_P0009331	*SENP8*	Homo sapiens SUMO/sentrin specific peptidase family member 8 (SENP8), transcript variant 2, mRNA [NM_145204]	-0.8967012	0.016326603
A_19_P00315452	*TMEM92-AS1*	Homo sapiens TMEM92 antisense RNA 1 (TMEM92-AS1), long non-coding RNA [NR_125805]	13701622	0.0040017776
A_21_P0008404	*PI4KB*	Homo sapiens phosphatidylinositol 4-kinase, catalytic, beta (PI4KB), transcript variant 2, mRNA [NM_001198773]	0.69332665	0.010986003
A_33_P3414422	*LOC100130938*	PREDICTED: Homo sapiens uncharacterized LOC100130938 (LOC100130938), transcript variant X1, ncRNA [XR_110148]	0.5037562	0.013250427

A_22_P00007790	*LOC102723354*	Homo sapiens uncharacterized LOC102723354 (LOC102723354), long non-coding RNA [NR_110546]	-0.87162226	0.0063477047
A_23_P206960	*GPHN*	Homo sapiens gephyrin (GPHN), transcript variant 1, mRNA [NM_020806]	-0.7596175	0.006262615
A_23_P51699	*HNRNPU-AS1*	Homo sapiens HNRNPU antisense RNA 1 (HNRNPU-AS1), long non-coding RNA [NR_026778]	-15999374	0.004359316
A_24_P364296	*SEC14L1*	Homo sapiens SEC14-like 1 (S. cerevisiae) (SEC14L1), transcript variant 1, mRNA [NM_003003]	-0.78193945	0.0051410845
A_22_P00025404	*ARHGEF2*	Homo sapiens Rho/Rac guanine nucleotide exchange factor (GEF) 2 (ARHGEF2), transcript variant 3, mRNA [NM_004723]	-0.48097444	0.01042595
A_33_P3671291	*STX2*	Homo sapiens syntaxin 2 (STX2), transcript variant 1, mRNA [NM_001980]	-0.620315	0.013453215
A_23_P149281	*LSP1*	Homo sapiens lymphocyte-specific protein 1 (LSP1), transcript variant 5, mRNA [NM_001242932]	10244806	0.0050508454
A_23_P141394	*SNORA12*	EST91069 Synovial sarcoma Homo sapiens cDNA 5' end, mRNA sequence [AA378382]	-13372539	0.0066119307
A_32_P190049	*EPHA2*	Homo sapiens EPH receptor A2 (EPHA2), mRNA [NM_004431]	10311551	0.0066194455
A_21_P0013846	*WIPI1*	Homo sapiens WD repeat domain, phosphoinositide interacting 1 (WIPI1), mRNA [NM_017983]	-13351883	0.00324898
A_24_P2648	*LRRC58*	Homo sapiens leucine rich repeat containing 58 (LRRC58), mRNA [NM_001099678]	-0.33594844	0.017103462
A_19_P00318725	*LINC01420*	Homo sapiens long intergenic non-protein coding RNA 1420 (LINC01420), long non-coding RNA [NR_015367]	-0.8483832	0.010894719
A_23_P24375	*PTPN14*	Homo sapiens protein tyrosine phosphatase, non-receptor type 14 (PTPN14), mRNA [NM_005401]	-0.8347375	0.016087124
A_21_P0014915	*LINC01204*	long intergenic non-protein coding RNA 1204 [Source:HGNC Symbol;Acc:HGNC:49635] [ENST00000422047]	-2108688	0.005192162
A_24_P924862	*OTUB1*	Homo sapiens OTU deubiquitinase, ubiquitin aldehyde binding 1 (OTUB1), transcript variant 1, mRNA [NM_017670]	0.68366265	0.01268465
A_23_P52031	*RAPH1*	Homo sapiens Ras association (RalGDS/AF-6) and pleckstrin homology domains 1 (RAPH1), transcript variant 1, mRNA [NM_213589]	-13312635	0.004026994
A_23_P33791	*SMCO2*	Homo sapiens single-pass membrane protein with coiled-coil domains 2 (SMCO2), mRNA [NM_001145010]	-0.8495021	0.008427347
A_24_P141332	*PGM1*	Homo sapiens phosphoglucomutase 1 (PGM1), transcript variant 1, mRNA [NM_002633]	-13937078	0.009185285

A_19_P00315753	*SSBP2*	Homo sapiens single-stranded DNA binding protein 2 (SSBP2), transcript variant 2, mRNA [NM_012446]	-0.93589085	0.011958462
A_32_P220715	*CAMK2G*	Homo sapiens calcium/calmodulin-dependent protein kinase II gamma (CAMK2G), transcript variant 1, mRNA [NM_172171]	-10364399	0.005800329
A_32_P58074	*LINC00883*	Homo sapiens long intergenic non-protein coding RNA 883 (LINC00883), transcript variant 1, long non-coding RNA [NR_028301]	-0.70335305	0.009334625
A_33_P3351894	*MAP1LC3B*	Homo sapiens microtubule-associated protein 1 light chain 3 beta (MAP1LC3B), mRNA [NM_022818]	-0.6907599	0.008110185
A_23_P99260	*RPS3A*	Homo sapiens ribosomal protein S3A (RPS3A), transcript variant 1, mRNA [NM_001006]	-0.33975977	0.009938745
A_24_P77947	*MIF-AS1*	Homo sapiens MIF antisense RNA 1 (MIF-AS1), long non-coding RNA [NR_038911]	0.31559724	0.008564741
A_23_P204782	*METAP2*	Homo sapiens methionyl aminopeptidase 2 (METAP2), mRNA [NM_006838]	-0.6702914	0.014981894
A_23_P251916	*CCDC132*	Homo sapiens coiled-coil domain containing 132 (CCDC132), transcript variant 2, mRNA [NM_024553]	-11760342	0.012626196
A_23_P55616	*MDM1*	Homo sapiens Mdm1 nuclear protein homolog (mouse) (MDM1), transcript variant 2, mRNA [NM_020128]	-0.8186787	0.0061189965
A_33_P3275255	*RMDN1*	Homo sapiens regulator of microtubule dynamics 1 (RMDN1), transcript variant 1, mRNA [NM_016033]	-0.9756028	0.009864289
A_33_P3363271	*SLC14A1*	Homo sapiens solute carrier family 14 (urea transporter), member 1 (Kidd blood group) (SLC14A1), transcript variant 4, mRNA [NM_001146037]	33865445	0.003538446
A_22_P00005074	*CACNB2*	Homo sapiens calcium channel, voltage-dependent, beta 2 subunit (CACNB2), transcript variant 1, mRNA [NM_000724]	12482768	0.01524383
A_23_P337917	*lnc-DES-1*	Q4U2V9_CHLRE (Q4U2V9) Hydroxyproline-rich glycoprotein GAS30 precursor, partial (5%) [THC2602224]	0.37277886	0.016973978
A_22_P00009281	*HAS1*	Homo sapiens hyaluronan synthase 1 (HAS1), transcript variant 1, mRNA [NM_001523]	3112728	0.0027348737
A_22_P00021391	*PPFIBP1*	Homo sapiens PTPRF interacting protein, binding protein 1 (liprin beta 1) (PPFIBP1), transcript variant 2, mRNA [NM_177444]	-13938526	0.0056819725
A_23_P359897	*LRRC26*	Homo sapiens leucine rich repeat containing 26 (LRRC26), mRNA [NM_001013653]	12166526	0.010986003
A_33_P3271395	*DIP2A-IT1*	Homo sapiens DIP2A intronic transcript 1 (non-protein coding) (DIP2A-IT1), long non-coding RNA [NR_046400]	14920428	0.005058204

A_24_P352445	*KDM7A*	Homo sapiens lysine (K)-specific demethylase 7A (KDM7A), mRNA [NM_030647]	-0.6844921	0.002804883
A_22_P00010710	*LOC100129534*	Homo sapiens small nuclear ribonucleoprotein polypeptide N pseudogene (LOC100129534), non-coding RNA [NR_024489]	-16904337	0.0052243667
A_33_P3345608	*MRPL42*	Homo sapiens mitochondrial ribosomal protein L42 (MRPL42), transcript variant 2, mRNA [NM_172177]	-0.6753669	0.004829709
A_33_P3336915	*lnc-NFYB-1*	LNCipedia lincRNA (lnc-NFYB-1), lincRNA [lnc-NFYB-1:1]	-0.7538912	0.0049456456
A_23_P17345	*EDRF1*	Homo sapiens erythroid differentiation regulatory factor 1 (EDRF1), transcript variant 1, mRNA [NM_001202438]	0.40653357	0.012818519
A_21_P0007442	*MAFB*	Homo sapiens v-maf avian musculoaponeurotic fibrosarcoma oncogene homolog B (MAFB), mRNA [NM_005461]	-27146783	0.00431384
A_22_P00012507	*ZNF383*	Homo sapiens zinc finger protein 383 (ZNF383), mRNA [NM_152604]	-0.5951509	0.006161988
A_33_P3268234	*lnc-FOLR3-1*	LNCipedia lincRNA (lnc-FOLR3-1), lincRNA [lnc-FOLR3-1:2]	0.63339835	0.01447346
A_23_P351342	*LOC102724795*	PREDICTED: Homo sapiens uncharacterized LOC102724795 (LOC102724795), ncRNA [XR_424475]	-0.5506492	0.00338883
A_33_P3211443	*KRT39*	Homo sapiens keratin 39, type I (KRT39), mRNA [NM_213656]	0.37785524	0.013021452
A_23_P67339	*PLEKHM2*	Homo sapiens pleckstrin homology domain containing, family M (with RUN domain) member 2 (PLEKHM2), mRNA [NM_015164]	0.9380411	0.014307808
A_23_P56314	*LRRC8B*	Homo sapiens leucine rich repeat containing 8 family, member B (LRRC8B), transcript variant 1, mRNA [NM_015350]	-0.9893813	0.01042464
A_33_P3413741	*RCN3*	Homo sapiens reticulocalbin 3, EF-hand calcium binding domain (RCN3), mRNA [NM_020650]	13291868	0.0048015774
A_33_P3311458	*UQCR11*	Homo sapiens ubiquinol-cytochrome c reductase, complex III subunit XI (UQCR11), mRNA [NM_006830]	-0.7378504	0.0029471128
A_24_P233944	*OXTR*	Homo sapiens oxytocin receptor (OXTR), mRNA [NM_000916]	-25432916	0.0027248112
A_21_P0003208	*CEPT1*	Homo sapiens choline/ethanolamine phosphotransferase 1 (CEPT1), transcript variant 2, mRNA [NM_001007794]	-0.83415174	0.011618324
A_33_P3322353	*lnc-C20orf197-3*	LNCipedia lincRNA (lnc-C20orf197-3), lincRNA [lnc-C20orf197-3:13]	2062397	0.0051130913
A_23_P257668	*lnc-KIAA0226-3*	LNCipedia lincRNA (lnc-KIAA0226-3), lincRNA [lnc-KIAA0226-3:1]	-14866674	0.0070324177

A_21_P0001374	*CAPZA2*	Homo sapiens capping protein (actin filament) muscle Z-line, alpha 2 (CAPZA2), mRNA [NM_006136]	-10731231	0.010052036
A_23_P101319	*TTC26*	Homo sapiens tetratricopeptide repeat domain 26 (TTC26), transcript variant 1, mRNA [NM_024926]	-0.70603895	0.009524396
A_24_P681301	*NBPF15*	neuroblastoma breakpoint family, member 15 [Source:HGNC Symbol;Acc:HGNC:28791] [ENST00000584793]	0.2995448	0.014687806
A_33_P3252196	*ZNF285*	Homo sapiens zinc finger protein 285 (ZNF285), transcript variant 2, mRNA [NM_152354]	-0.58721817	0.0062876423
A_22_P00013907	*UBC*	Homo sapiens ubiquitin C (UBC), mRNA [NM_021009]	0.62610394	0.015678963
A_21_P0008390	*EZH2*	Homo sapiens enhancer of zeste homolog 2 (Drosophila) (EZH2), transcript variant 1, mRNA [NM_004456]	-0.2952049	0.013510108
A_23_P138352	*LINC00595*	long intergenic non-protein coding RNA 595 [Source:HGNC Symbol;Acc:HGNC:31430] [ENST00000459633]	-0.8528249	0.009968179
A_33_P3331376	*lnc-DLK1-6*	LNCipedia lincRNA (lnc-DLK1-6), lincRNA [lnc-DLK1-6:9]	0.5117166	0.00584425
A_24_P170753	*WNT2B*	Homo sapiens wingless-type MMTV integration site family, member 2B (WNT2B), transcript variant WNT-2B1, mRNA [NM_004185]	-19312818	0.0054341624
A_24_P171268	*EPHB2*	Homo sapiens EPH receptor B2 (EPHB2), transcript variant 2, mRNA [NM_004442]	16361291	0.0039856653
A_33_P3315724	*EP400NL*	EP400 N-terminal like [Source:HGNC Symbol;Acc:HGNC:26602] [ENST00000443539]	0.5856868	0.012873199
A_32_P138617	*RASSF5*	Homo sapiens Ras association (RalGDS/AF-6) domain family member 5 (RASSF5), transcript variant 1, mRNA [NM_182663]	0.8117832	0.016956452
A_32_P208403	*KIAA2018*	Homo sapiens KIAA2018 (KIAA2018), transcript variant 1, mRNA [NM_001009899]	-0.9409278	0.010480952
A_33_P3385062	*CDA*	Homo sapiens cytidine deaminase (CDA), mRNA [NM_001785]	16276416	0.005644507
A_23_P76350	*GNG2*	Homo sapiens guanine nucleotide binding protein (G protein), gamma 2 (GNG2), transcript variant 1, mRNA [NM_053064]	-11706853	0.0044319443
A_33_P3260455	*HAUS3*	HAUS augmin-like complex, subunit 3 [Source:HGNC Symbol;Acc:HGNC:28719] [ENST00000243706]	-0.7448597	0.0145022655
A_23_P372834	*GNB3*	Homo sapiens guanine nucleotide binding protein (G protein), beta polypeptide 3 (GNB3), transcript variant 1, mRNA [NM_002075]	11240079	0.009421056

A_33_P3405168	*AQP1*	Homo sapiens aquaporin 1 (Colton blood group) (AQP1), transcript variant 1, mRNA [NM_198098]	-18844459	0.0025001846
A_24_P227069	*AP2A1*	Homo sapiens adaptor-related protein complex 2, alpha 1 subunit (AP2A1), transcript variant 1, mRNA [NM_014203]	11224229	0.013248024
A_33_P7465707	*MMAA*	Homo sapiens methylmalonic aciduria (cobalamin deficiency) cblA type (MMAA), mRNA [NM_172250]	-13591361	0.010707185
A_32_P149492	*GPAM*	Homo sapiens glycerol-3-phosphate acyltransferase, mitochondrial (GPAM), transcript variant 2, mRNA [NM_020918]	-0.8165938	0.008242901
A_23_P61674	*KRAS*	Homo sapiens Kirsten rat sarcoma viral oncogene homolog (KRAS), transcript variant b, mRNA [NM_004985]	-10312743	0.004437345
A_24_P174824	*NBPF11*	Homo sapiens neuroblastoma breakpoint family, member 11 (NBPF11), transcript variant 3, mRNA [NM_183372]	-0.64587027	0.017006246
A_33_P3410351	*CLK4*	Homo sapiens CDC-like kinase 4 (CLK4), mRNA [NM_020666]	-1318176	0.00590614
A_23_P12884	*SLU7*	Homo sapiens SLU7 splicing factor homolog (S. cerevisiae) (SLU7), mRNA [NM_006425]	-10387001	0.00690275
A_24_P319354	*GSTM2*	Homo sapiens glutathione S-transferase mu 2 (muscle) (GSTM2), transcript variant 1, mRNA [NM_000848]	0.8673794	0.0122815035
A_24_P411815	*GRK5*	Homo sapiens G protein-coupled receptor kinase 5 (GRK5), mRNA [NM_005308]	-0.96078944	0.002538878
A_23_P307400	*SUMO1*	Homo sapiens small ubiquitin-like modifier 1 (SUMO1), transcript variant 2, mRNA [NM_001005781]	-0.73586273	0.015919259
A_23_P119627	*MPLKIP*	Homo sapiens M-phase specific PLK1 interacting protein (MPLKIP), mRNA [NM_138701]	-0.6764576	0.012832334
A_33_P3393927	*CEP95*	Homo sapiens centrosomal protein 95kDa (CEP95), mRNA [NM_138363]	-11382899	0.0027874883
A_32_P69465	*NDUFA13*	Homo sapiens NADH dehydrogenase (ubiquinone) 1 alpha subcomplex, 13 (NDUFA13), mRNA [NM_015965]	-0.39639547	0.016604615
A_33_P3296372	*MTMR11*	Homo sapiens myotubularin related protein 11 (MTMR11), transcript variant 2, mRNA [NM_181873]	-1329514	0.0052430327
A_24_P335305	*MORN2*	Homo sapiens MORN repeat containing 2 (MORN2), mRNA [NM_001145450]	-0.6080958	0.0073443917
A_23_P108751	*EIF2B3*	Homo sapiens eukaryotic translation initiation factor 2B, subunit 3 gamma, 58kDa (EIF2B3), transcript variant 1, mRNA [NM_020365]	-0.7164786	0.008106991
A_23_P386364	*OAS3*	Homo sapiens 2'-5'-oligoadenylate synthetase 3, 100kDa (OAS3), mRNA [NM_006187]	14537753	0.004055852

A_33_P3255304	*FHL2*	Homo sapiens four and a half LIM domains 2 (FHL2), transcript variant 5, mRNA [NM_001039492]	-0.91268444	0.0031941293
A_33_P3379726	*HDX*	Homo sapiens highly divergent homeobox (HDX), transcript variant 2, mRNA [NM_144657]	-0.80472016	0.002535607
A_33_P3315314	*GGT5*	Homo sapiens gamma-glutamyltransferase 5 (GGT5), transcript variant 1, mRNA [NM_001099781]	40380564	0.0031941293
A_23_P63870	*CCDC106*	Homo sapiens coiled-coil domain containing 106 (CCDC106), mRNA [NM_013301]	-10125421	0.011427788
A_24_P244356	*MT1HL1*	Homo sapiens metallothionein 1H-like 1 (MT1HL1), mRNA [NM_001276687]	16771712	0.0029691157
A_33_P3257222	*SAMD8*	Homo sapiens sterile alpha motif domain containing 8 (SAMD8), transcript variant 2, mRNA [NM_144660]	-0.6561049	0.012686283
A_23_P37347	*NLRX1*	Homo sapiens NLR family member X1 (NLRX1), transcript variant 4, mRNA [NM_001282144]	-12337453	0.010880077
A_33_P3251989	*COMTD1*	Homo sapiens catechol-O-methyltransferase domain containing 1 (COMTD1), mRNA [NM_144589]	-14472947	0.0051266714
A_23_P135239	*SNW1*	Homo sapiens SNW domain containing 1 (SNW1), mRNA [NM_012245]	-0.5970588	0.014183129
A_33_P3333033	*CRIPT*	Homo sapiens cysteine-rich PDZ-binding protein (CRIPT), mRNA [NM_014171]	-11518786	0.006587778
A_21_P0012455	*TLE1*	Homo sapiens transducin-like enhancer of split 1 (E(sp1) homolog, Drosophila) (TLE1), transcript variant 2, mRNA [NM_005077]	-1915986	0.0032224318
A_33_P3341365	*SGSM2*	small G protein signaling modulator 2 [Source:HGNC Symbol;Acc:HGNC:29026] [ENST00000574857]	-0.8752525	0.014370709
A_33_P3414574	*LOC101927056*	PREDICTED: Homo sapiens uncharacterized LOC101927056 (RP11-379B18.5), transcript variant X3, ncRNA [XR_425623]	-1210035	0.009630487
A_23_P82674	*RNF216*	Homo sapiens ring finger protein 216 (RNF216), transcript variant 1, mRNA [NM_207111]	-0.7693219	0.008959291
A_22_P00013817	*DNAJA2*	Homo sapiens DnaJ (Hsp40) homolog, subfamily A, member 2 (DNAJA2), mRNA [NM_005880]	-0.42282867	0.015203984
A_33_P3217689	*GBAS*	Homo sapiens glioblastoma amplified sequence (GBAS), transcript variant 1, mRNA [NM_001483]	-0.88180685	0.0031941293
A_23_P75299	*lnc-RPA2-1*	LNCipedia lincRNA (lnc-RPA2-1), lincRNA [lnc-RPA2-1:1]	-0.7900253	0.004160909
A_23_P404134	*JMY*	Homo sapiens junction mediating and regulatory protein, p53 cofactor (JMY), mRNA [NM_152405]	-12021006	0.0060627027

A_33_P3330099	*LHPP*	Homo sapiens phospholysine phosphohistidine inorganic pyrophosphate phosphatase (LHPP), transcript variant 1, mRNA [NM_022126]	-1627289	0.004359316
A_23_P26294	*TOX4*	TOX high mobility group box family member 4 [Source:HGNC Symbol;Acc:HGNC:20161] [ENST00000448790]	-0.8514473	0.009895355
A_23_P321959	*ARSD*	Homo sapiens arylsulfatase D (ARSD), mRNA [NM_001669]	-0.5766685	0.0066119307
A_22_P00015399	*TPSG1*	Homo sapiens tryptase gamma 1 (TPSG1), mRNA [NM_012467]	0.48623967	0.011487663
A_23_P129695	*SFT2D1*	Homo sapiens SFT2 domain containing 1 (SFT2D1), transcript variant 1, mRNA [NM_145169]	-0.7239749	0.003948561
A_32_P70135	*lnc-SRP9-1*	LNCipedia lincRNA (lnc-SRP9-1), lincRNA [lnc-SRP9-1:3]	-12705599	0.005787274
A_23_P97810	*VASN*	Homo sapiens vasorin (VASN), mRNA [NM_138440]	-0.4207008	0.010056441
A_23_P160154	*PROSER1*	Homo sapiens proline and serine rich 1 (PROSER1), mRNA [NM_025138]	-0.55836815	0.006080751
A_33_P3211604	*GALE*	Homo sapiens UDP-galactose-4-epimerase (GALE), transcript variant 1, mRNA [NM_000403]	135868	0.008559404
A_32_P29118	*CCDC28A*	Homo sapiens coiled-coil domain containing 28A (CCDC28A), mRNA [NM_015439]	-0.84828967	0.0073443917
A_23_P25994	*RAP1B*	Homo sapiens RAP1B, member of RAS oncogene family (RAP1B), transcript variant 1, mRNA [NM_015646]	-0.6461541	0.007332902
A_23_P201655	*SEMA3D*	Homo sapiens sema domain, immunoglobulin domain (Ig), short basic domain, secreted, (semaphorin) 3D (SEMA3D), mRNA [NM_152754]	-2305985	0.003190129
A_33_P3272352	*LGMN*	Homo sapiens legumain (LGMN), transcript variant 2, mRNA [NM_001008530]	-13429161	0.006727872
A_23_P336644	*MYCBP*	Homo sapiens MYC binding protein (MYCBP), transcript variant 1, mRNA [NM_012333]	-0.7876382	0.009784609
A_23_P20804	*TOR1AIP2*	Homo sapiens torsin A interacting protein 2 (TOR1AIP2), transcript variant 2, mRNA [NM_145034]	-0.48339647	0.014267268
A_24_P283320	*PRKRA*	Homo sapiens protein kinase, interferon-inducible double stranded RNA dependent activator (PRKRA), transcript variant 1, mRNA [NM_003690]	-0.26908898	0.008808054
A_24_P360078	*FAM219A*	Homo sapiens family with sequence similarity 219, member A (FAM219A), transcript variant 4, mRNA [NM_147202]	0.61460596	0.014260212
A_33_P3318187	*PCMT1*	Homo sapiens protein-L-isoaspartate (D-aspartate) O-methyltransferase (PCMT1), transcript variant 1, mRNA [NM_005389]	-0.63619465	0.0144334

A_23_P79842	*LRBA*	Homo sapiens LPS-responsive vesicle trafficking, beach and anchor containing (LRBA), transcript variant 2, mRNA [NM_006726]	-0.94064665	0.003381037
A_23_P79247	*CENPBD1*	Homo sapiens CENPB DNA-binding domains containing 1 (CENPBD1), mRNA [NM_145039]	-0.8184022	0.011949872
A_32_P113812	*PIGT*	Homo sapiens phosphatidylinositol glycan anchor biosynthesis, class T (PIGT), transcript variant 1, mRNA [NM_015937]	-12465508	0.0041523096
A_33_P3327587	*PGAP1*	Homo sapiens post-GPI attachment to proteins 1 (PGAP1), mRNA [NM_024989]	-0.69130623	0.014104453
A_23_P89030	*lnc-EIF2D-1*	Q9HBY7_HUMAN (Q9HBY7) Regulatory erythroid kinase long form (Dual-specificity tyrosine-phosphorylation regulated kinase 3 long isoform) (Dual-specificity tyrosine-(Y)-phosphorylation regulated kinase 3), partial (4%) [THC2689950]	0.38273007	0.014505501
A_33_P3327961	*LOC100127904*	Homo sapiens cDNA clone IMAGE:30403562, partial cds. [BC065739]	-0.92078334	0.009873189
A_24_P358245	*C16orf95*	Homo sapiens chromosome 16 open reading frame 95 (C16orf95), transcript variant 1, mRNA [NM_001195124]	-0.9332731	0.005236083
A_33_P8579064	*ZNF615*	Homo sapiens zinc finger protein 615 (ZNF615), transcript variant 1, mRNA [NM_001199324]	-10031621	0.010642556
A_21_P0006521	*ATP8B5P*	Homo sapiens ATPase, class I, type 8B, member 5, pseudogene (ATP8B5P), transcript variant 2, non-coding RNA [NR_003582]	-0.5428178	0.012818519
A_33_P3447304	*ZNF518A*	zinc finger protein 518A [Source:HGNC Symbol;Acc:HGNC:29009] [ENST00000484770]	-11619909	0.007854403
A_22_P00005682	*LOC101928092*	PREDICTED: Homo sapiens uncharacterized LOC101928092 (LOC101928092), ncRNA [XR_247293]	-22505698	0.0034792
A_23_P153945	*OXR1*	Homo sapiens oxidation resistance 1 (OXR1), transcript variant 4, mRNA [NM_001198533]	-12841095	0.004000086
A_23_P29994	*lnc-EIF6-1*	LNCipedia lincRNA (lnc-EIF6-1), lincRNA [lnc-EIF6-1:9]	-13762629	0.0064965826
A_33_P3367970	*GTDC1*	Homo sapiens glycosyltransferase-like domain containing 1 (GTDC1), transcript variant 1, mRNA [NM_001006636]	-18358653	0.0035432973
A_23_P39517	*RBPJ*	Homo sapiens recombination signal binding protein for immunoglobulin kappa J region (RBPJ), transcript variant 4, mRNA [NM_203284]	-0.25880077	0.011749808
A_33_P3390207	*SRBD1*	Homo sapiens S1 RNA binding domain 1 (SRBD1), mRNA [NM_018079]	-0.55796075	0.013744105

A_33_P3224971	*UTY*	ubiquitously transcribed tetratricopeptide repeat containing, Y-linked [Source:HGNC Symbol;Acc:HGNC:12638] [ENST00000382893]	-0.88875484	0.0059903036
A_23_P133585	*TMEM155*	Homo sapiens transmembrane protein 155 (TMEM155), mRNA [NM_152399]	19147918	0.0027248112
A_33_P3235891	*CDK7*	Homo sapiens cyclin-dependent kinase 7 (CDK7), mRNA [NM_001799]	-0.9799461	0.0050485265
A_23_P32903	*IQSEC1*	Homo sapiens IQ motif and Sec7 domain 1 (IQSEC1), transcript variant 1, mRNA [NM_001134382]	-0.75669646	0.017047122
A_21_P0001085	*OCRL*	Homo sapiens oculocerebrorenal syndrome of Lowe (OCRL), transcript variant a, mRNA [NM_000276]	-0.909916	0.0064385477
A_23_P91468	*TRAF3IP2-AS1*	Homo sapiens TRAF3IP2 antisense RNA 1 (TRAF3IP2-AS1), transcript variant 4, long non-coding RNA [NR_034111]	-0.7251273	0.0063919895
A_23_P201808	*LOC100130417*	Homo sapiens uncharacterized LOC100130417 (LOC100130417), transcript variant 1, long non-coding RNA [NR_026874]	14379479	0.00477571
A_33_P3214635	*PSMA7*	Homo sapiens proteasome (prosome, macropain) subunit, alpha type, 7 (PSMA7), mRNA [NM_002792]	-0.8334789	0.00549504
A_23_P257201	*PPAP2B*	Homo sapiens phosphatidic acid phosphatase type 2B (PPAP2B), mRNA [NM_003713]	-1144867	0.0034964236
A_24_P313096	*FECH*	Homo sapiens ferrochelatase (FECH), transcript variant 1, mRNA [NM_001012515]	-0.8676281	0.013841187
A_33_P3222105	*RNF146*	Homo sapiens ring finger protein 146 (RNF146), transcript variant 2, mRNA [NM_030963]	-12047863	0.002909564
A_24_P363745	*TUBGCP3*	Homo sapiens tubulin, gamma complex associated protein 3 (TUBGCP3), transcript variant 1, mRNA [NM_006322]	-0.45547032	0.009421056
A_22_P00009876	*EIF1AX*	Homo sapiens eukaryotic translation initiation factor 1A, X-linked (EIF1AX), mRNA [NM_001412]	-0.4270606	0.011772038
A_32_P221748	*LRRC14*	Homo sapiens leucine rich repeat containing 14 (LRRC14), transcript variant 2, mRNA [NM_014665]	-2814129	0.008298705
A_24_P406060	*lnc-MFAP5-1*	Q88DJ8_PSEPK (Q88DJ8) Precorrin-2 C20-methyltransferase, partial (7%) [THC2536936]	-17184064	0.004834548
A_23_P121250	*RNF144B*	Homo sapiens ring finger protein 144B (RNF144B), mRNA [NM_182757]	-173169	0.003956951
A_33_P6806144	*TEK*	Homo sapiens TEK tyrosine kinase, endothelial (TEK), transcript variant 1, mRNA [NM_000459]	-2499082	0.002804883
A_23_P417282	*EIF4A2*	Homo sapiens eukaryotic translation initiation factor 4A2 (EIF4A2), mRNA [NM_001967]	-14180032	0.0022181747

A_23_P69791	*LINC00476*	Homo sapiens long intergenic non-protein coding RNA 476 (LINC00476), transcript variant 1, long non-coding RNA [NR_023390]	-11294363	0.013079763
A_24_P487736	*IGF1R*	Homo sapiens insulin-like growth factor 1 receptor (IGF1R), transcript variant 1, mRNA [NM_000875]	-0.56888074	0.010402669
A_33_P3363420	*AP1AR*	Homo sapiens adaptor-related protein complex 1 associated regulatory protein (AP1AR), transcript variant 1, mRNA [NM_018569]	-0.7483939	0.01262842
A_19_P00320119	*CXorf23*	Homo sapiens chromosome X open reading frame 23 (CXorf23), mRNA [NM_198279]	-15002643	0.0044240346
A_23_P204564	*FRMD3*	Homo sapiens FERM domain containing 3 (FRMD3), transcript variant 1, mRNA [NM_174938]	-0.80393314	0.0116504505
A_32_P191004	*TUG1*	Homo sapiens taurine up-regulated 1 (non-protein coding) (TUG1), transcript variant 1, long non-coding RNA [NR_110492]	-0.7556596	0.010447919
A_23_P32064	*PPP1R12A*	Homo sapiens protein phosphatase 1, regulatory subunit 12A (PPP1R12A), transcript variant 1, mRNA [NM_002480]	-0.92659235	0.0044853417
A_23_P91910	*ATAD2B*	Homo sapiens ATPase family, AAA domain containing 2B (ATAD2B), transcript variant 1, mRNA [NM_017552]	-0.846908	0.01179784
A_22_P00016991	*NSMF*	Homo sapiens NMDA receptor synaptonuclear signaling and neuronal migration factor (NSMF), transcript variant 2, mRNA [NM_015537]	1492643	0.0029691157
A_33_P3407618	*PLSCR4*	Homo sapiens phospholipid scramblase 4 (PLSCR4), transcript variant 2, mRNA [NM_020353]	-10414225	0.001417249
A_23_P142994	*lnc-TTC26-2*	LNCipedia lincRNA (lnc-TTC26-2), lincRNA [lnc-TTC26-2:1]	1363471	0.0054365364
A_33_P3335845	*LOC727751*	Homo sapiens golgin A2 pseudogene (LOC727751), transcript variant 1, non-coding RNA [NR_102747]	11572106	0.009593207
A_23_P376096	*RBM45*	Homo sapiens RNA binding motif protein 45 (RBM45), mRNA [NM_152945]	-0.55337685	0.0100471685
A_22_P00012561	*WDR33*	Homo sapiens WD repeat domain 33 (WDR33), transcript variant 2, mRNA [NM_001006622]	-0.91453314	0.008298705
A_23_P373708	*TICAM1*	Homo sapiens toll-like receptor adaptor molecule 1 (TICAM1), mRNA [NM_182919]	11344316	0.003956951
A_21_P0010857	*LOC100506476*	Homo sapiens uncharacterized LOC100506476 (LOC100506476), long non-coding RNA [NR_109995]	-26080847	0.0058377855
A_24_P397515	*KRT18P55*	Homo sapiens keratin 18 pseudogene 55 (KRT18P55), non-coding RNA [NR_028334]	21422153	0.0037583082

A_23_P209564	*HMGN4*	Homo sapiens high mobility group nucleosomal binding domain 4 (HMGN4), mRNA [NM_006353]	-0.5443676	0.013127201
A_33_P3274199	*lnc-FAS-1*	PREDICTED: Homo sapiens Fas cell surface death receptor (FAS), transcript variant X1, mRNA [XM_006717819]	-13522813	0.010519677
A_21_P0002358	*CYBRD1*	Homo sapiens cytochrome b reductase 1 (CYBRD1), transcript variant 1, mRNA [NM_024843]	-14590094	0.0018723286
A_23_P28375	*TP53I13*	Homo sapiens tumor protein p53 inducible protein 13 (TP53I13), mRNA [NM_138349]	-0.8296859	0.012061465
A_23_P148969	*GMCL1*	Homo sapiens germ cell-less, spermatogenesis associated 1 (GMCL1), mRNA [NM_178439]	-0.7601391	0.004326644
A_23_P51996	*EFCAB11*	Homo sapiens EF-hand calcium binding domain 11 (EFCAB11), transcript variant 1, mRNA [NM_145231]	-0.97395486	0.006485358
A_24_P36868	*LRRC40*	Homo sapiens leucine rich repeat containing 40 (LRRC40), mRNA [NM_017768]	-0.66972613	0.003162355
A_33_P3379506	*STXBP3*	Homo sapiens syntaxin binding protein 3 (STXBP3), mRNA [NM_007269]	-0.8570564	0.0039633843
A_33_P3413335	*WDR26*	Homo sapiens WD repeat domain 26 (WDR26), transcript variant 1, mRNA [NM_025160]	-0.7460031	0.008559404
A_33_P3292307	*MITD1*	Homo sapiens MIT, microtubule interacting and transport, domain containing 1 (MITD1), mRNA [NM_138798]	-0.81053394	0.0070101344
A_33_P3303066	*UBIAD1*	Homo sapiens UbiA prenyltransferase domain containing 1 (UBIAD1), mRNA [NM_013319]	0.7488085	0.012756575
A_23_P73702	*CDK17*	Homo sapiens cyclin-dependent kinase 17 (CDK17), transcript variant 2, mRNA [NM_001170464]	0.68609494	0.014392089
A_33_P3309271	*BTBD2*	Homo sapiens BTB (POZ) domain containing 2 (BTBD2), mRNA [NM_017797]	12658691	0.0067226742
A_33_P3293207	*MED12*	Homo sapiens mediator complex subunit 12 (MED12), mRNA [NM_005120]	10950863	0.01268465
A_21_P0006653	*BLOC1S5*	Homo sapiens biogenesis of lysosomal organelles complex-1, subunit 5, muted (BLOC1S5), transcript variant 3, mRNA [NM_001199323]	-12336766	0.009338006
A_24_P135322	*C8orf37*	Homo sapiens chromosome 8 open reading frame 37 (C8orf37), mRNA [NM_177965]	-0.9991996	0.005612091
A_33_P3227551	*NRP1*	Homo sapiens neuropilin 1 (NRP1), transcript variant 3, mRNA [NM_001024629]	-10841697	0.006812377
A_23_P54116	*GATSL2*	Homo sapiens GATS protein-like 2 (GATSL2), mRNA [NM_001145064]	0.84303474	0.009032192
A_21_P0002265	*ZNF331*	zinc finger protein 331 [Source:HGNC Symbol;Acc:HGNC:15489] [ENST00000509069]	28057532	0.0029471128

A_24_P645765	*DAAM1*	Homo sapiens dishevelled associated activator of morphogenesis 1 (DAAM1), transcript variant 1, mRNA [NM_014992]	-183519	0.005348777
A_23_P301360	*lnc-MRPS9-2*	LNCipedia lincRNA (lnc-MRPS9-2), lincRNA [lnc-MRPS9-2:2]	0.46911165	0.015249878
A_33_P3216372	*KLHL42*	Homo sapiens kelch-like family member 42 (KLHL42), mRNA [NM_020782]	-0.6370053	0.008570872
A_23_P2423	*ZNF572*	Homo sapiens zinc finger protein 572 (ZNF572), mRNA [NM_152412]	-18917627	0.004359316
A_24_P691826	*IQGAP2*	Homo sapiens IQ motif containing GTPase activating protein 2 (IQGAP2), transcript variant 1, mRNA [NM_006633]	11487956	0.0057806885
A_22_P00000258	*MAGOHB*	Homo sapiens mago-nashi homolog B (Drosophila) (MAGOHB), transcript variant 1, mRNA [NM_018048]	-0.94732857	0.0073193884
A_24_P81947	*WFDC21P*	WAP four-disulfide core domain 21, pseudogene [Source:HGNC Symbol;Acc:HGNC:50357] [ENST00000587298]	0.46310785	0.0073443917
A_23_P13065	*lnc-AC007405.2.1-3*	DA688068 NT2NE2 Homo sapiens cDNA clone NT2NE2006764 5', mRNA sequence [DA688068]	10466481	0.0024890031
A_19_P00316396	*CORO1C*	Homo sapiens coronin, actin binding protein, 1C (CORO1C), transcript variant 2, mRNA [NM_014325]	-0.64909554	0.0040017776
A_21_P0001911	*ZDHHC13*	Homo sapiens zinc finger, DHHC-type containing 13 (ZDHHC13), transcript variant 1, mRNA [NM_019028]	-0.7313993	0.0037583082
A_23_P34930	*MEG3*	Homo sapiens maternally expressed 3 (non-protein coding) (MEG3), transcript variant 16, long non-coding RNA [NR_046473]	0.9479181	0.009784609
A_23_P368225	*NIFK-AS1*	Homo sapiens NIFK antisense RNA 1 (NIFK-AS1), transcript variant 2, long non-coding RNA [NR_037858]	-0.8594453	0.009742341
A_33_P3281613	*BCAS2*	Homo sapiens breast carcinoma amplified sequence 2 (BCAS2), mRNA [NM_005872]	-0.5430312	0.0058555133
A_33_P3277988	*EME1*	Homo sapiens essential meiotic structure-specific endonuclease 1 (EME1), transcript variant 2, mRNA [NM_152463]	16099802	0.0041847117
A_24_P181506	*KCNT1*	Homo sapiens cDNA FLJ41282 fis, clone BRAMY2037823. [AK123276]	-0.7890807	0.00431082
A_22_P00018345	*IFNAR1*	Homo sapiens interferon (alpha, beta and omega) receptor 1 (IFNAR1), mRNA [NM_000629]	-0.50888973	0.010860952
A_23_P502350	*ZNF646*	Homo sapiens zinc finger protein 646 (ZNF646), mRNA [NM_014699]	-0.90326446	0.009630487
A_23_P102060	*ZNF205-AS1*	Homo sapiens ZNF205 antisense RNA 1 (ZNF205-AS1), transcript variant 1, long non-coding RNA [NR_024166]	0.4857027	0.011026111

A_33_P3866631	*RFX2*	Homo sapiens regulatory factor X, 2 (influences HLA class II expression) (RFX2), transcript variant 1, mRNA [NM_000635]	-11509287	0.012643002
A_23_P128744	*SSFA2*	Homo sapiens sperm specific antigen 2 (SSFA2), transcript variant 2, mRNA [NM_006751]	-0.26366135	0.006004045
A_24_P320328	*DKFZP564C152*	Homo sapiens mRNA; cDNA DKFZp564C152 (from clone DKFZp564C152) [AL049980]	-13310316	0.00418896
A_32_P514790	*BDKRB1*	Homo sapiens bradykinin receptor B1 (BDKRB1), mRNA [NM_000710]	2892513	0.003414849
A_24_P940135	*SUB1*	Homo sapiens SUB1 homolog (S. cerevisiae) (SUB1), mRNA [NM_006713]	-0.57467747	0.009917321
A_23_P27983	*UNK*	Homo sapiens unkempt family zinc finger (UNK), transcript variant 1, mRNA [NM_001080419]	-12750962	0.009924296
A_33_P3222664	*CTBS*	Homo sapiens chitobiase, di-N-acetyl- (CTBS), mRNA [NM_004388]	-0.98038363	0.009074582
A_23_P421526	*APLP1*	Homo sapiens amyloid beta (A4) precursor-like protein 1 (APLP1), transcript variant 2, mRNA [NM_005166]	10459512	0.013802298
A_23_P154345	*ODF4*	Homo sapiens outer dense fiber of sperm tails 4 (ODF4), mRNA [NM_153007]	0.79483473	0.01571288
A_23_P323272	*HIBCH*	Homo sapiens 3-hydroxyisobutyryl-CoA hydrolase (HIBCH), transcript variant 1, mRNA [NM_014362]	-0.473969	0.007115449
A_23_P19210	*PBRM1*	Homo sapiens polybromo 1 (PBRM1), mRNA [NM_018313]	-0.7032737	0.002804883
A_23_P149992	*OSR1*	Homo sapiens odd-skipped related transciption factor 1 (OSR1), mRNA [NM_145260]	-1636417	0.0044240346
A_33_P3411885	*RPF2*	Homo sapiens ribosome production factor 2 homolog (S. cerevisiae) (RPF2), transcript variant 1, mRNA [NM_032194]	-0.36674947	0.0140619585
A_33_P3406702	*PDLIM1*	Homo sapiens PDZ and LIM domain 1 (PDLIM1), mRNA [NM_020992]	0.5958276	0.010158614
A_33_P3360097	*LOC100128851*	Homo sapiens cDNA FLJ45515 fis, clone BRTHA2022914. [AK127423]	0.76486874	0.009507545
A_32_P108254	*APRT*	Homo sapiens adenine phosphoribosyltransferase (APRT), transcript variant 1, mRNA [NM_000485]	-0.70080394	0.012609954
A_23_P126844	*CA5BP1*	Homo sapiens carbonic anhydrase VB pseudogene 1 (CA5BP1), non-coding RNA [NR_026551]	13908743	0.012525387
A_23_P63038	*FAM20A*	Homo sapiens family with sequence similarity 20, member A (FAM20A), transcript variant 1, mRNA [NM_017565]	-20750544	0.007768678

A_21_P0010084	*TNFRSF25*	Homo sapiens tumor necrosis factor receptor superfamily, member 25 (TNFRSF25), transcript variant 1, mRNA [NM_148965]	-0.93160766	0.012131026
A_33_P3262670	*P3H1*	Homo sapiens prolyl 3-hydroxylase 1 (P3H1), transcript variant 1, mRNA [NM_022356]	-0.77550197	0.011680649
A_33_P3384667	*LOC100270804*	Homo sapiens uncharacterized LOC100270804 (LOC100270804), long non-coding RNA [NR_026885]	-10135963	0.011937011
A_23_P26024	*MAP7D3*	Homo sapiens MAP7 domain containing 3 (MAP7D3), transcript variant 3, mRNA [NM_001173517]	-12768043	0.0046024295
A_33_P3317123	*DCAF5*	Homo sapiens DDB1 and CUL4 associated factor 5 (DCAF5), transcript variant 4, mRNA [NM_001284208]	-0.71584773	0.0046812603
A_23_P317324	*C15orf48*	Homo sapiens chromosome 15 open reading frame 48 (C15orf48), transcript variant 2, mRNA [NM_032413]	24372025	0.0025001846
A_23_P131375	*SEC24C*	Homo sapiens SEC24 family member C (SEC24C), transcript variant 1, mRNA [NM_004922]	-0.4386949	0.01685728
A_23_P147383	*MECOM*	Homo sapiens MDS1 and EVI1 complex locus (MECOM), transcript variant 2, mRNA [NM_005241]	27802262	0.0027248112
A_21_P0005923	*PQLC3*	Homo sapiens PQ loop repeat containing 3 (PQLC3), transcript variant 1, mRNA [NM_152391]	-0.9683139	0.0044240346
A_33_P3397579	*GPAA1*	Homo sapiens glycosylphosphatidylinositol anchor attachment 1 (GPAA1), mRNA [NM_003801]	10444194	0.012994969
A_23_P43248	*LOC100506990*	Homo sapiens uncharacterized LOC100506990 (LOC100506990), transcript variant 2, long non-coding RNA [NR_040092]	-0.96799767	0.009711265
A_33_P3315355	*TAF2*	Homo sapiens TAF2 RNA polymerase II, TATA box binding protein (TBP)-associated factor, 150kDa (TAF2), mRNA [NM_003184]	-0.7008281	0.004220053
A_23_P18465	*MARC2*	Homo sapiens mitochondrial amidoxime reducing component 2 (MARC2), mRNA [NM_017898]	-10932052	0.0118191205
A_33_P3415500	*SETD6*	Homo sapiens SET domain containing 6 (SETD6), transcript variant 1, mRNA [NM_001160305]	-0.65534365	0.011722619
A_23_P215956	*RFC1*	Homo sapiens replication factor C (activator 1) 1, 145kDa (RFC1), transcript variant 1, mRNA [NM_002913]	-0.5702436	0.012746598
A_24_P358131	*TRIO*	trio Rho guanine nucleotide exchange factor [Source:HGNC Symbol;Acc:HGNC:12303] [ENST00000620511]	-11923449	0.004810816

A_23_P80954	*MYC*	Homo sapiens v-myc avian myelocytomatosis viral oncogene homolog (MYC), mRNA [NM_002467]	-0.8859947	0.006871239
A_33_P3379947	*SLC26A1*	Homo sapiens solute carrier family 26 (anion exchanger), member 1 (SLC26A1), transcript variant 1, mRNA [NM_022042]	0.32841212	0.0062257075
A_33_P3394198	*MED18*	Homo sapiens mediator complex subunit 18 (MED18), transcript variant 1, mRNA [NM_017638]	-26845765	0.0034903844
A_23_P145376	*HLA-B*	Homo sapiens major histocompatibility complex, class I, B (HLA-B), mRNA [NM_005514]	-13236728	0.013281833
A_24_P121271	*FPGS*	Homo sapiens folylpolyglutamate synthase (FPGS), transcript variant 2, mRNA [NM_001018078]	0.76639324	0.010383267
A_22_P00007550	*MAPK13*	Homo sapiens mitogen-activated protein kinase 13 (MAPK13), transcript variant 1, mRNA [NM_002754]	15998693	0.007103236
A_23_P324523	*CPSF6*	Homo sapiens cleavage and polyadenylation specific factor 6, 68kDa (CPSF6), transcript variant 1, mRNA [NM_007007]	-0.5202779	0.009938745
A_23_P14105	*lnc-HAPLN1-1*	LNCipedia lincRNA (lnc-HAPLN1-1), lincRNA [lnc-HAPLN1-1:1]	-11163538	0.0035097655
A_32_P831725	*IQCK*	Homo sapiens IQ motif containing K (IQCK), mRNA [NM_153208]	-11229403	0.010518581
A_23_P389102	*RCBTB2*	Homo sapiens regulator of chromosome condensation (RCC1) and BTB (POZ) domain containing protein 2 (RCBTB2), transcript variant 2, mRNA [NM_001268]	-0.6124339	0.010274393
A_21_P0005092	*C3orf62*	Homo sapiens chromosome 3 open reading frame 62 (C3orf62), mRNA [NM_198562]	10178992	0.0066283424
A_33_P3333667	*MYO1D*	Homo sapiens myosin ID (MYO1D), transcript variant 1, mRNA [NM_015194]	-19736216	0.005132046
A_24_P38276	*LOC101929484*	PREDICTED: Homo sapiens uncharacterized LOC101929484 (LOC101929484), ncRNA [XR_245642]	-31183991	0.004835474
A_23_P348383	*XRRA1*	Homo sapiens X-ray radiation resistance associated 1 (XRRA1), transcript variant 2, mRNA [NM_001270380]	1465182	0.013143453
A_21_P0006503	*FZD1*	Homo sapiens frizzled class receptor 1 (FZD1), mRNA [NM_003505]	-0.58730793	0.006165438
A_23_P200493	*CC2D2A*	Homo sapiens coiled-coil and C2 domain containing 2A (CC2D2A), transcript variant 1, mRNA [NM_001080522]	-0.78100306	0.002804883
A_33_P3451157	*ZC3H12B*	zinc finger CCCH-type containing 12B [Source:HGNC Symbol;Acc:HGNC:17407] [ENST00000617377]	11277502	0.014739164

A_23_P116414	*LBR*	Homo sapiens lamin B receptor (LBR), transcript variant 1, mRNA [NM_002296]	-0.5190344	0.010272158
A_23_P209740	*PLA2G16*	Homo sapiens phospholipase A2, group XVI (PLA2G16), transcript variant 1, mRNA [NM_007069]	-2425326	0.0024890031
A_33_P3246007	*NAIF1*	Homo sapiens nuclear apoptosis inducing factor 1 (NAIF1), mRNA [NM_197956]	-0.9377418	0.006586824
A_32_P89679	*PSMD1*	Homo sapiens proteasome (prosome, macropain) 26S subunit, non-ATPase, 1 (PSMD1), transcript variant 1, mRNA [NM_002807]	-0.54894114	0.00687431
A_33_P3216008	*APOA1BP*	Homo sapiens apolipoprotein A-I binding protein (APOA1BP), mRNA [NM_144772]	-0.80163455	0.003969615
A_33_P3339066	*ALG10B*	Homo sapiens ALG10B, alpha-1,2-glucosyltransferase (ALG10B), mRNA [NM_001013620]	-0.579765	0.006543285
A_23_P90099	*SKA3*	Homo sapiens spindle and kinetochore associated complex subunit 3 (SKA3), transcript variant 1, mRNA [NM_145061]	-0.5524771	0.015576043
A_21_P0009196	*RNPC3*	Homo sapiens RNA-binding region (RNP1, RRM) containing 3 (RNPC3), mRNA [NM_017619]	-0.7419351	0.0134738535
A_23_P397293	*TMEM205*	Homo sapiens transmembrane protein 205 (TMEM205), transcript variant 1, mRNA [NM_198536]	-0.71500707	0.015330245
A_33_P3299510	*TMEM92-AS1*	Homo sapiens TMEM92 antisense RNA 1 (TMEM92-AS1), long non-coding RNA [NR_125805]	13667036	0.0044813035
A_33_P3404448	*LY6K*	Homo sapiens lymphocyte antigen 6 complex, locus K (LY6K), transcript variant 1, mRNA [NM_017527]	18323724	0.004297874
A_21_P0011461	*SCX*	Homo sapiens scleraxis basic helix-loop-helix transcription factor (SCX), mRNA [NM_001080514]	17134957	0.0035939496
A_23_P89621	*SNRNP48*	Homo sapiens small nuclear ribonucleoprotein 48kDa (U11/U12) (SNRNP48), mRNA [NM_152551]	-0.5234921	0.0160096
A_32_P88415	*XLOC_l2_005438*	BROAD Institute lincRNA (XLOC_l2_005438), lincRNA [TCONS_l2_00010087]	0.31532025	0.011363052
A_23_P122375	*CBX4*	Homo sapiens chromobox homolog 4 (CBX4), mRNA [NM_003655]	1100878	0.01613243
A_23_P54477	*MYOZ3*	Homo sapiens myozenin 3 (MYOZ3), transcript variant 2, mRNA [NM_133371]	-0.980489	0.008634653
A_22_P00008771	*ZFAND3*	Homo sapiens zinc finger, AN1-type domain 3 (ZFAND3), mRNA [NM_021943]	0.50859004	0.0062159016
A_21_P0011359	*NOP10*	Homo sapiens NOP10 ribonucleoprotein (NOP10), mRNA [NM_018648]	-0.63241315	0.0071450765

A_24_P940166	*LOC101927979*	PREDICTED: Homo sapiens uncharacterized LOC101927979 (RP5-1050D4.5), ncRNA [XR_243582]	-0.70661277	0.008976597
A_24_P3005	*PAPSS2*	Homo sapiens 3'-phosphoadenosine 5'-phosphosulfate synthase 2 (PAPSS2), transcript variant 2, mRNA [NM_001015880]	-12688305	0.006632856
A_33_P3323999	*NR4A2*	Homo sapiens nuclear receptor subfamily 4, group A, member 2 (NR4A2), mRNA [NM_006186]	317101	0.0029691157
A_22_P00008143	*SCN9A*	Homo sapiens sodium channel, voltage gated, type IX alpha subunit (SCN9A), mRNA [NM_002977]	2605662	0.00340924
A_32_P95823	*SBF1*	Homo sapiens SET binding factor 1 (SBF1), mRNA [NM_002972]	-11472726	0.0056838957
DCP_22_0	*LOC101927841*		0.9845761	0.009161951
A_23_P202219	*UBXN4*	Homo sapiens UBX domain protein 4 (UBXN4), mRNA [NM_014607]	-0.38036224	0.010827594
A_33_P3393821	*CALHM2*	Homo sapiens calcium homeostasis modulator 2 (CALHM2), transcript variant 1, mRNA [NM_015916]	-0.8660126	0.0057647754
A_23_P41908	*DAK*	Homo sapiens dihydroxyacetone kinase 2 homolog (S. cerevisiae) (DAK), mRNA [NM_015533]	-10164719	0.0044319443
A_24_P48898	*C1R*	Homo sapiens complement component 1, r subcomponent (C1R), mRNA [NM_001733]	-0.6739824	0.0025001846
A_23_P68717	*FAM114A2*	Homo sapiens family with sequence similarity 114, member A2 (FAM114A2), mRNA [NM_018691]	-0.72213584	0.009425872
A_23_P168229	*APOL2*	Homo sapiens apolipoprotein L, 2 (APOL2), transcript variant beta, mRNA [NM_145637]	-10573914	0.010760281
A_33_P3386506	*PSMG1*	Homo sapiens proteasome (prosome, macropain) assembly chaperone 1 (PSMG1), transcript variant 1, mRNA [NM_003720]	-0.2747956	0.005936798
A_23_P79622	*TXNDC5*	Homo sapiens thioredoxin domain containing 5 (endoplasmic reticulum) (TXNDC5), transcript variant 1, mRNA [NM_030810]	-0.9194202	0.0048452313
A_22_P00025397	*NFKBIL1*	Homo sapiens nuclear factor of kappa light polypeptide gene enhancer in B-cells inhibitor-like 1 (NFKBIL1), transcript variant 2, mRNA [NM_001144961]	11330564	0.007610532
A_32_P23010	*FKBP7*	Homo sapiens FK506 binding protein 7 (FKBP7), transcript variant 1, mRNA [NM_181342]	-0.7379715	0.012250454
A_23_P385217	*lnc-OR1Q1-1*	603073365F1 NIH_MGC_119 Homo sapiens cDNA clone IMAGE:5165105 5', mRNA sequence [BI830485]	10068223	0.0166364

A_33_P3290748	*SDHAF1*	Homo sapiens succinate dehydrogenase complex assembly factor 1 (SDHAF1), mRNA [NM_001042631]	-0.80170006	0.011672338
A_21_P0000469	*ARL8B*	Homo sapiens ADP-ribosylation factor-like 8B (ARL8B), mRNA [NM_018184]	-0.60741645	0.0052900035
A_23_P17012	*LOC648044*	PREDICTED: Homo sapiens guanine nucleotide binding protein (G protein), gamma 12-like (LOC648044), mRNA [XM_003959952]	10009433	0.010503048
A_23_P213832	*SNORD11B*	Homo sapiens small nucleolar RNA, C/D box 11B (SNORD11B), small nucleolar RNA [NR_003694]	14306785	0.009480673
A_33_P3404729	*SCRN3*	Homo sapiens secernin 3 (SCRN3), transcript variant 1, mRNA [NM_024583]	-0.4582947	0.015704028
A_23_P203790	*SPINK7*	Homo sapiens serine peptidase inhibitor, Kazal type 7 (putative) (SPINK7), mRNA [NM_032566]	0.9572244	0.013774471
A_33_P3357843	*OS9*	Homo sapiens osteosarcoma amplified 9, endoplasmic reticulum lectin (OS9), transcript variant 1, mRNA [NM_006812]	-0.70924705	0.012609954
A_23_P200143	*SGCB*	Homo sapiens sarcoglycan, beta (43kDa dystrophin-associated glycoprotein) (SGCB), mRNA [NM_000232]	-0.7645194	0.012524939
A_24_P408321	*FSCN1*	Homo sapiens fascin actin-bundling protein 1 (FSCN1), mRNA [NM_003088]	0.9800217	0.0075709717
A_23_P156471	*DCAF8*	Homo sapiens DDB1 and CUL4 associated factor 8 (DCAF8), transcript variant 1, mRNA [NM_015726]	0.21796183	0.014118111
A_33_P3319937	*OSBPL2*	Homo sapiens oxysterol binding protein-like 2 (OSBPL2), transcript variant 2, mRNA [NM_144498]	-0.7191414	0.012438123
A_23_P141779	*CDC5L*	Homo sapiens cell division cycle 5-like (CDC5L), mRNA [NM_001253]	-0.6879606	0.0031941293
A_23_P257538	*CXXC1*	Homo sapiens CXXC finger protein 1 (CXXC1), transcript variant 2, mRNA [NM_014593]	10145247	0.010066772
A_22_P00010891	*CTSC*	Homo sapiens cathepsin C (CTSC), transcript variant 3, mRNA [NM_001114173]	-15079482	0.004225475
A_23_P41765	*RBBP9*	Homo sapiens retinoblastoma binding protein 9 (RBBP9), mRNA [NM_006606]	-0.86499286	0.014849294
A_21_P0005934	*LINC00116*	Homo sapiens long intergenic non-protein coding RNA 116 (LINC00116), long non-coding RNA [NR_027063]	-10749099	0.0059353677
A_23_P419107	*IRF1*	Homo sapiens interferon regulatory factor 1 (IRF1), mRNA [NM_002198]	16452746	0.008471989
A_32_P172141	*lnc-HMBOX1-1*	PREDICTED: Homo sapiens homeobox containing 1 (HMBOX1), transcript variant X6, mRNA [XM_005273639]	-0.9232075	0.011965513

A_23_P53345	*TCP11L2*	Homo sapiens t-complex 11, testis-specific-like 2 (TCP11L2), transcript variant 1, mRNA [NM_152772]	-15137671	0.0075343307
A_23_P45345	*CDON*	Homo sapiens cell adhesion associated, oncogene regulated (CDON), transcript variant 2, mRNA [NM_016952]	-16382698	0.015417507
A_33_P3219720	*ARNTL2*	Homo sapiens aryl hydrocarbon receptor nuclear translocator-like 2 (ARNTL2), transcript variant 1, mRNA [NM_020183]	0.5952522	0.011449041
A_23_P161644	*HTATSF1*	Homo sapiens HIV-1 Tat specific factor 1 (HTATSF1), transcript variant 2, mRNA [NM_014500]	-0.7197361	0.013665716
A_33_P3377209	*ZNF248*	zinc finger protein 248 [Source:HGNC Symbol;Acc:HGNC:13041] [ENST00000485560]	-0.79267263	0.01042595
A_23_P254472	*RBM14*	Homo sapiens RNA binding motif protein 14 (RBM14), transcript variant 1, mRNA [NM_006328]	0.8820406	0.0047990954
A_23_P17811	*ENSA*	Homo sapiens endosulfine alpha (ENSA), transcript variant 8, mRNA [NM_207168]	-0.34785625	0.004034385
A_33_P3296482	*C6orf211*	Homo sapiens chromosome 6 open reading frame 211 (C6orf211), transcript variant 1, mRNA [NM_024573]	-0.55599165	0.0054341624
A_21_P0010535	*SEC14L2*	Homo sapiens SEC14-like 2 (S. cerevisiae) (SEC14L2), transcript variant 1, mRNA [NM_012429]	11352873	0.0073443917
A_33_P3632937	*PTPRK*	Homo sapiens protein tyrosine phosphatase, receptor type, K (PTPRK), transcript variant 4, mRNA [NM_001291982]	13839092	0.016931534
A_33_P3288219	*XLOC_l2_000297*	BROAD Institute lincRNA (XLOC_l2_000297), lincRNA [TCONS_l2_00000385]	-0.77100205	0.012283598
A_33_P3359753	*LOC100131262*	PREDICTED: Homo sapiens uncharacterized LOC100131262 (LOC100131262), misc_RNA [XR_132952]	-0.34716082	0.015868904
A_23_P205713	*CCSAP*	Homo sapiens centriole, cilia and spindle-associated protein (CCSAP), mRNA [NM_145257]	-0.8059003	0.005438366
A_24_P56252	*CFLAR*	Homo sapiens CASP8 and FADD-like apoptosis regulator (CFLAR), transcript variant 1, mRNA [NM_003879]	-0.74150133	0.0059353677
A_33_P3364443	*STXBP6*	Homo sapiens syntaxin binding protein 6 (amisyn) (STXBP6), mRNA [NM_014178]	-24227242	0.0037656627
A_33_P3236310	*PPP1R12A*	Homo sapiens protein phosphatase 1, regulatory subunit 12A (PPP1R12A), transcript variant 1, mRNA [NM_002480]	-10450239	0.0024890031
A_23_P209689	*CRNDE*	colorectal neoplasia differentially expressed (non-protein coding) [Source:HGNC Symbol;Acc:HGNC:37078] [ENST00000560208]	-13279488	0.0024890031

A_23_P119362	*NOBOX*	Homo sapiens NOBOX oogenesis homeobox (NOBOX), mRNA [NM_001080413]	0.79102075	0.014418281
A_22_P00021369	*ROCK2*	Homo sapiens Rho-associated, coiled-coil containing protein kinase 2 (ROCK2), mRNA [NM_004850]	-0.549429	0.009543431
A_24_P392022	*EMP3*	Homo sapiens epithelial membrane protein 3 (EMP3), mRNA [NM_001425]	0.7335791	0.009266543
A_23_P59855	*EEF2KMT*	Homo sapiens eukaryotic elongation factor 2 lysine methyltransferase (EEF2KMT), transcript variant 1, mRNA [NM_201400]	-10150652	0.0042682863
A_22_P00004404	*ZNF322*	Homo sapiens zinc finger protein 322 (ZNF322), transcript variant 1, mRNA [NM_001242797]	-12393951	0.0034534817
A_23_P77145	*ZNF138*	Homo sapiens zinc finger protein 138 (ZNF138), transcript variant 1, mRNA [NM_006524]	-0.5131699	0.012870585
A_23_P400465	*lnc-COMMD2-1*	ALU6_HUMAN (P39193) Alu subfamily SP sequence contamination warning entry, partial (9%) [THC2705040]	13010255	0.009958825
A_33_P3372941	*RAB11A*	Homo sapiens RAB11A, member RAS oncogene family (RAB11A), transcript variant 1, mRNA [NM_004663]	-0.773139	0.003609745
A_32_P395879	*GTF3C6*	Homo sapiens general transcription factor IIIC, polypeptide 6, alpha 35kDa (GTF3C6), mRNA [NM_138408]	-0.46963027	0.010567525
A_23_P48358	*TINF2*	Homo sapiens TERF1 (TRF1)-interacting nuclear factor 2 (TINF2), transcript variant 2, mRNA [NM_012461]	-0.805362	0.013054549
A_33_P3324909	*CCDC171*	Homo sapiens coiled-coil domain containing 171 (CCDC171), mRNA [NM_173550]	-12211848	0.013700735
A_33_P3419691	*PCCA*	Homo sapiens propionyl CoA carboxylase, alpha polypeptide (PCCA), transcript variant 1, mRNA [NM_000282]	-0.99917674	0.0059353677
A_23_P1206	*JUND*	Homo sapiens jun D proto-oncogene (JUND), transcript variant 1, mRNA [NM_001286968]	15220094	0.008217507
A_23_P120435	*GATS*	Homo sapiens GATS, stromal antigen 3 opposite strand (GATS), transcript variant 2, non-coding RNA [NR_028038]	-13210092	0.0071290513
A_24_P72646	*RPS24*	Homo sapiens ribosomal protein S24 (RPS24), transcript variant c, mRNA [NM_001026]	-0.3515348	0.01324755
A_23_P156620	*WFDC3*	Homo sapiens WAP four-disulfide core domain 3 (WFDC3), mRNA [NM_080614]	-0.8372514	0.006111203
A_23_P151970	*BIN3-IT1*	Homo sapiens BIN3 intronic transcript 1 (non-protein coding) (BIN3-IT1), long non-coding RNA [NR_027715]	0.6746761	0.0066133677
A_21_P0014716	*ZNF184*	Homo sapiens zinc finger protein 184 (ZNF184), mRNA [NM_007149]	-0.5213975	0.011204273

A_21_P0000750	*FEM1B*	Homo sapiens fem-1 homolog b (C. elegans) (FEM1B), mRNA [NM_015322]	-155358	0.007324666
A_24_P117138	*MFI2-AS1*	Homo sapiens MFI2 antisense RNA 1 (MFI2-AS1), long non-coding RNA [NR_038285]	-13309579	0.011153647
A_21_P0000350	*NKTR*	Homo sapiens natural killer cell triggering receptor (NKTR), mRNA [NM_005385]	-0.37941167	0.008307647
A_33_P3324651	*ZNF626*	Homo sapiens zinc finger protein 626 (ZNF626), transcript variant 2, mRNA [NM_145297]	-0.27888966	0.01232182
A_23_P144911	*SCARNA20*	Homo sapiens small Cajal body-specific RNA 20 (SCARNA20), guide RNA [NR_002999]	-0.7981229	0.0063605034
A_23_P118254	*RBM39*	Homo sapiens RNA binding motif protein 39 (RBM39), transcript variant 4, mRNA [NM_001242600]	-0.6046851	0.0054868753
A_23_P64560	*EGFLAM*	Homo sapiens EGF-like, fibronectin type III and laminin G domains (EGFLAM), transcript variant 1, mRNA [NM_152403]	5096386	0.0025001846
A_24_P327815	*FOXF1*	Homo sapiens forkhead box F1 (FOXF1), mRNA [NM_001451]	55456605	0.0025001846
A_23_P411851	*PGAP2*	Homo sapiens post-GPI attachment to proteins 2 (PGAP2), transcript variant 1, mRNA [NM_014489]	-0.5270531	0.00955837
A_23_P163458	*STIP1*	Homo sapiens stress-induced phosphoprotein 1 (STIP1), transcript variant 2, mRNA [NM_006819]	13143319	0.0066917622
A_23_P120243	*SYNE3*	Homo sapiens spectrin repeat containing, nuclear envelope family member 3 (SYNE3), mRNA [NM_152592]	-0.64123183	0.0143041955
A_23_P169117	*EHD4*	Homo sapiens EH-domain containing 4 (EHD4), mRNA [NM_139265]	-0.46446896	0.0077072685
A_24_P21410	*HOXD1*	Homo sapiens homeobox D1 (HOXD1), mRNA [NM_024501]	-0.44696918	0.0056601833
A_24_P331904	*RRAGA*	Homo sapiens Ras-related GTP binding A (RRAGA), mRNA [NM_006570]	-0.4184339	0.016362116
A_33_P3308101	*NOL6*	Homo sapiens nucleolar protein 6 (RNA-associated) (NOL6), transcript variant alpha, mRNA [NM_022917]	10311326	0.009938745
A_23_P73012	*COMMD4*	Homo sapiens COMM domain containing 4 (COMMD4), transcript variant 1, mRNA [NM_017828]	-0.578634	0.009898471
A_33_P3234571	*C9orf3*	Homo sapiens chromosome 9 open reading frame 3 (C9orf3), transcript variant 2, mRNA [NM_032823]	-23377647	0.0037353234
A_21_P0010663	*TOLLIP*	Homo sapiens toll interacting protein (TOLLIP), mRNA [NM_019009]	-15736246	0.005595418
A_32_P50066	*MAP2K3*	Homo sapiens mitogen-activated protein kinase kinase 3 (MAP2K3), transcript variant B, mRNA [NM_145109]	11158692	0.0075625386

A_33_P3216714	*XLOC_l2_001206*	BROAD Institute lincRNA (XLOC_l2_001206), lincRNA [TCONS_l2_00001638]	0.81437796	0.0047538285
A_23_P331813	*MAP9*	Homo sapiens microtubule-associated protein 9 (MAP9), mRNA [NM_001039580]	-1151698	0.007571981
A_23_P24365	*DNAJC6*	Homo sapiens DnaJ (Hsp40) homolog, subfamily C, member 6 (DNAJC6), transcript variant 1, mRNA [NM_001256864]	-0.9243544	0.010495637
A_33_P3261610	*ZNF687*	Homo sapiens zinc finger protein 687 (ZNF687), mRNA [NM_020832]	0.81091183	0.004993122
A_23_P134835	*ANKRD49*	Homo sapiens ankyrin repeat domain 49 (ANKRD49), mRNA [NM_017704]	-11521561	0.005581195
A_24_P226116	*POLR3GL*	Homo sapiens polymerase (RNA) III (DNA directed) polypeptide G (32kD)-like (POLR3GL), mRNA [NM_032305]	-0.8983945	0.0046119415
A_23_P117694	*CSGALNACT1*	Homo sapiens chondroitin sulfate N-acetylgalactosaminyltransferase 1 (CSGALNACT1), transcript variant 2, mRNA [NM_018371]	-39350824	0.0063254745
A_23_P22350	*NAA15*	Homo sapiens N(alpha)-acetyltransferase 15, NatA auxiliary subunit (NAA15), mRNA [NM_057175]	0.35984966	0.008819748
A_24_P301557	*CORO2B*	Homo sapiens coronin, actin binding protein, 2B (CORO2B), transcript variant 1, mRNA [NM_006091]	25448947	0.0031750489
A_33_P3238215	*GRAMD3*	Homo sapiens GRAM domain containing 3 (GRAMD3), transcript variant 2, mRNA [NM_023927]	-14474487	0.0052543716
A_23_P37514	*LPIN2*	Homo sapiens lipin 2 (LPIN2), mRNA [NM_014646]	-0.73894376	0.011728736
A_23_P94660	*COBLL1*	Homo sapiens cordon-bleu WH2 repeat protein-like 1 (COBLL1), transcript variant 1, mRNA [NM_001278458]	-0.94433546	0.008893439
A_23_P429082	*C15orf39*	Homo sapiens chromosome 15 open reading frame 39 (C15orf39), mRNA [NM_015492]	-10812571	0.0051268074
A_32_P194779	*TBC1D13*	Homo sapiens TBC1 domain family, member 13 (TBC1D13), transcript variant 1, mRNA [NM_018201]	-0.5508621	0.013631432
A_23_P18684	*SPTY2D1*	Homo sapiens SPT2, Suppressor of Ty, domain containing 1 (S. cerevisiae) (SPTY2D1), mRNA [NM_194285]	-0.52607304	0.0147644505
A_33_P3396607	*ZBTB34*	Homo sapiens zinc finger and BTB domain containing 34 (ZBTB34), mRNA [NM_001099270]	-0.88891256	0.014448771
A_33_P3399318	*CLGN*	Homo sapiens calmegin (CLGN), transcript variant 1, mRNA [NM_004362]	14334674	0.0029015075
A_23_P22548	*UGDH*	Homo sapiens UDP-glucose 6-dehydrogenase (UGDH), transcript variant 1, mRNA [NM_003359]	-12092547	0.0044508153

A_33_P3275846	*GNG12*	Homo sapiens guanine nucleotide binding protein (G protein), gamma 12 (GNG12), mRNA [NM_018841]	-0.984735	0.0072907214
A_32_P142028	*CHM*	Homo sapiens choroideremia (Rab escort protein 1) (CHM), transcript variant 1, mRNA [NM_000390]	-0.55305177	0.008785306
A_23_P166826	*CACNA1A*	Homo sapiens calcium channel, voltage-dependent, P/Q type, alpha 1A subunit (CACNA1A), transcript variant 2, mRNA [NM_023035]	-0.9438875	0.01023743
A_33_P3421490	*HNRNPC*	Homo sapiens heterogeneous nuclear ribonucleoprotein C (C1/C2) (HNRNPC), transcript variant 1, mRNA [NM_031314]	-0.37891078	0.0070802537
A_33_P3268618	*DCP1A*	Homo sapiens decapping mRNA 1A (DCP1A), transcript variant 1, mRNA [NM_018403]	-0.783084	0.0096938675
A_23_P341325	*KIAA1024*	Homo sapiens KIAA1024 (KIAA1024), mRNA [NM_015206]	-11769476	0.0044319443
A_33_P3237235	*WDR19*	Homo sapiens WD repeat domain 19 (WDR19), mRNA [NM_025132]	-12691565	0.0052430327
A_22_P00017960	*RPL10L*	Homo sapiens ribosomal protein L10-like (RPL10L), mRNA [NM_080746]	0.49233818	0.0028913463
A_33_P3284888	*FBXO44*	Homo sapiens F-box protein 44 (FBXO44), transcript variant 4, mRNA [NM_001014765]	0.7750114	0.012283598
A_33_P3350748	*lnc-ZKSCAN1-1*	LNCipedia lincRNA (lnc-ZKSCAN1-1), lincRNA [lnc-ZKSCAN1-1:4]	0.88988954	0.011605179
A_23_P87351	*DLEU7*	Homo sapiens deleted in lymphocytic leukemia, 7 (DLEU7), mRNA [NM_198989]	0.5736423	0.00881269
A_23_P82588	*KRT7*	Homo sapiens keratin 7, type II (KRT7), mRNA [NM_005556]	-0.56274223	0.011672903
A_23_P107283	*RRM1*	Homo sapiens ribonucleotide reductase M1 (RRM1), mRNA [NM_001033]	-0.2930739	0.009503543
A_24_P115774	*C7orf55*	Homo sapiens chromosome 7 open reading frame 55 (C7orf55), transcript variant 1, mRNA [NM_197964]	-0.90319204	0.0056002573
A_33_P3215517	*HOXB2*	Homo sapiens homeobox B2 (HOXB2), mRNA [NM_002145]	-0.62072366	0.008412432
A_24_P346368	*BIRC2*	Homo sapiens baculoviral IAP repeat containing 2 (BIRC2), transcript variant 1, mRNA [NM_001166]	-0.6787901	0.0029691157
A_23_P155857	*RCC2*	Homo sapiens regulator of chromosome condensation 2 (RCC2), transcript variant 1, mRNA [NM_018715]	-0.6397222	0.016331382
A_24_P319736	*TMEM248*	Homo sapiens transmembrane protein 248 (TMEM248), mRNA [NM_017994]	-0.5010021	0.007466854
A_21_P0007398	*NUDT6*	Homo sapiens nudix (nucleoside diphosphate linked moiety X)-type motif 6 (NUDT6), transcript variant 2, mRNA [NM_198041]	-0.4898694	0.014915451

A_23_P66891	*MEIS1*	Homo sapiens Meis homeobox 1 (MEIS1), mRNA [NM_002398]	-12626718	0.00324898
A_23_P424002	*CDC42EP4*	Homo sapiens CDC42 effector protein (Rho GTPase binding) 4 (CDC42EP4), mRNA [NM_012121]	-0.79266244	0.011612434
A_24_P354496	*MAPRE1*	Homo sapiens microtubule-associated protein, RP/EB family, member 1 (MAPRE1), mRNA [NM_012325]	-10290723	0.011486457
A_33_P3350207	*POU2F1*	Homo sapiens POU class 2 homeobox 1 (POU2F1), transcript variant 1, mRNA [NM_002697]	-0.8807633	0.0032224318
A_33_P3369760	*WWC2-AS2*	Homo sapiens WWC2 antisense RNA 2 (WWC2-AS2), long non-coding RNA [NR_024008]	-0.82979333	0.016822075
A_32_P182388	*HCFC1R1*	Homo sapiens host cell factor C1 regulator 1 (XPO1 dependent) (HCFC1R1), transcript variant 1, mRNA [NM_017885]	-0.7892997	0.0073988605
A_33_P3300877	*GLIPR2*	Homo sapiens GLI pathogenesis-related 2 (GLIPR2), transcript variant 3, mRNA [NM_001287011]	-15630362	0.005136951
A_33_P3329607	*ZNF77*	Homo sapiens zinc finger protein 77 (ZNF77), mRNA [NM_021217]	-10818015	0.012215228
A_22_P00015287	*RAB1B*	Homo sapiens RAB1B, member RAS oncogene family (RAB1B), mRNA [NM_030981]	12364306	0.008330631
A_24_P77364	*CNOT2*	Homo sapiens CCR4-NOT transcription complex, subunit 2 (CNOT2), transcript variant 2, mRNA [NM_014515]	-0.7321996	0.01571288
A_33_P3739260	*lnc-SPATA9-1*	BC041337 rho-related BTB domain containing 3 {Homo sapiens} (exp=-1; wgp=0; cg=0), partial (8%) [THC2753743]	-0.3083068	0.009291781
A_33_P3397693	*BOLA3*	Homo sapiens bolA family member 3 (BOLA3), transcript variant 1, mRNA [NM_212552]	-0.65266466	0.008957529
A_33_P3396553	*CAP2*	Homo sapiens CAP, adenylate cyclase-associated protein, 2 (yeast) (CAP2), mRNA [NM_006366]	-17049837	0.008647297
A_23_P16817	*AGPS*	Homo sapiens alkylglycerone phosphate synthase (AGPS), mRNA [NM_003659]	-0.71846104	0.013844987
A_22_P00002012	*CLK1*	Homo sapiens CDC-like kinase 1 (CLK1), transcript variant 1, mRNA [NM_004071]	-0.7528901	0.002804883
A_23_P115922	*NCAPG2*	Homo sapiens non-SMC condensin II complex, subunit G2 (NCAPG2), transcript variant 2, mRNA [NM_001281932]	-0.47526056	0.014717759
A_23_P303671	*lnc-BDKRB1-1*	LNCipedia lincRNA (lnc-BDKRB1-1), lincRNA [lnc-BDKRB1-1:1]	24441662	0.0070324177
A_24_P759477	*EIF4EBP2*	Homo sapiens eukaryotic translation initiation factor 4E binding protein 2 (EIF4EBP2), mRNA [NM_004096]	-0.78348947	0.01649374

A_23_P354798	*ECM2*	Homo sapiens extracellular matrix protein 2, female organ and adipocyte specific (ECM2), transcript variant 1, mRNA [NM_001393]	-1938739	0.0028913463
A_23_P149664	*ITGB8*	Homo sapiens integrin, beta 8 (ITGB8), mRNA [NM_002214]	10686079	0.0144334
A_24_P182122	*COQ10A*	Homo sapiens coenzyme Q10 homolog A (S. cerevisiae) (COQ10A), transcript variant 1, mRNA [NM_144576]	-0.8336189	0.005976681
A_22_P00007202	*TMEM183B*	Homo sapiens transmembrane protein 183B (TMEM183B), mRNA [NM_001079809]	-0.7315927	0.00983551
A_23_P95930	*ND1*	mitochondrially encoded NADH dehydrogenase 1 [Source:HGNC Symbol;Acc:HGNC:7455] [ENST00000361390]	16088432	0.0032554762
A_33_P3358898	*lnc-GPBP1L1-1*	AGENCOURT_37009809 NIH_MGC_280 Homo sapiens cDNA clone IMAGE:7505078 5', mRNA sequence [CV800748]	-0.97108364	0.012539437
A_33_P3521643	*HMGA2*	Homo sapiens high mobility group AT-hook 2 (HMGA2), transcript variant 1, mRNA [NM_003483]	15889678	0.006506821
A_23_P152548	*SSC5D*	Homo sapiens scavenger receptor cysteine rich family, 5 domains (SSC5D), transcript variant 1, mRNA [NM_001144950]	-0.8474767	0.015334945
A_23_P41487	*PPP1R12C*	Homo sapiens protein phosphatase 1, regulatory subunit 12C (PPP1R12C), transcript variant 2, mRNA [NM_001271618]	-0.5436909	0.008220364
A_23_P25735	*SCPEP1*	Homo sapiens serine carboxypeptidase 1 (SCPEP1), mRNA [NM_021626]	-0.7704346	0.004609424
A_22_P00009238	*TBC1D9*	Homo sapiens TBC1 domain family, member 9 (with GRAM domain) (TBC1D9), mRNA [NM_015130]	-0.5650837	0.0059040254
A_21_P0000731	*PSMA6*	Homo sapiens proteasome (prosome, macropain) subunit, alpha type, 6 (PSMA6), transcript variant 1, mRNA [NM_002791]	-0.35392308	0.00477571
A_22_P00013002	*lnc-LRIG2-3*	Homo sapiens cDNA clone IMAGE:4798168. [BC030759]	-10189925	0.0068771048
A_33_P3646051	*LINC00968*	Homo sapiens long intergenic non-protein coding RNA 968 (LINC00968), long non-coding RNA [NR_038236]	-0.76126087	0.005215599
A_23_P399797	*lnc-ZNF345-1*	Homo sapiens cDNA FLJ37005 fis, clone BRACE2009122. [AK094324]	-11296976	0.010971502
A_24_P157926	*SHQ1*	Homo sapiens SHQ1, H/ACA ribonucleoprotein assembly factor (SHQ1), mRNA [NM_018130]	-0.6564897	0.011969343
A_23_P202939	*SMAD5-AS1*	Homo sapiens SMAD5 antisense RNA 1 (SMAD5-AS1), long non-coding RNA [NR_026763]	-11984125	0.01273111
A_24_P228579	*TNFAIP3*	Homo sapiens tumor necrosis factor, alpha-induced protein 3 (TNFAIP3), transcript variant 3, mRNA [NM_006290]	0.7003124	0.0067678746

A_33_P3244872	*APLP2*	Homo sapiens amyloid beta (A4) precursor-like protein 2 (APLP2), transcript variant 1, mRNA [NM_001642]	-0.89440054	0.006533246
A_33_P3335391	*NDUFAF5*	Homo sapiens NADH dehydrogenase (ubiquinone) complex I, assembly factor 5 (NDUFAF5), transcript variant 1, mRNA [NM_024120]	-0.78622127	0.007885695
A_22_P00003232	*CEP350*	Homo sapiens centrosomal protein 350kDa (CEP350), mRNA [NM_014810]	-0.5558061	0.011376762
A_23_P203115	*FAM83G*	Homo sapiens family with sequence similarity 83, member G (FAM83G), mRNA [NM_001039999]	14682302	0.0072358185
A_23_P396353	*LINC00900*	Homo sapiens long intergenic non-protein coding RNA 900 (LINC00900), long non-coding RNA [NR_034148]	11946083	0.0105877705
A_23_P411162	*TMEM25*	Homo sapiens transmembrane protein 25 (TMEM25), transcript variant 1, mRNA [NM_032780]	-0.80498666	0.007932796
A_22_P00000865	*NIN*	Homo sapiens ninein (GSK3B interacting protein) (NIN), transcript variant 2, mRNA [NM_020921]	-0.6531196	0.0046520224
A_33_P3381948	*PER2*	Homo sapiens period circadian clock 2 (PER2), mRNA [NM_022817]	1513335	0.013345465
A_33_P3389658	*LOC101927609*	PREDICTED: Homo sapiens uncharacterized LOC101927609 (LOC101927609), transcript variant X2, ncRNA [XR_428101]	-0.61454123	0.013688232
A_23_P17021	*WTIP*	Homo sapiens Wilms tumor 1 interacting protein (WTIP), mRNA [NM_001080436]	-0.62413627	0.012012772
A_22_P00019588	*PDE4D*	Homo sapiens phosphodiesterase 4D, cAMP-specific (PDE4D), transcript variant 3, mRNA [NM_001165899]	16063015	0.010133606
A_33_P3408244	*SCRN3*	Homo sapiens secernin 3 (SCRN3), transcript variant 1, mRNA [NM_024583]	-0.5994751	0.013623725
A_23_P85180	*ZC3H6*	Homo sapiens zinc finger CCCH-type containing 6 (ZC3H6), mRNA [NM_198581]	-16626866	0.0067226742
A_24_P230916	*SPRNP1*	Homo sapiens shadow of prion protein homolog (zebrafish) pseudogene 1 (SPRNP1), non-coding RNA [NR_033789]	0.936785	0.004508921
A_21_P0003137	*TMEM187*	Homo sapiens transmembrane protein 187 (TMEM187), mRNA [NM_003492]	-1076529	0.0037947425
A_23_P75430	*MIER1*	Homo sapiens mesoderm induction early response 1, transcriptional regulator (MIER1), transcript variant 4, mRNA [NM_001077702]	-0.5585473	0.013357834
A_23_P214222	*MTRNR2L2*	Homo sapiens MT-RNR2-like 2 (MTRNR2L2), mRNA [NM_001190470]	10504516	0.010198214
A_23_P90172	*SMCO4*	Homo sapiens single-pass membrane protein with coiled-coil domains 4 (SMCO4), mRNA [NM_020179]	-10219915	0.008966905

A_23_P10647	*MARCKS*	myristoylated alanine-rich protein kinase C substrate [Source:HGNC Symbol;Acc:HGNC:6759] [ENST00000612661]	-0.7693853	0.014302255
A_23_P35725	*PPP1R15A*	Homo sapiens protein phosphatase 1, regulatory subunit 15A (PPP1R15A), mRNA [NM_014330]	12475387	0.0041868715
DCP_1_0	*CYTL1*	Homo sapiens cytokine-like 1 (CYTL1), mRNA [NM_018659]	-17529881	0.008282745
A_21_P0014028	*ANO3*	Homo sapiens anoctamin 3 (ANO3), mRNA [NM_031418]	-10818889	0.007740612
A_33_P3264846	*PP12719*	Homo sapiens pp12719 mRNA, complete cds. [AF318328]	-18013825	0.006405189
A_21_P0001366	*HOXC9*	Homo sapiens homeobox C9 (HOXC9), mRNA [NM_006897]	-0.96022964	0.008427824
A_24_P270460	*SAMD9L*	sterile alpha motif domain containing 9-like [Source:HGNC Symbol;Acc:HGNC:1349] [ENST00000610760]	-11457728	0.005947757
A_33_P3395758	*lnc-TSHB-2*	LNCipedia lincRNA (lnc-TSHB-2), lincRNA [lnc-TSHB-2:1]	-11425291	0.0086486135
A_24_P40721	*IFI27*	Homo sapiens interferon, alpha-inducible protein 27 (IFI27), transcript variant 2, mRNA [NM_005532]	-0.6643265	0.009172592
A_19_P00321977	*C14orf28*	Homo sapiens chromosome 14 open reading frame 28 (C14orf28), mRNA [NM_001017923]	-19586122	0.0022181747
A_23_P34233	*SPTLC3*	Homo sapiens serine palmitoyltransferase, long chain base subunit 3 (SPTLC3), mRNA [NM_018327]	-15635415	0.015044886
A_21_P0013656	*lnc-LTBP3-2*	Homo sapiens clone FLC0165 mRNA sequence. [AF130094]	2059631	0.0039566266
A_33_P3240115	*QPRT*	Homo sapiens quinolinate phosphoribosyltransferase (QPRT), mRNA [NM_014298]	12311683	0.009097663
A_23_P97283	*XLOC_l2_015213*	BROAD Institute lincRNA (XLOC_l2_015213), lincRNA [TCONS_l2_00029360]	-0.46597087	0.008095165
A_32_P18159	*PAQR6*	Homo sapiens progestin and adipoQ receptor family member VI (PAQR6), transcript variant 1, mRNA [NM_024897]	-0.8308878	0.010782863
A_23_P158053	*PSMG4*	Homo sapiens proteasome (prosome, macropain) assembly chaperone 4 (PSMG4), transcript variant 1, mRNA [NM_001128592]	-0.41326237	0.008159728
A_32_P466514	*LYRM7*	Homo sapiens LYR motif containing 7 (LYRM7), transcript variant 1, mRNA [NM_181705]	-0.79344493	0.0025001846
A_33_P3346331	*C9orf16*	Homo sapiens chromosome 9 open reading frame 16 (C9orf16), mRNA [NM_024112]	10217216	0.008298705
A_23_P259586	*IRF2BPL*	Homo sapiens interferon regulatory factor 2 binding protein-like (IRF2BPL), mRNA [NM_024496]	0.9279214	0.010885659

A_23_P113572	*TTK*	Homo sapiens TTK protein kinase (TTK), transcript variant 1, mRNA [NM_003318]	-0.7471645	0.008273812
A_32_P31771	*DCUN1D4*	Homo sapiens DCN1, defective in cullin neddylation 1, domain containing 4 (DCUN1D4), transcript variant 1, mRNA [NM_001040402]	-0.6311682	0.004851867
A_22_P00002317	*CD19*	Homo sapiens CD19 molecule (CD19), transcript variant 2, mRNA [NM_001770]	17184519	0.00881236
A_24_P342632	*KIAA1715*	Homo sapiens KIAA1715 (KIAA1715), mRNA [NM_030650]	-0.74694705	0.008208044
A_33_P3282556	*lnc-C11orf44-2*	LNCipedia lincRNA (lnc-C11orf44-2), lincRNA [lnc-C11orf44-2:1]	10773398	0.005365543
A_23_P82249	*AK5*	Homo sapiens adenylate kinase 5 (AK5), transcript variant 1, mRNA [NM_174858]	-0.3230069	0.011995826
A_23_P20777	*TMEM204*	Homo sapiens transmembrane protein 204 (TMEM204), transcript variant 1, mRNA [NM_024600]	-0.8723132	0.012334684
A_33_P3535175	*ABCB8*	Homo sapiens ATP-binding cassette, sub-family B (MDR/TAP), member 8 (ABCB8), transcript variant 2, mRNA [NM_007188]	1471396	0.012095497
A_33_P3283231	*RBM18*	Homo sapiens RNA binding motif protein 18 (RBM18), transcript variant 1, mRNA [NM_033117]	-0.69950837	0.013657404
A_23_P111672	*YY2*	Homo sapiens YY2 transcription factor (YY2), mRNA [NM_206923]	-0.38755327	0.011421206
A_21_P0012528	*KIRREL3*	Homo sapiens kin of IRRE like 3 (Drosophila) (KIRREL3), transcript variant 1, mRNA [NM_032531]	-20415645	0.0044600195
A_23_P76109	*TES*	Homo sapiens testis derived transcript (3 LIM domains) (TES), transcript variant 2, mRNA [NM_152829]	-23884883	0.0050712093
A_33_P3251984	*RILPL2*	Rab interacting lysosomal protein-like 2 [Source:HGNC Symbol;Acc:HGNC:28787] [ENST00000280571]	-0.8322167	0.005241681
A_24_P225719	*RBM12B*	Homo sapiens RNA binding motif protein 12B (RBM12B), mRNA [NM_203390]	-0.81962705	0.006573993
A_33_P3310276	*MRGPRF*	Homo sapiens MAS-related GPR, member F (MRGPRF), transcript variant 1, mRNA [NM_001098515]	12387072	0.008873757
A_24_P910297	*MOB4*	Homo sapiens MOB family member 4, phocein (MOB4), transcript variant 1, mRNA [NM_015387]	-0.551039	0.0075457073
A_22_P00014818	*PIWIL2*	PREDICTED: Homo sapiens piwi-like RNA-mediated gene silencing 2 (PIWIL2), transcript variant X2, mRNA [XM_005273551]	23186722	0.0047538285
A_23_P200404	*GOLGA2*	Homo sapiens golgin A2 (GOLGA2), mRNA [NM_004486]	-0.82330054	0.010885659

A_21_P0011620	*LOC101927809*	PREDICTED: Homo sapiens uncharacterized LOC101927809 (LOC101927809), ncRNA [XR_243869]	-11101676	0.007392104
A_23_P254741	*AK2*	Homo sapiens adenylate kinase 2 (AK2), transcript variant 1, mRNA [NM_001625]	-0.53780174	0.008654175
A_32_P203013	*SOD3*	Homo sapiens superoxide dismutase 3, extracellular (SOD3), mRNA [NM_003102]	-15099308	0.0044319443
A_32_P132477	*SLC44A1*	Homo sapiens solute carrier family 44 (choline transporter), member 1 (SLC44A1), transcript variant 1, mRNA [NM_080546]	-0.78622675	0.0058474606
A_33_P3381943	*RPS10P7*	Homo sapiens ribosomal protein S10 pseudogene 7 (RPS10P7), non-coding RNA [NR_026667]	-0.9995925	0.011282458
A_24_P494454	*DMTF1*	Homo sapiens cyclin D binding myb-like transcription factor 1 (DMTF1), transcript variant 4, non-coding RNA [NR_024549]	-11884359	0.013185801
A_23_P79488	*CYP1B1*	Homo sapiens cytochrome P450, family 1, subfamily B, polypeptide 1 (CYP1B1), mRNA [NM_000104]	-23264713	0.0022181747
A_24_P137522	*SPIN3*	Homo sapiens spindlin family, member 3 (SPIN3), transcript variant 1, mRNA [NM_001010862]	-0.8745044	0.008437608
A_23_P68031	*STON1-GTF2A1L*	Homo sapiens STON1-GTF2A1L readthrough (STON1-GTF2A1L), transcript variant 1, mRNA [NM_172311]	-11062891	0.012647403
A_22_P00017401	*USP53*	Homo sapiens ubiquitin specific peptidase 53 (USP53), mRNA [NM_019050]	-1464778	0.0039856653
A_21_P0011938	*STAT4*	Homo sapiens signal transducer and activator of transcription 4 (STAT4), transcript variant 1, mRNA [NM_003151]	-2875701	0.002804883
A_23_P422718	*lnc-VCAN-1*	LNCipedia lincRNA (lnc-VCAN-1), lincRNA [lnc-VCAN-1:1]	16976838	0.00549504
A_21_P0012398	*ANKRD36B*	Homo sapiens ankyrin repeat domain 36B (ANKRD36B), mRNA [NM_025190]	-1121906	0.0057806885
A_33_P3381870	*POLH*	Homo sapiens polymerase (DNA directed), eta (POLH), transcript variant 1, mRNA [NM_006502]	-13561599	0.0044240346
A_33_P3272347	*DMTN*	Homo sapiens dematin actin binding protein (DMTN), transcript variant 1, mRNA [NM_001978]	-21089873	0.0064146123
A_23_P410312	*GULP1*	Homo sapiens GULP, engulfment adaptor PTB domain containing 1 (GULP1), transcript variant 1, mRNA [NM_016315]	13059928	0.0072648157
A_33_P3333826	*lnc-CCDC8-1*	Homo sapiens cDNA FLJ41726 fis, clone HLUNG2014449. [AK123720]	11188071	0.006573993
A_23_P416751	*C12orf76*	Homo sapiens chromosome 12 open reading frame 76 (C12orf76), mRNA [NM_207435]	-13531837	0.0025001846

A_33_P3321342	*UBE2Q2*	Homo sapiens ubiquitin-conjugating enzyme E2Q family member 2 (UBE2Q2), transcript variant 1, mRNA [NM_173469]	-0.7842295	0.009450744
A_33_P3322430	*ZNF610*	Homo sapiens zinc finger protein 610 (ZNF610), transcript variant 3, mRNA [NM_173530]	-0.84724367	0.012173611
A_23_P204751	*INSIG2*	Homo sapiens insulin induced gene 2 (INSIG2), mRNA [NM_016133]	-13120937	0.004225475
A_23_P80773	*ASIC1*	Homo sapiens acid sensing (proton gated) ion channel 1 (ASIC1), transcript variant 1, mRNA [NM_020039]	-17154318	0.0038144474
A_24_P943301	*SLC40A1*	Homo sapiens solute carrier family 40 (iron-regulated transporter), member 1 (SLC40A1), mRNA [NM_014585]	-0.8049799	0.00847175
A_33_P3369190	*SRPRB*	Homo sapiens signal recognition particle receptor, B subunit (SRPRB), mRNA [NM_021203]	-0.5065164	0.01693894
A_33_P3232995	*PEAR1*	Homo sapiens platelet endothelial aggregation receptor 1 (PEAR1), mRNA [NM_001080471]	-44943914	0.00978609
A_23_P109442	*PAM*	Homo sapiens peptidylglycine alpha-amidating monooxygenase (PAM), transcript variant 5, mRNA [NM_001177306]	-10517484	0.0046609533
A_23_P115190	*TDRD1*	Homo sapiens tudor domain containing 1 (TDRD1), mRNA [NM_198795]	-18707099	0.016549561
A_33_P3441060	*HPS4*	Homo sapiens Hermansky-Pudlak syndrome 4 (HPS4), transcript variant 1, mRNA [NM_022081]	-0.6570456	0.006749785
A_23_P99642	*NGF*	Homo sapiens nerve growth factor (beta polypeptide) (NGF), mRNA [NM_002506]	-0.57126427	0.011680649
A_23_P68922	*TMEM242*	Homo sapiens transmembrane protein 242 (TMEM242), mRNA [NM_018452]	-0.9209046	0.005776505
A_23_P20045	*SLC7A7*	Homo sapiens solute carrier family 7 (amino acid transporter light chain, y+L system), member 7 (SLC7A7), transcript variant 3, mRNA [NM_001126106]	0.29322535	0.0052499557
A_21_P0000296	*MICALL1*	Homo sapiens MICAL-like 1 (MICALL1), mRNA [NM_033386]	0.81567526	0.010072436
A_23_P83234	*PEX1*	Homo sapiens peroxisomal biogenesis factor 1 (PEX1), transcript variant 1, mRNA [NM_000466]	-0.8610976	0.0066162148
A_23_P73801	*SNORD63*	Homo sapiens small nucleolar RNA, C/D box 63 (SNORD63), small nucleolar RNA [NR_002913]	0.5179568	0.013616534
A_23_P145501	*ZBTB6*	Homo sapiens zinc finger and BTB domain containing 6 (ZBTB6), mRNA [NM_006626]	-1087236	0.010586884
A_23_P157449	*TCEAL1*	Homo sapiens transcription elongation factor A (SII)-like 1 (TCEAL1), transcript variant 3, mRNA [NM_001006640]	-0.9043236	0.013450395

A_23_P424316	*MED23*	Homo sapiens mediator complex subunit 23 (MED23), transcript variant 1, mRNA [NM_004830]	-0.46446946	0.009582734
A_24_P181944	*POLR2K*	Homo sapiens polymerase (RNA) II (DNA directed) polypeptide K, 7.0kDa (POLR2K), mRNA [NM_005034]	-0.6541748	0.0024890031
A_33_P3290949	*TCF20*	Homo sapiens transcription factor 20 (AR1) (TCF20), transcript variant 1, mRNA [NM_005650]	-0.9580241	0.003365343
A_23_P25638	*PHF20*	Homo sapiens PHD finger protein 20 (PHF20), mRNA [NM_016436]	-0.99342465	0.006155321
A_33_P3691615	*PTCD3*	pentatricopeptide repeat domain 3 [Source:HGNC Symbol;Acc:HGNC:24717] [ENST00000487043]	-0.99562615	0.010310044
A_23_P35564	*RNF219*	Homo sapiens ring finger protein 219 (RNF219), mRNA [NM_024546]	-0.44093174	0.013513609
A_23_P70794	*lnc-RP11-389E17.1.1-1*	Homo sapiens cDNA FLJ37626 fis, clone BRCOC2014748. [AK094945]	0.79531276	0.013152762
A_33_P3333995	*SEC31B*	Homo sapiens SEC31 homolog B (S. cerevisiae) (SEC31B), mRNA [NM_015490]	-0.48764968	0.008976597
A_33_P3417459	*RAB23*	Homo sapiens RAB23, member RAS oncogene family (RAB23), transcript variant 1, mRNA [NM_016277]	-0.8591974	0.010827594
A_22_P00019616	*KIFC3*	kinesin family member C3 [Source:HGNC Symbol;Acc:HGNC:6326] [ENST00000564204]	0.73236156	0.007889792
A_33_P3332081	*SCARNA9L*	Homo sapiens small Cajal body-specific RNA 9-like (SCARNA9L), guide RNA [NR_023358]	0.39670277	0.014728144
A_23_P77286	*lnc-KBTBD5-1*	DA134497 BRALZ2 Homo sapiens cDNA clone BRALZ2007118 5', mRNA sequence [DA134497]	0.7067666	0.014104654
A_23_P152218	*KHDRBS3*	Homo sapiens KH domain containing, RNA binding, signal transduction associated 3 (KHDRBS3), mRNA [NM_006558]	-10929074	0.0057916977
A_33_P3713357	*KATNBL1*	Homo sapiens katanin p80 subunit B-like 1 (KATNBL1), mRNA [NM_024713]	-0.62191904	0.00915229
A_33_P3347452	*E2F4*	Homo sapiens E2F transcription factor 4, p107/p130-binding (E2F4), mRNA [NM_001950]	15124123	0.0039856653
A_23_P208835	*ALCAM*	Homo sapiens activated leukocyte cell adhesion molecule (ALCAM), transcript variant 1, mRNA [NM_001627]	-12670435	0.00626104
A_24_P66125	*RPS6KA2*	Homo sapiens ribosomal protein S6 kinase, 90kDa, polypeptide 2 (RPS6KA2), transcript variant 1, mRNA [NM_021135]	-14716179	0.008106991
A_22_P00004542	*MAP2K2*	Homo sapiens mitogen-activated protein kinase kinase 2 (MAP2K2), mRNA [NM_030662]	0.9519315	0.013332267

A_33_P3363425	*STAG2*	Homo sapiens stromal antigen 2 (STAG2), transcript variant 1, mRNA [NM_001042749]	-11463566	0.013849912
A_23_P259580	*PARD3-AS1*	Homo sapiens PARD3 antisense RNA 1 (PARD3-AS1), long non-coding RNA [NR_108043]	-131816	0.008765474
A_24_P91472	*FRMD3*	Homo sapiens FERM domain containing 3 (FRMD3), transcript variant 1, mRNA [NM_174938]	-0.6229648	0.004299623
A_33_P3245183	*TAPBP*	Homo sapiens TAP binding protein (tapasin) (TAPBP), transcript variant 2, mRNA [NM_172208]	0.928796	0.01574856
A_33_P3351388	*ALKBH7*	Homo sapiens alkB, alkylation repair homolog 7 (E. coli) (ALKBH7), mRNA [NM_032306]	-0.70193505	0.015932376
A_21_P0000356	*HRH1*	Homo sapiens histamine receptor H1 (HRH1), transcript variant 1, mRNA [NM_001098213]	0.4866709	0.009779963
A_23_P418083	*SCARNA11*	Homo sapiens small Cajal body-specific RNA 11 (SCARNA11), guide RNA [NR_003012]	-10319569	0.012536314
A_23_P350234	*POLE*	Homo sapiens polymerase (DNA directed), epsilon, catalytic subunit (POLE), mRNA [NM_006231]	0.5327442	0.009170467
A_32_P202703	*LCA5*	Homo sapiens Leber congenital amaurosis 5 (LCA5), transcript variant 1, mRNA [NM_181714]	-2029771	0.002804883
A_33_P3378509	*UBE2NL*	Homo sapiens ubiquitin-conjugating enzyme E2N-like (gene/pseudogene) (UBE2NL), transcript variant coding, mRNA [NM_001012989]	-0.26550773	0.01559477
A_24_P941038	*LOC389831*	Homo sapiens uncharacterized LOC389831 (LOC389831), mRNA [NM_001242480]	-1602009	0.006352343
A_23_P119196	*USP38*	Homo sapiens ubiquitin specific peptidase 38 (USP38), transcript variant 3, mRNA [NM_001290326]	-0.93368506	0.005348777
A_33_P3329023	*VSTM4*	Homo sapiens V-set and transmembrane domain containing 4 (VSTM4), transcript variant 1, mRNA [NM_001031746]	-11671791	0.011276983
A_23_P19592	*KLF2*	Homo sapiens Kruppel-like factor 2 (KLF2), mRNA [NM_016270]	-13538511	0.00407791
A_32_P99347	*FAM69A*	Homo sapiens family with sequence similarity 69, member A (FAM69A), transcript variant 5, mRNA [NM_001252273]	-11930255	0.016675575
A_22_P00002982	*PGM3*	Homo sapiens phosphoglucomutase 3 (PGM3), transcript variant 2, mRNA [NM_015599]	-0.65457803	0.011910412
A_23_P213431	*FAM225B*	Homo sapiens family with sequence similarity 225, member B (non-protein coding) (FAM225B), long non-coding RNA [NR_024376]	18072993	0.004055852
A_23_P53663	*GFM2*	Homo sapiens G elongation factor, mitochondrial 2 (GFM2), transcript variant 1, mRNA [NM_032380]	-0.6491725	0.0040942165

A_32_P85539	*SNX3*	Homo sapiens sorting nexin 3 (SNX3), transcript variant 1, mRNA [NM_003795]	-0.96604276	0.0041847117
A_33_P3298062	*PAWR*	Homo sapiens PRKC, apoptosis, WT1, regulator (PAWR), mRNA [NM_002583]	-12022408	0.005377644
A_33_P3346866	*HCFC2*	Homo sapiens host cell factor C2 (HCFC2), mRNA [NM_013320]	-11910295	0.00590614
A_23_P24623	*ABCC5*	Homo sapiens ATP-binding cassette, sub-family C (CFTR/MRP), member 5 (ABCC5), transcript variant 2, mRNA [NM_001023587]	-0.6802148	0.01268465
A_33_P3403708	*KANSL2*	Homo sapiens KAT8 regulatory NSL complex subunit 2 (KANSL2), mRNA [NM_017822]	-0.71374583	0.008908852
A_24_P252043	*ELP4*	Homo sapiens elongator acetyltransferase complex subunit 4 (ELP4), transcript variant 1, mRNA [NM_019040]	-0.93032694	0.008208044
A_23_P389525	*PTP4A1*	Homo sapiens protein tyrosine phosphatase type IVA, member 1 (PTP4A1), mRNA [NM_003463]	0.30277207	0.011376762
A_22_P00023241	*SNHG7*	Homo sapiens small nucleolar RNA host gene 7 (non-protein coding) (SNHG7), transcript variant 3, long non-coding RNA [NR_024543]	-13746321	0.006503996
A_33_P3401301	*COMMD1*	Homo sapiens copper metabolism (Murr1) domain containing 1 (COMMD1), mRNA [NM_152516]	-0.88077927	0.009778202
A_21_P0000409	*lnc-SLC24A5-3*	Q4HW52_GIBZE (Q4HW52) Predicted protein, partial (12%) [THC2788552]	16066291	0.0071724257
A_24_P246963	*RPL39*	Homo sapiens ribosomal protein L39 (RPL39), mRNA [NM_001000]	-0.5581785	0.0072648157
A_33_P3264528	*SNORD114-12*	Homo sapiens small nucleolar RNA, C/D box 114-12 (SNORD114-12), small nucleolar RNA [NR_003205]	0.27360767	0.007115449
A_33_P3229256	*HOXA11*	Homo sapiens homeobox A11 (HOXA11), mRNA [NM_005523]	-0.76827973	0.01509341
A_23_P156826	*COX2*	mitochondrially encoded cytochrome c oxidase II [Source:HGNC Symbol;Acc:HGNC:7421] [ENST00000361739]	0.7097053	0.011788845
A_33_P3210875	*NT5C*	Homo sapiens 5', 3'-nucleotidase, cytosolic (NT5C), transcript variant 1, mRNA [NM_014595]	-0.74617887	0.010402669
A_22_P00008521	*ADTRP*	Homo sapiens androgen-dependent TFPI-regulating protein (ADTRP), transcript variant 2, mRNA [NM_032744]	11884549	0.004359316
A_22_P00019271	*ATP7A*	Homo sapiens ATPase, Cu++ transporting, alpha polypeptide (ATP7A), transcript variant 1, mRNA [NM_000052]	-1240234	0.0044240346
A_33_P3341329	*lnc-KCNJ12-1*	LNCipedia lincRNA (lnc-KCNJ12-1), lincRNA [lnc-KCNJ12-1:1]	0.5629523	0.012393208

A_22_P00022471	*LOC100507091*	Homo sapiens uncharacterized LOC100507091 (LOC100507091), long non-coding RNA [NR_038290]	0.85523915	0.0039854827
A_23_P424712	*ZCRB1*	Homo sapiens zinc finger CCHC-type and RNA binding motif 1 (ZCRB1), mRNA [NM_033114]	-0.7325644	0.011118512
A_22_P00023080	*CCDC142*	Homo sapiens coiled-coil domain containing 142 (CCDC142), mRNA [NM_032779]	-0.8116769	0.011087365
A_33_P3245290	*AK2*	Homo sapiens adenylate kinase 2 (AK2), transcript variant 2, mRNA [NM_013411]	-0.724561	0.011282458
A_33_P3349651	*LRIG2*	leucine-rich repeats and immunoglobulin-like domains 2 [Source:HGNC Symbol;Acc:HGNC:20889] [ENST00000361127]	-14866648	0.010971502
A_33_P3230073	*AQP7P1*	Homo sapiens aquaporin 7 pseudogene 1 (AQP7P1), non-coding RNA [NR_002817]	-23748665	0.003365343
A_23_P144656	*SWAP70*	Homo sapiens SWAP switching B-cell complex 70kDa subunit (SWAP70), transcript variant 1, mRNA [NM_015055]	-12884583	0.0030924478
A_33_P3262495	*CDH10*	Homo sapiens cadherin 10, type 2 (T2-cadherin) (CDH10), mRNA [NM_006727]	-41931663	0.005776505
A_23_P214211	*LOC400927-CSNK1E*	Homo sapiens LOC400927-CSNK1E readthrough (LOC400927-CSNK1E), mRNA [NM_001289912]	0.7740605	0.00945292
A_33_P3272117	*ZNF503*	Homo sapiens zinc finger protein 503 (ZNF503), transcript variant 1, mRNA [NM_032772]	-15054481	0.0035939496
A_23_P122906	*RARS2*	Homo sapiens arginyl-tRNA synthetase 2, mitochondrial (RARS2), mRNA [NM_020320]	-0.9657831	0.0074376203
A_23_P102412	*ASPSCR1*	Homo sapiens alveolar soft part sarcoma chromosome region, candidate 1 (ASPSCR1), transcript variant 1, mRNA [NM_024083]	0.7931123	0.015334634
A_21_P0000528	*AUTS2*	Homo sapiens autism susceptibility candidate 2 (AUTS2), transcript variant 1, mRNA [NM_015570]	-15588799	0.0065866495
A_33_P3320748	*PLEKHA3*	Homo sapiens pleckstrin homology domain containing, family A (phosphoinositide binding specific) member 3 (PLEKHA3), mRNA [NM_019091]	-0.63692594	0.008745013
A_23_P162970	*PRR26*	Homo sapiens proline rich 26 (PRR26), transcript variant 1, long non-coding RNA [NR_027152]	-0.29116142	0.0069250474
A_21_P0004024	*FDX1L*	Homo sapiens ferredoxin 1-like (FDX1L), mRNA [NM_001031734]	0.4988229	0.014267268
A_33_P3349145	*IPO4*	Homo sapiens importin 4 (IPO4), transcript variant 1, mRNA [NM_024658]	1111183	0.010871661
A_33_P3307660	*lnc-RASA1-3*	LNCipedia lincRNA (lnc-RASA1-3), lincRNA [lnc-RASA1-3:1]	-11442763	0.0031941293

A_24_P145122	*TTLL1*	Homo sapiens tubulin tyrosine ligase-like family member 1 (TTLL1), transcript variant 1, mRNA [NM_012263]	-20869775	0.0042918096
A_23_P11664	*KLHDC4*	Homo sapiens kelch domain containing 4 (KLHDC4), transcript variant 3, mRNA [NM_001184854]	0.92474014	0.014023312
A_33_P3259507	*NUCKS1*	Homo sapiens nuclear casein kinase and cyclin-dependent kinase substrate 1 (NUCKS1), mRNA [NM_022731]	-0.9547001	0.0144334
A_23_P383132	*SRSF11*	Homo sapiens serine/arginine-rich splicing factor 11 (SRSF11), transcript variant 1, mRNA [NM_004768]	-0.81353307	0.002804883
A_21_P0000100	*FBXO10*	Homo sapiens F-box protein 10 (FBXO10), mRNA [NM_012166]	-12521367	0.015334945
A_33_P3396200	*HIC2*	Homo sapiens hypermethylated in cancer 2 (HIC2), mRNA [NM_015094]	-0.97619367	0.012878467
A_22_P00017386	*MTRNR2L1*	Homo sapiens MT-RNR2-like 1 (MTRNR2L1), mRNA [NM_001190452]	10669771	0.010472552
A_22_P00005821	*ZASP*	Homo sapiens ZO-2 associated speckle protein (ZASP), mRNA [NM_001289933]	0.662365	0.007797447
A_33_P3370284	*lnc-VAMP1-1*	LNCipedia lincRNA (lnc-VAMP1-1), lincRNA [lnc-VAMP1-1:5]	-14412377	0.0069110035
A_22_P00018594	*lnc-EPSTI1-1*	LNCipedia lincRNA (lnc-EPSTI1-1), lincRNA [lnc-EPSTI1-1:1]	-10599723	0.015448156
A_33_P3317253	*EPN1*	Homo sapiens epsin 1 (EPN1), transcript variant 1, mRNA [NM_001130071]	-0.81290674	0.005792148
A_19_P00812349	*PTER*	Homo sapiens phosphotriesterase related (PTER), transcript variant 1, mRNA [NM_001001484]	0.69208354	0.008064267
A_33_P3278911	*RHOQ*	Homo sapiens ras homolog family member Q (RHOQ), mRNA [NM_012249]	-15786768	0.0032522907
A_32_P138004	*ZNF805*	Homo sapiens zinc finger protein 805 (ZNF805), transcript variant 1, mRNA [NM_001023563]	-11237528	0.0076697893
A_23_P215931	*PHF20L1*	PHD finger protein 20-like 1 [Source:HGNC Symbol;Acc:HGNC:24280] [ENST00000486199]	-13610057	0.005496824
A_23_P99917	*FAM45A*	Homo sapiens family with sequence similarity 45, member A (FAM45A), transcript variant 1, mRNA [NM_207009]	-0.6702296	0.0122815035
A_24_P99071	*LEPROTL1*	Homo sapiens leptin receptor overlapping transcript-like 1 (LEPROTL1), transcript variant 1, mRNA [NM_015344]	-0.61209893	0.007501246
A_32_P89310	*WDR73*	Homo sapiens WD repeat domain 73 (WDR73), mRNA [NM_032856]	-0.9226653	0.007286966
A_23_P217428	*IPO5*	Homo sapiens importin 5 (IPO5), mRNA [NM_002271]	-0.7468164	0.0071487525

A_33_P3399181	*PLEKHM3*	Homo sapiens pleckstrin homology domain containing, family M, member 3 (PLEKHM3), mRNA [NM_001080475]	-0.92204046	0.0057921284
A_33_P3315504	*ARHGAP6*	Homo sapiens Rho GTPase activating protein 6 (ARHGAP6), transcript variant 1, mRNA [NM_013427]	-12170229	0.0095815705
A_33_P3301876	*ERI1*	Homo sapiens exoribonuclease 1 (ERI1), mRNA [NM_153332]	-0.5309963	0.0075780195
A_33_P3396527	*WBP5*	Homo sapiens WW domain binding protein 5 (WBP5), transcript variant 1, mRNA [NM_016303]	-0.46187308	0.0037856582
A_23_P132405	*POLR3G*	Homo sapiens polymerase (RNA) III (DNA directed) polypeptide G (32kD) (POLR3G), mRNA [NM_006467]	0.65071934	0.016708879
A_23_P111188	*PNPLA7*	Homo sapiens patatin-like phospholipase domain containing 7 (PNPLA7), transcript variant 1, mRNA [NM_001098537]	-0.5629787	0.015839092
A_21_P0008252	*ACAD9*	Homo sapiens acyl-CoA dehydrogenase family, member 9 (ACAD9), transcript variant 1, mRNA [NM_014049]	-0.73794293	0.009683536
A_21_P0012887	*ZBTB22*	Homo sapiens zinc finger and BTB domain containing 22 (ZBTB22), transcript variant 1, mRNA [NM_005453]	0.46004236	0.010512188
A_23_P34375	*lnc-TSC22D1-1*	LNCipedia lincRNA (lnc-TSC22D1-1), lincRNA [lnc-TSC22D1-1:4]	0.61359364	0.016842965
A_22_P00010315	*TCEA3*	Homo sapiens transcription elongation factor A (SII), 3 (TCEA3), mRNA [NM_003196]	-14465165	0.010468031
A_21_P0012436	*MT1E*	Homo sapiens metallothionein 1E (MT1E), mRNA [NM_175617]	11873157	0.004453573
A_33_P3413541	*lnc-MUC20-4*	Homo sapiens cDNA FLJ36796 fis, clone ADRGL2006817. [AK094115]	0.7621828	0.013128275
A_21_P0013655	*XLOC_l2_010139*	BROAD Institute lincRNA (XLOC_l2_010139), lincRNA [TCONS_l2_00019209]	17710445	0.005255513
A_33_P3358641	*RNF138P1*	Homo sapiens ring finger protein 138, E3 ubiquitin protein ligase pseudogene 1 (RNF138P1), non-coding RNA [NR_001575]	-0.7256269	0.0024890031
A_24_P350200	*XLOC_l2_015213*	BROAD Institute lincRNA (XLOC_l2_015213), lincRNA [TCONS_l2_00029359]	-0.9677673	0.0061524836
A_24_P160466	*LRRFIP1*	Homo sapiens leucine rich repeat (in FLII) interacting protein 1 (LRRFIP1), transcript variant 3, mRNA [NM_001137552]	-12126724	0.011079362
A_22_P00013803	*LOC101929612*	mitochondrially encoded cytochrome c oxidase III [Source:HGNC Symbol;Acc:HGNC:7422] [ENST00000362079]	1088783	0.008204192
A_23_P137173	*GPRIN1*	Homo sapiens G protein regulated inducer of neurite outgrowth 1 (GPRIN1), mRNA [NM_052899]	16436665	0.0055431053

A_24_P30194	*TMSB15A*	Homo sapiens thymosin beta 15a (TMSB15A), mRNA [NM_021992]	-11850317	0.008267581
A_23_P406025	*RNASEH2B*	Homo sapiens ribonuclease H2, subunit B (RNASEH2B), transcript variant 2, mRNA [NM_001142279]	-0.798165	0.008908852
A_33_P3233010	*IFIT5*	Homo sapiens interferon-induced protein with tetratricopeptide repeats 5 (IFIT5), mRNA [NM_012420]	-0.9823718	0.0052314615
A_19_P00322583	*PRUNE2*	Homo sapiens prune homolog 2 (Drosophila) (PRUNE2), mRNA [NM_015225]	-54842024	0.0025739188
A_33_P3287584	*KIR3DL2*	Homo sapiens killer cell immunoglobulin-like receptor, three domains, long cytoplasmic tail, 2 (KIR3DL2), transcript variant 2, mRNA [NM_001242867]	0.33428133	0.014929396
A_22_P00012482	*PPP1R18*	Homo sapiens protein phosphatase 1, regulatory subunit 18 (PPP1R18), transcript variant 1, mRNA [NM_133471]	0.6053078	0.015388233
A_23_P112429	*VEGFB*	Homo sapiens vascular endothelial growth factor B (VEGFB), transcript variant VEGFB-186, mRNA [NM_003377]	-0.5184021	0.015963586
A_33_P3408212	*LOC100506368*	Homo sapiens uncharacterized LOC100506368 (LOC100506368), long non-coding RNA [NR_038905]	0.7804384	0.012999866
A_23_P44264	*RPL35*	Homo sapiens ribosomal protein L35 (RPL35), mRNA [NM_007209]	-0.40247488	0.0056962976
A_23_P74290	*HSP90B1*	Homo sapiens heat shock protein 90kDa beta (Grp94), member 1 (HSP90B1), mRNA [NM_003299]	-0.60626173	0.008242901
A_23_P364478	*EMX2*	Homo sapiens empty spiracles homeobox 2 (EMX2), transcript variant 1, mRNA [NM_004098]	-1795188	0.006262615
A_23_P52207	*GBP5*	Homo sapiens guanylate binding protein 5 (GBP5), transcript variant 1, mRNA [NM_052942]	-22839398	0.0039856653
A_33_P3313595	*FAM175B*	Homo sapiens family with sequence similarity 175, member B (FAM175B), mRNA [NM_032182]	-0.59956795	0.014448771
A_32_P208350	*BAMBI*	Homo sapiens BMP and activin membrane-bound inhibitor (BAMBI), mRNA [NM_012342]	-463375	0.0048015774
A_24_P675386	*CDK11B*	Homo sapiens cyclin-dependent kinase 11B (CDK11B), transcript variant 3, mRNA [NM_033487]	-0.92580503	0.013441296
A_23_P89941	*TDRD9*	Homo sapiens tudor domain containing 9 (TDRD9), mRNA [NM_153046]	22574546	0.00477571
A_32_P409222	*C11orf58*	Homo sapiens chromosome 11 open reading frame 58 (C11orf58), mRNA [NM_014267]	-0.45934942	0.005057235

A_24_P374382	*CDKN2D*	Homo sapiens cyclin-dependent kinase inhibitor 2D (p19, inhibits CDK4) (CDKN2D), transcript variant 1, mRNA [NM_001800]	-10910174	0.0033094941
A_23_P357794	*ZNF628*	Homo sapiens zinc finger protein 628 (ZNF628), mRNA [NM_033113]	1007974	0.012120488
A_23_P46369	*TOP1P2*	Homo sapiens topoisomerase (DNA) I pseudogene 2 (TOP1P2), non-coding RNA [NR_001283]	0.5146388	0.009484502
A_23_P90933	*MTBP*	Homo sapiens MDM2 binding protein (MTBP), mRNA [NM_022045]	-0.5518284	0.015405921
A_24_P124662	*RAB13*	Homo sapiens RAB13, member RAS oncogene family (RAB13), transcript variant 1, mRNA [NM_002870]	-0.39368516	0.014013276
A_23_P211244	*DUSP19*	Homo sapiens dual specificity phosphatase 19 (DUSP19), transcript variant 1, mRNA [NM_080876]	-0.6802169	0.005640775
A_24_P201381	*MAPKAPK5*	Homo sapiens mitogen-activated protein kinase-activated protein kinase 5 (MAPKAPK5), transcript variant 2, mRNA [NM_139078]	-0.448828	0.0064782333
A_21_P0003606	*PRMT2*	Homo sapiens protein arginine methyltransferase 2 (PRMT2), transcript variant 1, mRNA [NM_206962]	-0.8593375	0.01251397
A_23_P35444	*FGFBP3*	Homo sapiens fibroblast growth factor binding protein 3 (FGFBP3), mRNA [NM_152429]	-18746605	0.0029691157
A_23_P19369	*INA*	Homo sapiens internexin neuronal intermediate filament protein, alpha (INA), mRNA [NM_032727]	14165015	0.013078078
A_33_P3353365	*MCEE*	Homo sapiens methylmalonyl CoA epimerase (MCEE), mRNA [NM_032601]	-11040945	0.006573993
A_22_P00003470	*LRRC16A*	Homo sapiens leucine rich repeat containing 16A (LRRC16A), transcript variant 1, mRNA [NM_017640]	-2128367	0.0061139446
A_24_P350759	*SLC17A4*	solute carrier family 17, member 4 [Source:HGNC Symbol;Acc:HGNC:10932] [ENST00000397076]	0.3347336	0.009334625
A_33_P3417745	*lnc-CCDC68-2*	Homo sapiens cDNA FLJ27183 fis, clone SYN02322. [AK130693]	-14226606	0.0046320097
A_24_P6083	*SLC1A2*	Homo sapiens solute carrier family 1 (glial high affinity glutamate transporter), member 2 (SLC1A2), transcript variant 1, mRNA [NM_004171]	12445961	0.006573993
A_22_P00022768	*IQGAP1*	Homo sapiens IQ motif containing GTPase activating protein 1 (IQGAP1), mRNA [NM_003870]	-0.85592866	0.00557203
A_24_P166094	*CHCHD10*	Homo sapiens coiled-coil-helix-coiled-coil-helix domain containing 10 (CHCHD10), transcript variant 2, mRNA [NM_213720]	-0.89684075	0.009731291

A_23_P201279	*ARFIP1*	Homo sapiens ADP-ribosylation factor interacting protein 1 (ARFIP1), transcript variant 1, mRNA [NM_001025595]	-0.85921866	0.005669954
A_24_P37409	*SERPINB2*	Homo sapiens serpin peptidase inhibitor, clade B (ovalbumin), member 2 (SERPINB2), transcript variant 2, mRNA [NM_002575]	-31627724	0.004213048
A_23_P78458	*UBE4B*	Homo sapiens ubiquitination factor E4B (UBE4B), transcript variant 2, mRNA [NM_006048]	-11776457	0.008813848
A_21_P0011477	*DUSP2*	Homo sapiens dual specificity phosphatase 2 (DUSP2), mRNA [NM_004418]	1986141	0.010203489
A_23_P92281	*ZNF350*	Homo sapiens zinc finger protein 350 (ZNF350), mRNA [NM_021632]	-13436987	0.013197923
A_21_P0014479	*BMS1P17*	Homo sapiens BMS1 pseudogene 17 (BMS1P17), non-coding RNA [NR_073460]	-0.76472646	0.01012583
A_23_P315364	*GTPBP8*	Homo sapiens GTP-binding protein 8 (putative) (GTPBP8), transcript variant 1, mRNA [NM_014170]	-0.9081227	0.007854403
A_33_P3862354	*lnc-DNASE1L3-1*	Homo sapiens cDNA clone IMAGE:5272221. [BC041347]	-0.873296	0.01491013
A_33_P3211809	*CXCL2*	Homo sapiens chemokine (C-X-C motif) ligand 2 (CXCL2), mRNA [NM_002089]	31622505	0.0026564114
A_24_P238118	*TRABD*	Homo sapiens TraB domain containing (TRABD), mRNA [NM_025204]	19745044	0.0046609533
A_22_P00001826	*RUNX1*	Homo sapiens runt-related transcription factor 1 (RUNX1), transcript variant 3, mRNA [NM_001122607]	12428335	0.013849912
A_24_P74571	*PP12613*	Homo sapiens uncharacterized LOC100192379 (PP12613), long non-coding RNA [NR_024365]	18347923	0.0038144474
A_33_P3333507	*lnc-ATP6V1G3-2*	LNCipedia lincRNA (lnc-ATP6V1G3-2), lincRNA [lnc-ATP6V1G3-2:1]	-3204483	0.002419416
A_24_P928052	*CBY1*	Homo sapiens chibby homolog 1 (Drosophila) (CBY1), transcript variant 2, mRNA [NM_001002880]	-0.9994376	0.008064267
A_24_P185029	*SENP7*	Homo sapiens SUMO1/sentrin specific peptidase 7 (SENP7), transcript variant 1, mRNA [NM_020654]	-0.7146968	0.015481674
A_23_P104201	*NRP1*	Homo sapiens neuropilin 1 (NRP1), transcript variant 1, mRNA [NM_003873]	-0.68506	0.0083268415
A_32_P8546	*SH2B1*	Homo sapiens SH2B adaptor protein 1 (SH2B1), transcript variant 2, mRNA [NM_015503]	0.67593646	0.012164148
A_24_P405002	*YME1L1*	Homo sapiens YME1-like 1 ATPase (YME1L1), transcript variant 1, mRNA [NM_139312]	-0.5600383	0.008541028
A_24_P264943	*LINC00473*	Homo sapiens long intergenic non-protein coding RNA 473 (LINC00473), transcript variant 1, long non-coding RNA [NR_026860]	3839722	0.0028288076

A_22_P00022246	*PDIK1L*	Homo sapiens PDLIM1 interacting kinase 1 like (PDIK1L), transcript variant 1, mRNA [NM_152835]	-12500402	0.004599146
DCP_20_1	*COMP*	Homo sapiens cartilage oligomeric matrix protein (COMP), mRNA [NM_000095]	0.9334636	0.014345706
A_33_P3296024	*lnc-IRX3-4*	Q5CSV7_CRYPV (Q5CSV7) Predicted secreted protein, signal peptide, partial (5%) [THC2662545]	-21343336	0.003956336
A_23_P163059	*SNAI2*	Homo sapiens snail family zinc finger 2 (SNAI2), mRNA [NM_003068]	-0.9309411	0.007103236
A_22_P00005230	*SALL3*	Homo sapiens spalt-like transcription factor 3 (SALL3), mRNA [NM_171999]	0.5410321	0.011027956
A_23_P90533	*PCNXL4*	Homo sapiens pecanex-like 4 (Drosophila) (PCNXL4), mRNA [NM_022495]	-0.3907902	0.011013887
A_32_P218355	*SNHG23*	small nucleolar RNA host gene 23 (non-protein coding) [Source:HGNC Symbol;Acc:HGNC:50622] [ENST00000427085]	0.93610674	0.010314908
A_23_P76488	*POP4*	Homo sapiens processing of precursor 4, ribonuclease P/MRP subunit (S. cerevisiae) (POP4), transcript variant 1, mRNA [NM_006627]	-0.4596505	0.013216448
A_24_P694760	*C6orf132*	Homo sapiens chromosome 6 open reading frame 132 (C6orf132), mRNA [NM_001164446]	-12870364	0.0072230194
A_33_P3240693	*EMP1*	Homo sapiens epithelial membrane protein 1 (EMP1), mRNA [NM_001423]	-0.9018449	0.0031941293
A_32_P52785	*ERC1*	Homo sapiens ELKS/RAB6-interacting/CAST family member 1 (ERC1), transcript variant epsilon, mRNA [NM_178040]	-0.62257224	0.013214039
A_22_P00013715	*THSD4*	Homo sapiens thrombospondin, type I, domain containing 4 (THSD4), transcript variant 2, mRNA [NM_001286429]	14394507	0.0070571043
A_22_P00023257	*DAAM2*	Homo sapiens dishevelled associated activator of morphogenesis 2 (DAAM2), transcript variant 2, mRNA [NM_015345]	-60008354	0.009210265
A_33_P3423600	*EWSAT1*	long intergenic non-protein coding RNA 277 [Source:HGNC Symbol;Acc:HGNC:26596] [ENST00000559914]	0.55564034	0.016499978
A_23_P40718	*lnc-IFRD2-2*	Homo sapiens cDNA FLJ43511 fis, clone PERIC2003957. [AK125500]	0.4788387	0.008570872
A_33_P3294217	*OXLD1*	Homo sapiens oxidoreductase-like domain containing 1 (OXLD1), mRNA [NM_001039842]	-12485486	0.005644507
A_24_P937405	*PARVB*	Homo sapiens parvin, beta (PARVB), transcript variant 1, mRNA [NM_001003828]	0.73092717	0.011087365
A_24_P333663	*UTF1*	Homo sapiens undifferentiated embryonic cell transcription factor 1 (UTF1), mRNA [NM_003577]	0.6999068	0.00862906
A_24_P181295	*PRSS23*	Homo sapiens protease, serine, 23 (PRSS23), transcript variant 1, mRNA [NM_007173]	-16571732	0.004016376

A_23_P161719	*MAPK6*	Homo sapiens mitogen-activated protein kinase 6 (MAPK6), mRNA [NM_002748]	-0.8124278	0.015349476
A_33_P3415895	*C14orf37*	Homo sapiens chromosome 14 open reading frame 37 (C14orf37), mRNA [NM_001001872]	-14762219	0.006173301
A_33_P3226202	*CWF19L2*	Homo sapiens CWF19-like 2, cell cycle control (S. pombe) (CWF19L2), mRNA [NM_152434]	-0.5688919	0.011118512
A_24_P106357	*NRBF2*	Homo sapiens nuclear receptor binding factor 2 (NRBF2), transcript variant 1, mRNA [NM_030759]	-0.96176577	0.010503048
A_23_P74349	*FAM179B*	Homo sapiens family with sequence similarity 179, member B (FAM179B), mRNA [NM_015091]	-11972857	0.010181901
A_33_P3390773	*WDR36*	Homo sapiens WD repeat domain 36 (WDR36), mRNA [NM_139281]	-0.57680875	0.005842833
A_33_P3304107	*NUF2*	Homo sapiens NUF2, NDC80 kinetochore complex component (NUF2), transcript variant 1, mRNA [NM_145697]	-0.69709873	0.0029242039
A_23_P212696	*TRIM66*	Homo sapiens tripartite motif containing 66 (TRIM66), mRNA [NM_014818]	-0.2887315	0.0115006585
A_24_P941930	*KLHL28*	Homo sapiens kelch-like family member 28 (KLHL28), mRNA [NM_017658]	-1044491	0.0027248112
A_32_P108826	*FSTL1*	Homo sapiens follistatin-like 1 (FSTL1), mRNA [NM_007085]	-0.4656791	0.007103236
A_23_P59787	*EAF1*	Homo sapiens ELL associated factor 1 (EAF1), mRNA [NM_033083]	-0.6834071	0.003609745
A_23_P170587	*ZBTB41*	Homo sapiens zinc finger and BTB domain containing 41 (ZBTB41), mRNA [NM_194314]	-0.7497365	0.0031941293
A_23_P99614	*LUC7L2*	Homo sapiens LUC7-like 2 (S. cerevisiae) (LUC7L2), transcript variant 1, mRNA [NM_016019]	-0.8468318	0.010848315
A_33_P3805090	*SMYD2*	Homo sapiens SET and MYND domain containing 2 (SMYD2), mRNA [NM_020197]	-0.9868028	0.007889792
A_32_P234827	*BTBD6*	Homo sapiens BTB (POZ) domain containing 6 (BTBD6), mRNA [NM_033271]	-0.5537147	0.010328656
A_23_P391725	*FNIP2*	Homo sapiens folliculin interacting protein 2 (FNIP2), mRNA [NM_020840]	-11271895	0.009898471
A_23_P4353	*ARMC1*	Homo sapiens armadillo repeat containing 1 (ARMC1), transcript variant 1, mRNA [NM_018120]	-0.6872737	0.0063823937
A_23_P19352	*DR1*	Homo sapiens down-regulator of transcription 1, TBP-binding (negative cofactor 2) (DR1), mRNA [NM_001938]	-0.64336056	0.011466815
A_24_P48177	*WSB1*	Homo sapiens WD repeat and SOCS box containing 1 (WSB1), transcript variant 1, mRNA [NM_015626]	-0.6204238	0.011182366
A_24_P328231	*CNPY3*	Homo sapiens canopy FGF signaling regulator 3 (CNPY3), mRNA [NM_006586]	0.5215905	0.007078222

A_22_P00018346	*ST3GAL2*	Homo sapiens ST3 beta-galactoside alpha-2,3-sialyltransferase 2 (ST3GAL2), mRNA [NM_006927]	-0.42851135	0.016157847
A_23_P259207	*CPSF3L*	Homo sapiens cleavage and polyadenylation specific factor 3-like (CPSF3L), transcript variant 2, mRNA [NM_017871]	11989416	0.005169007
A_23_P63816	*ZNF213-AS1*	PREDICTED: Homo sapiens uncharacterized LOC100507458 (RP11-473M20.14), transcript variant X2, ncRNA [XR_243341]	0.8841719	0.007711294
A_33_P3247072	*THNSL2*	Homo sapiens threonine synthase-like 2 (S. cerevisiae) (THNSL2), transcript variant 1, mRNA [NM_018271]	11094491	0.013460425
DCP_22_4	*NRBF2*	Homo sapiens nuclear receptor binding factor 2 (NRBF2), transcript variant 1, mRNA [NM_030759]	-11856437	0.0070606796
A_24_P21752	*PCID2*	Homo sapiens PCI domain containing 2 (PCID2), transcript variant 5, mRNA [NM_001258213]	-0.6301557	0.008963034
A_33_P3328837	*EHBP1*	Homo sapiens EH domain binding protein 1 (EHBP1), transcript variant 1, mRNA [NM_015252]	-0.6848872	0.0057489234
A_23_P31765	*TBRG1*	Homo sapiens transforming growth factor beta regulator 1 (TBRG1), transcript variant 1, mRNA [NM_032811]	-0.68709135	0.010658397
A_23_P11461	*PKIA*	Homo sapiens protein kinase (cAMP-dependent, catalytic) inhibitor alpha (PKIA), transcript variant 1, mRNA [NM_006823]	0.7410512	0.015710928
A_24_P241183	*TRIM3*	Homo sapiens tripartite motif containing 3 (TRIM3), transcript variant 1, mRNA [NM_006458]	15576056	0.004220053
A_33_P3318069	*UBE2V1*	Homo sapiens ubiquitin-conjugating enzyme E2 variant 1 (UBE2V1), transcript variant 4, mRNA [NM_001032288]	-0.75083226	0.014728144
A_23_P205489	*CLEC2D*	Homo sapiens C-type lectin domain family 2, member D (CLEC2D), transcript variant 1, mRNA [NM_013269]	-0.6609401	0.010735533
A_33_P3315303	*SLC7A8*	Homo sapiens solute carrier family 7 (amino acid transporter light chain, L system), member 8 (SLC7A8), transcript variant 2, mRNA [NM_182728]	-11851122	0.004270451
A_32_P207243	*CCT4*	Homo sapiens chaperonin containing TCP1, subunit 4 (delta) (CCT4), transcript variant 1, mRNA [NM_006430]	-0.5865953	0.006543285
A_23_P15669	*KRT73*	Homo sapiens keratin 73, type II (KRT73), mRNA [NM_175068]	0.5867949	0.016991401
A_23_P386420	*C17orf85*	Homo sapiens chromosome 17 open reading frame 85 (C17orf85), mRNA [NM_001114118]	-0.874244	0.005937971

A_23_P94159	*TMEM11*	Homo sapiens transmembrane protein 11 (TMEM11), transcript variant 1, mRNA [NM_003876]	-0.4322781	0.01066717
A_23_P30464	*GTF2H3*	Homo sapiens general transcription factor IIH, polypeptide 3, 34kDa (GTF2H3), transcript variant 1, mRNA [NM_001516]	-0.40358186	0.0063605034
A_23_P255153	*FBXO25*	Homo sapiens F-box protein 25 (FBXO25), transcript variant 1, mRNA [NM_183421]	-14389937	0.0029471128
A_23_P327698	*PRR7*	Homo sapiens proline rich 7 (synaptic) (PRR7), transcript variant 1, mRNA [NM_030567]	0.29851347	0.011368536
A_21_P0005840	*RBMX2*	Homo sapiens RNA binding motif protein, X-linked 2 (RBMX2), mRNA [NM_016024]	-0.6413625	0.0049246643
A_23_P129085	*LMBRD2*	Homo sapiens LMBR1 domain containing 2 (LMBRD2), mRNA [NM_001007527]	-0.50639755	0.013475479
A_33_P3224070	*LINC01301*	Homo sapiens long intergenic non-protein coding RNA 1301 (LINC01301), long non-coding RNA [NR_103854]	-0.78201324	0.004055852
A_22_P00000606	*SPESP1*	Homo sapiens sperm equatorial segment protein 1 (SPESP1), mRNA [NM_145658]	20520625	0.0029015075
A_24_P49383	*CSRNP1*	Homo sapiens cysteine-serine-rich nuclear protein 1 (CSRNP1), mRNA [NM_033027]	27729704	0.0066917622
A_32_P208424	*lnc-ACER2-1*	Homo sapiens cDNA: FLJ21245 fis, clone COL01184. [AK024898]	-0.94641083	0.007282378
A_23_P72584	*AAMDC*	Homo sapiens adipogenesis associated, Mth938 domain containing (AAMDC), mRNA [NM_024684]	-0.8739631	0.0090518845
A_23_P356122	*H3F3A*	Homo sapiens H3 histone, family 3A (H3F3A), mRNA [NM_002107]	-0.5470429	0.013161252
A_21_P0007464	*ACBD7*	Homo sapiens acyl-CoA binding domain containing 7 (ACBD7), mRNA [NM_001039844]	3230649	0.003609745
A_24_P72479	*ZNF451*	Homo sapiens zinc finger protein 451 (ZNF451), transcript variant 1, mRNA [NM_001031623]	-0.9278049	0.008357089
A_33_P3245389	*ARPC1A*	Homo sapiens actin related protein 2/3 complex, subunit 1A, 41kDa (ARPC1A), transcript variant 1, mRNA [NM_006409]	-0.8082051	0.009153984
A_33_P3272080	*BMP2K*	Homo sapiens BMP2 inducible kinase (BMP2K), transcript variant 2, mRNA [NM_017593]	-0.59517086	0.014169819
A_22_P00018680	*TMEM260*	Homo sapiens transmembrane protein 260 (TMEM260), mRNA [NM_017799]	-10955138	0.005787274
A_23_P42335	*QRFP*	pyroglutamylated RFamide peptide [Source:HGNC Symbol;Acc:HGNC:29982] [ENST00000623824]	0.9769716	0.0044707763
A_23_P59261	*FANCE*	Homo sapiens Fanconi anemia, complementation group E (FANCE), mRNA [NM_021922]	-0.6234679	0.009696543

A_33_P3218188	*PDK3*	Homo sapiens pyruvate dehydrogenase kinase, isozyme 3 (PDK3), transcript variant 1, mRNA [NM_001142386]	-0.48557347	0.007415985
A_24_P355493	*TPBG*	Homo sapiens trophoblast glycoprotein (TPBG), transcript variant 1, mRNA [NM_006670]	13697277	0.0036234485
A_23_P130974	*SPSB2*	Homo sapiens splA/ryanodine receptor domain and SOCS box containing 2 (SPSB2), transcript variant 1, mRNA [NM_032641]	-0.9761567	0.005701378
A_24_P44596	*LHPP*	Homo sapiens phospholysine phosphohistidine inorganic pyrophosphate phosphatase (LHPP), transcript variant 1, mRNA [NM_022126]	-11294278	0.005512448
A_23_P385034	*KIAA1683*	Homo sapiens KIAA1683 (KIAA1683), transcript variant 2, mRNA [NM_025249]	-13606762	0.012391117
A_33_P3290343	*MON1B*	Homo sapiens MON1 secretory trafficking family member B (MON1B), transcript variant 1, mRNA [NM_014940]	-0.76363015	0.0074795843
A_23_P119344	*E2F3*	Homo sapiens E2F transcription factor 3 (E2F3), transcript variant 1, mRNA [NM_001949]	-0.8326714	0.006637152
A_23_P259098	*CYP1B1*	Homo sapiens cytochrome P450, family 1, subfamily B, polypeptide 1 (CYP1B1), mRNA [NM_000104]	-24091425	0.010851944
A_23_P91001	*TEAD2*	Homo sapiens TEA domain family member 2 (TEAD2), transcript variant 5, mRNA [NM_003598]	0.6966598	0.0133898035
A_23_P132341	*ZSCAN16*	Homo sapiens zinc finger and SCAN domain containing 16 (ZSCAN16), mRNA [NM_025231]	0.65577143	0.012788202
A_33_P3312030	*ASNSD1*	Homo sapiens asparagine synthetase domain containing 1 (ASNSD1), mRNA [NM_019048]	-0.571538	0.007843497
A_33_P3405068	*C22orf46*	Homo sapiens chromosome 22 open reading frame 46 (C22orf46), mRNA [NM_001142964]	0.6521731	0.011328784
A_23_P90659	*NAV1*	Homo sapiens neuron navigator 1 (NAV1), transcript variant 1, mRNA [NM_020443]	-0.4848626	0.0070324177
A_33_P3329344	*TCF7L1*	Homo sapiens transcription factor 7-like 1 (T-cell specific, HMG-box) (TCF7L1), mRNA [NM_031283]	-17374629	0.007392104
A_32_P131050	*LAPTM4A*	Homo sapiens lysosomal protein transmembrane 4 alpha (LAPTM4A), mRNA [NM_014713]	-0.3736811	0.011376762
A_23_P419764	*FASN*	Homo sapiens fatty acid synthase (FASN), mRNA [NM_004104]	10024227	0.012164148
A_23_P381714	*ZNF148*	Homo sapiens zinc finger protein 148 (ZNF148), mRNA [NM_021964]	-0.6234032	0.014497109
A_23_P156049	*CCDC149*	coiled-coil domain containing 149 [Source:HGNC Symbol;Acc:HGNC:25405] [ENST00000324309]	-0.6308184	0.011617846

A_32_P132396	*CA13*	Homo sapiens carbonic anhydrase XIII (CA13), mRNA [NM_198584]	-11392592	0.0051727663
A_24_P323635	*HEXB*	Homo sapiens hexosaminidase B (beta polypeptide) (HEXB), transcript variant 1, mRNA [NM_000521]	-0.98955655	0.002819256
A_23_P43049	*FAM86JP*	Homo sapiens family with sequence similarity 86, member J, pseudogene (FAM86JP), transcript variant 1, non-coding RNA [NR_024251]	10065424	0.015759172
A_22_P00020535	*DCTN6*	Homo sapiens dynactin 6 (DCTN6), mRNA [NM_006571]	-10736916	0.005822303
A_19_P00319476	*lnc-ECEL1-2*	LNCipedia lincRNA (lnc-ECEL1-2), lincRNA [lnc-ECEL1-2:1]	1380502	0.010468031
A_33_P3214310	*lnc-ENPP1-2*	DB576317 RIKEN full-length enriched human cDNA library, hypothalamus Homo sapiens cDNA clone H033093N20 3', mRNA sequence [DB576317]	-24057703	0.0126397945
A_21_P0013894	*lnc-LTBP3-2*	Homo sapiens clone FLC0165 mRNA sequence. [AF130094]	18137234	0.0037583082
A_23_P360079	*FOXP1*	Homo sapiens forkhead box P1 (FOXP1), transcript variant 2, mRNA [NM_001012505]	-14765176	0.0077268425
A_33_P3220663	*XLOC_l2_015938*	BROAD Institute lincRNA (XLOC_l2_015938), lincRNA [TCONS_l2_00030929]	27255807	0.011307727
A_23_P141917	*NCKAP5*	Homo sapiens NCK-associated protein 5 (NCKAP5), transcript variant 1, mRNA [NM_207363]	-0.7045495	0.009938745
A_21_P0012456	*STAC3*	Homo sapiens SH3 and cysteine rich domain 3 (STAC3), transcript variant 1, mRNA [NM_145064]	-0.84709406	0.0037583082
A_23_P304171	*TYK2*	Homo sapiens tyrosine kinase 2 (TYK2), mRNA [NM_003331]	12558634	0.006020368
A_24_P135483	*LOC101927056*	Homo sapiens uncharacterized LOC101927056 (LOC101927056), transcript variant 1, long non-coding RNA [NR_125395]	-28230872	0.011569153
A_24_P4877	*KIAA0226*	Homo sapiens KIAA0226 (KIAA0226), transcript variant 1, mRNA [NM_001145642]	-0.7107129	0.0036240483
A_23_P127652	*TSPAN14*	Homo sapiens tetraspanin 14 (TSPAN14), transcript variant 1, mRNA [NM_030927]	12349617	0.013595847
A_23_P89062	*ZCRB1*	Homo sapiens zinc finger CCHC-type and RNA binding motif 1 (ZCRB1), mRNA [NM_033114]	-0.44791523	0.005734989
A_22_P00002805	*ZNF202*	Homo sapiens zinc finger protein 202 (ZNF202), transcript variant 1, mRNA [NM_003455]	-0.39626545	0.0063667204
A_33_P3375145	*CLUHP3*	Homo sapiens clustered mitochondria (cluA/CLU1) homolog pseudogene 3 (CLUHP3), long non-coding RNA [NR_024034]	11912409	0.013496047
A_22_P00002061	*PCBP1-AS1*	Homo sapiens PCBP1 antisense RNA 1 (PCBP1-AS1), long non-coding RNA [NR_033872]	0.7307739	0.007312003

A_32_P11894	*LURAP1L*	Homo sapiens leucine rich adaptor protein 1-like (LURAP1L), mRNA [NM_203403]	-20496309	0.01307041
A_23_P77048	*lnc-BLID-1*	LNCipedia lincRNA (lnc-BLID-1), lincRNA [lnc-BLID-1:8]	-20568795	0.01221547
A_21_P0010032	*C12orf65*	Homo sapiens chromosome 12 open reading frame 65 (C12orf65), transcript variant 1, mRNA [NM_152269]	-15280962	0.0034400926
A_32_P62571	*SLC25A29*	Homo sapiens solute carrier family 25 (mitochondrial carnitine/acylcarnitine carrier), member 29 (SLC25A29), transcript variant 1, mRNA [NM_001039355]	-0.76959777	0.008208044
A_33_P3269824	*RBM8A*	Homo sapiens RNA binding motif protein 8A (RBM8A), mRNA [NM_005105]	-0.5392425	0.012864231
A_21_P0014173	*ALDH1L2*	Homo sapiens aldehyde dehydrogenase 1 family, member L2 (ALDH1L2), transcript variant 1, mRNA [NM_001034173]	-0.7579691	0.0108927535
A_19_P00808586	*LRRC66*	Homo sapiens leucine rich repeat containing 66 (LRRC66), mRNA [NM_001024611]	-1818794	0.0032831472
A_22_P00020726	*LOC102724889*	PREDICTED: Homo sapiens uncharacterized LOC102724889 (LOC102724889), ncRNA [XR_425995]	-0.5462997	0.015050633
A_23_P160992	*LINC01085*	Homo sapiens long intergenic non-protein coding RNA 1085 (LINC01085), long non-coding RNA [NR_033931]	-36822042	0.003639613
A_22_P00004717	*lnc-ZNF8-1*	ALU8_HUMAN (P39195) Alu subfamily SX sequence contamination warning entry, partial (8%) [THC2532393]	-0.41539544	0.016401727
A_23_P204269	*FMO4*	Homo sapiens flavin containing monooxygenase 4 (FMO4), mRNA [NM_002022]	-1786535	0.0055818916
A_24_P328969	*lnc-CTDSPL2-2*	UI-H-DF0-bet-o-06-0-UI.s1 NCI_CGAP_DF0 Homo sapiens cDNA clone UI-H-DF0-bet-o-06-0-UI 3', mRNA sequence [BU617929]	-14787	0.0065142275
A_24_P233786	*USP15*	Homo sapiens ubiquitin specific peptidase 15 (USP15), transcript variant 2, mRNA [NM_006313]	-0.78269184	0.01658581
A_19_P00324470	*YIPF5*	Homo sapiens Yip1 domain family, member 5 (YIPF5), transcript variant 2, mRNA [NM_030799]	-10255064	0.004236098
A_22_P00017310	*FAM129A*	Homo sapiens family with sequence similarity 129, member A (FAM129A), mRNA [NM_052966]	-0.5966296	0.012243193
A_23_P418015	*MIR143HG*	Homo sapiens MIR143 host gene (non-protein coding) (MIR143HG), transcript variant 1, long non-coding RNA [NR_105059]	-51087885	0.0029471128
A_21_P0007153	*MIR99AHG*	Homo sapiens mir-99a-let-7c cluster host gene (non-protein coding) (MIR99AHG), transcript variant 2, long non-coding RNA [NR_027791]	-0.74704695	0.004880698

A_22_P00022911	*MAPRE2*	Homo sapiens microtubule-associated protein, RP/EB family, member 2 (MAPRE2), transcript variant 1, mRNA [NM_014268]	-0.7572546	0.011376762
A_33_P3214298	*LOC143666*	Homo sapiens uncharacterized LOC143666 (LOC143666), long non-coding RNA [NR_026967]	-0.44976616	0.010942655
A_21_P0010595	*lnc-GATAD1-2*	LNCipedia lincRNA (lnc-GATAD1-2), lincRNA [lnc-GATAD1-2:1]	-0.7342432	0.010773812
A_33_P3292332	*IMPDH2*	Homo sapiens IMP (inosine 5'-monophosphate) dehydrogenase 2 (IMPDH2), mRNA [NM_000884]	-0.8843241	0.010683997
A_33_P3238815	*FBXO25*	Homo sapiens F-box protein 25 (FBXO25), transcript variant 2, mRNA [NM_183420]	-13296366	0.0076046344
A_21_P0004902	*KIF5B*	Homo sapiens kinesin family member 5B (KIF5B), mRNA [NM_004521]	-0.71157384	0.0025001846
A_24_P75072	*FRMD5*	Homo sapiens FERM domain containing 5 (FRMD5), transcript variant 4, mRNA [NM_001286491]	0.5491582	0.015719123
A_23_P371613	*SMUG1*	Homo sapiens single-strand-selective monofunctional uracil-DNA glycosylase 1 (SMUG1), transcript variant 1, mRNA [NM_014311]	-0.5338132	0.014332605
A_24_P4334	*NSUN3*	Homo sapiens NOP2/Sun domain family, member 3 (NSUN3), mRNA [NM_022072]	-0.7677887	0.005339123
A_19_P00321388	*CHCHD1*	Homo sapiens coiled-coil-helix-coiled-coil-helix domain containing 1 (CHCHD1), mRNA [NM_203298]	-0.6154709	0.009074582
A_23_P111000	*RNF38*	Homo sapiens ring finger protein 38 (RNF38), transcript variant 2, mRNA [NM_194328]	-0.6197654	0.010370596
A_22_P00023023	*PSMB9*	Homo sapiens proteasome (prosome, macropain) subunit, beta type, 9 (PSMB9), mRNA [NM_002800]	-0.84235424	0.007211793
A_33_P3232173	*RPGRIP1L*	Homo sapiens RPGRIP1-like (RPGRIP1L), transcript variant 1, mRNA [NM_015272]	-1092446	0.014341361
A_24_P653603	*FLJ32255*	Homo sapiens uncharacterized LOC643977 (FLJ32255), long non-coding RNA [NR_104643]	0.80325806	0.0166108
A_23_P127533	*PSPC1*	paraspeckle component 1 [Source:HGNC Symbol;Acc:HGNC:20320] [ENST00000338910]	-11460232	0.006474282
A_22_P00021561	*C17orf89*	Homo sapiens chromosome 17 open reading frame 89 (C17orf89), mRNA [NM_001086521]	-0.5463465	0.007024488
A_23_P63896	*DCUN1D5*	Homo sapiens DCN1, defective in cullin neddylation 1, domain containing 5 (DCUN1D5), mRNA [NM_032299]	-0.6169011	0.01047417
A_23_P394166	*FAS*	Homo sapiens Fas cell surface death receptor (FAS), transcript variant 1, mRNA [NM_000043]	-1359839	0.006090102
A_23_P38154	*NOX4*	Homo sapiens NADPH oxidase 4 (NOX4), transcript variant 1, mRNA [NM_016931]	-0.5611007	0.016869314

A_21_P0012457	*CNOT7*	Homo sapiens CCR4-NOT transcription complex, subunit 7 (CNOT7), transcript variant 1, mRNA [NM_013354]	-0.41058657	0.015884457
A_23_P129556	*FDXR*	Homo sapiens ferredoxin reductase (FDXR), transcript variant 2, mRNA [NM_004110]	-13869061	0.0037685214
A_23_P62741	*XLOC_l2_010330*	BROAD Institute lincRNA (XLOC_l2_010330), lincRNA [TCONS_l2_00019490]	-1069377	0.0044240346
A_33_P3330503	*IL4R*	Homo sapiens interleukin 4 receptor (IL4R), transcript variant 1, mRNA [NM_000418]	0.39471245	0.010560428
A_23_P217384	*ELTD1*	Homo sapiens EGF, latrophilin and seven transmembrane domain containing 1 (ELTD1), mRNA [NM_022159]	19946898	0.003363001
A_21_P0014256	*ALDH7A1*	Homo sapiens aldehyde dehydrogenase 7 family, member A1 (ALDH7A1), transcript variant 1, mRNA [NM_001182]	-15268929	9,05E+03
A_23_P435610	*AP1S2*	Homo sapiens adaptor-related protein complex 1, sigma 2 subunit (AP1S2), transcript variant 2, mRNA [NM_003916]	-0.28406185	0.009765222
A_24_P43681	*STK32C*	serine/threonine kinase 32C [Source:HGNC Symbol;Acc:HGNC:21332] [ENST00000456004]	-0.77856535	0.00916624
A_23_P53267	*EMC1*	Homo sapiens ER membrane protein complex subunit 1 (EMC1), transcript variant 1, mRNA [NM_015047]	-0.63842297	0.01559477
A_21_P0014503	*DBNL*	Homo sapiens drebrin-like (DBNL), transcript variant 1, mRNA [NM_014063]	0.631449	0.010447919
A_21_P0007230	*RSRC2*	Homo sapiens arginine/serine-rich coiled-coil 2 (RSRC2), transcript variant 1, mRNA [NM_023012]	-0.5444097	0.01609013
A_33_P3380056	*INTS6-AS1*	Homo sapiens INTS6 antisense RNA 1 (INTS6-AS1), long non-coding RNA [NR_103812]	-0.9650544	0.0031827379
A_33_P3394599	*lnc-CCND1-1*	LNCipedia lincRNA (lnc-CCND1-1), lincRNA [lnc-CCND1-1:1]	0.44783452	0.016875746
A_22_P00015296	*MSANTD3*	Homo sapiens Myb/SANT-like DNA-binding domain containing 3 (MSANTD3), transcript variant 4, mRNA [NM_001198807]	1058009	0.005651698
A_33_P3371663	*HMG20B*	Homo sapiens high mobility group 20B (HMG20B), mRNA [NM_006339]	0.70003676	0.012288998
A_33_P3292679	*EIF3J-AS1*	Homo sapiens EIF3J antisense RNA 1 (head to head) (EIF3J-AS1), transcript variant 1, long non-coding RNA [NR_034170]	-0.8689363	0.012416433
A_23_P142950	*LTK*	Homo sapiens leukocyte receptor tyrosine kinase (LTK), transcript variant 1, mRNA [NM_002344]	10977007	0.011021474
A_24_P261383	*SNX6*	Homo sapiens sorting nexin 6 (SNX6), transcript variant 1, mRNA [NM_021249]	-13123431	0.0049456456
A_33_P3335966	*GIGYF2*	Homo sapiens GRB10 interacting GYF protein 2 (GIGYF2), transcript variant 2, mRNA [NM_015575]	-0.6666818	0.003269423

A_23_P89410	*TAF1D*	Homo sapiens TATA box binding protein (TBP)-associated factor, RNA polymerase I, D, 41kDa (TAF1D), mRNA [NM_024116]	-10869243	0.002804883
A_23_P60002	*TPM1*	Homo sapiens tropomyosin 1 (alpha) (TPM1), transcript variant Tpm1.1, mRNA [NM_001018005]	-15176548	0.003162355
A_21_P0007249	*BECN1*	Homo sapiens beclin 1, autophagy related (BECN1), mRNA [NM_003766]	-0.70824045	0.006664567
A_23_P69437	*EMC2*	Homo sapiens ER membrane protein complex subunit 2 (EMC2), mRNA [NM_014673]	-0.6422226	0.014758866
A_23_P40108	*RAB30-AS1*	RAB30 antisense RNA 1 (head to head) [Source:HGNC Symbol;Acc:HGNC:48672] [ENST00000530270]	-13128911	0.009240182
A_33_P3429575	*YEATS2*	Homo sapiens YEATS domain containing 2 (YEATS2), mRNA [NM_018023]	-11338587	0.0048260237
A_23_P60458	*COL9A3*	Homo sapiens collagen, type IX, alpha 3 (COL9A3), mRNA [NM_001853]	4457892	0.0032831472
A_33_P6461662	*LOC643454*	Homo sapiens adaptor-related protein complex 3, sigma 1 subunit pseudogene, mRNA (cDNA clone IMAGE:8862705). [BC157883]	-0.95110244	0.0027248112
A_23_P111487	*PPP2R4*	Homo sapiens protein phosphatase 2A activator, regulatory subunit 4 (PPP2R4), transcript variant 1, mRNA [NM_178001]	10871606	0.0088250805
A_23_P420942	*VPS11*	Homo sapiens vacuolar protein sorting 11 homolog (S. cerevisiae) (VPS11), transcript variant 2, mRNA [NM_001290185]	-0.9937601	0.006543285
A_32_P53524	*SRRT*	Homo sapiens serrate, RNA effector molecule (SRRT), transcript variant 4, mRNA [NM_001128853]	0.5477686	0.015931543
A_23_P114883	*MT1E*	PREDICTED: Homo sapiens metallothionein 1E (MT1E), transcript variant X1, mRNA [XM_005255956]	10383507	0.0130611155
A_22_P00020247	*NTN1*	Homo sapiens netrin 1 (NTN1), mRNA [NM_004822]	-0.7058948	0.014332605
A_23_P65278	*FMOD*	Homo sapiens fibromodulin (FMOD), transcript variant 1, mRNA [NM_002023]	-27204497	0.0051764864
A_33_P3290909	*LMO7-AS1*	Homo sapiens LMO7 antisense RNA 1 (LMO7-AS1), transcript variant 1, long non-coding RNA [NR_120410]	-19603176	0.0047855447
A_23_P31921	*NBEA*	Homo sapiens neurobeachin (NBEA), transcript variant 1, mRNA [NM_015678]	-16742897	0.01172656
A_23_P52266	*SMC1A*	Homo sapiens structural maintenance of chromosomes 1A (SMC1A), transcript variant 2, mRNA [NM_001281463]	-0.47753593	0.011628436
A_23_P218637	*ASS1*	Homo sapiens argininosuccinate synthase 1 (ASS1), transcript variant 1, mRNA [NM_000050]	14210525	0.007870282

A_22_P00004521	*IFIT1*	Homo sapiens interferon-induced protein with tetratricopeptide repeats 1 (IFIT1), transcript variant 1, mRNA [NM_001548]	-1596417	0.0050769406
A_21_P0014306	*RGPD5*	Homo sapiens RANBP2-like and GRIP domain containing 5 (RGPD5), transcript variant 1, mRNA [NM_005054]	-0.7025423	0.01047417
A_23_P145388	*lnc-CR392000.1-2*	LNCipedia lincRNA (lnc-CR392000.1-2), lincRNA [lnc-CR392000.1-2:1]	13326191	0.00943937
A_33_P3235990	*CAPN15*	calpain 15 [Source:HGNC Symbol;Acc:HGNC:11182] [ENST00000567216]	0.8302428	0.008829352
A_23_P161352	*MTCH1*	Homo sapiens mitochondrial carrier 1 (MTCH1), transcript variant 1, mRNA [NM_014341]	-0.6516885	0.0042201984
A_23_P351667	*PTPN21*	Homo sapiens protein tyrosine phosphatase, non-receptor type 21 (PTPN21), mRNA [NM_007039]	-0.738152	0.0063919895
A_23_P154938	*PTPLA*	Homo sapiens protein tyrosine phosphatase-like (proline instead of catalytic arginine), member A (PTPLA), mRNA [NM_014241]	-0.7591727	0.010203489
A_23_P151820	*ADAM23*	Homo sapiens ADAM metallopeptidase domain 23 (ADAM23), mRNA [NM_003812]	-12345796	0.0062795784
A_23_P128215	*HIRA*	Homo sapiens histone cell cycle regulator (HIRA), mRNA [NM_003325]	-1065954	0.005377644
A_21_P0011380	*RIN3*	Homo sapiens Ras and Rab interactor 3 (RIN3), mRNA [NM_024832]	0.8419357	0.0072230194
A_24_P62860	*SOCS2*	Homo sapiens suppressor of cytokine signaling 2 (SOCS2), transcript variant 1, mRNA [NM_003877]	-13896203	0.0041847117
A_23_P120227	*lnc-STOML1-1*	Q4RDL2_TETNG (Q4RDL2) Chromosome undetermined SCAF16222, whole genome shotgun sequence, partial (5%) [THC2552880]	-12130711	0.009690877
A_23_P121082	*STAM2*	Homo sapiens signal transducing adaptor molecule (SH3 domain and ITAM motif) 2 (STAM2), mRNA [NM_005843]	-10173792	0.014629385
A_23_P317184	*LBH*	Homo sapiens limb bud and heart development (LBH), mRNA [NM_030915]	-39049392	0.0046119415
A_32_P524014	*GBE1*	Homo sapiens glucan (1,4-alpha-), branching enzyme 1 (GBE1), mRNA [NM_000158]	-15038345	0.006185927
A_22_P00005193	*LRRFIP2*	Homo sapiens leucine rich repeat (in FLII) interacting protein 2 (LRRFIP2), transcript variant 1, mRNA [NM_006309]	-0.7488923	0.006816371
A_21_P0014345	*UTRN*	Homo sapiens utrophin (UTRN), mRNA [NM_007124]	-14845853	0.003956951
A_24_P252078	*lnc-DKK4-1*	LNCipedia lincRNA (lnc-DKK4-1), lincRNA [lnc-DKK4-1:1]	-0.74598897	0.012994969
A_33_P3256425	*BTN3A2*	Homo sapiens butyrophilin, subfamily 3, member A2 (BTN3A2), transcript variant 1, mRNA [NM_007047]	-0.39443037	0.014260212

A_21_P0014781	*lnc-UQCRFS1-7*	Q982T4_RHILO (Q982T4) Mll8506 protein, partial (12%) [THC2612479]	-13125114	0.0067047477
A_33_P3424803	*BICD1*	Homo sapiens bicaudal D homolog 1 (Drosophila) (BICD1), transcript variant 1, mRNA [NM_001714]	-0.52899486	0.008366524
A_23_P252201	*FAM220A*	Homo sapiens family with sequence similarity 220, member A (FAM220A), mRNA [NM_001037163]	-0.72217965	0.002804883
A_23_P103837	*HLA-C*	Human MHC class I HLA-Cw1 gene, complete cds. [M26429]	-0.21147469	0.009433551
A_23_P324490	*EAF2*	Homo sapiens ELL associated factor 2 (EAF2), mRNA [NM_018456]	-0.344024	0.0120555395
A_23_P28420	*UBAP2L*	Homo sapiens ubiquitin associated protein 2-like (UBAP2L), transcript variant 3, mRNA [NM_001287815]	10592062	0.0055012475
A_23_P128408	*KIAA0355*	Homo sapiens KIAA0355 (KIAA0355), mRNA [NM_014686]	-10609238	0.0070447545
A_33_P3262133	*OLA1*	Homo sapiens Obg-like ATPase 1 (OLA1), transcript variant 1, mRNA [NM_013341]	0.42005798	0.009582734
A_32_P20523	*TRIAP1*	Homo sapiens TP53 regulated inhibitor of apoptosis 1 (TRIAP1), mRNA [NM_016399]	-10341673	0.010593901
A_23_P200507	*GFM1*	G elongation factor, mitochondrial 1 [Source:HGNC Symbol;Acc:HGNC:13780] [ENST00000312756]	0.7523743	0.008066471
A_33_P3415042	*TIGD2*	Homo sapiens tigger transposable element derived 2 (TIGD2), mRNA [NM_145715]	-0.920367	0.006173301
A_23_P40078	*CNIH4*	Homo sapiens cornichon family AMPA receptor auxiliary protein 4 (CNIH4), transcript variant 1, mRNA [NM_014184]	-10166938	0.006573993
A_24_P625382	*ACLY*	Homo sapiens ATP citrate lyase (ACLY), transcript variant 3, mRNA [NM_001303274]	-0.46006325	0.012006794
A_23_P150189	*XPO1*	Homo sapiens exportin 1 (XPO1), mRNA [NM_003400]	-0.55402213	0.00340924
A_23_P257043	*YBX3*	Homo sapiens Y box binding protein 3 (YBX3), transcript variant 1, mRNA [NM_003651]	-12941222	0.016493676
A_33_P3382769	*MRE11A*	Homo sapiens MRE11 meiotic recombination 11 homolog A (S. cerevisiae) (MRE11A), transcript variant 2, mRNA [NM_005590]	-0.72685766	0.0035523935
A_21_P0009265	*GEM*	Homo sapiens GTP binding protein overexpressed in skeletal muscle (GEM), transcript variant 1, mRNA [NM_005261]	11437993	0.0025001846
A_23_P162766	*LOC100507002*	Homo sapiens uncharacterized LOC100507002 (LOC100507002), long non-coding RNA [NR_110801]	0.8829849	0.01430104
A_23_P213678	*WDR3*	Homo sapiens WD repeat domain 3 (WDR3), mRNA [NM_006784]	-0.39596578	0.016837673

A_33_P3245620	*DOCK9*	Homo sapiens dedicator of cytokinesis 9 (DOCK9), transcript variant 1, mRNA [NM_015296]	-0.75925857	0.005652718
A_33_P3888365	*PAM*	Homo sapiens peptidylglycine alpha-amidating monooxygenase (PAM), transcript variant 1, mRNA [NM_000919]	-12909732	0.0043440815
A_33_P3233160	*RSBN1*	Homo sapiens round spermatid basic protein 1 (RSBN1), mRNA [NM_018364]	-10983918	0.007479662
A_22_P00002482	*C10orf53*	Homo sapiens chromosome 10 open reading frame 53 (C10orf53), transcript variant 1, mRNA [NM_182554]	0.8530746	0.009484502
A_23_P64799	*CFAP36*	Homo sapiens cilia and flagella associated protein 36 (CFAP36), transcript variant 1, mRNA [NM_001282761]	-0.8777282	0.0056601833
A_24_P386323	*GDPGP1*	GDP-D-glucose phosphorylase 1 [Source:HGNC Symbol;Acc:HGNC:34360] [ENST00000558017]	-0.6614774	0.014465799
A_32_P170925	*AAAS*	Homo sapiens achalasia, adrenocortical insufficiency, alacrimia (AAAS), transcript variant 1, mRNA [NM_015665]	0.821795	0.010833076
A_33_P3319134	*RABEPK*	Homo sapiens Rab9 effector protein with kelch motifs (RABEPK), transcript variant 1, mRNA [NM_005833]	-0.8683095	0.0044370755
A_23_P784	*TXNRD3*	Homo sapiens thioredoxin reductase 3 (TXNRD3), transcript variant 1, mRNA [NM_052883]	-0.6136917	0.013963695
A_23_P252764	*LOC100506191*	Homo sapiens clone pp9372 unknown mRNA. [AF289610]	-1188214	0.010052235
A_33_P3708413	*LZIC*	Homo sapiens leucine zipper and CTNNBIP1 domain containing (LZIC), mRNA [NM_032368]	-0.7860484	0.006586824
A_21_P0013143	*SMARCA2*	Homo sapiens SWI/SNF related, matrix associated, actin dependent regulator of chromatin, subfamily a, member 2 (SMARCA2), transcript variant 2, mRNA [NM_139045]	-14063513	0.0069511216
A_33_P3416937	*MFAP5*	Homo sapiens microfibrillar associated protein 5 (MFAP5), transcript variant 1, mRNA [NM_003480]	-3432675	0.0042201984
A_23_P159125	*FAM206A*	family with sequence similarity 206, member A [Source:HGNC Symbol;Acc:HGNC:1364] [ENST00000374624]	-14735781	0.0049677542
A_24_P942002	*TMEM141*	Homo sapiens transmembrane protein 141 (TMEM141), mRNA [NM_032928]	-0.9225096	0.009586496
A_24_P766208	*SLC16A5*	Homo sapiens solute carrier family 16 (monocarboxylate transporter), member 5 (SLC16A5), transcript variant 1, mRNA [NM_004695]	-0.9058881	0.004327546
A_23_P397417	*ACAP2*	Homo sapiens ArfGAP with coiled-coil, ankyrin repeat and PH domains 2 (ACAP2), mRNA [NM_012287]	-0.8052931	0.0027874883

A_33_P3389133	*RPL3*	Homo sapiens ribosomal protein L3 (RPL3), transcript variant 1, mRNA [NM_000967]	-0.32657048	0.0066119307
A_23_P22614	*CNEP1R1*	Homo sapiens CTD nuclear envelope phosphatase 1 regulatory subunit 1 (CNEP1R1), transcript variant 1, mRNA [NM_153261]	-0.96452945	0.002804883
A_33_P3452003	*MINPP1*	Homo sapiens multiple inositol-polyphosphate phosphatase 1 (MINPP1), transcript variant 2, mRNA [NM_001178117]	0.3924969	0.009782756
A_21_P0014075	*LOC143286*	Homo sapiens mRNA; cDNA DKFZp586E171 (from clone DKFZp586E171) [AL049428]	10484885	0.015933463
A_32_P10133	*LOC403323*	Homo sapiens uncharacterized LOC403323 (LOC403323), transcript variant 2, long non-coding RNA [NR_122077]	-0.720069	0.010839213
A_32_P149536	*FLG-AS1*	PREDICTED: Homo sapiens FLG antisense RNA 1 (FLG-AS1), misc_RNA [XR_108352]	-12932928	0.010730268
A_23_P72025	*lnc-THNSL1-2*	Homo sapiens cDNA FLJ11983 fis, clone HEMBB1001337. [AK022045]	-26790795	0.0038056036
A_23_P343411	*SUMO2*	Homo sapiens small ubiquitin-like modifier 2 (SUMO2), transcript variant 1, mRNA [NM_006937]	-0.6758299	0.015448156
A_33_P3728979	*SLC25A20*	Homo sapiens solute carrier family 25 (carnitine/acylcarnitine translocase), member 20 (SLC25A20), mRNA [NM_000387]	-0.73515034	0.008908852
A_21_P0014206	*AGRN*	Homo sapiens agrin (AGRN), mRNA [NM_198576]	3182146	0.0035432973
A_22_P00003431	*FAM151B*	Homo sapiens family with sequence similarity 151, member B (FAM151B), mRNA [NM_205548]	-11933662	0.00407791
A_24_P861009	*BBOX1-AS1*	Homo sapiens BBOX1 antisense RNA 1 (BBOX1-AS1), transcript variant 3, long non-coding RNA [NR_125768]	-15020545	0.010402669
A_23_P253350	*ZNF112*	Homo sapiens zinc finger protein 112 (ZNF112), transcript variant 2, mRNA [NM_013380]	-10597352	0.0069639594
A_22_P00015597	*BRWD1*	Homo sapiens bromodomain and WD repeat domain containing 1 (BRWD1), transcript variant 3, mRNA [NM_001007246]	-0.67924166	0.014228801
A_23_P132139	*C8orf4*	Homo sapiens chromosome 8 open reading frame 4 (C8orf4), mRNA [NM_020130]	2340032	0.014394993
A_21_P0004992	*STXBP5*	Homo sapiens syntaxin binding protein 5 (tomosyn) (STXBP5), transcript variant 2, mRNA [NM_001127715]	-0.3890247	0.016128872
A_23_P46170	*C21orf58*	Homo sapiens chromosome 21 open reading frame 58 (C21orf58), transcript variant 2, mRNA [NM_001286462]	0.74309385	0.0134437205
A_23_P9293	*lnc-C6orf146-2*	LNCipedia lincRNA (lnc-C6orf146-2), lincRNA [lnc-C6orf146-2:1]	0.5583274	0.010091486

A_33_P3247392	*MED8*	Homo sapiens mediator complex subunit 8 (MED8), transcript variant 5, mRNA [NM_201542]	-0.7276597	0.0072499304
A_22_P00004919	*TJP2*	Homo sapiens tight junction protein 2 (TJP2), transcript variant 1, mRNA [NM_004817]	0.60648245	0.014939495
A_22_P00008885	*TPTE*	Homo sapiens transmembrane phosphatase with tensin homology (TPTE), transcript variant 1, mRNA [NM_199261]	-18936566	0.008122174
A_23_P214969	*lnc-DARS2-1*	Homo sapiens cDNA: FLJ22193 fis, clone HRC01108. [AK025846]	10315909	0.010860726
A_23_P125771	*CITED2*	Homo sapiens Cbp/p300-interacting transactivator, with Glu/Asp-rich carboxy-terminal domain, 2 (CITED2), transcript variant 1, mRNA [NM_006079]	-20551379	0.0067226742
A_23_P306933	*RAD52*	Homo sapiens RAD52 homolog (S. cerevisiae) (RAD52), transcript variant 1, mRNA [NM_134424]	-0.49674213	0.013110992
A_33_P3290888	*HCFC1*	Homo sapiens host cell factor C1 (HCFC1), mRNA [NM_005334]	0.6240866	0.0093986625
A_33_P3336700	*PPTC7*	Homo sapiens PTC7 protein phosphatase homolog (S. cerevisiae) (PPTC7), mRNA [NM_139283]	-0.65007967	0.0065866495
A_33_P3347040	*CPNE1*	Homo sapiens copine I (CPNE1), transcript variant 3, mRNA [NM_003915]	0.9742641	0.009467851
A_33_P3369766	*SHROOM3*	Homo sapiens shroom family member 3 (SHROOM3), mRNA [NM_020859]	-20755322	0.0028703364
A_24_P19175	*DPP9-AS1*	Homo sapiens DPP9 antisense RNA 1 (DPP9-AS1), mRNA [NM_001242901]	19005517	0.0063397386
A_33_P3231262	*MED4*	Homo sapiens mediator complex subunit 4 (MED4), transcript variant 1, mRNA [NM_014166]	-12132802	0.0054549226
A_21_P0013775	*ZNF358*	Homo sapiens zinc finger protein 358 (ZNF358), mRNA [NM_018083]	1318068	0.008875075
A_24_P160001	*LOC257396*	Homo sapiens uncharacterized LOC257396 (LOC257396), transcript variant 1, long non-coding RNA [NR_034107]	0.50449693	0.00556604
A_32_P114215	*ARMCX4*	armadillo repeat containing, X-linked 4 [Source:HGNC Symbol;Acc:HGNC:28615] [ENST00000431677]	-0.79545605	0.011363052
A_33_P3334108	*FKBP1A*	Homo sapiens FK506 binding protein 1A, 12kDa (FKBP1A), transcript variant 2, mRNA [NM_054014]	10624306	0.00180587
A_24_P150486	*COMMD6*	Homo sapiens COMM domain containing 6 (COMMD6), transcript variant 1, mRNA [NM_203497]	-10994608	0.002804883
A_23_P61426	*ZNF740*	Homo sapiens zinc finger protein 740 (ZNF740), mRNA [NM_001004304]	-0.98850715	0.006474282

A_21_P0010370	*SPTLC2*	Homo sapiens serine palmitoyltransferase, long chain base subunit 2 (SPTLC2), mRNA [NM_004863]	-0.85712075	0.0067145773
A_23_P114903	*MSRA*	Homo sapiens methionine sulfoxide reductase A (MSRA), transcript variant 1, mRNA [NM_012331]	-0.9283669	0.011969343
A_21_P0000063	*HSPA6*	Homo sapiens heat shock 70kDa protein 6 (HSP70B') (HSPA6), mRNA [NM_002155]	0.9515413	0.005652718
A_33_P3240941	*UMOD*	Homo sapiens uromodulin (UMOD), transcript variant 1, mRNA [NM_003361]	0.44541007	0.0038773962
A_33_P3416448	*MDFIC*	Homo sapiens MyoD family inhibitor domain containing (MDFIC), transcript variant 2, mRNA [NM_001166346]	0.89592457	0.0029471128
A_23_P42331	*BACE1*	Homo sapiens beta-site APP-cleaving enzyme 1 (BACE1), transcript variant a, mRNA [NM_012104]	-1113944	0.010396678
A_23_P118	*BTBD7*	Homo sapiens BTB (POZ) domain containing 7 (BTBD7), transcript variant 1, mRNA [NM_001002860]	0.40534276	0.013489481
A_23_P136012	*HMGA1*	Homo sapiens high mobility group AT-hook 1 (HMGA1), transcript variant 3, mRNA [NM_145901]	18240423	0.0036402496
A_23_P339480	*EXTL2*	Homo sapiens exostosin-like glycosyltransferase 2 (EXTL2), transcript variant 1, mRNA [NM_001439]	-0.41487837	0.014329321
A_23_P1014	*FBXO8*	Homo sapiens F-box protein 8 (FBXO8), mRNA [NM_012180]	-11727107	0.0056123743
A_23_P97309	*HAT1*	Homo sapiens histone acetyltransferase 1 (HAT1), transcript variant 1, mRNA [NM_003642]	-0.37944525	0.011796224
A_32_P14744	*LINC00467*	Homo sapiens long intergenic non-protein coding RNA 467 (LINC00467), long non-coding RNA [NR_026761]	-0.9657108	0.009113876
A_21_P0006829	*CASP9*	Homo sapiens caspase 9, apoptosis-related cysteine peptidase (CASP9), transcript variant alpha, mRNA [NM_001229]	-13521575	0.006914849
A_24_P277367	*RPS15A*	Homo sapiens ribosomal protein S15a (RPS15A), transcript variant 2, mRNA [NM_001019]	-0.6190323	0.00995973
A_21_P0010743	*LINC00707*	Homo sapiens long intergenic non-protein coding RNA 707 (LINC00707), long non-coding RNA [NR_038291]	16725628	0.013589013
A_23_P250283	*CXCL5*	Homo sapiens chemokine (C-X-C motif) ligand 5 (CXCL5), mRNA [NM_002994]	3680357	0.008621566
A_33_P3264444	*RAB1A*	Homo sapiens RAB1A, member RAS oncogene family (RAB1A), transcript variant 1, mRNA [NM_004161]	-0.81760687	0.0028703364

A_23_P4572	*GPN3*	Homo sapiens GPN-loop GTPase 3 (GPN3), transcript variant 2, mRNA [NM_001164372]	-0.70095533	0.0074138297
A_23_P42116	*MYL12A*	Homo sapiens myosin, light chain 12A, regulatory, non-sarcomeric (MYL12A), transcript variant 1, mRNA [NM_006471]	-0.96875376	0.0025079036
A_23_P209694	*AMY1C*	Homo sapiens amylase, alpha 1C (salivary) (AMY1C), mRNA [NM_001008219]	-11877345	0.0067755687
A_21_P0014532	*PPT2*	Homo sapiens palmitoyl-protein thioesterase 2 (PPT2), transcript variant 1, mRNA [NM_005155]	-0.8118791	0.015328013
A_32_P43050	*PAPOLG*	Homo sapiens poly(A) polymerase gamma (PAPOLG), mRNA [NM_022894]	-0.9723269	0.010931163
A_24_P268160	*FRG1*	Homo sapiens FSHD region gene 1 (FRG1), mRNA [NM_004477]	-0.8663277	0.0074443575
A_21_P0012979	*MTCL1*	Homo sapiens microtubule crosslinking factor 1 (MTCL1), mRNA [NM_015210]	-1264516	0.010827594
A_23_P361773	*DRAM2*	Homo sapiens DNA-damage regulated autophagy modulator 2 (DRAM2), mRNA [NM_178454]	-12718208	0.005509873
A_23_P168771	*CCND3*	Homo sapiens cyclin D3 (CCND3), transcript variant 2, mRNA [NM_001760]	10147183	0.00946831
A_24_P784765	*BMPER*	Homo sapiens BMP binding endothelial regulator (BMPER), mRNA [NM_133468]	-12038287	0.0065866495
A_23_P77593	*CCDC146*	Homo sapiens coiled-coil domain containing 146 (CCDC146), mRNA [NM_020879]	-11484287	0.008541028
A_23_P214026	*CD59*	Homo sapiens CD59 molecule, complement regulatory protein (CD59), transcript variant 1, mRNA [NM_203330]	-0.71171325	0.003162355
A_33_P3323822	*VAC14*	Homo sapiens Vac14 homolog (S. cerevisiae) (VAC14), mRNA [NM_018052]	18033519	0.0044240346
A_32_P83049	*FBN2*	Homo sapiens fibrillin 2 (FBN2), mRNA [NM_001999]	-34538674	0.0054810056
A_23_P257795	*GATAD2B*	Homo sapiens GATA zinc finger domain containing 2B (GATAD2B), mRNA [NM_020699]	-1609183	0.012795951
A_23_P15466	*EFR3B*	Homo sapiens EFR3 homolog B (S. cerevisiae) (EFR3B), mRNA [NM_014971]	-21463907	0.009704711
A_33_P3272330	*NDUFA2*	Homo sapiens NADH dehydrogenase (ubiquinone) 1 alpha subcomplex, 2, 8kDa (NDUFA2), transcript variant 1, mRNA [NM_002488]	-0.78862643	0.008626249
A_24_P370471	*SCO1*	Homo sapiens SCO1 cytochrome c oxidase assembly protein (SCO1), mRNA [NM_004589]	-0.48213413	0.011749359
A_33_P3259938	*DNMT3A*	Homo sapiens DNA (cytosine-5-)-methyltransferase 3 alpha (DNMT3A), transcript variant 1, mRNA [NM_175629]	-0.49900618	0.011902722
A_32_P76720	*ATG10*	Homo sapiens autophagy related 10 (ATG10), transcript variant 2, mRNA [NM_031482]	-10070987	0.0075928494

A_23_P68949	*lnc-RNF208-1*	Homo sapiens cDNA FLJ46276 fis, clone TESTI4029676. [AK128153]	-0.964665	0.012609954
A_24_P387321	*NT5DC3*	Homo sapiens 5'-nucleotidase domain containing 3 (NT5DC3), mRNA [NM_001031701]	0.54613036	0.00602263
A_24_P402836	*ST13*	Homo sapiens suppression of tumorigenicity 13 (colon carcinoma) (Hsp70 interacting protein) (ST13), transcript variant 1, mRNA [NM_003932]	-0.93792987	0.011969343
A_23_P39386	*ZNF44*	Homo sapiens zinc finger protein 44 (ZNF44), transcript variant 2, mRNA [NM_016264]	-0.5707216	0.003969615
A_33_P3324206	*ZNF141*	Homo sapiens zinc finger protein 141 (ZNF141), mRNA [NM_003441]	0.31600526	0.009862303
A_23_P24926	*HCST*	Homo sapiens hematopoietic cell signal transducer (HCST), transcript variant 1, mRNA [NM_014266]	-0.8863411	0.0147644505
A_23_P400449	*HR*	Homo sapiens hair growth associated (HR), transcript variant 1, mRNA [NM_005144]	-0.81379706	0.015236547
A_33_P3366146	*FNTA*	Homo sapiens farnesyltransferase, CAAX box, alpha (FNTA), transcript variant 1, mRNA [NM_002027]	-0.9232003	0.008703407
A_23_P98282	*VAT1L*	Homo sapiens vesicle amine transport 1-like (VAT1L), mRNA [NM_020927]	40430937	0.0025451183
A_23_P81048	*SRSF5*	Homo sapiens serine/arginine-rich splicing factor 5 (SRSF5), transcript variant 1, mRNA [NM_001039465]	-0.33269194	0.010960915
A_23_P216476	*SPTBN2*	Homo sapiens spectrin, beta, non-erythrocytic 2 (SPTBN2), mRNA [NM_006946]	13677417	0.010848315
A_22_P00012752	*STIM2*	Homo sapiens stromal interaction molecule 2 (STIM2), transcript variant 2, mRNA [NM_020860]	10593839	0.008122174
A_21_P0000637	*ZBTB5*	Homo sapiens zinc finger and BTB domain containing 5 (ZBTB5), mRNA [NM_014872]	-12012668	0.013286371
A_23_P417331	*lnc-RAD1-2*	PREDICTED: Homo sapiens uncharacterized LOC401180 (LOC401180), mRNA [XM_379325]	0.74368095	0.011061571
A_22_P00009438	*TRAF3IP2-AS1*	Homo sapiens TRAF3IP2 antisense RNA 1 (TRAF3IP2-AS1), transcript variant 4, long non-coding RNA [NR_034111]	-0.5794506	0.011025315
A_24_P236445	*RPS6KA3*	Homo sapiens ribosomal protein S6 kinase, 90kDa, polypeptide 3 (RPS6KA3), mRNA [NM_004586]	-0.8030955	0.014241321
A_23_P56759	*SVIL-AS1*	Homo sapiens SVIL antisense RNA 1 (SVIL-AS1), transcript variant 10, long non-coding RNA [NR_110928]	0.66115624	0.015050037
A_23_P255896	*DYNC1LI2*	Homo sapiens dynein, cytoplasmic 1, light intermediate chain 2 (DYNC1LI2), transcript variant 1, mRNA [NM_006141]	-0.843358	0.00690275

A_23_P37598	*KRCC1*	Homo sapiens lysine-rich coiled-coil 1 (KRCC1), mRNA [NM_016618]	-10435548	0.0057247286
A_33_P3218925	*TRABD2A*	Homo sapiens TraB domain containing 2A (TRABD2A), transcript variant 2, mRNA [NM_001080824]	26105576	0.0072983294
A_33_P3260605	*NPTN*	Homo sapiens neuroplastin (NPTN), transcript variant b, mRNA [NM_012428]	-0.6744759	0.007900263
A_33_P3668839	*STEAP2*	Homo sapiens STEAP family member 2, metalloreductase (STEAP2), transcript variant 4, mRNA [NM_001244944]	0.37792817	0.01208643
A_24_P295999	*CTNNAL1*	Homo sapiens catenin (cadherin-associated protein), alpha-like 1 (CTNNAL1), transcript variant 1, mRNA [NM_003798]	-0.7081106	0.0044853417
A_33_P3240263	*LOC644656*	Homo sapiens uncharacterized LOC644656 (LOC644656), long non-coding RNA [NR_036539]	-0.6717643	0.014061069
A_33_P3398922	*CD4*	Homo sapiens CD4 molecule (CD4), transcript variant 1, mRNA [NM_000616]	0.6957464	0.0108927535
A_33_P3346098	*POMP*	Homo sapiens proteasome maturation protein (POMP), mRNA [NM_015932]	-0.7215974	0.01574856
A_23_P253921	*MBD1*	Homo sapiens methyl-CpG binding domain protein 1 (MBD1), transcript variant 11, mRNA [NM_001204142]	-0.7216893	0.0057506533
A_24_P260440	*TAOK2*	Homo sapiens TAO kinase 2 (TAOK2), transcript variant 1, mRNA [NM_016151]	0.94206375	0.01620439
A_23_P207399	*ZKSCAN7*	Homo sapiens zinc finger with KRAB and SCAN domains 7 (ZKSCAN7), transcript variant 1, mRNA [NM_018651]	-10027347	0.007811618
A_33_P3336696	*TNPO1*	Homo sapiens transportin 1 (TNPO1), transcript variant 1, mRNA [NM_002270]	-0.6851258	0.0104775475
A_33_P3363355	*NBR1*	Homo sapiens neighbor of BRCA1 gene 1 (NBR1), transcript variant 1, mRNA [NM_005899]	-0.97397566	0.0043846127
A_23_P102988	*ND4L*	mitochondrially encoded NADH dehydrogenase 4L [Source:HGNC Symbol;Acc:HGNC:7460] [ENST00000361335]	0.9221942	0.004026994
A_33_P3235716	*ICAM4*	Homo sapiens intercellular adhesion molecule 4 (Landsteiner-Wiener blood group) (ICAM4), transcript variant 2, mRNA [NM_022377]	21457944	0.004884975
A_23_P424582	*TXNRD2*	Homo sapiens thioredoxin reductase 2 (TXNRD2), transcript variant 1, mRNA [NM_006440]	0.61084753	0.008947276
A_23_P162087	*SPSB3*	Homo sapiens splA/ryanodine receptor domain and SOCS box containing 3 (SPSB3), mRNA [NM_080861]	-10374022	0.010606769
A_33_P3218797	*EGFL8*	Homo sapiens EGF-like-domain, multiple 8 (EGFL8), transcript variant 1, mRNA [NM_030652]	-10973184	0.011069937

A_22_P00005708	*TMEM9B*	Homo sapiens TMEM9 domain family, member B (TMEM9B), transcript variant 1, mRNA [NM_020644]	-0.94120836	0.0032224318
A_23_P110531	*PPDPF*	pancreatic progenitor cell differentiation and proliferation factor [Source:HGNC Symbol;Acc:HGNC:16142] [ENST00000370177]	0.6119206	0.012237778
A_23_P28538	*lnc-ELMOD3-1*	Homo sapiens cDNA FLJ46789 fis, clone TRACH3029139. [AK128630]	-10437155	0.007479662
A_22_P00020737	*FST*	Homo sapiens follistatin (FST), transcript variant FST344, mRNA [NM_013409]	0.7198107	0.008778114
A_33_P3230723	*MRPS5*	Homo sapiens mitochondrial ribosomal protein S5 (MRPS5), mRNA [NM_031902]	-0.56321293	0.014323062
A_23_P88740	*lnc-C10orf71-1*	17000600021474 GRN_PRENEU Homo sapiens cDNA 5', mRNA sequence [CN291113]	0.6043763	0.016245075
A_33_P3353170	*KIF1B*	Homo sapiens kinesin family member 1B (KIF1B), transcript variant 2, mRNA [NM_183416]	-0.86820877	0.010094895
A_33_P3308105	*CENPN*	Homo sapiens centromere protein N (CENPN), transcript variant 3, mRNA [NM_018455]	-0.59428996	0.011587933
A_33_P3291998	*CLTC*	Homo sapiens clathrin, heavy chain (Hc), mRNA (cDNA clone MGC:57689 IMAGE:6187185), complete cds. [BC051800]	-0.61444724	0.010519677
A_23_P98645	*GGH*	Homo sapiens gamma-glutamyl hydrolase (conjugase, folylpolygammaglutamyl hydrolase) (GGH), mRNA [NM_003878]	-0.46061587	0.013963695
A_33_P3343155	*RALGPS2*	Ral GEF with PH domain and SH3 binding motif 2 [Source:HGNC Symbol;Acc:HGNC:30279] [ENST00000495034]	-1999793	0.004324298
A_21_P0010998	*DCHS1*	Homo sapiens dachsous cadherin-related 1 (DCHS1), mRNA [NM_003737]	10232004	0.0117289275
A_23_P89710	*GNAQ*	Homo sapiens guanine nucleotide binding protein (G protein), q polypeptide (GNAQ), mRNA [NM_002072]	-0.6076784	0.01002859
A_23_P148990	*CEP192*	Homo sapiens centrosomal protein 192kDa (CEP192), mRNA [NM_032142]	-0.63204837	0.009630487
A_23_P422732	*SAMD14*	Homo sapiens sterile alpha motif domain containing 14 (SAMD14), transcript variant 1, mRNA [NM_174920]	17515695	0.010958166
A_22_P00013960	*HMCN1*	Homo sapiens hemicentin 1 (HMCN1), mRNA [NM_031935]	-2217311	0.013439266
A_22_P00011800	*WDR63*	Homo sapiens WD repeat domain 63 (WDR63), transcript variant 1, mRNA [NM_145172]	-30295663	0.005787274
A_22_P00023374	*lnc-RTL1-1*	Homo sapiens cDNA FLJ44703 fis, clone BRACE3016788. [AK126659]	1956466	0.014523874
A_23_P26810	*lnc-PGPEP1L-3*	Homo sapiens cDNA FLJ26811 fis, clone PRS06268. [AK130321]	-0.68683505	0.0067115854
A_23_P29803	*INAFM2*	Homo sapiens InaF-motif containing 2 (INAFM2), mRNA [NM_001301268]	-11628467	0.0067849406

A_21_P0013240	*TP53*	Homo sapiens tumor protein p53 (TP53), transcript variant 1, mRNA [NM_000546]	0.6034748	0.0031941293
A_33_P3245218	*POLR2H*	Homo sapiens polymerase (RNA) II (DNA directed) polypeptide H (POLR2H), transcript variant 2, mRNA [NM_006232]	-0.8442442	0.0051897555
A_24_P724040	*GTF2IRD1*	Homo sapiens GTF2I repeat domain containing 1 (GTF2IRD1), transcript variant 3, mRNA [NM_001199207]	15155686	0.0066283424
A_23_P2129	*TENM2*	Homo sapiens teneurin transmembrane protein 2 (TENM2), mRNA [NM_001122679]	-36164002	0.0025001846
A_24_P288836	*SNRPB2*	Homo sapiens small nuclear ribonucleoprotein polypeptide B (SNRPB2), transcript variant 1, mRNA [NM_003092]	-0.6169335	0.015754001
A_21_P0013885	*TMEM126B*	Homo sapiens transmembrane protein 126B (TMEM126B), transcript variant 1, mRNA [NM_018480]	-0.99118876	0.005313822
A_33_P3388501	*HLA-DPB2*	Homo sapiens major histocompatibility complex, class II, DP beta 2 (pseudogene) (HLA-DPB2), non-coding RNA [NR_001435]	-0.8819431	0.013030782
A_33_P3391290	*CHIT1*	Homo sapiens chitinase 1 (chitotriosidase) (CHIT1), transcript variant 1, mRNA [NM_003465]	-0.44968343	0.00602263
A_33_P3270668	*LINC01122*	Homo sapiens long intergenic non-protein coding RNA 1122 (LINC01122), long non-coding RNA [NR_033873]	0.97268796	0.0039365552
A_23_P93311	*NREP*	Homo sapiens neuronal regeneration related protein (NREP), transcript variant 11, mRNA [NM_001142483]	-17354735	0.0037479755
A_33_P3392325	*FAM131B*	Homo sapiens family with sequence similarity 131, member B (FAM131B), transcript variant a, mRNA [NM_001031690]	-11507332	0.010237655
A_24_P322709	*DDR1*	Homo sapiens discoidin domain receptor tyrosine kinase 1 (DDR1), transcript variant 2, mRNA [NM_013993]	-10954846	0.009938745
A_23_P253046	*CDC16*	Homo sapiens cell division cycle 16 (CDC16), transcript variant 2, mRNA [NM_001078645]	-1087986	0.007479662
A_33_P3401084	*SNTA1*	Homo sapiens syntrophin, alpha 1 (SNTA1), mRNA [NM_003098]	0.77268857	0.013595847
A_23_P399501	*UGP2*	Homo sapiens UDP-glucose pyrophosphorylase 2 (UGP2), transcript variant 1, mRNA [NM_006759]	-0.5789211	0.0064010993
A_23_P50241	*PKM*	Homo sapiens pyruvate kinase, muscle (PKM), transcript variant 2, mRNA [NM_182470]	13312035	0.0037856582
A_23_P11800	*EPC1*	Homo sapiens enhancer of polycomb homolog 1 (Drosophila) (EPC1), transcript variant 1, mRNA [NM_025209]	-0.47522095	0.010494636

A_33_P3449097	*CLPTM1*	Homo sapiens cleft lip and palate associated transmembrane protein 1 (CLPTM1), transcript variant 2, mRNA [NM_001294]	10738463	0.009847393
A_23_P4754	*CAMK2N1*	Homo sapiens calcium/calmodulin-dependent protein kinase II inhibitor 1 (CAMK2N1), mRNA [NM_018584]	11553805	0.008289811
A_23_P99186	*TSPAN10*	Homo sapiens tetraspanin 10 (TSPAN10), transcript variant 1, mRNA [NM_001290212]	-0.78626513	0.009533832
A_32_P7204	*CLPP*	Homo sapiens caseinolytic mitochondrial matrix peptidase proteolytic subunit (CLPP), mRNA [NM_006012]	-0.5939116	0.0047151954
A_23_P156687	*UBE3B*	Homo sapiens ubiquitin protein ligase E3B (UBE3B), transcript variant 3, mRNA [NM_183415]	-0.38778174	0.0064965826
A_22_P00018308	*RAB28*	Homo sapiens RAB28, member RAS oncogene family (RAB28), transcript variant 2, mRNA [NM_004249]	-0.72083634	0.007196166
A_33_P3250953	*CFB*	Homo sapiens complement factor B (CFB), mRNA [NM_001710]	2629652	0.003162355
A_23_P131683	*lnc-ZNF841-1*	Homo sapiens full length insert cDNA clone ZD41C11. [AF086256]	-12051837	0.006163671
A_23_P37375	*SLC35B4*	Homo sapiens solute carrier family 35 (UDP-xylose/UDP-N-acetylglucosamine transporter), member B4 (SLC35B4), mRNA [NM_032826]	-10285287	0.0062159016
A_33_P3354267	*MAPRE3*	Homo sapiens microtubule-associated protein, RP/EB family, member 3 (MAPRE3), transcript variant 1, mRNA [NM_012326]	19758086	0.009898471
A_33_P3345474	*RPS6KA5*	Homo sapiens ribosomal protein S6 kinase, 90kDa, polypeptide 5 (RPS6KA5), transcript variant 1, mRNA [NM_004755]	-15085186	0.0032831472
A_23_P43726	*AKIRIN1*	Homo sapiens akirin 1 (AKIRIN1), transcript variant 1, mRNA [NM_024595]	-0.58460784	0.0065723537
A_24_P123720	*NUP160*	Homo sapiens nucleoporin 160kDa (NUP160), mRNA [NM_015231]	-0.48931047	0.0134738535
A_33_P3256500	*VAPB*	Homo sapiens VAMP (vesicle-associated membrane protein)-associated protein B and C (VAPB), transcript variant 1, mRNA [NM_004738]	-0.8883259	0.005641348
A_23_P204423	*SEC63*	Homo sapiens SEC63 homolog (S. cerevisiae) (SEC63), mRNA [NM_007214]	-0.28598264	0.0049917083
A_23_P130158	*ATXN2*	ataxin 2 [Source:HGNC Symbol;Acc:HGNC:10555] [ENST00000392645]	-11571131	0.016938152
A_21_P0014903	*PPP1CC*	Homo sapiens protein phosphatase 1, catalytic subunit, gamma isozyme (PPP1CC), transcript variant 1, mRNA [NM_002710]	-0.47855115	0.010827251
A_33_P3368675	*WNT3*	Homo sapiens wingless-type MMTV integration site family, member 3 (WNT3), mRNA [NM_030753]	-0.5798833	0.0133306645

A_33_P3296169	*C6orf120*	Homo sapiens chromosome 6 open reading frame 120 (C6orf120), mRNA [NM_001029863]	-0.6036019	0.013815334
A_23_P70148	*POC1B*	Homo sapiens POC1 centriolar protein B (POC1B), transcript variant 1, mRNA [NM_172240]	-1042828	0.0025001846
A_33_P3265749	*CDCA7*	Homo sapiens cell division cycle associated 7 (CDCA7), transcript variant 1, mRNA [NM_031942]	0.65335155	0.013971014
A_19_P00322220	*NNT*	Homo sapiens nicotinamide nucleotide transhydrogenase (NNT), transcript variant 2, mRNA [NM_182977]	-0.46537358	0.0056002573
A_24_P200023	*PTGER3*	Homo sapiens prostaglandin E receptor 3 (subtype EP3) (PTGER3), transcript variant 7, mRNA [NM_198717]	15140783	0.008795671
A_33_P3383762	*SNHG17*	Homo sapiens small nucleolar RNA host gene 17 (non-protein coding) (SNHG17), transcript variant 2, long non-coding RNA [NR_027241]	-10584993	0.008392971
A_33_P3238920	*IL1R1*	Homo sapiens interleukin 1 receptor, type I (IL1R1), transcript variant 1, mRNA [NM_000877]	-0.58026636	0.014244717
A_33_P3338360	*INPP5F*	Homo sapiens inositol polyphosphate-5-phosphatase F (INPP5F), transcript variant 3, mRNA [NM_001243195]	-0.78704906	0.009074582
A_23_P170088	*FAM106A*	Homo sapiens family with sequence similarity 106, member A (FAM106A), long non-coding RNA [NR_026809]	14706734	0.012053988
A_24_P80181	*SCARNA13*	Homo sapiens small Cajal body-specific RNA 13 (SCARNA13), guide RNA [NR_003002]	-16155368	0.004296912
A_33_P3422030	*EXD3*	Homo sapiens exonuclease 3'-5' domain containing 3 (EXD3), transcript variant 4, non-coding RNA [NR_104599]	0.4512013	0.010773812
A_21_P0001711	*TMEM127*	Homo sapiens transmembrane protein 127 (TMEM127), transcript variant 1, mRNA [NM_017849]	-0.8123197	0.007683989
A_33_P3344733	*FXYD5*	FXYD domain containing ion transport regulator 5 [Source:HGNC Symbol;Acc:HGNC:4029] [ENST00000392218]	0.7050838	0.005701378
A_33_P3247848	*PCAT6*	Homo sapiens prostate cancer associated transcript 6 (non-protein coding) (PCAT6), transcript variant 2, long non-coding RNA [NR_046326]	0.38799718	0.008414938
A_33_P3504659	*NUBPL*	Homo sapiens nucleotide binding protein-like (NUBPL), transcript variant 1, mRNA [NM_025152]	-0.8663928	0.006639663
A_22_P00004708	*ZNF814*	Homo sapiens zinc finger protein 814 (ZNF814), mRNA [NM_001144989]	-0.45042884	0.010136075

A_22_P00018327	*CASP10*	Homo sapiens caspase 10, apoptosis-related cysteine peptidase (CASP10), transcript variant 1, mRNA [NM_032977]	-0.9808075	0.0029691157
A_24_P122921	*lnc-CTD-3080F16.3.1-1*	LNCipedia lincRNA (lnc-CTD-3080F16.3.1-1), lincRNA [lnc-CTD-3080F16.3.1-1:1]	1424243	0.0029471128
A_23_P328766	*lnc-ZNF91-2*	LNCipedia lincRNA (lnc-ZNF91-2), lincRNA [lnc-ZNF91-2:1]	-17878721	0.00556941
A_33_P3292297	*BCL2L11*	Homo sapiens BCL2-like 11 (apoptosis facilitator) (BCL2L11), transcript variant 1, mRNA [NM_138621]	-12648402	0.0053870557
A_33_P3416622	*ZNF519*	Homo sapiens zinc finger protein 519 (ZNF519), transcript variant 1, mRNA [NM_145287]	-0.9329681	0.013926392
A_23_P95130	*COPS7B*	Homo sapiens COP9 signalosome subunit 7B (COPS7B), transcript variant 1, mRNA [NM_001282950]	0.9082962	0.0027348737
A_33_P3271470	*LINC00322*	Homo sapiens cDNA FLJ16545 fis, clone OCBBF3004972. [AK131425]	0.5761886	0.016549561
A_23_P15348	*SLC37A3*	Homo sapiens solute carrier family 37, member 3 (SLC37A3), transcript variant 1, mRNA [NM_207113]	-0.965811	0.007836227
A_22_P00003533	*GREB1L*	Homo sapiens growth regulation by estrogen in breast cancer-like (GREB1L), mRNA [NM_001142966]	24434109	0.006059527
A_21_P0013771	*MPRIP*	Homo sapiens myosin phosphatase Rho interacting protein (MPRIP), transcript variant 1, mRNA [NM_015134]	-0.76627755	0.014774532
A_23_P1292	*lnc-CCNE2-1*	LNCipedia lincRNA (lnc-CCNE2-1), lincRNA [lnc-CCNE2-1:1]	-0.5112982	0.010931163
A_23_P347059	*XLOC_l2_015561*	BROAD Institute lincRNA (XLOC_l2_015561), lincRNA [TCONS_l2_00030252]	11756251	0.0044240346
A_23_P316582	*ERCC6*	Homo sapiens excision repair cross-complementation group 6 (ERCC6), mRNA [NM_000124]	-18560696	0.0025001846
A_23_P254702	*MOB1B*	Homo sapiens MOB kinase activator 1B (MOB1B), transcript variant 2, mRNA [NM_173468]	-0.73693705	0.0034766807
A_24_P32887	*ZNF441*	Homo sapiens zinc finger protein 441 (ZNF441), mRNA [NM_152355]	-0.8615921	0.007103236
A_33_P3246010	*DEK*	Homo sapiens DEK proto-oncogene (DEK), transcript variant 1, mRNA [NM_003472]	-0.633882	0.007819978
A_23_P210330	*BRD3*	Homo sapiens bromodomain containing 3 (BRD3), mRNA [NM_007371]	-1032411	0.014951782
A_23_P374351	*LGALSL*	Homo sapiens lectin, galactoside-binding-like (LGALSL), mRNA [NM_014181]	-25942402	0.0049768128
A_24_P940149	*FAM65B*	Homo sapiens family with sequence similarity 65, member B (FAM65B), transcript variant 2, mRNA [NM_015864]	-30680864	0.010541298

A_33_P3380098	*KCTD20*	Homo sapiens potassium channel tetramerization domain containing 20 (KCTD20), transcript variant 1, mRNA [NM_173562]	-16048281	0.005787274
A_23_P85604	*C2CD2*	Homo sapiens C2 calcium-dependent domain containing 2 (C2CD2), transcript variant 1, mRNA [NM_015500]	0.91813785	0.014591436
A_23_P162596	*PXN*	Homo sapiens paxillin (PXN), transcript variant 3, mRNA [NM_001243756]	13744879	0.00556941
A_23_P115645	*WDR26*	Homo sapiens WD repeat domain 26 (WDR26), transcript variant 1, mRNA [NM_025160]	-0.47902173	0.008977069
A_22_P00007929	*ACTR6*	Homo sapiens ARP6 actin-related protein 6 homolog (yeast) (ACTR6), transcript variant 1, mRNA [NM_022496]	-10098858	0.0025144578
A_23_P25626	*CELF2*	Homo sapiens CUGBP, Elav-like family member 2 (CELF2), transcript variant 1, mRNA [NM_001025076]	-0.9608666	0.008208044
A_33_P3380462	*lnc-HTR1B-1*	LNCipedia lincRNA (lnc-HTR1B-1), lincRNA [lnc-HTR1B-1:1]	16669769	0.008899145
A_19_P00319404	*BORA*	Homo sapiens bora, aurora kinase A activator (BORA), transcript variant 1, mRNA [NM_024808]	-0.71268034	0.010061895
A_33_P3316639	*GPR88*	Homo sapiens G protein-coupled receptor 88 (GPR88), mRNA [NM_022049]	0.60249263	0.009429358
A_22_P00017432	*LINC00472*	Homo sapiens long intergenic non-protein coding RNA 472 (LINC00472), transcript variant 1, long non-coding RNA [NR_121612]	-13373224	0.009291781
A_23_P50697	*CHMP3*	Homo sapiens charged multivesicular body protein 3 (CHMP3), transcript variant 3, mRNA [NM_001193517]	-0.75995183	0.01329452
A_23_P329740	*lnc-VN1R2-1*	LNCipedia lincRNA (lnc-VN1R2-1), lincRNA [lnc-VN1R2-1:1]	13577927	0.005229406
A_24_P161973	*PSG1*	Homo sapiens pregnancy specific beta-1-glycoprotein 1 (PSG1), transcript variant 1, mRNA [NM_006905]	1485573	0.010447919
A_21_P0013303	*UBC*	Homo sapiens ubiquitin C (UBC), mRNA [NM_021009]	-0.51362276	0.0036402496
A_23_P23748	*ATP11A*	Homo sapiens ATPase, class VI, type 11A (ATP11A), transcript variant 1, mRNA [NM_015205]	-0.52137184	0.009582734
A_33_P3629678	*SEPT7P2*	Homo sapiens septin 7 pseudogene 2 (SEPT7P2), non-coding RNA [NR_024271]	-10765307	0.009794734
A_23_P217319	*WDR47*	Homo sapiens WD repeat domain 47 (WDR47), transcript variant 2, mRNA [NM_014969]	-0.5937721	0.009576729
A_23_P403745	*COL5A1*	Homo sapiens collagen, type V, alpha 1 (COL5A1), transcript variant 1, mRNA [NM_000093]	-1028478	0.008312506

A_33_P3590259	*FGF13*	Homo sapiens fibroblast growth factor 13 (FGF13), transcript variant 1, mRNA [NM_004114]	-12648146	0.0038456172
A_23_P358662	*MTSS1L*	Homo sapiens metastasis suppressor 1-like (MTSS1L), mRNA [NM_138383]	-134693	0.014548385
A_23_P145485	*CXCL14*	Homo sapiens chemokine (C-X-C motif) ligand 14 (CXCL14), mRNA [NM_004887]	-5821286	0.011497865
A_23_P58647	*CEP44*	Homo sapiens centrosomal protein 44kDa (CEP44), transcript variant 1, mRNA [NM_001040157]	-0.89413726	0.006573993
A_33_P3645465	*ULBP2*	Homo sapiens UL16 binding protein 2 (ULBP2), mRNA [NM_025217]	-0.7936795	0.016513823
A_23_P411881	*CTNNA1*	Homo sapiens catenin (cadherin-associated protein), alpha 1, 102kDa (CTNNA1), transcript variant 1, mRNA [NM_001903]	-0.8004236	0.01211171
A_32_P155416	*PDCD4-AS1*	Homo sapiens PDCD4 antisense RNA 1 (PDCD4-AS1), long non-coding RNA [NR_026932]	-15385102	0.006500368
A_21_P0011948	*PIBF1*	Homo sapiens progesterone immunomodulatory binding factor 1 (PIBF1), mRNA [NM_006346]	-0.58289593	0.0072200163
A_33_P3393341	*ERI3*	Homo sapiens ERI1 exoribonuclease family member 3 (ERI3), transcript variant 1, mRNA [NM_024066]	0.8849067	0.011851147
A_33_P3310929	*LPIN3*	Homo sapiens lipin 3 (LPIN3), transcript variant 1, mRNA [NM_001301860]	-0.685672	0.008782249
A_33_P3409124	*ZNF197*	Homo sapiens zinc finger protein 197 (ZNF197), transcript variant 1, mRNA [NM_006991]	-0.5300707	0.009862303
A_33_P3346688	*ADAM12*	Homo sapiens ADAM metallopeptidase domain 12 (ADAM12), transcript variant 1, mRNA [NM_003474]	-15360348	0.0025001846
A_32_P76060	*NR1D2*	Homo sapiens nuclear receptor subfamily 1, group D, member 2 (NR1D2), transcript variant 1, mRNA [NM_005126]	-0.5915847	0.011054281
A_23_P113161	*CTDSPL2*	Homo sapiens CTD (carboxy-terminal domain, RNA polymerase II, polypeptide A) small phosphatase like 2 (CTDSPL2), mRNA [NM_016396]	-11209209	0.0060682436
A_22_P00016430	*lnc-UXS1-4*	LNCipedia lincRNA (lnc-UXS1-4), lincRNA [lnc-UXS1-4:1]	0.60587734	0.0163094
A_23_P56736	*C1orf21*	Homo sapiens chromosome 1 open reading frame 21 (C1orf21), mRNA [NM_030806]	-12438544	0.0059317574
A_33_P3372368	*TUBA3D*	Homo sapiens tubulin, alpha 3d (TUBA3D), mRNA [NM_080386]	0.58535993	0.008781871
A_32_P69930	*BRK1*	Homo sapiens BRICK1, SCAR/WAVE actin-nucleating complex subunit (BRK1), mRNA [NM_018462]	-0.8264346	0.007826014

A_23_P108785	*KRT8*	Homo sapiens keratin 8, type II (KRT8), transcript variant 4, non-coding RNA [NR_045962]	17849331	0.003993975
A_23_P501770	*CCDC96*	Homo sapiens coiled-coil domain containing 96 (CCDC96), mRNA [NM_153376]	-11143085	0.010827594
A_23_P64611	*ACTR3*	Homo sapiens ARP3 actin-related protein 3 homolog (yeast) (ACTR3), transcript variant 1, mRNA [NM_005721]	-11698527	0.004370471
A_33_P3308050	*ATRIP*	Homo sapiens ATR interacting protein (ATRIP), transcript variant 2, mRNA [NM_032166]	0.6236049	0.012481278
A_32_P116660	*P2RY6*	Homo sapiens pyrimidinergic receptor P2Y, G-protein coupled, 6 (P2RY6), transcript variant 2, mRNA [NM_176798]	-1428268	0.010237655
A_21_P0013500	*EIF4E2*	Homo sapiens eukaryotic translation initiation factor 4E family member 2 (EIF4E2), transcript variant 1, mRNA [NM_004846]	-0.7936984	0.013076766
A_21_P0014427	*RBM43*	Homo sapiens RNA binding motif protein 43 (RBM43), mRNA [NM_198557]	-0.7596343	0.00862906
A_33_P3231162	*XLOC_l2_014294*	BROAD Institute lincRNA (XLOC_l2_014294), lincRNA [TCONS_l2_00027902]	0.30983797	0.012873199
DCP_20_0	*lnc-CHADL-1*	Homo sapiens cDNA FLJ32615 fis, clone STOMA2000148. [AK057177]	-13557477	0.010617712
A_24_P72518	*ZNF776*	Homo sapiens zinc finger protein 776 (ZNF776), mRNA [NM_173632]	-0.9250844	0.014162805
A_24_P408736	*AHCYL2*	Homo sapiens adenosylhomocysteinase-like 2 (AHCYL2), transcript variant 1, mRNA [NM_015328]	-11834109	0.006812377
A_33_P3302255	*NKAPL*	Homo sapiens NFKB activating protein-like (NKAPL), mRNA [NM_001007531]	0.68789685	0.007384838
A_23_P105409	*GALNT5*	Homo sapiens polypeptide N-acetylgalactosaminyltransferase 5 (GALNT5), mRNA [NM_014568]	-11105411	0.0037583082
A_32_P46840	*ITM2B*	Homo sapiens integral membrane protein 2B (ITM2B), mRNA [NM_021999]	-0.96327925	0.0047272886
A_23_P357284	*MAP3K12*	Homo sapiens mitogen-activated protein kinase kinase kinase 12 (MAP3K12), transcript variant 2, mRNA [NM_006301]	10580384	0.010665993
A_33_P3267640	*LOC729680*	Homo sapiens cDNA FLJ33345 fis, clone BRACE2003713. [AK090664]	12462639	0.008064267
A_23_P413634	*GPR4*	Homo sapiens G protein-coupled receptor 4 (GPR4), mRNA [NM_005282]	-18707728	0.0064337924
A_23_P54758	*HGFAC*	Homo sapiens HGF activator (HGFAC), transcript variant 1, mRNA [NM_001297439]	0.6284463	0.01213097
A_23_P211957	*ZNF329*	Homo sapiens zinc finger protein 329 (ZNF329), mRNA [NM_024620]	-11717117	0.0046320097
A_23_P81369	*GDE1*	Homo sapiens glycerophosphodiester phosphodiesterase 1 (GDE1), mRNA [NM_016641]	-0.6560778	0.015203984

A_21_P0011773	*TGFBR2*	Homo sapiens transforming growth factor, beta receptor II (70/80kDa) (TGFBR2), transcript variant 1, mRNA [NM_001024847]	-0.81090134	0.009717243
A_21_P0014396	*ADAM19*	Homo sapiens ADAM metallopeptidase domain 19 (ADAM19), mRNA [NM_033274]	13827642	0.016171295
A_23_P115743	*CENPBD1P1*	CENPBD1 pseudogene 1 [Source:HGNC Symbol;Acc:HGNC:28421] [ENST00000492393]	-0.74282175	0.015734134
A_32_P205944	*IPO5P1*	Homo sapiens importin 5 pseudogene 1 (IPO5P1), transcript variant 1, non-coding RNA [NR_103741]	-0.50628614	0.016667983
A_22_P00017797	*FAM204A*	Homo sapiens family with sequence similarity 204, member A (FAM204A), transcript variant 1, mRNA [NM_022063]	-0.8297199	0.010252496
A_33_P3259148	*RGPD5*	Homo sapiens RANBP2-like and GRIP domain containing 5 (RGPD5), transcript variant 1, mRNA [NM_005054]	-12395653	0.003956951
A_21_P0014535	*ZNF286B*	Homo sapiens zinc finger protein 286B (ZNF286B), mRNA [NM_001145045]	-0.6220738	0.0100634415
A_23_P385063	*COX6C*	Homo sapiens cytochrome c oxidase subunit VIc (COX6C), mRNA [NM_004374]	-0.6541171	0.0033868244
A_24_P261724	*LOC102724348*	PREDICTED: Homo sapiens uncharacterized LOC102724348 (LOC102724348), ncRNA [XR_429392]	-11946218	0.0073276176
A_21_P0005578	*DNAJB6*	Homo sapiens DnaJ (Hsp40) homolog, subfamily B, member 6 (DNAJB6), transcript variant 1, mRNA [NM_058246]	-0.4025238	0.01418328
A_22_P00014777	*RNF10*	Homo sapiens ring finger protein 10 (RNF10), mRNA [NM_014868]	-0.7407005	0.00955837
A_33_P3870056	*lnc-EXOC4-2*	LNCipedia lincRNA (lnc-EXOC4-2), lincRNA [lnc-EXOC4-2:1]	0.7102229	0.012795951
A_22_P00006888	*lnc-SLC38A6-1*	Homo sapiens cDNA FLJ27270 fis, clone TMS00491. [AK130780]	-14229101	0.013529562
A_23_P52738	*LOC283335*	Homo sapiens uncharacterized LOC283335 (LOC283335), long non-coding RNA [NR_033854]	35987542	0.00978609
A_23_P115792	*DCPS*	Homo sapiens decapping enzyme, scavenger (DCPS), mRNA [NM_014026]	-0.45740944	0.0044240346
A_33_P3319026	*MGST3*	Homo sapiens microsomal glutathione S-transferase 3 (MGST3), mRNA [NM_004528]	-10965667	0.004613805
A_24_P85775	*PLEKHA1*	Homo sapiens pleckstrin homology domain containing, family A (phosphoinositide binding specific) member 1 (PLEKHA1), transcript variant 2, mRNA [NM_001001974]	-1186141	0.003162355
A_33_P3319041	*ZZZ3*	Homo sapiens zinc finger, ZZ-type containing 3 (ZZZ3), mRNA [NM_015534]	-0.62817883	0.006077557
A_32_P234935	*THEMIS2*	Homo sapiens thymocyte selection associated family member 2 (THEMIS2), transcript variant 2, mRNA [NM_001039477]	0.9971394	0.011258477

A_33_P3334443	*HMGB3*	Homo sapiens high mobility group box 3 (HMGB3), transcript variant 1, mRNA [NM_001301228]	-0.28460103	0.011422752
A_23_P259166	*TARDBP*	Homo sapiens TAR DNA binding protein (TARDBP), mRNA [NM_007375]	-0.49650505	0.0046812603
A_33_P3221034	*FAM69A*	Homo sapiens family with sequence similarity 69, member A (FAM69A), transcript variant 4, mRNA [NM_001252271]	-14012544	0.005377644
A_33_P3537875	*TCEAL4*	Homo sapiens transcription elongation factor A (SII)-like 4 (TCEAL4), transcript variant 1, mRNA [NM_024863]	-11797183	0.008274833
A_33_P3292126	*SMIM17*	Homo sapiens small integral membrane protein 17 (SMIM17), mRNA [NM_001193628]	-0.76902956	0.0068576415
A_33_P3291776	*MGC10814*	Homo sapiens hypothetical protein MGC10814, mRNA (cDNA clone MGC:10814 IMAGE:3613095), complete cds. [BC004943]	-0.790539	0.010060375
A_24_P178444	*XLOC_l2_013837*	BROAD Institute lincRNA (XLOC_l2_013837), lincRNA [TCONS_l2_00026575]	0.9158936	0.011476698
A_23_P417113	*NANS*	Homo sapiens N-acetylneuraminic acid synthase (NANS), mRNA [NM_018946]	-0.9181525	0.008691653
A_22_P00021205	*RBM33*	RNA binding motif protein 33 [Source:HGNC Symbol;Acc:HGNC:27223] [ENST00000287912]	-0.52583843	0.008289811
A_23_P205567	*TARID*	Homo sapiens TCF21 antisense RNA inducing promoter demethylation (TARID), long non-coding RNA [NR_109982]	1660565	0.003956951
A_22_P00010235	*HAGLROS*	Homo sapiens HAGLR opposite strand (non-protein coding) (HAGLROS), long non-coding RNA [NR_110457]	-0.34785125	0.01666397
A_21_P0000833	*PRKCH*	Homo sapiens protein kinase C, eta (PRKCH), mRNA [NM_006255]	-0.7889616	0.015456718
A_23_P251043	*MT1B*	Homo sapiens metallothionein 1B (MT1B), mRNA [NM_005947]	15779829	0.008298705
A_23_P113613	*SENCR*	Homo sapiens smooth muscle and endothelial cell enriched migration/differentiation-associated long non-coding RNA (SENCR), long non-coding RNA [NR_038908]	-241538	0.014476213
A_21_P0000363	*SYNDIG1*	Homo sapiens synapse differentiation inducing 1 (SYNDIG1), mRNA [NM_024893]	-3451566	0.004156154
A_33_P3245321	*CDCP1*	Homo sapiens CUB domain containing protein 1 (CDCP1), transcript variant 1, mRNA [NM_022842]	3841755	0.0025001846
A_23_P64792	*SNORA25*	Homo sapiens small nucleolar RNA, H/ACA box 25 (SNORA25), small nucleolar RNA [NR_003028]	-17672783	0.003667498
A_22_P00016780	*CENPP*	Homo sapiens centromere protein P (CENPP), transcript variant 1, mRNA [NM_001012267]	-0.77175903	0.0140423635

A_23_P147845	*KCNMB4*	Homo sapiens potassium channel subfamily M regulatory beta subunit 4 (KCNMB4), mRNA [NM_014505]	-13962617	0.0041987738
A_23_P354027	*lnc-TRIM41-3*	LNCipedia lincRNA (lnc-TRIM41-3), lincRNA [lnc-TRIM41-3:1]	12708173	0.011276983
A_21_P0014699	*PDXDC1*	Homo sapiens pyridoxal-dependent decarboxylase domain containing 1 (PDXDC1), transcript variant 1, mRNA [NM_015027]	-0.6838212	0.010396678
A_32_P63848	*KCTD11*	Homo sapiens potassium channel tetramerization domain containing 11 (KCTD11), mRNA [NM_001002914]	-0.98959875	0.0070605814
A_33_P3256334	*ZNF528-AS1*	PREDICTED: Homo sapiens uncharacterized LOC102724105 (LOC102724105), ncRNA [XR_430257]	-10726833	0.004016376
A_21_P0004593	*OXCT1*	Homo sapiens 3-oxoacid CoA transferase 1 (OXCT1), mRNA [NM_000436]	-0.7732377	0.00910823
A_21_P0003359	*PRB3*	Homo sapiens proline-rich protein BstNI subfamily 3 (PRB3), mRNA [NM_006249]	0.9278828	0.0059536803
A_23_P29855	*LOC283335*	Homo sapiens uncharacterized LOC283335 (LOC283335), long non-coding RNA [NR_033854]	-0.89997125	0.0046024295
A_23_P59677	*TMCO1*	Homo sapiens transmembrane and coiled-coil domains 1 (TMCO1), transcript variant 1, mRNA [NM_019026]	-0.93536973	0.0044319443
A_32_P117422	*USO1*	Homo sapiens USO1 vesicle transport factor (USO1), transcript variant 2, mRNA [NM_003715]	-0.53211975	0.0152104935
A_33_P3311551	*RINT1*	Homo sapiens RAD50 interactor 1 (RINT1), mRNA [NM_021930]	-0.6765282	0.009626979
A_33_P3381513	*PMS2P4*	Homo sapiens postmeiotic segregation increased 2 pseudogene 4 (PMS2P4), transcript variant 2, non-coding RNA [NR_022007]	-0.49043423	0.0073845843
A_23_P300033	*WLS*	Homo sapiens mRNA for putative NFkB activating protein, complete cds, clone: 373. [AB097018]	0.9253156	0.011106028
A_33_P3384710	*CD274*	Homo sapiens CD274 molecule (CD274), transcript variant 1, mRNA [NM_014143]	20486784	0.012896936
A_23_P47777	*PDGFRA*	Homo sapiens platelet-derived growth factor receptor, alpha polypeptide (PDGFRA), mRNA [NM_006206]	-0.629306	0.004288771
A_33_P3334948	*FAM222B*	Homo sapiens family with sequence similarity 222, member B (FAM222B), transcript variant 3, mRNA [NM_001288631]	0.8450205	0.0034766807
A_23_P427760	*MARCH9*	Homo sapiens membrane-associated ring finger (C3HC4) 9 (MARCH9), mRNA [NM_138396]	10592581	0.0075625386
A_24_P270525	*PIWIL4*	Homo sapiens piwi-like RNA-mediated gene silencing 4 (PIWIL4), mRNA [NM_152431]	-17722354	0.0055521973

A_23_P331943	*LINC00961*	Homo sapiens long intergenic non-protein coding RNA 961 (LINC00961), long non-coding RNA [NR_024283]	-13106885	0.0033094941
A_23_P391275	*GTF2A2*	Homo sapiens general transcription factor IIA, 2, 12kDa (GTF2A2), mRNA [NM_004492]	-0.23362787	0.0038144474
A_23_P122863	*TMEM181*	Homo sapiens transmembrane protein 181 (TMEM181), mRNA [NM_020823]	-0.54123133	0.013327314
A_24_P655268	*RCAN3*	Homo sapiens RCAN family member 3 (RCAN3), transcript variant 1, mRNA [NM_013441]	-0.74858737	0.008298705
A_23_P25030	*GRB10*	Homo sapiens growth factor receptor-bound protein 10 (GRB10), transcript variant 4, mRNA [NM_001001555]	-14930863	0.0034884813
A_33_P3418158	*OIP5-AS1*	Homo sapiens OIP5 antisense RNA 1 (OIP5-AS1), long non-coding RNA [NR_026757]	-0.26985762	0.011672903
A_23_P206140	*HSD17B6*	Homo sapiens hydroxysteroid (17-beta) dehydrogenase 6 (HSD17B6), mRNA [NM_003725]	-24458642	0.0025001846
A_21_P0005534	*SLC2A8*	Homo sapiens solute carrier family 2 (facilitated glucose transporter), member 8 (SLC2A8), transcript variant 2, mRNA [NM_001271711]	14569981	0.008755959
A_22_P00020428	*DNAJA4*	Homo sapiens DnaJ (Hsp40) homolog, subfamily A, member 4 (DNAJA4), transcript variant 1, mRNA [NM_018602]	-10668397	0.0071290513
A_33_P3223678	*lnc-MRPS33-1*	LNCipedia lincRNA (lnc-MRPS33-1), lincRNA [lnc-MRPS33-1:1]	0.8628285	0.006816371
A_24_P142743	*LHX3*	Homo sapiens LIM homeobox 3 (LHX3), transcript variant 2, mRNA [NM_014564]	0.88903284	0.002804883
A_23_P40192	*NIPSNAP3A*	Homo sapiens nipsnap homolog 3A (C. elegans) (NIPSNAP3A), mRNA [NM_015469]	-17343225	0.003365343
A_23_P44836	*CNN2*	calponin 2 [Source:HGNC Symbol;Acc:HGNC:2156] [ENST00000564572]	-17713366	0.0057647754
A_33_P3255782	*CDH22*	Homo sapiens cadherin 22, type 2 (CDH22), mRNA [NM_021248]	0.9918357	0.009835869
A_23_P412321	*NT5DC2*	Homo sapiens 5'-nucleotidase domain containing 2 (NT5DC2), transcript variant 2, mRNA [NM_022908]	13470908	0.0066793696
A_33_P3264346	*CCR5*	Homo sapiens chemokine (C-C motif) receptor 5 (gene/pseudogene) (CCR5), transcript variant A, mRNA [NM_000579]	0.5702336	0.013806593
A_33_P3252359	*SESN3*	Homo sapiens sestrin 3 (SESN3), transcript variant 1, mRNA [NM_144665]	-15811234	0.006573993
A_33_P3260969	*BDH1*	Homo sapiens 3-hydroxybutyrate dehydrogenase, type 1 (BDH1), transcript variant 3, mRNA [NM_203314]	-0.95625436	0.00978609

A_23_P45389	*LRRC20*	Homo sapiens leucine rich repeat containing 20 (LRRC20), transcript variant 3, mRNA [NM_018205]	-12515327	0.005192162
A_33_P3240043	*PRAP1*	Homo sapiens proline-rich acidic protein 1 (PRAP1), transcript variant 1, mRNA [NM_145202]	0.6669962	0.0151486425
A_32_P101031	*RAB9A*	Homo sapiens RAB9A, member RAS oncogene family (RAB9A), transcript variant 1, mRNA [NM_004251]	-12115712	0.009938401
A_23_P167595	*lnc-AC005323.1.1-1*	LNCipedia lincRNA (lnc-AC005323.1.1-1), lincRNA [lnc-AC005323.1.1-1:1]	-1994174	0.012288998
A_24_P376322	*LYPD1*	Homo sapiens LY6/PLAUR domain containing 1 (LYPD1), transcript variant 1, mRNA [NM_144586]	-19732611	0.0069250786
A_23_P371266	*UBE2B*	Homo sapiens ubiquitin-conjugating enzyme E2B (UBE2B), mRNA [NM_003337]	-0.8957596	0.011156819
A_24_P161463	*MBD1*	Homo sapiens methyl-CpG binding domain protein 1 (MBD1), transcript variant 2, mRNA [NM_015845]	0.7584365	0.0027519173
A_23_P68007	*DNM3*	Homo sapiens dynamin 3 (DNM3), transcript variant 1, mRNA [NM_015569]	-11129296	0.007871983
A_33_P7477724	*ZFAS1*	Homo sapiens ZNFX1 antisense RNA 1 (ZFAS1), transcript variant 2, long non-coding RNA [NR_003605]	-0.9074566	0.0026564114
A_33_P3393941	*ATP1B3*	Homo sapiens ATPase, Na+/K+ transporting, beta 3 polypeptide (ATP1B3), mRNA [NM_001679]	-0.20844573	0.016331382
A_22_P00009075	*lnc-VAMP1-1*	LNCipedia lincRNA (lnc-VAMP1-1), lincRNA [lnc-VAMP1-1:6]	-13424897	0.0043161632
A_33_P3319581	*MBTPS1*	Homo sapiens membrane-bound transcription factor peptidase, site 1 (MBTPS1), mRNA [NM_003791]	-0.5754358	0.013286371
A_21_P0011017	*lnc-LETM2-1*	LNCipedia lincRNA (lnc-LETM2-1), lincRNA [lnc-LETM2-1:1]	0.4877196	0.014847625
A_33_P3367917	*FIGNL2*	Homo sapiens fidgetin-like 2 (FIGNL2), mRNA [NM_001013690]	0.6955008	0.007234553
A_23_P210496	*XLOC_l2_002767*	BROAD Institute lincRNA (XLOC_l2_002767), lincRNA [TCONS_l2_00005184]	-13179489	0.0037664857
A_33_P3249529	*SSH2*	Homo sapiens slingshot protein phosphatase 2 (SSH2), transcript variant 3, mRNA [NM_001282130]	0.8027382	0.004788691
A_23_P120953	*PCIF1*	Homo sapiens PDX1 C-terminal inhibiting factor 1 (PCIF1), mRNA [NM_022104]	13878124	0.0039497027
A_32_P37360	*PCNX*	Homo sapiens pecanex homolog (Drosophila) (PCNX), mRNA [NM_014982]	-0.3415168	0.010591297

A_24_P127719	*SERHL2*	Homo sapiens serine hydrolase-like 2 (SERHL2), transcript variant 1, mRNA [NM_014509]	-0.65382415	0.010052235
A_23_P151746	*LACE1*	Homo sapiens lactation elevated 1 (LACE1), mRNA [NM_145315]	-0.94940656	0.01147035
A_33_P3246418	*MAFA*	Homo sapiens v-maf avian musculoaponeurotic fibrosarcoma oncogene homolog A (MAFA), mRNA [NM_201589]	0.6257747	0.013386562
A_23_P340251	*ISCA2*	Homo sapiens iron-sulfur cluster assembly 2 (ISCA2), transcript variant 1, mRNA [NM_194279]	-1027118	0.008079168
A_33_P3519683	*MDFI*	Homo sapiens MyoD family inhibitor (MDFI), transcript variant 1, mRNA [NM_001300804]	-31155305	0.0048989807
A_33_P3317948	*RAB2A*	Homo sapiens RAB2A, member RAS oncogene family (RAB2A), transcript variant 1, mRNA [NM_002865]	-0.8997374	0.009682345
A_32_P25357	*ZBTB8OS*	Homo sapiens zinc finger and BTB domain containing 8 opposite strand (ZBTB8OS), mRNA [NM_178547]	-0.48181653	0.012512322
A_21_P0000520	*TP73-AS1*	Homo sapiens TP73 antisense RNA 1 (TP73-AS1), transcript variant 1, long non-coding RNA [NR_033711]	-0.6819924	0.008080026
A_19_P00315551	*CDH15*	Homo sapiens cadherin 15, type 1, M-cadherin (myotubule) (CDH15), mRNA [NM_004933]	-0.7898454	0.013955389
A_32_P230547	*LOC400043*	Homo sapiens uncharacterized LOC400043 (LOC400043), long non-coding RNA [NR_026656]	-10622157	0.012581895
A_19_P00322948	*LIFR*	Homo sapiens leukemia inhibitory factor receptor alpha (LIFR), transcript variant 2, mRNA [NM_002310]	-14968898	0.009029172
A_24_P915806	*DOCK7*	Homo sapiens dedicator of cytokinesis 7 (DOCK7), transcript variant 2, mRNA [NM_033407]	-12157335	0.006708236
A_24_P56689	*SNHG6*	Homo sapiens small nucleolar RNA host gene 6 (non-protein coding) (SNHG6), long non-coding RNA [NR_002599]	-0.6869971	0.0031512622
A_22_P00004210	*HNMT*	Homo sapiens histamine N-methyltransferase (HNMT), transcript variant 2, mRNA [NM_001024074]	-0.553816	0.0071450765
A_23_P217968	*ZNF205*	Homo sapiens zinc finger protein 205 (ZNF205), transcript variant 1, mRNA [NM_003456]	0.9027074	0.0075928494
A_33_P3351606	*SRD5A3-AS1*	Homo sapiens SRD5A3 antisense RNA 1 (SRD5A3-AS1), long non-coding RNA [NR_037969]	-0.88426125	0.013169497
A_33_P3271455	*SUV420H1*	Homo sapiens suppressor of variegation 4-20 homolog 1 (Drosophila) (SUV420H1), transcript variant 2, mRNA [NM_016028]	-0.5504781	0.014959743

A_33_P3214314	*MIR124-2HG*	Homo sapiens MIR124-2 host gene (non-protein coding) (MIR124-2HG), transcript variant 2, long non-coding RNA [NR_034102]	11011393	0.003162355
A_33_P3406505	*PXDN*	Homo sapiens peroxidasin (PXDN), mRNA [NM_012293]	-0.42540377	0.0070658927
A_33_P3242493	*CCSER2*	Homo sapiens coiled-coil serine-rich protein 2 (CCSER2), transcript variant 3, mRNA [NM_001284241]	-10346453	0.00915229
A_23_P82762	*ELL*	Homo sapiens elongation factor RNA polymerase II (ELL), mRNA [NM_006532]	11896627	0.010116311
A_33_P3368830	*PIBF1*	Homo sapiens progesterone immunomodulatory binding factor 1 (PIBF1), mRNA [NM_006346]	-0.72275794	0.011257593
A_33_P3336720	*ZNF596*	Homo sapiens zinc finger protein 596 (ZNF596), transcript variant 1, mRNA [NM_001042416]	-10082853	0.009524396
A_22_P00024512	*LY9*	Homo sapiens lymphocyte antigen 9 (LY9), transcript variant 2, mRNA [NM_001033667]	0.8845553	0.015939081
A_23_P434352	*HAMP*	Homo sapiens hepcidin antimicrobial peptide (HAMP), mRNA [NM_021175]	0.5318238	0.0036402496
A_24_P65098	*JARID2-AS1*	Homo sapiens JARID2 antisense RNA 1 (JARID2-AS1), long non-coding RNA [NR_120502]	14105064	0.007698106
A_23_P301521	*CAST*	Homo sapiens calpastatin (CAST), transcript variant 6, mRNA [NM_001042440]	-0.68092895	0.0037705272
A_23_P325501	*TMEM87A*	Homo sapiens transmembrane protein 87A (TMEM87A), transcript variant 2, mRNA [NM_001110503]	0.857016	0.0037583082
A_23_P111311	*KIAA1462*	Homo sapiens KIAA1462 (KIAA1462), mRNA [NM_020848]	-0.6916809	0.011047582
A_23_P19624	*MORC3*	Homo sapiens MORC family CW-type zinc finger 3 (MORC3), mRNA [NM_015358]	-0.65085346	0.0072350414
A_22_P00016144	*AKAP12*	Homo sapiens A kinase (PRKA) anchor protein 12 (AKAP12), transcript variant 2, mRNA [NM_144497]	-203951	0.0051764864
A_23_P66525	*BMP6*	Homo sapiens bone morphogenetic protein 6 (BMP6), mRNA [NM_001718]	-21662087	0.009099136
A_24_P810697	*PSMA3-AS1*	Homo sapiens PSMA3 antisense RNA 1 (PSMA3-AS1), transcript variant 2, long non-coding RNA [NR_029435]	-0.6479655	0.013163641
A_24_P226962	*HS3ST3A1*	heparan sulfate (glucosamine) 3-O-sulfotransferase 3A1 [Source:HGNC Symbol;Acc:HGNC:5196] [ENST00000284110]	0.81034154	0.0050531467
A_23_P76918	*MXRA7*	Homo sapiens matrix-remodelling associated 7 (MXRA7), transcript variant 2, mRNA [NM_001008529]	0.33399266	0.011490571
A_23_P91487	*KIAA0368*	Homo sapiens KIAA0368 (KIAA0368), mRNA [NM_001080398]	0.36391163	0.008018198

A_23_P203013	*TIMM9*	Homo sapiens translocase of inner mitochondrial membrane 9 homolog (yeast) (TIMM9), mRNA [NM_012460]	-0.5219066	0.007900263
A_23_P44674	*RWDD2B*	Homo sapiens RWD domain containing 2B (RWDD2B), mRNA [NM_016940]	-0.5154033	0.016973978
A_23_P255317	*NPAT*	Homo sapiens nuclear protein, ataxia-telangiectasia locus (NPAT), mRNA [NM_002519]	-0.5349293	0.011821381
A_33_P3303562	*CRIP1*	Homo sapiens cysteine-rich protein 1 (intestinal) (CRIP1), mRNA [NM_001311]	-13972971	0.0037353234
A_23_P123563	*RNF14*	Homo sapiens ring finger protein 14 (RNF14), transcript variant 1, mRNA [NM_004290]	-0.69307446	0.009938745
A_23_P501007	*RPS6*	Homo sapiens ribosomal protein S6 (RPS6), mRNA [NM_001010]	-0.35887867	0.0067849406
A_23_P129169	*APCDD1L-AS1*	Homo sapiens APCDD1L antisense RNA 1 (head to head) (APCDD1L-AS1), long non-coding RNA [NR_034147]	0.69474185	0.014902025
A_32_P755542	*EFEMP1*	Homo sapiens EGF containing fibulin-like extracellular matrix protein 1 (EFEMP1), transcript variant 2, mRNA [NM_001039348]	-2408025	0.0018723286
A_23_P31055	*CYP11A1*	Homo sapiens cytochrome P450, family 11, subfamily A, polypeptide 1 (CYP11A1), transcript variant 1, mRNA [NM_000781]	0.91548496	0.009853304
A_24_P21266	*TAMM41*	Homo sapiens TAM41, mitochondrial translocator assembly and maintenance protein, homolog (S. cerevisiae) (TAMM41), transcript variant 2, mRNA [NM_138807]	-11219585	0.0034534817
A_23_P373568	*NUP43*	Homo sapiens nucleoporin 43kDa (NUP43), transcript variant 1, mRNA [NM_198887]	-10398576	0.010911651
A_33_P3304888	*ERLIN2*	Homo sapiens ER lipid raft associated 2 (ERLIN2), transcript variant 2, mRNA [NM_001003790]	-10082738	0.012773474
A_33_P3280044	*FUZ*	Homo sapiens fuzzy planar cell polarity protein (FUZ), transcript variant 1, mRNA [NM_025129]	0.36793646	0.0059758257
A_32_P448360	*USP32*	Homo sapiens ubiquitin specific peptidase 32 (USP32), mRNA [NM_032582]	-0.89537954	0.009630487
A_22_P00018556	*ANKRD11*	Homo sapiens ankyrin repeat domain 11 (ANKRD11), transcript variant 1, mRNA [NM_001256182]	-0.5746322	0.013936194
A_23_P138835	*ASB16-AS1*	Homo sapiens ASB16 antisense RNA 1 (ASB16-AS1), transcript variant 1, long non-coding RNA [NR_049729]	0.62260956	0.0132579645
A_33_P3364498	*lnc-AAAS-1*	ALU6_HUMAN (P39193) Alu subfamily SP sequence contamination warning entry, partial (24%) [THC2661571]	-0.58213943	0.010000725

A_33_P3325262	*CAPN1*	Homo sapiens calpain 1, (mu/I) large subunit (CAPN1), transcript variant 2, mRNA [NM_005186]	11791813	0.0032554762
A_33_P3353816	*SLC6A8*	Homo sapiens solute carrier family 6 (neurotransmitter transporter), member 8 (SLC6A8), transcript variant 1, mRNA [NM_005629]	0.9960713	0.010455912
A_33_P3791123	*BMS1P20*	BMS1 pseudogene 8 [Source:HGNC Symbol;Acc:HGNC:49152] [ENST00000565156]	-15685425	0.013705582
A_33_P3539345	*ITGA4*	Homo sapiens integrin, alpha 4 (antigen CD49D, alpha 4 subunit of VLA-4 receptor) (ITGA4), mRNA [NM_000885]	-25686584	0.0045890645
A_21_P0000369	*ATP5L2*	Homo sapiens ATP synthase, H+ transporting, mitochondrial Fo complex, subunit G2 (ATP5L2), mRNA [NM_001165877]	-0.43249348	0.009208136
A_24_P12413	*MYO6*	Homo sapiens myosin VI (MYO6), transcript variant 1, mRNA [NM_004999]	-13558171	0.0140423635
A_33_P6469777	*SNORA16A*	Homo sapiens small nucleolar RNA, H/ACA box 16A (SNORA16A), small nucleolar RNA [NR_003035]	-0.54875374	0.004206936
A_22_P00012113	*TRAM2*	Homo sapiens translocation associated membrane protein 2 (TRAM2), mRNA [NM_012288]	-12372041	0.005512448
A_21_P0000835	*SNHG7*	Homo sapiens small nucleolar RNA host gene 7 (non-protein coding) (SNHG7), transcript variant 2, long non-coding RNA [NR_024542]	-12919039	0.005701378
A_23_P433791	*lnc-POLD3-2*	LNCipedia lincRNA (lnc-POLD3-2), lincRNA [lnc-POLD3-2:1]	0.4290306	0.011877211
A_21_P0010220	*PCOLCE-AS1*	Homo sapiens PCOLCE antisense RNA 1 (PCOLCE-AS1), long non-coding RNA [NR_038910]	-0.729626	0.0064343205
A_23_P131149	*SPRTN*	Homo sapiens SprT-like N-terminal domain (SPRTN), transcript variant 2, mRNA [NM_001010984]	-0.41321754	0.0040572607
A_23_P420256	*ORC4*	Homo sapiens origin recognition complex, subunit 4 (ORC4), transcript variant 2, mRNA [NM_002552]	-0.62443596	0.01262842
A_23_P113623	*CLDN12*	Homo sapiens claudin 12 (CLDN12), transcript variant 3, mRNA [NM_012129]	0.90130913	0.011747539
A_23_P358009	*C2orf43*	Homo sapiens chromosome 2 open reading frame 43 (C2orf43), transcript variant 1, mRNA [NM_021925]	-10168649	0.012164067
A_33_P3702364	*MRPL45*	Homo sapiens mitochondrial ribosomal protein L45 (MRPL45), transcript variant 1, mRNA [NM_032351]	-10005232	0.006543285
A_24_P317450	*DNAJC16*	Homo sapiens DnaJ (Hsp40) homolog, subfamily C, member 16 (DNAJC16), transcript variant 1, mRNA [NM_015291]	-0.7214737	0.015953625

A_33_P3418576	*SNX24*	Homo sapiens sorting nexin 24 (SNX24), mRNA [NM_014035]	-0.49177286	0.009974877
A_33_P3283619	*OSTCP1*	Homo sapiens oligosaccharyltransferase complex subunit pseudogene 1 (OSTCP1), non-coding RNA [NR_028496]	-1415424	0.0056025535
A_23_P380998	*SERAC1*	Homo sapiens serine active site containing 1 (SERAC1), transcript variant 2, non-coding RNA [NR_073096]	-2171802	0.0059050764
A_23_P97632	*SH2D1A*	Homo sapiens SH2 domain containing 1A (SH2D1A), transcript variant 2, mRNA [NM_001114937]	12362437	0.015447556
A_33_P3227320	*R3HDM1*	Homo sapiens R3H domain containing 1 (R3HDM1), transcript variant 2, mRNA [NM_015361]	-0.56858474	0.015647039
A_33_P3324692	*EPRS*	Homo sapiens glutamyl-prolyl-tRNA synthetase (EPRS), mRNA [NM_004446]	-0.78114176	0.011416787
A_22_P00000129	*MIER3*	Homo sapiens mesoderm induction early response 1, family member 3 (MIER3), transcript variant 1, mRNA [NM_001297598]	-0.8743386	0.00324898
A_21_P0001177	*ALG1L2*	Homo sapiens ALG1, chitobiosyldiphosphodolichol beta-mannosyltransferase-like 2 (ALG1L2), mRNA [NM_001136152]	0.9851699	0.013216448
A_33_P3331451	*RIMKLB*	Homo sapiens ribosomal modification protein rimK-like family member B (RIMKLB), transcript variant 3, non-coding RNA [NR_123740]	-21938438	0.012994969
A_23_P16743	*lnc-FAM72C-2*	PREDICTED: Homo sapiens myomegalin-like (LOC100996723), mRNA [XM_006711090]	-1793638	0.014982433
A_33_P3269000	*TGFBR1*	Homo sapiens transforming growth factor, beta receptor 1 (TGFBR1), transcript variant 1, mRNA [NM_004612]	-14465632	0.01497713
A_24_P942517	*MGAT5*	Homo sapiens mannosyl (alpha-1,6-)-glycoprotein beta-1,6-N-acetyl-glucosaminyltransferase (MGAT5), mRNA [NM_002410]	14297975	0.003956951
A_33_P3220565	*C8orf48*	Homo sapiens chromosome 8 open reading frame 48 (C8orf48), mRNA [NM_001007090]	-10097152	0.002419416
A_23_P33759	*TMX4*	Homo sapiens thioredoxin-related transmembrane protein 4 (TMX4), mRNA [NM_021156]	-0.7277572	0.010839669
A_33_P3397147	*UBTD2*	Homo sapiens ubiquitin domain containing 2 (UBTD2), mRNA [NM_152277]	-0.7472829	0.0040527363
A_33_P3397298	*DHRS3*	Homo sapiens dehydrogenase/reductase (SDR family) member 3 (DHRS3), mRNA [NM_004753]	-25966682	0.0039856653
A_22_P00008038	*FAM168B*	Homo sapiens family with sequence similarity 168, member B (FAM168B), mRNA [NM_001009993]	0.85673213	0.00628389

A_22_P00019757	*IGFBP7*	Homo sapiens insulin-like growth factor binding protein 7 (IGFBP7), transcript variant 2, mRNA [NM_001253835]	-17644298	0.004327546
A_23_P15564	*LINC00597*	Homo sapiens long intergenic non-protein coding RNA 597 (LINC00597), long non-coding RNA [NR_026813]	-12960271	0.012094951
A_21_P0014892	*AMZ2*	Homo sapiens archaelysin family metallopeptidase 2 (AMZ2), transcript variant 1, mRNA [NM_016627]	-0.616781	0.0045764185
A_33_P3445805	*SLC35E2*	Homo sapiens solute carrier family 35, member E2 (SLC35E2), transcript variant 1, mRNA [NM_182838]	-0.6049581	0.014834527
A_23_P349566	*PPP6R3*	Homo sapiens protein phosphatase 6, regulatory subunit 3 (PPP6R3), transcript variant 4, mRNA [NM_001164160]	-1050945	0.005344626
A_23_P28969	*lnc-C5orf42-2*	Homo sapiens cDNA FLJ10039 fis, clone HEMBA1000975. [AK000901]	-11042383	0.008647109
A_32_P97169	*CCDC85A*	Homo sapiens coiled-coil domain containing 85A (CCDC85A), mRNA [NM_001080433]	-37713835	0.0023340243
A_24_P221092	*CHMP4B*	Homo sapiens charged multivesicular body protein 4B (CHMP4B), mRNA [NM_176812]	-0.5372484	0.009788225
A_33_P3256868	*GPC6*	Homo sapiens glypican 6 (GPC6), mRNA [NM_005708]	-42353897	0.01329306
A_32_P104000	*ZNF83*	Homo sapiens zinc finger protein 83 (ZNF83), transcript variant 10, mRNA [NM_001277945]	-0.5282206	0.012304476
A_24_P196499	*DCUN1D3*	Homo sapiens DCN1, defective in cullin neddylation 1, domain containing 3 (DCUN1D3), mRNA [NM_173475]	-1076689	0.0029085083
A_24_P40626	*RBM14-RBM4*	Homo sapiens RBM14-RBM4 readthrough (RBM14-RBM4), transcript variant 1, mRNA [NM_001198845]	-0.6900603	0.005822303
A_33_P3333523	*ARID4B*	AT rich interactive domain 4B (RBP1-like) [Source:HGNC Symbol;Acc:HGNC:15550] [ENST00000471257]	-0.48096925	0.012112936
A_23_P254179	*GREM2*	Homo sapiens gremlin 2, DAN family BMP antagonist (GREM2), mRNA [NM_022469]	-12842326	0.0058047334
A_23_P500206	*ADNP*	Homo sapiens activity-dependent neuroprotector homeobox (ADNP), transcript variant 1, mRNA [NM_015339]	-0.88630533	0.0041946988
A_21_P0002674	*SNORD114-10*	Homo sapiens small nucleolar RNA, C/D box 114-10 (SNORD114-10), small nucleolar RNA [NR_003203]	12282519	0.0069110035
A_33_P3867534	*IL17RE*	Homo sapiens interleukin 17 receptor E (IL17RE), transcript variant 5, mRNA [NM_153483]	0.78398955	0.009765222
A_32_P7316	*FLJ42351*	Homo sapiens uncharacterized LOC400999 (FLJ42351), long non-coding RNA [NR_033871]	-0.9952229	0.0071645253

A_33_P3300395	*MCM10*	Homo sapiens minichromosome maintenance complex component 10 (MCM10), transcript variant 1, mRNA [NM_182751]	0.57599217	0.012529562
A_24_P631848	*BDNF*	Homo sapiens brain-derived neurotrophic factor (BDNF), transcript variant 1, mRNA [NM_170735]	14594024	0.0029471128
A_32_P45493	*APITD1*	Homo sapiens apoptosis-inducing, TAF9-like domain 1 (APITD1), transcript variant A, mRNA [NM_199294]	-0.33879852	0.010237655
A_24_P169343	*SRSF10*	Homo sapiens serine/arginine-rich splicing factor 10 (SRSF10), transcript variant 1, mRNA [NM_006625]	-0.50613856	0.013596905
A_24_P371399	*BRMS1L*	Homo sapiens breast cancer metastasis-suppressor 1-like (BRMS1L), mRNA [NM_032352]	-0.86634	0.011394095
A_23_P66487	*AAED1*	Homo sapiens AhpC/TSA antioxidant enzyme domain containing 1 (AAED1), mRNA [NM_153698]	-0.8643405	0.0055521973
A_33_P3322363	*C3orf58*	Homo sapiens chromosome 3 open reading frame 58 (C3orf58), transcript variant 1, mRNA [NM_173552]	-0.5125185	0.011969343
A_24_P53282	*SMARCD2*	Homo sapiens SWI/SNF related, matrix associated, actin dependent regulator of chromatin, subfamily d, member 2 (SMARCD2), mRNA [NM_001098426]	0.48429483	0.009161951
A_23_P26336	*HMSD*	Homo sapiens histocompatibility (minor) serpin domain containing (HMSD), mRNA [NM_001123366]	-24061728	0.004437345
A_23_P371765	*CPD*	Homo sapiens carboxypeptidase D (CPD), transcript variant 1, mRNA [NM_001304]	-11181357	0.004296912
A_23_P44724	*ZC3H7A*	Homo sapiens zinc finger CCCH-type containing 7A (ZC3H7A), mRNA [NM_014153]	-11756563	0.003190129
A_33_P3233125	*SPATC1L*	Homo sapiens spermatogenesis and centriole associated 1-like (SPATC1L), transcript variant 2, mRNA [NM_032261]	10594801	0.0046744854
A_21_P0007229	*CSRP2*	Homo sapiens cysteine and glycine-rich protein 2 (CSRP2), transcript variant 1, mRNA [NM_001321]	-10606084	0.005377644
A_21_P0003704	*PSD*	Homo sapiens pleckstrin and Sec7 domain containing (PSD), transcript variant 1, mRNA [NM_002779]	12840025	0.011658678
A_33_P3250398	*lnc-FRG1-5*	LNCipedia lincRNA (lnc-FRG1-5), lincRNA [lnc-FRG1-5:3]	11167097	0.012030367
A_24_P58727	*LINC01063*	PREDICTED: Homo sapiens long intergenic non-protein coding RNA 1063 (LINC01063), ncRNA [XR_246052]	-12268708	0.009658549
A_32_P171328	*CTSZ*	cathepsin Z [Source:HGNC Symbol;Acc:HGNC:2547] [ENST00000503833]	-0.7554519	0.011949872

A_23_P2181	*UBE2S*	Homo sapiens ubiquitin-conjugating enzyme E2S (UBE2S), mRNA [NM_014501]	0.38611245	0.008798942
A_23_P20558	*DOK7*	Homo sapiens docking protein 7 (DOK7), transcript variant 1, mRNA [NM_173660]	29560862	0.0028913463
A_32_P142818	*CYB5R2*	Homo sapiens cytochrome b5 reductase 2 (CYB5R2), transcript variant 2, mRNA [NM_016229]	-0.48771003	0.012095497
A_32_P118250	*CDC37L1*	cell division cycle 37-like 1 [Source:HGNC Symbol;Acc:HGNC:17179] [ENST00000381854]	-13442173	0.0053870557
A_23_P48029	*DLX1*	Homo sapiens distal-less homeobox 1 (DLX1), transcript variant 1, mRNA [NM_178120]	142611	0.0043867165
A_22_P00017580	*DHRS4-AS1*	Homo sapiens DHRS4 antisense RNA 1 (DHRS4-AS1), transcript variant 1, long non-coding RNA [NR_023921]	-0.81961274	0.0036517929
A_23_P110243	*CLEC4A*	Homo sapiens C-type lectin domain family 4, member A (CLEC4A), transcript variant 1, mRNA [NM_016184]	-12006001	0.005512336
A_23_P140648	*lnc-WDR67-2*	LNCipedia lincRNA (lnc-WDR67-2), lincRNA [lnc-WDR67-2:1]	-10132791	0.0029691157
A_33_P3424143	*METTL14*	methyltransferase like 14 [Source:HGNC Symbol;Acc:HGNC:29330] [ENST00000388822]	-0.71829516	0.0070605814
A_21_P0001273	*CYFIP1*	Homo sapiens cytoplasmic FMR1 interacting protein 1 (CYFIP1), transcript variant 1, mRNA [NM_014608]	-0.6292066	0.013598395
A_24_P673063	*AREL1*	human full-length cDNA 5-PRIME end of clone CS0CAP004YO05 of Thymus of Homo sapiens (human). [BX248747]	0.49904782	0.015330245
A_24_P398323	*lnc-LDLRAP1-1*	PREDICTED: Homo sapiens low density lipoprotein receptor adaptor protein 1 (LDLRAP1), transcript variant X3, mRNA [XM_006710560]	-0.67904574	0.016549561
A_33_P3883912	*FABP5*	Homo sapiens fatty acid binding protein 5 (psoriasis-associated) (FABP5), mRNA [NM_001444]	-0.35333276	0.014241321
A_23_P121011	*TRIM34*	Homo sapiens tripartite motif containing 34 (TRIM34), transcript variant 4, mRNA [NM_001003827]	-0.64551026	0.008570872
A_23_P58538	*ZCCHC10*	Homo sapiens zinc finger, CCHC domain containing 10 (ZCCHC10), transcript variant 4, mRNA [NM_017665]	-10668142	0.005800329
A_32_P201979	*CSRNP1*	Homo sapiens cysteine-serine-rich nuclear protein 1 (CSRNP1), mRNA [NM_033027]	25833888	0.0035238392
A_21_P0010724	*EPB41L4A-AS1*	Homo sapiens EPB41L4A antisense RNA 1 (EPB41L4A-AS1), long non-coding RNA [NR_015370]	-12795737	0.0030159114
A_23_P93046	*HECTD2*	Homo sapiens HECT domain containing E3 ubiquitin protein ligase 2 (HECTD2), transcript variant 1, mRNA [NM_182765]	-0.9012275	0.0058047334

A_23_P155848	*LOC645166*	Homo sapiens lymphocyte-specific protein 1 pseudogene (LOC645166), transcript variant 1, non-coding RNA [NR_027354]	-10539229	0.009881715
A_23_P70748	*EXOC3*	Homo sapiens exocyst complex component 3 (EXOC3), mRNA [NM_007277]	-0.33100984	0.015448156
A_23_P409541	*DKK2*	Homo sapiens dickkopf WNT signaling pathway inhibitor 2 (DKK2), mRNA [NM_014421]	23277316	0.004026994
A_23_P24077	*REPS1*	Homo sapiens RALBP1 associated Eps domain containing 1 (REPS1), transcript variant 1, mRNA [NM_031922]	-0.54054195	0.0117895575
A_23_P43779	*POLR1D*	Homo sapiens polymerase (RNA) I polypeptide D, 16kDa (POLR1D), transcript variant 2, mRNA [NM_152705]	-0.6816611	0.01526397
A_32_P177097	*C10orf54*	Homo sapiens chromosome 10 open reading frame 54 (C10orf54), mRNA [NM_022153]	-22620494	0.011667079
A_23_P259272	*COPS4*	Homo sapiens COP9 signalosome subunit 4 (COPS4), transcript variant 1, mRNA [NM_016129]	-0.9544296	0.008153665
A_33_P3321130	*ZNF557*	Homo sapiens zinc finger protein 557 (ZNF557), transcript variant 1, mRNA [NM_024341]	-0.4711304	0.013071153
A_23_P74716	*WSB2*	Homo sapiens WD repeat and SOCS box containing 2 (WSB2), transcript variant 2, mRNA [NM_018639]	-10746088	0.006812377
A_23_P23102	*DENND3*	DENN/MADD domain containing 3 [Source:HGNC Symbol;Acc:HGNC:29134] [ENST00000520482]	-0.65102154	0.014260212
A_23_P312718	*DEDD*	Homo sapiens death effector domain containing (DEDD), transcript variant 1, mRNA [NM_032998]	-0.7055185	0.0150995245
A_23_P60565	*ZSCAN20*	Homo sapiens zinc finger and SCAN domain containing 20 (ZSCAN20), mRNA [NM_145238]	-0.8726451	0.006513625
A_23_P55706	*PNPLA8*	Homo sapiens patatin-like phospholipase domain containing 8 (PNPLA8), transcript variant 1, mRNA [NM_015723]	-0.77286834	0.009924993
A_22_P00019795	*ZNF354A*	Homo sapiens zinc finger protein 354A (ZNF354A), mRNA [NM_005649]	-0.3659287	0.014104453
A_24_P228717	*RELB*	Homo sapiens v-rel avian reticuloendotheliosis viral oncogene homolog B (RELB), mRNA [NM_006509]	0.6222987	0.0036240483
A_19_P00811196	*RAC2*	Homo sapiens ras-related C3 botulinum toxin substrate 2 (rho family, small GTP binding protein Rac2) (RAC2), mRNA [NM_002872]	16783133	0.009296599
A_33_P3413732	*SIX4*	Homo sapiens SIX homeobox 4 (SIX4), mRNA [NM_017420]	0.76791507	0.012873199
A_33_P3736195	*APTR*	Homo sapiens Alu-mediated CDKN1A/p21 transcriptional regulator (non-protein coding) (APTR), long non-coding RNA [NR_038361]	-0.7839937	0.010138355

A_23_P398491	*DKFZp686M1136*	Homo sapiens mRNA; cDNA DKFZp686M1136 (from clone DKFZp686M1136). [BX647230]	-0.8334663	0.013286371
A_19_P00319099	*N4BP2*	Homo sapiens NEDD4 binding protein 2 (N4BP2), mRNA [NM_018177]	-0.6016909	0.01232594
A_21_P0000414	*C15orf57*	Homo sapiens chromosome 15 open reading frame 57 (C15orf57), transcript variant 4, mRNA [NM_001289132]	-24208078	0.0071601756
A_23_P389118	*lnc-RAD23B-2*	LNCipedia lincRNA (lnc-RAD23B-2), lincRNA [lnc-RAD23B-2:1]	0.7704871	0.007745343
A_23_P413193	*SNORD114-17*	Homo sapiens small nucleolar RNA, C/D box 114-17 (SNORD114-17), small nucleolar RNA [NR_003210]	11519417	0.004220053
A_23_P360964	*ANO6*	Homo sapiens anoctamin 6 (ANO6), transcript variant 1, mRNA [NM_001025356]	-0.9847183	0.008539769
A_33_P3328426	*SFR1*	Homo sapiens SWI5-dependent recombination repair 1 (SFR1), transcript variant 2, mRNA [NM_145247]	0.38844776	0.009433551
A_32_P129419	*DACT3*	Homo sapiens dishevelled-binding antagonist of beta-catenin 3 (DACT3), transcript variant 1, mRNA [NM_145056]	-0.97539353	0.013456115
A_23_P13885	*ANO10*	Homo sapiens anoctamin 10 (ANO10), transcript variant 2, mRNA [NM_001204831]	-12302401	0.015268567
A_33_P3308481	*ICE2*	Homo sapiens interactor of little elongation complex ELL subunit 2 (ICE2), transcript variant 1, mRNA [NM_024611]	-15979922	0.015403497
A_33_P3418611	*ATN1*	Homo sapiens atrophin 1 (ATN1), transcript variant 1, mRNA [NM_001007026]	13423839	0.005057235
A_33_P3334185	*HYMAI*	Homo sapiens hydatidiform mole associated and imprinted (non-protein coding) (HYMAI), long non-coding RNA [NR_002768]	-0.79590154	0.013209052
A_33_P3390102	*ZNF729*	Homo sapiens zinc finger protein 729 (ZNF729), mRNA [NM_001242680]	0.3832948	0.0075145615
A_24_P943957	*AGTPBP1*	Homo sapiens ATP/GTP binding protein 1 (AGTPBP1), transcript variant 2, mRNA [NM_015239]	-0.51654524	0.005872996
A_24_P320759	*GIPC1*	Homo sapiens GIPC PDZ domain containing family, member 1 (GIPC1), transcript variant 1, mRNA [NM_005716]	-0.52761143	0.013159623
A_33_P3257165	*PIKFYVE*	Homo sapiens phosphoinositide kinase, FYVE finger containing (PIKFYVE), transcript variant 2, mRNA [NM_015040]	-0.938025	0.0050253686
A_24_P302998	*PRKRIP1*	Homo sapiens PRKR interacting protein 1 (IL11 inducible) (PRKRIP1), mRNA [NM_024653]	-0.66068196	0.010735533
A_24_P239606	*YPEL1*	Homo sapiens yippee-like 1 (Drosophila) (YPEL1), mRNA [NM_013313]	-12748063	0.013367701
A_24_P658427	*ATP5I*	Homo sapiens ATP synthase, H+ transporting, mitochondrial Fo complex, subunit E (ATP5I), transcript variant 1, mRNA [NM_007100]	-0.72572094	0.00557203

A_33_P3293798	*GADD45B*	Homo sapiens growth arrest and DNA-damage-inducible, beta (GADD45B), mRNA [NM_015675]	-16892327	0.0034964236
A_33_P3418917	*NFIB*	Homo sapiens nuclear factor I/B (NFIB), transcript variant 3, mRNA [NM_005596]	-26713307	0.0044240346
A_19_P00806707	*KRTAP10-1*	Homo sapiens keratin associated protein 10-1 (KRTAP10-1), mRNA [NM_198691]	0.5840738	0.0076440917
A_32_P24709	*RUSC1-AS1*	Homo sapiens RUSC1 antisense RNA 1 (RUSC1-AS1), mRNA [NM_001039517]	-0.61361414	0.00854209
A_24_P75718	*XLOC_l2_014579*	BROAD Institute lincRNA (XLOC_l2_014579), lincRNA [TCONS_l2_00028290]	11629045	0.01208643
A_24_P64039	*ZFP69*	Homo sapiens ZFP69 zinc finger protein (ZFP69), mRNA [NM_198494]	-10222982	0.013819061
A_24_P232365	*AP3M2*	Homo sapiens adaptor-related protein complex 3, mu 2 subunit (AP3M2), transcript variant 2, mRNA [NM_006803]	-0.78429914	0.0046919836
A_33_P3286536	*lnc-WDR1-1*	Homo sapiens mRNA; cDNA DKFZp667E0512 (from clone DKFZp667E0512). [AL713660]	-12553049	0.005612162
A_33_P3386723	*APBB1IP*	Homo sapiens amyloid beta (A4) precursor protein-binding, family B, member 1 interacting protein (APBB1IP), mRNA [NM_019043]	-2930255	0.0043220706
A_24_P191971	*FUT4*	Homo sapiens fucosyltransferase 4 (alpha (1,3) fucosyltransferase, myeloid-specific) (FUT4), mRNA [NM_002033]	-0.5441229	0.012479078
A_33_P3299977	*SAP30L*	Homo sapiens SAP30-like (SAP30L), transcript variant 1, mRNA [NM_024632]	-0.94026744	0.013753385
A_23_P354074	*TRIM2*	Homo sapiens tripartite motif containing 2 (TRIM2), transcript variant 1, mRNA [NM_015271]	-0.74097633	0.008208044
A_33_P3210848	*NAA35*	Homo sapiens N(alpha)-acetyltransferase 35, NatC auxiliary subunit (NAA35), mRNA [NM_024635]	-0.8881673	0.0034766807
A_22_P00001137	*LYST*	Homo sapiens lysosomal trafficking regulator (LYST), transcript variant 1, mRNA [NM_000081]	-12541726	0.0032831472
A_24_P356406	*ELFN1*	Homo sapiens extracellular leucine-rich repeat and fibronectin type III domain containing 1 (ELFN1), mRNA [NM_001128636]	-13519318	0.012219373
A_23_P250002	*HNRNPKP3*	Homo sapiens heterogeneous nuclear ribonucleoprotein K pseudogene 3 (HNRNPKP3), non-coding RNA [NR_033868]	-0.5257023	0.01590382
A_32_P108655	*PAFAH1B1*	Homo sapiens platelet-activating factor acetylhydrolase 1b, regulatory subunit 1 (45kDa) (PAFAH1B1), mRNA [NM_000430]	-0.58347267	0.0088629555
A_22_P00004066	*HACE1*	Homo sapiens HECT domain and ankyrin repeat containing E3 ubiquitin protein ligase 1	-11421756	0.0031474202

		(HACE1), transcript variant 1, mRNA [NM_020771]		
A_24_P945147	*AK4*	Homo sapiens adenylate kinase 4 (AK4), transcript variant 1, mRNA [NM_001005353]	-21037745	0.0024890031
A_33_P3305571	*lnc-CHSY1-5*	PREDICTED: Homo sapiens uncharacterized LOC101927751 (LOC101927751), mRNA [XM_005255009]	-10064304	0.011568712
A_24_P360763	*RABEP1*	Homo sapiens rabaptin, RAB GTPase binding effector protein 1 (RABEP1), transcript variant 1, mRNA [NM_004703]	-0.92279804	0.011604218
A_23_P250274	*TNFRSF6B*	Homo sapiens tumor necrosis factor receptor superfamily, member 6b, decoy (TNFRSF6B), mRNA [NM_003823]	13279948	0.010986003
A_23_P387071	*CREM*	Homo sapiens cAMP responsive element modulator (CREM), transcript variant 2, mRNA [NM_001881]	-0.7812479	0.0122815035
A_19_P00321361	*LRRC8A*	Homo sapiens leucine rich repeat containing 8 family, member A (LRRC8A), transcript variant 2, mRNA [NM_019594]	-0.64547205	0.013828529
A_23_P69586	*ZNF830*	Homo sapiens zinc finger protein 830 (ZNF830), mRNA [NM_052857]	-0.90181756	0.009901111
A_24_P126741	*LINC-PINT*	Homo sapiens long intergenic non-protein coding RNA, p53 induced transcript (LINC-PINT), transcript variant 4, long non-coding RNA [NR_109851]	-0.97824556	0.011355202
A_23_P309803	*FAT1*	Homo sapiens FAT atypical cadherin 1 (FAT1), mRNA [NM_005245]	-0.82293814	0.015620706
A_23_P356554	*ZNF777*	Homo sapiens zinc finger protein 777 (ZNF777), mRNA [NM_015694]	-0.5763893	0.008539769
A_23_P74981	*ZNF562*	Homo sapiens zinc finger protein 562 (ZNF562), transcript variant 3, mRNA [NM_017656]	-0.2674155	0.0031941293
A_23_P132595	*BAG2*	Homo sapiens BCL2-associated athanogene 2 (BAG2), mRNA [NM_004282]	-0.5742514	0.0072648157
A_23_P41114	*ZNF670*	Homo sapiens zinc finger protein 670 (ZNF670), transcript variant 1, mRNA [NM_033213]	-0.44576293	0.009811661
A_24_P106681	*VGLL4*	Homo sapiens vestigial-like family member 4 (VGLL4), transcript variant 2, mRNA [NM_014667]	-11621507	0.006814677
A_32_P17635	*CSTA*	Homo sapiens cystatin A (stefin A) (CSTA), mRNA [NM_005213]	19707093	0.00422039
A_24_P787897	*YWHAG*	Homo sapiens tyrosine 3-monooxygenase/tryptophan 5-monooxygenase activation protein, gamma (YWHAG), mRNA [NM_012479]	-0.41390175	0.011821381

A_33_P3306153	*SRSF8*	Homo sapiens serine/arginine-rich splicing factor 8 (SRSF8), transcript variant 1, mRNA [NM_032102]	-0.54387707	0.0039497027
A_21_P0011721	*XYLT1*	Homo sapiens xylosyltransferase I (XYLT1), mRNA [NM_022166]	-0.6857603	0.008956738
A_33_P3404697	*KIAA1841*	Homo sapiens KIAA1841 (KIAA1841), transcript variant 2, mRNA [NM_032506]	-0.78828746	0.0050508454
A_22_P00001553	*PSTK*	Homo sapiens phosphoseryl-tRNA kinase (PSTK), mRNA [NM_153336]	-0.72931683	0.01147035
A_24_P412156	*OPA1*	Homo sapiens optic atrophy 1 (autosomal dominant) (OPA1), transcript variant 8, mRNA [NM_130837]	-0.45902705	0.011054281
A_24_P402415	*lnc-ARID2-1*	Homo sapiens cDNA clone IMAGE:5303182. [BC039429]	0.707229	0.0129454555
A_23_P380181	*CXCL12*	Homo sapiens chemokine (C-X-C motif) ligand 12 (CXCL12), transcript variant 2, mRNA [NM_000609]	-0.96499896	0.0072350414
A_23_P79518	*SYT14*	Homo sapiens synaptotagmin XIV (SYT14), transcript variant 4, mRNA [NM_153262]	16894562	0.0060495283
A_21_P0012050	*LMO4*	Homo sapiens LIM domain only 4 (LMO4), mRNA [NM_006769]	-0.81993383	0.016076444
A_21_P0000785	*IL1B*	Homo sapiens interleukin 1, beta (IL1B), mRNA [NM_000576]	38742456	0.0024890031
A_21_P0000525	*DNM3OS*	Homo sapiens DNM3 opposite strand/antisense RNA (DNM3OS), transcript variant 1, long non-coding RNA [NR_038397]	-1186818	0.002819256
A_23_P143817	*RPL39*	Homo sapiens ribosomal protein L39 (RPL39), mRNA [NM_001000]	-0.24720377	0.015953625
A_33_P3414487	*LOC285847*	Homo sapiens uncharacterized LOC285847 (LOC285847), long non-coding RNA [NR_027117]	0.6227889	0.010369088
A_21_P0012880	*MYLK*	Homo sapiens myosin light chain kinase (MYLK), transcript variant 1, mRNA [NM_053025]	-1447845	0.004896935
A_33_P3276519	*WBSCR22*	Homo sapiens Williams Beuren syndrome chromosome region 22 (WBSCR22), transcript variant 1, mRNA [NM_001202560]	-0.71860236	0.010237655
A_21_P0014670	*BRD9*	bromodomain containing 9 [Source:HGNC Symbol;Acc:HGNC:25818] [ENST00000493082]	10750501	0.008216877
A_32_P14894	*MLST8*	Homo sapiens MTOR associated protein, LST8 homolog (S. cerevisiae) (MLST8), transcript variant 1, mRNA [NM_022372]	13930671	0.007797447
A_23_P38219	*RPS10*	Homo sapiens ribosomal protein S10 (RPS10), transcript variant 2, mRNA [NM_001014]	-0.3661442	0.010730268
A_23_P79816	*DEF6*	Homo sapiens differentially expressed in FDCP 6 homolog (mouse) (DEF6), mRNA [NM_022047]	16866031	0.016005075

A_33_P3347320	*PRPF8*	Homo sapiens pre-mRNA processing factor 8 (PRPF8), mRNA [NM_006445]	0.8003843	0.017113892
A_32_P75094	*EMILIN3*	Homo sapiens elastin microfibril interfacer 3 (EMILIN3), mRNA [NM_052846]	0.81977147	0.0059911786
A_24_P381199	*ADIRF-AS1*	Homo sapiens, clone IMAGE:3913679, mRNA. [BC017676]	18137193	0.005428736
A_33_P3358037	*AIFM2*	Homo sapiens apoptosis-inducing factor, mitochondrion-associated, 2 (AIFM2), transcript variant 2, mRNA [NM_032797]	-0.88507414	0.005595418
A_19_P00803685	*TRIM6*	Homo sapiens tripartite motif containing 6 (TRIM6), transcript variant 1, mRNA [NM_001003818]	-0.5880349	0.011464132
A_24_P395621	*ANKRD33B*	Homo sapiens ankyrin repeat domain 33B (ANKRD33B), mRNA [NM_001164440]	-18920631	0.0028288076
A_23_P143906	*TRIM22*	Homo sapiens tripartite motif containing 22 (TRIM22), transcript variant 1, mRNA [NM_006074]	-12864177	0.0039416323
A_23_P131825	*RUNDC1*	Homo sapiens RUN domain containing 1 (RUNDC1), mRNA [NM_173079]	-0.899611	0.005512448
A_33_P3353190	*MLF1*	Homo sapiens myeloid leukemia factor 1 (MLF1), transcript variant 1, mRNA [NM_022443]	-17711033	0.0034400926
A_23_P52986	*TNNC2*	Homo sapiens troponin C type 2 (fast) (TNNC2), mRNA [NM_003279]	-0.87029743	0.007502627
A_32_P115505	*LOC101930611*	PREDICTED: Homo sapiens uncharacterized LOC101930611 (LOC101930611), ncRNA [XR_250546]	-27214558	0.0034156272
A_24_P261567	*VWCE*	Homo sapiens von Willebrand factor C and EGF domains (VWCE), mRNA [NM_152718]	-30960584	0.0029691157
A_33_P3239759	*LTN1*	Homo sapiens listerin E3 ubiquitin protein ligase 1 (LTN1), mRNA [NM_015565]	-0.5118167	0.011558667
A_33_P3271711	*GDPD5*	Homo sapiens glycerophosphodiester phosphodiesterase domain containing 5 (GDPD5), mRNA [NM_030792]	-15896659	0.005182173
A_33_P3240843	*PPAN-P2RY11*	Homo sapiens PPAN-P2RY11 readthrough (PPAN-P2RY11), transcript variant 2, mRNA [NM_001198690]	17197326	0.007050734
A_24_P328492	*TMEM71*	Homo sapiens transmembrane protein 71 (TMEM71), transcript variant 1, mRNA [NM_144649]	0.65701175	0.0067479513
A_33_P3301559	*CCDC176*	Homo sapiens coiled-coil domain containing 176 (CCDC176), mRNA [NM_025057]	-18149539	0.007380132
A_24_P73943	*SOCS5*	Homo sapiens suppressor of cytokine signaling 5 (SOCS5), transcript variant 2, mRNA [NM_144949]	-11604923	0.005894816
A_33_P3282693	*CLUAP1*	Homo sapiens clusterin associated protein 1 (CLUAP1), transcript variant 1, mRNA [NM_015041]	-0.9150457	0.014780859

A_22_P00011692	*COX11*	Homo sapiens COX11 cytochrome c oxidase copper chaperone (COX11), transcript variant 1, mRNA [NM_004375]	-0.8607971	0.013618745
A_23_P421423	*OR3A4P*	Homo sapiens olfactory receptor, family 3, subfamily A, member 4 pseudogene (OR3A4P), non-coding RNA [NR_024128]	0.8637472	0.012252959
A_33_P3423830	*lnc-PDHX-1*	Homo sapiens full length insert cDNA clone ZE10H04. [AF086543]	1440942	0.005701378
A_24_P22079	*TNFAIP2*	Homo sapiens tumor necrosis factor, alpha-induced protein 2 (TNFAIP2), mRNA [NM_006291]	1024672	0.011458563
A_33_P3389113	*TCAF1*	Homo sapiens family with sequence similarity 115, member A (FAM115A), transcript variant 1, mRNA [NM_014719]	16037985	0.010505919
A_33_P3393106	*FOXO1*	Homo sapiens forkhead box O1 (FOXO1), mRNA [NM_002015]	-20429182	0.0033094941
A_33_P3238166	*TRAF3IP2-AS1*	Homo sapiens TRAF3IP2 antisense RNA 1 (TRAF3IP2-AS1), transcript variant 1, long non-coding RNA [NR_034108]	-17594767	0.008897745
A_23_P115573	*FAM188A*	Homo sapiens family with sequence similarity 188, member A (FAM188A), mRNA [NM_024948]	-11809976	0.010640448
A_33_P3297399	*PXDN*	Homo sapiens peroxidasin (PXDN), mRNA [NM_012293]	-0.3620246	0.005831737
A_23_P3849	*SHISA4*	Homo sapiens shisa family member 4 (SHISA4), transcript variant 1, mRNA [NM_198149]	-13857393	0.007323471
A_23_P91390	*KCTD9*	Homo sapiens potassium channel tetramerization domain containing 9 (KCTD9), mRNA [NM_017634]	-0.8374309	0.005325502
A_23_P112554	*TRAP1*	Homo sapiens TNF receptor-associated protein 1 (TRAP1), transcript variant 1, mRNA [NM_016292]	10089417	0.012368307
A_23_P370544	*THBD*	Homo sapiens thrombomodulin (THBD), mRNA [NM_000361]	-1459032	0.003392081
A_24_P223604	*COL15A1*	Homo sapiens collagen, type XV, alpha 1 (COL15A1), mRNA [NM_001855]	14909384	0.0042201984
A_33_P3246068	*ANKAR*	Homo sapiens ankyrin and armadillo repeat containing (ANKAR), mRNA [NM_144708]	-1371151	0.006155321
A_22_P00019844	*KIAA1429*	Homo sapiens KIAA1429 (KIAA1429), transcript variant 1, mRNA [NM_015496]	-11043103	0.005787274
A_33_P3235189	*LAMTOR2*	Homo sapiens late endosomal/lysosomal adaptor, MAPK and MTOR activator 2 (LAMTOR2), transcript variant 1, mRNA [NM_014017]	-10578673	0.0026049376
A_21_P0014865	*lnc-KLRG2-1*	Homo sapiens mRNA; cDNA DKFZp451B0818 (from clone DKFZp451B0818). [AL832624]	-0.86481124	0.01696804
A_33_P3243175	*ANKRD13B*	Homo sapiens ankyrin repeat domain 13B (ANKRD13B), mRNA [NM_152345]	0.6125683	0.004055852

A_23_P125717	*LOC100288160*	Homo sapiens esophagus cancer-related gene-2 interaction susceptibility protein mRNA, partial cds. [AF422192]	14103708	0.007696588
A_23_P434301	*PNPO*	Homo sapiens pyridoxamine 5'-phosphate oxidase (PNPO), mRNA [NM_018129]	-0.8965485	0.0140300365
A_33_P3392213	*NAP1L3*	Homo sapiens nucleosome assembly protein 1-like 3 (NAP1L3), mRNA [NM_004538]	-16201354	0.009406834
A_24_P941441	*PTMA*	Homo sapiens prothymosin, alpha (PTMA), transcript variant 2, mRNA [NM_002823]	0.2267895	0.0038302224
A_24_P734953	*GNA13*	Homo sapiens guanine nucleotide binding protein (G protein), alpha 13 (GNA13), transcript variant 1, mRNA [NM_006572]	-0.6867509	0.005377644
A_21_P0000607	*ISG20*	Homo sapiens interferon stimulated exonuclease gene 20kDa (ISG20), transcript variant 1, mRNA [NM_002201]	0.6687527	0.012274017
A_33_P3368636	*TRNP1*	Homo sapiens TMF1-regulated nuclear protein 1 (TRNP1), mRNA [NM_001013642]	0.93622637	0.008787238
A_22_P00002639	*ST3GAL4-AS1*	Homo sapiens ST3GAL4 antisense RNA 1 (head to head) (ST3GAL4-AS1), long non-coding RNA [NR_033839]	-10781778	0.011969343
A_23_P137035	*lnc-C1orf185-2*	LNCipedia lincRNA (lnc-C1orf185-2), lincRNA [lnc-C1orf185-2:1]	-0.8480729	0.012866833
A_33_P3337896	*CYBRD1*	Homo sapiens cytochrome b reductase 1 (CYBRD1), transcript variant 1, mRNA [NM_024843]	-10324143	0.012093684
A_23_P148556	*PIR*	Homo sapiens pirin (iron-binding nuclear protein) (PIR), transcript variant 1, mRNA [NM_003662]	0.74070853	0.0095736785
A_23_P31116	*ABCD1*	Homo sapiens ATP-binding cassette, sub-family D (ALD), member 1 (ABCD1), mRNA [NM_000033]	11091651	0.009820808
A_24_P264909	*PI16*	Homo sapiens peptidase inhibitor 16 (PI16), transcript variant 1, mRNA [NM_153370]	-0.83536625	0.0157461
A_24_P242609	*ACOT13*	Homo sapiens acyl-CoA thioesterase 13 (ACOT13), transcript variant 1, mRNA [NM_018473]	-10791531	0.0066119307
A_23_P415827	*NDFIP2*	Homo sapiens Nedd4 family interacting protein 2 (NDFIP2), transcript variant 1, mRNA [NM_019080]	-0.9388993	0.005842833
A_33_P3298105	*KLHL12*	Homo sapiens kelch-like family member 12 (KLHL12), transcript variant 2, mRNA [NM_021633]	-11940885	0.00652649
A_24_P200848	*SPATA13*	Homo sapiens spermatogenesis associated 13 (SPATA13), transcript variant 3, mRNA [NM_001286792]	16379623	0.0074182227
A_33_P3213992	*RTP4*	Homo sapiens receptor (chemosensory) transporter protein 4 (RTP4), mRNA [NM_022147]	-0.52343726	0.011087365

A_33_P3286774	*SNX13*	Homo sapiens sorting nexin 13 (SNX13), mRNA [NM_015132]	-12790867	0.00910823
A_33_P3354021	*MIIP*	Homo sapiens migration and invasion inhibitory protein (MIIP), mRNA [NM_021933]	-10110109	0.0050497777
A_33_P3236177	*lnc-SLC35A5-1*	ALU1_HUMAN (P39188) Alu subfamily J sequence contamination warning entry, partial (13%) [THC2669783]	-18668602	0.0063660103
A_32_P198923	*RPL23AP53*	Homo sapiens ribosomal protein L23a pseudogene 53 (RPL23AP53), non-coding RNA [NR_003572]	-0.718833	0.010920335
A_33_P3238479	*ANG*	Homo sapiens angiogenin, ribonuclease, RNase A family, 5 (ANG), transcript variant 1, mRNA [NM_001145]	-12841125	0.01273111
A_24_P373152	*YWHAZ*	Homo sapiens tyrosine 3-monooxygenase/tryptophan 5-monooxygenase activation protein, zeta (YWHAZ), transcript variant 2, mRNA [NM_145690]	-0.712945	0.007111782
A_24_P365807	*CFL2*	Homo sapiens cofilin 2 (muscle) (CFL2), transcript variant 1, mRNA [NM_021914]	-16584975	0.0024890031
A_33_P3269740	*MEGF8*	Homo sapiens multiple EGF-like-domains 8 (MEGF8), transcript variant 1, mRNA [NM_001271938]	-0.56580925	0.014051499
A_23_P215454	*EFNB1*	Homo sapiens ephrin-B1 (EFNB1), mRNA [NM_004429]	-13256786	0.005581195
A_24_P255314	*FAM86B2*	Homo sapiens family with sequence similarity 86, member B2 (FAM86B2), mRNA [NM_001137610]	-0.5229621	0.012742897
A_23_P51202	*ELN*	Homo sapiens elastin (ELN), transcript variant 13, mRNA [NM_001278939]	-202819	0.009259553
A_23_P66402	*ZNF436*	Homo sapiens zinc finger protein 436 (ZNF436), transcript variant 1, mRNA [NM_001077195]	-0.90011996	0.006520828
A_33_P3292896	*lnc-TUBA1C-1*	zr02f09.s1 Stratagene NT2 neuronal precursor 937230 Homo sapiens cDNA clone IMAGE:650345 3' similar to gb:K00558 TUBULIN ALPHA-1 CHAIN (HUMAN);, mRNA sequence [AA218968]	16422484	0.006048972
A_23_P18604	*MED9*	Homo sapiens mediator complex subunit 9 (MED9), mRNA [NM_018019]	-0.71515274	0.011376762
A_32_P26376	*SFXN5*	Homo sapiens sideroflexin 5 (SFXN5), mRNA [NM_144579]	1212464	0.0035939496
A_21_P0013292	*LAP3*	Homo sapiens leucine aminopeptidase 3 (LAP3), mRNA [NM_015907]	-0.9489414	0.0024890031
A_23_P115919	*POGLUT1*	Homo sapiens protein O-glucosyltransferase 1 (POGLUT1), transcript variant 1, mRNA [NM_152305]	-0.60296375	0.011499655

A_22_P00002996	*EIF4H*	Homo sapiens eukaryotic translation initiation factor 4H (EIF4H), transcript variant 1, mRNA [NM_022170]	0.28542084	0.005313822
A_33_P3213377	*PHYH*	Homo sapiens phytanoyl-CoA 2-hydroxylase (PHYH), transcript variant 2, mRNA [NM_001037537]	-0.7144811	0.0050054174
A_24_P941625	*lnc-C6orf221-2*	Homo sapiens cDNA clone IMAGE:4819744. [BC031876]	-0.40692717	0.009332055
A_33_P3421827	*IMMP2L*	Homo sapiens IMP2 inner mitochondrial membrane peptidase-like (S. cerevisiae) (IMMP2L), transcript variant 1, mRNA [NM_032549]	-13489422	0.0055521973
A_33_P3280875	*ZNF70*	Homo sapiens zinc finger protein 70 (ZNF70), mRNA [NM_021916]	-0.6912285	0.009104556
A_32_P43465	*HBZ*	Homo sapiens hemoglobin, zeta (HBZ), mRNA [NM_005332]	0.69149053	0.015603284
A_23_P145134	*IVNS1ABP*	Homo sapiens influenza virus NS1A binding protein (IVNS1ABP), mRNA [NM_006469]	0.8991604	0.013367701
A_21_P0009305	*ZNF182*	Homo sapiens zinc finger protein 182 (ZNF182), transcript variant 1, mRNA [NM_006962]	-11869849	0.003956951
A_33_P3213997	*FGFR1OP*	Homo sapiens FGFR1 oncogene partner (FGFR1OP), transcript variant 1, mRNA [NM_007045]	-0.6732202	0.003609745
A_24_P382489	*lnc-MFAP4-4*	LNCipedia lincRNA (lnc-MFAP4-4), lincRNA [lnc-MFAP4-4:1]	0.5297289	0.010495637
A_21_P0010997	*DHRS4L2*	Homo sapiens dehydrogenase/reductase (SDR family) member 4 like 2 (DHRS4L2), transcript variant 3, mRNA [NM_001193636]	-0.7109825	0.0037353234
A_23_P22672	*SLC27A1*	solute carrier family 27 (fatty acid transporter), member 1 [Source:HGNC Symbol;Acc:HGNC:10995] [ENST00000594962]	-21702514	0.0031941293
A_22_P00023561	*LOC283140*		-15893469	0.005514184
A_23_P153022	*ALG13*	Homo sapiens ALG13, UDP-N-acetylglucosaminyltransferase subunit (ALG13), transcript variant 7, mRNA [NM_001257231]	-10129392	0.0053870557
A_24_P320254	*KRTAP2-4*	Homo sapiens keratin associated protein 2-4 (KRTAP2-4), mRNA [NM_033184]	11115417	0.009792738
A_33_P3417313	*JAG1*	Homo sapiens jagged 1 (JAG1), mRNA [NM_000214]	-29432387	0.009713893
A_23_P91221	*HNRNPH1*	Homo sapiens heterogeneous nuclear ribonucleoprotein H1 (H) (HNRNPH1), transcript variant 2, mRNA [NM_005520]	-0.1765098	0.010474825
A_24_P54174	*PYCRL*	Homo sapiens pyrroline-5-carboxylate reductase-like (PYCRL), mRNA [NM_023078]	1371048	0.007794005

A_23_P133596	*PKIG*	Homo sapiens protein kinase (cAMP-dependent, catalytic) inhibitor gamma (PKIG), transcript variant 1, mRNA [NM_181805]	-17174628	0.003121324
A_22_P00015994	*TNFRSF1B*	Homo sapiens tumor necrosis factor receptor superfamily, member 1B (TNFRSF1B), mRNA [NM_001066]	1797205	0.0034766807
A_33_P6812270	*DROSHA*	Homo sapiens drosha, ribonuclease type III (DROSHA), transcript variant 1, mRNA [NM_013235]	-11645589	0.005348777
A_21_P0014898	*ATP6V1B1-AS1*	Homo sapiens ATP6V1B1 antisense RNA 1 (ATP6V1B1-AS1), transcript variant 2, long non-coding RNA [NR_110274]	14529595	0.0034903844
A_23_P30363	*LOC100506844*	Homo sapiens uncharacterized LOC100506844 (LOC100506844), long non-coding RNA [NR_038269]	-0.97429866	0.009582734
A_24_P944588	*P4HA2*	Homo sapiens prolyl 4-hydroxylase, alpha polypeptide II (P4HA2), transcript variant 1, mRNA [NM_004199]	-0.9584029	0.0055521973
A_23_P59397	*KIF23*	Homo sapiens kinesin family member 23 (KIF23), transcript variant 1, mRNA [NM_138555]	-0.390786	0.0069863
A_33_P3222917	*ZNF682*	Homo sapiens zinc finger protein 682 (ZNF682), transcript variant 1, mRNA [NM_033196]	-0.64510953	0.009516893
A_33_P3221284	*RSPH3*	Homo sapiens radial spoke 3 homolog (Chlamydomonas) (RSPH3), mRNA [NM_031924]	-1157331	0.0041063502
A_33_P3277965	*CD276*	Homo sapiens CD276 molecule (CD276), transcript variant 1, mRNA [NM_001024736]	-11420703	0.0029471128
A_33_P3319596	*CCDC80*	Homo sapiens coiled-coil domain containing 80 (CCDC80), transcript variant 1, mRNA [NM_199511]	-2137158	0.0064965826
A_23_P35066	*NFIC*	Homo sapiens nuclear factor I/C (CCAAT-binding transcription factor) (NFIC), transcript variant 2, mRNA [NM_205843]	0.6287896	0.011870703
A_23_P416191	*TANGO2*	Homo sapiens transport and golgi organization 2 homolog (Drosophila) (TANGO2), transcript variant 1, mRNA [NM_001283106]	-10109086	0.0059872055
A_23_P116264	*SNX7*	Homo sapiens sorting nexin 7 (SNX7), transcript variant 1, mRNA [NM_015976]	-14273313	0.0034707354
A_23_P132417	*TAS2R31*	Homo sapiens taste receptor, type 2, member 31 (TAS2R31), mRNA [NM_176885]	-0.68670356	0.010891201
A_33_P3362611	*NRGN*	Homo sapiens neurogranin (protein kinase C substrate, RC3) (NRGN), transcript variant 1, mRNA [NM_006176]	12041626	0.011324697
A_23_P109133	*LSG1*	Homo sapiens large 60S subunit nuclear export GTPase 1 (LSG1), mRNA [NM_018385]	-0.45234895	0.008346524

A_22_P00008004	*ELTD1*	Homo sapiens EGF, latrophilin and seven transmembrane domain containing 1 (ELTD1), mRNA [NM_022159]	17701122	0.003578794
A_33_P3264121	*AVP*	Homo sapiens arginine vasopressin (AVP), mRNA [NM_000490]	0.9078752	0.009172592
A_23_P38254	*MIR31HG*	Homo sapiens MIR31 host gene (non-protein coding) (MIR31HG), long non-coding RNA [NR_027054]	0.72875744	0.013946563
A_23_P96165	*TBCA*	Homo sapiens tubulin folding cofactor A (TBCA), transcript variant 2, mRNA [NM_004607]	-0.3499086	0.00800842
A_33_P3412975	*ELAC2*	Homo sapiens elaC ribonuclease Z 2 (ELAC2), transcript variant 1, mRNA [NM_018127]	-0.662303	0.008639274
A_24_P312692	*C11orf80*	Homo sapiens chromosome 11 open reading frame 80 (C11orf80), transcript variant 1, mRNA [NM_024650]	13732932	0.0073443917
A_23_P158925	*SIX5*	Homo sapiens SIX homeobox 5 (SIX5), mRNA [NM_175875]	-11188921	0.009761497
A_22_P00005087	*API5*	Homo sapiens apoptosis inhibitor 5 (API5), transcript variant 2, mRNA [NM_006595]	-0.48546413	0.0044508153
A_22_P00010857	*GPR125*	Homo sapiens G protein-coupled receptor 125 (GPR125), mRNA [NM_145290]	0.91065	0.010237655
A_23_P58489	*DGAT1*	Homo sapiens diacylglycerol O-acyltransferase 1 (DGAT1), mRNA [NM_012079]	0.65250176	0.0141058015
A_33_P3397865	*MIR4522*	Homo sapiens microRNA 4522 (MIR4522), microRNA [NR_039748]	0.5908194	0.0042551304
A_23_P43800	*FASTKD3*	Homo sapiens FAST kinase domains 3 (FASTKD3), transcript variant 1, mRNA [NM_024091]	-0.5832843	0.005057235
A_33_P3499102	*TNNT1*	Homo sapiens troponin T type 1 (skeletal, slow) (TNNT1), transcript variant 4, mRNA [NM_001291774]	-1379644	0.008282093
A_23_P78795	*BOP1*	Homo sapiens block of proliferation 1 (BOP1), mRNA [NM_015201]	13233747	0.007479662
A_33_P3314659	*RBM10*	Homo sapiens cDNA FLJ40796 fis, clone TRACH2007754, highly similar to DXS8237E PROTEIN. [AK098115]	3172666	0.0041916566
A_33_P3287611	*MEIS3*	Homo sapiens Meis homeobox 3 (MEIS3), transcript variant 2, mRNA [NM_001009813]	0.9278084	0.011296243
A_24_P322635	*SPEF2*	Homo sapiens sperm flagellar 2 (SPEF2), transcript variant 2, mRNA [NM_144722]	-12718176	0.006632856
A_23_P29079	*KRT3*	Homo sapiens keratin 3, type II (KRT3), mRNA [NM_057088]	0.55728537	0.013805346
A_23_P128930	*ELMO2*	Homo sapiens engulfment and cell motility 2 (ELMO2), transcript variant 2, mRNA [NM_182764]	-12077987	0.0029695062

A_21_P0007044	*PFKL*	Homo sapiens phosphofructokinase, liver (PFKL), transcript variant 1, mRNA [NM_001002021]	11548138	0.005712846
A_23_P46275	*PSMC6*	Homo sapiens proteasome (prosome, macropain) 26S subunit, ATPase, 6 (PSMC6), mRNA [NM_002806]	-0.54760647	0.01251397
A_33_P3379341	*LINC00707*	Homo sapiens long intergenic non-protein coding RNA 707 (LINC00707), long non-coding RNA [NR_038291]	10542392	0.008539769
A_23_P149529	*ATP5F1*	Homo sapiens ATP synthase, H+ transporting, mitochondrial Fo complex, subunit B1 (ATP5F1), mRNA [NM_001688]	-0.5822873	0.0065950155
A_24_P469641	*LOC391322*	Homo sapiens D-dopachrome tautomerase-like (LOC391322), mRNA [NM_001144931]	-0.70799	0.009635388
A_23_P38408	*TACSTD2*	Homo sapiens tumor-associated calcium signal transducer 2 (TACSTD2), mRNA [NM_002353]	-0.84935355	0.0020435157
A_23_P168556	*RNF216*	Homo sapiens ring finger protein 216 (RNF216), transcript variant 1, mRNA [NM_207111]	-0.5909785	0.011140108
A_23_P74799	*GGNBP2*	Homo sapiens gametogenetin binding protein 2 (GGNBP2), mRNA [NM_024835]	-0.73145574	0.009251071
A_22_P00002126	*STX1A*	Homo sapiens syntaxin 1A (brain) (STX1A), transcript variant 1, mRNA [NM_004603]	0.7749359	0.0044240346
A_21_P0000251	*SLC25A24*	Homo sapiens solute carrier family 25 (mitochondrial carrier; phosphate carrier), member 24 (SLC25A24), transcript variant 2, mRNA [NM_213651]	-0.5170295	0.004225475
A_33_P3217700	*DGUOK-AS1*	Homo sapiens DGUOK antisense RNA 1 (DGUOK-AS1), transcript variant 2, long non-coding RNA [NR_104030]	2084651	0.0029691157
A_23_P39223	*SNORA65*	Homo sapiens small nucleolar RNA, H/ACA box 65 (SNORA65), small nucleolar RNA [NR_002449]	-0.89301735	0.003365343
A_24_P356601	*USP9Y*	Homo sapiens ubiquitin specific peptidase 9, Y-linked (USP9Y), mRNA [NM_004654]	-0.7420823	0.016101459
A_24_P364057	*ZNF2*	Homo sapiens zinc finger protein 2 (ZNF2), transcript variant 1, mRNA [NM_021088]	2930408	0.004993122
A_33_P3421520	*HEXIM1*	Homo sapiens hexamethylene bis-acetamide inducible 1 (HEXIM1), mRNA [NM_006460]	-0.5986921	0.008298705
A_24_P355649	*C11orf70*	Homo sapiens chromosome 11 open reading frame 70 (C11orf70), transcript variant 1, mRNA [NM_032930]	-17498171	0.0028149292
A_24_P831309	*IKZF5*	Homo sapiens IKAROS family zinc finger 5 (Pegasus) (IKZF5), transcript variant 1, mRNA [NM_001271840]	-0.6768022	0.015881207
A_23_P81880	*FLI1*	Homo sapiens Fli-1 proto-oncogene, ETS transcription factor (FLI1), transcript variant 1, mRNA [NM_002017]	-3374635	0.003121324

A_23_P57807	*C1orf229*	Homo sapiens chromosome 1 open reading frame 229 (C1orf229), mRNA [NM_207401]	0.83867496	0.0064343205
A_33_P3415092	*CTDSP2*	Homo sapiens CTD (carboxy-terminal domain, RNA polymerase II, polypeptide A) small phosphatase 2 (CTDSP2), mRNA [NM_005730]	-12457882	0.014020975
A_23_P10858	*MKRN2*	Homo sapiens makorin ring finger protein 2 (MKRN2), transcript variant 1, mRNA [NM_014160]	-0.6744015	0.00435618
A_33_P3414964	*CLCN5*	Homo sapiens chloride channel, voltage-sensitive 5 (CLCN5), transcript variant 1, mRNA [NM_001127899]	-0.54030365	0.015324681
A_33_P3257903	*ANKLE2*	Homo sapiens ankyrin repeat and LEM domain containing 2 (ANKLE2), mRNA [NM_015114]	-0.41944164	0.005154336
A_33_P3365611	*PTPRS*	Homo sapiens protein tyrosine phosphatase, receptor type, S, mRNA (cDNA clone IMAGE:5272670), complete cds. [BC029496]	0.5845458	0.009240695
A_24_P91916	*GSTA4*	Homo sapiens glutathione S-transferase alpha 4 (GSTA4), mRNA [NM_001512]	-18168553	0.0029471128
A_23_P393620	*BTF3L4*	Homo sapiens basic transcription factor 3-like 4 (BTF3L4), transcript variant 1, mRNA [NM_152265]	-0.6835925	0.0050508454
A_22_P00024420	*NXT2*	Homo sapiens nuclear transport factor 2-like export factor 2 (NXT2), transcript variant 1, mRNA [NM_018698]	-0.7494336	0.006736978
A_21_P0000080	*TFPI2*	Homo sapiens tissue factor pathway inhibitor 2 (TFPI2), transcript variant 1, mRNA [NM_006528]	0.4247961	0.00556941
A_33_P3214690	*lnc-GCGR-1*	AU155391 OVARC1 Homo sapiens cDNA clone OVARC1001844 3', mRNA sequence [AU155391]	0.84425443	0.009515716
A_32_P159445	*FAM122C*	Homo sapiens family with sequence similarity 122C (FAM122C), transcript variant 4, mRNA [NM_001170781]	-0.78920525	0.0073643485
A_23_P42322	*NLGN1*	Homo sapiens neuroligin 1 (NLGN1), mRNA [NM_014932]	-33221128	0.0029276514
A_21_P0011339	*IQSEC2*	Homo sapiens IQ motif and Sec7 domain 2 (IQSEC2), transcript variant 2, mRNA [NM_015075]	10371931	0.0062004877
A_24_P124672	*COL11A2*	Homo sapiens collagen, type XI, alpha 2 (COL11A2), transcript variant 1, mRNA [NM_080680]	-10404497	0.016369263
A_33_P3286724	*XLOC_l2_004772*	BROAD Institute lincRNA (XLOC_l2_004772), lincRNA [TCONS_l2_00008799]	-11907306	0.0041847117
A_23_P395566	*DYNLL1*	Homo sapiens dynein, light chain, LC8-type 1 (DYNLL1), transcript variant 1, mRNA [NM_001037494]	-0.38532686	0.005402907
A_24_P47547	*PLD2*	Homo sapiens phospholipase D2 (PLD2), transcript variant 1, mRNA [NM_002663]	-0.99451846	0.004359316

A_23_P58521	*FBXO31*	Homo sapiens F-box protein 31 (FBXO31), transcript variant 1, mRNA [NM_024735]	-0.61582214	0.011631283
A_24_P49447	*RAN*	Homo sapiens RAN, member RAS oncogene family (RAN), transcript variant 1, mRNA [NM_006325]	-0.35821536	0.006694939
A_33_P3357530	*ERCC8*	Homo sapiens excision repair cross-complementation group 8 (ERCC8), transcript variant 2, mRNA [NM_001007233]	-0.7062348	0.0029691157
A_23_P134814	*RIC1*	Homo sapiens RAB6A GEF complex partner 1 (RIC1), transcript variant 1, mRNA [NM_020829]	-10860314	0.0066106957
A_33_P3283713	*SLC12A7*	Homo sapiens solute carrier family 12 (potassium/chloride transporter), member 7 (SLC12A7), mRNA [NM_006598]	-15593566	0.006573993
A_22_P00003353	*THAP1*	Homo sapiens THAP domain containing, apoptosis associated protein 1 (THAP1), transcript variant 1, mRNA [NM_018105]	-0.75620383	0.00381338
A_32_P67623	*ABHD14A*	Homo sapiens abhydrolase domain containing 14A (ABHD14A), mRNA [NM_015407]	-15723977	0.005428736
A_33_P3265514	*LINC00882*	Homo sapiens long intergenic non-protein coding RNA 882 (LINC00882), long non-coding RNA [NR_028303]	-11273088	0.009543431
A_33_P3287959	*FAM120C*	Homo sapiens family with sequence similarity 120C (FAM120C), transcript variant 3, mRNA [NM_001300788]	-10716894	0.013706757
A_24_P398781	*TPTE*	Homo sapiens transmembrane phosphatase with tensin homology (TPTE), transcript variant 1, mRNA [NM_199261]	0.54322445	0.013534785
A_22_P00012644	*RASA4*	Homo sapiens RAS p21 protein activator 4 (RASA4), transcript variant 1, mRNA [NM_006989]	-16017078	0.0034256727
A_23_P376599	*IFT43*	Homo sapiens intraflagellar transport 43 (IFT43), transcript variant 1, mRNA [NM_052873]	-12025738	0.005583561
A_21_P0014745	*RALBP1*	Homo sapiens ralA binding protein 1 (RALBP1), mRNA [NM_006788]	-0.7492552	0.016099177
A_23_P352291	*LOC728554*	Homo sapiens cDNA, FLJ17316. [AK310274]	17210287	0.003917791
A_33_P3253440	*SRSF10*	Homo sapiens serine/arginine-rich splicing factor 10 (SRSF10), transcript variant 2, mRNA [NM_054016]	-0.37193042	0.005377644
A_22_P00013376	*ZNF302*	Homo sapiens zinc finger protein 302 (ZNF302), transcript variant 15, mRNA [NM_018675]	-17526971	0.004102526
A_23_P213718	*PQLC3*	Homo sapiens PQ loop repeat containing 3 (PQLC3), transcript variant 1, mRNA [NM_152391]	-0.5245116	0.009152931

A_23_P37327	*lnc-RP11-17M16.1.1-1*	PREDICTED: Homo sapiens uncharacterized LOC101927989 (LOC101927989), mRNA [XM_006722594]	-17724097	0.003956951
A_24_P808100	*UQCRQ*	Homo sapiens ubiquinol-cytochrome c reductase, complex III subunit VII, 9.5kDa (UQCRQ), mRNA [NM_014402]	-0.6284004	0.0029691157
A_33_P3364051	*ABCD4*	Homo sapiens ATP-binding cassette, sub-family D (ALD), member 4 (ABCD4), transcript variant 1, mRNA [NM_005050]	-0.963886	0.006543285
A_33_P3347147	*MCTS1*	Homo sapiens malignant T cell amplified sequence 1 (MCTS1), transcript variant 1, mRNA [NM_014060]	-14195279	0.006573993
A_23_P6708	*TOP3B*	Homo sapiens topoisomerase (DNA) III beta (TOP3B), transcript variant 1, mRNA [NM_003935]	0.90371466	0.016401727
A_23_P140423	*SUPT3H*	Homo sapiens suppressor of Ty 3 homolog (S. cerevisiae) (SUPT3H), transcript variant 2, mRNA [NM_181356]	-12640291	0.004701671
A_33_P3407042	*NCBP2-AS2*	Homo sapiens NCBP2 antisense RNA 2 (head to head) (NCBP2-AS2), long non-coding RNA [NR_024388]	-0.36652392	0.010438802
A_23_P353014	*NDUFB1*	Homo sapiens NADH dehydrogenase (ubiquinone) 1 beta subcomplex, 1, 7kDa (NDUFB1), mRNA [NM_004545]	-0.5759423	0.006513625
A_33_P3364864	*B3GALT6*	Homo sapiens UDP-Gal:betaGal beta 1,3-galactosyltransferase polypeptide 6 (B3GALT6), mRNA [NM_080605]	-0.7300939	0.013823056
A_22_P00008020	*CACNA2D4*	Homo sapiens calcium channel, voltage-dependent, alpha 2/delta subunit 4 (CACNA2D4), mRNA [NM_172364]	-10470291	0.007907491
A_24_P389517	*NAMPT*	Homo sapiens nicotinamide phosphoribosyltransferase (NAMPT), mRNA [NM_005746]	19174252	0.0057417424
A_24_P942354	*LOC101929089*	Homo sapiens uncharacterized LOC101929089 (LOC101929089), transcript variant 5, long non-coding RNA [NR_120576]	0.993958	0.006080751
A_22_P00015868	*HNRNPK*	Homo sapiens heterogeneous nuclear ribonucleoprotein K (HNRNPK), transcript variant 3, mRNA [NM_031262]	-0.3323267	0.008029206
A_23_P56553	*PITPNA*	Homo sapiens phosphatidylinositol transfer protein, alpha (PITPNA), mRNA [NM_006224]	-0.4115486	0.0035432973
A_23_P21618	*TBX18*	T-box 18 [Source:HGNC Symbol;Acc:HGNC:11595] [ENST00000330469]	-14536725	0.0061534033
A_23_P140069	*METTL8*	Homo sapiens methyltransferase like 8 (METTL8), mRNA [NM_024770]	-0.6172665	0.009965863
A_24_P151498	*PDZRN3*	Homo sapiens PDZ domain containing ring finger 3 (PDZRN3), transcript variant 1, mRNA [NM_015009]	-0.7852816	0.0063791247

A_33_P3295108	*FBXL3*	Homo sapiens F-box and leucine-rich repeat protein 3 (FBXL3), mRNA [NM_012158]	-0.95114344	0.003609745
A_23_P257003	*PRMT2*	Homo sapiens protein arginine methyltransferase 2 (PRMT2), transcript variant 1, mRNA [NM_206962]	-0.82839274	0.015868904
A_21_P0008391	*GANC*	Homo sapiens glucosidase, alpha; neutral C (GANC), transcript variant 1, mRNA [NM_198141]	-11038716	0.012076056
A_23_P46095	*PCSK5*	Homo sapiens proprotein convertase subtilisin/kexin type 5 (PCSK5), transcript variant 2, mRNA [NM_006200]	-33466787	0.0033199077
A_23_P140170	*lnc-DLK1-9*	LNCipedia lincRNA (lnc-DLK1-9), lincRNA [lnc-DLK1-9:1]	16854676	0.0042506265
A_24_P20746	*C1orf131*	Homo sapiens chromosome 1 open reading frame 131 (C1orf131), transcript variant 1, mRNA [NM_152379]	-0.96569717	0.0057647754
A_24_P322369	*SEC23A*	Homo sapiens Sec23 homolog A (S. cerevisiae) (SEC23A), mRNA [NM_006364]	-0.6270795	0.0025001846
A_21_P0014940	*PRR16*	Homo sapiens proline rich 16 (PRR16), transcript variant 2, mRNA [NM_016644]	-0.5745412	0.004355401
A_33_P3259135	*VPS4B*	Homo sapiens vacuolar protein sorting 4 homolog B (S. cerevisiae) (VPS4B), mRNA [NM_004869]	-0.5867028	0.0117895575
A_33_P3413114	*lnc-TSC1-1*	Homo sapiens mRNA expressed only in placental villi, clone SMAP83. [AB019568]	23279471	0.0034534817
A_23_P206324	*NSG1*	Homo sapiens neuron specific gene family member 1 (NSG1), transcript variant 3, mRNA [NM_001287763]	10258824	0.01080114
A_22_P00010449	*ADAMTSL1*	Homo sapiens ADAMTS-like 1 (ADAMTSL1), transcript variant 2, mRNA [NM_052866]	-13465022	0.0060600177
A_24_P145629	*HSDL1*	Homo sapiens hydroxysteroid dehydrogenase like 1 (HSDL1), transcript variant 1, mRNA [NM_031463]	-0.80226135	0.010056441
A_24_P99216	*CLUAP1*	Homo sapiens clusterin associated protein 1 (CLUAP1), transcript variant 1, mRNA [NM_015041]	-0.76247275	0.013770719
A_21_P0003678	*SERINC2*	Homo sapiens serine incorporator 2 (SERINC2), transcript variant 1, mRNA [NM_178865]	32573237	0.0036923971
A_32_P38637	*LRP10*	Homo sapiens low density lipoprotein receptor-related protein 10 (LRP10), mRNA [NM_014045]	0.6352029	0.015841465
A_23_P160689	*lnc-SAP30-2*	LNCipedia lincRNA (lnc-SAP30-2), lincRNA [lnc-SAP30-2:2]	-10817189	0.0089123435
A_33_P3390570	*KRBA1*	Homo sapiens KRAB-A domain containing 1 (KRBA1), transcript variant 1, mRNA [NM_032534]	-0.8467826	0.0070515345

A_23_P39453	*LRIF1*	Homo sapiens ligand dependent nuclear receptor interacting factor 1 (LRIF1), transcript variant 1, mRNA [NM_018372]	-0.58027786	0.0077546863
A_22_P00006478	*KMT2A*	Homo sapiens lysine (K)-specific methyltransferase 2A (KMT2A), transcript variant 2, mRNA [NM_005933]	-0.71637464	0.0145504605
A_21_P0014394	*MEX3D*	Homo sapiens mex-3 RNA binding family member D (MEX3D), transcript variant 1, mRNA [NM_203304]	0.7720077	0.011443248
A_33_P3396692	*PTPRG-AS1*	Homo sapiens PTPRG antisense RNA 1 (PTPRG-AS1), transcript variant 1, long non-coding RNA [NR_038281]	-0.7064315	0.0034534817
A_23_P111092	*SSR1*	Homo sapiens signal sequence receptor, alpha (SSR1), transcript variant 1, mRNA [NM_003144]	-0.64570934	0.01477476
A_22_P00012701	*ANXA10*	Homo sapiens annexin A10 (ANXA10), mRNA [NM_007193]	0.63330257	0.014989517
A_21_P0011406	*OR2H1*	Homo sapiens olfactory receptor, family 2, subfamily H, member 1 (OR2H1), mRNA [NM_030883]	14688674	0.005612091
A_23_P101407	*lnc-RAB1A-4*	LNCipedia lincRNA (lnc-RAB1A-4), lincRNA [lnc-RAB1A-4:1]	0.7989673	0.015933463
A_33_P3224212	*C3*	Homo sapiens complement component 3 (C3), mRNA [NM_000064]	5262832	0.0025001846
A_22_P00010200	*C11orf52*	Homo sapiens chromosome 11 open reading frame 52 (C11orf52), mRNA [NM_080659]	-16188102	0.007479662
A_23_P203743	*R3HDM1*	Homo sapiens R3H domain containing 1 (R3HDM1), transcript variant 1, mRNA [NM_001282798]	0.6386738	0.014307376
A_33_P3215575	*LOC101927895*	PREDICTED: Homo sapiens uncharacterized LOC101927895 (LOC101927895), transcript variant X1, ncRNA [XR_241220]	-10255386	0.005255513
A_33_P3344816	*GAB2*	Homo sapiens GRB2-associated binding protein 2 (GAB2), transcript variant 2, mRNA [NM_012296]	-13557842	0.0057806885
A_33_P3405680	*ARHGEF10L*	Homo sapiens Rho guanine nucleotide exchange factor (GEF) 10-like (ARHGEF10L), transcript variant 1, mRNA [NM_018125]	-10807378	0.0062032775
A_22_P00012106	*ZNF813*	Homo sapiens zinc finger protein 813 (ZNF813), mRNA [NM_001004301]	-12240741	0.0067145773
A_23_P35820	*ARHGEF3-AS1*	Homo sapiens ARHGEF3 antisense RNA 1 (ARHGEF3-AS1), long non-coding RNA [NR_046572]	-0.9406776	0.013942816
A_22_P00015920	*lnc-PODN-1*	Homo sapiens cDNA FLJ36729 fis, clone UTERU2012581. [AK094048]	11003882	0.004670025
A_33_P3393766	*CFL1*	Homo sapiens cofilin 1 (non-muscle) (CFL1), mRNA [NM_005507]	0.5894885	0.010237655

A_33_P3307980	*C17orf96*	Homo sapiens chromosome 17 open reading frame 96 (C17orf96), mRNA [NM_001130677]	-11746157	0.006110945
A_23_P110961	*SSPO*	Homo sapiens SCO-spondin (SSPO), mRNA [NM_198455]	0.7095891	0.010642556
A_21_P0011550	*lnc-FAM133B-1*	Homo sapiens cDNA FLJ16364 fis, clone THYMU2032976. [AK131345]	-0.8569529	0.012953318
A_23_P350512	*MPC1*	Homo sapiens mitochondrial pyruvate carrier 1 (MPC1), transcript variant 1, mRNA [NM_016098]	-16244688	0.0037479755
A_22_P00017547	*LINC00674*	Homo sapiens long intergenic non-protein coding RNA 674 (LINC00674), long non-coding RNA [NR_027418]	-12556378	0.009690877
A_33_P3297978	*ADAM12*	Homo sapiens ADAM metallopeptidase domain 12 (ADAM12), transcript variant 2, mRNA [NM_021641]	-23313355	0.011969343
A_24_P294124	*ADARB2-AS1*	Homo sapiens ADARB2 antisense RNA 1 (ADARB2-AS1), long non-coding RNA [NR_033387]	0.88977045	0.009841122
A_23_P355385	*MYO1E*	Homo sapiens myosin IE (MYO1E), mRNA [NM_004998]	-19874856	0.0061896956
A_23_P97932	*SERTAD2*	Homo sapiens SERTA domain containing 2 (SERTAD2), mRNA [NM_014755]	-0.9275608	0.006143828
A_33_P3378737	*PPP6C*	Homo sapiens protein phosphatase 6, catalytic subunit (PPP6C), transcript variant 2, mRNA [NM_002721]	-0.70809716	0.012329381
A_23_P218770	*MSRB2*	Homo sapiens methionine sulfoxide reductase B2 (MSRB2), mRNA [NM_012228]	-0.7507963	0.016256094
A_32_P4403	*RAC2*	Homo sapiens ras-related C3 botulinum toxin substrate 2 (rho family, small GTP binding protein Rac2) (RAC2), mRNA [NM_002872]	0.8927493	0.014997627
A_33_P3344282	*lnc-FUS-1*	Homo sapiens mRNA; cDNA DKFZp667F205 (from clone DKFZp667F205). [AL713703]	17515101	0.009503543
A_24_P370096	*C16orf72*	Homo sapiens chromosome 16 open reading frame 72 (C16orf72), mRNA [NM_014117]	-0.91696924	0.007740612
A_33_P3342563	*HS1BP3*	Homo sapiens HCLS1 binding protein 3, mRNA (cDNA clone IMAGE:6056200), with apparent retained intron. [BC038847]	-1007449	0.011443763
A_33_P3331726	*ZNF230*	Homo sapiens zinc finger protein 230 (ZNF230), mRNA [NM_006300]	-0.31684303	0.0050640735
A_23_P37545	*PITPNM3*	Homo sapiens PITPNM family member 3 (PITPNM3), transcript variant 1, mRNA [NM_031220]	-12554041	0.010766392
A_33_P3340164	*LINC00657*	Homo sapiens long intergenic non-protein coding RNA 657 (LINC00657), long non-coding RNA [NR_027451]	0.39051798	0.010182504
A_23_P87500	*AAGAB*	Homo sapiens alpha- and gamma-adaptin binding protein (AAGAB), transcript variant 1, mRNA [NM_024666]	-0.64343786	0.010254725

A_23_P134237	*NDUFB5*	Homo sapiens NADH dehydrogenase (ubiquinone) 1 beta subcomplex, 5, 16kDa (NDUFB5), transcript variant 3, mRNA [NM_001199958]	-1057545	0.004588558
A_23_P157147	*ORMDL2*	Homo sapiens ORMDL sphingolipid biosynthesis regulator 2 (ORMDL2), mRNA [NM_014182]	-0.77541536	0.005037577 4
A_23_P79221	*RARRES2*	Homo sapiens retinoic acid receptor responder (tazarotene induced) 2 (RARRES2), mRNA [NM_002889]	6795907	0.001417249
A_24_P87114	*ZDHHC4*	Homo sapiens zinc finger, DHHC-type containing 4 (ZDHHC4), transcript variant 3, mRNA [NM_018106]	-12114019	0.008311857
A_22_P000061 87	*ACVR1*	Homo sapiens activin A receptor, type I (ACVR1), transcript variant 1, mRNA [NM_001105]	-0.6929992	0.014939495
A_33_P326426 9	*USP30*	Homo sapiens ubiquitin specific peptidase 30 (USP30), transcript variant 1, mRNA [NM_032663]	0.49190447	0.014391575
A_23_P63209	*KCND3-IT1*	Homo sapiens KCND3 intronic transcript 1 (non-protein coding) (KCND3-IT1), long non-coding RNA [NR_046783]	0.7352567	0.012864231
A_33_P339523 7	*MOV10*	Homo sapiens Mov10 RISC complex RNA helicase (MOV10), transcript variant 3, mRNA [NM_001286072]	15183642	0.004611096
A_33_P335738 2	*HSD11B1*	Homo sapiens hydroxysteroid (11-beta) dehydrogenase 1 (HSD11B1), transcript variant 2, mRNA [NM_181755]	-18967339	0.002334024 3
A_32_P194246	*POGZ*	pogo transposable element with ZNF domain [Source:HGNC Symbol;Acc:HGNC:18801] [ENST00000485040]	-13111306	0.006474282
A_23_P39955	*ABCB4*	Homo sapiens ATP-binding cassette, sub-family B (MDR/TAP), member 4 (ABCB4), transcript variant B, mRNA [NM_018849]	-3805861	0.002489003 1
A_23_P90589	*CLEC16A*	Homo sapiens C-type lectin domain family 16, member A (CLEC16A), transcript variant 1, mRNA [NM_015226]	-0.5526066	0.011794303
A_33_P330027 3	*ACTG2*	Homo sapiens actin, gamma 2, smooth muscle, enteric (ACTG2), transcript variant 1, mRNA [NM_001615]	16247008	0.006500368
A_33_P335324 2	*MRPL44*	Homo sapiens mitochondrial ribosomal protein L44 (MRPL44), mRNA [NM_022915]	-0.64938325	0.009835709
A_21_P000581 8	*C1orf52*	Homo sapiens chromosome 1 open reading frame 52 (C1orf52), transcript variant 1, mRNA [NM_198077]	-0.7298358	0.004431944 3
A_32_P167396	*HSPB1*	Homo sapiens heat shock 27kDa protein 1 (HSPB1), mRNA [NM_001540]	0.7161717	0.006681883 7

A_33_P3351982	*LOC100507420*	Homo sapiens uncharacterized LOC100507420 (LOC100507420), transcript variant 2, long non-coding RNA [NR_121621]	-15876155	0.0044074184
A_33_P3396270	*MED14OS*	Homo sapiens MED14 opposite strand (MED14OS), mRNA [NM_001289773]	-0.37549967	0.017161094
A_23_P44244	*COA5*	Homo sapiens cytochrome c oxidase assembly factor 5 (COA5), mRNA [NM_001008215]	-10881798	0.0043581175
A_23_P258621	*SLC9A4*	Homo sapiens solute carrier family 9, subfamily A (NHE4, cation proton antiporter 4), member 4 (SLC9A4), mRNA [NM_001011552]	17303758	0.011958462
A_23_P97250	*SMARCA1*	Homo sapiens SWI/SNF related, matrix associated, actin dependent regulator of chromatin, subfamily a, member 1 (SMARCA1), transcript variant 1, mRNA [NM_003069]	-0.9606135	0.0070031113
A_33_P3289113	*RTCA*	Homo sapiens RNA 3'-terminal phosphate cyclase (RTCA), transcript variant 2, mRNA [NM_003729]	-0.8111112	0.003162355
A_33_P3307447	*GON4L*	Homo sapiens gon-4-like (C. elegans) (GON4L), transcript variant 3, mRNA [NM_001282860]	-10392685	0.0035935708
A_24_P290354	*COX11*	cytochrome c oxidase assembly homolog 11 (yeast) [Source:HGNC Symbol;Acc:HGNC:2261] [ENST00000572088]	-16002898	0.007304192
A_23_P47497	*RPL32P3*	ribosomal protein L32 pseudogene 3 [Source:HGNC Symbol;Acc:HGNC:27024] [ENST00000359025]	-0.8913779	0.0057969303
A_33_P3361851	*ZFAND5*	Homo sapiens zinc finger, AN1-type domain 5 (ZFAND5), transcript variant c, mRNA [NM_006007]	-0.3384101	0.016537301
A_33_P3305173	*MRPL16*	Homo sapiens mitochondrial ribosomal protein L16 (MRPL16), mRNA [NM_017840]	-0.86847657	0.012276114
A_23_P134100	*RAB7A*	Homo sapiens RAB7A, member RAS oncogene family (RAB7A), mRNA [NM_004637]	-0.438129	0.013062467
A_21_P0004622	*RGAG4*	Homo sapiens retrotransposon gag domain containing 4 (RGAG4), mRNA [NM_001024455]	-1218307	0.004026994
A_33_P3247624	*ULBP3*	Homo sapiens UL16 binding protein 3 (ULBP3), mRNA [NM_024518]	10103941	0.012495794
A_24_P152968	*lnc-AL035696.1-5*	LNCipedia lincRNA (lnc-AL035696.1-5), lincRNA [lnc-AL035696.1-5:1]	-11196985	0.011137466
A_23_P12526	*REP15*	Homo sapiens RAB15 effector protein (REP15), mRNA [NM_001029874]	-0.9624189	0.014707766
A_33_P3674851	*AKR1C1*	Homo sapiens aldo-keto reductase family 1, member C1 (AKR1C1), mRNA [NM_001353]	0.8502772	0.01600397
A_21_P0005715	*TP53BP2*	Homo sapiens tumor protein p53 binding protein 2 (TP53BP2), transcript variant 2, mRNA [NM_005426]	-0.53763	0.011778491
A_23_P28886	*VPS36*	Homo sapiens vacuolar protein sorting 36 homolog (S. cerevisiae) (VPS36), transcript variant 3, mRNA [NM_001282169]	-0.8474269	0.0034903844

A_23_P44684	*LOC101926892*	Homo sapiens uncharacterized LOC101926892 (LOC101926892), long non-coding RNA [NR_110653]	19180211	0.0047432994
A_23_P329573	*PCNA*	Homo sapiens proliferating cell nuclear antigen (PCNA), transcript variant 1, mRNA [NM_002592]	-0.2943595	0.0054538557
A_33_P3531857	*ECT2*	Homo sapiens epithelial cell transforming 2 (ECT2), transcript variant 3, mRNA [NM_018098]	-0.38103697	0.009630487
A_23_P65518	*ITGB2*	Homo sapiens integrin, beta 2 (complement component 3 receptor 3 and 4 subunit) (ITGB2), transcript variant 1, mRNA [NM_000211]	-474338	0.0050554653
A_23_P99515	*DARS*	Homo sapiens aspartyl-tRNA synthetase (DARS), transcript variant 1, mRNA [NM_001349]	-0.6355669	0.012830978
A_23_P96271	*DACT1*	Homo sapiens dishevelled-binding antagonist of beta-catenin 1 (DACT1), transcript variant 1, mRNA [NM_016651]	-0.66669774	0.010986003
A_33_P3287785	*MEDAG*	Homo sapiens mesenteric estrogen-dependent adipogenesis (MEDAG), mRNA [NM_032849]	-11126451	0.008259308
A_33_P3362812	*MYOM1*	Homo sapiens myomesin 1 (MYOM1), transcript variant 1, mRNA [NM_003803]	-0.8489114	0.017157309
A_24_P14010	*NEFL*	Homo sapiens neurofilament, light polypeptide (NEFL), mRNA [NM_006158]	-12190402	0.0052430327
A_23_P329375	*NMT1*	Homo sapiens N-myristoyltransferase 1 (NMT1), mRNA [NM_021079]	-0.70640945	0.008055986
A_21_P0011495	*MCCC1*	Homo sapiens methylcrotonoyl-CoA carboxylase 1 (alpha) (MCCC1), transcript variant 1, mRNA [NM_020166]	-0.98923206	0.0034693098
A_33_P3249185	*POU6F1*	Homo sapiens POU class 6 homeobox 1 (POU6F1), transcript variant 1, mRNA [NM_002702]	-0.80120075	0.007700591
A_22_P00009395	*DDX11L16*	Homo sapiens DEAD/H (Asp-Glu-Ala-Asp/His) box helicase 11 like 16 (DDX11L16), non-coding RNA [NR_110561]	0.9692719	0.007549738
A_23_P204929	*SAR1A*	secretion associated, Ras related GTPase 1A [Source:HGNC Symbol;Acc:HGNC:10534] [ENST00000373236]	-0.7892678	0.0048260237
A_33_P3283900	*SSSCA1-AS1*	Homo sapiens SSSCA1 antisense RNA 1 (head to head) (SSSCA1-AS1), long non-coding RNA [NR_038923]	-1217761	0.007571981
A_24_P205130	*AKAP11*	Homo sapiens A kinase (PRKA) anchor protein 11 (AKAP11), mRNA [NM_016248]	-0.23083785	0.008897745
A_23_P100001	*CTNS*	Homo sapiens cystinosin, lysosomal cystine transporter (CTNS), transcript variant 2, mRNA [NM_004937]	13608185	0.012866833

A_23_P351467	*FNBP1*	Homo sapiens formin binding protein 1 (FNBP1), mRNA [NM_015033]	-0.8562937	0.010974453
A_22_P00002248	*FAM174B*	Homo sapiens family with sequence similarity 174, member B (FAM174B), mRNA [NM_207446]	2749456	0.0061608143
A_23_P303260	*CMAHP*	Homo sapiens cytidine monophospho-N-acetylneuraminic acid hydroxylase, pseudogene (CMAHP), transcript variant 1, non-coding RNA [NR_002174]	-2077992	0.010370998
A_33_P3290562	*FLJ37035*	Homo sapiens uncharacterized LOC399821 (FLJ37035), long non-coding RNA [NR_033847]	-1088794	0.0055521973
A_23_P69100	*STX7*	Homo sapiens syntaxin 7 (STX7), mRNA [NM_003569]	-0.97476393	0.0065950155
A_23_P48056	*GLI3*	Homo sapiens GLI family zinc finger 3 (GLI3), mRNA [NM_000168]	-15352545	0.004288771
A_21_P0000584	*ARHGEF26*	Homo sapiens Rho guanine nucleotide exchange factor (GEF) 26 (ARHGEF26), transcript variant 2, mRNA [NM_015595]	0.82076716	0.014949956
A_24_P333802	*CKAP4*	Homo sapiens cytoskeleton-associated protein 4 (CKAP4), mRNA [NM_006825]	-0.2411182	0.0109699825
A_23_P53957	*VTRNA2-1*	Homo sapiens vault RNA 2-1 (VTRNA2-1), vault RNA [NR_030583]	-0.8288077	0.013706757
A_33_P3363260	*MAGT1*	Homo sapiens magnesium transporter 1 (MAGT1), mRNA [NM_032121]	-0.856168	0.00407791
A_23_P56170	*COX16*	Homo sapiens COX16 cytochrome c oxidase assembly homolog (S. cerevisiae) (COX16), transcript variant 1, mRNA [NM_016468]	-0.5123496	0.010584611
A_33_P3350566	*PGM2L1*	Homo sapiens phosphoglucomutase 2-like 1 (PGM2L1), mRNA [NM_173582]	-10713891	0.005073928
A_33_P3303372	*MED16*	Homo sapiens mediator complex subunit 16 (MED16), mRNA [NM_005481]	0.8958013	0.011200304
A_23_P154874	*CACNA1I*	Homo sapiens calcium channel, voltage-dependent, T type, alpha 1I subunit (CACNA1I), transcript variant 1, mRNA [NM_021096]	0.6578	0.007549738
A_19_P00322711	*PARD3*	Homo sapiens par-3 family cell polarity regulator (PARD3), transcript variant 9, mRNA [NM_001184792]	-0.693629	0.011646048
A_33_P3271111	*DSCR3*	Homo sapiens Down syndrome critical region 3 (DSCR3), mRNA [NM_006052]	-0.667973	0.012737636
A_33_P3275310	*LINC00662*	Homo sapiens long intergenic non-protein coding RNA 662 (LINC00662), long non-coding RNA [NR_027301]	-0.8680527	0.009915023
A_24_P153511	*NINJ1*	Homo sapiens ninjurin 1 (NINJ1), mRNA [NM_004148]	11210089	0.005852893
A_23_P157352	*ATXN3*	Homo sapiens ataxin 3 (ATXN3), transcript variant reference, mRNA [NM_004993]	-0.6697203	0.00556941

A_33_P3252191	*OSBPL8*	Homo sapiens oxysterol binding protein-like 8 (OSBPL8), transcript variant 1, mRNA [NM_020841]	-0.70554084	0.006573993
A_33_P3400943	*MRPS33*	Homo sapiens mitochondrial ribosomal protein S33 (MRPS33), transcript variant 1, mRNA [NM_016071]	-0.5942497	0.006740723
A_33_P3408962	*CDIP1*	Homo sapiens cell death-inducing p53 target 1 (CDIP1), transcript variant 1, mRNA [NM_001199054]	-22633166	0.0046812603
A_32_P81768	*ZNF236*	Homo sapiens zinc finger protein 236 (ZNF236), mRNA [NM_007345]	-0.56766796	0.007860159
A_24_P693321	*SCN8A*	Homo sapiens sodium channel, voltage gated, type VIII alpha subunit (SCN8A), transcript variant 1, mRNA [NM_014191]	-11847227	0.012529562
A_23_P17490	*TMEM167A*	Homo sapiens transmembrane protein 167A (TMEM167A), mRNA [NM_174909]	-0.53841805	0.0039856653
A_32_P31182	*LOC100190986*	Homo sapiens uncharacterized LOC100190986 (LOC100190986), long non-coding RNA [NR_024456]	0.5871687	0.008289811
A_19_P00807336	*TMEM230*	Homo sapiens transmembrane protein 230 (TMEM230), transcript variant 2, mRNA [NM_001009924]	-12423525	0.0037583082
A_23_P74778	*RPL7*	Homo sapiens ribosomal protein L7 (RPL7), mRNA [NM_000971]	-0.7766597	0.013441296
A_23_P46063	*PPA1*	Homo sapiens pyrophosphatase (inorganic) 1 (PPA1), mRNA [NM_021129]	-0.58040524	0.006371145
A_33_P3423610	*C1orf54*	Homo sapiens chromosome 1 open reading frame 54 (C1orf54), transcript variant 3, mRNA [NM_024579]	-0.9741657	0.0047642486
A_23_P58506	*NME7*	Homo sapiens NME/NM23 family member 7 (NME7), transcript variant 1, mRNA [NM_013330]	-0.78782505	0.005543548
A_23_P320290	*ELL2*	Homo sapiens elongation factor, RNA polymerase II, 2 (ELL2), mRNA [NM_012081]	-0.9803838	0.013411169
A_23_P23380	*BRINP1*	Homo sapiens bone morphogenetic protein/retinoic acid inducible neural-specific 1 (BRINP1), mRNA [NM_014618]	18604943	0.009264408
A_24_P165656	*ZNF827*	Homo sapiens zinc finger protein 827 (ZNF827), mRNA [NM_178835]	0.71354467	0.014571856
A_23_P135257	*ELOVL1*	Homo sapiens ELOVL fatty acid elongase 1 (ELOVL1), transcript variant 1, mRNA [NM_022821]	0.88999504	0.00690275
A_33_P3287223	*PRKD3*	Homo sapiens protein kinase D3 (PRKD3), mRNA [NM_005813]	-10231497	0.0073988605
A_23_P91140	*PRSS3*	Homo sapiens protease, serine, 3 (PRSS3), transcript variant 2, mRNA [NM_002771]	27135844	0.003162355
A_24_P123408	*DPP4*	Homo sapiens dipeptidyl-peptidase 4 (DPP4), mRNA [NM_001935]	-0.73035693	0.009276546

A_24_P256654	*PECR*	Homo sapiens peroxisomal trans-2-enoyl-CoA reductase (PECR), mRNA [NM_018441]	-13477002	0.009291781
A_22_P00001270	*ABLIM3*	Homo sapiens actin binding LIM protein family, member 3 (ABLIM3), transcript variant 2, mRNA [NM_014945]	0.7244654	0.0093693705
A_24_P116242	*ZNF674-AS1*	Homo sapiens ZNF674 antisense RNA 1 (head to head) (ZNF674-AS1), long non-coding RNA [NR_015378]	-0.7298833	0.009475495
A_24_P113686	*lnc-ANKRD53-1*	BX110621 Soares_testis_NHT Homo sapiens cDNA clone IMAGp998E034493, mRNA sequence [BX110621]	12180164	0.0067360536
A_24_P339429	*KLHDC2*	Homo sapiens kelch domain containing 2 (KLHDC2), mRNA [NM_014315]	-12307451	0.0038773962
A_23_P6303	*ZNHIT6*	Homo sapiens zinc finger, HIT-type containing 6 (ZNHIT6), transcript variant 1, mRNA [NM_017953]	-0.32089308	0.016667983
A_23_P434890	*KCNJ12*	Homo sapiens potassium channel, inwardly rectifying subfamily J, member 12 (KCNJ12), mRNA [NM_021012]	-4134994	0.0031750489
A_22_P00002240	*U2AF1*	Homo sapiens U2 small nuclear RNA auxiliary factor 1 (U2AF1), transcript variant c, mRNA [NM_001025204]	-0.77200127	0.004016376
A_22_P00017033	*CARD10*	Homo sapiens caspase recruitment domain family, member 10 (CARD10), mRNA [NM_014550]	-2425519	0.010920335
A_33_P3349597	*lnc-C10orf114-1*	LNCipedia lincRNA (lnc-C10orf114-1), lincRNA [lnc-C10orf114-1:1]	0.858567	0.0107146185
A_23_P96976	*lnc-TTLL11-1*	PREDICTED: Homo sapiens tubulin tyrosine ligase-like family, member 11 (TTLL11), transcript variant X4, mRNA [XM_006716967]	-1492398	0.004701671
A_33_P3405424	*ZCCHC17*	Homo sapiens zinc finger, CCHC domain containing 17 (ZCCHC17), transcript variant 3, mRNA [NM_016505]	-0.84299356	0.005879462
A_23_P56703	*MTERF4*	Homo sapiens mitochondrial transcription termination factor 4 (MTERF4), transcript variant 2, non-coding RNA [NR_028049]	-13588784	0.005215599
A_23_P133995	*IL4I1*	Homo sapiens interleukin 4 induced 1 (IL4I1), transcript variant 1, mRNA [NM_152899]	29966712	0.0027262743
A_23_P126970	*TRABD2A*	Homo sapiens TraB domain containing 2A (TRABD2A), transcript variant 1, mRNA [NM_001277053]	14299879	0.009987734
A_33_P3256550	*PPIL1*	Homo sapiens peptidylprolyl isomerase (cyclophilin)-like 1 (PPIL1), mRNA [NM_016059]	-0.55755424	0.010801365
A_23_P345942	*TIMM17A*	Homo sapiens translocase of inner mitochondrial membrane 17 homolog A (yeast) (TIMM17A), mRNA [NM_006335]	-0.72067523	0.005861645

A_33_P3399840	*MST152*	Homo sapiens MSTP152 (MST152) mRNA, complete cds. [AF190155]	0.5616231	0.014768076
A_33_P3400653	*NDUFAF2*	Homo sapiens NADH dehydrogenase (ubiquinone) complex I, assembly factor 2 (NDUFAF2), mRNA [NM_174889]	-0.54434085	0.012731291
A_23_P301925	*USP39*	Homo sapiens ubiquitin specific peptidase 39 (USP39), transcript variant 1, mRNA [NM_006590]	-0.7295506	0.009510488
A_22_P00011184	*BNIP2*	BCL2/adenovirus E1B 19kDa interacting protein 2 [Source:HGNC Symbol;Acc:HGNC:1083] [ENST00000267859]	-0.9164982	0.0077072685
A_21_P0007566	*COX1*	mitochondrially encoded cytochrome c oxidase I [Source:HGNC Symbol;Acc:HGNC:7419] [ENST00000361624]	0.77136827	0.0024890031
A_24_P95154	*lnc-NAB2-1*	LNCipedia lincRNA (lnc-NAB2-1), lincRNA [lnc-NAB2-1:1]	1121956	0.011566584
A_33_P3288159	*DHRS12*	Homo sapiens dehydrogenase/reductase (SDR family) member 12 (DHRS12), transcript variant 1, mRNA [NM_001031719]	-13029246	0.009918909
A_33_P3323347	*TUSC3*	Homo sapiens tumor suppressor candidate 3 (TUSC3), transcript variant 2, mRNA [NM_178234]	-0.26820472	0.015647039
A_24_P222599	*ASPM*	Homo sapiens asp (abnormal spindle) homolog, microcephaly associated (Drosophila) (ASPM), transcript variant 1, mRNA [NM_018136]	-0.4020362	0.008307647
A_33_P3414312	*ZNF417*	Homo sapiens zinc finger protein 417 (ZNF417), transcript variant 2, mRNA [NM_001297734]	-0.8998118	0.00511559
DCP_22_2	*PDPK1*	Homo sapiens 3-phosphoinositide dependent protein kinase 1 (PDPK1), transcript variant 1, mRNA [NM_002613]	-0.7743597	0.0034964236
A_21_P0010732	*USP32*	Homo sapiens ubiquitin specific peptidase 32 (USP32), mRNA [NM_032582]	-0.97047937	0.01181994
A_23_P252306	*DLC1*	Homo sapiens DLC1 Rho GTPase activating protein (DLC1), transcript variant 1, mRNA [NM_182643]	-0.57722735	0.0065208646
A_23_P420692	*PAN3*	Homo sapiens PAN3 poly(A) specific ribonuclease subunit (PAN3), mRNA [NM_175854]	-0.6755838	0.009690877
A_33_P3809328	*ID1*	Homo sapiens inhibitor of DNA binding 1, dominant negative helix-loop-helix protein (ID1), transcript variant 1, mRNA [NM_002165]	0.88226604	0.003422245
A_33_P3215864	*PPFIA4*	protein tyrosine phosphatase, receptor type, f polypeptide (PTPRF), interacting protein (liprin), alpha 4 [Source:HGNC Symbol;Acc:HGNC:9248] [ENST00000295706]	-16488898	0.0053216834

A_21_P0002612	*FAM3A*	Homo sapiens family with sequence similarity 3, member A (FAM3A), transcript variant 4, mRNA [NM_001171134]	10305858	0.0046744854
A_24_P385611	*HOXD9*	Homo sapiens homeobox D9 (HOXD9), mRNA [NM_014213]	0.6358633	0.0066500553
A_24_P252794	*lnc-GPR39-2*	LNCipedia lincRNA (lnc-GPR39-2), lincRNA [lnc-GPR39-2:3]	10140419	0.0033199077
A_23_P215669	*SP100*	Homo sapiens SP100 nuclear antigen (SP100), transcript variant 2, mRNA [NM_003113]	-0.72964436	0.00970286
A_23_P119130	*CUL2*	Homo sapiens cullin 2 (CUL2), transcript variant 3, mRNA [NM_003591]	-0.7328075	0.012620988
A_33_P3351615	*POLR2J2*	Homo sapiens polymerase (RNA) II (DNA directed) polypeptide J2 (POLR2J2), mRNA [NM_032959]	2597911	0.005860685
A_24_P165423	*RPS19*	Homo sapiens ribosomal protein S19 (RPS19), mRNA [NM_001022]	-0.35851356	0.0036402496
A_33_P3881056	*RBP7*	Homo sapiens retinol binding protein 7, cellular (RBP7), mRNA [NM_052960]	-0.9833603	0.009679106
A_33_P3406493	*SNORA21*	Homo sapiens small nucleolar RNA, H/ACA box 21 (SNORA21), small nucleolar RNA [NR_002576]	-0.7689483	0.004803986
A_33_P3286754	*LOC100996405*	PREDICTED: Homo sapiens uncharacterized LOC100996405 (LOC100996405), mRNA [XM_003846604]	-15047151	0.0037124872
A_23_P46378	*GABBR2*	Homo sapiens gamma-aminobutyric acid (GABA) B receptor, 2 (GABBR2), mRNA [NM_005458]	-0.45986456	0.014825912
A_23_P412029	*SEC14L2*	Homo sapiens SEC14-like 2 (S. cerevisiae) (SEC14L2), transcript variant 2, mRNA [NM_033382]	-11120863	0.008095165
A_21_P0014748	*PIGV*	Homo sapiens phosphatidylinositol glycan anchor biosynthesis, class V (PIGV), transcript variant 2, mRNA [NM_017837]	-0.7616853	0.008307647
A_33_P3210516	*PUS10*	Homo sapiens pseudouridylate synthase 10 (PUS10), mRNA [NM_144709]	-0.9984354	0.010536704
A_21_P0013497	*COL27A1*	Homo sapiens collagen, type XXVII, alpha 1 (COL27A1), mRNA [NM_032888]	-15003431	0.005208127
A_23_P141738	*LOC642236*	Homo sapiens FSHD region gene 1 pseudogene (LOC642236), non-coding RNA [NR_033907]	-0.85744905	0.010271231
A_23_P390596	*XLOC_l2_014245*	BROAD Institute lincRNA (XLOC_l2_014245), lincRNA [TCONS_l2_00027829]	-13566198	0.004785329
A_33_P3303414	*SS18*	Homo sapiens synovial sarcoma translocation, chromosome 18 (SS18), transcript variant 1, mRNA [NM_001007559]	-0.7221248	0.015953625
A_21_P0013930	*PSKH1*	Homo sapiens protein serine kinase H1 (PSKH1), mRNA [NM_006742]	-0.7368798	0.014904488
A_23_P422794	*MAN1A1*	Homo sapiens mannosidase, alpha, class 1A, member 1 (MAN1A1), mRNA [NM_005907]	-0.87705016	0.0037353234

A_24_P333733	*NSMCE2*	Homo sapiens non-SMC element 2, MMS21 homolog (S. cerevisiae) (NSMCE2), mRNA [NM_173685]	-0.5778887	0.009591824
A_23_P102122	*lnc-CACNA1B-1*	LNCipedia lincRNA (lnc-CACNA1B-1), lincRNA [lnc-CACNA1B-1:3]	-11030434	0.0072139124
A_33_P3209869	*ATP6V0A1*	Homo sapiens ATPase, H+ transporting, lysosomal V0 subunit a1 (ATP6V0A1), transcript variant 3, mRNA [NM_005177]	-14493911	0.005776505
A_33_P3252989	*ARPC2*	Homo sapiens actin related protein 2/3 complex, subunit 2, 34kDa (ARPC2), transcript variant 1, mRNA [NM_152862]	-0.24564177	0.010660367
A_33_P3234855	*TCP11L1*	Homo sapiens t-complex 11, testis-specific-like 1 (TCP11L1), transcript variant 1, mRNA [NM_018393]	-12184253	0.010396678
A_23_P92954	*ZSWIM8*	Homo sapiens zinc finger, SWIM-type containing 8 (ZSWIM8), transcript variant 1, mRNA [NM_015037]	-0.89615506	0.005501472
A_24_P42136	*UTRN*	Homo sapiens utrophin (UTRN), mRNA [NM_007124]	-0.8320822	0.008307647
A_33_P3371889	*HSD17B4*	Homo sapiens hydroxysteroid (17-beta) dehydrogenase 4 (HSD17B4), transcript variant 2, mRNA [NM_000414]	-0.88098526	0.008271377
A_33_P3232277	*KRT18*	Homo sapiens keratin 18, type I (KRT18), transcript variant 1, mRNA [NM_000224]	16174654	0.0104427505
A_24_P357465	*NUAK1*	Homo sapiens NUAK family, SNF1-like kinase, 1 (NUAK1), mRNA [NM_014840]	-0.12123059	0.012584146
A_23_P98015	*CCNG1*	Homo sapiens cyclin G1 (CCNG1), transcript variant 1, mRNA [NM_004060]	-0.5912186	0.009512793
A_33_P3240295	*TP53INP2*	Homo sapiens tumor protein p53 inducible nuclear protein 2 (TP53INP2), mRNA [NM_021202]	0.39859393	0.008174585
A_33_P3365932	*CUTC*	Homo sapiens cutC copper transporter (CUTC), mRNA [NM_015960]	-0.7250765	0.012617188
A_33_P3387646	*WASH1*	Homo sapiens WAS protein family homolog 1 (WASH1), mRNA [NM_182905]	0.92027235	0.01506669
A_33_P3330498	*PVRL3*	Homo sapiens poliovirus receptor-related 3 (PVRL3), transcript variant 3, mRNA [NM_001243288]	-0.24664478	0.010986003
A_32_P220696	*LOC643201*	Homo sapiens centrosomal protein 192kDa pseudogene (LOC643201), non-coding RNA [NR_036494]	0.89810854	0.012864231
A_33_P3423825	*ALDH7A1*	Homo sapiens aldehyde dehydrogenase 7 family, member A1 (ALDH7A1), transcript variant 1, mRNA [NM_001182]	-15380455	0.0059536803
A_23_P255884	*TERF1*	Homo sapiens telomeric repeat binding factor (NIMA-interacting) 1 (TERF1), transcript variant 1, mRNA [NM_017489]	-0.5511532	0.014185274

A_23_P415706	*GSN*	Homo sapiens gelsolin (GSN), transcript variant 2, mRNA [NM_198252]	-13054345	0.006472185
A_23_P201086	*OLFM1*	Homo sapiens olfactomedin 1 (OLFM1), transcript variant 2, mRNA [NM_006334]	-0.84050775	0.0090693105
A_23_P151405	*GPR133*	Homo sapiens G protein-coupled receptor 133 (GPR133), mRNA [NM_198827]	-26530228	0.0043220706
A_21_P0000274	*ARF1*	Homo sapiens ADP-ribosylation factor 1 (ARF1), transcript variant 1, mRNA [NM_001024227]	0.6110081	0.0140439235
DCP_20_5	*CKAP2*	Homo sapiens cytoskeleton associated protein 2 (CKAP2), transcript variant 1, mRNA [NM_018204]	-0.6164542	0.013858067
A_33_P3862375	*SNORD95*	Homo sapiens small nucleolar RNA, C/D box 95 (SNORD95), small nucleolar RNA [NR_002591]	-0.91492295	0.0066114054
A_23_P118065	*USP45*	Homo sapiens ubiquitin specific peptidase 45 (USP45), mRNA [NM_001080481]	-0.38895693	0.005470758
A_22_P00009056	*HSD17B2*	Homo sapiens hydroxysteroid (17-beta) dehydrogenase 2 (HSD17B2), mRNA [NM_002153]	9375082	0.0025001846
A_33_P3216059	*TTC16*	Homo sapiens tetratricopeptide repeat domain 16 (TTC16), mRNA [NM_144965]	0.87038183	0.010480952
A_22_P00025578	*lnc-LEMD3-1*	Homo sapiens mRNA; cDNA DKFZp313L2229 (from clone DKFZp313L2229). [AL833114]	-197498	0.01121547
A_33_P3705884	*ASPN*	Homo sapiens asporin (ASPN), transcript variant 2, mRNA [NM_001193335]	-12216254	0.0077676554
A_23_P160297	*lnc-UNC93B1-1*	Homo sapiens mRNA; cDNA DKFZp434E145 (from clone DKFZp434E145). [AL137634]	10953625	0.009452373
A_23_P133770	*LINC00662*	long intergenic non-protein coding RNA 662 [Source:HGNC Symbol;Acc:HGNC:27122] [ENST00000561521]	-14114468	0.0065801656
A_33_P3613516	*C1orf94*	Homo sapiens chromosome 1 open reading frame 94 (C1orf94), transcript variant 2, mRNA [NM_032884]	29355464	0.0070571043
A_22_P00018375	*CCDC167*	Homo sapiens coiled-coil domain containing 167 (CCDC167), mRNA [NM_138493]	-0.8542134	0.01047417
A_23_P350451	*GATA2-AS1*	Homo sapiens cDNA: FLJ21000 fis, clone CAE03359. [AK024653]	0.77857566	0.008208044
A_19_P00322911	*lnc-AVEN-1*	Q9ARE2_FLABI (Q9ARE2) ZF-HD homeobox protein (Fragment), partial (5%) [THC2763885]	0.44367355	0.003956951
A_23_P131954	*PRDM1*	Homo sapiens PR domain containing 1, with ZNF domain (PRDM1), transcript variant 1, mRNA [NM_001198]	0.7718379	0.013460425
A_24_P135276	*TWIST2*	Homo sapiens twist family bHLH transcription factor 2 (TWIST2), transcript variant 2, mRNA [NM_057179]	-0.86588645	0.0102659585
A_24_P377489	*SNX5*	Homo sapiens sorting nexin 5 (SNX5), transcript variant 2, mRNA [NM_014426]	-0.61518025	0.011025315

A_33_P3317593	*USP42*	Homo sapiens ubiquitin specific peptidase 42 (USP42), mRNA [NM_032172]	-0.847095	0.012113696
A_21_P0005120	*CCZ1*	Homo sapiens CCZ1 vacuolar protein trafficking and biogenesis associated homolog (S. cerevisiae) (CCZ1), mRNA [NM_015622]	-0.9773938	0.0029471128
A_33_P3338693	*AP1B1*	Homo sapiens adaptor-related protein complex 1, beta 1 subunit (AP1B1), transcript variant 1, mRNA [NM_001127]	-0.53119224	0.008804243
A_33_P3369258	*SNAP25*	Homo sapiens synaptosomal-associated protein, 25kDa (SNAP25), transcript variant 1, mRNA [NM_003081]	4583374	0.0027248112
A_32_P96134	*ZNF284*	zinc finger protein 284 [Source:HGNC Symbol;Acc:HGNC:13078] [ENST00000421176]	-1007765	0.00626104
A_22_P00018617	*FGFR1OP2*	Homo sapiens FGFR1 oncogene partner 2 (FGFR1OP2), transcript variant 3, mRNA [NM_001171888]	-0.72088134	0.0138932085
A_23_P28434	*DPY19L1*	Homo sapiens dpy-19-like 1 (C. elegans) (DPY19L1), mRNA [NM_015283]	-0.7601652	0.008860189
A_23_P208373	*LOC101927027*	Homo sapiens uncharacterized LOC101927027 (LOC101927027), transcript variant 1, long non-coding RNA [NR_110204]	-0.41175613	0.00746454
A_24_P127021	*VAMP8*	Homo sapiens vesicle-associated membrane protein 8 (VAMP8), mRNA [NM_003761]	-11983976	0.004884975
A_23_P77415	*CYP2B6*	Homo sapiens cytochrome P450, family 2, subfamily B, polypeptide 6 (CYP2B6), mRNA [NM_000767]	15455935	0.007994212
A_23_P113777	*ST13*	Homo sapiens suppression of tumorigenicity 13 (colon carcinoma) (Hsp70 interacting protein) (ST13), transcript variant 1, mRNA [NM_003932]	-10130944	0.010446652
A_23_P6561	*OSGIN1*	Homo sapiens oxidative stress induced growth inhibitor 1 (OSGIN1), mRNA [NM_182981]	19953527	0.014074878
A_23_P24529	*ITGBL1*	Homo sapiens integrin, beta-like 1 (with EGF-like repeat domains) (ITGBL1), transcript variant 1, mRNA [NM_004791]	-16356268	0.0041946988
A_22_P00019045	*EBLN2*	Homo sapiens endogenous Bornavirus-like nucleoprotein 2 (EBLN2), mRNA [NM_018029]	-11556824	0.007501246
A_33_P3290179	*C11orf57*	Homo sapiens chromosome 11 open reading frame 57 (C11orf57), transcript variant 1, mRNA [NM_018195]	-1021646	0.0065142275
A_33_P3257528	*MIR181A2HG*	Homo sapiens MIR181A2 host gene (non-protein coding) (MIR181A2HG), long non-coding RNA [NR_038975]	-16462723	0.009811661
A_23_P21409	*DEGS1*	Homo sapiens delta(4)-desaturase, sphingolipid 1 (DEGS1), mRNA [NM_003676]	-0.7716446	0.009792738
A_23_P55518	*ZNF451*	Homo sapiens zinc finger protein 451 (ZNF451), transcript variant 2, mRNA [NM_015555]	-0.49532342	0.014728144

A_23_P86570	*CEP63*	Homo sapiens centrosomal protein 63kDa (CEP63), transcript variant 4, mRNA [NM_001042384]	-10431895	0.0062310593
A_23_P144896	*SMAD7*	Homo sapiens SMAD family member 7 (SMAD7), transcript variant 1, mRNA [NM_005904]	0.90667003	0.016038679
A_33_P3218625	*ANXA7*	Homo sapiens annexin A7 (ANXA7), transcript variant 2, mRNA [NM_004034]	-0.7260573	0.0059217354
A_22_P00004317	*PDLIM7*	Homo sapiens PDZ and LIM domain 7 (enigma) (PDLIM7), transcript variant 1, mRNA [NM_005451]	12410979	0.0074561136
A_22_P00011317	*CACFD1*	Homo sapiens calcium channel flower domain containing 1 (CACFD1), transcript variant 3, mRNA [NM_001242369]	-10554509	0.011371365
A_23_P101342	*PSMD5-AS1*	Homo sapiens PSMD5 antisense RNA 1 (head to head) (PSMD5-AS1), long non-coding RNA [NR_024408]	-1805978	0.0025001846
A_24_P943781	*LOC101926940*	Homo sapiens uncharacterized LOC101926940 (LOC101926940), long non-coding RNA [NR_104632]	0.5676551	0.0122020515
A_21_P0014351	*ATG4D*	Homo sapiens autophagy related 4D, cysteine peptidase (ATG4D), transcript variant 1, mRNA [NM_032885]	0.95662236	0.0067755687
A_33_P3211804	*CPED1*	Homo sapiens cadherin-like and PC-esterase domain containing 1 (CPED1), transcript variant 1, mRNA [NM_024913]	-0.3651597	0.014183129
A_21_P0004310	*SP2-AS1*	Homo sapiens SP2 antisense RNA 1 (SP2-AS1), transcript variant 1, long non-coding RNA [NR_103856]	-14556434	0.010033392
A_22_P00014972	*RUNX1*	Homo sapiens runt-related transcription factor 1 (RUNX1), transcript variant 2, mRNA [NM_001001890]	-0.590578	0.012082841
A_23_P214743	*lnc-ARHGAP26-4*	LNCipedia lincRNA (lnc-ARHGAP26-4), lincRNA [lnc-ARHGAP26-4:4]	0.6220319	0.008440512
A_24_P219114	*LOC101928156*	PREDICTED: Homo sapiens uncharacterized LOC101928156 (LOC101928156), ncRNA [XR_241404]	-0.73905104	0.014625823
A_23_P106844	*SIM1*	Homo sapiens single-minded family bHLH transcription factor 1 (SIM1), mRNA [NM_005068]	-15548189	0.012525387
A_23_P119040	*SEL1L*	Homo sapiens sel-1 suppressor of lin-12-like (C. elegans) (SEL1L), transcript variant 1, mRNA [NM_005065]	-11481023	0.01704795
A_23_P91015	*MT2A*	Homo sapiens metallothionein 2A (MT2A), mRNA [NM_005953]	12110124	0.010288888
A_33_P3264895	*GREB1L*	Homo sapiens growth regulation by estrogen in breast cancer-like (GREB1L), mRNA [NM_001142966]	28721297	0.0057247286

A_23_P92320	*SMYD5*	Homo sapiens SMYD family member 5 (SMYD5), mRNA [NM_006062]	11326939	0.0116860205
A_33_P3306504	*RHEBL1*	Homo sapiens Ras homolog enriched in brain like 1 (RHEBL1), transcript variant 2, mRNA [NM_001303126]	-12924998	0.0034534817
A_24_P385134	*NUP54*	Homo sapiens nucleoporin 54kDa (NUP54), transcript variant 1, mRNA [NM_017426]	-0.48193428	0.0052084886
A_21_P0002805	*ISYNA1*	Homo sapiens inositol-3-phosphate synthase 1 (ISYNA1), transcript variant 1, mRNA [NM_016368]	16254892	0.012177928
A_24_P98411	*SCD5*	Homo sapiens stearoyl-CoA desaturase 5 (SCD5), transcript variant 1, mRNA [NM_001037582]	-0.8551442	0.008488166
A_23_P433152	*LOC102724479*	PREDICTED: Homo sapiens uncharacterized LOC102724479 (LOC102724479), ncRNA [XR_427436]	-12420065	0.012928972
A_23_P403081	*HSPA5*	Homo sapiens heat shock 70kDa protein 5 (glucose-regulated protein, 78kDa) (HSPA5), mRNA [NM_005347]	-0.9742093	0.007264689
A_24_P35891	*C4orf33*	Homo sapiens chromosome 4 open reading frame 33 (C4orf33), transcript variant 1, mRNA [NM_173487]	-0.8065793	0.010397241
A_24_P235266	*C5orf34*	Homo sapiens chromosome 5 open reading frame 34 (C5orf34), mRNA [NM_198566]	-0.49910963	0.012368307
A_23_P34537	*ZNF219*	Homo sapiens zinc finger protein 219 (ZNF219), transcript variant 1, mRNA [NM_016423]	-17607503	0.0033065486
A_22_P00014497	*GRB10*	Homo sapiens growth factor receptor-bound protein 10 (GRB10), transcript variant 4, mRNA [NM_001001555]	-1740356	0.0037583082
A_23_P152087	*EPHX1*	Homo sapiens epoxide hydrolase 1, microsomal (xenobiotic) (EPHX1), transcript variant 1, mRNA [NM_000120]	16206445	0.003365343
A_33_P3311750	*lnc-SHISA9-1*	Q9W099_DROME (Q9W099) CG18171-PA, partial (3%) [THC2634032]	0.38187823	0.0126507105
A_23_P413641	*RMDN3*	Homo sapiens regulator of microtubule dynamics 3 (RMDN3), mRNA [NM_018145]	-0.59128094	0.016424606
A_33_P3397233	*CCDC144A*	Homo sapiens coiled-coil domain containing 144A (CCDC144A), transcript variant 1, mRNA [NM_014695]	-0.8829262	0.013746894
A_24_P241276	*PREX1*	Homo sapiens phosphatidylinositol-3,4,5-trisphosphate-dependent Rac exchange factor 1 (PREX1), mRNA [NM_020820]	-13236969	0.008459822
A_23_P109171	*LOC101929256*	PREDICTED: Homo sapiens uncharacterized LOC101929256 (LOC101929256), ncRNA [XR_242748]	-13249136	0.006595777
A_21_P0000598	*EXOSC8*	Homo sapiens exosome component 8 (EXOSC8), mRNA [NM_181503]	-0.7677574	0.0042201984

A_22_P00020469	*BFSP1*	Homo sapiens beaded filament structural protein 1, filensin (BFSP1), transcript variant 1, mRNA [NM_001195]	0.44013298	0.01564335
A_33_P3321369	*FBXW4*	Homo sapiens F-box and WD repeat domain containing 4 (FBXW4), mRNA [NM_022039]	-0.55480486	0.0028913463
A_23_P136355	*lnc-OLFML2A-1*	Q8NE53_HUMAN (Q8NE53) Zinedin, partial (3%) [THC2628554]	10487019	0.009808998
A_23_P162807	*HHAT*	Homo sapiens hedgehog acyltransferase (HHAT), transcript variant 1, mRNA [NM_018194]	-19261504	0.010667602
A_21_P0014314	*ULK4*	Homo sapiens unc-51 like kinase 4 (ULK4), mRNA [NM_017886]	-18012904	0.0072648157
A_33_P3280796	*MRPS31*	Homo sapiens mitochondrial ribosomal protein S31 (MRPS31), mRNA [NM_005830]	-0.7551505	0.0064857528
A_32_P169735	*lnc-ZSCAN10-3*	LNCipedia lincRNA (lnc-ZSCAN10-3), lincRNA [lnc-ZSCAN10-3:4]	-0.8859565	0.009924457
A_21_P0000397	*LMO7*	PREDICTED: Homo sapiens LIM domain 7 (LMO7), transcript variant X22, mRNA [XM_005266396]	-0.8686936	0.0118191205
A_21_P0013250	*TTC8*	Homo sapiens tetratricopeptide repeat domain 8 (TTC8), transcript variant 1, mRNA [NM_144596]	-0.307014	0.011376762
A_23_P1280	*SCARNA18*	Homo sapiens small Cajal body-specific RNA 18 (SCARNA18), guide RNA [NR_003139]	-0.49794695	0.009170467
A_23_P77493	*XLOC_l2_013485*	BROAD Institute lincRNA (XLOC_l2_013485), lincRNA [TCONS_l2_00026055]	-0.81145257	0.004437345
A_22_P00011346	*ASCC1*	Homo sapiens activating signal cointegrator 1 complex subunit 1 (ASCC1), transcript variant 1, mRNA [NM_001198799]	-0.8488505	0.005142579
A_24_P73730	*TUBB3*	Homo sapiens tubulin, beta 3 class III (TUBB3), transcript variant 1, mRNA [NM_006086]	10555241	0.0068968083
A_23_P500734	*lnc-OTUD7B-1*	LNCipedia lincRNA (lnc-OTUD7B-1), lincRNA [lnc-OTUD7B-1:1]	-0.91325665	0.012095497
A_33_P3226492	*CCDC113*	Homo sapiens coiled-coil domain containing 113 (CCDC113), transcript variant 1, mRNA [NM_014157]	-0.8876358	0.010133606
A_23_P571	*POGZ*	Homo sapiens pogo transposable element with ZNF domain (POGZ), transcript variant 1, mRNA [NM_015100]	-0.92994785	0.004573394
A_24_P223124	*RAB7A*	Homo sapiens PRO2706 mRNA, complete cds. [AF119891]	0.4618541	0.015931543
A_33_P3414789	*SLC2A1*	Homo sapiens solute carrier family 2 (facilitated glucose transporter), member 1 (SLC2A1), mRNA [NM_006516]	-0.6611955	0.008216877
A_23_P74887	*FNDC3B*	Homo sapiens fibronectin type III domain containing 3B (FNDC3B), transcript variant 1, mRNA [NM_022763]	-0.8976803	0.010370596

A_33_P3317543	*FSD1*	Homo sapiens fibronectin type III and SPRY domain containing 1 (FSD1), mRNA [NM_024333]	15787476	0.004158264
A_24_P886040	*SDC3*	Homo sapiens syndecan 3 (SDC3), mRNA [NM_014654]	11362184	0.004577453
A_33_P3401701	*GTF2IRD2B*	Homo sapiens GTF2I repeat domain containing 2B (GTF2IRD2B), mRNA [NM_001003795]	-0.75620437	0.0065142275
A_22_P00019571	*DCP2*	Homo sapiens decapping mRNA 2 (DCP2), transcript variant 1, mRNA [NM_152624]	-12294319	0.0053449664
A_33_P3379916	*EDNRA*	Homo sapiens endothelin receptor type A (EDNRA), transcript variant 1, mRNA [NM_001957]	13057451	0.011788845
A_23_P153236	*SNAPC1*	Homo sapiens small nuclear RNA activating complex, polypeptide 1, 43kDa (SNAPC1), mRNA [NM_003082]	-11107923	0.00460523
A_24_P912372	*GLS*	Homo sapiens glutaminase (GLS), transcript variant 1, mRNA [NM_014905]	-27874238	0.0025001846
A_33_P3410194	*CHMP2A*	Homo sapiens charged multivesicular body protein 2A (CHMP2A), transcript variant 2, mRNA [NM_014453]	-0.9801788	0.007206317
A_33_P3231140	*KIAA0754*	Homo sapiens KIAA0754 (KIAA0754), mRNA [NM_015038]	-0.84226716	0.009483101
A_24_P225534	*ANKRD50*	Homo sapiens ankyrin repeat domain 50 (ANKRD50), transcript variant 2, mRNA [NM_001167882]	-11556399	0.0034766807
A_33_P3278801	*RHBDL2*	Homo sapiens rhomboid, veinlet-like 2 (Drosophila) (RHBDL2), mRNA [NM_017821]	-0.76596487	0.012001763
A_23_P360874	*SENP5*	Homo sapiens SUMO1/sentrin specific peptidase 5 (SENP5), mRNA [NM_152699]	-12246804	0.015431974
A_33_P3398533	*LRCH3*	Homo sapiens cDNA FLJ44976 fis, clone BRAWH3001833. [AK128758]	0.89164495	0.00752686
A_33_P3319870	*LRWD1*	Homo sapiens leucine-rich repeats and WD repeat domain containing 1 (LRWD1), mRNA [NM_152892]	-0.23822	0.014660574
A_23_P253484	*PROSER1*	Homo sapiens proline and serine rich 1 (PROSER1), mRNA [NM_025138]	0.67521334	0.014580026
A_23_P140057	*GREM1*	Homo sapiens gremlin 1, DAN family BMP antagonist (GREM1), transcript variant 2, mRNA [NM_001191323]	-0.7295272	0.009543431
A_21_P0010026	*AADAT*	Homo sapiens aminoadipate aminotransferase (AADAT), transcript variant 1, mRNA [NM_016228]	-0.67801946	0.009679106
A_22_P00007299	*TNFRSF19*	Homo sapiens tumor necrosis factor receptor superfamily, member 19 (TNFRSF19), transcript variant 1, mRNA [NM_018647]	14342629	0.004701671
A_24_P154948	*lnc-GGTLC1-4*	LNCipedia lincRNA (lnc-GGTLC1-4), lincRNA [lnc-GGTLC1-4:1]	0.7134428	0.007901905

A_32_P103291	*GPR37L1*	G protein-coupled receptor 37 like 1 [Source:HGNC Symbol;Acc:HGNC:14923] [ENST00000367282]	0.66364354	0.0120555395
A_23_P166333	*GARS*	Homo sapiens glycyl-tRNA synthetase (GARS), mRNA [NM_002047]	-0.6466033	0.0039416323
A_33_P3252915	*SMYD3*	Homo sapiens SET and MYND domain containing 3 (SMYD3), transcript variant 2, mRNA [NM_022743]	-19810302	0.0034310205
A_33_P3247988	*UFD1L*	Homo sapiens ubiquitin fusion degradation 1 like (yeast) (UFD1L), transcript variant 1, mRNA [NM_005659]	-0.44518778	0.012079458
A_22_P00004712	*RNF7*	Homo sapiens ring finger protein 7 (RNF7), transcript variant 5, non-coding RNA [NR_037702]	-0.6050749	0.0057262382
A_23_P218375	*PCSK4*	Homo sapiens proprotein convertase subtilisin/kexin type 4 (PCSK4), mRNA [NM_017573]	-0.58699495	0.013078078
A_33_P3249524	*LOC100506844*	Homo sapiens uncharacterized LOC100506844 (LOC100506844), long non-coding RNA [NR_038269]	-10122557	0.0060077314
A_23_P160787	*ITGAE*	Homo sapiens integrin, alpha E (antigen CD103, human mucosal lymphocyte antigen 1; alpha polypeptide) (ITGAE), mRNA [NM_002208]	-12054462	0.005822303
A_22_P00012830	*SPDYE2*	Homo sapiens speedy/RINGO cell cycle regulator family member E2 (SPDYE2), mRNA [NM_001031618]	0.6410909	0.011297126
A_33_P3284951	*PEX14*	Homo sapiens peroxisomal biogenesis factor 14 (PEX14), mRNA [NM_004565]	-0.8954673	0.004332739
A_33_P3269879	*TMEM161B-AS1*	Homo sapiens TMEM161B antisense RNA 1 (TMEM161B-AS1), transcript variant 3, long non-coding RNA [NR_039995]	-0.4191824	0.010976066
A_22_P00024977	*MCM5*	Homo sapiens minichromosome maintenance complex component 5 (MCM5), mRNA [NM_006739]	12355671	0.0075455266
A_33_P3423401	*FAM160B2*	Homo sapiens family with sequence similarity 160, member B2 (FAM160B2), mRNA [NM_022749]	0.767701	0.007748667
A_33_P3229617	*lnc-CETP-1*	Homo sapiens, clone IMAGE:4426859, mRNA. [BC015447]	-10671252	0.0072718766
A_33_P3281930	*ZNF790*	zinc finger protein 790 [Source:HGNC Symbol;Acc:HGNC:33114] [ENST00000356725]	-0.9514496	0.008857327
A_23_P72503	*SGCE*	Homo sapiens sarcoglycan, epsilon (SGCE), transcript variant 1, mRNA [NM_001099401]	-0.8333092	0.004994266
A_33_P3280965	*LRRFIP2*	Homo sapiens leucine rich repeat (in FLII) interacting protein 2 (LRRFIP2), transcript variant 1, mRNA [NM_006309]	-0.8291464	0.006513625

A_21_P0009551	*KLHL2*	Homo sapiens kelch-like family member 2 (KLHL2), transcript variant 1, mRNA [NM_007246]	-11798345	0.007929221
A_23_P156445	*SNHG9*	Homo sapiens small nucleolar RNA host gene 9 (non-protein coding) (SNHG9), long non-coding RNA [NR_003142]	-0.57710236	0.008045824
A_24_P419211	*lnc-RIT2-1*	LNCipedia lincRNA (lnc-RIT2-1), lincRNA [lnc-RIT2-1:1]	0.5438392	0.0044371844
A_33_P3214432	*DDX43*	Homo sapiens DEAD (Asp-Glu-Ala-Asp) box polypeptide 43 (DDX43), mRNA [NM_018665]	-11234521	0.009560995
A_23_P32165	*MTMR6*	Homo sapiens myotubularin related protein 6 (MTMR6), mRNA [NM_004685]	-0.36604267	0.013143453
A_33_P3252785	*ZC3HAV1L*	Homo sapiens zinc finger CCCH-type, antiviral 1-like (ZC3HAV1L), mRNA [NM_080660]	0.9101162	0.014951782
A_22_P00021513	*LHX2*	Homo sapiens LIM homeobox 2 (LHX2), mRNA [NM_004789]	-31302924	0.005098191
A_23_P90311	*PLAC9*	placenta-specific 9 [Source:HGNC Symbol;Acc:HGNC:19255] [ENST00000372263]	-35893383	0.0025001846
A_33_P3795707	*TICAM1*	Homo sapiens toll-like receptor adaptor molecule 1 (TICAM1), mRNA [NM_182919]	-0.95213795	0.0025001846
A_33_P3270485	*APCDD1L-AS1*	Homo sapiens APCDD1L antisense RNA 1 (head to head) (APCDD1L-AS1), long non-coding RNA [NR_034147]	0.9084846	0.006906742
A_19_P00322962	*SNORA16B*	CU457294 Homo sapiens ORESTES from keratinocytes Homo sapiens cDNA, mRNA sequence [CU457294]	-0.59988505	0.014426552
A_23_P127964	*CBX6*	chromobox homolog 6 [Source:HGNC Symbol;Acc:HGNC:1556] [ENST00000407418]	-0.61925584	0.013587454
A_23_P59022	*SPIN3*	Homo sapiens spindlin family, member 3 (SPIN3), transcript variant 2, non-coding RNA [NR_027139]	-0.79928005	0.0038144474
A_23_P21485	*PRCP*	Homo sapiens prolylcarboxypeptidase (angiotensinase C) (PRCP), transcript variant 2, mRNA [NM_199418]	-0.878647	0.005428736
A_22_P00001695	*TRERF1*	Homo sapiens transcriptional regulating factor 1 (TRERF1), transcript variant 2, mRNA [NM_033502]	-0.47529915	0.015276223
A_33_P3238323	*PID1*	Homo sapiens phosphotyrosine interaction domain containing 1 (PID1), transcript variant 1, mRNA [NM_017933]	-0.915194	0.007093148
A_33_P3346669	*RBCK1*	Homo sapiens RanBP-type and C3HC4-type zinc finger containing 1 (RBCK1), transcript variant 2, mRNA [NM_031229]	11583326	0.0065142275
A_33_P3544887	*C19orf12*	Homo sapiens chromosome 19 open reading frame 12 (C19orf12), transcript variant 2, mRNA [NM_031448]	-0.65543586	0.014345706

A_23_P501754	*PLCE1*	Homo sapiens phospholipase C, epsilon 1 (PLCE1), transcript variant 1, mRNA [NM_016341]	-2104514	0.008031949
A_21_P0010950	*TTC28*	Homo sapiens tetratricopeptide repeat domain 28 (TTC28), mRNA [NM_001145418]	3099496	0.010272991
A_23_P45851	*CSF3*	Homo sapiens colony stimulating factor 3 (granulocyte) (CSF3), transcript variant 1, mRNA [NM_000759]	2412849	0.0117289275
A_33_P3268124	*lnc-SFTPD-2*	LNCipedia lincRNA (lnc-SFTPD-2), lincRNA [lnc-SFTPD-2:9]	-0.91964275	0.008423122
A_33_P3271241	*HIAT1*	Homo sapiens hippocampus abundant transcript 1 (HIAT1), mRNA [NM_033055]	-0.9103644	0.006352343
A_23_P124024	*ZNF654*	Homo sapiens zinc finger protein 654 (ZNF654), mRNA [NM_018293]	-0.8319854	0.003365343
A_22_P00017772	*PPA1*	Homo sapiens pyrophosphatase (inorganic) 1 (PPA1), mRNA [NM_021129]	-0.64431477	0.01211171
A_33_P3293858	*MED10*	Homo sapiens mediator complex subunit 10 (MED10), mRNA [NM_032286]	-0.3216643	0.014260212
A_33_P3328736	*lnc-YWHAZ-1*	Homo sapiens full length insert cDNA clone ZD79H01. [AF086429]	-11953465	0.008755959
A_22_P00023546	*MTUS2*	Homo sapiens microtubule associated tumor suppressor candidate 2 (MTUS2), transcript variant 1, mRNA [NM_001033602]	-27432017	0.0048452313
A_23_P127579	*CCDC23*	Homo sapiens coiled-coil domain containing 23 (CCDC23), mRNA [NM_199342]	-0.9451263	0.0037353234
A_23_P56505	*lnc-PANK4-1*	UI-E-CI1-afo-j-24-0-UI.r1 UI-E-CI1 Homo sapiens cDNA clone UI-E-CI1-afo-j-24-0-UI 5', mRNA sequence [BM694243]	0.7181048	0.016435314
A_33_P3229177	*PTS*	Homo sapiens 6-pyruvoyltetrahydropterin synthase (PTS), mRNA [NM_000317]	-0.8267558	0.010383267
A_21_P0001152	*ITGA4*	Homo sapiens integrin, alpha 4 (antigen CD49D, alpha 4 subunit of VLA-4 receptor) (ITGA4), mRNA [NM_000885]	-2000989	0.004701671
A_32_P141768	*POLE4*	Homo sapiens polymerase (DNA-directed), epsilon 4, accessory subunit (POLE4), mRNA [NM_019896]	-0.55928034	0.012199728
A_23_P3956	*lnc-TMED5-1*	LNCipedia lincRNA (lnc-TMED5-1), lincRNA [lnc-TMED5-1:21]	-0.6853254	0.010822477
A_24_P350307	*AGPAT4*	Homo sapiens 1-acylglycerol-3-phosphate O-acyltransferase 4 (AGPAT4), mRNA [NM_020133]	-10755209	0.008921625
A_33_P3357678	*C1QTNF1*	Homo sapiens C1q and tumor necrosis factor related protein 1 (C1QTNF1), transcript variant 4, mRNA [NM_198594]	34404528	0.003190129
A_21_P0010545	*LCTL*	Homo sapiens lactase-like (LCTL), transcript variant 1, mRNA [NM_207338]	-17897328	0.0057647754

A_23_P342744	*TACC1*	Homo sapiens transforming, acidic coiled-coil containing protein 1 (TACC1), transcript variant 1, mRNA [NM_006283]	-0.4508069	0.0120937945
A_23_P21747	*LAMTOR5-AS1*	Homo sapiens LAMTOR5 antisense RNA 1 (LAMTOR5-AS1), long non-coding RNA [NR_102697]	-0.9412694	0.008355834
A_33_P3372297	*LIX1L*	Homo sapiens Lix1 homolog (chicken) like (LIX1L), mRNA [NM_153713]	-0.9281332	0.013211504
A_23_P200936	*CABP5*	Homo sapiens calcium binding protein 5 (CABP5), mRNA [NM_019855]	0.63060117	0.007479662
A_33_P3268487	*ABR*	Homo sapiens active BCR-related (ABR), transcript variant 3, mRNA [NM_001159746]	0.65559053	0.0147644505
A_23_P170337	*MTR*	Homo sapiens 5-methyltetrahydrofolate-homocysteine methyltransferase (MTR), transcript variant 1, mRNA [NM_000254]	-0.35401243	0.009626979
A_23_P103703	*ABAT*	Homo sapiens 4-aminobutyrate aminotransferase (ABAT), transcript variant 2, mRNA [NM_000663]	-15934018	0.0032554762
A_23_P99226	*ALDH4A1*	Homo sapiens aldehyde dehydrogenase 4 family, member A1 (ALDH4A1), transcript variant P5CDhL, mRNA [NM_003748]	-0.7947132	0.009629584
A_32_P86150	*HSPB7*	Homo sapiens heat shock 27kDa protein family, member 7 (cardiovascular) (HSPB7), mRNA [NM_014424]	-21539776	0.0033094941
A_23_P212497	*SIRT4*	Homo sapiens sirtuin 4 (SIRT4), mRNA [NM_012240]	-13326803	0.0053870557
A_23_P46315	*CTRB2*	Homo sapiens chymotrypsinogen B2 (CTRB2), mRNA [NM_001025200]	0.875751	0.009468359
A_33_P3326817	*ACAD11*	Homo sapiens acyl-CoA dehydrogenase family, member 11 (ACAD11), mRNA [NM_032169]	-0.2977152	0.010606769
A_33_P3383816	*DENND2C*	DENN/MADD domain containing 2C [Source:HGNC Symbol;Acc:HGNC:24748] [ENST00000393277]	-11240361	0.008559404
A_33_P3369956	*LINC00909*	Homo sapiens long intergenic non-protein coding RNA 909 (LINC00909), long non-coding RNA [NR_024484]	-11068531	0.0072763516
A_33_P3387696	*PHYKPL*	Homo sapiens 5-phosphohydroxy-L-lysine phospho-lyase (PHYKPL), transcript variant 2, mRNA [NM_153373]	-0.7700703	0.011778318
A_24_P58054	*ATXN2L*	Homo sapiens ataxin 2-like (ATXN2L), transcript variant E, mRNA [NM_148416]	10351746	0.005344626
A_21_P0000404	*TMBIM4*	Homo sapiens transmembrane BAX inhibitor motif containing 4 (TMBIM4), transcript variant 1, mRNA [NM_001282606]	-10649515	0.003850731
A_33_P3394727	*SLC9A8*	Homo sapiens solute carrier family 9, subfamily A (NHE8, cation proton antiporter 8), member 8 (SLC9A8), transcript variant 2, mRNA [NM_015266]	-0.6753603	0.0117289275

A_23_P403588	*SNORD114-7*	Homo sapiens small nucleolar RNA, C/D box 114-7 (SNORD114-7), small nucleolar RNA [NR_003199]	0.87410134	0.012416433
A_33_P330894 9	*KHDRBS3*	Homo sapiens KH domain containing, RNA binding, signal transduction associated 3 (KHDRBS3), mRNA [NM_006558]	-10684873	0.007521825 4
A_22_P000241 27	*USP47*	Homo sapiens ubiquitin specific peptidase 47 (USP47), transcript variant 2, mRNA [NM_017944]	-0.58998513	0.006595015 5
A_33_P332077 2	*DBT*	Homo sapiens dihydrolipoamide branched chain transacylase E2 (DBT), mRNA [NM_001918]	-14097983	0.006433792 4
A_33_P336356 0	*ATXN1*	Homo sapiens ataxin 1 (ATXN1), transcript variant 1, mRNA [NM_000332]	-11089231	0.005726238 2
A_33_P332049 9	*RNF214*	Homo sapiens ring finger protein 214 (RNF214), transcript variant 1, mRNA [NM_001077239]	-0.55731493	0.008159728
A_33_P337408 5	*TMEM51*	Homo sapiens transmembrane protein 51 (TMEM51), transcript variant 1, mRNA [NM_001136216]	1512965	0.004288771
A_21_P001149 4	*RAI1*	Homo sapiens retinoic acid induced 1 (RAI1), mRNA [NM_030665]	13659792	0.016642986
A_23_P202345	*LRRFIP1*	Homo sapiens leucine rich repeat (in FLII) interacting protein 1 (LRRFIP1), transcript variant 3, mRNA [NM_001137552]	-0.8162816	0.010435922
A_22_P000118 80	*XLOC_l2_0056 06*	BROAD Institute lincRNA (XLOC_l2_005606), lincRNA [TCONS_l2_00010379]	0.46887767	0.009639497
A_23_P341700	*ADO*	Homo sapiens 2-aminoethanethiol (cysteamine) dioxygenase (ADO), mRNA [NM_032804]	-11237254	0.008750267
A_22_P000034 65	*DNM3OS*	Homo sapiens DNM3 opposite strand/antisense RNA (DNM3OS), transcript variant 2, long non-coding RNA [NR_103486]	-0.6992638	0.011959745
A_23_P761	*ZNF484*	Homo sapiens zinc finger protein 484 (ZNF484), transcript variant 2, mRNA [NM_001007101]	-0.71516824	0.007576738 5
A_24_P357726	*MMP25-AS1*	Homo sapiens MMP25 antisense RNA 1 (MMP25-AS1), long non-coding RNA [NR_123723]	1626323	0.008909963
A_21_P000084 7	*PSMB4*	Homo sapiens proteasome (prosome, macropain) subunit, beta type, 4 (PSMB4), mRNA [NM_002796]	-0.36530855	0.00942855
A_24_P46334	*PSG8*	Homo sapiens pregnancy specific beta-1-glycoprotein 8 (PSG8), transcript variant 1, mRNA [NM_182707]	2351818	0.013823056
A_24_P412734	*LOC100630918*	Homo sapiens uncharacterized LOC100630918 (LOC100630918), long non-coding RNA [NR_038942]	-0.6025608	0.008647297

A_33_P3396951	*SCOC*	Homo sapiens short coiled-coil protein (SCOC), transcript variant 4, mRNA [NM_032547]	-0.80863094	0.0030069214
A_33_P3210900	*PRSS36*	Homo sapiens protease, serine, 36 (PRSS36), transcript variant 1, mRNA [NM_173502]	0.6863524	0.008017601
A_33_P3780901	*BNIP3L*	Homo sapiens BCL2/adenovirus E1B 19kDa interacting protein 3-like (BNIP3L), mRNA [NM_004331]	-0.42312026	0.00800842
A_22_P00008731	*SEC13*	SEC13 homolog (S. cerevisiae) [Source:HGNC Symbol;Acc:HGNC:10697] [ENST00000397099]	0.91988295	0.014904488
A_22_P00006143	*SBNO1*	Homo sapiens strawberry notch homolog 1 (Drosophila) (SBNO1), transcript variant 1, mRNA [NM_001167856]	-0.9238665	0.007324666
A_21_P0006548	*lnc-FAM189A1-3*	Homo sapiens mRNA full length insert cDNA clone EUROIMAGE 295344. [AL109707]	-0.27188942	0.015492334
A_23_P302672	*NFATC4*	Homo sapiens nuclear factor of activated T-cells, cytoplasmic, calcineurin-dependent 4 (NFATC4), transcript variant 1, mRNA [NM_001136022]	-0.8825493	0.014821658
A_23_P83438	*lnc-FAM70A-1*	LNCipedia lincRNA (lnc-FAM70A-1), lincRNA [lnc-FAM70A-1:5]	-0.7240431	0.0053998437
A_22_P00024273	*DDIT4L*	Homo sapiens DNA-damage-inducible transcript 4-like, mRNA (cDNA clone MGC:9960 IMAGE:3877854), complete cds. [BC013592]	0.74252623	0.008427824
A_23_P215227	*UBE2Z*	Homo sapiens ubiquitin-conjugating enzyme E2Z (UBE2Z), mRNA [NM_023079]	-0.7464883	0.008570872
A_23_P88626	*DNAJB6*	Homo sapiens DnaJ (Hsp40) homolog, subfamily B, member 6 (DNAJB6), transcript variant 2, mRNA [NM_005494]	-0.515213	0.010637393
A_23_P55682	*NTNG2*	Homo sapiens netrin G2 (NTNG2), mRNA [NM_032536]	-18824922	0.0066818837
A_32_P14610	*ANPEP*	Homo sapiens alanyl (membrane) aminopeptidase (ANPEP), mRNA [NM_001150]	0.9504192	0.014125317
A_33_P3384287	*ZSCAN18*	Homo sapiens zinc finger and SCAN domain containing 18 (ZSCAN18), transcript variant 3, mRNA [NM_023926]	-0.7187097	0.011382023
A_33_P3306983	*PDLIM5*	Homo sapiens PDZ and LIM domain 5 (PDLIM5), transcript variant 1, mRNA [NM_006457]	-0.3832373	0.01211404
A_21_P0014938	*PALM*	Homo sapiens paralemmin (PALM), transcript variant 1, mRNA [NM_002579]	-13093517	0.008755959
A_22_P00013910	*C11orf31*	Homo sapiens chromosome 11 open reading frame 31 (C11orf31), mRNA [NM_170746]	-0.5001099	0.007180822
A_24_P8494	*SLC37A4*	Homo sapiens solute carrier family 37 (glucose-6-phosphate transporter), member 4 (SLC37A4), transcript variant 1, mRNA [NM_001164277]	0.5907483	0.0107158525
A_33_P3822503	*AR*	Homo sapiens androgen receptor (AR), transcript variant 1, mRNA [NM_000044]	13988717	0.0054810056

A_22_P00017937	*SEC61B*	Homo sapiens Sec61 beta subunit (SEC61B), mRNA [NM_006808]	-0.74862075	0.0077867797
A_33_P3315074	*CTF1*	Homo sapiens cardiotrophin 1 (CTF1), transcript variant 1, mRNA [NM_001330]	-15485152	0.008647297
A_23_P71908	*EIF2S3*	eukaryotic translation initiation factor 2, subunit 3 gamma, 52kDa [Source:HGNC Symbol;Acc:HGNC:3267] [ENST00000460032]	-11164021	0.015082767
A_19_P00316701	*SNAPC3*	Homo sapiens small nuclear RNA activating complex, polypeptide 3, 50kDa (SNAPC3), mRNA [NM_001039697]	-0.66291	0.0147644505
A_24_P940125	*IFT81*	Homo sapiens intraflagellar transport 81 (IFT81), transcript variant 2, mRNA [NM_031473]	-1138411	0.007210647
A_33_P3350823	*SNHG5*	Homo sapiens small nucleolar RNA host gene 5 (non-protein coding) (SNHG5), long non-coding RNA [NR_003038]	-0.32741284	0.011013887
A_23_P427502	*CNOT6*	Homo sapiens CCR4-NOT transcription complex, subunit 6 (CNOT6), mRNA [NM_001303241]	-0.31201196	0.010125467
A_33_P3366540	*ZNF639*	Homo sapiens zinc finger protein 639 (ZNF639), transcript variant 1, mRNA [NM_016331]	-0.5904292	0.011937011
A_23_P31489	*ZNRF3*	Homo sapiens zinc and ring finger 3 (ZNRF3), transcript variant 1, mRNA [NM_001206998]	0.6901036	0.012581895
A_23_P350574	*CSGALNACT1*	Homo sapiens chondroitin sulfate N-acetylgalactosaminyltransferase 1 (CSGALNACT1), transcript variant 1, mRNA [NM_001130518]	-33298807	0.016687684
A_24_P331560	*URGCP*	Homo sapiens upregulator of cell proliferation (URGCP), transcript variant 2, mRNA [NM_001077664]	0.7780435	0.013635898
A_23_P43613	*FCRLB*	Homo sapiens Fc receptor-like B (FCRLB), transcript variant 1, mRNA [NM_001002901]	-13354976	0.00556941
A_33_P3290403	*STS*	Homo sapiens steroid sulfatase (microsomal), isozyme S (STS), mRNA [NM_000351]	-0.9354925	0.010252496
A_23_P252700	*NDUFB6*	Homo sapiens NADH dehydrogenase (ubiquinone) 1 beta subcomplex, 6, 17kDa (NDUFB6), transcript variant 2, mRNA [NM_182739]	-0.6511083	0.00888951
A_24_P214598	*IMPA2*	Homo sapiens inositol(myo)-1(or 4)-monophosphatase 2 (IMPA2), mRNA [NM_014214]	13989497	0.004370471
A_23_P138168	*UNC50*	Homo sapiens unc-50 homolog (C. elegans) (UNC50), mRNA [NM_014044]	-0.6483423	0.005255513
A_33_P3474859	*PPM1K*	Homo sapiens protein phosphatase, Mg2+/Mn2+ dependent, 1K (PPM1K), mRNA [NM_152542]	-14438007	0.0031166414

A_24_P377499	*CNN3*	Homo sapiens calponin 3, acidic (CNN3), transcript variant 1, mRNA [NM_001839]	-0.8346839	0.011772038
DCP_1_1	*lnc-RP11-195B21.3.1-2*	Homo sapiens cDNA clone IMAGE:5498355. [BC110369]	0.56535405	0.0083268415
A_24_P80135	*OSBPL3*	Homo sapiens oxysterol binding protein-like 3 (OSBPL3), transcript variant 1, mRNA [NM_015550]	-0.6536341	0.006571875
A_23_P12816	*PTPN18*	Homo sapiens protein tyrosine phosphatase, non-receptor type 18 (brain-derived) (PTPN18), transcript variant 1, mRNA [NM_014369]	-0.9271512	0.0062516355
A_21_P0004566	*LOC101927326*	PREDICTED: Homo sapiens uncharacterized LOC101927326 (LOC101927326), ncRNA [XR_244183]	10167762	0.009334625
A_33_P7289027	*HELLS*	Homo sapiens helicase, lymphoid-specific (HELLS), transcript variant 1, mRNA [NM_018063]	0.4511852	0.008967493
A_23_P125348	*lnc-ARRDC3-1*	LNCipedia lincRNA (lnc-ARRDC3-1), lincRNA [lnc-ARRDC3-1:6]	0.8453836	0.007923661
A_23_P214139	*LOC401320*	Homo sapiens uncharacterized LOC401320 (LOC401320), long non-coding RNA [NR_038889]	-12978903	0.008857441
A_22_P00016741	*TRAPPC1*	Homo sapiens trafficking protein particle complex 1 (TRAPPC1), transcript variant 1, mRNA [NM_021210]	0.9963999	0.0074661016
A_33_P3423570	*REV3L*	Homo sapiens REV3-like, polymerase (DNA directed), zeta, catalytic subunit (REV3L), transcript variant 1, mRNA [NM_002912]	-1407368	0.0041475594
A_21_P0005657	*lnc-TRH-2*	ALU6_HUMAN (P39193) Alu subfamily SP sequence contamination warning entry, partial (12%) [THC2498486]	-13877255	0.01273111
A_23_P39050	*METRN*	Homo sapiens meteorin, glial cell differentiation regulator (METRN), mRNA [NM_024042]	-10706367	0.005098191
A_23_P8754	*ZNF823*	Homo sapiens zinc finger protein 823 (ZNF823), transcript variant 1, mRNA [NM_001080493]	-10322272	0.012164148
A_21_P0009107	*PDCD6*	Homo sapiens programmed cell death 6 (PDCD6), transcript variant 6, non-coding RNA [NR_073609]	-10538185	0.009765222
A_33_P3226832	*AASS*	Homo sapiens aminoadipate-semialdehyde synthase (AASS), mRNA [NM_005763]	-19738684	0.0032224318
A_23_P40059	*lnc-CDH5-4*	LNCipedia lincRNA (lnc-CDH5-4), lincRNA [lnc-CDH5-4:2]	-12151215	0.013441296
A_23_P25964	*F3*	Homo sapiens coagulation factor III (thromboplastin, tissue factor) (F3), transcript variant 1, mRNA [NM_001993]	-0.72454405	0.007952653

A_33_P3235360	*PMS1*	Homo sapiens PMS1 postmeiotic segregation increased 1 (S. cerevisiae) (PMS1), transcript variant 1, mRNA [NM_000534]	-0.5836951	0.01089484
A_33_P3312842	*GALC*	Homo sapiens galactosylceramidase (GALC), transcript variant 1, mRNA [NM_000153]	-10105256	0.0051764864
A_21_P0000315	*ZNRF2*	Homo sapiens zinc and ring finger 2, E3 ubiquitin protein ligase (ZNRF2), mRNA [NM_147128]	-0.8935409	0.013808693
A_23_P52727	*GAS5*	Homo sapiens growth arrest-specific 5 (non-protein coding) (GAS5), long non-coding RNA [NR_002578]	-0.774703	0.0095815705
A_22_P00016146	*SNORA18*	Homo sapiens small nucleolar RNA, H/ACA box 18 (SNORA18), small nucleolar RNA [NR_002959]	-14889481	0.0037353234
A_33_P3330264	*NAV2*	Homo sapiens neuron navigator 2 (NAV2), transcript variant 1, mRNA [NM_182964]	-0.87172073	0.009778202
A_24_P59667	*lnc-TIMM9-2*	Homo sapiens mRNA; cDNA DKFZp781K21161 (from clone DKFZp781K21161). [CR627086]	-0.9415869	0.016435314
A_33_P3255409	*CXCL1*	Homo sapiens chemokine (C-X-C motif) ligand 1 (melanoma growth stimulating activity, alpha) (CXCL1), transcript variant 1, mRNA [NM_001511]	28949049	0.00917936
A_24_P105747	*JAK3*	Homo sapiens Janus kinase 3 (JAK3), mRNA [NM_000215]	-20555387	0.0054910174
A_22_P00014860	*PIH1D1*	Homo sapiens PIH1 domain containing 1 (PIH1D1), mRNA [NM_017916]	-0.98632765	0.005958252
A_23_P500130	*AP1S3*	Homo sapiens adaptor-related protein complex 1, sigma 3 subunit (AP1S3), transcript variant 1, mRNA [NM_001039569]	0.7366269	0.0041847117
A_33_P3416563	*KANK1*	Homo sapiens KN motif and ankyrin repeat domains 1 (KANK1), transcript variant 2, mRNA [NM_153186]	-0.8518018	0.011672903
A_33_P3265075	*SOS2*	Homo sapiens son of sevenless homolog 2 (Drosophila) (SOS2), mRNA [NM_006939]	-11216519	0.005255513
A_23_P310022	*AIG1*	Homo sapiens androgen-induced 1 (AIG1), transcript variant 1, mRNA [NM_016108]	-10494356	0.0079629375
A_33_P3383149	*PAAF1*	Homo sapiens proteasomal ATPase-associated factor 1 (PAAF1), transcript variant 1, mRNA [NM_001267803]	-0.5946428	0.009543431
A_23_P84140	*KIAA1217*	Homo sapiens KIAA1217 (KIAA1217), transcript variant 1, mRNA [NM_019590]	-13396915	0.0037856582
A_21_P0011499	*ZCWPW2*	zinc finger, CW type with PWWP domain 2 [Source:HGNC Symbol;Acc:HGNC:23574] [ENST00000383768]	-0.6272345	0.01232118
A_33_P3369213	*IQCB1*	Homo sapiens IQ motif containing B1 (IQCB1), transcript variant 3, mRNA [NM_001023571]	-0.83933854	0.011846851

A_24_P832426	*LOC101928203*	Homo sapiens uncharacterized LOC101928203 (LOC101928203), long non-coding RNA [NR_110934]	14983513	0.0093763685
A_33_P3360867	*LINC00999*	Homo sapiens long intergenic non-protein coding RNA 999 (LINC00999), long non-coding RNA [NR_024497]	16514974	0.013097991
A_23_P52311	*B3GALTL*	Homo sapiens beta 1,3-galactosyltransferase-like (B3GALTL), mRNA [NM_194318]	-0.976408	0.006111203
A_23_P329152	*TAF5*	Homo sapiens TAF5 RNA polymerase II, TATA box binding protein (TBP)-associated factor, 100kDa (TAF5), mRNA [NM_006951]	-0.82576185	0.003945209
A_21_P0014134	*TRAF3IP2-AS1*	Homo sapiens TRAF3IP2 antisense RNA 1 (TRAF3IP2-AS1), transcript variant 3, long non-coding RNA [NR_034110]	-0.7451624	0.013529562
A_33_P3409154	*ILF3*	Homo sapiens interleukin enhancer binding factor 3, 90kDa (ILF3), transcript variant 1, mRNA [NM_012218]	0.7937519	0.016789315
A_23_P31064	*EVC*	PREDICTED: Homo sapiens Ellis van Creveld syndrome (EVC), transcript variant X11, misc_RNA [XR_427477]	-13453876	0.01039718
A_33_P3229390	*SLC22A23*	Homo sapiens solute carrier family 22, member 23 (SLC22A23), transcript variant 1, mRNA [NM_015482]	-27163258	0.0131854955
A_33_P3300965	*MOXD1*	Homo sapiens monooxygenase, DBH-like 1 (MOXD1), mRNA [NM_015529]	-17454121	0.01654334
A_32_P23795	*HOXC6*	Homo sapiens homeobox C6 (HOXC6), transcript variant 1, mRNA [NM_004503]	-16354373	0.005265734
A_22_P00012589	*MOSPD1*	Homo sapiens motile sperm domain containing 1 (MOSPD1), mRNA [NM_019556]	-0.5276227	0.0064337924
A_33_P3318377	*XLOC_l2_010916*	BROAD Institute lincRNA (XLOC_l2_010916), lincRNA [TCONS_l2_00020852]	-0.87310976	0.007711294
A_23_P321320	*lnc-PTPN23-1*	Homo sapiens cDNA clone IMAGE:6384488, partial cds. [BC067356]	0.7074403	0.013130262
A_24_P13475	*STK11IP*	Homo sapiens serine/threonine kinase 11 interacting protein (STK11IP), mRNA [NM_052902]	-10092031	0.007422046
A_23_P385206	*MRPL15*	Homo sapiens mitochondrial ribosomal protein L15 (MRPL15), mRNA [NM_014175]	-0.60080785	0.012512322
A_23_P200670	*SPATA13*	Homo sapiens spermatogenesis associated 13 (SPATA13), transcript variant 3, mRNA [NM_001286792]	10576129	0.005154336
A_23_P110362	*STX12*	Homo sapiens syntaxin 12 (STX12), mRNA [NM_177424]	-1129921	0.0052211415
A_21_P0003138	*WDR78*	Homo sapiens WD repeat domain 78 (WDR78), transcript variant 2, mRNA [NM_207014]	-0.9284214	0.007848112
A_23_P50897	*LAMTOR3*	Homo sapiens late endosomal/lysosomal adaptor, MAPK and MTOR activator 3	-11846287	0.0031750489

		(LAMTOR3), transcript variant 1, mRNA [NM_021970]		
A_23_P435697	*lnc-RPL24-2*	LNCipedia lincRNA (lnc-RPL24-2), lincRNA [lnc-RPL24-2:4]	0.71839654	0.012006794
A_33_P3258712	*NIFK*	Homo sapiens nucleolar protein interacting with the FHA domain of MKI67 (NIFK), mRNA [NM_032390]	-0.6847203	0.005808847
A_33_P3878772	*EDRF1*	Homo sapiens erythroid differentiation regulatory factor 1 (EDRF1), transcript variant 2, mRNA [NM_015608]	-0.835452	0.00860804
A_33_P3260634	*LOC101928000*	PREDICTED: Homo sapiens uncharacterized LOC101928000 (LOC101928000), ncRNA [XR_243583]	0.33725357	0.014448771
A_33_P3292560	*JAK2*	Homo sapiens Janus kinase 2 (JAK2), mRNA [NM_004972]	-0.7289382	0.009294179
A_24_P222835	*PLCB4*	Homo sapiens phospholipase C, beta 4 (PLCB4), transcript variant 3, mRNA [NM_001172646]	-11288198	0.0065142275
A_23_P70448	*SURF1*	Homo sapiens surfeit 1 (SURF1), transcript variant 1, mRNA [NM_003172]	-10431496	0.007066105
A_33_P3311403	*S100PBP*	Homo sapiens S100P binding protein (S100PBP), transcript variant 1, mRNA [NM_022753]	-0.69628084	0.011079763
A_21_P0002606	*HIST1H1A*	Homo sapiens histone cluster 1, H1a (HIST1H1A), mRNA [NM_005325]	10232363	0.0054418826
A_24_P397107	*MRVI1*	Homo sapiens murine retrovirus integration site 1 homolog (MRVI1), transcript variant 2, mRNA [NM_130385]	-2386129	0.0030924478
A_24_P239177	*lnc-FOXD4L1-1*	LNCipedia lincRNA (lnc-FOXD4L1-1), lincRNA [lnc-FOXD4L1-1:3]	0.8033265	0.01039718
A_21_P0012078	*CDC25A*	Homo sapiens cell division cycle 25A (CDC25A), transcript variant 1, mRNA [NM_001789]	0.8169023	0.010288888
A_32_P205553	*MUC4*	Homo sapiens mucin 4, cell surface associated (MUC4), transcript variant 1, mRNA [NM_018406]	0.5202637	0.012368504
A_23_P30283	*MIR4435-1HG*	Homo sapiens MIR4435-1 host gene (non-protein coding) (MIR4435-1HG), transcript variant 1, long non-coding RNA [NR_015395]	-0.97481513	0.006262615
A_33_P3221293	*RPL26L1*	Homo sapiens ribosomal protein L26-like 1 (RPL26L1), mRNA [NM_016093]	-10022337	0.0057806885
A_32_P507710	*FAM174A*	family with sequence similarity 174, member A [Source:HGNC Symbol;Acc:HGNC:24943] [ENST00000312637]	-11012377	0.007180822
A_23_P304450	*DFNA5*	Homo sapiens deafness, autosomal dominant 5 (DFNA5), transcript variant 1, mRNA [NM_004403]	-13328454	0.0064857528

A_23_P413803	*PI4KAP2*	Homo sapiens phosphatidylinositol 4-kinase, catalytic, alpha pseudogene 2 (PI4KAP2), non-coding RNA [NR_003700]	0.8439468	0.0061524836
A_24_P662366	*GATA6*	Homo sapiens GATA binding protein 6 (GATA6), mRNA [NM_005257]	-54877377	0.00556786
A_23_P140029	*POC5*	Homo sapiens POC5 centriolar protein (POC5), transcript variant 2, mRNA [NM_152408]	-0.7796027	0.008362636
A_23_P109768	*UBL3*	Homo sapiens ubiquitin-like 3 (UBL3), mRNA [NM_007106]	-10912744	0.004026994
A_21_P0000492	*CDH13*	Homo sapiens cadherin 13 (CDH13), transcript variant 5, mRNA [NM_001220491]	-27970285	0.002804883
A_33_P3306823	*RNF7*	Homo sapiens ring finger protein 7 (RNF7), transcript variant 1, mRNA [NM_014245]	-0.7450089	0.0078559
A_21_P0011085	*SNAR-F*	Homo sapiens small ILF3/NF90-associated RNA F (SNAR-F), small nuclear RNA [NR_004384]	-0.7031317	0.009040769
A_33_P3260342	*ZNF846*	Homo sapiens zinc finger protein 846 (ZNF846), mRNA [NM_001077624]	-0.7496075	0.009222329
A_23_P75453	*NFASC*	Homo sapiens neurofascin (NFASC), transcript variant 1, mRNA [NM_001005388]	-1510924	0.0024890031
A_23_P214798	*SULF2*	Homo sapiens sulfatase 2 (SULF2), transcript variant 1, mRNA [NM_018837]	-3529759	0.0022181747
A_23_P18413	*MEN1*	Homo sapiens multiple endocrine neoplasia I (MEN1), transcript variant e1E, mRNA [NM_130803]	0.94659257	0.016359627
A_23_P42265	*SYNCRIP*	Homo sapiens synaptotagmin binding, cytoplasmic RNA interacting protein (SYNCRIP), transcript variant 1, mRNA [NM_006372]	-0.4493241	0.013300074
A_24_P382017	*TIMMDC1*	Homo sapiens translocase of inner mitochondrial membrane domain containing 1 (TIMMDC1), mRNA [NM_016589]	-0.547072	0.0126051465
A_23_P115407	*APOM*	Homo sapiens apolipoprotein M (APOM), transcript variant 1, mRNA [NM_019101]	-0.9272021	0.008672548
A_23_P384635	*RRN3*	Homo sapiens RRN3 RNA polymerase I transcription factor homolog (S. cerevisiae) (RRN3), transcript variant 1, mRNA [NM_018427]	-0.47469395	0.012226211
A_33_P3222937	*GSTM1*	Homo sapiens glutathione S-transferase mu 1 (GSTM1), transcript variant 2, mRNA [NM_146421]	17668675	0.00393248
A_33_P3388771	*DZIP1L*	Homo sapiens DAZ interacting zinc finger protein 1-like (DZIP1L), transcript variant 1, mRNA [NM_173543]	-10310868	0.0040699136
A_22_P00016257	*TMEM214*	Homo sapiens transmembrane protein 214 (TMEM214), transcript variant 1, mRNA [NM_017727]	-0.40748456	0.007774276
A_33_P3238573	*LOC101060810*	PREDICTED: Homo sapiens zinc finger protein 492-like (LOC101060810), mRNA [XM_006710044]	10923673	0.0028703364

A_33_P3315929	*lnc-TMED5-1*	LNCipedia lincRNA (lnc-TMED5-1), lincRNA [lnc-TMED5-1:27]	-0.5933935	0.005543548
A_33_P3699445	*DENND4B*	Homo sapiens DENN/MADD domain containing 4B (DENND4B), mRNA [NM_014856]	-0.5875223	0.01270892
A_23_P68529	*PTBP3*	Homo sapiens polypyrimidine tract binding protein 3 (PTBP3), transcript variant 5, mRNA [NM_001244897]	-0.6513205	0.009429358
A_24_P148043	*ITPA*	Homo sapiens inosine triphosphatase (nucleoside triphosphate pyrophosphatase) (ITPA), transcript variant 1, mRNA [NM_033453]	-0.7254359	0.0155405095
A_23_P22671	*HHLA3*	Homo sapiens HERV-H LTR-associating 3 (HHLA3), transcript variant 3, mRNA [NM_001036646]	0.5863461	0.009588725
A_33_P3798739	*FAM20B*	Homo sapiens family with sequence similarity 20, member B (FAM20B), mRNA [NM_014864]	-12724496	0.0031941293
A_33_P3311917	*VAMP7*	Homo sapiens vesicle-associated membrane protein 7 (VAMP7), transcript variant 1, mRNA [NM_005638]	-0.9960663	0.0056601833
A_23_P213908	*LOC286382*	Homo sapiens cDNA FLJ37037 fis, clone BRACE2011611. [AK094356]	0.23789804	0.013354078
A_33_P3210278	*PHAX*	Homo sapiens phosphorylated adaptor for RNA export (PHAX), mRNA [NM_032177]	-0.636882	0.007861275
A_33_P3407742	*GLI4*	GLI family zinc finger 4 [Source:HGNC Symbol;Acc:HGNC:4320] [ENST00000523812]	10917776	0.0034310205
A_21_P0000238	*SYNE2*	Homo sapiens spectrin repeat containing, nuclear envelope 2 (SYNE2), transcript variant 5, mRNA [NM_182914]	-0.83118457	0.006946239
A_24_P171182	*PPM1B*	Homo sapiens protein phosphatase, Mg2+/Mn2+ dependent, 1B (PPM1B), transcript variant 1, mRNA [NM_002706]	-0.43453735	0.013322001
A_24_P225635	*SNORD12C*	Homo sapiens small nucleolar RNA, C/D box 12C (SNORD12C), small nucleolar RNA [NR_002433]	0.6509547	0.008420444
A_23_P55477	*ACBD3*	Homo sapiens acyl-CoA binding domain containing 3 (ACBD3), mRNA [NM_022735]	-10587658	0.003609745
A_23_P200710	*HNRNPLL*	heterogeneous nuclear ribonucleoprotein L-like [Source:HGNC Symbol;Acc:HGNC:25127] [ENST00000449105]	-0.8807292	0.012244734
A_23_P59138	*ADORA2B*	Homo sapiens adenosine A2b receptor (ADORA2B), mRNA [NM_000676]	-11021426	0.0025001846
A_24_P186986	*PIK3C2B*	Homo sapiens phosphatidylinositol-4-phosphate 3-kinase, catalytic subunit type 2 beta (PIK3C2B), mRNA [NM_002646]	-18878056	0.008559404
A_33_P3251073	*POU5F1*	Homo sapiens POU class 5 homeobox 1 (POU5F1), transcript variant 1, mRNA [NM_002701]	-11562488	0.0024890031

A_33_P3384562	*TMEM52*	Homo sapiens transmembrane protein 52 (TMEM52), mRNA [NM_178545]	-0.8673258	0.0037856582
A_23_P365738	*ZNF189*	Homo sapiens zinc finger protein 189 (ZNF189), transcript variant 2, mRNA [NM_197977]	-0.857505	0.008358917
A_33_P3419621	*PDLIM7*	Homo sapiens PDZ and LIM domain 7 (enigma) (PDLIM7), transcript variant 4, mRNA [NM_213636]	-0.51926756	0.016981635
A_21_P0011968	*ARC*	Homo sapiens activity-regulated cytoskeleton-associated protein (ARC), mRNA [NM_015193]	-23484535	0.009921474
A_33_P3269338	*LOC100289580*	Homo sapiens uncharacterized LOC100289580 (LOC100289580), long non-coding RNA [NR_103774]	13031845	0.015728846
A_23_P207367	*XLOC_l2_008270*	BROAD Institute lincRNA (XLOC_l2_008270), lincRNA [TCONS_l2_00014908]	1472102	0.012379245
A_24_P217758	*HAUS5*	Homo sapiens HAUS augmin-like complex, subunit 5 (HAUS5), mRNA [NM_015302]	-0.5498118	0.00689503
A_33_P3283213	*STAT5A*	Homo sapiens signal transducer and activator of transcription 5A (STAT5A), transcript variant 2, mRNA [NM_003152]	-10518591	0.0077676554
A_24_P76358	*MFAP3*	Homo sapiens microfibrillar-associated protein 3 (MFAP3), transcript variant 3, mRNA [NM_001242336]	-0.5361743	0.016310623
A_23_P302005	*MGC57346*	Homo sapiens ADP-ribosylation factor pseudogene (MGC57346), transcript variant 2, non-coding RNA [NR_027295]	18974935	0.0062326672
A_32_P197340	*STON1*	Homo sapiens stonin 1 (STON1), transcript variant 2, mRNA [NM_006873]	-21621141	0.012620306
A_23_P166536	*BHLHE41*	Homo sapiens basic helix-loop-helix family, member e41 (BHLHE41), mRNA [NM_030762]	-30263066	0.015948854
A_23_P157580	*ERICH2*	Homo sapiens glutamate-rich 2 (ERICH2), transcript variant 1, mRNA [NM_001290030]	-1451237	0.013054549
A_33_P3405531	*BRD1*	Homo sapiens bromodomain containing 1 (BRD1), mRNA [NM_014577]	-0.50890493	0.015505246
A_24_P98975	*SDCBP*	Homo sapiens syndecan binding protein (syntenin) (SDCBP), transcript variant 1, mRNA [NM_005625]	-0.79859656	0.007530967
A_24_P175176	*TMF1*	Homo sapiens TATA element modulatory factor 1 (TMF1), mRNA [NM_007114]	-0.4604514	0.0064359996
A_33_P3402570	*ANKRD13A*	Homo sapiens ankyrin repeat domain 13A (ANKRD13A), mRNA [NM_033121]	-0.42132854	0.009630487
A_23_P107421	*PHTF2*	Homo sapiens putative homeodomain transcription factor 2 (PHTF2), transcript variant 2, mRNA [NM_020432]	-0.38582304	0.00978742
A_33_P3266873	*CCDC59*	Homo sapiens coiled-coil domain containing 59 (CCDC59), transcript variant 1, mRNA [NM_014167]	-0.52628106	0.0053727482

A_21_P0011314	*TK1*	Homo sapiens thymidine kinase 1, soluble (TK1), mRNA [NM_003258]	-0.22244662	0.0140423635
A_21_P0003308	*MSL1*	Homo sapiens male-specific lethal 1 homolog (Drosophila) (MSL1), mRNA [NM_001012241]	-0.7509162	0.008358917
A_23_P79231	*INAFM2*	Homo sapiens InaF-motif containing 2 (INAFM2), mRNA [NM_001301268]	-0.78511137	0.012993722
A_21_P0010211	*lnc-MCCC1-1*	AF292100 RP42 protein {Homo sapiens} (exp=-1; wgp=0; cg=0), partial (34%) [THC2670542]	0.40639913	0.010951968
A_22_P00000254	*CREB1*	Homo sapiens cAMP responsive element binding protein 1 (CREB1), transcript variant B, mRNA [NM_134442]	-0.9329574	0.0044319443
A_24_P203315	*LOC101928399*	PREDICTED: Homo sapiens uncharacterized LOC101928399 (LOC101928399), transcript variant X3, ncRNA [XR_244328]	11318718	0.014297511
A_23_P142407	*CNIH2*	Homo sapiens cornichon family AMPA receptor auxiliary protein 2 (CNIH2), transcript variant 1, mRNA [NM_182553]	18367298	0.00795879
A_23_P52826	*CDK12*	Homo sapiens cyclin-dependent kinase 12 (CDK12), transcript variant 1, mRNA [NM_016507]	0.6810098	0.014681147
A_23_P101811	*ZNF101*	Homo sapiens zinc finger protein 101 (ZNF101), transcript variant 1, mRNA [NM_033204]	-0.4355876	0.013054549
A_33_P3241756	*BUD13*	Homo sapiens BUD13 homolog (S. cerevisiae) (BUD13), transcript variant 1, mRNA [NM_032725]	-0.6813598	0.00477571
A_33_P3342628	*ZNF14*	Homo sapiens zinc finger protein 14 (ZNF14), mRNA [NM_021030]	-10421443	0.0075455266
A_23_P123727	*IKBKB*	inhibitor of kappa light polypeptide gene enhancer in B-cells, kinase beta [Source:HGNC Symbol;Acc:HGNC:5960] [ENST00000517917]	-11085916	0.009898471
A_24_P49260	*HES4*	Homo sapiens hes family bHLH transcription factor 4 (HES4), transcript variant 2, mRNA [NM_021170]	0.9216599	0.0064680316
A_24_P15754	*ZCCHC6*	Homo sapiens zinc finger, CCHC domain containing 6 (ZCCHC6), transcript variant 1, mRNA [NM_024617]	-0.63092065	0.006714085
A_23_P39840	*SPTLC3*	Homo sapiens serine palmitoyltransferase, long chain base subunit 3 (SPTLC3), mRNA [NM_018327]	-30224564	0.003162355
A_23_P121222	*TOMM40*	Homo sapiens translocase of outer mitochondrial membrane 40 homolog (yeast) (TOMM40), transcript variant 3, mRNA [NM_006114]	1709966	0.0056439545
A_23_P215819	*VAMP5*	Homo sapiens vesicle-associated membrane protein 5 (VAMP5), mRNA [NM_006634]	-0.9976947	0.006776806
A_23_P81993	*RAD18*	Homo sapiens RAD18 E3 ubiquitin protein ligase (RAD18), mRNA [NM_020165]	-0.51050574	0.010390857

A_22_P00000477	*ZNF655*	Homo sapiens zinc finger protein 655 (ZNF655), transcript variant 2, mRNA [NM_024061]	0.60497123	0.0134437205
A_21_P0000478	*C6orf1*	Homo sapiens chromosome 6 open reading frame 1 (C6orf1), transcript variant 1, mRNA [NM_178508]	-0.6666932	0.0060077314
A_23_P401700	*SNORA11C*	Homo sapiens small nucleolar RNA, H/ACA box 11C (SNORA11C), small nucleolar RNA [NR_003710]	-16475204	0.013589366
A_33_P3251289	*COL4A1*	Homo sapiens collagen, type IV, alpha 1 (COL4A1), transcript variant 1, mRNA [NM_001845]	-30053904	0.004803986
A_22_P00005562	*APBB1IP*	Homo sapiens amyloid beta (A4) precursor protein-binding, family B, member 1 interacting protein (APBB1IP), mRNA [NM_019043]	-3065855	0.0056098294
A_24_P941988	*VIMP*	Homo sapiens VCP-interacting membrane protein (VIMP), transcript variant 2, mRNA [NM_018445]	-0.73040557	0.012498083
A_33_P3343106	*GATA2-AS1*	Homo sapiens GATA2 antisense RNA 1 (GATA2-AS1), long non-coding RNA [NR_125398]	16275729	0.00836265
A_33_P3300635	*SPAST*	Homo sapiens spastin (SPAST), transcript variant 1, mRNA [NM_014946]	-0.8105792	0.003365343
A_23_P62901	*ETV2*	Homo sapiens ets variant 2 (ETV2), transcript variant 1, mRNA [NM_014209]	-0.75021374	0.007944492
A_22_P00021641	*PFKFB2*	Homo sapiens 6-phosphofructo-2-kinase/fructose-2,6-biphosphatase 2 (PFKFB2), transcript variant 1, mRNA [NM_006212]	-18356719	0.0034156272
A_23_P303423	*BTG2*	Homo sapiens BTG family, member 2 (BTG2), mRNA [NM_006763]	-14859133	0.0039856653
A_33_P3579984	*lnc-TMEM160-1*	LNCipedia lincRNA (lnc-TMEM160-1), lincRNA [lnc-TMEM160-1:1]	-0.977911	0.007208255
A_23_P151710	*SLAIN2*	Homo sapiens SLAIN motif family, member 2 (SLAIN2), mRNA [NM_020846]	-0.80527055	0.014391575
A_24_P398092	*PTGER2*	Homo sapiens prostaglandin E receptor 2 (subtype EP2), 53kDa (PTGER2), mRNA [NM_000956]	0.7445047	0.010838023
A_23_P59192	*TM2D2*	Homo sapiens TM2 domain containing 2 (TM2D2), transcript variant 2, mRNA [NM_031940]	-0.41590446	0.014842539
A_23_P326204	*RAD23B*	Homo sapiens RAD23 homolog B (S. cerevisiae) (RAD23B), transcript variant 1, mRNA [NM_002874]	-0.82370657	0.0032831472
A_33_P3570223	*SNRPC*	Homo sapiens small nuclear ribonucleoprotein polypeptide C (SNRPC), transcript variant 1, mRNA [NM_003093]	-0.41746098	0.0166364

A_33_P3359984	*SGMS2*	Homo sapiens sphingomyelin synthase 2 (SGMS2), transcript variant 1, mRNA [NM_152621]	-0.9083691	0.013286371
A_24_P406601	*ACTG1P20*	Homo sapiens mRNA; cDNA DKFZp686I06131 (from clone DKFZp686I06131). [BX648289]	10605345	0.01607819
A_33_P3210338	*OLFM1*	Homo sapiens olfactomedin 1 (OLFM1), transcript variant 1, mRNA [NM_014279]	-0.99563456	0.0052883234
A_23_P118536	*MTA3*	Homo sapiens metastasis associated 1 family, member 3 (MTA3), transcript variant 3, mRNA [NM_020744]	-0.9673783	0.005377644
A_23_P385861	*RFX7*	Homo sapiens regulatory factor X, 7 (RFX7), mRNA [NM_022841]	-0.8473923	0.006772767
A_23_P422115	*SLFN12*	Homo sapiens schlafen family member 12 (SLFN12), transcript variant 1, mRNA [NM_018042]	-0.7331285	0.01504611
A_33_P3338909	*CDCA2*	Homo sapiens cell division cycle associated 2 (CDCA2), mRNA [NM_152562]	-0.7555218	0.015575268
A_33_P3362088	*C9orf116*	Homo sapiens chromosome 9 open reading frame 116 (C9orf116), transcript variant 1, mRNA [NM_001048265]	-14468348	0.013185801
A_23_P70843	*ARPC5*	Homo sapiens actin related protein 2/3 complex, subunit 5, 16kDa (ARPC5), transcript variant 2, mRNA [NM_001270439]	-0.9047909	0.00455649
A_33_P3352432	*P2RX4*	Homo sapiens purinergic receptor P2X, ligand gated ion channel, 4 (P2RX4), transcript variant 1, mRNA [NM_001256796]	-1034709	0.007103236
A_33_P3306452	*BPGM*	Homo sapiens 2,3-bisphosphoglycerate mutase (BPGM), transcript variant 2, mRNA [NM_199186]	-0.9293244	0.00856985
A_22_P00023764	*KRBOX4*	Homo sapiens KRAB box domain containing 4 (KRBOX4), transcript variant 3, mRNA [NM_001129899]	-0.76963186	0.006754764
A_33_P3665553	*NEXN-AS1*	Homo sapiens NEXN antisense RNA 1 (NEXN-AS1), long non-coding RNA [NR_103535]	16441193	0.0050497777
A_23_P92057	*MPP1*	Homo sapiens membrane protein, palmitoylated 1, 55kDa (MPP1), transcript variant 1, mRNA [NM_002436]	-0.9238495	0.0063477047
A_23_P23221	*LOC100233156*	Homo sapiens cDNA FLJ44253 fis, clone TKIDN2009092. [AK126241]	-0.9224347	0.0041847117
A_33_P3406030	*PIK3CA*	Homo sapiens phosphatidylinositol-4,5-bisphosphate 3-kinase, catalytic subunit alpha (PIK3CA), mRNA [NM_006218]	-0.7125971	0.0029355595
A_23_P138706	*GADD45A*	Homo sapiens growth arrest and DNA-damage-inducible, alpha (GADD45A), transcript variant 1, mRNA [NM_001924]	-1060587	0.0044074184
A_33_P3399090	*LRRC8C*	Homo sapiens leucine rich repeat containing 8 family, member C (LRRC8C), mRNA [NM_032270]	21766708	0.0034684502

A_24_P327499	*ADRA2A*	Homo sapiens adrenoceptor alpha 2A (ADRA2A), mRNA [NM_000681]	-23346658	0.0042764777
A_19_P00321009	*DIXDC1*	Homo sapiens DIX domain containing 1 (DIXDC1), transcript variant 1, mRNA [NM_001037954]	-2292971	0.0040243655
A_22_P00014057	*TTI1*	TELO2 interacting protein 1 [Source:HGNC Symbol;Acc:HGNC:29029] [ENST00000449821]	0.832253	0.014739808
A_33_P3245489	*LINC01133*	Homo sapiens long intergenic non-protein coding RNA 1133 (LINC01133), long non-coding RNA [NR_038849]	-359087	0.0027248112
A_33_P3288110	*ADAMTSL5*	Homo sapiens ADAMTS-like 5 (ADAMTSL5), mRNA [NM_213604]	0.9727268	0.002909564
A_33_P3210965	*DPF2*	Homo sapiens D4, zinc and double PHD fingers family 2 (DPF2), mRNA [NM_006268]	-0.65926284	0.006500368
A_23_P45108	*PKP4*	Homo sapiens plakophilin 4 (PKP4), transcript variant 2, mRNA [NM_001005476]	-12169697	0.007929221
A_21_P0000354	*TCTN1*	Homo sapiens tectonic family member 1 (TCTN1), transcript variant 1, mRNA [NM_001082538]	-12772453	0.005787274
A_33_P3271121	*QRICH1*	Homo sapiens glutamine-rich 1 (QRICH1), transcript variant 1, mRNA [NM_017730]	-0.64671963	0.0046024295
A_33_P3309643	*SCARNA8*	Homo sapiens small Cajal body-specific RNA 8 (SCARNA8), guide RNA [NR_003009]	-0.9395802	0.008392573
A_33_P3388815	*NIT2*	Homo sapiens nitrilase family, member 2 (NIT2), mRNA [NM_020202]	-0.74135876	0.014149188
A_33_P3358957	*HEBP2*	Homo sapiens heme binding protein 2 (HEBP2), mRNA [NM_014320]	-14307632	0.008320085
A_21_P0014718	*FPGT*	Homo sapiens fucose-1-phosphate guanylyltransferase (FPGT), transcript variant 1, mRNA [NM_003838]	-15782193	0.013330584
A_23_P162037	*PAPL*	Homo sapiens iron/zinc purple acid phosphatase-like protein (PAPL), mRNA [NM_001004318]	2030266	0.0038056036
A_21_P0014791	*PACRGL*	PARK2 co-regulated-like [Source:HGNC Symbol;Acc:HGNC:28442] [ENST00000467997]	-14273984	0.009295065
A_24_P226970	*ARNTL*	Homo sapiens aryl hydrocarbon receptor nuclear translocator-like (ARNTL), transcript variant 3, mRNA [NM_001030273]	-1042685	0.006405189
A_24_P330303	*ZNF365*	Homo sapiens zinc finger protein 365 (ZNF365), transcript variant A, mRNA [NM_014951]	13228014	0.004994266
A_33_P3221448	*TGFB3*	Homo sapiens transforming growth factor, beta 3 (TGFB3), mRNA [NM_003239]	-0.61736804	0.015113612
A_32_P168247	*FRMD6*	Homo sapiens FERM domain containing 6 (FRMD6), transcript variant 1, mRNA [NM_001042481]	-14565749	0.003639613
A_22_P00008304	*ZNF264*	Homo sapiens zinc finger protein 264 (ZNF264), mRNA [NM_003417]	-0.79098624	0.0045172833

A_23_P53193	*COX6A1*	Homo sapiens cytochrome c oxidase subunit VIa polypeptide 1 (COX6A1), mRNA [NM_004373]	-0.5246403	0.004829709
A_33_P3394378	*lnc-ITGAL-3*	LNCipedia lincRNA (lnc-ITGAL-3), lincRNA [lnc-ITGAL-3:1]	0.42149255	0.016047947
A_23_P215491	*SYTL2*	Homo sapiens synaptotagmin-like 2 (SYTL2), transcript variant a, mRNA [NM_032943]	0.81800056	0.00868837
A_23_P23141	*CCL24*	Homo sapiens chemokine (C-C motif) ligand 24 (CCL24), mRNA [NM_002991]	0.7832667	0.0053998437
A_23_P64954	*DOHH*	Homo sapiens deoxyhypusine hydroxylase/monooxygenase (DOHH), transcript variant 2, mRNA [NM_031304]	0.6061788	0.014260212
A_23_P431388	*H3F3A*	Homo sapiens H3 histone, family 3A (H3F3A), mRNA [NM_002107]	-0.7786603	0.005182173
A_24_P136182	*USP5*	Homo sapiens ubiquitin specific peptidase 5 (isopeptidase T) (USP5), transcript variant 2, mRNA [NM_003481]	0.97786474	0.0067838193
A_23_P33154	*SPOCD1*	Homo sapiens SPOC domain containing 1 (SPOCD1), transcript variant 1, mRNA [NM_144569]	-12011589	0.0042337696
A_21_P0003710	*RPS2P32*	Homo sapiens ribosomal protein S2 pseudogene 32 (RPS2P32), non-coding RNA [NR_026676]	-0.25631064	0.0118191205
A_24_P807031	*STAU2*	Homo sapiens staufen double-stranded RNA binding protein 2 (STAU2), transcript variant 5, mRNA [NM_014393]	-0.87939954	0.0069311536
A_23_P84448	*lnc-FAM53A-1*	LNCipedia lincRNA (lnc-FAM53A-1), lincRNA [lnc-FAM53A-1:2]	-16001389	0.016028663
A_24_P98251	*ATP6AP1L*	Homo sapiens ATPase, H+ transporting, lysosomal accessory protein 1-like (ATP6AP1L), mRNA [NM_001017971]	-11110746	0.0113136545
A_21_P0009773	*TUBA4A*	Homo sapiens tubulin, alpha 4a (TUBA4A), transcript variant 1, mRNA [NM_006000]	22438526	0.0044927304
A_22_P00023275	*VPS13B*	Homo sapiens vacuolar protein sorting 13 homolog B (yeast) (VPS13B), transcript variant 5, mRNA [NM_017890]	-0.9656326	0.008601822
A_23_P27795	*lnc-BBC3-1*	LNCipedia lincRNA (lnc-BBC3-1), lincRNA [lnc-BBC3-1:1]	-14047321	0.009244242
A_33_P3357658	*SPINT2*	Homo sapiens serine peptidase inhibitor, Kunitz type, 2 (SPINT2), transcript variant a, mRNA [NM_021102]	-37410936	0.003948561
A_23_P39034	*BNIP3*	Homo sapiens BCL2/adenovirus E1B 19kDa interacting protein 3 (BNIP3), mRNA [NM_004052]	-0.8090427	0.004016376
A_33_P3297030	*HMGA2*	Homo sapiens high mobility group AT-hook 2 (HMGA2), transcript variant 4, mRNA [NM_001300919]	10676328	0.0060101966

A_32_P164246	*SMARCA4*	Homo sapiens SWI/SNF related, matrix associated, actin dependent regulator of chromatin, subfamily a, member 4 (SMARCA4), transcript variant 3, mRNA [NM_003072]	-0.28318092	0.0093693705
A_21_P0014785	*VPS53*	vacuolar protein sorting 53 homolog (S. cerevisiae) [Source:HGNC Symbol;Acc:HGNC:25608] [ENST00000570359]	0.901842	0.008274833
A_33_P3220152	*FOXQ1*	Homo sapiens forkhead box Q1 (FOXQ1), mRNA [NM_033260]	111478	0.0054365364
A_21_P0014312	*VIM-AS1*	Homo sapiens VIM antisense RNA 1 (VIM-AS1), transcript variant 1, long non-coding RNA [NR_108061]	12740254	0.0029471128
A_23_P429998	*SBNO2*	Homo sapiens strawberry notch homolog 2 (Drosophila) (SBNO2), transcript variant 1, mRNA [NM_014963]	19032493	0.0072648157
A_33_P3353996	*SNHG19*	small nucleolar RNA host gene 19 (non-protein coding) [Source:HGNC Symbol;Acc:HGNC:49574] [ENST00000563192]	0.39646432	0.009683536
A_22_P00004142	*FOSB*	Homo sapiens FBJ murine osteosarcoma viral oncogene homolog B (FOSB), transcript variant 1, mRNA [NM_006732]	22930381	0.0029355595
A_23_P67980	*PPP1R3G*	Homo sapiens protein phosphatase 1, regulatory subunit 3G (PPP1R3G), mRNA [NM_001145115]	-12269676	0.01205956
A_33_P3254634	*lnc-CLDN6-2*	LNCipedia lincRNA (lnc-CLDN6-2), lincRNA [lnc-CLDN6-2:1]	10777383	0.007797447
A_33_P3318661	*KLF7*	Homo sapiens Kruppel-like factor 7 (ubiquitous) (KLF7), transcript variant 1, mRNA [NM_003709]	-14996433	0.0034890627
A_23_P76078	*PDIA5*	Homo sapiens protein disulfide isomerase family A, member 5 (PDIA5), transcript variant 1, mRNA [NM_006810]	-12230074	0.005255513
A_23_P218646	*E2F7*	Homo sapiens E2F transcription factor 7 (E2F7), mRNA [NM_203394]	11044129	0.0039342875
A_33_P3347055	*IL23A*	Homo sapiens interleukin 23, alpha subunit p19 (IL23A), mRNA [NM_016584]	-0.5197229	0.005947757
A_33_P3240200	*TNFRSF6B*	Homo sapiens tumor necrosis factor receptor superfamily, member 6b, decoy (TNFRSF6B), mRNA [NM_003823]	10187262	0.014799829
A_33_P3209522	*DPCD*	Homo sapiens deleted in primary ciliary dyskinesia homolog (mouse) (DPCD), mRNA [NM_015448]	-14579594	0.007092758
A_23_P145874	*LOC286068*	Homo sapiens, clone IMAGE:3909165, mRNA. [BC015720]	-0.7100996	0.013275614
A_33_P3217147	*SLFN5*	Homo sapiens schlafen family member 5 (SLFN5), mRNA [NM_144975]	-15886942	0.0098649

A_22_P00001001	*SAMD9L*	sterile alpha motif domain containing 9-like [Source:HGNC Symbol;Acc:HGNC:1349] [ENST00000411955]	-0.9755089	0.00906859
A_24_P343377	*GPATCH3*	Homo sapiens G patch domain containing 3 (GPATCH3), mRNA [NM_022078]	0.59145373	0.009039211
A_23_P17955	*lnc-AL353698.1-1*	Q14754_HUMAN (Q14754) ORFII, partial (4%) [THC2714876]	-14923359	0.0042815204
A_33_P3286293	*ATP5J2*	Homo sapiens ATP synthase, H+ transporting, mitochondrial Fo complex, subunit F2 (ATP5J2), transcript variant 3, mRNA [NM_001003714]	-0.5456719	0.0171135
A_23_P38346	*FBXL2*	Homo sapiens F-box and leucine-rich repeat protein 2 (FBXL2), transcript variant 1, mRNA [NM_012157]	-21730137	0.005015031
A_23_P54376	*CTNNBIP1*	Homo sapiens catenin, beta interacting protein 1 (CTNNBIP1), transcript variant 1, mRNA [NM_020248]	10269295	0.0029355595
A_24_P362646	*DHX58*	Homo sapiens DEXH (Asp-Glu-X-His) box polypeptide 58 (DHX58), mRNA [NM_024119]	-14336705	0.004634905
A_19_P00809902	*STOML1*	Homo sapiens stomatin (EPB72)-like 1 (STOML1), transcript variant 1, mRNA [NM_004809]	-0.91250324	0.011376762
A_23_P165984	*TXNDC9*	Homo sapiens thioredoxin domain containing 9 (TXNDC9), mRNA [NM_005783]	-0.75134015	0.0060495283
A_33_P3318465	*LINC01006*	Homo sapiens long intergenic non-protein coding RNA 1006 (LINC01006), long non-coding RNA [NR_103858]	11327407	0.006878185
A_19_P00801735	*ZSWIM3*	Homo sapiens zinc finger, SWIM-type containing 3 (ZSWIM3), transcript variant 1, mRNA [NM_080752]	-0.8222706	0.014070397
A_21_P0012358	*SUMO1*	Homo sapiens small ubiquitin-like modifier 1 (SUMO1), transcript variant 1, mRNA [NM_003352]	-0.54967856	0.0038773962
A_21_P0000339	*SEPT7-AS1*	SEPT7 antisense RNA 1 (head to head) [Source:HGNC Symbol;Acc:HGNC:51153] [ENST00000437235]	12417328	0.0140423635
A_24_P914513	*SNORA58*	Homo sapiens small nucleolar RNA, H/ACA box 58 (SNORA58), small nucleolar RNA [NR_002985]	-11434735	0.013513609
A_33_P3421243	*BCKDHB*	Homo sapiens branched chain keto acid dehydrogenase E1, beta polypeptide (BCKDHB), transcript variant 1, mRNA [NM_183050]	-14346714	0.0058383252
A_21_P0000218	*SIAH2*	Homo sapiens siah E3 ubiquitin protein ligase 2 (SIAH2), mRNA [NM_005067]	-10457398	0.009690877
A_33_P3288189	*AFP*	Homo sapiens alpha-fetoprotein (AFP), mRNA [NM_001134]	-11214105	0.0063397386

A_21_P0008460	*SNORD35A*	Homo sapiens small nucleolar RNA, C/D box 35A (SNORD35A), small nucleolar RNA [NR_000018]	0.8198899	0.010128013
A_23_P417918	*RHOBTB3*	Homo sapiens Rho-related BTB domain containing 3 (RHOBTB3), mRNA [NM_014899]	0.55777407	0.009545371
A_23_P217737	*PENK*	Homo sapiens proenkephalin (PENK), transcript variant 2, mRNA [NM_006211]	-32341475	0.0060077314
A_24_P71468	*TPGS1*	Homo sapiens tubulin polyglutamylase complex subunit 1 (TPGS1), mRNA [NM_033513]	1629043	0.0043634414
A_33_P3215023	*ATP7A*	Homo sapiens ATPase, Cu++ transporting, alpha polypeptide (ATP7A), transcript variant 1, mRNA [NM_000052]	-0.71879894	0.010660999
A_23_P124927	*QPCT*	Homo sapiens glutaminyl-peptide cyclotransferase (QPCT), mRNA [NM_012413]	-13192511	0.004755269
A_33_P3223535	*C6orf89*	Homo sapiens chromosome 6 open reading frame 89 (C6orf89), transcript variant 1, mRNA [NM_152734]	-0.92103213	0.0070324177
A_23_P17593	*RGS14*	Homo sapiens regulator of G-protein signaling 14 (RGS14), mRNA [NM_006480]	10660561	0.005842833
A_23_P146209	*CDH4*	Homo sapiens cadherin 4, type 1, R-cadherin (retinal) (CDH4), transcript variant 1, mRNA [NM_001794]	0.9389187	0.01179784
A_22_P00008884	*GRAMD1A*	Homo sapiens GRAM domain containing 1A (GRAMD1A), transcript variant 2, mRNA [NM_001136199]	1318321	0.007973369
A_33_P3343812	*RNF170*	Homo sapiens ring finger protein 170 (RNF170), transcript variant 2, mRNA [NM_030954]	-0.57435584	0.0031827379
A_33_P3313283	*LOC101929641*	PREDICTED: Homo sapiens uncharacterized LOC101929641 (LOC101929641), ncRNA [XR_243223]	0.7668536	0.008897602
A_23_P86100	*OR1L6*	Homo sapiens olfactory receptor, family 1, subfamily L, member 6 (OR1L6), mRNA [NM_001004453]	0.8142769	0.005039057
A_24_P219266	*CILP2*	Homo sapiens cartilage intermediate layer protein 2 (CILP2), mRNA [NM_153221]	13323556	0.013050359
A_32_P42925	*KLHDC9*	Homo sapiens kelch domain containing 9 (KLHDC9), transcript variant 2, mRNA [NM_001007255]	-1338625	0.009811661
A_23_P312536	*LRRC57*	Homo sapiens leucine rich repeat containing 57 (LRRC57), mRNA [NM_153260]	-0.838306	0.014167799
A_33_P3283906	*MYL12B*	Homo sapiens myosin, light chain 12B, regulatory (MYL12B), transcript variant 2, mRNA [NM_033546]	-0.64179224	0.0035894548
A_23_P147025	*ARSK*	Homo sapiens arylsulfatase family, member K (ARSK), mRNA [NM_198150]	-0.5014284	0.010986003

A_21_P0000682	*NIP7*	Homo sapiens NIP7, nucleolar pre-rRNA processing protein (NIP7), transcript variant 1, mRNA [NM_016101]	0.38166255	0.006736978
A_23_P201319	*RAB33A*	Homo sapiens RAB33A, member RAS oncogene family (RAB33A), mRNA [NM_004794]	-202732	0.004478291
A_32_P84728	*LHX4-AS1*	Homo sapiens LHX4 antisense RNA 1 (LHX4-AS1), long non-coding RNA [NR_037642]	-0.6841168	0.013050359
A_23_P161183	*DISP1*	Homo sapiens dispatched homolog 1 (Drosophila) (DISP1), mRNA [NM_032890]	-0.7975346	0.006414832
A_24_P216253	*ZDHHC6*	Homo sapiens zinc finger, DHHC-type containing 6 (ZDHHC6), transcript variant 1, mRNA [NM_022494]	-0.7723174	0.008208044
A_24_P210399	*DLGAP4*	Homo sapiens discs, large (Drosophila) homolog-associated protein 4 (DLGAP4), transcript variant 1, mRNA [NM_014902]	-0.95522785	0.014661159
A_33_P3286046	*BRCC3*	Homo sapiens BRCA1/BRCA2-containing complex, subunit 3 (BRCC3), transcript variant 2, mRNA [NM_001018055]	-0.53296477	0.010503048
A_33_P3397399	*TM9SF2*	Homo sapiens transmembrane 9 superfamily member 2 (TM9SF2), mRNA [NM_004800]	-0.72953653	0.005255513
A_23_P88865	*DPCD*	Homo sapiens deleted in primary ciliary dyskinesia homolog (mouse) (DPCD), mRNA [NM_015448]	-0.96543765	0.009795713
A_33_P3265704	*PPP3CB*	Homo sapiens protein phosphatase 3, catalytic subunit, beta isozyme (PPP3CB), transcript variant 5, mRNA [NM_001289969]	-0.4655061	0.0052206037
A_23_P151614	*CMTM3*	Homo sapiens CKLF-like MARVEL transmembrane domain containing 3 (CMTM3), transcript variant 1, mRNA [NM_144601]	-11730022	0.011432728
A_33_P3366073	*UFD1L*	Homo sapiens ubiquitin fusion degradation 1 like (yeast) (UFD1L), transcript variant 1, mRNA [NM_005659]	-0.75849843	0.009656577
A_24_P400355	*PSME1*	Homo sapiens proteasome (prosome, macropain) activator subunit 1 (PA28 alpha) (PSME1), transcript variant 1, mRNA [NM_006263]	-0.96341276	0.012887069
A_32_P192376	*EBPL*	Homo sapiens emopamil binding protein-like (EBPL), transcript variant 3, non-coding RNA [NR_103802]	-0.62634027	0.011964846
A_33_P3400547	*GET4*	Homo sapiens golgi to ER traffic protein 4 homolog (S. cerevisiae) (GET4), mRNA [NM_015949]	-0.7017038	0.0115904575
A_23_P11843	*ENPP1*	Homo sapiens ectonucleotide pyrophosphatase/phosphodiesterase 1 (ENPP1), mRNA [NM_006208]	-0.6293497	0.013596905

A_33_P3261700	*MED21*	Homo sapiens mediator complex subunit 21 (MED21), transcript variant 1, mRNA [NM_004264]	-0.96450067	0.0054365364
A_33_P3292417	*LRRN2*	Homo sapiens leucine rich repeat neuronal 2 (LRRN2), transcript variant 2, mRNA [NM_201630]	0.6048147	0.005851159
A_33_P3221055	*ANKRD13C*	Homo sapiens ankyrin repeat domain 13C (ANKRD13C), mRNA [NM_030816]	-0.38766095	0.011575981
A_32_P137266	*WDR5*	Homo sapiens WD repeat domain 5 (WDR5), transcript variant 1, mRNA [NM_017588]	-0.4925863	0.016295526
A_33_P3254946	*LOC101927270*	PREDICTED: Homo sapiens uncharacterized LOC101927270 (LOC101927270), transcript variant X4, misc_RNA [XR_432672]	-0.64650685	0.016238803
A_21_P0014589	*EFCAB7*	EF-hand calcium binding domain 7 [Source:HGNC Symbol;Acc:HGNC:29379] [ENST00000461039]	-0.8315036	0.0072222846
A_23_P143958	*PLEKHH3*	Homo sapiens pleckstrin homology domain containing, family H (with MyTH4 domain) member 3 (PLEKHH3), transcript variant 1, mRNA [NM_024927]	-0.6608805	0.015453676
A_23_P108303	*LOC101929112*	PREDICTED: Homo sapiens uncharacterized LOC101929112 (LOC101929112), transcript variant X1, ncRNA [XR_246129]	-0.66747963	0.005559843
A_33_P3387050	*RPL22L1*	Homo sapiens ribosomal protein L22-like 1 (RPL22L1), mRNA [NM_001099645]	-0.54630655	0.0035432973
A_23_P212475	*NDUFA7*	Homo sapiens NADH dehydrogenase (ubiquinone) 1 alpha subcomplex, 7, 14.5kDa (NDUFA7), mRNA [NM_005001]	-0.861146	0.007929221
A_23_P66948	*C8orf82*	Homo sapiens chromosome 8 open reading frame 82 (C8orf82), mRNA [NM_001001795]	-0.70331544	0.009431794
A_23_P307310	*SHISA5*	Homo sapiens shisa family member 5 (SHISA5), transcript variant 1, mRNA [NM_016479]	-0.4181819	0.010053306
A_33_P3233550	*GAREM*	Homo sapiens GRB2 associated, regulator of MAPK1 (GAREM), transcript variant 2, mRNA [NM_022751]	-0.5846429	0.016826278
A_33_P3301955	*ACAN*	Homo sapiens aggrecan (ACAN), transcript variant 2, mRNA [NM_013227]	-8002324	0.0029691157
A_23_P377214	*RTN4*	Homo sapiens reticulon 4 (RTN4), transcript variant 1, mRNA [NM_020532]	-0.37200496	0.0075457073
A_23_P54055	*PDE7A*	Homo sapiens phosphodiesterase 7A (PDE7A), transcript variant 3, mRNA [NM_001242318]	-12016586	0.011149755
A_24_P74070	*HEXIM2*	hexamethylene bis-acetamide inducible 2 [Source:HGNC Symbol;Acc:HGNC:28591] [ENST00000592695]	-0.93687034	0.006077557
A_24_P84008	*AJUBA*	Homo sapiens ajuba LIM protein (AJUBA), transcript variant 1, mRNA [NM_032876]	-0.7951641	0.009811661

A_22_P00023453	*PARD6G*	Homo sapiens par-6 family cell polarity regulator gamma (PARD6G), mRNA [NM_032510]	0.599312	0.013124348
A_22_P00023397	*lnc-LRMP-3*	DB455794 RIKEN full-length enriched human cDNA library, testis Homo sapiens cDNA clone H013070L12 5', mRNA sequence [DB455794]	0.67105013	0.014792068
A_22_P00014326	*CASP7*	Homo sapiens caspase 7, apoptosis-related cysteine peptidase (CASP7), transcript variant d, mRNA [NM_033338]	-0.5989441	0.01083596
A_32_P10272	*lnc-TMEM159-2*	LNCipedia lincRNA (lnc-TMEM159-2), lincRNA [lnc-TMEM159-2:1]	12211572	0.011091872
A_23_P390190	*lnc-SERPINC1-1*	LNCipedia lincRNA (lnc-SERPINC1-1), lincRNA [lnc-SERPINC1-1:24]	-0.54604346	0.004849151
A_23_P204630	*PMS2P5*	Homo sapiens postmeiotic segregation increased 2 pseudogene 5 (PMS2P5), transcript variant 1, non-coding RNA [NR_027775]	-0.4494712	0.013475039
A_21_P0014085	*NUPL1*	Homo sapiens nucleoporin like 1 (NUPL1), transcript variant 1, mRNA [NM_014089]	-0.56076336	0.0037341104
A_21_P0012676	*NTN4*	Homo sapiens netrin 4 (NTN4), mRNA [NM_021229]	-23868053	0.0028703364
A_21_P0000515	*ID2-AS1*	Homo sapiens ID2 antisense RNA 1 (head to head) (ID2-AS1), transcript variant 2, long non-coding RNA [NR_110154]	-0.79348403	0.016027637
A_23_P22119	*GK3P*	Homo sapiens glycerol kinase 3 pseudogene (GK3P), non-coding RNA [NR_026575]	12239108	0.013327314
A_21_P0006549	*VTRNA1-3*	Homo sapiens vault RNA 1-3 (VTRNA1-3), vault RNA [NR_026705]	-13911158	0.0032710456
A_22_P00023347	*PLEC*	Homo sapiens plectin (PLEC), transcript variant 6, mRNA [NM_201380]	0.795383	0.0097784065
A_23_P5601	*lnc-APLN-1*	LNCipedia lincRNA (lnc-APLN-1), lincRNA [lnc-APLN-1:1]	-22798262	0.0038144474
A_23_P42080	*lnc-C12orf49-2*	ALU1_HUMAN (P39188) Alu subfamily J sequence contamination warning entry, partial (10%) [THC2724097]	-0.70064753	0.009560995
A_33_P3363305	*DOK1*	Homo sapiens docking protein 1, 62kDa (downstream of tyrosine kinase 1) (DOK1), transcript variant 1, mRNA [NM_001381]	-10866952	0.010148608
A_33_P3367293	*TMEM14A*	Homo sapiens transmembrane protein 14A (TMEM14A), mRNA [NM_014051]	-12485567	0.0024890031
A_23_P419213	*MT1IP*	Homo sapiens metallothionein 1I, pseudogene (MT1IP), non-coding RNA [NR_003669]	0.35126373	0.015561968
A_21_P0003055	*CCDC85C*	Homo sapiens coiled-coil domain containing 85C (CCDC85C), mRNA [NM_001144995]	-0.30621582	0.0068582953
A_32_P99032	*KIAA1407*	Homo sapiens KIAA1407 (KIAA1407), mRNA [NM_020817]	-0.95517266	0.009555101
A_33_P3218741	*lnc-SERPINI1-9*	LNCipedia lincRNA (lnc-SERPINI1-9), lincRNA [lnc-SERPINI1-9:5]	-0.7526715	0.004701671

A_23_P86195	*LOC100240734*	Homo sapiens uncharacterized LOC100240734 (LOC100240734), long non-coding RNA [NR_026657]	-2496026	0.0033065486
A_33_P3412294	*SLC44A3*	Homo sapiens solute carrier family 44, member 3 (SLC44A3), transcript variant 2, mRNA [NM_152369]	-20346456	0.0122128595
A_33_P3326382	*PGP*	Homo sapiens phosphoglycolate phosphatase (PGP), mRNA [NM_001042371]	12356343	0.0063422406
A_23_P154675	*ZSCAN2*	Homo sapiens zinc finger and SCAN domain containing 2 (ZSCAN2), transcript variant 1, mRNA [NM_181877]	36459465	0.0044370755
A_24_P380132	*STX1B*	syntaxin 1B [Source:HGNC Symbol;Acc:HGNC:18539] [ENST00000215095]	10652729	0.015647968
A_23_P44942	*SNRPB*	Homo sapiens small nuclear ribonucleoprotein polypeptides B and B1 (SNRPB), transcript variant 1, mRNA [NM_198216]	0.8342913	0.0050375774
A_33_P3551349	*G3BP2*	Homo sapiens GTPase activating protein (SH3 domain) binding protein 2 (G3BP2), transcript variant 1, mRNA [NM_203505]	-0.39339373	0.012680636
A_23_P109410	*CCDC15*	Homo sapiens coiled-coil domain containing 15 (CCDC15), mRNA [NM_025004]	-0.51064974	0.010380393
A_23_P393034	*THAP7*	Homo sapiens THAP domain containing 7 (THAP7), transcript variant 1, mRNA [NM_030573]	0.94390786	0.0055675246
A_22_P00001957	*PRL*	Homo sapiens prolactin (PRL), transcript variant 1, mRNA [NM_000948]	-13948004	0.010138943
A_33_P3257808	*HAS3*	Homo sapiens hyaluronan synthase 3 (HAS3), transcript variant 1, mRNA [NM_005329]	-20056534	0.013751258
A_22_P00022494	*SRP54-AS1*	PREDICTED: Homo sapiens uncharacterized LOC100506157 (LOC100506157), transcript variant X4, ncRNA [XR_424491]	-0.8214324	0.012893396
A_32_P180971	*FANCD2*	Homo sapiens Fanconi anemia, complementation group D2 (FANCD2), transcript variant 2, mRNA [NM_001018115]	-0.9975977	0.015416404
A_21_P0011343	*LOC728323*		-11402445	0.00687431
A_33_P3267356	*OTUD6B-AS1*	Homo sapiens OTUD6B antisense RNA 1 (head to head) (OTUD6B-AS1), transcript variant 1, long non-coding RNA [NR_110438]	-11985157	0.0039856653
A_22_P00025917	*LOC100128108*	PREDICTED: Homo sapiens uncharacterized LOC100128108 (LOC100128108), transcript variant X1, mRNA [XM_006709989]	-0.55077827	0.010231521
A_33_P3259203	*GLS*	Homo sapiens glutaminase (GLS), transcript variant 2, mRNA [NM_001256310]	-14797407	0.0046609533
A_21_P0001971	*LOC100506603*	Homo sapiens uncharacterized LOC100506603 (LOC100506603), transcript variant 1, long non-coding RNA [NR_104183]	-12544068	0.005173042

A_33_P3619171	*TMEM255B*	transmembrane protein 255B [Source:HGNC Symbol;Acc:HGNC:28297] [ENST00000375353]	-14527357	0.0051655783
A_33_P3373185	*PMAIP1*	Homo sapiens phorbol-12-myristate-13-acetate-induced protein 1 (PMAIP1), mRNA [NM_021127]	12316222	0.00371792
A_33_P3270019	*CDS2*	Homo sapiens CDP-diacylglycerol synthase (phosphatidate cytidylyltransferase) 2 (CDS2), mRNA [NM_003818]	-0.5129463	0.015141116
A_23_P208288	*LINC00999*	Homo sapiens long intergenic non-protein coding RNA 999 (LINC00999), long non-coding RNA [NR_024497]	0.581313	0.0114650065
A_23_P79155	*NCOA2*	Homo sapiens nuclear receptor coactivator 2 (NCOA2), mRNA [NM_006540]	12483886	0.0073775975
A_23_P18276	*ZNF304*	Homo sapiens zinc finger protein 304 (ZNF304), transcript variant 2, mRNA [NM_020657]	-0.6523904	0.0037583082
A_33_P3348494	*GPR39*	Homo sapiens G protein-coupled receptor 39 (GPR39), mRNA [NM_001508]	25654817	0.005377644
A_22_P00003258	*RBM5*	Homo sapiens RNA binding motif protein 5 (RBM5), transcript variant 1, mRNA [NM_005778]	-0.8521862	0.010314042
A_23_P204277	*PCID2*	Homo sapiens PCI domain containing 2 (PCID2), transcript variant 5, mRNA [NM_001258213]	-0.53655005	0.007698106
A_33_P3247858	*HOXB-AS1*	Homo sapiens HOXB cluster antisense RNA 1 (HOXB-AS1), long non-coding RNA [NR_102279]	-0.30838722	0.0059353677
A_33_P3355266	*H2AFJ*	Homo sapiens H2A histone family, member J (H2AFJ), transcript variant 1, mRNA [NM_177925]	-10299659	0.0055105817
A_33_P3236340	*MPP1*	Homo sapiens membrane protein, palmitoylated 1, 55kDa (MPP1), transcript variant 3, mRNA [NM_001166461]	-0.9304849	0.011021474
A_24_P54390	*TINAGL1*	Homo sapiens tubulointerstitial nephritis antigen-like 1 (TINAGL1), transcript variant 1, mRNA [NM_022164]	-0.41203326	0.010911651
A_33_P3243649	*RASGRP3*	Homo sapiens RAS guanyl releasing protein 3 (calcium and DAG-regulated) (RASGRP3), transcript variant 2, mRNA [NM_170672]	16943161	0.007103236
A_24_P217572	*DRD4*	Homo sapiens dopamine receptor D4 (DRD4), mRNA [NM_000797]	-0.64547086	0.010182504
A_23_P38365	*LOC100131831*	Homo sapiens cDNA FLJ26174 fis, clone ADG03920. [AK129685]	11553562	0.009512793
A_33_P3284345	*EDNRA*	Homo sapiens endothelin receptor type A (EDNRA), transcript variant 1, mRNA [NM_001957]	2003203	0.0037353234
A_23_P212310	*TLK2*	Homo sapiens tousled-like kinase 2 (TLK2), transcript variant A, mRNA [NM_006852]	-0.8300325	0.012406482

A_23_P53567	*NRG1*	Homo sapiens neuregulin 1 (NRG1), transcript variant HRG-gamma, mRNA [NM_004495]	-33858156	0.0032554762
A_33_P3395738	*ELP6*	Homo sapiens elongator acetyltransferase complex subunit 6 (ELP6), mRNA [NM_001031703]	-0.52536106	0.016474942
A_33_P3329597	*CCDC53*	Homo sapiens coiled-coil domain containing 53 (CCDC53), transcript variant 1, mRNA [NM_016053]	-0.6777575	0.011530268
A_33_P3266828	*BCAR1*	Homo sapiens cDNA FLJ45059 fis, clone BRAWH3023274. [AK127004]	0.8382127	0.015811017
A_23_P17503	*BLOC1S3*	Homo sapiens biogenesis of lysosomal organelles complex-1, subunit 3 (BLOC1S3), mRNA [NM_212550]	-0.8960364	0.008685628
A_21_P0012925	*TMEM8B*	Homo sapiens transmembrane protein 8B (TMEM8B), transcript variant 3, mRNA [NM_016446]	-14068577	0.0050497777
A_32_P151544	*KIF16B*	Homo sapiens kinesin family member 16B (KIF16B), transcript variant 2, mRNA [NM_024704]	13872744	0.007415985
A_23_P85053	*XLOC_l2_012210*	BROAD Institute lincRNA (XLOC_l2_012210), lincRNA [TCONS_l2_00023087]	-0.4391883	0.0100634415
A_33_P3302070	*KRT18*	Homo sapiens keratin 18, type I (KRT18), transcript variant 1, mRNA [NM_000224]	11772339	0.0075625386
A_21_P0011479	*ZRSR2*	Homo sapiens zinc finger (CCCH type), RNA-binding motif and serine/arginine rich 2 (ZRSR2), mRNA [NM_005089]	-0.60494894	0.011570765
A_32_P231086	*XLOC_l2_005465*	BROAD Institute lincRNA (XLOC_l2_005465), lincRNA [TCONS_l2_00010135]	-0.35507843	0.012500278
A_24_P191312	*TSEN15*	Homo sapiens TSEN15 tRNA splicing endonuclease subunit (TSEN15), transcript variant 1, mRNA [NM_052965]	-12744648	0.0039497027
A_23_P502930	*GOLGA6L9*	Homo sapiens golgin A6 family-like 9 (GOLGA6L9), transcript variant 1, mRNA [NM_198181]	-0.73909307	0.01120723
A_33_P3219429	*SLC1A4*	Homo sapiens solute carrier family 1 (glutamate/neutral amino acid transporter), member 4 (SLC1A4), transcript variant 1, mRNA [NM_003038]	-20616922	0.006111203
A_23_P169278	*FASTK*	Homo sapiens Fas-activated serine/threonine kinase (FASTK), transcript variant 1, mRNA [NM_006712]	-0.8110513	0.009918909
A_21_P0001790	*SMNDC1*	Homo sapiens survival motor neuron domain containing 1 (SMNDC1), mRNA [NM_005871]	-0.5523228	0.004912971
A_24_P331704	*AGTPBP1*	Homo sapiens ATP/GTP binding protein 1 (AGTPBP1), transcript variant 2, mRNA [NM_015239]	-13754284	0.004359316

A_23_P44295	*LOC101927438*	Homo sapiens uncharacterized LOC101927438 (LOC101927438), long non-coding RNA [NR_110224]	0.2192158	0.00942879
A_23_P133075	*KRT80*	Homo sapiens keratin 80, type II (KRT80), transcript variant 1, mRNA [NM_182507]	1014504	0.007065473
A_23_P332399	*CLASP2*	Homo sapiens cytoplasmic linker associated protein 2 (CLASP2), transcript variant 1, mRNA [NM_015097]	-0.6246693	0.014525657
A_33_P3718152	*TBCK*	Homo sapiens TBC1 domain containing kinase (TBCK), transcript variant 4, mRNA [NM_033115]	-14093245	0.006360746
A_23_P160318	*GULP1*	Homo sapiens GULP, engulfment adaptor PTB domain containing 1 (GULP1), transcript variant 1, mRNA [NM_016315]	0.9276275	0.0043163924
A_23_P40307	*lnc-ACOT1-3*	Homo sapiens cDNA FLJ31314 fis, clone LIVER1000278. [AK055876]	1131196	0.010284574
A_33_P3365117	*COL16A1*	Homo sapiens collagen, type XVI, alpha 1 (COL16A1), mRNA [NM_001856]	-0.6225452	0.0057666874
A_33_P3280521	*SNRPB2*	Homo sapiens small nuclear ribonucleoprotein polypeptide B (SNRPB2), transcript variant 1, mRNA [NM_003092]	-0.8121536	0.0076440917
A_22_P00013357	*AKR1C1*	aldo-keto reductase family 1, member C1 [Source:HGNC Symbol;Acc:HGNC:384] [ENST00000380859]	22161121	0.010607272
A_24_P879740	*MFAP3L*	Homo sapiens microfibrillar-associated protein 3-like (MFAP3L), transcript variant 1, mRNA [NM_021647]	-2820716	0.006161988
A_23_P121875	*lnc-RP11-166B2.1.1-1*	LNCipedia lincRNA (lnc-RP11-166B2.1.1-1), lincRNA [lnc-RP11-166B2.1.1-1:1]	12962018	0.01541599
A_23_P146830	*MAP1B*	Homo sapiens microtubule-associated protein 1B (MAP1B), mRNA [NM_005909]	-0.39153218	0.010519677
A_33_P3221414	*C5orf28*	Homo sapiens chromosome 5 open reading frame 28 (C5orf28), mRNA [NM_022483]	-10106556	0.0053354
A_22_P00023618	*SLC25A10*	Homo sapiens solute carrier family 25 (mitochondrial carrier; dicarboxylate transporter), member 10 (SLC25A10), transcript variant 2, mRNA [NM_012140]	13386667	0.007415985
A_23_P316460	*NTNG1*	netrin G1 [Source:HGNC Symbol;Acc:HGNC:23319] [ENST00000370068]	-20300145	0.009988937
A_33_P3412722	*ATP13A5-AS1*	Homo sapiens ATP13A5 antisense RNA 1 (ATP13A5-AS1), long non-coding RNA [NR_046758]	0.2179086	0.017063787
A_23_P302787	*ZBED6CL*	Homo sapiens ZBED6 C-terminal like (ZBED6CL), mRNA [NM_138434]	0.8861682	0.004282122
A_32_P113114	*SNX8*	Homo sapiens sorting nexin 8 (SNX8), mRNA [NM_013321]	12313712	0.0062071052

A_23_P30050	*LINC01116*	Homo sapiens long intergenic non-protein coding RNA 1116 (LINC01116), long non-coding RNA [NR_040001]	-0.5895057	0.008437608
A_23_P98310	*ZNF561*	Homo sapiens zinc finger protein 561 (ZNF561), mRNA [NM_152289]	-0.6070724	0.0107158525
A_23_P152666	*SLC30A9*	Homo sapiens solute carrier family 30 (zinc transporter), member 9 (SLC30A9), mRNA [NM_006345]	-0.4227788	0.011010366
A_21_P0013670	*CSTF3*	Homo sapiens cleavage stimulation factor, 3' pre-RNA, subunit 3, 77kDa (CSTF3), transcript variant 1, mRNA [NM_001326]	-0.7593565	0.006773419
A_24_P313186	*COX11*	Homo sapiens COX11 cytochrome c oxidase copper chaperone (COX11), transcript variant 1, mRNA [NM_004375]	-0.8028675	0.005787274
A_23_P44974	*XLOC_l2_015295*	BROAD Institute lincRNA (XLOC_l2_015295), lincRNA [TCONS_l2_00029480]	13547206	0.010402669
A_23_P150510	*CALM1*	Homo sapiens calmodulin 1 (phosphorylase kinase, delta) (CALM1), mRNA [NM_006888]	-0.49660805	0.011313176
A_32_P128701	*MRPL13*	Homo sapiens mitochondrial ribosomal protein L13 (MRPL13), mRNA [NM_014078]	-0.79694295	0.006814677
A_23_P75921	*MED19*	Homo sapiens mediator complex subunit 19 (MED19), mRNA [NM_153450]	-0.97200584	0.0064337924
A_33_P3382423	*USP53*	Homo sapiens ubiquitin specific peptidase 53 (USP53), mRNA [NM_019050]	-1464283	0.0041847117
A_33_P3332006	*TRAF6*	Homo sapiens TNF receptor-associated factor 6, E3 ubiquitin protein ligase (TRAF6), transcript variant 1, mRNA [NM_145803]	-0.80867845	0.010168999
A_22_P00016898	*ZNF428*	Homo sapiens zinc finger protein 428 (ZNF428), mRNA [NM_182498]	-10564286	0.0063838474
A_23_P43946	*CBX1*	Homo sapiens chromobox homolog 1 (CBX1), transcript variant 1, mRNA [NM_006807]	-0.6085691	0.005325502
A_23_P207742	*SARNP*	Homo sapiens SAP domain containing ribonucleoprotein (SARNP), transcript variant 1, mRNA [NM_033082]	-0.8236282	0.005236083
A_33_P3369761	*NDUFAF7*	Homo sapiens NADH dehydrogenase (ubiquinone) complex I, assembly factor 7 (NDUFAF7), transcript variant 1, mRNA [NM_144736]	-0.92076224	0.007923661
A_23_P158662	*THRA*	Homo sapiens thyroid hormone receptor, alpha (THRA), transcript variant 2, mRNA [NM_003250]	-13431157	0.0039856653
A_33_P3603812	*PDP1*	Homo sapiens pyruvate dehyrogenase phosphatase catalytic subunit 1 (PDP1), transcript variant 2, mRNA [NM_001161779]	-0.8904722	0.0024890031
A_33_P3223869	*GPS1*	Homo sapiens G protein pathway suppressor 1 (GPS1), transcript variant 1, mRNA [NM_212492]	0.9730389	0.004884975

A_33_P3399208	*FRMD8*	Homo sapiens FERM domain containing 8 (FRMD8), transcript variant 3, mRNA [NM_001300833]	0.71217597	0.0086922655
A_33_P3381483	*HLA-B*	major histocompatibility complex, class I, B [Source:HGNC Symbol;Acc:HGNC:4932] [ENST00000466304]	-0.86204726	0.009603145
A_22_P00008429	*PDE4DIP*	Homo sapiens phosphodiesterase 4D interacting protein (PDE4DIP), transcript variant 1, mRNA [NM_014644]	-0.85847783	0.0063477047
A_22_P00007112	*ZNF331*	Homo sapiens zinc finger protein 331 (ZNF331), transcript variant 3, mRNA [NM_001253798]	0.4809716	0.015810816
A_33_P3262205	*SGOL1-AS1*	Homo sapiens SGOL1 antisense RNA 1 (SGOL1-AS1), long non-coding RNA [NR_046723]	10345377	0.0075276187
A_33_P3296067	*lnc-GLUD1-3*	CU454073 Homo sapiens ORESTES from keratinocytes Homo sapiens cDNA, mRNA sequence [CU454073]	17787052	0.0037856582
A_24_P350649	*CPNE1*	Homo sapiens copine I (CPNE1), transcript variant 3, mRNA [NM_003915]	11111164	0.007990462
A_33_P3235117	*CDC14C*	Homo sapiens cell division cycle 14C (CDC14C), non-coding RNA [NR_003595]	0.81432176	0.007889792
A_23_P385105	*PDPR*	Homo sapiens pyruvate dehydrogenase phosphatase regulatory subunit (PDPR), mRNA [NM_017990]	-0.94790405	0.015511605
A_23_P410965	*LOC100133286*	Homo sapiens uncharacterized LOC100133286 (LOC100133286), long non-coding RNA [NR_040084]	0.95662546	0.0037685214
A_23_P41066	*PLCD4*	Homo sapiens phospholipase C, delta 4 (PLCD4), mRNA [NM_032726]	11633303	0.008488166
A_23_P147109	*KIAA1522*	Homo sapiens KIAA1522 (KIAA1522), transcript variant 1, mRNA [NM_020888]	-0.8375754	0.009952812
A_24_P268786	*RASSF1*	Homo sapiens Ras association (RalGDS/AF-6) domain family member 1 (RASSF1), transcript variant C, mRNA [NM_170713]	-0.8391683	0.0065390505
A_23_P46606	*TOR4A*	Homo sapiens torsin family 4, member A (TOR4A), mRNA [NM_017723]	23912847	0.003162355
A_23_P96209	*MYNN*	Homo sapiens myoneurin (MYNN), transcript variant 1, mRNA [NM_018657]	-0.89402837	0.007700591
A_24_P942730	*LPGAT1*	Homo sapiens lysophosphatidylglycerol acyltransferase 1 (LPGAT1), mRNA [NM_014873]	-0.99062085	0.00862906
A_23_P214330	*REEP4*	Homo sapiens receptor accessory protein 4 (REEP4), mRNA [NM_025232]	19267178	0.0044240346
A_23_P363163	*ZSCAN29*	Homo sapiens zinc finger and SCAN domain containing 29 (ZSCAN29), mRNA [NM_152455]	-1056368	0.010565531
A_33_P3244803	*SERPINB1*	Homo sapiens serpin peptidase inhibitor, clade B (ovalbumin), member 1 (SERPINB1), transcript variant 1, mRNA [NM_030666]	-10292995	0.0072506997

A_33_P3243230	*CGGBP1*	Homo sapiens CGG triplet repeat binding protein 1 (CGGBP1), transcript variant 1, mRNA [NM_001008390]	-10037732	0.009525487
A_23_P56833	*ACOX1*	Homo sapiens acyl-CoA oxidase 1, palmitoyl (ACOX1), transcript variant 3, mRNA [NM_001185039]	-0.7155204	0.010426067
A_24_P926783	*ASB3*	Homo sapiens ankyrin repeat and SOCS box containing 3 (ASB3), transcript variant 1, mRNA [NM_016115]	-0.9424634	0.013213625
A_33_P3330443	*TRIB2*	Homo sapiens tribbles pseudokinase 2 (TRIB2), transcript variant 1, mRNA [NM_021643]	-0.73295045	0.013657404
A_33_P3250845	*ZNF880*	Homo sapiens zinc finger protein 880 (ZNF880), mRNA [NM_001145434]	-0.44171286	0.011102258
A_33_P3267118	*FAM110B*	Homo sapiens family with sequence similarity 110, member B (FAM110B), mRNA [NM_147189]	-13177708	0.005735339
A_23_P380965	*NIPA2*	Homo sapiens non imprinted in Prader-Willi/Angelman syndrome 2 (NIPA2), transcript variant 5, mRNA [NM_001184889]	-0.39005923	0.011759551
A_23_P130304	*EIF4G3*	eukaryotic translation initiation factor 4 gamma, 3 [Source:HGNC Symbol;Acc:HGNC:3298] [ENST00000374933]	-0.6648829	0.013460425
A_33_P3398437	*JKAMP*	Homo sapiens JNK1/MAPK8-associated membrane protein (JKAMP), transcript variant 1, mRNA [NM_016475]	-0.5574603	0.010402669
A_23_P113701	*TXNL4A*	thioredoxin-like 4A [Source:HGNC Symbol;Acc:HGNC:30551] [ENST00000585769]	-0.47915053	0.01396833
A_23_P137578	*MGA*	MGA, MAX dimerization protein [Source:HGNC Symbol;Acc:HGNC:14010] [ENST00000619521]	28742082	0.0044519836
A_21_P0010960	*PDGFA*	Homo sapiens platelet-derived growth factor alpha polypeptide (PDGFA), transcript variant 1, mRNA [NM_002607]	-13813336	0.009431794
A_23_P210829	*FBXO28*	Homo sapiens F-box protein 28 (FBXO28), transcript variant 1, mRNA [NM_015176]	-0.71010685	0.008570872
A_21_P0010328	*XLOC_l2_002204*	BROAD Institute lincRNA (XLOC_l2_002204), lincRNA [TCONS_l2_00004345]	0.8094908	0.007111782
A_23_P128174	*PCMTD2*	Homo sapiens protein-L-isoaspartate (D-aspartate) O-methyltransferase domain containing 2 (PCMTD2), transcript variant 1, mRNA [NM_018257]	-11794249	0.0058555133
A_33_P3354322	*LOC101928891*	Homo sapiens uncharacterized LOC101928891 (LOC101928891), long non-coding RNA [NR_110537]	-12365162	0.011366253
A_23_P200001	*RAB3IP*	Homo sapiens RAB3A interacting protein (RAB3IP), transcript variant alpha 2, mRNA [NM_175623]	-13331634	0.0114562

A_23_P70231	*GPX1*	Homo sapiens glutathione peroxidase 1 (GPX1), transcript variant 2, mRNA [NM_201397]	-0.8519237	0.0025001846
A_23_P424513	*NEXN*	Homo sapiens nexilin (F actin binding protein) (NEXN), transcript variant 1, mRNA [NM_144573]	-0.6363167	0.011870703
A_33_P3324383	*ALDH7A1*	Homo sapiens aldehyde dehydrogenase 7 family, member A1 (ALDH7A1), transcript variant 1, mRNA [NM_001182]	-17232429	0.003365343
A_22_P00025954	*RANBP9*	Homo sapiens RAN binding protein 9 (RANBP9), mRNA [NM_005493]	-0.7609031	0.0046812603
A_24_P936122	*FIGNL2*	Homo sapiens fidgetin-like 2 (FIGNL2), mRNA [NM_001013690]	-19375142	0.015820691
A_21_P0013717	*LOC101929771*	Homo sapiens uncharacterized LOC101929771 (LOC101929771), long non-coding RNA [NR_125989]	-11625786	0.009121571
A_21_P0000015	*STX16*	Homo sapiens syntaxin 16 (STX16), transcript variant 1, mRNA [NM_001001433]	-0.5872311	0.016538722
A_33_P3377459	*PARP9*	Homo sapiens poly (ADP-ribose) polymerase family, member 9 (PARP9), transcript variant 6, mRNA [NM_001146106]	-12272214	0.016400624
A_23_P258251	*IL6ST*	Homo sapiens interleukin 6 signal transducer (IL6ST), transcript variant 3, mRNA [NM_001190981]	0.7727694	0.010567525
A_21_P0007936	*PAPD5*	Homo sapiens PAP associated domain containing 5 (PAPD5), transcript variant 1, mRNA [NM_001040284]	-12396761	0.013451378
A_21_P0010578	*ENOX2*	Homo sapiens ecto-NOX disulfide-thiol exchanger 2 (ENOX2), transcript variant 2, mRNA [NM_182314]	-0.6845302	0.010986003
A_23_P105368	*lnc-GTF2F2-2*	LNCipedia lincRNA (lnc-GTF2F2-2), lincRNA [lnc-GTF2F2-2:14]	-0.63667417	0.0054365364
A_24_P319613	*LOC101926980*	PREDICTED: Homo sapiens septin-7-like (LOC101926980), transcript variant X6, misc_RNA [XR_431358]	-10239025	0.010827594
A_24_P237757	*NEK2*	Homo sapiens NIMA-related kinase 2 (NEK2), transcript variant 1, mRNA [NM_002497]	-0.5783553	0.007610532
A_33_P3253807	*LOC389906*	Homo sapiens zinc finger protein 839 pseudogene (LOC389906), non-coding RNA [NR_034031]	0.536135	0.013169497
A_23_P211039	*ZMYM2*	zinc finger, MYM-type 2 [Source:HGNC Symbol;Acc:HGNC:12989] [ENST00000382881]	-0.5048208	0.008447855
A_23_P26386	*CEBPG*	Homo sapiens CCAAT/enhancer binding protein (C/EBP), gamma (CEBPG), transcript variant 1, mRNA [NM_001806]	-12583625	0.005512448
A_23_P93988	*ADAMTS1*	Homo sapiens ADAM metallopeptidase with thrombospondin type 1 motif, 1 (ADAMTS1), mRNA [NM_006988]	-0.7594461	0.008204847

A_33_P3560679	*TPPP3*	Homo sapiens tubulin polymerization-promoting protein family member 3 (TPPP3), transcript variant 1, mRNA [NM_016140]	0.95722854	0.0070460853
A_23_P146908	*ARHGEF5*	Homo sapiens Rho guanine nucleotide exchange factor (GEF) 5 (ARHGEF5), mRNA [NM_005435]	-0.9264052	0.0075709717
A_24_P356130	*STX8*	Homo sapiens syntaxin 8 (STX8), transcript variant 1, mRNA [NM_004853]	-0.4936874	0.0096947495
A_33_P3269723	*lnc-CHD9-1*	ALU4_HUMAN (P39191) Alu subfamily SB2 sequence contamination warning entry, partial (4%) [THC2709209]	-0.96548784	0.009630487
A_22_P00004635	*MAP2K5*	Homo sapiens mitogen-activated protein kinase kinase 5 (MAP2K5), transcript variant 2, mRNA [NM_002757]	-0.36621907	0.014244717
A_24_P68819	*ZSWIM7*	Homo sapiens zinc finger, SWIM-type containing 7 (ZSWIM7), transcript variant 1, mRNA [NM_001042697]	-0.8585255	0.010124778
A_33_P3382709	*LOC101927571*	Homo sapiens uncharacterized LOC101927571 (LOC101927571), long non-coding RNA [NR_110782]	-0.36494362	0.014013276
A_23_P19576	*PPP1R21*	Homo sapiens protein phosphatase 1, regulatory subunit 21 (PPP1R21), transcript variant 2, mRNA [NM_152994]	-0.8988179	0.012007281
A_33_P3311498	*LEMD3*	Homo sapiens LEM domain containing 3 (LEMD3), transcript variant 1, mRNA [NM_014319]	-0.78319377	0.010328656
A_23_P205031	*SF3B5*	Homo sapiens splicing factor 3b, subunit 5, 10kDa (SF3B5), mRNA [NM_031287]	-0.55626464	0.01479099
A_23_P94053	*TRHDE-AS1*	Homo sapiens TRHDE antisense RNA 1 (TRHDE-AS1), transcript variant 1, long non-coding RNA [NR_026837]	0.522905	0.016416559
A_22_P00021984	*COL4A2*	Homo sapiens collagen, type IV, alpha 2 (COL4A2), mRNA [NM_001846]	-23067617	0.0041916566
A_21_P0013462	*TRRAP*	Homo sapiens transformation/transcription domain-associated protein (TRRAP), transcript variant 2, mRNA [NM_003496]	-0.20603853	0.012320746
A_23_P143016	*ABCB7*	Homo sapiens ATP-binding cassette, sub-family B (MDR/TAP), member 7 (ABCB7), transcript variant 1, mRNA [NM_004299]	-0.565413	0.0058047334
A_21_P0003986	*CENPA*	Homo sapiens centromere protein A (CENPA), transcript variant 1, mRNA [NM_001809]	-0.5559526	0.010308166
A_33_P3270509	*ARID5A*	Homo sapiens AT rich interactive domain 5A (MRF1-like) (ARID5A), mRNA [NM_212481]	-0.7864413	0.013289528
A_33_P3240333	*GDNF-AS1*	Homo sapiens GDNF antisense RNA 1 (head to head) (GDNF-AS1), transcript variant 1, mRNA [NM_001278094]	-16061314	0.0032589228

A_23_P334864	*SIAH1*	Homo sapiens siah E3 ubiquitin protein ligase 1 (SIAH1), transcript variant 1, mRNA [NM_003031]	-0.44249934	0.016753407
A_32_P147078	*PITX1*	Homo sapiens paired-like homeodomain 1 (PITX1), mRNA [NM_002653]	24357162	0.0037124872
A_24_P108738	*FAM126B*	Homo sapiens family with sequence similarity 126, member B (FAM126B), mRNA [NM_173822]	-0.8016358	0.004324298
A_23_P1602	*SLC8A1*	Homo sapiens solute carrier family 8 (sodium/calcium exchanger), member 1 (SLC8A1), transcript variant A, mRNA [NM_021097]	-0.6521158	0.0051819514
A_24_P196117	*SCARF2*	Homo sapiens scavenger receptor class F, member 2 (SCARF2), transcript variant 1, mRNA [NM_153334]	0.99596274	0.012334684
A_21_P0014753	*CDC42EP2*	Homo sapiens CDC42 effector protein (Rho GTPase binding) 2 (CDC42EP2), mRNA [NM_006779]	-0.7699497	0.0029471128
A_33_P3234410	*GTF2H5*	Homo sapiens general transcription factor IIH, polypeptide 5 (GTF2H5), mRNA [NM_207118]	-16276689	0.007423588
A_23_P41204	*CAPZB*	Homo sapiens capping protein (actin filament) muscle Z-line, beta (CAPZB), transcript variant 3, mRNA [NM_001206541]	-0.86199045	0.013286371
A_24_P316257	*BOD1L1*	Homo sapiens biorientation of chromosomes in cell division 1-like 1 (BOD1L1), mRNA [NM_148894]	-0.86331856	0.0076919207
A_23_P165494	*FAM131A*	Homo sapiens family with sequence similarity 131, member A (FAM131A), transcript variant 1, mRNA [NM_144635]	0.5556141	0.004473207
A_23_P333951	*NHLRC4*	Homo sapiens NHL repeat containing 4 (NHLRC4), transcript variant 2, mRNA [NM_176677]	0.84123677	0.015141116
A_23_P144071	*GORASP2*	Homo sapiens golgi reassembly stacking protein 2, 55kDa (GORASP2), transcript variant 1, mRNA [NM_015530]	-0.5684127	0.0103437295
A_22_P00007155	*DNAH14*	Homo sapiens dynein, axonemal, heavy chain 14 (DNAH14), transcript variant 3, mRNA [NM_144989]	-0.51595926	0.013662781
A_23_P135357	*COL7A1*	Homo sapiens collagen, type VII, alpha 1 (COL7A1), mRNA [NM_000094]	27354689	0.0043927003
A_23_P140928	*LOC101927269*	Homo sapiens uncharacterized LOC101927269 (LOC101927269), long non-coding RNA [NR_110075]	-0.21032175	0.015011511
A_23_P369666	*RNMTL1*	Homo sapiens RNA methyltransferase like 1 (RNMTL1), mRNA [NM_018146]	-0.5563007	0.0070313537
A_22_P00008681	*TMC7*	Homo sapiens transmembrane channel-like 7 (TMC7), transcript variant 1, mRNA [NM_024847]	-0.7098332	0.016331382

A_21_P0011603	*ZMYND8*	zinc finger, MYND-type containing 8 [Source:HGNC Symbol;Acc:HGNC:9397] [ENST00000468376]	0.5524736	0.0053998437
A_23_P84334	*NUP50-AS1*	Homo sapiens NUP50 antisense RNA 1 (head to head) (NUP50-AS1), transcript variant 1, long non-coding RNA [NR_038956]	-0.69489527	0.010503048
A_33_P3339336	*AMZ2*	Homo sapiens archaelysin family metallopeptidase 2 (AMZ2), transcript variant 1, mRNA [NM_016627]	-10246463	0.0057247286
A_33_P3413840	*TMEM39A*	Homo sapiens transmembrane protein 39A (TMEM39A), transcript variant 1, mRNA [NM_018266]	-0.8367238	0.0064965826
A_33_P3300297	*FAM86FP*	Homo sapiens family with sequence similarity 86, member F, pseudogene (FAM86FP), non-coding RNA [NR_024254]	-10069001	0.009630487
A_22_P00009594	*GK*	Homo sapiens glycerol kinase (GK), transcript variant 4, mRNA [NM_001205019]	14335692	0.006610701
A_23_P17130	*FAM25A*	Homo sapiens family with sequence similarity 25, member A (FAM25A), mRNA [NM_001146157]	18862524	0.007928829
A_23_P51231	*TSPO2*	Homo sapiens translocator protein 2 (TSPO2), transcript variant 1, mRNA [NM_001010873]	0.67786264	0.015756385
A_23_P100420	*C2orf88*	Homo sapiens chromosome 2 open reading frame 88 (C2orf88), transcript variant 1, mRNA [NM_001042519]	-0.7983164	0.015502214
A_23_P44505	*RUNX3*	Homo sapiens runt-related transcription factor 3 (RUNX3), transcript variant 1, mRNA [NM_001031680]	-30562515	0.0042506265
A_24_P408740	*ZCCHC14*	Homo sapiens zinc finger, CCHC domain containing 14 (ZCCHC14), mRNA [NM_015144]	-0.96801376	0.009748889
A_33_P3399248	*KLF11*	Homo sapiens Kruppel-like factor 11 (KLF11), transcript variant 1, mRNA [NM_003597]	-0.62088346	0.013688232
A_24_P18146	*CMC1*	Homo sapiens C-x(9)-C motif containing 1 (CMC1), mRNA [NM_182523]	-0.6224327	0.010105655
A_33_P3407324	*UFM1*	Homo sapiens ubiquitin-fold modifier 1 (UFM1), transcript variant 5, mRNA [NM_001286706]	-0.69070727	0.004832007
A_23_P120557	*PSD3*	Homo sapiens pleckstrin and Sec7 domain containing 3 (PSD3), transcript variant 1, mRNA [NM_015310]	-11185898	0.00736203
A_33_P3266410	*GULP1*	Homo sapiens GULP, engulfment adaptor PTB domain containing 1 (GULP1), transcript variant 2, mRNA [NM_001252668]	11361861	0.0041847117
A_32_P165116	*TASP1*	Homo sapiens taspase, threonine aspartase, 1 (TASP1), mRNA [NM_017714]	-0.8575506	0.0067145773
A_33_P3257187	*MAZ*	Homo sapiens MYC-associated zinc finger protein (purine-binding transcription factor)	12079985	0.010468031

		(MAZ), transcript variant 1, mRNA [NM_002383]		
A_23_P93543	*WDR92*	Homo sapiens WD repeat domain 92 (WDR92), transcript variant 1, mRNA [NM_138458]	-0.6942375	0.0077072685
A_23_P16915	*PRKAA1*	Homo sapiens protein kinase, AMP-activated, alpha 1 catalytic subunit (PRKAA1), transcript variant 2, mRNA [NM_206907]	-0.90301704	0.0038056036
A_24_P414376	*PEX7*	Homo sapiens peroxisomal biogenesis factor 7 (PEX7), mRNA [NM_000288]	-0.90349454	0.00697878
A_23_P400945	*QPCT*	Homo sapiens glutaminyl-peptide cyclotransferase (QPCT), mRNA [NM_012413]	-11087689	0.006933903
A_24_P233570	*KLF3*	Homo sapiens Kruppel-like factor 3 (basic) (KLF3), mRNA [NM_016531]	-0.89162946	0.009765222
A_33_P3392525	*ETV3*	Homo sapiens ets variant 3 (ETV3), transcript variant 2, mRNA [NM_005240]	18125303	0.0070447545
A_24_P129277	*PSMB1*	Homo sapiens proteasome (prosome, macropain) subunit, beta type, 1 (PSMB1), mRNA [NM_002793]	-0.33220553	0.0107979635
A_23_P310274	*ARL4D*	Homo sapiens ADP-ribosylation factor-like 4D (ARL4D), mRNA [NM_001661]	-1976837	0.0029497955
A_23_P18692	*NOD1*	Homo sapiens nucleotide-binding oligomerization domain containing 1 (NOD1), mRNA [NM_006092]	-0.8753799	0.0063477047
A_23_P363826	*PRSS3P2*	Homo sapiens protease, serine, 3 pseudogene 2 (PRSS3P2), non-coding RNA [NR_001296]	2148883	0.003190129
A_22_P00003261	*ADH5*	Homo sapiens alcohol dehydrogenase 5 (class III), chi polypeptide (ADH5), mRNA [NM_000671]	-0.9028332	0.00431082
A_23_P202156	*FBXO3*	Homo sapiens F-box protein 3 (FBXO3), transcript variant 2, mRNA [NM_033406]	-10629536	0.0030041896
A_24_P127159	*lnc-CALHM3-2*	Homo sapiens mRNA; cDNA DKFZp686B1431 (from clone DKFZp686B1431). [CR749568]	0.7885511	0.011740745
A_19_P00316219	*NFKB2*	Homo sapiens nuclear factor of kappa light polypeptide gene enhancer in B-cells 2 (p49/p100) (NFKB2), transcript variant 3, mRNA [NM_001288724]	2357242	0.003922092
A_24_P10233	*DDX11*	Homo sapiens DEAD/H (Asp-Glu-Ala-Asp/His) box helicase 11 (DDX11), transcript variant 5, mRNA [NM_001257145]	17049584	0.016256094
A_23_P390032	*lnc-EYS-2*	Homo sapiens cDNA clone IMAGE:4830172. [BC040303]	-14730698	0.016474942
A_23_P35684	*DAPK2*	Homo sapiens death-associated protein kinase 2 (DAPK2), mRNA [NM_014326]	-21955602	0.0037856582
A_33_P3359518	*SLC35G1*	Homo sapiens solute carrier family 35, member G1 (SLC35G1), transcript variant 2, mRNA [NM_153226]	-0.22042024	0.006573993

A_33_P322130 3	*INPP5F*	Homo sapiens inositol polyphosphate-5-phosphatase F (INPP5F), transcript variant 1, mRNA [NM_014937]	-0.78335935	0.010931163
A_23_P401106	*MYSM1*	Homo sapiens Myb-like, SWIRM and MPN domains 1 (MYSM1), mRNA [NM_001085487]	-0.6590861	0.013050359
A_33_P340792 5	*CCR10*	Homo sapiens chemokine (C-C motif) receptor 10 (CCR10), mRNA [NM_016602]	0.74016863	0.012030219
A_23_P368259	*PDE2A*	Homo sapiens phosphodiesterase 2A, cGMP-stimulated (PDE2A), transcript variant 1, mRNA [NM_002599]	-12955283	0.008273812
A_33_P341567 8	*VMP1*	Homo sapiens vacuole membrane protein 1 (VMP1), mRNA [NM_030938]	-12142942	0.005635523 2
A_33_P324624 4	*EID2B*	EP300 interacting inhibitor of differentiation 2B [Source:HGNC Symbol;Acc:HGNC:26796] [ENST00000326282]	-0.36780998	0.014154868
A_33_P336215 3	*ATF4*	Homo sapiens activating transcription factor 4 (ATF4), transcript variant 1, mRNA [NM_001675]	-0.5379743	0.007718046 7
A_23_P253958	*TMEM238*	Homo sapiens transmembrane protein 238 (TMEM238), mRNA [NM_001190764]	0.99129605	0.004424034 6
A_24_P370970	*MT1E*	Homo sapiens metallothionein 1E (MT1E), mRNA [NM_175617]	0.5476379	0.014834769
A_23_P47879	*LRRC17*	Homo sapiens leucine rich repeat containing 17 (LRRC17), transcript variant 2, mRNA [NM_005824]	2268224	0.003194129 3
A_33_P334581 6	*NSL1*	Homo sapiens NSL1, MIS12 kinetochore complex component (NSL1), transcript variant 1, mRNA [NM_015471]	-0.61210674	0.012215228
A_33_P332238 8	*STAT6*	Homo sapiens signal transducer and activator of transcription 6, interleukin-4 induced (STAT6), transcript variant 2, mRNA [NM_003153]	-1.6363006	0.004818115 4
A_24_P36097	*GPER1*	Homo sapiens G protein-coupled estrogen receptor 1 (GPER1), transcript variant 3, mRNA [NM_001039966]	-1.0491427	0.010274393
A_23_P315589	*SPRR2D*	Homo sapiens small proline-rich protein 2D (SPRR2D), mRNA [NM_006945]	0.8272072	0.011843565
A_23_P432034	*RPAIN*	Homo sapiens RPA interacting protein (RPAIN), transcript variant 5, mRNA [NM_001160266]	-0.41608196	0.012476785
A_23_P6914	*KIAA0930*	Homo sapiens KIAA0930 (KIAA0930), transcript variant 1, mRNA [NM_015264]	1.0093819	0.011636669
A_23_P34983	*CCDC117*	Homo sapiens coiled-coil domain containing 117 (CCDC117), transcript variant 1, mRNA [NM_173510]	-1.3232465	0.003640249 6
A_21_P001257 1	*OSBPL11*	Homo sapiens oxysterol binding protein-like 11 (OSBPL11), mRNA [NM_022776]	-0.6850488	0.005907049 4
A_24_P182539	*JTB*	Homo sapiens jumping translocation breakpoint (JTB), mRNA [NM_006694]	-0.47807166	0.015841465

A_23_P256223	*ATG4C*	Homo sapiens autophagy related 4C, cysteine peptidase (ATG4C), transcript variant 1, mRNA [NM_032852]	-0.5211155	0.010783317
A_24_P316305	*DSP*	Homo sapiens desmoplakin (DSP), transcript variant 1, mRNA [NM_004415]	-3.1334207	0.0024890031
A_33_P3269806	*VBP1*	von Hippel-Lindau binding protein 1 [Source:HGNC Symbol;Acc:HGNC:12662] [ENST00000460509]	-0.48752862	0.0058383252
A_24_P131392	*AQR*	aquarius intron-binding spliceosomal factor [Source:HGNC Symbol;Acc:HGNC:29513] [ENST00000156471]	-0.93701553	0.013354005
A_23_P214091	*ZNF616*	Homo sapiens zinc finger protein 616 (ZNF616), mRNA [NM_178523]	-1.2061464	0.011193861
A_24_P111912	*RMDN2*	Homo sapiens regulator of microtubule dynamics 2 (RMDN2), transcript variant 1, mRNA [NM_144713]	-0.82140183	0.0023340243
A_33_P3299066	*LYPLA1*	Homo sapiens lysophospholipase I (LYPLA1), transcript variant 1, mRNA [NM_006330]	-0.551218	0.006266305
A_33_P3233580	*FAM172A*	Homo sapiens family with sequence similarity 172, member A (FAM172A), transcript variant 1, mRNA [NM_032042]	-1.0399966	0.012965681
A_19_P00809682	*NR4A2*	Homo sapiens nuclear receptor subfamily 4, group A, member 2 (NR4A2), mRNA [NM_006186]	4.1945543	0.0025001846
A_32_P168464	*KIAA1217*	Homo sapiens KIAA1217 (KIAA1217), transcript variant 6, mRNA [NM_001282769]	-1.1460866	0.01273111
A_33_P3343573	*lnc-FAM105B-1*	Homo sapiens cDNA clone IMAGE:4309177, **** WARNING: chimeric clone ****. [BC014023]	-0.5299855	0.008066016
A_32_P157208	*CASK*	Homo sapiens calcium/calmodulin-dependent serine protein kinase (MAGUK family) (CASK), transcript variant 1, mRNA [NM_003688]	-0.8037696	0.007479662
A_21_P0011306	*PGM5P3-AS1*	Homo sapiens PGM5P3 antisense RNA 1 (PGM5P3-AS1), transcript variant 1, long non-coding RNA [NR_121188]	2.9037466	0.00180587
A_23_P414913	*PRPF18*	Homo sapiens pre-mRNA processing factor 18 (PRPF18), mRNA [NM_003675]	-0.8480234	0.0038487043
A_33_P3214129	*SYNGR2*	Homo sapiens synaptogyrin 2 (SYNGR2), mRNA [NM_004710]	1.4939916	0.007049496
A_23_P55926	*GLIPR2*	Homo sapiens GLI pathogenesis-related 2 (GLIPR2), transcript variant 1, mRNA [NM_022343]	-1.8300965	0.0026564114
A_23_P61524	*LOC728061*	Homo sapiens cDNA: FLJ21498 fis, clone COL05627. [AK025151]	-2.44437	0.0030069214
A_24_P33982	*NR1H2*	Homo sapiens nuclear receptor subfamily 1, group H, member 2 (NR1H2), transcript variant 1, mRNA [NM_007121]	0.8766808	0.009667313

A_22_P00004217	*CCDC71*	Homo sapiens coiled-coil domain containing 71 (CCDC71), mRNA [NM_022903]	-0.8841584	0.01478167
A_23_P128919	*MILR1*	Homo sapiens mast cell immunoglobulin-like receptor 1 (MILR1), transcript variant S2, mRNA [NM_001291317]	1.8111498	0.0044707763
A_24_P150874	*C1RL-AS1*	Homo sapiens C1RL antisense RNA 1 (C1RL-AS1), long non-coding RNA [NR_026947]	0.44430164	0.016487706
A_23_P18493	*LGALS3*	Homo sapiens lectin, galactoside-binding, soluble, 3 (LGALS3), transcript variant 1, mRNA [NM_002306]	-0.30450034	0.007502627
A_33_P3266315	*GNA13*	Homo sapiens guanine nucleotide binding protein (G protein), alpha 13 (GNA13), transcript variant 1, mRNA [NM_006572]	-0.903008	0.004098441
A_33_P3284290	*PTPN13*	Homo sapiens protein tyrosine phosphatase, non-receptor type 13 (APO-1/CD95 (Fas)-associated phosphatase) (PTPN13), transcript variant 4, mRNA [NM_080685]	-1.1253653	0.0036402496
A_23_P40847	*SH3GLB1*	Homo sapiens SH3-domain GRB2-like endophilin B1 (SH3GLB1), transcript variant 2, mRNA [NM_001206651]	-0.58671254	0.01211171
A_33_P3412716	*ZNF778*	Homo sapiens zinc finger protein 778 (ZNF778), transcript variant 1, mRNA [NM_001201407]	-0.9767716	0.015141116
A_23_P333705	*CHST2*	Homo sapiens carbohydrate (N-acetylglucosamine-6-O) sulfotransferase 2 (CHST2), mRNA [NM_004267]	-1.9484061	0.0052314615
A_21_P0000408	*NEK3*	Homo sapiens NIMA-related kinase 3 (NEK3), transcript variant 1, mRNA [NM_002498]	-0.93276334	0.010328656
A_23_P154457	*TMEM106B*	Homo sapiens transmembrane protein 106B (TMEM106B), transcript variant 1, mRNA [NM_018374]	-0.21106364	0.008366524
A_23_P401606	*SNORD114-11*	Homo sapiens small nucleolar RNA, C/D box 114-11 (SNORD114-11), small nucleolar RNA [NR_003204]	0.6674713	0.0031941293
A_33_P3340105	*NOL10*	Homo sapiens nucleolar protein 10 (NOL10), transcript variant 1, mRNA [NM_024894]	-0.5859089	0.010750983
A_24_P118011	*EDIL3*	Homo sapiens EGF-like repeats and discoidin I-like domains 3 (EDIL3), transcript variant 1, mRNA [NM_005711]	1.4272581	0.0034534817
A_33_P3888629	*BASP1P1*	Homo sapiens brain abundant, membrane attached signal protein 1 pseudogene 1 (BASP1P1), non-coding RNA [NR_033774]	1.0660833	0.0070711365
A_23_P337934	*PRORSD1P*	Homo sapiens prolyl-tRNA synthetase associated domain containing 1, pseudogene (PRORSD1P), non-coding RNA [NR_027258]	-0.973074	0.013327314
A_32_P131143	*MECOM*	Homo sapiens MDS1 and EVI1 complex locus (MECOM), transcript variant 6, mRNA [NM_001164000]	2.6455607	0.0029471128

A_33_P3294881	*FBLIM1*	Homo sapiens filamin binding LIM protein 1 (FBLIM1), transcript variant 1, mRNA [NM_017556]	-1.3207605	0.007177978
A_33_P3217834	*CECR5-AS1*	Homo sapiens CECR5 antisense RNA 1 (CECR5-AS1), transcript variant 2, long non-coding RNA [NR_024482]	-1.3777219	0.007111782
A_23_P21473	*CCDC174*	Homo sapiens coiled-coil domain containing 174 (CCDC174), mRNA [NM_016474]	-1.0656084	0.00628389
A_23_P337201	*SMARCAL1*	Homo sapiens SWI/SNF related, matrix associated, actin dependent regulator of chromatin, subfamily a-like 1 (SMARCAL1), transcript variant 1, mRNA [NM_014140]	-1.1554383	0.00687448
A_23_P164451	*CEP70*	Homo sapiens centrosomal protein 70kDa (CEP70), transcript variant 1, mRNA [NM_024491]	-0.7144474	0.0057916977
A_22_P00008958	*SRP72*	Homo sapiens signal recognition particle 72kDa (SRP72), transcript variant 1, mRNA [NM_006947]	-0.5487389	0.0059050764
A_23_P255695	*TBX2*	Homo sapiens T-box 2 (TBX2), mRNA [NM_005994]	3.2077339	0.003363001
A_23_P259506	*SLC17A3*	Homo sapiens solute carrier family 17 (organic anion transporter), member 3 (SLC17A3), transcript variant 2, mRNA [NM_006632]	-0.6308619	0.011949872
A_24_P243776	*XLOC_l2_005438*	BROAD Institute lincRNA (XLOC_l2_005438), lincRNA [TCONS_l2_00010088]	1.2784113	0.002802766
A_21_P0013621	*CYSTM1*	Homo sapiens cysteine-rich transmembrane module containing 1 (CYSTM1), mRNA [NM_032412]	-0.7842431	0.011106028
A_23_P409626	*ZNF277*	Homo sapiens zinc finger protein 277 (ZNF277), mRNA [NM_021994]	-1.1085538	0.002819256
A_33_P3372281	*XLOC_l2_015121*	BROAD Institute lincRNA (XLOC_l2_015121), lincRNA [TCONS_l2_00029212]	0.93332684	0.0108927535
A_24_P416997	*HNRNPUL2*	Homo sapiens heterogeneous nuclear ribonucleoprotein U-like 2 (HNRNPUL2), mRNA [NM_001079559]	-0.890764	0.016750982
A_33_P3293247	*MRS2*	Homo sapiens MRS2 magnesium transporter (MRS2), transcript variant 3, mRNA [NM_001286265]	-1.2891206	0.0073845843
A_33_P3243153	*APOL3*	Homo sapiens apolipoprotein L, 3 (APOL3), transcript variant beta/a, mRNA [NM_145641]	-1.0872755	0.016539587
A_23_P65558	*GPSM1*	Homo sapiens G-protein signaling modulator 1 (GPSM1), transcript variant 2, mRNA [NM_015597]	-0.7499275	0.008976597
A_23_P39602	*GFPT1*	Homo sapiens glutamine--fructose-6-phosphate transaminase 1 (GFPT1), transcript variant 1, mRNA [NM_001244710]	-0.61488074	0.0117289275
A_24_P82880	*MGAT2*	Homo sapiens mannosyl (alpha-1,6-)-glycoprotein beta-1,2-N-	-0.659533	0.0031750489

		acetylglucosaminyltransferase (MGAT2), mRNA [NM_002408]		
A_33_P3326432	*NCOA1*	Homo sapiens nuclear receptor coactivator 1 (NCOA1), transcript variant 3, mRNA [NM_147233]	-0.97319984	0.008392573
A_24_P21447	*TPM4*	Homo sapiens tropomyosin 4 (TPM4), transcript variant Tpm4.2, mRNA [NM_003290]	-0.72817516	0.014154771
A_21_P0013768	*SEPW1*	Homo sapiens selenoprotein W, 1 (SEPW1), mRNA [NM_003009]	-1.4561431	0.0059758257
A_23_P156025	*SURF6*	Homo sapiens surfeit 6 (SURF6), transcript variant 1, mRNA [NM_006753]	-0.47925714	0.01704795
A_24_P360722	*XLOC_l2_015542*	BROAD Institute lincRNA (XLOC_l2_015542), lincRNA [TCONS_l2_00030232]	-0.27379274	0.010397241
A_23_P128698	*IRX2*	Homo sapiens iroquois homeobox 2 (IRX2), transcript variant 1, mRNA [NM_033267]	-1.567856948477316	0.0032589228
A_23_P56709	*DIP2C*	Homo sapiens DIP2 disco-interacting protein 2 homolog C (Drosophila) (DIP2C), mRNA [NM_014974]	-0.8030295	0.006740723
A_24_P333857	*SPRY2*	Homo sapiens sprouty homolog 2 (Drosophila) (SPRY2), mRNA [NM_005842]	1.153188	0.004359316
A_23_P216596	*RNF103*	Homo sapiens ring finger protein 103 (RNF103), transcript variant 1, mRNA [NM_005667]	-1.0096396	0.005192215
A_33_P3363012	*SGIP1*	Homo sapiens SH3-domain GRB2-like (endophilin) interacting protein 1 (SGIP1), mRNA [NM_032291]	0.51033765	0.01172006
A_23_P125519	*SVEP1*	Homo sapiens sushi, von Willebrand factor type A, EGF and pentraxin domain containing 1 (SVEP1), mRNA [NM_153366]	0.70908666	0.014448771
A_23_P259314	*PRPF19*	Homo sapiens pre-mRNA processing factor 19 (PRPF19), mRNA [NM_014502]	-0.46784928	0.007332902
A_22_P00008062	*RPS4X*	Homo sapiens ribosomal protein S4, X-linked (RPS4X), mRNA [NM_001007]	-0.15534218	0.013286371
A_33_P3422113	*RPS4Y1*	Homo sapiens ribosomal protein S4, Y-linked 1 (RPS4Y1), mRNA [NM_001008]	-0.9011263	0.004437345
A_23_P333592	*AADACL2-AS1*	Homo sapiens AADACL2 antisense RNA 1 (AADACL2-AS1), transcript variant 2, long non-coding RNA [NR_110203]	-0.9487294	0.016068412
A_33_P3321150	*ZSCAN12P1*	Homo sapiens zinc finger and SCAN domain containing 12 pseudogene 1 (ZSCAN12P1), non-coding RNA [NR_024063]	0.5642443	0.012535833
A_23_P125624	*ZNF547*	zinc finger protein 547 [Source:HGNC Symbol;Acc:HGNC:26432] [ENST00000282282]	-1.5391709	0.0043846127
A_33_P3364268	*CUL3*	Homo sapiens cullin 3 (CUL3), transcript variant 1, mRNA [NM_003590]	-0.5968809	0.0057496065

A_33_P3265744	*ACOT9*	Homo sapiens acyl-CoA thioesterase 9 (ACOT9), transcript variant 1, mRNA [NM_001037171]	-0.94574165	0.0047719665
A_23_P10591	*LBH*	Homo sapiens limb bud and heart development (LBH), mRNA [NM_030915]	-3.3363233	0.0030069214
A_33_P3257150	*PTGER3*	Homo sapiens prostaglandin E receptor 3 (subtype EP3) (PTGER3), transcript variant 5, mRNA [NM_198715]	1.0717167	0.0065723537
A_24_P6903	*METRNL*	Homo sapiens meteorin, glial cell differentiation regulator-like (METRNL), mRNA [NM_001004431]	-1.5935738	0.0031941293
A_33_P3241521	*CDC42*	Homo sapiens cell division cycle 42 (CDC42), transcript variant 3, mRNA [NM_001039802]	-0.8959601	0.010822477
A_33_P3349334	*ACTBL2*	Homo sapiens actin, beta-like 2 (ACTBL2), mRNA [NM_001017992]	0.7555101	0.015833624
A_33_P3258846	*EBF1*	Homo sapiens early B-cell factor 1 (EBF1), transcript variant 1, mRNA [NM_001290360]	-2.816972	0.013522089
A_22_P00022364	*SOCS4*	Homo sapiens suppressor of cytokine signaling 4 (SOCS4), transcript variant 1, mRNA [NM_199421]	-0.6705911	0.00942879
A_23_P170820	*LOC100507054*	PREDICTED: Homo sapiens uncharacterized LOC100507054 (LOC100507054), ncRNA [XR_108535]	-1.3964788	0.0040942165
A_33_P3370751	*AKAP1*	Homo sapiens A kinase (PRKA) anchor protein 1 (AKAP1), transcript variant 1, mRNA [NM_003488]	-0.61604565	0.011656639
A_21_P0005552	*PUM1*	Homo sapiens pumilio RNA-binding family member 1 (PUM1), transcript variant 1, mRNA [NM_001020658]	-0.7326705	0.014424864
A_33_P3229335	*SMURF2*	Homo sapiens SMAD specific E3 ubiquitin protein ligase 2 (SMURF2), mRNA [NM_022739]	0.6548298	0.010058273
A_33_P3309471	*lnc-EVX1-5*	LNCipedia lincRNA (lnc-EVX1-5), lincRNA [lnc-EVX1-5:3]	-0.59070915	0.015840316
A_23_P154306	*HIST3H2BB*	Homo sapiens histone cluster 3, H2bb (HIST3H2BB), mRNA [NM_175055]	1.149993	0.00856985
A_24_P29445	*PTPRQ*	Homo sapiens protein tyrosine phosphatase, receptor type, Q (PTPRQ), mRNA [NM_001145026]	2.21722	0.0031941293
A_21_P0000595	*TANK*	Homo sapiens TRAF family member-associated NFKB activator (TANK), transcript variant 1, mRNA [NM_004180]	-0.96481436	0.0066501293
A_33_P3269598	*TMEM14B*	Homo sapiens transmembrane protein 14B (TMEM14B), transcript variant 1, mRNA [NM_030969]	-0.69592446	0.0031941293
A_32_P100439	*SNORA70E*	Homo sapiens small nucleolar RNA, H/ACA box 70E (SNORA70E), small nucleolar RNA [NR_033347]	0.71377206	0.011308836

A_23_P339079	*ERCC6L2*	Homo sapiens excision repair cross-complementation group 6-like 2 (ERCC6L2), transcript variant 1, mRNA [NM_020207]	-0.59312993	0.015820691
A_23_P321703	*MTURN*	Homo sapiens maturin, neural progenitor differentiation regulator homolog (Xenopus) (MTURN), mRNA [NM_152793]	-0.8694885	0.010567525
A_21_P0003919	*ZNF573*	Homo sapiens zinc finger protein 573 (ZNF573), transcript variant 1, mRNA [NM_152360]	-0.8904171	0.004016376
A_24_P250922	*BCL2A1*	Homo sapiens BCL2-related protein A1 (BCL2A1), transcript variant 1, mRNA [NM_004049]	-0.5077819	0.014941534
A_23_P141005	*lnc-FRG2-5*	LNCipedia lincRNA (lnc-FRG2-5), lincRNA [lnc-FRG2-5:2]	0.67234665	0.012854842
A_33_P3209404	*PTGS2*	Homo sapiens prostaglandin-endoperoxide synthase 2 (prostaglandin G/H synthase and cyclooxygenase) (PTGS2), mRNA [NM_000963]	1.0602975	0.0029015075
A_33_P3292218	*AMFR*	Homo sapiens autocrine motility factor receptor, E3 ubiquitin protein ligase (AMFR), mRNA [NM_001144]	-10343187	0.0067678746
A_24_P280378	*XLOC_l2_006173*	BROAD Institute lincRNA (XLOC_l2_006173), lincRNA [TCONS_l2_00011427]	1.322135	0.004993122
A_23_P166910	*AQR*	Homo sapiens aquarius intron-binding spliceosomal factor (AQR), mRNA [NM_014691]	-0.86655855	0.008749787
A_23_P316812	*GNAS-AS1*	Homo sapiens GNAS antisense RNA 1 (GNAS-AS1), antisense RNA [NR_002785]	-0.9649571	0.0066818837
A_33_P3305915	*DNAJC13*	Homo sapiens DnaJ (Hsp40) homolog, subfamily C, member 13 (DNAJC13), mRNA [NM_015268]	-0.5901322	0.008298705
A_21_P0012379	*AKR7A2P1*	Homo sapiens aldo-keto reductase family 7, member A2 pseudogene 1 (AKR7A2P1), non-coding RNA [NR_002796]	0.5152797	0.014391575
A_33_P3333992	*GGA1*	Homo sapiens golgi-associated, gamma adaptin ear containing, ARF binding protein 1 (GGA1), transcript variant 5, mRNA [NM_001172688]	0.6940862	0.015048716
A_24_P393958	*XLOC_l2_009773*	BROAD Institute lincRNA (XLOC_l2_009773), lincRNA [TCONS_l2_00018665]	0.8990722	0.006888116
A_33_P3281572	*LOC100132495*	Homo sapiens cDNA FLJ45506 fis, clone BRTHA2020695. [AK127414]	-1.0691736	0.0059353677
A_23_P90333	*DNAJB4*	Homo sapiens DnaJ (Hsp40) homolog, subfamily B, member 4 (DNAJB4), mRNA [NM_007034]	-1.099776	0.0034156272
A_23_P169838	*CMAHP*	Homo sapiens cytidine monophospho-N-acetylneuraminic acid hydroxylase, pseudogene (CMAHP), transcript variant 1, non-coding RNA [NR_002174]	-2.0941503	0.0040841834

A_21_P0008398	*ZNF404*	Homo sapiens zinc finger protein 404 (ZNF404), mRNA [NM_001033719]	-1.0093082	0.009450744
A_23_P362637	*SUN1*	Homo sapiens Sad1 and UNC84 domain containing 1 (SUN1), transcript variant 2, mRNA [NM_025154]	-0.9211753	0.008106991
A_33_P3290955	*lnc-CTD-2134A5.2.1-2*	LNCipedia lincRNA (lnc-CTD-2134A5.2.1-2), lincRNA [lnc-CTD-2134A5.2.1-2:1]	1.3273847	0.0028288076
A_33_P3275381	*UBR2*	Homo sapiens ubiquitin protein ligase E3 component n-recognin 2 (UBR2), transcript variant 1, mRNA [NM_015255]	-0.93593115	0.0062257075
A_33_P3333712	*KIAA1875*	Homo sapiens KIAA1875 (KIAA1875), long non-coding RNA [NR_024207]	3.1070886	0.004626585
A_19_P00803499	*MAP9*	Homo sapiens microtubule-associated protein 9 (MAP9), mRNA [NM_001039580]	-1.2209578	0.005428736
A_23_P142671	*SCYL2*	Homo sapiens SCY1-like 2 (S. cerevisiae) (SCYL2), mRNA [NM_017988]	-0.64377904	0.010272158
A_23_P46539	*ZNF513*	Homo sapiens zinc finger protein 513 (ZNF513), transcript variant 1, mRNA [NM_144631]	0.963004	0.009485806
A_33_P3390758	*lnc-PPA2-1*	Homo sapiens cDNA clone IMAGE:4309177, **** WARNING: chimeric clone ****. [BC014023]	-0.30908465	0.0037856582
A_21_P0000328	*PSRC1*	Homo sapiens proline/serine-rich coiled-coil 1 (PSRC1), transcript variant 1, mRNA [NM_032636]	-0.7302773	0.013286371
A_21_P0006566	*HSPA8*	Homo sapiens heat shock 70kDa protein 8 (HSPA8), transcript variant 2, mRNA [NM_153201]	0.68175334	0.010971502
A_33_P3411145	*SNORA40*	Homo sapiens small nucleolar RNA, H/ACA box 40 (SNORA40), small nucleolar RNA [NR_002973]	-1.1955506	0.00975317
A_33_P3280030	*lnc-RAB40AL-1*	BX114424 Soares placenta Nb2HP Homo sapiens cDNA clone IMAGp998L02197, mRNA sequence [BX114424]	-0.79795265	0.00324898
A_33_P3346483	*FAM200B*	Homo sapiens family with sequence similarity 200, member B (FAM200B), mRNA [NM_001145191]	-1.0714173	0.016205462
A_23_P211136	*FRA10AC1*	Homo sapiens fragile site, folic acid type, rare, fra(10)(q23.3) or fra(10)(q24.2) candidate 1 (FRA10AC1), mRNA [NM_145246]	-0.9742112	0.014424864
A_23_P92202	*SYNPO2L*	Homo sapiens synaptopodin 2-like (SYNPO2L), transcript variant 1, mRNA [NM_001114133]	2.5342689	0.007479662
A_23_P111860	*BRWD1*	Homo sapiens bromodomain and WD repeat domain containing 1 (BRWD1), transcript variant 1, mRNA [NM_018963]	-0.8180318	0.003392081
A_22_P00000104	*GMPPB*	Homo sapiens GDP-mannose pyrophosphorylase B (GMPPB), transcript variant 2, mRNA [NM_021971]	0.6222918	0.016620774

A_23_P70213	*RADIL*	Homo sapiens Ras association and DIL domains (RADIL), mRNA [NM_018059]	-1.1141775	0.0031941293
A_22_P00020936	*lnc-NDFIP1-3*	Homo sapiens cDNA: FLJ20903 fis, clone ADSE00222. [AK024556]	0.97202414	0.0074661016
A_23_P201483	*APC*	Homo sapiens adenomatous polyposis coli (APC), transcript variant 3, mRNA [NM_000038]	-0.91854674	0.013124348
A_23_P63847	*lnc-UNC93B1-2*	LNCipedia lincRNA (lnc-UNC93B1-2), lincRNA [lnc-UNC93B1-2:1]	-1.169146	0.009784609
A_33_P3416772	*MAPKAPK2*	Homo sapiens mitogen-activated protein kinase-activated protein kinase 2 (MAPKAPK2), transcript variant 1, mRNA [NM_004759]	0.74371576	0.011187486
A_23_P43425	*SUPV3L1*	Homo sapiens suppressor of var1, 3-like 1 (S. cerevisiae) (SUPV3L1), transcript variant 1, mRNA [NM_003171]	-1.1316596	0.0037583082
A_21_P0014920	*C1orf159*	Homo sapiens chromosome 1 open reading frame 159 (C1orf159), mRNA [NM_017891]	0.89681983	0.009387307
A_23_P255653	*C9orf40*	Homo sapiens chromosome 9 open reading frame 40 (C9orf40), mRNA [NM_017998]	-0.49244526	0.015505246
A_23_P203645	*TNFRSF10A*	Homo sapiens tumor necrosis factor receptor superfamily, member 10a (TNFRSF10A), mRNA [NM_003844]	-0.7287373	0.016825441
A_33_P3494748	*CREBZF*	Homo sapiens CREB/ATF bZIP transcription factor (CREBZF), transcript variant 1, mRNA [NM_001039618]	-0.82164925	0.0062516355
A_33_P3222243	*MIR100HG*	Homo sapiens mir-100-let-7a-2 cluster host gene (non-protein coding) (MIR100HG), long non-coding RNA [NR_024430]	-0.7280554	0.014951782
A_24_P472455	*TMEM65*	Homo sapiens transmembrane protein 65 (TMEM65), mRNA [NM_194291]	-1.865504	0.0023561441
A_23_P64617	*MED15P9*	mediator complex subunit 15 pseudogene 9 [Source:HGNC Symbol;Acc:HGNC:44130] [ENST00000424716]	1.1901097	0.005852893
A_21_P0011577	*ARF6*	Homo sapiens ADP-ribosylation factor 6 (ARF6), mRNA [NM_001663]	-0.9701133	0.004723954
A_21_P0004569	*FZD4*	Homo sapiens frizzled class receptor 4 (FZD4), mRNA [NM_012193]	-1.019089	0.008574389
A_33_P3297155	*TNPO1*	Homo sapiens transportin 1 (TNPO1), transcript variant 1, mRNA [NM_002270]	-0.63186765	0.00929522
A_32_P70724	*LUCAT1*	Homo sapiens lung cancer associated transcript 1 (non-protein coding) (LUCAT1), transcript variant 2, long non-coding RNA [NR_103549]	0.94914615	0.0055521973
A_33_P3220470	*GEMIN2*	Homo sapiens gem (nuclear organelle) associated protein 2 (GEMIN2), transcript variant alpha, mRNA [NM_003616]	-0.927828	0.009765222
A_22_P00011122	*KDM5B*	Homo sapiens lysine (K)-specific demethylase 5B (KDM5B), mRNA [NM_006618]	-0.8989723	0.009182041

A_23_P65768	*SMAD6*	Homo sapiens SMAD family member 6 (SMAD6), transcript variant 1, mRNA [NM_005585]	-1.1102433	0.006146274
A_23_P5441	*lnc-NUMB-2*	BX101396 Soares_testis_NHT Homo sapiens cDNA clone IMAGp998D114108, mRNA sequence [BX101396]	1.5413623	0.0046764575
A_33_P3308903	*RSL24D1*	Homo sapiens ribosomal L24 domain containing 1 (RSL24D1), mRNA [NM_016304]	-0.7367492	0.004197023
A_33_P3258617	*ABCB6*	Homo sapiens ATP-binding cassette, sub-family B (MDR/TAP), member 6 (Langereis blood group) (ABCB6), mRNA [NM_005689]	-0.73711175	0.009545733
A_23_P4190	*MIB2*	Homo sapiens mindbomb E3 ubiquitin protein ligase 2 (MIB2), transcript variant 1, mRNA [NM_080875]	0.7664199	0.008771556
A_23_P122052	*PLIN2*	perilipin 2 [Source:HGNC Symbol;Acc:HGNC:248] [ENST00000380464]	0.5860032	0.010804776
A_23_P8763	*ACSF2*	Homo sapiens acyl-CoA synthetase family member 2 (ACSF2), transcript variant 2, mRNA [NM_025149]	-1.014122	0.010782863
A_22_P00018086	*GPX8*	Homo sapiens glutathione peroxidase 8 (putative) (GPX8), mRNA [NM_001008397]	-0.2624268	0.0063667204
A_33_P3243717	*PTPN12*	Homo sapiens protein tyrosine phosphatase, non-receptor type 12 (PTPN12), transcript variant 1, mRNA [NM_002835]	-0.8361771	0.011558667
A_22_P00008083	*ZNF248*	Homo sapiens zinc finger protein 248 (ZNF248), transcript variant 1, mRNA [NM_021045]	-0.774976	0.0058377855
A_32_P48615	*TMEM71*	Homo sapiens transmembrane protein 71 (TMEM71), transcript variant 1, mRNA [NM_144649]	1.0579783	0.0077268425
A_19_P00315780	*LOC101929761*	PREDICTED: Homo sapiens uncharacterized LOC101929761 (LOC101929761), transcript variant X2, ncRNA [XR_428452]	-1.3704246	0.005348777
A_23_P40588	*LOC101929709*		-0.6136309	0.011969343
A_23_P121506	*WDR20*	Homo sapiens WD repeat domain 20 (WDR20), transcript variant 1, mRNA [NM_181291]	-0.75593835	0.012134408
A_21_P0006135	*HSCB*	Homo sapiens HscB mitochondrial iron-sulfur cluster co-chaperone (HSCB), mRNA [NM_172002]	-0.35391662	0.0160096
A_33_P3286616	*FAM193A*	Homo sapiens family with sequence similarity 193, member A (FAM193A), transcript variant 1, mRNA [NM_003704]	0.4381747	0.015905993
A_23_P58396	*lnc-IFNK-1*	LNCipedia lincRNA (lnc-IFNK-1), lincRNA [lnc-IFNK-1:1]	-1.0031395	0.014904488
A_22_P00019988	*IRAK1*	Homo sapiens interleukin-1 receptor-associated kinase 1 (IRAK1), transcript variant 1, mRNA [NM_001569]	1.5685085	0.0044240346

A_23_P218731	*PDGFC*	Homo sapiens platelet derived growth factor C (PDGFC), transcript variant 1, mRNA [NM_016205]	-1.071883	0.0037583082
A_21_P0000572	*lnc-GTPBP1-2*	Q504T5_HUMAN (Q504T5) UNC84B protein, partial (3%) [THC2558827]	0.91492796	0.009507545
A_23_P259328	*CRYZL1*	Homo sapiens crystallin, zeta (quinone reductase)-like 1 (CRYZL1), mRNA [NM_145858]	-0.97524476	0.011289068
A_22_P00000010	*PITPNA-AS1*	Homo sapiens PITPNA antisense RNA 1 (PITPNA-AS1), long non-coding RNA [NR_028514]	-0.5648701	0.014922057
A_24_P360269	*PEX3*	Homo sapiens peroxisomal biogenesis factor 3 (PEX3), mRNA [NM_003630]	-0.84441334	0.010612081
A_33_P3338968	*ADAM12*	Homo sapiens ADAM metallopeptidase domain 12 (ADAM12), transcript variant 1, mRNA [NM_003474]	-2.1143215	0.005701378
A_23_P101950	*RNASET2*	Homo sapiens ribonuclease T2 (RNASET2), mRNA [NM_003730]	-1.1155593	0.0100634415
A_32_P109922	*MDH1*	Homo sapiens malate dehydrogenase 1, NAD (soluble) (MDH1), transcript variant 2, mRNA [NM_005917]	-0.6443054	0.008564741
A_23_P164623	*LOC389033*	Homo sapiens placenta-specific 9 pseudogene (LOC389033), non-coding RNA [NR_026740]	-2.372497	0.004465531
A_24_P38347	*ZFAT*	Homo sapiens zinc finger and AT hook domain containing (ZFAT), transcript variant 1, mRNA [NM_020863]	-0.8374219	0.01387464
A_23_P105664	*ZBTB45*	Homo sapiens zinc finger and BTB domain containing 45 (ZBTB45), mRNA [NM_032792]	-0.3740912	0.007794005
A_22_P00013800	*DPYSL2*	Homo sapiens dihydropyrimidinase-like 2 (DPYSL2), transcript variant 2, mRNA [NM_001386]	-0.67362404	0.004832007
A_23_P46222	*CCDC59*	Homo sapiens coiled-coil domain containing 59 (CCDC59), transcript variant 1, mRNA [NM_014167]	-0.6132498	0.012365967
A_33_P3332438	*lnc-RP4-604K5.1.1-2*	Homo sapiens cDNA clone IMAGE:4831215. [BC034638]	-0.7989173	0.0069863
A_33_P3319765	*TRIM46*	Homo sapiens tripartite motif containing 46 (TRIM46), transcript variant 8, mRNA [NM_001282379]	0.32549143	0.010593901
A_23_P318300	*GINM1*	Homo sapiens glycoprotein integral membrane 1 (GINM1), mRNA [NM_138785]	-0.8061237	0.01211171
A_33_P3372217	*FANCC*	Homo sapiens Fanconi anemia, complementation group C (FANCC), transcript variant 3, mRNA [NM_001243744]	-1.27953	0.0038222359
A_33_P3412353	*ZAK*	Homo sapiens sterile alpha motif and leucine zipper containing kinase AZK (ZAK), transcript variant 2, mRNA [NM_133646]	-0.63787794	0.013711676

A_24_P371053	*PSG5*	pregnancy specific beta-1-glycoprotein 5 [Source:HGNC Symbol;Acc:HGNC:9522] [ENST00000401992]	1.7338877	0.005400047
A_24_P251841	*ZNF268*	Homo sapiens zinc finger protein 268 (ZNF268), transcript variant 3, mRNA [NM_152943]	-0.8302679	0.014008534
A_23_P115885	*ORMDL1*	Homo sapiens ORMDL sphingolipid biosynthesis regulator 1 (ORMDL1), transcript variant 1, mRNA [NM_016467]	-0.84365964	0.005172653
A_23_P202458	*SETD7*	Homo sapiens SET domain containing (lysine methyltransferase) 7 (SETD7), mRNA [NM_030648]	-0.7670305	0.0057496065
A_24_P154037	*MINPP1*	Homo sapiens multiple inositol-polyphosphate phosphatase 1 (MINPP1), transcript variant 1, mRNA [NM_004897]	-0.47336513	0.0066500553
A_24_P926960	*ZNF22*	Homo sapiens zinc finger protein 22 (ZNF22), mRNA [NM_006963]	-1.050705	0.010371308
A_21_P0000542	*IRS2*	Homo sapiens insulin receptor substrate 2 (IRS2), mRNA [NM_003749]	-1.3613716	0.0037458793
A_22_P00023864	*MEGF6*	Homo sapiens multiple EGF-like-domains 6 (MEGF6), mRNA [NM_001409]	-2.103662	0.005787274
A_32_P47988	*CMAHP*	Homo sapiens cytidine monophospho-N-acetylneuraminic acid hydroxylase, pseudogene (CMAHP), transcript variant 2, non-coding RNA [NR_027626]	-0.7980677	0.0041847117
A_23_P347528	*LOC101929723*	Homo sapiens uncharacterized LOC101929723 (LOC101929723), transcript variant 1, long non-coding RNA [NR_110262]	-1.5742345	0.011779548
A_24_P50753	*CAMK2D*	Homo sapiens calcium/calmodulin-dependent protein kinase II delta (CAMK2D), transcript variant 3, mRNA [NM_001221]	-0.74036956	0.012680636
A_23_P17204	*TFCP2*	Homo sapiens transcription factor CP2 (TFCP2), transcript variant 1, mRNA [NM_005653]	-1.0327264	0.003417016
A_33_P3374957	*NUDT4*	Homo sapiens nudix (nucleoside diphosphate linked moiety X)-type motif 4 (NUDT4), transcript variant 2, mRNA [NM_199040]	-1.0703032	0.003485545
A_32_P144421	*ANAPC1*	Homo sapiens anaphase promoting complex subunit 1 (ANAPC1), mRNA [NM_022662]	-0.79259944	0.00886592
A_24_P280029	*KLF16*	Homo sapiens Kruppel-like factor 16 (KLF16), mRNA [NM_031918]	0.87515455	0.012476755
A_24_P201153	*ZNF518B*	Homo sapiens zinc finger protein 518B (ZNF518B), mRNA [NM_053042]	-0.6805882	0.011743996
A_33_P3329878	*PDXP*	Homo sapiens pyridoxal (pyridoxine, vitamin B6) phosphatase (PDXP), mRNA [NM_020315]	1.4745406	0.0073988605
A_23_P330999	*TJP2*	Homo sapiens tight junction protein 2 (TJP2), transcript variant 2, mRNA [NM_201629]	1.160726	0.004804688

A_23_P256375	*RNF216*	Homo sapiens ring finger protein 216 (RNF216), transcript variant 1, mRNA [NM_207111]	-0.60916495	0.007877756
A_33_P3344603	*MED23*	Homo sapiens mediator complex subunit 23 (MED23), transcript variant 2, mRNA [NM_015979]	0.39051023	0.014370709
A_24_P391568	*STX4*	Homo sapiens syntaxin 4 (STX4), transcript variant 3, mRNA [NM_004604]	-0.6443072	0.013833903
A_23_P134176	*ZNF280D*	Homo sapiens zinc finger protein 280D (ZNF280D), transcript variant 5, mRNA [NM_001288589]	-0.40477398	0.0155452425
A_33_P3405103	*ARNT*	Homo sapiens aryl hydrocarbon receptor nuclear translocator (ARNT), transcript variant 1, mRNA [NM_001668]	-0.46429282	0.016340334
A_23_P342934	*SOD2*	Homo sapiens superoxide dismutase 2, mitochondrial (SOD2), transcript variant 2, mRNA [NM_001024465]	1.5414107	0.0056093615
A_33_P3276997	*FLYWCH1*	Homo sapiens FLYWCH-type zinc finger 1 (FLYWCH1), transcript variant 2, mRNA [NM_020912]	-0.9723671	0.013467389
A_32_P156892	*TLE3*	Homo sapiens transducin-like enhancer of split 3 (TLE3), transcript variant 1, mRNA [NM_005078]	-0.40128982	0.007681782
A_21_P0000148	*MAP6*	Homo sapiens microtubule-associated protein 6 (MAP6), transcript variant 2, mRNA [NM_207577]	-1.9345843	0.0051130913
A_23_P156852	*ST13*	Homo sapiens suppression of tumorigenicity 13 (colon carcinoma) (Hsp70 interacting protein) (ST13), transcript variant 1, mRNA [NM_003932]	-0.9007094	0.010169998
A_21_P0010740	*NDUFB6*	Homo sapiens NADH dehydrogenase (ubiquinone) 1 beta subcomplex, 6, 17kDa (NDUFB6), transcript variant 1, mRNA [NM_002493]	-0.8104119	0.0095815705
A_23_P160567	*ECI2*	Homo sapiens enoyl-CoA delta isomerase 2 (ECI2), transcript variant 2, mRNA [NM_206836]	-0.5493141	0.012873199
A_23_P140035	*ZMYND12*	Homo sapiens zinc finger, MYND-type containing 12 (ZMYND12), transcript variant 1, mRNA [NM_032257]	-1.5782129	0.01581842
A_23_P94141	*RIOK3*	Homo sapiens RIO kinase 3 (RIOK3), mRNA [NM_003831]	-1.3696196	0.0024890031
A_24_P248240	*WBP4*	Homo sapiens WW domain binding protein 4 (WBP4), mRNA [NM_007187]	-0.9232735	0.0031941293
A_23_P125042	*RAD54B*	Homo sapiens RAD54 homolog B (S. cerevisiae) (RAD54B), transcript variant 2, mRNA [NM_001205262]	0.34109786	0.0102413045

A_32_P230828	*SYT11*	Homo sapiens synaptotagmin XI (SYT11), mRNA [NM_152280]	-0.8472346	0.0062631676
A_22_P00004590	*ZNF222*	Homo sapiens zinc finger protein 222 (ZNF222), transcript variant 2, mRNA [NM_013360]	-0.49450675	0.0055521973
A_33_P3251054	*GAS5*	Homo sapiens growth arrest-specific 5 (non-protein coding) (GAS5), long non-coding RNA [NR_002578]	-0.79060316	0.0062618116
A_23_P149892	*lnc-CS-1*	LNCipedia lincRNA (lnc-CS-1), lincRNA [lnc-CS-1:1]	1.0765122	0.004884975
A_23_P80817	*RNF38*	Homo sapiens ring finger protein 38 (RNF38), transcript variant 2, mRNA [NM_194328]	-0.8328473	0.0070313537
A_23_P77459	*CSGALNACT2*	Homo sapiens chondroitin sulfate N-acetylgalactosaminyltransferase 2 (CSGALNACT2), mRNA [NM_018590]	-0.75540847	0.0028149292
A_24_P80532	*TAGLN3*	Homo sapiens transgelin 3 (TAGLN3), transcript variant 1, mRNA [NM_013259]	3.337998	0.0027348737
A_33_P3297444	*NAE1*	Homo sapiens NEDD8 activating enzyme E1 subunit 1 (NAE1), transcript variant 2, mRNA [NM_001018159]	-0.35369107	0.007871983
A_23_P390116	*CCNG2*	Homo sapiens cyclin G2 (CCNG2), mRNA [NM_004354]	-1.0355871	0.00975317
A_22_P00005733	*ABTB1*	Homo sapiens ankyrin repeat and BTB (POZ) domain containing 1 (ABTB1), transcript variant 1, mRNA [NM_032548]	-0.57902056	0.012240485
A_23_P48175	*SPATA13*	Homo sapiens spermatogenesis associated 13 (SPATA13), transcript variant 2, mRNA [NM_153023]	1.2318667	0.015448156
A_21_P0003259	*lnc-EMILIN2-1*	cl79c10.z1 Hembase; Erythroid Precursor Cells (LCB:cl library) Homo sapiens cDNA clone cl79c10 5', mRNA sequence [BU661971]	-1.3552594	0.004404579
A_23_P408094	*TMEM106C*	Homo sapiens transmembrane protein 106C (TMEM106C), transcript variant 2, mRNA [NM_024056]	-0.6971488	0.004454847
A_23_P214897	*lnc-SERPINI1-9*	LNCipedia lincRNA (lnc-SERPINI1-9), lincRNA [lnc-SERPINI1-9:6]	-0.98066056	0.007740612
A_33_P3313785	*MXD1*	Homo sapiens MAX dimerization protein 1 (MXD1), transcript variant 1, mRNA [NM_002357]	-0.66160417	0.009241496
A_22_P00015791	*AKAP12*	Homo sapiens A kinase (PRKA) anchor protein 12 (AKAP12), transcript variant 2, mRNA [NM_144497]	-2.0191038	0.0035935708
A_33_P3402056	*CCDC14*	Homo sapiens coiled-coil domain containing 14 (CCDC14), mRNA [NM_022757]	-0.9042079	0.009627439
A_23_P348159	*lnc-TARBP2-1*	zf31f06.s1 Soares_fetal_heart_NbHH19W Homo sapiens cDNA clone IMAGE:378563 3' similar to contains Alu repetitive	0.4925609	0.0171262

		element;contains element PTR7 repetitive element ;, mRNA sequence [AA775737]		
A_33_P3307197	*LOC389831*	Homo sapiens uncharacterized LOC389831 (LOC389831), mRNA [NM_001242480]	-1.215027	0.005415712
A_33_P7476087	*DST*	dystonin [Source:HGNC Symbol;Acc:HGNC:1090] [ENST00000439203]	-0.6013941	0.014349793
A_21_P0000629	*PTGFRN*	Homo sapiens prostaglandin F2 receptor inhibitor (PTGFRN), mRNA [NM_020440]	-3.965034	0.0023340243
A_23_P38732	*THUMPD3-AS1*	Homo sapiens THUMPD3 antisense RNA 1 (THUMPD3-AS1), long non-coding RNA [NR_027007]	-0.36898822	0.014192907
A_23_P162918	*SARS*	Homo sapiens seryl-tRNA synthetase (SARS), transcript variant 2, non-coding RNA [NR_034072]	-0.93638086	0.012580898
A_24_P390070	*CDH2*	Homo sapiens cadherin 2, type 1, N-cadherin (neuronal) (CDH2), mRNA [NM_001792]	-2.1134055	0.003145806
A_23_P92230	*SERPINA3*	Homo sapiens serpin peptidase inhibitor, clade A (alpha-1 antiproteinase, antitrypsin), member 3 (SERPINA3), mRNA [NM_001085]	1.8854531	0.0046008946
A_23_P146050	*SRSF9*	Homo sapiens serine/arginine-rich splicing factor 9 (SRSF9), mRNA [NM_003769]	-0.22898716	0.0063147643
A_22_P00001321	*TSC22D2*	Homo sapiens TSC22 domain family, member 2 (TSC22D2), transcript variant 1, mRNA [NM_014779]	-1.1100471	0.0037458793
A_23_P309865	*ZFAND1*	Homo sapiens zinc finger, AN1-type domain 1 (ZFAND1), transcript variant 1, mRNA [NM_024699]	-0.7507836	0.0044240346
A_33_P3280721	*lnc-ANTXRL-2*	Homo sapiens cDNA clone IMAGE:4825811. [BC031882]	0.9810769	0.014728144
A_33_P3382399	*ZNF449*	Homo sapiens zinc finger protein 449 (ZNF449), mRNA [NM_152695]	-1.7852553	0.007920593
A_24_P203056	*WLS*	Homo sapiens wntless Wnt ligand secretion mediator (WLS), transcript variant 2, mRNA [NM_001002292]	1.9961534	0.0048641856
A_33_P3403082	*KRTAP3-1*	Homo sapiens keratin associated protein 3-1 (KRTAP3-1), mRNA [NM_031958]	0.8658211	0.016391506
A_32_P154830	*BCL7A*	Homo sapiens B-cell CLL/lymphoma 7A (BCL7A), transcript variant 1, mRNA [NM_020993]	-0.9316871	0.005612091
A_23_P214080	*LINC00176*	Homo sapiens long intergenic non-protein coding RNA 176 (LINC00176), transcript variant 1, long non-coding RNA [NR_027686]	0.6424438	0.012575773
A_33_P3262191	*OSTM1*	Homo sapiens osteopetrosis associated transmembrane protein 1 (OSTM1), mRNA [NM_014028]	-0.9275432	0.009811661
A_23_P90845	*EGR1*	Homo sapiens early growth response 1 (EGR1), mRNA [NM_001964]	0.14259245	0.008755959

A_24_P917866	*CPNE7*	Homo sapiens copine VII (CPNE7), transcript variant 2, mRNA [NM_014427]	1.3007247	0.004160909
A_33_P3329477	*TIA1*	Homo sapiens TIA1 cytotoxic granule-associated RNA binding protein (TIA1), transcript variant 2, mRNA [NM_022173]	-0.6029549	0.014260212
A_23_P145397	*SET*	Homo sapiens SET nuclear proto-oncogene (SET), transcript variant 2, mRNA [NM_003011]	0.7845742	0.014613322
A_23_P430181	*RPL13P5*	Homo sapiens ribosomal protein L13 pseudogene 5 (RPL13P5), non-coding RNA [NR_002803]	-0.83270615	0.009830751
A_24_P298877	*CCNC*	Homo sapiens cyclin C (CCNC), transcript variant 1, mRNA [NM_005190]	-0.5051456	0.0046609533
A_21_P0014236	*ZBTB3*	Homo sapiens zinc finger and BTB domain containing 3 (ZBTB3), mRNA [NM_024784]	-1.4003017	0.006773419
A_24_P93452	*C1orf174*	Homo sapiens chromosome 1 open reading frame 174 (C1orf174), mRNA [NM_207356]	-0.5591472	0.009460012
A_23_P76159	*C9orf3*	chromosome 9 open reading frame 3 [Source:HGNC Symbol;Acc:HGNC:1361] [ENST00000482056]	-0.78072375	0.008090729
A_21_P0014732	*EEA1*	Homo sapiens early endosome antigen 1 (EEA1), mRNA [NM_003566]	-0.4888539	0.009784609
A_33_P3365142	*ZNF414*	Homo sapiens zinc finger protein 414 (ZNF414), transcript variant 2, mRNA [NM_032370]	1.5449518	0.003606253
A_24_P187351	*GAD1*	Homo sapiens glutamate decarboxylase 1 (brain, 67kDa) (GAD1), transcript variant GAD25, mRNA [NM_013445]	-1.6781061	0.014982433
A_33_P3424297	*ARHGAP17*	Homo sapiens Rho GTPase activating protein 17 (ARHGAP17), transcript variant 1, mRNA [NM_001006634]	-1.068774	0.007683969
A_32_P69368	*CNOT4*	Homo sapiens CCR4-NOT transcription complex, subunit 4 (CNOT4), transcript variant 1, mRNA [NM_013316]	1.1804215	0.011307727
A_23_P426140	*SELT*	Homo sapiens selenoprotein T (SELT), mRNA [NM_016275]	-0.46802038	0.005057235
A_33_P3262555	*ID2*	Homo sapiens inhibitor of DNA binding 2, dominant negative helix-loop-helix protein (ID2), mRNA [NM_002166]	-0.56175965	0.015403497
A_23_P388900	*ZYG11B*	Homo sapiens zyg-11 family member B, cell cycle regulator (ZYG11B), mRNA [NM_024646]	-0.8174245	0.0024890031
A_22_P00025708	*MEX3D*	Homo sapiens mex-3 RNA binding family member D (MEX3D), transcript variant 2, mRNA [NM_001174118]	1.0818053	0.005428736
A_33_P3423425	*SLC22A15*	Homo sapiens solute carrier family 22, member 15 (SLC22A15), mRNA [NM_018420]	-1.5439699	0.004577453
A_33_P3361393	*ZNF770*	Homo sapiens zinc finger protein 770 (ZNF770), mRNA [NM_014106]	-2.2321393	0.0049677542

A_21_P0000691	*SCARNA17*	Homo sapiens small Cajal body-specific RNA 17 (SCARNA17), guide RNA [NR_003003]	-0.77083445	0.008868601
A_22_P00017379	*EIF1AX*	Homo sapiens eukaryotic translation initiation factor 1A, X-linked (EIF1AX), mRNA [NM_001412]	-0.42285782	0.009649863
A_24_P13230	*LOC115110*	Homo sapiens uncharacterized LOC115110 (LOC115110), long non-coding RNA [NR_037844]	-1.0187752	0.013669192
A_23_P85952	*lnc-UVRAG-2*	DA940890 SPLEN2 Homo sapiens cDNA clone SPLEN2014945 5', mRNA sequence [DA940890]	0.38169688	0.016568046
A_33_P3393796	*RAB6A*	Homo sapiens RAB6A, member RAS oncogene family (RAB6A), transcript variant 1, mRNA [NM_002869]	-0.6066589	0.01263861
A_23_P134147	*DENND2D*	Homo sapiens DENN/MADD domain containing 2D (DENND2D), transcript variant 1, mRNA [NM_024901]	-1.4397299	0.005514184
A_23_P259333	*TRMT5*	Homo sapiens tRNA methyltransferase 5 (TRMT5), mRNA [NM_020810]	-0.5465064	0.0042201984
A_23_P408095	*ZBTB24*	Homo sapiens zinc finger and BTB domain containing 24 (ZBTB24), transcript variant 1, mRNA [NM_014797]	-0.72506684	0.009534265
A_33_P3250133	*C6orf203*	Homo sapiens chromosome 6 open reading frame 203 (C6orf203), transcript variant 1, mRNA [NM_016487]	-0.9166834	0.0040635793
A_21_P0004817	*DSTN*	Homo sapiens destrin (actin depolymerizing factor) (DSTN), transcript variant 2, mRNA [NM_001011546]	-0.84776014	0.00422039
A_33_P3261818	*VSIG10*	Homo sapiens V-set and immunoglobulin domain containing 10 (VSIG10), mRNA [NM_019086]	-0.52505904	0.011949872
A_33_P3392245	*lnc-WRNIP1-2*	LNCipedia lincRNA (lnc-WRNIP1-2), lincRNA [lnc-WRNIP1-2:9]	-0.63127434	0.004327546
A_23_P372988	*MDH1*	Homo sapiens malate dehydrogenase 1, NAD (soluble) (MDH1), transcript variant 2, mRNA [NM_005917]	-0.5322972	0.0061524836
A_23_P109235	*FAM178A*	Homo sapiens family with sequence similarity 178, member A (FAM178A), transcript variant 2, mRNA [NM_001136123]	-0.66184807	0.004155979
A_33_P3229241	*PRR22*	Homo sapiens proline rich 22 (PRR22), mRNA [NM_001134316]	1.3678186	0.010942945
A_23_P202737	*RALY*	Homo sapiens RALY heterogeneous nuclear ribonucleoprotein (RALY), transcript variant 1, mRNA [NM_016732]	0.7779227	0.011768655
A_22_P00021553	*HIST2H2BF*	histone cluster 2, H2bf [Source:HGNC Symbol;Acc:HGNC:24700] [ENST00000369167]	-1.0741837	0.0046958732
A_23_P41143	*JRKL*	Homo sapiens JRK-like (JRKL), transcript variant 1, mRNA [NM_003772]	-0.9142841	0.006080751

A_22_P00023384	*PPP4R1-AS1*	Homo sapiens PPP4R1 antisense RNA 1 (PPP4R1-AS1), transcript variant 1, long non-coding RNA [NR_110773]	-1.357299	0.005612091
A_33_P3242014	*APEH*	Homo sapiens acylaminoacyl-peptide hydrolase (APEH), mRNA [NM_001640]	0.84405017	0.009859882
A_23_P143935	*PHC1*	Homo sapiens polyhomeotic homolog 1 (Drosophila) (PHC1), mRNA [NM_004426]	-0.74369	0.0073615913
A_23_P89921	*C1orf27*	Homo sapiens chromosome 1 open reading frame 27 (C1orf27), transcript variant 1, mRNA [NM_017847]	-0.60546494	0.007196166
A_23_P334870	*PIGZ*	Homo sapiens phosphatidylinositol glycan anchor biosynthesis, class Z (PIGZ), mRNA [NM_025163]	-0.8420613	0.0098649
A_33_P3421515	*ZNF180*	Homo sapiens zinc finger protein 180 (ZNF180), transcript variant 1, mRNA [NM_013256]	-0.952198	0.008220364
A_33_P3715909	*TMEM217*	Homo sapiens transmembrane protein 217 (TMEM217), transcript variant 1, mRNA [NM_145316]	-1.8566769	0.00549504
A_24_P106794	*CDK16*	Homo sapiens cyclin-dependent kinase 16 (CDK16), transcript variant 1, mRNA [NM_006201]	-0.59522104	0.0038456172
A_33_P3217704	*SNAI1*	Homo sapiens snail family zinc finger 1 (SNAI1), mRNA [NM_005985]	1.7742274	0.0037353234
A_22_P00010310	*NUDCD1*	Homo sapiens NudC domain containing 1 (NUDCD1), transcript variant 1, mRNA [NM_032869]	-1.2099017	0.0056601833
A_23_P206018	*FAM214B*	Homo sapiens family with sequence similarity 214, member B (FAM214B), mRNA [NM_025182]	-0.65736073	0.00344329
A_33_P3379381	*LOC101927503*	PREDICTED: Homo sapiens uncharacterized LOC101927503 (LOC101927503), transcript variant X2, misc_RNA [XR_428869]	0.6569459	0.0038773962
A_33_P3387170	*TPM1*	Homo sapiens tropomyosin 1 (alpha) (TPM1), transcript variant Tpm1.6, mRNA [NM_001018004]	-1.2754428	0.003969615
A_23_P57588	*WDR78*	Homo sapiens WD repeat domain 78 (WDR78), transcript variant 2, mRNA [NM_207014]	-0.4668469	0.0037011201
A_21_P0012868	*MATN1-AS1*	Homo sapiens MATN1 antisense RNA 1 (MATN1-AS1), long non-coding RNA [NR_034182]	1.062707	0.0059948512
A_32_P230825	*GTSE1*	Homo sapiens G-2 and S-phase expressed 1 (GTSE1), mRNA [NM_016426]	0.5149815	0.015397574
A_33_P3306113	*LOC100128340*	PREDICTED: Homo sapiens uncharacterized LOC100128340 (RP11-423H2.3), misc_RNA [XR_427818]	-1.049407	0.008242901

A_23_P118571	*JMJD4*	Homo sapiens jumonji domain containing 4 (JMJD4), transcript variant 1, mRNA [NM_023007]	-0.8339648	0.013513609
A_33_P3349827	*SOST*	Homo sapiens sclerostin (SOST), mRNA [NM_025237]	-1.79582	0.004155979
A_24_P261929	*MLF1*	Homo sapiens myeloid leukemia factor 1 (MLF1), transcript variant 4, mRNA [NM_001195432]	-1.5760112	0.009378251
A_33_P3377060	*RBM3*	Homo sapiens RNA binding motif (RNP1, RRM) protein 3 (RBM3), mRNA [NM_006743]	-0.8436563	0.005348777
A_21_P0000651	*IFI27L1*	Homo sapiens interferon, alpha-inducible protein 27-like 1 (IFI27L1), transcript variant 2, mRNA [NM_206949]	-0.6584704	0.0072350414
A_22_P00003334	*SSR1*	Homo sapiens signal sequence receptor, alpha (SSR1), transcript variant 1, mRNA [NM_003144]	-0.31247666	0.016171217
A_33_P3293446	*HERC2P7*	Homo sapiens hect domain and RLD 2 pseudogene 7 (HERC2P7), non-coding RNA [NR_036470]	-1.5190554	0.0075218254
A_33_P3296074	*lnc-CAST-4*	LNCipedia lincRNA (lnc-CAST-4), lincRNA [lnc-CAST-4:1]	-1.4339492	0.010833076
A_23_P253068	*KIAA1462*	Homo sapiens KIAA1462 (KIAA1462), mRNA [NM_020848]	-1.0709753	0.006155321
A_33_P3378051	*ZNF131*	Homo sapiens zinc finger protein 131 (ZNF131), transcript variant 1, mRNA [NM_001297548]	-0.71862596	0.0065241847
A_24_P333421	*ANAPC2*	Homo sapiens anaphase promoting complex subunit 2 (ANAPC2), mRNA [NM_013366]	1.3934593	0.0031941293
A_23_P355824	*SESTD1*	Homo sapiens SEC14 and spectrin domains 1 (SESTD1), mRNA [NM_178123]	-0.85766804	0.0041997624
A_23_P167040	*ZNF862*	Homo sapiens zinc finger protein 862 (ZNF862), mRNA [NM_001099220]	-0.9795792	0.0073443917
A_33_P3289860	*MGRN1*	Homo sapiens mahogunin ring finger 1, E3 ubiquitin protein ligase (MGRN1), transcript variant 1, mRNA [NM_015246]	-0.7923793	0.007920593
A_22_P00002059	*PDIA5*	Homo sapiens protein disulfide isomerase family A, member 5 (PDIA5), transcript variant 1, mRNA [NM_006810]	-1.7789376	0.005641728
A_23_P420196	*MIR100HG*	Homo sapiens mir-100-let-7a-2 cluster host gene (non-protein coding) (MIR100HG), long non-coding RNA [NR_024430]	-0.9007074	0.010442942
A_33_P7278111	*C6orf89*	Homo sapiens chromosome 6 open reading frame 89 (C6orf89), transcript variant 1, mRNA [NM_152734]	-0.8865471	0.0051266714
A_21_P0011252	*SOCS1*	Homo sapiens suppressor of cytokine signaling 1 (SOCS1), mRNA [NM_003745]	1.4689603	0.0067319144

A_23_P121282	*LAPTM4B*	Homo sapiens lysosomal protein transmembrane 4 beta (LAPTM4B), mRNA [NM_018407]	-0.59939665	0.011747599
A_33_P3274105	*TMEM89*	Homo sapiens transmembrane protein 89 (TMEM89), mRNA [NM_001008269]	1.3321937	0.008153157
A_24_P128683	*RDH11*	Homo sapiens retinol dehydrogenase 11 (all-trans/9-cis/11-cis) (RDH11), transcript variant 1, mRNA [NM_016026]	-0.52056617	0.0126051465
A_33_P3703501	*CLHC1*	clathrin heavy chain linker domain containing 1 [Source:HGNC Symbol;Acc:HGNC:26453] [ENST00000403506]	0.5924542	0.01214474
A_23_P28878	*CNPPD1*	Homo sapiens cyclin Pas1/PHO80 domain containing 1 (CNPPD1), mRNA [NM_015680]	0.52347475	0.013991326
A_23_P401774	*GALNT10*	Homo sapiens polypeptide N-acetylgalactosaminyltransferase 10 (GALNT10), mRNA [NM_198321]	-1.2012584	0.007905212
A_33_P3377380	*C20orf27*	Homo sapiens chromosome 20 open reading frame 27 (C20orf27), transcript variant 1, mRNA [NM_001039140]	1.1862087	0.0068865013
A_22_P00018124	*ELMOD1*	Homo sapiens ELMO/CED-12 domain containing 1 (ELMOD1), transcript variant 1, mRNA [NM_018712]	3.1162615	0.008159287
A_33_P3213362	*lnc-PABPC4-2*	Q5TGA2_HUMAN (Q5TGA2) Peptidylprolyl isomerase E (Cyclophilin E), partial (29%) [THC2586785]	0.76217985	0.0069110035
A_23_P128554	*ZNF32-AS1*	Homo sapiens ZNF32 antisense RNA 1 (ZNF32-AS1), long non-coding RNA [NR_047557]	-0.8156364	0.0057351408
A_23_P143650	*CASC2*	Homo sapiens cancer susceptibility candidate 2 (non-protein coding) (CASC2), transcript variant 1, long non-coding RNA [NR_026939]	-1.5587549	0.00910823
A_32_P42574	*EBPL*	Homo sapiens emopamil binding protein-like (EBPL), transcript variant 1, mRNA [NM_032565]	-0.5029206	0.013483029
A_23_P47517	*DUSP18*	Homo sapiens dual specificity phosphatase 18 (DUSP18), mRNA [NM_152511]	-1.0032097	0.016336935
A_33_P3406836	*C1orf198*	Homo sapiens chromosome 1 open reading frame 198 (C1orf198), transcript variant 1, mRNA [NM_032800]	-1.6814343	0.005057235
A_24_P791829	*VPS37C*	Homo sapiens vacuolar protein sorting 37 homolog C (S. cerevisiae) (VPS37C), mRNA [NM_017966]	-0.8157003	0.0047776042
A_23_P152768	*HTR1F*	Homo sapiens 5-hydroxytryptamine (serotonin) receptor 1F, G protein-coupled (HTR1F), mRNA [NM_000866]	1.1879029	0.0031941293
A_21_P0000382	*NUFIP2*	Homo sapiens nuclear fragile X mental retardation protein interacting protein 2 (NUFIP2), mRNA [NM_020772]	-0.7912557	0.007986457

A_23_P203920	*TUBG1*	Homo sapiens tubulin, gamma 1 (TUBG1), mRNA [NM_001070]	0.9759176	0.010827728
A_33_P3217465	*SNORD71*	Homo sapiens small nucleolar RNA, C/D box 71 (SNORD71), small nucleolar RNA [NR_003059]	-0.9111624	0.0132579645
A_22_P00003340	*SSPN*	Homo sapiens sarcospan (SSPN), transcript variant 1, mRNA [NM_005086]	-2.0584579	0.0032831472
A_33_P3272527	*MID1IP1*	Homo sapiens MID1 interacting protein 1 (MID1IP1), transcript variant 1, mRNA [NM_021242]	-1.3546965	0.0067283837
A_23_P130836	*lnc-CATSPERB-1*	Homo sapiens cDNA FLJ11511 fis, clone HEMBA1002189. [AK021573]	1.0757694	0.01284574
A_23_P135995	*MAVS*	Homo sapiens mitochondrial antiviral signaling protein (MAVS), transcript variant 3, mRNA [NM_001206491]	0.6378205	0.007920593
A_23_P368154	*GZMM*	Homo sapiens granzyme M (lymphocyte met-ase 1) (GZMM), transcript variant 1, mRNA [NM_005317]	0.4875004	0.0059536803
A_23_P200222	*ARV1*	Homo sapiens ARV1 homolog (S. cerevisiae) (ARV1), mRNA [NM_022786]	-0.59539884	0.010769289
A_33_P3267375	*PODN*	Homo sapiens podocan (PODN), transcript variant 1, mRNA [NM_153703]	1.5319573	0.0029871191
A_23_P363406	*LRP8*	Homo sapiens low density lipoprotein receptor-related protein 8, apolipoprotein e receptor (LRP8), transcript variant 2, mRNA [NM_033300]	0.50678444	0.012795951
A_33_P3284129	*SGCD*	Homo sapiens sarcoglycan, delta (35kDa dystrophin-associated glycoprotein) (SGCD), transcript variant 1, mRNA [NM_000337]	-2.4996529	0.003190129
A_24_P152404	*ANGEL1*	Homo sapiens angel homolog 1 (Drosophila) (ANGEL1), mRNA [NM_015305]	-1.0304796	0.016238803
A_24_P880043	*LYPD1*	Homo sapiens LY6/PLAUR domain containing 1 (LYPD1), transcript variant 1, mRNA [NM_144586]	-1.301658	0.00626446
A_33_P3611762	*C10orf76*	chromosome 10 open reading frame 76 [Source:HGNC Symbol;Acc:HGNC:25788] [ENST00000311122]	-0.5106956	0.016442578
A_33_P3254666	*PCGF5*	Homo sapiens polycomb group ring finger 5 (PCGF5), transcript variant 1, mRNA [NM_032373]	-1.0922903	0.0053046974
A_33_P3405022	*TRAM2-AS1*	Homo sapiens TRAM2 antisense RNA 1 (head to head) (TRAM2-AS1), transcript variant 1, long non-coding RNA [NR_103446]	-0.86669326	0.009259001
A_24_P67806	*LZTS2*	Homo sapiens cDNA FLJ40678 fis, clone THYMU2022660. [AK097997]	2.5713954	0.008472113
A_33_P3234593	*LETM1*	Homo sapiens leucine zipper-EF-hand containing transmembrane protein 1 (LETM1), mRNA [NM_012318]	3.173757	0.005400047

A_32_P437004	*MTR*	Homo sapiens 5-methyltetrahydrofolate-homocysteine methyltransferase (MTR), transcript variant 1, mRNA [NM_000254]	-0.77161235	0.011489915
A_23_P412409	*BRD7*	bromodomain containing 7 [Source:HGNC Symbol;Acc:HGNC:14310] [ENST00000401491]	0.95010936	0.005014313
A_23_P157404	*XG*	Homo sapiens Xg blood group (XG), transcript variant 1, mRNA [NM_175569]	0.6490817	0.004508921
A_23_P152125	*PRRC2C*	Homo sapiens proline-rich coiled-coil 2C (PRRC2C), mRNA [NM_015172]	-0.23118469	0.012773474
A_22_P00004148	*AP1S1*	Homo sapiens adaptor-related protein complex 1, sigma 1 subunit (AP1S1), mRNA [NM_001283]	-0.8341879	0.009794768
A_23_P75283	*MVD*	Homo sapiens mevalonate (diphospho) decarboxylase (MVD), mRNA [NM_002461]	0.8907888	0.009924993
A_23_P207058	*lnc-CLEC18B-2*	LNCipedia lincRNA (lnc-CLEC18B-2), lincRNA [lnc-CLEC18B-2:2]	-0.57008094	0.016435314
A_33_P6518002	*RBP4*	Homo sapiens retinol binding protein 4, plasma (RBP4), mRNA [NM_006744]	1.4806517	0.009731291
A_33_P3294237	*SOCS3*	Homo sapiens suppressor of cytokine signaling 3 (SOCS3), mRNA [NM_003955]	1.445468	0.003121324
A_33_P3377239	*CREB1*	Homo sapiens cAMP responsive element binding protein 1 (CREB1), transcript variant B, mRNA [NM_134442]	-0.8791041	0.002419416
A_22_P00015401	*MTF1*	Homo sapiens metal-regulatory transcription factor 1 (MTF1), mRNA [NM_005955]	-1.0207353	0.00410343
A_24_P627306	*LOC100270804*	Homo sapiens uncharacterized LOC100270804 (LOC100270804), long non-coding RNA [NR_026885]	-1.0359141	0.016537301
A_23_P131227	*KMT2E-AS1*	Homo sapiens KMT2E antisense RNA 1 (head to head) (KMT2E-AS1), long non-coding RNA [NR_024586]	0.73325264	0.00860804
A_33_P3307820	*EDEM2*	Homo sapiens ER degradation enhancer, mannosidase alpha-like 2 (EDEM2), transcript variant 1, mRNA [NM_018217]	-0.61671585	0.015061039
A_32_P235796	*TTC27*	Homo sapiens tetratricopeptide repeat domain 27 (TTC27), transcript variant 1, mRNA [NM_017735]	-0.58074427	0.011794303
A_33_P3295358	*ZC2HC1B*	Homo sapiens zinc finger, C2HC-type containing 1B (ZC2HC1B), mRNA [NM_001013623]	-1.0373666	0.013618745
A_23_P6651	*BBS12*	Homo sapiens Bardet-Biedl syndrome 12 (BBS12), transcript variant 2, mRNA [NM_152618]	-1.7060807	0.0057666874
A_24_P269814	*ANGPTL4*	Homo sapiens angiopoietin-like 4 (ANGPTL4), transcript variant 1, mRNA [NM_139314]	1.2483636	0.0071605598
A_21_P0000694	*FAM208A*	Homo sapiens family with sequence similarity 208, member A (FAM208A), transcript variant 2, mRNA [NM_015224]	-0.86646515	0.010237655

A_24_P58337	*PLEKHA1*	Homo sapiens pleckstrin homology domain containing, family A (phosphoinositide binding specific) member 1 (PLEKHA1), transcript variant 2, mRNA [NM_001001974]	-1.3898467	0.005255513
A_21_P0007586	*NIFK-AS1*	Homo sapiens NIFK antisense RNA 1 (NIFK-AS1), transcript variant 2, long non-coding RNA [NR_037858]	-0.69381636	0.010735307
A_21_P0011231	*FTH1*	Homo sapiens ferritin, heavy polypeptide 1 (FTH1), mRNA [NM_002032]	1.0129988	0.011680649
A_23_P71433	*LINC00448*	Homo sapiens long intergenic non-protein coding RNA 448 (LINC00448), transcript variant 1, long non-coding RNA [NR_047029]	0.697185	0.012554233
A_23_P201368	*UBE2W*	Homo sapiens ubiquitin-conjugating enzyme E2W (putative) (UBE2W), transcript variant 1, mRNA [NM_001001481]	-0.76784325	0.005512448
A_33_P3223749	*RNF167*	Homo sapiens ring finger protein 167 (RNF167), mRNA [NM_015528]	1.1373708	0.0031512622
A_33_P3295283	*CTBS*	Homo sapiens chitobiase, di-N-acetyl- (CTBS), mRNA [NM_004388]	-0.9113223	0.00886592
A_23_P211445	*MED14*	Homo sapiens mediator complex subunit 14 (MED14), mRNA [NM_004229]	-0.95889425	0.0053998437
A_32_P201773	*LPCAT3*	Homo sapiens lysophosphatidylcholine acyltransferase 3 (LPCAT3), mRNA [NM_005768]	-0.65744233	0.016876886
A_33_P3237225	*LIMK2*	Homo sapiens LIM domain kinase 2 (LIMK2), transcript variant 2b, mRNA [NM_016733]	-0.57803035	0.016324468
A_23_P25253	*AMMECR1*	Homo sapiens Alport syndrome, mental retardation, midface hypoplasia and elliptocytosis chromosomal region gene 1 (AMMECR1), transcript variant 1, mRNA [NM_015365]	-0.57350355	0.014035798
A_21_P0014336	*FBXO34*	Homo sapiens F-box protein 34 (FBXO34), transcript variant 2, mRNA [NM_152231]	-1.1429745	0.005014313
A_23_P131646	*DERA*	Homo sapiens deoxyribose-phosphate aldolase (putative) (DERA), transcript variant 1, mRNA [NM_015954]	-0.87941027	0.00556786
A_33_P3390708	*lnc-CA5A-4*	Homo sapiens cDNA FLJ41560 fis, clone CTONG1000088. [AK123554]	1.6558213	0.0081354305
A_23_P1782	*RPIA*	Homo sapiens ribose 5-phosphate isomerase A (RPIA), mRNA [NM_144563]	-0.4848964	0.009765222
A_23_P134809	*TMEM2*	Homo sapiens transmembrane protein 2 (TMEM2), transcript variant 1, mRNA [NM_013390]	1.3004417	0.007771992
A_23_P126135	*CD82*	Homo sapiens CD82 molecule (CD82), transcript variant 1, mRNA [NM_002231]	2.4275553	0.0037583082
A_33_P3211956	*NSMAF*	Homo sapiens neutral sphingomyelinase (N-SMase) activation associated factor (NSMAF), transcript variant 1, mRNA [NM_003580]	-0.62060666	0.016810067

A_33_P3268408	*MFN2*	Homo sapiens mitofusin 2 (MFN2), transcript variant 1, mRNA [NM_014874]	-0.71629906	0.0076496457
A_33_P3351934	*RGS19*	Homo sapiens regulator of G-protein signaling 19 (RGS19), transcript variant 1, mRNA [NM_005873]	-0.58865356	0.014086517
A_33_P3833256	*MSTO1*	Homo sapiens misato homolog 1 (Drosophila), mRNA (cDNA clone IMAGE:4828998). [BC070067]	-0.8308961	0.014514313
A_23_P212706	*MAD1L1*	Homo sapiens MAD1 mitotic arrest deficient-like 1 (yeast) (MAD1L1), transcript variant 1, mRNA [NM_003550]	1.2377939	0.008367874
A_33_P3342430	*LOC440028*	Homo sapiens uncharacterized LOC440028 (LOC440028), long non-coding RNA [NR_033972]	-0.41295058	0.0031941293
A_33_P3304983	*ATG3*	Homo sapiens autophagy related 3 (ATG3), transcript variant 1, mRNA [NM_022488]	-0.5605902	0.0108927535
A_24_P336705	*PRKAR2B*	Homo sapiens protein kinase, cAMP-dependent, regulatory, type II, beta (PRKAR2B), mRNA [NM_002736]	0.9927342	0.0085187815
A_24_P405205	*RABGGTB*	Homo sapiens Rab geranylgeranyltransferase, beta subunit (RABGGTB), transcript variant 1, mRNA [NM_004582]	-0.9853983	0.008627961
A_24_P22746	*CEP41*	Homo sapiens centrosomal protein 41kDa (CEP41), transcript variant 1, mRNA [NM_018718]	-0.80742264	0.011285475
A_33_P3276308	*ATP2B4*	Homo sapiens ATPase, Ca++ transporting, plasma membrane 4 (ATP2B4), transcript variant 1, mRNA [NM_001001396]	-1.2026386	0.0025001846
A_23_P21092	*ZADH2*	Homo sapiens zinc binding alcohol dehydrogenase domain containing 2 (ZADH2), mRNA [NM_175907]	-0.7962529	0.004804688
A_23_P171249	*FLJ44124*	PREDICTED: Homo sapiens uncharacterized LOC641737 (FLJ44124), misc_RNA [XR_252152]	0.86960983	0.0069250474
A_33_P3269588	*CALB2*	Homo sapiens calbindin 2 (CALB2), transcript variant CALB2, mRNA [NM_001740]	-1.8573161	0.015330245
A_23_P133474	*IGBP1*	Homo sapiens immunoglobulin (CD79A) binding protein 1 (IGBP1), mRNA [NM_001551]	-0.6336834	0.006262615
A_21_P0000410	*GABPA*	Homo sapiens GA binding protein transcription factor, alpha subunit 60kDa (GABPA), transcript variant 1, mRNA [NM_002040]	-1.048619	0.0029691157
A_21_P0000885	*GPX3*	Homo sapiens glutathione peroxidase 3 (plasma) (GPX3), mRNA [NM_002084]	2.1122713	0.0039497027
A_33_P3304655	*SNORD114-13*	Homo sapiens small nucleolar RNA, C/D box 114-13 (SNORD114-13), small nucleolar RNA [NR_003206]	0.82946104	0.0065723537

A_23_P109254	*ZNF790-AS1*	Homo sapiens ZNF790 antisense RNA 1 (ZNF790-AS1), transcript variant 1, long non-coding RNA [NR_040027]	-0.88758373	0.0059821224
A_23_P386320	*LTBP1*	Homo sapiens latent transforming growth factor beta binding protein 1 (LTBP1), transcript variant 1, mRNA [NM_206943]	0.8630233	0.0055054883
A_32_P88635	*RBM39*	Homo sapiens RNA binding motif protein 39 (RBM39), transcript variant 1, mRNA [NM_184234]	-0.8533304	0.014930988
A_33_P3399019	*MFI2*	Homo sapiens antigen p97 (melanoma associated) identified by monoclonal antibodies 133.2 and 96.5 (MFI2), transcript variant 2, mRNA [NM_033316]	-1.2384499	0.01456425
A_24_P312189	*NBPF3*	Homo sapiens neuroblastoma breakpoint family, member 3 (NBPF3), transcript variant 1, mRNA [NM_032264]	-0.6889079	0.0070571043
A_33_P3412900	*ZNF816*	Homo sapiens zinc finger protein 816 (ZNF816), transcript variant 1, mRNA [NM_001031665]	-1.2280478	0.006573993
A_22_P00010751	*MEPCE*	Homo sapiens methylphosphate capping enzyme (MEPCE), transcript variant 1, mRNA [NM_019606]	0.58688766	0.011745691
A_33_P3349444	*CBLN3*	Homo sapiens cerebellin 3 precursor (CBLN3), mRNA [NM_001039771]	-1.0042546	0.0041847117
A_33_P3346826	*YTHDF3*	Homo sapiens YTH N(6)-methyladenosine RNA binding protein 3 (YTHDF3), transcript variant 5, mRNA [NM_001277816]	0.69289684	0.01367917
A_33_P3383912	*NAGA*	Homo sapiens N-acetylgalactosaminidase, alpha- (NAGA), mRNA [NM_000262]	-0.79514176	0.014864522
A_32_P517749	*IL32*	Homo sapiens interleukin 32 (IL32), transcript variant 4, mRNA [NM_001012633]	2.905571	0.0034534817
A_19_P00806930	*HLA-DRB3*	Homo sapiens major histocompatibility complex, class II, DR beta 3 (HLA-DRB3), mRNA [NM_022555]	0.9686124	0.013185053
A_32_P156851	*RPS6KA3*	Homo sapiens ribosomal protein S6 kinase, 90kDa, polypeptide 3 (RPS6KA3), mRNA [NM_004586]	-1.2611322	0.0062618116
A_32_P24581	*XLOC_l2_003627*	BROAD Institute lincRNA (XLOC_l2_003627), lincRNA [TCONS_l2_00006718]	0.8676297	0.0048452313
A_23_P103775	*RCAN2*	Homo sapiens regulator of calcineurin 2 (RCAN2), transcript variant 1, mRNA [NM_005822]	-1.7352428	0.0035734554
A_22_P00013430	*RPS27A*	Homo sapiens ribosomal protein S27a (RPS27A), transcript variant 1, mRNA [NM_002954]	0.5924144	0.010237655
A_23_P92093	*LRRC8C*	Homo sapiens leucine rich repeat containing 8 family, member C (LRRC8C), mRNA [NM_032270]	1.1459187	0.0039416323

A_33_P3291454	*BTG1*	Homo sapiens B-cell translocation gene 1, anti-proliferative (BTG1), mRNA [NM_001731]	-0.9483234	0.005763634
A_33_P3358312	*CELSR3*	Homo sapiens cadherin, EGF LAG seven-pass G-type receptor 3 (CELSR3), mRNA [NM_001407]	0.48225576	0.009457829
A_23_P161338	*CCDC172*	Homo sapiens coiled-coil domain containing 172 (CCDC172), mRNA [NM_198515]	1.0528648	0.014660574
A_23_P70060	*OTUD6B*	Homo sapiens OTU domain containing 6B (OTUD6B), transcript variant 1, mRNA [NM_016023]	-0.6575799	0.015764253
A_33_P3251227	*PPA1*	Homo sapiens pyrophosphatase (inorganic) 1 (PPA1), mRNA [NM_021129]	-0.6677418	0.00817131
A_22_P00019101	*PPAP2A*	Homo sapiens phosphatidic acid phosphatase type 2A (PPAP2A), transcript variant 2, mRNA [NM_176895]	-1.2914128	0.007603999
A_21_P0005182	*C1GALT1*	core 1 synthase, glycoprotein-N-acetylgalactosamine 3-beta-galactosyltransferase 1 [Source:HGNC Symbol;Acc:HGNC:24337] [ENST00000402468]	-0.5833757	0.013456115
A_24_P271049	*CREB1*	Homo sapiens cAMP responsive element binding protein 1 (CREB1), transcript variant B, mRNA [NM_134442]	-0.9885316	0.0056962976
A_21_P0001049	*lnc-SDIM1-3*	LNCipedia lincRNA (lnc-SDIM1-3), lincRNA [lnc-SDIM1-3:7]	2.832468	0.010986003
A_33_P3260053	*C18orf32*	Homo sapiens chromosome 18 open reading frame 32 (C18orf32), transcript variant 2, mRNA [NM_001035005]	-0.9196154	0.0052543716
A_33_P3227269	*AIF1L*	Homo sapiens allograft inflammatory factor 1-like (AIF1L), transcript variant 3, mRNA [NM_001185095]	1.8408146	0.0046717683
A_23_P128613	*LAMP2*	Homo sapiens lysosomal-associated membrane protein 2 (LAMP2), transcript variant B, mRNA [NM_013995]	-0.78697467	0.0031941293
A_33_P3338928	*GATA3-AS1*	Homo sapiens GATA3 antisense RNA 1 (GATA3-AS1), transcript variant 2, long non-coding RNA [NR_024256]	4.352149	0.005057235
A_23_P92543	*KDELC1*	Homo sapiens KDEL (Lys-Asp-Glu-Leu) containing 1 (KDELC1), mRNA [NM_024089]	-0.69461846	0.0098649
A_23_P337270	*DAB2*	Homo sapiens Dab, mitogen-responsive phosphoprotein, homolog 2 (Drosophila) (DAB2), transcript variant 1, mRNA [NM_001343]	-1.4715362	0.0027348737
A_33_P3318861	*SCFD2*	Homo sapiens sec1 family domain containing 2 (SCFD2), mRNA [NM_152540]	-0.9617123	0.016855255
A_21_P0000011	*NEK10*	NIMA-related kinase 10 [Source:HGNC Symbol;Acc:HGNC:18592] [ENST00000383771]	-0.57807064	0.0071450765
A_24_P169843	*DYTN*	Homo sapiens dystrotelin (DYTN), mRNA [NM_001093730]	-0.5384993	0.015505246

A_33_P3421203	*AMDHD2*	Homo sapiens amidohydrolase domain containing 2 (AMDHD2), transcript variant 2, mRNA [NM_001145815]	0.9631295	0.011893636
A_23_P43566	*FAM86B3P*	Homo sapiens family with sequence similarity 86, member B3, pseudogene (FAM86B3P), transcript variant 1, non-coding RNA [NR_024362]	-1.3777698	0.004659113
A_24_P276816	*INPP5F*	Homo sapiens inositol polyphosphate-5-phosphatase F (INPP5F), transcript variant 3, mRNA [NM_001243195]	-0.97013545	0.009918909
A_23_P99906	*NDUFA8*	Homo sapiens NADH dehydrogenase (ubiquinone) 1 alpha subcomplex, 8, 19kDa (NDUFA8), mRNA [NM_014222]	-0.88841623	0.0029015075
A_23_P167269	*RCOR3*	Homo sapiens REST corepressor 3 (RCOR3), transcript variant 4, mRNA [NM_018254]	-0.7094423	0.011517759
A_23_P23074	*HOMER2*	Homo sapiens homer homolog 2 (Drosophila) (HOMER2), transcript variant 2, mRNA [NM_199330]	-0.2860825	0.016238803
A_21_P0004503	*TMA16*	Homo sapiens translation machinery associated 16 homolog (S. cerevisiae) (TMA16), mRNA [NM_018352]	-0.33170465	0.0090693105
A_21_P0007430	*IFI44*	Homo sapiens interferon-induced protein 44 (IFI44), mRNA [NM_006417]	0.6185382	0.0025001846
A_23_P38723	*LOC257396*	Homo sapiens uncharacterized LOC257396 (LOC257396), transcript variant 1, long non-coding RNA [NR_034107]	0.24263	0.016893262
A_24_P360529	*LOC494141*	Homo sapiens cDNA clone IMAGE:4152388, partial cds. [BC013074]	-1.5158182	0.010301823
A_33_P3405904	*SMCHD1*	Homo sapiens structural maintenance of chromosomes flexible hinge domain containing 1 (SMCHD1), mRNA [NM_015295]	-0.3237758	0.013076766
A_23_P345830	*PDE7A*	Homo sapiens cAMP-specific cyclic nucleotide phosphodiesterase PDE7A3 mRNA, complete cds. [AF332652]	-1.6728588	0.0056065638
A_33_P3221788	*FASTKD2*	Homo sapiens FAST kinase domains 2 (FASTKD2), transcript variant 3, mRNA [NM_014929]	-0.42355078	0.015419179
A_23_P404091	*SNORD114-3*	Homo sapiens small nucleolar RNA, C/D box 114-3 (SNORD114-3), small nucleolar RNA [NR_003195]	0.5186319	0.012437972
A_21_P0011382	*RAB22A*	Homo sapiens RAB22A, member RAS oncogene family (RAB22A), mRNA [NM_020673]	-0.5819392	0.0058361553
A_33_P3299436	*GRPEL2*	Homo sapiens GrpE-like 2, mitochondrial (E. coli) (GRPEL2), mRNA [NM_152407]	-0.9447584	0.0036240483
A_23_P89755	*UBE2Q2P1*	ubiquitin-conjugating enzyme E2Q family member 2 pseudogene 1 [Source:HGNC Symbol;Acc:HGNC:37439] [ENST00000354771]	-0.80190164	0.009421056

A_33_P3209497	*RNF138*	Homo sapiens ring finger protein 138, E3 ubiquitin protein ligase (RNF138), transcript variant 1, mRNA [NM_016271]	-0.4914086	0.010402669
A_23_P252082	*COPB1*	Homo sapiens coatomer protein complex, subunit beta 1 (COPB1), transcript variant 1, mRNA [NM_016451]	-0.78733563	0.0067319144
A_22_P00024313	*TMEM176A*	Homo sapiens transmembrane protein 176A (TMEM176A), mRNA [NM_018487]	3.1292715	0.0029015075
A_33_P3298535	*TM2D1*	Homo sapiens TM2 domain containing 1 (TM2D1), mRNA [NM_032027]	-1.293309	0.0046155252
A_22_P00017171	*lnc-MFSD2A-1*	LNCipedia lincRNA (lnc-MFSD2A-1), lincRNA [lnc-MFSD2A-1:1]	-1.162662	0.010092017
A_23_P108673	*ROMO1*	Homo sapiens reactive oxygen species modulator 1 (ROMO1), mRNA [NM_080748]	-0.36900634	0.01263861
A_33_P3406899	*lnc-UBLCP1-3*	601309061F1 NIH_MGC_44 Homo sapiens cDNA clone IMAGE:3630806 5', mRNA sequence [BE393031]	0.8807808	0.011656639
A_23_P500773	*EVA1A*	Homo sapiens eva-1 homolog A (C. elegans) (EVA1A), transcript variant 2, mRNA [NM_032181]	-0.88860315	0.005947757
A_33_P3278152	*TRAK1*	Homo sapiens trafficking protein, kinesin binding 1 (TRAK1), transcript variant 3, mRNA [NM_001265608]	-2.124828	0.0036517929
A_24_P89512	*MAP3K7*	Homo sapiens mitogen-activated protein kinase kinase kinase 7 (MAP3K7), transcript variant A, mRNA [NM_003188]	-0.7087247	0.004609308
A_23_P406702	*BCLAF1*	Homo sapiens BCL2-associated transcription factor 1 (BCLAF1), transcript variant 1, mRNA [NM_014739]	-0.48078132	0.008785588
A_23_P132536	*lnc-CCKAR-1*	LNCipedia lincRNA (lnc-CCKAR-1), lincRNA [lnc-CCKAR-1:1]	1.371983	0.009294259
A_33_P3485760	*CAMSAP2*	Homo sapiens calmodulin regulated spectrin-associated protein family, member 2 (CAMSAP2), transcript variant 2, mRNA [NM_203459]	-0.5804248	0.009057305
A_23_P130444	*TRAK1*	Homo sapiens trafficking protein, kinesin binding 1 (TRAK1), transcript variant 1, mRNA [NM_001042646]	-1.8396192	0.0054418826
A_24_P129232	*SNORA19*	AGENCOURT_14540417 NIA Human H1 Embryonic Stem Cell cDNA Library (Long) Homo sapiens cDNA clone IMAGE:30423983 5', mRNA sequence [CD654100]	1.5865955	0.00847175
A_22_P00008940	*ZNF701*	Homo sapiens zinc finger protein 701 (ZNF701), transcript variant 2, mRNA [NM_018260]	-0.95415306	0.010879834
A_33_P3282181	*SERINC1*	Homo sapiens serine incorporator 1 (SERINC1), mRNA [NM_020755]	-0.84466314	0.0071928417

A_23_P202978	*ARHGAP4*	Homo sapiens Rho GTPase activating protein 4 (ARHGAP4), transcript variant 1, mRNA [NM_001164741]	1.270467	0.005057235
A_21_P0006496	*PRDM10*	Homo sapiens PR domain containing 10 (PRDM10), transcript variant 2, mRNA [NM_199437]	-0.59923714	0.014517222
A_23_P73114	*CASP1*	Homo sapiens caspase 1, apoptosis-related cysteine peptidase (CASP1), transcript variant alpha, mRNA [NM_033292]	1.6218503	0.0024890031
A_23_P259172	*GAGE2B*	Homo sapiens G antigen 2B (GAGE2B), mRNA [NM_001098411]	-2.1605773	0.0029497955
A_23_P163143	*PROS1*	Homo sapiens protein S (alpha) (PROS1), mRNA [NM_000313]	-1.5547693	0.005239873
A_24_P627984	*SSR4*	Homo sapiens signal sequence receptor, delta (SSR4), transcript variant 2, mRNA [NM_006280]	-0.8924341	0.009690877
A_23_P360797	*ACYP1*	Homo sapiens acylphosphatase 1, erythrocyte (common) type (ACYP1), transcript variant 4, mRNA [NM_001302617]	-0.57579494	0.011566051
A_21_P0012584	*EPM2AIP1*	Homo sapiens EPM2A (laforin) interacting protein 1 (EPM2AIP1), mRNA [NM_014805]	-1.067019	0.0105530135
A_23_P329890	*NTF3*	Homo sapiens neurotrophin 3 (NTF3), transcript variant 2, mRNA [NM_002527]	-2.18528	0.0035935708
A_33_P3637909	*LOC101060498*	Homo sapiens uncharacterized LOC101060498 (LOC101060498), transcript variant 1, long non-coding RNA [NR_121640]	0.7562313	0.0039486005
A_22_P00025545	*TMEM136*	Homo sapiens transmembrane protein 136 (TMEM136), transcript variant 2, mRNA [NM_174926]	-0.77164096	0.0044371844
A_33_P3337719	*UBE2D4*	Homo sapiens ubiquitin-conjugating enzyme E2D 4 (putative) (UBE2D4), mRNA [NM_015983]	-0.9962775	0.011654807
A_33_P3282434	*CECR7*	Homo sapiens cat eye syndrome chromosome region, candidate 7 (non-protein coding) (CECR7), long non-coding RNA [NR_015352]	-2.2055295	0.003392081
A_24_P381604	*LOC100134868*	Homo sapiens uncharacterized LOC100134868 (LOC100134868), long non-coding RNA [NR_004846]	-1.0517848	0.012512322
A_33_P3225567	*SLC16A1*	Homo sapiens solute carrier family 16 (monocarboxylate transporter), member 1 (SLC16A1), transcript variant 2, mRNA [NM_001166496]	-0.40563244	0.011879074
A_33_P3365676	*ITM2B*	Homo sapiens integral membrane protein 2B (ITM2B), mRNA [NM_021999]	-1.3110231	0.014260212
A_33_P3316293	*TRMT2B*	Homo sapiens tRNA methyltransferase 2 homolog B (S. cerevisiae) (TRMT2B), transcript variant 1, mRNA [NM_024917]	-0.61369246	0.015893938

A_23_P94216	*PTPLAD1*	Homo sapiens protein tyrosine phosphatase-like A domain containing 1 (PTPLAD1), mRNA [NM_016395]	-0.48827264	0.008978059
A_33_P3367565	*CAB39*	Homo sapiens calcium binding protein 39 (CAB39), transcript variant 2, mRNA [NM_001130849]	-0.3314138	0.011456508
A_33_P3303212	*LONRF1*	Homo sapiens LON peptidase N-terminal domain and ring finger 1 (LONRF1), mRNA [NM_152271]	-0.7961807	0.0039522685
A_23_P68665	*FNBP1*	PREDICTED: Homo sapiens formin binding protein 1 (FNBP1), transcript variant X1, mRNA [XM_005251815]	-1.0669202	0.0037479755
A_24_P372217	*CCDC74A*	Homo sapiens coiled-coil domain containing 74A (CCDC74A), transcript variant 2, mRNA [NM_001258304]	-0.5490657	0.009988937
A_24_P351420	*ADRM1*	Homo sapiens adhesion regulating molecule 1 (ADRM1), transcript variant 1, mRNA [NM_007002]	1.1516037	0.0067923404
A_33_P3350488	*ERLIN2*	Homo sapiens ER lipid raft associated 2 (ERLIN2), transcript variant 1, mRNA [NM_007175]	-1.020199	0.004275752
A_23_P213085	*ZDHHC3*	Homo sapiens zinc finger, DHHC-type containing 3 (ZDHHC3), transcript variant 2, mRNA [NM_016598]	-0.660347	0.0039486005
A_23_P161727	*NUSAP1*	Homo sapiens nucleolar and spindle associated protein 1 (NUSAP1), transcript variant 1, mRNA [NM_016359]	-0.58911633	0.009811661
A_24_P269619	*SLC10A7*	Homo sapiens solute carrier family 10, member 7 (SLC10A7), transcript variant 4, mRNA [NM_001300842]	-0.59941983	0.0059070494
A_33_P3258467	*HSPB2*	Homo sapiens heat shock 27kDa protein 2 (HSPB2), mRNA [NM_001541]	-2.4259343	0.0029471128
A_33_P3244578	*DECR1*	Homo sapiens 2,4-dienoyl CoA reductase 1, mitochondrial (DECR1), mRNA [NM_001359]	-0.8082965	0.004288771
A_22_P00016355	*SPTBN1*	Homo sapiens spectrin, beta, non-erythrocytic 1 (SPTBN1), transcript variant 2, mRNA [NM_178313]	-1.5175273	0.0031941293
A_33_P3265016	*LOC100129675*	Homo sapiens cDNA FLJ43071 fis, clone BRTHA3009291. [AK125061]	1.4070662	0.010942903
A_23_P65089	*LOC728730*	Homo sapiens uncharacterized LOC728730 (LOC728730), long non-coding RNA [NR_037875]	1.1322447	0.007771992
A_24_P419276	*PEX6*	Homo sapiens peroxisomal biogenesis factor 6 (PEX6), mRNA [NM_000287]	0.64472723	0.015419179
A_19_P00318813	*DDX47*	Homo sapiens DEAD (Asp-Glu-Ala-Asp) box polypeptide 47 (DDX47), transcript variant 1, mRNA [NM_016355]	-0.8379876	0.00856985

A_22_P00019165	*ZNF248*	Homo sapiens zinc finger protein 248 (ZNF248), transcript variant 1, mRNA [NM_021045]	-0.48303807	0.01481567
A_23_P430161	*FOXN3*	forkhead box N3 [Source:HGNC Symbol;Acc:HGNC:1928] [ENST00000553904]	0.5162789	0.0068410696
A_23_P118842	*PCOLCE2*	Homo sapiens procollagen C-endopeptidase enhancer 2 (PCOLCE2), mRNA [NM_013363]	-5.1528435	0.0024890031
A_21_P0014195	*EXOC8*	Homo sapiens exocyst complex component 8 (EXOC8), mRNA [NM_175876]	-1.006928	0.007205146
A_33_P3273020	*KRTAP1-5*	Homo sapiens keratin associated protein 1-5 (KRTAP1-5), mRNA [NM_031957]	-1.0580778	0.005842833
A_24_P161036	*FKBP2*	Homo sapiens FK506 binding protein 2, 13kDa (FKBP2), transcript variant 1, mRNA [NM_004470]	-0.9197259	0.0025739188
A_23_P109143	*XLOC_l2_013873*	BROAD Institute lincRNA (XLOC_l2_013873), lincRNA [TCONS_l2_00026658]	-1.0499887	0.010495637
A_22_P00004645	*ACOT1*	Homo sapiens acyl-CoA thioesterase 1 (ACOT1), mRNA [NM_001037161]	-1.1107407	0.010134365
A_23_P412427	*PRNP*	Homo sapiens prion protein (PRNP), transcript variant 1, mRNA [NM_000311]	-0.26655608	0.006812377
A_23_P102262	*lnc-CTBP2-1*	LNCipedia lincRNA (lnc-CTBP2-1), lincRNA [lnc-CTBP2-1:1]	-0.8986042	0.006606754
A_33_P3217119	*LOC171391*	Homo sapiens NS3 binding protein (NS3BP) mRNA, complete cds. [AF435951]	1.0944215	0.0058047334
A_23_P28590	*MRPL19*	Homo sapiens mitochondrial ribosomal protein L19 (MRPL19), mRNA [NM_014763]	-0.6956069	0.0051764864
A_23_P217151	*OSCP1*	Homo sapiens organic solute carrier partner 1 (OSCP1), transcript variant 2, mRNA [NM_206837]	-1.1414281	0.0027874883
A_23_P83751	*FASTKD1*	Homo sapiens FAST kinase domains 1 (FASTKD1), transcript variant 1, mRNA [NM_024622]	-0.67466503	0.0072139124
A_33_P3269718	*KRBOX4*	Homo sapiens KRAB box domain containing 4 (KRBOX4), transcript variant 2, mRNA [NM_017776]	-0.71013904	0.010168826
A_33_P3211153	*C11orf63*	Homo sapiens chromosome 11 open reading frame 63 (C11orf63), transcript variant 1, mRNA [NM_024806]	-2.21064	0.0041475594
A_23_P80643	*IVD*	Homo sapiens isovaleryl-CoA dehydrogenase (IVD), transcript variant 2, mRNA [NM_001159508]	-0.89759207	0.0090693105
A_33_P3377005	*ANKS3*	Homo sapiens ankyrin repeat and sterile alpha motif domain containing 3 (ANKS3), transcript variant 1, mRNA [NM_133450]	-0.36315247	0.014392089
A_33_P3320804	*SETMAR*	Homo sapiens SET domain and mariner transposase fusion gene (SETMAR), transcript variant 1, mRNA [NM_006515]	-0.91880226	0.008649646

A_23_P150281	*LMNTD2*	Homo sapiens lamin tail domain containing 2 (LMNTD2), mRNA [NM_173573]	-0.43170276	0.015625326
A_32_P108156	*IP6K1*	Homo sapiens inositol hexakisphosphate kinase 1 (IP6K1), transcript variant 3, mRNA [NM_001242829]	-0.6076396	0.013985718
A_24_P365322	*TP53I11*	Homo sapiens tumor protein p53 inducible protein 11 (TP53I11), transcript variant 1, mRNA [NM_001258320]	1.4103984	0.006395745
A_33_P3282489	*MIR155HG*	Homo sapiens MIR155 host gene (non-protein coding) (MIR155HG), long non-coding RNA [NR_001458]	0.9593793	0.004120151
A_23_P69109	*ZNF709*	Homo sapiens zinc finger protein 709 (ZNF709), mRNA [NM_152601]	-0.6844532	0.0065241847
A_23_P396858	*GCNT1*	Homo sapiens glucosaminyl (N-acetyl) transferase 1, core 2 (GCNT1), transcript variant 1, mRNA [NM_001097634]	-2.3165598	0.0024890031
A_24_P250765	*PLSCR1*	Homo sapiens phospholipid scramblase 1 (PLSCR1), mRNA [NM_021105]	-0.54134053	0.011054281
A_24_P396197	*FZD8*	Homo sapiens frizzled class receptor 8 (FZD8), mRNA [NM_031866]	0.5045232	0.012620988
A_21_P0008039	*PRKG1*	Homo sapiens protein kinase, cGMP-dependent, type I (PRKG1), transcript variant 2, mRNA [NM_006258]	-0.9182382	0.0106466925
A_21_P0014365	*PRKCSH*	Homo sapiens protein kinase C substrate 80K-H (PRKCSH), transcript variant 1, mRNA [NM_002743]	1.5234921	0.005712846
A_23_P29655	*lnc-WASF3-1*	LNCipedia lincRNA (lnc-WASF3-1), lincRNA [lnc-WASF3-1:1]	-0.61830425	0.012334684
A_23_P106481	*GACAT2*	Homo sapiens gastric cancer associated transcript 2 (non-protein coding) (GACAT2), long non-coding RNA [NR_120598]	-2.1539774	0.0038144474
A_33_P3402763	*C3orf14*	Homo sapiens chromosome 3 open reading frame 14 (C3orf14), transcript variant 1, mRNA [NM_020685]	-0.70571154	0.0035238392
A_23_P251316	*EMC4*	Homo sapiens ER membrane protein complex subunit 4 (EMC4), transcript variant 1, mRNA [NM_016454]	-0.8330206	0.005428736
A_21_P0004274	*NIFK*	Homo sapiens nucleolar protein interacting with the FHA domain of MKI67 (NIFK), mRNA [NM_032390]	-0.6691766	0.005514184
A_33_P3244122	*CTNND1*	Homo sapiens catenin (cadherin-associated protein), delta 1 (CTNND1), transcript variant 3, mRNA [NM_001331]	-0.83198994	0.014448771
A_23_P157726	*lnc-RASA1-3*	LNCipedia lincRNA (lnc-RASA1-3), lincRNA [lnc-RASA1-3:12]	-0.7383526	0.009099136
A_33_P3422787	*HAAO*	Homo sapiens 3-hydroxyanthranilate 3,4-dioxygenase (HAAO), mRNA [NM_012205]	-1.7500821	0.014731476

A_23_P81158	*DENND4C*	DENN/MADD domain containing 4C [Source:HGNC Symbol;Acc:HGNC:26079] [ENST00000380437]	-0.78638935	0.008447855
A_24_P55225	*lnc-TM4SF4-2*	Homo sapiens cDNA, FLJ99482. [AK309441]	0.7705028	0.010735533
A_24_P370156	*ADH1C*	Homo sapiens alcohol dehydrogenase 1C (class I), gamma polypeptide (ADH1C), mRNA [NM_000669]	2.037057	0.0032379
A_22_P00003894	*RSPH9*	Homo sapiens radial spoke head 9 homolog (Chlamydomonas) (RSPH9), transcript variant 1, mRNA [NM_152732]	-0.97986245	0.010822477
A_33_P3306964	*MAN2B1*	Homo sapiens mannosidase, alpha, class 2B, member 1 (MAN2B1), transcript variant 1, mRNA [NM_000528]	0.97938913	0.0073898793
A_23_P108376	*lnc-CEP170-5*	Q86TS6_HUMAN (Q86TS6) Full-length cDNA 5-PRIME end of clone CS0CAP004YO05 of Thymus of Homo sapiens (human) (Fragment), partial (21%) [THC2770348]	-0.832615	0.010931163
A_33_P3421418	*PPP1R2*	Homo sapiens protein phosphatase 1, regulatory (inhibitor) subunit 2 (PPP1R2), transcript variant 2, mRNA [NM_006241]	-0.90462196	0.013124348
A_21_P0011084	*THADA*	Homo sapiens thyroid adenoma associated (THADA), transcript variant 1, mRNA [NM_022065]	-0.66692793	0.011376762
A_33_P3415551	*XLOC_l2_003293*	BROAD Institute lincRNA (XLOC_l2_003293), lincRNA [TCONS_l2_00006125]	0.6972219	0.016251752
A_24_P341019	*INPP5B*	Homo sapiens inositol polyphosphate-5-phosphatase, 75kDa (INPP5B), transcript variant 1, mRNA [NM_005540]	-0.76415986	0.014904488
A_32_P10396	*GPAT2*	Homo sapiens glycerol-3-phosphate acyltransferase 2, mitochondrial (GPAT2), mRNA [NM_207328]	0.40253994	0.011439164
A_23_P332374	*TMEM230*	Homo sapiens transmembrane protein 230 (TMEM230), transcript variant 2, mRNA [NM_001009924]	-0.74842614	0.0057647754
A_33_P3315425	*WDFY3*	Homo sapiens WD repeat and FYVE domain containing 3 (WDFY3), mRNA [NM_014991]	-0.904921	0.0070605814
A_23_P37983	*ZNF175*	Homo sapiens zinc finger protein 175 (ZNF175), mRNA [NM_007147]	-0.6515259	0.01225594
A_33_P3394517	*METTL4*	Homo sapiens methyltransferase like 4 (METTL4), mRNA [NM_022840]	-0.7776699	0.006299558
A_21_P0005474	*MT1B*	Homo sapiens metallothionein 1B (MT1B), mRNA [NM_005947]	1.803966	0.005377644
A_33_P3399581	*SMG1*	Homo sapiens SMG1 phosphatidylinositol 3-kinase-related kinase (SMG1), mRNA [NM_015092]	0.46313554	0.014792068
A_24_P71649	*TWIST1*	PREDICTED: Homo sapiens twist family bHLH transcription factor 1 (TWIST1), transcript variant X2, misc_RNA [XR_428085]	-0.7317409	0.016899958

A_33_P3267160	*TRDMT1*	Homo sapiens tRNA aspartic acid methyltransferase 1 (TRDMT1), mRNA [NM_004412]	-0.53698456	0.0071605598
A_19_P00327354	*CNTN3*	Homo sapiens contactin 3 (plasmacytoma associated) (CNTN3), mRNA [NM_020872]	-1.2133889	0.0050497777
A_32_P35512	*CYB5R4*	cytochrome b5 reductase 4 [Source:HGNC Symbol;Acc:HGNC:20147] [ENST00000369681]	-0.48078156	0.016964827
A_23_P15864	*LINC00963*	long intergenic non-protein coding RNA 963 [Source:HGNC Symbol;Acc:HGNC:48716] [ENST00000419300]	-0.6997572	0.011962944
A_32_P96692	*SSB*	Homo sapiens Sjogren syndrome antigen B (autoantigen La) (SSB), transcript variant 1, mRNA [NM_003142]	-0.46290734	0.008273267
A_19_P00318280	*MBD2*	Homo sapiens methyl-CpG binding domain protein 2 (MBD2), transcript variant 1, mRNA [NM_003927]	-0.9454465	0.008427824
A_33_P3361701	*POLH*	Homo sapiens polymerase (DNA directed), eta (POLH), transcript variant 1, mRNA [NM_006502]	-1.7333076	0.005776505
A_24_P178175	*LOC100506860*	Homo sapiens uncharacterized LOC100506860 (LOC100506860), long non-coding RNA [NR_109780]	-1.5606161	0.0067036957
A_33_P3421571	*UBAP2L*	Homo sapiens ubiquitin associated protein 2-like (UBAP2L), transcript variant 3, mRNA [NM_001287815]	1.1308048	0.007811618
A_23_P82047	*GGT1*	Homo sapiens gamma-glutamyltransferase 1 (GGT1), transcript variant 6, mRNA [NM_001288833]	0.41746998	0.014934347
A_24_P289299	*RAPH1*	Homo sapiens Ras association (RalGDS/AF-6) and pleckstrin homology domains 1 (RAPH1), transcript variant 1, mRNA [NM_213589]	1.3133557	0.006605609
A_22_P00005343	*STXBP5*	Homo sapiens syntaxin binding protein 5 (tomosyn) (STXBP5), transcript variant 2, mRNA [NM_001127715]	-0.57058024	0.0044707763
A_23_P251705	*ARHGEF25*	Homo sapiens Rho guanine nucleotide exchange factor (GEF) 25 (ARHGEF25), transcript variant 1, mRNA [NM_182947]	0.8654637	0.0120555395
A_33_P3252695	*KCNG1*	Homo sapiens potassium channel, voltage gated modifier subfamily G, member 1 (KCNG1), mRNA [NM_002237]	-1.0239737	0.008346524
A_23_P165722	*ARHGEF9*	Homo sapiens Cdc42 guanine nucleotide exchange factor (GEF) 9 (ARHGEF9), transcript variant 1, mRNA [NM_015185]	-0.50321066	0.012706064
A_23_P25913	*CYTL1*	Homo sapiens cytokine-like 1 (CYTL1), mRNA [NM_018659]	-1.7621574	0.005057235
A_24_P101561	*EIF4E2*	Homo sapiens eukaryotic translation initiation factor 4E family member 2 (EIF4E2), transcript variant 1, mRNA [NM_004846]	-0.78957087	0.007180822

A_33_P3226600	*DNAL1*	Homo sapiens dynein, axonemal, light chain 1 (DNAL1), transcript variant 1, mRNA [NM_031427]	-1.0539806	0.0050712093
A_23_P254498	*GPAA1*	Homo sapiens glycosylphosphatidylinositol anchor attachment 1 (GPAA1), mRNA [NM_003801]	-0.84636736	0.010013196
A_23_P329261	*HYAL1*	Homo sapiens hyaluronoglucosaminidase 1 (HYAL1), transcript variant 8, mRNA [NM_153281]	-0.9802366	0.009259001
A_32_P120895	*RANGRF*	Homo sapiens RAN guanine nucleotide release factor (RANGRF), transcript variant 1, mRNA [NM_016492]	-0.7499008	0.009690877
A_33_P3404489	*KCNJ2*	Homo sapiens potassium channel, inwardly rectifying subfamily J, member 2 (KCNJ2), mRNA [NM_000891]	2.1933594	0.0032831163
A_33_P3382118	*LYSMD2*	Homo sapiens LysM, putative peptidoglycan-binding, domain containing 2 (LYSMD2), transcript variant 1, mRNA [NM_153374]	-0.3793523	0.016977986
A_33_P3308686	*MAMLD1*	Homo sapiens mastermind-like domain containing 1 (MAMLD1), transcript variant 1, mRNA [NM_001177465]	-1.3845942	0.0058361553
A_33_P3550894	*CC2D2A*	Homo sapiens coiled-coil and C2 domain containing 2A (CC2D2A), transcript variant 2, mRNA [NM_020785]	-1.1586412	0.009649863
A_33_P3247022	*NR4A3*	Homo sapiens nuclear receptor subfamily 4, group A, member 3 (NR4A3), transcript variant 3, mRNA [NM_173200]	0.8401888	0.005961688
A_23_P374767	*GATA2*	Homo sapiens GATA binding protein 2 (GATA2), transcript variant 1, mRNA [NM_001145661]	3.136744	0.0020435157
A_19_P00803960	*CCNE2*	Homo sapiens cyclin E2 (CCNE2), mRNA [NM_057749]	0.68077874	0.009466768
A_24_P70117	*UBE2D4*	Homo sapiens ubiquitin-conjugating enzyme E2D 4 (putative) (UBE2D4), mRNA [NM_015983]	-0.82976174	0.013858261
A_24_P225679	*THAP9-AS1*	Homo sapiens THAP9 antisense RNA 1 (THAP9-AS1), transcript variant 1, long non-coding RNA [NR_034075]	-0.6279233	0.012250454
A_32_P180265	*DTWD1*	Homo sapiens DTW domain containing 1 (DTWD1), transcript variant 1, mRNA [NM_020234]	-1.008821	0.004437345
A_23_P126057	*IRS1*	Homo sapiens insulin receptor substrate 1 (IRS1), mRNA [NM_005544]	0.7244723	0.009556777
A_22_P00012024	*SCP2*	Homo sapiens sterol carrier protein 2 (SCP2), transcript variant 1, mRNA [NM_002979]	-0.768466	0.0053083743
A_23_P335905	*GCOM1*	Homo sapiens GRINL1A complex locus 1 (GCOM1), transcript variant 1, mRNA [NM_001018090]	-0.86846375	0.006772767

A_23_P159650	*TTN-AS1*	Homo sapiens TTN antisense RNA 1 (TTN-AS1), transcript variant 2, long non-coding RNA [NR_038271]	0.7145354	0.015540036
A_23_P315815	*STK36*	Homo sapiens serine/threonine kinase 36 (STK36), transcript variant 1, mRNA [NM_015690]	-0.3612511	0.01530907
A_33_P3386459	*COX7B*	Homo sapiens cytochrome c oxidase subunit VIIb (COX7B), mRNA [NM_001866]	-0.5318389	0.008537068
A_19_P00322339	*NRG1*	Homo sapiens neuregulin 1 (NRG1), transcript variant HRG-gamma, mRNA [NM_004495]	-3.6228538	0.0033868244
A_33_P3254801	*ARHGAP5*	Homo sapiens Rho GTPase activating protein 5 (ARHGAP5), transcript variant 1, mRNA [NM_001030055]	-1.1516073	0.0041916566
A_23_P29555	*LINC00707*	Homo sapiens long intergenic non-protein coding RNA 707 (LINC00707), long non-coding RNA [NR_038291]	2.6741364	0.0033868244
A_24_P49371	*OGFR*	Homo sapiens opioid growth factor receptor (OGFR), mRNA [NM_007346]	1.1076969	0.0066995043
A_33_P3332970	*SEC13*	Homo sapiens SEC13 homolog (S. cerevisiae) (SEC13), transcript variant 1, mRNA [NM_183352]	-0.6076298	0.0049014823
A_24_P231104	*C4orf27*	Homo sapiens chromosome 4 open reading frame 27 (C4orf27), mRNA [NM_017867]	-0.7320955	0.014970456
A_33_P3225882	*CLEC2B*	Homo sapiens C-type lectin domain family 2, member B (CLEC2B), mRNA [NM_005127]	1.2880993	0.0066432646
A_32_P155631	*LEPR*	Homo sapiens leptin receptor (LEPR), transcript variant 3, mRNA [NM_001003679]	-1.8600825	0.0028913463
A_22_P00021090	*BET1L*	Homo sapiens Bet1 golgi vesicular membrane trafficking protein-like (BET1L), transcript variant 1, mRNA [NM_001098787]	-0.42968562	0.013706757
A_23_P80827	*SDHD*	Homo sapiens succinate dehydrogenase complex, subunit D, integral membrane protein (SDHD), transcript variant 1, mRNA [NM_003002]	-1.0329881	0.003609745
A_33_P3362068	*lnc-MDH1B-2*	LNCipedia lincRNA (lnc-MDH1B-2), lincRNA [lnc-MDH1B-2:1]	-1.8854138	0.0037856582
A_23_P258018	*FYTTD1*	Homo sapiens forty-two-three domain containing 1 (FYTTD1), transcript variant 2, mRNA [NM_001011537]	-0.7586868	0.0030638003
A_24_P408424	*MYL5*	Homo sapiens myosin, light chain 5, regulatory (MYL5), mRNA [NM_002477]	-0.8324724	0.01252379
A_33_P3331307	*BPTF*	Homo sapiens bromodomain PHD finger transcription factor (BPTF), transcript variant 1, mRNA [NM_182641]	-0.61582726	0.0041847117
A_33_P3382489	*MYH9*	Homo sapiens myosin, heavy chain 9, non-muscle (MYH9), mRNA [NM_002473]	-0.8381348	0.011356648

A_22_P00003242	*CDC42EP3*	Homo sapiens CDC42 effector protein (Rho GTPase binding) 3 (CDC42EP3), transcript variant 1, mRNA [NM_006449]	-1.4222697	0.006812377
A_23_P28598	*GSN*	Homo sapiens gelsolin (GSN), transcript variant 4, mRNA [NM_001127663]	-1.181978	0.004803986
A_23_P86182	*LINC00969*	Homo sapiens long intergenic non-protein coding RNA 969 (LINC00969), long non-coding RNA [NR_122105]	0.67618126	0.015050633
A_23_P141847	*DLX2*	Homo sapiens distal-less homeobox 2 (DLX2), mRNA [NM_004405]	2.0058331	0.003121324
A_32_P206293	*MRPS21*	Homo sapiens mitochondrial ribosomal protein S21 (MRPS21), transcript variant 1, mRNA [NM_031901]	-0.46957424	0.0095815705
A_23_P169197	*UBE2M*	Homo sapiens ubiquitin-conjugating enzyme E2M (UBE2M), mRNA [NM_003969]	0.7753959	0.012007281
A_24_P724153	*ZNF322*	Homo sapiens zinc finger protein 322 (ZNF322), transcript variant 1, mRNA [NM_001242797]	-1.280396	0.008497114
A_33_P3313660	*HSDL2*	Homo sapiens hydroxysteroid dehydrogenase like 2 (HSDL2), transcript variant 1, mRNA [NM_032303]	-0.5312977	0.0072840904
A_23_P81408	*BRD7P3*	Homo sapiens bromodomain containing 7 pseudogene 3 (BRD7P3), non-coding RNA [NR_002730]	-1.3759124	0.0060495283
A_24_P114617	*STAT5B*	Homo sapiens signal transducer and activator of transcription 5B (STAT5B), mRNA [NM_012448]	-0.71618414	0.006295563
A_23_P386411	*MAT2B*	Homo sapiens methionine adenosyltransferase II, beta (MAT2B), transcript variant 2, mRNA [NM_182796]	-0.89031196	0.0056123743
A_24_P295010	*CHMP2B*	Homo sapiens charged multivesicular body protein 2B (CHMP2B), transcript variant 1, mRNA [NM_014043]	-1.137765	0.005612091
A_22_P00013330	*PPID*	Homo sapiens peptidylprolyl isomerase D (PPID), mRNA [NM_005038]	-0.794749	0.005192162
A_23_P60227	*SERPINB9*	Homo sapiens serpin peptidase inhibitor, clade B (ovalbumin), member 9 (SERPINB9), mRNA [NM_004155]	-1.9075221	0.0057647754
A_23_P207280	*lnc-RP11-127H5.1.1-1*	BX088883 Soares_testis_NHT Homo sapiens cDNA clone IMAGp998E183518 ; IMAGE:1393145, mRNA sequence [BX088883]	1.6367161	0.0064965826
A_23_P97860	*CCIN*	Homo sapiens calicin (CCIN), mRNA [NM_005893]	-1.1583202	0.009861905
A_33_P3348239	*APPBP2*	Homo sapiens amyloid beta precursor protein (cytoplasmic tail) binding protein 2 (APPBP2), transcript variant 1, mRNA [NM_006380]	-1.2107561	0.012362548

A_23_P152727	*LIPA*	Homo sapiens lipase A, lysosomal acid, cholesterol esterase (LIPA), transcript variant 2, mRNA [NM_000235]	-1.9180247	0.0028288076
A_24_P282343	*FBN1*	Homo sapiens fibrillin 1 (FBN1), mRNA [NM_000138]	-0.83043766	0.005734989
A_23_P54840	*PLEKHM1*	Homo sapiens pleckstrin homology domain containing, family M (with RUN domain) member 1 (PLEKHM1), transcript variant 1, mRNA [NM_014798]	-0.96868557	0.0118191205
A_23_P39185	*CDKL5*	Homo sapiens cyclin-dependent kinase-like 5 (CDKL5), transcript variant I, mRNA [NM_003159]	-1.3164396	0.0029471128
A_21_P0002684	*MT1A*	Homo sapiens metallothionein 1A (MT1A), mRNA [NM_005946]	1.5275785	0.0035894548
A_23_P65609	*RDH13*	Homo sapiens retinol dehydrogenase 13 (all-trans/9-cis) (RDH13), transcript variant 2, mRNA [NM_138412]	1.0738846	0.0078017237
A_23_P144677	*LINC01116*	Homo sapiens long intergenic non-protein coding RNA 1116 (LINC01116), long non-coding RNA [NR_040001]	-0.7259478	0.011739143
A_23_P32722	*GTF2A1*	Homo sapiens general transcription factor IIA, 1, 19/37kDa (GTF2A1), transcript variant 1, mRNA [NM_015859]	-0.4392696	0.007700591
A_23_P19291	*LNPEP*	leucyl/cystinyl aminopeptidase [Source:HGNC Symbol;Acc:HGNC:6656] [ENST00000231368]	-1.1318841	0.0044240346
A_22_P00014120	*ZNF408*	Homo sapiens zinc finger protein 408 (ZNF408), transcript variant 1, mRNA [NM_024741]	1.3105165	0.010822477
A_33_P3331641	*TUBB2A*	Homo sapiens tubulin, beta 2A class IIa (TUBB2A), mRNA [NM_001069]	-0.86299366	0.0063443403
A_23_P61398	*lnc-SCAP-1*	Homo sapiens cDNA clone IMAGE:5272313. [BC036224]	-1.2830018	0.008947276
A_33_P3235766	*ZNF503-AS2*	Homo sapiens ZNF503 antisense RNA 2 (ZNF503-AS2), transcript variant 5, long non-coding RNA [NR_110301]	-1.5485027	0.0028288076
A_33_P3219870	*PIM3*	Homo sapiens Pim-3 proto-oncogene, serine/threonine kinase (PIM3), mRNA [NM_001001852]	-0.7344758	0.0071432865
A_33_P3287770	*QSER1*	Homo sapiens glutamine and serine rich 1 (QSER1), mRNA [NM_001076786]	-0.5347238	0.011501758
A_24_P113221	*PNPLA2*	Homo sapiens patatin-like phospholipase domain containing 2 (PNPLA2), mRNA [NM_020376]	0.6121864	0.011604
A_23_P94319	*TELO2*	Homo sapiens telomere maintenance 2 (TELO2), mRNA [NM_016111]	0.1321142	0.017046493
A_33_P3319940	*ZNF24*	Homo sapiens zinc finger protein 24 (ZNF24), mRNA [NM_006965]	-0.7511522	0.008454407

A_23_P338952	*KBTBD11*	Homo sapiens kelch repeat and BTB (POZ) domain containing 11 (KBTBD11), mRNA [NM_014867]	-3.9299061	0.0025001846
A_33_P3297562	*ATRX*	Homo sapiens alpha thalassemia/mental retardation syndrome X-linked (ATRX), transcript variant 1, mRNA [NM_000489]	-0.55501956	0.0057806885
A_23_P141479	*S100PBP*	Homo sapiens S100P binding protein (S100PBP), transcript variant 1, mRNA [NM_022753]	-0.85952044	0.010369088
A_23_P30175	*IRX2*	Homo sapiens iroquois homeobox 2 (IRX2), transcript variant 1, mRNA [NM_033267]	-1.9213789	0.0041847117
A_22_P00006917	*SUPT6H*	Homo sapiens suppressor of Ty 6 homolog (S. cerevisiae) (SUPT6H), mRNA [NM_003170]	0.7642107	0.011371365
A_32_P184279	*ERBB2IP*	Homo sapiens erbb2 interacting protein (ERBB2IP), transcript variant 2, mRNA [NM_018695]	-0.7395403	0.0044813035
A_24_P115511	*lnc-GAS6-5*	EST182079 Jurkat T-cells V Homo sapiens cDNA 5' end similar to EST containing repeat, mRNA sequence [AA311247]	1.0332638	0.016991401
A_33_P3219821	*CCDC6*	Homo sapiens coiled-coil domain containing 6 (CCDC6), mRNA [NM_005436]	-1.1355824	0.003417016
A_33_P3246833	*RAB14*	Homo sapiens RAB14, member RAS oncogene family (RAB14), mRNA [NM_016322]	-1.3046545	0.0058361553
A_24_P252497	*FMN2*	Homo sapiens formin 2 (FMN2), mRNA [NM_020066]	-0.81029576	0.014034924
A_23_P203299	*IL1RN*	Homo sapiens interleukin 1 receptor antagonist (IL1RN), transcript variant 4, mRNA [NM_173843]	1.0229528	0.0057417424
A_33_P3281036	*TRIB1*	Homo sapiens tribbles pseudokinase 1 (TRIB1), transcript variant 1, mRNA [NM_025195]	1.3618314	0.0051410845
A_23_P148047	*RCN1*	Homo sapiens reticulocalbin 1, EF-hand calcium binding domain (RCN1), mRNA [NM_002901]	-0.7269669	0.0066194455
A_24_P865	*HCG11*	Homo sapiens HLA complex group 11 (non-protein coding) (HCG11), long non-coding RNA [NR_026790]	0.34026006	0.004993122
A_33_P3416946	*PTGER4*	Homo sapiens prostaglandin E receptor 4 (subtype EP4) (PTGER4), mRNA [NM_000958]	0.8663076	0.008153665
A_33_P3370424	*CEP85L*	Homo sapiens centrosomal protein 85kDa-like (CEP85L), transcript variant 2, mRNA [NM_206921]	-0.802542	0.009222329
A_22_P00017233	*ACTR10*	Homo sapiens actin-related protein 10 homolog (S. cerevisiae) (ACTR10), mRNA [NM_018477]	-0.7129228	0.01477476
A_33_P3344673	*NOTCH1*	Homo sapiens notch 1 (NOTCH1), mRNA [NM_017617]	0.62040496	0.015461617

A_22_P00003244	*ENTPD3-AS1*	Homo sapiens ENTPD3 antisense RNA 1 (ENTPD3-AS1), long non-coding RNA [NR_040100]	-1.0481381	0.0057247286
A_24_P713668	*PPP1R37*	Homo sapiens protein phosphatase 1, regulatory subunit 37 (PPP1R37), mRNA [NM_019121]	0.8201202	0.015448156
A_24_P309594	*lnc-CADPS-1*	LNCipedia lincRNA (lnc-CADPS-1), lincRNA [lnc-CADPS-1:1]	1.2251389	0.0058217365
A_24_P920979	*BZW1*	Homo sapiens basic leucine zipper and W2 domains 1 (BZW1), transcript variant 4, mRNA [NM_014670]	-0.288352	0.0050253686
A_32_P144220	*SLC48A1*	Homo sapiens solute carrier family 48 (heme transporter), member 1 (SLC48A1), mRNA [NM_017842]	-0.61400175	0.0075928494
A_33_P3409886	*ASB7*	Homo sapiens ankyrin repeat and SOCS box containing 7 (ASB7), transcript variant 2, mRNA [NM_198243]	-0.9654239	0.0053643263
A_24_P245298	*VAMP2*	Homo sapiens vesicle-associated membrane protein 2 (synaptobrevin 2) (VAMP2), mRNA [NM_014232]	1.0236475	0.009630889
A_19_P00320729	*THAP2*	Homo sapiens THAP domain containing, apoptosis associated protein 2 (THAP2), mRNA [NM_031435]	-1.3411595	0.0043846127
A_21_P0009134	*TNFSF12*	Homo sapiens tumor necrosis factor (ligand) superfamily, member 12 (TNFSF12), transcript variant 1, mRNA [NM_003809]	-1.5386267	0.005852893
A_23_P101054	*CDYL2*	chromodomain protein, Y-like 2 [Source:HGNC Symbol;Acc:HGNC:23030] [ENST00000570137]	-0.9484632	0.0036517929
A_23_P216108	*lnc-CLEC18B-1*	LNCipedia lincRNA (lnc-CLEC18B-1), lincRNA [lnc-CLEC18B-1:1]	1.0523206	0.0068771048
A_23_P74653	*KRT34*	Homo sapiens keratin 34, type I (KRT34), mRNA [NM_021013]	-3.660894	0.0029691157
A_33_P3377851	*ANK1*	Homo sapiens ankyrin 1, erythrocytic (ANK1), transcript variant 3, mRNA [NM_000037]	1.4475679	0.005947757
A_33_P3286318	*NUDC*	Homo sapiens nudC nuclear distribution protein (NUDC), mRNA [NM_006600]	0.45944074	0.011568039
A_23_P166698	*PNMAL2*	Homo sapiens paraneoplastic Ma antigen family-like 2 (PNMAL2), mRNA [NM_020709]	-1.5543123	0.013991326
A_32_P192823	*SS18L2*	Homo sapiens synovial sarcoma translocation gene on chromosome 18-like 2 (SS18L2), mRNA [NM_016305]	-0.5658625	0.011140108
A_24_P237878	*CXCL6*	Homo sapiens chemokine (C-X-C motif) ligand 6 (CXCL6), mRNA [NM_002993]	0.94882256	0.0048114373
A_33_P3365805	*PRPS1L1*	Homo sapiens phosphoribosyl pyrophosphate synthetase 1-like 1 (PRPS1L1), mRNA [NM_175886]	-1.8223423	0.00431082

A_23_P120442	*FAM195B*	Homo sapiens family with sequence similarity 195, member B (FAM195B), transcript variant 1, mRNA [NM_207368]	-1.1166482	0.005098191
A_23_P431268	*CLN5*	Homo sapiens ceroid-lipofuscinosis, neuronal 5 (CLN5), mRNA [NM_006493]	-0.5897156	0.016888179
A_24_P212443	*NCOA3*	Homo sapiens nuclear receptor coactivator 3 (NCOA3), transcript variant 1, mRNA [NM_181659]	-0.6406299	0.008208044
A_22_P00006069	*PLEKHA6*	Homo sapiens pleckstrin homology domain containing, family A member 6 (PLEKHA6), mRNA [NM_014935]	1.549702	0.0029071842
A_21_P0012376	*UBE3C*	Homo sapiens ubiquitin protein ligase E3C (UBE3C), mRNA [NM_014671]	0.73562664	0.003363001
A_22_P00018344	*lnc-FAM13B-1*	601905253F1 NIH_MGC_54 Homo sapiens cDNA clone IMAGE:4133101 5', mRNA sequence [BF238994]	0.8736607	0.0140423635
A_23_P144916	*GEMIN2*	Homo sapiens gem (nuclear organelle) associated protein 2 (GEMIN2), transcript variant gamma, mRNA [NM_001009183]	-0.9122523	0.0076747234
A_33_P3276718	*GFPT2*	Homo sapiens glutamine-fructose-6-phosphate transaminase 2 (GFPT2), mRNA [NM_005110]	-1.268719	0.007304192
A_22_P00022873	*NPC2*	Homo sapiens Niemann-Pick disease, type C2 (NPC2), mRNA [NM_006432]	-1.1006019	0.0054810056
A_23_P330419	*HGF*	Homo sapiens hepatocyte growth factor (hepapoietin A; scatter factor) (HGF), transcript variant 5, mRNA [NM_001010934]	5.378997	0.002535607
A_24_P181149	*LOC102724532*	PREDICTED: Homo sapiens uncharacterized LOC102724532 (LOC102724532), transcript variant X1, ncRNA [XR_424843]	-1.2023602	0.004026994
A_33_P3407400	*ACPT*	Homo sapiens acid phosphatase, testicular (ACPT), mRNA [NM_033068]	0.781084	0.006901566
A_33_P3502315	*BBS10*	Homo sapiens Bardet-Biedl syndrome 10 (BBS10), mRNA [NM_024685]	-1.5403864	0.007092758
A_33_P3775741	*SPEF2*	Homo sapiens sperm flagellar 2 (SPEF2), transcript variant 2, mRNA [NM_144722]	-1.4851402	0.0034903844
A_24_P49517	*CRLS1*	Homo sapiens cardiolipin synthase 1 (CRLS1), transcript variant 1, mRNA [NM_019095]	-0.93700194	0.0034766807
A_23_P34788	*RPL32P3*	Homo sapiens ribosomal protein L32 pseudogene 3 (RPL32P3), non-coding RNA [NR_003111]	-0.39044765	0.0054271906
A_21_P0012430	*ZNF251*	Homo sapiens zinc finger protein 251 (ZNF251), mRNA [NM_138367]	-1.0160584	0.008273267
A_33_P3849275	*KIF2C*	Homo sapiens kinesin family member 2C (KIF2C), transcript variant 1, mRNA [NM_006845]	-0.637995	0.013654007
A_33_P3295423	*LOC101930159*	PREDICTED: Homo sapiens uncharacterized LOC101930159 (LOC101930159), ncRNA [XR_251533]	-0.83945704	0.009480673

A_23_P157117	*FHL1*	Homo sapiens four and a half LIM domains 1 (FHL1), transcript variant 4, mRNA [NM_001159704]	-1.6187539	0.00477571
A_22_P00000650	*ZXDC*	Homo sapiens ZXD family zinc finger C (ZXDC), transcript variant 2, mRNA [NM_001040653]	-0.43437865	0.013746894
A_33_P3235400	*CREB5*	Homo sapiens cAMP responsive element binding protein 5 (CREB5), transcript variant 1, mRNA [NM_182898]	-1.8585448	0.009334625
A_24_P344711	*lnc-ACSS3-2*	LNCipedia lincRNA (lnc-ACSS3-2), lincRNA [lnc-ACSS3-2:1]	0.68289787	0.007797447
A_23_P98631	*HDGF*	Homo sapiens hepatoma-derived growth factor (HDGF), transcript variant 1, mRNA [NM_004494]	1.4156276	0.0134437205
A_24_P35169	*AGPAT3*	Homo sapiens 1-acylglycerol-3-phosphate O-acyltransferase 3 (AGPAT3), transcript variant 1, mRNA [NM_020132]	0.5183117	0.012485394
A_19_P00320047	*HPS5*	Homo sapiens Hermansky-Pudlak syndrome 5 (HPS5), transcript variant 1, mRNA [NM_181507]	-0.8454528	0.015843993
A_33_P3224105	*GATAD1*	Homo sapiens GATA zinc finger domain containing 1 (GATAD1), transcript variant 1, mRNA [NM_021167]	-0.7035496	0.0075455266
A_33_P3320953	*lnc-AC069257.9.1-4*	LNCipedia lincRNA (lnc-AC069257.9.1-4), lincRNA [lnc-AC069257.9.1-4:16]	0.5184206	0.01482335
A_21_P0014514	*KNSTRN*	Homo sapiens kinetochore-localized astrin/SPAG5 binding protein (KNSTRN), transcript variant 2, mRNA [NM_001142761]	-0.69241136	0.008122174
A_23_P314755	*CTXN1*	Homo sapiens cortexin 1 (CTXN1), mRNA [NM_206833]	1.3585296	0.0072456384
A_23_P41872	*STC1*	Homo sapiens stanniocalcin 1 (STC1), mRNA [NM_003155]	0.99348557	0.007327463
A_21_P0000341	*ALKBH8*	Homo sapiens alkB, alkylation repair homolog 8 (E. coli) (ALKBH8), transcript variant 2, mRNA [NM_138775]	-0.68856174	0.0065241847
A_33_P3303380	*B4GALT7*	Homo sapiens xylosylprotein beta 1,4-galactosyltransferase, polypeptide 7 (B4GALT7), mRNA [NM_007255]	0.62558335	0.014758866
A_23_P144578	*SNORA61*	Homo sapiens small nucleolar RNA, H/ACA box 61 (SNORA61), small nucleolar RNA [NR_002987]	-0.64532906	0.013453215
A_33_P3327592	*PARD3*	par-3 family cell polarity regulator [Source:HGNC Symbol;Acc:HGNC:16051] [ENST00000374768]	0.8522859	0.008947276
A_23_P323924	*GNPDA2*	Homo sapiens glucosamine-6-phosphate deaminase 2 (GNPDA2), transcript variant 1, mRNA [NM_138335]	-1.1113942	0.005014313

A_33_P3369286	*RNF13*	Homo sapiens ring finger protein 13 (RNF13), transcript variant 1, mRNA [NM_007282]	-0.70971847	0.004935436
A_23_P119353	*ZFYVE27*	Homo sapiens zinc finger, FYVE domain containing 27 (ZFYVE27), transcript variant 1, mRNA [NM_001002261]	-0.8821099	0.0075611626
A_32_P96036	*IPO8*	Homo sapiens importin 8 (IPO8), transcript variant 1, mRNA [NM_006390]	-0.6281751	0.00371792
A_33_P3298960	*RASIP1*	Homo sapiens Ras interacting protein 1 (RASIP1), mRNA [NM_017805]	-1.7573324	0.004829709
A_33_P3351524	*MEX3A*	Homo sapiens mex-3 RNA binding family member A (MEX3A), mRNA [NM_001093725]	-0.91935325	0.008553962
A_33_P3392698	*KDELC2*	Homo sapiens KDEL (Lys-Asp-Glu-Leu) containing 2 (KDELC2), mRNA [NM_153705]	-0.5869994	0.010308166
A_23_P31747	*SPIN4*	Homo sapiens spindlin family, member 4 (SPIN4), mRNA [NM_001012968]	0.49225172	0.005800329
A_23_P162211	*PIGK*	Homo sapiens phosphatidylinositol glycan anchor biosynthesis, class K (PIGK), mRNA [NM_005482]	-1.1176989	0.0060000643
A_23_P25403	*C8orf76*	Homo sapiens chromosome 8 open reading frame 76 (C8orf76), mRNA [NM_032847]	-0.7855263	0.008559404
A_22_P00011386	*MANSC1*	Homo sapiens MANSC domain containing 1 (MANSC1), mRNA [NM_018050]	-1.9322906	0.011376762
A_23_P67271	*HCFC2*	Homo sapiens host cell factor C2 (HCFC2), mRNA [NM_013320]	-1.029587	0.0031941293
A_22_P00003430	*lnc-P4HA2-2*	Homo sapiens mRNA; cDNA DKFZp779A135 (from clone DKFZp779A135). [BX538252]	0.4063253	0.005512448
A_23_P154108	*PKN1*	Homo sapiens protein kinase N1 (PKN1), transcript variant 2, mRNA [NM_002741]	1.4212227	0.006372418
A_24_P677634	*lnc-CCDC33-2*	AGENCOURT_13620477 NIH_MGC_148 Homo sapiens cDNA clone IMAGE:30335914 5', mRNA sequence [CB994296]	-0.48714253	0.011745691
A_21_P0004420	*DYNC1I2*	Homo sapiens dynein, cytoplasmic 1, intermediate chain 2 (DYNC1I2), transcript variant 1, mRNA [NM_001378]	-0.70087814	0.006650803
A_24_P134319	*LOC493754*	Homo sapiens RAB guanine nucleotide exchange factor (GEF) 1 pseudogene (LOC493754), transcript variant 6, non-coding RNA [NR_111977]	-0.45150304	0.011824575
A_21_P0014186	*lnc-ARRDC3-1*	LNCipedia lincRNA (lnc-ARRDC3-1), lincRNA [lnc-ARRDC3-1:14]	1.4086612	0.0056601833
A_23_P101737	*ADNP*	Homo sapiens activity-dependent neuroprotector homeobox (ADNP), transcript variant 3, mRNA [NM_001282531]	-0.69462323	0.010827251
A_23_P55011	*LOC101927811*	Homo sapiens uncharacterized LOC101927811 (LOC101927811), transcript variant 1, long non-coding RNA [NR_110119]	-1.1408771	0.010606176

A_23_P406986	*DOT1L*	Homo sapiens DOT1-like histone H3K79 methyltransferase (DOT1L), mRNA [NM_032482]	1.0158526	0.008427824
A_24_P137434	*SLC38A10*	Homo sapiens solute carrier family 38, member 10 (SLC38A10), transcript variant 1, mRNA [NM_001037984]	-0.80687976	0.0077546863
A_23_P428248	*MOSPD2*	Homo sapiens motile sperm domain containing 2 (MOSPD2), transcript variant 1, mRNA [NM_152581]	-0.7595737	0.0027248112
A_33_P3352349	*DCBLD2*	Homo sapiens discoidin, CUB and LCCL domain containing 2 (DCBLD2), mRNA [NM_080927]	0.91898155	0.0028630516
A_23_P166051	*TTC21A*	Homo sapiens tetratricopeptide repeat domain 21A (TTC21A), transcript variant 2, mRNA [NM_145755]	-2.0793297	0.0026564114
A_21_P0008215	*KRTAP9-1*	Homo sapiens keratin associated protein 9-1 (KRTAP9-1), mRNA [NM_001190460]	1.380342	0.0022181747
A_24_P171058	*RBCK1*	Homo sapiens RanBP-type and C3HC4-type zinc finger containing 1 (RBCK1), transcript variant 2, mRNA [NM_031229]	1.0224137	0.004160909
A_23_P69058	*LOC101927712*	PREDICTED: Homo sapiens uncharacterized LOC101927712 (LOC101927712), ncRNA [XR_243074]	-0.95473075	0.0102659585
A_24_P186346	*TMEM64*	Homo sapiens transmembrane protein 64 (TMEM64), transcript variant 1, mRNA [NM_001008495]	-0.51516396	0.0033094941
A_24_P269062	*MLH1*	Homo sapiens mutL homolog 1 (MLH1), transcript variant 1, mRNA [NM_000249]	-0.6307883	0.0071605598
A_33_P3211423	*ANAPC1*	Homo sapiens anaphase promoting complex subunit 1 (ANAPC1), mRNA [NM_022662]	-0.74352616	0.011383506
A_24_P942630	*SPRY4*	Homo sapiens sprouty homolog 4 (Drosophila) (SPRY4), transcript variant 1, mRNA [NM_030964]	2.360104	0.0053998437
A_23_P11859	*PANK3*	pantothenate kinase 3 [Source:HGNC Symbol;Acc:HGNC:19365] [ENST00000239231]	-0.89173985	0.0070736636
A_33_P3216297	*KDM6B*	Homo sapiens lysine (K)-specific demethylase 6B (KDM6B), mRNA [NM_001080424]	1.4439983	0.005632443
A_23_P116037	*HSD17B7*	Homo sapiens hydroxysteroid (17-beta) dehydrogenase 7 (HSD17B7), mRNA [NM_016371]	-0.81268555	0.009410995
A_23_P37283	*NR3C1*	Homo sapiens nuclear receptor subfamily 3, group C, member 1 (glucocorticoid receptor) (NR3C1), transcript variant 5, mRNA [NM_001018077]	-1.0819914	0.0073443917
A_22_P00015794	*TM7SF2*	Homo sapiens transmembrane 7 superfamily member 2 (TM7SF2), transcript variant 1, mRNA [NM_003273]	1.2985474	0.0031941293

A_33_P3282898	*CGRRF1*	Homo sapiens cell growth regulator with ring finger domain 1 (CGRRF1), mRNA [NM_006568]	-0.89839286	0.010645894
A_23_P406438	*lnc-TARDBP-1*	Homo sapiens cDNA FLJ30609 fis, clone CTONG2000480. [AK055171]	0.40053707	0.0059821224
A_33_P3272461	*TLN1*	talin 1 [Source:HGNC Symbol;Acc:HGNC:11845] [ENST00000378192]	-1.4111191	0.0059623835
A_24_P76078	*SRPK2*	Homo sapiens SRSF protein kinase 2 (SRPK2), transcript variant 2, mRNA [NM_182691]	-1.1103541	0.0038736917
A_33_P6471837	*METTL5*	Homo sapiens methyltransferase like 5 (METTL5), transcript variant 1, mRNA [NM_014168]	-0.4502048	0.006104359
A_23_P59202	*LBX2-AS1*	Homo sapiens LBX2 antisense RNA 1 (LBX2-AS1), long non-coding RNA [NR_024606]	1.4171855	0.011091811
A_33_P3394769	*SIKE1*	Homo sapiens suppressor of IKBKE 1 (SIKE1), transcript variant 1, mRNA [NM_001102396]	-0.93989515	0.0076952893
A_23_P149813	*TAF11*	Homo sapiens TAF11 RNA polymerase II, TATA box binding protein (TBP)-associated factor, 28kDa (TAF11), transcript variant 1, mRNA [NM_005643]	-0.6736292	0.005015031
A_23_P20876	*TMEM8C*	Homo sapiens transmembrane protein 8C (TMEM8C), mRNA [NM_001080483]	0.9912932	0.0037583082
A_24_P380679	*RSU1*	Homo sapiens Ras suppressor protein 1 (RSU1), transcript variant 1, mRNA [NM_012425]	-1.0001097	0.012053236
A_21_P0000116	*PTPDC1*	Homo sapiens protein tyrosine phosphatase domain containing 1 (PTPDC1), transcript variant 2, mRNA [NM_177995]	-0.5070553	0.010801365
A_33_P3284854	*LSMEM1*	Homo sapiens leucine-rich single-pass membrane protein 1 (LSMEM1), transcript variant 1, mRNA [NM_182597]	-1.88193	0.004763325
A_23_P120002	*MID1*	Homo sapiens midline 1 (MID1), transcript variant 6, mRNA [NM_001193278]	-0.88424337	0.005925039
A_33_P3335124	*SP110*	Homo sapiens SP110 nuclear body protein (SP110), transcript variant b, mRNA [NM_004510]	-0.73581576	0.0077072685
A_23_P103864	*SFSWAP*	Homo sapiens splicing factor, suppressor of white-apricot family (SFSWAP), transcript variant 2, mRNA [NM_004592]	-0.74811643	0.007300493
A_23_P435407	*RPS2*	Homo sapiens ribosomal protein S2 (RPS2), mRNA [NM_002952]	-0.8145749	0.0086045945
A_24_P171873	*TTC13*	Homo sapiens tetratricopeptide repeat domain 13 (TTC13), transcript variant 1, mRNA [NM_024525]	-0.77612793	0.0036759654
A_24_P303420	*GPC4*	Homo sapiens glypican 4 (GPC4), mRNA [NM_001448]	-4.0099764	0.0043846127
A_24_P941148	*FBXO4*	Homo sapiens F-box protein 4 (FBXO4), transcript variant 1, mRNA [NM_012176]	-1.1373084	0.0031941293

A_24_P111511	*ADCY10P1*	Homo sapiens adenylate cyclase 10 (soluble) pseudogene 1 (ADCY10P1), non-coding RNA [NR_026938]	-0.49302217	0.016854454
A_33_P3300975	*HAUS6*	Homo sapiens HAUS augmin-like complex, subunit 6 (HAUS6), transcript variant 1, mRNA [NM_017645]	-0.67146635	0.006515337
A_21_P0013960	*TTC21B*	Homo sapiens tetratricopeptide repeat domain 21B (TTC21B), mRNA [NM_024753]	-0.78672975	0.0069220536
A_33_P3260669	*HOXC4*	Homo sapiens homeobox C4 (HOXC4), transcript variant 1, mRNA [NM_014620]	-1.5774784	0.003728358
A_33_P3384775	*LOC101930246*	PREDICTED: Homo sapiens uncharacterized LOC101930246 (LOC101930246), transcript variant X2, ncRNA [XR_250239]	0.81102705	0.009195471
A_33_P3414122	*STK3*	Homo sapiens serine/threonine kinase 3 (STK3), transcript variant 2, mRNA [NM_001256312]	-1.0850585	0.010822477
A_24_P388786	*ZBTB21*	zinc finger and BTB domain containing 21 [Source:HGNC Symbol;Acc:HGNC:13083] [ENST00000398497]	2.511331	0.0037583082
A_33_P3309429	*ZNF260*	Homo sapiens zinc finger protein 260 (ZNF260), transcript variant 1, mRNA [NM_001012756]	-0.55831665	0.008303243
A_23_P213093	*DNAH5*	Homo sapiens dynein, axonemal, heavy chain 5 (DNAH5), mRNA [NM_001369]	1.0959523	0.0044240346
A_23_P43690	*NUPL2*	nucleoporin like 2 [Source:HGNC Symbol;Acc:HGNC:17010] [ENST00000477844]	2.6109595	0.0041383547
A_23_P73208	*OCIAD1*	Homo sapiens OCIA domain containing 1 (OCIAD1), transcript variant 2, mRNA [NM_001079839]	-0.5965137	9,05E+03
A_21_P0012186	*BNC2*	Homo sapiens basonuclin 2 (BNC2), mRNA [NM_017637]	0.9557629	0.0072456384
A_23_P307955	*GABPB2*	Homo sapiens GA binding protein transcription factor, beta subunit 2 (GABPB2), mRNA [NM_144618]	-1.0189092	0.004659113
A_21_P0014327	*XLOC_l2_009139*	BROAD Institute lincRNA (XLOC_l2_009139), lincRNA [TCONS_l2_00017151]	-0.57405734	0.011153647
A_23_P359588	*SIRT5*	Homo sapiens sirtuin 5 (SIRT5), transcript variant 2, mRNA [NM_031244]	-0.74823725	0.010455912
A_22_P00021933	*FTO-IT1*	Homo sapiens FTO intronic transcript 1 (non-protein coding) (FTO-IT1), long non-coding RNA [NR_103838]	-0.5163613	0.011694485
A_22_P00011429	*PCDHGB4*	Homo sapiens protocadherin gamma subfamily B, 4 (PCDHGB4), transcript variant 2, mRNA [NM_032098]	-0.97897565	0.0074182227
A_23_P204579	*P4HA2*	Homo sapiens prolyl 4-hydroxylase, alpha polypeptide II (P4HA2), transcript variant 1, mRNA [NM_004199]	-1.117733	0.01089527

A_24_P148094	*GLIS2-AS1*	Homo sapiens GLIS2 antisense RNA 1 (GLIS2-AS1), long non-coding RNA [NR_110901]	-2.167139	0.0070324177
A_33_P3271945	*TDG*	Homo sapiens thymine-DNA glycosylase (TDG), mRNA [NM_003211]	-1.0701425	0.0052430327
A_23_P71094	*LEPROT*	Homo sapiens leptin receptor overlapping transcript (LEPROT), transcript variant 1, mRNA [NM_017526]	-0.9992604	0.004804688
A_24_P414371	*HAUS6*	HAUS augmin-like complex, subunit 6 [Source:HGNC Symbol;Acc:HGNC:25948] [ENST00000380496]	-0.66299754	0.0059353677
A_32_P49848	*GCC1*	Homo sapiens GRIP and coiled-coil domain containing 1 (GCC1), mRNA [NM_024523]	-0.7963876	0.008298705
A_21_P0007548	*PPP3CA*	Homo sapiens protein phosphatase 3, catalytic subunit, alpha isozyme (PPP3CA), transcript variant 1, mRNA [NM_000944]	-0.7095482	0.007843468
A_23_P62276	*RHOQ*	Homo sapiens ras homolog family member Q (RHOQ), mRNA [NM_012249]	-15110843	0.0034684502
A_32_P94660	*lnc-FAM113B-5*	LNCipedia lincRNA (lnc-FAM113B-5), lincRNA [lnc-FAM113B-5:1]	1.1513128	0.0073130694
A_33_P3289696	*NKAP*	Homo sapiens NFKB activating protein (NKAP), mRNA [NM_024528]	-0.79596066	0.009576729
A_22_P00020375	*LRRC37B*	Homo sapiens leucine rich repeat containing 37B (LRRC37B), mRNA [NM_052888]	-0.51935285	0.014970456
A_23_P91590	*OSCP1*	Homo sapiens organic solute carrier partner 1 (OSCP1), transcript variant 1, mRNA [NM_145047]	-0.9133636	0.010194437
A_32_P226786	*ZNF192P1*	Homo sapiens zinc finger protein 192 pseudogene 1 (ZNF192P1), non-coding RNA [NR_103448]	-1.1583284	0.0069803307
A_33_P3216292	*RANBP1*	Homo sapiens RAN binding protein 1 (RANBP1), transcript variant 2, mRNA [NM_002882]	-0.20081826	0.004454897
A_23_P57760	*FAM126B*	Homo sapiens family with sequence similarity 126, member B (FAM126B), mRNA [NM_173822]	-1.1629083	0.003557021
A_33_P3377261	*TBC1D2*	Homo sapiens TBC1 domain family, member 2 (TBC1D2), transcript variant 1, mRNA [NM_001267571]	-0.8996351	0.009792738
A_19_P00322058	*PXYLP1*	Homo sapiens 2-phosphoxylose phosphatase 1 (PXYLP1), transcript variant 1, mRNA [NM_152282]	-2.3275926	0.0029471128
A_23_P8961	*RNF7*	Homo sapiens ring finger protein 7 (RNF7), transcript variant 1, mRNA [NM_014245]	-0.46357277	0.01042595
A_21_P0001704	*LINC01013*	Homo sapiens long intergenic non-protein coding RNA 1013 (LINC01013), long non-coding RNA [NR_038981]	-4.1917105	0.012911957
A_33_P3397655	*IL7*	Homo sapiens interleukin 7 (IL7), transcript variant 1, mRNA [NM_000880]	-1.199695	0.008702064

A_33_P6821973	*lnc-NBPF16-4*	LNCipedia lincRNA (lnc-NBPF16-4), lincRNA [lnc-NBPF16-4:2]	-0.5804555	0.014244717
A_23_P39364	*SYNPO*	Homo sapiens synaptopodin (SYNPO), transcript variant 3, mRNA [NM_001166208]	0.64987814	0.013250718
A_24_P273157	*PCBP1-AS1*	Homo sapiens PCBP1 antisense RNA 1 (PCBP1-AS1), long non-coding RNA [NR_033872]	-0.9931759	0.010795945
A_24_P414256	*HOMER3*	Homo sapiens homer homolog 3 (Drosophila) (HOMER3), transcript variant 2, mRNA [NM_004838]	-0.5148162	0.010894719
A_24_P306561	*OBSCN*	Homo sapiens obscurin, cytoskeletal calmodulin and titin-interacting RhoGEF (OBSCN), transcript variant 1, mRNA [NM_052843]	0.7755283	0.01581842
A_23_P306215	*TMA7*	translation machinery associated 7 homolog (S. cerevisiae) [Source:HGNC Symbol;Acc:HGNC:26932] [ENST00000477624]	-0.6791895	0.004763325
A_33_P3272948	*TCF25*	Homo sapiens transcription factor 25 (basic helix-loop-helix) (TCF25), mRNA [NM_014972]	-0.9686668	0.0056444393
A_33_P3883985	*FAM84A*	Homo sapiens family with sequence similarity 84, member A (FAM84A), mRNA [NM_145175]	1.144437	0.0069564725
A_33_P3404651	*C17orf107*	Homo sapiens chromosome 17 open reading frame 107 (C17orf107), mRNA [NM_001145536]	-1.7137004	0.004026994
A_24_P103686	*LMF1*	Homo sapiens lipase maturation factor 1 (LMF1), transcript variant 4, non-coding RNA [NR_036442]	1.7142649	0.008106991
A_33_P3373203	*TTC7A*	Homo sapiens tetratricopeptide repeat domain 7A (TTC7A), transcript variant 1, mRNA [NM_001288951]	-0.8762772	0.008679666
A_24_P589266	*AGPAT6*	Homo sapiens 1-acylglycerol-3-phosphate O-acyltransferase 6 (AGPAT6), mRNA [NM_178819]	0.5594732	0.0118191205
A_33_P3256560	*SH3RF3*	Homo sapiens SH3 domain containing ring finger 3 (SH3RF3), mRNA [NM_001099289]	0.8922174	0.015991567
A_33_P3365437	*ATP9A*	Homo sapiens ATPase, class II, type 9A (ATP9A), mRNA [NM_006045]	-1.3660994	0.004626585
A_23_P300728	*ZER1*	Homo sapiens zyg-11 related, cell cycle regulator (ZER1), mRNA [NM_006336]	-1.0018489	0.008216877
A_23_P363647	*LHX9*	Homo sapiens LIM homeobox 9 (LHX9), transcript variant 1, mRNA [NM_020204]	-1.6783924	0.007986457
A_33_P3355944	*LMBR1*	Homo sapiens limb development membrane protein 1 (LMBR1), mRNA [NM_022458]	-0.5621669	0.016474942
A_23_P139704	*DDX26B*	Homo sapiens DEAD/H (Asp-Glu-Ala-Asp/His) box polypeptide 26B (DDX26B), mRNA [NM_182540]	-0.66131717	0.0034684502
A_23_P164237	*LINC00908*	long intergenic non-protein coding RNA 908 [Source:HGNC Symbol;Acc:HGNC:27599] [ENST00000582452]	0.9981655	0.011772038

A_33_P3313622	*DUSP6*	Homo sapiens dual specificity phosphatase 6 (DUSP6), transcript variant 1, mRNA [NM_001946]	0.42770836	0.009332474
A_23_P131676	*UTP6*	Homo sapiens UTP6, small subunit (SSU) processome component, homolog (yeast) (UTP6), mRNA [NM_018428]	-0.7383997	0.0035470822
A_24_P570049	*MIR17HG*	Homo sapiens miR-17-92 cluster host gene (non-protein coding) (MIR17HG), transcript variant 1, long non-coding RNA [NR_027350]	0.8535708	0.008356209
A_24_P271527	*ACKR3*	Homo sapiens atypical chemokine receptor 3 (ACKR3), mRNA [NM_020311]	-2.2349818	0.005306498
A_22_P00015933	*PPARA*	Homo sapiens peroxisome proliferator-activated receptor alpha (PPARA), transcript variant 5, mRNA [NM_005036]	-0.7963056	0.010871661
A_23_P142022	*JOSD1*	Homo sapiens Josephin domain containing 1 (JOSD1), mRNA [NM_014876]	-0.74899197	0.00746454
A_23_P256342	*LOC101929132*	PREDICTED: Homo sapiens uncharacterized LOC101929132 (LOC101929132), transcript variant X1, ncRNA [XR_247847]	-1.0949649	0.0035826967
A_24_P363005	*PRPF31*	Homo sapiens pre-mRNA processing factor 31 (PRPF31), mRNA [NM_015629]	0.6996424	0.0072215945
A_21_P0007030	*SNX13*	Homo sapiens sorting nexin 13 (SNX13), mRNA [NM_015132]	-1.0130157	0.0043220706
A_24_P391853	*UBE2D3*	Homo sapiens ubiquitin-conjugating enzyme E2D 3 (UBE2D3), transcript variant 2, mRNA [NM_181886]	-0.48348406	0.004026994
A_23_P124486	*lnc-UROS-1*	LNCipedia lincRNA (lnc-UROS-1), lincRNA [lnc-UROS-1:1]	0.35438162	0.0140423635
A_33_P3242483	*PTPN9*	Homo sapiens protein tyrosine phosphatase, non-receptor type 9 (PTPN9), mRNA [NM_002833]	0.6082961	0.005057235
A_24_P294719	*ARHGAP5*	Homo sapiens Rho GTPase activating protein 5 (ARHGAP5), transcript variant 1, mRNA [NM_001030055]	-0.88554585	0.0070736636
A_33_P3315634	*KIDINS220*	Homo sapiens kinase D-interacting substrate, 220kDa (KIDINS220), mRNA [NM_020738]	-1.0511734	0.0031166414
A_23_P91829	*FAF2*	Homo sapiens Fas associated factor family member 2 (FAF2), mRNA [NM_014613]	-0.5904622	0.008488166
A_32_P198731	*DCBLD2*	Homo sapiens discoidin, CUB and LCCL domain containing 2 (DCBLD2), mRNA [NM_080927]	1.1277286	0.003614092
A_23_P130811	*NEURL1B*	Homo sapiens neuralized E3 ubiquitin protein ligase 1B (NEURL1B), mRNA [NM_001142651]	-1.2109504	0.0075457073
A_24_P658584	*NHSL1*	Homo sapiens NHS-like 1 (NHSL1), transcript variant 1, mRNA [NM_020464]	1.013449	0.0057647754
A_23_P118834	*ZNF536*	Homo sapiens zinc finger protein 536 (ZNF536), mRNA [NM_014717]	1.1418277	0.01251812
A_33_P3311076	*SASH1*	Homo sapiens SAM and SH3 domain containing 1 (SASH1), mRNA [NM_015278]	-1.0134358	0.0028703364

A_23_P164536	*TOP2A*	Homo sapiens topoisomerase (DNA) II alpha 170kDa (TOP2A), mRNA [NM_001067]	-0.5832378	0.005622158
A_33_P3214874	*CYB5A*	Homo sapiens cytochrome b5 type A (microsomal) (CYB5A), transcript variant 3, mRNA [NM_001190807]	-0.86515063	0.009765215
A_24_P178963	*PIK3C3*	Homo sapiens phosphatidylinositol 3-kinase, catalytic subunit type 3 (PIK3C3), mRNA [NM_002647]	-0.83616066	0.008749787
A_24_P213228	*KPNA6*	Homo sapiens karyopherin alpha 6 (importin alpha 7) (KPNA6), mRNA [NM_012316]	-0.6117556	0.009182542
A_23_P419239	*TCEANC2*	Homo sapiens transcription elongation factor A (SII) N-terminal and central domain containing 2 (TCEANC2), mRNA [NM_153035]	0.3696164	0.010986003
A_33_P3352134	*LYPLA1*	Homo sapiens lysophospholipase I (LYPLA1), transcript variant 1, mRNA [NM_006330]	-0.7356086	0.005712846
A_22_P00021963	*ETNK1*	Homo sapiens ethanolamine kinase 1 (ETNK1), transcript variant 1, mRNA [NM_018638]	-0.25281006	0.013080299
A_23_P45913	*ATG16L1*	Homo sapiens autophagy related 16-like 1 (S. cerevisiae) (ATG16L1), transcript variant 1, mRNA [NM_030803]	0.6393925	0.0140619585
A_24_P299996	*lnc-NLRP12-2*	LNCipedia lincRNA (lnc-NLRP12-2), lincRNA [lnc-NLRP12-2:1]	-0.92100906	0.005659222
A_23_P112201	*SNRNP40*	Homo sapiens small nuclear ribonucleoprotein 40kDa (U5) (SNRNP40), mRNA [NM_004814]	0.2251889	0.016152125
A_23_P205997	*SHQ1*	Homo sapiens SHQ1, H/ACA ribonucleoprotein assembly factor (SHQ1), mRNA [NM_018130]	-0.38210693	0.010062054
A_23_P256965	*KDM4C*	Homo sapiens lysine (K)-specific demethylase 4C (KDM4C), transcript variant 1, mRNA [NM_015061]	-1.0884025	0.0037926938
A_32_P149060	*APH1B*	Homo sapiens APH1B gamma secretase subunit (APH1B), transcript variant 1, mRNA [NM_031301]	-0.86680716	0.0075317346
A_24_P136641	*FAM167A-AS1*	Homo sapiens FAM167A antisense RNA 1 (FAM167A-AS1), long non-coding RNA [NR_026814]	0.85912603	0.011066167
A_33_P3302354	*LINC00515*	Homo sapiens long intergenic non-protein coding RNA 515 (LINC00515), long non-coding RNA [NR_024092]	-0.2639262	0.009429358
A_21_P0005656	*TOPORS-AS1*	Homo sapiens TOPORS antisense RNA 1 (TOPORS-AS1), transcript variant 1, long non-coding RNA [NR_033991]	-1.000126	0.011566
A_23_P159027	*CABIN1*	Homo sapiens calcineurin binding protein 1 (CABIN1), transcript variant 1, mRNA [NM_001199281]	1.290174	0.0029691157
A_24_P152635	*LOC100506990*	Homo sapiens uncharacterized LOC100506990 (LOC100506990), transcript variant 2, long non-coding RNA [NR_040092]	-1.3104941	0.008908852

A_23_P210210	*ZNF521*	Homo sapiens zinc finger protein 521 (ZNF521), mRNA [NM_015461]	0.64842343	0.00847175
A_23_P388433	*TMX2*	Homo sapiens thioredoxin-related transmembrane protein 2 (TMX2), transcript variant 1, mRNA [NM_015959]	0.6662707	0.010714154
A_23_P324754	*EPAS1*	Homo sapiens endothelial PAS domain protein 1 (EPAS1), mRNA [NM_001430]	-0.57227254	0.015427458
A_32_P206839	*C4orf3*	Homo sapiens chromosome 4 open reading frame 3 (C4orf3), transcript variant 1, mRNA [NM_001170330]	-1.0728893	0.009630487
A_24_P241792	*CEMIP*	Homo sapiens cell migration inducing protein, hyaluronan binding (CEMIP), transcript variant 3, mRNA [NM_018689]	-1.388465	0.003969615
A_33_P3223648	*LOC100288911*	Homo sapiens uncharacterized LOC100288911 (LOC100288911), long non-coding RNA [NR_037631]	-1.1172485	0.009240182
A_32_P11471	*ZNF160*	Homo sapiens zinc finger protein 160 (ZNF160), transcript variant 2, mRNA [NM_198893]	-0.8105149	0.0057916977
A_24_P75158	*CEBPZOS*	Homo sapiens CEBPZ opposite strand (CEBPZOS), transcript variant 1, long non-coding RNA [NR_037879]	-0.51405895	0.009765222
A_33_P3260777	*FAU*	Homo sapiens Finkel-Biskis-Reilly murine sarcoma virus (FBR-MuSV) ubiquitously expressed (FAU), mRNA [NM_001997]	-0.3621809	0.003956951
A_23_P304489	*PTAR1*	Homo sapiens protein prenyltransferase alpha subunit repeat containing 1 (PTAR1), mRNA [NM_001099666]	-0.6881869	0.017157309
A_33_P3239854	*ARF5*	Homo sapiens ADP-ribosylation factor 5 (ARF5), mRNA [NM_001662]	-0.85761684	0.004188518
A_23_P28953	*FAM19A5*	Homo sapiens family with sequence similarity 19 (chemokine (C-C motif)-like), member A5 (FAM19A5), transcript variant 2, mRNA [NM_015381]	-4.531445	0.0027714507
A_23_P107735	*ALKBH5*	Homo sapiens AlkB family member 5, RNA demethylase (ALKBH5), mRNA [NM_017758]	0.42651224	0.016435314
A_33_P3290394	*DNMT3B*	Homo sapiens DNA (cytosine-5-)-methyltransferase 3 beta (DNMT3B), transcript variant 6, mRNA [NM_175850]	-1.388045	0.0044864463
A_23_P94118	*CD79A*	Homo sapiens CD79a molecule, immunoglobulin-associated alpha (CD79A), transcript variant 1, mRNA [NM_001783]	1.079704	0.009056631
A_21_P0011065	*IL2RG*	Homo sapiens interleukin 2 receptor, gamma (IL2RG), mRNA [NM_000206]	0.5423227	0.014307808
A_23_P376735	*GTF2E2*	Homo sapiens general transcription factor IIE, polypeptide 2, beta 34kDa (GTF2E2), mRNA [NM_002095]	-0.42308113	0.008477025

A_33_P3389728	*ZNF524*	Homo sapiens zinc finger protein 524 (ZNF524), mRNA [NM_153219]	-0.4266443	0.015534843
A_22_P00005677	*LOC101928000*	PREDICTED: Homo sapiens uncharacterized LOC101928000 (LOC101928000), ncRNA [XR_243583]	0.37155268	0.013585372
A_23_P21734	*NR5A2*	Homo sapiens nuclear receptor subfamily 5, group A, member 2 (NR5A2), transcript variant 1, mRNA [NM_205860]	1.6403474	0.0052543716
A_33_P3413463	*lnc-EIF4EBP1-1*	Homo sapiens, clone IMAGE:4431109, mRNA. [BC015455]	-1.1892717	0.0042764777
A_24_P222997	*AK6*	Homo sapiens adenylate kinase 6 (AK6), transcript variant 3, mRNA [NM_001015891]	-0.95151186	0.004788691
A_23_P32029	*BRF1*	Homo sapiens BRF1, RNA polymerase III transcription initiation factor 90 kDa subunit (BRF1), transcript variant 1, mRNA [NM_001519]	-0.43346447	0.0068250843
A_32_P76156	*ZRANB3*	Homo sapiens zinc finger, RAN-binding domain containing 3 (ZRANB3), transcript variant 1, mRNA [NM_032143]	-0.32605308	0.014768076
A_33_P3238866	*SLC35D2*	Homo sapiens solute carrier family 35 (UDP-GlcNAc/UDP-glucose transporter), member D2 (SLC35D2), transcript variant 1, mRNA [NM_007001]	-0.33073926	0.0036861196
A_21_P0000590	*RWDD4*	Homo sapiens RWD domain containing 4 (RWDD4), mRNA [NM_152682]	-0.7483979	0.0057262382
A_33_P3251703	*SBDS*	Homo sapiens Shwachman-Bodian-Diamond syndrome (SBDS), mRNA [NM_016038]	-1.3159068	0.013143453
A_33_P3411388	*ZEB2*	Homo sapiens zinc finger E-box binding homeobox 2 (ZEB2), transcript variant 3, non-coding RNA [NR_033258]	-0.9574543	0.0063667204
A_21_P0012185	*CRIP1*	Homo sapiens cysteine-rich protein 1 (intestinal) (CRIP1), mRNA [NM_001311]	-1.3331084	0.0037947425
A_33_P3223759	*UNCX*	Homo sapiens UNC homeobox (UNCX), mRNA [NM_001080461]	0.85539985	0.008864373
A_33_P3400828	*MED13*	Homo sapiens mediator complex subunit 13 (MED13), mRNA [NM_005121]	-10075363	0.0053253504
A_21_P0013825	*GBP2*	Homo sapiens guanylate binding protein 2, interferon-inducible (GBP2), mRNA [NM_004120]	-0.7700671	0.0037856582
A_33_P3255631	*NXNL2*	Homo sapiens nucleoredoxin-like 2 (NXNL2), transcript variant 1, mRNA [NM_001161625]	-0.8788396	0.009938745
A_33_P3243657	*STAU2*	Homo sapiens staufen double-stranded RNA binding protein 2 (STAU2), transcript variant 1, mRNA [NM_001164380]	-0.81786734	0.009924993
A_24_P158421	*ZNF780A*	Homo sapiens zinc finger protein 780A (ZNF780A), transcript variant 2, mRNA [NM_001010880]	-0.9534662	0.006816371

A_23_P77073	*TIMM10B*	Homo sapiens translocase of inner mitochondrial membrane 10 homolog B (yeast) (TIMM10B), mRNA [NM_012192]	-0.5907371	0.01294906
A_23_P93431	*SAR1A*	Homo sapiens secretion associated, Ras related GTPase 1A (SAR1A), transcript variant 2, mRNA [NM_020150]	-0.89130634	0.006199031
A_23_P36689	*SPPL2A*	Homo sapiens signal peptide peptidase like 2A (SPPL2A), mRNA [NM_032802]	-0.78549385	0.010750983
A_24_P26897	*AIG1*	Homo sapiens androgen-induced 1 (AIG1), transcript variant 1, mRNA [NM_016108]	-1.179337	0.005182173
A_24_P341674	*LRRC23*	Homo sapiens leucine rich repeat containing 23 (LRRC23), transcript variant 2, mRNA [NM_006992]	-0.9072567	0.01264908
A_22_P00013992	*INPP5A*	Homo sapiens inositol polyphosphate-5-phosphatase, 40kDa (INPP5A), mRNA [NM_005539]	-0.8323818	0.00825568
A_23_P9523	*DIO2*	Homo sapiens deiodinase, iodothyronine, type II (DIO2), transcript variant 1, mRNA [NM_013989]	-0.9153975	0.003609745
A_33_P3277328	*lnc-RUNDC3A-1*	AGENCOURT_8824882 NIH_MGC_18 Homo sapiens cDNA clone IMAGE:6423362 5', mRNA sequence [BQ943064]	0.5264673	0.005303325
A_23_P205913	*RBKS*	Homo sapiens ribokinase (RBKS), transcript variant 1, mRNA [NM_022128]	-1.4325314	0.006052523
A_23_P91657	*LOC730102*	Homo sapiens quinone oxidoreductase-like protein 2 pseudogene (LOC730102), non-coding RNA [NR_037167]	-0.38609812	0.0144273415
A_33_P3324980	*SLC24A1*	Homo sapiens solute carrier family 24 (sodium/potassium/calcium exchanger), member 1 (SLC24A1), transcript variant 1, mRNA [NM_004727]	-0.6340169	0.010804776
A_24_P303874	*EWSR1*	Homo sapiens EWS RNA-binding protein 1 (EWSR1), transcript variant 1, mRNA [NM_013986]	0.7621846	0.011828812
A_21_P0005573	*ARID5B*	Homo sapiens AT rich interactive domain 5B (MRF1-like) (ARID5B), transcript variant 1, mRNA [NM_032199]	1.1806455	0.01039718
A_21_P0000583	*C9orf62*	Homo sapiens chromosome 9 open reading frame 62 (C9orf62), mRNA [NM_173520]	1.0571865	0.011708419
A_33_P6505283	*lnc-PIK3CG-2*	LNCipedia lincRNA (lnc-PIK3CG-2), lincRNA [lnc-PIK3CG-2:3]	1.228625	0.016549561
A_33_P3213752	*LOC439994*	Homo sapiens uncharacterized LOC439994 (LOC439994), long non-coding RNA [NR_029408]	-1.0565189	0.011110318
A_21_P0004247	*LOC100507316*	Homo sapiens uncharacterized LOC100507316 (LOC100507316), transcript variant 2, long non-coding RNA [NR_120683]	-0.9111614	0.0054365364

A_24_P132099	*CDK5RAP1*	Homo sapiens CDK5 regulatory subunit associated protein 1 (CDK5RAP1), transcript variant 1, mRNA [NM_016408]	-0.6186502	0.010369088
A_24_P376422	*lnc-AC114947.1.1-2*	LNCipedia lincRNA (lnc-AC114947.1.1-2), lincRNA [lnc-AC114947.1.1-2:5]	-0.84115416	0.0093693705
A_32_P61857	*HMGN3*	Homo sapiens high mobility group nucleosomal binding domain 3 (HMGN3), transcript variant 2, mRNA [NM_138730]	-0.7355539	0.007929221
A_23_P203137	*KIAA1468*	Homo sapiens KIAA1468 (KIAA1468), mRNA [NM_020854]	-0.56866115	0.0055431053
A_24_P333571	*FAM206A*	Homo sapiens family with sequence similarity 206, member A (FAM206A), mRNA [NM_017832]	-0.9666485	0.009014498
A_32_P232559	*UBE4A*	Homo sapiens ubiquitination factor E4A (UBE4A), transcript variant 1, mRNA [NM_004788]	-0.7289429	0.0068771048
A_33_P3212804	*ARHGAP29*	Rho GTPase activating protein 29 [Source:HGNC Symbol;Acc:HGNC:30207] [ENST00000370217]	-1.8981644	0.00324898
A_24_P364838	*PRKCQ-AS1*	Homo sapiens PRKCQ antisense RNA 1 (PRKCQ-AS1), transcript variant 1, long non-coding RNA [NR_036502]	1.5119594	0.009182041
A_33_P3399433	*C22orf42*	Homo sapiens chromosome 22 open reading frame 42 (C22orf42), mRNA [NM_001010859]	-0.8776994	0.0029471128
A_22_P00012072	*SLC9A3R2*	Homo sapiens solute carrier family 9, subfamily A (NHE3, cation proton antiporter 3), member 3 regulator 2 (SLC9A3R2), transcript variant 2, mRNA [NM_004785]	0.9420921	0.011739143
A_23_P34968	*C20orf27*	Homo sapiens chromosome 20 open reading frame 27 (C20orf27), transcript variant 3, mRNA [NM_001258430]	-0.39019677	0.014629385
A_23_P98085	*lnc-PMEL-1*	LNCipedia lincRNA (lnc-PMEL-1), lincRNA [lnc-PMEL-1:1]	-0.46094528	0.0148595255
A_33_P3383261	*SCNM1*	Homo sapiens sodium channel modifier 1 (SCNM1), transcript variant 2, mRNA [NM_001204856]	-0.9296975	0.006814677
A_33_P3380101	*PTEN*	Homo sapiens phosphatase and tensin homolog (PTEN), mRNA [NM_000314]	-0.75012755	0.008425284
A_23_P404045	*SIKE1*	Homo sapiens suppressor of IKBKE 1 (SIKE1), transcript variant 1, mRNA [NM_001102396]	-1.0971582	0.005640775
A_24_P920521	*MAP7D1*	Homo sapiens MAP7 domain containing 1 (MAP7D1), transcript variant 1, mRNA [NM_018067]	0.6167927	0.009784609
A_23_P44257	*MTHFR*	Homo sapiens methylenetetrahydrofolate reductase (NAD(P)H) (MTHFR), mRNA [NM_005957]	0.58540154	0.0031941293

A_23_P201386	*COMMD6*	Homo sapiens COMM domain containing 6 (COMMD6), transcript variant 1, mRNA [NM_203497]	-0.5318163	0.014885985
A_22_P00000676	*COMMD8*	Homo sapiens COMM domain containing 8 (COMMD8), mRNA [NM_017845]	-0.958586	0.0041847117
A_23_P18317	*DDAH1*	Homo sapiens dimethylarginine dimethylaminohydrolase 1 (DDAH1), transcript variant 1, mRNA [NM_012137]	-15202451	0.0040017776
A_23_P162734	*SLC41A3*	Homo sapiens solute carrier family 41, member 3 (SLC41A3), transcript variant 2, mRNA [NM_017836]	-1.1674495	0.0070515345
A_24_P914817	*SLC26A2*	Homo sapiens solute carrier family 26 (anion exchanger), member 2 (SLC26A2), mRNA [NM_000112]	-0.46693203	0.010735533
A_24_P290502	*RNF6*	Homo sapiens ring finger protein (C3H2C3 type) 6 (RNF6), transcript variant 1, mRNA [NM_005977]	-0.58058167	0.0123029845
A_23_P104109	*MKLN1*	Homo sapiens muskelin 1, intracellular mediator containing kelch motifs (MKLN1), transcript variant 2, mRNA [NM_013255]	-0.4703205	0.014323314
A_33_P3235390	*GCC2*	Homo sapiens GRIP and coiled-coil domain containing 2 (GCC2), transcript variant 1, mRNA [NM_181453]	-0.7504196	0.0070515345
A_23_P106433	*RPS6KC1*	Homo sapiens ribosomal protein S6 kinase, 52kDa, polypeptide 1 (RPS6KC1), transcript variant 1, mRNA [NM_012424]	-0.6371002	0.0073898793
A_33_P3283201	*UBA5*	Homo sapiens ubiquitin-like modifier activating enzyme 5 (UBA5), transcript variant 1, mRNA [NM_024818]	-0.7010892	0.009271572
A_24_P105564	*RCCD1*	Homo sapiens RCC1 domain containing 1 (RCCD1), transcript variant 1, mRNA [NM_033544]	-0.47571325	0.011787466
A_33_P3321055	*PRKAB2*	Homo sapiens protein kinase, AMP-activated, beta 2 non-catalytic subunit (PRKAB2), transcript variant 1, mRNA [NM_005399]	-1.7202439	0.0037353234
A_23_P136172	*PDZK1*	Homo sapiens PDZ domain containing 1 (PDZK1), transcript variant 1, mRNA [NM_002614]	-1.3295228	0.006382261
A_23_P162719	*TNFRSF19*	tumor necrosis factor receptor superfamily, member 19 [Source:HGNC Symbol;Acc:HGNC:11915] [ENST00000464735]	1.1460435	0.010091486
A_33_P3441576	*TMEM68*	Homo sapiens transmembrane protein 68 (TMEM68), transcript variant 2, mRNA [NM_152417]	-0.6003374	0.008031949
A_33_P3212700	*DIAPH3*	Homo sapiens diaphanous-related formin 3 (DIAPH3), transcript variant 2, mRNA [NM_030932]	-0.743144	0.007503526

A_23_P32217	*SRGAP2C*	Homo sapiens SLIT-ROBO Rho GTPase activating protein 2C (SRGAP2C), mRNA [NM_001271872]	-0.9197886	0.00910823
A_22_P00010173	*TOPORS*	Homo sapiens topoisomerase I binding, arginine/serine-rich, E3 ubiquitin protein ligase (TOPORS), transcript variant 1, mRNA [NM_005802]	-0.57130027	0.004016376
A_33_P3840512	*SYMPK*	Homo sapiens symplekin (SYMPK), mRNA [NM_004819]	-0.62263536	0.005822303
A_22_P00005227	*FGD5-AS1*	Homo sapiens FGD5 antisense RNA 1 (FGD5-AS1), transcript variant 1, long non-coding RNA [NR_046251]	-0.7217269	0.0056601833
A_23_P72059	*SLC25A15*	Homo sapiens solute carrier family 25 (mitochondrial carrier; ornithine transporter) member 15 (SLC25A15), mRNA [NM_014252]	-0.7605833	0.013532116
A_33_P3285893	*SNHG23*	small nucleolar RNA host gene 23 (non-protein coding) [Source:HGNC Symbol;Acc:HGNC:50622] [ENST00000556720]	1.0092477	0.0041523096
A_23_P382775	*VSIG10*	Homo sapiens V-set and immunoglobulin domain containing 10 (VSIG10), mRNA [NM_019086]	-0.7400056	0.010480952
A_21_P0000399	*SCRN1*	Homo sapiens secernin 1 (SCRN1), transcript variant 1, mRNA [NM_001145513]	0.8481389	0.013623725
A_33_P3306267	*BBC3*	Homo sapiens BCL2 binding component 3 (BBC3), transcript variant 4, mRNA [NM_014417]	-1.6594603	0.0054085264
A_33_P3411315	*SNORD114-2*	Homo sapiens small nucleolar RNA, C/D box 114-2 (SNORD114-2), small nucleolar RNA [NR_003194]	0.47648638	0.0053727482
A_33_P3314550	*PAX6*	Homo sapiens paired box 6 (PAX6), transcript variant 1, mRNA [NM_000280]	3.0932097	0.0037583082
A_33_P3382177	*KRTAP3-3*	Homo sapiens keratin associated protein 3-3 (KRTAP3-3), mRNA [NM_033185]	1.181652	0.0142584685
A_24_P159648	*RAB3A*	Homo sapiens RAB3A, member RAS oncogene family (RAB3A), mRNA [NM_002866]	2.4867437	0.0052438728
A_24_P299318	*TIMP2*	Homo sapiens TIMP metallopeptidase inhibitor 2 (TIMP2), mRNA [NM_003255]	-0.38184693	0.009574626
A_33_P3393135	*BAIAP2*	Homo sapiens BAI1-associated protein 2 (BAIAP2), transcript variant 3, mRNA [NM_006340]	1.3837606	0.0072648157
A_32_P228501	*FAM101B*	Homo sapiens family with sequence similarity 101, member B (FAM101B), mRNA [NM_182705]	-1.3646715	0.0041523096
A_33_P3210521	*HIST1H2AI*	Homo sapiens histone cluster 1, H2ai, mRNA (cDNA clone MGC:138459 IMAGE:8327722), complete cds. [BC112254]	0.670808	0.011006847
A_21_P0014677	*CDC26*	Homo sapiens cell division cycle 26 (CDC26), mRNA [NM_139286]	-0.39927194	0.0114650065

A_24_P277155	*C2orf43*	Homo sapiens chromosome 2 open reading frame 43 (C2orf43), transcript variant 1, mRNA [NM_021925]	-0.5150252	0.010931163
A_21_P0008550	*RAB21*	RAB21, member RAS oncogene family [Source:HGNC Symbol;Acc:HGNC:18263] [ENST00000261263]	-0.564857	0.014103071
A_32_P194115	*HLTF*	Homo sapiens helicase-like transcription factor (HLTF), transcript variant 1, mRNA [NM_003071]	-0.9858308	0.016219413
A_21_P0009973	*lnc-BCL11B-1*	LNCipedia lincRNA (lnc-BCL11B-1), lincRNA [lnc-BCL11B-1:3]	1.2092488	0.00847175
A_23_P71821	*SUN2*	Homo sapiens Sad1 and UNC84 domain containing 2 (SUN2), transcript variant 2, mRNA [NM_015374]	0.3955621	0.013453215
A_23_P366328	*lnc-PTPN1-2*	LNCipedia lincRNA (lnc-PTPN1-2), lincRNA [lnc-PTPN1-2:1]	1.2205738	0.0070447545
A_33_P3242743	*PBX3*	Homo sapiens pre-B-cell leukemia homeobox 3 (PBX3), transcript variant 1, mRNA [NM_006195]	-0.37213108	0.007885157
A_24_P106112	*VPS37A*	Homo sapiens vacuolar protein sorting 37 homolog A (S. cerevisiae) (VPS37A), transcript variant 1, mRNA [NM_152415]	-0.7523658	0.008459498
A_24_P49533	*ARFRP1*	Homo sapiens ADP-ribosylation factor related protein 1 (ARFRP1), transcript variant 8, mRNA [NM_001267549]	-0.6826287	0.012896412
A_24_P185314	*PKD2*	Homo sapiens polycystic kidney disease 2 (autosomal dominant) (PKD2), mRNA [NM_000297]	-1.063683	0.0072222846
A_23_P214168	*ZYG11B*	Homo sapiens zyg-11 family member B, cell cycle regulator (ZYG11B), mRNA [NM_024646]	-0.95820254	0.0070571043
A_23_P405707	*NAPG*	Homo sapiens N-ethylmaleimide-sensitive factor attachment protein, gamma (NAPG), mRNA [NM_003826]	-0.69659257	0.0090518845
A_33_P3349693	*COL12A1*	Homo sapiens collagen, type XII, alpha 1 (COL12A1), transcript variant long, mRNA [NM_004370]	-0.51741815	0.004912971
A_19_P00322929	*BCOR*	BCL6 corepressor [Source:HGNC Symbol;Acc:HGNC:20893] [ENST00000615339]	0.8869899	0.0033202018
A_22_P00016450	*LCLAT1*	Homo sapiens lysocardiolipin acyltransferase 1 (LCLAT1), transcript variant 1, mRNA [NM_182551]	-1.1583434	0.005509873
A_21_P0012439	*lnc-TMEM63A-1*	BC019442 transmembrane protein 63a {Mus musculus} (exp=-1; wgp=0; cg=0), partial (16%) [THC2541010]	-1.3593794	0.004834548
A_24_P47467	*NBR2*	Homo sapiens neighbor of BRCA1 gene 2 (non-protein coding) (NBR2), long non-coding RNA [NR_003108]	0.59675324	0.014704332

A_33_P3576797	*TRANK1*	Homo sapiens tetratricopeptide repeat and ankyrin repeat containing 1 (TRANK1), mRNA [NM_014831]	-0.38202658	0.0074381046
A_33_P3404749	*TMEM109*	Homo sapiens transmembrane protein 109 (TMEM109), mRNA [NM_024092]	-0.4690904	0.01581842
A_24_P297888	*LOC158863*	Homo sapiens mRNA; cDNA DKFZp586J1922 (from clone DKFZp586J1922) [AL110203]	-1.0687282	0.008639274
A_24_P56467	*FMN1*	Homo sapiens formin 1 (FMN1), transcript variant 1, mRNA [NM_001277313]	-1.8030151	0.0057647754
A_32_P145153	*MTAP*	Homo sapiens methylthioadenosine phosphorylase (MTAP), mRNA [NM_002451]	0.9773576	0.014125317
A_33_P3397323	*GMPR2*	Homo sapiens guanosine monophosphate reductase 2 (GMPR2), transcript variant 2, mRNA [NM_001002000]	-0.9322834	0.0053870557
A_22_P00001459	*RPL31*	Homo sapiens ribosomal protein L31 (RPL31), transcript variant 1, mRNA [NM_000993]	0.8379524	0.013050359
A_24_P310756	*ZNF441*	Homo sapiens zinc finger protein 441 (ZNF441), mRNA [NM_152355]	-1.420471	0.006324384
A_32_P170444	*lnc-AQP11-2*	LNCipedia lincRNA (lnc-AQP11-2), lincRNA [lnc-AQP11-2:1]	0.5498151	0.009421056
A_24_P109432	*FBXO28*	Homo sapiens F-box protein 28 (FBXO28), transcript variant 1, mRNA [NM_015176]	-1.0307589	0.010201849
A_22_P00001466	*SUB1*	Homo sapiens SUB1 homolog (S. cerevisiae) (SUB1), mRNA [NM_006713]	-0.44323522	0.014361322
A_23_P8522	*NBEAL1*	Homo sapiens neurobeachin-like 1 (NBEAL1), mRNA [NM_001114132]	-1.0647645	0.003363001
A_32_P234604	*lnc-AQP9-1*	Homo sapiens hypothetical protein LOC283665, mRNA (cDNA clone IMAGE:4826990). [BC034958]	0.7939472	0.01301878
A_23_P30275	*TMEM106B*	Homo sapiens transmembrane protein 106B (TMEM106B), transcript variant 1, mRNA [NM_018374]	-0.34040526	0.008403154
A_23_P82823	*PFN1*	Homo sapiens profilin 1 (PFN1), mRNA [NM_005022]	0.76629585	0.004940129
A_33_P3248654	*PCYOX1L*	Homo sapiens prenylcysteine oxidase 1 like (PCYOX1L), transcript variant 1, mRNA [NM_024028]	-1.761084	0.0065888464
A_22_P00014723	*PINX1*	Homo sapiens PIN2/TERF1 interacting, telomerase inhibitor 1 (PINX1), transcript variant 1, mRNA [NM_017884]	-0.7098005	0.007445825
A_21_P0002319	*SARS*	Homo sapiens seryl-tRNA synthetase (SARS), transcript variant 1, mRNA [NM_006513]	-1.0246494	0.0033198062
A_23_P48771	*TARID*	Homo sapiens TCF21 antisense RNA inducing promoter demethylation (TARID), long non-coding RNA [NR_109982]	2.1654296	0.0054923543
A_33_P3311637	*LINC01117*	Homo sapiens long intergenic non-protein coding RNA 1117 (LINC01117), long non-coding RNA [NR_110599]	-0.61148864	0.010567244

A_21_P0007523	*C14orf159*	Homo sapiens chromosome 14 open reading frame 159 (C14orf159), transcript variant 3, mRNA [NM_024952]	-0.42532924	0.012579966
A_21_P0008857	*SCFD1*	Homo sapiens sec1 family domain containing 1 (SCFD1), transcript variant 1, mRNA [NM_016106]	-0.4576604	0.005612091
A_33_P3243997	*DDX11-AS1*	Homo sapiens DDX11 antisense RNA 1 (DDX11-AS1), long non-coding RNA [NR_038927]	1.1589284	0.0062957765
A_23_P161698	*lnc-PEAK1.1-1*	LNCipedia lincRNA (lnc-PEAK1.1-1), lincRNA [lnc-PEAK1.1-1:3]	-1.2107081	0.016837673
A_23_P215787	*PSME4*	Homo sapiens proteasome (prosome, macropain) activator subunit 4 (PSME4), mRNA [NM_014614]	-0.8227375	0.008362665
A_21_P0000142	*MMP3*	Homo sapiens matrix metallopeptidase 3 (stromelysin 1, progelatinase) (MMP3), mRNA [NM_002422]	-1.2052348	0.0057262382
A_33_P3297888	*HBP1*	Homo sapiens HMG-box transcription factor 1 (HBP1), transcript variant 2, mRNA [NM_012257]	-0.82475716	0.009924993
A_23_P410613	*RNF103*	Homo sapiens ring finger protein 103 (RNF103), transcript variant 3, mRNA [NM_001198952]	0.82670593	0.008208044
A_23_P346006	*CCDC125*	Homo sapiens coiled-coil domain containing 125 (CCDC125), transcript variant 1, mRNA [NM_176816]	-0.48287687	0.0053727482
A_23_P56567	*TMEM263*	Homo sapiens transmembrane protein 263 (TMEM263), mRNA [NM_152261]	-1.0316741	0.009296599
A_32_P125549	*CCPG1*	Homo sapiens cell cycle progression 1 (CCPG1), transcript variant 2, mRNA [NM_020739]	-1.2411691	0.0035186775
A_23_P79069	*GEMIN6*	Homo sapiens gem (nuclear organelle) associated protein 6 (GEMIN6), mRNA [NM_024775]	-0.5275028	0.011788845
A_21_P0006372	*RASAL3*	Homo sapiens RAS protein activator like 3 (RASAL3), mRNA [NM_022904]	2.5629435	0.0027974492
A_24_P108242	*MANEA*	Homo sapiens mannosidase, endo-alpha (MANEA), mRNA [NM_024641]	0.17205189	0.011568712
A_23_P344531	*LOC101929014*	PREDICTED: Homo sapiens uncharacterized LOC101929014 (LOC101929014), ncRNA [XR_242676]	1.2453816	0.008276945
A_23_P314191	*GOSR2*	Homo sapiens golgi SNAP receptor complex member 2 (GOSR2), transcript variant A, mRNA [NM_004287]	-0.8393759	0.007549738
A_24_P170095	*SYNPO*	Homo sapiens synaptopodin (SYNPO), transcript variant 1, mRNA [NM_007286]	0.7129609	0.014939495
A_33_P3320368	*ZDHHC17*	Homo sapiens zinc finger, DHHC-type containing 17 (ZDHHC17), mRNA [NM_015336]	-1.0421798	0.013587645

A_23_P3368	*TMEM161A*	Homo sapiens transmembrane protein 161A (TMEM161A), transcript variant 1, mRNA [NM_017814]	-0.41368267	0.01012583
A_24_P43391	*LOC100506990*	Homo sapiens uncharacterized LOC100506990 (LOC100506990), transcript variant 2, long non-coding RNA [NR_040092]	-1.1140112	0.009503543
A_24_P212152	*FURIN*	Homo sapiens furin (paired basic amino acid cleaving enzyme) (FURIN), transcript variant 1, mRNA [NM_002569]	0.7265281	0.0072648157
A_32_P187617	*TMEM165*	Homo sapiens transmembrane protein 165 (TMEM165), transcript variant 1, mRNA [NM_018475]	-0.87487745	0.008750102
A_23_P39774	*MZT2B*	Homo sapiens mitotic spindle organizing protein 2B (MZT2B), mRNA [NM_025029]	-0.6025655	0.011678774
A_23_P396666	*TDRD3*	Homo sapiens tudor domain containing 3 (TDRD3), transcript variant 2, mRNA [NM_030794]	-1.0649897	0.011745691
A_23_P331700	*SNRNP27*	Homo sapiens small nuclear ribonucleoprotein 27kDa (U4/U6.U5) (SNRNP27), transcript variant 1, mRNA [NM_006857]	-0.5589674	0.014982028
A_32_P360193	*TBC1D2B*	Homo sapiens TBC1 domain family, member 2B (TBC1D2B), transcript variant 2, mRNA [NM_015079]	-0.89629644	0.013460425
A_23_P110345	*SRRM3*	Homo sapiens serine/arginine repetitive matrix 3 (SRRM3), transcript variant 1, mRNA [NM_001291831]	1.260979	0.008554032
A_23_P435833	*DNHD1*	Homo sapiens dynein heavy chain domain 1 (DNHD1), transcript variant 1, mRNA [NM_144666]	0.5764081	0.010782863
A_21_P0011384	*CHIC2*	Homo sapiens cysteine-rich hydrophobic domain 2 (CHIC2), mRNA [NM_012110]	-1.0772452	0.0038144474
A_23_P110430	*FUBP3*	Homo sapiens far upstream element (FUSE) binding protein 3 (FUBP3), mRNA [NM_003934]	0.5263564	0.016152125
A_24_P402690	*UBE2Q2P1*	Homo sapiens ubiquitin-conjugating enzyme E2Q family member 2 pseudogene 1 (UBE2Q2P1), non-coding RNA [NR_003661]	-0.74789834	0.012699999
A_23_P134601	*MSX1*	Homo sapiens msh homeobox 1 (MSX1), mRNA [NM_002448]	-0.8645523	0.013248894
A_23_P316064	*ITM2C*	Homo sapiens integral membrane protein 2C (ITM2C), transcript variant 1, mRNA [NM_030926]	-0.6565659	0.008483943
A_19_P00808453	*WNT16*	Homo sapiens wingless-type MMTV integration site family, member 16 (WNT16), transcript variant 1, mRNA [NM_057168]	-1.5558643	0.0057522994
A_23_P403335	*ZACN*	Homo sapiens zinc activated ligand-gated ion channel (ZACN), mRNA [NM_180990]	-0.84525925	0.009988937

A_24_P358305	*COX5A*	Homo sapiens cytochrome c oxidase subunit Va (COX5A), mRNA [NM_004255]	-0.42818546	0.011788845
A_24_P219552	*EXPH5*	Homo sapiens exophilin 5 (EXPH5), mRNA [NM_015065]	-1.013004	0.0073811873
A_33_P3376379	*NFE2L1*	Homo sapiens nuclear factor, erythroid 2-like 1 (NFE2L1), mRNA [NM_003204]	-0.71881866	0.009490355
A_23_P89799	*FAM72D*	Homo sapiens family with sequence similarity 72, member D (FAM72D), mRNA [NM_207418]	-0.6100135	0.010274393
A_23_P89727	*CNST*	Homo sapiens consortin, connexin sorting protein (CNST), transcript variant 2, mRNA [NM_001139459]	-0.6877361	0.009939401
A_32_P18250	*ACAA2*	Homo sapiens acetyl-CoA acyltransferase 2 (ACAA2), mRNA [NM_006111]	-1.6285458	0.0055521973
A_23_P98382	*CTDP1*	Homo sapiens CTD (carboxy-terminal domain, RNA polymerase II, polypeptide A) phosphatase, subunit 1 (CTDP1), transcript variant 2, mRNA [NM_048368]	0.8288819	0.006632856
A_24_P12281	*FUNDC2P2*	Homo sapiens FUN14 domain containing 2 pseudogene 2 (FUNDC2P2), non-coding RNA [NR_003663]	-0.41469026	0.004220053
A_33_P3286334	*TIMM8B*	Homo sapiens translocase of inner mitochondrial membrane 8 homolog B (yeast) (TIMM8B), transcript variant 1, mRNA [NM_012459]	-0.7094996	0.0027348737
A_33_P3295650	*AP4S1*	Homo sapiens adaptor-related protein complex 4, sigma 1 subunit (AP4S1), transcript variant 1, mRNA [NM_007077]	-0.52956825	0.008427824
A_33_P3362239	*SH3BP1*	Homo sapiens SH3-domain binding protein 1 (SH3BP1), mRNA [NM_018957]	1.0585593	0.0076989555
A_24_P255218	*APBA1*	Homo sapiens amyloid beta (A4) precursor protein-binding, family A, member 1 (APBA1), mRNA [NM_001163]	1.4693519	0.0029471128
A_22_P00017022	*MYO5A*	Homo sapiens myosin VA (heavy chain 12, myoxin) (MYO5A), transcript variant 1, mRNA [NM_000259]	-0.56778485	0.014313585
A_24_P274795	*TPM3*	Homo sapiens tropomyosin 3 (TPM3), transcript variant Tpm3.12, mRNA [NM_152263]	-0.6860396	0.012878467
A_22_P00018895	*HAPLN3*	Homo sapiens hyaluronan and proteoglycan link protein 3 (HAPLN3), mRNA [NM_178232]	-2.70567028272314	0.0030114138
A_24_P259922	*CDCA7L*	Homo sapiens cell division cycle associated 7-like (CDCA7L), transcript variant 1, mRNA [NM_018719]	-0.6787936	0.007836227
A_21_P0014571	*LANCL1*	Homo sapiens LanC lantibiotic synthetase component C-like 1 (bacterial) (LANCL1), transcript variant 1, mRNA [NM_006055]	-0.9289691	0.0066432646

A_32_P46571	*NGRN*	neugrin, neurite outgrowth associated [Source:HGNC Symbol;Acc:HGNC:18077] [ENST00000379095]	-0.5602659	0.016789315
A_32_P115558	*lnc-NT5DC3-1*	AF160476 CD44-like precursor FELL {Homo sapiens} (exp=-1; wgp=0; cg=0), partial (5%) [THC2518757]	-1.013987	0.014228801
A_23_P399146	*RHBDL2*	Homo sapiens rhomboid, veinlet-like 2 (Drosophila) (RHBDL2), mRNA [NM_017821]	-1.3384109	0.012982396
A_24_P244706	*ZNF549*	Homo sapiens zinc finger protein 549 (ZNF549), transcript variant 2, mRNA [NM_153263]	-0.6366747	0.015581156
A_21_P0014808	*GPR68*	Homo sapiens G protein-coupled receptor 68 (GPR68), transcript variant 2, mRNA [NM_003485]	2.1675894	0.0033868244
A_22_P00014292	*SSTR1*	Homo sapiens somatostatin receptor 1 (SSTR1), mRNA [NM_001049]	1.0690197	0.0077546863
A_23_P201432	*lnc-SERAC1-2*	LNCipedia lincRNA (lnc-SERAC1-2), lincRNA [lnc-SERAC1-2:1]	0.4353124	0.010510407
A_22_P00025801	*SHC4*	Homo sapiens SHC (Src homology 2 domain containing) family, member 4 (SHC4), mRNA [NM_203349]	1.370194	0.0039486005
A_22_P00016826	*ADSS*	Homo sapiens adenylosuccinate synthase (ADSS), mRNA [NM_001126]	-0.8103266	0.0047857375
A_32_P121674	*TMEM210*	transmembrane protein 210 [Source:HGNC Symbol;Acc:HGNC:34059] [ENST00000535352]	0.897316	0.006465216
A_24_P63347	*lnc-TRIP4-1*	LNCipedia lincRNA (lnc-TRIP4-1), lincRNA [lnc-TRIP4-1:1]	-0.3336111	0.014822511
A_33_P3275801	*lnc-PAFAH1B1-2*	AV709080 ADC Homo sapiens cDNA clone ADCAZF07 5', mRNA sequence [AV709080]	-0.24000633	0.016260965
A_22_P00014171	*PF4V1*	Homo sapiens platelet factor 4 variant 1 (PF4V1), mRNA [NM_002620]	3.9137504	0.0030069214
A_23_P336015	*DES*	Homo sapiens desmin (DES), mRNA [NM_001927]	-0.8353019	0.009630487
A_32_P75284	*MALAT1*	Homo sapiens metastasis associated lung adenocarcinoma transcript 1 (non-protein coding) (MALAT1), long non-coding RNA [NR_002819]	0.17266968	0.00919727
A_32_P155460	*NOC2L*	Homo sapiens nucleolar complex associated 2 homolog (S. cerevisiae) (NOC2L), mRNA [NM_015658]	1.2776529	0.014681147
A_33_P3258362	*KATNAL1*	Homo sapiens katanin p60 subunit A-like 1 (KATNAL1), transcript variant 1, mRNA [NM_032116]	-0.8574536	0.009334625
A_23_P32135	*LINC00162*	Homo sapiens long intergenic non-protein coding RNA 162 (LINC00162), long non-coding RNA [NR_024089]	-0.94493145	0.0088963
A_33_P3305487	*HBA2*	Homo sapiens hemoglobin, alpha 2 (HBA2), mRNA [NM_000517]	1.5259771	0.0050554653

A_23_P209232	*C9orf9*	Homo sapiens chromosome 9 open reading frame 9 (C9orf9), mRNA [NM_018956]	-1.2752745	0.008819748
A_23_P53724	*REM2*	Homo sapiens RAS (RAD and GEM)-like GTP binding 2 (REM2), mRNA [NM_173527]	-0.7965197	0.015279996
A_24_P780052	*CLIP4*	Homo sapiens CAP-GLY domain containing linker protein family, member 4 (CLIP4), transcript variant 1, mRNA [NM_024692]	-0.7149527	0.012089522
A_23_P17512	*CLSTN3*	Homo sapiens calsyntenin 3 (CLSTN3), mRNA [NM_014718]	1.3306116	0.011443248
A_33_P3352562	*RPSAP58*	Homo sapiens ribosomal protein SA pseudogene 58 (RPSAP58), non-coding RNA [NR_003662]	-0.36709663	0.009154653
A_33_P3231557	*DTD1*	Homo sapiens D-tyrosyl-tRNA deacylase 1 (DTD1), mRNA [NM_080820]	-0.7924685	0.007286966
A_24_P342096	*MFSD12*	Homo sapiens major facilitator superfamily domain containing 12 (MFSD12), transcript variant 3, mRNA [NM_174983]	0.9044516	0.013591491
A_23_P50108	*CDC14B*	Homo sapiens cell division cycle 14B (CDC14B), transcript variant 1, mRNA [NM_003671]	-1.0743597	0.0044889734
A_21_P0000556	*FAM27C*	Homo sapiens family with sequence similarity 27, member C (FAM27C), long non-coding RNA [NR_027421]	-1.8249288	0.004577453
A_21_P0000696	*NDC80*	Homo sapiens NDC80 kinetochore complex component (NDC80), mRNA [NM_006101]	-0.7157518	0.006405189
A_24_P203622	*LINC00883*	Homo sapiens long intergenic non-protein coding RNA 883 (LINC00883), transcript variant 1, long non-coding RNA [NR_028301]	-0.30240282	0.01403796
A_24_P100673	*SLIT2-IT1*	Homo sapiens SLIT2 intronic transcript 1 (non-protein coding) (SLIT2-IT1), long non-coding RNA [NR_037876]	1.5728847	0.010500704
A_23_P44932	*DOCK7*	Homo sapiens dedicator of cytokinesis 7 (DOCK7), transcript variant 2, mRNA [NM_033407]	-0.5201598	0.014981894
A_22_P00012919	*EMC4*	Homo sapiens ER membrane protein complex subunit 4 (EMC4), transcript variant 1, mRNA [NM_016454]	-0.7337802	0.006926426
A_33_P3318696	*EIF2A*	Homo sapiens eukaryotic translation initiation factor 2A, 65kDa (EIF2A), mRNA [NM_032025]	-0.65369767	0.005947757
A_24_P256063	*NMNAT1*	Homo sapiens nicotinamide nucleotide adenylyltransferase 1 (NMNAT1), transcript variant 2, mRNA [NM_001297778]	-0.6328135	0.010519677
A_33_P3359543	*CCBL2*	Homo sapiens cysteine conjugate-beta lyase 2 (CCBL2), transcript variant 1, mRNA [NM_001008661]	-0.7879896	0.0057506533
A_23_P209426	*ZNF561*	Homo sapiens zinc finger protein 561 (ZNF561), mRNA [NM_152289]	-0.74141884	0.016236924
A_23_P166663	*ZFHX3*	Homo sapiens zinc finger homeobox 3 (ZFHX3), transcript variant A, mRNA [NM_006885]	-1.0100405	0.008425284

A_23_P83098	*TRAK2*	Homo sapiens trafficking protein, kinesin binding 2 (TRAK2), mRNA [NM_015049]	-0.5184655	0.0049246643
A_23_P314145	*APPL1*	Homo sapiens adaptor protein, phosphotyrosine interaction, PH domain and leucine zipper containing 1 (APPL1), mRNA [NM_012096]	-1.0605366	0.007946748
A_23_P54649	*ALDH1A1*	Homo sapiens aldehyde dehydrogenase 1 family, member A1 (ALDH1A1), mRNA [NM_000689]	-3.1442857	0.0027248112
A_24_P106591	*UST*	Homo sapiens uronyl-2-sulfotransferase (UST), mRNA [NM_005715]	-1.314942	0.003539263
A_33_P3309075	*TRADD*	Homo sapiens TNFRSF1A-associated via death domain (TRADD), mRNA [NM_003789]	-1.1740229	0.009560995
A_23_P166122	*BLOC1S5*	Homo sapiens biogenesis of lysosomal organelles complex-1, subunit 5, muted (BLOC1S5), transcript variant 1, mRNA [NM_201280]	-1.4039755	0.003162355
A_24_P71700	*TBC1D8*	Homo sapiens TBC1 domain family, member 8 (with GRAM domain) (TBC1D8), mRNA [NM_001102426]	1.3082074	0.0031941293
A_23_P406928	*POLR3F*	Homo sapiens polymerase (RNA) III (DNA directed) polypeptide F, 39 kDa (POLR3F), transcript variant 1, mRNA [NM_006466]	-0.7130746	0.015262972
A_23_P110076	*ZBTB47*	Homo sapiens zinc finger and BTB domain containing 47 (ZBTB47), mRNA [NM_145166]	-1.4697856	0.0085387705
A_21_P0014423	*ND5*	mitochondrially encoded NADH dehydrogenase 5 [Source:HGNC Symbol;Acc:HGNC:7461] [ENST00000361567]	1.2024175	0.0075857607
A_23_P333029	*WDR53*	Homo sapiens WD repeat domain 53 (WDR53), mRNA [NM_182627]	-0.408581	0.010891201
A_33_P3425356	*ERICH5*	Homo sapiens glutamate-rich 5 (ERICH5), transcript variant 1, mRNA [NM_173549]	-0.5532468	0.0057647754
A_23_P83094	*DNTTIP1*	Homo sapiens deoxynucleotidyltransferase, terminal, interacting protein 1 (DNTTIP1), mRNA [NM_052951]	-0.61736465	0.015600941
A_33_P3387463	*ARMC8*	Homo sapiens armadillo repeat containing 8 (ARMC8), transcript variant 2, mRNA [NM_015396]	-0.99838656	0.005735339
A_23_P62371	*TLE4*	Homo sapiens transducin-like enhancer of split 4 (TLE4), transcript variant 3, mRNA [NM_007005]	-1.2779942	0.004359316
A_23_P28263	*ATP6V1D*	Homo sapiens ATPase, H+ transporting, lysosomal 34kDa, V1 subunit D (ATP6V1D), mRNA [NM_015994]	-0.75397253	0.0046609533
A_23_P207927	*TAZ*	tafazzin [Source:HGNC Symbol;Acc:HGNC:11577] [ENST00000498029]	1.026989	0.009524396
A_21_P0000236	*CTDSP1*	Homo sapiens CTD (carboxy-terminal domain, RNA polymerase II, polypeptide A) small	-0.9885432	0.0078061516

		phosphatase 1 (CTDSP1), transcript variant 1, mRNA [NM_021198]		
A_23_P218858	*C17orf80*	Homo sapiens chromosome 17 open reading frame 80 (C17orf80), transcript variant 4, mRNA [NM_001288770]	-0.8848541	0.005208127
A_23_P205830	*SNORA64*	Homo sapiens small nucleolar RNA, H/ACA box 64 (SNORA64), small nucleolar RNA [NR_002326]	-1.0984868	0.0085082175
A_33_P3408898	*ABI3BP*	Homo sapiens ABI family, member 3 (NESH) binding protein (ABI3BP), mRNA [NM_015429]	-3.9433188	0.0029471128
A_33_P3371904	*BTBD1*	Homo sapiens BTB (POZ) domain containing 1 (BTBD1), transcript variant 1, mRNA [NM_025238]	-0.6667065	0.0070324177
A_23_P253464	*C6orf226*	Homo sapiens chromosome 6 open reading frame 226 (C6orf226), mRNA [NM_001008739]	-0.827703	0.010331959
A_22_P00005423	*ASTN2*	Homo sapiens astrotactin 2 (ASTN2), transcript variant 4, mRNA [NM_198188]	-0.98001486	0.011171237
A_23_P372331	*FAM175A*	Homo sapiens family with sequence similarity 175, member A (FAM175A), mRNA [NM_139076]	-0.7461219	0.0029691157
A_23_P22433	*DTWD1*	DTW domain containing 1 [Source:HGNC Symbol;Acc:HGNC:30926] [ENST00000251250]	-1.139876	0.008940561
A_33_P3299872	*TRNT1*	Homo sapiens tRNA nucleotidyl transferase, CCA-adding, 1 (TRNT1), transcript variant 1, mRNA [NM_182916]	-0.37533605	0.0033202018
A_33_P3245709	*RP2*	Homo sapiens retinitis pigmentosa 2 (X-linked recessive) (RP2), mRNA [NM_006915]	-1.0849429	0.0065241847
A_23_P165698	*HINT3*	Homo sapiens histidine triad nucleotide binding protein 3 (HINT3), mRNA [NM_138571]	-0.8423992	0.008923579
A_33_P3237634	*RALGAPB*	Homo sapiens Ral GTPase activating protein, beta subunit (non-catalytic) (RALGAPB), transcript variant 1, mRNA [NM_020336]	-0.7492234	0.01217032
A_23_P156732	*C2orf49*	Homo sapiens chromosome 2 open reading frame 49 (C2orf49), transcript variant 1, mRNA [NM_024093]	-1.1094509	0.0044785915
A_33_P3374952	*TSC22D3*	Homo sapiens TSC22 domain family, member 3 (TSC22D3), transcript variant 2, mRNA [NM_004089]	-1.6329241	0.003392081
A_24_P126557	*PHF1*	Homo sapiens PHD finger protein 1 (PHF1), transcript variant 2, mRNA [NM_024165]	0.90167457	0.004332961
A_23_P217109	*KLF17*	Homo sapiens Kruppel-like factor 17 (KLF17), mRNA [NM_173484]	-0.71376866	0.009778202
A_23_P368773	*RAVER1*	Homo sapiens ribonucleoprotein, PTB-binding 1 (RAVER1), mRNA [NM_133452]	1.7852482	0.003956951
A_23_P250102	*SLC31A2*	Homo sapiens solute carrier family 31 (copper transporter), member 2 (SLC31A2), mRNA [NM_001860]	-1.072757	0.003969615

A_24_P194000	*ZNF560*	Homo sapiens zinc finger protein 560 (ZNF560), mRNA [NM_152476]	-0.6330003	0.005052648
A_21_P0010888	*CAND2*	Homo sapiens cullin-associated and neddylation-dissociated 2 (putative) (CAND2), transcript variant 2, mRNA [NM_012298]	-10844011	0.008017601
A_23_P80098	*SAE1*	Homo sapiens SUMO1 activating enzyme subunit 1 (SAE1), transcript variant 1, mRNA [NM_005500]	-0.44998863	0.01150098
A_24_P153576	*NUTM2B-AS1*	Homo sapiens NUTM2B antisense RNA 1 (NUTM2B-AS1), transcript variant 3, long non-coding RNA [NR_120613]	-0.8622032	0.0110383965
A_32_P54475	*GART*	Homo sapiens phosphoribosylglycinamide formyltransferase, phosphoribosylglycinamide synthetase, phosphoribosylaminoimidazole synthetase (GART), transcript variant 1, mRNA [NM_000819]	-0.45985106	0.0070324177
A_33_P3219785	*SHPRH*	Homo sapiens SNF2 histone linker PHD RING helicase, E3 ubiquitin protein ligase (SHPRH), transcript variant 2, mRNA [NM_173082]	-0.7970163	0.006888116
A_33_P3412560	*SOWAHC*	Homo sapiens sosondowah ankyrin repeat domain family member C (SOWAHC), mRNA [NM_023016]	-0.50885177	0.012554233
A_24_P303193	*MRFAP1*	Homo sapiens Morf4 family associated protein 1 (MRFAP1), transcript variant 1, mRNA [NM_033296]	-0.77565163	0.011679733
A_23_P16058	*HNRNPA0*	heterogeneous nuclear ribonucleoprotein A0 [Source:HGNC Symbol;Acc:HGNC:5030] [ENST00000314940]	-0.77608234	0.009761497
A_23_P156748	*CFAP36*	Homo sapiens cilia and flagella associated protein 36 (CFAP36), transcript variant 2, mRNA [NM_080667]	-0.9919328	0.004804688
A_23_P156809	*ZNF296*	Homo sapiens zinc finger protein 296 (ZNF296), mRNA [NM_145288]	1.5500704	0.006490918
A_33_P3209581	*ANKS1A*	Homo sapiens ankyrin repeat and sterile alpha motif domain containing 1A (ANKS1A), mRNA [NM_015245]	-0.93893594	0.013080299
A_21_P0005608	*METTL21A*	Homo sapiens methyltransferase like 21A (METTL21A), transcript variant 2, mRNA [NM_001127395]	1.1427642	0.011416787
A_22_P00011709	*IQSEC3*	Homo sapiens IQ motif and Sec7 domain 3 (IQSEC3), transcript variant 1, mRNA [NM_001170738]	1.3371667	0.0058555133
A_33_P3353791	*LOC101927525*	PREDICTED: Homo sapiens uncharacterized LOC101927525 (LOC101927525), transcript variant X1, ncRNA [XR_242314]	-0.7557709	0.0061700954
A_21_P0002980	*EMX2OS*	Homo sapiens EMX2 opposite strand/antisense RNA (EMX2OS), long non-coding RNA [NR_002791]	-2.0672204	0.0049749194

A_23_P6963	*ITGA1*	Homo sapiens integrin, alpha 1 (ITGA1), mRNA [NM_181501]	-0.6467397	0.0053870557
A_33_P3361636	*LOC100506990*	Homo sapiens uncharacterized LOC100506990 (LOC100506990), transcript variant 2, long non-coding RNA [NR_040092]	-1.1717952	0.011149755
A_33_P3212555	*UBE2E1*	Homo sapiens ubiquitin-conjugating enzyme E2E 1 (UBE2E1), transcript variant 1, mRNA [NM_003341]	-0.9491311	0.0057506533
A_23_P30223	*MGP*	Homo sapiens matrix Gla protein (MGP), transcript variant 1, mRNA [NM_001190839]	-3.5845718	0.009792738
A_33_P3395713	*PROS1*	Homo sapiens protein S (alpha) (PROS1), mRNA [NM_000313]	-1.4701676	0.004437345
A_23_P77779	*SRD5A1*	Homo sapiens steroid-5-alpha-reductase, alpha polypeptide 1 (3-oxo-5 alpha-steroid delta 4-dehydrogenase alpha 1) (SRD5A1), mRNA [NM_001047]	-1.2138357	0.0039659217
A_24_P924202	*BRWD1*	Homo sapiens bromodomain and WD repeat domain containing 1 (BRWD1), transcript variant 1, mRNA [NM_018963]	-1.1646806	0.011745691
A_23_P162879	*RPL19*	Homo sapiens ribosomal protein L19 (RPL19), mRNA [NM_000981]	0.5889606	0.015685366
A_21_P0014801	*PCNXL3*	Homo sapiens pecanex-like 3 (Drosophila) (PCNXL3), mRNA [NM_032223]	0.88449717	0.01456425
A_33_P3244808	*APOPT1*	Homo sapiens apoptogenic 1, mitochondrial (APOPT1), transcript variant 1, mRNA [NM_032374]	-0.640809	0.009630487
A_21_P0010525	*KLHDC4*	kelch domain containing 4 [Source:HGNC Symbol;Acc:HGNC:25272] [ENST00000567298]	-0.4329109	0.014842539
A_23_P213102	*BEST4*	Homo sapiens bestrophin 4 (BEST4), mRNA [NM_153274]	-0.980824	0.00884821
A_33_P3270852	*XLOC_l2_000123*	BROAD Institute lincRNA (XLOC_l2_000123), lincRNA [TCONS_l2_00000181]	-0.3860643	0.0037479755
A_23_P216038	*PALLD*	Homo sapiens palladin, cytoskeletal associated protein (PALLD), transcript variant 2, mRNA [NM_016081]	-0.5182771	0.007840032
A_22_P00006508	*RPS29*	Homo sapiens ribosomal protein S29 (RPS29), transcript variant 1, mRNA [NM_001032]	-0.5203369	0.005920916
A_24_P935491	*PHF20L1*	PHD finger protein 20-like 1 [Source:HGNC Symbol;Acc:HGNC:24280] [ENST00000395383]	-1.9222739	0.0034534817
A_23_P23855	*LOC102723716*	PREDICTED: Homo sapiens uncharacterized LOC102723716 (LOC102723716), ncRNA [XR_426296]	1.4809263	0.0050320392
A_23_P50137	*COL3A1*	Homo sapiens collagen, type III, alpha 1 (COL3A1), mRNA [NM_000090]	-0.5529937	0.00596182
A_24_P309360	*POLR3GL*	Homo sapiens polymerase (RNA) III (DNA directed) polypeptide G (32kD)-like (POLR3GL), mRNA [NM_032305]	-1.2235548	0.0058474606

A_23_P154330	*MEX3C*	Homo sapiens mex-3 RNA binding family member C (MEX3C), mRNA [NM_016626]	-1.1909207	0.008755959
A_33_P6607913	*TM9SF3*	Homo sapiens transmembrane 9 superfamily member 3 (TM9SF3), mRNA [NM_020123]	-0.33104867	0.0025001846
A_24_P402898	*TXNDC9*	Homo sapiens thioredoxin domain containing 9 (TXNDC9), mRNA [NM_005783]	-0.731095	0.0075767385
A_32_P159651	*CWC15*	CWC15 spliceosome-associated protein [Source:HGNC Symbol;Acc:HGNC:26939] [ENST00000542165]	-0.3014701	0.006948726
A_24_P365327	*OTUD4*	Homo sapiens OTU deubiquitinase 4 (OTUD4), transcript variant 3, mRNA [NM_001102653]	-0.42041466	0.014613322
A_23_P398172	*KAT2B*	Homo sapiens K(lysine) acetyltransferase 2B (KAT2B), mRNA [NM_003884]	-1.2019349	0.0033015371
A_33_P3311618	*LSM14A*	Homo sapiens LSM14A, SCD6 homolog A (S. cerevisiae) (LSM14A), transcript variant 2, mRNA [NM_015578]	-0.74710935	0.003609745
A_33_P3392350	*FAM135A*	Homo sapiens family with sequence similarity 135, member A (FAM135A), transcript variant 2, mRNA [NM_020819]	-0.6943306	0.007479662
A_33_P3240538	*RABGGTB*	Homo sapiens Rab geranylgeranyltransferase, beta subunit (RABGGTB), transcript variant 1, mRNA [NM_004582]	-0.6746812	0.0052206037
A_22_P00019592	*TUBE1*	Homo sapiens tubulin, epsilon 1 (TUBE1), mRNA [NM_016262]	-1.3844835	0.0039856653
A_22_P00023815	*FAM171B*	Homo sapiens family with sequence similarity 171, member B (FAM171B), mRNA [NM_177454]	1.386398	0.0040911012
A_23_P254442	*MRPL9*	Homo sapiens mitochondrial ribosomal protein L9 (MRPL9), transcript variant 2, mRNA [NM_001300733]	0.49547008	0.014332605
A_33_P3396891	*LOC155060*	Homo sapiens AI894139 pseudogene (LOC155060), non-coding RNA [NR_036573]	-1.2834988	0.00706395
A_23_P142322	*lnc-CCKAR-1*	LNCipedia lincRNA (lnc-CCKAR-1), lincRNA [lnc-CCKAR-1:2]	1.2546275	0.013050359
A_23_P215214	*AVPI1*	Homo sapiens arginine vasopressin-induced 1 (AVPI1), mRNA [NM_021732]	1.6050336	0.0057806885
A_33_P3318581	*CIRBP*	Homo sapiens cold inducible RNA binding protein (CIRBP), transcript variant 1, mRNA [NM_001280]	-0.5293228	0.005062231
A_23_P3453	*LMBR1*	Homo sapiens limb development membrane protein 1 (LMBR1), mRNA [NM_022458]	-0.6776179	0.005516214
A_33_P3360301	*PLOD2*	Homo sapiens procollagen-lysine, 2-oxoglutarate 5-dioxygenase 2 (PLOD2), transcript variant 1, mRNA [NM_182943]	-0.4804256	0.011656639
A_23_P214977	*RPAP1*	Homo sapiens RNA polymerase II associated protein 1 (RPAP1), mRNA [NM_015540]	-0.5254964	0.016463466

A_32_P52911	*USP19*	Homo sapiens ubiquitin specific peptidase 19 (USP19), transcript variant 2, mRNA [NM_001199161]	-0.7793603	0.014674634
A_33_P3251617	*SEC63*	Homo sapiens SEC63 homolog (S. cerevisiae) (SEC63), mRNA [NM_007214]	-0.91319674	0.0038144474
A_22_P00007635	*ADI1*	Homo sapiens acireductone dioxygenase 1 (ADI1), mRNA [NM_018269]	-0.682836	0.0053998437
A_33_P3375665	*LINC01118*	Homo sapiens long intergenic non-protein coding RNA 1118 (LINC01118), long non-coding RNA [NR_034099]	-1.5272201	0.0059536803
A_23_P250982	*FAM13A-AS1*	Homo sapiens FAM13A antisense RNA 1 (FAM13A-AS1), long non-coding RNA [NR_002806]	-0.94877666	0.006747443
A_24_P416411	*FOXO3*	Homo sapiens forkhead box O3 (FOXO3), transcript variant 1, mRNA [NM_001455]	-0.7408125	0.005860685
A_23_P259189	*ISOC1*	Homo sapiens isochorismatase domain containing 1 (ISOC1), mRNA [NM_016048]	0.42657906	0.004701671
A_22_P00000026	*PEX12*	Homo sapiens peroxisomal biogenesis factor 12 (PEX12), mRNA [NM_000286]	-0.6528253	0.012967146
A_32_P192545	*CLIC4*	Homo sapiens chloride intracellular channel 4 (CLIC4), mRNA [NM_013943]	-0.88593125	0.0031941293
A_21_P0014738	*EMX2OS*	Homo sapiens EMX2 opposite strand/antisense RNA (EMX2OS), long non-coding RNA [NR_002791]	-2.4016166	0.0056962976
A_22_P00021279	*TCEAL6*	Homo sapiens transcription elongation factor A (SII)-like 6 (TCEAL6), mRNA [NM_001006938]	-1.2757373	0.008189544
A_23_P53646	*LOC101930282*	PREDICTED: Homo sapiens uncharacterized LOC101930282 (LOC101930282), ncRNA [XR_250610]	0.8241122	0.009504336
A_33_P3327519	*NAP1L1*	Homo sapiens nucleosome assembly protein 1-like 1 (NAP1L1), transcript variant 1, mRNA [NM_139207]	-0.7522998	0.0029691157
A_24_P358146	*SNORA74A*	Homo sapiens small nucleolar RNA, H/ACA box 74A (SNORA74A), small nucleolar RNA [NR_002915]	0.55986416	0.014658366
A_23_P115011	*MAML1*	mastermind-like 1 (Drosophila) [Source:HGNC Symbol;Acc:HGNC:13632] [ENST00000503050]	0.6492784	0.016359903
A_23_P84910	*ADAMTSL4*	Homo sapiens ADAMTS-like 4 (ADAMTSL4), transcript variant 1, mRNA [NM_019032]	1.2845902	0.0029873664
A_23_P2601	*ASAP2*	Homo sapiens ArfGAP with SH3 domain, ankyrin repeat and PH domain 2 (ASAP2), transcript variant 1, mRNA [NM_003887]	-0.8665943	0.008649646
A_33_P3812669	*ZNF157*	Homo sapiens zinc finger protein 157 (ZNF157), mRNA [NM_003446]	2.0296125	0.008336323
A_23_P40240	*HSP90B1*	Homo sapiens heat shock protein 90kDa beta (Grp94), member 1 (HSP90B1), mRNA [NM_003299]	-0.66743803	0.004160909

A_33_P3320082	*GABARAPL1*	Homo sapiens GABA(A) receptor-associated protein like 1 (GABARAPL1), mRNA [NM_031412]	-0.7756117	0.0070606796
A_33_P3394833	*CTSZ*	Homo sapiens cathepsin Z (CTSZ), mRNA [NM_001336]	-2.0399368	0.007900263
A_24_P373286	*NFIB*	Homo sapiens nuclear factor I/B (NFIB), transcript variant 3, mRNA [NM_005596]	-1.5693977	0.015759172
A_22_P00021971	*FOXL2NB*	Homo sapiens FOXL2 neighbor (FOXL2NB), mRNA [NM_001040061]	-5.0421515	0.006573993
A_32_P389118	*CCP110*	Homo sapiens centriolar coiled coil protein 110kDa (CCP110), transcript variant 2, mRNA [NM_014711]	-0.5477733	0.0122128595
A_33_P3317664	*lnc-EIF4E3-1*	Homo sapiens cDNA FLJ12191 fis, clone MAMMA1000843. [AK022253]	-2.2894444	0.0035935708
A_23_P27005	*HEATR5B*	Homo sapiens HEAT repeat containing 5B (HEATR5B), mRNA [NM_019024]	-0.8636135	0.0051253513
A_24_P245815	*MGAT1*	Homo sapiens mannosyl (alpha-1,3-)-glycoprotein beta-1,2-N-acetylglucosaminyltransferase (MGAT1), transcript variant 1, mRNA [NM_001114618]	0.8185699	0.011575981
A_23_P255215	*DHRS11*	Homo sapiens dehydrogenase/reductase (SDR family) member 11 (DHRS11), mRNA [NM_024308]	1.0533525	0.01040563
A_23_P8664	*ASPHD2*	Homo sapiens aspartate beta-hydroxylase domain containing 2 (ASPHD2), mRNA [NM_020437]	-1.7206163	0.008948642
A_23_P134953	*RP9*	Homo sapiens retinitis pigmentosa 9 (autosomal dominant) (RP9), mRNA [NM_203288]	-1.3702772	0.0072648157
A_21_P0002614	*DMTF1*	Homo sapiens cyclin D binding myb-like transcription factor 1 (DMTF1), transcript variant 1, mRNA [NM_021145]	-0.9109824	0.008811762
A_24_P181101	*PLIN2*	Homo sapiens perilipin 2 (PLIN2), transcript variant 1, mRNA [NM_001122]	-0.8310952	0.010438802
A_21_P0001371	*lnc-LYPD6-1*	LNCipedia lincRNA (lnc-LYPD6-1), lincRNA [lnc-LYPD6-1:2]	0.43756324	0.0118191205
A_33_P3242578	*TMEM135*	Homo sapiens transmembrane protein 135 (TMEM135), transcript variant 1, mRNA [NM_022918]	-0.72770745	0.005752781
A_23_P152344	*ZNF717*	Homo sapiens zinc finger protein 717 (ZNF717), transcript variant 3, mRNA [NM_001290209]	-0.8239964	0.0077888486
A_22_P00001788	*LOC100506731*	PREDICTED: Homo sapiens uncharacterized LOC100506731 (LOC100506731), ncRNA [XR_110231]	-0.576377	0.011376762
A_23_P145463	*GTF3C1*	Homo sapiens general transcription factor IIIC, polypeptide 1, alpha 220kDa (GTF3C1), transcript variant 1, mRNA [NM_001520]	1.5563519	0.009938745

A_33_P3371819	*lnc-ATP4B-1*	Homo sapiens cDNA FLJ39037 fis, clone NT2RP7008544. [AK096356]	-0.44447732	0.011926476
A_21_P0009481	*SLC35B3*	Homo sapiens solute carrier family 35 (adenosine 3'-phospho 5'-phosphosulfate transporter), member B3 (SLC35B3), transcript variant 1, mRNA [NM_015948]	-0.8167535	0.010149606
A_22_P00021328	*PTCD2*	Homo sapiens pentatricopeptide repeat domain 2 (PTCD2), transcript variant 1, mRNA [NM_024754]	-0.8918112	0.016660124
A_23_P135857	*RAB27B*	Homo sapiens RAB27B, member RAS oncogene family (RAB27B), mRNA [NM_004163]	2.075785	0.0054418826
A_33_P3322415	*TSC22D2*	TSC22 domain family, member 2 [Source:HGNC Symbol;Acc:HGNC:29095] [ENST00000361875]	-2.088818	0.006485141
A_33_P3389153	*EIF2AK3*	Homo sapiens eukaryotic translation initiation factor 2-alpha kinase 3 (EIF2AK3), mRNA [NM_004836]	-1.0201628	0.004896935
A_23_P5568	*MARK4*	Homo sapiens MAP/microtubule affinity-regulating kinase 4 (MARK4), transcript variant 2, mRNA [NM_031417]	1.1774145	0.0037705272
A_23_P70047	*STK10*	Homo sapiens serine/threonine kinase 10 (STK10), mRNA [NM_005990]	-1.1954049	0.0045487867
A_23_P3193	*SFT2D3*	Homo sapiens SFT2 domain containing 3 (SFT2D3), mRNA [NM_032740]	-0.7226145	0.007637737
A_33_P3213493	*MATR3*	Homo sapiens matrin 3 (MATR3), transcript variant 1, mRNA [NM_199189]	-0.61524534	0.009135723
A_33_P3385387	*GOLGA5*	Homo sapiens golgin A5 (GOLGA5), mRNA [NM_005113]	-1.0880351	0.002804883
A_33_P3324086	*VAMP2*	Homo sapiens vesicle-associated membrane protein 2 (synaptobrevin 2) (VAMP2), mRNA [NM_014232]	1.4027703	0.004895583
A_24_P194931	*MCUR1*	Homo sapiens mitochondrial calcium uniporter regulator 1 (MCUR1), mRNA [NM_001031713]	-0.8017564	0.005377644
A_33_P3224307	*ZNF532*	Homo sapiens zinc finger protein 532 (ZNF532), mRNA [NM_018181]	-1.0667577	0.0057262382
A_24_P247273	*SMEK2*	Homo sapiens SMEK homolog 2, suppressor of mek1 (Dictyostelium) (SMEK2), transcript variant 2, mRNA [NM_020463]	-0.86956924	0.013441296
A_23_P73747	*SCARNA12*	Homo sapiens small Cajal body-specific RNA 12 (SCARNA12), guide RNA [NR_003010]	-0.93494153	0.008812241
A_33_P3371175	*PRR33*	proline rich 33 [Source:HGNC Symbol;Acc:HGNC:35118] [ENST00000391480]	1.0378612	0.008080026
A_23_P72961	*ARMCX2*	Homo sapiens armadillo repeat containing, X-linked 2 (ARMCX2), transcript variant 2, mRNA [NM_014782]	-1.2096432	0.007777545
A_33_P3381305	*CYP20A1*	Homo sapiens cytochrome P450, family 20, subfamily A, polypeptide 1 (CYP20A1), mRNA [NM_177538]	-1.1940086	0.004723954

A_33_P3239087	*PRPS1*	Homo sapiens phosphoribosyl pyrophosphate synthetase 1 (PRPS1), transcript variant 1, mRNA [NM_002764]	-1.6193788	0.0049172705
A_23_P69720	*FAM87A*	Homo sapiens family with sequence similarity 87, member A (FAM87A), long non-coding RNA [NR_103537]	0.7509502	0.016255163
A_33_P3284686	*ZYG11A*	Homo sapiens zyg-11 family member A, cell cycle regulator (ZYG11A), mRNA [NM_001004339]	1.0637408	0.015520415
A_33_P3260100	*ANXA5*	Homo sapiens annexin A5 (ANXA5), mRNA [NM_001154]	-1.3642755	0.0028791232
A_33_P3406866	*SSBP3*	Homo sapiens single stranded DNA binding protein 3 (SSBP3), transcript variant 1, mRNA [NM_145716]	1.1511892	0.009036186
A_23_P43476	*CCDC167*	Homo sapiens coiled-coil domain containing 167 (CCDC167), mRNA [NM_138493]	-0.9584553	0.011650059
A_33_P3330149	*NFASC*	Homo sapiens neurofascin (NFASC), transcript variant 1, mRNA [NM_001005388]	-1.9922807	0.005154915
A_24_P137376	*VLDLR*	Homo sapiens very low density lipoprotein receptor (VLDLR), transcript variant 1, mRNA [NM_003383]	-0.72659516	0.007464364
A_33_P3326772	*PAX6*	Homo sapiens paired box 6 (PAX6), transcript variant 1, mRNA [NM_000280]	1.985339	0.0042125084
A_33_P3292387	*ATP2C1*	Homo sapiens ATPase, Ca++ transporting, type 2C, member 1 (ATP2C1), transcript variant 3, mRNA [NM_001001485]	-0.80686456	0.0065241847
A_24_P200162	*LAMTOR4*	Homo sapiens late endosomal/lysosomal adaptor, MAPK and MTOR activator 4 (LAMTOR4), mRNA [NM_001008395]	-0.9456107	0.013460425
A_21_P0004312	*LRRIQ3*	Homo sapiens leucine-rich repeats and IQ motif containing 3 (LRRIQ3), mRNA [NM_001105659]	-0.75427854	0.016077448
A_33_P3388588	*HIGD1A*	Homo sapiens HIG1 hypoxia inducible domain family, member 1A (HIGD1A), transcript variant 3, mRNA [NM_014056]	-0.7960252	0.0029675725
A_23_P111452	*MIR143HG*	MIR143 host gene (non-protein coding) [Source:HGNC Symbol;Acc:HGNC:42872] [ENST00000519898]	-2.6953926	0.003162355
A_23_P77310	*LOC400558*	Homo sapiens uncharacterized LOC400558 (LOC400558), long non-coding RNA [NR_046200]	0.6724906	0.009697046
A_22_P00009654	*AGAP3*	Homo sapiens ArfGAP with GTPase domain, ankyrin repeat and PH domain 3 (AGAP3), transcript variant 1, mRNA [NM_031946]	-0.6513099	0.01009471
A_23_P348257	*ZNF106*	Homo sapiens zinc finger protein 106 (ZNF106), transcript variant 1, mRNA [NM_022473]	0.4642022	0.005947757

A_23_P368645	*lnc-MAT2B-3*	Homo sapiens, clone IMAGE:4479080, mRNA, partial cds. [BC011998]	1.3045529	0.00558687
A_23_P214144	*NUAK1*	Homo sapiens NUAK family, SNF1-like kinase, 1 (NUAK1), mRNA [NM_014840]	-1.4962246	0.0029497955
A_32_P352358	*AGPS*	Homo sapiens alkylglycerone phosphate synthase (AGPS), mRNA [NM_003659]	-0.32011342	0.011087365
A_33_P3318841	*COL10A1*	Homo sapiens collagen, type X, alpha 1 (COL10A1), mRNA [NM_000493]	2.0339508	0.0037305711
A_24_P943922	*LOC650293*	Homo sapiens seven transmembrane helix receptor (LOC650293), mRNA [NM_001040071]	-1.5477515	0.005512448
A_23_P122852	*C5orf63*	Homo sapiens chromosome 5 open reading frame 63 (C5orf63), transcript variant 1, mRNA [NM_001164479]	-0.69240093	0.012773474
A_23_P202071	*CACHD1*	Homo sapiens cache domain containing 1 (CACHD1), transcript variant 1, mRNA [NM_020925]	-0.7657872	0.006632856
A_32_P134290	*SMARCD3*	Homo sapiens SWI/SNF related, matrix associated, actin dependent regulator of chromatin, subfamily d, member 3 (SMARCD3), transcript variant 2, mRNA [NM_003078]	-0.5895388	0.015534843
A_24_P167642	*CELF2*	Homo sapiens CUGBP, Elav-like family member 2 (CELF2), transcript variant 3, mRNA [NM_001025077]	-1.4821655	0.005985026
A_23_P152305	*ZCCHC2*	Homo sapiens zinc finger, CCHC domain containing 2 (ZCCHC2), transcript variant 1, mRNA [NM_017742]	-1.0030057	0.0063259676
A_33_P3416124	*GCH1*	Homo sapiens GTP cyclohydrolase 1 (GCH1), transcript variant 1, mRNA [NM_000161]	-0.64127266	0.013800824
A_22_P00008088	*CDH11*	Homo sapiens cadherin 11, type 2, OB-cadherin (osteoblast) (CDH11), mRNA [NM_001797]	-1.0567724	0.0056355232
A_23_P363831	*LOC100130872*	Homo sapiens uncharacterized LOC100130872 (LOC100130872), long non-coding RNA [NR_024569]	1.077204	0.006573993
A_33_P3258013	*lnc-IL15RA-1*	AGENCOURT_6621625 NIH_MGC_125 Homo sapiens cDNA clone IMAGE:5722280 5', mRNA sequence [BM906553]	0.36039594	0.011604
A_23_P100292	*FBXO3*	Homo sapiens F-box protein 3 (FBXO3), transcript variant 2, mRNA [NM_033406]	-0.8342459	0.010203489
A_32_P123629	*UBE3B*	Homo sapiens ubiquitin protein ligase E3B (UBE3B), transcript variant 4, mRNA [NM_001270449]	-0.8248152	0.015534843
A_33_P3260623	*ZNF598*	Homo sapiens zinc finger protein 598 (ZNF598), mRNA [NM_178167]	-0.71843505	0.010791786
A_23_P88095	*TTC39C*	Homo sapiens tetratricopeptide repeat domain 39C (TTC39C), transcript variant 4, mRNA [NM_001292030]	-1.0020161	0.008090729

A_33_P3403748	*PLCB3*	Homo sapiens phospholipase C, beta 3 (phosphatidylinositol-specific) (PLCB3), transcript variant 1, mRNA [NM_000932]	0.9840795	0.012346
A_33_P3416503	*TBC1D4*	Homo sapiens TBC1 domain family, member 4 (TBC1D4), transcript variant 1, mRNA [NM_014832]	-1.0107746	0.0067707044
A_33_P3419419	*UQCC3*	Homo sapiens ubiquinol-cytochrome c reductase complex assembly factor 3 (UQCC3), mRNA [NM_001085372]	-0.7270947	0.0065241847
A_23_P70670	*NUDT8*	Homo sapiens nudix (nucleoside diphosphate linked moiety X)-type motif 8 (NUDT8), transcript variant 2, mRNA [NM_181843]	0.73670083	0.00968922
A_23_P118061	*PIK3C2B*	phosphatidylinositol-4-phosphate 3-kinase, catalytic subunit type 2 beta [Source:HGNC Symbol;Acc:HGNC:8972] [ENST00000367184]	0.5796193	0.010827594
A_24_P655849	*CD83*	Homo sapiens CD83 molecule (CD83), transcript variant 1, mRNA [NM_004233]	0.99740994	0.006155321
A_33_P3350643	*CKLF*	Homo sapiens chemokine-like factor (CKLF), transcript variant 4, mRNA [NM_181641]	-0.5871756	0.0067226742
A_23_P252740	*SMAD9*	Homo sapiens SMAD family member 9 (SMAD9), transcript variant a, mRNA [NM_001127217]	-1.0107263	0.0072350414
A_23_P93464	*LMX1B*	Homo sapiens LIM homeobox transcription factor 1, beta (LMX1B), transcript variant 3, mRNA [NM_001174146]	1.0651579	0.013353969
A_22_P00001341	*DSCC1*	Homo sapiens DNA replication and sister chromatid cohesion 1 (DSCC1), mRNA [NM_024094]	-0.6741825	0.0072139124
A_22_P00001583	*BCKDHB*	Homo sapiens branched chain keto acid dehydrogenase E1, beta polypeptide (BCKDHB), transcript variant 2, mRNA [NM_000056]	-1.5191706	0.0038487043
A_33_P3394933	*MIR612*	Homo sapiens microRNA 612 (MIR612), microRNA [NR_030343]	0.7132691	0.013369118
A_22_P00006790	*lnc-ARL6IP5-1*	RST28380 Athersys RAGE Library Homo sapiens cDNA, mRNA sequence [BG208742]	0.41233325	0.01039718
A_33_P3216232	*P4HA2*	Homo sapiens prolyl 4-hydroxylase, alpha polypeptide II (P4HA2), transcript variant 2, mRNA [NM_001017973]	-0.8253328	0.009794768
A_23_P67785	*lnc-FZD4-1*	LNCipedia lincRNA (lnc-FZD4-1), lincRNA [lnc-FZD4-1:1]	-1.5577248	0.0053998437
A_33_P3374643	*ITGB1BP1*	Homo sapiens integrin beta 1 binding protein 1 (ITGB1BP1), transcript variant 1, mRNA [NM_004763]	-1.5078261	0.009924993
A_24_P386334	*SPAG16*	Homo sapiens sperm associated antigen 16 (SPAG16), transcript variant 2, mRNA [NM_001025436]	-0.78436875	0.0074376203

A_33_P3329740	*PAAF1*	Homo sapiens proteasomal ATPase-associated factor 1 (PAAF1), transcript variant 1, mRNA [NM_001267803]	-0.6605597	0.010851504
A_33_P3240328	*SH3GLB2*	Homo sapiens SH3-domain GRB2-like endophilin B2 (SH3GLB2), transcript variant 2, mRNA [NM_020145]	1.641912	0.0065033673
A_19_P00321259	*PITX1*	Homo sapiens paired-like homeodomain 1 (PITX1), mRNA [NM_002653]	1.7338912	0.0058377855
A_22_P00003809	*S100A4*	Homo sapiens S100 calcium binding protein A4 (S100A4), transcript variant 1, mRNA [NM_002961]	-0.23783737	0.003381037
A_33_P3424861	*LINC01133*	Homo sapiens long intergenic non-protein coding RNA 1133 (LINC01133), long non-coding RNA [NR_038849]	-3.0421736	0.0029471128
A_19_P00804777	*lnc-CDKN2C-1*	LNCipedia lincRNA (lnc-CDKN2C-1), lincRNA [lnc-CDKN2C-1:2]	-1.398077	0.0055582817
A_23_P21363	*FAM118A*	Homo sapiens family with sequence similarity 118, member A (FAM118A), transcript variant 1, mRNA [NM_001104595]	2.88762	0.0050769406
A_23_P342668	*LINC00662*	long intergenic non-protein coding RNA 662 [Source:HGNC Symbol;Acc:HGNC:27122] [ENST00000586954]	-1.3403206	0.00477571
A_33_P3590279	*AHNAK*	Homo sapiens AHNAK nucleoprotein (AHNAK), transcript variant 2, mRNA [NM_024060]	-1.4993505	0.0031941293
A_33_P3308534	*AKAP17A*	Homo sapiens A kinase (PRKA) anchor protein 17A (AKAP17A), transcript variant 1, mRNA [NM_005088]	-0.7584479	0.01083596
A_33_P3390853	*OSBPL1A*	Homo sapiens oxysterol binding protein-like 1A (OSBPL1A), transcript variant 2, mRNA [NM_080597]	-0.81625515	0.012094951
A_32_P50522	*LOC101928714*	PREDICTED: Homo sapiens uncharacterized LOC101928714 (LOC101928714), ncRNA [XR_241865]	-0.9504192	0.016780324
A_24_P201064	*LINC01420*	Homo sapiens long intergenic non-protein coding RNA 1420 (LINC01420), long non-coding RNA [NR_015367]	-0.8474025	0.00711267
A_23_P96965	*FKBP1A*	Homo sapiens FK506 binding protein 1A, 12kDa (FKBP1A), transcript variant 1, mRNA [NM_000801]	1.1067436	0.0042908904
A_23_P36183	*PPP1R3B*	Homo sapiens protein phosphatase 1, regulatory subunit 3B (PPP1R3B), transcript variant 1, mRNA [NM_001201329]	-1.7261751	0.0024890031
A_32_P352697	*SYNC*	Homo sapiens syncoilin, intermediate filament protein (SYNC), transcript variant 1, mRNA [NM_030786]	-1.232593	0.003897398
A_23_P137470	*GTF2H1*	Homo sapiens general transcription factor IIH, polypeptide 1, 62kDa (GTF2H1), transcript variant 1, mRNA [NM_005316]	-0.6930728	0.0064385477

A_33_P3231156	*YTHDC1*	Homo sapiens YTH domain containing 1 (YTHDC1), transcript variant 1, mRNA [NM_001031732]	-1.1800213	0.0059911786
A_23_P256391	*SIPA1L2*	Homo sapiens signal-induced proliferation-associated 1 like 2 (SIPA1L2), mRNA [NM_020808]	-1.8302289	0.00477571
A_23_P308097	*GOLGA4*	Homo sapiens golgin A4 (GOLGA4), transcript variant 2, mRNA [NM_002078]	-0.33627036	0.015442503
A_33_P3365596	*EBP*	Homo sapiens emopamil binding protein (sterol isomerase) (EBP), mRNA [NM_006579]	-0.43617243	0.009425872
A_24_P259154	*PPP2CB*	Homo sapiens protein phosphatase 2, catalytic subunit, beta isozyme (PPP2CB), mRNA [NM_001009552]	-0.5278967	0.016512543
A_23_P149121	*NAPB*	Homo sapiens N-ethylmaleimide-sensitive factor attachment protein, beta (NAPB), transcript variant 1, mRNA [NM_001283018]	-1.3611089	0.004169345
A_24_P141214	*TUBA3FP*	Homo sapiens tubulin, alpha 3f, pseudogene (TUBA3FP), non-coding RNA [NR_003608]	0.97671896	0.014822511
A_23_P142560	*DIRAS3*	Homo sapiens DIRAS family, GTP-binding RAS-like 3 (DIRAS3), mRNA [NM_004675]	2.0290074	0.00324898
A_24_P940921	*STOM*	Homo sapiens stomatin (STOM), transcript variant 2, mRNA [NM_198194]	-1.7468145	0.00584425
A_24_P365901	*ZEB2*	Homo sapiens zinc finger E-box binding homeobox 2 (ZEB2), transcript variant 1, mRNA [NM_014795]	-1.769381	0.0022181747
A_33_P3268783	*SETX*	Homo sapiens senataxin (SETX), mRNA [NM_015046]	-0.92529464	0.008559404
A_23_P158007	*TSPAN33*	Homo sapiens tetraspanin 33 (TSPAN33), mRNA [NM_178562]	0.80797935	0.009378251
A_23_P259874	*AMZ1*	Homo sapiens archaelysin family metallopeptidase 1 (AMZ1), transcript variant 1, mRNA [NM_133463]	1.9576349	0.008208044
A_23_P105730	*ZDHHC12*	Homo sapiens zinc finger, DHHC-type containing 12 (ZDHHC12), mRNA [NM_032799]	0.8083	0.016256094
A_24_P84048	*SEC22A*	Homo sapiens SEC22 vesicle trafficking protein homolog A (S. cerevisiae) (SEC22A), mRNA [NM_012430]	-0.972671	0.0034534817
A_23_P256205	*MDM1*	Homo sapiens Mdm1 nuclear protein homolog (mouse) (MDM1), transcript variant 2, mRNA [NM_020128]	-0.8486129	0.015843209
A_21_P0009580	*ABLIM3*	Homo sapiens actin binding LIM protein family, member 3 (ABLIM3), transcript variant 2, mRNA [NM_014945]	0.28731698	0.016236924
A_22_P00011248	*RUNX3*	Homo sapiens runt-related transcription factor 3 (RUNX3), transcript variant 1, mRNA [NM_001031680]	-3.2770348	0.0031941293
A_24_P405298	*lnc-FBXO15-3*	LNCipedia lincRNA (lnc-FBXO15-3), lincRNA [lnc-FBXO15-3:3]	1.1252079	0.012042751

A_33_P3288805	*lnc-OR1Q1-1*	603073365F1 NIH_MGC_119 Homo sapiens cDNA clone IMAGE:5165105 5', mRNA sequence [BI830485]	1.191797	0.00942855
A_23_P392126	*PPP1CB*	Homo sapiens protein phosphatase 1, catalytic subunit, beta isozyme (PPP1CB), transcript variant 1, mRNA [NM_002709]	-0.9101786	0.0036517929
A_33_P3879920	*KLC2*	Homo sapiens kinesin light chain 2 (KLC2), transcript variant 2, mRNA [NM_001134774]	1.757214	0.009938745
A_33_P3272698	*LYRM9*	Homo sapiens LYR motif containing 9 (LYRM9), mRNA [NM_001076680]	-1.5745368	0.004155979
A_24_P396720	*TRIM39-RPP21*	Homo sapiens TRIM39-RPP21 readthrough (TRIM39-RPP21), mRNA [NM_001199119]	-0.73139477	0.010057722
A_24_P12626	*DUSP23*	Homo sapiens dual specificity phosphatase 23 (DUSP23), mRNA [NM_017823]	-1.1326623	0.0044240346
A_23_P147255	*PPP1CB*	Homo sapiens protein phosphatase 1, catalytic subunit, beta isozyme (PPP1CB), transcript variant 1, mRNA [NM_002709]	-0.6988999	0.011656639
A_33_P3252093	*CAV1*	Homo sapiens caveolin 1, caveolae protein, 22kDa (CAV1), transcript variant 1, mRNA [NM_001753]	-1.3163688	0.003363001
A_33_P3368453	*PCBP3*	Homo sapiens poly(rC) binding protein 3 (PCBP3), transcript variant 1, mRNA [NM_020528]	0.562436	0.015049158
A_33_P3807593	*TMED10*	Homo sapiens transmembrane emp24-like trafficking protein 10 (yeast) (TMED10), mRNA [NM_006827]	-0.95836896	0.0032831472
A_24_P298360	*MICA*	Homo sapiens MHC class I polypeptide-related sequence A (MICA), transcript variant 1*001, mRNA [NM_000247]	-0.9171319	0.008897602
A_23_P89310	*LOC286437*	Homo sapiens uncharacterized LOC286437 (LOC286437), long non-coding RNA [NR_039980]	-1.465523	0.010618303
A_23_P212383	*LTBP3*	Homo sapiens latent transforming growth factor beta binding protein 3 (LTBP3), transcript variant 2, mRNA [NM_021070]	0.7731068	0.0057247286
A_33_P3667484	*EPN2*	Homo sapiens epsin 2 (EPN2), transcript variant 2, mRNA [NM_014964]	-0.63538885	0.012452464
A_33_P3271126	*SACM1L*	Homo sapiens SAC1 suppressor of actin mutations 1-like (yeast) (SACM1L), mRNA [NM_014016]	-0.8126103	0.0044240346
A_21_P0007761	*ACTG1P4*	Homo sapiens actin gamma 1 pseudogene 4 (ACTG1P4), non-coding RNA [NR_024438]	-2.6655285	0.004359316
A_33_P3244669	*MAPKAPK5-AS1*	Homo sapiens MAPKAPK5 antisense RNA 1 (MAPKAPK5-AS1), long non-coding RNA [NR_015404]	-0.5350209	0.014625823
A_23_P135616	*AKR1C1*	Homo sapiens aldo-keto reductase family 1, member C1 (AKR1C1), mRNA [NM_001353]	0.8819077	0.010687508

A_22_P00020011	*TMEM30A*	Homo sapiens transmembrane protein 30A (TMEM30A), transcript variant 1, mRNA [NM_018247]	-0.8663235	0.0067682164
A_23_P205046	*STX18*	Homo sapiens syntaxin 18 (STX18), mRNA [NM_016930]	-0.6960651	0.013664689
A_33_P3261828	*ANKRD10*	Homo sapiens ankyrin repeat domain 10 (ANKRD10), transcript variant 1, mRNA [NM_017664]	-0.79061735	0.006515337
A_33_P3289561	*PLCL2*	Homo sapiens phospholipase C-like 2 (PLCL2), transcript variant 2, mRNA [NM_015184]	-1.3497875	0.006020368
A_23_P20463	*ADAMTS7P1*	Homo sapiens ADAMTS7 pseudogene 1 (ADAMTS7P1), non-coding RNA [NR_045529]	-1.718841	0.010716798
A_21_P0014392	*RAD21*	Homo sapiens RAD21 homolog (S. pombe) (RAD21), mRNA [NM_006265]	-0.5572955	0.010369088
A_32_P28402	*ORAI3*	Homo sapiens ORAI calcium release-activated calcium modulator 3 (ORAI3), mRNA [NM_152288]	-0.84753567	0.0106436545
A_22_P00008012	*ZNF330*	Homo sapiens zinc finger protein 330 (ZNF330), transcript variant 1, mRNA [NM_014487]	-1.3796875	0.005842833
A_33_P3317321	*LRRC37BP1*	Homo sapiens leucine rich repeat containing 37B pseudogene 1 (LRRC37BP1), non-coding RNA [NR_015341]	-0.9395132	0.0039522685
A_23_P342709	*SEMA3B-AS1*	Homo sapiens SEMA3B antisense RNA 1 (head to head) (SEMA3B-AS1), long non-coding RNA [NR_110702]	-1.604648	0.0037685214
A_22_P00001017	*TPMT*	Homo sapiens thiopurine S-methyltransferase (TPMT), mRNA [NM_000367]	-1.0632148	0.0035238392
A_23_P218476	*FBXO15*	Homo sapiens F-box protein 15 (FBXO15), transcript variant 1, mRNA [NM_152676]	0.51481134	0.009560995
A_21_P0011898	*LINC00842*	Homo sapiens long intergenic non-protein coding RNA 842 (LINC00842), long non-coding RNA [NR_033957]	0.70803213	0.010612081
A_24_P98555	*B9D2*	Homo sapiens B9 protein domain 2 (B9D2), mRNA [NM_030578]	-0.7355089	0.0102766305
A_23_P33364	*FAM45A*	Homo sapiens family with sequence similarity 45, member A (FAM45A), transcript variant 2, mRNA [NM_001303111]	-0.74474454	0.010871661
A_19_P00322166	*MARCKSL1*	Homo sapiens MARCKS-like 1 (MARCKSL1), transcript variant 1, mRNA [NM_023009]	1.0500771	0.005255513
A_24_P101101	*SH3D19*	Homo sapiens SH3 domain containing 19 (SH3D19), transcript variant 1, mRNA [NM_001009555]	-0.82750773	0.005303325
A_33_P3285470	*GDNF-AS1*	Homo sapiens GDNF antisense RNA 1 (head to head) (GDNF-AS1), transcript variant 1, mRNA [NM_001278094]	-1.7755219	0.0046024295
A_23_P4611	*BAI1*	Homo sapiens brain-specific angiogenesis inhibitor 1 (BAI1), mRNA [NM_001702]	-0.824108	0.01302682

A_33_P3420816	*AKR1C3*	Homo sapiens aldo-keto reductase family 1, member C3 (AKR1C3), transcript variant 1, mRNA [NM_003739]	-0.6736219	0.010871661
A_33_P3239587	*SLC27A5*	Homo sapiens solute carrier family 27 (fatty acid transporter), member 5 (SLC27A5), mRNA [NM_012254]	-0.8246584	0.010284574
A_33_P3332492	*GDF1*	Homo sapiens growth differentiation factor 1 (GDF1), mRNA [NM_001492]	1.236372	0.012477598
A_24_P500422	*MXRA7*	Homo sapiens matrix-remodelling associated 7 (MXRA7), transcript variant 2, mRNA [NM_001008529]	-0.8206115	0.002909564
A_23_P130343	*FANK1*	Homo sapiens fibronectin type III and ankyrin repeat domains 1 (FANK1), mRNA [NM_145235]	-1.9088377	0.0068240776
A_24_P347480	*LOC730101*	Homo sapiens uncharacterized LOC730101 (LOC730101), transcript variant 1, long non-coding RNA [NR_024403]	-1.2351973	0.0029471128
A_33_P3227944	*KCTD1*	Homo sapiens potassium channel tetramerization domain containing 1 (KCTD1), transcript variant 2, mRNA [NM_198991]	-0.5475888	0.010833967
A_23_P166616	*NEK9*	Homo sapiens NIMA-related kinase 9 (NEK9), mRNA [NM_033116]	-1.0291606	0.0065241847
A_33_P3216442	*CRHR1-IT1*	Homo sapiens CRHR1 intronic transcript 1 (non-protein coding) (CRHR1-IT1), transcript variant 2, long non-coding RNA [NR_026906]	2.50286	0.005822303
A_22_P00012145	*AGTR1*	Homo sapiens angiotensin II receptor, type 1 (AGTR1), transcript variant 4, mRNA [NM_031850]	-2.6821122	0.0054135993
A_33_P3239849	*COL11A2*	Homo sapiens collagen, type XI, alpha 2 (COL11A2), transcript variant 1, mRNA [NM_080680]	-0.74663967	0.011409725
A_24_P156490	*lnc-POLR3E-2*	Homo sapiens hypothetical LOC440346, mRNA (cDNA clone IMAGE:5265545). [BC035163]	-0.92498714	0.017078122
A_23_P26704	*GPX1*	Homo sapiens glutathione peroxidase 1 (GPX1), transcript variant 2, mRNA [NM_201397]	-1.1638646	0.0062257075
A_23_P63432	*KCNMA1*	Homo sapiens potassium channel, calcium activated large conductance subfamily M alpha, member 1 (KCNMA1), transcript variant 2, mRNA [NM_002247]	-0.9263778	0.0052084886
A_23_P135787	*MFSD11*	Homo sapiens major facilitator superfamily domain containing 11 (MFSD11), transcript variant 5, mRNA [NM_024311]	-1.0533079	0.0050201877
A_23_P125078	*RHBDL2*	Homo sapiens rhomboid, veinlet-like 2 (Drosophila) (RHBDL2), mRNA [NM_017821]	-0.9192184	0.015589794
A_33_P3236632	*GOLGB1*	Homo sapiens golgin B1 (GOLGB1), transcript variant 3, mRNA [NM_001256487]	0.78265655	0.0023340243

A_23_P218579	*SLC26A11*	Homo sapiens solute carrier family 26 (anion exchanger), member 11 (SLC26A11), transcript variant 2, mRNA [NM_173626]	-0.8010078	0.009276546
A_33_P3366987	*MIA3*	Homo sapiens melanoma inhibitory activity family, member 3 (MIA3), transcript variant 1, mRNA [NM_198551]	0.63748235	0.0149709545
A_21_P0000305	*GLB1L*	Homo sapiens galactosidase, beta 1-like (GLB1L), transcript variant 2, mRNA [NM_024506]	1.2105947	0.012350057
A_23_P118174	*GPR125*	Homo sapiens G protein-coupled receptor 125 (GPR125), mRNA [NM_145290]	1.4160097	0.0047066417
A_24_P246467	*PDCL3*	Homo sapiens phosducin-like 3 (PDCL3), mRNA [NM_024065]	-0.87780166	0.0040527363
A_23_P107211	*PLK1*	polo-like kinase 1 [Source:HGNC Symbol;Acc:HGNC:9077] [ENST00000562272]	1.5541265	0.0049312185
A_33_P3242508	*ATF2*	Homo sapiens activating transcription factor 2 (ATF2), transcript variant 1, mRNA [NM_001880]	-0.7780679	0.009731291
A_19_P00805950	*RAB5C*	Homo sapiens RAB5C, member RAS oncogene family (RAB5C), transcript variant 1, mRNA [NM_201434]	0.7964153	0.0059664235
A_21_P0014786	*ZNF365*	Homo sapiens zinc finger protein 365 (ZNF365), transcript variant B, mRNA [NM_199450]	1.7023892	0.016991401
A_33_P3344399	*LOC101928673*	Homo sapiens uncharacterized LOC101928673 (LOC101928673), long non-coding RNA [NR_125960]	0.83949655	0.0048260237
A_23_P200298	*LOC100507663*	Homo sapiens cDNA FLJ26385 fis, clone HRT07145. [AK129895]	-0.9114944	0.010971502
A_23_P206228	*LRRC49*	Homo sapiens leucine rich repeat containing 49 (LRRC49), transcript variant 3, mRNA [NM_001199018]	-0.57941425	0.011454393
A_19_P00810085	*AGL*	Homo sapiens amylo-alpha-1, 6-glucosidase, 4-alpha-glucanotransferase (AGL), transcript variant 4, mRNA [NM_000028]	-1.1904665	0.008768279
A_33_P3278293	*VPS13C*	Homo sapiens vacuolar protein sorting 13 homolog C (S. cerevisiae) (VPS13C), transcript variant 2A, mRNA [NM_020821]	-0.9326121	0.0054341624
A_24_P232790	*LINC00607*	long intergenic non-protein coding RNA 607 [Source:HGNC Symbol;Acc:HGNC:43944] [ENST00000419922]	-2.419065	0.0031941293
A_23_P431853	*METTL16*	Homo sapiens methyltransferase like 16 (METTL16), mRNA [NM_024086]	-0.55968237	0.009630487
A_21_P0008851	*CCDC177*	Homo sapiens coiled-coil domain containing 177 (CCDC177), mRNA [NM_001271507]	1.0499665	0.007697845
A_23_P59616	*ND2*	mitochondrially encoded NADH dehydrogenase 2 [Source:HGNC Symbol;Acc:HGNC:7456] [ENST00000361453]	1.5307956	0.0059872055

A_22_P00013710	*lnc-ITGA11-2*	LNCipedia lincRNA (lnc-ITGA11-2), lincRNA [lnc-ITGA11-2:3]	0.856939	0.012893396
A_33_P3323101	*GTF2IRD2*	Homo sapiens GTF2I repeat domain containing 2 (GTF2IRD2), transcript variant 1, mRNA [NM_173537]	-0.9665426	0.0145678455
A_33_P3265224	*HAS2-AS1*	Homo sapiens HAS2 antisense RNA 1 (HAS2-AS1), antisense RNA [NR_002835]	-1.2580972	0.006472185
A_33_P3422679	*ZNF33A*	Homo sapiens zinc finger protein 33A (ZNF33A), transcript variant 4, mRNA [NM_001278171]	-0.83407795	0.015770173
A_24_P641130	*CEP68*	centrosomal protein 68kDa [Source:HGNC Symbol;Acc:HGNC:29076] [ENST00000537589]	-1.1498533	0.0062257075
A_22_P00014756	*FAM149B1*	Homo sapiens family with sequence similarity 149, member B1 (FAM149B1), mRNA [NM_173348]	-1.5935876	0.0044240346
A_23_P96542	*PRRC2A*	Homo sapiens proline-rich coiled-coil 2A (PRRC2A), transcript variant 1, mRNA [NM_080686]	1.7070936	0.0038456172
A_24_P203710	*LOC101060091*	Homo sapiens uncharacterized LOC101060091 (LOC101060091), long non-coding RNA [NR_110174]	-1.3288348	0.00641094
A_33_P3276222	*VMA21*	Homo sapiens VMA21 vacuolar H+-ATPase homolog (S. cerevisiae) (VMA21), mRNA [NM_001017980]	-0.67933255	0.008437608
A_23_P370924	*FUBP3*	Homo sapiens far upstream element (FUSE) binding protein 3 (FUBP3), mRNA [NM_003934]	-0.6441006	0.0031166414
A_23_P153098	*SMIM4*	Homo sapiens small integral membrane protein 4 (SMIM4), mRNA [NM_001124767]	-0.8012483	0.0047719665
A_33_P3339051	*DCP1B*	Homo sapiens decapping mRNA 1B (DCP1B), mRNA [NM_152640]	-1.1219032	0.0067226742
A_22_P00003781	*HDHD2*	Homo sapiens haloacid dehalogenase-like hydrolase domain containing 2 (HDHD2), mRNA [NM_032124]	-0.9735842	0.0044519836
A_23_P104996	*ZNF428*	Homo sapiens zinc finger protein 428 (ZNF428), mRNA [NM_182498]	-1.206631	0.0029471128
A_24_P854913	*CDK3*	Homo sapiens cyclin-dependent kinase 3 (CDK3), mRNA [NM_001258]	0.97390676	0.008366524
A_24_P212127	*BEST1*	Homo sapiens bestrophin 1 (BEST1), transcript variant 1, mRNA [NM_004183]	-0.90875685	0.0058047334
A_23_P213166	*METTL21A*	Homo sapiens methyltransferase like 21A (METTL21A), transcript variant 2, mRNA [NM_001127395]	-0.7862706	0.013970144
A_23_P138693	*ZNF33A*	Homo sapiens zinc finger protein 33A (ZNF33A), transcript variant 1, mRNA [NM_006954]	-0.62583566	0.0081642615
A_23_P208009	*ZGRF1*	Homo sapiens zinc finger, GRF-type containing 1 (ZGRF1), mRNA [NM_018392]	-0.19848222	0.015313101

A_33_P3367126	*NMT2*	Homo sapiens N-myristoyltransferase 2 (NMT2), mRNA [NM_004808]	-0.7839854	0.0054708454
A_23_P107073	*SEC11C*	Homo sapiens SEC11 homolog C (S. cerevisiae) (SEC11C), mRNA [NM_033280]	-1.1252172	0.0029691157
A_33_P3327200	*ARHGEF28*	Homo sapiens Rho guanine nucleotide exchange factor (GEF) 28 (ARHGEF28), transcript variant 2, mRNA [NM_001177693]	2.7975774	0.0037353234
A_23_P416581	*RPA1*	Homo sapiens replication protein A1, 70kDa (RPA1), mRNA [NM_002945]	-1.0062913	0.006573993
A_22_P00017019	*ATP2B4*	Homo sapiens ATPase, Ca++ transporting, plasma membrane 4 (ATP2B4), transcript variant 1, mRNA [NM_001001396]	-1.1288292	0.008536579
A_22_P00012594	*GNAZ*	Homo sapiens guanine nucleotide binding protein (G protein), alpha z polypeptide (GNAZ), mRNA [NM_002073]	2.5401168	0.0020435157
A_23_P128783	*lnc-TTC40-3*	LNCipedia lincRNA (lnc-TTC40-3), lincRNA [lnc-TTC40-3:7]	1.8315467	0.015238087
A_22_P00006381	*SNUPN*	Homo sapiens snurportin 1 (SNUPN), transcript variant 1, mRNA [NM_005701]	-0.6941483	0.01211404
A_24_P135902	*EAPP*	Homo sapiens E2F-associated phosphoprotein (EAPP), mRNA [NM_018453]	-0.9483876	0.009433551
A_21_P0000591	*RPS2*	Homo sapiens ribosomal protein S2 (RPS2), mRNA [NM_002952]	-0.40197462	0.012873199
A_21_P0000370	*LIAS*	Homo sapiens lipoic acid synthetase (LIAS), transcript variant 1, mRNA [NM_006859]	-0.81616235	0.015484922
A_24_P189533	*SNORA70F*	Homo sapiens small nucleolar RNA, H/ACA box 70F (SNORA70F), small nucleolar RNA [NR_033309]	0.52675164	0.00856985
A_33_P3318763	*SNORD6*	Homo sapiens small nucleolar RNA, C/D box 6 (SNORD6), small nucleolar RNA [NR_003036]	-0.83055377	0.0033015371
A_23_P128641	*ENDOD1*	Homo sapiens endonuclease domain containing 1 (ENDOD1), mRNA [NM_015036]	-0.98776364	0.013450244
A_33_P3415617	*LOC101927497*	Homo sapiens uncharacterized LOC101927497 (LOC101927497), transcript variant 1, long non-coding RNA [NR_110086]	-2.4428587	0.0050880876
A_22_P00019311	*USPL1*	Homo sapiens ubiquitin specific peptidase like 1 (USPL1), mRNA [NM_005800]	-0.66906434	0.014173936
A_24_P827491	*lnc-MARVELD3-2*	LNCipedia lincRNA (lnc-MARVELD3-2), lincRNA [lnc-MARVELD3-2:1]	0.78097564	0.004094892
A_33_P3351955	*KLHDC7B*	Homo sapiens kelch domain containing 7B (KLHDC7B), mRNA [NM_138433]	1.7413257	0.009468359
A_24_P344961	*PA2G4*	Homo sapiens proliferation-associated 2G4, 38kDa (PA2G4), mRNA [NM_006191]	0.06849638	0.013944771
A_24_P372625	*EGFR*	Homo sapiens epidermal growth factor receptor (EGFR), transcript variant 2, mRNA [NM_201282]	0.89062357	0.007451174
A_33_P3264955	*AMOT*	Homo sapiens angiomotin (AMOT), transcript variant 2, mRNA [NM_133265]	-0.77440673	0.007370545

A_23_P254863	*RNF141*	Homo sapiens ring finger protein 141 (RNF141), mRNA [NM_016422]	-1.0653276	0.0072506047
A_23_P139958	*NIM1K*	Homo sapiens NIM1 serine/threonine protein kinase (NIM1K), transcript variant 1, mRNA [NM_153361]	-1.0790232	0.012040703
A_23_P126266	*RAB11FIP5*	Homo sapiens RAB11 family interacting protein 5 (class I) (RAB11FIP5), mRNA [NM_015470]	-0.8305312	0.010042705
A_23_P70583	*CDK8*	Homo sapiens cyclin-dependent kinase 8 (CDK8), mRNA [NM_001260]	-0.5148018	0.011207995
A_22_P00007786	*HLX*	Homo sapiens H2.0-like homeobox (HLX), mRNA [NM_021958]	0.5036502	0.0031941293
A_22_P00023433	*VPS52*	Homo sapiens vacuolar protein sorting 52 homolog (S. cerevisiae) (VPS52), transcript variant 1, mRNA [NM_022553]	0.50059146	0.011443248
A_23_P210482	*LINC00641*	Homo sapiens long intergenic non-protein coding RNA 641 (LINC00641), transcript variant 1, long non-coding RNA [NR_038970]	0.5685642	0.011747599
A_23_P255376	*ADA*	Homo sapiens adenosine deaminase (ADA), mRNA [NM_000022]	-2.0779238	0.0044240346
A_23_P157607	*SCAMP1-AS1*	Homo sapiens SCAMP1 antisense RNA 1 (SCAMP1-AS1), long non-coding RNA [NR_105014]	-0.5524218	0.0068236426
A_23_P8582	*CCDC109B*	Homo sapiens coiled-coil domain containing 109B (CCDC109B), mRNA [NM_017918]	-1.1476734	0.0050698896
A_32_P182439	*INTS10*	Homo sapiens integrator complex subunit 10 (INTS10), mRNA [NM_018142]	-0.97640055	0.0070802537
A_22_P00005316	*FAM126A*	Homo sapiens family with sequence similarity 126, member A (FAM126A), mRNA [NM_032581]	-0.71357465	0.011353592
A_33_P3327333	*POLD3*	Homo sapiens polymerase (DNA-directed), delta 3, accessory subunit (POLD3), transcript variant 1, mRNA [NM_006591]	-0.7928831	0.00735003
A_23_P408455	*lnc-DNASE1-3*	LNCipedia lincRNA (lnc-DNASE1-3), lincRNA [lnc-DNASE1-3:1]	0.8472649	0.0076440917
A_23_P426809	*IRF3*	Homo sapiens interferon regulatory factor 3 (IRF3), transcript variant 3, mRNA [NM_001197123]	1.5780642	0.0054923543
A_23_P406131	*SLC25A36*	Homo sapiens solute carrier family 25 (pyrimidine nucleotide carrier), member 36 (SLC25A36), transcript variant 1, mRNA [NM_001104647]	-0.6977567	0.010974453
A_22_P00009571	*ARHGEF11*	Homo sapiens Rho guanine nucleotide exchange factor (GEF) 11 (ARHGEF11), transcript variant 2, mRNA [NM_198236]	0.97784424	0.0057647754
A_23_P53530	*TMEM159*	Homo sapiens transmembrane protein 159 (TMEM159), transcript variant 2, mRNA [NM_020422]	-0.8644719	0.010495637

A_33_P3209356	*lnc-MAP6-1*	BX378454 Homo sapiens PLACENTA COT 25-NORMALIZED Homo sapiens cDNA clone CS0DI020YB19 3-PRIME, mRNA sequence [BX378454]	1.4122204	0.0031941293
A_23_P217528	*MTERF2*	Homo sapiens mitochondrial transcription termination factor 2 (MTERF2), transcript variant 1, mRNA [NM_001033050]	-0.7756624	0.012476755
A_24_P197964	*RFWD2*	Homo sapiens ring finger and WD repeat domain 2, E3 ubiquitin protein ligase (RFWD2), transcript variant 1, mRNA [NM_022457]	-0.8156852	0.008447855
A_23_P386450	*KLF8*	Homo sapiens Kruppel-like factor 8 (KLF8), transcript variant 1, mRNA [NM_007250]	-1.0096996	0.0140423635
A_19_P00325768	*TRIM14*	Homo sapiens tripartite motif containing 14 (TRIM14), transcript variant 1, mRNA [NM_014788]	-0.68128514	0.0068824533
A_24_P213548	*POLK*	Homo sapiens polymerase (DNA directed) kappa (POLK), mRNA [NM_016218]	-0.9587004	0.008153665
A_23_P65230	*MAN1A2*	Homo sapiens mannosidase, alpha, class 1A, member 2 (MAN1A2), mRNA [NM_006699]	-0.5481731	0.015750945
A_24_P91310	*HOMEZ*	Homo sapiens homeobox and leucine zipper encoding (HOMEZ), mRNA [NM_020834]	-0.85076	0.0107146185
A_23_P216689	*TMTC4*	Homo sapiens transmembrane and tetratricopeptide repeat containing 4 (TMTC4), transcript variant 1, mRNA [NM_032813]	-0.5642763	0.0070571043
A_23_P80551	*PSMC4*	Homo sapiens proteasome (prosome, macropain) 26S subunit, ATPase, 4 (PSMC4), transcript variant 1, mRNA [NM_006503]	0.6570522	0.0068771048
A_33_P3405995	*BRD3*	Homo sapiens bromodomain containing 3 (BRD3), mRNA [NM_007371]	-0.6734934	0.009449825
A_24_P402588	*KRBOX1*	Homo sapiens KRAB box domain containing 1 (KRBOX1), mRNA [NM_001205272]	-1.0926477	0.0054708454
A_24_P5305	*YBEY*	Homo sapiens ybeY metallopeptidase (putative) (YBEY), transcript variant 1, mRNA [NM_058181]	-0.85937077	0.004034385
A_24_P190877	*BCL11A*	Homo sapiens B-cell CLL/lymphoma 11A (zinc finger protein) (BCL11A), transcript variant 2, mRNA [NM_018014]	-2.7933629	0.0035470822
A_21_P0000830	*SPG7*	Homo sapiens spastic paraplegia 7 (pure and complicated autosomal recessive) (SPG7), transcript variant 1, mRNA [NM_003119]	1.0121707	0.0037801727
A_21_P0010961	*TRMT1L*	Homo sapiens tRNA methyltransferase 1 homolog (S. cerevisiae)-like (TRMT1L), transcript variant 1, mRNA [NM_030934]	-0.8265288	0.0055818916
A_23_P97365	*RAB30-AS1*	Homo sapiens RAB30 antisense RNA 1 (head to head) (RAB30-AS1), long non-coding RNA [NR_038903]	-0.92977464	0.0060600177

A_33_P3394075	*TSSC2*	tumor suppressing subtransferable candidate 2 pseudogene [Source:HGNC Symbol;Acc:HGNC:12384] [ENST00000526488]	0.95928705	0.014041002
A_21_P0010655	*LRRC8D*	Homo sapiens leucine rich repeat containing 8 family, member D (LRRC8D), transcript variant 2, mRNA [NM_018103]	-0.88721436	0.0072648157
A_24_P227927	*RIT1*	Homo sapiens Ras-like without CAAX 1 (RIT1), transcript variant 2, mRNA [NM_006912]	-1.1757648	0.011182691
A_23_P13524	*IL21R*	Homo sapiens interleukin 21 receptor (IL21R), transcript variant 2, mRNA [NM_181078]	3.3693461	0.0076077557
A_23_P213754	*TMEM126A*	Homo sapiens transmembrane protein 126A (TMEM126A), transcript variant 1, mRNA [NM_032273]	-0.59354657	0.009366913
A_21_P0003809	*PCDH18*	Homo sapiens protocadherin 18 (PCDH18), transcript variant 1, mRNA [NM_019035]	-2.4880939	0.0036517929
A_24_P280113	*PAIP2*	Homo sapiens poly(A) binding protein interacting protein 2 (PAIP2), transcript variant 1, mRNA [NM_001033112]	-0.8190758	0.005811904
A_23_P408271	*lnc-CLDN24-2*	LNCipedia lincRNA (lnc-CLDN24-2), lincRNA [lnc-CLDN24-2:3]	-1.1309962	0.007415985
A_23_P314222	*IL13RA1*	Homo sapiens interleukin 13 receptor, alpha 1 (IL13RA1), mRNA [NM_001560]	-1.1408637	0.0046812603
A_33_P3411907	*HSD17B11*	Homo sapiens hydroxysteroid (17-beta) dehydrogenase 11 (HSD17B11), mRNA [NM_016245]	-0.92068386	0.004670025
A_23_P29365	*LEO1*	Homo sapiens Leo1, Paf1/RNA polymerase II complex component, homolog (S. cerevisiae) (LEO1), transcript variant 1, mRNA [NM_138792]	-0.31686348	0.013345465
A_22_P00019874	*FGF5*	Homo sapiens fibroblast growth factor 5 (FGF5), transcript variant 2, mRNA [NM_033143]	1.3067861	0.013680298
A_21_P0010322	*RYBP*	Homo sapiens RING1 and YY1 binding protein (RYBP), mRNA [NM_012234]	-0.7219236	0.008106991
A_22_P00017083	*TMEM56-RWDD3*	Homo sapiens TMEM56-RWDD3 readthrough (TMEM56-RWDD3), mRNA [NM_001199691]	-1.0273448	0.00511448
A_33_P3370076	*ADCY3*	Homo sapiens adenylate cyclase 3 (ADCY3), mRNA [NM_004036]	-0.7573316	0.008193496
A_33_P3321993	*lnc-TWSG1-1*	Homo sapiens cDNA FLJ44360 fis, clone TRACH3007541. [AK126333]	-1.2458088	0.006169501
A_23_P17074	*TFE3*	Homo sapiens transcription factor binding to IGHM enhancer 3 (TFE3), transcript variant 1, mRNA [NM_006521]	0.71232724	0.010519517
A_33_P3273409	*CCDC115*	Homo sapiens coiled-coil domain containing 115 (CCDC115), transcript variant 1, mRNA [NM_032357]	-0.9607296	0.008064267

A_21_P0013709	*GMPPA*	Homo sapiens GDP-mannose pyrophosphorylase A (GMPPA), transcript variant 1, mRNA [NM_013335]	0.9924463	0.0114709465
A_24_P414332	*SLC35E1*	Homo sapiens solute carrier family 35, member E1 (SLC35E1), mRNA [NM_024881]	-0.65043944	0.009425872
A_23_P99927	*LINC01503*	long intergenic non-protein coding RNA 1503 [Source:HGNC Symbol;Acc:HGNC:51184] [ENST00000423122]	0.7289312	0.006605609
A_23_P55107	*AFF1*	Homo sapiens AF4/FMR2 family, member 1 (AFF1), transcript variant 2, mRNA [NM_005935]	-0.5459657	0.0026564114
A_22_P00002736	*PTPLAD1*	Homo sapiens protein tyrosine phosphatase-like A domain containing 1 (PTPLAD1), mRNA [NM_016395]	-0.62310743	0.0069260783
A_21_P0014563	*ULK2*	Homo sapiens unc-51 like autophagy activating kinase 2 (ULK2), transcript variant 1, mRNA [NM_014683]	-1.0253572	0.0033199077
A_23_P212522	*TRIM24*	tripartite motif containing 24 [Source:HGNC Symbol;Acc:HGNC:11812] [ENST00000343526]	-1.0595572	0.0028288076
A_24_P317874	*CADPS*	Homo sapiens Ca++-dependent secretion activator (CADPS), transcript variant 3, mRNA [NM_183393]	-3.347773	0.0035939496
A_23_P55376	*ATP11B*	Homo sapiens ATPase, class VI, type 11B (ATP11B), mRNA [NM_014616]	-0.8332002	0.0028768703
A_24_P414952	*KIN*	Homo sapiens Kin17 DNA and RNA binding protein (KIN), transcript variant 1, mRNA [NM_012311]	-0.6474127	0.009276546
A_33_P3390456	*KANSL1*	Homo sapiens KAT8 regulatory NSL complex subunit 1 (KANSL1), transcript variant 2, mRNA [NM_015443]	-0.5730495	0.015340515
A_23_P147388	*TMEM168*	Homo sapiens transmembrane protein 168 (TMEM168), transcript variant 2, mRNA [NM_022484]	-0.73710847	0.0076856846
A_22_P00002998	*YAF2*	Homo sapiens YY1 associated factor 2 (YAF2), transcript variant 1, mRNA [NM_001190979]	-1.1131817	0.006772767
A_24_P336417	*KIF13B*	Homo sapiens kinesin family member 13B (KIF13B), mRNA [NM_015254]	-1.2336286	0.0052543716
A_33_P3587611	*LOC102723648*	PREDICTED: Homo sapiens uncharacterized LOC102723648 (LOC102723648), transcript variant X3, ncRNA [XR_427920]	0.5476404	0.017126175
A_23_P130149	*XPR1*	Homo sapiens xenotropic and polytropic retrovirus receptor 1 (XPR1), transcript variant 1, mRNA [NM_004736]	-1.0557585	0.008159287
A_23_P122068	*RPL36AP33*	Homo sapiens cDNA clone MGC:23914 IMAGE:4769647, complete cds. [BC020828]	-0.88291687	0.015771633
A_24_P284353	*ENO3*	Homo sapiens enolase 3 (beta, muscle) (ENO3), transcript variant 1, mRNA [NM_001976]	-1.0839732	0.01348601

A_23_P98092	*C1QTNF3*	Homo sapiens C1q and tumor necrosis factor related protein 3 (C1QTNF3), transcript variant 2, mRNA [NM_181435]	-1.191499	0.009591824
A_23_P394836	*TMEM107*	Homo sapiens transmembrane protein 107 (TMEM107), transcript variant 1, mRNA [NM_032354]	-1.2329304	0.0073548867
A_23_P36305	*OAT*	Homo sapiens ornithine aminotransferase (OAT), transcript variant 1, mRNA [NM_000274]	-0.61429816	0.010839669
A_33_P3346533	*INF2*	Homo sapiens inverted formin, FH2 and WH2 domain containing (INF2), transcript variant 1, mRNA [NM_022489]	0.8495374	0.013288616
A_23_P98232	*ATG16L2*	Homo sapiens autophagy related 16-like 2 (S. cerevisiae) (ATG16L2), mRNA [NM_033388]	-0.5436005	0.010060375
A_33_P3210671	*ZFPL1*	Homo sapiens zinc finger protein-like 1 (ZFPL1), mRNA [NM_006782]	0.84494674	0.013715877
A_23_P92687	*MAGI3*	Homo sapiens membrane associated guanylate kinase, WW and PDZ domain containing 3 (MAGI3), transcript variant 2, mRNA [NM_152900]	-0.7412835	0.009894935
A_24_P687594	*GPAT2*	Homo sapiens glycerol-3-phosphate acyltransferase 2, mitochondrial (GPAT2), mRNA [NM_207328]	1.6132635	0.00395888
A_32_P205110	*DAP*	Homo sapiens death-associated protein (DAP), transcript variant 2, mRNA [NM_004394]	0.91684484	0.016465655
A_33_P3399064	*LIX1L*	Homo sapiens Lix1 homolog (chicken) like (LIX1L), mRNA [NM_153713]	-0.7415855	0.012609292
A_33_P3212092	*FOXC1*	Homo sapiens forkhead box C1 (FOXC1), mRNA [NM_001453]	-1.4430243	0.005632443
A_23_P29046	*RNA5-8S5*	Homo sapiens RNA, 5.8S ribosomal 5 (RNA5-8S5), ribosomal RNA [NR_003285]	0.9886472	0.011965513
A_24_P344295	*PDCD4*	Homo sapiens programmed cell death 4 (neoplastic transformation inhibitor) (PDCD4), transcript variant 2, mRNA [NM_145341]	-0.7092264	0.0067838193
A_33_P3258702	*CBR1*	Homo sapiens carbonyl reductase 1 (CBR1), transcript variant 1, mRNA [NM_001757]	-0.6020083	0.008853051
A_24_P311604	*RNF167*	Homo sapiens ring finger protein 167 (RNF167), mRNA [NM_015528]	1.3988612	0.0046812603
A_33_P3281033	*SEC22C*	Homo sapiens SEC22 vesicle trafficking protein homolog C (S. cerevisiae) (SEC22C), transcript variant 4, mRNA [NM_001201584]	-0.9402043	0.014260212
A_23_P362228	*PACRGL*	Homo sapiens PARK2 co-regulated-like (PACRGL), transcript variant 1, mRNA [NM_145048]	-0.9762147	0.013367701
A_33_P3350703	*MACROD2*	Homo sapiens chromosome 20 open reading frame 133, mRNA (cDNA clone IMAGE:4513167). [BC035876]	0.37897637	0.008320085

A_24_P277349	*ZNF436-AS1*	Homo sapiens ZNF436 antisense RNA 1 (ZNF436-AS1), transcript variant 1, long non-coding RNA [NR_033690]	-1.0444522	0.005365543
A_24_P941643	*ERO1LB*	Homo sapiens ERO1-like beta (S. cerevisiae) (ERO1LB), mRNA [NM_019891]	-0.8284838	0.0166364
A_21_P0000362	*SEC31A*	Homo sapiens SEC31 homolog A (S. cerevisiae) (SEC31A), transcript variant 5, mRNA [NM_001077207]	-0.87364215	0.013663553
A_21_P0000152	*PLCB1*	Homo sapiens phospholipase C, beta 1 (phosphoinositide-specific) (PLCB1), transcript variant 2, mRNA [NM_182734]	-0.7032134	0.0034534817
A_23_P67748	*SNORA1*	Homo sapiens small nucleolar RNA, H/ACA box 1 (SNORA1), small nucleolar RNA [NR_003026]	-1.2251524	0.0054923543
A_33_P3323699	*CD44*	Homo sapiens CD44 molecule (Indian blood group) (CD44), transcript variant 8, mRNA [NM_001202557]	1.3579444	0.0039522685
A_23_P115785	*RAB11B*	Homo sapiens RAB11B, member RAS oncogene family (RAB11B), mRNA [NM_004218]	1.4876825	0.005192162
A_23_P362183	*NPPA*	natriuretic peptide A [Source:HGNC Symbol;Acc:HGNC:7939] [ENST00000376476]	1.1462595	0.007474992
A_32_P19840	*FANK1*	Homo sapiens fibronectin type III and ankyrin repeat domains 1 (FANK1), mRNA [NM_145235]	-1.9853846	0.004454847
A_24_P7157	*ANKS6*	Homo sapiens ankyrin repeat and sterile alpha motif domain containing 6 (ANKS6), mRNA [NM_173551]	-1.0723083	0.006514206
A_23_P161022	*lnc-EPHA1-1*	PREDICTED: Homo sapiens uncharacterized LOC100507507 (LOC100507507), mRNA [XM_006710194]	-1.1444097	0.0046744854
A_24_P341222	*RIMKLB*	Homo sapiens ribosomal modification protein rimK-like family member B (RIMKLB), transcript variant 1, mRNA [NM_020734]	-1.6640854	0.004609308
A_19_P00322941	*SNAPIN*	Homo sapiens SNAP-associated protein (SNAPIN), transcript variant 1, mRNA [NM_012437]	-0.5883674	0.015534843
A_23_P65068	*SMIM20*	Homo sapiens small integral membrane protein 20 (SMIM20), mRNA [NM_001145432]	-0.8313494	0.009820808
A_23_P117163	*MIR143HG*	MIR143 host gene (non-protein coding) [Source:HGNC Symbol;Acc:HGNC:42872] [ENST00000518014]	-4.5876536	0.0033015371
A_23_P165676	*EID3*	Homo sapiens EP300 interacting inhibitor of differentiation 3 (EID3), mRNA [NM_001008394]	0.63817793	0.0078082304
A_21_P0007412	*RCBTB1*	Homo sapiens regulator of chromosome condensation (RCC1) and BTB (POZ) domain containing protein 1 (RCBTB1), mRNA [NM_018191]	-0.86655325	0.010397241

A_33_P3242355	*lnc-BLID-1*	LNCipedia lincRNA (lnc-BLID-1), lincRNA [lnc-BLID-1:18]	-1.0828178	0.0054365364
A_23_P100455	*NOSTRIN*	Homo sapiens nitric oxide synthase trafficking (NOSTRIN), transcript variant 1, mRNA [NM_052946]	-2.1632624	0.0025001846
A_23_P430902	*MTHFSD*	Homo sapiens methenyltetrahydrofolate synthetase domain containing (MTHFSD), transcript variant 3, mRNA [NM_022764]	-1.1819961	0.004993122
A_23_P393425	*ZNF217*	Homo sapiens zinc finger protein 217 (ZNF217), mRNA [NM_006526]	-0.4533842	0.00978742
A_24_P925062	*MORN4*	Homo sapiens MORN repeat containing 4 (MORN4), transcript variant 1, mRNA [NM_178832]	-0.8715807	0.01649328
A_33_P3369939	*PAPD4*	Homo sapiens PAP associated domain containing 4 (PAPD4), transcript variant 3, mRNA [NM_173797]	-1.0854855	0.0047990954
A_24_P117177	*MXRA7*	Homo sapiens matrix-remodelling associated 7 (MXRA7), transcript variant 1, mRNA [NM_001008528]	-1.1476468	0.015442503
A_33_P3261433	*DNAJC21*	Homo sapiens DnaJ (Hsp40) homolog, subfamily C, member 21 (DNAJC21), transcript variant 1, mRNA [NM_194283]	-0.57926744	0.007479662
A_33_P3382157	*SNX21*	Homo sapiens sorting nexin family member 21 (SNX21), transcript variant 2, mRNA [NM_152897]	0.5181261	0.0062257075
A_23_P216708	*PICALM*	Homo sapiens phosphatidylinositol binding clathrin assembly protein (PICALM), transcript variant 1, mRNA [NM_007166]	-0.8299565	0.002804883
A_23_P156970	*SLIT3*	Homo sapiens slit homolog 3 (Drosophila) (SLIT3), transcript variant 2, mRNA [NM_003062]	-2.105854	0.0027248112
A_23_P162127	*RFK*	Homo sapiens riboflavin kinase (RFK), mRNA [NM_018339]	-0.56447524	0.016991401
A_24_P409595	*MEST*	Homo sapiens mesoderm specific transcript (MEST), transcript variant 1, mRNA [NM_002402]	-2.0518034	0.0042908904
A_33_P3310533	*CCDC90B*	Homo sapiens coiled-coil domain containing 90B (CCDC90B), transcript variant 1, mRNA [NM_021825]	-0.92440194	0.005937971
A_23_P215060	*MAPK8IP1*	Homo sapiens mitogen-activated protein kinase 8 interacting protein 1 (MAPK8IP1), mRNA [NM_005456]	1.6479337	0.0046701035
A_33_P3764802	*DNAJC21*	Homo sapiens DnaJ (Hsp40) homolog, subfamily C, member 21 (DNAJC21), transcript variant 2, mRNA [NM_001012339]	-0.6813744	0.0049456456
A_23_P213840	*PODXL*	Homo sapiens podocalyxin-like (PODXL), transcript variant 1, mRNA [NM_001018111]	-2.669595	0.002419416

A_33_P3296303	*SIRT5*	Homo sapiens sirtuin 5 (SIRT5), transcript variant 1, mRNA [NM_012241]	-1.5352597	0.0041881003
A_32_P831181	*ATP6V0E1*	Homo sapiens ATPase, H+ transporting, lysosomal 9kDa, V0 subunit e1 (ATP6V0E1), mRNA [NM_003945]	-1.2725868	0.0040017776
A_22_P00001561	*CHMP5*	Homo sapiens charged multivesicular body protein 5 (CHMP5), transcript variant 1, mRNA [NM_016410]	-1.1222839	0.0029471128
A_33_P3691916	*BRI3BP*	Homo sapiens BRI3 binding protein (BRI3BP), mRNA [NM_080626]	1.040634	0.010971502
A_21_P0013977	*FAM13A*	Homo sapiens family with sequence similarity 13, member A (FAM13A), transcript variant 1, mRNA [NM_014883]	-0.5450528	0.0057647754
A_33_P3309684	*PAK2*	Homo sapiens p21 protein (Cdc42/Rac)-activated kinase 2 (PAK2), mRNA [NM_002577]	-0.5611328	0.013900776
A_19_P00809417	*PCID2*	PCI domain containing 2 [Source:HGNC Symbol;Acc:HGNC:25653] [ENST00000493650]	-0.913934	0.013802298
A_22_P00006711	*ATF7IP2*	activating transcription factor 7 interacting protein 2 [Source:HGNC Symbol;Acc:HGNC:20397] [ENST00000570163]	1.230274	0.012353942
A_33_P3306264	*NCOA4*	Homo sapiens nuclear receptor coactivator 4 (NCOA4), transcript variant 1, mRNA [NM_001145260]	-0.75283283	0.009512793
A_33_P3422812	*lnc-FRMD1-3*	602494735F1 NIH_MGC_75 Homo sapiens cDNA clone IMAGE:4608392 5', mRNA sequence [BG428602]	-0.80527914	0.012832334
A_32_P179676	*LYPD3*	Homo sapiens LY6/PLAUR domain containing 3 (LYPD3), mRNA [NM_014400]	2.2916698	0.0031941293
A_33_P3854030	*C7orf73*	Homo sapiens chromosome 7 open reading frame 73 (C7orf73), mRNA [NM_001130929]	-1.0458095	0.004609308
A_33_P3386716	*TOB2*	Homo sapiens transducer of ERBB2, 2 (TOB2), mRNA [NM_016272]	-1.2564702	0.0029471128
A_23_P361584	*MBD5*	Homo sapiens methyl-CpG binding domain protein 5 (MBD5), mRNA [NM_018328]	-1.0729506	0.0066162148
A_33_P3262665	*PPP1R15B*	Homo sapiens protein phosphatase 1, regulatory subunit 15B (PPP1R15B), mRNA [NM_032833]	-0.548791	0.009574714
A_23_P5831	*TMEM154*	Homo sapiens transmembrane protein 154 (TMEM154), mRNA [NM_152680]	-1.2579951	0.0074381046
A_33_P3318122	*MAP7D3*	Homo sapiens MAP7 domain containing 3 (MAP7D3), transcript variant 1, mRNA [NM_024597]	-1.3622234	0.007392104
A_23_P132121	*HPCAL1*	Homo sapiens hippocalcin-like 1 (HPCAL1), transcript variant 2, mRNA [NM_134421]	1.2487781	0.009069191
A_23_P34307	*C16orf62*	Homo sapiens chromosome 16 open reading frame 62 (C16orf62), transcript variant 1, mRNA [NM_020314]	-0.82413405	0.016474942

A_23_P43157	*SIK1*	Homo sapiens salt-inducible kinase 1 (SIK1), mRNA [NM_173354]	3.2909327	0.0029675725
A_33_P3301219	*PIGK*	Homo sapiens phosphatidylinositol glycan anchor biosynthesis, class K (PIGK), mRNA [NM_005482]	-1.1441784	0.005776624
A_32_P116840	*MYBL1*	Homo sapiens v-myb avian myeloblastosis viral oncogene homolog-like 1 (MYBL1), transcript variant 1, mRNA [NM_001080416]	-0.8036457	0.011420157
A_33_P3384543	*FAM27C*	Homo sapiens family with sequence similarity 27, member C (FAM27C), long non-coding RNA [NR_027421]	-13411525	0.011493016
A_33_P3397795	*CTAGE5*	Homo sapiens CTAGE family, member 5 (CTAGE5), transcript variant 4, mRNA [NM_203356]	-0.9174868	0.010147684
A_23_P214459	*POMGNT1*	Homo sapiens protein O-linked mannose N-acetylglucosaminyltransferase 1 (beta 1,2-) (POMGNT1), transcript variant 2, mRNA [NM_001243766]	-0.6880584	0.010804776
A_33_P3331346	*PCNXL4*	Homo sapiens cDNA FLJ38170 fis, clone FCBBF1000024. [AK095489]	-0.6930971	0.007854403
A_33_P3224423	*PRPH2*	Homo sapiens peripherin 2 (retinal degeneration, slow) (PRPH2), mRNA [NM_000322]	1.3418072	0.006814677
A_24_P497186	*TRIM65*	Homo sapiens tripartite motif containing 65 (TRIM65), transcript variant 1, mRNA [NM_173547]	-0.877635	0.0065241847
A_23_P339119	*POLM*	Homo sapiens polymerase (DNA directed), mu (POLM), transcript variant 1, mRNA [NM_013284]	0.67868286	0.0024890031
A_23_P210348	*IRF2BP2*	Homo sapiens interferon regulatory factor 2 binding protein 2 (IRF2BP2), transcript variant 1, mRNA [NM_182972]	-1.5325624	0.0031941293
A_23_P153930	*ACSS3*	Homo sapiens acyl-CoA synthetase short-chain family member 3 (ACSS3), mRNA [NM_024560]	-1.327917	0.0072350414
A_24_P160874	*COX7A2L*	Homo sapiens cytochrome c oxidase subunit VIIa polypeptide 2 like (COX7A2L), mRNA [NM_004718]	-0.8086972	0.0031474202
A_23_P325690	*ACVR2A*	Homo sapiens activin A receptor, type IIA (ACVR2A), transcript variant 2, mRNA [NM_001616]	-1.145681	0.0044508153
A_33_P3305855	*DUT*	Homo sapiens deoxyuridine triphosphatase (DUT), transcript variant 1, mRNA [NM_001025248]	-1.0884426	0.01305673
A_32_P191895	*ANKRD35*	Homo sapiens ankyrin repeat domain 35 (ANKRD35), transcript variant 1, mRNA [NM_144698]	0.4334781	0.006474282
A_23_P97394	*RPS16*	Homo sapiens ribosomal protein S16 (RPS16), mRNA [NM_001020]	-0.31618926	0.005294463

A_33_P3214720	*KIFC3*	Homo sapiens kinesin family member C3 (KIFC3), transcript variant 1, mRNA [NM_005550]	1.187926	0.007920593
A_23_P124327	*BCAR3*	Homo sapiens breast cancer anti-estrogen resistance 3 (BCAR3), transcript variant 2, mRNA [NM_003567]	-0.7400546	0.005644507
A_24_P239811	*ZC3H12A*	Homo sapiens zinc finger CCCH-type containing 12A (ZC3H12A), mRNA [NM_025079]	2.689751	0.003948561
A_32_P130265	*NSD1*	Homo sapiens nuclear receptor binding SET domain protein 1 (NSD1), transcript variant 2, mRNA [NM_022455]	-0.8929115	0.017015142
A_32_P112592	*UBXN11*	Homo sapiens UBX domain protein 11 (UBXN11), transcript variant 2, mRNA [NM_183008]	1.2023844	0.011318375
A_23_P309967	*ZC3H11A*	Homo sapiens zinc finger CCCH-type containing 11A (ZC3H11A), mRNA [NM_014827]	-0.44805	0.006020368
A_22_P00002045	*LINC01140*	Homo sapiens long intergenic non-protein coding RNA 1140 (LINC01140), transcript variant 5, long non-coding RNA [NR_026989]	-0.57509553	0.014070397
A_23_P408675	*DCTN5*	Homo sapiens dynactin 5 (p25) (DCTN5), transcript variant 1, mRNA [NM_032486]	0.40165895	0.010822477
A_23_P359870	*SCAMP1*	Homo sapiens secretory carrier membrane protein 1 (SCAMP1), transcript variant 1, mRNA [NM_004866]	-0.6540007	0.0105530135
A_23_P134125	*BTAF1*	Homo sapiens BTAF1 RNA polymerase II, B-TFIID transcription factor-associated, 170kDa (BTAF1), mRNA [NM_003972]	-0.800198	0.010150118
A_23_P259251	*MAP3K5*	Homo sapiens mitogen-activated protein kinase kinase kinase 5 (MAP3K5), mRNA [NM_005923]	1.2897252	0.0072230194
A_32_P46214	*CLIC4*	Homo sapiens chloride intracellular channel 4 (CLIC4), mRNA [NM_013943]	-1.5120711	0.0022181747
A_33_P3389653	*KCND2*	Homo sapiens potassium channel, voltage gated Shal related subfamily D, member 2 (KCND2), mRNA [NM_012281]	0.4119676	0.007868093
A_22_P00012285	*SLC9A9*	Homo sapiens solute carrier family 9, subfamily A (NHE9, cation proton antiporter 9), member 9 (SLC9A9), mRNA [NM_173653]	-3.6730604	0.0025001846
A_23_P251075	*PDE4D*	Homo sapiens phosphodiesterase 4D, cAMP-specific (PDE4D), transcript variant 3, mRNA [NM_001165899]	1.7047031	0.002419416
A_24_P115507	*TCERG1L-AS1*	Homo sapiens TCERG1L antisense RNA 1 (TCERG1L-AS1), long non-coding RNA [NR_120623]	0.5898535	0.014491717
A_24_P802145	*MAMLD1*	Homo sapiens mastermind-like domain containing 1 (MAMLD1), transcript variant 2, mRNA [NM_005491]	-2.4598098	0.0033094941

A_24_P132470	*SARDH*	Homo sapiens sarcosine dehydrogenase (SARDH), transcript variant 1, mRNA [NM_007101]	-0.15623109	0.010735533
A_22_P00005771	*IRS1*	Homo sapiens insulin receptor substrate 1 (IRS1), mRNA [NM_005544]	0.6672324	0.0056355232
A_23_P120472	*ESYT2*	Homo sapiens extended synaptotagmin-like protein 2 (ESYT2), mRNA [NM_020728]	-1.4500148	0.0053691682
A_24_P726336	*lnc-ENPP1-2*	LNCipedia lincRNA (lnc-ENPP1-2), lincRNA [lnc-ENPP1-2:1]	-0.6574688	0.005365543
A_33_P3342295	*TFAP2C*	Homo sapiens transcription factor AP-2 gamma (activating enhancer binding protein 2 gamma) (TFAP2C), mRNA [NM_003222]	1.3330102	0.0050847097
A_23_P501538	*PHACTR2*	Homo sapiens phosphatase and actin regulator 2 (PHACTR2), transcript variant 1, mRNA [NM_001100164]	-0.8259887	0.009851655
A_23_P431179	*VSX1*	Homo sapiens visual system homeobox 1 (VSX1), transcript variant 2, mRNA [NM_199425]	1.2340438	0.0029695062
A_23_P116942	*HOXA3*	Homo sapiens homeobox A3 (HOXA3), transcript variant 2, mRNA [NM_153631]	1.2706051	0.0044240346
A_22_P00004418	*HIST1H4A*	Homo sapiens histone cluster 1, H4a (HIST1H4A), mRNA [NM_003538]	0.5616654	0.012414032
A_23_P405129	*LAG3*	Homo sapiens lymphocyte-activation gene 3 (LAG3), mRNA [NM_002286]	-1.0659884	0.00860804
A_33_P3352103	*lnc-COPS7B-1*	Homo sapiens cDNA clone IMAGE:4300887, partial cds. [BC008846]	-1.504389	0.010827594
A_24_P29686	*LTBP2*	Homo sapiens latent transforming growth factor beta binding protein 2 (LTBP2), mRNA [NM_000428]	-0.6954307	0.0024890031
A_24_P363100	*LYPLAL1*	Homo sapiens lysophospholipase-like 1 (LYPLAL1), transcript variant 1, mRNA [NM_138794]	-0.60890913	0.011324697
A_21_P0014379	*ZMIZ2*	Homo sapiens zinc finger, MIZ-type containing 2 (ZMIZ2), transcript variant 1, mRNA [NM_031449]	-1.0227628	0.010149606
A_23_P34460	*RGMB*	Homo sapiens repulsive guidance molecule family member b (RGMB), mRNA [NM_001012761]	1.0494262	0.017030599
A_33_P3309054	*ZNF793-AS1*	Homo sapiens ZNF793 antisense RNA 1 (head to head) (ZNF793-AS1), long non-coding RNA [NR_110723]	-1.4501214	0.011749359
A_33_P3424472	*SLC35A3*	Homo sapiens solute carrier family 35 (UDP-N-acetylglucosamine (UDP-GlcNAc) transporter), member A3 (SLC35A3), transcript variant 1, mRNA [NM_012243]	-0.5913825	0.0033094941
A_23_P110571	*TNPO3*	Homo sapiens transportin 3 (TNPO3), transcript variant 1, mRNA [NM_012470]	-1.0424926	0.014687651

A_33_P3268716	*LINC01540*	Homo sapiens long intergenic non-protein coding RNA 1540 (LINC01540), transcript variant 1, long non-coding RNA [NR_110429]	1.1804252	0.012581895
A_33_P3269578	*MAST4*	Homo sapiens microtubule associated serine/threonine kinase family member 4 (MAST4), transcript variant 3, mRNA [NM_001164664]	1.0525577	0.013806973
A_22_P00014361	*SNX1*	Homo sapiens sorting nexin 1 (SNX1), transcript variant 1, mRNA [NM_003099]	-1.1824327	0.008061535
A_24_P64393	*PBRM1*	Homo sapiens polybromo 1 (PBRM1), mRNA [NM_018313]	-1.0075877	0.012276114
A_33_P3364904	*CWC15*	Homo sapiens CWC15 spliceosome-associated protein (CWC15), mRNA [NM_016403]	-0.49585465	0.014989517
A_24_P418408	*MARCH5*	Homo sapiens membrane-associated ring finger (C3HC4) 5 (MARCH5), mRNA [NM_017824]	-0.6767645	0.0084265405
A_33_P3407937	*ZNF662*	Homo sapiens zinc finger protein 662 (ZNF662), transcript variant 1, mRNA [NM_207404]	-1.6173279	0.0043846127
A_33_P7883685	*FAM89A*	Homo sapiens family with sequence similarity 89, member A (FAM89A), mRNA [NM_198552]	1.8631934	0.006155321
A_23_P211840	*PLCXD1*	Homo sapiens phosphatidylinositol-specific phospholipase C, X domain containing 1 (PLCXD1), transcript variant 1, mRNA [NM_018390]	0.9241576	0.0166364
A_33_P3294317	*TSIX*	Homo sapiens TSIX transcript, XIST antisense RNA (TSIX), antisense RNA [NR_003255]	0.63211125	0.00910458
A_23_P23194	*UBA3*	Homo sapiens ubiquitin-like modifier activating enzyme 3 (UBA3), transcript variant 1, mRNA [NM_003968]	-0.42871976	0.009421056
A_22_P00005221	*LOC101928880*	Homo sapiens uncharacterized LOC101928880 (LOC101928880), long non-coding RNA [NR_110944]	0.78062165	0.011728736
A_33_P3282669	*PINK1*	Homo sapiens PTEN induced putative kinase 1 (PINK1), mRNA [NM_032409]	-0.84999084	0.010545495
A_33_P3388016	*MEG3*	Homo sapiens maternally expressed 3 (non-protein coding) (MEG3), transcript variant 4, long non-coding RNA [NR_033358]	1.9011556	0.0043059364
A_33_P3618429	*POLL*	Homo sapiens polymerase (DNA directed), lambda (POLL), transcript variant 1, mRNA [NM_001174084]	1.0835528	0.0024890031
A_23_P361841	*TMA7*	Homo sapiens translation machinery associated 7 homolog (S. cerevisiae) (TMA7), mRNA [NM_015933]	-0.73858	0.0063397386
A_33_P3289705	*PRKXP1*	Homo sapiens protein kinase, X-linked, pseudogene 1 (PRKXP1), non-coding RNA [NR_073405]	-0.6069224	0.015620706

A_19_P00322182	*RIMKLB*	Homo sapiens ribosomal modification protein rimK-like family member B (RIMKLB), transcript variant 1, mRNA [NM_020734]	-1.4041526	0.0054418826
A_22_P00002869	*GOLGB1*	Homo sapiens golgin B1 (GOLGB1), transcript variant 3, mRNA [NM_001256487]	-0.93951625	0.008048185
A_32_P95067	*RALGPS2*	Homo sapiens Ral GEF with PH domain and SH3 binding motif 2 (RALGPS2), transcript variant 10, mRNA [NM_152663]	-1.2135608	0.007415985
A_33_P3231242	*ST6GALNAC5*	Homo sapiens ST6 (alpha-N-acetyl-neuraminyl-2,3-beta-galactosyl-1,3)-N-acetylgalactosaminide alpha-2,6-sialyltransferase 5 (ST6GALNAC5), mRNA [NM_030965]	-2.5123467	0.007843468
A_24_P313822	*AK4*	Homo sapiens adenylate kinase 4 (AK4), transcript variant 1, mRNA [NM_001005353]	-1.572118	0.008143171
A_24_P68585	*PAK4*	Homo sapiens p21 protein (Cdc42/Rac)-activated kinase 4 (PAK4), transcript variant 1, mRNA [NM_005884]	1.0891526	0.012320746
A_33_P3244828	*MX2*	Homo sapiens MX dynamin-like GTPase 2 (MX2), mRNA [NM_002463]	2.7738519	0.0031941293
A_23_P74114	*SHC1*	Homo sapiens SHC (Src homology 2 domain containing) transforming protein 1 (SHC1), transcript variant 1, mRNA [NM_183001]	1.8526425	0.0129849
A_32_P9842	*ZNF713*	Homo sapiens zinc finger protein 713 (ZNF713), mRNA [NM_182633]	2.6277564	0.0072200163
A_24_P381555	*MTMR14*	Homo sapiens myotubularin related protein 14 (MTMR14), transcript variant 1, mRNA [NM_001077526]	-0.90075487	0.0044240346
A_21_P0000666	*C21orf59*	Homo sapiens chromosome 21 open reading frame 59 (C21orf59), transcript variant 1, mRNA [NM_021254]	-0.50609714	0.015992101
A_23_P348264	*SAP18*	Homo sapiens Sin3A-associated protein, 18kDa (SAP18), mRNA [NM_005870]	-1.1463337	0.0039486005
A_33_P3267263	*LOC100129361*	Homo sapiens chromosome X open reading frame 69-like (LOC100129361), mRNA [NM_001271592]	-0.39072105	0.008273812
A_32_P12327	*LETM2*	Homo sapiens leucine zipper-EF-hand containing transmembrane protein 2 (LETM2), transcript variant 2, mRNA [NM_144652]	2.913946	0.0042509567
A_19_P00808348	*RNVU1-18*	Homo sapiens RNA, variant U1 small nuclear 18 (RNVU1-18), small nuclear RNA [NR_004400]	0.83585477	0.0039486005
A_33_P3839334	*USP46-AS1*	Homo sapiens USP46 antisense RNA 1 (USP46-AS1), long non-coding RNA [NR_125363]	-0.5528875	0.015453676
A_21_P0008626	*RETSAT*	Homo sapiens retinol saturase (all-trans-retinol 13,14-reductase) (RETSAT), mRNA [NM_017750]	0.32680044	0.014497109

A_22_P00017804	*ZNF547*	zinc finger protein 547 [Source:HGNC Symbol;Acc:HGNC:26432] [ENST00000282282]	-1.3907242	0.011053785
A_23_P11598	*OIP5-AS1*	Homo sapiens OIP5 antisense RNA 1 (OIP5-AS1), long non-coding RNA [NR_026757]	-0.5767809	0.011087365
A_21_P0009964	*ZBTB20*	Homo sapiens zinc finger and BTB domain containing 20 (ZBTB20), transcript variant 3, mRNA [NM_001164343]	-0.77218103	0.011499655
A_33_P3358938	*ZNF684*	Homo sapiens zinc finger protein 684 (ZNF684), mRNA [NM_152373]	-1.1205052	0.014286892
A_33_P3326225	*lnc-NCOA3-10*	DKFZp686M2229_r1 686 (synonym: hlcc3) Homo sapiens cDNA clone DKFZp686M2229 5', mRNA sequence [BX489122]	0.3008516	0.0070571043
A_19_P00813077	*CLOCK*	Homo sapiens clock circadian regulator (CLOCK), transcript variant 1, mRNA [NM_001267843]	-0.68978024	0.00975317
A_33_P3378880	*SAMD12*	Homo sapiens sterile alpha motif domain containing 12 (SAMD12), transcript variant 1, mRNA [NM_001101676]	1.041244	0.0102121765
A_23_P141960	*FAM228B*	Homo sapiens family with sequence similarity 228, member B (FAM228B), transcript variant 1, mRNA [NM_001145710]	-1.0027105	0.011110267
A_23_P101796	*HIST1H4C*	histone cluster 1, H4c [Source:HGNC Symbol;Acc:HGNC:4787] [ENST00000377803]	0.75359756	0.013195482
A_23_P201636	*SERTAD3*	Homo sapiens SERTA domain containing 3 (SERTAD3), transcript variant 2, mRNA [NM_203344]	-0.3628698	0.005640775
A_33_P3346046	*SYDE1*	Homo sapiens synapse defective 1, Rho GTPase, homolog 1 (C. elegans) (SYDE1), transcript variant 1, mRNA [NM_033025]	1.0131378	0.01338585
A_32_P30831	*LAMC2*	Homo sapiens laminin, gamma 2 (LAMC2), transcript variant 1, mRNA [NM_005562]	-2.8343925	0.0050554653
A_23_P5757	*RAD51-AS1*	Homo sapiens RAD51 antisense RNA 1 (head to head) (RAD51-AS1), long non-coding RNA [NR_040058]	-1.1150882	0.0062618116
A_23_P254756	*ORAOV1*	Homo sapiens oral cancer overexpressed 1 (ORAOV1), mRNA [NM_153451]	-1.1057117	0.0031750489
A_23_P148629	*TPRKB*	Homo sapiens TP53RK binding protein (TPRKB), mRNA [NM_016058]	-0.31754702	0.013806973
A_23_P418597	*CD164*	Homo sapiens CD164 molecule, sialomucin (CD164), transcript variant 1, mRNA [NM_006016]	-0.5232363	0.007103236
A_23_P110643	*EIF1AY*	Homo sapiens eukaryotic translation initiation factor 1A, Y-linked (EIF1AY), transcript variant 1, mRNA [NM_004681]	-0.38750055	0.0077072685
A_22_P00002753	*TNKS1BP1*	Homo sapiens tankyrase 1 binding protein 1, 182kDa (TNKS1BP1), mRNA [NM_033396]	1.3740143	0.003667498

A_23_P350895	*CDKL3*	Homo sapiens cyclin-dependent kinase-like 3 (CDKL3), transcript variant 2, mRNA [NM_016508]	-1.2169429	0.006773419
A_23_P254573	*lnc-C21orf62-1*	LNCipedia lincRNA (lnc-C21orf62-1), lincRNA [lnc-C21orf62-1:1]	-0.23434207	0.008248755
A_24_P91405	*DNAJC2*	Homo sapiens DnaJ (Hsp40) homolog, subfamily C, member 2 (DNAJC2), transcript variant 1, mRNA [NM_014377]	-0.2683975	0.014221296
A_23_P319719	*HOXC8*	Homo sapiens homeobox C8 (HOXC8), mRNA [NM_022658]	-1.9138013	0.005365543
A_22_P00001217	*ZNF709*	Homo sapiens zinc finger protein 709 (ZNF709), mRNA [NM_152601]	-0.5069269	0.011140108
A_32_P60459	*PGAM5*	Homo sapiens phosphoglycerate mutase family member 5 (PGAM5), transcript variant 3, mRNA [NM_138575]	1.2349868	0.010062054
A_33_P3389286	*lnc-ANKRD11-1*	AGENCOURT_7892117 NIH_MGC_67 Homo sapiens cDNA clone IMAGE:6139956 5', mRNA sequence [BU187512]	-0.63724715	0.01582957
A_23_P10815	*OTUD1*	Homo sapiens OTU deubiquitinase 1 (OTUD1), mRNA [NM_001145373]	-1.0136019	0.011140108
A_22_P00012038	*SFN*	Homo sapiens stratifin (SFN), mRNA [NM_006142]	5.4791813	0.003162355
A_23_P8281	*PUM1*	Homo sapiens pumilio RNA-binding family member 1 (PUM1), transcript variant 1, mRNA [NM_001020658]	-0.7605857	0.01395132
A_33_P3259620	*MTURN*	Homo sapiens maturin, neural progenitor differentiation regulator homolog (Xenopus) (MTURN), mRNA [NM_152793]	-1.4160385	0.0071947672
A_23_P115636	*IFNGR1*	Homo sapiens interferon gamma receptor 1 (IFNGR1), mRNA [NM_000416]	-1.3528898	0.0046675983
A_21_P0013753	*TTC37*	Homo sapiens tetratricopeptide repeat domain 37 (TTC37), mRNA [NM_014639]	-0.71492577	0.009113876
A_33_P3246883	*TRDMT1*	Homo sapiens tRNA aspartic acid methyltransferase 1 (TRDMT1), mRNA [NM_004412]	-12266994	0.0025001846
A_22_P00013673	*XLOC_l2_015451*	BROAD Institute lincRNA (XLOC_l2_015451), lincRNA [TCONS_l2_00030147]	-0.40773505	0.005098191
A_23_P139396	*DMKN*	Homo sapiens dermokine (DMKN), transcript variant 2, mRNA [NM_033317]	-0.9968463	0.012965681
A_23_P159833	*lnc-RP11-688I9.2.1-1*	UI-HF-ES0-avz-o-20-0-UI.r1 NIH_MGC_213 Homo sapiens cDNA clone IMAGE:30561667 5', mRNA sequence [CF129872]	0.8383268	0.00180587
A_33_P3419481	*C11orf73*	Homo sapiens chromosome 11 open reading frame 73 (C11orf73), transcript variant 1, mRNA [NM_016401]	-0.93839526	0.007078901
A_23_P15542	*NDUFA1*	Homo sapiens NADH dehydrogenase (ubiquinone) 1 alpha subcomplex, 1, 7.5kDa (NDUFA1), mRNA [NM_004541]	-0.7355461	0.0024890031

A_24_P915692	*HSD17B1*	Homo sapiens hydroxysteroid (17-beta) dehydrogenase 1 (HSD17B1), mRNA [NM_000413]	-0.9398814	0.014448771
A_24_P247454	*ELF1*	Homo sapiens E74-like factor 1 (ets domain transcription factor) (ELF1), transcript variant 2, mRNA [NM_001145353]	-1.1943648	0.0073844297
A_24_P392690	*PHLDA1*	Homo sapiens pleckstrin homology-like domain, family A, member 1 (PHLDA1), mRNA [NM_007350]	1.3856993	0.00410343
A_23_P78053	*MALL*	Homo sapiens mal, T-cell differentiation protein-like (MALL), mRNA [NM_005434]	-3.8308477	0.0024890031
A_23_P39814	*FAM117A*	Homo sapiens family with sequence similarity 117, member A (FAM117A), mRNA [NM_030802]	-2.1759179	0.0062013934
A_23_P377376	*MAF1*	Homo sapiens MAF1 homolog (S. cerevisiae) (MAF1), mRNA [NM_032272]	1.2307721	0.0143041955
A_33_P3280927	*CIR1*	Homo sapiens corepressor interacting with RBPJ, 1 (CIR1), mRNA [NM_004882]	-0.4735394	0.006573993
A_23_P200741	*ACTR2*	Homo sapiens ARP2 actin-related protein 2 homolog (yeast) (ACTR2), transcript variant 1, mRNA [NM_001005386]	-0.45678967	0.004327546
A_22_P00008116	*SNHG7*	Homo sapiens small nucleolar RNA host gene 7 (non-protein coding) (SNHG7), transcript variant 2, long non-coding RNA [NR_024542]	-1.3371111	0.003956951
A_23_P500410	*DPT*	Homo sapiens dermatopontin (DPT), mRNA [NM_001937]	-3.047867	0.0041847117
A_33_P3406904	*lnc-IL23A-2*	LNCipedia lincRNA (lnc-IL23A-2), lincRNA [lnc-IL23A-2:1]	-0.7803076	0.011409725
A_24_P297098	*ATP6V1G2*	Homo sapiens ATPase, H+ transporting, lysosomal 13kDa, V1 subunit G2 (ATP6V1G2), transcript variant 1, mRNA [NM_130463]	-1.5195612	0.00842156
A_19_P00315649	*LOC101928561*	PREDICTED: Homo sapiens uncharacterized LOC101928561 (LOC101928561), ncRNA [XR_246464]	-0.9750383	0.013419309
A_23_P251268	*PHF20*	Homo sapiens PHD finger protein 20 (PHF20), mRNA [NM_016436]	-0.74369556	0.016733099
A_33_P3231653	*LOC100507165*	PREDICTED: Homo sapiens uncharacterized LOC100507165 (LOC100507165), ncRNA [XR_110530]	-2.5222707	0.0025001846
A_24_P915371	*LINC00852*	Homo sapiens long intergenic non-protein coding RNA 852 (LINC00852), long non-coding RNA [NR_026829]	1.8604927	0.009765222
A_33_P3256257	*GOLM1*	Homo sapiens golgi membrane protein 1 (GOLM1), transcript variant 1, mRNA [NM_016548]	-1.009185	0.0108471485
A_23_P59988	*MYEOV2*	Homo sapiens myeloma overexpressed 2 (MYEOV2), transcript variant 2, mRNA [NM_001163424]	-0.64723057	0.005612091

A_21_P0014877	*KRTAP4-7*	Homo sapiens keratin associated protein 4-7 (KRTAP4-7), mRNA [NM_033061]	0.66578686	0.014842539
A_33_P3275835	*SLC35G5*	Homo sapiens solute carrier family 35, member G5 (SLC35G5), mRNA [NM_054028]	-1.0039227	0.0073845843
A_23_P106391	*TOR2A*	Homo sapiens torsin family 2, member A (TOR2A), transcript variant 3, mRNA [NM_001134430]	0.900743	0.0041847117
A_22_P00019097	*ETAA1*	Homo sapiens Ewing tumor-associated antigen 1 (ETAA1), mRNA [NM_019002]	-0.9352716	0.0063101933
A_21_P0000115	*THAP10*	Homo sapiens THAP domain containing 10 (THAP10), mRNA [NM_020147]	-1.0512013	0.0060941027
A_33_P3360555	*CNPY2*	Homo sapiens canopy FGF signaling regulator 2 (CNPY2), transcript variant 2, mRNA [NM_001190991]	-0.8687377	0.010719198
A_33_P3230264	*ARFIP1*	Homo sapiens ADP-ribosylation factor interacting protein 1 (ARFIP1), transcript variant 1, mRNA [NM_001025595]	-0.9204207	0.013890939
A_21_P0014483	*ZNF444*	Homo sapiens zinc finger protein 444 (ZNF444), transcript variant 2, mRNA [NM_001253792]	-0.83779496	0.015000365
A_24_P317762	*GPC3*	Homo sapiens glypican 3 (GPC3), transcript variant 1, mRNA [NM_001164617]	-0.4486938	0.00557203
A_23_P7282	*FGD5-AS1*	Homo sapiens FGD5 antisense RNA 1 (FGD5-AS1), transcript variant 1, long non-coding RNA [NR_046251]	-1.0273498	0.0095999
A_33_P3354683	*LY6E*	Homo sapiens lymphocyte antigen 6 complex, locus E (LY6E), transcript variant 1, mRNA [NM_002346]	1.3937259	0.0057647754
A_23_P133058	*ELMOD2*	Homo sapiens ELMO/CED-12 domain containing 2 (ELMOD2), mRNA [NM_153702]	-1.117993	0.0042201984
A_33_P3215803	*PIGS*	Homo sapiens phosphatidylinositol glycan anchor biosynthesis, class S (PIGS), mRNA [NM_033198]	-1.0248255	0.007474992
A_33_P3379456	*MRFAP1L1*	Homo sapiens Morf4 family associated protein 1-like 1 (MRFAP1L1), mRNA [NM_203462]	-1.1311418	0.01083596
A_23_P27332	*EMR1*	Homo sapiens egf-like module containing, mucin-like, hormone receptor-like 1 (EMR1), transcript variant 1, mRNA [NM_001974]	0.75134313	0.016477155
A_21_P0003244	*SH3YL1*	Homo sapiens SH3 and SYLF domain containing 1 (SH3YL1), transcript variant 4, mRNA [NM_001282687]	-1.2591879	0.008153665
A_23_P202594	*TCF4*	Homo sapiens transcription factor 4 (TCF4), transcript variant 2, mRNA [NM_003199]	-1.3174129	0.005822303
A_24_P255303	*MCMBP*	Homo sapiens minichromosome maintenance complex binding protein (MCMBP), transcript variant 1, mRNA [NM_024834]	-0.38443306	0.013419309
A_32_P129752	*lnc-PEAK1.1-1*	LNCipedia lincRNA (lnc-PEAK1.1-1), lincRNA [lnc-PEAK1.1-1:4]	-0.4317975	0.0140619585

A_24_P356453	*TMEM30B*	Homo sapiens transmembrane protein 30B (TMEM30B), mRNA [NM_001017970]	-2.5148873	0.0040526874
A_24_P311926	*TLDC1*	Homo sapiens TBC/LysM-associated domain containing 1 (TLDC1), mRNA [NM_020947]	-0.9582228	0.015811017
A_22_P00015679	*COA3*	Homo sapiens cytochrome c oxidase assembly factor 3 (COA3), mRNA [NM_001040431]	-0.72110385	0.014748011
A_33_P3378284	*HLA-G*	Homo sapiens major histocompatibility complex, class I, G (HLA-G), mRNA [NM_002127]	-0.5694986	0.011110267
A_23_P210608	*COX6B2*	Homo sapiens cytochrome c oxidase subunit VIb polypeptide 2 (testis) (COX6B2), mRNA [NM_144613]	2.9436707	0.0041946988
A_23_P17471	*SNORA79*	Homo sapiens small nucleolar RNA, H/ACA box 79 (SNORA79), small nucleolar RNA [NR_003021]	-1.2058583	0.0035894548
A_23_P256473	*ZNF217*	Homo sapiens zinc finger protein 217 (ZNF217), mRNA [NM_006526]	-1.432266	0.0029015075
A_23_P103672	*PCED1A*	Homo sapiens PC-esterase domain containing 1A (PCED1A), transcript variant 1, mRNA [NM_022760]	1.1486958	0.0064200144
A_23_P48099	*SEMA3C*	Homo sapiens sema domain, immunoglobulin domain (Ig), short basic domain, secreted, (semaphorin) 3C (SEMA3C), mRNA [NM_006379]	-0.6482551	0.008153665
A_23_P206371	*NES*	Homo sapiens nestin (NES), mRNA [NM_006617]	1.1426728	0.014360646
A_21_P0004054	*NUP37*	Homo sapiens nucleoporin 37kDa (NUP37), mRNA [NM_024057]	-0.7123053	0.0105506545
A_23_P146512	*NOL3*	Homo sapiens nucleolar protein 3 (apoptosis repressor with CARD domain) (NOL3), transcript variant 2, mRNA [NM_003946]	-0.7529356	0.009591824
A_33_P3311473	*GOLM1*	Homo sapiens golgi membrane protein 1 (GOLM1), transcript variant 1, mRNA [NM_016548]	-0.9781046	0.0054365364
A_24_P257416	*ENC1*	Homo sapiens ectodermal-neural cortex 1 (with BTB domain) (ENC1), transcript variant 1, mRNA [NM_003633]	-0.9762724	0.0039856653
A_23_P79836	*ATP10D*	Homo sapiens ATPase, class V, type 10D (ATP10D), mRNA [NM_020453]	-1.2664138	0.0058197915
A_19_P00319095	*CXCL2*	Homo sapiens chemokine (C-X-C motif) ligand 2 (CXCL2), mRNA [NM_002089]	3.5809116	0.003956951
A_32_P193322	*SERINC3*	Homo sapiens serine incorporator 3 (SERINC3), transcript variant 1, mRNA [NM_006811]	-1.0120679	0.012965635
A_23_P303155	*SNHG5*	Homo sapiens small nucleolar RNA host gene 5 (non-protein coding) (SNHG5), long non-coding RNA [NR_003038]	-0.49928898	0.009218792

A_21_P0004468	*RICTOR*	Homo sapiens RPTOR independent companion of MTOR, complex 2 (RICTOR), transcript variant 1, mRNA [NM_152756]	-0.8195641	0.0043705306
A_32_P82475	*TMEM87B*	Homo sapiens transmembrane protein 87B (TMEM87B), mRNA [NM_032824]	1.0313275	0.0074739116
A_22_P00016911	*lnc-FBXW11-2*	LNCipedia lincRNA (lnc-FBXW11-2), lincRNA [lnc-FBXW11-2:1]	-0.8230657	0.0035082079
A_23_P97990	*SNHG10*	Homo sapiens small nucleolar RNA host gene 10 (non-protein coding) (SNHG10), transcript variant 1, long non-coding RNA [NR_003138]	-0.39057648	0.010272158
A_23_P107653	*RNU4ATAC*	Homo sapiens RNA, U4atac small nuclear (U12-dependent splicing) (RNU4ATAC), small nuclear RNA [NR_023343]	0.98907894	0.01530907
A_33_P3249534	*HTRA1*	Homo sapiens HtrA serine peptidase 1 (HTRA1), mRNA [NM_002775]	-1.5112648	0.0048015774
A_33_P3342285	*ETFB*	Homo sapiens electron-transfer-flavoprotein, beta polypeptide (ETFB), transcript variant 2, mRNA [NM_001014763]	-0.69934654	0.0118191205
A_24_P49687	*NEFM*	Homo sapiens neurofilament, medium polypeptide (NEFM), transcript variant 1, mRNA [NM_005382]	-1.7012038	0.0052045966
A_24_P286013	*RAP1A*	Homo sapiens RAP1A, member of RAS oncogene family (RAP1A), transcript variant 1, mRNA [NM_001010935]	-0.86235124	0.008357089
A_33_P3241269	*UBE2K*	Homo sapiens ubiquitin-conjugating enzyme E2K (UBE2K), transcript variant 1, mRNA [NM_005339]	-0.7638886	0.007923661
A_21_P0001029	*LOH12CR1*	Homo sapiens loss of heterozygosity, 12, chromosomal region 1 (LOH12CR1), transcript variant 1, mRNA [NM_058169]	-0.8158615	0.007070614
A_33_P3325102	*CES1*	Homo sapiens carboxylesterase 1 (CES1), transcript variant 1, mRNA [NM_001025195]	0.9414674	0.013367055
A_23_P87049	*CD274*	Homo sapiens CD274 molecule (CD274), transcript variant 1, mRNA [NM_014143]	2.0944102	0.0034903844
A_33_P3269539	*XLOC_l2_015812*	BROAD Institute lincRNA (XLOC_l2_015812), lincRNA [TCONS_l2_00030590]	0.8044013	0.010732792
A_22_P00015975	*SORL1*	Homo sapiens sortilin-related receptor, L(DLR class) A repeats containing (SORL1), mRNA [NM_003105]	1.70874	0.006173301
A_21_P0007273	*COL6A2*	Homo sapiens collagen, type VI, alpha 2 (COL6A2), transcript variant 2C2a, mRNA [NM_058174]	0.9194312	0.00626104
A_23_P258769	*lnc-TELO2-3*	LNCipedia lincRNA (lnc-TELO2-3), lincRNA [lnc-TELO2-3:1]	0.741916	0.011087365
A_23_P78014	*lnc-ATM-1*	LNCipedia lincRNA (lnc-ATM-1), lincRNA [lnc-ATM-1:1]	-0.7793125	0.00959983

A_33_P3418731	*HLA-DPB1*	Homo sapiens major histocompatibility complex, class II, DP beta 1 (HLA-DPB1), mRNA [NM_002121]	-2.1119332	0.0044813035
A_23_P50735	*TUBG2*	Homo sapiens tubulin, gamma 2 (TUBG2), mRNA [NM_016437]	0.7963024	0.015866216
A_23_P151267	*ZNF542P*	Homo sapiens zinc finger protein 542, pseudogene (ZNF542P), transcript variant 4, non-coding RNA [NR_003127]	-0.8126383	0.016092481
A_22_P00002500	*ZNF181*	Homo sapiens zinc finger protein 181 (ZNF181), transcript variant 1, mRNA [NM_001029997]	-1.031969	0.011743996
A_23_P34144	*LIMA1*	Homo sapiens LIM domain and actin binding 1 (LIMA1), transcript variant 2, mRNA [NM_016357]	-1.2584305	0.0054438785
A_33_P3304764	*lnc-C16orf61-2*	Homo sapiens cDNA clone IMAGE:4248887, partial cds. [BC017893]	-1.2297691	0.006632856
A_32_P150856	*MAGEH1*	Homo sapiens melanoma antigen family H, 1 (MAGEH1), mRNA [NM_014061]	-0.77603245	0.010220027
A_23_P127840	*LOC407835*	Homo sapiens mitogen-activated protein kinase kinase 2 pseudogene (LOC407835), non-coding RNA [NR_002144]	0.84853953	0.0114562
A_21_P0014192	*ATL1*	Homo sapiens atlastin GTPase 1 (ATL1), transcript variant 2, mRNA [NM_181598]	-1.7354695	0.009231221
A_23_P211212	*ZNF214*	Homo sapiens zinc finger protein 214 (ZNF214), mRNA [NM_013249]	-1.0082977	0.0098156035
A_23_P79302	*COL18A1*	Homo sapiens collagen, type XVIII, alpha 1 (COL18A1), transcript variant 1, mRNA [NM_030582]	1.237865	0.0033066468
A_33_P3229161	*C2CD5*	Homo sapiens C2 calcium-dependent domain containing 5 (C2CD5), transcript variant 6, mRNA [NM_014802]	-0.9453771	0.0058197915
A_21_P0014096	*LYPD6B*	Homo sapiens LY6/PLAUR domain containing 6B (LYPD6B), mRNA [NM_177964]	1.0758085	0.011118167
A_23_P212213	*UTP14C*	Homo sapiens UTP14, U3 small nucleolar ribonucleoprotein, homolog C (yeast) (UTP14C), mRNA [NM_021645]	-0.85335183	0.011870703
A_33_P3244181	*THUMPD3*	Homo sapiens THUMP domain containing 3 (THUMPD3), transcript variant 1, mRNA [NM_015453]	-0.83535093	0.009924457
A_33_P3243832	*TMEM14C*	Homo sapiens transmembrane protein 14C (TMEM14C), transcript variant 1, mRNA [NM_001165258]	-0.78405595	0.005842833
A_33_P3330413	*HSBP1*	Homo sapiens heat shock factor binding protein 1 (HSBP1), mRNA [NM_001537]	-0.7352335	0.0060600177
A_24_P808522	*ZEB2*	Homo sapiens zinc finger E-box binding homeobox 2 (ZEB2), transcript variant 1, mRNA [NM_014795]	-1.5495632	0.0060695945

A_22_P00017915	*RPS14*	Homo sapiens ribosomal protein S14 (RPS14), transcript variant 3, mRNA [NM_005617]	0.34322217	0.007835731
A_22_P00009936	*LOC101929484*	PREDICTED: Homo sapiens uncharacterized LOC101929484 (LOC101929484), ncRNA [XR_245642]	-2.8808403	0.006024328
A_33_P3360823	*ZNF883*	Homo sapiens zinc finger protein 883 (ZNF883), mRNA [NM_001101338]	1.5321399	0.01666397
A_23_P384698	*HCP5*	Homo sapiens HLA complex P5 (non-protein coding) (HCP5), long non-coding RNA [NR_040662]	0.48610303	0.011383067
A_22_P00016181	*C10orf88*	Homo sapiens chromosome 10 open reading frame 88 (C10orf88), mRNA [NM_024942]	-0.55428505	0.007300493
A_33_P3214597	*EMC3-AS1*	Homo sapiens EMC3 antisense RNA 1 (EMC3-AS1), long non-coding RNA [NR_103821]	1.2542362	0.0054972414
A_32_P157481	*RP9P*	Homo sapiens retinitis pigmentosa 9 pseudogene (RP9P), non-coding RNA [NR_003500]	-1.0633007	0.013441296
A_33_P3329652	*GAA*	Homo sapiens glucosidase, alpha; acid (GAA), transcript variant 1, mRNA [NM_000152]	-2.4792776	0.003945209
A_23_P47704	*ERCC4*	Homo sapiens excision repair cross-complementation group 4 (ERCC4), mRNA [NM_005236]	-0.67603517	0.011772038
A_33_P3355371	*ZFP90*	Homo sapiens ZFP90 zinc finger protein (ZFP90), mRNA [NM_133458]	-0.8584342	0.010822477
A_24_P153043	*UCP2*	Homo sapiens uncoupling protein 2 (mitochondrial, proton carrier) (UCP2), mRNA [NM_003355]	3.113482	0.003956951
A_33_P3219469	*TTC9C*	tetratricopeptide repeat domain 9C [Source:HGNC Symbol;Acc:HGNC:28432] [ENST00000294161]	-1.9580386	0.005612091
A_33_P3216090	*RPL23AP7*	Homo sapiens ribosomal protein L23a pseudogene 7 (RPL23AP7), transcript variant 3, non-coding RNA [NR_000029]	-0.7361367	0.011152497
A_23_P314101	*SCAF11*	Homo sapiens SR-related CTD-associated factor 11 (SCAF11), mRNA [NM_004719]	-0.75791335	0.004370471
A_24_P672240	*SHC3*	Homo sapiens SHC (Src homology 2 domain containing) transforming protein 3 (SHC3), mRNA [NM_016848]	4.4816556	0.0025451183
A_33_P3593546	*SUSD2*	Homo sapiens sushi domain containing 2 (SUSD2), mRNA [NM_019601]	2.3214726	0.008559404
A_24_P374586	*FRMPD4*	Homo sapiens FERM and PDZ domain containing 4 (FRMPD4), mRNA [NM_014728]	-1.6099029	0.004993122
A_32_P32413	*MARK3*	Homo sapiens MAP/microtubule affinity-regulating kinase 3 (MARK3), transcript variant 1, mRNA [NM_001128918]	-0.682543	0.011730079
A_33_P3245248	*PMS2*	Homo sapiens PMS2 postmeiotic segregation increased 2 (S. cerevisiae) (PMS2), transcript variant 1, mRNA [NM_000535]	-0.49632424	0.012211056

A_23_P145114	*SETBP1*	Homo sapiens SET binding protein 1 (SETBP1), transcript variant 1, mRNA [NM_015559]	-2.4850864	0.004160909
A_33_P3372099	*TERC*	Homo sapiens telomerase RNA component (TERC), telomerase RNA [NR_001566]	0.36882374	0.00962867
A_21_P0002422	*GCLC*	Homo sapiens glutamate-cysteine ligase, catalytic subunit (GCLC), transcript variant 1, mRNA [NM_001498]	-0.76140755	0.005457504
A_21_P0011010	*DDIT4L*	Homo sapiens DNA-damage-inducible transcript 4-like (DDIT4L), mRNA [NM_145244]	0.68332225	0.007607114
A_23_P169050	*LINC00211*	Homo sapiens long intergenic non-protein coding RNA 211 (LINC00211), transcript variant 1, long non-coding RNA [NR_110011]	-1.4077482	0.005651698
A_21_P0000140	*XLOC_l2_002651*	BROAD Institute lincRNA (XLOC_l2_002651), lincRNA [TCONS_l2_00005011]	-1.1509194	0.0055675246
A_23_P20022	*MRPS28*	Homo sapiens mitochondrial ribosomal protein S28 (MRPS28), mRNA [NM_014018]	-0.43951565	0.008437608
A_23_P212511	*LEPROT*	Homo sapiens leptin receptor overlapping transcript (LEPROT), transcript variant 3, mRNA [NM_001198683]	-0.4190364	0.0056098294
A_21_P0011619	*HILPDA*	Homo sapiens hypoxia inducible lipid droplet-associated (HILPDA), transcript variant 1, mRNA [NM_013332]	-0.70593995	0.008244355
A_33_P3215128	*TTC14*	Homo sapiens tetratricopeptide repeat domain 14 (TTC14), transcript variant 2, mRNA [NM_001042601]	-0.4828872	0.015309069
A_23_P110941	*TBC1D3P5*	Homo sapiens TBC1 domain family, member 3 pseudogene 5 (TBC1D3P5), non-coding RNA [NR_033892]	-1.1295792	0.0035935708
A_33_P3262717	*IBA57*	Homo sapiens IBA57, iron-sulfur cluster assembly homolog (S. cerevisiae) (IBA57), mRNA [NM_001010867]	-0.8720921	0.009630889
A_24_P166443	*GSTA4*	Homo sapiens glutathione S-transferase alpha 4 (GSTA4), mRNA [NM_001512]	-1.6520703	0.005796753
A_33_P3219055	*HLA-DPB1*	Homo sapiens major histocompatibility complex, class II, DP beta 1 (HLA-DPB1), mRNA [NM_002121]	-1.8969368	0.0038456172
A_24_P398130	*C16orf13*	Homo sapiens chromosome 16 open reading frame 13 (C16orf13), transcript variant 8, non-coding RNA [NR_109978]	1.4004165	0.0056025535
A_22_P00009695	*C15orf61*	Homo sapiens chromosome 15 open reading frame 61 (C15orf61), mRNA [NM_001143936]	-1.3130455	0.004510206
A_33_P3257170	*USP6NL*	Homo sapiens USP6 N-terminal like (USP6NL), transcript variant 1, mRNA [NM_014688]	-0.7000581	0.017064169
A_23_P204364	*PRR3*	proline rich 3 [Source:HGNC Symbol;Acc:HGNC:21149] [ENST00000461523]	-0.8322441	0.003365343
A_32_P86739	*PFKFB3*	Homo sapiens 6-phosphofructo-2-kinase/fructose-2,6-biphosphatase 3 (PFKFB3), transcript variant 1, mRNA [NM_004566]	1.1900309	0.015868904

A_21_P0011262	*NOP2*	Homo sapiens NOP2 nucleolar protein (NOP2), transcript variant 2, mRNA [NM_001033714]	0.9809086	0.012479078
A_23_P99253	*CASC10*	Homo sapiens cancer susceptibility candidate 10 (CASC10), mRNA [NM_001010911]	-1.1180607	0.01352988
A_22_P00023898	*XPO6*	Homo sapiens exportin 6 (XPO6), transcript variant 1, mRNA [NM_001270940]	1.3802732	0.008437608
A_22_P00017323	*LIN7A*	Homo sapiens lin-7 homolog A (C. elegans) (LIN7A), mRNA [NM_004664]	1.3334948	0.0053870557
A_21_P0014740	*lnc-PHYHD1-1*	LNCipedia lincRNA (lnc-PHYHD1-1), lincRNA [lnc-PHYHD1-1:1]	-0.73219866	0.012368504
A_19_P00809076	*KCTD21-AS1*	Homo sapiens KCTD21 antisense RNA 1 (KCTD21-AS1), transcript variant 2, long non-coding RNA [NR_102281]	0.78983575	0.0105506545
A_23_P423197	*KANSL1-AS1*	Homo sapiens KANSL1 antisense RNA 1 (KANSL1-AS1), long non-coding RNA [NR_034172]	1.0447731	0.006052523
A_22_P00015929	*FAM110A*	Homo sapiens family with sequence similarity 110, member A (FAM110A), transcript variant 4, mRNA [NM_001289145]	0.74305016	0.0076440917
A_33_P3218832	*RXRA*	Homo sapiens retinoid X receptor, alpha (RXRA), transcript variant 1, mRNA [NM_002957]	-1.3507395	0.0044240346
A_23_P420610	*lnc-TCN2-1*	LNCipedia lincRNA (lnc-TCN2-1), lincRNA [lnc-TCN2-1:1]	0.76738876	0.009135723
A_33_P3256347	*RIMS1*	Homo sapiens regulating synaptic membrane exocytosis 1 (RIMS1), transcript variant 1, mRNA [NM_014989]	-3.743723	0.0053354
A_32_P136402	*FCHO2*	Homo sapiens FCH domain only 2 (FCHO2), transcript variant 1, mRNA [NM_138782]	-0.9635477	0.015445575
A_33_P3327454	*TIMM8B*	Homo sapiens translocase of inner mitochondrial membrane 8 homolog B (yeast) (TIMM8B), transcript variant 1, mRNA [NM_012459]	-0.8034448	0.004699213
A_24_P268993	*THOC7*	Homo sapiens THO complex 7 homolog (Drosophila) (THOC7), transcript variant 1, mRNA [NM_025075]	-0.6735246	0.005198761
A_24_P226755	*RSPRY1*	Homo sapiens ring finger and SPRY domain containing 1 (RSPRY1), mRNA [NM_133368]	-1.1189277	0.003162355
A_23_P110266	*LEAP2*	Homo sapiens liver expressed antimicrobial peptide 2 (LEAP2), mRNA [NM_052971]	-0.8766608	0.013163641
A_23_P25121	*TOX*	Homo sapiens thymocyte selection-associated high mobility group box (TOX), mRNA [NM_014729]	-0.8656083	0.0065241847
A_33_P3370094	*NDNF*	Homo sapiens neuron-derived neurotrophic factor (NDNF), mRNA [NM_024574]	-4.586475	0.0025001846
A_23_P206396	*FKBP11*	Homo sapiens FK506 binding protein 11, 19 kDa (FKBP11), transcript variant 1, mRNA [NM_016594]	-1.4338888	0.005595418

A_23_P408285	*MME*	Homo sapiens membrane metallo-endopeptidase (MME), transcript variant 2b, mRNA [NM_007289]	-0.6789833	0.005438366
A_23_P6909	*CKLF*	Homo sapiens chemokine-like factor (CKLF), transcript variant 5, mRNA [NM_001040138]	-0.7842994	0.0065698107
A_33_P3364493	*PRICKLE1*	Homo sapiens prickle homolog 1 (Drosophila) (PRICKLE1), transcript variant 1, mRNA [NM_153026]	1.2011507	0.013467389
A_23_P418031	*ACKR4*	Homo sapiens atypical chemokine receptor 4 (ACKR4), transcript variant 1, mRNA [NM_178445]	-0.7435324	0.009160365
A_23_P364517	*IFFO2*	Homo sapiens intermediate filament family orphan 2 (IFFO2), mRNA [NM_001136265]	-0.9342184	0.009501149
A_33_P3223093	*DTX3*	Homo sapiens deltex 3, E3 ubiquitin ligase (DTX3), transcript variant 1, mRNA [NM_178502]	1.4090163	0.005936798
A_23_P7727	*SELK*	Homo sapiens selenoprotein K (SELK), mRNA [NM_021237]	-0.9730406	0.008471989
A_33_P3363799	*HAPLN1*	Homo sapiens hyaluronan and proteoglycan link protein 1 (HAPLN1), mRNA [NM_001884]	0.60103893	0.0077551813
A_22_P00003484	*lnc-ZPLD1-1*	LNCipedia lincRNA (lnc-ZPLD1-1), lincRNA [lnc-ZPLD1-1:1]	-1.1758873	0.014548385
A_33_P3278159	*NCAM1*	Homo sapiens neural cell adhesion molecule 1 (NCAM1), transcript variant 5, mRNA [NM_001242607]	-3.5180678	0.004055852
A_23_P57856	*JRKL-AS1*	Homo sapiens JRKL antisense RNA 1 (JRKL-AS1), long non-coding RNA [NR_047481]	-0.6081482	0.005852893
A_24_P281988	*BCL6*	Homo sapiens B-cell CLL/lymphoma 6 (BCL6), transcript variant 2, mRNA [NM_001130845]	-1.1081977	0.014416893
A_33_P3265374	*METTL25*	Homo sapiens methyltransferase like 25 (METTL25), mRNA [NM_032230]	-1.0178924	0.0043846127
A_24_P79153	*PLCL2*	Homo sapiens phospholipase C-like 2 (PLCL2), transcript variant 2, mRNA [NM_015184]	-0.79841965	0.008923173
A_23_P396328	*HOMER3*	Homo sapiens homer homolog 3 (Drosophila) (HOMER3), transcript variant 1, mRNA [NM_001145722]	-0.6069105	0.009794734
A_33_P3327479	*SCAMP4*	Homo sapiens secretory carrier membrane protein 4 (SCAMP4), mRNA [NM_079834]	1.0040653	0.00789076
A_23_P215778	*MPDZ*	Homo sapiens multiple PDZ domain protein (MPDZ), transcript variant 1, mRNA [NM_003829]	-0.5115764	0.0093693705
A_22_P00012339	*ZDHHC3*	Homo sapiens zinc finger, DHHC-type containing 3 (ZDHHC3), transcript variant 2, mRNA [NM_016598]	-1.2492311	0.0044785915
A_23_P141302	*DUS4L*	Homo sapiens dihydrouridine synthase 4-like (S. cerevisiae) (DUS4L), transcript variant 1, mRNA [NM_181581]	-0.76655114	0.008367874

A_21_P0002505	*lnc-PRDM13-4*	LNCipedia lincRNA (lnc-PRDM13-4), lincRNA [lnc-PRDM13-4:1]	-1.4326684	0.013076766
A_23_P347541	*ZNF18*	Homo sapiens zinc finger protein 18 (ZNF18), transcript variant 1, mRNA [NM_144680]	-1.1789808	0.005911952
A_32_P68533	*lnc-C2orf27B-3*	LNCipedia lincRNA (lnc-C2orf27B-3), lincRNA [lnc-C2orf27B-3:2]	-0.95612603	0.006901566
A_21_P0011609	*GRIN3A*	Homo sapiens glutamate receptor, ionotropic, N-methyl-D-aspartate 3A (GRIN3A), mRNA [NM_133445]	0.9991322	0.015141116
A_21_P0000082	*FAM161A*	Homo sapiens family with sequence similarity 161, member A (FAM161A), transcript variant 2, mRNA [NM_032180]	-0.56339866	0.010443572
A_24_P346431	*LOC100130520*	PREDICTED: Homo sapiens CD300c molecule-like (LOC100130520), transcript variant X3, mRNA [XM_005275830]	0.62569404	0.006540455
A_23_P845	*LARP4*	Homo sapiens La ribonucleoprotein domain family, member 4 (LARP4), transcript variant 2, mRNA [NM_199188]	-0.77052635	0.0056601833
A_23_P47614	*TNS3*	Homo sapiens tensin 3 (TNS3), mRNA [NM_022748]	1.5551593	0.005142579
A_32_P224522	*TOMM40L*	Homo sapiens translocase of outer mitochondrial membrane 40 homolog (yeast)-like (TOMM40L), transcript variant 1, mRNA [NM_032174]	-0.8759167	0.011618324
A_23_P96087	*PHLDA2*	Homo sapiens pleckstrin homology-like domain, family A, member 2 (PHLDA2), mRNA [NM_003311]	0.685383	0.005640775
A_32_P213330	*SLC25A23*	Homo sapiens solute carrier family 25 (mitochondrial carrier; phosphate carrier), member 23 (SLC25A23), mRNA [NM_024103]	-0.9295714	0.006474282
A_23_P202565	*H1FX*	Homo sapiens H1 histone family, member X (H1FX), mRNA [NM_006026]	1.3886262	0.0031750489
A_33_P3403937	*ARHGEF28*	Homo sapiens Rho guanine nucleotide exchange factor (GEF) 28 (ARHGEF28), transcript variant 2, mRNA [NM_001177693]	2.4622378	0.0040699136
A_24_P142024	*SHOC2*	Homo sapiens soc-2 suppressor of clear homolog (C. elegans) (SHOC2), transcript variant 1, mRNA [NM_007373]	-1.3932784	0.00180587
A_24_P456723	*LOC100129399*	Homo sapiens cDNA FLJ42017 fis, clone SPLEN2033153. [AK124011]	-0.83999586	0.0163094
A_33_P3886707	*CHMP4A*	Homo sapiens charged multivesicular body protein 4A (CHMP4A), mRNA [NM_014169]	-0.8793025	0.012626196
A_22_P00012424	*LINC01125*	Homo sapiens long intergenic non-protein coding RNA 1125 (LINC01125), long non-coding RNA [NR_038386]	-0.896543	0.004738647
A_22_P00024857	*PPA2*	Homo sapiens pyrophosphatase (inorganic) 2 (PPA2), transcript variant 1, mRNA [NM_176869]	-0.9590161	0.0038456172

A_33_P3400728	*LOC101927071*	PREDICTED: Homo sapiens uncharacterized LOC101927071 (LOC101927071), ncRNA [XR_243350]	0.8580542	0.008465735
A_23_P365189	*LOC100288798*	Homo sapiens uncharacterized LOC100288798 (LOC100288798), transcript variant 1, long non-coding RNA [NR_125377]	0.96874505	0.013656562
A_23_P369899	*RIPK4*	Homo sapiens receptor-interacting serine-threonine kinase 4 (RIPK4), mRNA [NM_020639]	0.6599339	0.015419179
A_23_P167812	*CAMTA2*	Homo sapiens calmodulin binding transcription activator 2 (CAMTA2), transcript variant 1, mRNA [NM_015099]	1.0921319	0.016739154
A_23_P41948	*TMEM158*	Homo sapiens transmembrane protein 158 (gene/pseudogene) (TMEM158), mRNA [NM_015444]	2.523854	0.0016880932
A_33_P3414851	*RBM24*	Homo sapiens RNA binding motif protein 24 (RBM24), transcript variant 2, mRNA [NM_153020]	-1.018396	0.005092817
A_23_P435657	*SPDL1*	Homo sapiens spindle apparatus coiled-coil protein 1 (SPDL1), mRNA [NM_017785]	-0.6250293	0.007867977
A_23_P37560	*RNF220*	Homo sapiens ring finger protein 220 (RNF220), mRNA [NM_018150]	1.0725487	0.0080731055
A_23_P142750	*ALMS1P*	Homo sapiens Alstrom syndrome 1 pseudogene (ALMS1P), non-coding RNA [NR_003683]	-0.45967236	0.010548865
A_21_P0010771	*PEX11A*	Homo sapiens peroxisomal biogenesis factor 11 alpha (PEX11A), transcript variant 1, mRNA [NM_003847]	-0.69158614	0.009152166
A_22_P00016058	*EIF2AK2*	Homo sapiens eukaryotic translation initiation factor 2-alpha kinase 2 (EIF2AK2), transcript variant 1, mRNA [NM_002759]	-0.66729546	0.016782451
A_33_P3395146	*ANKRD20A2*	Homo sapiens ankyrin repeat domain 20 family, member A2 (ANKRD20A2), mRNA [NM_001012421]	-1.2174171	0.0047719665
A_33_P3378126	*LOC100506047*	PREDICTED: Homo sapiens uncharacterized LOC100506047 (LOC100506047), ncRNA [XR_109976]	0.6665115	0.014352553
A_21_P0011960	*LOC646762*	Homo sapiens uncharacterized LOC646762 (LOC646762), long non-coding RNA [NR_024278]	-0.6121676	0.01692151
A_23_P160025	*FBXO32*	Homo sapiens F-box protein 32 (FBXO32), transcript variant 1, mRNA [NM_058229]	-2.7290072	0.0029471128
A_32_P71113	*LOC100506797*	PREDICTED: Homo sapiens uncharacterized LOC100506797 (LOC100506797), transcript variant X1, ncRNA [XR_112377]	-1.1184962	0.013573022
A_33_P3330323	*IFI16*	Homo sapiens interferon, gamma-inducible protein 16 (IFI16), transcript variant 2, mRNA [NM_005531]	-1.2754617	0.002819256

A_21_P0000756	*SCAI*	Homo sapiens suppressor of cancer cell invasion (SCAI), transcript variant 1, mRNA [NM_173690]	-0.8854042	0.01456425
A_23_P60591	*MGA*	Homo sapiens MGA, MAX dimerization protein (MGA), transcript variant 2, mRNA [NM_001080541]	-0.7045551	0.006818124
A_33_P3269803	*LINC01012*	Homo sapiens long intergenic non-protein coding RNA 1012 (LINC01012), transcript variant 2, long non-coding RNA [NR_038293]	1.0083095	0.004657079
A_23_P65442	*DNAJC7*	Homo sapiens DnaJ (Hsp40) homolog, subfamily C, member 7 (DNAJC7), transcript variant 1, mRNA [NM_003315]	-0.6002784	0.009512793
A_24_P149124	*CLSTN3*	Homo sapiens calsyntenin 3 (CLSTN3), mRNA [NM_014718]	-0.68872905	0.006059527
A_33_P3676746	*IRF9*	Homo sapiens interferon regulatory factor 9 (IRF9), mRNA [NM_006084]	-0.9909472	0.011100154
A_23_P250735	*NREP*	Homo sapiens neuronal regeneration related protein (NREP), transcript variant 1, mRNA [NM_004772]	-2.3060482	0.0025001846
A_33_P3683362	*YLPM1*	Homo sapiens YLP motif containing 1 (YLPM1), mRNA [NM_019589]	-0.46604607	0.011189605
A_23_P43175	*CBX7*	Homo sapiens chromobox homolog 7 (CBX7), mRNA [NM_175709]	-1.0245259	0.008208155
A_22_P00001465	*lnc-RNF125-2*	Homo sapiens, clone IMAGE:5745916, mRNA. [BC035844]	-0.65702987	0.00371792
A_24_P55465	*lnc-AQP8-1*	LNCipedia lincRNA (lnc-AQP8-1), lincRNA [lnc-AQP8-1:1]	-0.7515146	0.009291781
A_21_P0013986	*PLXNB2*	Homo sapiens plexin B2 (PLXNB2), mRNA [NM_012401]	0.7676235	0.015416404
A_23_P5389	*MTPN*	Homo sapiens myotrophin (MTPN), mRNA [NM_145808]	-0.81319803	0.0086922655
A_24_P29277	*DPY30*	Homo sapiens dpy-30 homolog (C. elegans) (DPY30), mRNA [NM_032574]	-0.88529587	0.008208044
A_24_P399009	*lnc-AFG3L2-1*	LNCipedia lincRNA (lnc-AFG3L2-1), lincRNA [lnc-AFG3L2-1:1]	0.5477194	0.01368361
A_23_P397208	*COL4A3BP*	Homo sapiens collagen, type IV, alpha 3 (Goodpasture antigen) binding protein (COL4A3BP), transcript variant 3, mRNA [NM_001130105]	-1.096091	0.0046744854
A_23_P128624	*EDC4*	Homo sapiens enhancer of mRNA decapping 4 (EDC4), mRNA [NM_014329]	0.99175537	0.010402669
A_24_P310630	*GSTM2*	Homo sapiens glutathione S-transferase mu 2 (muscle) (GSTM2), transcript variant 1, mRNA [NM_000848]	0.88196576	0.015505246
A_23_P154740	*CARS2*	Homo sapiens cysteinyl-tRNA synthetase 2, mitochondrial (putative) (CARS2), mRNA [NM_024537]	-1.2606771	0.004206936

A_33_P3252781	*UPF3B*	Homo sapiens UPF3 regulator of nonsense transcripts homolog B (yeast) (UPF3B), transcript variant 1, mRNA [NM_080632]	-0.8517996	0.00881236
A_33_P3423285	*KIZ*	Homo sapiens kizuna centrosomal protein (KIZ), transcript variant 1, mRNA [NM_018474]	-1.0668899	0.004701671
A_24_P93855	*PLAC9*	Homo sapiens placenta-specific 9 (PLAC9), mRNA [NM_001012973]	-3.9842165	0.0031941293
A_32_P22257	*lnc-SRGAP3-1*	Homo sapiens hypothetical gene supported by AK128398, mRNA (cDNA clone IMAGE:40146702). [BC132680]	0.9958173	0.013623725
A_24_P38702	*FOXP4*	Homo sapiens forkhead box P4 (FOXP4), transcript variant 1, mRNA [NM_001012426]	1.2554258	0.0034903844
A_23_P148297	*HMGN1*	Homo sapiens high mobility group nucleosome binding domain 1 (HMGN1), mRNA [NM_004965]	-0.72473055	0.005015031
A_24_P117323	*NKX2-3*	Homo sapiens NK2 homeobox 3 (NKX2-3), mRNA [NM_145285]	0.8126458	0.006111203
A_24_P827037	*SH3BGRL*	Homo sapiens SH3 domain binding glutamate-rich protein like (SH3BGRL), mRNA [NM_003022]	-1.1343434	0.005822303
A_32_P101689	*KLHL22*	Homo sapiens kelch-like family member 22 (KLHL22), transcript variant 1, mRNA [NM_032775]	1.0662365	0.007794005
A_23_P123172	*LRRC15*	Homo sapiens leucine rich repeat containing 15 (LRRC15), transcript variant 2, mRNA [NM_130830]	-2.2208512	0.0039655184
A_24_P231829	*FAM3C*	Homo sapiens family with sequence similarity 3, member C (FAM3C), transcript variant 1, mRNA [NM_014888]	-1.0073004	0.004935436
A_33_P3339036	*OR2A9P*	Homo sapiens olfactory receptor, family 2, subfamily A, member 9 pseudogene (OR2A9P), non-coding RNA [NR_002157]	-1.136675	0.013221312
A_24_P228875	*BHMT2*	Homo sapiens betaine--homocysteine S-methyltransferase 2 (BHMT2), transcript variant 1, mRNA [NM_017614]	0.6025779	0.016216509
A_23_P94009	*MECP2*	Homo sapiens methyl CpG binding protein 2 (MECP2), transcript variant 2, mRNA [NM_001110792]	-1.0276902	0.0073958626
A_33_P3322879	*ARHGEF6*	Homo sapiens Rac/Cdc42 guanine nucleotide exchange factor (GEF) 6 (ARHGEF6), mRNA [NM_004840]	-1.3741163	0.003162355
A_23_P253221	*LSM8*	Homo sapiens LSM8 homolog, U6 small nuclear RNA associated (S. cerevisiae) (LSM8), mRNA [NM_016200]	-1.4570665	0.002804883
A_33_P3417695	*LOC102724484*	Homo sapiens uncharacterized LOC102724484 (LOC102724484), long non-coding RNA [NR_120522]	-1.2826146	0.012274017

A_33_P3402725	*ARHGEF4*	Homo sapiens Rho guanine nucleotide exchange factor (GEF) 4 (ARHGEF4), transcript variant 2, mRNA [NM_032995]	-0.99478984	0.008808054
A_33_P3289976	*ODF3B*	Homo sapiens outer dense fiber of sperm tails 3B (ODF3B), mRNA [NM_001014440]	-1.086518	0.011104006
A_23_P141032	*ASH1L*	Homo sapiens ash1 (absent, small, or homeotic)-like (Drosophila) (ASH1L), mRNA [NM_018489]	-0.8464768	0.010827594
A_24_P418619	*COX4I1*	Homo sapiens cytochrome c oxidase subunit IV isoform 1 (COX4I1), mRNA [NM_001861]	-0.5678718	0.008705128
A_21_P0010536	*PRPS1*	Homo sapiens phosphoribosyl pyrophosphate synthetase 1 (PRPS1), transcript variant 1, mRNA [NM_002764]	-1.9398127	0.003637067
A_33_P3321781	*RPS10*	Homo sapiens ribosomal protein S10 (RPS10), transcript variant 2, mRNA [NM_001014]	-0.50371987	0.016260965
A_22_P00025334	*GBP3*	Homo sapiens guanylate binding protein 3 (GBP3), mRNA [NM_018284]	-0.67780757	0.009906083
A_22_P00001935	*LINC01119*	Homo sapiens long intergenic non-protein coding RNA 1119 (LINC01119), long non-coding RNA [NR_024452]	-2.2057397	0.003637067
A_21_P0011387	*H2AFY2*	Homo sapiens H2A histone family, member Y2 (H2AFY2), mRNA [NM_018649]	-0.84093666	0.004169345
A_33_P3332112	*GPR89B*	Homo sapiens G protein-coupled receptor 89B (GPR89B), mRNA [NM_016334]	-0.42808792	0.007111782
A_33_P3231297	*GABRE*	Homo sapiens gamma-aminobutyric acid (GABA) A receptor, epsilon (GABRE), mRNA [NM_004961]	-1.3345115	0.0031166414
A_33_P3301286	*FAS*	Homo sapiens Fas cell surface death receptor (FAS), transcript variant 1, mRNA [NM_000043]	-1.3362008	0.013209052
A_19_P00315493	*CREG1*	Homo sapiens cellular repressor of E1A-stimulated genes 1 (CREG1), mRNA [NM_003851]	-1.2692237	0.0038056036
A_23_P65262	*CUL5*	Homo sapiens cullin 5 (CUL5), mRNA [NM_003478]	-0.8079422	0.005695989
A_24_P387869	*LOC145474*	Homo sapiens uncharacterized LOC145474 (LOC145474), long non-coding RNA [NR_027046]	0.42127153	0.012138538
A_33_P3407065	*N4BP2L2*	Homo sapiens NEDD4 binding protein 2-like 2 (N4BP2L2), transcript variant 3, mRNA [NM_001278432]	-0.9196196	0.00950681
A_21_P0011142	*PKN2*	Homo sapiens protein kinase N2 (PKN2), mRNA [NM_006256]	-0.69019675	0.0067047477
A_23_P309515	*KIF2A*	Homo sapiens kinesin heavy chain member 2A (KIF2A), transcript variant 3, mRNA [NM_001243952]	-0.7208886	0.009014158

A_23_P53363	*NAP1L1*	Homo sapiens nucleosome assembly protein 1-like 1 (NAP1L1), transcript variant 1, mRNA [NM_139207]	-0.9638321	0.003162355
A_33_P3410449	*KCTD18*	Homo sapiens potassium channel tetramerization domain containing 18 (KCTD18), mRNA [NM_152387]	-0.83176756	0.009383018
A_21_P0000479	*XRCC6BP1*	Homo sapiens XRCC6 binding protein 1 (XRCC6BP1), mRNA [NM_033276]	-0.9076252	0.011255692
A_23_P82748	*SCARF2*	Homo sapiens scavenger receptor class F, member 2 (SCARF2), transcript variant 1, mRNA [NM_153334]	0.87628216	0.008358917
A_33_P3320079	*SNORA11D*	Homo sapiens small nucleolar RNA, H/ACA box 11D (SNORA11D), small nucleolar RNA [NR_003711]	-1.1601019	0.0029675725
A_33_P3271323	*ENY2*	Homo sapiens enhancer of yellow 2 homolog (Drosophila) (ENY2), transcript variant 1, mRNA [NM_020189]	-0.6502674	0.010374721
A_24_P273203	*NFIB*	Homo sapiens nuclear factor I/B (NFIB), transcript variant 3, mRNA [NM_005596]	-1.9221288	0.0034766807
A_21_P0000078	*RHOQ*	Homo sapiens ras homolog family member Q (RHOQ), mRNA [NM_012249]	-1.3976045	0.003542592
A_23_P145895	*SPATA22*	Homo sapiens spermatogenesis associated 22 (SPATA22), transcript variant 1, mRNA [NM_001170699]	5.000063	0.013163641
A_23_P135184	*TP53TG1*	Homo sapiens TP53 target 1 (non-protein coding) (TP53TG1), long non-coding RNA [NR_015381]	-0.6598361	0.007683989
A_22_P00010236	*ZNF100*	Homo sapiens zinc finger protein 100 (ZNF100), mRNA [NM_173531]	-0.6397191	0.011575981
A_23_P14876	*RALGDS*	Homo sapiens ral guanine nucleotide dissociation stimulator (RALGDS), transcript variant 2, mRNA [NM_001042368]	-0.4726653	0.0052084886
A_23_P255714	*SRP14*	Homo sapiens signal recognition particle 14kDa (homologous Alu RNA binding protein) (SRP14), transcript variant 1, mRNA [NM_003134]	-0.51575226	0.0029471128
A_23_P144465	*CDV3*	Homo sapiens CDV3 homolog (mouse) (CDV3), transcript variant 2, mRNA [NM_017548]	-0.685554	0.010706546
A_24_P182764	*IFT74*	Homo sapiens intraflagellar transport 74 (IFT74), transcript variant 1, mRNA [NM_025103]	-0.8778306	0.006485141
A_23_P208788	*PAPSS1*	Homo sapiens 3'-phosphoadenosine 5'-phosphosulfate synthase 1 (PAPSS1), mRNA [NM_005443]	-0.36945504	0.008521206
A_24_P290013	*ATG4B*	Homo sapiens autophagy related 4B, cysteine peptidase (ATG4B), transcript variant 2, mRNA [NM_178326]	-0.84576267	0.006155321

A_24_P24790	*C19orf33*	Homo sapiens chromosome 19 open reading frame 33 (C19orf33), mRNA [NM_033520]	-2.3526258	0.0035734554
A_32_P133072	*FAM86B3P*	Homo sapiens family with sequence similarity 86, member B3, pseudogene (FAM86B3P), transcript variant 3, non-coding RNA [NR_024363]	-1.434777	0.006946239
A_23_P86470	*SPON1*	Homo sapiens spondin 1, extracellular matrix protein (SPON1), mRNA [NM_006108]	-3.0004468	0.0029471128
A_22_P00013078	*DCAF6*	Homo sapiens DDB1 and CUL4 associated factor 6 (DCAF6), transcript variant 1, mRNA [NM_018442]	-0.8100951	0.003609745
A_32_P210642	*CH25H*	Homo sapiens cholesterol 25-hydroxylase (CH25H), mRNA [NM_003956]	-3.9914572	0.0029471128
A_33_P3261182	*LINC01089*	Homo sapiens long intergenic non-protein coding RNA 1089 (LINC01089), long non-coding RNA [NR_002809]	1.2121726	0.014450768
A_23_P255569	*EGFL7*	Homo sapiens EGF-like-domain, multiple 7 (EGFL7), transcript variant 2, mRNA [NM_201446]	-1.0437161	0.008853051
A_23_P155765	*POC5*	Homo sapiens POC5 centriolar protein (POC5), transcript variant 1, mRNA [NM_001099271]	-0.76592827	0.0048641856
A_23_P156807	*DUS1L*	Homo sapiens dihydrouridine synthase 1-like (S. cerevisiae) (DUS1L), mRNA [NM_022156]	0.99268293	0.009958825
A_23_P358597	*HMGB2*	Homo sapiens high mobility group box 2 (HMGB2), transcript variant 1, mRNA [NM_002129]	-0.6398859	0.0034964236
A_23_P143047	*CFLAR*	Homo sapiens CASP8 and FADD-like apoptosis regulator (CFLAR), transcript variant 8, mRNA [NM_001202519]	1.2597376	0.0035238392
A_24_P940517	*POPDC3*	Homo sapiens popeye domain containing 3 (POPDC3), transcript variant 1, mRNA [NM_022361]	-0.85206556	0.013327314
A_32_P148796	*ATP6V1E2*	Homo sapiens ATPase, H+ transporting, lysosomal 31kDa, V1 subunit E2 (ATP6V1E2), mRNA [NM_080653]	-0.785387	0.012683343
A_23_P413761	*PCYOX1*	Homo sapiens prenylcysteine oxidase 1 (PCYOX1), mRNA [NM_016297]	-1.0760161	0.005595418
A_32_P101860	*UBXN2B*	Homo sapiens UBX domain protein 2B (UBXN2B), mRNA [NM_001077619]	-0.49939582	0.010859876
A_33_P3217347	*SRSF3*	Homo sapiens serine/arginine-rich splicing factor 3 (SRSF3), transcript variant 1, mRNA [NM_003017]	-0.45729306	0.015446468
A_22_P00023307	*TMEM106A*	Homo sapiens transmembrane protein 106A (TMEM106A), transcript variant 1, mRNA [NM_001291586]	-0.5555722	0.012508982
A_23_P63929	*PJA1*	Homo sapiens praja ring finger 1, E3 ubiquitin protein ligase (PJA1), transcript variant 1, mRNA [NM_145119]	-1.3229635	0.0056962976

A_23_P411814	*lnc-IRF2BP2-3*	LNCipedia lincRNA (lnc-IRF2BP2-3), lincRNA [lnc-IRF2BP2-3:1]	-0.5079468	0.007279599
A_33_P3225685	*WDR11*	Homo sapiens WD repeat domain 11 (WDR11), mRNA [NM_018117]	-0.77522844	0.0057806885
A_22_P00010163	*OSTC*	Homo sapiens oligosaccharyltransferase complex subunit (non-catalytic) (OSTC), transcript variant 1, mRNA [NM_021227]	-1.4115188	0.0024890031
A_23_P87575	*B3GAT3*	Homo sapiens beta-1,3-glucuronyltransferase 3 (B3GAT3), transcript variant 4, mRNA [NM_001288723]	0.6408773	0.010052548
A_33_P3214785	*lnc-MRPS17-1*	Homo sapiens cDNA FLJ12163 fis, clone MAMMA1000594. [AK022225]	-1.1280642	0.0064698174
A_33_P3372466	*CCNT1*	Homo sapiens cyclin T1 (CCNT1), transcript variant a, mRNA [NM_001240]	0.633976	0.014535421
A_23_P350045	*LEPROT*	Homo sapiens cDNA FLJ37482 fis, clone BRAWH2013941. [AK094801]	-13796207	0.006419502
A_23_P9614	*TECR*	Homo sapiens trans-2,3-enoyl-CoA reductase (TECR), transcript variant 1, mRNA [NM_138501]	1.2296326	0.007797447
A_33_P3256888	*REEP5*	Homo sapiens receptor accessory protein 5 (REEP5), mRNA [NM_005669]	-0.8086178	0.005155042
A_23_P165061	*NDUFA4L2*	Homo sapiens NADH dehydrogenase (ubiquinone) 1 alpha subcomplex, 4-like 2 (NDUFA4L2), mRNA [NM_020142]	-0.5421825	0.012237778
A_33_P3396807	*AES*	Homo sapiens amino-terminal enhancer of split (AES), transcript variant 1, mRNA [NM_198969]	-0.7942793	0.006219354
A_21_P0004592	*SERGEF*	Homo sapiens secretion regulating guanine nucleotide exchange factor (SERGEF), transcript variant 1, mRNA [NM_012139]	-1.3194621	0.0054341624
A_33_P3248052	*ATG4C*	Homo sapiens autophagy related 4C, cysteine peptidase (ATG4C), transcript variant 1, mRNA [NM_032852]	-0.2604982	0.015390707
A_24_P203678	*NLGN4Y*	Homo sapiens neuroligin 4, Y-linked (NLGN4Y), transcript variant 2, mRNA [NM_001164238]	-0.95910126	0.009610021
A_23_P70897	*GMFB*	Homo sapiens glia maturation factor, beta (GMFB), mRNA [NM_004124]	-0.6256532	0.007860159
A_33_P3306616	*ACAT1*	Homo sapiens acetyl-CoA acetyltransferase 1 (ACAT1), mRNA [NM_000019]	-0.6490047	0.005514184
A_33_P3424364	*ZCWPW1*	Homo sapiens zinc finger, CW type with PWWP domain 1 (ZCWPW1), transcript variant 1, mRNA [NM_017984]	-1.0755814	0.008437608
A_23_P129801	*CCDC152*	Homo sapiens coiled-coil domain containing 152 (CCDC152), mRNA [NM_001134848]	1.5341729	0.015589794
A_33_P3464555	*KLHDC1*	Homo sapiens kelch domain containing 1 (KLHDC1), mRNA [NM_172193]	-0.9594386	0.0053857304
A_33_P3882659	*RAB40B*	Homo sapiens RAB40B, member RAS oncogene family (RAB40B), mRNA [NM_006822]	-0.62927055	0.012887069

A_24_P921366	*CAMK1D*	calcium/calmodulin-dependent protein kinase ID [Source:HGNC Symbol;Acc:HGNC:19341] [ENST00000619168]	2.407627	0.007373151
A_24_P201879	*CALD1*	Homo sapiens caldesmon 1 (CALD1), transcript variant 1, mRNA [NM_033138]	-0.5984425	0.002178149
A_23_P359904	*GUSBP1*	Homo sapiens glucuronidase, beta pseudogene 1 (GUSBP1), transcript variant 2, non-coding RNA [NR_027027]	-0.23702538	0.008959019
A_24_P288323	*UTP14C*	Homo sapiens UTP14, U3 small nucleolar ribonucleoprotein, homolog C (yeast) (UTP14C), mRNA [NM_021645]	-0.8436243	0.010369088
A_22_P00017623	*PDDC1*	Homo sapiens Parkinson disease 7 domain containing 1 (PDDC1), mRNA [NM_182612]	-0.7090676	0.016810067
A_33_P3209866	*DPP9*	Homo sapiens dipeptidyl-peptidase 9 (DPP9), mRNA [NM_139159]	1.3348988	0.010735533
A_24_P196019	*SNHG21*	Homo sapiens small nucleolar RNA host gene 21 (non-protein coding) (SNHG21), transcript variant 5, long non-coding RNA [NR_110100]	-0.4730912	0.0029471128
A_23_P166087	*KANK1*	Homo sapiens KN motif and ankyrin repeat domains 1 (KANK1), transcript variant 4, mRNA [NM_001256877]	-0.4648016	0.015659679
A_24_P207139	*RASSF2*	Homo sapiens Ras association (RalGDS/AF-6) domain family member 2 (RASSF2), transcript variant 1, mRNA [NM_014737]	1.4774444	0.0044240346
A_21_P0011052	*PML*	Homo sapiens promyelocytic leukemia (PML), transcript variant 1, mRNA [NM_033238]	-0.5575918	0.017157309
A_21_P0008224	*RHOBTB1*	Homo sapiens Rho-related BTB domain containing 1 (RHOBTB1), transcript variant 4, mRNA [NM_001242359]	-2.1978052	0.0031941293
A_21_P0000476	*XLOC_l2_014001*	BROAD Institute lincRNA (XLOC_l2_014001), lincRNA [TCONS_l2_00026880]	0.71712935	0.013286371
A_23_P61280	*SNORA70C*	Homo sapiens small nucleolar RNA, H/ACA box 70C (SNORA70C), small nucleolar RNA [NR_003708]	0.68240434	0.013806373
A_21_P0011580	*PBK*	Homo sapiens PDZ binding kinase (PBK), transcript variant 1, mRNA [NM_018492]	-0.5084695	0.012215228
A_21_P0005985	*COPG2*	Homo sapiens coatomer protein complex, subunit gamma 2 (COPG2), transcript variant 1, mRNA [NM_012133]	-1.292142	0.0051410845
A_23_P341567	*XLOC_l2_006025*	BROAD Institute lincRNA (XLOC_l2_006025), lincRNA [TCONS_l2_00011143]	1.1644651	0.00697878
A_24_P63563	*SUGT1P1*	Homo sapiens SUGT1 pseudogene 1 (SUGT1P1), non-coding RNA [NR_003667]	-0.7291801	0.0064149695
A_33_P3408632	*SLC9B2*	Homo sapiens solute carrier family 9, subfamily B (NHA2, cation proton antiporter 2), member 2 (SLC9B2), transcript variant 1, mRNA [NM_178833]	-0.6907039	0.01572897

A_33_P6579294	*KIAA0141*	Homo sapiens KIAA0141 (KIAA0141), transcript variant 1, mRNA [NM_014773]	-1.0028634	0.016315734
A_33_P3387365	*LMF2*	Homo sapiens lipase maturation factor 2 (LMF2), mRNA [NM_033200]	1.8871166	0.004626977
A_23_P20852	*ELP2*	elongator acetyltransferase complex subunit 2 [Source:HGNC Symbol;Acc:HGNC:18248] [ENST00000540766]	3.5729885	0.004404579
A_23_P331049	*PXMP4*	Homo sapiens peroxisomal membrane protein 4, 24kDa (PXMP4), transcript variant 1, mRNA [NM_007238]	-1.1866193	0.013607847
A_32_P85999	*AUH*	Homo sapiens AU RNA binding protein/enoyl-CoA hydratase (AUH), mRNA [NM_001698]	-0.6200163	0.01102822
A_33_P3265185	*DPYSL4*	Homo sapiens dihydropyrimidinase-like 4 (DPYSL4), mRNA [NM_006426]	2.123839	0.005512448
A_24_P811704	*CDH13*	Homo sapiens cadherin 13 (CDH13), transcript variant 1, mRNA [NM_001257]	-2.4877675	0.002819256
A_24_P307580	*PPFIBP1*	Homo sapiens PTPRF interacting protein, binding protein 1 (liprin beta 1) (PPFIBP1), transcript variant 1, mRNA [NM_003622]	-1.4331387	0.008755959
A_23_P69497	*BTN2A1*	Homo sapiens butyrophilin, subfamily 2, member A1 (BTN2A1), transcript variant 4, mRNA [NM_001197234]	-0.8508459	0.007257218
A_23_P25003	*HTATIP2*	Homo sapiens HIV-1 Tat interactive protein 2, 30kDa (HTATIP2), transcript variant 5, mRNA [NM_001098523]	-0.68657476	0.013347209
A_32_P69166	*CLEC3B*	Homo sapiens C-type lectin domain family 3, member B (CLEC3B), mRNA [NM_003278]	-1.7743628	0.005695306
A_22_P00014957	*CLIP1*	Homo sapiens CAP-GLY domain containing linker protein 1 (CLIP1), transcript variant 1, mRNA [NM_002956]	-0.56814194	0.015728846
A_22_P00018544	*ANKRD42*	Homo sapiens ankyrin repeat domain 42 (ANKRD42), transcript variant 4, mRNA [NM_182603]	-1.3564005	0.0037982434
A_23_P157715	*lnc-SLTM-3*	LNCipedia lincRNA (lnc-SLTM-3), lincRNA [lnc-SLTM-3:1]	-0.8667916	0.011332268
A_24_P84898	*lnc-TBC1D12-1*	RABX5_HUMAN (Q9UJ41) Rab5 GDP/GTP exchange factor (Rabaptin 5-associated exchange factor for Rab5) (Rabex-5), partial (4%) [THC2562510]	0.76573277	0.008488166
A_32_P49844	*PPP1R16A*	Homo sapiens protein phosphatase 1, regulatory subunit 16A (PPP1R16A), mRNA [NM_032902]	-0.6272235	0.011568712
A_33_P3359223	*FEN1*	Homo sapiens flap structure-specific endonuclease 1 (FEN1), mRNA [NM_004111]	0.96175337	0.0069863
A_33_P3242820	*RHOQ*	Homo sapiens ras homolog family member Q (RHOQ), mRNA [NM_012249]	-1.1076928	0.0072215945

A_32_P114284	*C9orf173*	Homo sapiens chromosome 9 open reading frame 173 (C9orf173), transcript variant 1, mRNA [NM_001256699]	0.51129603	0.0128832515
A_23_P200772	*LOC115110*	Homo sapiens uncharacterized LOC115110 (LOC115110), long non-coding RNA [NR_037844]	-1.1102452	0.009724618
A_32_P62863	*IKZF2*	Homo sapiens IKAROS family zinc finger 2 (Helios) (IKZF2), transcript variant 2, mRNA [NM_001079526]	0.8615668	0.011974697
A_24_P306469	*ZNF644*	Homo sapiens zinc finger protein 644 (ZNF644), transcript variant 1, mRNA [NM_201269]	-0.35323662	0.007887163
A_24_P703462	*SCHIP1*	Homo sapiens schwannomin interacting protein 1 (SCHIP1), transcript variant 1, mRNA [NM_014575]	-1.3322577	0.004659113
A_24_P576506	*lnc-OPN4-2*	Homo sapiens cDNA FLJ33309 fis, clone BNGH42004538. [AK090628]	0.8287255	0.01071198
A_33_P3286958	*KIRREL3*	Homo sapiens kin of IRRE like 3 (Drosophila) (KIRREL3), transcript variant 2, mRNA [NM_001161707]	-1.7687169	0.014260212
A_23_P421401	*SPRN*	Homo sapiens shadow of prion protein homolog (zebrafish) (SPRN), mRNA [NM_001012508]	1.508648	0.014881293
A_24_P399942	*B3GALT2*	Homo sapiens UDP-Gal:betaGlcNAc beta 1,3-galactosyltransferase, polypeptide 2 (B3GALT2), mRNA [NM_003783]	-3.283422	0.012327818
A_33_P3295870	*PDGFRB*	Homo sapiens platelet-derived growth factor receptor, beta polypeptide (PDGFRB), mRNA [NM_002609]	-1.0686977	0.0083699105
A_33_P3234641	*ATP11C*	Homo sapiens ATPase, class VI, type 11C (ATP11C), transcript variant 1, mRNA [NM_173694]	-0.6905815	0.007049496
A_33_P3319155	*FAM166A*	Homo sapiens family with sequence similarity 166, member A (FAM166A), mRNA [NM_001001710]	0.83426625	0.014118111
A_23_P74229	*ACP1*	Homo sapiens acid phosphatase 1, soluble (ACP1), transcript variant 4, mRNA [NM_001040649]	-0.73615074	0.007549738
A_23_P361569	*TMEM242*	Homo sapiens transmembrane protein 242 (TMEM242), mRNA [NM_018452]	-1.0556167	0.011082943
A_21_P0011714	*STK40*	Homo sapiens serine/threonine kinase 40 (STK40), transcript variant 3, mRNA [NM_032017]	-0.8647921	0.009588725
A_21_P0005891	*SLC35F5*	Homo sapiens solute carrier family 35, member F5 (SLC35F5), transcript variant 1, mRNA [NM_025181]	-0.76874584	0.0032869747
A_21_P0008304	*LILRA6*	Homo sapiens leukocyte immunoglobulin-like receptor, subfamily A (with TM domain),	0.57740724	0.011910412

		member 6 (LILRA6), transcript variant 1, mRNA [NM_024318]		
A_33_P3415859	*lnc-EXT1-3*	LNCipedia lincRNA (lnc-EXT1-3), lincRNA [lnc-EXT1-3:1]	-16059433	0.0066500553
A_33_P3292602	*lnc-FOXG1-6*	LNCipedia lincRNA (lnc-FOXG1-6), lincRNA [lnc-FOXG1-6:7]	0.86736	0.0057247286
A_32_P87568	*NLRC3*	Homo sapiens mRNA for FLJ00398 protein. [AK090476]	0.91808933	0.0044240346
A_23_P45324	*ENAH*	Homo sapiens enabled homolog (Drosophila) (ENAH), transcript variant 1, mRNA [NM_001008493]	-0.76371574	0.011566584
A_23_P313389	*ST13*	Homo sapiens suppression of tumorigenicity 13 (colon carcinoma) (Hsp70 interacting protein) (ST13), transcript variant 1, mRNA [NM_003932]	-0.5506578	0.011030013
A_32_P435367	*TMEM35*	Homo sapiens transmembrane protein 35 (TMEM35), mRNA [NM_021637]	-0.30546397	0.017047122
A_24_P56240	*UGCG*	Homo sapiens UDP-glucose ceramide glucosyltransferase (UGCG), mRNA [NM_003358]	-0.99535465	0.009010301
A_23_P300076	*PSMD4*	Homo sapiens proteasome (prosome, macropain) 26S subunit, non-ATPase, 4 (PSMD4), mRNA [NM_002810]	-0.5866673	0.00511448
A_33_P3411097	*CPNE8*	Homo sapiens copine VIII (CPNE8), mRNA [NM_153634]	-2.773685	0.0025001846
A_23_P93009	*IQCG*	Homo sapiens IQ motif containing G (IQCG), transcript variant 1, mRNA [NM_032263]	-1.0936103	0.0044240346
A_32_P192615	*GRID2IP*	Homo sapiens glutamate receptor, ionotropic, delta 2 (Grid2) interacting protein (GRID2IP), mRNA [NM_001145118]	0.8331549	0.016991401
A_33_P3281333	*SRP19*	Homo sapiens signal recognition particle 19kDa (SRP19), transcript variant 6, mRNA [NM_001204199]	-0.70825845	0.016789315
A_23_P89073	*TAF1*	Homo sapiens TAF1 RNA polymerase II, TATA box binding protein (TBP)-associated factor, 250kDa (TAF1), transcript variant 3, mRNA [NM_001286074]	-0.9119157	0.004370471
A_22_P00007948	*SNTB2*	Homo sapiens syntrophin, beta 2 (dystrophin-associated protein A1, 59kDa, basic component 2) (SNTB2), mRNA [NM_006750]	-0.645497	0.014526816
A_23_P43276	*ZNF23*	Homo sapiens zinc finger protein 23 (ZNF23), mRNA [NM_145911]	-0.86455476	0.005255513
A_21_P0005034	*lnc-IBTK-2*	LNCipedia lincRNA (lnc-IBTK-2), lincRNA [lnc-IBTK-2:1]	-1.2696915	0.016824888
A_24_P268893	*GPR124*	Homo sapiens G protein-coupled receptor 124 (GPR124), mRNA [NM_032777]	-0.9350326	0.0048260237
A_23_P171359	*lnc-HTR1B-2*	LNCipedia lincRNA (lnc-HTR1B-2), lincRNA [lnc-HTR1B-2:1]	0.7133821	0.010937513

A_23_P126716	*THAP6*	Homo sapiens THAP domain containing 6 (THAP6), mRNA [NM_144721]	-0.7284796	0.0066818837
A_24_P185986	*WDR44*	Homo sapiens WD repeat domain 44 (WDR44), transcript variant 1, mRNA [NM_019045]	-0.9328127	0.003365343
A_23_P201596	*ATPIF1*	Homo sapiens ATPase inhibitory factor 1 (ATPIF1), transcript variant 3, mRNA [NM_178191]	-0.80253834	0.005365543
A_22_P00005122	*UPRT*	Homo sapiens uracil phosphoribosyltransferase (FUR1) homolog (S. cerevisiae) (UPRT), transcript variant 1, mRNA [NM_145052]	-0.57262725	0.0057647754
A_23_P381261	*AMPD2*	Homo sapiens adenosine monophosphate deaminase 2 (AMPD2), transcript variant 1, mRNA [NM_004037]	0.8595993	0.014086517
A_23_P395911	*lnc-DHX15-1*	PREDICTED: Homo sapiens uncharacterized LOC729175 (LOC729175), mRNA [XM_001129558]	1.2026266	0.003956951
A_23_P202501	*ADCY4*	Homo sapiens adenylate cyclase 4 (ADCY4), transcript variant 2, mRNA [NM_139247]	-1.1372075	0.009859882
A_24_P374634	*FBXO17*	F-box protein 17 [Source:HGNC Symbol;Acc:HGNC:18754] [ENST00000601394]	0.3891622	0.010495637
A_23_P19987	*RNLS*	Homo sapiens renalase, FAD-dependent amine oxidase (RNLS), transcript variant 1, mRNA [NM_001031709]	-1.1119591	0.0054723793
A_22_P00024264	*STAU2*	Homo sapiens staufen double-stranded RNA binding protein 2 (STAU2), transcript variant 1, mRNA [NM_001164380]	-0.948521	0.0047990954
A_32_P16854	*IGF2BP3*	Homo sapiens insulin-like growth factor 2 mRNA binding protein 3 (IGF2BP3), mRNA [NM_006547]	0.8250985	0.01559477
A_33_P3211818	*lnc-ADD1-1*	Homo sapiens mRNA, exon 1, 2, 3, 4, clone:RES4-24B. [AB000465]	1.0512421	0.010874326
A_33_P3326927	*ANAPC13*	Homo sapiens anaphase promoting complex subunit 13 (ANAPC13), transcript variant 1, mRNA [NM_015391]	-1.2962457	0.0042280685
A_21_P0000865	*RUNX1*	Homo sapiens runt-related transcription factor 1 (RUNX1), transcript variant 2, mRNA [NM_001001890]	1.2544047	0.0074561136
A_21_P0011184	*ZNF19*	Homo sapiens zinc finger protein 19 (ZNF19), mRNA [NM_006961]	-16793511	0.004437345
A_33_P3242965	*LINC01016*	Homo sapiens long intergenic non-protein coding RNA 1016 (LINC01016), long non-coding RNA [NR_038989]	1.1506068	0.008437608
A_24_P236799	*XLOC_l2_003992*	BROAD Institute lincRNA (XLOC_l2_003992), lincRNA [TCONS_l2_00007206]	-0.67960185	0.0077279983
A_33_P3289416	*ATAD3C*	Homo sapiens ATPase family, AAA domain containing 3C (ATAD3C), mRNA [NM_001039211]	1.5572052	0.0034502172

A_33_P3766959	*RAB31*	Homo sapiens RAB31, member RAS oncogene family (RAB31), mRNA [NM_006868]	-1.1867152	0.0075928494
A_23_P133902	*ZNF785*	Homo sapiens zinc finger protein 785 (ZNF785), mRNA [NM_152458]	-0.5382349	0.011974697
A_33_P3381751	*TDP1*	Homo sapiens tyrosyl-DNA phosphodiesterase 1 (TDP1), transcript variant 1, mRNA [NM_018319]	-0.7115875	0.0080137085
A_33_P3210059	*PSORS1C1*	Homo sapiens psoriasis susceptibility 1 candidate 1 (PSORS1C1), mRNA [NM_014068]	-2.088656	0.010398194
A_23_P115036	*TIAM1*	Homo sapiens T-cell lymphoma invasion and metastasis 1 (TIAM1), mRNA [NM_003253]	-5.585654	0.0020435157
A_23_P204801	*SF3A1*	Homo sapiens splicing factor 3a, subunit 1, 120kDa (SF3A1), mRNA [NM_005877]	-0.40903997	0.014738179
A_33_P3263170	*IPO13*	Homo sapiens importin 13 (IPO13), mRNA [NM_014652]	1.0832379	0.0045487867
A_23_P204550	*SLC41A2*	Homo sapiens solute carrier family 41 (magnesium transporter), member 2 (SLC41A2), mRNA [NM_032148]	-0.8686426	0.008288629
A_23_P254888	*SNRPG*	Homo sapiens small nuclear ribonucleoprotein polypeptide G (SNRPG), mRNA [NM_003096]	-0.31214026	0.01211404
A_33_P3268892	*SCYL2*	Homo sapiens SCY1-like 2 (S. cerevisiae) (SCYL2), mRNA [NM_017988]	-0.8310509	0.010084194
A_24_P85942	*ZYX*	Homo sapiens zyxin (ZYX), transcript variant 1, mRNA [NM_003461]	0.9500439	0.011732056
A_22_P00015395	*GDNF*	Homo sapiens glial cell derived neurotrophic factor (GDNF), transcript variant 3, mRNA [NM_001190468]	0.6185289	0.008870552
A_21_P0014492	*GCC2*	Homo sapiens GRIP and coiled-coil domain containing 2 (GCC2), transcript variant 1, mRNA [NM_181453]	-1.1215332	0.005470758
A_23_P13772	*PHF3*	Homo sapiens PHD finger protein 3 (PHF3), transcript variant 1, mRNA [NM_015153]	-0.5072489	0.011171237
A_23_P58819	*TXNIP*	Homo sapiens thioredoxin interacting protein (TXNIP), mRNA [NM_006472]	-2.0685234	0.0055012475
A_23_P25605	*TBX3*	Homo sapiens T-box 3 (TBX3), transcript variant 2, mRNA [NM_016569]	2.6791334	0.008208044
A_23_P501831	*RANBP17*	RAN binding protein 17 [Source:HGNC Symbol;Acc:HGNC:14428] [ENST00000521759]	1.2519617	0.0057496065
A_21_P0003242	*KBTBD7*	Homo sapiens kelch repeat and BTB (POZ) domain containing 7 (KBTBD7), mRNA [NM_032138]	-1.0041372	0.0057506533
A_23_P14302	*FAXDC2*	Homo sapiens fatty acid hydroxylase domain containing 2 (FAXDC2), mRNA [NM_032385]	-2.017581	0.0034766807
A_23_P47116	*lnc-EPHA6-1*	LNCipedia lincRNA (lnc-EPHA6-1), lincRNA [lnc-EPHA6-1:1]	1.7014905	0.010220027
A_23_P211561	*LINC00341*	Homo sapiens long intergenic non-protein coding RNA 341 (LINC00341), long non-coding RNA [NR_026779]	-1.0032125	0.012873304

A_23_P92730	*RASSF7*	Homo sapiens Ras association (RalGDS/AF-6) domain family (N-terminal) member 7 (RASSF7), transcript variant 1, mRNA [NM_003475]	1.3459072	0.007629789
A_23_P116614	*MEI1*	Homo sapiens meiosis inhibitor 1 (MEI1), mRNA [NM_152513]	-2.5868287	0.0025001846
A_24_P194934	*HSPB3*	Homo sapiens heat shock 27kDa protein 3 (HSPB3), mRNA [NM_006308]	-3.8126626	0.002804883
A_33_P3333158	*ME3*	Homo sapiens malic enzyme 3, NADP(+)-dependent, mitochondrial (ME3), transcript variant 2, mRNA [NM_001014811]	-1.0674714	0.011087365
A_23_P208293	*CPXM2*	Homo sapiens carboxypeptidase X (M14 family), member 2 (CPXM2), mRNA [NM_198148]	1.2643664	0.008853142
A_21_P0014380	*ANXA4*	Homo sapiens annexin A4 (ANXA4), mRNA [NM_001153]	-1.3364632	0.0037856582
A_24_P820037	*PVRL2*	Homo sapiens poliovirus receptor-related 2 (herpesvirus entry mediator B) (PVRL2), transcript variant delta, mRNA [NM_001042724]	-1.356638	0.004055852
A_23_P395595	*SLC6A17*	Homo sapiens solute carrier family 6 (neutral amino acid transporter), member 17 (SLC6A17), mRNA [NM_001010898]	1.3433356	0.008216877
A_24_P251962	*MRPS35*	Homo sapiens mitochondrial ribosomal protein S35 (MRPS35), transcript variant 1, mRNA [NM_021821]	-0.6420984	0.0060537914
A_33_P3226070	*FNBP4*	Homo sapiens formin binding protein 4 (FNBP4), mRNA [NM_015308]	-0.9742708	0.0050261226
A_23_P146765	*PCDHB2*	Homo sapiens protocadherin beta 2 (PCDHB2), mRNA [NM_018936]	1.3217472	0.009576729
A_33_P3230269	*lnc-CRIPAK-1*	LNCipedia lincRNA (lnc-CRIPAK-1), lincRNA [lnc-CRIPAK-1:1]	0.43940875	0.016888233
A_23_P374844	*RNF139*	Homo sapiens ring finger protein 139 (RNF139), mRNA [NM_007218]	-0.6487296	0.013997362
A_33_P3385750	*GRHL1*	Homo sapiens grainyhead-like 1 (Drosophila) (GRHL1), mRNA [NM_198182]	-2.7480524	0.0050253686
A_24_P212819	*GAL*	Homo sapiens galanin/GMAP prepropeptide (GAL), mRNA [NM_015973]	-1.7658447	0.005808847
A_22_P00002037	*ZNF793*	Homo sapiens zinc finger protein 793 (ZNF793), mRNA [NM_001013659]	-1.4689873	0.004804688
A_23_P358470	*HAR1B*	Homo sapiens highly accelerated region 1B (non-protein coding) (HAR1B), long non-coding RNA [NR_003245]	0.32151556	0.0071345614
A_23_P309850	*DIAPH1*	Homo sapiens diaphanous-related formin 1 (DIAPH1), transcript variant 1, mRNA [NM_005219]	-0.80200243	0.008285209

A_23_P132294	*PRIMPOL*	Homo sapiens primase and polymerase (DNA-directed) (PRIMPOL), transcript variant 1, mRNA [NM_152683]	-0.90369457	0.0057247286
A_23_P73589	*RPUSD2*	Homo sapiens RNA pseudouridylate synthase domain containing 2 (RPUSD2), transcript variant 1, mRNA [NM_152260]	-0.6922137	0.014970456
A_21_P0012473	*GGA1*	Homo sapiens golgi-associated, gamma adaptin ear containing, ARF binding protein 1 (GGA1), transcript variant 2, mRNA [NM_001001560]	-1.0150728	0.010212298
A_33_P3378430	*MSN*	Homo sapiens moesin (MSN), mRNA [NM_002444]	-0.48092464	0.006603455
A_32_P157965	*VEZF1*	Homo sapiens vascular endothelial zinc finger 1 (VEZF1), mRNA [NM_007146]	0.5549361	0.015843209
A_33_P3264875	*NOM1*	Homo sapiens nucleolar protein with MIF4G domain 1 (NOM1), mRNA [NM_138400]	-0.8532193	0.0064680316
A_23_P50591	*EIF2S2*	Homo sapiens eukaryotic translation initiation factor 2, subunit 2 beta, 38kDa (EIF2S2), mRNA [NM_003908]	-1.1019142	0.003538446
A_23_P105625	*GRK6*	Homo sapiens G protein-coupled receptor kinase 6 (GRK6), transcript variant 3, mRNA [NM_001004105]	0.9246118	0.016815627
A_24_P366526	*KCNK6*	Homo sapiens potassium channel, two pore domain subfamily K, member 6 (KCNK6), mRNA [NM_004823]	-2.9342153	0.004220053
A_33_P3298251	*COQ5*	Homo sapiens coenzyme Q5 homolog, methyltransferase (S. cerevisiae) (COQ5), mRNA [NM_032314]	-1.1233573	0.0118191205
A_33_P3403773	*SYNGR2*	Homo sapiens synaptogyrin 2, mRNA (cDNA clone MGC:102914 IMAGE:4746277), complete cds. [BC105992]	0.52661824	0.0086922655
A_33_P3415440	*ZNF569*	Homo sapiens zinc finger protein 569 (ZNF569), mRNA [NM_152484]	-0.8504815	0.008471989
A_23_P135769	*MAP3K2*	Homo sapiens mitogen-activated protein kinase kinase kinase 2 (MAP3K2), mRNA [NM_006609]	-0.7838394	0.0076440917
A_22_P00000562	*GPATCH8*	Homo sapiens G patch domain containing 8 (GPATCH8), transcript variant 1, mRNA [NM_001002909]	-0.53070587	0.013353969
A_23_P216429	*ACTB*	Homo sapiens actin, beta (ACTB), mRNA [NM_001101]	2.22869	0.007897626
A_23_P149206	*lnc-AC127496.3-6*	DKFZp686P2320_r1 686 (synonym: hlcc3) Homo sapiens cDNA clone DKFZp686P2320 5', mRNA sequence [AL603067]	0.85623926	0.005640775
A_21_P0008403	*ASPN*	Homo sapiens asporin (ASPN), transcript variant 1, mRNA [NM_017680]	-1.4226729	0.0036517929
A_33_P3230528	*B4GALT2*	Homo sapiens UDP-Gal:betaGlcNAc beta 1,4-galactosyltransferase, polypeptide 2	-0.53662914	0.012020139

		(B4GALT2), transcript variant 2, mRNA [NM_003780]		
A_23_P154037	*lnc-C14orf79-2*	LNCipedia lincRNA (lnc-C14orf79-2), lincRNA [lnc-C14orf79-2:4]	-0.85694265	0.0057806885
A_21_P0000298	*MPRIP*	myosin phosphatase Rho interacting protein [Source:HGNC Symbol;Acc:HGNC:30321] [ENST00000395807]	-10269121	0.0074182227
A_24_P801079	*AOX1*	Homo sapiens aldehyde oxidase 1 (AOX1), mRNA [NM_001159]	-0.8517854	0.012514508
A_24_P88800	*SNORD8*	Homo sapiens small nucleolar RNA, C/D box 8 (SNORD8), small nucleolar RNA [NR_002916]	-0.7259624	0.0140423635
A_33_P3404032	*COX7A2L*	Homo sapiens cytochrome c oxidase subunit VIIa polypeptide 2 like (COX7A2L), mRNA [NM_004718]	-0.6992943	0.005377644
A_33_P3368139	*RWDD3*	Homo sapiens RWD domain containing 3 (RWDD3), transcript variant 1, mRNA [NM_015485]	-1.1786953	0.0028149292
A_33_P3306545	*HIST3H2A*	Homo sapiens histone cluster 3, H2a (HIST3H2A), mRNA [NM_033445]	1.141183	0.0088629555
A_32_P18547	*MAP3K1*	Homo sapiens mitogen-activated protein kinase kinase kinase 1, E3 ubiquitin protein ligase (MAP3K1), mRNA [NM_005921]	-1.0008105	0.004288771
A_33_P3371055	*EIF4G2*	Homo sapiens eukaryotic translation initiation factor 4 gamma, 2 (EIF4G2), transcript variant 3, mRNA [NM_001172705]	-0.10683255	0.013071273
A_33_P3244361	*YBEY*	Homo sapiens ybeY metallopeptidase (putative) (YBEY), transcript variant 1, mRNA [NM_058181]	-0.6366174	0.010404932
A_24_P329635	*RAB1A*	Homo sapiens RAB1A, member RAS oncogene family (RAB1A), transcript variant 1, mRNA [NM_004161]	-0.56185985	0.0046155252
A_23_P321452	*TSC1*	Homo sapiens tuberous sclerosis 1 (TSC1), transcript variant 1, mRNA [NM_000368]	-0.9297581	0.013152009
A_21_P0010912	*LOC284930*	Homo sapiens uncharacterized LOC284930 (LOC284930), long non-coding RNA [NR_122046]	0.7897179	0.015505246
A_32_P78491	*PDZD8*	Homo sapiens PDZ domain containing 8 (PDZD8), mRNA [NM_173791]	-0.6314194	0.004701671
A_22_P00014477	*XLOC_l2_002176*	BROAD Institute lincRNA (XLOC_l2_002176), lincRNA [TCONS_l2_00003853]	0.5129955	0.014732352
A_23_P205611	*ETV1*	Homo sapiens ets variant 1 (ETV1), transcript variant 1, mRNA [NM_004956]	0.85162216	0.010237655
A_21_P0004244	*lnc-SHARPIN-2*	Homo sapiens cDNA clone IMAGE:4392840. [BC023010]	-0.7312437	0.00975317
A_23_P6381	*GMFB*	Homo sapiens glia maturation factor, beta (GMFB), mRNA [NM_004124]	-0.8367527	0.0039728633
A_21_P0003219	*lnc-CCDC152-1*	LNCipedia lincRNA (lnc-CCDC152-1), lincRNA [lnc-CCDC152-1:1]	-0.39274925	0.014345706

A_23_P340188	*MN1*	Homo sapiens meningioma (disrupted in balanced translocation) 1 (MN1), mRNA [NM_002430]	-0.7970161	0.015349476
A_24_P100551	*lnc-OXNAD1-1*	LNCipedia lincRNA (lnc-OXNAD1-1), lincRNA [lnc-OXNAD1-1:2]	0.48670357	0.00602263
A_23_P70571	*FAM122C*	Homo sapiens family with sequence similarity 122C (FAM122C), transcript variant 2, mRNA [NM_138819]	-1.1727569	0.0059724282
A_33_P3340990	*SH3RF1*	Homo sapiens SH3 domain containing ring finger 1 (SH3RF1), mRNA [NM_020870]	-1.6366465	0.003162355
A_23_P373054	*SLC39A7*	Homo sapiens solute carrier family 39 (zinc transporter), member 7 (SLC39A7), transcript variant 1, mRNA [NM_006979]	-0.5761328	0.00842156
A_24_P10214	*AKTIP*	Homo sapiens AKT interacting protein (AKTIP), transcript variant 1, mRNA [NM_001012398]	-1.299134	0.0030924478
A_33_P3318288	*TCAIM*	Homo sapiens T cell activation inhibitor, mitochondrial (TCAIM), transcript variant 1, mRNA [NM_173826]	-0.60203093	0.009276546
A_21_P0014222	*STXBP6*	Homo sapiens syntaxin binding protein 6 (amisyn) (STXBP6), mRNA [NM_014178]	-2.232245	0.0050497777
A_23_P424080	*CFH*	Homo sapiens complement factor H (CFH), transcript variant 2, mRNA [NM_001014975]	1.5298438	0.0067319144
A_33_P3252939	*LOC100505501*	PREDICTED: Homo sapiens uncharacterized LOC100505501 (LOC100505501), misc_RNA [XR_171725]	-1.1606123	0.0037353234
A_32_P117170	*YIPF4*	Homo sapiens Yip1 domain family, member 4 (YIPF4), mRNA [NM_032312]	-0.8906307	0.0031941293
A_21_P0001655	*MYLK4*	Homo sapiens myosin light chain kinase family, member 4 (MYLK4), mRNA [NM_001012418]	-0.75837165	0.009398277
A_33_P3298942	*NAPEPLD*	Homo sapiens N-acyl phosphatidylethanolamine phospholipase D (NAPEPLD), transcript variant 1, mRNA [NM_001122838]	-1.1100471	0.005014313
A_32_P197561	*lnc-AL669831.1-4*	LNCipedia lincRNA (lnc-AL669831.1-4), lincRNA [lnc-AL669831.1-4:1]	1.4434737	0.015963586
A_23_P28485	*ATP10B*	ATPase, class V, type 10B [Source:HGNC Symbol;Acc:HGNC:13543] [ENST00000326831]	1.5406686	0.0057496065
A_33_P3390357	*EBF1*	Homo sapiens early B-cell factor 1 (EBF1), transcript variant 1, mRNA [NM_001290360]	-3.1727266	0.005449271
A_24_P114255	*GCA*	Homo sapiens grancalcin, EF-hand calcium binding protein (GCA), mRNA [NM_012198]	-2.467953	0.0039633843
A_23_P53217	*SPIRE1*	Homo sapiens spire-type actin nucleation factor 1 (SPIRE1), transcript variant 1, mRNA [NM_001128626]	-0.71963835	0.014008534
A_23_P63897	*MBOAT2*	Homo sapiens membrane bound O-acyltransferase domain containing 2 (MBOAT2), mRNA [NM_138799]	-0.5299738	0.015883092

A_33_P3315600	*EED*	Homo sapiens embryonic ectoderm development (EED), transcript variant 2, mRNA [NM_152991]	-0.65655166	0.011701991
A_24_P341504	*CTBP2*	Homo sapiens C-terminal binding protein 2 (CTBP2), transcript variant 2, mRNA [NM_022802]	-0.7670913	0.012215175
A_33_P3305458	*YY1*	Homo sapiens YY1 transcription factor (YY1), mRNA [NM_003403]	-0.81960845	0.008976597
A_24_P276490	*RNPC3*	Homo sapiens RNA-binding region (RNP1, RRM) containing 3 (RNPC3), mRNA [NM_017619]	-0.8158212	0.008045824
A_23_P201731	*ATF6B*	Homo sapiens activating transcription factor 6 beta (ATF6B), transcript variant 1, mRNA [NM_004381]	-1.181088	0.005215599
A_23_P250571	*LYPLA2*	Homo sapiens lysophospholipase II (LYPLA2), mRNA [NM_007260]	1.3295107	0.0034117412
A_21_P0013022	*TRAF5*	Homo sapiens TNF receptor-associated factor 5 (TRAF5), transcript variant 1, mRNA [NM_004619]	-1.9125314	0.0044240346
A_23_P122615	*DMXL1*	Homo sapiens Dmx-like 1 (DMXL1), transcript variant 2, mRNA [NM_005509]	-0.8279237	0.008346524
A_33_P3325467	*LOC101929309*	PREDICTED: Homo sapiens uncharacterized LOC101929309 (LOC101929309), transcript variant X2, ncRNA [XR_242007]	1.3372904	0.01232594
A_23_P5742	*PNISR*	Homo sapiens PNN-interacting serine/arginine-rich protein (PNISR), transcript variant 1, mRNA [NM_032870]	-0.7227886	0.009545733
A_33_P3403549	*SMIM12*	Homo sapiens small integral membrane protein 12 (SMIM12), transcript variant 1, mRNA [NM_001164824]	-0.63054276	0.0075530736
A_23_P65217	*CCDC121*	Homo sapiens coiled-coil domain containing 121 (CCDC121), transcript variant 3, mRNA [NM_024584]	-0.66211015	0.010091486
A_24_P96403	*CNEP1R1*	Homo sapiens CTD nuclear envelope phosphatase 1 regulatory subunit 1 (CNEP1R1), transcript variant 1, mRNA [NM_153261]	-0.6422702	0.008236777
A_22_P00004408	*TGDS*	Homo sapiens TDP-glucose 4,6-dehydratase (TGDS), mRNA [NM_014305]	-0.96618503	0.005039057
A_23_P72117	*RUNX1*	Homo sapiens runt-related transcription factor 1 (RUNX1), transcript variant 2, mRNA [NM_001001890]	-0.7664473	0.016087124
A_23_P205098	*LMO7-AS1*	Homo sapiens LMO7 antisense RNA 1 (LMO7-AS1), transcript variant 3, long non-coding RNA [NR_120412]	-1.3014807	0.011079362
A_21_P0000740	*SMPDL3A*	Homo sapiens sphingomyelin phosphodiesterase, acid-like 3A (SMPDL3A), transcript variant 1, mRNA [NM_006714]	-0.8999772	0.008265211

A_33_P3370364	*PDS5B*	Homo sapiens PDS5 cohesin associated factor B (PDS5B), mRNA [NM_015032]	-0.68393886	0.015262972
A_33_P3364741	*HIVEP3*	Homo sapiens human immunodeficiency virus type I enhancer binding protein 3 (HIVEP3), transcript variant 3, non-coding RNA [NR_038260]	-1.4992429	0.0076440917
A_23_P82950	*PRLHR*	Homo sapiens prolactin releasing hormone receptor (PRLHR), mRNA [NM_004248]	0.76565707	0.010839213
A_33_P3323742	*MRC2*	Homo sapiens mannose receptor, C type 2 (MRC2), mRNA [NM_006039]	-0.42421126	0.015385319
A_23_P259692	*PCM1*	Homo sapiens pericentriolar material 1 (PCM1), mRNA [NM_006197]	-0.6376105	0.014118111
A_33_P3316379	*PNRC2*	Homo sapiens proline-rich nuclear receptor coactivator 2 (PNRC2), mRNA [NM_017761]	-1.0497587	0.013368177
A_23_P97623	*PSAT1*	Homo sapiens phosphoserine aminotransferase 1 (PSAT1), transcript variant 1, mRNA [NM_058179]	-0.76168936	0.013681735
A_23_P90911	*RRAGC*	Homo sapiens Ras-related GTP binding C (RRAGC), transcript variant 1, mRNA [NM_022157]	-1.0000048	0.0048260237
A_33_P3418120	*ABCA11P*	Homo sapiens ATP-binding cassette, sub-family A (ABC1), member 11, pseudogene (ABCA11P), non-coding RNA [NR_002451]	-0.84580004	0.009626979
A_24_P296254	*RDH14*	Homo sapiens retinol dehydrogenase 14 (all-trans/9-cis/11-cis) (RDH14), mRNA [NM_020905]	-0.59909606	0.0130220065
A_23_P308603	*IPPK*	Homo sapiens inositol 1,3,4,5,6-pentakisphosphate 2-kinase (IPPK), mRNA [NM_022755]	-0.58692324	0.009355827
A_23_P201951	*ARHGAP11A*	Homo sapiens Rho GTPase activating protein 11A (ARHGAP11A), transcript variant 1, mRNA [NM_014783]	-0.45664066	0.0091418745
A_24_P55295	*SRC*	Homo sapiens SRC proto-oncogene, non-receptor tyrosine kinase (SRC), transcript variant 1, mRNA [NM_005417]	-0.9821357	0.012940846
A_23_P19663	*ARID4B*	Homo sapiens AT rich interactive domain 4B (RBP1-like) (ARID4B), transcript variant 1, mRNA [NM_016374]	-0.7693014	0.0045212996
A_24_P107941	*GJA1*	Homo sapiens gap junction protein, alpha 1, 43kDa (GJA1), mRNA [NM_000165]	-0.9789965	0.0031827379
A_23_P334798	*CTGF*	Homo sapiens connective tissue growth factor (CTGF), mRNA [NM_001901]	-1.5008304	0.002819256
A_23_P39110	*USB1*	Homo sapiens U6 snRNA biogenesis 1 (USB1), transcript variant 1, mRNA [NM_024598]	-0.86745316	0.00477571
A_33_P3347343	*LRRC2*	Homo sapiens leucine rich repeat containing 2 (LRRC2), mRNA [NM_024512]	-2.5537784	0.003162355
A_33_P7354526	*RUVBL2*	Homo sapiens RuvB-like AAA ATPase 2 (RUVBL2), mRNA [NM_006666]	0.78783584	0.01204538

A_24_P118231	*CCDC102B*	Homo sapiens coiled-coil domain containing 102B (CCDC102B), transcript variant 1, mRNA [NM_001093729]	3.1419892	0.016463466
A_23_P356526	*SMAD2*	SMAD family member 2 [Source:HGNC Symbol;Acc:HGNC:6768] [ENST00000262160]	-0.7149491	0.010153778
A_23_P391778	*ATP5S*	Homo sapiens ATP synthase, H+ transporting, mitochondrial Fo complex, subunit s (factor B) (ATP5S), transcript variant 1, mRNA [NM_001003803]	-0.9742608	0.004149754
A_22_P00024535	*TRIM5*	Homo sapiens tripartite motif containing 5 (TRIM5), transcript variant gamma, mRNA [NM_033092]	-0.79763883	0.013805346
A_33_P3237150	*MACF1*	Homo sapiens microtubule-actin crosslinking factor 1 (MACF1), mRNA [NM_012090]	-0.49929738	0.010073093
A_33_P3273969	*BMP2*	Homo sapiens bone morphogenetic protein 2 (BMP2), mRNA [NM_001200]	2.5514553	0.008274833
A_32_P92505	*NAA38*	Homo sapiens N(alpha)-acetyltransferase 38, NatC auxiliary subunit (NAA38), mRNA [NM_032356]	-0.67837757	0.007868093
A_23_P48339	*ZC3H11A*	Homo sapiens zinc finger CCCH-type containing 11A (ZC3H11A), mRNA [NM_014827]	-0.8850801	0.0141507
A_33_P3277198	*LCLAT1*	Homo sapiens lysocardiolipin acyltransferase 1 (LCLAT1), transcript variant 1, mRNA [NM_182551]	-1.222389	0.003945209
A_23_P66694	*IFT88*	Homo sapiens intraflagellar transport 88 (IFT88), transcript variant 1, mRNA [NM_175605]	-0.86619884	0.006485141
A_22_P00024462	*CHST10*	Homo sapiens carbohydrate sulfotransferase 10 (CHST10), mRNA [NM_004854]	0.9698234	0.0086922655
A_24_P305938	*EVI2B*	Homo sapiens ecotropic viral integration site 2B (EVI2B), mRNA [NM_006495]	1.1585459	0.010612072
A_33_P3392077	*RARA-AS1*	Homo sapiens RARA antisense RNA 1 (RARA-AS1), long non-coding RNA [NR_110861]	-1.6834135	0.0024890031
A_23_P106016	*RAB9BP1*	Homo sapiens RAB9B, member RAS oncogene family pseudogene 1 (RAB9BP1), non-coding RNA [NR_000039]	-0.93766564	0.010974453
A_23_P423427	*TP53I3*	Homo sapiens tumor protein p53 inducible protein 3 (TP53I3), transcript variant 1, mRNA [NM_004881]	-1.3433516	0.0084265405
A_21_P0014114	*PRKD1*	Homo sapiens protein kinase D1 (PRKD1), mRNA [NM_002742]	-1.4389766	0.0045487867
A_33_P3387561	*FAM171B*	Homo sapiens family with sequence similarity 171, member B (FAM171B), mRNA [NM_177454]	1.343157	0.010804776
A_33_P3423931	*GATA2-AS1*	Homo sapiens GATA2 antisense RNA 1 (GATA2-AS1), long non-coding RNA [NR_125398]	1.1019014	0.0050253686

A_23_P99996	*OSCAR*	Homo sapiens osteoclast associated, immunoglobulin-like receptor (OSCAR), transcript variant 1, mRNA [NM_206818]	0.845907	0.0070515345
A_33_P3384322	*LCN9*	Homo sapiens lipocalin 9 (LCN9), mRNA [NM_001001676]	0.80652404	0.011496922
A_24_P265856	*MAGEL2*	Homo sapiens MAGE-like 2 (MAGEL2), mRNA [NM_019066]	1.7595162	0.015141116
A_24_P88801	*METTL22*	methyltransferase like 22 [Source:HGNC Symbol;Acc:HGNC:28368] [ENST00000381920]	-1.085862	0.014349793
A_24_P233850	*SENP7*	Homo sapiens SUMO1/sentrin specific peptidase 7 (SENP7), transcript variant 1, mRNA [NM_020654]	-1.0624187	0.004016376
A_23_P71037	*NPHP1*	Homo sapiens nephronophthisis 1 (juvenile) (NPHP1), transcript variant 1, mRNA [NM_000272]	-1.5398113	0.01039718
A_24_P134266	*SDHC*	Homo sapiens succinate dehydrogenase complex, subunit C, integral membrane protein, 15kDa (SDHC), transcript variant 1, mRNA [NM_003001]	-0.5781455	0.008425284
A_33_P3420068	*IL6*	Homo sapiens interleukin 6 (IL6), mRNA [NM_000600]	3.6483552	0.0025001846
A_33_P3661631	*BSG*	Homo sapiens basigin (Ok blood group) (BSG), transcript variant 1, mRNA [NM_001728]	1.1813843	0.0039139553
A_23_P10081	*GOLGA7*	Homo sapiens golgin A7 (GOLGA7), transcript variant 2, mRNA [NM_001002296]	-0.64739376	0.008691653
A_23_P99741	*PCBD2*	Homo sapiens pterin-4 alpha-carbinolamine dehydratase/dimerization cofactor of hepatocyte nuclear factor 1 alpha (TCF1) 2 (PCBD2), mRNA [NM_032151]	-0.49698064	0.0114562
A_32_P176550	*ARL13B*	Homo sapiens ADP-ribosylation factor-like 13B (ARL13B), transcript variant 1, mRNA [NM_182896]	-0.73920786	0.004016376
A_33_P3256095	*CDKL1*	Homo sapiens cyclin-dependent kinase-like 1 (CDC2-related kinase) (CDKL1), transcript variant 1, mRNA [NM_004196]	-1.220083	0.0073916297
A_22_P00018457	*JMY*	Homo sapiens junction mediating and regulatory protein, p53 cofactor (JMY), mRNA [NM_152405]	-0.90744406	0.005905037
A_33_P3216898	*XLOC_l2_001324*	BROAD Institute lincRNA (XLOC_l2_001324), lincRNA [TCONS_l2_00001793]	1.0173061	0.010827594
A_33_P3554318	*lnc-CCDC51-1*	LNCipedia lincRNA (lnc-CCDC51-1), lincRNA [lnc-CCDC51-1:2]	1.1315304	0.0038056036
A_24_P743802	*LOC286367*	Homo sapiens FP944 (LOC286367), long non-coding RNA [NR_024011]	-0.78028214	0.011846851
A_22_P00024629	*TRIM3*	Homo sapiens tripartite motif containing 3 (TRIM3), transcript variant 1, mRNA [NM_006458]	1.3564954	0.007677732

A_33_P3243023	*ZNF618*	Homo sapiens zinc finger protein 618 (ZNF618), mRNA [NM_133374]	-0.9081528	0.0039139553
A_23_P252145	*lnc-CDKAL1-2*	tt17e11.x1 NCI_CGAP_GC6 Homo sapiens cDNA clone IMAGE:2241068 3', mRNA sequence [AI699576]	-0.35398552	0.012795951
A_21_P0014103	*ADRA1D*	Homo sapiens adrenoceptor alpha 1D (ADRA1D), mRNA [NM_000678]	-1.6589091	0.0084265405
A_19_P00808668	*C1GALT1*	Homo sapiens core 1 synthase, glycoprotein-N-acetylgalactosamine 3-beta-galactosyltransferase 1 (C1GALT1), mRNA [NM_020156]	-0.39987203	0.011382023
A_32_P216426	*LOC101929947*	PREDICTED: Homo sapiens uncharacterized LOC101929947 (LOC101929947), transcript variant X3, ncRNA [XR_251219]	-0.9192664	0.0027519173
A_23_P26759	*WDR82*	Homo sapiens WD repeat domain 82 (WDR82), mRNA [NM_025222]	-0.81583637	0.010769289
A_21_P0013587	*HNRNPA1L2*	Homo sapiens heterogeneous nuclear ribonucleoprotein A1-like 2 (HNRNPA1L2), transcript variant 1, mRNA [NM_001011724]	-0.39376947	0.0054271906
A_24_P313334	*CANT1*	Homo sapiens calcium activated nucleotidase 1 (CANT1), transcript variant 1, mRNA [NM_138793]	1.0255436	0.007264689
A_22_P00014395	*XLOC_l2_014835*	BROAD Institute lincRNA (XLOC_l2_014835), lincRNA [TCONS_l2_00028817]	-1.5669292	0.005920916
A_32_P73217	*SERF2*	Homo sapiens small EDRK-rich factor 2 (SERF2), transcript variant 4, mRNA [NM_001199877]	-0.8809581	0.008046024
A_33_P3347622	*LOC102723346*	PREDICTED: Homo sapiens uncharacterized LOC102723346 (LOC102723346), transcript variant X1, ncRNA [XR_427907]	-0.86403024	0.0052084886
A_23_P103131	*TMEM167B*	Homo sapiens transmembrane protein 167B (TMEM167B), mRNA [NM_020141]	-1.0357641	0.0055686985
A_23_P55880	*MOB1B*	Homo sapiens MOB kinase activator 1B (MOB1B), transcript variant 3, mRNA [NM_001244767]	0.53398603	0.014644525
A_33_P3416366	*MIEF1*	Homo sapiens mitochondrial elongation factor 1 (MIEF1), mRNA [NM_019008]	-0.54479855	0.011171237
A_33_P3281191	*ZNF564*	Homo sapiens zinc finger protein 564 (ZNF564), mRNA [NM_144976]	-1.3954176	0.00942855
A_24_P38572	*CHAF1A*	Homo sapiens chromatin assembly factor 1, subunit A (p150) (CHAF1A), mRNA [NM_005483]	1.3940295	0.008427824
A_23_P44768	*NID1*	Homo sapiens nidogen 1 (NID1), mRNA [NM_002508]	-0.8574729	0.008159287
A_22_P00014378	*NOL6*	Homo sapiens nucleolar protein 6 (RNA-associated) (NOL6), transcript variant alpha, mRNA [NM_022917]	1.2634721	0.012007281

A_33_P3257297	*TBK1*	Homo sapiens TANK-binding kinase 1 (TBK1), mRNA [NM_013254]	-0.90671253	0.00180587
A_21_P0005217	*lnc-SETDB1-1*	LNCipedia lincRNA (lnc-SETDB1-1), lincRNA [lnc-SETDB1-1:2]	1.1127483	0.009938745
A_22_P00008187	*CFAP58*	Homo sapiens cilia and flagella associated protein 58 (CFAP58), mRNA [NM_001008723]	-1.2649662	0.004155979
A_33_P3274164	*lnc-IL6-3*	HTM1-598F HTM1 Homo sapiens cDNA, mRNA sequence [BE439741]	1.2367605	0.0070439377
A_23_P339818	*DOT1L*	PREDICTED: Homo sapiens DOT1-like histone H3K79 methyltransferase (DOT1L), transcript variant X2, mRNA [XM_005259660]	0.7385298	0.0032831472
A_24_P194154	*NET1*	Homo sapiens neuroepithelial cell transforming 1 (NET1), transcript variant 1, mRNA [NM_001047160]	0.26199818	0.002804883
A_23_P389692	*ARRDC4*	Homo sapiens arrestin domain containing 4 (ARRDC4), mRNA [NM_183376]	-1.1840132	0.00180587
A_33_P3261084	*UPF1*	Homo sapiens UPF1 regulator of nonsense transcripts homolog (yeast) (UPF1), transcript variant 2, mRNA [NM_002911]	-0.81910133	0.006007334
A_33_P3248749	*KIAA1328*	Homo sapiens KIAA1328 (KIAA1328), mRNA [NM_020776]	-0.8751018	0.013354078
A_24_P366535	*IDH3B*	Homo sapiens isocitrate dehydrogenase 3 (NAD+) beta (IDH3B), transcript variant 2, mRNA [NM_174855]	0.6155258	0.009856158
A_33_P3691860	*RSPH9*	radial spoke head 9 homolog (Chlamydomonas) [Source:HGNC Symbol;Acc:HGNC:21057] [ENST00000372165]	1.0784312	0.005822303
A_24_P143127	*FAM122B*	Homo sapiens family with sequence similarity 122B (FAM122B), transcript variant 2, mRNA [NM_001166599]	-0.6947086	0.006020368
A_33_P3247644	*TLDC1*	Homo sapiens TBC/LysM-associated domain containing 1 (TLDC1), mRNA [NM_020947]	-0.8578091	0.013002109
A_33_P3284463	*GRIA3*	Homo sapiens glutamate receptor, ionotropic, AMPA 3 (GRIA3), transcript variant 2, mRNA [NM_000828]	-1.8705372	0.0056123743
A_21_P0000493	*MTURN*	Homo sapiens maturin, neural progenitor differentiation regulator homolog (Xenopus) (MTURN), mRNA [NM_152793]	-1.2067142	0.003728358
A_23_P129786	*ASAH1*	Homo sapiens N-acylsphingosine amidohydrolase (acid ceramidase) 1 (ASAH1), transcript variant 1, mRNA [NM_177924]	-0.86086607	0.012237778
A_22_P00015282	*SCARNA14*	Homo sapiens small Cajal body-specific RNA 14 (SCARNA14), guide RNA [NR_004388]	-1.0889242	0.0054365364
A_23_P86330	*SREBF1*	Homo sapiens sterol regulatory element binding transcription factor 1 (SREBF1), transcript variant 1, mRNA [NM_001005291]	-1.0824234	0.005879462

A_21_P0012077	*NBPF1*	Homo sapiens neuroblastoma breakpoint family, member 1 (NBPF1), mRNA [NM_017940]	-1.1618788	0.007050734
A_23_P69877	*IER5*	Homo sapiens immediate early response 5 (IER5), mRNA [NM_016545]	-0.67245024	0.0041847117
A_24_P28295	*ZFP62*	Homo sapiens ZFP62 zinc finger protein (ZFP62), transcript variant 1, mRNA [NM_152283]	-0.95908755	0.0050508454
A_33_P3233005	*PLS3*	Homo sapiens plastin 3 (PLS3), transcript variant 1, mRNA [NM_005032]	-1.189435	0.0039856653
A_24_P229531	*RABGAP1L*	Homo sapiens RAB GTPase activating protein 1-like (RABGAP1L), transcript variant 4, mRNA [NM_001243765]	-1.01092	0.006352343
A_24_P156886	*MBTD1*	Homo sapiens mbt domain containing 1 (MBTD1), mRNA [NM_017643]	-0.62821245	0.006584295
A_33_P3217213	*NABP1*	Homo sapiens nucleic acid binding protein 1 (NABP1), transcript variant 1, mRNA [NM_001031716]	-0.69396776	0.0135373
A_23_P356565	*DDX59*	Homo sapiens DEAD (Asp-Glu-Ala-Asp) box polypeptide 59 (DDX59), mRNA [NM_001031725]	-0.8576352	0.0052045966
A_33_P3342598	*PDLIM7*	Homo sapiens PDZ and LIM domain 7 (enigma) (PDLIM7), transcript variant 1, mRNA [NM_005451]	1.4717212	0.0063397386
A_33_P3338417	*RRP8*	Homo sapiens ribosomal RNA processing 8, methyltransferase, homolog (yeast) (RRP8), mRNA [NM_015324]	-0.53420466	0.016302144
A_33_P3702281	*PRKG2*	Homo sapiens protein kinase, cGMP-dependent, type II (PRKG2), transcript variant 1, mRNA [NM_006259]	2.0986595	0.0038302224
A_21_P0000429	*WARS2*	Homo sapiens tryptophanyl tRNA synthetase 2, mitochondrial (WARS2), transcript variant 2, mRNA [NM_201263]	-0.49838468	0.011828812
A_33_P3214056	*LOC284014*	Homo sapiens cDNA FLJ38248 fis, clone FCBBF2007556. [AK095567]	-0.8993139	0.009555101
A_22_P00023356	*SNORD113-1*	Homo sapiens small nucleolar RNA, C/D box 113-1 (SNORD113-1), small nucleolar RNA [NR_003229]	1.4717102	0.0025451183
A_23_P3562	*LINC00869*	Homo sapiens long intergenic non-protein coding RNA 869 (LINC00869), transcript variant 8, long non-coding RNA [NR_111952]	-0.84856915	0.006474282
A_32_P34920	*lnc-C1orf222-2*	LNCipedia lincRNA (lnc-C1orf222-2), lincRNA [lnc-C1orf222-2:2]	1.2563593	0.00556941
A_23_P15876	*SLC7A6OS*	Homo sapiens solute carrier family 7, member 6 opposite strand (SLC7A6OS), mRNA [NM_032178]	-0.6740318	0.008488166
A_33_P3423185	*FOXD1*	Homo sapiens forkhead box D1 (FOXD1), mRNA [NM_004472]	-0.961544	0.003417016

A_24_P139208	*ALPK2*	Homo sapiens alpha-kinase 2 (ALPK2), mRNA [NM_052947]	-1.6099098	0.004659113
A_23_P311020	*LOC102725171*	PREDICTED: Homo sapiens uncharacterized LOC102725171 (LOC102725171), ncRNA [XR_432995]	0.94482124	0.009658549
A_22_P00017352	*USP25*	Homo sapiens ubiquitin specific peptidase 25 (USP25), transcript variant 3, mRNA [NM_013396]	-0.5103334	0.006586824
A_22_P00020501	*MITD1*	Homo sapiens MIT, microtubule interacting and transport, domain containing 1 (MITD1), mRNA [NM_138798]	-0.78522223	0.008427824
A_23_P114232	*GABPB1-AS1*	Homo sapiens GABPB1 antisense RNA 1 (GABPB1-AS1), long non-coding RNA [NR_024490]	1.1983994	0.0032554762
A_23_P111381	*PHKG2*	Homo sapiens phosphorylase kinase, gamma 2 (testis) (PHKG2), transcript variant 2, mRNA [NM_001172432]	1.2221195	0.01304696
A_24_P274270	*PRDX4*	Homo sapiens peroxiredoxin 4 (PRDX4), mRNA [NM_006406]	-0.82766956	0.0049917083
A_24_P108311	*ATG5*	Homo sapiens autophagy related 5 (ATG5), transcript variant 1, mRNA [NM_004849]	-0.46121863	0.012320746
A_33_P3327108	*STAT1*	Homo sapiens signal transducer and activator of transcription 1, 91kDa (STAT1), transcript variant beta, mRNA [NM_139266]	-0.78385353	0.0166364
A_23_P31903	*NEDD4L*	Homo sapiens neural precursor cell expressed, developmentally down-regulated 4-like, E3 ubiquitin protein ligase (NEDD4L), transcript variant d, mRNA [NM_015277]	-26606822	0.003365343
A_23_P86838	*NLK*	Homo sapiens nemo-like kinase (NLK), mRNA [NM_016231]	-0.7124686	0.009808879
A_22_P00023941	*VPS28*	Homo sapiens vacuolar protein sorting 28 homolog (S. cerevisiae) (VPS28), transcript variant 2, mRNA [NM_183057]	-0.75047535	0.0144334
A_23_P65699	*SLC36A4*	Homo sapiens solute carrier family 36 (proton/amino acid symporter), member 4 (SLC36A4), transcript variant 1, mRNA [NM_152313]	-0.7052026	0.011745691
A_24_P398064	*lnc-GABPA-5*	LNCipedia lincRNA (lnc-GABPA-5), lincRNA [lnc-GABPA-5:1]	-0.60072064	0.016238803
A_24_P67988	*SPG11*	Homo sapiens spastic paraplegia 11 (autosomal recessive) (SPG11), transcript variant 1, mRNA [NM_025137]	-0.9076412	0.01047417
A_21_P0006502	*RGP1*	RGP1 retrograde golgi transport homolog (S. cerevisiae) [Source:HGNC Symbol;Acc:HGNC:21965] [ENST00000378078]	-0.7409401	0.0027348737
A_33_P3229725	*FRMD8*	Homo sapiens FERM domain containing 8 (FRMD8), transcript variant 1, mRNA [NM_031904]	-0.42657188	0.013353969

A_22_P00017320	*lnc-ZC3H12B-2*	LNCipedia lincRNA (lnc-ZC3H12B-2), lincRNA [lnc-ZC3H12B-2:2]	0.7149765	0.015600941
A_33_P3880302	*ARSA*	Homo sapiens arylsulfatase A (ARSA), transcript variant 1, mRNA [NM_000487]	-1.0488288	0.0025743134
A_32_P117313	*LOC339803*	Homo sapiens uncharacterized LOC339803 (LOC339803), long non-coding RNA [NR_036496]	-0.9339578	0.0050497777
A_22_P00009237	*EPHB2*	Homo sapiens EPH receptor B2 (EPHB2), transcript variant 2, mRNA [NM_004442]	1.3569818	0.0072620907
A_23_P137896	*TRIQK*	Homo sapiens triple QxxK/R motif containing (TRIQK), transcript variant 1, mRNA [NM_001171796]	-0.9150243	0.006901566
A_23_P257503	*OXCT2*	Homo sapiens 3-oxoacid CoA transferase 2 (OXCT2), mRNA [NM_022120]	0.7901269	0.004884975
A_24_P166613	*METTL18*	Homo sapiens methyltransferase like 18 (METTL18), mRNA [NM_033418]	-0.50258297	0.016676214
A_23_P86917	*HERC1*	Homo sapiens HECT and RLD domain containing E3 ubiquitin protein ligase family member 1 (HERC1), mRNA [NM_003922]	-0.6723972	0.0066119307
A_23_P257583	*EPDR1*	Homo sapiens ependymin related 1 (EPDR1), transcript variant 1, mRNA [NM_017549]	-22839036	0.004055852
A_33_P3234989	*FADD*	Homo sapiens Fas (TNFRSF6)-associated via death domain (FADD), mRNA [NM_003824]	-0.91034126	0.011763987
A_33_P3384721	*DENND2A*	Homo sapiens DENN/MADD domain containing 2A (DENND2A), mRNA [NM_015689]	3.7494788	0.0022181747
A_32_P226646	*IFT81*	Homo sapiens intraflagellar transport 81 (IFT81), transcript variant 1, mRNA [NM_014055]	-0.8483672	0.014591436
A_23_P126197	*NDUFB8*	Homo sapiens NADH dehydrogenase (ubiquinone) 1 beta subcomplex, 8, 19kDa (NDUFB8), transcript variant 1, mRNA [NM_005004]	-0.7135339	0.016295526
A_24_P239176	*LOC100129781*	PREDICTED: Homo sapiens uncharacterized LOC100129781 (LOC100129781), transcript variant X2, ncRNA [XR_109259]	-1.5279962	0.010124778
A_33_P3341586	*SRSF4*	Homo sapiens serine/arginine-rich splicing factor 4 (SRSF4), mRNA [NM_005626]	-0.7055132	0.010149606
A_23_P14515	*MUC4*	Homo sapiens mucin 4, cell surface associated (MUC4), transcript variant 1, mRNA [NM_018406]	0.35365647	0.015446468
A_23_P95594	*SIL1*	Homo sapiens SIL1 nucleotide exchange factor (SIL1), transcript variant 1, mRNA [NM_001037633]	-1.1121925	0.012592122
A_23_P312358	*ACOT4*	Homo sapiens acyl-CoA thioesterase 4 (ACOT4), mRNA [NM_152331]	-1.7418334	0.0034766807
A_33_P3306159	*NAT1*	Homo sapiens N-acetyltransferase 1 (arylamine N-acetyltransferase) (NAT1), transcript variant 5, mRNA [NM_000662]	-0.8667095	0.010801375

A_33_P3424112	*BEND7*	Homo sapiens BEN domain containing 7 (BEND7), transcript variant 1, mRNA [NM_152751]	-0.9987937	0.010950393
A_24_P371194	*TNRC18*	Homo sapiens trinucleotide repeat containing 18 (TNRC18), mRNA [NM_001080495]	0.90328276	0.015140831
A_23_P99604	*DHX40*	Homo sapiens DEAH (Asp-Glu-Ala-His) box polypeptide 40 (DHX40), transcript variant 1, mRNA [NM_024612]	-0.53802997	0.009014158
A_22_P00016779	*MRPL53*	Homo sapiens mitochondrial ribosomal protein L53 (MRPL53), mRNA [NM_053050]	-0.682599	0.015346599
A_23_P422851	*G2E3*	Homo sapiens G2/M-phase specific E3 ubiquitin protein ligase (G2E3), mRNA [NM_017769]	-0.6737132	0.0039497027
A_21_P0014809	*lnc-TRIM41-3*	LNCipedia lincRNA (lnc-TRIM41-3), lincRNA [lnc-TRIM41-3:2]	-0.7059302	0.012507492
A_33_P3354940	*CABLES1*	Homo sapiens Cdk5 and Abl enzyme substrate 1 (CABLES1), transcript variant 1, mRNA [NM_138375]	-0.54491466	0.01047417
A_22_P00016333	*FAM136A*	Homo sapiens family with sequence similarity 136, member A (FAM136A), mRNA [NM_032822]	-0.7120983	0.014448771
A_24_P358425	*CSF1*	Homo sapiens colony stimulating factor 1 (macrophage) (CSF1), transcript variant 1, mRNA [NM_000757]	0.9144466	0.009750865
A_33_P3235053	*lnc-TMEM159-2*	BX282506 NIH_MGC_37 Homo sapiens cDNA clone IMAGp998M037681 ; IMAGE:3058346, mRNA sequence [BX282506]	1.9347531	0.011072234
A_23_P7732	*GPATCH11*	Homo sapiens G patch domain containing 11 (GPATCH11), transcript variant 1, mRNA [NM_174931]	-0.61223984	0.0074175163
A_22_P00022302	*TRIM47*	Homo sapiens tripartite motif containing 47 (TRIM47), mRNA [NM_033452]	0.8041065	0.013152762
A_33_P3401826	*CETN3*	Homo sapiens centrin, EF-hand protein, 3 (CETN3), transcript variant 2, mRNA [NM_004365]	-0.910141	0.0039426656
A_22_P00012835	*CMPK2*	Homo sapiens cytidine monophosphate (UMP-CMP) kinase 2, mitochondrial (CMPK2), transcript variant 1, mRNA [NM_207315]	-0.6511714	0.0041946988
A_24_P944640	*RPS27L*	Homo sapiens ribosomal protein S27-like (RPS27L), mRNA [NM_015920]	-1.0549138	0.0041868715
A_23_P69988	*lnc-RASA1-3*	LNCipedia lincRNA (lnc-RASA1-3), lincRNA [lnc-RASA1-3:6]	-0.84992623	0.00951162
A_24_P193295	*EPB41L5*	Homo sapiens erythrocyte membrane protein band 4.1 like 5 (EPB41L5), transcript variant 1, mRNA [NM_020909]	1.1964852	0.0066503813
A_23_P373521	*RARS*	Homo sapiens arginyl-tRNA synthetase (RARS), mRNA [NM_002887]	-0.5839417	0.007099076

A_23_P138805	*RAB15*	Homo sapiens RAB15, member RAS oncogene family (RAB15), mRNA [NM_198686]	-1.1813042	0.008385109
A_24_P358381	*HAND2*	Homo sapiens heart and neural crest derivatives expressed 2 (HAND2), mRNA [NM_021973]	2.5284357	0.0076546753
A_21_P0003595	*CHORDC1*	Homo sapiens cysteine and histidine-rich domain (CHORD) containing 1 (CHORDC1), transcript variant 1, mRNA [NM_012124]	-0.14914083	0.0062004877
A_33_P3283054	*GTPBP6*	Homo sapiens GTP binding protein 6 (putative) (GTPBP6), mRNA [NM_012227]	1.0187896	0.0037134301
A_24_P152356	*lnc-PCDH7-13*	LNCipedia lincRNA (lnc-PCDH7-13), lincRNA [lnc-PCDH7-13:1]	-0.84706163	0.0043846127
A_24_P409800	*ZIC1*	Human clone 23814 mRNA sequence. [U79264]	0.66792816	0.010060375
A_33_P3241184	*SLC35E3*	Homo sapiens solute carrier family 35, member E3 (SLC35E3), mRNA [NM_018656]	-0.66754436	0.006506821
A_24_P90216	*KCTD16*	Homo sapiens potassium channel tetramerization domain containing 16 (KCTD16), mRNA [NM_020768]	-1.6664306	0.00338883
A_33_P3402171	*LGR4*	Homo sapiens leucine-rich repeat containing G protein-coupled receptor 4 (LGR4), mRNA [NM_018490]	-3.1640954	0.0025001846
A_23_P42257	*AK2*	Homo sapiens adenylate kinase 2 (AK2), transcript variant 1, mRNA [NM_001625]	-0.6570046	0.009218792
A_23_P29575	*EIF2S1*	Homo sapiens eukaryotic translation initiation factor 2, subunit 1 alpha, 35kDa (EIF2S1), mRNA [NM_004094]	-0.62782264	0.010040388
A_22_P00009473	*IER3*	Homo sapiens immediate early response 3 (IER3), mRNA [NM_003897]	0.6677713	0.011182366
A_33_P3388948	*MRPS36*	Homo sapiens mitochondrial ribosomal protein S36 (MRPS36), mRNA [NM_033281]	-0.88397115	0.005612091
A_33_P3381338	*PSMG3-AS1*	Homo sapiens PSMG3 antisense RNA 1 (head to head) (PSMG3-AS1), transcript variant 3, long non-coding RNA [NR_021487]	0.39940023	0.014332747
A_32_P113584	*SNX30*	Homo sapiens sorting nexin family member 30 (SNX30), mRNA [NM_001012994]	-0.6624461	0.009630487
A_23_P90369	*TNXB*	Homo sapiens tenascin XB (TNXB), transcript variant XB, mRNA [NM_019105]	-1.590956	0.0066119307
A_33_P3352522	*ZNF292*	Homo sapiens zinc finger protein 292 (ZNF292), mRNA [NM_015021]	-0.59193736	0.00884821
A_23_P63219	*TMEM259*	Homo sapiens transmembrane protein 259 (TMEM259), transcript variant 1, mRNA [NM_001033026]	-0.5832998	0.011257593
A_23_P111343	*GAS6-AS1*	Homo sapiens cDNA FLJ35543 fis, clone SPLEN2002957. [AK092862]	-1.4272823	0.008427824
A_21_P0008856	*POGZ*	Homo sapiens pogo transposable element with ZNF domain (POGZ), transcript variant 1, mRNA [NM_015100]	-0.96026844	0.0061486606

A_24_P64918	*BCLAF1*	Homo sapiens BCL2-associated transcription factor 1 (BCLAF1), transcript variant 1, mRNA [NM_014739]	-0.46397132	0.013495644
A_23_P62659	*PEAK1*	pseudopodium-enriched atypical kinase 1 [Source:HGNC Symbol;Acc:HGNC:29431] [ENST00000560626]	-0.9275086	0.0035523935
A_23_P360245	*ZBTB1*	Homo sapiens zinc finger and BTB domain containing 1 (ZBTB1), transcript variant 2, mRNA [NM_014950]	-0.29499465	0.014525657
A_33_P3313796	*PPT1*	Homo sapiens palmitoyl-protein thioesterase 1 (PPT1), transcript variant 1, mRNA [NM_000310]	-0.8485703	0.0050531467
A_23_P15305	*NUDCD2*	Homo sapiens NudC domain containing 2 (NUDCD2), mRNA [NM_145266]	-0.8518488	0.0026564114
A_33_P3322553	*CCDC34*	Homo sapiens coiled-coil domain containing 34 (CCDC34), transcript variant 1, mRNA [NM_030771]	-1.1561577	0.0031941293
A_33_P3508717	*PRPSAP1*	Homo sapiens phosphoribosyl pyrophosphate synthetase-associated protein 1 (PRPSAP1), mRNA [NM_002766]	-0.9957113	0.008031949
A_24_P130962	*MED25*	Homo sapiens mediator complex subunit 25 (MED25), mRNA [NM_030973]	1.2498417	0.0068023712
A_33_P3278684	*LINC00847*	Homo sapiens long intergenic non-protein coding RNA 847 (LINC00847), transcript variant 1, long non-coding RNA [NR_027183]	-0.8365362	0.010986003
A_24_P338187	*TOR3A*	Homo sapiens torsin family 3, member A (TOR3A), mRNA [NM_022371]	0.7874544	0.0069863
A_23_P42514	*HNRNPH3*	Homo sapiens heterogeneous nuclear ribonucleoprotein H3 (2H9) (HNRNPH3), transcript variant 2H9, mRNA [NM_012207]	-0.7036755	0.0038302224
A_21_P0014833	*CDH2*	Homo sapiens cadherin 2, type 1, N-cadherin (neuronal) (CDH2), mRNA [NM_001792]	-2.3326523	0.0025001846
A_23_P210690	*C6orf62*	Homo sapiens chromosome 6 open reading frame 62 (C6orf62), mRNA [NM_030939]	-0.7474773	0.0025001846
A_21_P0007068	*LOC100507209*	Homo sapiens cDNA FLJ27418 fis, clone WMC07037. [AK130928]	-1.6256622	0.0037705272
A_23_P124164	*TRIB3*	Homo sapiens tribbles pseudokinase 3 (TRIB3), transcript variant 1, mRNA [NM_021158]	-1.4189601	0.0031941293
A_33_P3323368	*LOC101929506*	PREDICTED: Homo sapiens uncharacterized LOC101929506 (LOC101929506), transcript variant X2, ncRNA [XR_252776]	-0.71274495	0.012223227
A_23_P56673	*ISY1*	Homo sapiens ISY1 splicing factor homolog (S. cerevisiae) (ISY1), transcript variant 2, mRNA [NM_020701]	-1.0897794	0.0075276187
A_33_P3503937	*LINC00921*	Homo sapiens long intergenic non-protein coding RNA 921 (LINC00921), long non-coding RNA [NR_033904]	-1.3689154	0.0045670583

A_33_P3358648	*INO80B*	Homo sapiens INO80 complex subunit B (INO80B), mRNA [NM_031288]	0.7288461	0.0132846935
A_33_P3391796	*LOC284581*	Homo sapiens cDNA FLJ37107 fis, clone BRACE2020157. [AK094426]	-0.86494535	0.007103236
A_33_P3210585	*LRRFIP1*	Homo sapiens leucine rich repeat (in FLII) interacting protein 1 (LRRFIP1), transcript variant 3, mRNA [NM_001137552]	-0.75257224	0.012909207
A_23_P23575	*NOG*	Homo sapiens noggin (NOG), mRNA [NM_005450]	-2.6312466	0.0025001846
A_23_P200203	*AAK1*	Homo sapiens AP2 associated kinase 1 (AAK1), mRNA [NM_014911]	-0.8738324	0.0061896956
A_32_P64038	*SLC39A1*	Homo sapiens solute carrier family 39 (zinc transporter), member 1 (SLC39A1), transcript variant 1, mRNA [NM_014437]	0.6775346	0.01211404
A_33_P3320062	*ECHDC2*	Homo sapiens enoyl CoA hydratase domain containing 2 (ECHDC2), transcript variant 2, mRNA [NM_018281]	-0.94282293	0.0043705306
A_32_P160045	*LOC400590*	Homo sapiens hypothetical LOC400590, mRNA (cDNA clone MGC:70830 IMAGE:5248762), complete cds. [BC062632]	1.2271032	0.004402272
A_23_P144959	*PLD1*	Homo sapiens phospholipase D1, phosphatidylcholine-specific (PLD1), transcript variant 1, mRNA [NM_002662]	-1.0851433	0.013143453
A_32_P175539	*TCTEX1D1*	Homo sapiens Tctex1 domain containing 1 (TCTEX1D1), mRNA [NM_152665]	-0.7035275	0.008222596
A_33_P3289356	*VCAN*	Homo sapiens versican (VCAN), transcript variant 1, mRNA [NM_004385]	-1.5833315	0.0044240346
A_24_P661641	*RCN2*	Homo sapiens reticulocalbin 2, EF-hand calcium binding domain (RCN2), transcript variant 1, mRNA [NM_002902]	-1.149544	0.0070515345
A_24_P19810	*CD58*	Homo sapiens CD58 molecule (CD58), transcript variant 1, mRNA [NM_001779]	-1.6018008	0.0043705306
A_33_P3349716	*LINC00938*	Homo sapiens long intergenic non-protein coding RNA 938 (LINC00938), long non-coding RNA [NR_028408]	-0.5122675	0.010296312
A_33_P3231888	*PPCS*	Homo sapiens phosphopantothenoylcysteine synthetase (PPCS), transcript variant 1, mRNA [NM_024664]	-0.8409023	0.0071450765
A_33_P3360728	*ZNF766*	Homo sapiens zinc finger protein 766 (ZNF766), mRNA [NM_001010851]	-1.0573694	0.006506821
A_33_P3242543	*C5orf15*	Homo sapiens chromosome 5 open reading frame 15 (C5orf15), mRNA [NM_020199]	-0.91861135	0.0047990954
A_33_P3232557	*BLVRB*	Homo sapiens biliverdin reductase B (BLVRB), mRNA [NM_000713]	-0.22864418	0.014497755
A_33_P3385436	*MAOA*	Homo sapiens monoamine oxidase A (MAOA), transcript variant 2, mRNA [NM_001270458]	-3.1590536	0.0031941293

A_24_P69691	*DLGAP3*	Homo sapiens discs, large (Drosophila) homolog-associated protein 3 (DLGAP3), mRNA [NM_001080418]	0.8828386	0.013936194
A_22_P00011316	*PLAC8L1*	Homo sapiens PLAC8-like 1 (PLAC8L1), mRNA [NM_001029869]	-1.1881502	0.003392081
A_23_P3592	*ZNF25*	Homo sapiens zinc finger protein 25 (ZNF25), mRNA [NM_145011]	-0.9414954	0.010190244
A_24_P142473	*HSF4*	Homo sapiens heat shock transcription factor 4 (HSF4), transcript variant 2, mRNA [NM_001040667]	0.6262142	0.007603999
A_24_P286054	*GOLGA8R*	Homo sapiens golgin A8 family, member R (GOLGA8R), mRNA [NM_001282484]	-0.30569926	0.009479944
A_23_P134426	*CTDNEP1*	Homo sapiens CTD nuclear envelope phosphatase 1 (CTDNEP1), transcript variant 1, mRNA [NM_015343]	0.5631184	0.013529562
A_22_P00014812	*ZFYVE16*	Homo sapiens zinc finger, FYVE domain containing 16 (ZFYVE16), transcript variant 2, mRNA [NM_001105251]	-0.3974211	0.009679106
A_22_P00000693	*GPNMB*	Homo sapiens glycoprotein (transmembrane) nmb (GPNMB), transcript variant 1, mRNA [NM_001005340]	-0.92694306	0.0069260783
A_23_P346302	*lnc-ADAM21-2*	LNCipedia lincRNA (lnc-ADAM21-2), lincRNA [lnc-ADAM21-2:1]	-2.5440178	0.009939401
A_33_P3421984	*ZNRD1-AS1*	Homo sapiens ZNRD1 antisense RNA 1 (ZNRD1-AS1), long non-coding RNA [NR_026751]	-1.2868099	0.016331382
A_33_P3408877	*ZMAT2*	Homo sapiens zinc finger, matrin-type 2 (ZMAT2), mRNA [NM_144723]	-0.65547174	0.004690428
A_23_P56578	*HYAL4*	Homo sapiens hyaluronoglucosaminidase 4 (HYAL4), mRNA [NM_012269]	2.754865	0.0035097655
A_22_P00005232	*VIT*	Homo sapiens vitrin (VIT), transcript variant 1, mRNA [NM_053276]	-1.7524618	0.0075857607
A_33_P3252605	*CCDC77*	Homo sapiens coiled-coil domain containing 77 (CCDC77), transcript variant 1, mRNA [NM_032358]	-0.67927045	0.013612673
A_24_P140827	*CYP2U1*	Homo sapiens cytochrome P450, family 2, subfamily U, polypeptide 1 (CYP2U1), mRNA [NM_183075]	-0.74838114	0.008510122
A_24_P254949	*AFTPH*	Homo sapiens aftiphilin (AFTPH), transcript variant 1, mRNA [NM_203437]	-0.76241374	0.0046636313
A_33_P3281196	*PCMT1*	Homo sapiens protein-L-isoaspartate (D-aspartate) O-methyltransferase (PCMT1), transcript variant 1, mRNA [NM_005389]	-0.4655421	0.009782756
A_33_P3243929	*PGM5*	Homo sapiens phosphoglucomutase 5 (PGM5), mRNA [NM_021965]	1.8280967	0.0062610074
A_23_P138725	*MRPS36*	Homo sapiens mitochondrial ribosomal protein S36 (MRPS36), mRNA [NM_033281]	-0.73475766	0.007637267
A_32_P150391	*FLJ42022*	Homo sapiens cDNA FLJ42022 fis, clone SPLEN2034678. [AK124016]	-0.55248934	0.009282967

A_23_P402000	*MARVELD1*	Homo sapiens MARVEL domain containing 1 (MARVELD1), mRNA [NM_031484]	-0.80759525	0.0032795896
A_24_P923251	*C17orf100*	Homo sapiens chromosome 17 open reading frame 100 (C17orf100), mRNA [NM_001105520]	-1.170841	0.0060077314
A_33_P3387090	*ZNF527*	Homo sapiens zinc finger protein 527 (ZNF527), mRNA [NM_032453]	-0.83472186	0.011851147
A_33_P3374293	*TGM2*	Homo sapiens transglutaminase 2 (TGM2), transcript variant 2, mRNA [NM_198951]	1.358251	0.010801365
A_21_P0014707	*PYROXD1*	Homo sapiens pyridine nucleotide-disulphide oxidoreductase domain 1 (PYROXD1), mRNA [NM_024854]	-1.062236	0.006573993
A_24_P117942	*TMEM38B*	Homo sapiens transmembrane protein 38B (TMEM38B), mRNA [NM_018112]	-0.5238656	0.007337258
A_33_P3284557	*TOMM20L*	Homo sapiens translocase of outer mitochondrial membrane 20 homolog (yeast)-like (TOMM20L), mRNA [NM_207377]	-0.6646239	0.009222329
A_33_P3269278	*TMEM128*	Homo sapiens transmembrane protein 128 (TMEM128), transcript variant 3, mRNA [NM_032927]	-0.7013077	0.009765173
A_23_P156284	*ZAK*	Homo sapiens sterile alpha motif and leucine zipper containing kinase AZK (ZAK), transcript variant 1, mRNA [NM_016653]	-0.32988077	0.01294906
A_33_P3278906	*ZNF461*	Homo sapiens zinc finger protein 461 (ZNF461), transcript variant 1, mRNA [NM_153257]	-0.79328376	0.003422245
A_23_P103256	*DBN1*	Homo sapiens drebrin 1 (DBN1), transcript variant 2, mRNA [NM_080881]	-0.6106886	0.014279916
A_33_P3239347	*PHF20L1*	Homo sapiens PHD finger protein 20-like 1 (PHF20L1), transcript variant 3, mRNA [NM_198513]	-0.5365383	0.005057235
A_24_P256380	*CFHR3*	Homo sapiens complement factor H-related 3 (CFHR3), transcript variant 1, mRNA [NM_021023]	1.7734028	0.005236083
A_33_P3278211	*NKX3-1*	Homo sapiens NK3 homeobox 1 (NKX3-1), transcript variant 1, mRNA [NM_006167]	-10505446	0.010397241
A_23_P394605	*WLS*	Homo sapiens wntless Wnt ligand secretion mediator (WLS), transcript variant 1, mRNA [NM_024911]	2.147121	0.00180587
A_21_P0014157	*MMRN2*	PREDICTED: Homo sapiens multimerin 2 (MMRN2), transcript variant X2, mRNA [XM_006717970]	1.4148583	0.01477367
A_23_P129577	*SEC24A*	Homo sapiens SEC24 family member A (SEC24A), transcript variant 1, mRNA [NM_021982]	-0.50782466	0.012731291
A_24_P85181	*TIGD7*	Homo sapiens tigger transposable element derived 7 (TIGD7), mRNA [NM_033208]	-0.6709536	0.0041946988

A_24_P7594	*TRHDE-AS1*	Homo sapiens TRHDE antisense RNA 1 (TRHDE-AS1), transcript variant 2, long non-coding RNA [NR_026836]	1.5289828	0.0073155
A_33_P3422289	*RBSN*	Homo sapiens rabenosyn, RAB effector (RBSN), transcript variant 1, mRNA [NM_022340]	-0.92602915	0.008452045
A_23_P368691	*APOL6*	Homo sapiens apolipoprotein L, 6 (APOL6), mRNA [NM_030641]	-1.3881282	0.0134437205
A_33_P3246995	*lnc-TMED5-1*	LNCipedia lincRNA (lnc-TMED5-1), lincRNA [lnc-TMED5-1:28]	-0.5870227	0.007479662
A_23_P211126	*ABCC9*	Homo sapiens ATP-binding cassette, sub-family C (CFTR/MRP), member 9 (ABCC9), transcript variant SUR2A, mRNA [NM_005691]	0.9406811	0.006573993
A_23_P83579	*DYRK1A*	Homo sapiens dual-specificity tyrosine-(Y)-phosphorylation regulated kinase 1A (DYRK1A), transcript variant 2, mRNA [NM_130436]	-0.47299743	0.009591459
A_22_P00008499	*CDX1*	Homo sapiens caudal type homeobox 1 (CDX1), mRNA [NM_001804]	0.7633938	0.014707766
A_22_P00014083	*ARNT2*	Homo sapiens aryl-hydrocarbon receptor nuclear translocator 2 (ARNT2), mRNA [NM_014862]	-0.9568832	0.0051692408
A_24_P414719	*lnc-KCNE4-1*	Homo sapiens cDNA FLJ39762 fis, clone SPLEN1000156. [AK097081]	0.69161767	0.013432872
A_24_P410797	*lnc-SAP30-5*	LNCipedia lincRNA (lnc-SAP30-5), lincRNA [lnc-SAP30-5:33]	1.4967383	0.013327314
A_23_P214046	*NFYA*	Homo sapiens nuclear transcription factor Y, alpha (NFYA), transcript variant 1, mRNA [NM_002505]	-0.51620954	0.013341529
A_23_P71867	*KALRN*	Homo sapiens cDNA FLJ43991 fis, clone TESTI4019843, highly similar to Rattus norvegicus huntingtin-associated protein interacting protein (duo) (Hapip). [AK125979]	-2.1615753	0.0108264685
A_23_P35219	*FBXW11*	Homo sapiens F-box and WD repeat domain containing 11 (FBXW11), transcript variant 3, mRNA [NM_012300]	-0.8997555	0.007885157
A_33_P3331687	*IL11RA*	Homo sapiens interleukin 11 receptor, alpha (IL11RA), transcript variant 3, mRNA [NM_001142784]	-0.82177544	0.015980396
A_23_P148308	*NEK2*	Homo sapiens NIMA-related kinase 2 (NEK2), transcript variant 1, mRNA [NM_002497]	-0.57655376	0.008729154
A_23_P255805	*GPSM1*	Homo sapiens G-protein signaling modulator 1 (GPSM1), transcript variant 1, mRNA [NM_001145638]	1.1209327	0.0054447073
A_23_P410017	*RBM3*	Homo sapiens RNA binding motif (RNP1, RRM) protein 3 (RBM3), mRNA [NM_006743]	-0.75992745	0.003609745
A_33_P3239143	*ZNF7*	Homo sapiens zinc finger protein 7 (ZNF7), transcript variant 2, mRNA [NM_003416]	-0.72354645	0.01571288

A_23_P43504	*TBCEL*	Homo sapiens tubulin folding cofactor E-like (TBCEL), transcript variant 1, mRNA [NM_152715]	-1.1796991	0.0055054883
A_23_P64121	*ZNF497*	Homo sapiens zinc finger protein 497 (ZNF497), transcript variant 1, mRNA [NM_198458]	0.42534563	0.0041475594
A_23_P113748	*ABCA2*	Homo sapiens ATP-binding cassette, sub-family A (ABC1), member 2 (ABCA2), transcript variant 1, mRNA [NM_001606]	1.8139515	0.0057916977
A_24_P39101	*KIAA1549L*	Homo sapiens KIAA1549-like (KIAA1549L), mRNA [NM_012194]	-1.3270042	0.0044240346
A_23_P8452	*ZNF385D*	Homo sapiens zinc finger protein 385D (ZNF385D), mRNA [NM_024697]	-1.5953604	0.0037856582
A_33_P3221999	*KCTD10*	Homo sapiens potassium channel tetramerization domain containing 10 (KCTD10), mRNA [NM_031954]	-0.98862165	0.0045487867
A_23_P257905	*LFNG*	Homo sapiens LFNG O-fucosylpeptide 3-beta-N-acetylglucosaminyltransferase (LFNG), transcript variant 1, mRNA [NM_001040167]	0.44351295	0.013562597
A_23_P163942	*GSDMB*	Homo sapiens gasdermin B (GSDMB), transcript variant 3, mRNA [NM_001165958]	-1.6632166	0.008973613
A_24_P241815	*CENPB*	Homo sapiens centromere protein B, 80kDa (CENPB), mRNA [NM_001810]	1.7240882	0.004359316
A_23_P55990	*SUPT4H1*	Homo sapiens suppressor of Ty 4 homolog 1 (S. cerevisiae) (SUPT4H1), transcript variant 1, mRNA [NM_003168]	-0.7657995	0.005306498
A_23_P214474	*JUNB*	Homo sapiens jun B proto-oncogene (JUNB), mRNA [NM_002229]	2.370483	0.004160909
A_21_P0006149	*NAPA*	Homo sapiens N-ethylmaleimide-sensitive factor attachment protein, alpha (NAPA), transcript variant 1, mRNA [NM_003827]	0.91725206	0.010740848
A_23_P330836	*TOMM6*	Homo sapiens translocase of outer mitochondrial membrane 6 homolog (yeast) (TOMM6), mRNA [NM_001134493]	-0.23835284	0.012215386
A_33_P3329522	*lnc-ANKRD20A1-2*	DA714598 NT2RI2 Homo sapiens cDNA clone NT2RI2021603 5', mRNA sequence [DA714598]	-2.1986256	0.004359316
A_33_P3320493	*LINC01560*	Homo sapiens long intergenic non-protein coding RNA 1560 (LINC01560), long non-coding RNA [NR_126059]	-0.29328683	0.0073988605
A_23_P314070	*LRRC17*	Homo sapiens leucine rich repeat containing 17 (LRRC17), transcript variant 1, mRNA [NM_001031692]	2.3737311	0.0034156272
A_23_P139919	*RAI1*	Homo sapiens mRNA for retinoid-acid induced protein 1 (RAI1 gene). [AJ271790]	0.9265308	0.012479078
A_24_P278460	*ARHGAP1*	Homo sapiens Rho GTPase activating protein 1 (ARHGAP1), mRNA [NM_004308]	-1.1684587	0.0071645253

A_33_P3249135	*CHST11*	Homo sapiens carbohydrate (chondroitin 4) sulfotransferase 11 (CHST11), transcript variant 1, mRNA [NM_018413]	-1.8232484	0.008137207
A_23_P73540	*FAR1*	Homo sapiens fatty acyl CoA reductase 1 (FAR1), mRNA [NM_032228]	-0.46456742	0.01217032
A_24_P405190	*NBEAL1*	Homo sapiens neurobeachin-like 1 (NBEAL1), mRNA [NM_001114132]	-0.8287231	0.003993975
A_23_P318646	*CCDC120*	Homo sapiens coiled-coil domain containing 120 (CCDC120), transcript variant 3, mRNA [NM_033626]	-2.1568902	0.0029471128
A_33_P3254191	*USP33*	Homo sapiens ubiquitin specific peptidase 33 (USP33), transcript variant 3, mRNA [NM_201626]	-0.67930096	0.009679106
A_24_P244410	*RPS10*	Homo sapiens ribosomal protein S10 (RPS10), transcript variant 2, mRNA [NM_001014]	-0.45901942	0.016465655
A_24_P108291	*OSBPL8*	Homo sapiens oxysterol binding protein-like 8 (OSBPL8), transcript variant 1, mRNA [NM_020841]	-0.5371955	0.0072230194
A_33_P3318292	*ANAPC15*	Homo sapiens anaphase promoting complex subunit 15 (ANAPC15), transcript variant 3, mRNA [NM_014042]	0.42192382	0.0059217354
A_22_P00008813	*IMPACT*	Homo sapiens impact RWD domain protein (IMPACT), mRNA [NM_018439]	-1.1759917	0.0048989807
A_21_P0014285	*SFPQ*	Homo sapiens splicing factor proline/glutamine-rich (SFPQ), mRNA [NM_005066]	0.34315968	0.00951954
A_33_P3420466	*LOC102725022*	PREDICTED: Homo sapiens uncharacterized LOC102725022 (LOC102725022), transcript variant X3, misc_RNA [XR_430889]	0.8326565	0.00628389
A_22_P00012882	*LINC01234*	Homo sapiens long intergenic non-protein coding RNA 1234 (LINC01234), transcript variant 1, long non-coding RNA [NR_110026]	0.7478487	0.00978609
A_24_P408047	*MATN3*	Homo sapiens matrilin 3 (MATN3), mRNA [NM_002381]	-0.90316176	0.014825912
A_23_P78018	*lnc-RBM11-1*	LNCipedia lincRNA (lnc-RBM11-1), lincRNA [lnc-RBM11-1:1]	0.68640506	0.008749787
A_23_P259090	*PLEKHA4*	Homo sapiens pleckstrin homology domain containing, family A (phosphoinositide binding specific) member 4 (PLEKHA4), transcript variant 1, mRNA [NM_020904]	-1.0302266	0.0057647754
A_32_P78816	*ABCA5*	Homo sapiens ATP-binding cassette, sub-family A (ABC1), member 5 (ABCA5), transcript variant 1, mRNA [NM_018672]	-0.8804384	0.003162355
A_21_P0000711	*NUDT12*	Homo sapiens nudix (nucleoside diphosphate linked moiety X)-type motif 12 (NUDT12), transcript variant 1, mRNA [NM_031438]	-0.73478216	0.0059070494
A_33_P3358347	*PSPH*	Homo sapiens phosphoserine phosphatase (PSPH), mRNA [NM_004577]	-1.0824983	0.0043581175

A_33_P3272990	*SRD5A3-AS1*	Homo sapiens SRD5A3 antisense RNA 1 (SRD5A3-AS1), long non-coding RNA [NR_037969]	-1.6724075	0.005559843
A_23_P20743	*FKBP8*	Homo sapiens FK506 binding protein 8, 38kDa (FKBP8), mRNA [NM_012181]	1.3546884	0.0074561136
A_33_P3387621	*INADL*	Homo sapiens InaD-like (Drosophila) (INADL), mRNA [NM_176877]	-0.9228642	0.00978609
A_24_P329597	*TMEM246*	Homo sapiens transmembrane protein 246 (TMEM246), transcript variant 1, mRNA [NM_032342]	-3.1641617	0.0025001846
A_33_P3288839	*RHPN2*	Homo sapiens rhophilin, Rho GTPase binding protein 2 (RHPN2), mRNA [NM_033103]	1.5788836	0.0066194455
A_23_P216610	*UBQLN1*	Homo sapiens ubiquilin 1 (UBQLN1), transcript variant 1, mRNA [NM_013438]	-0.94816184	0.007050734
A_21_P0006958	*C14orf37*	Homo sapiens chromosome 14 open reading frame 37 (C14orf37), mRNA [NM_001001872]	-2.1304991	0.005640208
A_23_P4007	*SUSD1*	Homo sapiens sushi domain containing 1 (SUSD1), transcript variant 2, mRNA [NM_022486]	-0.8918936	0.013163641
A_21_P0000316	*lnc-AKR1C2-3*	LNCipedia lincRNA (lnc-AKR1C2-3), lincRNA [lnc-AKR1C2-3:2]	1.628156	0.0073067984
A_24_P178093	*FXR2*	Homo sapiens fragile X mental retardation, autosomal homolog 2 (FXR2), mRNA [NM_004860]	0.8881588	0.0083268415
A_23_P73530	*SNORA20*	Homo sapiens small nucleolar RNA, H/ACA box 20 (SNORA20), small nucleolar RNA [NR_002960]	0.64175963	0.012893396
A_19_P00805954	*TOMM40*	Homo sapiens translocase of outer mitochondrial membrane 40 homolog (yeast) (TOMM40), transcript variant 3, mRNA [NM_006114]	1.6719383	0.005947757
A_33_P3262452	*MTMR1*	Homo sapiens myotubularin related protein 1 (MTMR1), mRNA [NM_003828]	-1.1941612	0.004359828
A_23_P117933	*LOC100129461*	Homo sapiens uncharacterized LOC100129461 (LOC100129461), transcript variant 2, long non-coding RNA [NR_126016]	-0.55171496	0.015810816
A_23_P125668	*PEX26*	Homo sapiens peroxisomal biogenesis factor 26 (PEX26), transcript variant 2, mRNA [NM_001127649]	-0.6198797	0.014349793
A_33_P3229037	*GCSH*	Homo sapiens glycine cleavage system protein H (aminomethyl carrier) (GCSH), transcript variant 1, mRNA [NM_004483]	-0.59241414	0.015868282
A_33_P3333488	*SLC25A43*	Homo sapiens solute carrier family 25, member 43 (SLC25A43), mRNA [NM_145305]	-1.5613865	0.0037583082
A_33_P3357753	*HAGH*	Homo sapiens hydroxyacylglutathione hydrolase (HAGH), transcript variant 2, mRNA [NM_001040427]	-1.3787684	0.011110267

A_23_P42935	*S100A13*	Homo sapiens S100 calcium binding protein A13 (S100A13), transcript variant 1, mRNA [NM_001024210]	-0.53655386	0.008122174
A_33_P3263417	*NUTM2A-AS1*	Homo sapiens NUTM2A antisense RNA 1 (NUTM2A-AS1), long non-coding RNA [NR_024397]	-0.5790546	0.0038456172
A_23_P67661	*BRAF*	Homo sapiens B-Raf proto-oncogene, serine/threonine kinase (BRAF), mRNA [NM_004333]	-0.47657898	0.0108927535
A_33_P3355831	*LINC-PINT*	Homo sapiens long intergenic non-protein coding RNA, p53 induced transcript (LINC-PINT), transcript variant 4, long non-coding RNA [NR_109851]	-0.8962194	0.0060804365
A_21_P0005172	*COX7A1*	cytochrome c oxidase subunit VIIa polypeptide 1 (muscle) [Source:HGNC Symbol;Acc:HGNC:2287] [ENST00000292907]	-1.280139	0.004935436
A_32_P148345	*ZNF213*	Homo sapiens zinc finger protein 213 (ZNF213), transcript variant 1, mRNA [NM_004220]	1.0785123	0.011411947
A_23_P338233	*LINC00472*	Homo sapiens long intergenic non-protein coding RNA 472 (LINC00472), transcript variant 1, long non-coding RNA [NR_121612]	-0.89812857	0.005377644
A_24_P220058	*ANXA2*	Homo sapiens annexin A2 (ANXA2), transcript variant 2, mRNA [NM_001002857]	-1.2409104	0.004884975
A_23_P213375	*BCDIN3D*	Homo sapiens BCDIN3 domain containing (BCDIN3D), mRNA [NM_181708]	-1.23493	0.004890946
A_24_P125283	*MAPRE1*	Homo sapiens microtubule-associated protein, RP/EB family, member 1 (MAPRE1), mRNA [NM_012325]	-0.7473747	0.006173301
A_24_P551842	*PCDHB2*	Homo sapiens protocadherin beta 2 (PCDHB2), mRNA [NM_018936]	1.2712047	0.008541028
A_24_P414786	*HDAC5*	Homo sapiens histone deacetylase 5 (HDAC5), transcript variant 3, mRNA [NM_001015053]	-1.1830559	0.006040495
A_33_P3465247	*CYTB*	mitochondrially encoded cytochrome b [Source:HGNC Symbol;Acc:HGNC:7427] [ENST00000361789]	0.67654604	0.0040860237
A_23_P377434	*CDC40*	Homo sapiens cell division cycle 40 (CDC40), mRNA [NM_015891]	-0.8473007	0.005052031
A_33_P3323842	*KIF3A*	Homo sapiens kinesin family member 3A (KIF3A), transcript variant 1, mRNA [NM_001300791]	-0.5579992	0.008437608
A_24_P186379	*TNRC18*	Homo sapiens trinucleotide repeat containing 18 (TNRC18), mRNA [NM_001080495]	-0.9170254	0.004437345
A_23_P218807	*BDNF-AS*	Homo sapiens BDNF antisense RNA (BDNF-AS), transcript variant BT2B, long non-coding RNA [NR_002832]	-1.3062292	0.005501472
A_24_P48403	*FUOM*	Homo sapiens fucose mutarotase (FUOM), transcript variant 2, mRNA [NM_198472]	-0.7843253	0.012552783

A_23_P83149	*ZC3H7B*	Homo sapiens zinc finger CCCH-type containing 7B (ZC3H7B), mRNA [NM_017590]	-1.079988	0.0075782244
A_22_P00009023	*YES1*	Homo sapiens YES proto-oncogene 1, Src family tyrosine kinase (YES1), mRNA [NM_005433]	-0.6001239	0.005428736
A_33_P3322236	*ABHD17B*	Homo sapiens abhydrolase domain containing 17B (ABHD17B), transcript variant 1, mRNA [NM_016014]	-0.35035288	0.0130611155
A_23_P88201	*FLG-AS1*	Homo sapiens FLG antisense RNA 1 (FLG-AS1), transcript variant 1, long non-coding RNA [NR_103778]	-1.8685482	0.0057513886
A_33_P3256695	*PPP2R3C*	Homo sapiens protein phosphatase 2, regulatory subunit B'', gamma (PPP2R3C), mRNA [NM_017917]	-0.6895554	0.012353736
A_33_P3832852	*EEF1E1*	Homo sapiens eukaryotic translation elongation factor 1 epsilon 1 (EEF1E1), transcript variant 2, mRNA [NM_001135650]	-0.31543207	0.007264689
A_32_P110872	*PRPF40A*	Homo sapiens PRP40 pre-mRNA processing factor 40 homolog A (S. cerevisiae) (PRPF40A), mRNA [NM_017892]	-0.90517426	0.007402553
A_32_P36046	*HAUS2*	Homo sapiens HAUS augmin-like complex, subunit 2 (HAUS2), transcript variant 1, mRNA [NM_018097]	-0.79555964	0.011851147
A_23_P400078	*GGACT*	Homo sapiens gamma-glutamylamine cyclotransferase (GGACT), transcript variant 1, mRNA [NM_033110]	-1.7337469	0.0041063502
A_33_P3313030	*C2orf68*	Homo sapiens chromosome 2 open reading frame 68 (C2orf68), mRNA [NM_001013649]	-1.2293545	0.0051987534
A_33_P3242373	*MTHFR*	Homo sapiens methylenetetrahydrofolate reductase (NAD(P)H) (MTHFR), mRNA [NM_005957]	-1.2869486	0.004016376
A_22_P00018349	*TXNDC15*	Homo sapiens thioredoxin domain containing 15 (TXNDC15), mRNA [NM_024715]	-0.9810455	0.011007661
A_21_P0005703	*AFF1*	Homo sapiens AF4/FMR2 family, member 1 (AFF1), transcript variant 2, mRNA [NM_005935]	-0.6063678	0.015790157
A_24_P82135	*lnc-RAB2A-1*	LNCipedia lincRNA (lnc-RAB2A-1), lincRNA [lnc-RAB2A-1:1]	-0.83802485	0.0029675725
A_33_P3289192	*PCNA-AS1*	Homo sapiens PCNA antisense RNA 1 (PCNA-AS1), long non-coding RNA [NR_028370]	1.0401778	0.0077546863
A_24_P206776	*SECISBP2L*	Homo sapiens SECIS binding protein 2-like (SECISBP2L), transcript variant 2, mRNA [NM_014701]	-0.76026046	0.010752312
A_22_P00015717	*HBS1L*	Homo sapiens HBS1-like translational GTPase (HBS1L), transcript variant 1, mRNA [NM_006620]	-0.58878344	0.0060600177
A_23_P394448	*CRYAB*	Homo sapiens crystallin, alpha B (CRYAB), transcript variant 1, mRNA [NM_001885]	-2.3357	0.0039856653

A_23_P328621	*DPY19L1*	Homo sapiens dpy-19-like 1 (C. elegans) (DPY19L1), mRNA [NM_015283]	-0.49065554	0.014146073
A_23_P63026	*MTRNR2L7*	Homo sapiens MT-RNR2-like 7 (MTRNR2L7), mRNA [NM_001190489]	-0.8525425	0.010835073
A_24_P403561	*UBQLNL*	Homo sapiens ubiquilin-like (UBQLNL), mRNA [NM_145053]	-0.8890293	0.0075857607
A_22_P00021911	*LGALS8*	Homo sapiens lectin, galactoside-binding, soluble, 8 (LGALS8), transcript variant 1, mRNA [NM_006499]	-1.1870621	0.005182173
A_33_P3212615	*LRP4*	Homo sapiens low density lipoprotein receptor-related protein 4 (LRP4), mRNA [NM_002334]	-2.8364944	0.0034766807
A_23_P61127	*lnc-BRF1-2*	LNCipedia lincRNA (lnc-BRF1-2), lincRNA [lnc-BRF1-2:1]	-0.45641682	0.013926392
A_33_P3390546	*TFPI*	Homo sapiens tissue factor pathway inhibitor (lipoprotein-associated coagulation inhibitor) (TFPI), transcript variant 1, mRNA [NM_006287]	0.5456925	0.012487623
A_23_P167997	*APOO*	Homo sapiens apolipoprotein O (APOO), transcript variant 1, mRNA [NM_024122]	-1.0288569	0.008859262
A_33_P3260016	*HIST1H2BG*	Homo sapiens histone cluster 1, H2bg (HIST1H2BG), mRNA [NM_003518]	0.58667463	0.009431794
A_33_P3388938	*KCNRG*	Homo sapiens potassium channel regulator (KCNRG), transcript variant 2, mRNA [NM_199464]	-0.6255748	0.01047417
A_24_P302695	*NEDD1*	Homo sapiens neural precursor cell expressed, developmentally down-regulated 1 (NEDD1), transcript variant 2, mRNA [NM_152905]	-0.72850776	0.013149154
A_33_P3301620	*SNAI3-AS1*	Homo sapiens SNAI3 antisense RNA 1 (SNAI3-AS1), transcript variant 1, long non-coding RNA [NR_024402]	-0.9424149	0.01574856
A_23_P14273	*MRPL30*	Homo sapiens mitochondrial ribosomal protein L30 (MRPL30), transcript variant 1, mRNA [NM_145212]	-0.51981354	0.007797447
A_33_P3298861	*RNF32*	Homo sapiens ring finger protein 32 (RNF32), transcript variant 2, mRNA [NM_001184997]	1.300883	0.004803986
A_23_P13073	*ZFYVE21*	Homo sapiens zinc finger, FYVE domain containing 21 (ZFYVE21), transcript variant 2, mRNA [NM_024071]	-0.7420714	0.015868904
A_33_P3499174	*SLC12A8*	Homo sapiens solute carrier family 12, member 8 (SLC12A8), transcript variant 1, mRNA [NM_024628]	1.3208095	0.00969217
A_23_P397238	*PUS3*	Homo sapiens pseudouridylate synthase 3 (PUS3), transcript variant 1, mRNA [NM_031307]	-0.51118606	0.00975317
A_33_P3298128	*URM1*	Homo sapiens ubiquitin related modifier 1 (URM1), transcript variant 3, mRNA [NM_001265582]	-0.7204322	0.013770927

A_23_P139722	*FKBP1A*	Homo sapiens FK506 binding protein 1A, 12kDa (FKBP1A), transcript variant 2, mRNA [NM_054014]	0.75596833	0.009431794
A_33_P3362869	*ITPR2*	Homo sapiens inositol 1,4,5-trisphosphate receptor, type 2 (ITPR2), mRNA [NM_002223]	-1.4798388	0.0039416323
A_23_P90463	*TNFRSF1A*	Homo sapiens tumor necrosis factor receptor superfamily, member 1A (TNFRSF1A), mRNA [NM_001065]	-0.30222794	0.0031941293
A_21_P0000582	*DNAJC19*	Homo sapiens DnaJ (Hsp40) homolog, subfamily C, member 19 (DNAJC19), transcript variant 2, mRNA [NM_001190233]	-0.93928766	0.0037458793
A_33_P3271051	*LSM7*	Homo sapiens LSM7 homolog, U6 small nuclear RNA associated (S. cerevisiae) (LSM7), mRNA [NM_016199]	-0.61411804	0.014847365
A_33_P3224819	*LOC100134317*	Homo sapiens uncharacterized LOC100134317 (LOC100134317), long non-coding RNA [NR_029389]	2.5743623	0.0056355232
A_24_P787947	*CYTH3*	Homo sapiens cytohesin 3 (CYTH3), mRNA [NM_004227]	-1.5239453	0.0035238392
A_33_P3327822	*SLC45A4*	Homo sapiens solute carrier family 45, member 4 (SLC45A4), transcript variant 1, mRNA [NM_001286646]	-0.83649313	0.015089842
A_19_P00810899	*YPEL2*	Homo sapiens yippee-like 2 (Drosophila) (YPEL2), mRNA [NM_001005404]	-1.5228171	0.0038144474
A_33_P3284197	*SH3BGR*	Homo sapiens SH3 domain binding glutamate-rich protein (SH3BGR), transcript variant 1, mRNA [NM_007341]	-0.43217292	0.010822477
A_23_P397055	*SNHG20*	Homo sapiens small nucleolar RNA host gene 20 (non-protein coding) (SNHG20), long non-coding RNA [NR_027058]	-1.1335192	0.0049063917
A_33_P3367361	*POMGNT1*	Homo sapiens protein O-linked mannose N-acetylglucosaminyltransferase 1 (beta 1,2-) (POMGNT1), transcript variant 2, mRNA [NM_001243766]	-0.6562147	0.014023312
A_32_P153892	*CHPF2*	Homo sapiens chondroitin polymerizing factor 2 (CHPF2), transcript variant 1, mRNA [NM_019015]	1.2459815	0.0073096966
A_23_P202170	*FAM107B*	Homo sapiens family with sequence similarity 107, member B (FAM107B), transcript variant 1, mRNA [NM_001282695]	0.59427243	0.016440568
A_23_P218434	*TAF3*	Homo sapiens TAF3 RNA polymerase II, TATA box binding protein (TBP)-associated factor, 140kDa (TAF3), mRNA [NM_031923]	-0.32282764	0.007358156
A_23_P144627	*MGEA5*	Homo sapiens meningioma expressed antigen 5 (hyaluronidase) (MGEA5), transcript variant 1, mRNA [NM_012215]	-1.2754068	0.0049686665

A_24_P942481	*ZNF226*	Homo sapiens zinc finger protein 226 (ZNF226), transcript variant 3, mRNA [NM_001032374]	-0.6549837	0.0054868753
A_33_P3236661	*PCDHB13*	Homo sapiens protocadherin beta 13 (PCDHB13), mRNA [NM_018933]	0.5727804	0.0050054174
A_33_P3230548	*GPR180*	Homo sapiens G protein-coupled receptor 180 (GPR180), mRNA [NM_180989]	-0.2845361	0.010314042
A_23_P361469	*PCDH9*	Homo sapiens protocadherin 9, mRNA (cDNA clone MGC:167030 IMAGE:8860363), complete cds. [BC150296]	1.394947	0.0050554653
A_23_P171237	*KIF14*	Homo sapiens kinesin family member 14 (KIF14), mRNA [NM_014875]	-0.88753724	0.009649863
A_22_P00013922	*METTL6*	Homo sapiens methyltransferase like 6 (METTL6), transcript variant 1, mRNA [NM_152396]	-0.7491817	0.015328013
A_23_P202408	*ACRC*	Homo sapiens acidic repeat containing (ACRC), mRNA [NM_052957]	-2.549035	0.003363001
A_23_P320897	*HDX*	highly divergent homeobox [Source:HGNC Symbol;Acc:HGNC:26411] [ENST00000465509]	-0.9984305	0.013076766
A_23_P92042	*HERC4*	Homo sapiens HECT and RLD domain containing E3 ubiquitin protein ligase 4 (HERC4), transcript variant 3, mRNA [NM_001278185]	-0.7528155	0.003557021
A_22_P00015112	*DENND1B*	Homo sapiens DENN/MADD domain containing 1B (DENND1B), transcript variant 3, mRNA [NM_001195215]	-0.72388077	0.014448771
A_23_P257743	*ITPR1*	Homo sapiens inositol 1,4,5-trisphosphate receptor, type 1 (ITPR1), transcript variant 2, mRNA [NM_002222]	-0.7362257	0.005822303
A_33_P3375002	*lnc-SNX17-1*	ALU1_HUMAN (P39188) Alu subfamily J sequence contamination warning entry, partial (9%) [THC2525711]	0.95461476	0.007549738
A_21_P0007949	*SHB*	Homo sapiens Src homology 2 domain containing adaptor protein B (SHB), mRNA [NM_003028]	1.5756351	0.008307647
A_23_P431789	*SOCS2-AS1*	Homo sapiens SOCS2 antisense RNA 1 (SOCS2-AS1), long non-coding RNA [NR_038263]	-1.4979979	0.0059540337
A_33_P3273719	*lnc-SCEL-1*	LNCipedia lincRNA (lnc-SCEL-1), lincRNA [lnc-SCEL-1:1]	1.284131	0.014949756
A_23_P43150	*ARF4*	Homo sapiens ADP-ribosylation factor 4 (ARF4), mRNA [NM_001660]	-0.6100528	0.004404579
A_33_P3271350	*ELOVL5*	Homo sapiens ELOVL fatty acid elongase 5 (ELOVL5), transcript variant 4, mRNA [NM_001242831]	-0.6851318	0.008541028
A_21_P0014944	*ZHX1*	Homo sapiens zinc fingers and homeoboxes 1 (ZHX1), transcript variant 1, mRNA [NM_001017926]	-1.3162956	0.0026564114

A_33_P3411991	*IKBKAP*	Homo sapiens inhibitor of kappa light polypeptide gene enhancer in B-cells, kinase complex-associated protein (IKBKAP), mRNA [NM_003640]	-0.48706228	0.010986003
A_22_P00001250	*LOC102724156*	PREDICTED: Homo sapiens uncharacterized LOC102724156 (LOC102724156), transcript variant X1, ncRNA [XR_426407]	0.9599649	0.0064972234
A_24_P202581	*RABGAP1*	Homo sapiens RAB GTPase activating protein 1 (RABGAP1), mRNA [NM_012197]	-1.10555	0.011456508
A_33_P3420747	*ARHGEF1*	Homo sapiens Rho guanine nucleotide exchange factor (GEF) 1 (ARHGEF1), transcript variant 1, mRNA [NM_199002]	1.4654313	0.008651172
A_23_P47377	*MORF4L1*	Homo sapiens mortality factor 4 like 1 (MORF4L1), transcript variant 2, mRNA [NM_206839]	-1.1237504	0.0024890031
A_22_P00023586	*YTHDC2*	Homo sapiens YTH domain containing 2 (YTHDC2), mRNA [NM_022828]	-0.4898264	0.013728742
A_24_P130041	*HSD17B12*	Homo sapiens hydroxysteroid (17-beta) dehydrogenase 12 (HSD17B12), mRNA [NM_016142]	-0.65103364	0.010572384
A_23_P152028	*HOXA9*	Homo sapiens homeobox A9 (HOXA9), mRNA [NM_152739]	-0.35779667	0.0086486135
A_23_P301984	*CYP51A1*	Homo sapiens cytochrome P450, family 51, subfamily A, polypeptide 1 (CYP51A1), transcript variant 1, mRNA [NM_000786]	-0.35677674	0.0065117455
A_33_P3210218	*TRIP4*	Homo sapiens thyroid hormone receptor interactor 4 (TRIP4), mRNA [NM_016213]	-0.77847534	0.006175962
A_23_P145694	*C8orf48*	Homo sapiens chromosome 8 open reading frame 48 (C8orf48), mRNA [NM_001007090]	-0.7671539	0.0029071842
A_22_P00010174	*GALNT1*	Homo sapiens polypeptide N-acetylgalactosaminyltransferase 1 (GALNT1), mRNA [NM_020474]	-0.59222823	0.01489746
A_22_P00002410	*ASNS*	Homo sapiens asparagine synthetase (glutamine-hydrolyzing) (ASNS), transcript variant 2, mRNA [NM_001673]	-0.9212177	0.0025001846
A_24_P304154	*FGD5-AS1*	Homo sapiens FGD5 antisense RNA 1 (FGD5-AS1), transcript variant 1, long non-coding RNA [NR_046251]	-1.0914448	0.003667498
A_23_P89884	*VASH1*	Homo sapiens vasohibin 1 (VASH1), mRNA [NM_014909]	0.9023032	0.006173301
A_23_P166686	*AMPD3*	Homo sapiens adenosine monophosphate deaminase 3 (AMPD3), transcript variant 3, mRNA [NM_001025390]	-0.8817734	0.0085703395
A_22_P00001596	*TRIM28*	Homo sapiens tripartite motif containing 28 (TRIM28), mRNA [NM_005762]	1.3093193	0.008545737
A_23_P4474	*AMOTL2*	Homo sapiens angiomotin like 2 (AMOTL2), transcript variant 2, mRNA [NM_016201]	-0.9556451	0.008066016

A_24_P288448	*lnc-ARPC2-1*	Homo sapiens cDNA FLJ36582 fis, clone TRACH2013081. [AK093901]	-0.94073987	0.008560784
A_23_P19852	*IER3IP1*	Homo sapiens immediate early response 3 interacting protein 1 (IER3IP1), mRNA [NM_016097]	-0.54920053	0.008409678
A_22_P00007879	*RASSF2*	Ras association (RalGDS/AF-6) domain family member 2 [Source:HGNC Symbol;Acc:HGNC:9883] [ENST00000478553]	0.7982559	0.015665986
A_23_P207666	*IQCE*	Homo sapiens IQ motif containing E (IQCE), transcript variant 1, mRNA [NM_152558]	-1.0539103	0.006573993
A_23_P394917	*lnc-HSCB-1*	Homo sapiens cDNA clone IMAGE:5192449, partial cds. [BC060374]	-0.54112	0.011728736
A_33_P3317725	*USP32*	Homo sapiens ubiquitin specific peptidase 32 (USP32), mRNA [NM_032582]	-0.43529916	0.012866299
A_24_P217834	*SRCAP*	Homo sapiens Snf2-related CREBBP activator protein (SRCAP), mRNA [NM_006662]	1.5258669	0.0032831472
A_21_P0014048	*LINC00243*	Homo sapiens cDNA FLJ40693 fis, clone THYMU2025042. [AK098012]	-0.6961145	0.011794303
A_21_P0013952	*HIST1H3D*	Homo sapiens histone cluster 1, H3d (HIST1H3D), mRNA [NM_003530]	0.4822948	0.015044886
A_23_P24244	*RBFOX3*	Homo sapiens RNA binding protein, fox-1 homolog (C. elegans) 3 (RBFOX3), mRNA [NM_001082575]	0.8829689	0.007196166
A_22_P00008931	*CCT4*	Homo sapiens chaperonin containing TCP1, subunit 4 (delta) (CCT4), transcript variant 1, mRNA [NM_006430]	-0.4753933	0.012215228
A_24_P134356	*FAM208B*	Homo sapiens family with sequence similarity 208, member B (FAM208B), mRNA [NM_017782]	-0.61610454	0.016981635
A_24_P181677	*BTBD3*	Homo sapiens BTB (POZ) domain containing 3 (BTBD3), transcript variant 1, mRNA [NM_014962]	-0.730277	0.010573547
A_33_P3329549	*C19orf66*	Homo sapiens chromosome 19 open reading frame 66 (C19orf66), mRNA [NM_018381]	0.7517493	0.012288998
A_22_P00017353	*PQLC1*	Homo sapiens PQ loop repeat containing 1 (PQLC1), transcript variant 1, mRNA [NM_025078]	0.96800506	0.01386758
A_23_P93562	*FBRS*	Homo sapiens fibrosin (FBRS), mRNA [NM_001105079]	0.8963537	0.009811661
A_33_P3264419	*SESN1*	Homo sapiens sestrin 1 (SESN1), transcript variant 1, mRNA [NM_014454]	-1.4639885	0.0035894548
A_23_P7827	*LRCH2*	Homo sapiens leucine-rich repeats and calponin homology (CH) domain containing 2 (LRCH2), transcript variant 1, mRNA [NM_020871]	-0.48136216	0.014755079
A_23_P76882	*YTHDF3*	Homo sapiens YTH N(6)-methyladenosine RNA binding protein 3 (YTHDF3), transcript variant 2, mRNA [NM_001277813]	-0.6095236	0.012364148

A_24_P373126	*FAM26F*	Homo sapiens family with sequence similarity 26, member F (FAM26F), transcript variant 1, mRNA [NM_001010919]	-2.206303	0.004657079
A_23_P230	*CCNB1IP1*	Homo sapiens cyclin B1 interacting protein 1, E3 ubiquitin protein ligase (CCNB1IP1), transcript variant 4, mRNA [NM_182852]	-1.0270777	0.011326066
A_24_P218006	*TRMT61A*	Homo sapiens tRNA methyltransferase 61A (TRMT61A), mRNA [NM_152307]	0.8925892	0.015963586
A_23_P150609	*TTC4*	Homo sapiens tetratricopeptide repeat domain 4 (TTC4), transcript variant 1, mRNA [NM_004623]	0.56156844	0.016443476
A_23_P27306	*DNAJC30*	Homo sapiens DnaJ (Hsp40) homolog, subfamily C, member 30 (DNAJC30), mRNA [NM_032317]	-0.64581144	0.014578919
A_24_P269432	*IGF2*	Homo sapiens insulin-like growth factor 2 (IGF2), transcript variant 1, mRNA [NM_000612]	2.6674378	0.0050508454
A_21_P0012156	*COLEC12*	Homo sapiens collectin sub-family member 12 (COLEC12), mRNA [NM_130386]	-1.0649121	0.003993975
A_24_P217330	*BET1*	Homo sapiens Bet1 golgi vesicular membrane trafficking protein (BET1), mRNA [NM_005868]	-0.44063804	0.009228974
A_33_P3268310	*POMGNT2*	Homo sapiens protein O-linked mannose N-acetylglucosaminyltransferase 2 (beta 1,4-) (POMGNT2), mRNA [NM_032806]	0.88251126	0.015208612
A_33_P3291510	*DISP2*	Homo sapiens dispatched homolog 2 (Drosophila) (DISP2), mRNA [NM_033510]	-1.2056628	0.006514609
A_23_P44363	*LIMS3L*	Homo sapiens LIM and senescent cell antigen-like domains 3-like (LIMS3L), transcript variant 1, mRNA [NM_001205288]	1.7433383	0.0060066204
A_23_P410587	*VCY*	Homo sapiens variable charge, Y-linked (VCY), mRNA [NM_004679]	1.0112724	0.008716995
A_21_P0011595	*CASKIN2*	Homo sapiens CASK interacting protein 2 (CASKIN2), transcript variant 1, mRNA [NM_020753]	0.7771044	0.011328784
A_21_P0010155	*JADE1*	Homo sapiens jade family PHD finger 1 (JADE1), transcript variant 2, mRNA [NM_024900]	-1.0293248	0.0047719665
A_22_P00019273	*XLOC_l2_006101*	BROAD Institute lincRNA (XLOC_l2_006101), lincRNA [TCONS_l2_00011289]	1.2672687	0.008853051
A_23_P117734	*LOC284825*	Homo sapiens uncharacterized LOC284825 (LOC284825), transcript variant 2, long non-coding RNA [NR_126011]	2.559317	0.005651752
A_33_P3328659	*lnc-MED7-1*	Homo sapiens cDNA FLJ34312 fis, clone FEBRA2008265. [AK091631]	-1.2413249	0.0034903844
A_24_P307014	*LYSMD4*	Homo sapiens LysM, putative peptidoglycan-binding, domain containing 4 (LYSMD4), transcript variant 1, mRNA [NM_152449]	-1.1081519	0.008189544

A_24_P795371	*CELSR1*	Homo sapiens cadherin, EGF LAG seven-pass G-type receptor 1 (CELSR1), mRNA [NM_014246]	1.3283451	0.004522109
A_23_P323180	*BRD2*	Homo sapiens bromodomain containing 2 (BRD2), transcript variant 1, mRNA [NM_005104]	-0.67847365	0.013163641
A_24_P40417	*NR2F2-AS1*	Homo sapiens NR2F2 antisense RNA 1 (NR2F2-AS1), transcript variant 1, long non-coding RNA [NR_102743]	0.8437491	0.013079763
A_19_P00317237	*HOXD3*	Homo sapiens homeobox D3 (HOXD3), mRNA [NM_006898]	-2.6787379	0.004884975
A_33_P3283828	*FMR1*	Homo sapiens fragile X mental retardation 1 (FMR1), transcript variant ISO1, mRNA [NM_002024]	-0.7380441	0.007385569
A_33_P6592586	*lnc-AC114947.1.1-2*	Q5WDH4_BACSK (Q5WDH4) Phosphoglycolate phosphatase, partial (6%) [THC2668937]	-1.0785849	0.007848112
A_21_P0000799	*CCDC154*	Homo sapiens coiled-coil domain containing 154 (CCDC154), mRNA [NM_001143980]	-1.1041354	0.009724618
A_23_P4551	*STARD13*	StAR-related lipid transfer (START) domain containing 13 [Source:HGNC Symbol;Acc:HGNC:19164] [ENST00000344312]	1.7202071	0.0037856582
A_24_P365975	*RNASEH1-AS1*	Homo sapiens RNASEH1 antisense RNA 1 (RNASEH1-AS1), transcript variant 1, long non-coding RNA [NR_038430]	0.72369814	0.007479662
A_23_P216282	*SETBP1*	Homo sapiens SET binding protein 1 (SETBP1), transcript variant 1, mRNA [NM_015559]	-2.352007	0.003363001
A_23_P160354	*COL8A2*	Homo sapiens collagen, type VIII, alpha 2 (COL8A2), transcript variant 1, mRNA [NM_005202]	-1.6286398	0.0061534033
A_23_P252106	*ARHGEF10*	Homo sapiens Rho guanine nucleotide exchange factor (GEF) 10 (ARHGEF10), mRNA [NM_014629]	-0.9019668	0.008153665
A_24_P358205	*AKT3*	Homo sapiens v-akt murine thymoma viral oncogene homolog 3 (AKT3), transcript variant 2, mRNA [NM_181690]	-1.0490494	0.013451378
A_23_P102517	*RIPK2*	Homo sapiens receptor-interacting serine-threonine kinase 2 (RIPK2), mRNA [NM_003821]	0.7374916	0.011821381
A_33_P3279720	*PDE6D*	Homo sapiens phosphodiesterase 6D, cGMP-specific, rod, delta (PDE6D), transcript variant 1, mRNA [NM_002601]	-0.86856985	0.013163641
A_23_P212728	*FAM73A*	Homo sapiens family with sequence similarity 73, member A (FAM73A), transcript variant 2, mRNA [NM_001270384]	-0.69883585	0.007683834
A_21_P0006268	*ARFRP1*	ADP-ribosylation factor related protein 1 [Source:HGNC Symbol;Acc:HGNC:662] [ENST00000612772]	0.31151885	0.015552779

A_33_P3420904	*TBC1D23*	Homo sapiens TBC1 domain family, member 23 (TBC1D23), transcript variant 1, mRNA [NM_001199198]	-0.58747387	0.005229406
A_33_P3220390	*lnc-FAM75A6-1*	LNCipedia lincRNA (lnc-FAM75A6-1), lincRNA [lnc-FAM75A6-1:1]	0.67945033	0.012840108
A_24_P144784	*PATE3*	Homo sapiens prostate and testis expressed 3 (PATE3), mRNA [NM_001129883]	0.2791849	0.0117289275
A_22_P00003888	*WTAP*	Homo sapiens Wilms tumor 1 associated protein, mRNA (cDNA clone IMAGE:5399821), partial cds. [BC028180]	1.560193	0.009574714
A_23_P369701	*HILS1*	Homo sapiens histone linker H1 domain, spermatid-specific 1, pseudogene (HILS1), transcript variant 1, non-coding RNA [NR_024193]	-0.8669175	0.0046784063
A_24_P116606	*lnc-CENPV-2*	Q7ZU62_BRARE (Q7ZU62) Zgc:66168 protein (Fragment), partial (46%) [THC2592602]	1.3611618	0.008537068
A_21_P0000588	*ABHD17C*	Homo sapiens abhydrolase domain containing 17C (ABHD17C), mRNA [NM_021214]	0.41447738	0.008030646
A_23_P90014	*WWOX*	Homo sapiens WW domain containing oxidoreductase (WWOX), transcript variant 2, mRNA [NM_130791]	-1.2509987	0.0057247286
A_33_P3297642	*HOXB-AS3*	Homo sapiens HOXB cluster antisense RNA 3 (HOXB-AS3), transcript variant 8, long non-coding RNA [NR_110331]	-1.4247534	0.003956951
A_24_P203072	*GPR108*	Homo sapiens G protein-coupled receptor 108 (GPR108), mRNA [NM_001080452]	1.15093	0.010035188
A_23_P72157	*FAM204A*	Homo sapiens family with sequence similarity 204, member A (FAM204A), transcript variant 1, mRNA [NM_022063]	-0.8053274	0.008346524
A_33_P3318852	*JADE3*	Homo sapiens jade family PHD finger 3 (JADE3), transcript variant 1, mRNA [NM_014735]	-0.68036336	0.013492972
A_22_P00021723	*MFSD7*	Homo sapiens major facilitator superfamily domain containing 7 (MFSD7), transcript variant 2, mRNA [NM_032219]	-0.9312484	0.009276546
A_23_P4662	*TBC1D8B*	Homo sapiens TBC1 domain family, member 8B (with GRAM domain) (TBC1D8B), transcript variant 2, mRNA [NM_198881]	-0.8231697	0.012516303
A_22_P00007461	*LOC101927078*	PREDICTED: Homo sapiens uncharacterized LOC101927078 (RP11-492A10.1), ncRNA [XR_251845]	0.84733886	0.010136075
A_33_P3247933	*BCL3*	Homo sapiens B-cell CLL/lymphoma 3 (BCL3), mRNA [NM_005178]	-0.73845285	0.01208643
A_23_P100539	*lnc-GTF2A2-2*	BP394394 Homo sapiens pancreatic islet Homo sapiens cDNA clone hbt00084 3', mRNA sequence [BP394394]	-2.5982027	0.004055852

A_24_P321634	*GCC2*	Homo sapiens GRIP and coiled-coil domain containing 2 (GCC2), transcript variant 1, mRNA [NM_181453]	-0.87469935	0.0059911786
A_23_P256542	*ABCC6*	Homo sapiens ATP-binding cassette, sub-family C (CFTR/MRP), member 6 (ABCC6), transcript variant 1, mRNA [NM_001171]	-0.9523058	0.0130220065
A_19_P00322573	*ZMYM5*	Homo sapiens zinc finger, MYM-type 5 (ZMYM5), transcript variant 1, mRNA [NM_001039650]	-0.6354566	0.012620988
A_23_P32233	*FAM162A*	Homo sapiens family with sequence similarity 162, member A (FAM162A), mRNA [NM_014367]	-0.9960682	0.0053387037
A_22_P00014211	*LINC01372*	Homo sapiens long intergenic non-protein coding RNA 1372 (LINC01372), long non-coding RNA [NR_108104]	-1.5150031	0.010237655
A_22_P00025125	*KLF4*	Homo sapiens Kruppel-like factor 4 (gut) (KLF4), mRNA [NM_004235]	-0.8255782	0.015933463
A_23_P115824	*VIPR1-AS1*	Homo sapiens VIPR1 antisense RNA 1 (VIPR1-AS1), long non-coding RNA [NR_046654]	0.45563123	0.0086535355
A_33_P3405360	*lnc-FAM98A-1*	LNCipedia lincRNA (lnc-FAM98A-1), lincRNA [lnc-FAM98A-1:1]	-1.2633673	0.009296599
A_22_P00006547	*HNRNPH3*	Homo sapiens heterogeneous nuclear ribonucleoprotein H3 (2H9) (HNRNPH3), transcript variant 2H9, mRNA [NM_012207]	-0.71277875	0.01225594
A_33_P3306948	*PDCL*	Homo sapiens phosducin-like (PDCL), mRNA [NM_005388]	-0.7421876	0.00584425
A_21_P0000351	*lnc-FKBP2-1*	Q2AC61_9BURK (Q2AC61) HrpW, partial (3%) [THC2716184]	-1.0233932	0.009197153
A_23_P111583	*LRP6*	Homo sapiens low density lipoprotein receptor-related protein 6 (LRP6), mRNA [NM_002336]	-0.7851941	0.005787274
A_24_P315638	*SCARNA22*	Homo sapiens small Cajal body-specific RNA 22 (SCARNA22), guide RNA [NR_003004]	-0.6492086	0.0029734594
A_23_P168951	*CD36*	Homo sapiens CD36 molecule (thrombospondin receptor) (CD36), transcript variant 2, mRNA [NM_001001547]	-0.90271705	0.008471989
A_24_P29001	*MRGPRE*	Homo sapiens MAS-related GPR, member E (MRGPRE), mRNA [NM_001039165]	0.24321467	0.0043581175
A_33_P3341234	*ZHX2*	Homo sapiens zinc fingers and homeoboxes 2 (ZHX2), mRNA [NM_014943]	-1.1970932	0.0086466065
A_19_P00316375	*LSM3*	Homo sapiens LSM3 homolog, U6 small nuclear RNA associated (S. cerevisiae) (LSM3), mRNA [NM_014463]	-0.5131201	0.007698106
A_22_P00002938	*UBE2H*	Homo sapiens ubiquitin-conjugating enzyme E2H (UBE2H), transcript variant 1, mRNA [NM_003344]	-0.853853	0.010964888
A_22_P00016840	*ERI1*	exoribonuclease 1 [Source:HGNC Symbol;Acc:HGNC:23994] [ENST00000520332]	-0.93274677	0.006395745

A_24_P91165	*LOC648987*	Homo sapiens uncharacterized LOC648987 (LOC648987), transcript variant 2, long non-coding RNA [NR_034086]	-1.1729466	0.0027248112
A_23_P354827	*lnc-TRMT61B-2*	DA509635 FCBBF3 Homo sapiens cDNA clone FCBBF3026229 5', mRNA sequence [DA509635]	-0.5791284	0.0065390505
A_22_P00022161	*CACNB1*	Homo sapiens calcium channel, voltage-dependent, beta 1 subunit (CACNB1), transcript variant 3, mRNA [NM_199248]	-0.48958683	0.007323471
A_23_P144264	*ZNF550*	Homo sapiens zinc finger protein 550 (ZNF550), transcript variant 1, mRNA [NM_001277090]	-1.431129	0.0029242039
A_23_P9018	*ADNP-AS1*	Homo sapiens ADNP antisense RNA 1 (ADNP-AS1), transcript variant 1, long non-coding RNA [NR_110007]	-0.71217567	0.0062702326
A_33_P3368879	*TOMM70A*	Homo sapiens translocase of outer mitochondrial membrane 70 homolog A (S. cerevisiae) (TOMM70A), mRNA [NM_014820]	-0.4295126	0.0023561441
A_24_P160401	*DEFB104B*	Homo sapiens defensin, beta 104B (DEFB104B), mRNA [NM_001040702]	0.345205	0.014625823
A_23_P20494	*LMOD1*	Homo sapiens leiomodin 1 (smooth muscle) (LMOD1), mRNA [NM_012134]	-2.612002	0.0059911786
A_23_P145957	*CDCP1*	Homo sapiens CUB domain containing protein 1 (CDCP1), transcript variant 2, mRNA [NM_178181]	4.3049927	0.0025001846
A_32_P50417	*NDRG1*	Homo sapiens N-myc downstream regulated 1 (NDRG1), transcript variant 2, mRNA [NM_006096]	-1.7845681	0.0037705272
A_21_P0003911	*TPK1*	Homo sapiens thiamin pyrophosphokinase 1 (TPK1), transcript variant 1, mRNA [NM_022445]	-1.8806964	0.0037583082
A_33_P3410831	*lnc-DCTD-1*	LNCipedia lincRNA (lnc-DCTD-1), lincRNA [lnc-DCTD-1:1]	-0.71073043	0.012536314
A_33_P3330039	*THY1*	Homo sapiens Thy-1 cell surface antigen (THY1), mRNA [NM_006288]	0.22297189	0.016266534
A_23_P119593	*LOC729609*	Homo sapiens uncharacterized LOC729609 (LOC729609), long non-coding RNA [NR_024440]	-0.8825318	0.013246798
A_33_P3371769	*PLEKHO1*	Homo sapiens pleckstrin homology domain containing, family O member 1 (PLEKHO1), mRNA [NM_016274]	-1.105638	0.005640775
A_32_P743407	*EPHX3*	Homo sapiens epoxide hydrolase 3 (EPHX3), transcript variant 1, mRNA [NM_024794]	0.7406625	0.0063397386
A_22_P00016856	*GLYR1*	Homo sapiens glyoxylate reductase 1 homolog (Arabidopsis) (GLYR1), mRNA [NM_032569]	1.2698574	0.0064965826
A_33_P3257513	*MTDH*	Homo sapiens metadherin (MTDH), mRNA [NM_178812]	-0.57684565	0.003942978

A_23_P105144	*lnc-TRPM7-1*	Homo sapiens full length insert cDNA clone YB40C09. [AF147349]	-0.8558318	0.00652649
A_32_P722809	*FAT3*	Homo sapiens FAT atypical cadherin 3 (FAT3), mRNA [NM_001008781]	2.0754852	0.004522109
A_23_P257763	*SCUBE2*	Homo sapiens signal peptide, CUB domain, EGF-like 2 (SCUBE2), transcript variant 1, mRNA [NM_020974]	-0.6801455	0.016056836
A_22_P00015661	*BOC*	Homo sapiens BOC cell adhesion associated, oncogene regulated (BOC), transcript variant 2, mRNA [NM_033254]	-0.80007935	0.011728736
A_24_P319923	*TTC28*	Homo sapiens tetratricopeptide repeat domain 28 (TTC28), mRNA [NM_001145418]	-0.93536586	0.011624532
A_24_P307827	*LOC101930316*	PREDICTED: Homo sapiens uncharacterized LOC101930316 (LOC101930316), ncRNA [XR_247649]	-0.7512444	0.008159287
A_22_P00019908	*MYLK*	Homo sapiens myosin light chain kinase (MYLK), transcript variant 1, mRNA [NM_053025]	-1.4817741	0.0036759654
A_23_P148121	*WHAMM*	Homo sapiens WAS protein homolog associated with actin, golgi membranes and microtubules (WHAMM), mRNA [NM_001080435]	-0.7393208	0.008574389
A_23_P502224	*lnc-WNT1-2*	LNCipedia lincRNA (lnc-WNT1-2), lincRNA [lnc-WNT1-2:1]	-0.83375406	0.0059872055
A_21_P0011915	*EHBP1L1*	Homo sapiens EH domain binding protein 1-like 1 (EHBP1L1), mRNA [NM_001099409]	0.69092864	0.012495794
A_23_P373687	*CYB5R3*	Homo sapiens cytochrome b5 reductase 3 (CYB5R3), transcript variant 2, mRNA [NM_007326]	-0.8134613	0.0038450547
A_24_P262127	*XLOC_l2_008013*	BROAD Institute lincRNA (XLOC_l2_008013), lincRNA [TCONS_l2_00014567]	-0.8144305	0.0036402496
A_24_P41371	*PUS10*	Homo sapiens pseudouridylate synthase 10 (PUS10), mRNA [NM_144709]	-1.2301232	0.010271231
A_24_P62505	*RRAD*	Homo sapiens Ras-related associated with diabetes (RRAD), transcript variant 2, mRNA [NM_004165]	1.7125444	0.0046744854
A_21_P0008873	*CC2D1A*	Homo sapiens coiled-coil and C2 domain containing 1A (CC2D1A), mRNA [NM_017721]	1.173507	0.005559843
A_24_P303647	*COLGALT2*	Homo sapiens collagen beta(1-O)galactosyltransferase 2 (COLGALT2), transcript variant 1, mRNA [NM_015101]	-1.3198661	0.008437608
A_33_P3322274	*TP53TG3D*	Homo sapiens TP53 target 3D (TP53TG3D), transcript variant 1, mRNA [NM_001243722]	-0.9645928	0.014047869
A_23_P361820	*C7orf60*	Homo sapiens chromosome 7 open reading frame 60 (C7orf60), mRNA [NM_152556]	-0.3914016	0.009785405
A_33_P3210916	*TNFRSF1B*	Homo sapiens tumor necrosis factor receptor superfamily, member 1B (TNFRSF1B), mRNA [NM_001066]	1.3601764	0.0054549226

A_24_P221366	*ATG2A*	Homo sapiens autophagy related 2A (ATG2A), mRNA [NM_015104]	0.83831495	0.01208643
A_23_P390734	*SEC13*	SEC13 homolog (S. cerevisiae) [Source:HGNC Symbol;Acc:HGNC:10697] [ENST00000397102]	0.67244107	0.00866489
A_23_P69030	*RPS15A*	Homo sapiens ribosomal protein S15a (RPS15A), transcript variant 2, mRNA [NM_001019]	-0.5047562	0.004055852
A_21_P0000146	*FGFR1OP2*	Homo sapiens FGFR1 oncogene partner 2 (FGFR1OP2), transcript variant 1, mRNA [NM_015633]	-0.6806269	0.0031827379
A_32_P56713	*COL8A1*	Homo sapiens collagen, type VIII, alpha 1 (COL8A1), transcript variant 1, mRNA [NM_001850]	-1.205084	0.011435631
A_22_P00016110	*PDK2*	Homo sapiens pyruvate dehydrogenase kinase, isozyme 2 (PDK2), transcript variant 4, mRNA [NM_001199900]	-1.2379317	0.005833367
A_24_P280706	*BCR*	Homo sapiens breakpoint cluster region (BCR), transcript variant 1, mRNA [NM_004327]	1.2664984	0.0056287716
A_23_P171054	*THTPA*	Homo sapiens thiamine triphosphatase (THTPA), transcript variant 7, mRNA [NM_001256323]	0.6298747	0.0070571043
A_24_P944616	*SUGT1*	Homo sapiens SGT1, suppressor of G2 allele of SKP1 (S. cerevisiae) (SUGT1), transcript variant 1A, mRNA [NM_006704]	-0.78199655	0.0040942165
A_33_P3396434	*ELK1*	Homo sapiens ELK1, member of ETS oncogene family (ELK1), transcript variant 2, mRNA [NM_005229]	1.096912	0.0134437205
A_21_P0013818	*HP1BP3*	Homo sapiens heterochromatin protein 1, binding protein 3 (HP1BP3), mRNA [NM_016287]	-0.50485206	0.0067707044
A_32_P174365	*XLOC_l2_015752*	BROAD Institute lincRNA (XLOC_l2_015752), lincRNA [TCONS_l2_00030522]	0.75719607	0.010735533
A_23_P61960	*LOC101930053*	PREDICTED: Homo sapiens uncharacterized LOC101930053 (LOC101930053), transcript variant X1, ncRNA [XR_428440]	-0.96622604	0.010782863
A_19_P00321656	*SATB2*	Homo sapiens SATB homeobox 2 (SATB2), transcript variant 2, mRNA [NM_015265]	-0.86835194	0.009091813
A_23_P340158	*ATP6V0E2*	Homo sapiens ATPase, H+ transporting V0 subunit e2 (ATP6V0E2), transcript variant 1, mRNA [NM_145230]	1.8658668	0.0036240483
A_33_P3236030	*DDAH1*	Homo sapiens dimethylarginine dimethylaminohydrolase 1 (DDAH1), transcript variant 2, mRNA [NM_001134445]	-1.6837871	0.0026564114
A_24_P117410	*ISCU*	Homo sapiens iron-sulfur cluster assembly enzyme (ISCU), transcript variant 1, mRNA [NM_014301]	-0.9845724	0.0030042294

A_23_P92441	*ARHGAP24*	Rho GTPase activating protein 24 [Source:HGNC Symbol;Acc:HGNC:25361] [ENST00000509709]	-0.8519215	0.010134572
A_32_P103945	*KLHDC7B*	Homo sapiens kelch domain containing 7B (KLHDC7B), mRNA [NM_138433]	0.7907301	0.014904488
A_23_P159390	*MAD2L1*	Homo sapiens MAD2 mitotic arrest deficient-like 1 (yeast) (MAD2L1), mRNA [NM_002358]	-0.4394166	0.0025079036
A_23_P208674	*B4GALT4*	Homo sapiens UDP-Gal:betaGlcNAc beta 1,4-galactosyltransferase, polypeptide 4 (B4GALT4), transcript variant 1, mRNA [NM_212543]	-0.8582213	0.006812377
A_33_P3591972	*TOPBP1*	Homo sapiens topoisomerase (DNA) II binding protein 1 (TOPBP1), mRNA [NM_007027]	-0.16225456	0.013857105
A_21_P0014295	*EMC10*	Homo sapiens ER membrane protein complex subunit 10 (EMC10), transcript variant 1, mRNA [NM_175063]	-1.1616936	0.0062618116
A_24_P8768	*GLYCTK*	Homo sapiens glycerate kinase (GLYCTK), transcript variant 2, mRNA [NM_001144951]	0.572315	0.014821658
A_33_P3217230	*PIGBOS1*	PREDICTED: Homo sapiens uncharacterized LOC101928527 (LOC101928527), transcript variant X2, ncRNA [XR_243152]	-0.5931825	0.012857543
A_21_P0000506	*MRPL20*	Homo sapiens mitochondrial ribosomal protein L20 (MRPL20), mRNA [NM_017971]	-0.5927353	0.015355276
A_22_P00007240	*ZNF91*	Homo sapiens zinc finger protein 91 (ZNF91), transcript variant 1, mRNA [NM_003430]	-0.705537	0.014766825
A_21_P0000299	*SUZ12P1*	Homo sapiens suppressor of zeste 12 homolog pseudogene 1 (SUZ12P1), non-coding RNA [NR_024187]	-0.76552033	0.007050734
A_24_P14367	*lnc-GPR12-1*	LNCipedia lincRNA (lnc-GPR12-1), lincRNA [lnc-GPR12-1:1]	-0.6560681	0.010301823
A_23_P31135	*SNORA19*	Homo sapiens small nucleolar RNA, H/ACA box 19 (SNORA19), small nucleolar RNA [NR_002917]	-0.8180373	0.005852893
A_33_P3259865	*PTBP1*	Homo sapiens polypyrimidine tract binding protein 1 (PTBP1), transcript variant 1, mRNA [NM_002819]	0.8586691	0.0031941293
A_33_P3342076	*ACAT2*	Homo sapiens acetyl-CoA acetyltransferase 2 (ACAT2), transcript variant 1, mRNA [NM_005891]	0.18967512	0.016428085
A_23_P163647	*C1orf220*	Homo sapiens chromosome 1 open reading frame 220 (C1orf220), long non-coding RNA [NR_033186]	-1.3359035	0.008285209
A_33_P3489646	*PRDM2*	Homo sapiens PR domain containing 2, with ZNF domain (PRDM2), transcript variant 2, mRNA [NM_015866]	-0.7192373	0.010627036
A_23_P396981	*ECI1*	Homo sapiens enoyl-CoA delta isomerase 1 (ECI1), transcript variant 1, mRNA [NM_001919]	-0.99354506	0.0067024035

A_33_P3249595	*SPI1*	Homo sapiens Spi-1 proto-oncogene (SPI1), transcript variant 1, mRNA [NM_001080547]	-0.5035949	0.006573993
A_33_P3213119	*CCDC66*	Homo sapiens coiled-coil domain containing 66 (CCDC66), transcript variant 2, mRNA [NM_001012506]	-1.0015664	0.0126847
A_23_P63908	*BCL11A*	Homo sapiens B-cell CLL/lymphoma 11A (zinc finger protein) (BCL11A), transcript variant 3, mRNA [NM_138559]	-1.0748742	0.00847175
A_33_P3325497	*HAS2-AS1*	Homo sapiens HAS2 antisense RNA 1 (HAS2-AS1), antisense RNA [NR_002835]	-1.2923617	0.011824575
A_33_P3264577	*TRUB1*	Homo sapiens TruB pseudouridine (psi) synthase family member 1 (TRUB1), mRNA [NM_139169]	-0.7048769	0.003162355
A_23_P253571	*FIBIN*	Homo sapiens fin bud initiation factor homolog (zebrafish) (FIBIN), mRNA [NM_203371]	0.9090705	0.0025451183
A_33_P3230166	*DCTN1*	Homo sapiens dynactin 1 (DCTN1), transcript variant 1, mRNA [NM_004082]	0.7176068	0.014959743
A_23_P141802	*PHF5A*	Homo sapiens PHD finger protein 5A (PHF5A), mRNA [NM_032758]	-0.2556853	0.01031573
A_33_P3609431	*NALCN*	Homo sapiens sodium leak channel, non selective (NALCN), mRNA [NM_052867]	-1.4691237	0.00180587
A_23_P120194	*SERPINB7*	Homo sapiens serpin peptidase inhibitor, clade B (ovalbumin), member 7 (SERPINB7), transcript variant 2, mRNA [NM_001040147]	-1.3054762	0.002419416
A_21_P0012174	*ORMDL1*	Homo sapiens ORMDL sphingolipid biosynthesis regulator 1 (ORMDL1), transcript variant 1, mRNA [NM_016467]	-0.6354005	0.007677732
A_23_P217114	*SETD8*	Homo sapiens SET domain containing (lysine methyltransferase) 8 (SETD8), mRNA [NM_020382]	-1.0310134	0.015333281
A_32_P220472	*ALAD*	Homo sapiens aminolevulinate dehydratase (ALAD), mRNA [NM_000031]	-0.9587302	0.005132046
A_23_P59349	*UHRF1BP1*	Homo sapiens UHRF1 binding protein 1 (UHRF1BP1), mRNA [NM_017754]	-0.6854703	0.006155321
A_24_P915196	*ZFAND6*	Homo sapiens zinc finger, AN1-type domain 6 (ZFAND6), transcript variant 1, mRNA [NM_019006]	-0.45837432	0.009543431
A_33_P6822486	*HECA*	Homo sapiens headcase homolog (Drosophila) (HECA), mRNA [NM_016217]	-1.641672	0.004155979
A_23_P42975	*C9orf91*	Homo sapiens chromosome 9 open reading frame 91 (C9orf91), mRNA [NM_153045]	-0.9757445	0.010314908
A_21_P0013582	*LOC100506844*	Homo sapiens uncharacterized LOC100506844 (LOC100506844), long non-coding RNA [NR_038269]	-0.92022705	0.0062618116
A_32_P98298	*PRKAR2B*	Homo sapiens protein kinase, cAMP-dependent, regulatory, type II, beta (PRKAR2B), mRNA [NM_002736]	1.0679605	0.0060199546

A_22_P00008487	*DUSP8*	Homo sapiens dual specificity phosphatase 8 (DUSP8), mRNA [NM_004420]	0.62092924	0.011787466
A_33_P3378031	*ORM1*	Homo sapiens orosomucoid 1 (ORM1), mRNA [NM_000607]	1.7029359	0.0029691157
A_23_P71440	*lnc-KCNC2-1*	UI-HF-BL0-acc-h-09-0-UI.r1 NIH_MGC_37 Homo sapiens cDNA clone IMAGE:3058793 5', mRNA sequence [AW404679]	0.46202803	0.012742897
A_23_P151565	*TMEM18*	Homo sapiens transmembrane protein 18 (TMEM18), mRNA [NM_152834]	-1.0025674	0.0069110035
A_23_P213247	*GOLGA7*	Homo sapiens golgin A7 (GOLGA7), transcript variant 2, mRNA [NM_001002296]	-1.0317671	0.0076440917
A_33_P3224483	*RALGAPA1*	Homo sapiens Ral GTPase activating protein, alpha subunit 1 (catalytic) (RALGAPA1), transcript variant 1, mRNA [NM_014990]	-0.874579	0.0029355595
A_33_P3287388	*FBXL5*	Homo sapiens F-box and leucine-rich repeat protein 5 (FBXL5), transcript variant 1, mRNA [NM_012161]	-1.2951527	0.0047719665
A_33_P3332865	*RABL2B*	Homo sapiens RAB, member of RAS oncogene family-like 2B, mRNA (cDNA clone IMAGE:3902486), with apparent retained intron. [BC020495]	-1.1116022	0.0066995043
A_23_P168629	*NPEPL1*	Homo sapiens aminopeptidase-like 1 (NPEPL1), transcript variant 1, mRNA [NM_024663]	-0.86963195	0.0057647754
A_23_P93269	*TMEM151B*	Homo sapiens transmembrane protein 151B (TMEM151B), mRNA [NM_001137560]	1.0085847	0.0056355232
A_23_P85598	*RBM28*	Homo sapiens RNA binding motif protein 28 (RBM28), transcript variant 1, mRNA [NM_018077]	-0.52951336	0.011118167
A_24_P376339	*ZNF165*	Homo sapiens zinc finger protein 165 (ZNF165), mRNA [NM_003447]	-1.1566818	0.014173936
A_23_P7397	*ADCK3*	Homo sapiens aarF domain containing kinase 3 (ADCK3), mRNA [NM_020247]	-0.9890415	0.01210552
A_33_P3322499	*CCNL2*	Homo sapiens cyclin L2 (CCNL2), transcript variant 2, mRNA [NM_001039577]	-0.61436915	0.014025692
A_23_P170857	*PCDHB10*	Homo sapiens protocadherin beta 10 (PCDHB10), mRNA [NM_018930]	1.0260689	0.010859876
A_22_P00010745	*ZNF833P*	Homo sapiens zinc finger protein 833, pseudogene (ZNF833P), non-coding RNA [NR_028594]	2.5049942	0.0057262382
A_33_P3361202	*IL1RAP*	Homo sapiens interleukin 1 receptor accessory protein (IL1RAP), transcript variant 1, mRNA [NM_002182]	-0.5896593	0.014359909
A_23_P124122	*lnc-NIPSNAP3B-1*	Homo sapiens cDNA, FLJ18487. [AK311445]	-0.5295075	0.0047719665
A_33_P3252286	*CCSAP*	Homo sapiens centriole, cilia and spindle-associated protein (CCSAP), mRNA [NM_145257]	-0.6521339	0.008883942

A_23_P20363	*PXMP2*	Homo sapiens peroxisomal membrane protein 2, 22kDa (PXMP2), mRNA [NM_018663]	-0.7334487	0.0032831472
A_23_P34433	*CRLF1*	Homo sapiens cytokine receptor-like factor 1 (CRLF1), mRNA [NM_004750]	-2.1023924	0.0029355595
A_23_P29384	*ZC2HC1A*	Homo sapiens zinc finger, C2HC-type containing 1A (ZC2HC1A), mRNA [NM_016010]	-1.0426272	0.003557021
A_19_P00813593	*ZCCHC11*	Homo sapiens zinc finger, CCHC domain containing 11 (ZCCHC11), transcript variant 1, mRNA [NM_001009881]	-0.57594967	0.0037856582
A_23_P159741	*ZNF502*	Homo sapiens zinc finger protein 502 (ZNF502), transcript variant 1, mRNA [NM_033210]	-0.7023896	0.01109159
A_23_P38167	*LINC01116*	Homo sapiens long intergenic non-protein coding RNA 1116 (LINC01116), long non-coding RNA [NR_040001]	-0.77299666	0.015502214
A_33_P3397658	*BCOR*	Homo sapiens BCL6 corepressor (BCOR), transcript variant 1, mRNA [NM_017745]	-0.36296836	0.014260212
A_22_P00010804	*GPRC5C*	Homo sapiens G protein-coupled receptor, class C, group 5, member C (GPRC5C), transcript variant 1, mRNA [NM_022036]	1.7683815	0.002419416
A_23_P983	*SYNPO*	synaptopodin [Source:HGNC Symbol;Acc:HGNC:30672] [ENST00000394243]	0.56737405	0.0027262743
A_24_P113824	*lnc-NME3-1*	LNCipedia lincRNA (lnc-NME3-1), lincRNA [lnc-NME3-1:1]	-0.51952094	0.009801979
A_23_P345361	*PRDX6*	Homo sapiens peroxiredoxin 6 (PRDX6), mRNA [NM_004905]	-0.541497	0.015289479
A_33_P3219398	*TMEM50A*	Homo sapiens transmembrane protein 50A (TMEM50A), mRNA [NM_014313]	-0.9971506	0.010583646
A_33_P3361681	*C18orf25*	Homo sapiens chromosome 18 open reading frame 25 (C18orf25), transcript variant 1, mRNA [NM_145055]	-0.52350956	0.013411169
A_23_P51487	*ZNF521*	Homo sapiens zinc finger protein 521 (ZNF521), mRNA [NM_015461]	1.3172821	0.010735533
A_23_P60376	*VMA21*	Homo sapiens VMA21 vacuolar H+-ATPase homolog (S. cerevisiae) (VMA21), mRNA [NM_001017980]	-0.48199683	0.007740612
A_33_P3272160	*GBP3*	Homo sapiens guanylate binding protein 3 (GBP3), mRNA [NM_018284]	-1.0380204	0.0041847117
A_21_P0012276	*EDF1*	Homo sapiens endothelial differentiation-related factor 1 (EDF1), transcript variant beta, mRNA [NM_153200]	-0.35695148	0.010782863
A_33_P3403044	*REXO4*	Homo sapiens REX4, RNA exonuclease 4 homolog (S. cerevisiae) (REXO4), transcript variant 1, mRNA [NM_020385]	-0.32144135	0.011575981
A_24_P409857	*SLC25A15*	Homo sapiens solute carrier family 25 (mitochondrial carrier; ornithine transporter) member 15 (SLC25A15), mRNA [NM_014252]	-0.7767838	0.0065142275

A_23_P379614	*MAT2A*	Homo sapiens methionine adenosyltransferase II, alpha (MAT2A), mRNA [NM_005911]	1.3951104	0.005695989
A_24_P860703	*HMGN1*	Homo sapiens high mobility group nucleosome binding domain 1 (HMGN1), mRNA [NM_004965]	-0.6709838	0.0030359412
A_22_P00023949	*OIP5*	Homo sapiens Opa interacting protein 5 (OIP5), mRNA [NM_007280]	-0.7455306	0.0058944006
A_33_P3272563	*SNHG17*	Homo sapiens small nucleolar RNA host gene 17 (non-protein coding) (SNHG17), transcript variant 1, long non-coding RNA [NR_015366]	-0.9786572	0.012965681
A_32_P122793	*RUVBL1-AS1*	Homo sapiens RUVBL1 antisense RNA 1 (RUVBL1-AS1), long non-coding RNA [NR_046645]	0.48414212	0.013039871
A_33_P3319640	*NMT2*	Homo sapiens N-myristoyltransferase 2 (NMT2), mRNA [NM_004808]	-0.85504884	0.01571288
A_23_P133629	*MORC2-AS1*	Homo sapiens MORC2 antisense RNA 1 (MORC2-AS1), long non-coding RNA [NR_026920]	-0.86688244	0.009724618
A_23_P14458	*HDGFRP2*	Hepatoma-derived growth factor-related protein 2 [Source:UniProtKB/Swiss-Prot;Acc:Q7Z4V5] [ENST00000615225]	2.426725	0.005920916
A_21_P0011016	*CDC23*	Homo sapiens cell division cycle 23 (CDC23), mRNA [NM_004661]	-1.0907459	0.006924551
A_23_P105592	*L3HYPDH*	Homo sapiens L-3-hydroxyproline dehydratase (trans-) (L3HYPDH), mRNA [NM_144581]	-0.37623072	0.005428736
A_33_P3347330	*MVK*	Homo sapiens mevalonate kinase (MVK), transcript variant 1, mRNA [NM_000431]	-0.5510029	0.0027248112
A_21_P0013574	*lnc-SLC2A9-1*	LNCipedia lincRNA (lnc-SLC2A9-1), lincRNA [lnc-SLC2A9-1:1]	-0.80940485	0.014349793
A_19_P00812250	*TOMM7*	Homo sapiens translocase of outer mitochondrial membrane 7 homolog (yeast) (TOMM7), mRNA [NM_019059]	-1.016752	0.0044240346
A_24_P149902	*MTHFD1L*	Homo sapiens methylenetetrahydrofolate dehydrogenase (NADP+ dependent) 1-like (MTHFD1L), transcript variant 1, mRNA [NM_001242767]	-0.8539629	0.007501246
A_22_P00022845	*SMAD4*	Homo sapiens SMAD family member 4 (SMAD4), mRNA [NM_005359]	-0.89621663	0.011212511
A_23_P165402	*SUFU*	Homo sapiens suppressor of fused homolog (Drosophila) (SUFU), transcript variant 1, mRNA [NM_016169]	0.7188429	0.012773474
A_21_P0009075	*lnc-RPS27L-1*	BX373851 Homo sapiens PLACENTA COT 25-NORMALIZED Homo sapiens cDNA clone CS0DI086YN04 3-PRIME, mRNA sequence [BX373851]	1.7130036	0.0096269585
A_33_P3332627	*SF3B6*	Homo sapiens splicing factor 3b, subunit 6, 14kDa (SF3B6), mRNA [NM_016047]	-0.977556	0.0024890031

A_23_P392470	*lnc-ZDHHC7-1*	LNCipedia lincRNA (lnc-ZDHHC7-1), lincRNA [lnc-ZDHHC7-1:2]	1.1828603	0.005758863
A_23_P123071	*NR3C2*	Homo sapiens nuclear receptor subfamily 3, group C, member 2 (NR3C2), transcript variant 1, mRNA [NM_000901]	-0.9671561	0.014779506
A_33_P3416574	*COMMD10*	Homo sapiens COMM domain containing 10 (COMMD10), mRNA [NM_016144]	-0.9700012	0.0059573688
A_21_P0000436	*CAV2*	Homo sapiens caveolin 2 (CAV2), transcript variant 1, mRNA [NM_001233]	-0.91862345	0.010706546
A_23_P61531	*AIG1*	Homo sapiens androgen-induced 1 (AIG1), transcript variant 1, mRNA [NM_016108]	-1.1759179	0.0056601833
A_21_P0009771	*SNORD113-9*	Homo sapiens small nucleolar RNA, C/D box 113-9 (SNORD113-9), small nucleolar RNA [NR_003237]	0.83310837	0.010271231
A_23_P251232	*GLB1*	Homo sapiens galactosidase, beta 1 (GLB1), transcript variant 1, mRNA [NM_000404]	-0.91410685	0.011087365
A_23_P29594	*lnc-IGFL3-1*	LNCipedia lincRNA (lnc-IGFL3-1), lincRNA [lnc-IGFL3-1:1]	-1.3714033	0.010495637
A_23_P79942	*TTTY14*	Homo sapiens testis-specific transcript, Y-linked 14 (non-protein coding) (TTTY14), transcript variant 5, long non-coding RNA [NR_001543]	-1.2144703	0.010137935
A_33_P3233645	*RPL39L*	Homo sapiens ribosomal protein L39-like (RPL39L), mRNA [NM_052969]	-0.662523	0.011276983
A_33_P3304893	*PANK2*	Homo sapiens pantothenate kinase 2 (PANK2), transcript variant 1, mRNA [NM_153638]	-0.6988549	0.0042032786
A_22_P00015122	*MT1G*	Homo sapiens metallothionein 1G (MT1G), transcript variant 2, mRNA [NM_001301267]	4.2459345	0.0035894548
A_33_P3396389	*lnc-SNX20-5*	BX109892 Soares_testis_NHT Homo sapiens cDNA clone IMAGp998M071789, mRNA sequence [BX109892]	0.6202121	0.0044240346
A_23_P160503	*SNHG12*	Homo sapiens small nucleolar RNA host gene 12 (non-protein coding) (SNHG12), long non-coding RNA [NR_024127]	-1.0329225	0.008564741
A_23_P112251	*IL1R1*	Homo sapiens interleukin 1 receptor, type I (IL1R1), transcript variant 1, mRNA [NM_000877]	-0.7677474	0.0074246256
A_24_P341677	*GLRX2*	Homo sapiens glutaredoxin 2 (GLRX2), transcript variant 1, mRNA [NM_016066]	-0.6813224	0.007103236
A_21_P0008517	*GNG10*	Homo sapiens guanine nucleotide binding protein (G protein), gamma 10 (GNG10), transcript variant 1, mRNA [NM_001017998]	-0.7911884	0.0033199077
A_24_P25080	*LOC100506476*	Homo sapiens uncharacterized LOC100506476 (LOC100506476), long non-coding RNA [NR_109995]	-2.645844	0.003667498
A_24_P7584	*CBWD5*	Homo sapiens COBW domain containing 5 (CBWD5), transcript variant 1, mRNA [NM_001024916]	-0.81586266	0.0027709126

A_33_P3333156	*ILF3-AS1*	Homo sapiens ILF3 antisense RNA 1 (head to head) (ILF3-AS1), long non-coding RNA [NR_024333]	0.8002589	0.010272991
A_24_P28619	*LY6G5C*	Homo sapiens lymphocyte antigen 6 complex, locus G5C (LY6G5C), mRNA [NM_025262]	1.2549366	0.010004913
A_24_P146670	*C11orf70*	Homo sapiens chromosome 11 open reading frame 70 (C11orf70), transcript variant 2, mRNA [NM_001195005]	-1.8430113	0.0027248112
A_33_P3409159	*ANGEL2*	Homo sapiens angel homolog 2 (Drosophila) (ANGEL2), transcript variant 1, mRNA [NM_144567]	-0.4672906	0.014834527
A_23_P61778	*SLK*	Homo sapiens STE20-like kinase (SLK), mRNA [NM_014720]	-0.2994814	0.015044886
A_23_P124417	*SLC22A23*	Homo sapiens solute carrier family 22, member 23 (SLC22A23), transcript variant 1, mRNA [NM_015482]	-2.2342896	0.0025001846
A_33_P3296682	*NXN*	Homo sapiens nucleoredoxin (NXN), transcript variant 1, mRNA [NM_022463]	1.127157	0.006812377
A_23_P19590	*BUB1*	Homo sapiens BUB1 mitotic checkpoint serine/threonine kinase (BUB1), transcript variant 1, mRNA [NM_004336]	-0.48431438	0.008357089
A_23_P65022	*NDUFB5*	Homo sapiens NADH dehydrogenase (ubiquinone) 1 beta subcomplex, 5, 16kDa (NDUFB5), transcript variant 1, mRNA [NM_002492]	-0.7447662	0.0052760695
A_23_P152066	*EZR*	Homo sapiens ezrin (EZR), transcript variant 1, mRNA [NM_003379]	-0.5385077	0.01132647
A_21_P0013042	*ACADS*	Homo sapiens acyl-CoA dehydrogenase, C-2 to C-3 short chain (ACADS), transcript variant 1, mRNA [NM_000017]	0.6817724	0.008251486
A_33_P3326922	*UBR1*	Homo sapiens ubiquitin protein ligase E3 component n-recognin 1 (UBR1), mRNA [NM_174916]	-0.88198423	0.005255513
A_33_P3369631	*XLOC_l2_012748*	BROAD Institute lincRNA (XLOC_l2_012748), lincRNA [TCONS_l2_00024457]	1.600537	0.0056962976
A_22_P00015400	*EIF3J*	Homo sapiens eukaryotic translation initiation factor 3, subunit J (EIF3J), transcript variant 1, mRNA [NM_003758]	-0.5527997	0.011171237
A_32_P47554	*ZNF880*	zinc finger protein 880 [Source:HGNC Symbol;Acc:HGNC:37249] [ENST00000344085]	-0.78957343	0.006514206
A_24_P945194	*LOC102723834*	PREDICTED: Homo sapiens uncharacterized LOC102723834 (LOC102723834), ncRNA [XR_425103]	-1.3225675	0.0076398156
A_33_P3374563	*HINT1*	Homo sapiens histidine triad nucleotide binding protein 1 (HINT1), transcript variant 1, mRNA [NM_005340]	-0.497479	0.011673419

A_24_P401637	*PDCD6IP*	Homo sapiens programmed cell death 6 interacting protein (PDCD6IP), transcript variant 1, mRNA [NM_013374]	-0.7955942	0.00477571
A_33_P3265030	*SPATA7*	Homo sapiens spermatogenesis associated 7 (SPATA7), transcript variant 1, mRNA [NM_018418]	-1.0744328	0.004834548
A_24_P354337	*LOC100130856*	Homo sapiens cDNA FLJ38408 fis, clone FEBRA2009029. [AK095727]	-0.62972856	0.016260965
A_33_P3337977	*GP1BB*	Homo sapiens glycoprotein Ib (platelet), beta polypeptide (GP1BB), mRNA [NM_000407]	-1.4362755	0.0061486606
A_33_P3294053	*ARL6*	Homo sapiens ADP-ribosylation factor-like 6 (ARL6), transcript variant 1, mRNA [NM_032146]	-0.6455133	0.0098649
A_23_P259521	*FIG4*	FIG4 phosphoinositide 5-phosphatase [Source:HGNC Symbol;Acc:HGNC:16873] [ENST00000368941]	-0.7766225	0.013327314
A_33_P3409099	*ANKRD39*	Homo sapiens ankyrin repeat domain 39 (ANKRD39), mRNA [NM_016466]	-0.47892472	0.017022476
A_33_P3407746	*WDR41*	Homo sapiens WD repeat domain 41 (WDR41), mRNA [NM_018268]	-0.6484199	0.006878128
A_23_P26094	*ATXN1*	Homo sapiens ataxin 1 (ATXN1), transcript variant 1, mRNA [NM_000332]	-1.5993588	0.0044319485
A_24_P143171	*PPM1B*	Homo sapiens protein phosphatase, Mg2+/Mn2+ dependent, 1B (PPM1B), transcript variant 2, mRNA [NM_177968]	-0.79998916	0.010074285
A_24_P382253	*SLTM*	Homo sapiens SAFB-like, transcription modulator (SLTM), transcript variant 1, mRNA [NM_024755]	-0.50944847	0.008357089
A_21_P0005597	*TMEM47*	Homo sapiens transmembrane protein 47 (TMEM47), mRNA [NM_031442]	-2.1421294	0.0028703364
A_24_P261005	*RPRD1A*	Homo sapiens regulation of nuclear pre-mRNA domain containing 1A (RPRD1A), transcript variant 1, mRNA [NM_018170]	-0.88051647	0.009952643
A_22_P00003913	*TWIST1*	PREDICTED: Homo sapiens twist family bHLH transcription factor 1 (TWIST1), transcript variant X2, misc_RNA [XR_428085]	-1.9655756	0.0041868715
A_23_P257131	*SMIM19*	Homo sapiens small integral membrane protein 19 (SMIM19), transcript variant 3, mRNA [NM_138436]	-1.5035315	0.0044240346
A_33_P3333982	*lnc-CERS5-1*	Homo sapiens mRNA; cDNA DKFZp686E22244 (from clone DKFZp686E22244). [BX647194]	-2.5064778	0.002909564
A_24_P107291	*PEX13*	Homo sapiens peroxisomal biogenesis factor 13 (PEX13), mRNA [NM_002618]	-0.50794536	0.016187608
A_19_P00319225	*PDE4DIP*	Homo sapiens phosphodiesterase 4D interacting protein (PDE4DIP), transcript variant 3, mRNA [NM_022359]	-1.3102305	0.0049456456

A_23_P141035	*PPP2R1B*	Homo sapiens protein phosphatase 2, regulatory subunit A, beta (PPP2R1B), transcript variant 2, mRNA [NM_181699]	0.5858178	0.0066128806
A_33_P3227284	*ZNF300P1*	Homo sapiens zinc finger protein 300 pseudogene 1 (functional) (ZNF300P1), non-coding RNA [NR_026867]	-1.3669934	0.004581666
A_22_P00018968	*CHST4*	Homo sapiens carbohydrate (N-acetylglucosamine 6-O) sulfotransferase 4 (CHST4), transcript variant 1, mRNA [NM_005769]	0.35603252	0.006302727
A_24_P81841	*OGT*	Homo sapiens O-linked N-acetylglucosamine (GlcNAc) transferase (OGT), transcript variant 1, mRNA [NM_181672]	-0.9587478	0.007797447
A_23_P81212	*lnc-TMEM106C-2*	Homo sapiens cDNA FLJ26083 fis, clone RCT04690. [AK129594]	1.2741234	0.0043846127
A_33_P3294608	*CDKN1B*	Homo sapiens cyclin-dependent kinase inhibitor 1B (p27, Kip1) (CDKN1B), mRNA [NM_004064]	-1.5089462	0.0027248112
A_23_P79803	*MRPS18C*	Homo sapiens mitochondrial ribosomal protein S18C (MRPS18C), transcript variant 1, mRNA [NM_016067]	-0.59183025	0.008061535
A_33_P3229672	*MVP*	Homo sapiens major vault protein (MVP), transcript variant 1, mRNA [NM_017458]	-0.4463401	0.012157646
A_24_P566701	*VSTM2L*	Homo sapiens V-set and transmembrane domain containing 2 like (VSTM2L), mRNA [NM_080607]	-21864953	0.007794005
A_21_P0000737	*SKP1*	Homo sapiens S-phase kinase-associated protein 1 (SKP1), transcript variant 2, mRNA [NM_170679]	-0.9955888	0.0031941293
A_23_P74088	*BGN*	Homo sapiens biglycan (BGN), mRNA [NM_001711]	10011927	0.0040017776
A_23_P97296	*ZNF571-AS1*	Homo sapiens ZNF571 antisense RNA 1 (ZNF571-AS1), transcript variant 1, long non-coding RNA [NR_038247]	-1.1503801	0.007225359
A_23_P200560	*MMP23B*	Homo sapiens matrix metallopeptidase 23B (MMP23B), mRNA [NM_006983]	2.2852638	0.006532576
A_22_P00014722	*NECAP2*	Homo sapiens NECAP endocytosis associated 2 (NECAP2), transcript variant 1, mRNA [NM_018090]	-0.895391	0.0054447073
A_23_P255328	*CDC42*	Homo sapiens cell division cycle 42 (CDC42), transcript variant 3, mRNA [NM_001039802]	-0.8925705	0.0043846127
A_33_P3237784	*TARID*	Homo sapiens TCF21 antisense RNA inducing promoter demethylation (TARID), long non-coding RNA [NR_109982]	2.2571764	0.004026994
A_23_P420293	*PRDM8*	Homo sapiens PR domain containing 8 (PRDM8), transcript variant 1, mRNA [NM_020226]	1.0414479	0.013777289

A_23_P389987	*PORCN*	Homo sapiens porcupine homolog (Drosophila) (PORCN), transcript variant F, mRNA [NM_001282167]	-0.9882755	0.008636949
A_33_P6819918	*C11orf45*	Homo sapiens chromosome 11 open reading frame 45 (C11orf45), transcript variant 2, mRNA [NM_145013]	-1.1879354	0.007304854
A_33_P3330109	*TLX2*	Homo sapiens T-cell leukemia homeobox 2 (TLX2), mRNA [NM_016170]	-2.6186485	0.0035432973
A_23_P126752	*LOC100506844*	Homo sapiens uncharacterized LOC100506844 (LOC100506844), long non-coding RNA [NR_038269]	-0.8437047	0.0070571043
A_33_P3303015	*HLA-C*	Homo sapiens major histocompatibility complex, class I, C (HLA-C), transcript variant 2, mRNA [NM_001243042]	-1.7573612	0.0044240346
A_21_P0000219	*CAPZB*	Homo sapiens capping protein (actin filament) muscle Z-line, beta (CAPZB), transcript variant 1, mRNA [NM_004930]	0.29351878	0.008786314
A_22_P00005685	*RPS9*	Homo sapiens ribosomal protein S9 (RPS9), mRNA [NM_001013]	-0.3350156	0.008967493
A_21_P0000173	*SNORD34*	Homo sapiens small nucleolar RNA, C/D box 34 (SNORD34), small nucleolar RNA [NR_000019]	1.270253	0.008080026
A_33_P3289422	*lnc-ELAC2-1*	LNCipedia lincRNA (lnc-ELAC2-1), lincRNA [lnc-ELAC2-1:1]	0.66471875	0.016966365
A_23_P62634	*C5orf56*	Homo sapiens chromosome 5 open reading frame 56 (C5orf56), long non-coding RNA [NR_045116]	-1.2681546	0.0039728633
A_33_P3533001	*ZNF765*	Homo sapiens zinc finger protein 765 (ZNF765), mRNA [NM_001040185]	-0.67838854	0.015035079
A_21_P0000035	*RHCE*	Homo sapiens Rh blood group, CcEe antigens (RHCE), transcript variant 1, mRNA [NM_020485]	-1.3190978	0.015734134
A_23_P213518	*UBE2E4P*	AL568675 Homo sapiens PLACENTA Homo sapiens cDNA clone CS0DE004YG14 3-PRIME, mRNA sequence [AL568675]	1.4427022	0.004588558
A_33_P3342126	*TMEM217*	Homo sapiens transmembrane protein 217 (TMEM217), transcript variant 2, mRNA [NM_001162900]	-0.5317729	0.011845045
A_24_P106953	*CAST*	Homo sapiens calpastatin (CAST), transcript variant 6, mRNA [NM_001042440]	-0.80558467	0.0051253513
A_23_P26184	*PRDM8*	Homo sapiens PR domain containing 8 (PRDM8), transcript variant 1, mRNA [NM_020226]	1.2175646	0.005800329
A_23_P102351	*PTGES2*	Homo sapiens prostaglandin E synthase 2 (PTGES2), transcript variant 1, mRNA [NM_025072]	1.0210468	0.012610269
A_21_P0008629	*DET1*	Homo sapiens de-etiolated homolog 1 (Arabidopsis) (DET1), transcript variant 1, mRNA [NM_017996]	-1.1116134	0.007103236

A_33_P3241482	*CHST10*	Homo sapiens carbohydrate sulfotransferase 10 (CHST10), mRNA [NM_004854]	0.9545933	0.012911957
A_23_P409093	*lnc-CTDSPL2-1*	LNCipedia lincRNA (lnc-CTDSPL2-1), lincRNA [lnc-CTDSPL2-1:1]	0.5488621	0.006395745
A_33_P3372924	*ZNF346*	Homo sapiens zinc finger protein 346 (ZNF346), mRNA [NM_012279]	-0.46329898	0.015417507
A_22_P00021593	*ANO4*	Homo sapiens anoctamin 4 (ANO4), transcript variant 3, mRNA [NM_178826]	-2.139639	0.003365343
A_33_P3312682	*ADCK3*	aarF domain containing kinase 3 [Source:HGNC Symbol;Acc:HGNC:16812] [ENST00000366779]	0.960232	0.009367357
A_32_P175301	*lnc-C12orf66-3*	BX089237 Soares_NFL_T_GBC_S1 Homo sapiens cDNA clone IMAGp998K033715 ; IMAGE:1468922, mRNA sequence [BX089237]	0.58582217	0.015414173
A_23_P39766	*REXO1*	Homo sapiens REX1, RNA exonuclease 1 homolog (S. cerevisiae) (REXO1), mRNA [NM_020695]	1.0296836	0.0050508454
A_33_P3270109	*DENND3*	Homo sapiens DENN/MADD domain containing 3 (DENND3), mRNA [NM_014957]	-1.0171512	0.011575981
A_23_P60028	*GLS*	Homo sapiens glutaminase (GLS), transcript variant 1, mRNA [NM_014905]	-2.8607574	0.0020435157
A_33_P3371954	*LEO1*	Homo sapiens Leo1, Paf1/RNA polymerase II complex component, homolog (S. cerevisiae) (LEO1), transcript variant 2, mRNA [NM_001286430]	-0.6540744	0.007797447
A_23_P167227	*TCEB1*	Homo sapiens transcription elongation factor B (SIII), polypeptide 1 (15kDa, elongin C) (TCEB1), transcript variant 1, mRNA [NM_005648]	-0.55251783	0.016256094
A_33_P3228558	*IGSF8*	Homo sapiens immunoglobulin superfamily, member 8 (IGSF8), transcript variant 1, mRNA [NM_052868]	1.1390983	0.0054341624
A_33_P3258452	*HADH*	Homo sapiens hydroxyacyl-CoA dehydrogenase (HADH), transcript variant 2, mRNA [NM_005327]	-1.1922584	0.007312003
A_32_P42964	*ARHGAP27*	Homo sapiens Rho GTPase activating protein 27 (ARHGAP27), transcript variant 4, mRNA [NM_001282290]	-0.33659086	0.016892996
A_23_P119143	*C12orf5*	Homo sapiens chromosome 12 open reading frame 5 (C12orf5), mRNA [NM_020375]	-1.1962873	0.0035186775
A_21_P0013201	*NUPR1L*	Homo sapiens nuclear protein, transcriptional regulator, 1-like (NUPR1L), mRNA [NM_001145712]	-0.88698876	0.011313176
A_23_P159663	*ICAM5*	Homo sapiens intercellular adhesion molecule 5, telencephalin (ICAM5), mRNA [NM_003259]	2.785713	0.0051161773
A_22_P00008583	*UXT*	Homo sapiens ubiquitously-expressed, prefoldin-like chaperone (UXT), transcript variant 2, mRNA [NM_004182]	-0.9252061	0.010871661

A_23_P143127	*DNAAF2*	Homo sapiens dynein, axonemal, assembly factor 2 (DNAAF2), transcript variant 1, mRNA [NM_018139]	-0.69167566	0.007286966
A_23_P47058	*lnc-KCTD10-1*	LNCipedia lincRNA (lnc-KCTD10-1), lincRNA [lnc-KCTD10-1:1]	-1.026553	0.014038121
A_33_P3256858	*EML4*	echinoderm microtubule associated protein like 4 [Source:HGNC Symbol;Acc:HGNC:1316] [ENST00000318522]	-0.65590507	0.005612091
A_23_P147729	*CUZD1*	Homo sapiens CUB and zona pellucida-like domains 1 (CUZD1), transcript variant 1, mRNA [NM_022034]	-1.3322763	0.0043581175
A_23_P48237	*C14orf80*	Homo sapiens chromosome 14 open reading frame 80 (C14orf80), transcript variant 1, mRNA [NM_001134875]	-0.4316556	0.014552402
A_19_P00322743	*SLC35E3*	Homo sapiens solute carrier family 35, member E3 (SLC35E3), mRNA [NM_018656]	-0.97843266	0.01571288
A_22_P00009735	*MCRS1*	Homo sapiens microspherule protein 1 (MCRS1), transcript variant 1, mRNA [NM_006337]	0.71243	0.011383506
A_23_P258002	*lnc-SLC30A7-2*	Homo sapiens cDNA FLJ11489 fis, clone HEMBA1001915. [AK021551]	-1.0498744	0.012873199
A_33_P3544856	*BTBD9*	Homo sapiens BTB (POZ) domain containing 9 (BTBD9), transcript variant 1, mRNA [NM_052893]	-0.6115132	0.010000725
A_33_P3392000	*CDKN2AIP*	Homo sapiens CDKN2A interacting protein (CDKN2AIP), mRNA [NM_017632]	-0.63127685	0.012452464
A_19_P00315869	*lnc-ERC1-1*	Homo sapiens cDNA FLJ33297 fis, clone BNGH42001406. [AK090616]	0.78454256	0.015754001
A_33_P3283206	*LINC00963*	Homo sapiens long intergenic non-protein coding RNA 963 (LINC00963), long non-coding RNA [NR_038955]	2.91263	0.0038302224
A_33_P3265739	*lnc-DET1-1*	Homo sapiens cDNA FLJ33447 fis, clone BRAMY1000098. [AK090766]	1.561059	0.003609745
A_23_P56288	*LOC102723979*	PREDICTED: Homo sapiens uncharacterized LOC102723979 (LOC102723979), ncRNA [XR_426452]	0.34120837	0.008510122
A_21_P0006370	*PTGER3*	Homo sapiens prostaglandin E receptor 3 (subtype EP3) (PTGER3), transcript variant 9, mRNA [NM_198719]	1.2108437	0.005092817
A_33_P3291329	*LENG8*	Homo sapiens leukocyte receptor cluster (LRC) member 8 (LENG8), mRNA [NM_052925]	0.71470165	0.011479118
A_22_P00013902	*lnc-RABGAP1-1*	Q86TS6_HUMAN (Q86TS6) Full-length cDNA 5-PRIME end of clone CS0CAP004YO05 of Thymus of Homo sapiens (human) (Fragment), partial (13%) [THC2506250]	-0.74534065	0.014349793
A_21_P0004980	*lnc-RPS24-2*	LNCipedia lincRNA (lnc-RPS24-2), lincRNA [lnc-RPS24-2:1]	0.70182854	0.005092817

A_32_P91250	*FGF2*	Homo sapiens fibroblast growth factor 2 (basic) (FGF2), mRNA [NM_002006]	-1.4313614	0.004359316
A_23_P93881	*UBE2L3*	Homo sapiens ubiquitin-conjugating enzyme E2L 3 (UBE2L3), transcript variant 1, mRNA [NM_003347]	-0.54899955	0.009841122
A_22_P00017709	*F8*	Homo sapiens coagulation factor VIII, procoagulant component (F8), transcript variant 1, mRNA [NM_000132]	-1.0979118	0.005348777
A_33_P3246083	*SYPL1*	Homo sapiens synaptophysin-like 1 (SYPL1), transcript variant 2, mRNA [NM_182715]	-0.83332556	0.0044864463
A_24_P356373	*LOC101929172*	Homo sapiens uncharacterized LOC101929172 (LOC101929172), long non-coding RNA [NR_104677]	0.5272505	0.007974341
A_21_P0011841	*OXSM*	Homo sapiens 3-oxoacyl-ACP synthase, mitochondrial (OXSM), transcript variant 1, mRNA [NM_017897]	-1.183358	0.0059050764
A_33_P3362861	*HAGHL*	Homo sapiens hydroxyacylglutathione hydrolase-like (HAGHL), transcript variant 2, mRNA [NM_032304]	1.30864	0.011182366
A_22_P00004471	*PAFAH1B1*	Homo sapiens platelet-activating factor acetylhydrolase 1b, regulatory subunit 1 (45kDa) (PAFAH1B1), mRNA [NM_000430]	-0.5283408	0.016691668
A_32_P68504	*ZFP30*	Homo sapiens ZFP30 zinc finger protein (ZFP30), mRNA [NM_014898]	-0.7254634	0.012737636
A_33_P3237135	*ZDBF2*	Homo sapiens zinc finger, DBF-type containing 2 (ZDBF2), transcript variant 1, mRNA [NM_020923]	-0.9989881	0.0077551813
A_24_P633902	*FEZ2*	Homo sapiens fasciculation and elongation protein zeta 2 (zygin II) (FEZ2), transcript variant 2, mRNA [NM_001042548]	-1.5008233	0.002419416
A_32_P152767	*MMP2*	Homo sapiens matrix metallopeptidase 2 (gelatinase A, 72kDa gelatinase, 72kDa type IV collagenase) (MMP2), transcript variant 1, mRNA [NM_004530]	-0.84354067	0.014244717
A_33_P3258923	*RNF115*	Homo sapiens ring finger protein 115 (RNF115), mRNA [NM_014455]	-1.0487894	0.012362548
A_23_P27147	*SKIDA1*	Homo sapiens SKI/DACH domain containing 1 (SKIDA1), mRNA [NM_207371]	-0.78112704	0.01685728
A_21_P0000321	*MKRN3*	Homo sapiens makorin ring finger protein 3 (MKRN3), mRNA [NM_005664]	2.086085	0.0049917083
A_24_P145066	*ANAPC11*	Homo sapiens anaphase promoting complex subunit 11 (ANAPC11), transcript variant 1, mRNA [NM_001002244]	-0.69048667	0.0031941293
A_23_P135157	*SNORA31*	Homo sapiens small nucleolar RNA, H/ACA box 31 (SNORA31), small nucleolar RNA [NR_002967]	-0.52959436	0.008976597

A_33_P3412233	*KIF1B*	Homo sapiens kinesin family member 1B (KIF1B), transcript variant 2, mRNA [NM_183416]	-1.2118976	0.008972698
A_33_P3527721	*PLAA*	Homo sapiens phospholipase A2-activating protein (PLAA), mRNA [NM_001031689]	-0.6423855	0.01276926
A_33_P3243857	*POMZP3*	Homo sapiens POM121 and ZP3 fusion (POMZP3), transcript variant 2, mRNA [NM_152992]	1.0298578	0.007442623
A_33_P3399474	*LOC284219*	Homo sapiens cDNA FLJ37117 fis, clone BRACE2022270. [AK094436]	-1.529892	0.003850731
A_33_P3307363	*ADAM10*	Homo sapiens ADAM metallopeptidase domain 10 (ADAM10), mRNA [NM_001110]	-1.1298251	0.010066772
A_33_P3268564	*LPHN2*	Homo sapiens latrophilin 2 (LPHN2), transcript variant 3, mRNA [NM_001297705]	-3.6444092	0.0018723286
A_23_P161190	*lnc-N4BP2-1*	ALU6_HUMAN (P39193) Alu subfamily SP sequence contamination warning entry, partial (8%) [THC2533503]	-0.84630984	0.008437608
A_23_P130089	*NCK2*	Homo sapiens NCK adaptor protein 2 (NCK2), transcript variant 3, mRNA [NM_001004722]	-1.196964	0.006224155
A_33_P3236102	*VIM*	Homo sapiens vimentin (VIM), mRNA [NM_003380]	0.95640033	0.016633598
A_33_P3314902	*IFT20*	Homo sapiens intraflagellar transport 20 (IFT20), transcript variant 2, mRNA [NM_174887]	-1.1210597	0.0034684502
A_23_P502425	*IER5L*	Homo sapiens immediate early response 5-like (IER5L), mRNA [NM_203434]	-0.6325171	0.014345706
A_33_P3293668	*BIRC6*	Homo sapiens baculoviral IAP repeat containing 6 (BIRC6), mRNA [NM_016252]	-0.8387386	0.009172592
A_23_P394064	*MRPL47*	Homo sapiens mitochondrial ribosomal protein L47 (MRPL47), transcript variant 1, mRNA [NM_020409]	-0.6551716	0.005860685
A_33_P3362900	*DENND4C*	PREDICTED: Homo sapiens DENN/MADD domain containing 4C (DENND4C), transcript variant X1, mRNA [XM_006716804]	-0.95520115	0.0048340787
A_23_P346969	*PTRF*	Homo sapiens polymerase I and transcript release factor (PTRF), mRNA [NM_012232]	-0.43238208	0.004530034
A_23_P38795	*FAM228B*	Homo sapiens family with sequence similarity 228, member B (FAM228B), transcript variant 1, mRNA [NM_001145710]	-1.4094921	0.0063157394
A_23_P17144	*PIK3CB*	Homo sapiens phosphatidylinositol-4,5-bisphosphate 3-kinase, catalytic subunit beta (PIK3CB), transcript variant 1, mRNA [NM_006219]	0.20975082	0.011383506
A_33_P3334313	*FPR1*	Homo sapiens formyl peptide receptor 1 (FPR1), transcript variant 2, mRNA [NM_002029]	1.2820529	0.010062054
A_23_P14184	*GPN1*	Homo sapiens GPN-loop GTPase 1 (GPN1), transcript variant 1, mRNA [NM_007266]	-0.62659377	0.008437608

A_23_P360744	*ACTR3B*	Homo sapiens ARP3 actin-related protein 3 homolog B (yeast) (ACTR3B), transcript variant 1, mRNA [NM_020445]	-1.4508461	0.0055611515
A_23_P77714	*THSD1*	Homo sapiens thrombospondin, type I, domain containing 1 (THSD1), transcript variant 1, mRNA [NM_018676]	-1.6075552	0.0031941293
A_23_P10206	*RAG1*	Homo sapiens recombination activating gene 1 (RAG1), mRNA [NM_000448]	0.020526117	0.01154211
A_22_P00021588	*CLUAP1*	Homo sapiens clusterin associated protein 1 (CLUAP1), transcript variant 2, mRNA [NM_024793]	-1.0885558	0.0052045966
A_23_P215048	*HAS2*	Homo sapiens hyaluronan synthase 2 (HAS2), mRNA [NM_005328]	-0.8090067	0.012549863
A_33_P3385361	*LOC101930532*	PREDICTED: Homo sapiens uncharacterized LOC101930532 (LOC101930532), ncRNA [XR_248664]	0.47580504	0.0065142275
A_24_P14260	*KIAA0408*	Homo sapiens KIAA0408 (KIAA0408), mRNA [NM_014702]	-1.1390688	0.007206317
A_23_P327022	*NOL7*	Homo sapiens nucleolar protein 7, 27kDa (NOL7), mRNA [NM_016167]	-0.46624726	0.0062004877
A_19_P00802417	*CARD8*	Homo sapiens caspase recruitment domain family, member 8 (CARD8), transcript variant 2, mRNA [NM_014959]	-0.3869125	0.016251752
A_24_P227831	*MDFIC*	Homo sapiens MyoD family inhibitor domain containing (MDFIC), transcript variant 1, mRNA [NM_199072]	0.88227034	0.0051410845
A_23_P375549	*ABCC1*	Homo sapiens ATP-binding cassette, sub-family C (CFTR/MRP), member 1 (ABCC1), mRNA [NM_004996]	0.610065	0.013781849
A_23_P317800	*FUNDC1*	Homo sapiens FUN14 domain containing 1 (FUNDC1), mRNA [NM_173794]	-0.6627535	0.0029471128
A_23_P74663	*PEAK1*	Homo sapiens pseudopodium-enriched atypical kinase 1 (PEAK1), mRNA [NM_024776]	-0.83569324	0.005787274
A_33_P3413227	*ANAPC4*	Homo sapiens anaphase promoting complex subunit 4 (ANAPC4), transcript variant 2, mRNA [NM_013367]	-0.76629615	0.00856985
A_19_P00812135	*TAF1A*	Homo sapiens TATA box binding protein (TBP)-associated factor, RNA polymerase I, A, 48kDa (TAF1A), transcript variant 1, mRNA [NM_005681]	-0.75500095	0.0036556649
A_32_P46191	*LOC100132363*	Homo sapiens cDNA FLJ39193 fis, clone OCBBF2004866. [AK096512]	1.2999048	0.006970571
A_23_P1676	*LOC100130417*	Homo sapiens uncharacterized LOC100130417 (LOC100130417), transcript variant 1, long non-coding RNA [NR_026874]	1.2503018	0.012381021
A_32_P56143	*ZNF727*	Homo sapiens zinc finger protein 727 (ZNF727), mRNA [NM_001159522]	0.15382506	0.006814677

A_23_P60354	*TMEM218*	Homo sapiens transmembrane protein 218 (TMEM218), transcript variant 1, mRNA [NM_001258243]	-0.7649924	0.0022181747
A_24_P576174	*SMARCA2*	Homo sapiens SWI/SNF related, matrix associated, actin dependent regulator of chromatin, subfamily a, member 2 (SMARCA2), transcript variant 2, mRNA [NM_139045]	-1.0157294	0.0031941293
A_23_P24616	*CRNDE*	Homo sapiens colorectal neoplasia differentially expressed (non-protein coding) (CRNDE), transcript variant 4, long non-coding RNA [NR_110454]	-1.1512382	0.003381037
A_22_P00015751	*DCP1A*	Homo sapiens decapping mRNA 1A (DCP1A), transcript variant 3, mRNA [NM_001290205]	-1.0407405	0.013076766
A_24_P818268	*SIAE*	Homo sapiens sialic acid acetylesterase (SIAE), transcript variant 1, mRNA [NM_170601]	-0.8695386	0.007582027
A_21_P0014529	*lnc-TAF1C-1*	Homo sapiens SFPQ mRNA, complete cds, alternatively spliced. [DQ323994]	0.8063488	0.014696344
A_32_P115375	*RGS4*	Homo sapiens regulator of G-protein signaling 4 (RGS4), transcript variant 2, mRNA [NM_005613]	-2.3290486	0.0025001846
A_23_P250118	*SS18L1*	Homo sapiens synovial sarcoma translocation gene on chromosome 18-like 1 (SS18L1), transcript variant 1, mRNA [NM_198935]	-0.755636	0.0058047334
A_33_P3388865	*lnc-SMG6-1*	Synthetic construct Homo sapiens gateway clone IMAGE:100018904 3' read HNRPA1 mRNA. [CU677925]	1.2808833	0.002804883
A_33_P3325087	*HSPBAP1*	Homo sapiens HSPB (heat shock 27kDa) associated protein 1 (HSPBAP1), mRNA [NM_024610]	-0.76041275	0.011658678
A_24_P224998	*LRRC10*	leucine rich repeat containing 10 [Source:HGNC Symbol;Acc:HGNC:20264] [ENST00000361484]	0.69829357	0.014588932
A_23_P78734	*NSRP1*	Homo sapiens nuclear speckle splicing regulatory protein 1 (NSRP1), transcript variant 1, mRNA [NM_032141]	-0.4976651	0.0056888084
A_23_P132915	*MYH14*	Homo sapiens myosin, heavy chain 14, non-muscle (MYH14), transcript variant 1, mRNA [NM_001077186]	0.72051877	0.011357791
A_33_P3370555	*GPBP1*	Homo sapiens GC-rich promoter binding protein 1 (GPBP1), transcript variant 1, mRNA [NM_022913]	-0.6843927	0.005073928
A_33_P3271635	*FAM114A1*	Homo sapiens family with sequence similarity 114, member A1 (FAM114A1), transcript variant 1, mRNA [NM_138389]	-1.0686076	0.008403594
A_33_P3351566	*GALC*	galactosylceramidase [Source:HGNC Symbol;Acc:HGNC:4115] [ENST00000474294]	-1.4207968	0.009157587

A_23_P1962	*HLA-DPB1*	Homo sapiens major histocompatibility complex, class II, DP beta 1 (HLA-DPB1), mRNA [NM_002121]	-1.42762	0.007502627
A_23_P55256	*ETNK1*	Homo sapiens ethanolamine kinase 1 (ETNK1), transcript variant 2, mRNA [NM_001039481]	0.96487534	0.00590614
A_24_P46093	*RARRES3*	Homo sapiens retinoic acid receptor responder (tazarotene induced) 3 (RARRES3), mRNA [NM_004585]	-0.7466991	0.016148135
A_24_P65060	*ZNF652*	Homo sapiens zinc finger protein 652 (ZNF652), transcript variant 2, mRNA [NM_014897]	-1.1098486	0.0055521973
A_24_P204244	*SLC6A6*	Homo sapiens solute carrier family 6 (neurotransmitter transporter), member 6 (SLC6A6), transcript variant 1, mRNA [NM_003043]	-0.19425368	0.007058774
A_21_P0009797	*MEX3B*	Homo sapiens mex-3 RNA binding family member B (MEX3B), mRNA [NM_032246]	-2.173482	0.0031512622
A_33_P3361027	*ANXA2P1*	Homo sapiens annexin A2 pseudogene 1 (ANXA2P1), non-coding RNA [NR_001562]	-0.5052972	0.013460425
A_21_P0006358	*lnc-ZNF8-3*	LNCipedia lincRNA (lnc-ZNF8-3), lincRNA [lnc-ZNF8-3:1]	-0.34139273	0.0129454555
A_23_P98580	*TFIP11*	Homo sapiens tuftelin interacting protein 11 (TFIP11), transcript variant 1, mRNA [NM_001008697]	-0.8352077	0.009787606
A_23_P71241	*lnc-HIATL1-2*	LNCipedia lincRNA (lnc-HIATL1-2), lincRNA [lnc-HIATL1-2:2]	1.8249096	0.0029471128
A_24_P228637	*FADS2*	Homo sapiens fatty acid desaturase 2 (FADS2), transcript variant 1, mRNA [NM_004265]	2.2594063	0.0037656627
A_24_P565556	*SEC61G*	Homo sapiens Sec61 gamma subunit (SEC61G), transcript variant 1, mRNA [NM_014302]	-0.79531664	0.002419416
A_23_P204016	*SETD4*	Homo sapiens SET domain containing 4 (SETD4), transcript variant 2, mRNA [NM_001007259]	-0.8942194	0.007562944
A_23_P166779	*ALG1*	Homo sapiens ALG1, chitobiosyldiphosphodolichol beta-mannosyltransferase (ALG1), mRNA [NM_019109]	0.87603104	0.010971502
A_33_P3236441	*CACNB3*	Homo sapiens calcium channel, voltage-dependent, beta 3 subunit (CACNB3), transcript variant 1, mRNA [NM_000725]	1.0949285	0.0048260237
A_21_P0009420	*LINC00312*	Homo sapiens long intergenic non-protein coding RNA 312 (LINC00312), long non-coding RNA [NR_024065]	-3.1516924	0.0031750489
A_33_P3575854	*LDHAL6A*	Homo sapiens lactate dehydrogenase A-like 6A (LDHAL6A), transcript variant 1, mRNA [NM_144972]	1.3675755	0.008437608
A_33_P3240512	*lnc-TMEM105-2*	LNCipedia lincRNA (lnc-TMEM105-2), lincRNA [lnc-TMEM105-2:2]	-0.7317573	0.0102659585

A_24_P205019	*LOC642361*	Homo sapiens uncharacterized LOC642361 (LOC642361), long non-coding RNA [NR_029407]	-0.94307303	0.009690877
A_33_P3418025	*KCTD12*	Homo sapiens potassium channel tetramerization domain containing 12 (KCTD12), mRNA [NM_138444]	-0.80954164	0.009091813
A_22_P00015002	*ZNF250*	Homo sapiens zinc finger protein 250 (ZNF250), transcript variant 1, mRNA [NM_021061]	-1.2884312	0.0054295897
A_32_P165113	*CTSO*	Homo sapiens cathepsin O (CTSO), mRNA [NM_001334]	-1.7900953	0.003983638
A_23_P27215	*WDR92*	Homo sapiens WD repeat domain 92 (WDR92), transcript variant 1, mRNA [NM_138458]	-0.6707578	0.010519677
A_33_P3245160	*ARL17B*	Homo sapiens ADP-ribosylation factor-like 17B (ARL17B), transcript variant 1, mRNA [NM_001039083]	-0.6604253	0.008106991
A_33_P3274851	*UBB*	Homo sapiens ubiquitin B (UBB), transcript variant 1, mRNA [NM_018955]	0.39284605	0.009501149
A_23_P75288	*COX5B*	Homo sapiens cytochrome c oxidase subunit Vb (COX5B), mRNA [NM_001862]	-1.0651385	0.008087408
A_23_P216355	*RIN1*	Homo sapiens Ras and Rab interactor 1 (RIN1), mRNA [NM_004292]	0.55415845	0.0072350414
A_22_P00014155	*ATAD1*	Homo sapiens ATPase family, AAA domain containing 1 (ATAD1), mRNA [NM_032810]	-0.8510155	0.0020435157
A_24_P19752	*TONSL*	Homo sapiens tonsoku-like, DNA repair protein (TONSL), mRNA [NM_013432]	1.9258978	0.008339934
A_23_P132874	*PEX19*	Homo sapiens peroxisomal biogenesis factor 19 (PEX19), transcript variant 1, mRNA [NM_002857]	-0.57956856	0.015740072
A_23_P30338	*lnc-EMP3-1*	LNCipedia lincRNA (lnc-EMP3-1), lincRNA [lnc-EMP3-1:1]	0.93723345	0.008424041
A_23_P388812	*CMSS1*	Homo sapiens cms1 ribosomal small subunit homolog (yeast) (CMSS1), transcript variant 1, mRNA [NM_032359]	-0.54253316	0.0055054883
A_33_P3390778	*CCNH*	Homo sapiens cyclin H (CCNH), transcript variant 1, mRNA [NM_001239]	-0.62105685	0.0084265405
A_23_P157795	*CKAP2L*	cytoskeleton associated protein 2-like [Source:HGNC Symbol;Acc:HGNC:26877] [ENST00000435431]	-0.36748368	0.01041717
A_24_P128442	*TRIM46*	Homo sapiens tripartite motif containing 46 (TRIM46), transcript variant 2, mRNA [NM_001256599]	-1.2616707	0.010846277
A_23_P62890	*CTNNAL1*	Homo sapiens catenin (cadherin-associated protein), alpha-like 1 (CTNNAL1), transcript variant 1, mRNA [NM_003798]	-0.36533925	0.012953318
A_22_P00010274	*TBX15*	Homo sapiens T-box 15 (TBX15), mRNA [NM_152380]	-1.0904074	0.005057235

A_33_P3238856	*GBP1*	Homo sapiens guanylate binding protein 1, interferon-inducible (GBP1), mRNA [NM_002053]	-1.2224805	0.006203498
A_21_P0012336	*FARP1*	Homo sapiens FERM, RhoGEF (ARHGEF) and pleckstrin domain protein 1 (chondrocyte-derived) (FARP1), transcript variant 2, mRNA [NM_001001715]	-0.48675758	0.011969343
A_33_P3313889	*MYO1D*	Homo sapiens myosin ID (MYO1D), transcript variant 1, mRNA [NM_015194]	-1.5550202	0.008298705
A_22_P00003390	*GGTLC1*	Homo sapiens gamma-glutamyltransferase light chain 1 (GGTLC1), transcript variant A, mRNA [NM_178311]	-1.1590708	0.00904597
A_21_P0014904	*TOR1A*	Homo sapiens torsin family 1, member A (torsin A) (TOR1A), mRNA [NM_000113]	-0.58428645	0.01664532
A_23_P134204	*lnc-CCDC113-1*	LNCipedia lincRNA (lnc-CCDC113-1), lincRNA [lnc-CCDC113-1:1]	0.8650041	0.0041847117
A_33_P3246505	*FAM71F1*	Homo sapiens family with sequence similarity 71, member F1 (FAM71F1), transcript variant 1, mRNA [NM_032599]	-1.2098637	0.0052084886
A_33_P3228435	*TEP1*	Homo sapiens telomerase-associated protein 1 (TEP1), mRNA [NM_007110]	-1.7768945	0.005937971
A_24_P394368	*MAP3K8*	mitogen-activated protein kinase kinase kinase 8 [Source:HGNC Symbol;Acc:HGNC:6860] [ENST00000375322]	1.0702825	0.0028193828
A_23_P258570	*FXYD1*	Homo sapiens FXYD domain containing ion transport regulator 1 (FXYD1), transcript variant c, mRNA [NM_001278717]	0.8801962	0.0144334
A_33_P3279798	*WDR19*	Homo sapiens WD repeat domain 19 (WDR19), mRNA [NM_025132]	-0.84011406	0.008470881
A_23_P56213	*PSMD10*	Homo sapiens proteasome (prosome, macropain) 26S subunit, non-ATPase, 10 (PSMD10), transcript variant 1, mRNA [NM_002814]	-0.9013827	0.003190129
A_23_P203030	*ATP5S*	Homo sapiens ATP synthase, H+ transporting, mitochondrial Fo complex, subunit s (factor B) (ATP5S), transcript variant 3, mRNA [NM_015684]	-1.0045104	0.008032271
A_32_P98975	*GRAMD1A*	Homo sapiens GRAM domain containing 1A (GRAMD1A), transcript variant 1, mRNA [NM_020895]	1.3504066	0.0054923543
A_33_P3268567	*DLAT*	Homo sapiens dihydrolipoamide S-acetyltransferase (DLAT), mRNA [NM_001931]	-0.6819892	0.004437345
A_23_P4041	*C15orf57*	Homo sapiens chromosome 15 open reading frame 57 (C15orf57), transcript variant 2, mRNA [NM_052849]	-1.4199184	0.0074423784
A_22_P00007479	*NCK2*	Homo sapiens NCK adaptor protein 2 (NCK2), transcript variant 1, mRNA [NM_003581]	-1.1412363	0.0061695063

A_21_P0006347	*PCTP*	Homo sapiens phosphatidylcholine transfer protein (PCTP), transcript variant 1, mRNA [NM_021213]	-1.1432686	0.0027248112
A_33_P3269636	*lnc-GTPBP1-1*	yf55b03.s1 Soares infant brain 1NIB Homo sapiens cDNA clone IMAGE:25867 3', mRNA sequence [R37239]	1.3739371	0.010138355
A_23_P58082	*lnc-DCAF10-2*	LNCipedia lincRNA (lnc-DCAF10-2), lincRNA [lnc-DCAF10-2:1]	-1.2042086	0.013657404
A_24_P649624	*SBSN*	Homo sapiens suprabasin (SBSN), transcript variant 1, mRNA [NM_001166034]	-1.9495893	0.0025739188
A_24_P80138	*CCDC80*	Homo sapiens coiled-coil domain containing 80 (CCDC80), transcript variant 1, mRNA [NM_199511]	-2.3747957	0.0037926938
A_23_P146728	*KIF1B*	Homo sapiens kinesin family member 1B (KIF1B), transcript variant 2, mRNA [NM_183416]	-1.6692312	0.003162355
A_23_P210300	*PDCL3*	Homo sapiens phosducin-like 3 (PDCL3), mRNA [NM_024065]	-0.8515741	0.007474992
A_23_P94434	*ALG2*	Homo sapiens ALG2, alpha-1,3/1,6-mannosyltransferase (ALG2), transcript variant 1, mRNA [NM_033087]	-0.6322096	0.009874361
A_24_P379750	*ASXL2*	Homo sapiens additional sex combs like transcriptional regulator 2 (ASXL2), mRNA [NM_018263]	-0.5945565	0.008651172
A_23_P501877	*HRCT1*	Homo sapiens histidine rich carboxyl terminus 1 (HRCT1), mRNA [NM_001039792]	-1.4904501	0.005428736
A_23_P167159	*MXD1*	Homo sapiens MAX dimerization protein 1 (MXD1), transcript variant 1, mRNA [NM_002357]	-0.5633483	0.0069923652
A_33_P3330125	*ZFP64*	Homo sapiens ZFP64 zinc finger protein (ZFP64), transcript variant 1, mRNA [NM_018197]	-0.9094305	0.008956738
A_23_P64873	*SCRG1*	Homo sapiens stimulator of chondrogenesis 1 (SCRG1), mRNA [NM_007281]	-1.3088065	0.008236777
A_23_P79725	*DIABLO*	Homo sapiens diablo, IAP-binding mitochondrial protein (DIABLO), transcript variant 5, mRNA [NM_001278302]	0.620637	0.016498037
A_24_P58529	*DCN*	Homo sapiens decorin (DCN), transcript variant A1, mRNA [NM_001920]	0.4453113	0.011095965
A_23_P12635	*COPS7B*	Homo sapiens COP9 signalosome subunit 7B (COPS7B), transcript variant 2, mRNA [NM_022730]	0.8786504	0.016148135
A_22_P00015970	*TUBA1C*	Homo sapiens tubulin, alpha 1c (TUBA1C), transcript variant 5, mRNA [NM_032704]	0.4748547	0.010776369
A_22_P00011300	*FBXL15*	Homo sapiens F-box and leucine-rich repeat protein 15 (FBXL15), mRNA [NM_024326]	-0.90762806	0.012215228
A_23_P218988	*lnc-TEKT3-1*	Homo sapiens mRNA; cDNA DKFZp686P21116 (from clone DKFZp686P21116). [AL833462]	-1.9717761	0.0031512622

A_23_P318420	*TUG1*	Homo sapiens taurine up-regulated 1 (non-protein coding) (TUG1), transcript variant 2, long non-coding RNA [NR_110493]	0.6364705	0.0043705306
A_32_P18440	*SLC30A5*	Homo sapiens solute carrier family 30 (zinc transporter), member 5 (SLC30A5), transcript variant 1, mRNA [NM_022902]	-0.63485813	0.006111203
A_23_P137948	*CTTNBP2NL*	Homo sapiens CTTNBP2 N-terminal like (CTTNBP2NL), mRNA [NM_018704]	-0.9232464	0.009683536
A_23_P125423	*ARID5B*	Homo sapiens AT rich interactive domain 5B (MRF1-like) (ARID5B), transcript variant 1, mRNA [NM_032199]	-0.754983	0.0109699825
A_21_P0007219	*NENF*	Homo sapiens neudesin neurotrophic factor (NENF), transcript variant 1, mRNA [NM_013349]	-0.96324086	0.010182504
A_33_P3287735	*C1R*	Homo sapiens complement component 1, r subcomponent (C1R), mRNA [NM_001733]	-0.9846863	0.011641521
A_24_P303454	*lnc-AP000769.1-1*	LNCipedia lincRNA (lnc-AP000769.1-1), lincRNA [lnc-AP000769.1-1:1]	1.6252228	0.0051410845
A_23_P112512	*KAT6A*	Homo sapiens K(lysine) acetyltransferase 6A (KAT6A), transcript variant 1, mRNA [NM_001099412]	-0.6328698	0.005695989
A_33_P3241043	*TIAM2*	Homo sapiens T-cell lymphoma invasion and metastasis 2 (TIAM2), transcript variant 1, mRNA [NM_012454]	-1.1929433	0.0046717683
A_23_P65427	*SLC25A51*	Homo sapiens solute carrier family 25, member 51 (SLC25A51), transcript variant 1, mRNA [NM_033412]	-1.0235552	0.0090693105
A_33_P3592015	*PSME2*	Homo sapiens proteasome (prosome, macropain) activator subunit 2 (PA28 beta) (PSME2), mRNA [NM_002818]	-0.28326446	0.007867977
A_21_P0013814	*AURKC*	Homo sapiens aurora kinase C (AURKC), transcript variant 1, mRNA [NM_001015878]	1.174614	0.015795756
A_23_P96383	*HABP4*	Homo sapiens hyaluronan binding protein 4 (HABP4), mRNA [NM_014282]	-1.1955733	0.005842833
A_22_P00023587	*XLOC_l2_015722*	BROAD Institute lincRNA (XLOC_l2_015722), lincRNA [TCONS_l2_00030483]	-0.71464694	0.009690877
A_24_P294982	*SRPX*	Homo sapiens sushi-repeat containing protein, X-linked (SRPX), transcript variant 1, mRNA [NM_006307]	-0.7504968	0.006579529
A_23_P211878	*CDK3*	Homo sapiens cyclin-dependent kinase 3 (CDK3), mRNA [NM_001258]	-0.87310386	0.005861645
A_23_P150147	*VTA1*	Homo sapiens vesicle (multivesicular body) trafficking 1 (VTA1), transcript variant 1, mRNA [NM_016485]	-0.77791286	0.009129293
A_33_P3499692	*FLNB*	Homo sapiens filamin B, beta (FLNB), transcript variant 2, mRNA [NM_001457]	-0.9086217	0.009414093
A_23_P112260	*SSH3*	Homo sapiens slingshot protein phosphatase 3 (SSH3), mRNA [NM_017857]	1.3721858	0.009610021

A_32_P191527	*LOC645261*	Homo sapiens PP565 mRNA, complete cds. [AF258587]	0.62259895	0.012498083
A_24_P85158	*GNG10*	Homo sapiens guanine nucleotide binding protein (G protein), gamma 10 (GNG10), transcript variant 1, mRNA [NM_001017998]	-0.60104805	0.0038056036
A_23_P201996	*SETD8*	Homo sapiens SET domain containing (lysine methyltransferase) 8 (SETD8), mRNA [NM_020382]	-0.8821958	0.01582957
A_21_P0010577	*SIRT3*	Homo sapiens sirtuin 3 (SIRT3), transcript variant 1, mRNA [NM_012239]	-0.8150742	0.007882029
A_24_P71153	*WAC*	Homo sapiens WW domain containing adaptor with coiled-coil (WAC), transcript variant 2, mRNA [NM_100264]	-0.720881	0.002804883
A_23_P53668	*XLOC_l2_000641*	BROAD Institute lincRNA (XLOC_l2_000641), lincRNA [TCONS_l2_00000849]	-0.94537747	0.013708202
A_22_P00024620	*PAFAH2*	Homo sapiens platelet-activating factor acetylhydrolase 2, 40kDa (PAFAH2), mRNA [NM_000437]	-0.91979903	0.009771336
A_33_P3351474	*NFYB*	Homo sapiens nuclear transcription factor Y, beta (NFYB), mRNA [NM_006166]	-1.013063	0.005735339
A_22_P00021487	*lnc-CBLL1-1*	AY124817 At2g20920/F5H14.11 {Arabidopsis thaliana} (exp=-1; wgp=0; cg=0), partial (5%) [THC2628953]	0.6002888	0.016101176
A_21_P0000501	*SMARCAD1*	Homo sapiens SWI/SNF-related, matrix-associated actin-dependent regulator of chromatin, subfamily a, containing DEAD/H box 1 (SMARCAD1), transcript variant 1, mRNA [NM_001128429]	-0.40944505	0.008132637
A_23_P336565	*TRABD2B*	Homo sapiens TraB domain containing 2B (TRABD2B), mRNA [NM_001194986]	0.97300315	0.012414032
A_23_P67702	*SNORD97*	Homo sapiens small nucleolar RNA, C/D box 97 (SNORD97), small nucleolar RNA [NR_004403]	0.4158587	0.0039416323
A_23_P203994	*FAM185A*	Homo sapiens family with sequence similarity 185, member A (FAM185A), transcript variant 1, mRNA [NM_001145268]	-0.87537897	0.013705582
A_24_P322908	*ZNF85*	Homo sapiens zinc finger protein 85 (ZNF85), transcript variant 1, mRNA [NM_003429]	0.2784374	0.0033868244
A_33_P3294961	*PSMD9*	Homo sapiens proteasome (prosome, macropain) 26S subunit, non-ATPase, 9 (PSMD9), transcript variant 1, mRNA [NM_002813]	-0.562747	0.013618745
A_23_P20752	*USP27X*	Homo sapiens ubiquitin specific peptidase 27, X-linked (USP27X), mRNA [NM_001145073]	-1.0029447	0.006474282
A_32_P108474	*LAT2*	linker for activation of T cells family, member 2 [Source:HGNC Symbol;Acc:HGNC:12749] [ENST00000398475]	-0.71578765	0.010115223

A_32_P101844	*CDK20*	Homo sapiens cyclin-dependent kinase 20 (CDK20), transcript variant 3, mRNA [NM_001039803]	-1.2438142	0.0070658606
A_23_P1519	*ABCE1*	Homo sapiens ATP-binding cassette, sub-family E (OABP), member 1 (ABCE1), transcript variant 1, mRNA [NM_002940]	-0.5070577	0.016369263
A_23_P206901	*PITPNM1*	Homo sapiens phosphatidylinositol transfer protein, membrane-associated 1 (PITPNM1), transcript variant 1, mRNA [NM_004910]	0.96565825	0.009794768
A_23_P68970	*TMEM128*	Homo sapiens transmembrane protein 128 (TMEM128), transcript variant 3, mRNA [NM_032927]	-0.7406018	0.0056142956
A_33_P3226665	*NDE1*	Homo sapiens nudE neurodevelopment protein 1 (NDE1), transcript variant 2, mRNA [NM_017668]	-1.2987735	0.0057506533
A_24_P105391	*ARFGAP3*	Homo sapiens ADP-ribosylation factor GTPase activating protein 3 (ARFGAP3), transcript variant 1, mRNA [NM_014570]	-0.9044404	0.005215599
A_33_P3280841	*ZNF419*	Homo sapiens zinc finger protein 419 (ZNF419), transcript variant 10, mRNA [NM_001291745]	-0.7825692	0.0046320097
A_21_P0000668	*ERMAP*	Homo sapiens erythroblast membrane-associated protein (Scianna blood group) (ERMAP), transcript variant 1, mRNA [NM_001017922]	-0.73950756	0.015238087
A_33_P3401252	*LINC00671*	Homo sapiens long intergenic non-protein coding RNA 671 (LINC00671), long non-coding RNA [NR_027254]	0.5143069	0.009683546
A_21_P0000026	*ADAM1A*	Homo sapiens ADAM metallopeptidase domain 1A (pseudogene) (ADAM1A), non-coding RNA [NR_036636]	-0.9057389	0.0065950155
A_23_P2922	*C15orf40*	Homo sapiens chromosome 15 open reading frame 40 (C15orf40), transcript variant 4, mRNA [NM_001160115]	-0.5745205	0.015874637
A_23_P54846	*MXI1*	Homo sapiens MAX interactor 1, dimerization protein (MXI1), transcript variant 2, mRNA [NM_130439]	-2.3384728	0.005734989
A_33_P3258612	*MBIP*	Homo sapiens MAP3K12 binding inhibitory protein 1 (MBIP), transcript variant 1, mRNA [NM_016586]	-0.47827885	0.006541107
A_22_P00004713	*HERPUD1*	Homo sapiens homocysteine-inducible, endoplasmic reticulum stress-inducible, ubiquitin-like domain member 1 (HERPUD1), transcript variant 1, mRNA [NM_014685]	-11807554	0.003162355
A_23_P78122	*PCNA*	Homo sapiens proliferating cell nuclear antigen (PCNA), transcript variant 1, mRNA [NM_002592]	-0.516624	0.0057506533

A_33_P3323718	*LOC100506844*	Homo sapiens uncharacterized LOC100506844 (LOC100506844), long non-coding RNA [NR_038269]	-0.8676367	0.006161988
A_24_P160969	*MYO18A*	Homo sapiens myosin XVIIIA (MYO18A), transcript variant 1, mRNA [NM_078471]	1.0096776	0.010349238
A_19_P00803441	*UACA*	Homo sapiens uveal autoantigen with coiled-coil domains and ankyrin repeats (UACA), transcript variant 2, mRNA [NM_001008224]	0.87836826	0.0053998437
A_23_P144827	*TP53I11*	Homo sapiens tumor protein p53 inducible protein 11 (TP53I11), transcript variant 1, mRNA [NM_001258320]	0.90405554	0.015446468
A_24_P169544	*LOC100499194*	Homo sapiens uncharacterized LOC100499194 (LOC100499194), long non-coding RNA [NR_034130]	0.7783797	0.010859876
A_24_P234732	*FBXL7*	Homo sapiens F-box and leucine-rich repeat protein 7 (FBXL7), transcript variant 1, mRNA [NM_012304]	-1.0751694	0.009973396
A_23_P63402	*ZNF17*	Homo sapiens zinc finger protein 17 (ZNF17), mRNA [NM_006959]	-0.8956052	0.005255513
A_23_P406616	*MXD4*	Homo sapiens MAX dimerization protein 4 (MXD4), mRNA [NM_006454]	0.8595663	0.01226685
A_32_P167122	*GPSM2*	Homo sapiens G-protein signaling modulator 2 (GPSM2), mRNA [NM_013296]	-0.65203756	0.010004596
A_22_P00013109	*CCDC71L*	Homo sapiens coiled-coil domain containing 71-like (CCDC71L), mRNA [NM_175884]	0.8304589	0.015672265
A_24_P11965	*RCOR3*	Homo sapiens REST corepressor 3 (RCOR3), transcript variant 4, mRNA [NM_018254]	-1.0761579	0.0065170582
A_22_P00022745	*lnc-RIOK1-2*	Homo sapiens cDNA FLJ12098 fis, clone HEMBB1002623. [AK022160]	-1.3401158	0.005377644
A_33_P3321533	*MRFAP1*	Homo sapiens Morf4 family associated protein 1 (MRFAP1), transcript variant 1, mRNA [NM_033296]	-0.6970072	0.011814177
A_23_P6624	*lnc-LRGUK-1*	LNCipedia lincRNA (lnc-LRGUK-1), lincRNA [lnc-LRGUK-1:1]	0.44747135	0.011794303
A_24_P302584	*TMEM178B*	transmembrane protein 178B [Source:HGNC Symbol;Acc:HGNC:44112] [ENST00000563442]	0.41532463	0.0051764864
A_23_P214766	*UMPS*	Homo sapiens uridine monophosphate synthetase (UMPS), transcript variant 1, mRNA [NM_000373]	-0.45879295	0.013100813
A_21_P0000491	*SOX11*	Homo sapiens SRY (sex determining region Y)-box 11 (SOX11), mRNA [NM_003108]	-1.7669362	0.0039659217
A_22_P00019043	*HIVEP2*	Homo sapiens human immunodeficiency virus type I enhancer binding protein 2 (HIVEP2), mRNA [NM_006734]	-0.56933355	0.015151705
A_33_P3243887	*SNAR-G1*	Homo sapiens small ILF3/NF90-associated RNA G1 (SNAR-G1), small nuclear RNA [NR_004383]	-1.039735	0.010804776
A_23_P88470	*lnc-TTC8-1*	LNCipedia lincRNA (lnc-TTC8-1), lincRNA [lnc-TTC8-1:1]	0.6925638	0.009543431

A_22_P00004436	*IL11*	Homo sapiens interleukin 11 (IL11), transcript variant 1, mRNA [NM_000641]	2.5766041	0.0027248112
A_21_P0006206	*TRPM7*	Homo sapiens transient receptor potential cation channel, subfamily M, member 7 (TRPM7), transcript variant 1, mRNA [NM_017672]	-0.5970018	0.008389225
A_33_P3365878	*lnc-RAD23B-7*	LNCipedia lincRNA (lnc-RAD23B-7), lincRNA [lnc-RAD23B-7:1]	0.53679913	0.011958462
A_33_P3247599	*BMP8B*	Homo sapiens bone morphogenetic protein 8b (BMP8B), mRNA [NM_001720]	-0.7742772	0.0117895575
A_33_P3271651	*PRDM2*	Homo sapiens PR domain containing 2, with ZNF domain (PRDM2), transcript variant 2, mRNA [NM_015866]	-1.0778309	0.00558687
A_33_P3210866	*ZNF805*	Homo sapiens zinc finger protein 805 (ZNF805), transcript variant 1, mRNA [NM_001023563]	-1.0970459	0.0029015075
A_22_P00009777	*HLA-DPB1*	Homo sapiens major histocompatibility complex, class II, DP beta 1 (HLA-DPB1), mRNA [NM_002121]	-2.0940218	0.005154336
A_32_P126375	*ZNF808*	zinc finger protein 808 [Source:HGNC Symbol;Acc:HGNC:33230] [ENST00000487863]	-0.661982	0.013753385
A_23_P306105	*NHS*	Homo sapiens Nance-Horan syndrome (congenital cataracts and dental anomalies) (NHS), transcript variant 1, mRNA [NM_198270]	-0.6194861	0.0056002573
A_23_P254120	*SCARNA5*	Homo sapiens small Cajal body-specific RNA 5 (SCARNA5), guide RNA [NR_003008]	-0.8747236	0.013264849
A_22_P00024540	*GALNT1*	Homo sapiens polypeptide N-acetylgalactosaminyltransferase 1 (GALNT1), mRNA [NM_020474]	-1.252152	0.0041358355
A_33_P3320301	*FBXO9*	Homo sapiens F-box protein 9 (FBXO9), transcript variant 2, mRNA [NM_033480]	-0.93151474	0.012481136
A_32_P92783	*lnc-VN1R2-1*	LNCipedia lincRNA (lnc-VN1R2-1), lincRNA [lnc-VN1R2-1:4]	1.1458348	0.007500294
A_33_P3211078	*LINC00493*	Homo sapiens long intergenic non-protein coding RNA 493 (LINC00493), long non-coding RNA [NR_015432]	-0.8086607	0.005514184
A_33_P3341509	*STIP1*	Homo sapiens stress-induced phosphoprotein 1 (STIP1), transcript variant 2, mRNA [NM_006819]	1.2617403	0.003667498
A_23_P124190	*HMGA2*	Homo sapiens high mobility group AT-hook 2 (HMGA2), transcript variant 3, mRNA [NM_001300918]	1.6163887	0.0058474606
A_23_P134755	*TRIM34*	Homo sapiens tripartite motif containing 34 (TRIM34), transcript variant 3, mRNA [NM_130390]	-1.0549288	0.007442623
A_32_P54137	*NDUFB9*	Homo sapiens NADH dehydrogenase (ubiquinone) 1 beta subcomplex, 9, 22kDa	-0.44970635	0.0067226742

		(NDUFB9), transcript variant 1, mRNA [NM_005005]		
A_23_P137046	*TRPS1*	Homo sapiens trichorhinophalangeal syndrome I (TRPS1), transcript variant 1, mRNA [NM_014112]	-1.3797233	0.0028288076
A_23_P311885	*UQCRH*	Homo sapiens ubiquinol-cytochrome c reductase hinge protein (UQCRH), transcript variant 1, mRNA [NM_006004]	-0.42776346	0.014864522
A_23_P14062	*NYX*	Homo sapiens nyctalopin (NYX), mRNA [NM_022567]	1.213504	0.0069110035
A_33_P3257703	*L3MBTL3*	Homo sapiens l(3)mbt-like 3 (Drosophila) (L3MBTL3), transcript variant 1, mRNA [NM_032438]	-0.9559705	0.0083493525
A_22_P00022451	*NUP107*	Homo sapiens nucleoporin 107kDa (NUP107), mRNA [NM_020401]	-0.8421838	0.006961398
A_22_P00002862	*C9orf131*	Homo sapiens chromosome 9 open reading frame 131 (C9orf131), transcript variant 1, mRNA [NM_203299]	0.8089076	0.004588558
A_33_P3281940	*lnc-RP11-582J16.5.1-2*	LNCipedia lincRNA (lnc-RP11-582J16.5.1-2), lincRNA [lnc-RP11-582J16.5.1-2:1]	0.61707896	0.014842539
A_23_P76690	*lnc-C3orf52-1*	Homo sapiens cDNA FLJ35137 fis, clone PLACE6009419. [AK092456]	-1.1900592	0.0034534817
A_32_P5276	*RPE*	Homo sapiens ribulose-5-phosphate-3-epimerase (RPE), transcript variant 4, mRNA [NM_001278283]	-0.61231476	0.013572528
A_21_P0014576	*UCHL3*	Homo sapiens ubiquitin carboxyl-terminal esterase L3 (ubiquitin thiolesterase) (UCHL3), transcript variant 2, mRNA [NM_006002]	-0.5014716	0.015263157
A_23_P108028	*ARHGEF26*	Homo sapiens Rho guanine nucleotide exchange factor (GEF) 26 (ARHGEF26), transcript variant 1, mRNA [NM_001251962]	0.6288214	0.010822477
A_22_P00007468	*ZNF146*	Homo sapiens zinc finger protein 146 (ZNF146), transcript variant 1, mRNA [NM_007145]	-0.4934294	0.009645509
A_23_P163216	*KLHL5*	Homo sapiens kelch-like family member 5 (KLHL5), transcript variant 1, mRNA [NM_015990]	0.6291422	0.0140619585
A_23_P108932	*RBPMS-AS1*	Homo sapiens RBPMS antisense RNA 1 (RBPMS-AS1), long non-coding RNA [NR_046205]	-1.2178222	0.006181179
A_33_P3400324	*ATP8B4*	Homo sapiens ATPase, class I, type 8B, member 4 (ATP8B4), transcript variant 1, mRNA [NM_024837]	0.9363383	0.005920916
A_24_P132787	*RPL23AP32*	Homo sapiens ribosomal protein L23a pseudogene 32 (RPL23AP32), non-coding RNA [NR_002229]	-1.9966655	0.005640775
A_33_P3253832	*PHC3*	Homo sapiens polyhomeotic homolog 3 (Drosophila) (PHC3), mRNA [NM_024947]	-0.69979787	0.008386064

A_32_P169574	*RAB18*	Homo sapiens RAB18, member RAS oncogene family (RAB18), transcript variant 1, mRNA [NM_021252]	-0.619519	0.012579966
A_23_P16078	*OTUD5*	Homo sapiens OTU deubiquitinase 5 (OTUD5), transcript variant 1, mRNA [NM_017602]	0.67922246	0.010156224
A_23_P73097	*XLOC_l2_015894*	BROAD Institute lincRNA (XLOC_l2_015894), lincRNA [TCONS_l2_00030903]	0.7985403	0.0118191205
A_23_P85716	*PAFAH1B3*	Homo sapiens platelet-activating factor acetylhydrolase 1b, catalytic subunit 3 (29kDa) (PAFAH1B3), transcript variant 2, mRNA [NM_002573]	0.81934786	0.014779506
A_23_P76749	*RGS20*	Homo sapiens regulator of G-protein signaling 20 (RGS20), transcript variant 1, mRNA [NM_170587]	1.7847794	0.0047719665
A_24_P131522	*FCGR2A*	Homo sapiens Fc fragment of IgG, low affinity IIa, receptor (CD32) (FCGR2A), transcript variant 2, mRNA [NM_021642]	1.5221074	0.016793482
A_23_P170518	*GALNT16*	Homo sapiens polypeptide N-acetylgalactosaminyltransferase 16 (GALNT16), transcript variant 2, mRNA [NM_020692]	-2.3813205	0.0063986857
A_21_P0012886	*ANTXR1*	Homo sapiens anthrax toxin receptor 1 (ANTXR1), transcript variant 1, mRNA [NM_032208]	-0.6156966	0.015368708
A_23_P62731	*DYM*	Homo sapiens dymeclin (DYM), mRNA [NM_017653]	-1.2707331	0.004100789
A_33_P3213551	*XLOC_l2_011911*	BROAD Institute lincRNA (XLOC_l2_011911), lincRNA [TCONS_l2_00022753]	-2.8948379	0.0052438728
A_33_P3382125	*MRPS14*	Homo sapiens mitochondrial ribosomal protein S14 (MRPS14), transcript variant 1, mRNA [NM_022100]	-0.94737935	0.011222859
A_33_P3320548	*SHARPIN*	Homo sapiens SHANK-associated RH domain interactor (SHARPIN), transcript variant 1, mRNA [NM_030974]	-0.3830995	0.0041523096
A_33_P3218955	*NUPL2*	Homo sapiens nucleoporin like 2 (NUPL2), mRNA [NM_007342]	-0.54591423	0.010212298
A_33_P3334225	*HERC4*	Homo sapiens HECT and RLD domain containing E3 ubiquitin protein ligase 4 (HERC4), transcript variant 1, mRNA [NM_022079]	-0.9117393	0.0038144474
A_24_P38895	*KDM6A*	Homo sapiens lysine (K)-specific demethylase 6A (KDM6A), transcript variant 1, mRNA [NM_001291415]	-0.61952525	0.013635898
A_32_P25437	*ACACA*	Homo sapiens acetyl-CoA carboxylase alpha (ACACA), transcript variant 2, mRNA [NM_198839]	-0.59638166	0.015625326
A_24_P53976	*H2AFX*	Homo sapiens H2A histone family, member X (H2AFX), mRNA [NM_002105]	-0.58657044	0.0070658927

A_23_P69840	*SLC12A2*	Homo sapiens solute carrier family 12 (sodium/potassium/chloride transporter), member 2 (SLC12A2), transcript variant 1, mRNA [NM_001046]	-0.3662815	0.009566855
A_33_P3385461	*GLUL*	Homo sapiens glutamate-ammonia ligase (GLUL), transcript variant 1, mRNA [NM_002065]	-0.42587113	0.006474282
A_23_P31844	*SLC35A4*	Homo sapiens solute carrier family 35, member A4 (SLC35A4), mRNA [NM_080670]	0.9496894	0.014198785
A_21_P0000053	*BRCC3*	Homo sapiens BRCA1/BRCA2-containing complex, subunit 3 (BRCC3), transcript variant 1, mRNA [NM_024332]	-0.68296295	0.006607786
A_24_P196851	*ATP6V1B2*	Homo sapiens ATPase, H+ transporting, lysosomal 56/58kDa, V1 subunit B2 (ATP6V1B2), mRNA [NM_001693]	-0.7769434	0.008749787
A_22_P00014894	*C16orf52*	Homo sapiens chromosome 16 open reading frame 52 (C16orf52), mRNA [NM_001164579]	-0.71537876	0.013369118
A_24_P277657	*TLN1*	Homo sapiens talin 1 (TLN1), mRNA [NM_006289]	0.74242735	0.009724618
A_23_P216448	*lnc-SLC7A5-1*	LNCipedia lincRNA (lnc-SLC7A5-1), lincRNA [lnc-SLC7A5-1:1]	0.91114426	0.0038922254
A_33_P3287745	*GMPR*	Homo sapiens guanosine monophosphate reductase (GMPR), mRNA [NM_006877]	1.3814449	0.008459822
A_23_P65797	*NFIB*	Homo sapiens nuclear factor I/B (NFIB), transcript variant 3, mRNA [NM_005596]	-2.295282	0.0025001846
A_23_P151093	*KAT8*	Homo sapiens K(lysine) acetyltransferase 8 (KAT8), transcript variant 2, mRNA [NM_182958]	-0.77443427	0.011182366
A_33_P3241596	*KLHL25*	Homo sapiens kelch-like family member 25 (KLHL25), mRNA [NM_022480]	-1.0669467	0.0061486606
A_21_P0002211	*YARS2*	Homo sapiens tyrosyl-tRNA synthetase 2, mitochondrial (YARS2), mRNA [NM_001040436]	-0.8128572	0.0071601756
A_32_P20691	*RORB*	Homo sapiens RAR-related orphan receptor B (RORB), mRNA [NM_006914]	-0.74921227	0.003190129
A_33_P3332382	*KCNMA1*	Homo sapiens potassium channel, calcium activated large conductance subfamily M alpha, member 1 (KCNMA1), transcript variant 7, mRNA [NM_001271520]	1.0481412	0.007642386
A_23_P101351	*CLK3*	Homo sapiens CDC-like kinase 3 (CLK3), transcript variant 2, mRNA [NM_003992]	-0.74365157	0.014448771
A_32_P5480	*LOC100129406*	Homo sapiens cDNA FLJ35472 fis, clone SMINT2007062. [AK092791]	0.39628533	0.015769914
A_23_P343398	*ZNF426*	Homo sapiens zinc finger protein 426 (ZNF426), transcript variant 1, mRNA [NM_024106]	-1.0017691	0.008978059
A_23_P153360	*CERS6*	Homo sapiens ceramide synthase 6 (CERS6), transcript variant 2, mRNA [NM_203463]	-3.0816603	0.0030638003

A_22_P00022587	*CCR7*	Homo sapiens chemokine (C-C motif) receptor 7 (CCR7), mRNA [NM_001838]	1.6521139	0.0077495878
A_21_P0001128	*SMIM7*	Homo sapiens small integral membrane protein 7 (SMIM7), transcript variant 2, mRNA [NM_024104]	-0.39567062	0.016822075
A_33_P3407675	*lnc-NUMB-2*	BX101396 Soares_testis_NHT Homo sapiens cDNA clone IMAGp998D114108, mRNA sequence [BX101396]	1.8062406	0.0035470822
A_33_P3344477	*LINC01389*	Homo sapiens long intergenic non-protein coding RNA 1389 (LINC01389), long non-coding RNA [NR_126355]	0.56736207	0.01191373
A_24_P283928	*TIPRL*	Homo sapiens TOR signaling pathway regulator (TIPRL), transcript variant 2, mRNA [NM_001031800]	-0.6439101	0.014162805
A_23_P156890	*FERMT2*	Homo sapiens fermitin family member 2 (FERMT2), transcript variant 2, mRNA [NM_001134999]	-1.338691	0.0029691157
A_33_P3345501	*NABP2*	Homo sapiens nucleic acid binding protein 2 (NABP2), mRNA [NM_024068]	0.6619899	0.009733482
A_33_P3209096	*TCF21*	Homo sapiens transcription factor 21 (TCF21), transcript variant 2, mRNA [NM_003206]	4.5008793	0.0029015075
A_33_P3356290	*CD58*	Homo sapiens CD58 molecule (CD58), transcript variant 1, mRNA [NM_001779]	-1.4892031	0.0044240346
A_23_P500861	*RINL*	Homo sapiens Ras and Rab interactor-like (RINL), transcript variant 1, mRNA [NM_001195833]	-1.6841578	0.005365543
A_21_P0003243	*CADPS*	Homo sapiens Ca++-dependent secretion activator (CADPS), transcript variant 3, mRNA [NM_183393]	-1.7132779	0.0064680316
A_23_P99747	*SYNE1*	Homo sapiens spectrin repeat containing, nuclear envelope 1 (SYNE1), transcript variant 1, mRNA [NM_182961]	-1.4787085	0.0075457073
A_33_P3411427	*lnc-EPHA6-1*	LNCipedia lincRNA (lnc-EPHA6-1), lincRNA [lnc-EPHA6-1:2]	2.30265	0.0039322884
A_23_P218937	*CDKL1*	Homo sapiens cyclin-dependent kinase-like 1 (CDC2-related kinase) (CDKL1), transcript variant 1, mRNA [NM_004196]	-1.4828901	0.011958462
A_23_P100602	*ZNF837*	Homo sapiens zinc finger protein 837 (ZNF837), transcript variant 2, mRNA [NM_138466]	-0.57794565	0.006304432
A_23_P14649	*PCDHGC3*	Homo sapiens protocadherin gamma subfamily C, 3 (PCDHGC3), transcript variant 2, mRNA [NM_032402]	0.90897477	0.0059050764
A_23_P31273	*TBCD*	Homo sapiens tubulin folding cofactor D (TBCD), mRNA [NM_005993]	0.73377323	0.013348818
A_23_P145541	*ANP32A-IT1*	Homo sapiens ANP32A intronic transcript 1 (non-protein coding) (ANP32A-IT1), long non-coding RNA [NR_026808]	-0.46085906	0.012270588

A_33_P3294222	*AMPH*	Homo sapiens amphiphysin (AMPH), transcript variant 1, mRNA [NM_001635]	-1.2541203	0.012871345
A_23_P94501	*FIG4*	Homo sapiens FIG4 phosphoinositide 5-phosphatase (FIG4), mRNA [NM_014845]	-0.80434334	0.011974697
A_23_P143692	*RLTPR*	Homo sapiens RGD motif, leucine rich repeats, tropomodulin domain and proline-rich containing (RLTPR), mRNA [NM_001013838]	1.0227902	0.016256094
A_21_P0014623	*ANXA1*	Homo sapiens annexin A1 (ANXA1), mRNA [NM_000700]	-0.76407856	0.0042908904
A_21_P0011867	*SH3BP1*	Homo sapiens SH3-domain binding protein 1 (SH3BP1), mRNA [NM_018957]	1.1298823	0.0060495283
A_33_P3215487	*SNHG21*	Homo sapiens small nucleolar RNA host gene 21 (non-protein coding) (SNHG21), transcript variant 1, long non-coding RNA [NR_110096]	-0.61427104	0.007819016
A_23_P214638	*XLOC_l2_007656*	BROAD Institute lincRNA (XLOC_l2_007656), lincRNA [TCONS_l2_00014098]	-1.6682596	0.003993975
A_21_P0013652	*AP5Z1*	Homo sapiens adaptor-related protein complex 5, zeta 1 subunit (AP5Z1), mRNA [NM_014855]	-0.5354595	0.012898417
A_23_P152235	*EHMT2*	Homo sapiens euchromatic histone-lysine N-methyltransferase 2 (EHMT2), transcript variant 2, mRNA [NM_006709]	0.97069407	0.014118111
A_24_P337397	*XLOC_l2_015213*	BROAD Institute lincRNA (XLOC_l2_015213), lincRNA [TCONS_l2_00029348]	1.4176067	0.010232035
A_19_P00320971	*IRX3*	Homo sapiens iroquois homeobox 3 (IRX3), mRNA [NM_024336]	-2.497527	0.0028288076
A_23_P60166	*ANKRA2*	Homo sapiens ankyrin repeat, family A (RFXANK-like), 2 (ANKRA2), mRNA [NM_023039]	-1.024836	0.0044319443
A_33_P3212645	*LINC01192*	long intergenic non-protein coding RNA 1192 [Source:HGNC Symbol;Acc:HGNC:37197] [ENST00000488173]	0.44970798	0.010986003
A_23_P115608	*DEPTOR*	Homo sapiens DEP domain containing MTOR-interacting protein (DEPTOR), transcript variant 1, mRNA [NM_022783]	-3.3056593	0.0032589228
A_23_P79661	*NOTCH2NL*	Homo sapiens notch 2 N-terminal like (NOTCH2NL), mRNA [NM_203458]	-0.57448775	0.009160365
A_22_P00007883	*ARHGAP21*	Homo sapiens Rho GTPase activating protein 21 (ARHGAP21), mRNA [NM_020824]	-0.4933429	0.013029367
A_23_P115366	*CCDC93*	Homo sapiens coiled-coil domain containing 93 (CCDC93), mRNA [NM_019044]	-1.3047817	0.0044365884
A_33_P3407626	*HSD17B11*	Homo sapiens hydroxysteroid (17-beta) dehydrogenase 11 (HSD17B11), mRNA [NM_016245]	-1.0189434	0.003121324
A_33_P3303430	*CMPK1*	Homo sapiens cytidine monophosphate (UMP-CMP) kinase 1, cytosolic (CMPK1), transcript variant 1, mRNA [NM_016308]	-0.8727266	0.0044240346

A_33_P3340847	*QTRTD1*	Homo sapiens queuine tRNA-ribosyltransferase domain containing 1 (QTRTD1), transcript variant 1, mRNA [NM_024638]	-0.4684346	0.011212511
A_33_P3254460	*CHD6*	Homo sapiens chromodomain helicase DNA binding protein 6 (CHD6), mRNA [NM_032221]	-0.9985315	0.005936798
A_23_P77568	*CARD6*	Homo sapiens caspase recruitment domain family, member 6 (CARD6), mRNA [NM_032587]	-0.31228444	0.016409002
A_24_P916614	*DLK2*	Homo sapiens delta-like 2 homolog (Drosophila) (DLK2), transcript variant 2, mRNA [NM_206539]	1.2637452	0.012362548
A_23_P327069	*EIF3C*	Homo sapiens eukaryotic translation initiation factor 3, subunit C (EIF3C), transcript variant 2, mRNA [NM_001037808]	-0.39066505	0.011257593
A_24_P397247	*PTBP3*	Homo sapiens polypyrimidine tract binding protein 3 (PTBP3), transcript variant 1, mRNA [NM_005156]	-0.5932956	0.0065101455
A_23_P48803	*KIAA0232*	Homo sapiens KIAA0232 (KIAA0232), transcript variant 1, mRNA [NM_014743]	-0.8518672	0.004094079
A_24_P88850	*SDAD1*	Homo sapiens SDA1 domain containing 1 (SDAD1), transcript variant 1, mRNA [NM_018115]	-0.60554385	0.0056098294
A_24_P59247	*TMOD2*	Homo sapiens tropomodulin 2 (neuronal) (TMOD2), transcript variant 1, mRNA [NM_014548]	-1.3474039	0.011557543
A_21_P0000401	*MRAS*	Homo sapiens muscle RAS oncogene homolog (MRAS), transcript variant 1, mRNA [NM_012219]	-0.7943046	0.007929221
A_33_P3423240	*SNORD114-4*	Homo sapiens small nucleolar RNA, C/D box 114-4 (SNORD114-4), small nucleolar RNA [NR_003196]	0.77911913	0.0077676554
A_23_P315571	*G6PD*	Homo sapiens glucose-6-phosphate dehydrogenase (G6PD), transcript variant 1, mRNA [NM_000402]	2.135973	0.0034766807
A_33_P3213707	*RFTN1*	Homo sapiens raftlin, lipid raft linker 1 (RFTN1), mRNA [NM_015150]	-0.89755696	0.006632856
A_23_P41470	*MIS12*	Homo sapiens MIS12 kinetochore complex component (MIS12), transcript variant 2, mRNA [NM_024039]	-0.6140844	0.0080620805
A_23_P370454	*PDCD1LG2*	Homo sapiens programmed cell death 1 ligand 2 (PDCD1LG2), mRNA [NM_025239]	0.64123636	0.014448771
A_33_P3247175	*DDX60*	Homo sapiens DEAD (Asp-Glu-Ala-Asp) box polypeptide 60 (DDX60), mRNA [NM_017631]	-0.40098584	0.0050043953
A_21_P0011562	*KCNAB3*	Homo sapiens potassium channel, voltage gated subfamily A regulatory beta subunit 3 (KCNAB3), mRNA [NM_004732]	0.24520117	0.003392081
A_23_P48740	*C4orf47*	Homo sapiens chromosome 4 open reading frame 47 (C4orf47), mRNA [NM_001114357]	-2.159995	0.008976597

A_33_P3286254	*XLOC_l2_005933*	BROAD Institute lincRNA (XLOC_l2_005933), lincRNA [TCONS_l2_00011027]	1.0107841	0.007698106
A_24_P922357	*DIO2*	Homo sapiens deiodinase, iodothyronine, type II (DIO2), transcript variant 1, mRNA [NM_013989]	-1.92926	0.003162355
A_23_P358995	*AP3S1*	Homo sapiens adaptor-related protein complex 3, sigma 1 subunit (AP3S1), mRNA [NM_001284]	-0.60782975	0.004026994
A_23_P389281	*C22orf39*	Homo sapiens chromosome 22 open reading frame 39 (C22orf39), transcript variant 1, mRNA [NM_173793]	-0.9084823	0.010284574
A_22_P00020393	*IBTK*	Homo sapiens inhibitor of Bruton agammaglobulinemia tyrosine kinase (IBTK), transcript variant 1, mRNA [NM_015525]	-0.60736483	0.0052438728
A_24_P83808	*HOXA13*	Homo sapiens homeobox A13 (HOXA13), mRNA [NM_000522]	-0.6405053	0.011190785
A_23_P309381	*C10orf10*	Homo sapiens chromosome 10 open reading frame 10 (C10orf10), mRNA [NM_007021]	-2.2355726	0.0066283424
A_23_P88710	*PDHB*	Homo sapiens pyruvate dehydrogenase (lipoamide) beta (PDHB), transcript variant 1, mRNA [NM_000925]	-0.8080782	0.005182173
A_24_P325176	*HIST2H2AA4*	Homo sapiens histone cluster 2, H2aa4 (HIST2H2AA4), mRNA [NM_001040874]	1.0837137	0.0047066417
A_33_P3311493	*TMEM87A*	Homo sapiens transmembrane protein 87A (TMEM87A), transcript variant 1, mRNA [NM_015497]	-0.68811655	0.007222818
A_24_P355267	*KIAA1109*	Homo sapiens KIAA1109 (KIAA1109), mRNA [NM_015312]	-0.99227613	0.003365343
A_22_P00021229	*TRHDE-AS1*	Homo sapiens TRHDE antisense RNA 1 (TRHDE-AS1), transcript variant 2, long non-coding RNA [NR_026836]	1.4968902	0.00180587
A_24_P99838	*SLC25A25*	Homo sapiens solute carrier family 25 (mitochondrial carrier; phosphate carrier), member 25 (SLC25A25), transcript variant 2, mRNA [NM_001006641]	-0.5794637	0.0080137085
A_33_P3316223	*lnc-SHISA4-1*	Homo sapiens cDNA FLJ45279 fis, clone BRHIP3001338. [AK127212]	-1.1933509	0.0108264685
A_24_P136758	*ZNF223*	Homo sapiens zinc finger protein 223 (ZNF223), mRNA [NM_013361]	-1.0934901	0.0062715514
A_23_P381368	*SNAPC1*	Homo sapiens small nuclear RNA activating complex, polypeptide 1, 43kDa (SNAPC1), mRNA [NM_003082]	-1.0517479	0.0067226742
A_22_P00017827	*HOXD10*	Homo sapiens homeobox D10 (HOXD10), mRNA [NM_002148]	3.3387454	0.004288771
A_22_P00021268	*MFSD11*	major facilitator superfamily domain containing 11 [Source:HGNC Symbol;Acc:HGNC:25458] [ENST00000588460]	0.9516314	0.004160909

A_33_P3242369	*lnc-ZC3H12D-2*	RST31404 Athersys RAGE Library Homo sapiens cDNA, mRNA sequence [BG211832]	-0.9004349	0.0108836265
A_22_P00017555	*lnc-UBE2Z-1*	Synthetic construct Homo sapiens gateway clone IMAGE:100018633 3' read SNF8 mRNA. [CU679210]	0.65740395	0.00969217
A_23_P347432	*lnc-WDR45L-1*	Homo sapiens mRNA; cDNA DKFZp434D1428 (from clone DKFZp434D1428). [AL136830]	-0.7297565	0.009074582
A_23_P146946	*FLJ32255*	Homo sapiens uncharacterized LOC643977 (FLJ32255), long non-coding RNA [NR_104643]	0.95283717	0.006360746
A_23_P137381	*DVL1*	Homo sapiens dishevelled segment polarity protein 1 (DVL1), mRNA [NM_004421]	1.6581411	0.003948561
A_23_P84929	*CST6*	Homo sapiens cystatin E/M (CST6), mRNA [NM_001323]	-2.4913478	0.0025001846
A_33_P3314525	*ID3*	Homo sapiens inhibitor of DNA binding 3, dominant negative helix-loop-helix protein (ID3), mRNA [NM_002167]	1.1484748	0.0072854767
A_33_P3317277	*SLC38A5*	Homo sapiens solute carrier family 38, member 5 (SLC38A5), mRNA [NM_033518]	0.81340075	0.013936194
A_23_P390704	*CRYM-AS1*	Homo sapiens CRYM antisense RNA 1 (CRYM-AS1), long non-coding RNA [NR_026675]	-0.66461134	0.009921689
A_23_P46982	*DPF1*	Homo sapiens D4, zinc and double PHD fingers family 1 (DPF1), transcript variant 4, mRNA [NM_001289978]	0.9285898	0.009029172
A_21_P0005785	*STK38*	Homo sapiens serine/threonine kinase 38 (STK38), mRNA [NM_007271]	-0.6325891	0.01381281
A_24_P943095	*MMS19*	Homo sapiens MMS19 nucleotide excision repair homolog (S. cerevisiae) (MMS19), transcript variant 1, mRNA [NM_022362]	-0.75234866	0.0037781831
A_33_P3419998	*lnc-ERICH1-5*	LNCipedia lincRNA (lnc-ERICH1-5), lincRNA [lnc-ERICH1-5:1]	2.0013492	0.003416591
A_19_P00809992	*SPECC1*	Homo sapiens sperm antigen with calponin homology and coiled-coil domains 1 (SPECC1), transcript variant 1, mRNA [NM_001033553]	0.89283526	0.01664532
A_23_P129334	*SLC4A8*	Homo sapiens solute carrier family 4, sodium bicarbonate cotransporter, member 8 (SLC4A8), transcript variant 1, mRNA [NM_001039960]	-1.1896706	0.0033868244
A_23_P433079	*C11orf74*	Homo sapiens chromosome 11 open reading frame 74 (C11orf74), transcript variant 1, mRNA [NM_001276722]	-0.91994816	0.010474825
A_21_P0014472	*CLCN7*	Homo sapiens chloride channel, voltage-sensitive 7 (CLCN7), transcript variant 1, mRNA [NM_001287]	1.2320266	0.007774276
A_33_P3328485	*DNAJC14*	Homo sapiens DnaJ (Hsp40) homolog, subfamily C, member 14 (DNAJC14), mRNA [NM_032364]	-0.44853377	0.008404369

A_21_P0012357	*IKZF2*	Homo sapiens IKAROS family zinc finger 2 (Helios) (IKZF2), transcript variant 2, mRNA [NM_001079526]	1.2857519	0.011605183
A_23_P401	*FAM208A*	Homo sapiens family with sequence similarity 208, member A (FAM208A), transcript variant 2, mRNA [NM_015224]	-0.88791347	0.0057806885
A_24_P303480	*CENPF*	Homo sapiens centromere protein F, 350/400kDa (CENPF), mRNA [NM_016343]	-0.59177375	0.01381281
A_33_P3318746	*ZC3H6*	Homo sapiens zinc finger CCCH-type containing 6 (ZC3H6), mRNA [NM_198581]	-1.2471311	0.012791966

Table S3. Differentially expressed genes untreated vs siRNA3 treated FBs

Probe set ID	Gene symbol	Gene name	2Log Fold Change	p.value
A_23_P369471	*LCE3A*	Homo sapiens late cornified envelope 3A (LCE3A), mRNA [NM_178431]	-1.5107913	0.03650733
A_24_P292929	*CAMKK1*	Homo sapiens calcium/calmodulin-dependent protein kinase kinase 1, alpha (CAMKK1), transcript variant 3, mRNA [NM_172207]	-0.99820465	1,76E+02
A_33_P3302389	*FEM1A*	Homo sapiens fem-1 homolog a (C. elegans) (FEM1A), mRNA [NM_018708]	-0.994365	1,74E-06
A_19_P00802448	*lnc-ANKRA2-3*	LNCipedia lincRNA (lnc-ANKRA2-3), lincRNA [lnc-ANKRA2-3:1]	-0.9765941	0.0013620802
A_22_P00020225	*LOC100132215*	Homo sapiens uncharacterized LOC100132215 (LOC100132215), transcript variant 1, long non-coding RNA [NR_027069]	-0.96729976	0.031726398
A_21_P0012131	*MIR646HG*	Homo sapiens MIR646 host gene (non-protein coding) (MIR646HG), long non-coding RNA [NR_046099]	-0.9671868	7,06E-05
A_24_P606538	*GGNBP1*	Homo sapiens gametogenetin binding protein 1 (pseudogene) (GGNBP1), non-coding RNA [NR_028361]	0.9503849	0.03201747
A_33_P3364062	*FLJ31356*	Homo sapiens uncharacterized protein FLJ31356 (FLJ31356), long non-coding RNA [NR_103831]	-0.93246484	0.0014252557
A_21_P0000311	*SNORA14A*	Homo sapiens small nucleolar RNA, H/ACA box 14A (SNORA14A), small nucleolar RNA [NR_002955]	-0.92003155	0.014638112

A_33_P3821778	*LINC01021*	long intergenic non-protein coding RNA 1021 [Source:HGNC Symbol;Acc:HGNC:48995] [ENST00000512067]	-0.9045643	0.0014252557
A_22_P00005639	*lnc-EIF2AK3-4*	Q2S9U3_HAHCH (Q2S9U3) Tfp pilus assembly protein FimT, partial (8%) [THC2741911]	-0.9039704	7,20E-07
A_24_P286569	*LANCL2*	Homo sapiens LanC lantibiotic synthetase component C-like 2 (bacterial) (LANCL2), mRNA [NM_018697]	-0.90343505	0.033744548
A_23_P24903	*P2RY2*	Homo sapiens purinergic receptor P2Y, G-protein coupled, 2 (P2RY2), transcript variant 1, mRNA [NM_176072]	-0.8876952	0.020627521
A_22_P00006487	*LOC100506674*	Homo sapiens uncharacterized LOC100506674 (LOC100506674), transcript variant 4, long non-coding RNA [NR_109865]	-0.8811784	0.0031119252
A_23_P170649	*SBSPON*	Homo sapiens somatomedin B and thrombospondin, type 1 domain containing (SBSPON), mRNA [NM_153225]	-0.8724477	7,31E-02
A_21_P0007204	*LOC101928812*	Homo sapiens uncharacterized LOC101928812 (LOC101928812), transcript variant 1, long non-coding RNA [NR_120557]	0.8653424	1,37E+00
A_33_P3252800	*PTPRR*	Homo sapiens protein tyrosine phosphatase, receptor type, R (PTPRR), transcript variant 1, mRNA [NM_002849]	0.8642196	8,56E+02
A_33_P3361758	*FAM227B*	Homo sapiens family with sequence similarity 227, member B (FAM227B), mRNA [NM_152647]	-0.8560214	5,96E-03
A_21_P0003255	*lnc-P2RY1-3*	LNCipedia lincRNA (lnc-P2RY1-3), lincRNA [lnc-P2RY1-3:8]	0.8554616	0.033744548
A_21_P0006421	*LINC01204*	long intergenic non-protein coding RNA 1204 [Source:HGNC Symbol;Acc:HGNC:49635] [ENST00000422047]	-0.8521979	9,27E-07
A_23_P121480	*CD200*	Homo sapiens CD200 molecule (CD200), transcript variant 2, mRNA [NM_001004196]	-0.8521714	7,20E-07
A_23_P2942	*NPAS3*	Homo sapiens neuronal PAS domain protein 3 (NPAS3), transcript variant 2, mRNA [NM_022123]	-0.8447344	4,23E-05
A_23_P130547	*OR7D4*	Homo sapiens olfactory receptor, family 7, subfamily D, member 4 (OR7D4), mRNA [NM_001005191]	-0.84205115	4,43E-04
A_33_P3500167	*ACAN*	Homo sapiens aggrecan (ACAN), transcript variant 1, mRNA [NM_001135]	-0.8386695	0.0023199285
A_24_P413669	*PFKFB2*	Homo sapiens 6-phosphofructo-2-kinase/fructose-2,6-biphosphatase 2 (PFKFB2), transcript variant 2, mRNA [NM_001018053]	-0.8372427	0.03650733
A_23_P89302	*CACNG1*	Homo sapiens calcium channel, voltage-dependent, gamma subunit 1 (CACNG1), mRNA [NM_000727]	-0.8336058	0.049553927
A_21_P0004325	*LOC102724458*	PREDICTED: Homo sapiens uncharacterized LOC102724458 (LOC102724458), ncRNA [XR_425932]	-0.83046645	1,31E-04

A_33_P3217731	*TLE1*	transducin-like enhancer of split 1 (E(sp1) homolog, Drosophila) [Source:HGNC Symbol;Acc:HGNC:11837] [ENST00000376484]	-0.82630706	0.04633323
A_21_P0004219	*LINC01018*	Homo sapiens long intergenic non-protein coding RNA 1018 (LINC01018), transcript variant 1, long non-coding RNA [NR_024424]	-0.8177358	0.0013620802
A_21_P0000857	*MORF4L2-AS1*	Homo sapiens MORF4L2 antisense RNA 1 (MORF4L2-AS1), long non-coding RNA [NR_038978]	-0.81417805	6,76E-04
A_23_P6201	*LIPI*	Homo sapiens lipase, member I (LIPI), transcript variant 2, mRNA [NM_198996]	-0.81229424	3,07E+01
A_22_P00017729	*KIZ*	kizuna centrosomal protein [Source:HGNC Symbol;Acc:HGNC:15865] [ENST00000441136]	-0.78527915	0.0029840462
A_21_P0000060	*FRAS1*	Homo sapiens Fraser extracellular matrix complex subunit 1 (FRAS1), transcript variant 2, mRNA [NM_001166133]	-0.7850411	4,83E-06
A_21_P0008548	*LINC01550*	long intergenic non-protein coding RNA 1550 [Source:HGNC Symbol;Acc:HGNC:20111] [ENST00000554822]	-0.7796922	8,10E-06
A_33_P3333232	*CCDC162P*	coiled-coil domain containing 162, pseudogene [Source:HGNC Symbol;Acc:HGNC:21565] [ENST00000621947]	-0.77852273	5,63E-03
A_21_P0004532	*LOC102546299*	Homo sapiens uncharacterized LOC102546299 (LOC102546299), long non-coding RNA [NR_105065]	-0.77614135	2,85E+01
A_21_P0012110	*LOC101929395*	Homo sapiens uncharacterized LOC101929395 (LOC101929395), long non-coding RNA [NR_110611]	-0.7714431	1,49E+02
A_33_P3287636		T cell receptor alpha variable 21 [Source:HGNC Symbol;Acc:HGNC:12118] [ENST00000390449]	-0.768102	0.04722463
A_33_P3289865	*PLCL1*	Homo sapiens phospholipase C-like 1 (PLCL1), mRNA [NM_006226]	-0.7619654	0.03387884
A_33_P3306828		zinc finger protein 806 [Source:HGNC Symbol;Acc:HGNC:33228] [ENST00000438300]	-0.75759405	7,20E-07
A_22_P00009656	*lnc-MAT2B-3*	LNCipedia lincRNA (lnc-MAT2B-3), lincRNA [lnc-MAT2B-3:19]	-0.74989986	6,29E-06
A_21_P0010564	*XLOC_l2_000471*	BROAD Institute lincRNA (XLOC_l2_000471), lincRNA [TCONS_l2_00000660]	-0.74138707	7,03E-01
A_19_P00322599	*KIAA1671*	Homo sapiens KIAA1671 (KIAA1671), mRNA [NM_001145206]	-0.7300689	9,27E-07
A_21_P0012205	*XLOC_l2_009219*	BROAD Institute lincRNA (XLOC_l2_009219), lincRNA [TCONS_l2_00017238]	-0.7297345	0.0013620802
A_22_P00004405	*LOC100130992*	Homo sapiens uncharacterized LOC100130992 (LOC100130992), long non-coding RNA [NR_038921]	-0.7273326	3,07E-01
A_22_P00002758		Homo sapiens chromosome 21 open reading frame 135, mRNA (cDNA clone IMAGE:3891427), with apparent retained intron. [BC009635]	-0.72493625	1,14E+01

A_32_P351277	*lnc-JMJD8-1*	Homo sapiens, clone IMAGE:3543963, mRNA. [BC004968]	-0.7237616	0.0013620802
A_24_P82032	*RHOJ*	Homo sapiens ras homolog family member J (RHOJ), mRNA [NM_020663]	-0.7226467	1,34E-06
A_19_P00322746		PREDICTED: Homo sapiens uncharacterized LOC101928011 (LOC101928011), ncRNA [XR_244302]	-0.7196306	8,13E-02
A_21_P0006479	*lnc-CXorf28-1*	LNCipedia lincRNA (lnc-CXorf28-1), lincRNA [lnc-CXorf28-1:1]	-0.71803874	0.034170788
A_23_P251881	*NCR3*	Homo sapiens natural cytotoxicity triggering receptor 3 (NCR3), transcript variant 1, mRNA [NM_147130]	0.7173416	0.0013620802
A_22_P00016217	*LOC654841*	Homo sapiens uncharacterized LOC654841 (LOC654841), long non-coding RNA [NR_102371]	-0.71589947	8,17E-05
A_23_P45365	*COL4A5*	Homo sapiens collagen, type IV, alpha 5 (COL4A5), transcript variant 2, mRNA [NM_033380]	-0.71444225	6,21E-05
A_24_P228796	*GAGE7*	Homo sapiens G antigen 7 (GAGE7), mRNA [NM_021123]	-0.7128798	0.03740897
A_23_P23783	*MYOC*	Homo sapiens myocilin, trabecular meshwork inducible glucocorticoid response (MYOC), mRNA [NM_000261]	-0.7128688	1,47E-05
A_22_P00023543	*lnc-RNF38-1*	BX098108 Soares fetal liver spleen 1NFLS Homo sapiens cDNA clone IMAGp998F20117, mRNA sequence [BX098108]	-0.7107486	3,60E+00
A_33_P3232965	*TDRD6*	Homo sapiens tudor domain containing 6 (TDRD6), transcript variant 1, mRNA [NM_001010870]	-0.69747496	1,41E-06
A_21_P0007155	*lnc-RHOG-2*	602971010F1 NIH_MGC_12 Homo sapiens cDNA clone IMAGE:5110226 5', mRNA sequence [BI260813]	-0.6919969	2,74E+01
A_32_P486620	*IGSF22*	Homo sapiens immunoglobulin superfamily, member 22 (IGSF22), mRNA [NM_173588]	-0.687938	3,67E-08
A_23_P23669	*PALMD*	Homo sapiens palmdelphin (PALMD), mRNA [NM_017734]	0.68589973	0.035078954
A_19_P00802814	*LOC729296*	Homo sapiens uncharacterized LOC729296 (LOC729296), transcript variant 1, long non-coding RNA [NR_109916]	-0.6842911	0.033744548
A_33_P3279959	*PLXNA4*	Homo sapiens plexin A4 (PLXNA4), transcript variant 3, mRNA [NM_001105543]	-0.67819035	2,03E+02
A_21_P0003180	*lnc-MSL2-1*	LNCipedia lincRNA (lnc-MSL2-1), lincRNA [lnc-MSL2-1:1]	-0.6770178	0.008105122
A_23_P7313	*SPP1*	Homo sapiens secreted phosphoprotein 1 (SPP1), transcript variant 1, mRNA [NM_001040058]	-0.67593473	0.0013620802
A_24_P659836	*SYCE3*	Homo sapiens synaptonemal complex central element protein 3 (SYCE3), mRNA [NM_001123225]	-0.6716641	7,20E-07
A_21_P0012177	*XLOC_l2_009105*	BROAD Institute lincRNA (XLOC_l2_009105), lincRNA [TCONS_l2_00017094]	-0.6657797	2,39E-05

A_21_P0008446	*lnc-TMEM30B-3*	LNCipedia lincRNA (lnc-TMEM30B-3), lincRNA [lnc-TMEM30B-3:6]	-0.6609051	1,26E-04
A_21_P0004231	*lnc-FAM105B-3*	LNCipedia lincRNA (lnc-FAM105B-3), lincRNA [lnc-FAM105B-3:2]	0.651612	3,39E-06
A_19_P00801088	*ANKRD20A12P*	Homo sapiens ankyrin repeat domain 20 family, member A12, pseudogene (ANKRD20A12P), non-coding RNA [NR_046228]	-0.65063643	1,52E+02
A_23_P411825	*CCDC148*	Homo sapiens coiled-coil domain containing 148 (CCDC148), transcript variant 1, mRNA [NM_138803]	-0.64744204	4,06E-02
A_22_P00023071	*LOC102724587*	PREDICTED: Homo sapiens uncharacterized LOC102724587 (LOC102724587), transcript variant X2, ncRNA [XR_424628]	-0.6470509	1,60E-05
A_22_P00014988	*C3orf80*	Homo sapiens chromosome 3 open reading frame 80 (C3orf80), mRNA [NM_001168214]	-0.6401956	5,70E-06
A_24_P149445	*WEE2-AS1*	Homo sapiens WEE2 antisense RNA 1 (WEE2-AS1), long non-coding RNA [NR_015392]	-0.6394096	2,32E-04
A_23_P257231	*ETNPPL*	Homo sapiens ethanolamine-phosphate phospho-lyase (ETNPPL), transcript variant 1, mRNA [NM_031279]	-0.63842595	5,00E+02
A_33_P6818629	*PCBP1-AS1*	Homo sapiens PCBP1 antisense RNA 1 (PCBP1-AS1), long non-coding RNA [NR_033872]	-0.637059	0.048573185
A_33_P3323939	*LIPN*	Homo sapiens lipase, family member N (LIPN), mRNA [NM_001102469]	-0.63115364	0.033744548
A_23_P90626	*CYTIP*	Homo sapiens cytohesin 1 interacting protein (CYTIP), mRNA [NM_004288]	-0.62612355	6,51E-05
A_24_P304423	*IGF1*	Homo sapiens insulin-like growth factor 1 (somatomedin C) (IGF1), transcript variant 4, mRNA [NM_000618]	-0.6237413	0.0013620802
A_23_P49657	*MYH1*	Homo sapiens myosin, heavy chain 1, skeletal muscle, adult (MYH1), mRNA [NM_005963]	-0.6225416	7,20E-07
A_23_P373464	*AFF3*	Homo sapiens AF4/FMR2 family, member 3 (AFF3), transcript variant 1, mRNA [NM_002285]	-0.61676675	4,28E-05
A_21_P0000831	*LOC100506368*	Homo sapiens uncharacterized LOC100506368 (LOC100506368), long non-coding RNA [NR_038905]	-0.6106946	0.03578969
A_21_P0003877	*lnc-TRIML1-3*	LNCipedia lincRNA (lnc-TRIML1-3), lincRNA [lnc-TRIML1-3:10]	-0.61065847	3,40E+02
A_24_P140475	*SORBS2*	Homo sapiens sorbin and SH3 domain containing 2 (SORBS2), transcript variant 2, mRNA [NM_021069]	-0.6091638	0.004688134
A_21_P0009743	*lnc-ZNF100-3*	LNCipedia lincRNA (lnc-ZNF100-3), lincRNA [lnc-ZNF100-3:1]	-0.60725266	0.0013620802
A_23_P65789	*MCTP2*	Homo sapiens multiple C2 domains, transmembrane 2 (MCTP2), transcript variant 1, mRNA [NM_018349]	0.6036978	0.033744548
A_24_P303199	*ARL15*	Homo sapiens ADP-ribosylation factor-like 15 (ARL15), mRNA [NM_019087]	-0.6019606	1,60E-05

A_32_P193583	*FAM218A*	Homo sapiens family with sequence similarity 218, member A (FAM218A), mRNA [NM_153027]	-0.6011206	0.0013620802
A_33_P3360565	*ZNF474*	zinc finger protein 474 [Source:HGNC Symbol;Acc:HGNC:23245] [ENST00000296600]	0.59706557	0.033858534
A_22_P00001898	*lnc-B4GALT3-1*	LNCipedia lincRNA (lnc-B4GALT3-1), lincRNA [lnc-B4GALT3-1:1]	-0.58646756	0.03459761
A_24_P943113	*EIF4E3*	Homo sapiens eukaryotic translation initiation factor 4E family member 3 (EIF4E3), transcript variant 2, mRNA [NM_173359]	0.58247775	0.036519654
A_22_P00017000	*LOC100130691*	Homo sapiens uncharacterized LOC100130691 (LOC100130691), long non-coding RNA [NR_026966]	-0.58040786	0.033744548
A_22_P00010132	*lnc-MRPL3-2*	LNCipedia lincRNA (lnc-MRPL3-2), lincRNA [lnc-MRPL3-2:2]	-0.5778944	0.004134883
A_21_P0012197	*XLOC_l2_009182*	BROAD Institute lincRNA (XLOC_l2_009182), lincRNA [TCONS_l2_00017189]	-0.5758753	7,20E-07
A_33_P3252281	*EYA4*	Homo sapiens EYA transcriptional coactivator and phosphatase 4 (EYA4), transcript variant 1, mRNA [NM_004100]	0.5744577	1,58E+01
A_33_P3217490	*FAM230B*	Homo sapiens family with sequence similarity 230, member B (non-protein coding) (FAM230B), long non-coding RNA [NR_108107]	-0.5728114	0.03171026
A_24_P63380	*BMPR1B*	Homo sapiens bone morphogenetic protein receptor, type IB (BMPR1B), transcript variant 2, mRNA [NM_001203]	-0.5712677	0.0041201347
A_33_P3338300	*CASZ1*	Homo sapiens castor zinc finger 1 (CASZ1), transcript variant 1, mRNA [NM_001079843]	-0.5702155	0.010379838
A_33_P3408757	*FOXO6*	Homo sapiens forkhead box O6 (FOXO6), mRNA [NM_001291281]	-0.5677802	1,74E-06
A_22_P00006548	*lnc-FKBP3-1*	LNCipedia lincRNA (lnc-FKBP3-1), lincRNA [lnc-FKBP3-1:4]	-0.5588376	7,20E-07
A_33_P3404899	*LPHN2*	Homo sapiens latrophilin 2 (LPHN2), transcript variant 1, mRNA [NM_012302]	-0.5581433	1,44E-02
A_19_P00803334	*lnc-GALNTL4-2*	LNCipedia lincRNA (lnc-GALNTL4-2), lincRNA [lnc-GALNTL4-2:1]	-0.55153376	0.03459761
A_21_P0001167	*LOC100996263*	Homo sapiens uncharacterized LOC100996263 (LOC100996263), long non-coding RNA [NR_121626]	0.5373984	0.0013620802
A_21_P0004941	*LINC01013*	Homo sapiens long intergenic non-protein coding RNA 1013 (LINC01013), long non-coding RNA [NR_038981]	-0.5291774	3,09E+02
A_21_P0007980	*LINC00384*	long intergenic non-protein coding RNA 384 [Source:HGNC Symbol;Acc:HGNC:42711] [ENST00000437721]	-0.52906996	1,87E-05
A_22_P00020264	*LOC101929577*	Homo sapiens uncharacterized LOC101929577 (LOC101929577), long non-coding RNA [NR_125928]	0.5281336	5,64E+02

A_22_P00012842	*lnc-RASA1-7*	ALU1_HUMAN (P39188) Alu subfamily J sequence contamination warning entry, partial (13%) [THC2693066]	-0.5273968	4,96E+00
A_22_P00006603	*LINC01220*	Homo sapiens long intergenic non-protein coding RNA 1220 (LINC01220), long non-coding RNA [NR_038421]	-0.52707285	0.0018572797
A_22_P00009142	*SNHG22*	Homo sapiens small nucleolar RNA host gene 22 (non-protein coding) (SNHG22), long non-coding RNA [NR_117096]	-0.5243958	0.0013620802
A_21_P0012715	*LINC01085*	Homo sapiens long intergenic non-protein coding RNA 1085 (LINC01085), long non-coding RNA [NR_033931]	0.5144408	0.0143562
A_33_P3214665	*MAP2*	Homo sapiens microtubule-associated protein 2 (MAP2), transcript variant 1, mRNA [NM_002374]	-0.5142558	8,55E-02
A_23_P126031	*OR10J5*	Homo sapiens olfactory receptor, family 10, subfamily J, member 5 (OR10J5), mRNA [NM_001004469]	-0.4987741	0.03459761
A_21_P0008485	*LINC01550*	long intergenic non-protein coding RNA 1550 [Source:HGNC Symbol;Acc:HGNC:20111] [ENST00000554822]	-0.49851787	2,16E-05
A_21_P0008040	*lnc-GSX1-1*	LNCipedia lincRNA (lnc-GSX1-1), lincRNA [lnc-GSX1-1:1]	-0.4967447	0.033744548
A_23_P253446	*GAP43*	Homo sapiens growth associated protein 43 (GAP43), transcript variant 2, mRNA [NM_002045]	-0.49477184	7,20E-07
A_21_P0008049	*lnc-EXOSC8-1*	LNCipedia lincRNA (lnc-EXOSC8-1), lincRNA [lnc-EXOSC8-1:1]	-0.493996	0.0051669944
A_33_P3294921	*PRUNE2*	Homo sapiens prune homolog 2 (Drosophila) (PRUNE2), mRNA [NM_015225]	-0.49318907	0.00972379
A_22_P00019123	*SLC35E3*	solute carrier family 35, member E3 [Source:HGNC Symbol;Acc:HGNC:20864] [ENST00000399333]	-0.48791385	3,00E+03
A_21_P0006946	*lnc-PITRM1-7*	LNCipedia lincRNA (lnc-PITRM1-7), lincRNA [lnc-PITRM1-7:8]	-0.48530948	0.034143027
A_23_P37410	*CYP19A1*	Homo sapiens cytochrome P450, family 19, subfamily A, polypeptide 1 (CYP19A1), transcript variant 2, mRNA [NM_031226]	0.47651383	0.0024354951
A_21_P0006868	*lnc-TMEM72-1*	LNCipedia lincRNA (lnc-TMEM72-1), lincRNA [lnc-TMEM72-1:1]	-0.47603342	1,94E-05
A_22_P00019604	*lnc-KIF16B-1*	LNCipedia lincRNA (lnc-KIF16B-1), lincRNA [lnc-KIF16B-1:1]	-0.46696115	0.0048126606
A_24_P135769	*VWA5A*	Homo sapiens von Willebrand factor A domain containing 5A (VWA5A), transcript variant 2, mRNA [NM_198315]	-0.45501333	1,74E-06
A_24_P299685	*PDPN*	Homo sapiens podoplanin (PDPN), transcript variant 2, mRNA [NM_198389]	-0.43971246	5,41E+00
A_22_P00011751	*ZFHX4-AS1*	Homo sapiens ZFHX4 antisense RNA 1 (ZFHX4-AS1), long non-coding RNA [NR_024360]	-0.43783396	1,14E-06
A_24_P109417	*DRAXIN*	Homo sapiens dorsal inhibitory axon guidance protein (DRAXIN), mRNA [NM_198545]	-0.43708912	0.04722463

A_21_P0013271	*SMKR1*	Homo sapiens small lysine-rich protein 1 (SMKR1), mRNA [NM_001195243]	-0.43567178	4,83E-06
A_23_P415611	*SLC9B1*	Homo sapiens solute carrier family 9, subfamily B (NHA1, cation proton antiporter 1), member 1 (SLC9B1), transcript variant 1, mRNA [NM_139173]	0.43274972	0.03387884
A_33_P3415395	*SPTLC3*	Homo sapiens serine palmitoyltransferase, long chain base subunit 3 (SPTLC3), mRNA [NM_018327]	0.42457253	0.0029840462
A_23_P324813	*BCL6B*	Homo sapiens B-cell CLL/lymphoma 6, member B (BCL6B), mRNA [NM_181844]	-0.42340666	2,67E+02
A_21_P0007062	*LINC00844*	Homo sapiens long intergenic non-protein coding RNA 844 (LINC00844), long non-coding RNA [NR_108046]	0.41562727	2,27E-01
A_22_P00015215	*LINC01152*	Homo sapiens long intergenic non-protein coding RNA 1152 (LINC01152), transcript variant 2, long non-coding RNA [NR_110132]	-0.4141439	4,52E+00
A_33_P3327097	*MTMR1*	myotubularin related protein 1 [Source:HGNC Symbol;Acc:HGNC:7449] [ENST00000370387]	-0.4125312	0.005973542
A_23_P323243	*PNLDC1*	Homo sapiens poly(A)-specific ribonuclease (PARN)-like domain containing 1 (PNLDC1), transcript variant 2, mRNA [NM_173516]	-0.40839273	2,85E+01
A_21_P0009626	*lnc-CDH2-1*	LNCipedia lincRNA (lnc-CDH2-1), lincRNA [lnc-CDH2-1:2]	0.39796638	0.033744548
A_33_P3381255	*CLEC2A*	Homo sapiens C-type lectin domain family 2, member A (CLEC2A), transcript variant 2, mRNA [NM_207375]	-0.39074126	4,95E-04
A_23_P19020	*SNCAIP*	Homo sapiens synuclein, alpha interacting protein (SNCAIP), transcript variant 1, mRNA [NM_005460]	-0.38562164	7,20E-07
A_22_P00004840	*LOC100506258*	Homo sapiens uncharacterized LOC100506258 (LOC100506258), long non-coding RNA [NR_103855]	-0.37540805	4,96E-01
A_23_P384748	*PLEKHH2*	Homo sapiens pleckstrin homology domain containing, family H (with MyTH4 domain) member 2 (PLEKHH2), mRNA [NM_172069]	0.3753041	0.008864218
A_24_P261470	*NRXN2*	Homo sapiens neurexin 2 (NRXN2), transcript variant alpha-2, mRNA [NM_138732]	-0.37121257	0.0029129395
A_33_P3626301	*DPY19L2P1*	Homo sapiens DPY19L2 pseudogene 1 (DPY19L2P1), non-coding RNA [NR_002833]	0.36768654	0.03937545
A_23_P390148	*RALGPS1*	Homo sapiens Ral GEF with PH domain and SH3 binding motif 1 (RALGPS1), transcript variant 1, mRNA [NM_014636]	-0.36637503	0.0
A_23_P320242	*KIAA1324L*	Homo sapiens KIAA1324-like (KIAA1324L), transcript variant 2, mRNA [NM_152748]	-0.36299175	0.033744548
A_22_P00017344	*lnc-USP50-1*	LNCipedia lincRNA (lnc-USP50-1), lincRNA [lnc-USP50-1:1]	-0.362718	2,19E+02
A_21_P0005474	*TWIST1*	PREDICTED: Homo sapiens twist family bHLH transcription factor 1 (TWIST1), transcript variant X2, misc_RNA [XR_428085]	-0.35930654	0.046908163

A_21_P0010896	*LINC00864*	Homo sapiens long intergenic non-protein coding RNA 864 (LINC00864), long non-coding RNA [NR_046091]	0.35898083	4,74E-01
A_33_P3605269	*KRT8P41*	Homo sapiens keratin 8 pseudogene 41 (KRT8P41), non-coding RNA [NR_027713]	-0.35877004	0.008307837
A_22_P00007155	*LOC101927269*	Homo sapiens uncharacterized LOC101927269 (LOC101927269), long non-coding RNA [NR_110075]	-0.35187984	0.0013620802
A_33_P3270489	*CCDC170*	Homo sapiens coiled-coil domain containing 170 (CCDC170), mRNA [NM_025059]	-0.34749174	1,38E+02
A_32_P125338	*FAM43B*	Homo sapiens family with sequence similarity 43, member B (FAM43B), mRNA [NM_207334]	0.34552854	0.0013620802
A_22_P00005194	*lnc-DLAT-1*	LNCipedia lincRNA (lnc-DLAT-1), lincRNA [lnc-DLAT-1:1]	0.3384218	0.033744548
A_21_P0007995	*LOC103191607*	Homo sapiens uncharacterized LOC103191607 (LOC103191607), long non-coding RNA [NR_120400]	-0.3342119	0.0013620802
A_23_P416965	*FAM149A*	Homo sapiens family with sequence similarity 149, member A (FAM149A), transcript variant 1, mRNA [NM_015398]	-0.33334947	8,99E+01
A_33_P3372004	*IGSF6*	Homo sapiens immunoglobulin superfamily, member 6 (IGSF6), mRNA [NM_005849]	0.32752222	3,21E-05
A_21_P0003701	*LINC01060*	Homo sapiens long intergenic non-protein coding RNA 1060 (LINC01060), long non-coding RNA [NR_033869]	-0.32734567	0.0013620802
A_23_P111361	*PPIL6*	Homo sapiens peptidylprolyl isomerase (cyclophilin)-like 6 (PPIL6), transcript variant 1, mRNA [NM_173672]	-0.32404646	0.0013620802
A_33_P3415623	*LRRIQ1*	leucine-rich repeats and IQ motif containing 1 [Source:HGNC Symbol;Acc:HGNC:25708] [ENST00000525971]	-0.31786758	9,83E+02
A_33_P3226357	*FOXE1*	Homo sapiens forkhead box E1 (FOXE1), mRNA [NM_004473]	-0.31704378	0.0018957087
A_33_P3270009	*NCOA1*	Homo sapiens nuclear receptor coactivator 1 (NCOA1), transcript variant 2, mRNA [NM_147223]	-0.31623057	0.010077573
A_32_P131640	*SLITRK4*	Homo sapiens SLIT and NTRK-like family, member 4 (SLITRK4), transcript variant 2, mRNA [NM_173078]	-0.30327168	0.024874954
A_22_P00004758	*LOC101927932*	Homo sapiens uncharacterized LOC101927932 (LOC101927932), long non-coding RNA [NR_126334]	-0.30204758	0.009129492
A_32_P140489	*GDF6*	Homo sapiens growth differentiation factor 6 (GDF6), mRNA [NM_001001557]	-0.30087674	0.0071114134
A_21_P0007228	*lnc-MYEOV-3*	LNCipedia lincRNA (lnc-MYEOV-3), lincRNA [lnc-MYEOV-3:1]	-0.2987	0.0013620802
A_23_P399897	*KY*	Homo sapiens kyphoscoliosis peptidase (KY), mRNA [NM_178554]	0.29578307	0.0055006724

A_33_P3283430	*SEL1L2*	Homo sapiens sel-1 suppressor of lin-12-like 2 (C. elegans) (SEL1L2), transcript variant 1, mRNA [NM_001271539]	0.29333848	0.023173148
A_24_P31627	*KCNB1*	Homo sapiens potassium channel, voltage gated Shab related subfamily B, member 1 (KCNB1), mRNA [NM_004975]	-0.29297817	2,53E-03
A_23_P135742	*PERM1*	Homo sapiens PPARGC1 and ESRR induced regulator, muscle 1 (PERM1), transcript variant 1, mRNA [NM_001291366]	0.2901024	0.0013620802
A_24_P3249	*RARB*	Homo sapiens retinoic acid receptor, beta (RARB), transcript variant 1, mRNA [NM_000965]	0.2736764	0.028035743
A_23_P168847	*FBXO16*	Homo sapiens F-box protein 16 (FBXO16), transcript variant 1, mRNA [NM_172366]	-0.27179226	0.023244653
A_23_P130333	*TTR*	Homo sapiens transthyretin (TTR), mRNA [NM_000371]	-0.27101725	0.0096237
A_33_P3404814	*GNAO1*	Homo sapiens guanine nucleotide binding protein (G protein), alpha activating activity polypeptide O (GNAO1), transcript variant 1, mRNA [NM_020988]	-0.26866764	0.033744548
A_33_P3415336	*ALS2*	amyotrophic lateral sclerosis 2 (juvenile) [Source:HGNC Symbol;Acc:HGNC:443] [ENST00000462747]	0.26688632	0.033744548
A_23_P346912	*SPATA17*	Homo sapiens spermatogenesis associated 17 (SPATA17), mRNA [NM_138796]	0.25909814	7,20E-07
A_21_P0009023	*lnc-AC136428.1.1-2*	RC0-GN0275-060201-032-g10 GN0275 Homo sapiens cDNA, mRNA sequence [BI055436]	-0.24089248	1,96E+03
A_23_P150903	*FAR2*	Homo sapiens fatty acyl CoA reductase 2 (FAR2), transcript variant 2, mRNA [NM_018099]	-0.23990673	0.03167772
A_23_P201022	*PKLR*	Homo sapiens pyruvate kinase, liver and RBC (PKLR), transcript variant 1, mRNA [NM_000298]	0.23976792	1,97E+03
A_32_P108889	*DCLK1*	Homo sapiens doublecortin-like kinase 1 (DCLK1), transcript variant 1, mRNA [NM_004734]	0.23644155	0.033744548
A_33_P3850216	*TRIM55*	Homo sapiens tripartite motif containing 55 (TRIM55), transcript variant 2, mRNA [NM_033058]	0.23559503	1,47E-05
A_33_P3897698	*LOC100147773*	Homo sapiens uncharacterized LOC100147773 (LOC100147773), long non-coding RNA [NR_125374]	-0.23548153	0.033744548
A_22_P00005133	*LINC00944*	long intergenic non-protein coding RNA 944 [Source:HGNC Symbol;Acc:HGNC:48640] [ENST00000542248]	-0.23200452	0.0015534712
A_21_P0000675	*LOC100287846*	Homo sapiens patched 1 pseudogene (LOC100287846), non-coding RNA [NR_037168]	-0.23031822	2,94E+01
A_21_P0001767	*FTCDNL1*	Homo sapiens formiminotransferase cyclodeaminase N-terminal like (FTCDNL1), long non-coding RNA [NR_034096]	-0.22861703	0.034170788
A_24_P314477	*TUBB2B*	Homo sapiens tubulin, beta 2B class IIb (TUBB2B), mRNA [NM_178012]	-0.22857314	0.0013620802

A_23_P904	*BEND5*	Homo sapiens BEN domain containing 5 (BEND5), transcript variant 1, mRNA [NM_024603]	-0.22781084	0.03171026
A_33_P3353345	*SULT4A1*	Homo sapiens sulfotransferase family 4A, member 1 (SULT4A1), mRNA [NM_014351]	-0.22460866	0.0013620802
A_21_P0006076	*lnc-FAM27E1.1-1*	LNCipedia lincRNA (lnc-FAM27E1.1-1), lincRNA [lnc-FAM27E1.1-1:1]	0.2244192	0.033744548
A_23_P161218	*ANKRD1*	Homo sapiens ankyrin repeat domain 1 (cardiac muscle) (ANKRD1), mRNA [NM_014391]	0.22365206	0.0013620802
A_23_P748	*IRF6*	Homo sapiens interferon regulatory factor 6 (IRF6), transcript variant 1, mRNA [NM_006147]	-0.22252312	0.004583383
A_24_P11100	*ZMAT1*	Homo sapiens zinc finger, matrin-type 1 (ZMAT1), transcript variant 2, mRNA [NM_001282400]	-0.22232647	7,20E-07
A_33_P3422213	*NEK10*	NIMA-related kinase 10 [Source:HGNC Symbol;Acc:HGNC:18592] [ENST00000341435]	0.21926588	0.021781867
A_24_P93754	*KDM4C*	lysine (K)-specific demethylase 4C [Source:HGNC Symbol;Acc:HGNC:17071] [ENST00000381306]	-0.21723932	0.035580207
A_21_P0005075	*lnc-TMEM242-1*	LNCipedia lincRNA (lnc-TMEM242-1), lincRNA [lnc-TMEM242-1:1]	-0.2163323	0.033744548
A_21_P0008333	*lnc-ABHD12B-4*	LNCipedia lincRNA (lnc-ABHD12B-4), lincRNA [lnc-ABHD12B-4:1]	-0.21607189	4,79E-03
A_24_P113131	*BZRAP1*	Homo sapiens benzodiazepine receptor (peripheral) associated protein 1 (BZRAP1), transcript variant 1, mRNA [NM_004758]	-0.20541592	0.033744548
A_21_P0004562	*lnc-ANKRA2-3*	LNCipedia lincRNA (lnc-ANKRA2-3), lincRNA [lnc-ANKRA2-3:2]	-0.20486781	0.002563978
A_33_P3358469	*GLI2*	Homo sapiens GLI family zinc finger 2 (GLI2), mRNA [NM_005270]	-0.20136926	5,04E-03
A_33_P3241782	*ADD2*	Homo sapiens adducin 2 (beta) (ADD2), transcript variant 1, mRNA [NM_001617]	-0.1993051	0.0029840462
A_21_P0004412	*lnc-ZNF366-6*	LNCipedia lincRNA (lnc-ZNF366-6), lincRNA [lnc-ZNF366-6:1]	-0.19620033	0.023138246
A_33_P3310961	*ADD3-AS1*	Homo sapiens ADD3 antisense RNA 1 (ADD3-AS1), long non-coding RNA [NR_038943]	0.19298504	0.01773788
A_23_P133665	*FRK*	Homo sapiens fyn-related Src family tyrosine kinase (FRK), mRNA [NM_002031]	0.19092995	0.0018047945
A_24_P13083	*TSPAN18*	Homo sapiens tetraspanin 18 (TSPAN18), mRNA [NM_130783]	-0.18899232	0.0013620802
A_19_P00800061	*LOC100289026*	PREDICTED: Homo sapiens putative uncharacterized protein FLJ00310-like (LOC100289026), misc_RNA [XR_424060]	-0.18832111	0.020403102
A_24_P69538	*TLR4*	Homo sapiens toll-like receptor 4 (TLR4), transcript variant 1, mRNA [NM_138554]	-0.1869232	0.019402675
A_22_P00001448	*PRKCA-AS1*	Homo sapiens PRKCA antisense RNA 1 (PRKCA-AS1), long non-coding RNA [NR_110822]	0.18517277	0.033677556
A_21_P0006134	*lnc-TEK-2*	LNCipedia lincRNA (lnc-TEK-2), lincRNA [lnc-TEK-2:1]	-0.18397969	0.0016918951
A_23_P254768	*HCG4*	Homo sapiens HLA complex group 4 (non-protein coding) (HCG4), long non-coding RNA [NR_002139]	-0.17222147	0.0041389074

A_22_P00005439	*lnc-DUOXA1-2*	LNCipedia lincRNA (lnc-DUOXA1-2), lincRNA [lnc-DUOXA1-2:1]	-0.16894858	0.026608149
A_33_P3363804	*NCAM1*	Homo sapiens neural cell adhesion molecule 1 (NCAM1), transcript variant 4, mRNA [NM_001242608]	-0.16843754	0.03578969
A_22_P00008915	*A2M-AS1*	Homo sapiens A2M antisense RNA 1 (head to head) (A2M-AS1), long non-coding RNA [NR_026971]	-0.16843066	0.006028756
A_22_P00008538	*KCNK15*	Homo sapiens potassium channel, two pore domain subfamily K, member 15 (KCNK15), mRNA [NM_022358]	0.16761783	0.001440138
A_32_P75902	*MEIOB*	Homo sapiens meiosis specific with OB domains (MEIOB), transcript variant 2, mRNA [NM_152764]	-0.16648431	0.010058458
A_33_P6817603	*IRF6*	Homo sapiens interferon regulatory factor 6 (IRF6), transcript variant 1, mRNA [NM_006147]	-0.16322568	0.0013793856
A_33_P3278916	*PHF20L1*	PHD finger protein 20-like 1 [Source:HGNC Symbol;Acc:HGNC:24280] [ENST00000361997]	0.15816495	0.001765581
A_33_P3548860	*SEC1P*	Homo sapiens secretory blood group 1, pseudogene (SEC1P), non-coding RNA [NR_004401]	0.15668352	0.0064550955
A_23_P421436	*ADD2*	Homo sapiens adducin 2 (beta) (ADD2), transcript variant 4, mRNA [NM_017488]	0.15256956	0.0014308001
A_33_P3322814	*NTRK3*	Homo sapiens neurotrophic tyrosine kinase, receptor, type 3 (NTRK3), transcript variant 3, mRNA [NM_001007156]	-0.1486336	0.0015611758
A_33_P3335910	*SYNE1*	Homo sapiens spectrin repeat containing, nuclear envelope 1 (SYNE1), transcript variant 2, mRNA [NM_033071]	-0.1480343	0.001440138
A_23_P201747	*PADI2*	Homo sapiens peptidyl arginine deiminase, type II (PADI2), mRNA [NM_007365]	0.14587463	1,41E-06
A_23_P252062	*PPARG*	Homo sapiens peroxisome proliferator-activated receptor gamma (PPARG), transcript variant 3, mRNA [NM_138711]	0.14154339	0.0053243246
A_21_P0014689	*LOC100506974*	PREDICTED: Homo sapiens uncharacterized LOC100506974 (LOC100506974), ncRNA [XR_111668]	-0.1399953	0.0013731016
A_33_P3328051	*CCNT2-AS1*	Homo sapiens CCNT2 antisense RNA 1 (CCNT2-AS1), long non-coding RNA [NR_036549]	0.13668452	0.020403102
A_33_P3331853	*PDE1C*	Homo sapiens phosphodiesterase 1C, calmodulin-dependent 70kDa (PDE1C), transcript variant 1, mRNA [NM_001191056]	-0.13136762	0.0016918951
A_23_P121795	*SORBS2*	Homo sapiens sorbin and SH3 domain containing 2 (SORBS2), transcript variant 2, mRNA [NM_021069]	0.1297663	0.01303263
A_32_P515431	*LOC100132057*	Homo sapiens phosphodiesterase 4D interacting protein pseudogene (LOC100132057), non-coding RNA [NR_111930]	0.12958598	0.0018047945

A_23_P329962	*SUN3*	Homo sapiens Sad1 and UNC84 domain containing 3 (SUN3), transcript variant 1, mRNA [NM_001030019]	-0.1203431	0.028199319
A_23_P84860	*FAM107A*	Homo sapiens family with sequence similarity 107, member A (FAM107A), transcript variant 1, mRNA [NM_007177]	-0.11780161	1,16E+00
A_23_P16275	*TSKS*	Homo sapiens testis-specific serine kinase substrate (TSKS), mRNA [NM_021733]	-0.116511494	0.043470398
A_24_P928969	*PTPN3*	Homo sapiens protein tyrosine phosphatase, non-receptor type 3 (PTPN3), transcript variant 3, mRNA [NM_001145369]	0.1155495	0.0013620802
A_22_P00001817	*lnc-ATP6V1D-2*	oz04h06.x1 Soares_fetal_liver_spleen_1NFLS_S1 Homo sapiens cDNA clone IMAGE:1674395 3', mRNA sequence [AI079571]	0.112588406	0.035322282
A_22_P00000368	*BHLHE40-AS1*	Homo sapiens BHLHE40 antisense RNA 1 (BHLHE40-AS1), transcript variant 4, long non-coding RNA [NR_125916]	0.10971048	7,38E+02
A_23_P153964	*INHBB*	Homo sapiens inhibin, beta B (INHBB), mRNA [NM_002193]	0.10736252	0.011196064
A_21_P0002458	*lnc-CD207-1*	LNCipedia lincRNA (lnc-CD207-1), lincRNA [lnc-CD207-1:1]	-0.09894207	0.045859136
A_23_P34045	*EDA*	Homo sapiens ectodysplasin A (EDA), transcript variant 1, mRNA [NM_001399]	0.09388271	0.026776692
A_21_P0000486	*SNORD103A*	Homo sapiens small nucleolar RNA, C/D box 103A (SNORD103A), small nucleolar RNA [NR_004054]	0.09014053	0.018618442
A_21_P0013331	*XLOC_l2_013886*	BROAD Institute lincRNA (XLOC_l2_013886), lincRNA [TCONS_l2_00026680]	0.084344685	0.011384754
A_24_P497464	*SOX9-AS1*	Homo sapiens SOX9 antisense RNA 1 (SOX9-AS1), transcript variant 1, long non-coding RNA [NR_103738]	0.07917648	0.00534224
A_23_P167479	*IL17B*	Homo sapiens interleukin 17B (IL17B), mRNA [NM_014443]	-0.07552795	0.0013620802
A_23_P330561	*MCEMP1*	Homo sapiens mast cell-expressed membrane protein 1 (MCEMP1), mRNA [NM_174918]	-0.07218626	1,60E-05
A_24_P475349	*RAB6B*	Homo sapiens RAB6B, member RAS oncogene family (RAB6B), mRNA [NM_016577]	0.0671412	0.024863733
A_23_P54291	*DUOX1*	Homo sapiens dual oxidase 1 (DUOX1), transcript variant 1, mRNA [NM_017434]	-0.0629498	0.00972379
A_22_P00004460	*LINC01085*	Homo sapiens long intergenic non-protein coding RNA 1085 (LINC01085), long non-coding RNA [NR_033931]	-0.06246864	0.024874954
A_32_P199767	*ZNF630*	Homo sapiens zinc finger protein 630 (ZNF630), transcript variant 1, mRNA [NM_001037735]	-0.060797174	0.033858534
A_21_P0012241	*LOC102725394*	PREDICTED: Homo sapiens uncharacterized LOC102725394 (LOC102725394), transcript variant X4, ncRNA [XR_424435]	-0.058803152	0.0014978354
A_21_P0001418	*lnc-ATF3-1*	LNCipedia lincRNA (lnc-ATF3-1), lincRNA [lnc-ATF3-1:3]	-0.054881167	0.0074755806

A_21_P0008663	*lnc-RP11-597K23.2.1-2*	BC011509 Cox15 protein {Mus musculus} (exp=-1; wgp=0; cg=0), partial (5%) [THC2616036]	-0.04907761	0.001518706
A_22_P00015031	*HOXC-AS3*	Homo sapiens HOXC cluster antisense RNA 3 (HOXC-AS3), long non-coding RNA [NR_047506]	0.04110983	0.0029129395
A_19_P00316667	*LOC101929450*	Homo sapiens uncharacterized LOC101929450 (LOC101929450), transcript variant 2, long non-coding RNA [NR_125815]	-0.03971428	0.012412555
A_23_P59807	*WNT2*	Homo sapiens wingless-type MMTV integration site family member 2 (WNT2), transcript variant 1, mRNA [NM_003391]	0.039298613	0.001440138
A_21_P0012866	*XLOC_l2_011872*	BROAD Institute lincRNA (XLOC_l2_011872), lincRNA [TCONS_l2_00022657]	0.036524784	0.025125682
A_21_P0004387	*LOC102467080*	Homo sapiens uncharacterized LOC102467080 (LOC102467080), transcript variant 2, long non-coding RNA [NR_104659]	-0.03622376	0.0013620802
A_33_P3281028	*MACROD2*	Homo sapiens MACRO domain containing 2 (MACROD2), transcript variant 1, mRNA [NM_080676]	-0.031337075	0.019062221
A_33_P3298775	*PRSS57*	Homo sapiens protease, serine, 57 (PRSS57), mRNA [NM_214710]	0.030933296	0.0013620802
A_33_P3287967	*ANK2*	Homo sapiens ankyrin 2, neuronal (ANK2), transcript variant 1, mRNA [NM_001148]	-0.02952921	0.033744548
A_22_P00009680	*LINC01468*	Homo sapiens long intergenic non-protein coding RNA 1468 (LINC01468), transcript variant 1, long non-coding RNA [NR_120641]	0.025005529	0.013584298
A_21_P0000364	*SNORD9*	Homo sapiens small nucleolar RNA, C/D box 9 (SNORD9), small nucleolar RNA [NR_003029]	-0.023501247	2,37E+00
A_33_P3213645	*ERN2*	Homo sapiens endoplasmic reticulum to nucleus signaling 2 (ERN2), mRNA [NM_033266]	-0.017491318	0.0030991563
A_33_P3305749	*RARB*	Homo sapiens retinoic acid receptor, beta (RARB), transcript variant 1, mRNA [NM_000965]	-0.016810158	0.0014006961
A_24_P270460	*IFI27*	Homo sapiens interferon, alpha-inducible protein 27 (IFI27), transcript variant 2, mRNA [NM_005532]	0.014057259	0.01073377
A_23_P157914	*MAMDC2*	Homo sapiens MAM domain containing 2 (MAMDC2), transcript variant 1, mRNA [NM_153267]	-0.008509323	3,10E+01
A_22_P00013123	*lnc-RMI1-1*	LNCipedia lincRNA (lnc-RMI1-1), lincRNA [lnc-RMI1-1:1]	-0.0064639123	1,55E+02
A_22_P00025282	*lnc-COL9A1-1*	PREDICTED: Homo sapiens uncharacterized LOC100507172 (LOC100507172), mRNA [XM_003118552]	0.0058030467	0.0020144724
A_22_P00013748	*GATA6-AS1*	Homo sapiens GATA6 antisense RNA 1 (head to head) (GATA6-AS1), long non-coding RNA [NR_102763]	-8.319841	0.0013620802
A_21_P0001698	*lnc-UBL4B-1*	LNCipedia lincRNA (lnc-UBL4B-1), lincRNA [lnc-UBL4B-1:4]	-1.0823225	0.033744548

A_21_P0002371	*lnc-SH3BP4-3*	601817555F1 NIH_MGC_57 Homo sapiens cDNA clone IMAGE:4041036 5', mRNA sequence [BF185848]	-1.0994786	0.0013620802
A_33_P3327041	*PLCE1-AS1*	Homo sapiens PLCE1 antisense RNA 1 (PLCE1-AS1), long non-coding RNA [NR_033969]	-1.1456672	0.0015534712
A_23_P76774	*GSC*	Homo sapiens goosecoid homeobox (GSC), mRNA [NM_173849]	-1.1615574	0.0027548962
A_21_P0000129	*DCLK1*	Homo sapiens doublecortin-like kinase 1 (DCLK1), transcript variant 4, mRNA [NM_001195430]	-1.1749325	0.024207804
A_24_P183128	*PLAC8*	Homo sapiens placenta-specific 8 (PLAC8), transcript variant 2, mRNA [NM_016619]	-1.2127153	0.0152475005
A_23_P66481	*RTN4RL1*	Homo sapiens reticulon 4 receptor-like 1 (RTN4RL1), mRNA [NM_178568]	-1.2620338	0.0013620802
A_23_P258088	*PACSIN1*	Homo sapiens protein kinase C and casein kinase substrate in neurons 1 (PACSIN1), transcript variant 1, mRNA [NM_020804]	-1.2895532	0.006028756
A_33_P3247057	*SMIM5*	Homo sapiens small integral membrane protein 5 (SMIM5), transcript variant 1, mRNA [NM_001162995]	-1.3303874	0.0013620802
A_33_P3307810	*CCDC148*	Homo sapiens coiled-coil domain containing 148 (CCDC148), transcript variant 1, mRNA [NM_138803]	-1.4512222	0.033744548
A_22_P00022238	*LINC01391*	Homo sapiens long intergenic non-protein coding RNA 1391 (LINC01391), long non-coding RNA [NR_121649]	-1.4682915	0.035748977

Table S4**.** Characteristics of Anti-NSD1 siRNA-AT1, Anti-NSD1 siRNA-AT2, Anti-NSD1 siRNA-AT3, Anti PPIA and Anti-Cy3 siRNA Duplexes and Their Position in the *NSD1* and PPIA mRNAs.

Target	*Target sequence*	*Sequence 5'→3'*	*GC content*
Anti-NSD1 siRNA-AT1	5'-GTCAAAGAACCGAAAATGTATTC-3'	Sense: CAGACUGUCUCAAUCUGACC Antisense: GGUCAGAUUGAGACAGUCUG	50 %
Anti-NSD1 siRNA-AT2	5'-AAGGAAATTATGACAAAGTAAAC-3'	Sense: UUACUUUGUCAUAAUUUCCUU Antisense: GGAAAUUAUGACAAAGUAAAC	23.8 %
Anti-NSD1 siRNA-AT3	5'-TAGACAAAGACCAAATCCCAATC-3'	Sense: UUGGGAUUUGGUCUUUGUCUA Antisense: GACAAAGACCAAAUCCCAAUC	38.1 %
Anti-NSD1 siRNA control	5'- ATGATTTAGACCCTGGATTTATG-3'	Sense: UAAAUCCAGGGUCUAAAUCAU Antisense: GAUUUAGACCCUGGAUUUAUG	34.7 %
siRNA-AT3	5'- GTGGTGTTTGGCAAAGTGAAAGA-3'	Sense: UUUCACUUUGCCAAACACCAC Antisense: GGUGUUUGGCAAAGUGAAAGA	42.8%
Anti-Cy3 siRNA	5'-AATTCTCCGAACGTGTCACGT-3'	Sense: UUCUCCGAACGUGUCACGUdTdT Antisense: ACGUGACACGUUCGGAGAAdTdT	52.6%

Table S5. Primers of differentially expressed genes used for validation by real-time PCR

Gene Symbol	RefSeq Tran-script ID	Forward Primers	Reverse Primers	Pro duct size (bp)	Temp
KRAS	NM_004985	5'AGCCCTTTGTA CACCCTAA3'	5'GTCGTCATCGTCTTT GTAGTC3'	125	60°C
FGF1	NM_000800	5'GTGGATGGGAC AAGGGACAG3'	5'GGCAGGGGGAGAA ACAAGAT3'	120	60°C
FGF3	NM_005247	5'GGAAATTAAAG GGACTCGCG3'	5'GATCCCGCGCCTGG AGATG'3	188	60°C
SFN	NM_006142	5'CAGGCTACTTC TCCCCTCCT3'	5'TCAATCTCGGTCTT GCACTG3'	100	60°C
ACTR2	NM_001005386	5'GACTACACATTT GGACCAGAGA3'	5'CTTCTCTCTGTTTTT GGTTGGG3'	119	60°C
ACTR3	NM_005721	5'CTGGGTTGCGG AAGTGATAG3'	5'TGTATACCCCGTGC CACAGT'33'	145	60°C
ARPC2	NM_152862	5'TCCGACTCTAC CAGCTGATGC3'	5'AAGCTGGACTCATC CCACAGC3'	150	60°C
ARPC3	NM_001278556	5'GTGCAATTCCA AAAGCCAAGG3'	5'GGCTCTCATCACTT CATCTTCC3'	100	60°C
FEM1A	NM_018708	5'TGCATCCGCTT GTGGAAGTACG3'	5'AGCACGTAGGAGAA GAGTTCCG 3'	122	60°C
NEDD	NM_006403	5'CCCATCCAGAT ACCAAAAGGAC3' 3'	5'CACTGGAACTGAAA ACACAGGGC3'	128	60°C
GSC	NM_173849	5'GTTCAGCATCG ACAACATCC3'	5'TAGAAGGCGCCATA GTCCGA3'	150	60°C
NCAM1	NM_001242607	5'CATCACCTGGA GGACTTCTACC3'	5'CAGTGTACTGGATG CTCTTCAGG3'	140	60°C
NCOA1	NM_147223	5'TGATTTGTATTG CACGGCGAT3'	5'AAATGCACTTCCTCA CTA3'	150	60°C
